# Supplementary material for: Encapsulated Co–Ni alloy boosts high-temperature CO2 electroreduction
Source: Nature. 2025 May 14;641(8065):1156–61. doi: 10.1038/s41586-025-08978-0 (PMC12119355; doi:10.1038/s41586-025-08978-0)
Supplement: Supplementary file 1 — Supplementary Note 1, Figs. 1–47, Tables 1–17 and references. [file 41586_2025_8978_MOESM1_ESM.pdf]

Supplementary Information for

Encapsulated Co-Ni alloy boosts high-temperature CO_2 electroreduction

Authors: Wenchao Ma[1], Jordi Morales-Vidal[2], Jiaming Tian[1], Meng-Ting Liu[3], Seongmin Jin[4], Wenhao Ren[1], Julian Taubmann[5], Christodoulos Chatzichristodoulou[5], Jeremy Luterbacher[4], Hao Ming Chen[3], Núria López[2], Xile Hu[1]*

Affiliations:

[1]Laboratory of Inorganic Synthesis and Catalysis, Institute of Chemical Sciences and Engineering, Ecole Polytechnique Fédérale de Lausanne (EPFL), Lausanne 1015, Switzerland.

[2]Institute of Chemical Research of Catalonia (ICIQ-CERCA), The Barcelona Institute of Science and Technology, Tarragona 43007, Spain.

[3]Department of Chemistry and Center for Emerging Materials and Advanced Devices, National Taiwan University, Taipei 10617, Taiwan.

[4]Laboratory of Sustainable and Catalytic Processing, Institute of Chemical Sciences and Engineering, École Polytechnique Fédérale de Lausanne (EPFL), Lausanne 1015, Switzerland.

[5]Department of Energy Conversion and Storage, Technical University of Denmark, Kongens Lyngby 2800, Denmark.

*Corresponding author. Email: xile.hu@epfl.ch

Supplementary Note 1. Preliminary cost estimation

In this work, we employ a modified model based on Ref. [1] to roughly estimate the costs to produce 100 ton of CO per day, starting from CO_2 feedstock. We assume that the MEA plant operates at a CO FE of 92% (with the remaining 8% being H_2), a current density of 1.75 A/cm^2, a cell voltage of 3.5 V, a CO single-pass yield of 33%, and a catalyst and membrane lifetime of 210 h (although the system lifetime was only 12 h as the electrolyte needed to be refreshed every 12 h), based on the results of Ref. [2]. We assume that the SOEC plant operates at a CO FE of 100%, a current density of 0.5 A/cm^2, a cell voltage of 1.2 V, a CO single-pass yield of 45%, and a catalyst and electrolyte lifetime of 1,000 h, based on the results of Ref. [3]. Our system operates at a CO FE of 100%, a current density of 1.0 A/cm^2, a cell voltage of 1.2 V, a CO single-pass yield of 90%, and a catalyst and electrolyte lifetime of 2,000 h. The input parameters and output results are summarized in Supplementary Tables 16 and 17, respectively, while the detailed estimations and assumptions are described below for ease of reference:

CO_2 cost

We assume that the conversion efficiency from CO_2 feedstocks to CO products is 100%, and any losses of CO_2 will be factored into other calculations that involve recycling CO_2 back to the cathode. Based on a CO_2 price of 30 \$/ton (Ref. 1), the cost of input CO_2 per ton of CO can be estimated by eq. 1:

$$\boldsymbol{CO_2\ cost}\left[\frac{\$}{\text{ton CO}}\right] = \frac{Mr_{CO_2}\left[\frac{g}{mol}\right]}{Mr_{CO}\left[\frac{g}{mol}\right]} \times Molar\ ratio\left[\frac{CO_2}{\text{CO}}\right] \times CO_2\ price\left[\frac{\$}{\text{ton}}\right] \quad (1)$$

Electrolyser cost

The CO production rate in a unit of moles per second can be calculated by eq. 2:

$$CO\ production\left[\frac{\text{mol}}{\text{s}}\right] = \frac{CO\ production\left[\frac{g}{day}\right]}{Mr_{CO}\left[\frac{g}{mol}\right]\times 86400\left[\frac{s}{day}\right]} \quad (2)$$

The total current needed per day can be calculated by eq. 3:

$$Total\ current\left[\frac{\text{A}}{\text{day}}\right] = \frac{CO\ production\left[\frac{\text{mol}}{\text{s}}\right]\times 2\times 96485\left[\frac{\text{C}}{\text{mol}}\right]}{FE_{CO}[decimal]} \quad (3)$$

The total power consumed per day can be calculated by eq. 4:

$$Total\ power\left[\frac{\text{kW}}{\text{day}}\right] = Total\ current\left[\frac{\text{A}}{\text{day}}\right] \times Cell\ voltage\ [V] \times \frac{1kW}{1000W} \quad (4)$$

The total electrolyser cost can be estimated by eq. 5:

$$Total\ electrolyser\ cost[\$]$$

$$= Total\ power[kW] \times Base\ electrolyser\ price\ \left[\frac{\$}{kW}\right] \times \frac{Base\ current\ density\left[\frac{\text{A}}{cm^2}\right]}{Input\ current\ density\left[\frac{\text{A}}{cm^2}\right]} \quad (5)$$

For the MEA electrolyser, the base price is estimated to be 100 \$/kW, based on a 2050 target for AEM water electrolyser at a base current density of 2.0 A/cm^2 (Ref. 4). For the SOEC electrolyser, the base price is estimated to be 200 \$/kW, based on a 2050 target for SOEC water electrolyser at a base current density of 2.0 A/cm^2 (Ref. 4).

To estimate the electrolyser cost per ton of CO, we first calculate the annual cost of the plant, assuming zero salvage value at the end of its lifetime. We then divide this value by the number of operating days of the plant to get a daily cost. This estimation can be used for all capital costs and starts by calculating a capital recovery factor (*CRF*) based on discount rate (*i*) and lifetime of the materials:

$$CRF = \frac{i \times (1+i)^{lifetime[year]}}{(1+i)^{lifetime[year]} - 1} \quad (6)$$

Thus, the electrolyser cost per ton of CO can be estimated by eq. 7:

$$\boldsymbol{Electrolyser\ cost}\left[\frac{\$}{\textbf{ton CO}}\right] = \frac{CRF_{electrolyser} \times Total\ electrolyser\ cost[\$]}{Capacity\ factor \times 365\left[\frac{\text{days}}{year}\right] \times CO\ production\left[\frac{\text{ton}}{\text{day}}\right]} \quad (7)$$

For both MEA and SOEC electrolysers, we assume a discount rate (*i*) of 5%, a capacity factor of 0.9, and a lifetime of 30 years[1,4].

Catalyst and electrolyte costs:

The costs of catalyst and electrolyte are estimated by summing the total costs of all materials used in their synthesis, assuming a 100% atomic efficiency. The quantities and corresponding prices for each material were listed in Supplementary Table 15, based on data from Thermo Fisher Scientific, US[5]. For the MEA cells[2], the prices for Ag nanoparticles, gas diffusion electrodes, Ni foam and Nafion membranes are assumed to be 18.7, 500, 250, and 500 \$/m^2, respectively[5].

The total catalyst and electrolyte size can be estimated by eq. 8:

$$Catalyst\ and\ electrolyte\ size[m^2] = \frac{Total\ current[\text{A}]}{Input\ current\ density\left[\frac{\text{A}}{cm^2}\right] \times 10^4\ \frac{cm^2}{m^2}} \quad (8)$$

The total catalyst and electrolyte cost can be estimated by eq. 9:

$$Total\ catalyst\ and\ electrolyte\ cost[\$]$$

$$= (cathode\ cost + anode\ cost + electrolyte\ cost)\ \left[\frac{\$}{m^2}\right] \times Catalyst\ and\ electrolyte\ size[m^2] \quad (9)$$

Thus, the catalyst and membrane cost per ton of CO can be estimated by eq. 10:

$$\boldsymbol{Catalyst\ and\ membrane\ cost}\left[\frac{\$}{\textbf{ton CO}}\right] = \frac{Catalyt\ and\ membrane\ cost\left[\frac{\$}{m^2}\right] \times CRF_{catalyst\ and\ membrane}}{Capacity\ factor \times 365\left[\frac{\text{days}}{year}\right] \times CO\ production\left[\frac{\text{ton}}{\text{day}}\right]} \quad (10)$$

Separation cost:

For the separation cost of the cathode gas outlets, a pressure swing adsorption (PSA) separation unit is utilized with a base price of 1,989,043 \$, a base capacity of 1,000 m^3/h, a scaling factor of 0.7, and a base electricity requirement of 0.25 kWh/m^3 (Ref. 6). Since normally no CO_2 gas exits in the anode gas outlets for either MEA or SOEC, we assume that there is no cost associated with anode gas separation.

Although the SOEC gases are maintained at 800 °C, we assume that they have enough time to cool down to room temperature before entering the PSA unit. Thus, the output CO flow rates for both MEA and SOEC can be calculated by eq. 11:

$$CO\ flow\ rate\left[\frac{m^3}{h}\right] = \frac{CO\ production\left[\frac{\text{ton}}{\text{day}}\right] \times \frac{10^6 g}{1\ ton} \times 8.314 \frac{J}{mol\ K} \times 298K}{28 \frac{g}{mol} \times 101325Pa \times 24 \frac{h}{day}} \quad (11)$$

The output CO_2 flow rate can be calculated by eq. 12:

$$CO_2\ flow\ rate\left[\frac{m^3}{h}\right] = \frac{100 - CO\ single\ pass\ yield\ [\%]}{CO\ single\ pass\ yield\ [\%]} \times CO\ flow\ rate\left[\frac{m^3}{h}\right] \times Molar\ ratio\left[\frac{CO_2}{\text{CO}}\right] \quad (12)$$

The output H_2 flow rate can be calculated by eq. 13:

$$H_2\ flow\ rate\left[\frac{m^3}{h}\right] = \frac{FE_{H_2}[\%]}{FE_{CO}[\%]} \times CO\ flow\ rate\left[\frac{m^3}{h}\right] \times Molar\ ratio\left[\frac{H_2}{\text{CO}}\right] \quad (13)$$

For the MEA electrolysers, due to the generated OH^- in the cathode, there will be 1 mole of CO_2 that crossovers to anode in the form of carbonate for the production of 1 mole of CO or H_2. There is no CO_2 crossover issue in the SOEC electrolysers. The CO_2 crossover rate in MEA can be calculated by eq. 14:

$$CO_2\ crossover\ rate\left[\frac{m^3}{h}\right]$$

$$= \frac{FE_{H_2}[\%] + FE_{CO}[\%]}{FE_{CO}[\%]} \times CO\ single\ pass\ yield\ [decimal] \times CO\ flow\ rate\left[\frac{m^3}{h}\right] \quad (14)$$

Thus, the total cathode output gas flow rate can be calculated by eq. 15:

$$Total\ flow\ rate\left[\frac{m^3}{h}\right]$$

$$= (CO\ flow\ rate + CO_2\ flow\ rate + H_2\ flow\ rate - CO_2\ crossover\ rate)\left[\frac{m^3}{h}\right] \quad (15)$$

The total *PSA* capital cost can be estimated by eq. 16:

$$Total\ PSA\ capital\ cost[\$] = 1989043[\$] \times \left(\frac{Total\ flow\ rate\left[\frac{m^3}{h}\right]}{1000\left[\frac{m^3}{h}\right]}\right)^{0.7} \quad (16)$$

The *PSA* capital cost per ton of CO can be estimated by eq. 17:

$$PSA\ capital\ cost\left[\frac{\$}{\text{ton CO}}\right] = \frac{CRF_{electrolyser} \times Total\ PSA\ capital\ cost[\$]}{Capacity\ factor \times 365\left[\frac{\text{days}}{year}\right] \times CO\ production\left[\frac{\text{ton}}{\text{day}}\right]} \quad (17)$$

The *PSA* operating cost per ton of CO can be estimated by eq. 18:

$$PSA\ operating\ cost\left[\frac{\$}{\text{ton CO}}\right] = \frac{0.25\frac{kWh}{m^3} \times Total\ flow\ rate\left[\frac{m^3}{h}\right] \times 24\frac{h}{day} \times Electricity\ price\left[\frac{\$}{kWh}\right]}{CO\ production\left[\frac{\text{ton}}{\text{day}}\right]} \quad (18)$$

Therefore, the separation cost of per ton of CO can be estimated by eq. 19:

$$\boldsymbol{Separation\ cost}\left[\frac{\$}{\textbf{ton CO}}\right] = PSA\ capital\ cost\left[\frac{\$}{\text{ton CO}}\right] + PSA\ operating\ cost\left[\frac{\$}{\text{ton CO}}\right] \quad (19)$$

Carbonate regeneration cost:

To calculate the regeneration costs of CO_2 in the anolyte resulting from carbonate crossover in MEA, we use a calcium caustic loop model with a base energy requirement of 1,229 kWh per ton of CO_2 (Ref. 1,7). As there is no carbonate crossover issue in SOEC, we assume zero cost associated with carbonate regeneration.

The lost CO_2 amount to carbonate per day for MEA can be calculated by eq. 20:

$$Lost\ CO_2\left[\frac{ton}{day}\right] = \frac{101325\ Pa \times 44\frac{g}{mol} \times 24\ h}{8.314\frac{J}{mol\ K} \times 298K} \times CO_2\ crossover\ rate\left[\frac{m^3}{h}\right] \times \frac{1\ ton}{10^6\ g} \quad (20)$$

Therefore, the carbonate regeneration cost per ton of CO in MEA can be estimated by eq. 21:

$$\boldsymbol{Carbonate\ regeneration\ cost}\left[\frac{\$}{\textbf{ton CO}}\right] = \frac{1229\frac{kWh}{ton} \times Lost\ CO_2\left[\frac{ton}{day}\right] \times Electricity\ price\left[\frac{\$}{kWh}\right]}{CO\ production\left[\frac{\text{ton}}{\text{day}}\right]} \quad (21)$$

Heating cost:

To calculate the heating cost, we consider the costs of heating CO_2 and the electrolyser (where nichrome is assumed as the material) from room temperature to reaction temperature, with a heating efficiency of 90% (Ref. 8). The heat capacities of CO_2 and nichrome are 843 and 460 J/kg/K, respectively. The density of nichrome is 8,400 kg/m^3. We assume an electrolyser thickness of 5 cm.

The mass of CO_2 required to be heated per day can be calculated by eq. 22:

$$CO_2\ mass\ \left[\frac{\text{kg}}{\text{day}}\right] = CO\ production\left[\frac{\text{ton}}{\text{day}}\right] \times \frac{Mr_{CO_2}\left[\frac{g}{mol}\right]}{Mr_{CO_2}\left[\frac{g}{mol}\right]} \times \frac{1}{CO\ single\ pass\ yield\ [demical]} \times \frac{10^3\ kg}{1\ ton} \quad (22)$$

The energy required to heat CO_2 per day can be estimated by eq. 23:

$$Heating\ energy_{CO_2}\left[\frac{\text{GJ}}{\text{day}}\right] = 843\ \frac{J}{kg\times K} \times (T[K] - 298K) \times CO_2\ mass\ \left[\frac{\text{kg}}{\text{day}}\right] \times \frac{1\ GJ}{10^9\ J} \quad (23)$$

The surface area of the electrolyser can be calculated by eq. 24:

$$Electrolyser\ area[m^2] = \frac{Total\ current[A]}{Input\ current\ density\left[\frac{\text{A}}{cm^2}\right]} \times \frac{1\ m^2}{10000\ cm^2} \quad (24)$$

The mass of nichrome required to be heated per day can be calculated by eq. 25:

$$Nichrome\ mass\left[\frac{\text{kg}}{\text{day}}\right] = Electrolyser\ area[m^2] \times Electrolyser\ thickness[m] \times 8400\ \frac{kg}{m^3} \quad (25)$$

The energy required to heat nichrome per day can be estimated by eq. 26:

$$Heating\ energy_{nichrome}\left[\frac{\text{GJ}}{day}\right] = 460\ \frac{J}{kg\times K} \times (T[K] - 298K) \times Nichrome\ mass\ \left[\frac{\text{kg}}{\text{day}}\right] \times \frac{1\ GJ}{10^9\ J} \quad (26)$$

Therefore, the total heating cost per ton of CO can be estimated by eq. 27:

$$\boldsymbol{Heating\ cost}\left[\frac{\$}{\textbf{ton CO}}\right] = \frac{\left(Heating\ energy_{CO_2} + Heating\ energy_{nichrome}\right)\left[\frac{\text{GJ}}{day}\right] \times \frac{1\ kWh}{0.0036\ GJ} \times Electricity\ price\ \left[\frac{\$}{kWh}\right]}{CO\ production\left[\frac{\text{ton}}{\text{day}}\right] \times Heating\ efficiency\ [decimal]} \quad (27)$$

Electricity cost:

The electricity cost per ton of CO can be estimated by eq. 28:

$$\boldsymbol{Electricity\ cost}\left[\frac{\$}{\textbf{ton CO}}\right] = \frac{Total\ power\left[\frac{kW}{day}\right] \times \frac{24\ h}{1\ day} \times Electricity\ price\ \left[\frac{\$}{kWh}\right]}{CO\ production\left[\frac{\text{ton}}{\text{day}}\right]} \quad (28)$$

Other operating cost:

We add an additional 10% of the electricity costs to account for the labour and maintenance costs during plant operation[1]:

$$\boldsymbol{Other\ operating\ costs}\left[\frac{\$}{\textbf{ton CO}}\right] = 0.1 \times Electricity\ cost\left[\frac{\$}{\text{ton CO}}\right] \quad (29)$$

Balance of Plant (BoP) and installation costs:

The BoP and installation costs are estimated by adding 150% of all capital costs to the final cost of CO (Ref. 1,4). The total capital costs include the costs of electrolyser, catalyst and electrolyte, PSA capital, and carbonate regeneration. Therefore, the BoP cost per ton of CO can be estimated by eq. 30:

$$\boldsymbol{BoP\ and\ installation\ costs}\left[\frac{\$}{\textbf{ton CO}}\right]$$

$$= 1.5 \times (Electrolyser\ cost + Catalyst\ and\ electrolyte\ cost + PSA\ capital\ cost + Carbonate\ regeneration\ cost)\left[\frac{\$}{\text{ton CO}}\right] \quad (30)$$

Total costs:

By summing up all of the above costs, we can determine the total costs of producing 1 ton of CO.

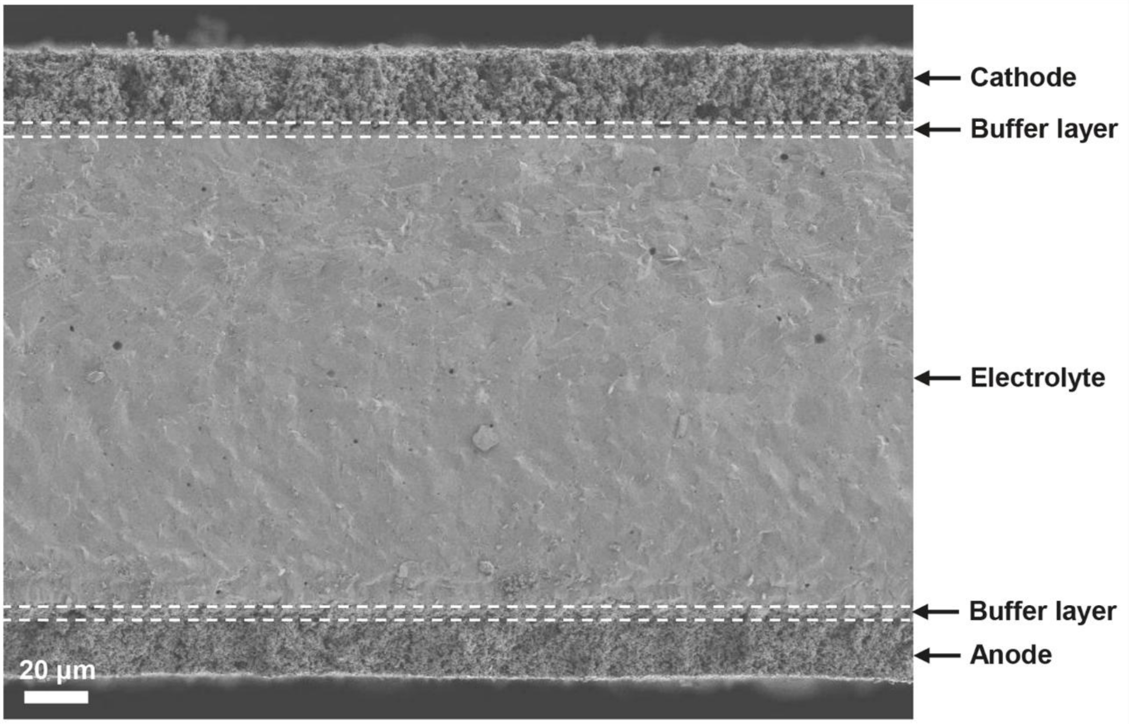

Supplementary Fig. 1 | Cross-section SEM view of our electrolyte-supported cell.

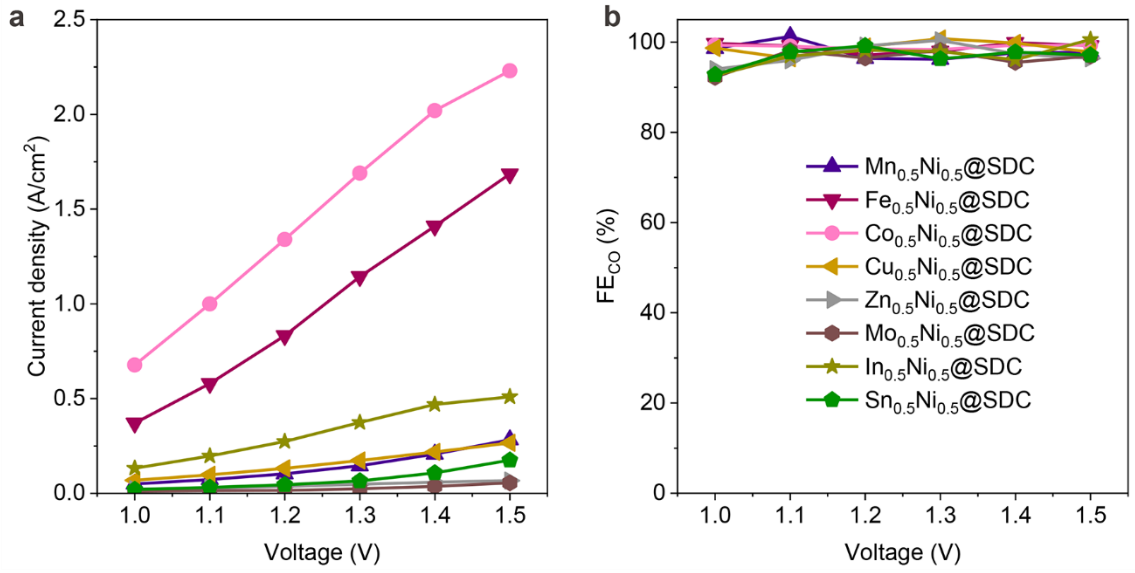

Supplementary Fig. 2 | The performance of CO_2 electroreduction for $M_{0.5}Ni_{0.5}$@SDC catalysts. a, Current densities. **b**, FEs of CO.

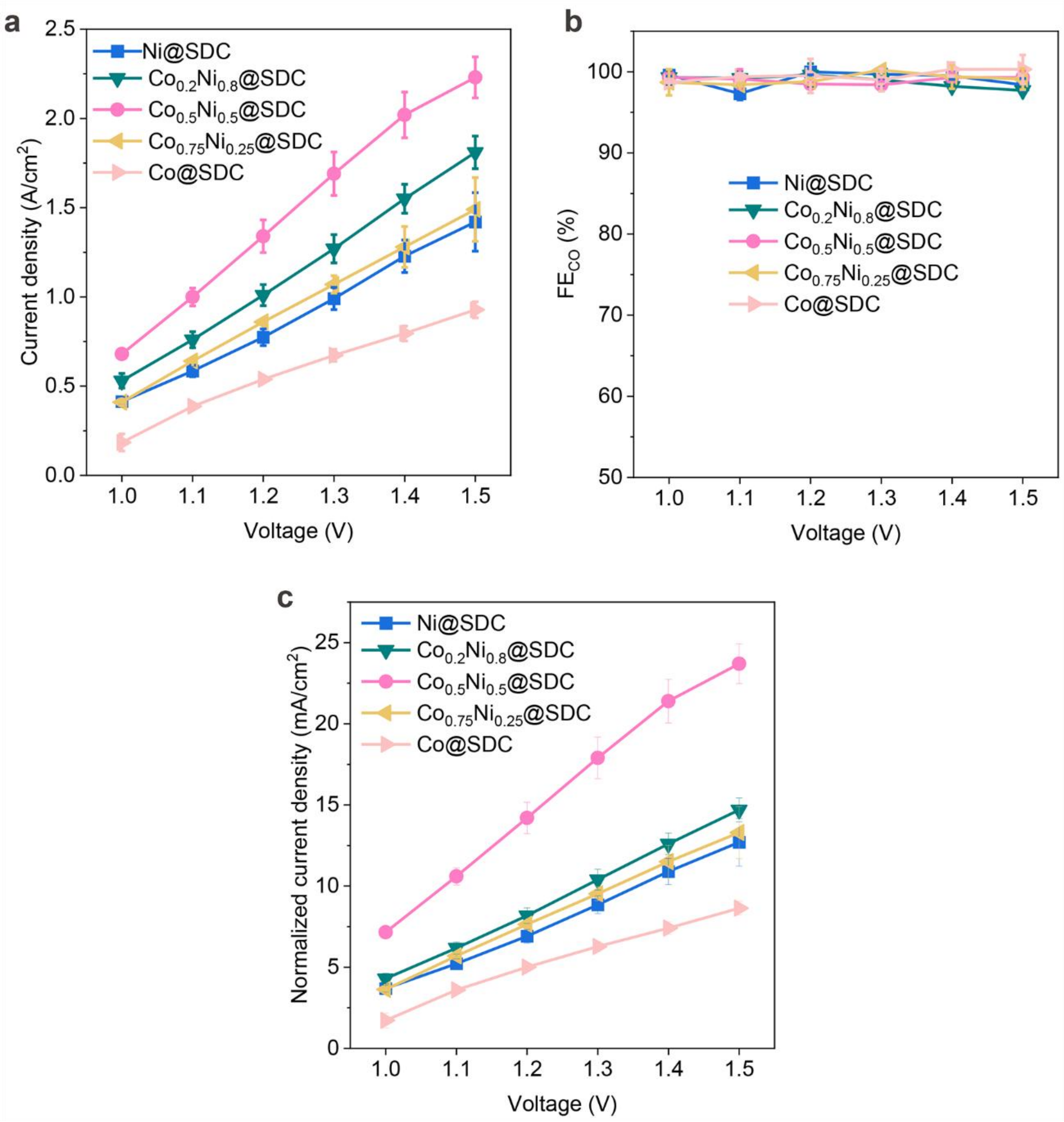

Supplementary Fig. 3 | The performance of CO_2 electroreduction for Co_xNi_{1-x}@SDC catalysts with different Co and Ni molar ratios. a, Current densities. **b**, FEs of CO. **c**, Normalized current densities based on ECSAs. The results are shown as mean ± s.d. based on three individual experiments.

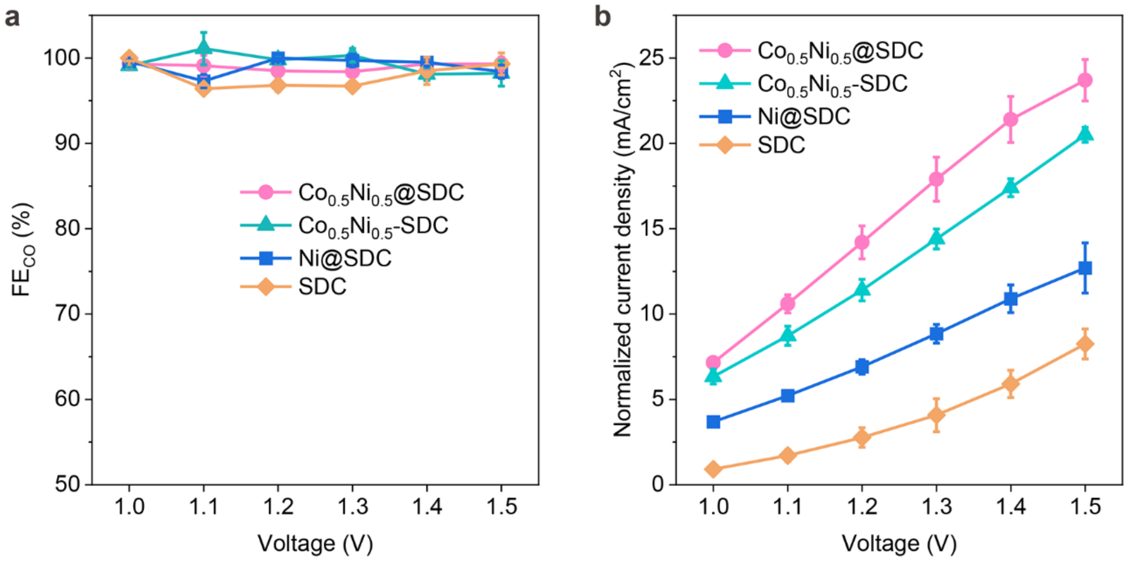

Supplementary Fig. 4 | The performance of CO_2 electroreduction for different catalysts. **a**, FEs of CO. **b**, Normalized current densities based on ECSAs. The results are shown as mean ± s.d. based on three individual experiments.

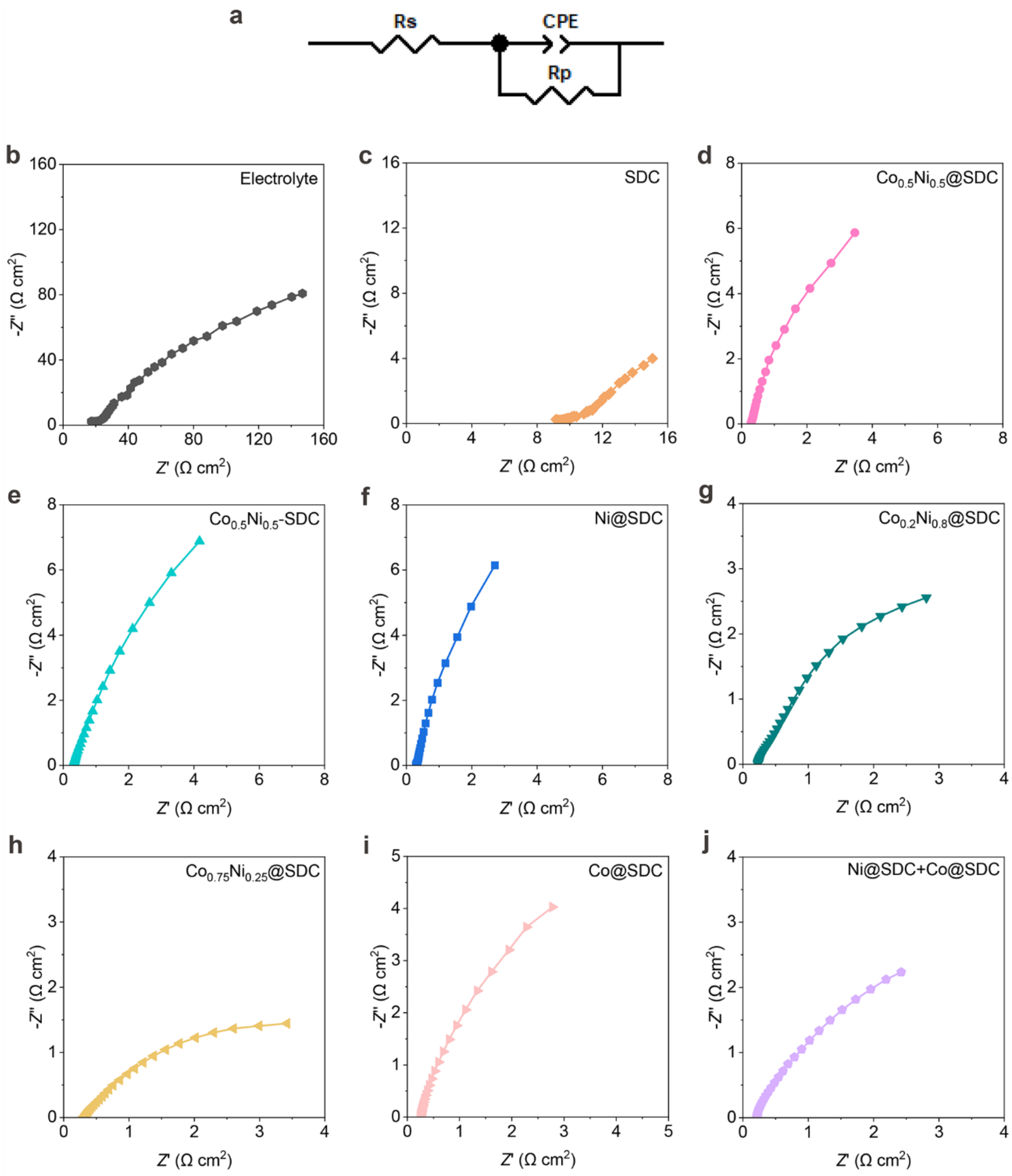

Supplementary Fig. 5 | Nyquist plots obtained from EIS for symmetrical cells. a, Equivalent Randles circuit for fitting. **b**, Electrolyte. **c**, SDC. **d**, $Co_{0.5}Ni_{0.5}$@SDC. **e**, $Co_{0.5}Ni_{0.5}$-SDC. **f**, Ni@SDC. **g**, $Co_{0.2}Ni_{0.8}$@SDC. **h**, $Co_{0.75}Ni_{0.25}$@SDC. **i**, Co@SDC. **j**, Ni@SDC+ Co@SDC.

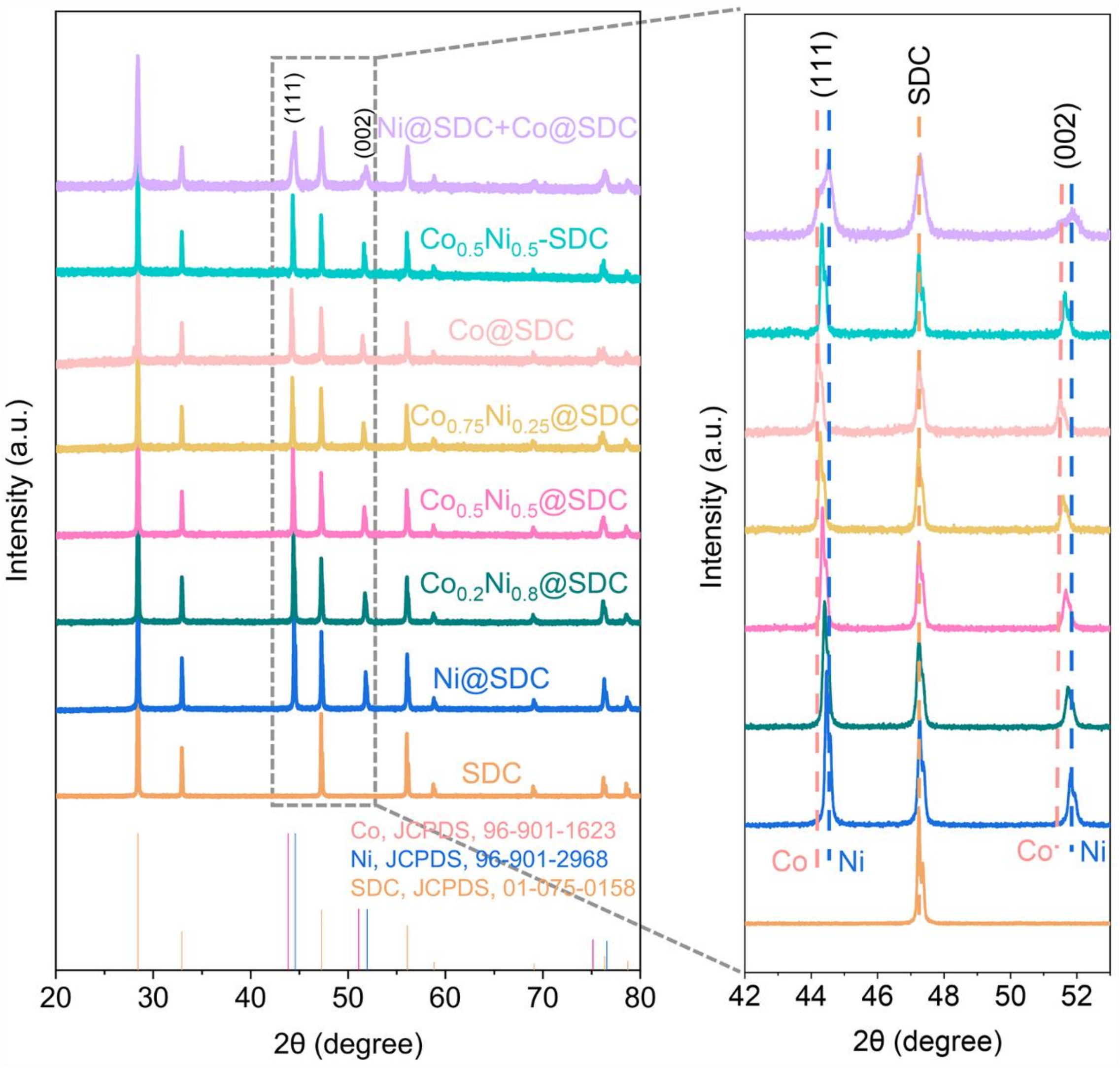

Supplementary Fig. 6 | XRD patterns of fresh samples.

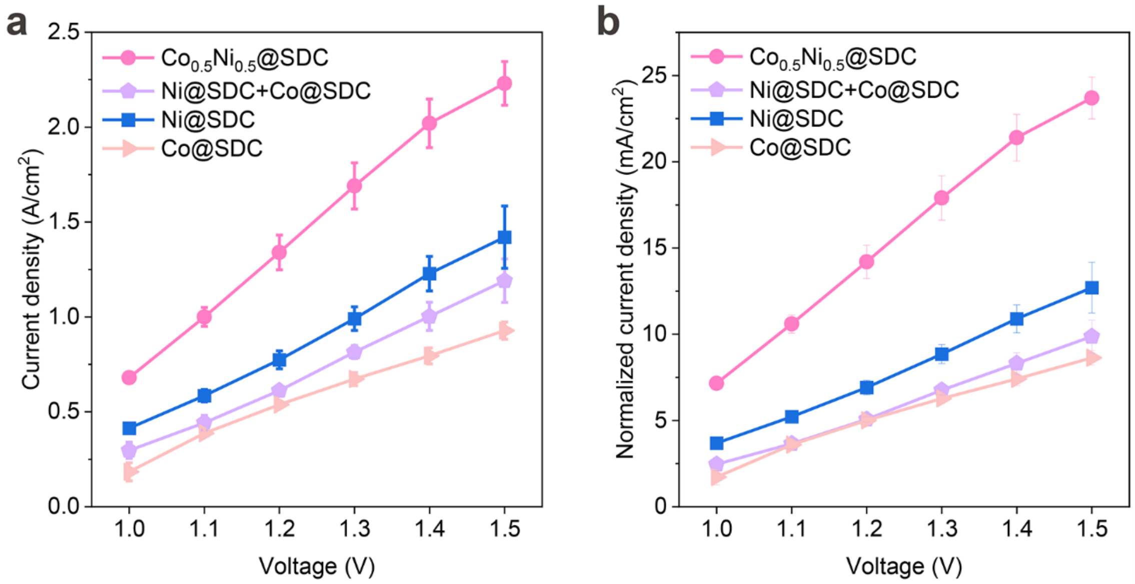

Supplementary Fig. 7 | The performance of CO_2 electroreduction for a physical mixture of Ni@SDC and Co@SDC. a, Current densities at different cell voltages. **b**, Normalized current densities based on ECSAs. The results are shown as mean ± s.d. based on three individual experiments.

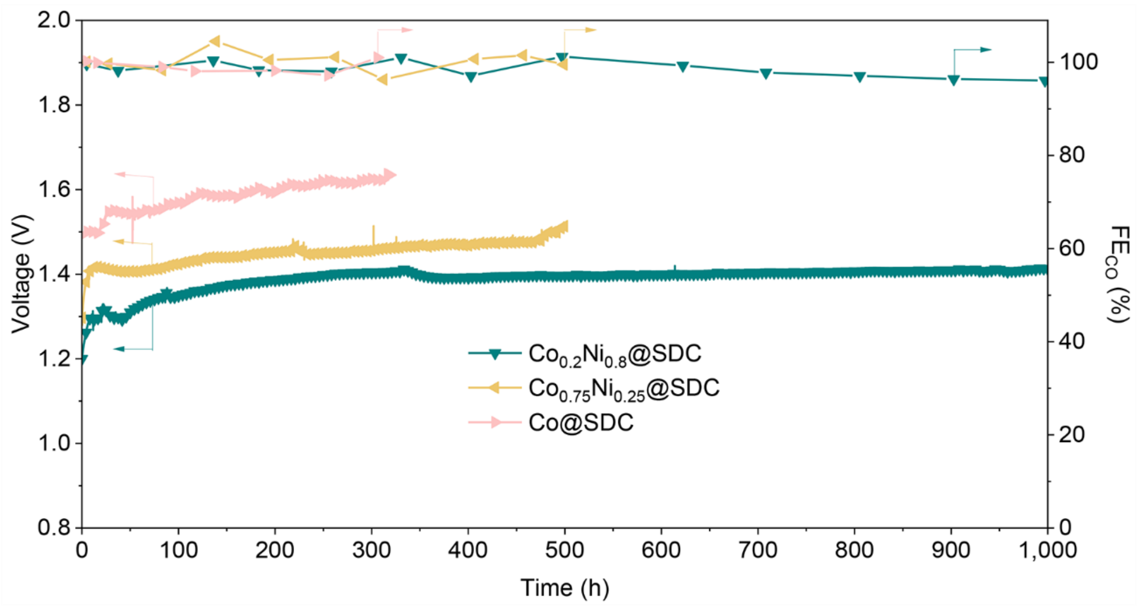

Supplementary Fig. 8 | Stability tests for $Co_{0.2}Ni_{0.8}$@SDC, $Co_{0.75}Ni_{0.25}$@SDC and Co@SDC at 1.0 A/cm^2.

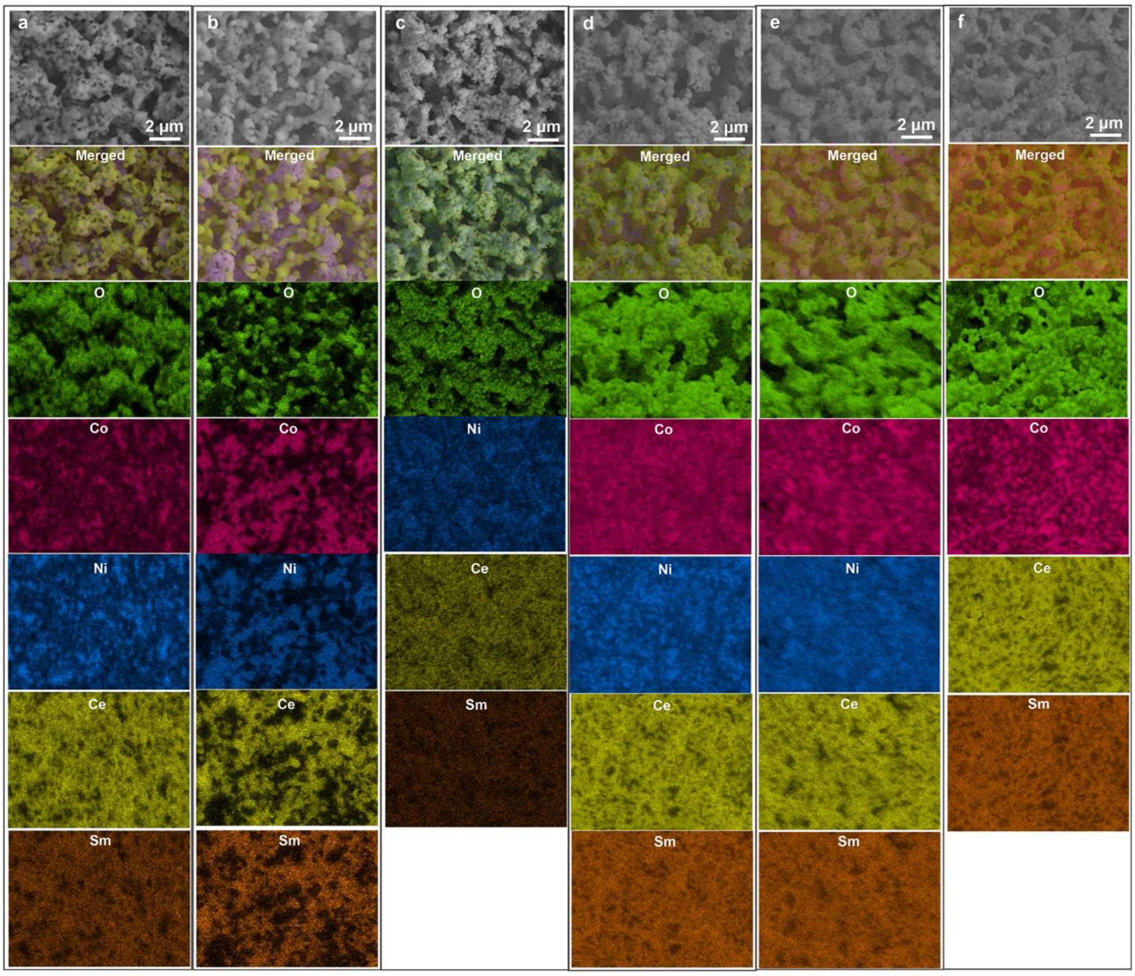

Supplementary Fig. 9 | SEM images and the corresponding EDX mappings of fresh samples. **a**, $Co_{0.5}Ni_{0.5}$@SDC. **b**, $Co_{0.5}Ni_{0.5}$-SDC. **c**, Ni@SDC. **d**, $Co_{0.2}Ni_{0.8}$@SDC. **e**, $Co_{0.75}Ni_{0.25}$@SDC. **f**, Co@SDC.

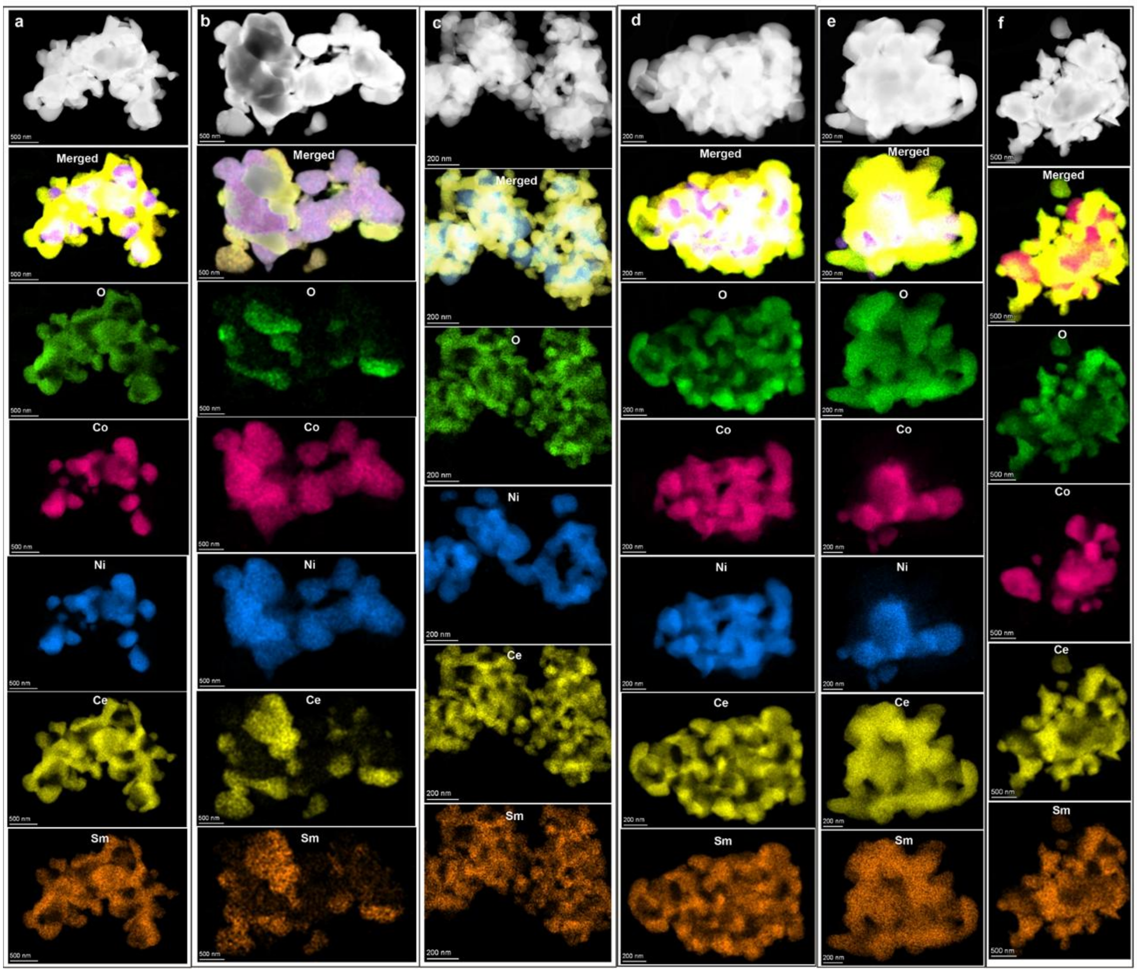

Supplementary Fig. 10 | TEM images and the corresponding EDX mappings of fresh samples. **a**, $Co_{0.5}Ni_{0.5}$@SDC. **b**, $Co_{0.5}Ni_{0.5}$-SDC. **c**, Ni@SDC. **d**, $Co_{0.2}Ni_{0.8}$@SDC. **e**, $Co_{0.75}Ni_{0.25}$@SDC. **f**, Co@SDC.

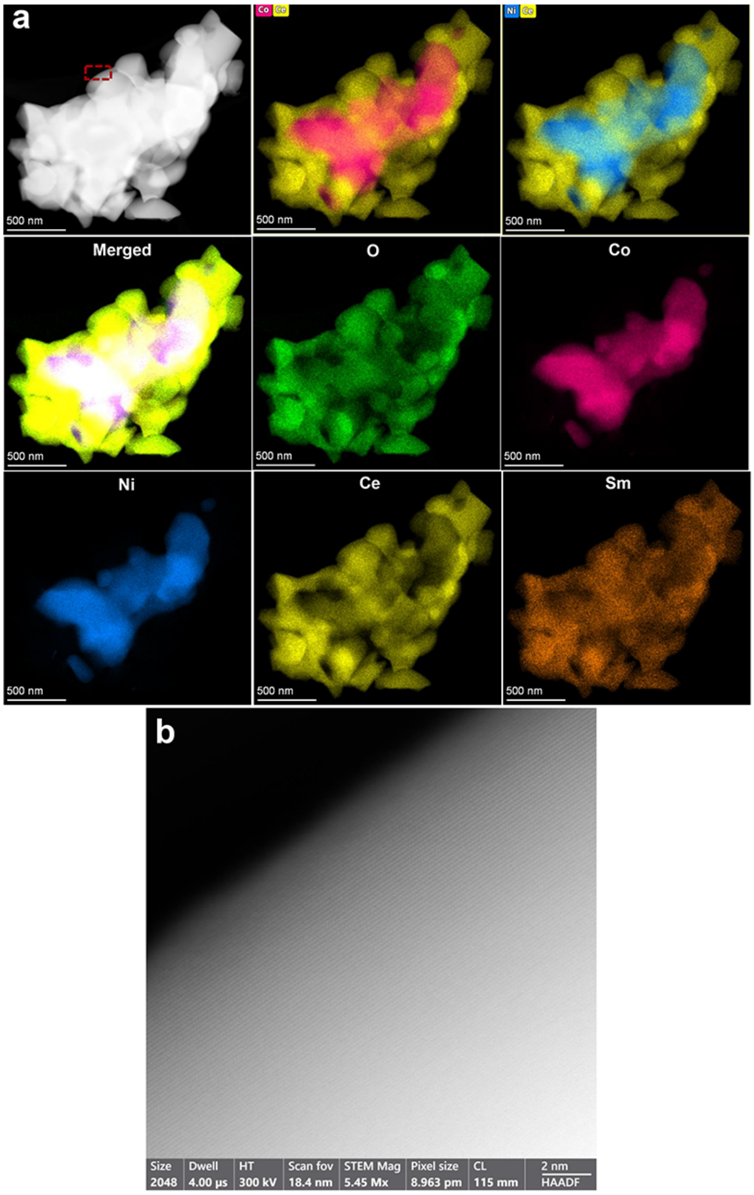

Supplementary Fig. 11 | Spherical aberration corrected TEM images of $Co_{0.5}Ni_{0.5}$@SDC. **a**, EDX mappings. **b**, High-angle annular dark-field scanning TEM (HAADF-STEM) for the red rectangle region in **a**.

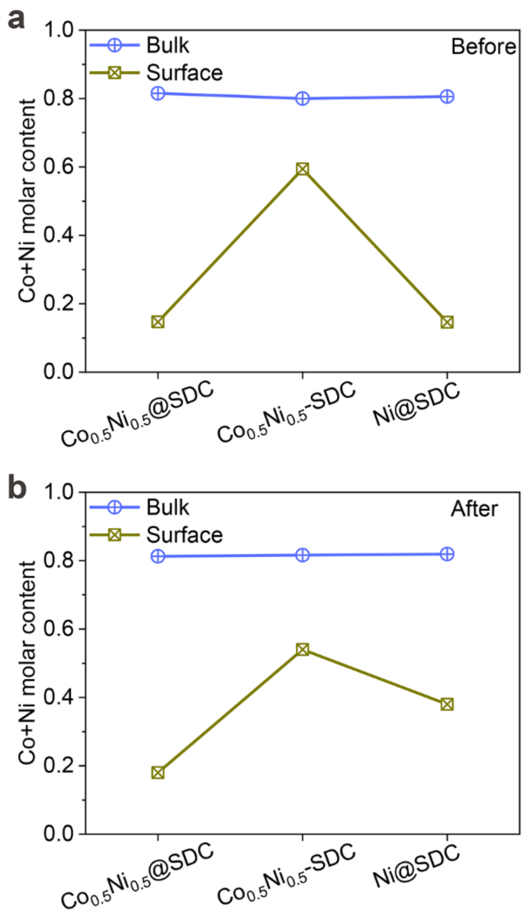

Supplementary Fig. 12 | Surface and bulk contents of Co and Ni. a, Fresh samples. **b**, Post-stability samples. The Co+Ni content was calculated by the molar ratio of Co+Ni and Co+Ni+Ce+Sm.

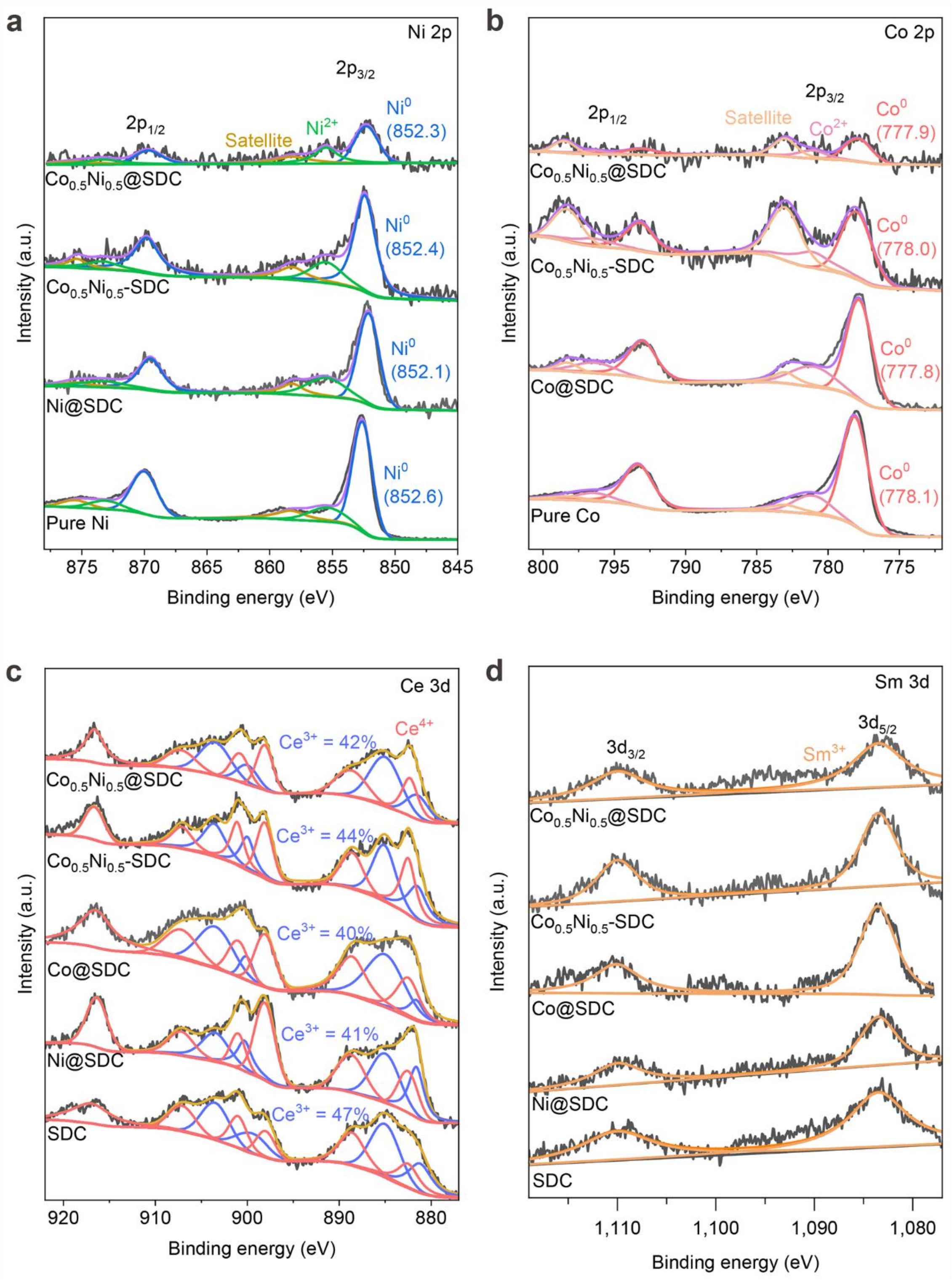

Supplementary Fig. 13 | Quasi in-situ XPS spectra of fresh samples. a, Ni 2p spectra. **b**, Co 2p spectra. **c**, Ce 3d spectra. **d**, Sm 3d spectra. The Ce^{3+} content was calculated by the ratio of Ce^{3+} peak areas and Ce^{3+}+Ce^{4+} peak areas.

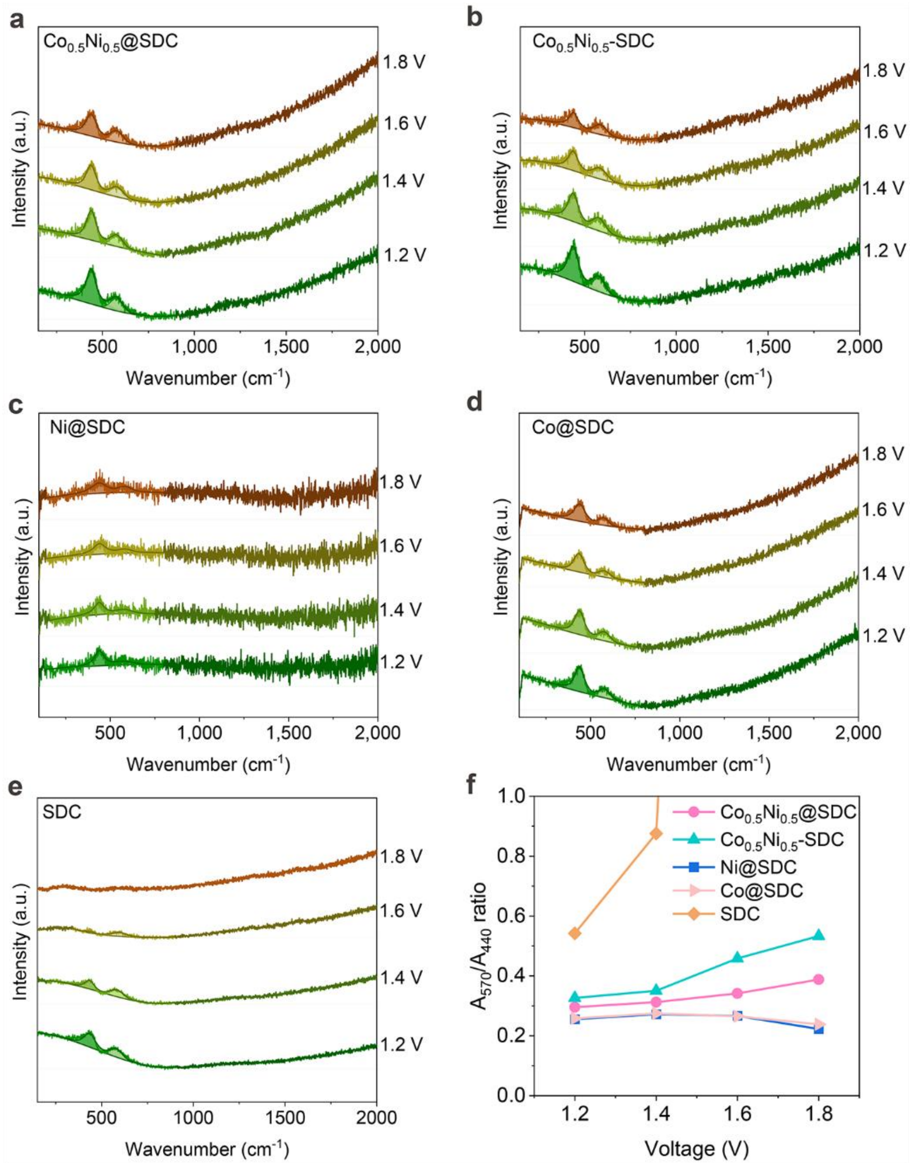

Supplementary Fig. 14 | Operando Raman spectra of different samples. a, $Co_{0.5}Ni_{0.5}$@SDC. **b**, $Co_{0.5}Ni_{0.5}$-SDC. **c**, Ni@SDC. **d**, Co@SDC. **e**, SDC. **f,** Comparison of A_{570}/A_{440} ratio on different samples. The Raman bands observed at ~570 cm^{-1} and ~440 cm^{-1} correspond to the oxygen vacancy and F_{2g} vibration mode of pure CeO_2, respectively[9]. Thus, the relative peak intensity ratio between these two bands (A_{570}/A_{440}) serves as a measure of the oxygen vacancy concentration of in CeO_2 (Ref. 9). The presence of oxygen vacancies in SDC arises from the influence of Sm^{3+} and Ce^{3+}. Given that the Sm^{3+} content remains constant across all samples, the concentration of oxygen vacancies directly mirrors the surface Ce^{3+} content.

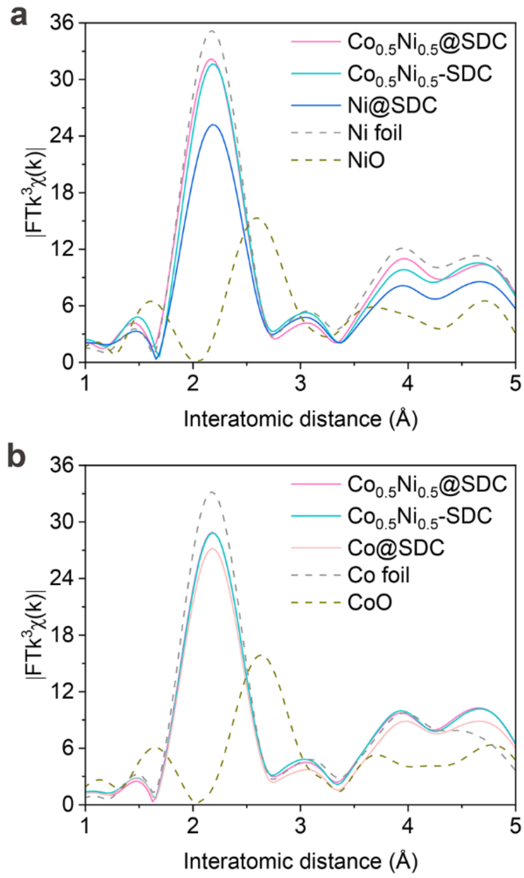

Supplementary Fig. 15 | EXAFS spectra of fresh samples. a, R-space for Ni K-edge. **b**, R-space for Co K-edge.

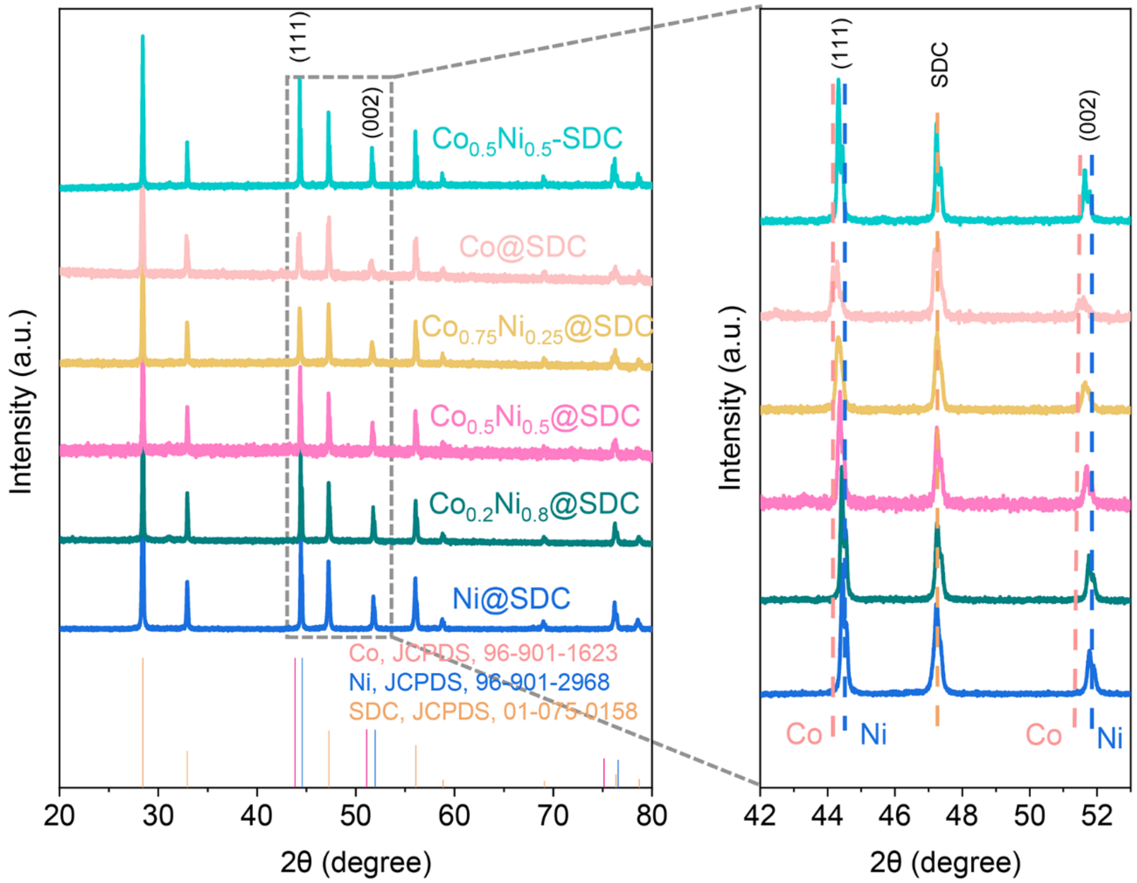

Supplementary Fig. 16 | XRD patterns after stability tests.

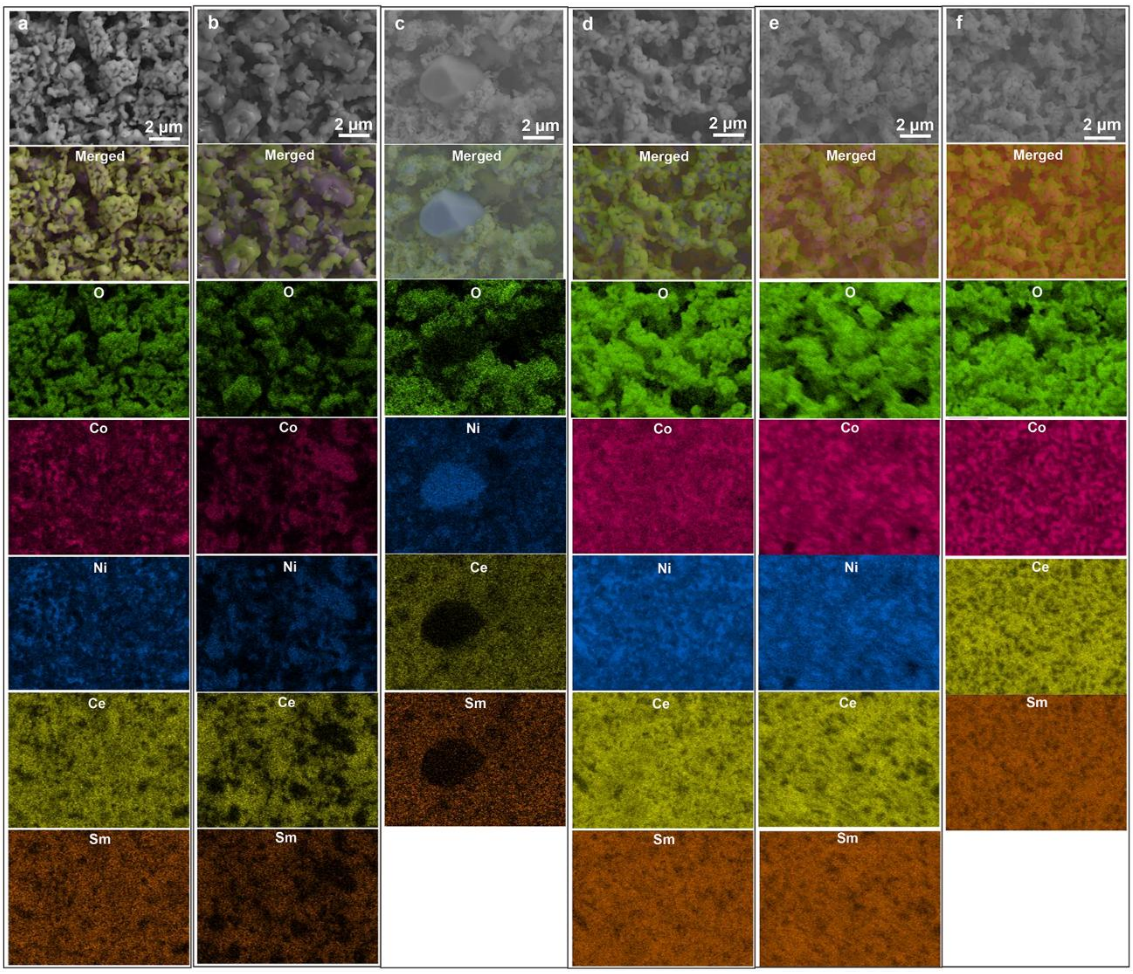

Supplementary Fig. 17 | SEM images and the corresponding EDX mappings after stability tests. **a**, $Co_{0.5}Ni_{0.5}$@SDC. **b**, $Co_{0.5}Ni_{0.5}$-SDC. **c**, Ni@SDC. **d**, $Co_{0.2}Ni_{0.8}$@SDC. **e**, $Co_{0.75}Ni_{0.25}$@SDC. **f**, Co@SDC.

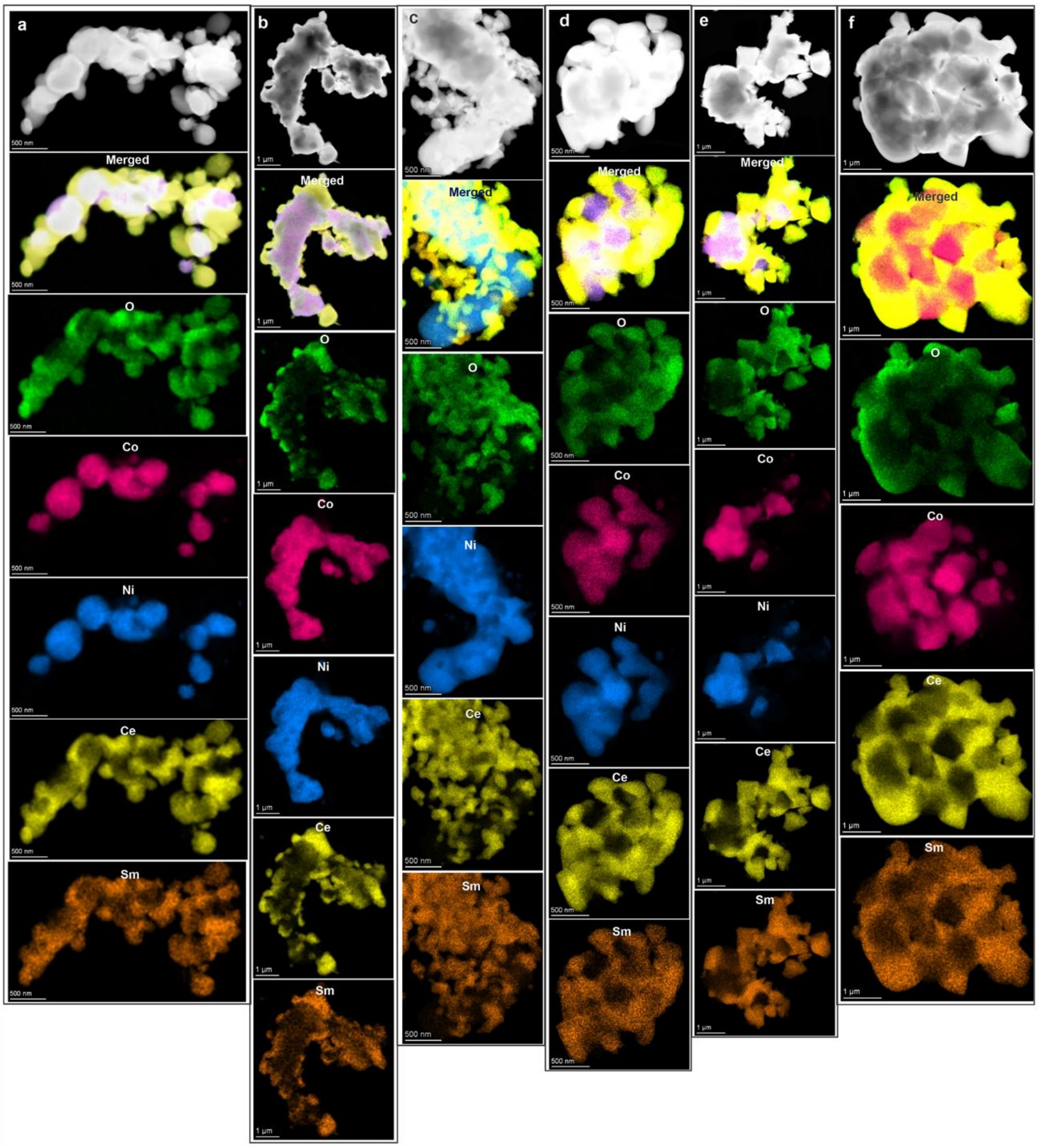

Supplementary Fig. 18 | TEM images and the corresponding EDX mappings after stability tests. a, $Co_{0.5}Ni_{0.5}$@SDC. **b**, $Co_{0.5}Ni_{0.5}$-SDC. **c**, Ni@SDC. **d**, $Co_{0.2}Ni_{0.8}$@SDC. **e**, $Co_{0.75}Ni_{0.25}$@SDC. **f**, Co@SDC.

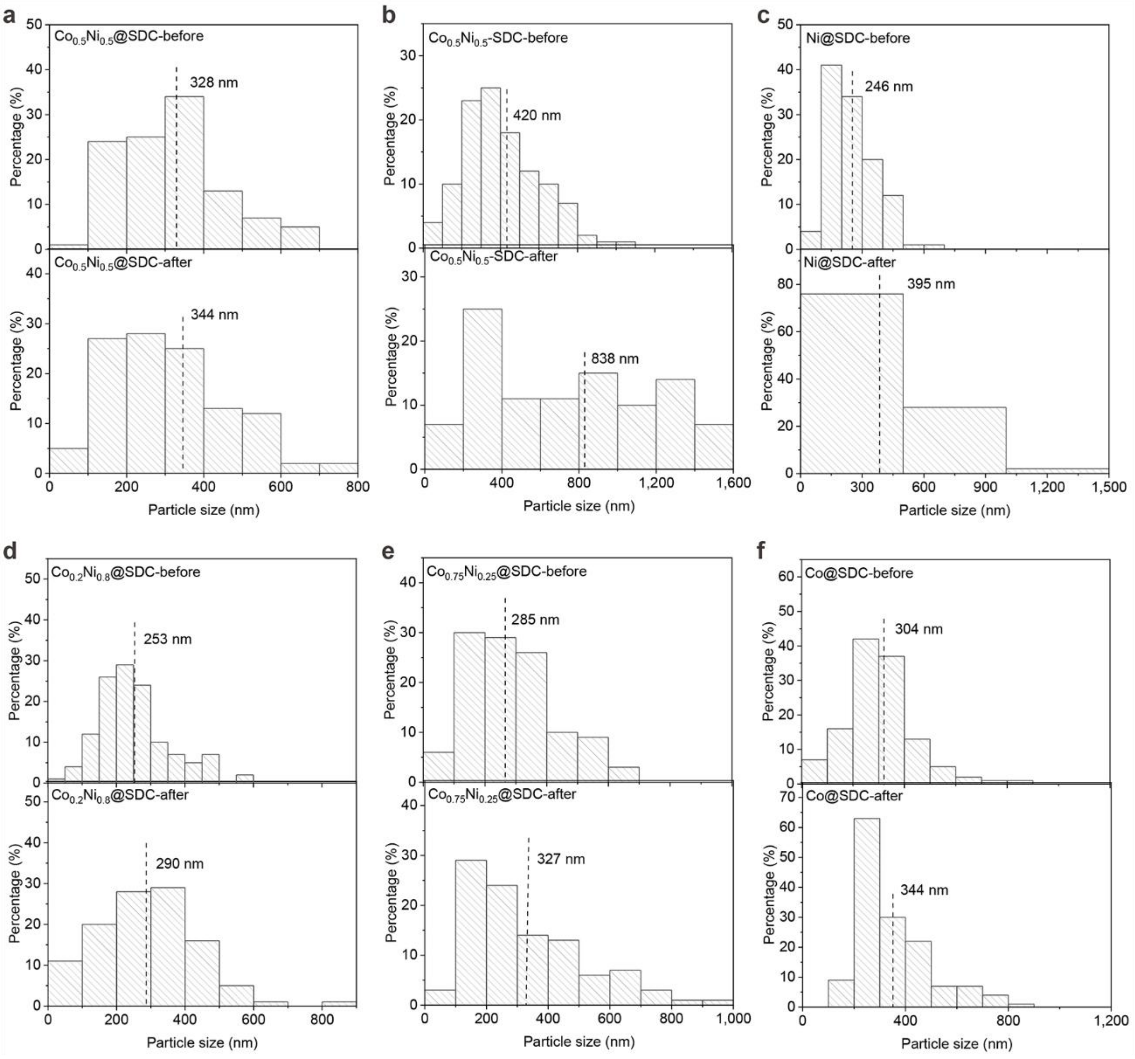

Supplementary Fig. 19 | Metal particle size distributions before and after stability tests. **a**, $Co_{0.5}Ni_{0.5}$@SDC. **b**, $Co_{0.5}Ni_{0.5}$-SDC. **c**, Ni@SDC. **d**, $Co_{0.2}Ni_{0.8}$@SDC. **e**, $Co_{0.75}Ni_{0.25}$@SDC. **f**, Co@SDC.

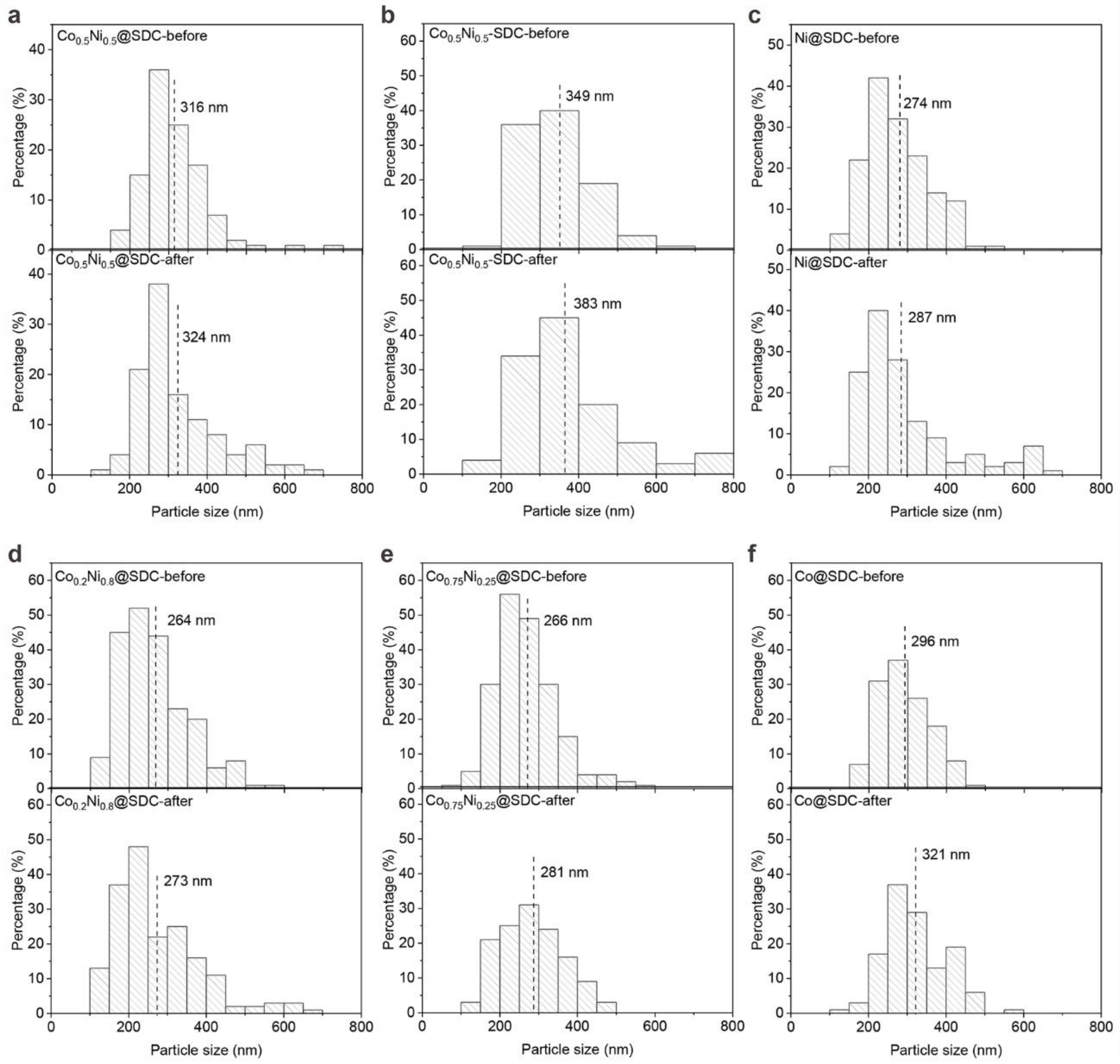

Supplementary Fig. 20 | SDC particle size distributions before and after stability tests. a, $Co_{0.5}Ni_{0.5}$@SDC. **b**, $Co_{0.5}Ni_{0.5}$-SDC. **c**, Ni@SDC. **d**, $Co_{0.2}Ni_{0.8}$@SDC. **e**, $Co_{0.75}Ni_{0.25}$@SDC. **f**, Co@SDC.

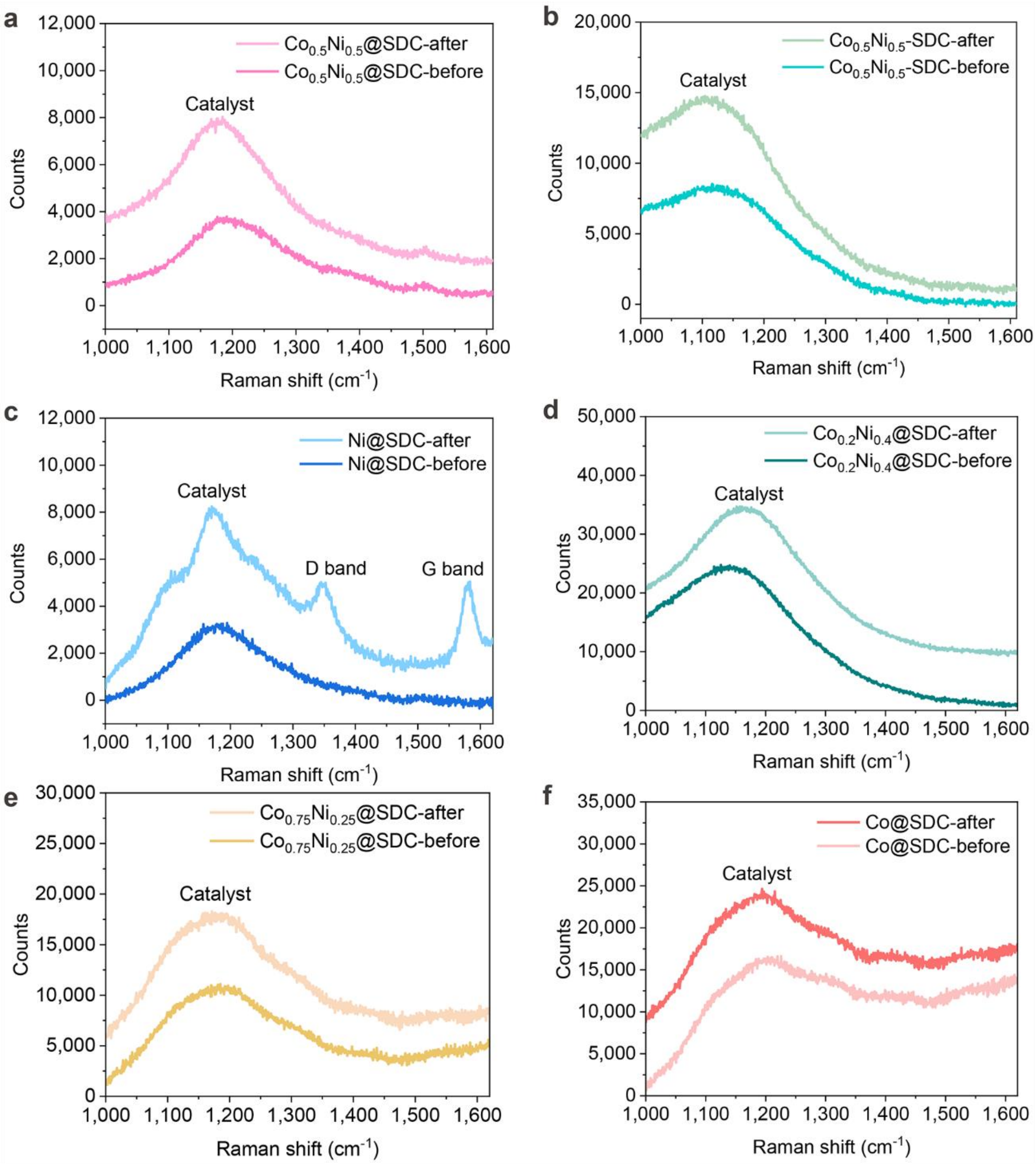

Supplementary Fig. 21 | Ex situ Raman spectra of different catalysts before and after stability tests. a, $Co_{0.5}Ni_{0.5}$@SDC. **b**, $Co_{0.5}Ni_{0.5}$-SDC. **c**, Ni@SDC. **d**, $Co_{0.2}Ni_{0.8}$@SDC. **e**, $Co_{0.75}Ni_{0.25}$@SDC. **f**, Co@SDC.

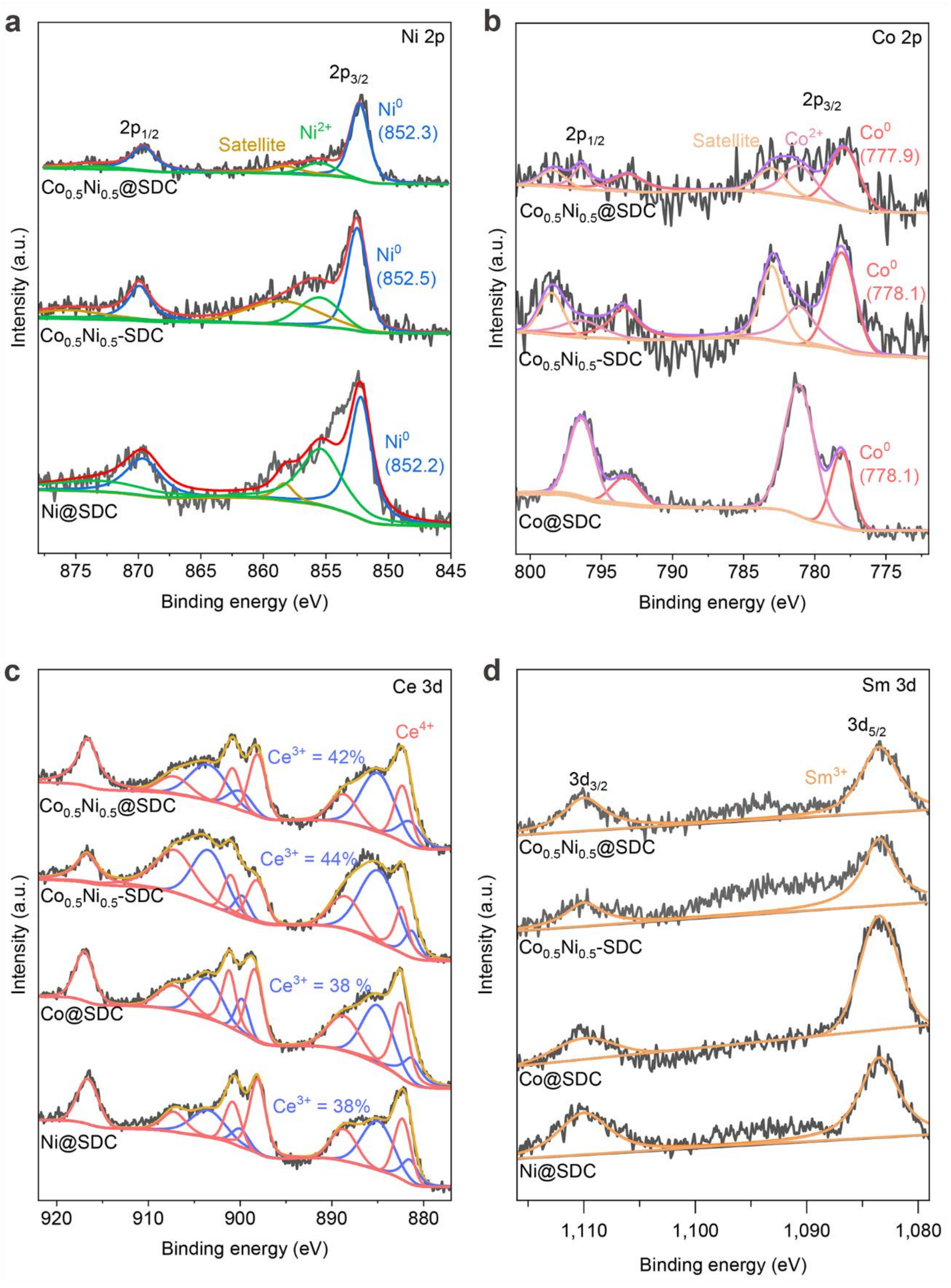

Supplementary Fig. 22 | XPS spectra after stability tests. a, Ni 2p spectra. **b**, Co 2p spectra. **c**, Ce 3d spectra. **d**, Sm 3d spectra.

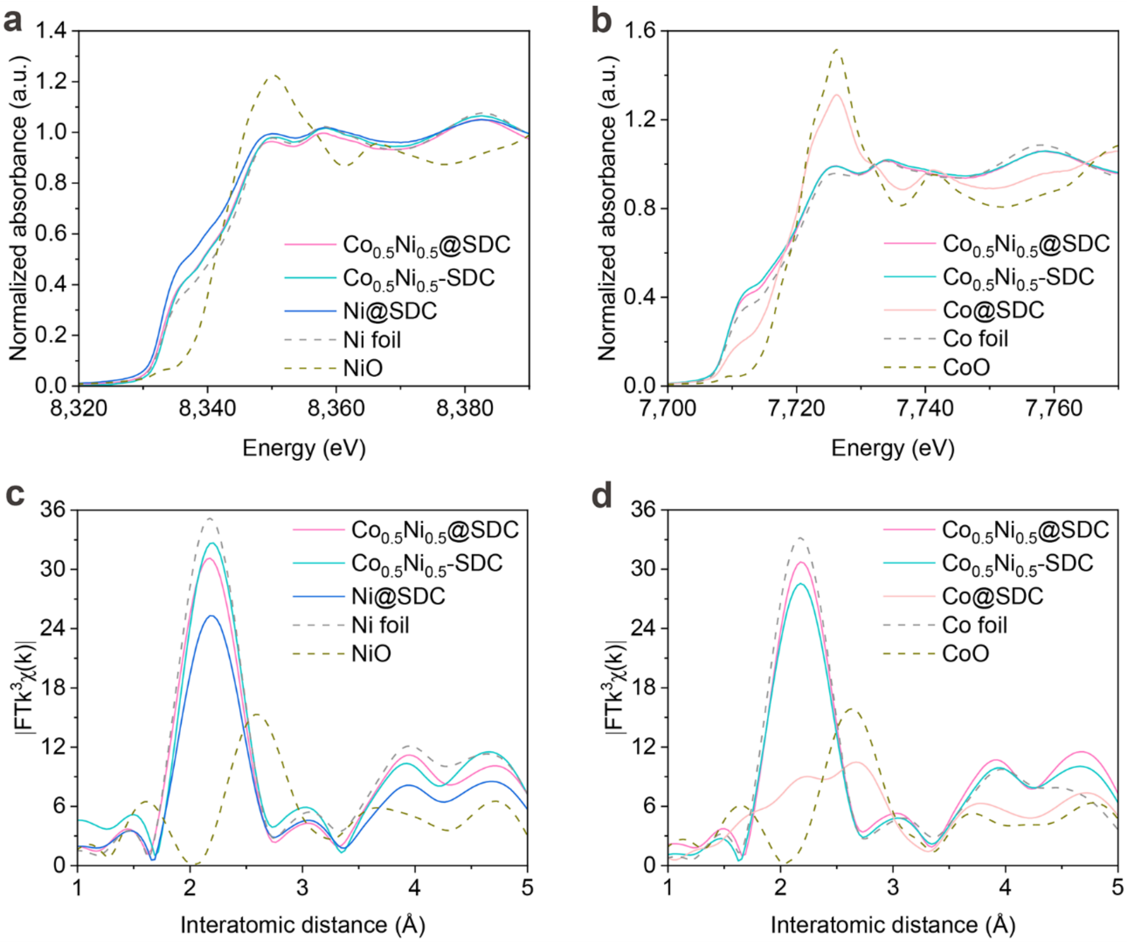

Supplementary Fig. 23 | XAS spectra after stability tests. a, Ni K-edge XANES spectra. **b**, Co K-edge XANES spectra. **c**, R-space for Ni K-edge EXAFS spectra. **d**, R-space for Co K-edge EXAFS spectra.

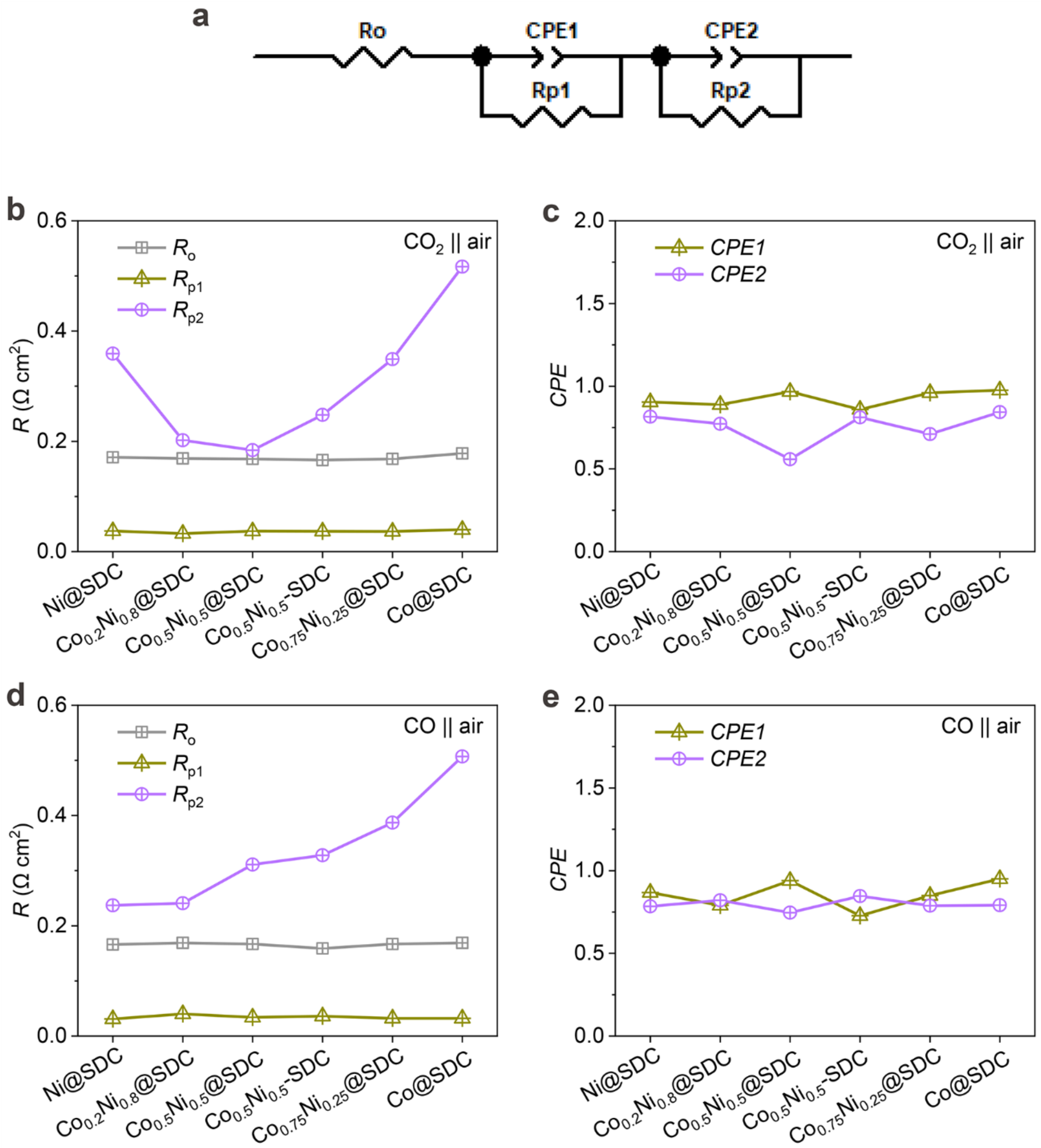

Supplementary Fig. 24 | Fitted EIS results for different samples. a, Equivalent circuit for fitting. **b**, Fitted resistances at 1.0 V under CO_2 atmosphere in cathode. **c**, Fitted capacitances at 1.0 V under CO_2 atmosphere in cathode. **d**, Fitted resistances at 0.9 V under CO atmosphere in anode. **e**, Fitted capacitances at 0.9 V under CO atmosphere in anode. It is established that R_0, R_{p1}, and R_{p2} correspond to the electrolyte resistance, air electrode polarization resistance, and fuel electrode polarization resistance, respectively[10]. *CPE1* and *CPE2* are the constant phase element for the air electrode and fuel electrode, respectively[10].

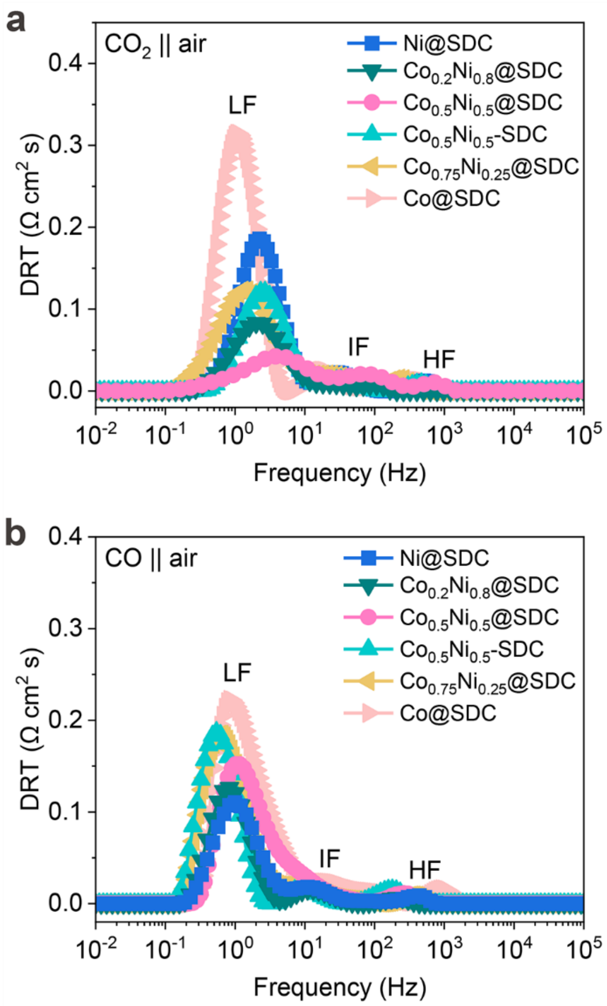

Supplementary Fig. 25 | DRT plots for different catalysts. a, DRT plots at 1.0 V under CO_2 atmosphere in cathode. **b**, DRT plots at 0.9 V under CO atmosphere in anode.

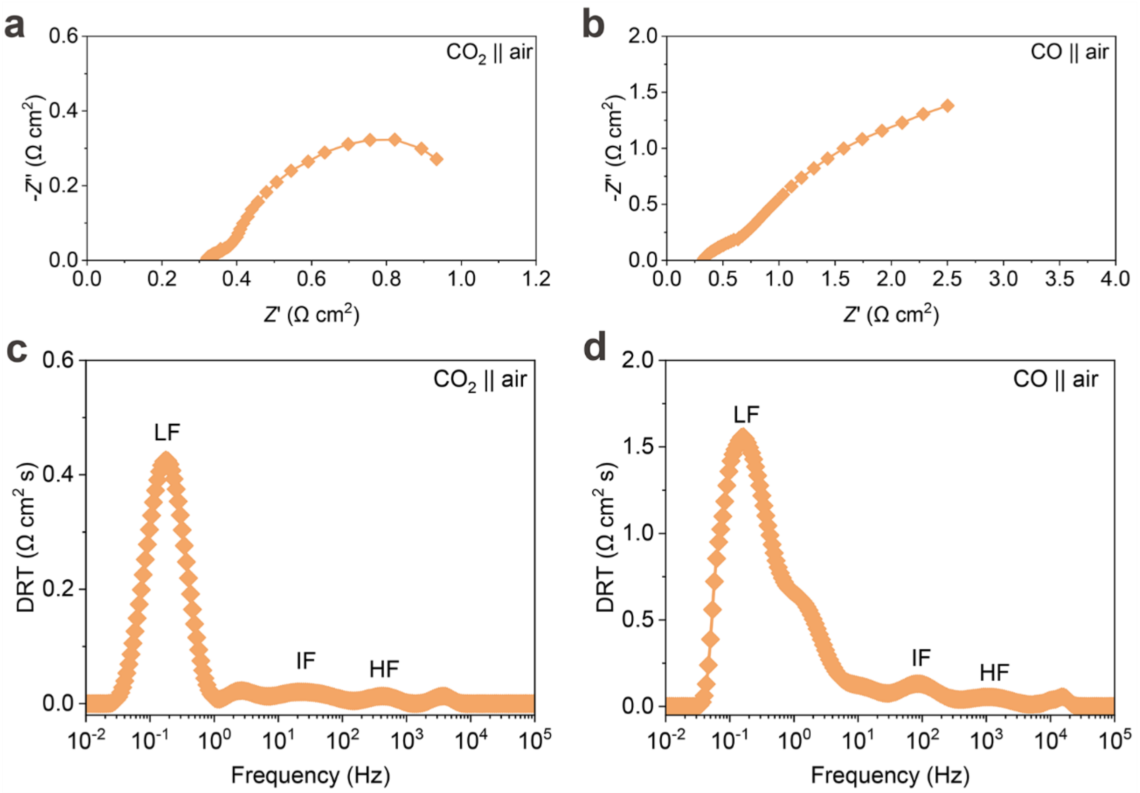

Supplementary Fig. 26 | Nyquist and DRT plots for SDC. a, Nyquist plots obtained from EIS at 1.0 V under CO_2 atmosphere in cathode. **b**, Nyquist plots obtained from EIS at 0.9 V under CO atmosphere in anode. **c**, DRT plots at 1.0 V under CO_2 atmosphere in cathode. **d**, DRT plots at 0.9 V under CO atmosphere in anode.

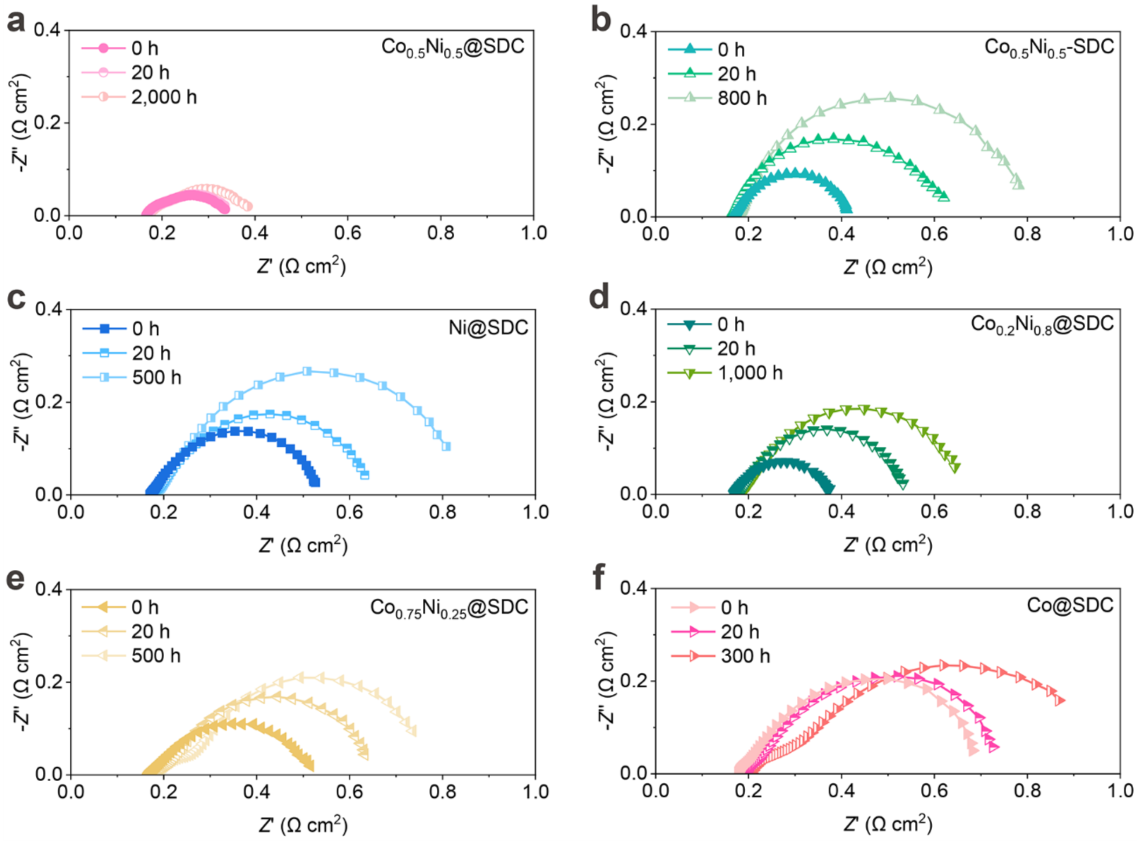

Supplementary Fig. 27 | Evolution of Nyquist plots during stability tests. a, $Co_{0.5}Ni_{0.5}$@SDC. **b**, $Co_{0.5}Ni_{0.5}$-SDC. **c**, Ni@SDC. **d**, $Co_{0.2}Ni_{0.8}$@SDC. **e**, $Co_{0.75}Ni_{0.25}$@SDC. **f**, Co@SDC. Cathode: CO_2; anode: air; cell voltage: 1.0 V.

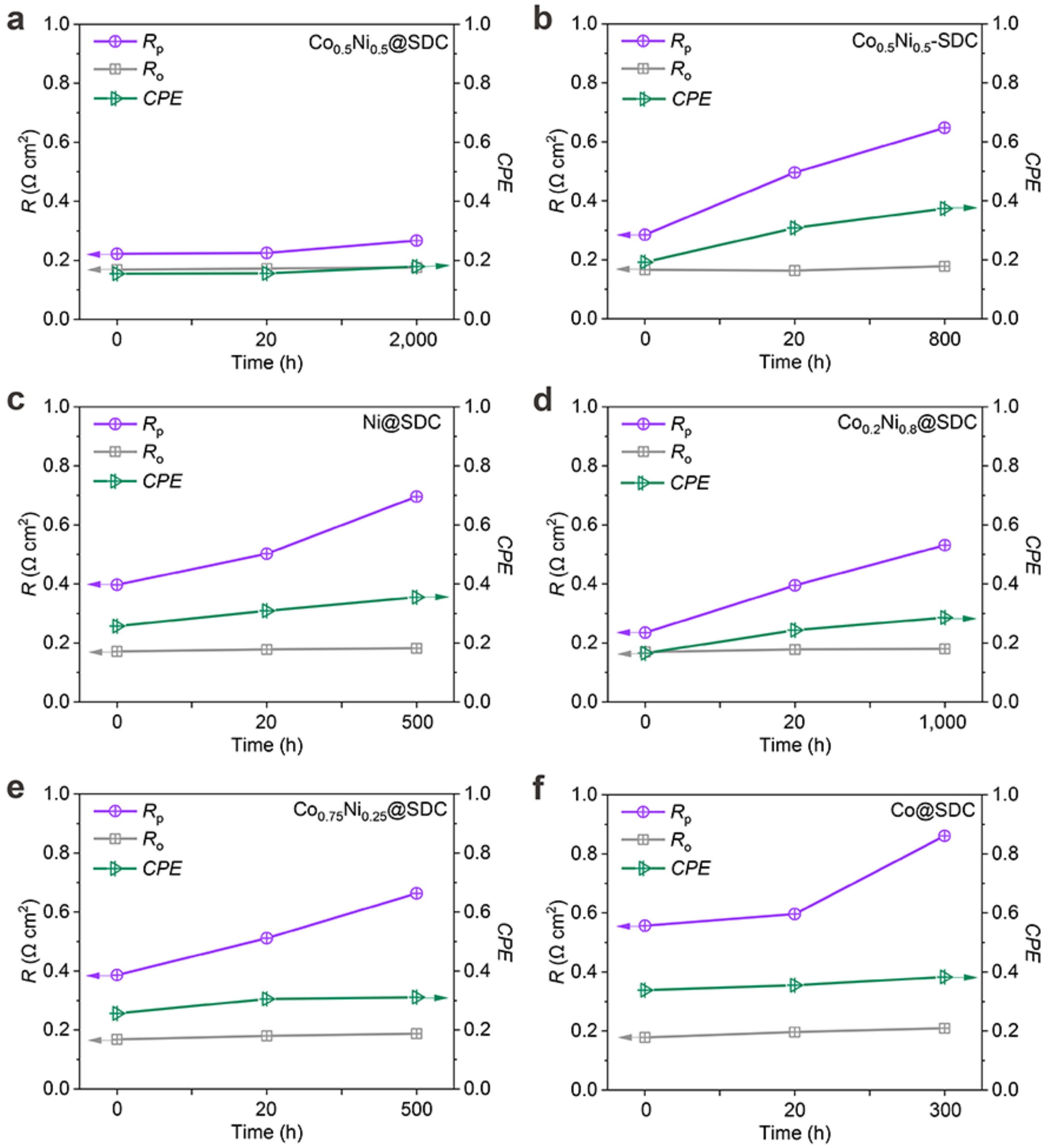

Supplementary Fig. 28 | Fitted EIS results during stability tests. a, $Co_{0.5}Ni_{0.5}$@SDC. **b**, $Co_{0.5}Ni_{0.5}$-SDC. **c**, Ni@SDC. **d**, $Co_{0.2}Ni_{0.8}$@SDC. **e**, $Co_{0.75}Ni_{0.25}$@SDC. **f**, Co@SDC. Cathode: CO_2; anode: air; cell voltage: 1.0 V. R_p is calculated by the equation of $R_p = R_{p1} + R_{p2}$. CPE is calculated by the equation of $1/CPE = 1/CPE1 + 1/CPE2$.

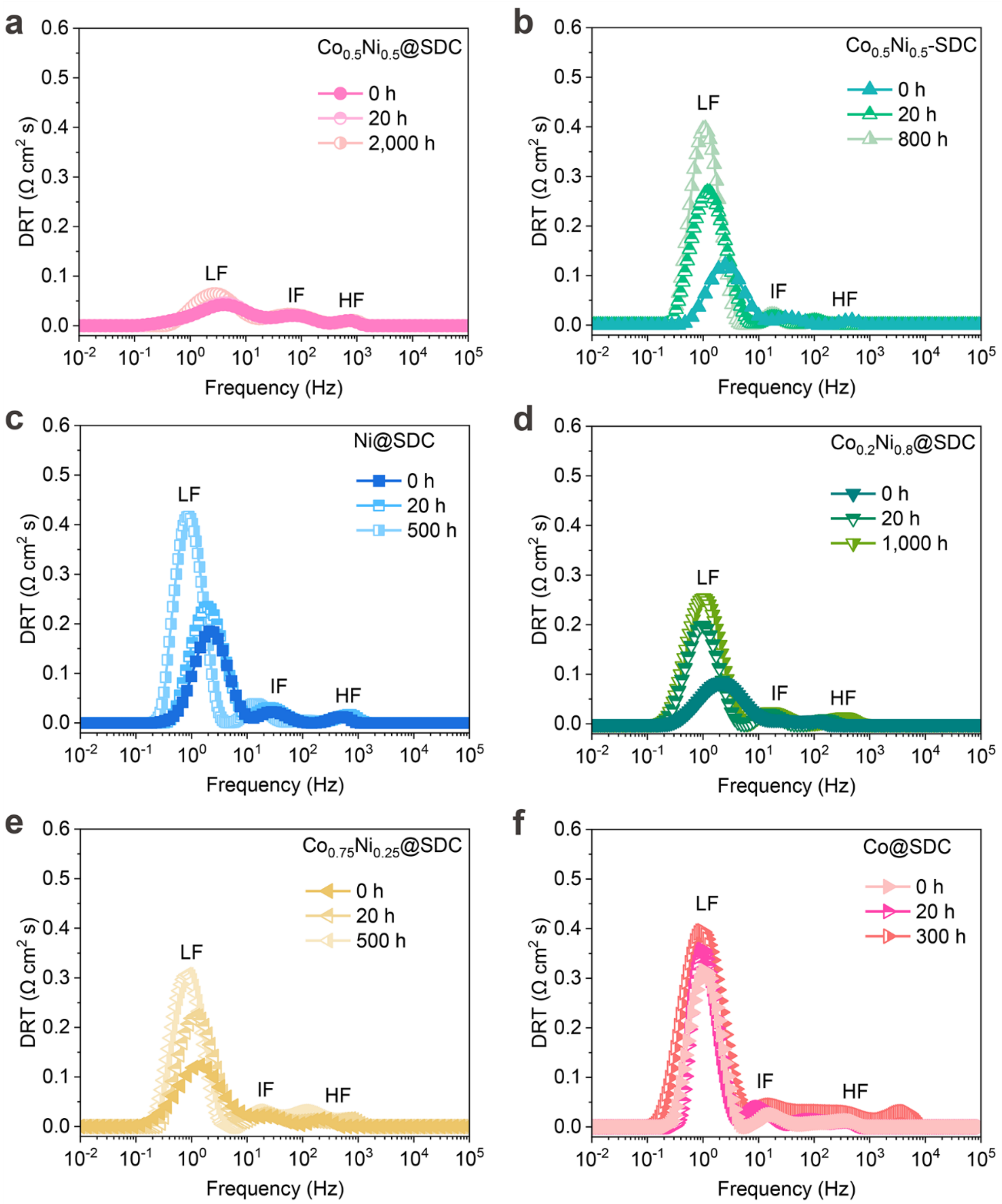

Supplementary Fig. 29 | Evolution of DRT plots during stability tests. a, $Co_{0.5}Ni_{0.5}$@SDC. **b**, $Co_{0.5}Ni_{0.5}$-SDC. **c**, Ni@SDC. **d**, $Co_{0.2}Ni_{0.8}$@SDC. **e**, $Co_{0.75}Ni_{0.25}$@SDC. **f**, Co@SDC. Cathode: CO_2; anode: air; cell voltage: 1.0 V.

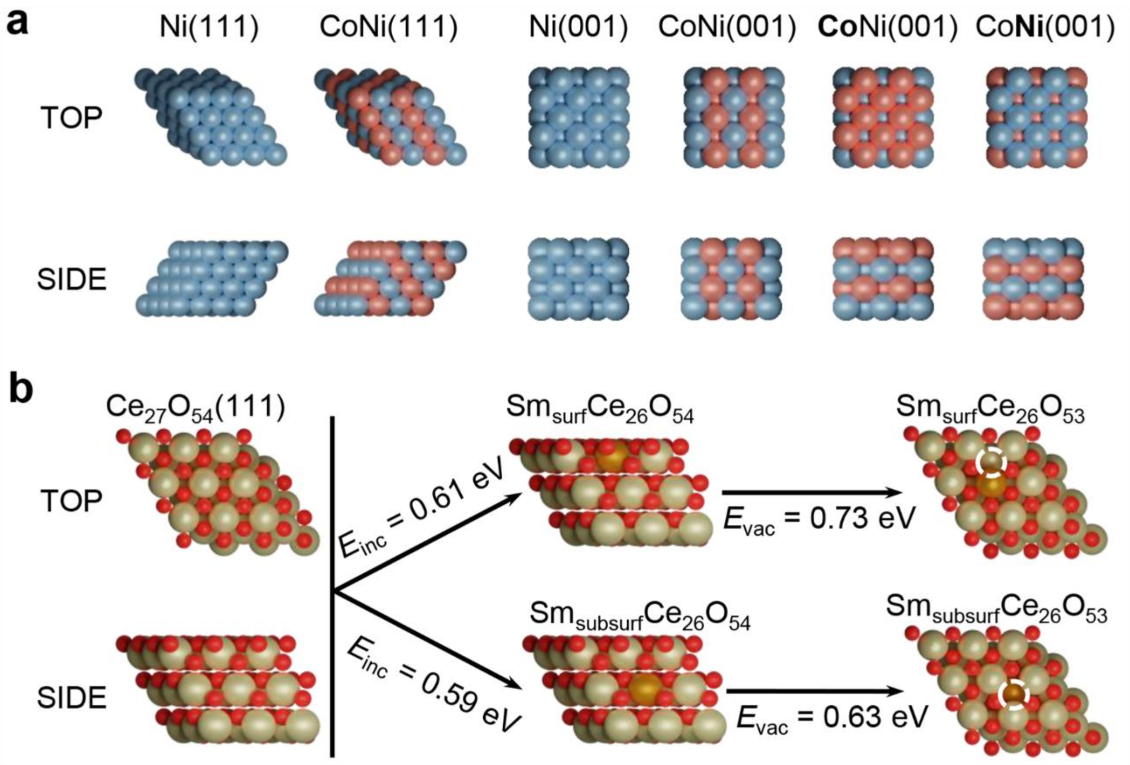

Supplementary Fig. 30 | Optimized structures for isolated metal and SDC models. a, Top and side views of different isolated metals. **b**, Top and side views of different isolated SDC. E_{inc} and E_{vac} were the computed energy for incorporating a Sm atom into CeO_2 and the formation energy of an oxygen vacancy, respectively. The E_{inc} and E_{vac} values were obtained using a $p(3\times3)$ $CeO_2(111)$ slab, molecular oxygen, bulk fluorite CeO_2, and bulk cubic Sm_2O_3 as references. Color code: Ni (blue) and Co (light pink), Ce (pale yellow), Sm (orange), O (red), and oxygen vacancy (dotted white circle).

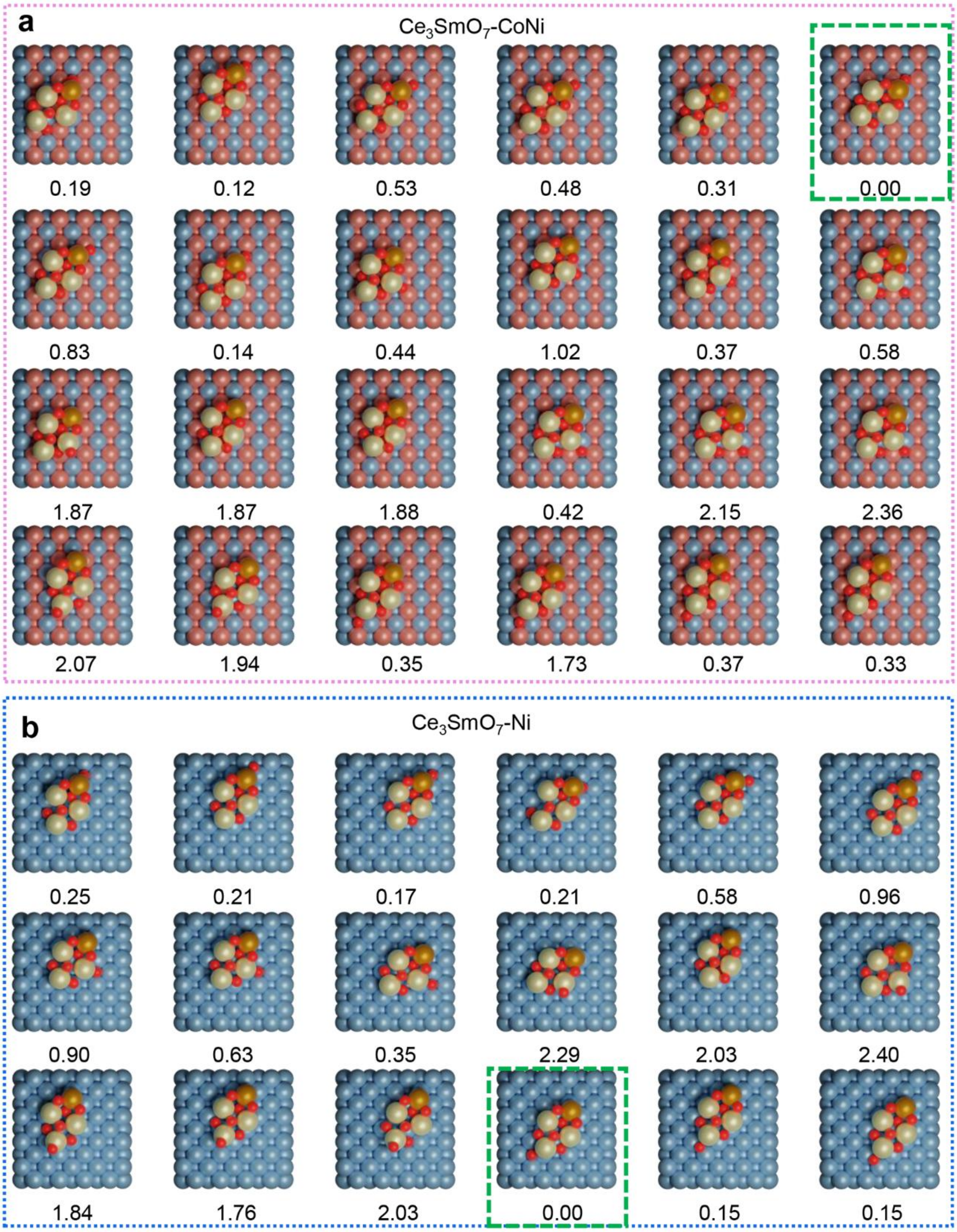

Supplementary Fig. 31 | Optimized structures for various metal-SDC models. Snapshots and relative energy (ΔE, eV) for various Ce_3SmO_7 clusters adsorbed on **a**, CoNi(001) and **b**, Ni(001) surfaces, with respect to the most stable configuration (framed by dotted green square). Color code: Ni (blue), Co (light pink), Ce (pale yellow), Sm (orange), and O (red).

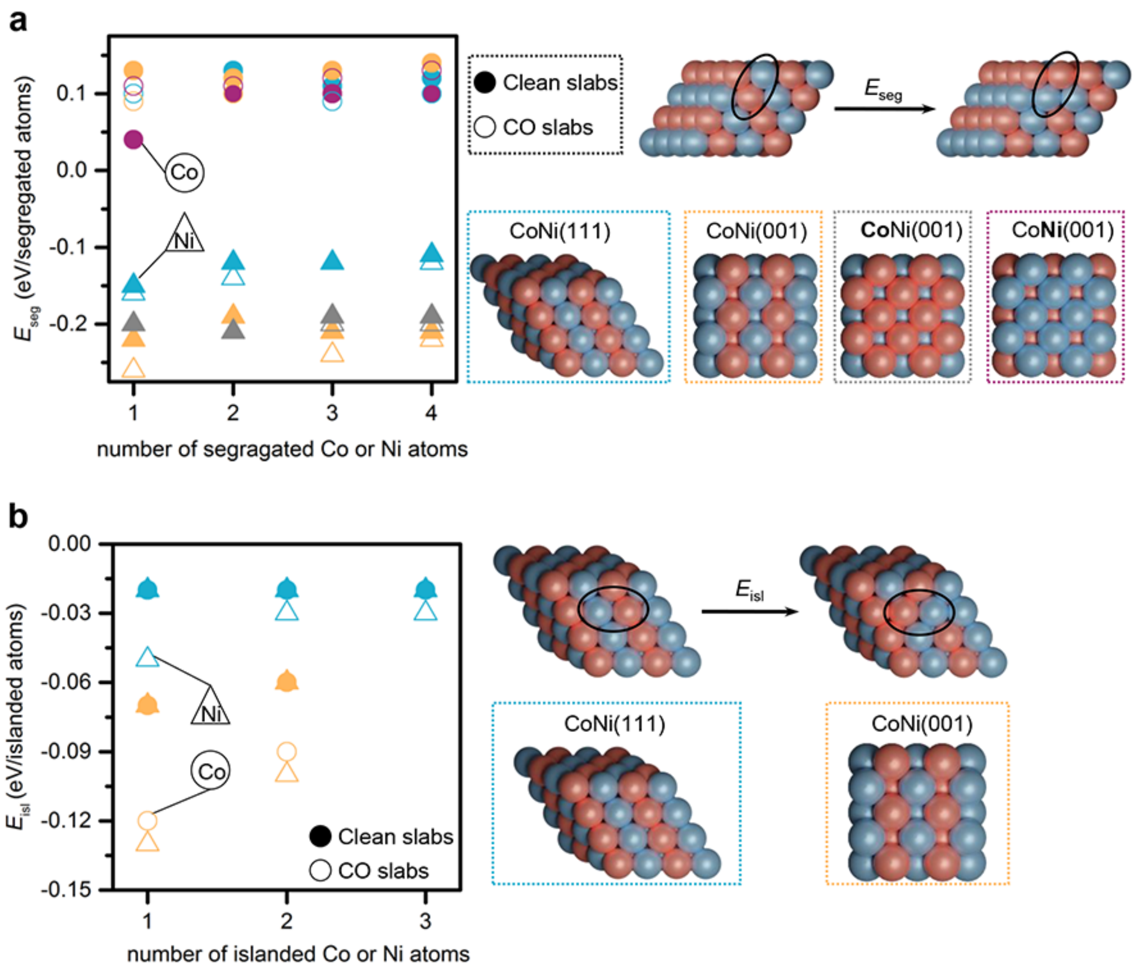

Supplementary Fig. 32 | Computed segregation energies (*E*$_{seg}$, eV/segregated atoms) and islanding energies (*E*$_{isl}$, eV/islanded atoms) for various Co-Ni alloys models with and without adsorbed CO. **a**, E_{seg}. E_{seg} is defined as the energy required to exchange a Ni (Co) atom of the subsurface with a Co (Ni) atom in the surface. A schematic representation of the segregation of a Co atom in the CoNi(111) surface was shown in the upper right. **b**, E_{isl}. E_{isl} is the energy needed to exchange a Ni atom with a Co atom in the surface. A schematic representation of the islanding of a Co atom in the CoNi(111) surface was shown in the upper right. The alloys models are framed with dotted lines in different colors, corresponding to the colors of the markers in the plot. Color code: Ni (blue), and Co (light pink).

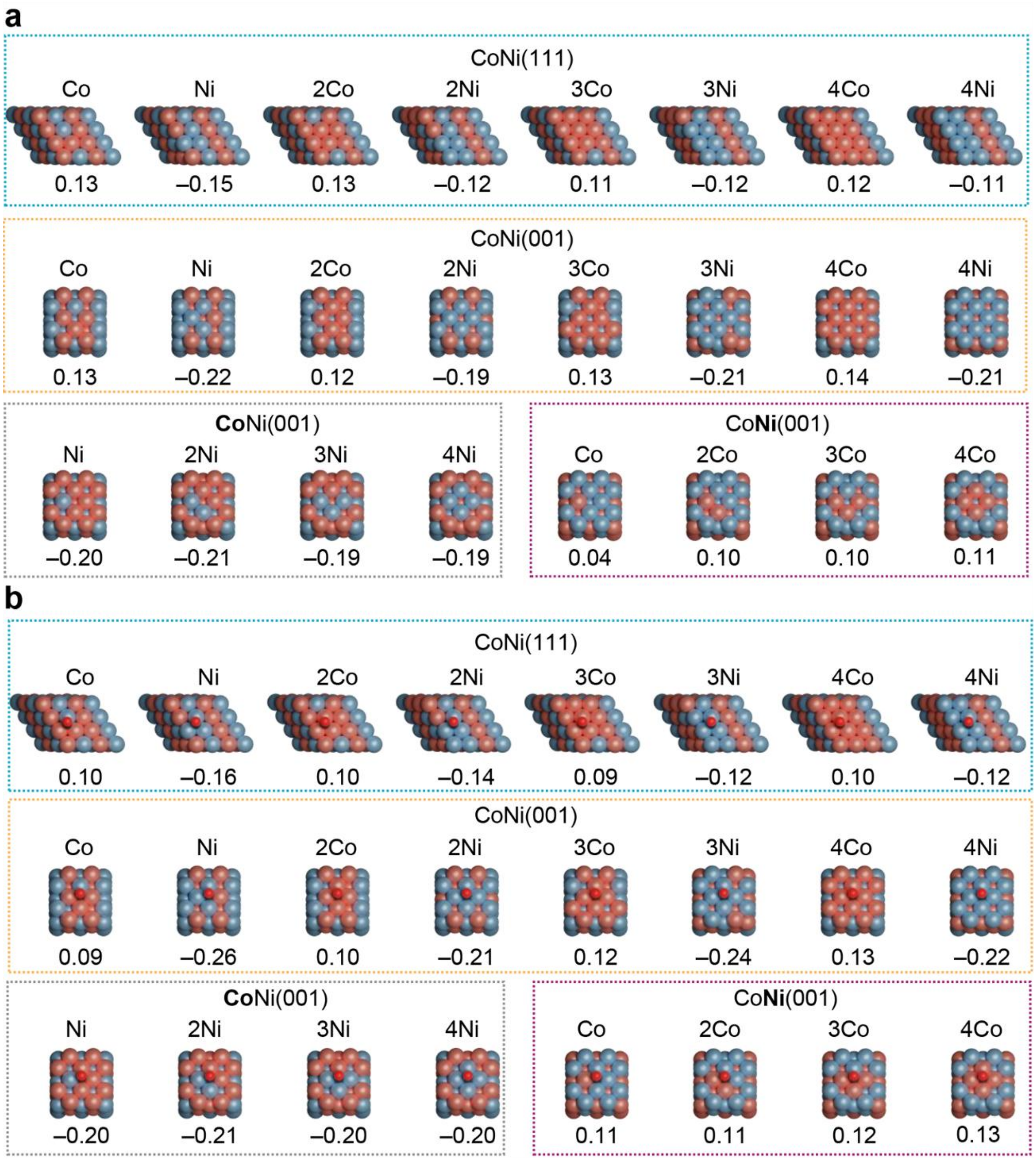

Supplementary Fig. 33 | DFT models employed to evaluate the segregation energy (E_{seg}, eV/segregated atoms) for various Co-Ni alloy models with and without adsorbed CO. a, Snapshots of bare Co-Ni alloys featuring different numbers of Co/Ni segregated atoms and their corresponding E_{seg} values. **b**, Snapshots of Co-Ni alloys with adsorbed CO, featuring different numbers of Co/Ni segregated atoms and their corresponding E_{seg} values. The alloys models were framed with dotted lines in different colors, corresponding to the colors of the markers in the plots of Supplementary Fig. 32. Color code: Ni (blue), Co (light pink), C (dark gray), and O (red).

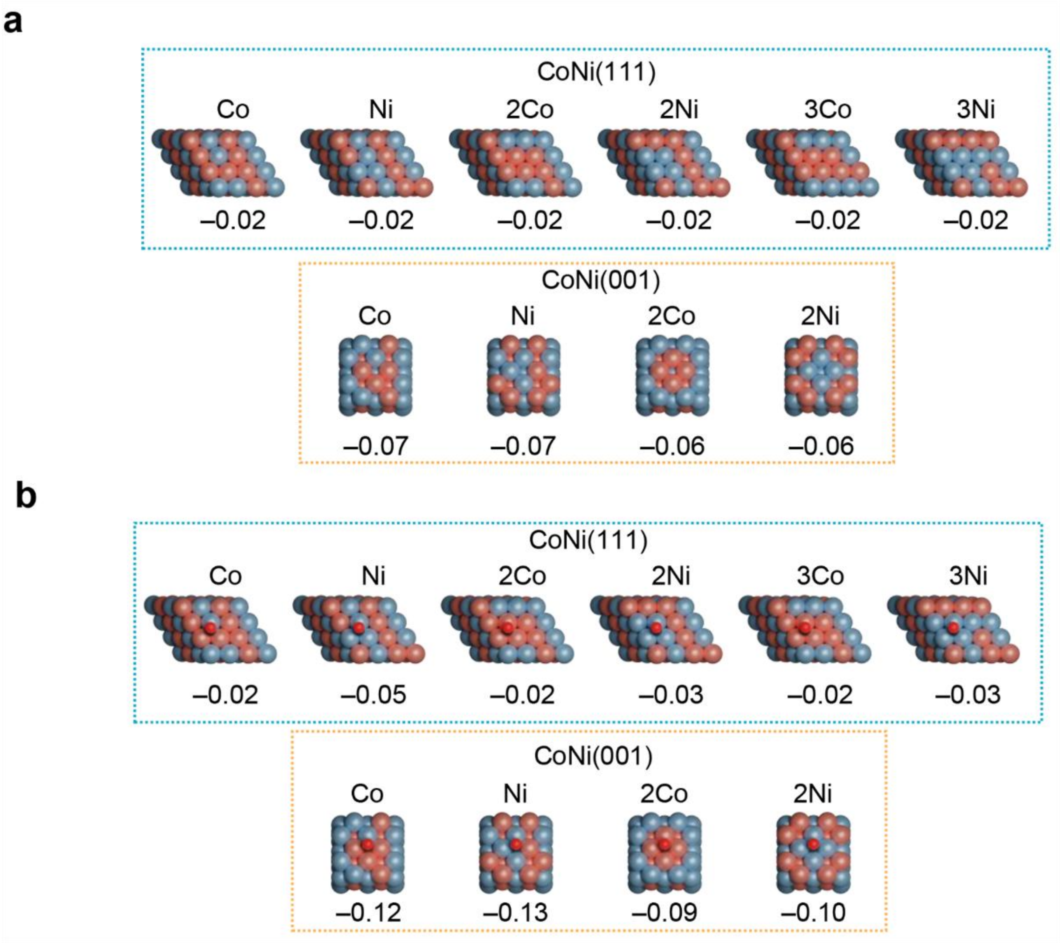

Supplementary Fig. 34 | DFT models employed to evaluate the islanding energy (E_{isl}, eV/islanded atoms) for various Co-Ni alloys models with and without adsorbed CO. a, Snapshots of bare Co-Ni alloys featuring different numbers of Co/Ni segregated atoms and their corresponding E_{isl} values. **b**, Snapshots of Co-Ni alloys with adsorbed CO, featuring different numbers of Co/Ni segregated atoms and their corresponding E_{isl} values. The alloys models were framed with dotted lines in different colors, corresponding to the colors of the markers in the plots of Supplementary Fig. 32. Color code: Ni (blue), Co (light pink)), C (dark gray), and O (red)..

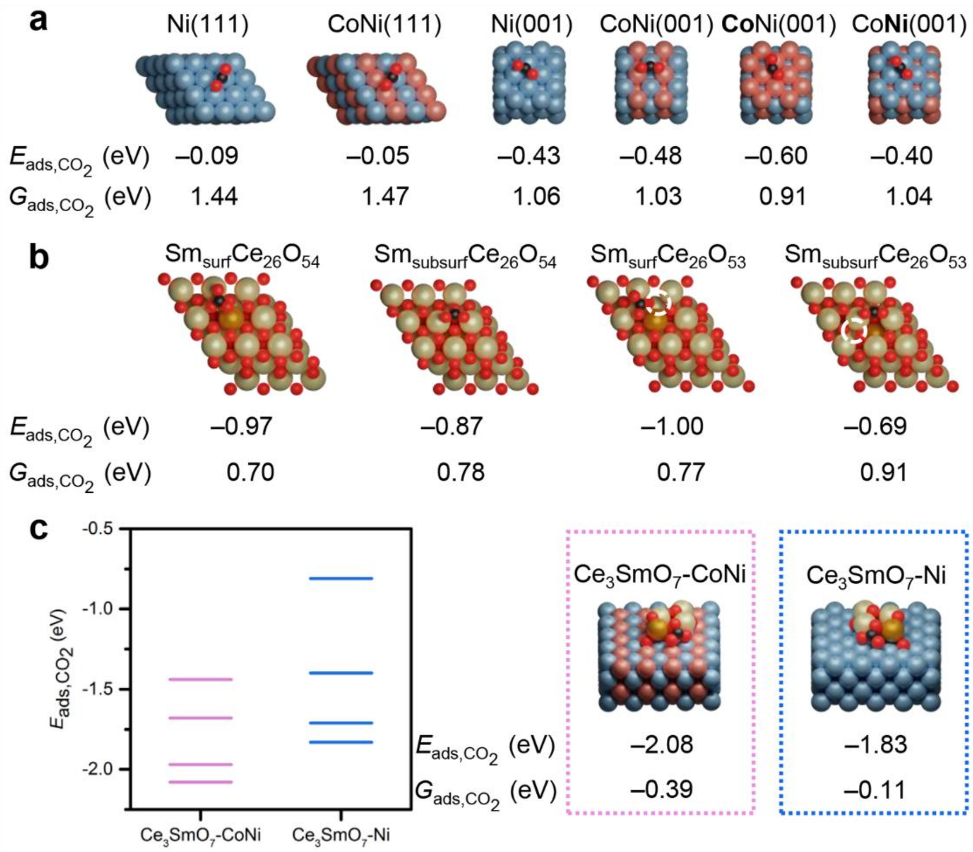

Supplementary Fig. 35 | Computed CO_2 adsorption energy (E_{ads,CO_2}, eV) and Gibbs free energy (G_{ads,CO_2}, eV) on different models. a, Isolated metal surfaces. **b**, Isolated SDC surfaces. Different models, incorporating Sm into surface (surf) and subsurface (subsurf) sites, along with the presence of oxygen vacancies, were employed to assess the adsorption of CO_2 on SDC. **c**, Metal-SDC surfaces. The structures associated with the most favoured CO_2 adsorptions were depicted in the right, while other structures were shown in Supplementary Fig. 36. The difference between E_{ads} and G_{ads} arises from significant entropic contributions at 800 ºC, particularly in adsorption and desorption processes. For gas-phase CO_2 and CO, both translational and rotational contributions were included (TS_{trans} = 1.73 eV and TS_{rot} = 0.61 eV for CO_2; TS_{trans} = 1.67 eV and TS_{rot} = 0.52 eV for CO, obtained from Gaussian 09)[11], while only vibrational contributions were considered for adsorbed CO_2 and CO. Color code: Ni (blue) and Co (light pink), Ce (pale yellow), Sm (orange), C (dark gray), O (red), and oxygen vacancy (dotted white circle).

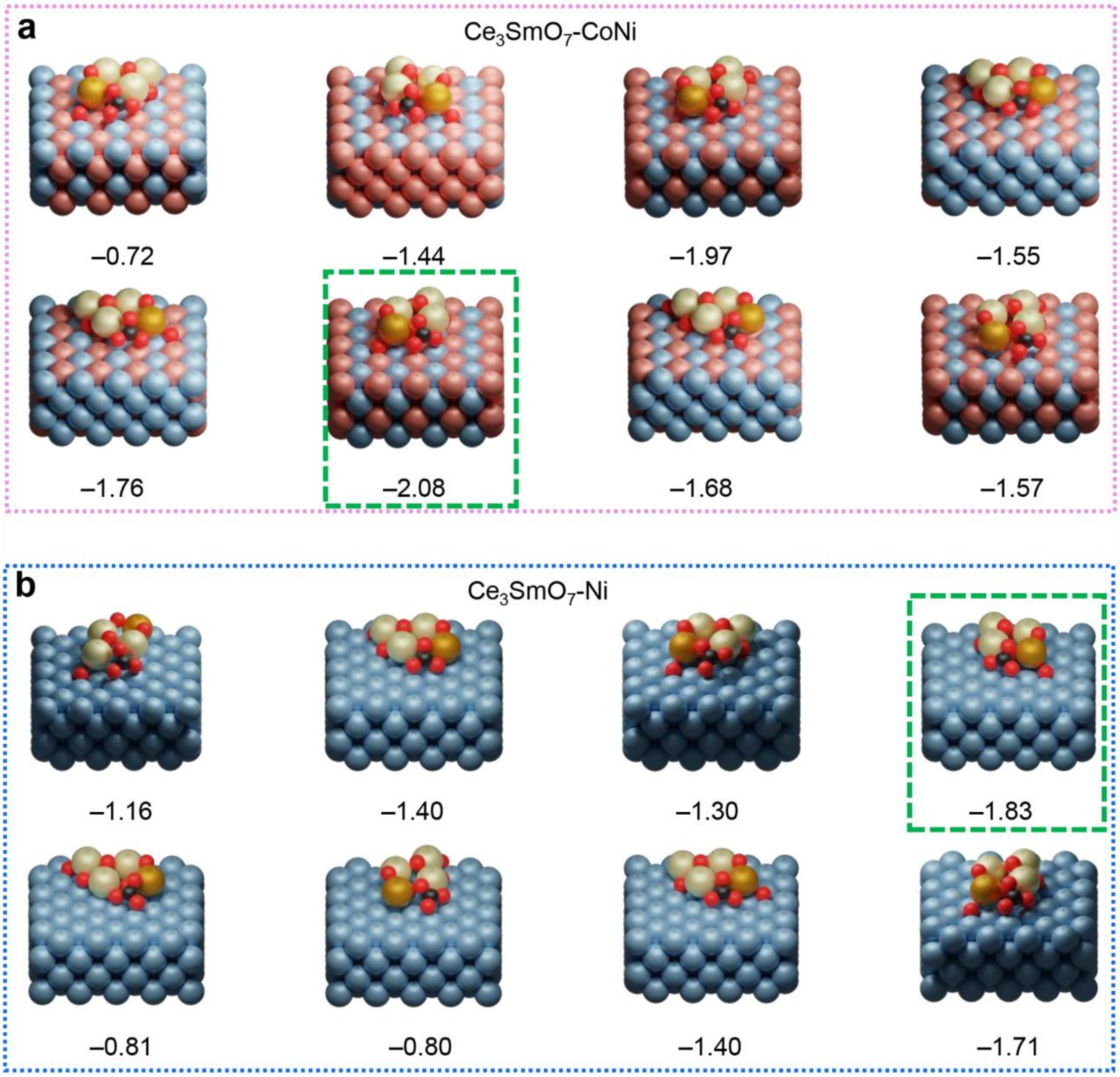

Supplementary Fig. 36 | Snapshots and E_{ads,CO_2} on the metal-SDC surfaces. Snapshots of different CO_2 adsorption configurations and their corresponding E_{ads,CO_2} values on **a**, Ce_3SmO_7-CoNi and **b**, Ce_3SmO_7-Ni models. The structure associated with the most favoured CO_2 adsorption was framed by dotted green square. Color code: Ni (blue), Co (light pink), Ce (pale yellow), Sm (orange), C (dark gray), and O (red).

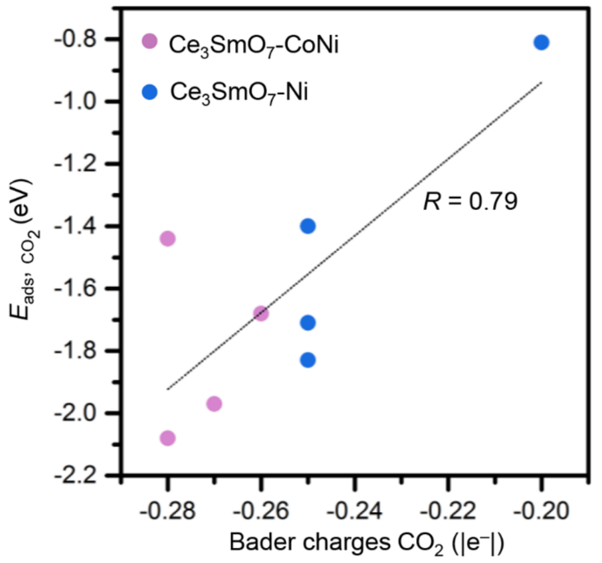

Supplementary Fig. 37 | The relationship between CO_2 adsorption energy and the degree of charge transfer. The total Bader charge of the adsorbed CO_2 molecule ranged from -0.20 to -0.30 $|e^-|$, compared to 0.03 $|e^-|$ for gas-phase CO_2, indicating a charge transfer from the catalyst surface to the CO_2 molecule. However, no strong correlation was observed between the CO_2 adsorption energy and the degree of charge transfer. This suggests that the enhanced CO_2 adsorption cannot be attributed solely to charge transfer. Other factors, such as the basicity of the oxygen site where the molecule is adsorbed and the acidity of the metal atoms interacting with the CO_2 molecule, also influence the interaction.

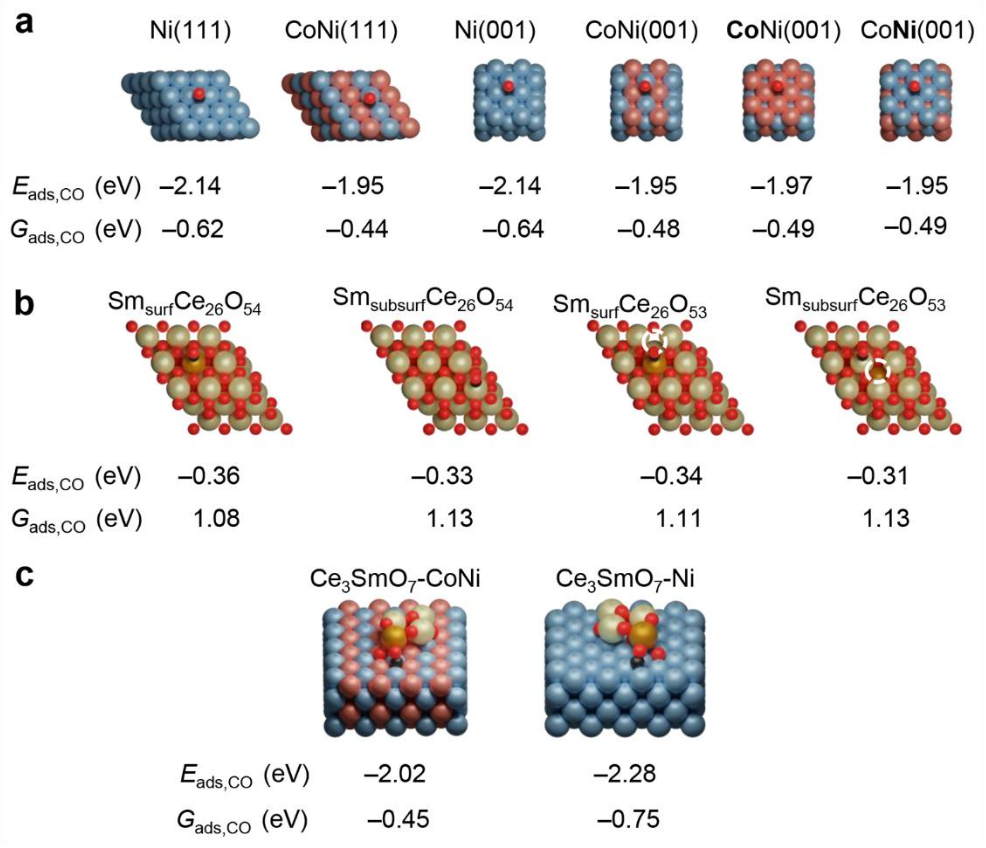

Supplementary Fig. 38 | Computed CO adsorption energy ($E_{ads,CO}$, eV) and Gibbs free energy ($G_{ads,CO}$, eV) on different models. a, Isolated metal surfaces. **b**, Isolated SDC surfaces. **c**, Metal-SDC surfaces. The difference between E_{ads} and G_{ads} arises from significant entropic contributions at 800 ºC, particularly in adsorption and desorption processes. For gas-phase CO_2 and CO, both translational and rotational contributions were included (TS_{trans} = 1.73 eV and TS_{rot} = 0.61 eV for CO_2; TS_{trans} = 1.67 eV and TS_{rot} = 0.52 eV for CO, obtained from Gaussian 09)[11], while only vibrational contributions were considered for adsorbed CO_2 and CO. Color code: Ni (blue) and Co (light pink), Ce (pale yellow), Sm (orange), C (dark gray), O (red), and oxygen vacancy (dotted white circle).

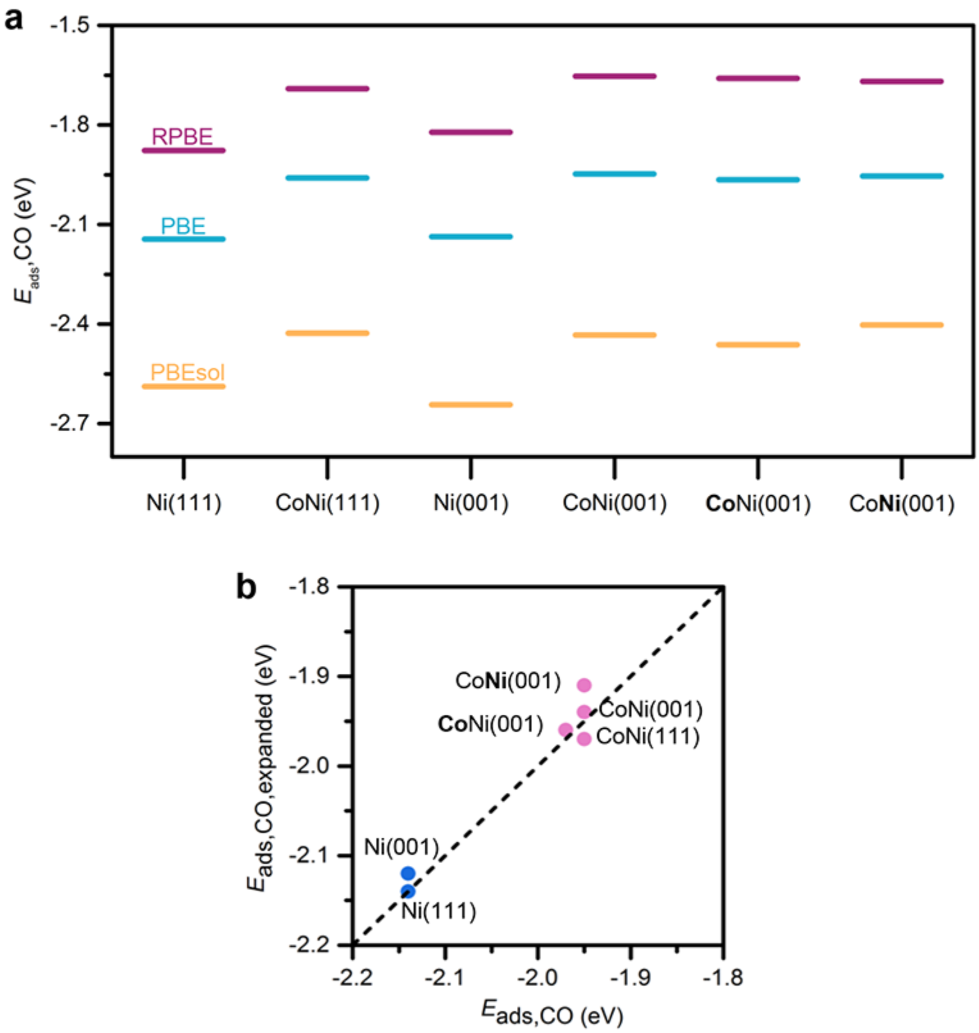

Supplementary Fig. 39 | Influence of functional and metal thermal expansion in the computation of CO adsorption energies on metal surfaces. a, Influence of functional. The adsorption energies obtained with PBE are overestimated and underestimated by RPBE and PBEsol, respectively[12,13]. Nevertheless, the trends in the adsorption energies along the different surfaces are preserved for the three functionals. **b**, Comparison between CO adsorption energies ($E_{ads,CO}$) and values associated with expanded models ($E_{ads,CO,expanded}$). The trends along metal surfaces persisted even when considering metal thermal expansions. To account for the metal thermal expansions at the reaction temperature (800 ºC), expanded models were built by increasing the volume of Ni and CoNi bulks and cleaving (111) and (001) terminations. The expansion was carried out in Ni bulk according to its linear expansion coefficient ($13\cdot10^{-6}$ K^{-1}), while the linear average of the coefficients of Co and Ni ($12.5\cdot10^{-6}$ K^{-1}) was employed for the CoNi model[14-16].

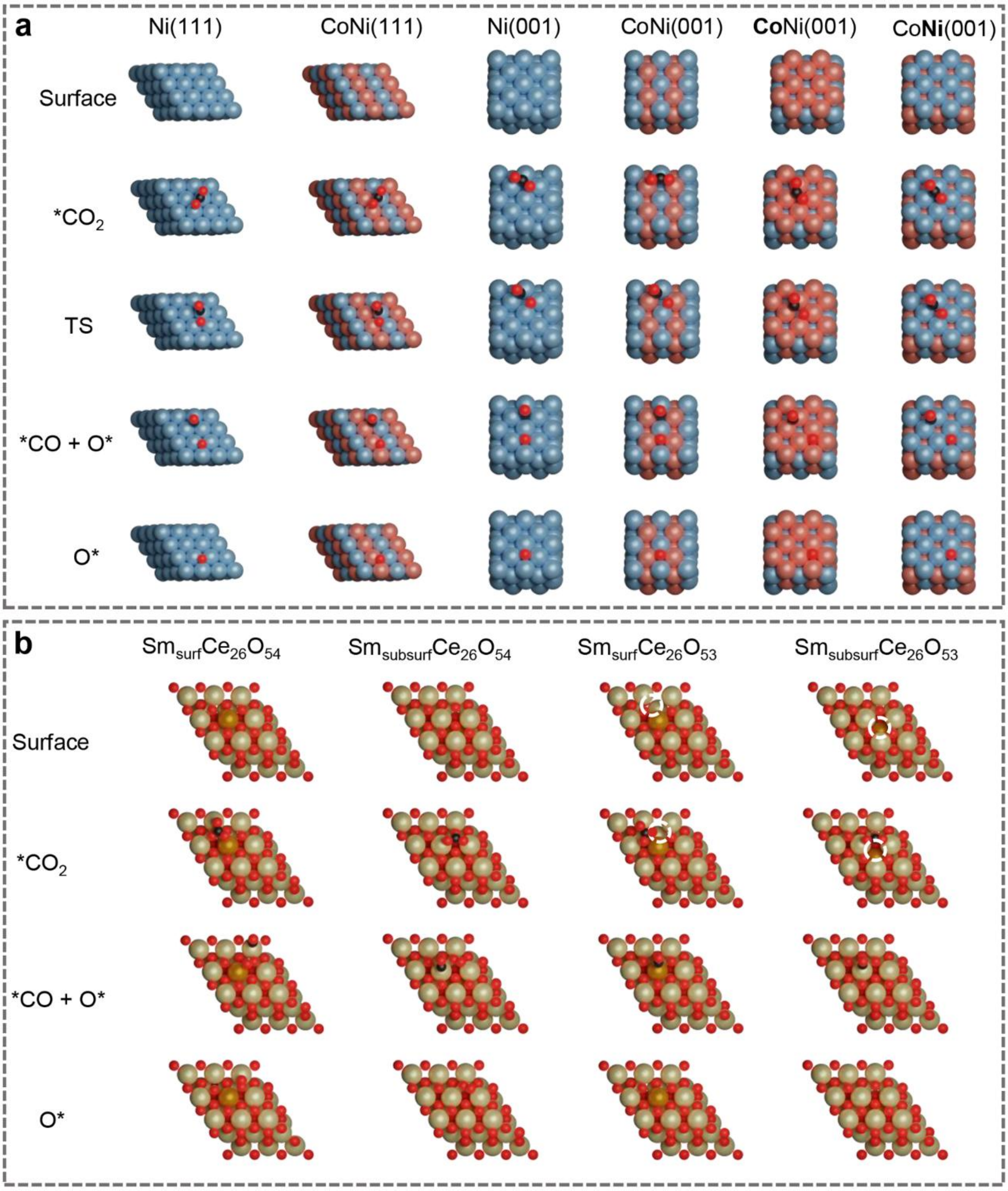

Supplementary Fig. 40 | Snapshots of intermediates for CO_2 electroreduction to CO on isolated metal and SDC surfaces. **a**, Isolated metal surfaces. **b**, Isolated SDC surfaces. Color code: Ni (blue), Co (light pink), Ce (pale yellow), Sm (orange), C (dark gray), O (red), and oxygen vacancy (dotted white circle).

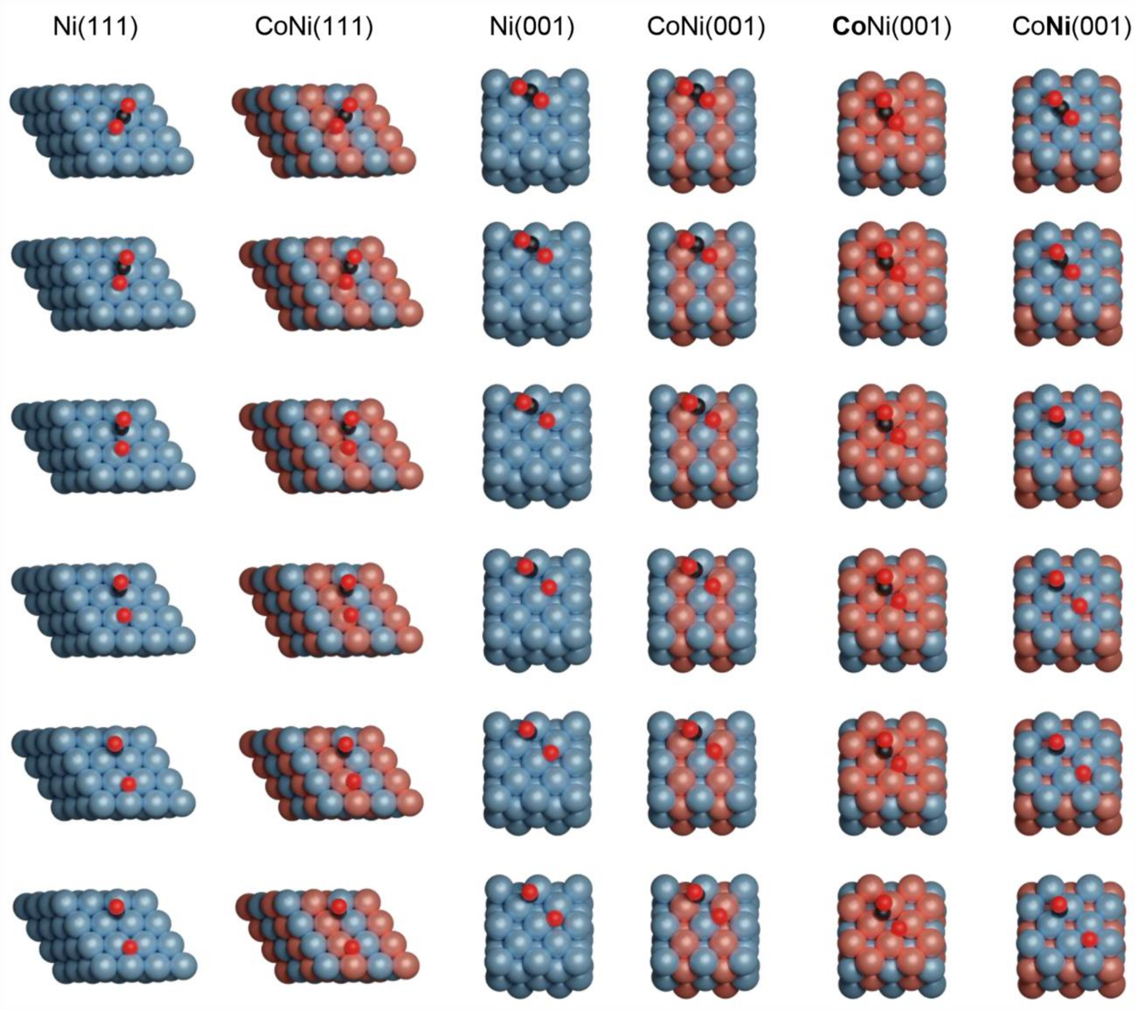

Supplementary Fig. 41 | Snapshots of the images used in the CI-NEB method to locate the transition states on the isolated metal surfaces. Color code: Ni (blue), Co (light pink), C (dark gray), and O (red).

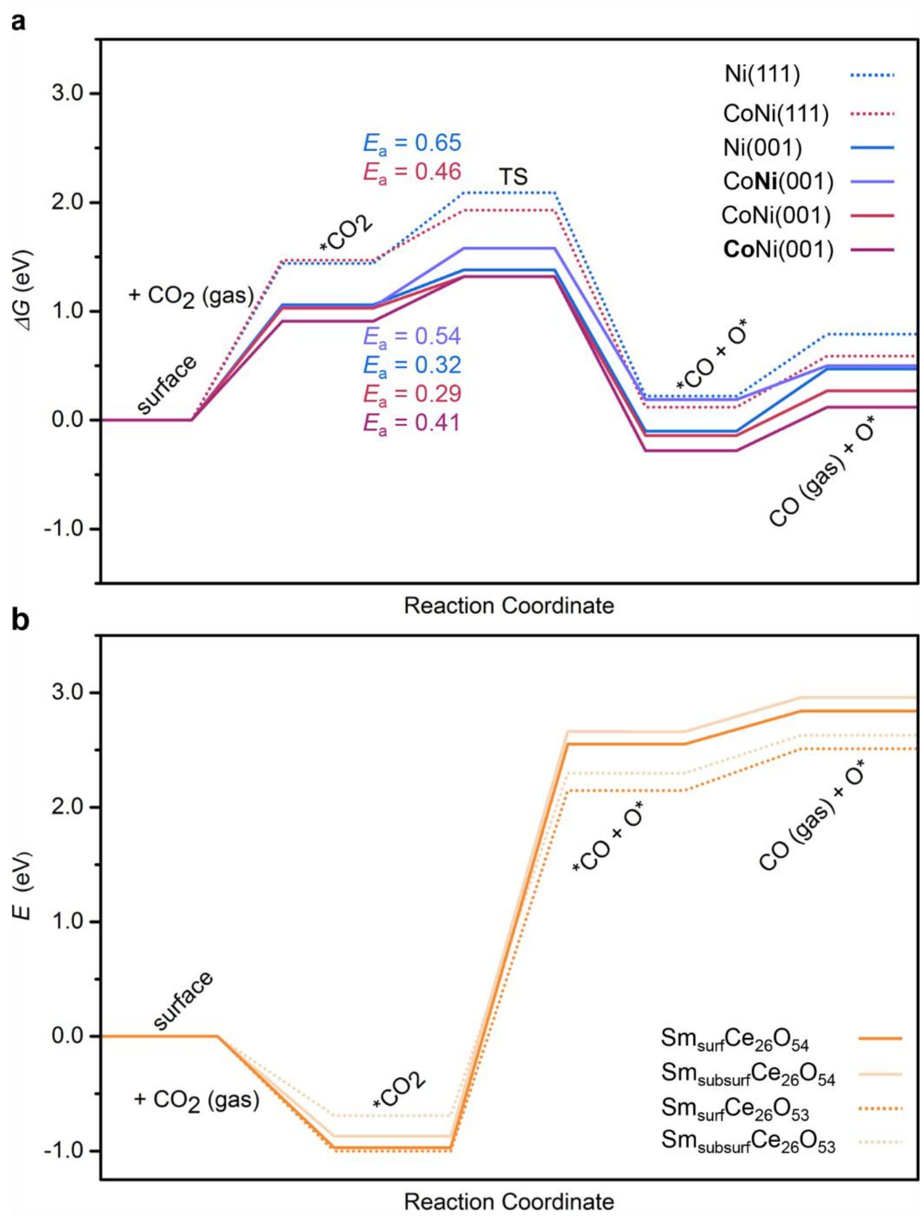

Supplementary Fig. 42 | Gibbs free energy profiles for CO_2 electroreduction to CO on isolated metal and SDC surfaces. a, Isolated metal surfaces. **b**, Isolated SDC surfaces. CO_2 reduction to CO is promoted by metal surfaces, though their CO_2 capture ability is limited. The activation energies (E_a) obtained for the metal surfaces indicate that the (001) surfaces of Ni and CoNi generally outperform the (111) surfaces. Moreover, Ni(001) and the most favourable CoNi(001) model exhibit similar E_a, within the margin of DFT errors. In contrast, SDC enhances CO_2 adsorption, but does not favor CO_2 reduction, in agreement with the low CO_2 electroreduction activity observed for pure SDC (Fig. 2a).

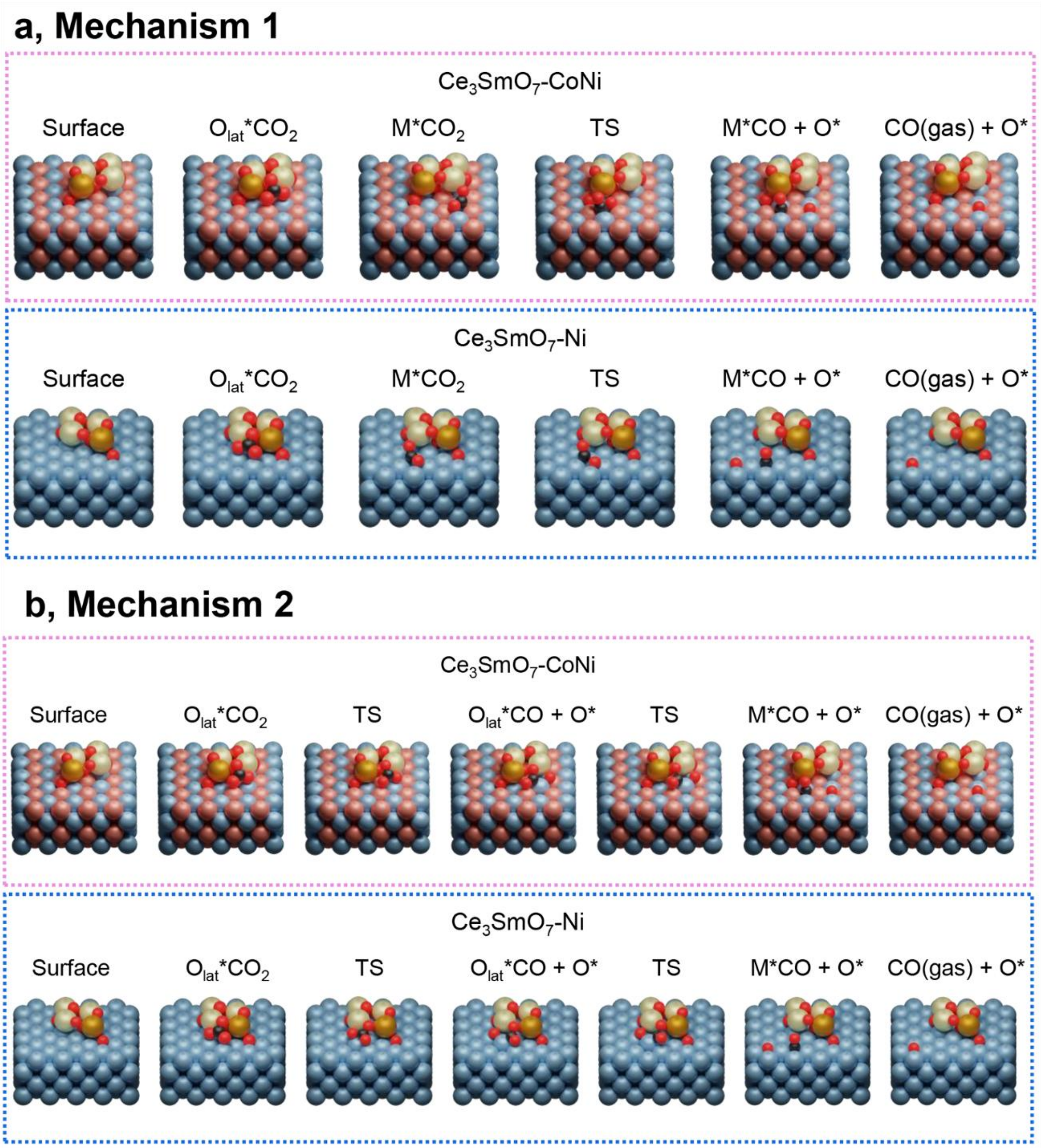

Supplementary Fig. 43 | Snapshots of intermediates for CO_2 electroreduction to CO on the metal-SDC surfaces via two different mechanisms. a, Mechanism 1: CO_2 is captured at the interface, diffuses to nearby metallic surfaces, and undergoes reduction to CO. **b**, Mechanism 2: CO_2 is captured and reduced directly at the metal-SDC interface. Color code: Ni (blue), Co (light pink), Ce (pale yellow), Sm (orange), C (dark gray), and O (red).

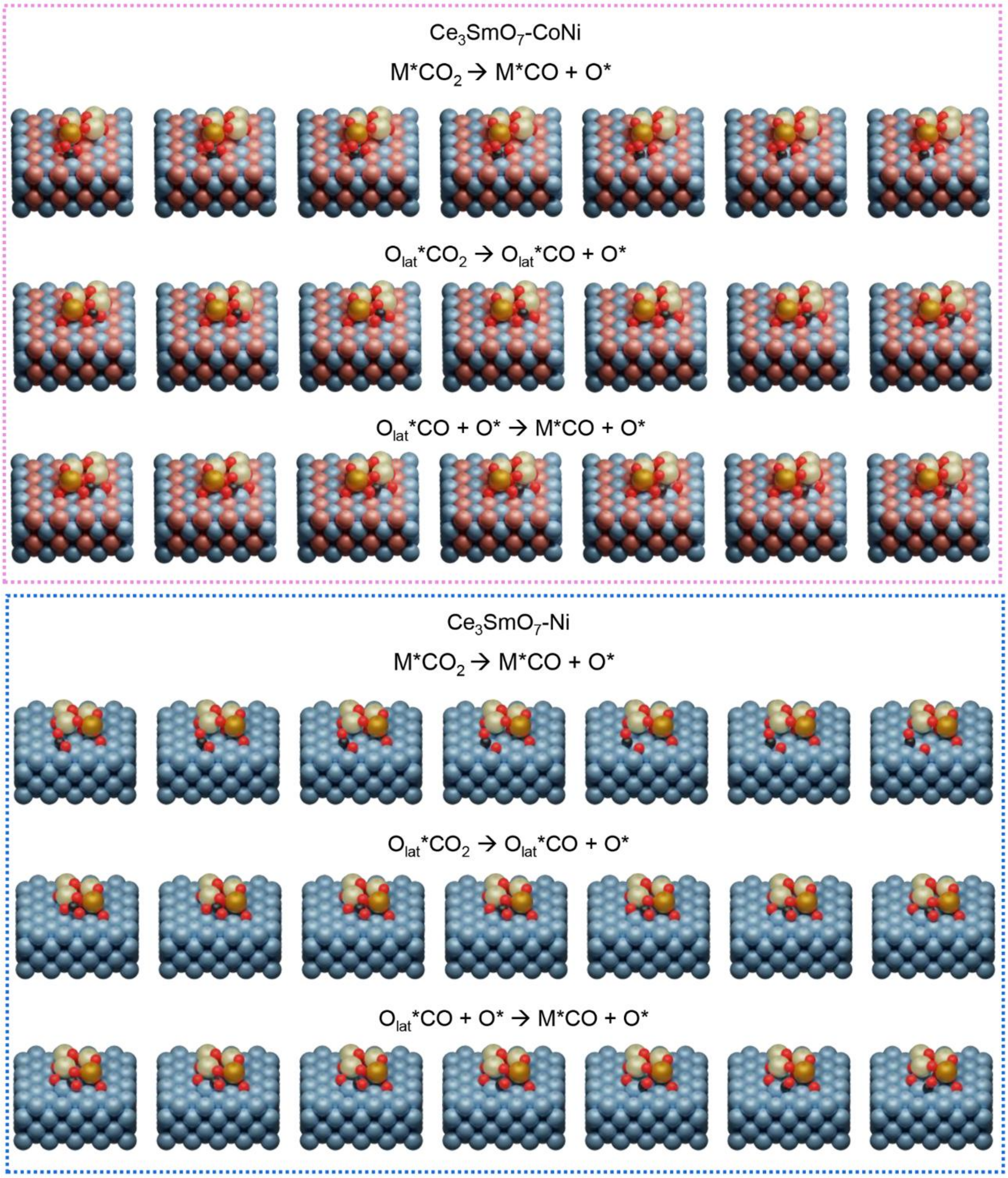

Supplementary Fig. 44 | Snapshots of the images used in the CI-NEB method to locate the transition states on the metal-SDC surfaces. Color code: Ni (blue), Co (light pink), Ce (pale yellow), Sm (orange), C (dark gray), and O (red).

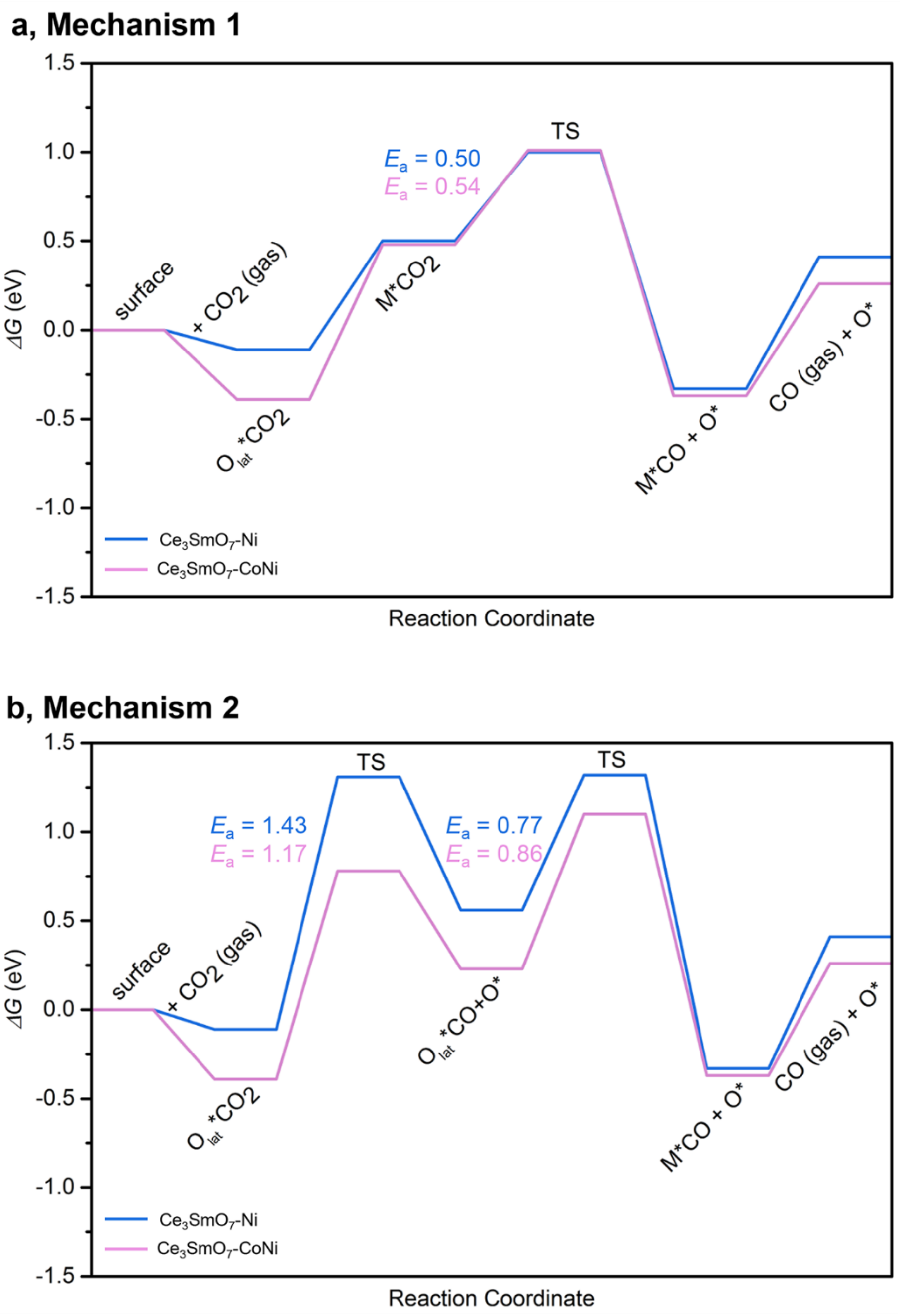

Supplementary Fig. 45 | Gibbs free energy profiles for CO_2 electroreduction to CO on the metal-SDC surfaces via two different mechanisms. a, Mechanism 1: CO_2 is captured at the interface, diffuses to nearby metallic surfaces, and undergoes reduction to CO. **b**, Mechanism 2: CO_2 is captured and reduced directly at the metal-SDC interface. Our results showed that the Mechanism 1 was more favourable.

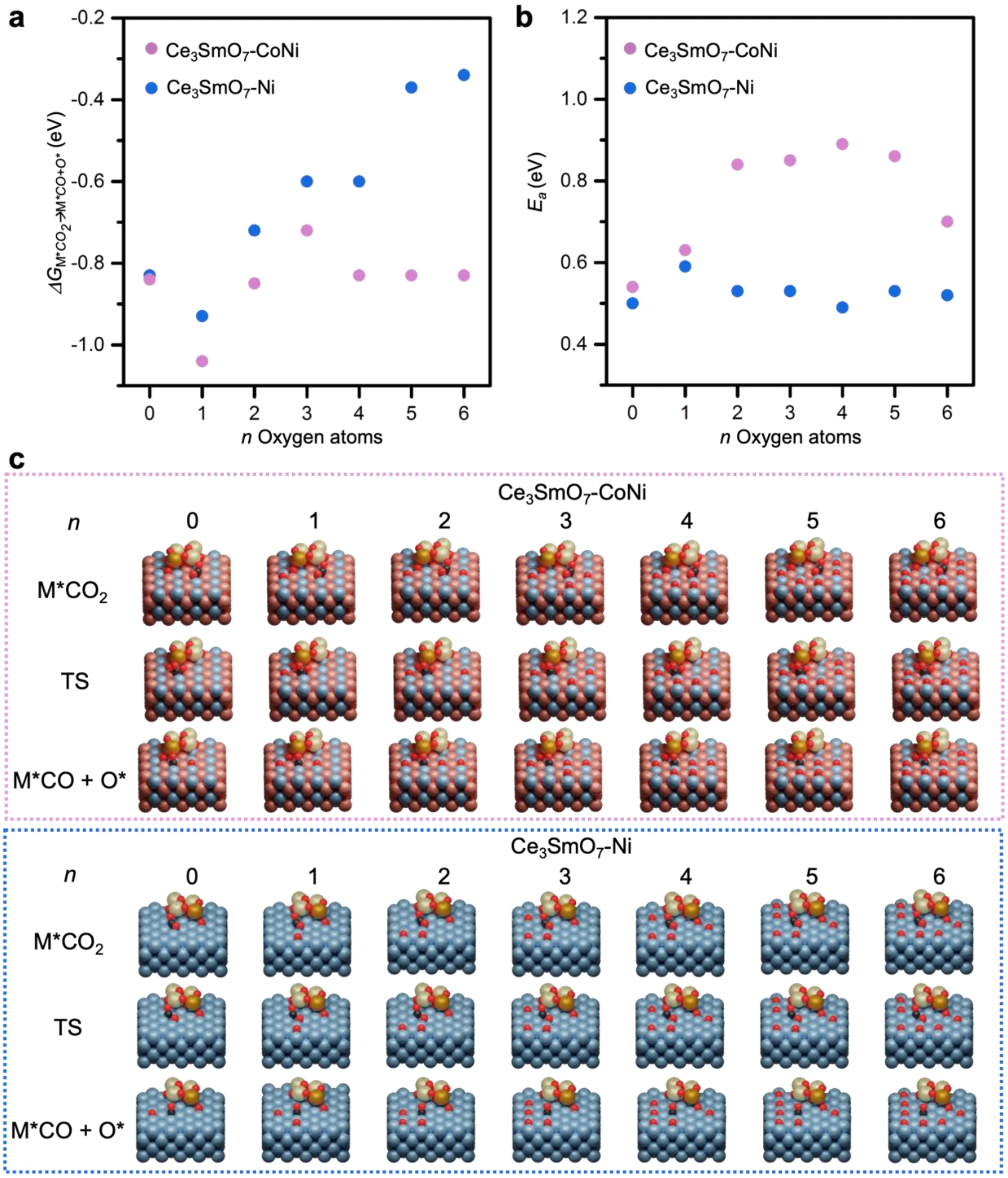

Supplementary Fig. 46 | Effect of oxygen coverage on CO_2 electroreduction on both CoNi-SDC and Ni-SDC surfaces. a,Gibbs free energy and **b**, activation energy for M*CO_2 → M*CO + O* on CoNi-SDC and Ni-SDC surfaces in the presence of 0 to 6 oxygen atoms. The results showed that oxygen accumulation on the catalyst surfaces was detrimental to CO_2 electroreduction. However, under the applied voltage, the adsorbed oxygen primarily migrates to the anode for the oxygen evolution reaction (Fig. 1a). This is supported by the observation that the oxidation states of our catalysts remain almost unchanged before and after stability tests (Supplementary Figs. 13, 15, 22 and 23). **c**, Snapshots of the different structures used to assess the influence of oxygen coverage. Color code: Ni (blue), Co (light pink), Ce (pale yellow), Sm (orange), C (dark gray), and O (red).

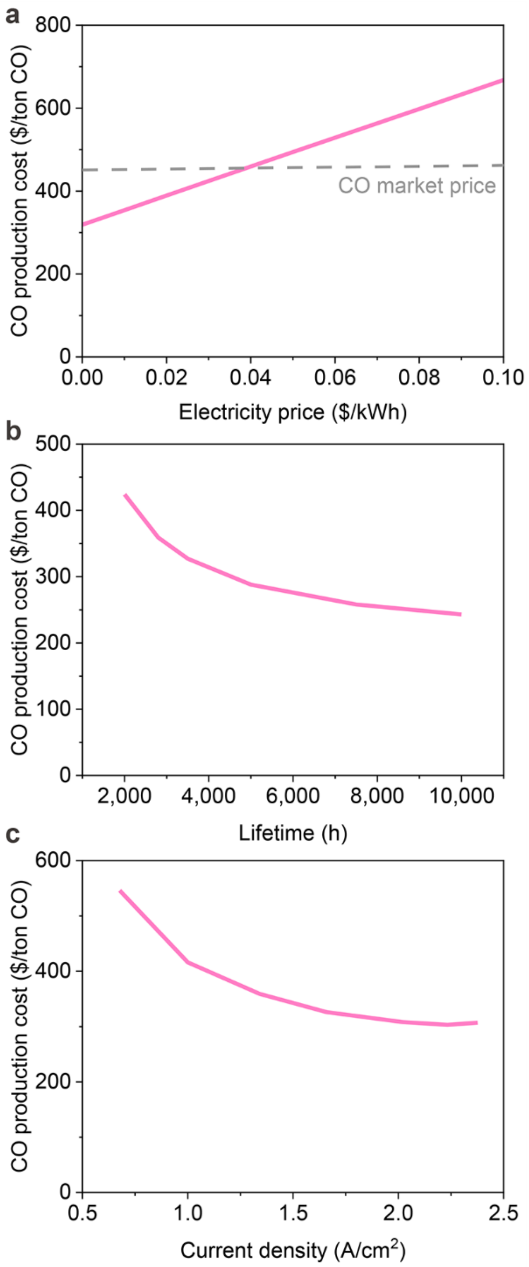

Supplementary Fig. 47 | Effects of electricity price, lifetime and current density on CO production costs. a, Effect of electricity price. Except for electricity price, all other input parameters in Supplementary Table 16 were assumed to be the same. **b**, Effect of catalyst and electrolyte lifetime. Except for catalyst and electrolyte lifetime, all other input parameters in Supplementary Table 16 were assumed to be the same. **c**, Effect of current density. The current density and cell voltage data were based on the performance of $Co_{0.5}Ni_{0.5}$@SDC in Fig. 2a, and all other input parameters in Supplementary Table 16 were assumed to be the same.

Supplementary Table 1 | Physical parameters of each layer of our SOEC.

**Layer**	**Thickness (μm)**	**Mass (mg/cm^2)**
**Cathode**	24	8.2
**Electrolyte**	145	119.0
**Anode**	23	7.8
**Buffer layer**	4.0	2.6

Supplementary Table 2 | Feeding molar ratios and molar ratios measured by ICP-MS of different samples.

Sample	Feeding molar ratio (%)				Molar ratio by ICP-MS (%)			
	Co	Ni	Sm	Ce	Co	Ni	Sm	Ce
$Co_{0.5}Ni_{0.5}$@SDC	40	40	4	16	40.7	40.8	3.6	14.9
$Co_{0.5}Ni_{0.5}$-SDC	40	40	4	16	39.8	40.2	3.8	16.2
Ni@SDC	0	80	4	16	0.0	80.6	3.7	15.7
SDC	0	0	20	80	0.0	0.0	19.3	80.7
$Co_{0.2}Ni_{0.8}$@SDC	16	64	4	16	16.2	64.8	3.7	15.3
$Co_{0.75}Ni_{0.25}$@SDC	60	20	4	16	61.2	20.3	3.6	14.9
Co@SDC	80	0	4	16	81.1	0.0	3.6	15.3
Ni@SDC+Co@SDC	40	40	4	16	40.5	40.3	3.7	15.5

Supplementary Table 3 | C_{dl} and ECSAs for different samples.

Sample	C_{dl} (mF/cm^2)	ECSA (cm^2)
**Electrolyte**	1.12	1.0
**SDC**	39.3	35.0
**$Co_{0.5}Ni_{0.5}$@SDC**	105.9	94.4
**$Co_{0.5}Ni_{0.5}$-SDC**	87.1	77.6
**Ni@SDC**	125.7	112.0
**$Co_{0.2}Ni_{0.8}$@SDC**	138.1	123.1
**$Co_{0.75}Ni_{0.25}$@SDC**	125.5	111.9
**Co@SDC**	120.3	107.3
**Ni@SDC+Co@SDC**	135.5	120.7

Supplementary Table 4 | Average oxidation states of Ni and Co centers based on XANES.

Sample	Ni	Co
$Co_{0.5}Ni_{0.5}$@SDC	–0.087	–0.004
$Co_{0.5}Ni_{0.5}$-SDC	–0.005	0.000
Ni@SDC	–0.093	-
Co@SDC	-	–0.030
$Co_{0.5}Ni_{0.5}$@SDC-after stability	–0.101	–0.005
$Co_{0.5}Ni_{0.5}$-SDC-after stability	0.035	0.026
Ni@SDC-after stability	–0.109	-
Co@SDC-after stability	-	1.2

**Supplementary Table 5 | ** Fitting parameters of Ni K-edge EXAFS spectra.

Sample	Path	N	R	dE	DW	R-factor
$Co_{0.5}Ni_{0.5}$@SDC	Ni-Ni/Co	11.1(2)	2.49(1)	10.5(2)	0.070(2)	3.391
$Co_{0.5}Ni_{0.5}$-SDC	Ni-Ni/Co	11.1(2)	2.49(1)	12.0(2)	0.076(2)	1.381
Ni@SDC	Ni-Ni	8.5(2)	2.49(1)	11.8(2)	0.072(2)	1.755
$Co_{0.5}Ni_{0.5}$@SDC -after stability	Ni-Ni/Co	11.0(2)	2.49(1)	10.4(2)	0.072(2)	3.051
$Co_{0.5}Ni_{0.5}$-SDC -after stability	Ni-Ni/Co	11.4(2)	2.49(1)	12.0(2)	0.077(2)	1.165
Ni@SDC -after stability	Ni-Ni	8.5(2)	2.49(1)	12.1(2)	0.072(2)	1.698

Supplementary Table 6 | Fitting parameters of Co K-edge EXAFS spectra.

Sample	Path	N	R	dE	DW	R-factor
$Co_{0.5}Ni_{0.5}$@SDC	Co-Co/Ni	9.4(2)	2.49(1)	10.6(2)	0.067(2)	2.823
$Co_{0.5}Ni_{0.5}$-SDC	Co-Co/Ni	9.5(2)	2.49(1)	10.7(2)	0.069(2)	2.325
Co@SDC	Co-Co/Ni	10.0(2)	2.50(1)	9.8(2)	0.075(2)	2.249
$Co_{0.5}Ni_{0.5}$@SDC -after stability	Co-Co/Ni	10.4(2)	2.50(1)	11.0(2)	0.071(2)	1.445
$Co_{0.5}Ni_{0.5}$-SDC -after stability	Co-Co/Ni	9.2(2)	2.49(1)	10.6(2)	0.067(2)	2.590
Co@SDC -after stability	Co-O(CoO)<br>Co-Co(Co foil)<br>Co-Co(CoO)	3.6(2)<br>6.2(3)<br>5.6(1)	2.13(1)<br>2.49(1)<br>3.00(1)	11.7(8)<br>14.7(5)<br>8.4(4)	0.081(7)<br>0.096(4)<br>0.063(2)	4.631

Supplementary Table 7 | Molar ratios measured by ICP-MS of different samples after stability tests.

Sample	Molar ratio by ICP-MS (%)			
	Co	Ni	Sm	Ce
$Co_{0.5}Ni_{0.5}$@SDC	40.6	40.7	3.6	15.1
$Co_{0.5}Ni_{0.5}$-SDC	40.8	40.8	3.6	14.8
Ni@SDC	0	81.9	3.5	14.6
$Co_{0.2}Ni_{0.8}$@SDC	16.2	65.5	3.7	14.6
$Co_{0.75}Ni_{0.25}$@SDC	62.4	18.3	3.6	15.7
Co@SDC	81.8	0	3.5	14.7

Supplementary Table 8 | Carbon elemental analysis of different samples before and after stability tests.

Sample	Carbon content (wt%)	
	Before stability	After stability
$Co_{0.5}Ni_{0.5}$@SDC	0	0
$Co_{0.5}Ni_{0.5}$-SDC	0	0
Ni@SDC	0	1.8
$Co_{0.2}Ni_{0.8}$@SDC	0	0
$Co_{0.75}Ni_{0.25}$@SDC	0	0
Co@SDC	0	0

Supplementary Table 9 | Total mass of different cells before and after stability tests.

Cell	Mass (mg/cm^2)	
	Before stability	After stability
$Co_{0.5}Ni_{0.5}$@SDC|| SDC || LSGM || SDC || LSCF	134.4	134.3
$Co_{0.5}Ni_{0.5}$-SDC|| SDC || LSGM || SDC || LSCF	143.3	143.2
Ni@SDC|| SDC || LSGM || SDC || LSCF	135.2	135.8
$Co_{0.2}Ni_{0.8}$@SDC|| SDC || LSGM || SDC || LSCF	138.5	138.7
$Co_{0.75}Ni_{0.25}$@SDC|| SDC || LSGM || SDC || LSCF	131.2	130.0
Co@SDC|| SDC || LSGM || SDC || LSCF	130.1	127.3

Supplementary Table 10 | Optimized lattice parameters of fcc metals and fluorite CeO_2.

**Model**	***a* = *b* = *c* (Å)**	**α = β = γ (º)**
Ni	3.475	90
CoNi	3.476	90
CeO_2	5.472	90

Supplementary Table 11 | Bader charges associated with the Co and Ni atoms of the outermost layer of different Co-Ni alloy models.

**Model**	**Atom**	**Bader charge (|e⁻|)**
CoNi(111)	Ni	–0.10
	Co	0.05
CoNi(001)	Ni	–0.21
	Co	0.19
Co**Ni**(001)	Ni	–0.07
**Co**Ni(001)	Co	0.08

Supplementary Table 12 | Interaction energy (E_{int}, eV) between two adsorbed CO molecules on different metal surfaces.

Model	$E_{ads, CO}$ (eV/mol)	$E_{ads, 2CO}$ (eV/mol)	E_{int} (eV)
Ni(111)	-2.14	-2.06	0.08
CoNi(111)	-1.96	-1.92	0.04
Ni(001)	-2.14	-2.04	0.10
CoNi(001)	-1.95	-1.94	0.01
**Co**Ni(001)	-1.97	-1.87	0.09
Co**Ni**(001)	-1.95	-1.84	0.11

$E_{ads, CO}$ and $E_{ads, 2CO}$ represnt the adsorption energies for one and two adsorbed CO molecules, respectively, normalized per CO molecule. E_{int} was calculated as the difference between $E_{ads, CO}$ and $E_{ads, 2CO}$.

Supplementary Table 13 | Comparison of our cell with other state-of-the-art SOECs for high-temperature CO_2 electroreduction to CO.

Cathode || electrolyte || anode	*U* (V)	*J* (A/cm^2)	Stability (h)	Ref.
NiFe || LSGM || BLC (electrolyte-supported)	1.52	0.98	12 (~1.0 A/cm^2)	[17]
NiFe-LSFM || LSGM || BLC (electrolyte-supported)	1.47	1.2	100 (0.5 A/cm^2)	[10]
Ni-LSTM || YSZ || LSM (electrolyte-supported)	1.6	0.87	100 (0.28 A/cm^2)	[18]
Ni-YSZ || YSZ || RuO_2 (cathode-supported)	1.46	1.0	60 (0.33 A/cm^2)	[19]
Co-LSCM || LSGM || LSCF* (electrolyte-supported)	1.5	0.78	12 (0.7 A/cm^2)	[20]
F-SFM || LSGM || LSCF (electrolyte-supported)	1.34	1.0	120 (1.1 A/cm^2)	[21]
CuNi-CeO_2 || LSGM || BSCF (electrolyte-supported)	1.37	1.0	100 (0.58 A/cm^2)	[22]
LSCoFeM || LSGM || BSCF (electrolyte-supported)	1.4	1.0	100 (0.55 A/cm^2)	[23]
Ni-YSZ || YSZ || LSC (cathode-supported)	1.48	1.17	55 (0.81 A/cm^2)	[1]
SFeRuM || LSGM || BSCF (electrolyte-supported)	1.45	1.0	1,000 (~0.5 A/cm^2)	[3]
CoNi@SDC || LSGM || LSCF (electrolyte-supported)	1.1	1.0	2,000 (1.0 A/cm^2)	This work

Reaction temperature: 800 °C. *Reaction temperature: 850 °C. LSFM: $La_{0.6}Sr_{0.4}Fe_{0.8}Mn_{0.2}O_3$; LSGM: $La_{0.8}Sr_{0.2}Ga_{0.8}Mg_{0.2}O_{3-\delta}$; BLC: $Ba_{0.6}La_{0.4}CoO_3$; LSTM: $La_{0.2}Sr_{0.8}Ti_{0.9}Mn_{0.1}O_{3+\delta}$; YSZ: Y_2O_3-stabilized ZrO_2; LSM: $(La_{0.8}Sr_{0.2})_{0.95}MnO_{3-\delta}$; LSCM: $La_{0.6}Sr_{0.4}Co_{0.7}Mn_{0.3}O_3$; LSCF: $La_{0.6}Sr_{0.4}Co_{0.2}Fe_{0.8}O_{3-\delta}$; SFM: $Sr_2Fe_{1.5}Mo_{0.5}O_{6-\delta}$; BSCF: $Ba_{0.6}Sr_{0.4}Co_{0.2}Fe_{0.8}O_{3-\delta}$; LSCoFeM: $La_{0.4}Sr_{0.6}Co_{0.2}Fe_{0.7}Mo_{0.1}O_{3-\delta}$; LSFeM: $La_{1.2}Sr_{0.8}Fe_{0.6}Mn_{0.4}O_{4-\delta}$; LSC: $La_{0.6}Sr_{0.4}CoO_3$; SFeRuM: $Sr_2Fe_{1.4}Ru_{0.1}Mo_{0.5}O_{6-\delta}$.

Supplementary Table 14 | State-of-the-art MEA systems for low-temperature CO_2 electroreduction to CO.

Cathode ǁ anode	*U* (V)	*J* (A/cm^2)	FE_{CO} (%)	Stability (h)	Ref.
Ag ǁ Ni foam	2.8	0.09	85	10	[24]
Au/MWNT ǁ IrO_x	2.0	0.1	98	8	[25]
Au/C ǁ IrO_x	2.3	0.1	85	100	[26]
Ag NP ǁ IrO_x	3.2	~0.5	90	224	[27]
Ag NP ǁ Ni foam	3.5	1.75	92	12	[2]
Ag NW ǁ IrO_x	3.5	0.1	~90	750	[28]

Reaction temperature: 25~60 °C.

Supplementary Table 15 | Precursor prices and feeding quantities for different samples.

Precursor	Price ($/kg)	Feeding quantity (kg/m^2)				
		$Co_{0.5}Ni_{0.5}$@SDC	SDC	LSGM	LSCF	SFeRuM[3]
Nickel(II) nitrate hexahydrate	78	0.116	-	-	-	-
Cobalt(II) nitrate hexahydrate	185	0.116	-	-	0.0208	-
Iron(III) nitrate nonahydrate	25	-	-	-	0.116	0.113
Cerium(III) nitrate hexahydrate	70	0.0694	0.0521	-	-	-
Samarium(III) nitrate hexahydrate	200	0.0186	0.0140	-	-	-
Lanthanum(III) nitrate hexahydrate	100	-	-	-	0.0923	-
Strontium nitrate	20	-	-	-	0.0303	0.0846
Citric acid	2.5	0.384	0.0576	-	0.206	0.1
Ethylene glycol	1.5	0.124	0.0186	-	-	-
Ethylenediaminetetraacetic acid	10	-	-	-	0.209	-
Polyvinyl alcohol	5	-	-	-	-	-
Ammonia solution (25%)	1	-	-	-	0.644	0.1
Lanthanum oxide	350	-	-	1.303	-	-
Strontium carbonate	34	-	-	0.147	-	-
Gallium(III) oxide	400	-	-	0.750	-	-
Magnesium oxide	1	-	-	0.0403	-	-
Rhenium(III) chloride	20000	-	-	-	-	0.0585
Ammonium heptamolybdate hydrate	273	-	-	-	-	0.0176
**Total price ($/m^2)**		**40.3**	**6.61**	**761.1**	**19.8**	**126.7**

The chemicals were roughly estimated based on the data from Thermo Fisher Scientific - US[5]. We assumed 100% atomic efficiencies for the material synthesis from precursors to the final products.

Supplementary Table 16 | Input parameters for cost estimations.

Parameters	MEA	SOEC	This work
Current density (A/cm^2)	1.75	0.5	1.0
CO FE (%)	92	100	100
H_2 FE (%)	8	0	0
Cell voltage (V)	3.5	1.2	1.2
CO single-pass yield (%)	33	45*	90
Plant lifetime (year)	30	30	30
Catalyst and electrolyte lifetime (h)	210	1,000	2,000
Operation temperature (°C)	25	800	800
CO_2 price ($/ton)	30	30	30
Electricity price ($/kWh)	0.03	0.03	0.03
Base electrolyser price ($/kW)	100	200	200
Discount rate (%)	5	5	5
Cathode price ($\$/m^2$)	518.7	126.7	40.34
Anode price ($\$/m^2$)	250	19.8	19.8
Electrolyte (or membrane) price ($\$/m^2$)	500	767.7	767.7
Capacity factor	0.9	0.9	0.9
Heating efficiency (%)	90	90	90
Other operating factor (%)	10	10	10
BoP factor (%)	150	150	150

*Since no CO single-pass yield was provided in paper[3], we assumed it to be half of our calculated value, i.e., 45%. This assumption is based on the observation that the current density in this paper is half of our current density.

Supplementary Table 17 | Output results of producing 1 ton of CO from cost estimations.

	**MEA**	**SOEC**	**This work**
CO_2 cost (\$/ton CO)	47.14	47.14	47.14
Electrolyser cost (\$/ton CO)	6.87	15.16	7.58
Catalyst and electrolyte costs (\$/ton CO)	818.45	399.68	90.73
Separation cost (\$/ton CO)	37.86	31.57	17.75
Carbonate regeneration cost (\$/ton CO)	20.78	0	0
Heating cost (\$/ton CO)	0	43.24	21.62
Electricity cost (\$/ton CO)	218.49	68.92	68.92
Other operating cost (\$/ton CO)	21.85	6.89	6.89
BoP and installation costs (\$/ton CO)	1298.84	647.78	163.18
Total costs (\$/ton CO)	2470	1260	424

Supplementary references

1. Ozden, A. *et al.* Cascade CO_2 electroreduction enables efficient carbonate-free production of ethylene. *Joule* **5**, 706-719 (2021).
2. Wen, G. *et al.* Continuous CO_2 electrolysis using a CO_2 exsolution-induced flow cell. *Nat. Energy* (2022).
3. Lv, H. *et al.* Promoting exsolution of RuFe alloy nanoparticles on $Sr_2Fe_{1.4}Ru_{0.1}Mo_{0.5}O_{6-\delta}$ via repeated redox manipulations for CO_2 electrolysis. *Nat. Commun.* **12**, 5665 (2021).
4. Jin, J. *et al.* Constrained C_2 adsorbate orientation enables CO-to-acetate electroreduction. *Nature*, **617**, 724–729 (2023).
5. Thermo Fisher Scientific, US (2024). www.thermofisher.com/ch/en/home.html
6. Jouny, M., Luc, W. & Jiao, F. General techno-economic analysis of CO_2 electrolysis systems. *Ind. Eng. Chem. Res.* **57**, 2165-2177 (2018).
7. Keith, D. W., Holmes, G., St. Angelo, D. & Heidel, K. A Process for capturing CO_2 from the atmosphere. *Joule* **2**, 1573-1594 (2018).
8. Wang, F. *et al.* Thermodynamic analysis of solid oxide electrolyzer integration with engine waste heat recovery for hydrogen production. *Case Stud. Therm. Eng.* **27**, 101240 (2021).
9. Wang, F. *et al.* Active site dependent reaction mechanism over Ru/CeO_2 catalyst toward CO_2 methanation. *J. Am. Chem. Soc.* **138**, 6298-6305 (2016).
10. Wang, S., Tsuruta, H., Asanuma, M. & Ishihara, T. Ni–Fe–La(Sr)Fe(Mn)O_3 as a new active cermet cathode for intermediate-temperature CO_2 electrolysis using a $LaGaO_3$-based electrolyte. *Adv. Energy Mater.* **5**, 1401003 (2015).
11. Frisch, M. J. *et al*. Gaussian 09, Revision A. 03, Gaussian, Inc.: Wallingford. (2016).
12. Hammer, B. H. L. B., Hansen, L. B. & Nørskov, J. K. Improved adsorption energetics within density-functional theory using revised Perdew-Burke-Ernzerhof functionals. *Phys. Rev. B* **59**, 7413 (1999).
13. Perdew, J. P. *et al.* Restoring the density-gradient expansion for exchange in solids and surfaces. *Phys. Rev. Lett.* **100**, 136406 (2008).
14. Kim, G. Quantitative calculations of thermal-expansion coefficient in Fe–Ni alloys: First-principles approach. *Curr. Appl. Phys.* **38**, 76-80 (2022)
15. Teslia, S. *et al*. Phase compatibility in (WC-W_2C)/AlFeCoNiCrTi composite produced by spark plasma sintering. *J. Alloy and Compd*. **921**, 166042 (2022)
16. Veleev, I.S. *et al.* Thermal Expansion of Cobalt in Various Structural States. *Phys. Solid State* **51**, 593-596 (2009).
17. Wang, S. *et al.* Ni–Fe bimetallic cathodes for intermediate temperature CO_2 electrolyzers using a $La_{0.9}Sr_{0.1}Ga_{0.8}Mg_{0.2}O_3$ electrolyte. *J. Mater. Chem. A* **1**, 12455-12461 (2013).

18. Ye, L. *et al.* Enhancing CO_2 electrolysis through synergistic control of non-stoichiometry and doping to tune cathode surface structures. *Nat. Commun.* **8**, 14785 (2017).
19. Song, Y. *et al.* Pure CO_2 electrolysis over an Ni/YSZ cathode in a solid oxide electrolysis cell. *J. Mater. Chem. A* **6**, 13661-13667 (2018).
20. Park, S. *et al.* In situ exsolved Co nanoparticles on Ruddlesden-Popper material as highly active catalyst for CO_2 electrolysis to CO. *Appl. Catal. B.* **248**, 147-156 (2019).
21. Li, Y. *et al.* Perovskite oxyfluoride electrode enabling direct electrolyzing carbon dioxide with excellent electrochemical performances. *Adv. Energy Mater.* **9**, 1803156 (2019).
22. Wang, W. *et al.* Enhanced carbon dioxide electrolysis at redox manipulated interfaces. *Nat. Commun.* **10**, 1550 (2019).
23. Lv, H. *et al.* Atomic-scale insight into exsolution of CoFe alloy nanoparticles in $La_{0.4}Sr_{0.6}Co_{0.2}Fe_{0.7}Mo_{0.1}O_{3-\delta}$ with efficient CO_2 electrolysis. *Angew. Chem. Int. Ed.* **59**, 15968-15973 (2020).
24. Gabardo, C. M. *et al.* Combined high alkalinity and pressurization enable efficient CO_2 electroreduction to CO. *Energy Environ. Sci.* **11**, 2531-2539 (2018).
25. Verma, S. *et al.* Insights into the low overpotential electroreduction of CO_2 to CO on a supported gold catalyst in an alkaline flow electrolyzer. *ACS Energy Lett.* **3**, 193-198 (2018).
26. Yin, Z. *et al.* An alkaline polymer electrolyte CO_2 electrolyzer operated with pure water. *Energy Environ. Sci.* **12**, 2455-2462 (2019).
27. Endrődi, B. *et al.* Operando cathode activation with alkali metal cations for high current density operation of water-fed zero-gap carbon dioxide electrolysers. *Nat. Energy* **6**, 439-448 (2021).
28. Kim, J. Y. T. *et al.* Recovering carbon losses in CO_2 electrolysis using a solid electrolyte reactor. *Nat. Catal.* **5**, 288-299 (2022).
