# Supplementary material for: Encapsulated Co–Ni alloy boosts high-temperature CO2 electroreduction
Source: Nature. 2025 May 14;641(8065):1156–61. doi: 10.1038/s41586-025-08978-0 (PMC12119355; doi:10.1038/s41586-025-08978-0)
Supplement: Supplementary file 2 — Atomic positions for all DFT models. [file 41586_2025_8978_MOESM2_ESM.pdf]

Supplementary Data

Atomic positions of DFT models for Encapsulated Co-Ni alloy boosts high-temperature CO_2 electroreduction

/db/jmorales/CoNi-alloy/References/CO_2

a =			20.0				
b =			20.0				
c =			20.0				
alpha =			90.0				
beta =			90.0				
gamma =			90.0				
	Atom	X	Y	Z	X	Y	Z
1	C	9.3465	11.4011	10.0000	0.4673	0.5701	0.5000
2	O	10.5065	11.4011	10.0000	0.5253	0.5701	0.5000
3	O	8.1865	11.4011	10.0000	0.4093	0.5701	0.5000

/db/jmorales/CoNi-alloy/References/Sm_2O_3-bulk

a =	10.930423264215735
b =	10.930423264215735
c =	10.930423264215735
alpha =	90.0
beta =	90.0
gamma =	90.0

	Atom	X	Y	Z	X	Y	Z
1	Sm	5.4652	0.0000	5.4652	0.5000	0.0000	0.5000
2	Sm	5.4652	0.0000	0.0000	0.5000	0.0000	0.0000
3	Sm	5.4652	5.4652	5.4652	0.5000	0.5000	0.5000
4	Sm	0.0000	0.0000	5.4652	0.0000	0.0000	0.5000
5	Sm	8.1978	2.3868	0.0000	0.7500	0.2184	0.0000
6	Sm	5.4652	2.7326	7.8520	0.5000	0.2500	0.7184
7	Sm	3.0784	0.0000	2.7326	0.2816	0.0000	0.2500
8	Sm	0.0000	2.7326	3.0784	0.0000	0.2500	0.2816
9	Sm	7.8520	5.4652	2.7326	0.7184	0.5000	0.2500
10	Sm	8.1978	8.5437	5.4652	0.7500	0.7816	0.5000
11	Sm	2.7326	8.5437	0.0000	0.2500	0.7816	0.0000
12	Sm	5.4652	8.1978	3.0784	0.5000	0.7500	0.2816
13	Sm	7.8520	0.0000	8.1978	0.7184	0.0000	0.7500
14	Sm	0.0000	8.1978	7.8520	0.0000	0.7500	0.7184
15	Sm	3.0784	5.4652	8.1978	0.2816	0.5000	0.7500
16	Sm	2.7326	2.3868	5.4652	0.2500	0.2184	0.5000
17	Sm	0.0000	5.4652	0.0000	0.0000	0.5000	0.0000
18	Sm	0.0000	5.4652	5.4652	0.0000	0.5000	0.5000
19	Sm	0.0000	0.0000	0.0000	0.0000	0.0000	0.0000
20	Sm	5.4652	5.4652	0.0000	0.5000	0.5000	0.0000
21	Sm	2.7326	7.8520	5.4652	0.2500	0.7184	0.5000
22	Sm	0.0000	8.1978	2.3868	0.0000	0.7500	0.2184
23	Sm	8.5437	5.4652	8.1978	0.7816	0.5000	0.7500
24	Sm	5.4652	8.1978	8.5437	0.5000	0.7500	0.7816
25	Sm	2.3868	0.0000	8.1978	0.2184	0.0000	0.7500
26	Sm	2.7326	3.0784	0.0000	0.2500	0.2816	0.0000
27	Sm	8.1978	3.0784	5.4652	0.7500	0.2816	0.5000
28	Sm	0.0000	2.7326	8.5437	0.0000	0.2500	0.7816
29	Sm	2.3868	5.4652	2.7326	0.2184	0.5000	0.2500
30	Sm	5.4652	2.7326	2.3868	0.5000	0.2500	0.2184
31	Sm	8.5437	0.0000	2.7326	0.7816	0.0000	0.2500
32	Sm	8.1978	7.8520	0.0000	0.7500	0.7184	0.0000
33	O	4.3773	1.5430	4.0575	0.4005	0.1412	0.3712
34	O	6.8730	1.0880	1.5430	0.6288	0.0995	0.1412
35	O	3.9222	4.0575	6.5532	0.3588	0.3712	0.5995
36	O	9.5227	1.0880	9.3874	0.8712	0.0995	0.8588
37	O	9.3874	1.4078	4.3773	0.8588	0.1288	0.4005
38	O	9.5227	9.8425	7.0082	0.8712	0.9005	0.6412
39	O	1.5430	4.0575	4.3773	0.1412	0.3712	0.4005
40	O	7.0082	1.4078	6.5532	0.6412	0.1288	0.5995

41	O	6.8730	9.8425	3.9222	0.6288	0.9005	0.3588
42	O	6.5532	3.9222	4.0575	0.5995	0.3588	0.3712
43	O	4.3773	9.3874	1.4078	0.4005	0.8588	0.1288
44	O	6.5532	7.0082	1.4078	0.5995	0.6412	0.1288
45	O	6.5532	9.3874	6.8730	0.5995	0.8588	0.6288
46	O	4.0575	9.8425	9.3874	0.3712	0.9005	0.8588
47	O	7.0082	6.8730	4.3773	0.6412	0.6288	0.4005
48	O	1.4078	9.8425	1.5430	0.1288	0.9005	0.1412
49	O	1.5430	9.5227	6.5532	0.1412	0.8712	0.5995
50	O	1.4078	1.0880	3.9222	0.1288	0.0995	0.3588
51	O	9.3874	6.8730	6.5532	0.8588	0.6288	0.5995
52	O	3.9222	9.5227	4.3773	0.3588	0.8712	0.4005
53	O	4.0575	1.0880	7.0082	0.3712	0.0995	0.6412
54	O	4.3773	7.0082	6.8730	0.4005	0.6412	0.6288
55	O	6.5532	1.5430	9.5227	0.5995	0.1412	0.8712
56	O	4.3773	3.9222	9.5227	0.4005	0.3588	0.8712
57	O	9.8425	7.0082	9.5227	0.9005	0.6412	0.8712
58	O	1.4078	6.5532	7.0082	0.1288	0.5995	0.6412
59	O	9.3874	9.5227	1.0880	0.8588	0.8712	0.0995
60	O	4.0575	6.5532	3.9222	0.3712	0.5995	0.3588
61	O	3.9222	6.8730	9.8425	0.3588	0.6288	0.9005
62	O	4.0575	4.3773	1.5430	0.3712	0.4005	0.1412
63	O	7.0082	9.5227	9.8425	0.6412	0.8712	0.9005
64	O	1.5430	6.8730	1.0880	0.1412	0.6288	0.0995
65	O	1.4078	4.3773	9.3874	0.1288	0.4005	0.8588
66	O	1.0880	9.3874	9.5227	0.0995	0.8588	0.8712
67	O	9.8425	3.9222	6.8730	0.9005	0.3588	0.6288
68	O	1.0880	1.5430	6.8730	0.0995	0.1412	0.6288
69	O	1.0880	3.9222	1.4078	0.0995	0.3588	0.1288
70	O	9.5227	4.3773	3.9222	0.8712	0.4005	0.3588
71	O	1.5430	1.4078	9.8425	0.1412	0.1288	0.9005
72	O	6.8730	4.3773	7.0082	0.6288	0.4005	0.6412
73	O	7.0082	4.0575	1.0880	0.6412	0.3712	0.0995
74	O	6.8730	6.5532	9.3874	0.6288	0.5995	0.8588
75	O	3.9222	1.4078	1.0880	0.3588	0.1288	0.0995
76	O	9.3874	4.0575	9.8425	0.8588	0.3712	0.9005
77	O	9.5227	6.5532	1.5430	0.8712	0.5995	0.1412
78	O	9.8425	1.5430	1.4078	0.9005	0.1412	0.1288
79	O	1.0880	7.0082	4.0575	0.0995	0.6412	0.3712
80	O	9.8425	9.3874	4.0575	0.9005	0.8588	0.3712

/db/jmorales/CoNi-alloy/References/CeO_2(111)

a =			11.6082000732				
b =			11.608200073110298				
c =			22.898399353				
alpha =			90.0				
beta =			90.0				
gamma =			120.0				
	Atom	X	Y	Z	X	Y	Z
1	Ce	1.9347	1.1170	0.7898	0.2222	0.1111	0.0345
2	Ce	0.0000	4.4680	0.7898	0.2222	0.4444	0.0345
3	Ce	-1.9347	7.8190	0.7898	0.2222	0.7778	0.0345
4	Ce	5.8041	1.1170	0.7898	0.5556	0.1111	0.0345
5	Ce	3.8694	4.4680	0.7898	0.5556	0.4444	0.0345
6	Ce	1.9347	7.8190	0.7898	0.5556	0.7778	0.0345
7	Ce	9.6735	1.1170	0.7898	0.8889	0.1111	0.0345
8	Ce	7.7388	4.4680	0.7898	0.8889	0.4444	0.0345
9	Ce	5.8041	7.8190	0.7898	0.8889	0.7778	0.0345
10	Ce	-0.0000	2.2340	3.9541	0.1111	0.2222	0.1727
11	Ce	-1.9347	5.5850	3.9541	0.1111	0.5556	0.1727
12	Ce	-3.8694	8.9360	3.9541	0.1111	0.8889	0.1727
13	Ce	3.8694	2.2340	3.9541	0.4444	0.2222	0.1727
14	Ce	1.9347	5.5850	3.9541	0.4444	0.5556	0.1727
15	Ce	-0.0000	8.9360	3.9541	0.4444	0.8889	0.1727
16	Ce	7.7388	2.2340	3.9541	0.7778	0.2222	0.1727
17	Ce	5.8041	5.5850	3.9541	0.7778	0.5556	0.1727
18	Ce	3.8694	8.9360	3.9541	0.7778	0.8889	0.1727
19	Ce	-0.0000	-0.0000	7.1054	-0.0000	-0.0000	0.3103
20	Ce	-1.9347	3.3510	7.1054	-0.0000	0.3333	0.3103
21	Ce	-3.8694	6.7020	7.1054	-0.0000	0.6667	0.3103
22	Ce	3.8694	-0.0000	7.1054	0.3333	-0.0000	0.3103
23	Ce	1.9347	3.3510	7.1054	0.3333	0.3333	0.3103
24	Ce	-0.0000	6.7020	7.1054	0.3333	0.6667	0.3103
25	Ce	7.7388	-0.0000	7.1054	0.6667	-0.0000	0.3103
26	Ce	5.8041	3.3510	7.1054	0.6667	0.3333	0.3103
27	Ce	3.8694	6.7020	7.1054	0.6667	0.6667	0.3103
28	O	0.0000	0.0000	0.0000	0.0000	0.0000	0.0000
29	O	-1.9347	3.3510	0.0000	-0.0000	0.3333	0.0000
30	O	-3.8694	6.7020	0.0000	-0.0000	0.6667	0.0000
31	O	3.8694	0.0000	0.0000	0.3333	0.0000	0.0000
32	O	1.9347	3.3510	0.0000	0.3333	0.3333	0.0000
33	O	0.0000	6.7020	0.0000	0.3333	0.6667	0.0000
34	O	7.7388	0.0000	0.0000	0.6667	0.0000	0.0000
35	O	5.8041	3.3510	0.0000	0.6667	0.3333	0.0000
36	O	3.8694	6.7020	0.0000	0.6667	0.6667	0.0000
37	O	-0.0000	2.2340	1.5798	0.1111	0.2222	0.0690
38	O	-1.9347	5.5850	1.5798	0.1111	0.5556	0.0690
39	O	-3.8694	8.9360	1.5798	0.1111	0.8889	0.0690
40	O	3.8694	2.2340	1.5798	0.4444	0.2222	0.0690

41	O	1.9347	5.5850	1.5798	0.4444	0.5556	0.0690
42	O	-0.0000	8.9360	1.5798	0.4444	0.8889	0.0690
43	O	7.7388	2.2340	1.5798	0.7778	0.2222	0.0690
44	O	5.8041	5.5850	1.5798	0.7778	0.5556	0.0690
45	O	3.8694	8.9360	1.5798	0.7778	0.8889	0.0690
46	O	1.9347	1.1170	3.1593	0.2222	0.1111	0.1380
47	O	0.0000	4.4680	3.1593	0.2222	0.4444	0.1380
48	O	-1.9347	7.8190	3.1593	0.2222	0.7778	0.1380
49	O	5.8041	1.1170	3.1593	0.5556	0.1111	0.1380
50	O	3.8694	4.4680	3.1593	0.5556	0.4444	0.1380
51	O	1.9347	7.8190	3.1593	0.5556	0.7778	0.1380
52	O	9.6735	1.1170	3.1593	0.8889	0.1111	0.1380
53	O	7.7388	4.4680	3.1593	0.8889	0.4444	0.1380
54	O	5.8041	7.8190	3.1593	0.8889	0.7778	0.1380
55	O	0.0000	0.0000	4.7625	0.0000	0.0000	0.2080
56	O	-1.9347	3.3510	4.7625	0.0000	0.3333	0.2080
57	O	-3.8694	6.7020	4.7625	0.0000	0.6667	0.2080
58	O	3.8694	0.0000	4.7625	0.3333	0.0000	0.2080
59	O	1.9347	3.3510	4.7625	0.3333	0.3333	0.2080
60	O	0.0000	6.7020	4.7625	0.3333	0.6667	0.2080
61	O	7.7388	0.0000	4.7625	0.6667	0.0000	0.2080
62	O	5.8041	3.3510	4.7625	0.6667	0.3333	0.2080
63	O	3.8694	6.7020	4.7625	0.6667	0.6667	0.2080
64	O	-0.0000	2.2340	6.3036	0.1111	0.2222	0.2753
65	O	-1.9347	5.5850	6.3036	0.1111	0.5556	0.2753
66	O	-3.8694	8.9360	6.3036	0.1111	0.8889	0.2753
67	O	3.8694	2.2340	6.3036	0.4444	0.2222	0.2753
68	O	1.9347	5.5850	6.3036	0.4444	0.5556	0.2753
69	O	-0.0000	8.9360	6.3036	0.4444	0.8889	0.2753
70	O	7.7388	2.2340	6.3036	0.7778	0.2222	0.2753
71	O	5.8041	5.5850	6.3036	0.7778	0.5556	0.2753
72	O	3.8694	8.9360	6.3036	0.7778	0.8889	0.2753
73	O	1.9347	1.1170	7.8931	0.2222	0.1111	0.3447
74	O	0.0000	4.4680	7.8931	0.2222	0.4444	0.3447
75	O	-1.9347	7.8190	7.8931	0.2222	0.7778	0.3447
76	O	5.8041	1.1170	7.8931	0.5556	0.1111	0.3447
77	O	3.8694	4.4680	7.8931	0.5556	0.4444	0.3447
78	O	1.9347	7.8190	7.8931	0.5556	0.7778	0.3447
79	O	9.6735	1.1170	7.8931	0.8889	0.1111	0.3447
80	O	7.7388	4.4680	7.8931	0.8889	0.4444	0.3447
81	O	5.8041	7.8190	7.8931	0.8889	0.7778	0.3447

/db/jmorales/CoNi-alloy/References/O_2

a =	18.0
b =	19.0
c =	20.0
alpha =	90.0
beta =	90.0
gamma =	90.0

	Atom	X	Y	Z	X	Y	Z
1	O	8.0411	11.4272	8.9897	0.4467	0.6014	0.4495
2	O	8.0411	12.2009	9.9503	0.4467	0.6422	0.4975

/db/jmorales/CoNi-alloy/References/CO

a =		20.0					
b =		20.0					
c =		20.0					
alpha =		90.0					
beta =		90.0					
gamma =		90.0					
	Atom	X	Y	Z	X	Y	Z
1	C	8.0411	11.0626	9.0000	0.4021	0.5531	0.4500
2	O	8.0411	12.1926	9.0000	0.4021	0.6096	0.4500

/db/jmorales/CoNi-alloy/Bulks/CoNi-bulk

a =	3.475834607
b =	3.475834607
c =	3.475834607
alpha =	90.0
beta =	90.0
gamma =	90.0

	Atom	X	Y	Z	X	Y	Z
1	Ni	0.0000	0.0000	0.0000	0.0000	0.0000	0.0000
2	Ni	0.0000	1.7379	1.7379	0.0000	0.5000	0.5000
3	Co	1.7379	0.0000	1.7379	0.5000	0.0000	0.5000
4	Co	1.7379	1.7379	0.0000	0.5000	0.5000	0.0000

/db/jmorales/CoNi-alloy/Bulks/Ni-bulk

a = 3.475495934
b = 3.475495934
c = 3.475495934
alpha = 90.0
beta = 90.0
gamma = 90.0

	Atom	X	Y	Z	X	Y	Z
1	Ni	0.0000	0.0000	0.0000	0.0000	0.0000	0.0000
2	Ni	0.0000	1.7378	1.7378	0.0000	0.5000	0.5000
3	Ni	1.7378	0.0000	1.7378	0.5000	0.0000	0.5000
4	Ni	1.7378	1.7378	0.0000	0.5000	0.5000	0.0000

/db/jmorales/CoNi-alloy/Bulks/Ni-bulk-expanded

a =	3.523982404
b =	3.523982404
c =	3.523982404
alpha =	90.0
beta =	90.0
gamma =	90.0

	Atom	X	Y	Z	X	Y	Z
1	Ni	0.0000	0.0000	0.0000	0.0000	0.0000	0.0000
2	Ni	0.0000	1.7620	1.7620	0.0000	0.5000	0.5000
3	Ni	1.7620	0.0000	1.7620	0.5000	0.0000	0.5000
4	Ni	1.7620	1.7620	0.0000	0.5000	0.5000	0.0000

/db/jmorales/CoNi-alloy/Bulks/CoNi-bulk-expanded

a =	3.522460756
b =	3.522460756
c =	3.522460756
alpha =	90.0
beta =	90.0
gamma =	90.0

	Atom	X	Y	Z	X	Y	Z
1	Ni	0.0000	0.0000	0.0000	0.0000	0.0000	0.0000
2	Ni	0.0000	1.7612	1.7612	0.0000	0.5000	0.5000
3	Co	1.7612	0.0000	1.7612	0.5000	0.0000	0.5000
4	Co	1.7612	1.7612	0.0000	0.5000	0.5000	0.0000

/db/jmorales/CoNi-alloy/Slabs/Segregation/Co-CoNi(001)/Co-CoNi(001)-2-Ni

a =	6.9516692132
b =	6.9516692132
c =	21.9516692133
alpha =	90.0
beta =	90.0
gamma =	90.0

	Atom	X	Y	Z	X	Y	Z
1	Ni	0.0000	0.0000	0.0000	0.0000	0.0000	0.0000
2	Ni	1.7379	1.7379	0.0000	0.2500	0.2500	0.0000
3	Ni	0.0000	3.4758	0.0000	0.0000	0.5000	0.0000
4	Ni	1.7379	5.2138	0.0000	0.2500	0.7500	0.0000
5	Ni	3.4758	0.0000	0.0000	0.5000	0.0000	0.0000
6	Ni	5.2138	1.7379	0.0000	0.7500	0.2500	0.0000
7	Ni	3.4758	3.4758	0.0000	0.5000	0.5000	0.0000
8	Ni	5.2138	5.2138	0.0000	0.7500	0.7500	0.0000
9	Ni	0.0032	0.0006	3.5190	0.0005	0.0001	0.1603
10	Ni	1.7301	1.7370	3.5253	0.2489	0.2499	0.1606
11	Ni	6.9424	3.4769	3.5239	0.9987	0.5002	0.1605
12	Ni	1.7293	5.2161	3.5176	0.2488	0.7503	0.1602
13	Ni	5.2261	1.7358	3.5198	0.7518	0.2497	0.1603
14	Ni	1.7220	3.4923	5.2498	0.2477	0.5024	0.2392
15	Ni	5.2209	5.2135	3.5088	0.7510	0.7500	0.1598
16	Ni	3.4929	1.7316	5.2405	0.5025	0.2491	0.2387
17	Co	1.7379	0.0000	1.7379	0.2500	0.0000	0.0792
18	Co	0.0000	1.7379	1.7379	0.0000	0.2500	0.0792
19	Co	1.7379	3.4758	1.7379	0.2500	0.5000	0.0792
20	Co	0.0000	5.2138	1.7379	0.0000	0.7500	0.0792
21	Co	5.2138	0.0000	1.7379	0.7500	0.0000	0.0792
22	Co	3.4758	1.7379	1.7379	0.5000	0.2500	0.0792
23	Co	5.2138	3.4758	1.7379	0.7500	0.5000	0.0792
24	Co	3.4758	5.2138	1.7379	0.5000	0.7500	0.0792
25	Co	1.7456	-0.0169	5.2441	0.2511	-0.0024	0.2389
26	Co	6.9340	1.7200	5.2572	0.9975	0.2474	0.2395
27	Co	3.4813	3.4795	3.5153	0.5008	0.5005	0.1601
28	Co	6.9274	5.2415	5.2538	0.9965	0.7540	0.2393
29	Co	5.2236	6.9226	5.2401	0.7514	0.9958	0.2387
30	Co	5.2216	3.4942	5.2448	0.7511	0.5026	0.2389
31	Co	3.4886	5.2197	5.2293	0.5018	0.7509	0.2382
32	Co	3.4762	-0.0072	3.5217	0.5001	-0.0010	0.1604

/db/jmorales/CoNi-alloy/Slabs/Segregation/Ni-CoNi(001)/Ni-CoNi(001)-3-Co-CO

a = 6.9516692132
b = 6.9516692132
c = 21.9516692133
alpha = 90.0
beta = 90.0
gamma = 90.0

	Atom	X	Y	Z	X	Y	Z
1	Co	0.0000	0.0000	0.0000	0.0000	0.0000	0.0000
2	Co	1.7379	1.7379	0.0000	0.2500	0.2500	0.0000
3	Co	0.0000	3.4758	0.0000	0.0000	0.5000	0.0000
4	Co	1.7379	5.2138	0.0000	0.2500	0.7500	0.0000
5	Co	3.4758	0.0000	0.0000	0.5000	0.0000	0.0000
6	Co	5.2138	1.7379	0.0000	0.7500	0.2500	0.0000
7	Co	3.4758	3.4758	0.0000	0.5000	0.5000	0.0000
8	Co	5.2138	5.2138	0.0000	0.7500	0.7500	0.0000
9	Co	0.0111	-0.0079	3.5559	0.0016	-0.0011	0.1620
10	Co	1.7385	1.7379	3.5560	0.2501	0.2500	0.1620
11	Co	0.0073	3.4809	3.5505	0.0011	0.5007	0.1617
12	Co	1.7245	5.2167	3.5616	0.2481	0.7504	0.1622
13	Co	1.7327	3.4660	5.2294	0.2493	0.4986	0.2382
14	Co	5.2213	5.2129	3.5438	0.7511	0.7499	0.1614
15	Co	3.4795	1.7872	5.2311	0.5005	0.2571	0.2383
16	Co	5.2080	3.4761	5.2250	0.7492	0.5000	0.2380
17	Ni	1.7379	0.0000	1.7379	0.2500	0.0000	0.0792
18	Ni	0.0000	1.7379	1.7379	0.0000	0.2500	0.0792
19	Ni	1.7379	3.4758	1.7379	0.2500	0.5000	0.0792
20	Ni	0.0000	5.2138	1.7379	0.0000	0.7500	0.0792
21	Ni	5.2138	0.0000	1.7379	0.7500	0.0000	0.0792
22	Ni	3.4758	1.7379	1.7379	0.5000	0.2500	0.0792
23	Ni	5.2138	3.4758	1.7379	0.7500	0.5000	0.0792
24	Ni	3.4758	5.2138	1.7379	0.5000	0.7500	0.0792
25	Ni	1.7524	0.0133	5.2306	0.2521	0.0019	0.2383
26	Ni	6.9442	1.7507	5.2199	0.9989	0.2518	0.2378
27	Ni	3.4719	3.4831	3.4871	0.4994	0.5010	0.1589
28	Ni	6.9415	5.2059	5.2278	0.9985	0.7489	0.2381
29	Ni	5.2037	6.9488	5.2193	0.7486	0.9996	0.2378
30	Ni	3.4761	5.1725	5.2779	0.5000	0.7441	0.2404
31	Ni	3.4732	6.9417	3.5456	0.4996	0.9986	0.1615
32	Ni	5.2138	1.7331	3.5324	0.7500	0.2493	0.1609
33	O	3.4545	3.4705	7.5800	0.4969	0.4992	0.3453
34	C	3.4663	3.4735	6.3645	0.4986	0.4997	0.2899

/db/jmorales/CoNi-alloy/Slabs/Segregation/Ni-CoNi(001)/Ni-CoNi(001)-3-Co

a =				6.9516692132			
b =				6.9516692132			
c =				21.9516692133			
alpha =				90.0			
beta =				90.0			
gamma =				90.0			
	Atom	X	Y	Z	X	Y	Z
1	Co	0.0000	0.0000	0.0000	0.0000	0.0000	0.0000
2	Co	1.7379	1.7379	0.0000	0.2500	0.2500	0.0000
3	Co	0.0000	3.4758	0.0000	0.0000	0.5000	0.0000
4	Co	1.7379	5.2138	0.0000	0.2500	0.7500	0.0000
5	Co	3.4758	0.0000	0.0000	0.5000	0.0000	0.0000
6	Co	5.2138	1.7379	0.0000	0.7500	0.2500	0.0000
7	Co	3.4758	3.4758	0.0000	0.5000	0.5000	0.0000
8	Co	5.2138	5.2138	0.0000	0.7500	0.7500	0.0000
9	Co	0.0071	-0.0097	3.5567	0.0010	-0.0014	0.1620
10	Co	1.7305	1.7393	3.5483	0.2489	0.2502	0.1616
11	Co	0.0069	3.4823	3.5526	0.0010	0.5009	0.1618
12	Co	1.7270	5.2143	3.5614	0.2484	0.7501	0.1622
13	Co	1.7451	3.4630	5.2029	0.2510	0.4982	0.2370
14	Co	5.2199	5.2103	3.5481	0.7509	0.7495	0.1616
15	Co	3.4716	1.7670	5.1957	0.4994	0.2542	0.2367
16	Co	5.2040	3.4783	5.2031	0.7486	0.5004	0.2370
17	Ni	1.7379	0.0000	1.7379	0.2500	0.0000	0.0792
18	Ni	0.0000	1.7379	1.7379	0.0000	0.2500	0.0792
19	Ni	1.7379	3.4758	1.7379	0.2500	0.5000	0.0792
20	Ni	0.0000	5.2138	1.7379	0.0000	0.7500	0.0792
21	Ni	5.2138	0.0000	1.7379	0.7500	0.0000	0.0792
22	Ni	3.4758	1.7379	1.7379	0.5000	0.2500	0.0792
23	Ni	5.2138	3.4758	1.7379	0.7500	0.5000	0.0792
24	Ni	3.4758	5.2138	1.7379	0.5000	0.7500	0.0792
25	Ni	1.7522	0.0127	5.2327	0.2520	0.0018	0.2384
26	Ni	-0.0060	1.7549	5.2331	-0.0009	0.2524	0.2384
27	Ni	3.4726	3.4826	3.5141	0.4995	0.5010	0.1601
28	Ni	6.9439	5.2026	5.2395	0.9989	0.7484	0.2387
29	Ni	5.1986	6.9506	5.2283	0.7478	0.9999	0.2382
30	Ni	3.4758	5.1849	5.2345	0.5000	0.7458	0.2385
31	Ni	3.4748	6.9475	3.5326	0.4999	0.9994	0.1609
32	Ni	5.2197	1.7369	3.5308	0.7509	0.2499	0.1608

/db/jmorales/CoNi-alloy/Slabs/Segregation/CoNi(001)/CoNi(001)-1-Ni-CO

a =	6.9516692132
b =	6.9516692132
c =	21.9516692133
alpha =	90.0
beta =	90.0
gamma =	90.0

	Atom	X	Y	Z	X	Y	Z
1	Ni	0.0000	0.0000	0.0000	0.0000	0.0000	0.0000
2	Ni	0.0000	3.4758	0.0000	0.0000	0.5000	0.0000
3	Ni	3.4758	0.0000	0.0000	0.5000	0.0000	0.0000
4	Ni	3.4758	3.4758	0.0000	0.5000	0.5000	0.0000
5	Ni	0.0000	1.7379	1.7379	0.0000	0.2500	0.0792
6	Ni	0.0000	5.2138	1.7379	0.0000	0.7500	0.0792
7	Ni	3.4758	1.7379	1.7379	0.5000	0.2500	0.0792
8	Ni	3.4758	5.2138	1.7379	0.5000	0.7500	0.0792
9	Ni	-0.0059	0.0003	3.4995	-0.0008	0.0000	0.1594
10	Ni	0.0049	3.4756	3.5022	0.0007	0.5000	0.1595
11	Ni	3.4813	0.0000	3.5137	0.5008	0.0000	0.1601
12	Ni	1.7650	3.4759	5.2704	0.2539	0.5000	0.2401
13	Ni	0.0001	1.7267	5.2011	0.0000	0.2484	0.2369
14	Ni	0.0001	5.2255	5.2009	0.0000	0.7517	0.2369
15	Ni	3.4751	1.7362	5.2495	0.4999	0.2498	0.2391
16	Ni	3.4752	5.2159	5.2488	0.4999	0.7503	0.2391
17	Co	1.7379	1.7379	0.0000	0.2500	0.2500	0.0000
18	Co	1.7379	5.2138	0.0000	0.2500	0.7500	0.0000
19	Co	5.2138	1.7379	0.0000	0.7500	0.2500	0.0000
20	Co	5.2138	5.2138	0.0000	0.7500	0.7500	0.0000
21	Co	1.7379	0.0000	1.7379	0.2500	0.0000	0.0792
22	Co	1.7379	3.4758	1.7379	0.2500	0.5000	0.0792
23	Co	5.2138	0.0000	1.7379	0.7500	0.0000	0.0792
24	Co	5.2138	3.4758	1.7379	0.7500	0.5000	0.0792
25	Co	1.7415	1.7269	3.5288	0.2505	0.2484	0.1608
26	Co	1.7414	5.2253	3.5286	0.2505	0.7517	0.1607
27	Co	5.2119	1.7439	3.5531	0.7497	0.2509	0.1619
28	Co	5.2119	5.2080	3.5528	0.7497	0.7492	0.1618
29	Co	1.7208	0.0000	5.2034	0.2475	0.0000	0.2370
30	Co	3.4817	3.4759	3.4651	0.5009	0.5000	0.1579
31	Co	5.2294	0.0000	5.2035	0.7523	0.0000	0.2370
32	Co	5.1929	3.4759	5.2215	0.7470	0.5000	0.2379
33	O	3.4787	3.4774	7.5599	0.5004	0.5002	0.3444
34	C	3.4568	3.4766	6.3446	0.4973	0.5001	0.2890

/db/jmorales/CoNi-alloy/Slabs/Islanding/CoNi(111)/CoNi(111)-2-Ni-CO

a = 9.831199646
b = 9.831199646075708
c = 21.0202999115
alpha = 90.0
beta = 90.0
gamma = 120.0

	Atom	X	Y	Z	X	Y	Z
1	Ni	0.0000	0.0000	0.0000	0.0000	0.0000	0.0000
2	Ni	-1.2289	2.1285	0.0000	0.0000	0.2500	0.0000
3	Ni	-2.4578	4.2570	0.0000	0.0000	0.5000	0.0000
4	Ni	-3.6867	6.3856	0.0000	0.0000	0.7500	0.0000
5	Ni	4.9156	0.0000	0.0000	0.5000	0.0000	0.0000
6	Ni	3.6867	2.1285	0.0000	0.5000	0.2500	0.0000
7	Ni	2.4578	4.2570	0.0000	0.5000	0.5000	0.0000
8	Ni	1.2289	6.3856	0.0000	0.5000	0.7500	0.0000
9	Ni	2.4578	1.4190	2.0068	0.3333	0.1667	0.0955
10	Ni	1.2289	3.5475	2.0068	0.3333	0.4167	0.0955
11	Ni	0.0000	5.6760	2.0068	0.3333	0.6667	0.0955
12	Ni	-1.2289	7.8045	2.0068	0.3333	0.9167	0.0955
13	Ni	7.3734	1.4190	2.0068	0.8333	0.1667	0.0955
14	Ni	6.1445	3.5475	2.0068	0.8333	0.4167	0.0955
15	Ni	4.9156	5.6760	2.0068	0.8333	0.6667	0.0955
16	Ni	3.6867	7.8045	2.0068	0.8333	0.9167	0.0955
17	Ni	0.0378	2.8589	4.0036	0.1717	0.3358	0.1905
18	Ni	1.2635	0.7298	4.0082	0.1714	0.0857	0.1907
19	Ni	-2.4304	7.1148	4.0164	0.1706	0.8357	0.1911
20	Ni	-1.1958	4.9837	4.0081	0.1710	0.5854	0.1907
21	Ni	4.9440	2.8559	4.0235	0.6706	0.3354	0.1914
22	Ni	6.1709	0.7304	4.0073	0.6706	0.0858	0.1906
23	Ni	2.4937	7.1142	4.0092	0.6714	0.8356	0.1907
24	Ni	3.7172	4.9810	4.0230	0.6706	0.5850	0.1914
25	Ni	-1.1833	2.1516	6.0361	0.0060	0.2527	0.2872
26	Ni	-2.4153	4.2934	6.0335	0.0065	0.5043	0.2870
27	Ni	-3.6424	6.4119	6.0278	0.0061	0.7531	0.2868
28	Ni	4.9719	0.0392	6.0269	0.5080	0.0046	0.2867
29	Ni	3.7492	2.1638	6.0870	0.5084	0.2541	0.2896
30	Ni	2.5206	4.2900	6.0840	0.5083	0.5039	0.2894
31	Ni	1.2779	2.1479	6.0936	0.2561	0.2523	0.2899
32	Ni	0.0303	4.3154	6.0371	0.2565	0.5068	0.2872
33	Co	1.2289	2.1285	0.0000	0.2500	0.2500	0.0000
34	Co	2.4578	0.0000	0.0000	0.2500	0.0000	0.0000
35	Co	-1.2289	6.3856	0.0000	0.2500	0.7500	0.0000
36	Co	0.0000	4.2570	0.0000	0.2500	0.5000	0.0000
37	Co	6.1445	2.1285	0.0000	0.7500	0.2500	0.0000
38	Co	7.3734	0.0000	0.0000	0.7500	0.0000	0.0000
39	Co	3.6867	6.3856	0.0000	0.7500	0.7500	0.0000
40	Co	4.9156	4.2570	0.0000	0.7500	0.5000	0.0000
41	Co	-1.2289	3.5475	2.0068	0.0833	0.4167	0.0955

42	Co	0.0000	1.4190	2.0068	0.0833	0.1667	0.0955
43	Co	-3.6867	7.8045	2.0068	0.0833	0.9167	0.0955
44	Co	-2.4578	5.6760	2.0068	0.0833	0.6667	0.0955
45	Co	3.6867	3.5475	2.0068	0.5833	0.4167	0.0955
46	Co	4.9156	1.4190	2.0068	0.5833	0.1667	0.0955
47	Co	1.2289	7.8045	2.0068	0.5833	0.9167	0.0955
48	Co	2.4578	5.6760	2.0068	0.5833	0.6667	0.0955
49	Co	3.7066	0.7321	4.0578	0.4200	0.0860	0.1930
50	Co	2.4964	2.8605	4.0027	0.4219	0.3360	0.1904
51	Co	1.2654	4.9862	4.0584	0.4215	0.5856	0.1931
52	Co	0.0325	7.1105	4.0588	0.4209	0.8351	0.1931
53	Co	8.6396	0.7276	4.0543	0.9215	0.0855	0.1929
54	Co	7.4049	2.8558	4.0646	0.9209	0.3354	0.1934
55	Co	6.1731	4.9806	4.0562	0.9204	0.5850	0.1930
56	Co	4.9413	7.1187	4.0552	0.9207	0.8361	0.1929
57	Co	2.5312	-0.0002	6.0041	0.2575	-0.0000	0.2856
58	Co	-1.1689	6.4381	6.0118	0.2592	0.7562	0.2860
59	Co	6.2171	2.1587	6.0150	0.7592	0.2535	0.2862
60	Co	7.4385	0.0267	6.0118	0.7582	0.0031	0.2860
61	Co	3.7462	6.4336	6.0183	0.7589	0.7556	0.2863
62	Co	5.0053	4.3139	6.0119	0.7625	0.5067	0.2860
63	Co	0.0272	0.0133	5.9925	0.0036	0.0016	0.2851
64	Co	1.2808	6.4078	5.9910	0.5066	0.7526	0.2850
65	O	2.4937	2.8569	8.6184	0.4214	0.3355	0.4100
66	C	2.4868	2.8443	7.4251	0.4200	0.3341	0.3532

/db/jmorales/CoNi-alloy/Slabs/Segregation/CoNi(001)/CoNi(001)-2-Co

a =	6.9516692132
b =	6.9516692132
c =	21.9516692133
alpha =	90.0
beta =	90.0
gamma =	90.0

	Atom	X	Y	Z	X	Y	Z
1	Ni	0.0000	0.0000	0.0000	0.0000	0.0000	0.0000
2	Ni	0.0000	3.4758	0.0000	0.0000	0.5000	0.0000
3	Ni	3.4758	0.0000	0.0000	0.5000	0.0000	0.0000
4	Ni	3.4758	3.4758	0.0000	0.5000	0.5000	0.0000
5	Ni	0.0000	1.7379	1.7379	0.0000	0.2500	0.0792
6	Ni	0.0000	5.2138	1.7379	0.0000	0.7500	0.0792
7	Ni	3.4758	1.7379	1.7379	0.5000	0.2500	0.0792
8	Ni	3.4758	5.2138	1.7379	0.5000	0.7500	0.0792
9	Ni	6.9493	-0.0015	3.5224	0.9997	-0.0002	0.1605
10	Ni	6.9490	3.4759	3.5220	0.9996	0.5000	0.1604
11	Ni	3.4740	-0.0001	3.5031	0.4997	-0.0000	0.1596
12	Ni	3.4744	3.4752	3.5036	0.4998	0.4999	0.1596
13	Ni	0.0148	1.7373	5.2329	0.0021	0.2499	0.2384
14	Ni	0.0070	5.2128	5.2337	0.0010	0.7499	0.2384
15	Ni	1.7360	1.7374	3.5309	0.2497	0.2499	0.1608
16	Ni	1.7364	5.2131	3.5320	0.2498	0.7499	0.1609
17	Co	1.7379	1.7379	0.0000	0.2500	0.2500	0.0000
18	Co	1.7379	5.2138	0.0000	0.2500	0.7500	0.0000
19	Co	5.2138	1.7379	0.0000	0.7500	0.2500	0.0000
20	Co	5.2138	5.2138	0.0000	0.7500	0.7500	0.0000
21	Co	1.7379	0.0000	1.7379	0.2500	0.0000	0.0792
22	Co	1.7379	3.4758	1.7379	0.2500	0.5000	0.0792
23	Co	5.2138	0.0000	1.7379	0.7500	0.0000	0.0792
24	Co	5.2138	3.4758	1.7379	0.7500	0.5000	0.0792
25	Co	3.4617	1.7368	5.2118	0.4980	0.2498	0.2374
26	Co	5.2122	1.7374	3.5585	0.7498	0.2499	0.1621
27	Co	5.2120	5.2133	3.5605	0.7498	0.7499	0.1622
28	Co	1.7786	6.9474	5.2540	0.2559	0.9994	0.2393
29	Co	1.7790	3.4781	5.2530	0.2559	0.5003	0.2393
30	Co	5.1758	0.0054	5.2382	0.7445	0.0008	0.2386
31	Co	5.1758	3.4692	5.2376	0.7445	0.4990	0.2386
32	Co	3.4697	5.2129	5.2124	0.4991	0.7499	0.2374

/db/jmorales/CoNi-alloy/Slabs/Segregation/CoNi(111)/CoNi(111)-2-Co

a =			9.831199646				
b =			9.831199646075708				
c =			21.0202999115				
alpha =			90.0				
beta =			90.0				
gamma =			120.0				
	Atom	X	Y	Z	X	Y	Z
1	Ni	0.0000	0.0000	0.0000	0.0000	0.0000	0.0000
2	Ni	-1.2289	2.1285	0.0000	0.0000	0.2500	0.0000
3	Ni	-2.4578	4.2570	0.0000	0.0000	0.5000	0.0000
4	Ni	-3.6867	6.3856	0.0000	0.0000	0.7500	0.0000
5	Ni	4.9156	0.0000	0.0000	0.5000	0.0000	0.0000
6	Ni	3.6867	2.1285	0.0000	0.5000	0.2500	0.0000
7	Ni	2.4578	4.2570	0.0000	0.5000	0.5000	0.0000
8	Ni	1.2289	6.3856	0.0000	0.5000	0.7500	0.0000
9	Ni	2.4578	1.4190	2.0068	0.3333	0.1667	0.0955
10	Ni	1.2289	3.5475	2.0068	0.3333	0.4167	0.0955
11	Ni	0.0000	5.6760	2.0068	0.3333	0.6667	0.0955
12	Ni	-1.2289	7.8045	2.0068	0.3333	0.9167	0.0955
13	Ni	7.3734	1.4190	2.0068	0.8333	0.1667	0.0955
14	Ni	6.1445	3.5475	2.0068	0.8333	0.4167	0.0955
15	Ni	4.9156	5.6760	2.0068	0.8333	0.6667	0.0955
16	Ni	3.6867	7.8045	2.0068	0.8333	0.9167	0.0955
17	Ni	0.0300	2.8515	4.0198	0.1705	0.3349	0.1912
18	Ni	1.2535	0.7309	4.0194	0.1704	0.0858	0.1912
19	Ni	-2.4304	7.1076	4.0158	0.1702	0.8348	0.1910
20	Ni	-1.2057	4.9865	4.0164	0.1702	0.5857	0.1911
21	Ni	4.9430	2.8594	4.0157	0.6707	0.3358	0.1910
22	Ni	6.1816	0.7262	4.0180	0.6714	0.0853	0.1911
23	Ni	2.4893	7.1220	4.0190	0.6715	0.8365	0.1912
24	Ni	3.7178	4.9827	4.0143	0.6708	0.5852	0.1910
25	Ni	0.0445	0.0242	6.0380	0.0059	0.0028	0.2872
26	Ni	-1.1876	2.1588	6.0389	0.0060	0.2536	0.2873
27	Ni	-2.4178	4.2848	6.0349	0.0057	0.5033	0.2871
28	Ni	-3.6446	6.4105	6.0342	0.0057	0.7529	0.2871
29	Ni	4.9634	0.0267	6.0259	0.5064	0.0031	0.2867
30	Ni	1.2734	6.4164	6.0258	0.5063	0.7536	0.2867
31	Ni	3.7104	0.7278	4.0399	0.4202	0.0855	0.1922
32	Ni	1.2553	4.9811	4.0391	0.4202	0.5850	0.1922
33	Co	1.2289	2.1285	0.0000	0.2500	0.2500	0.0000
34	Co	2.4578	0.0000	0.0000	0.2500	0.0000	0.0000
35	Co	-1.2289	6.3856	0.0000	0.2500	0.7500	0.0000
36	Co	0.0000	4.2570	0.0000	0.2500	0.5000	0.0000
37	Co	6.1445	2.1285	0.0000	0.7500	0.2500	0.0000
38	Co	7.3734	0.0000	0.0000	0.7500	0.0000	0.0000
39	Co	3.6867	6.3856	0.0000	0.7500	0.7500	0.0000
40	Co	4.9156	4.2570	0.0000	0.7500	0.5000	0.0000
41	Co	-1.2289	3.5475	2.0068	0.0833	0.4167	0.0955

42	Co	0.0000	1.4190	2.0068	0.0833	0.1667	0.0955
43	Co	-3.6867	7.8045	2.0068	0.0833	0.9167	0.0955
44	Co	-2.4578	5.6760	2.0068	0.0833	0.6667	0.0955
45	Co	3.6867	3.5475	2.0068	0.5833	0.4167	0.0955
46	Co	4.9156	1.4190	2.0068	0.5833	0.1667	0.0955
47	Co	1.2289	7.8045	2.0068	0.5833	0.9167	0.0955
48	Co	2.4578	5.6760	2.0068	0.5833	0.6667	0.0955
49	Co	2.4873	2.8566	4.0761	0.4208	0.3355	0.1939
50	Co	0.0251	7.1111	4.0548	0.4202	0.8352	0.1929
51	Co	8.6288	0.7273	4.0640	0.9204	0.0854	0.1933
52	Co	7.4003	2.8553	4.0580	0.9204	0.3354	0.1931
53	Co	6.1702	4.9826	4.0536	0.9202	0.5852	0.1928
54	Co	4.9428	7.1109	4.0584	0.9204	0.8352	0.1931
55	Co	1.2929	2.1675	6.0329	0.2588	0.2546	0.2870
56	Co	2.5130	0.0488	6.0323	0.2585	0.0057	0.2870
57	Co	-1.1642	6.4258	6.0166	0.2589	0.7547	0.2862
58	Co	0.0689	4.2830	6.0328	0.2585	0.5030	0.2870
59	Co	6.1939	2.1681	6.0164	0.7574	0.2547	0.2862
60	Co	7.4286	0.0352	6.0316	0.7577	0.0041	0.2869
61	Co	3.7434	6.4118	6.0175	0.7573	0.7531	0.2863
62	Co	4.9638	4.2883	6.0265	0.7567	0.5037	0.2867
63	Co	3.7265	2.1483	6.0042	0.5052	0.2523	0.2856
64	Co	2.4923	4.2849	6.0032	0.5051	0.5033	0.2856

/db/jmorales/CoNi-alloy/Slabs/Islanding/CoNi(111)/CoNi(111)-2-Co-CO

a =	9.831199646			
b =	9.831199646075708			
c =	21.0202999115			
alpha =	90.0			
beta =	90.0			
gamma =	120.0			

	Atom	X	Y	Z	X	Y	Z
1	Ni	0.0000	0.0000	0.0000	0.0000	0.0000	0.0000
2	Ni	-1.2289	2.1285	0.0000	0.0000	0.2500	0.0000
3	Ni	-2.4578	4.2570	0.0000	0.0000	0.5000	0.0000
4	Ni	-3.6867	6.3856	0.0000	0.0000	0.7500	0.0000
5	Ni	4.9156	0.0000	0.0000	0.5000	0.0000	0.0000
6	Ni	3.6867	2.1285	0.0000	0.5000	0.2500	0.0000
7	Ni	2.4578	4.2570	0.0000	0.5000	0.5000	0.0000
8	Ni	1.2289	6.3856	0.0000	0.5000	0.7500	0.0000
9	Ni	2.4578	1.4190	2.0068	0.3333	0.1667	0.0955
10	Ni	1.2289	3.5475	2.0068	0.3333	0.4167	0.0955
11	Ni	0.0000	5.6760	2.0068	0.3333	0.6667	0.0955
12	Ni	-1.2289	7.8045	2.0068	0.3333	0.9167	0.0955
13	Ni	7.3734	1.4190	2.0068	0.8333	0.1667	0.0955
14	Ni	6.1445	3.5475	2.0068	0.8333	0.4167	0.0955
15	Ni	4.9156	5.6760	2.0068	0.8333	0.6667	0.0955
16	Ni	3.6867	7.8045	2.0068	0.8333	0.9167	0.0955
17	Ni	0.0345	2.8556	4.0087	0.1712	0.3354	0.1907
18	Ni	1.2617	0.7283	4.0018	0.1711	0.0855	0.1904
19	Ni	-2.4268	7.1126	4.0073	0.1708	0.8354	0.1906
20	Ni	-1.1998	4.9829	4.0125	0.1706	0.5853	0.1909
21	Ni	4.9409	2.8571	4.0212	0.6704	0.3356	0.1913
22	Ni	6.1745	0.7269	4.0140	0.6707	0.0854	0.1910
23	Ni	2.4868	7.1112	3.9999	0.6706	0.8352	0.1903
24	Ni	3.7110	4.9762	4.0069	0.6697	0.5845	0.1906
25	Ni	0.0543	0.0329	6.0227	0.0075	0.0039	0.2865
26	Ni	-1.1754	2.1635	6.0220	0.0075	0.2541	0.2865
27	Ni	-2.4063	4.2786	6.0226	0.0065	0.5025	0.2865
28	Ni	-3.6344	6.4098	6.0253	0.0067	0.7528	0.2866
29	Ni	4.9701	0.0372	6.0190	0.5077	0.0044	0.2863
30	Ni	1.2912	6.4147	6.0199	0.5080	0.7534	0.2864
31	Ni	2.5255	0.0165	6.0381	0.2579	0.0019	0.2873
32	Ni	3.7461	6.4182	6.0455	0.7580	0.7538	0.2876
33	Co	1.2289	2.1285	0.0000	0.2500	0.2500	0.0000
34	Co	2.4578	0.0000	0.0000	0.2500	0.0000	0.0000
35	Co	-1.2289	6.3856	0.0000	0.2500	0.7500	0.0000
36	Co	0.0000	4.2570	0.0000	0.2500	0.5000	0.0000
37	Co	6.1445	2.1285	0.0000	0.7500	0.2500	0.0000
38	Co	7.3734	0.0000	0.0000	0.7500	0.0000	0.0000
39	Co	3.6867	6.3856	0.0000	0.7500	0.7500	0.0000
40	Co	4.9156	4.2570	0.0000	0.7500	0.5000	0.0000
41	Co	-1.2289	3.5475	2.0068	0.0833	0.4167	0.0955

42	Co	0.0000	1.4190	2.0068	0.0833	0.1667	0.0955
43	Co	-3.6867	7.8045	2.0068	0.0833	0.9167	0.0955
44	Co	-2.4578	5.6760	2.0068	0.0833	0.6667	0.0955
45	Co	3.6867	3.5475	2.0068	0.5833	0.4167	0.0955
46	Co	4.9156	1.4190	2.0068	0.5833	0.1667	0.0955
47	Co	1.2289	7.8045	2.0068	0.5833	0.9167	0.0955
48	Co	2.4578	5.6760	2.0068	0.5833	0.6667	0.0955
49	Co	3.7163	0.7347	4.0385	0.4212	0.0863	0.1921
50	Co	2.4859	2.8524	4.0688	0.4204	0.3350	0.1936
51	Co	1.2590	4.9790	4.0488	0.4205	0.5848	0.1926
52	Co	0.0254	7.1094	4.0524	0.4201	0.8350	0.1928
53	Co	8.6336	0.7233	4.0603	0.9207	0.0850	0.1932
54	Co	7.4027	2.8548	4.0440	0.9206	0.3353	0.1924
55	Co	6.1672	4.9821	4.0550	0.9199	0.5852	0.1929
56	Co	4.9534	7.1136	4.0395	0.9216	0.8355	0.1922
57	Co	1.2868	2.1622	6.0667	0.2579	0.2540	0.2886
58	Co	-1.1463	6.4124	6.0097	0.2600	0.7532	0.2859
59	Co	0.0576	4.3087	6.0077	0.2589	0.5061	0.2858
60	Co	6.2168	2.1707	6.0109	0.7598	0.2550	0.2860
61	Co	7.4322	0.0484	6.0138	0.7588	0.0057	0.2861
62	Co	4.9912	4.2893	6.0080	0.7596	0.5038	0.2858
63	Co	3.7571	2.1410	6.0375	0.5079	0.2515	0.2872
64	Co	2.5093	4.3033	6.0425	0.5080	0.5054	0.2875
65	O	2.5433	2.8833	8.5893	0.4280	0.3386	0.4086
66	C	2.4870	2.8677	7.3926	0.4214	0.3368	0.3517

/db/jmorales/CoNi-alloy/Slabs/CoNi(111)

a =		9.831199646
b =		9.831199645902503
c =		21.020299912
alpha =		90.0
beta =		90.0
gamma =		120.0

	Atom	X	Y	Z	X	Y	Z
1	Ni	0.0000	0.0000	0.0000	0.0000	0.0000	0.0000
2	Ni	-1.2289	2.1285	0.0000	0.0000	0.2500	0.0000
3	Ni	-2.4578	4.2570	0.0000	-0.0000	0.5000	0.0000
4	Ni	-3.6867	6.3856	0.0000	-0.0000	0.7500	0.0000
5	Ni	4.9156	0.0000	0.0000	0.5000	0.0000	0.0000
6	Ni	3.6867	2.1285	0.0000	0.5000	0.2500	0.0000
7	Ni	2.4578	4.2570	0.0000	0.5000	0.5000	0.0000
8	Ni	1.2289	6.3856	0.0000	0.5000	0.7500	0.0000
9	Ni	2.4578	1.4190	2.0068	0.3333	0.1667	0.0955
10	Ni	1.2289	3.5475	2.0068	0.3333	0.4167	0.0955
11	Ni	0.0000	5.6760	2.0068	0.3333	0.6667	0.0955
12	Ni	-1.2289	7.8046	2.0068	0.3333	0.9167	0.0955
13	Ni	7.3734	1.4190	2.0068	0.8333	0.1667	0.0955
14	Ni	6.1445	3.5475	2.0068	0.8333	0.4167	0.0955
15	Ni	4.9156	5.6760	2.0068	0.8333	0.6667	0.0955
16	Ni	3.6867	7.8046	2.0068	0.8333	0.9167	0.0955
17	Ni	-0.0000	2.8380	4.0136	0.1667	0.3333	0.1909
18	Ni	1.2289	0.7095	4.0136	0.1667	0.0833	0.1909
19	Ni	-2.4578	7.0951	4.0136	0.1667	0.8333	0.1909
20	Ni	-1.2289	4.9666	4.0136	0.1667	0.5833	0.1909
21	Ni	4.9156	2.8380	4.0136	0.6667	0.3333	0.1909
22	Ni	6.1445	0.7095	4.0136	0.6667	0.0833	0.1909
23	Ni	2.4578	7.0951	4.0136	0.6667	0.8333	0.1909
24	Ni	3.6867	4.9666	4.0136	0.6667	0.5833	0.1909
25	Ni	0.0000	0.0000	6.0202	0.0000	0.0000	0.2864
26	Ni	-1.2289	2.1285	6.0202	0.0000	0.2500	0.2864
27	Ni	-2.4578	4.2570	6.0202	-0.0000	0.5000	0.2864
28	Ni	-3.6867	6.3856	6.0202	-0.0000	0.7500	0.2864
29	Ni	4.9156	0.0000	6.0202	0.5000	0.0000	0.2864
30	Ni	3.6867	2.1285	6.0202	0.5000	0.2500	0.2864
31	Ni	2.4578	4.2570	6.0202	0.5000	0.5000	0.2864
32	Ni	1.2289	6.3856	6.0202	0.5000	0.7500	0.2864
33	Co	1.2289	2.1285	0.0000	0.2500	0.2500	0.0000
34	Co	2.4578	0.0000	0.0000	0.2500	0.0000	0.0000
35	Co	-1.2289	6.3856	0.0000	0.2500	0.7500	0.0000
36	Co	0.0000	4.2570	0.0000	0.2500	0.5000	0.0000
37	Co	6.1445	2.1285	0.0000	0.7500	0.2500	0.0000
38	Co	7.3734	0.0000	0.0000	0.7500	0.0000	0.0000
39	Co	3.6867	6.3856	0.0000	0.7500	0.7500	0.0000
40	Co	4.9156	4.2570	0.0000	0.7500	0.5000	0.0000
41	Co	-1.2289	3.5475	2.0068	0.0833	0.4167	0.0955

42	Co	0.0000	1.4190	2.0068	0.0833	0.1667	0.0955
43	Co	-3.6867	7.8046	2.0068	0.0833	0.9167	0.0955
44	Co	-2.4578	5.6760	2.0068	0.0833	0.6667	0.0955
45	Co	3.6867	3.5475	2.0068	0.5833	0.4167	0.0955
46	Co	4.9156	1.4190	2.0068	0.5833	0.1667	0.0955
47	Co	1.2289	7.8046	2.0068	0.5833	0.9167	0.0955
48	Co	2.4578	5.6760	2.0068	0.5833	0.6667	0.0955
49	Co	3.6867	0.7095	4.0136	0.4167	0.0833	0.1909
50	Co	2.4578	2.8380	4.0136	0.4167	0.3333	0.1909
51	Co	1.2289	4.9666	4.0136	0.4167	0.5833	0.1909
52	Co	-0.0000	7.0951	4.0136	0.4167	0.8333	0.1909
53	Co	8.6023	0.7095	4.0136	0.9167	0.0833	0.1909
54	Co	7.3734	2.8380	4.0136	0.9167	0.3333	0.1909
55	Co	6.1445	4.9666	4.0136	0.9167	0.5833	0.1909
56	Co	4.9156	7.0951	4.0136	0.9167	0.8333	0.1909
57	Co	1.2289	2.1285	6.0202	0.2500	0.2500	0.2864
58	Co	2.4578	0.0000	6.0202	0.2500	0.0000	0.2864
59	Co	-1.2289	6.3856	6.0202	0.2500	0.7500	0.2864
60	Co	0.0000	4.2570	6.0202	0.2500	0.5000	0.2864
61	Co	6.1445	2.1285	6.0202	0.7500	0.2500	0.2864
62	Co	7.3734	0.0000	6.0202	0.7500	0.0000	0.2864
63	Co	3.6867	6.3856	6.0202	0.7500	0.7500	0.2864
64	Co	4.9156	4.2570	6.0202	0.7500	0.5000	0.2864

/db/jmorales/CoNi-alloy/Slabs/Islanding/CoNi(111)/CoNi(111)-1-Co-CO

a =			9.831199646				
b =			9.831199646075708				
c =			21.0202999115				
alpha =			90.0				
beta =			90.0				
gamma =			120.0				
	Atom	X	Y	Z	X	Y	Z
1	Ni	0.0000	0.0000	0.0000	0.0000	0.0000	0.0000
2	Ni	-1.2289	2.1285	0.0000	0.0000	0.2500	0.0000
3	Ni	-2.4578	4.2570	0.0000	0.0000	0.5000	0.0000
4	Ni	-3.6867	6.3856	0.0000	0.0000	0.7500	0.0000
5	Ni	4.9156	0.0000	0.0000	0.5000	0.0000	0.0000
6	Ni	3.6867	2.1285	0.0000	0.5000	0.2500	0.0000
7	Ni	2.4578	4.2570	0.0000	0.5000	0.5000	0.0000
8	Ni	1.2289	6.3856	0.0000	0.5000	0.7500	0.0000
9	Ni	2.4578	1.4190	2.0068	0.3333	0.1667	0.0955
10	Ni	1.2289	3.5475	2.0068	0.3333	0.4167	0.0955
11	Ni	0.0000	5.6760	2.0068	0.3333	0.6667	0.0955
12	Ni	-1.2289	7.8045	2.0068	0.3333	0.9167	0.0955
13	Ni	7.3734	1.4190	2.0068	0.8333	0.1667	0.0955
14	Ni	6.1445	3.5475	2.0068	0.8333	0.4167	0.0955
15	Ni	4.9156	5.6760	2.0068	0.8333	0.6667	0.0955
16	Ni	3.6867	7.8045	2.0068	0.8333	0.9167	0.0955
17	Ni	0.0358	2.8560	4.0087	0.1714	0.3354	0.1907
18	Ni	1.2637	0.7249	3.9960	0.1711	0.0851	0.1901
19	Ni	-2.4283	7.1147	4.0057	0.1708	0.8356	0.1906
20	Ni	-1.1975	4.9839	4.0159	0.1709	0.5854	0.1910
21	Ni	4.9404	2.8585	4.0208	0.6704	0.3357	0.1913
22	Ni	6.1765	0.7274	4.0113	0.6710	0.0854	0.1908
23	Ni	2.4891	7.1099	4.0115	0.6707	0.8351	0.1908
24	Ni	3.7143	4.9796	4.0138	0.6702	0.5849	0.1910
25	Ni	0.0528	0.0364	6.0215	0.0075	0.0043	0.2865
26	Ni	-1.1759	2.1632	6.0213	0.0074	0.2541	0.2865
27	Ni	-2.4058	4.2819	6.0220	0.0067	0.5029	0.2865
28	Ni	-3.6317	6.4115	6.0288	0.0071	0.7530	0.2868
29	Ni	4.9775	0.0379	6.0191	0.5085	0.0044	0.2863
30	Ni	2.5239	4.2966	6.0789	0.5090	0.5046	0.2892
31	Ni	1.2925	6.4162	6.0213	0.5083	0.7536	0.2865
32	Ni	2.5250	0.0209	6.0412	0.2581	0.0025	0.2874
33	Co	1.2289	2.1285	0.0000	0.2500	0.2500	0.0000
34	Co	2.4578	0.0000	0.0000	0.2500	0.0000	0.0000
35	Co	-1.2289	6.3856	0.0000	0.2500	0.7500	0.0000
36	Co	0.0000	4.2570	0.0000	0.2500	0.5000	0.0000
37	Co	6.1445	2.1285	0.0000	0.7500	0.2500	0.0000
38	Co	7.3734	0.0000	0.0000	0.7500	0.0000	0.0000
39	Co	3.6867	6.3856	0.0000	0.7500	0.7500	0.0000
40	Co	4.9156	4.2570	0.0000	0.7500	0.5000	0.0000
41	Co	-1.2289	3.5475	2.0068	0.0833	0.4167	0.0955

42	Co	0.0000	1.4190	2.0068	0.0833	0.1667	0.0955
43	Co	-3.6867	7.8045	2.0068	0.0833	0.9167	0.0955
44	Co	-2.4578	5.6760	2.0068	0.0833	0.6667	0.0955
45	Co	3.6867	3.5475	2.0068	0.5833	0.4167	0.0955
46	Co	4.9156	1.4190	2.0068	0.5833	0.1667	0.0955
47	Co	1.2289	7.8045	2.0068	0.5833	0.9167	0.0955
48	Co	2.4578	5.6760	2.0068	0.5833	0.6667	0.0955
49	Co	3.7185	0.7324	4.0443	0.4212	0.0860	0.1924
50	Co	2.4897	2.8453	4.0605	0.4203	0.3342	0.1932
51	Co	1.2582	4.9787	4.0516	0.4204	0.5848	0.1927
52	Co	0.0277	7.1114	4.0529	0.4204	0.8353	0.1928
53	Co	8.6357	0.7272	4.0558	0.9211	0.0854	0.1929
54	Co	7.4029	2.8542	4.0451	0.9206	0.3352	0.1924
55	Co	6.1708	4.9842	4.0569	0.9204	0.5854	0.1930
56	Co	4.9475	7.1093	4.0582	0.9208	0.8350	0.1931
57	Co	1.2826	2.1617	6.0584	0.2574	0.2539	0.2882
58	Co	-1.1474	6.4167	6.0113	0.2601	0.7537	0.2860
59	Co	0.0534	4.3205	6.0068	0.2592	0.5075	0.2858
60	Co	6.2172	2.1727	6.0123	0.7600	0.2552	0.2860
61	Co	7.4449	0.0471	6.0146	0.7600	0.0055	0.2861
62	Co	3.7586	6.4279	6.0123	0.7598	0.7550	0.2860
63	Co	5.0031	4.3040	6.0119	0.7617	0.5055	0.2860
64	Co	3.7544	2.1536	6.0413	0.5084	0.2529	0.2874
65	O	2.5129	2.8579	8.6030	0.4234	0.3357	0.4093
66	C	2.4539	2.8415	7.4088	0.4165	0.3337	0.3525

/db/jmorales/CoNi-alloy/Slabs/Segregation/Co-CoNi(001)/Co-CoNi(001)-3-Ni-CO

a =				6.9516692132			
b =				6.9516692132			
c =				21.9516692133			
alpha =				90.0			
beta =				90.0			
gamma =				90.0			
	Atom	X	Y	Z	X	Y	Z
1	Ni	0.0000	0.0000	0.0000	0.0000	0.0000	0.0000
2	Ni	1.7379	1.7379	0.0000	0.2500	0.2500	0.0000
3	Ni	0.0000	3.4758	0.0000	0.0000	0.5000	0.0000
4	Ni	1.7379	5.2138	0.0000	0.2500	0.7500	0.0000
5	Ni	3.4758	0.0000	0.0000	0.5000	0.0000	0.0000
6	Ni	5.2138	1.7379	0.0000	0.7500	0.2500	0.0000
7	Ni	3.4758	3.4758	0.0000	0.5000	0.5000	0.0000
8	Ni	5.2138	5.2138	0.0000	0.7500	0.7500	0.0000
9	Ni	0.0007	-0.0033	3.5058	0.0001	-0.0005	0.1597
10	Ni	1.7276	1.7340	3.5142	0.2485	0.2494	0.1601
11	Ni	0.0011	3.4804	3.5438	0.0002	0.5007	0.1614
12	Ni	1.7309	5.2151	3.5071	0.2490	0.7502	0.1598
13	Ni	1.7643	3.4860	5.2825	0.2538	0.5015	0.2406
14	Ni	5.2177	5.2149	3.5088	0.7506	0.7502	0.1598
15	Ni	3.4783	1.7385	5.2743	0.5003	0.2501	0.2403
16	Ni	5.1800	3.4899	5.2775	0.7451	0.5020	0.2404
17	Co	1.7379	0.0000	1.7379	0.2500	0.0000	0.0792
18	Co	0.0000	1.7379	1.7379	0.0000	0.2500	0.0792
19	Co	1.7379	3.4758	1.7379	0.2500	0.5000	0.0792
20	Co	0.0000	5.2138	1.7379	0.0000	0.7500	0.0792
21	Co	5.2138	0.0000	1.7379	0.7500	0.0000	0.0792
22	Co	3.4758	1.7379	1.7379	0.5000	0.2500	0.0792
23	Co	5.2138	3.4758	1.7379	0.7500	0.5000	0.0792
24	Co	3.4758	5.2138	1.7379	0.5000	0.7500	0.0792
25	Co	1.7294	6.9246	5.2151	0.2488	0.9961	0.2376
26	Co	6.9277	1.7023	5.2375	0.9965	0.2449	0.2386
27	Co	3.4753	3.4789	3.4506	0.4999	0.5004	0.1572
28	Co	0.0047	5.2703	5.2500	0.0007	0.7581	0.2392
29	Co	5.2206	6.9286	5.2090	0.7510	0.9967	0.2373
30	Co	3.4684	5.2398	5.2432	0.4989	0.7537	0.2389
31	Co	3.4739	6.9379	3.5078	0.4997	0.9980	0.1598
32	Co	5.2291	1.7340	3.5160	0.7522	0.2494	0.1602
33	O	3.4703	3.4692	7.5814	0.4992	0.4990	0.3454
34	C	3.4666	3.4252	6.3683	0.4987	0.4927	0.2901

/db/jmorales/CoNi-alloy/Slabs/Segregation/CoNi(001)/CoNi(001)-3-Ni

a = 6.9516692132
b = 6.9516692132
c = 21.9516692133
alpha = 90.0
beta = 90.0
gamma = 90.0

	Atom	X	Y	Z	X	Y	Z
1	Ni	0.0000	0.0000	0.0000	0.0000	0.0000	0.0000
2	Ni	0.0000	3.4758	0.0000	0.0000	0.5000	0.0000
3	Ni	3.4758	0.0000	0.0000	0.5000	0.0000	0.0000
4	Ni	3.4758	3.4758	0.0000	0.5000	0.5000	0.0000
5	Ni	0.0000	1.7379	1.7379	0.0000	0.2500	0.0792
6	Ni	0.0000	5.2138	1.7379	0.0000	0.7500	0.0792
7	Ni	3.4758	1.7379	1.7379	0.5000	0.2500	0.0792
8	Ni	3.4758	5.2138	1.7379	0.5000	0.7500	0.0792
9	Ni	3.4832	0.0000	3.4976	0.5011	0.0000	0.1593
10	Ni	1.7514	3.4759	5.2053	0.2519	0.5000	0.2371
11	Ni	6.9476	1.7325	5.2109	0.9994	0.2492	0.2374
12	Ni	6.9476	5.2193	5.2109	0.9994	0.7508	0.2374
13	Ni	3.4798	1.7244	5.2015	0.5006	0.2481	0.2370
14	Ni	3.4796	5.2274	5.2016	0.5005	0.7520	0.2370
15	Ni	5.2024	3.4759	5.2062	0.7484	0.5000	0.2372
16	Ni	1.7310	-0.0000	5.2096	0.2490	-0.0000	0.2373
17	Co	1.7379	1.7379	0.0000	0.2500	0.2500	0.0000
18	Co	1.7379	5.2138	0.0000	0.2500	0.7500	0.0000
19	Co	5.2138	1.7379	0.0000	0.7500	0.2500	0.0000
20	Co	5.2138	5.2138	0.0000	0.7500	0.7500	0.0000
21	Co	1.7379	0.0000	1.7379	0.2500	0.0000	0.0792
22	Co	1.7379	3.4758	1.7379	0.2500	0.5000	0.0792
23	Co	5.2138	0.0000	1.7379	0.7500	0.0000	0.0792
24	Co	5.2138	3.4758	1.7379	0.7500	0.5000	0.0792
25	Co	1.7284	1.7372	3.5265	0.2486	0.2499	0.1606
26	Co	1.7286	5.2147	3.5265	0.2487	0.7501	0.1607
27	Co	5.2220	1.7425	3.5273	0.7512	0.2507	0.1607
28	Co	5.2220	5.2092	3.5275	0.7512	0.7493	0.1607
29	Co	3.4746	3.4760	3.5031	0.4998	0.5000	0.1596
30	Co	5.2334	0.0001	5.1782	0.7528	0.0000	0.2359
31	Co	0.0052	3.4759	3.5212	0.0007	0.5000	0.1604
32	Co	6.9430	-0.0000	3.5041	0.9988	-0.0000	0.1596

/db/jmorales/CoNi-alloy/Slabs/Segregation/CoNi(001)/CoNi(001)-3-Co

a =				6.9516692132			
b =				6.9516692132			
c =				21.9516692133			
alpha =				90.0			
beta =				90.0			
gamma =				90.0			
	Atom	X	Y	Z	X	Y	Z
1	Ni	0.0000	0.0000	0.0000	0.0000	0.0000	0.0000
2	Ni	0.0000	3.4758	0.0000	0.0000	0.5000	0.0000
3	Ni	3.4758	0.0000	0.0000	0.5000	0.0000	0.0000
4	Ni	3.4758	3.4758	0.0000	0.5000	0.5000	0.0000
5	Ni	0.0000	1.7379	1.7379	0.0000	0.2500	0.0792
6	Ni	0.0000	5.2138	1.7379	0.0000	0.7500	0.0792
7	Ni	3.4758	1.7379	1.7379	0.5000	0.2500	0.0792
8	Ni	3.4758	5.2138	1.7379	0.5000	0.7500	0.0792
9	Ni	0.0001	0.0010	3.5291	0.0000	0.0001	0.1608
10	Ni	0.0001	3.4750	3.5282	0.0000	0.4999	0.1607
11	Ni	3.4728	0.0017	3.5107	0.4996	0.0002	0.1599
12	Ni	3.4723	3.4741	3.5112	0.4995	0.4997	0.1599
13	Ni	0.0079	5.2139	5.2505	0.0011	0.7500	0.2392
14	Ni	1.7315	1.7377	3.5318	0.2491	0.2500	0.1609
15	Ni	1.7390	5.2136	3.5468	0.2502	0.7500	0.1616
16	Ni	5.2161	1.7376	3.5453	0.7503	0.2500	0.1615
17	Co	1.7379	1.7379	0.0000	0.2500	0.2500	0.0000
18	Co	1.7379	5.2138	0.0000	0.2500	0.7500	0.0000
19	Co	5.2138	1.7379	0.0000	0.7500	0.2500	0.0000
20	Co	5.2138	5.2138	0.0000	0.7500	0.7500	0.0000
21	Co	1.7379	0.0000	1.7379	0.2500	0.0000	0.0792
22	Co	1.7379	3.4758	1.7379	0.2500	0.5000	0.0792
23	Co	5.2138	0.0000	1.7379	0.7500	0.0000	0.0792
24	Co	5.2138	3.4758	1.7379	0.7500	0.5000	0.0792
25	Co	3.4711	1.7371	5.2276	0.4993	0.2499	0.2381
26	Co	5.2132	5.2139	3.5493	0.7499	0.7500	0.1617
27	Co	1.7621	0.0301	5.2576	0.2535	0.0043	0.2395
28	Co	1.7620	3.4445	5.2565	0.2535	0.4955	0.2395
29	Co	5.1881	-0.0101	5.2481	0.7463	-0.0015	0.2391
30	Co	5.1889	3.4860	5.2474	0.7464	0.5015	0.2390
31	Co	3.4790	5.2147	5.2292	0.5005	0.7501	0.2382
32	Co	0.0034	1.7378	5.2466	0.0005	0.2500	0.2390

/db/jmorales/CoNi-alloy/Slabs/Segregation/CoNi(001)/CoNi(001)-2-Ni-CO

a =	6.9516692132
b =	6.9516692132
c =	21.9516692133
alpha =	90.0
beta =	90.0
gamma =	90.0

	Atom	X	Y	Z	X	Y	Z
1	Ni	0.0000	0.0000	0.0000	0.0000	0.0000	0.0000
2	Ni	0.0000	3.4758	0.0000	0.0000	0.5000	0.0000
3	Ni	3.4758	0.0000	0.0000	0.5000	0.0000	0.0000
4	Ni	3.4758	3.4758	0.0000	0.5000	0.5000	0.0000
5	Ni	0.0000	1.7379	1.7379	0.0000	0.2500	0.0792
6	Ni	0.0000	5.2138	1.7379	0.0000	0.7500	0.0792
7	Ni	3.4758	1.7379	1.7379	0.5000	0.2500	0.0792
8	Ni	3.4758	5.2138	1.7379	0.5000	0.7500	0.0792
9	Ni	-0.0007	0.0002	3.4981	-0.0001	0.0000	0.1594
10	Ni	3.4754	0.0002	3.5088	0.4999	0.0000	0.1598
11	Ni	1.7523	3.4761	5.2559	0.2521	0.5000	0.2394
12	Ni	-0.0001	1.7103	5.2012	-0.0000	0.2460	0.2369
13	Ni	-0.0002	5.2416	5.2009	-0.0000	0.7540	0.2369
14	Ni	3.4755	1.7441	5.2511	0.5000	0.2509	0.2392
15	Ni	3.4756	5.2080	5.2507	0.5000	0.7492	0.2392
16	Ni	5.1993	3.4760	5.2552	0.7479	0.5000	0.2394
17	Co	1.7379	1.7379	0.0000	0.2500	0.2500	0.0000
18	Co	1.7379	5.2138	0.0000	0.2500	0.7500	0.0000
19	Co	5.2138	1.7379	0.0000	0.7500	0.2500	0.0000
20	Co	5.2138	5.2138	0.0000	0.7500	0.7500	0.0000
21	Co	1.7379	0.0000	1.7379	0.2500	0.0000	0.0792
22	Co	1.7379	3.4758	1.7379	0.2500	0.5000	0.0792
23	Co	5.2138	0.0000	1.7379	0.7500	0.0000	0.0792
24	Co	5.2138	3.4758	1.7379	0.7500	0.5000	0.0792
25	Co	1.7355	1.7368	3.5294	0.2496	0.2498	0.1608
26	Co	1.7353	5.2151	3.5293	0.2496	0.7502	0.1608
27	Co	5.2158	1.7369	3.5294	0.7503	0.2499	0.1608
28	Co	5.2158	5.2151	3.5291	0.7503	0.7502	0.1608
29	Co	1.7370	0.0001	5.1932	0.2499	0.0000	0.2366
30	Co	3.4754	3.4759	3.4614	0.4999	0.5000	0.1577
31	Co	5.2144	0.0002	5.1928	0.7501	0.0000	0.2366
32	Co	-0.0001	3.4758	3.5052	-0.0000	0.5000	0.1597
33	O	3.4756	3.4769	7.5538	0.5000	0.5002	0.3441
34	C	3.4758	3.4761	6.3397	0.5000	0.5000	0.2888

/db/jmorales/CoNi-alloy/Slabs/Islanding/CoNi(001)/CoNi(001)-2-Co-CO

a =			6.9516692132				
b =			6.9516692132				
c =			21.9516692133				
alpha =			90.0				
beta =			90.0				
gamma =			90.0				
	Atom	X	Y	Z	X	Y	Z
1	Ni	0.0000	0.0000	0.0000	0.0000	0.0000	0.0000
2	Ni	0.0000	3.4758	0.0000	0.0000	0.5000	0.0000
3	Ni	3.4758	0.0000	0.0000	0.5000	0.0000	0.0000
4	Ni	3.4758	3.4758	0.0000	0.5000	0.5000	0.0000
5	Ni	0.0000	1.7379	1.7379	0.0000	0.2500	0.0792
6	Ni	0.0000	5.2138	1.7379	0.0000	0.7500	0.0792
7	Ni	3.4758	1.7379	1.7379	0.5000	0.2500	0.0792
8	Ni	3.4758	5.2138	1.7379	0.5000	0.7500	0.0792
9	Ni	-0.0003	-0.0002	3.5025	-0.0000	-0.0000	0.1596
10	Ni	6.9511	3.4764	3.5014	0.9999	0.5001	0.1595
11	Ni	3.4754	-0.0003	3.5075	0.4999	-0.0000	0.1598
12	Ni	3.4756	3.4759	3.4511	0.5000	0.5000	0.1572
13	Ni	-0.0002	1.7532	5.2132	-0.0000	0.2522	0.2375
14	Ni	-0.0007	5.1973	5.2147	-0.0001	0.7476	0.2376
15	Ni	1.7475	-0.0002	5.2151	0.2514	-0.0000	0.2376
16	Ni	5.2026	-0.0000	5.2168	0.7484	-0.0000	0.2377
17	Co	1.7379	1.7379	0.0000	0.2500	0.2500	0.0000
18	Co	1.7379	5.2138	0.0000	0.2500	0.7500	0.0000
19	Co	5.2138	1.7379	0.0000	0.7500	0.2500	0.0000
20	Co	5.2138	5.2138	0.0000	0.7500	0.7500	0.0000
21	Co	1.7379	0.0000	1.7379	0.2500	0.0000	0.0792
22	Co	1.7379	3.4758	1.7379	0.2500	0.5000	0.0792
23	Co	5.2138	0.0000	1.7379	0.7500	0.0000	0.0792
24	Co	5.2138	3.4758	1.7379	0.7500	0.5000	0.0792
25	Co	1.7422	1.7453	3.5472	0.2506	0.2511	0.1616
26	Co	1.7429	5.2062	3.5476	0.2507	0.7489	0.1616
27	Co	5.2091	1.7464	3.5476	0.7493	0.2512	0.1616
28	Co	5.2081	5.2049	3.5485	0.7492	0.7487	0.1617
29	Co	1.7685	3.4767	5.2278	0.2544	0.5001	0.2382
30	Co	5.1807	3.4760	5.2295	0.7452	0.5000	0.2382
31	Co	3.4742	1.7663	5.2147	0.4998	0.2541	0.2376
32	Co	3.4750	5.1846	5.2149	0.4999	0.7458	0.2376
33	O	3.4681	3.4673	7.5832	0.4989	0.4988	0.3455
34	C	3.4732	3.4766	6.3673	0.4996	0.5001	0.2901

/db/jmorales/CoNi-alloy/Slabs/Segregation/CoNi(111)/CoNi(111)-3-Ni-CO

a = 9.831199646
b = 9.831199646075708
c = 21.0202999115
alpha = 90.0
beta = 90.0
gamma = 120.0

	Atom	X	Y	Z	X	Y	Z
1	Ni	0.0000	0.0000	0.0000	0.0000	0.0000	0.0000
2	Ni	-1.2289	2.1285	0.0000	0.0000	0.2500	0.0000
3	Ni	-2.4578	4.2570	0.0000	0.0000	0.5000	0.0000
4	Ni	-3.6867	6.3856	0.0000	0.0000	0.7500	0.0000
5	Ni	4.9156	0.0000	0.0000	0.5000	0.0000	0.0000
6	Ni	3.6867	2.1285	0.0000	0.5000	0.2500	0.0000
7	Ni	2.4578	4.2570	0.0000	0.5000	0.5000	0.0000
8	Ni	1.2289	6.3856	0.0000	0.5000	0.7500	0.0000
9	Ni	2.4578	1.4190	2.0068	0.3333	0.1667	0.0955
10	Ni	1.2289	3.5475	2.0068	0.3333	0.4167	0.0955
11	Ni	0.0000	5.6760	2.0068	0.3333	0.6667	0.0955
12	Ni	-1.2289	7.8045	2.0068	0.3333	0.9167	0.0955
13	Ni	7.3734	1.4190	2.0068	0.8333	0.1667	0.0955
14	Ni	6.1445	3.5475	2.0068	0.8333	0.4167	0.0955
15	Ni	4.9156	5.6760	2.0068	0.8333	0.6667	0.0955
16	Ni	3.6867	7.8045	2.0068	0.8333	0.9167	0.0955
17	Ni	-1.2007	4.9800	4.0021	0.1703	0.5849	0.1904
18	Ni	4.9423	2.8556	4.0175	0.6704	0.3354	0.1911
19	Ni	6.1716	0.7278	4.0058	0.6705	0.0855	0.1906
20	Ni	2.4862	7.1102	4.0098	0.6704	0.8351	0.1908
21	Ni	3.7122	4.9810	4.0189	0.6701	0.5850	0.1912
22	Ni	0.0385	0.0233	6.0370	0.0053	0.0027	0.2872
23	Ni	-1.1856	2.1546	6.0287	0.0059	0.2531	0.2868
24	Ni	-2.4202	4.2805	6.0276	0.0052	0.5028	0.2868
25	Ni	-3.6490	6.4079	6.0339	0.0051	0.7526	0.2871
26	Ni	4.9658	0.0302	6.0170	0.5069	0.0035	0.2862
27	Ni	3.7502	2.1655	6.0757	0.5086	0.2543	0.2890
28	Ni	2.5172	4.2935	6.0783	0.5082	0.5043	0.2892
29	Ni	1.2840	6.4132	6.0157	0.5072	0.7532	0.2862
30	Ni	1.2712	2.1554	6.0826	0.2559	0.2532	0.2894
31	Ni	2.5147	-0.0004	6.0169	0.2558	-0.0001	0.2862
32	Ni	0.0273	4.3091	6.0155	0.2558	0.5061	0.2862
33	Co	1.2289	2.1285	0.0000	0.2500	0.2500	0.0000
34	Co	2.4578	0.0000	0.0000	0.2500	0.0000	0.0000
35	Co	-1.2289	6.3856	0.0000	0.2500	0.7500	0.0000
36	Co	0.0000	4.2570	0.0000	0.2500	0.5000	0.0000
37	Co	6.1445	2.1285	0.0000	0.7500	0.2500	0.0000
38	Co	7.3734	0.0000	0.0000	0.7500	0.0000	0.0000
39	Co	3.6867	6.3856	0.0000	0.7500	0.7500	0.0000
40	Co	4.9156	4.2570	0.0000	0.7500	0.5000	0.0000
41	Co	-1.2289	3.5475	2.0068	0.0833	0.4167	0.0955

42	Co	0.0000	1.4190	2.0068	0.0833	0.1667	0.0955
43	Co	-3.6867	7.8045	2.0068	0.0833	0.9167	0.0955
44	Co	-2.4578	5.6760	2.0068	0.0833	0.6667	0.0955
45	Co	3.6867	3.5475	2.0068	0.5833	0.4167	0.0955
46	Co	4.9156	1.4190	2.0068	0.5833	0.1667	0.0955
47	Co	1.2289	7.8045	2.0068	0.5833	0.9167	0.0955
48	Co	2.4578	5.6760	2.0068	0.5833	0.6667	0.0955
49	Co	3.7050	0.7330	4.0331	0.4199	0.0861	0.1919
50	Co	2.4815	2.8493	4.0019	0.4197	0.3347	0.1904
51	Co	1.2617	4.9743	4.0362	0.4205	0.5842	0.1920
52	Co	0.0215	7.1109	4.0461	0.4198	0.8352	0.1925
53	Co	8.6427	0.7320	4.0552	0.9221	0.0860	0.1929
54	Co	7.4128	2.8569	4.0471	0.9218	0.3356	0.1925
55	Co	6.1781	4.9907	4.0474	0.9215	0.5862	0.1925
56	Co	4.9546	7.1209	4.0500	0.9221	0.8364	0.1927
57	Co	-1.1803	6.4243	5.9845	0.2572	0.7545	0.2847
58	Co	6.2096	2.1591	6.0027	0.7584	0.2536	0.2856
59	Co	7.4318	0.0330	6.0076	0.7579	0.0039	0.2858
60	Co	3.7436	6.4270	6.0059	0.7582	0.7549	0.2857
61	Co	5.0009	4.3045	5.9983	0.7615	0.5056	0.2854
62	Co	1.2522	0.7259	4.0035	0.1700	0.0853	0.1905
63	Co	-2.4285	7.1106	4.0197	0.1706	0.8352	0.1912
64	Co	0.0284	2.8519	3.9954	0.1704	0.3350	0.1901
65	O	2.4977	2.8518	8.6060	0.4215	0.3350	0.4094
66	C	2.4943	2.8506	7.4129	0.4211	0.3348	0.3527

/db/jmorales/CoNi-alloy/Slabs/Segregation/CoNi(111)/CoNi(111)-3-Co-CO

a =	9.831199646	
b =	9.831199646075708	
c =	21.0202999115	
alpha =	90.0	
beta =	90.0	
gamma =	120.0	

	Atom	X	Y	Z	X	Y	Z
1	Ni	0.0000	0.0000	0.0000	0.0000	0.0000	0.0000
2	Ni	-1.2289	2.1285	0.0000	0.0000	0.2500	0.0000
3	Ni	-2.4578	4.2570	0.0000	0.0000	0.5000	0.0000
4	Ni	-3.6867	6.3856	0.0000	0.0000	0.7500	0.0000
5	Ni	4.9156	0.0000	0.0000	0.5000	0.0000	0.0000
6	Ni	3.6867	2.1285	0.0000	0.5000	0.2500	0.0000
7	Ni	2.4578	4.2570	0.0000	0.5000	0.5000	0.0000
8	Ni	1.2289	6.3856	0.0000	0.5000	0.7500	0.0000
9	Ni	2.4578	1.4190	2.0068	0.3333	0.1667	0.0955
10	Ni	1.2289	3.5475	2.0068	0.3333	0.4167	0.0955
11	Ni	0.0000	5.6760	2.0068	0.3333	0.6667	0.0955
12	Ni	-1.2289	7.8045	2.0068	0.3333	0.9167	0.0955
13	Ni	7.3734	1.4190	2.0068	0.8333	0.1667	0.0955
14	Ni	6.1445	3.5475	2.0068	0.8333	0.4167	0.0955
15	Ni	4.9156	5.6760	2.0068	0.8333	0.6667	0.0955
16	Ni	3.6867	7.8045	2.0068	0.8333	0.9167	0.0955
17	Ni	0.0277	2.8513	4.0149	0.1703	0.3349	0.1910
18	Ni	1.2527	0.7266	4.0171	0.1701	0.0853	0.1911
19	Ni	-2.4401	7.1057	4.0221	0.1691	0.8346	0.1913
20	Ni	-1.2132	4.9760	4.0278	0.1688	0.5844	0.1916
21	Ni	4.9396	2.8600	4.0241	0.6704	0.3359	0.1914
22	Ni	6.1788	0.7320	4.0177	0.6715	0.0860	0.1911
23	Ni	2.4850	7.1094	4.0168	0.6703	0.8350	0.1911
24	Ni	3.7206	4.9724	4.0234	0.6705	0.5840	0.1914
25	Ni	0.0362	0.0218	6.0360	0.0050	0.0026	0.2871
26	Ni	-1.1891	2.1505	6.0373	0.0053	0.2526	0.2872
27	Ni	-2.4225	4.2776	6.0426	0.0048	0.5024	0.2875
28	Ni	-3.6498	6.4087	6.0417	0.0051	0.7527	0.2874
29	Ni	4.9595	0.0232	6.0187	0.5058	0.0027	0.2863
30	Ni	3.7005	0.7383	4.0363	0.4198	0.0867	0.1920
31	Ni	1.2619	4.9673	4.0453	0.4201	0.5834	0.1924
32	Ni	0.0276	7.1069	4.0324	0.4202	0.8347	0.1918
33	Co	1.2289	2.1285	0.0000	0.2500	0.2500	0.0000
34	Co	2.4578	0.0000	0.0000	0.2500	0.0000	0.0000
35	Co	-1.2289	6.3856	0.0000	0.2500	0.7500	0.0000
36	Co	0.0000	4.2570	0.0000	0.2500	0.5000	0.0000
37	Co	6.1445	2.1285	0.0000	0.7500	0.2500	0.0000
38	Co	7.3734	0.0000	0.0000	0.7500	0.0000	0.0000
39	Co	3.6867	6.3856	0.0000	0.7500	0.7500	0.0000
40	Co	4.9156	4.2570	0.0000	0.7500	0.5000	0.0000
41	Co	-1.2289	3.5475	2.0068	0.0833	0.4167	0.0955

42	Co	0.0000	1.4190	2.0068	0.0833	0.1667	0.0955
43	Co	-3.6867	7.8045	2.0068	0.0833	0.9167	0.0955
44	Co	-2.4578	5.6760	2.0068	0.0833	0.6667	0.0955
45	Co	3.6867	3.5475	2.0068	0.5833	0.4167	0.0955
46	Co	4.9156	1.4190	2.0068	0.5833	0.1667	0.0955
47	Co	1.2289	7.8045	2.0068	0.5833	0.9167	0.0955
48	Co	2.4578	5.6760	2.0068	0.5833	0.6667	0.0955
49	Co	2.4816	2.8512	4.0729	0.4199	0.3349	0.1938
50	Co	8.6275	0.7227	4.0621	0.9200	0.0849	0.1932
51	Co	7.3969	2.8507	4.0620	0.9198	0.3348	0.1932
52	Co	6.1692	4.9817	4.0616	0.9201	0.5851	0.1932
53	Co	4.9350	7.1080	4.0603	0.9194	0.8349	0.1932
54	Co	1.2739	2.1564	6.0727	0.2562	0.2533	0.2889
55	Co	2.5124	0.0126	6.0222	0.2563	0.0015	0.2865
56	Co	-1.1614	6.4100	6.0273	0.2583	0.7529	0.2867
57	Co	0.0380	4.3028	6.0315	0.2566	0.5054	0.2869
58	Co	6.1898	2.1542	6.0189	0.7561	0.2530	0.2863
59	Co	7.4172	0.0245	6.0308	0.7559	0.0029	0.2869
60	Co	3.7321	6.4064	6.0207	0.7558	0.7525	0.2864
61	Co	4.9748	4.2980	6.0227	0.7584	0.5048	0.2865
62	Co	3.7319	2.1499	6.0441	0.5058	0.2525	0.2875
63	Co	2.4995	4.2827	6.0456	0.5057	0.5030	0.2876
64	Co	1.2627	6.4068	5.9918	0.5047	0.7525	0.2850
65	O	2.5277	2.8634	8.6107	0.4253	0.3363	0.4096
66	C	2.4963	2.8536	7.4140	0.4215	0.3352	0.3527

/db/jmorales/CoNi-alloy/Slabs/Islanding/CoNi(001)/CoNi(001)-1-Co

a =		6.9516692132
b =		6.9516692132
c =		21.9516692133
alpha =		90.0
beta =		90.0
gamma =		90.0

	Atom	X	Y	Z	X	Y	Z
1	Ni	0.0000	0.0000	0.0000	0.0000	0.0000	0.0000
2	Ni	0.0000	3.4758	0.0000	0.0000	0.5000	0.0000
3	Ni	3.4758	0.0000	0.0000	0.5000	0.0000	0.0000
4	Ni	3.4758	3.4758	0.0000	0.5000	0.5000	0.0000
5	Ni	0.0000	1.7379	1.7379	0.0000	0.2500	0.0792
6	Ni	0.0000	5.2138	1.7379	0.0000	0.7500	0.0792
7	Ni	3.4758	1.7379	1.7379	0.5000	0.2500	0.0792
8	Ni	3.4758	5.2138	1.7379	0.5000	0.7500	0.0792
9	Ni	6.9515	0.0002	3.5045	1.0000	0.0000	0.1596
10	Ni	-0.0026	3.4775	3.5027	-0.0004	0.5002	0.1596
11	Ni	3.4754	-0.0059	3.5000	0.4999	-0.0008	0.1594
12	Ni	3.4763	3.4856	3.4931	0.5001	0.5014	0.1591
13	Ni	6.9455	1.7501	5.2105	0.9991	0.2518	0.2374
14	Ni	6.9501	5.2065	5.2139	0.9998	0.7490	0.2375
15	Ni	3.4796	5.2020	5.2107	0.5005	0.7483	0.2374
16	Ni	1.7490	0.0056	5.2286	0.2516	0.0008	0.2382
17	Co	1.7379	1.7379	0.0000	0.2500	0.2500	0.0000
18	Co	1.7379	5.2138	0.0000	0.2500	0.7500	0.0000
19	Co	5.2138	1.7379	0.0000	0.7500	0.2500	0.0000
20	Co	5.2138	5.2138	0.0000	0.7500	0.7500	0.0000
21	Co	1.7379	0.0000	1.7379	0.2500	0.0000	0.0792
22	Co	1.7379	3.4758	1.7379	0.2500	0.5000	0.0792
23	Co	5.2138	0.0000	1.7379	0.7500	0.0000	0.0792
24	Co	5.2138	3.4758	1.7379	0.7500	0.5000	0.0792
25	Co	1.7436	1.7491	3.5473	0.2508	0.2516	0.1616
26	Co	1.7360	5.2033	3.5392	0.2497	0.7485	0.1612
27	Co	5.2075	1.7373	3.5565	0.7491	0.2499	0.1620
28	Co	5.2144	5.2147	3.5516	0.7501	0.7501	0.1618
29	Co	1.7492	3.4690	5.2130	0.2516	0.4990	0.2375
30	Co	5.2038	0.0026	5.2130	0.7486	0.0004	0.2375
31	Co	5.1954	3.4671	5.2120	0.7474	0.4987	0.2374
32	Co	3.4818	1.7522	5.1912	0.5009	0.2521	0.2365

/db/jmorales/CoNi-alloy/Slabs/Segregation/CoNi(001)/CoNi(001)-1-Ni

a =			6.9516692132				
b =			6.9516692132				
c =			21.9516692133				
alpha =			90.0				
beta =			90.0				
gamma =			90.0				
	Atom	X	Y	Z	X	Y	Z
1	Ni	0.0000	0.0000	0.0000	0.0000	0.0000	0.0000
2	Ni	0.0000	3.4758	0.0000	0.0000	0.5000	0.0000
3	Ni	3.4758	0.0000	0.0000	0.5000	0.0000	0.0000
4	Ni	3.4758	3.4758	0.0000	0.5000	0.5000	0.0000
5	Ni	0.0000	1.7379	1.7379	0.0000	0.2500	0.0792
6	Ni	0.0000	5.2138	1.7379	0.0000	0.7500	0.0792
7	Ni	3.4758	1.7379	1.7379	0.5000	0.2500	0.0792
8	Ni	3.4758	5.2138	1.7379	0.5000	0.7500	0.0792
9	Ni	0.0005	0.0000	3.4984	0.0001	0.0000	0.1594
10	Ni	-0.0011	3.4758	3.5058	-0.0002	0.5000	0.1597
11	Ni	3.4788	0.0000	3.5000	0.5004	0.0000	0.1594
12	Ni	1.7533	3.4758	5.2206	0.2522	0.5000	0.2378
13	Ni	-0.0052	1.7308	5.2063	-0.0007	0.2490	0.2372
14	Ni	-0.0052	5.2208	5.2063	-0.0007	0.7510	0.2372
15	Ni	3.4858	1.7058	5.2112	0.5014	0.2454	0.2374
16	Ni	3.4858	5.2458	5.2112	0.5014	0.7546	0.2374
17	Co	1.7379	1.7379	0.0000	0.2500	0.2500	0.0000
18	Co	1.7379	5.2138	0.0000	0.2500	0.7500	0.0000
19	Co	5.2138	1.7379	0.0000	0.7500	0.2500	0.0000
20	Co	5.2138	5.2138	0.0000	0.7500	0.7500	0.0000
21	Co	1.7379	0.0000	1.7379	0.2500	0.0000	0.0792
22	Co	1.7379	3.4758	1.7379	0.2500	0.5000	0.0792
23	Co	5.2138	0.0000	1.7379	0.7500	0.0000	0.0792
24	Co	5.2138	3.4758	1.7379	0.7500	0.5000	0.0792
25	Co	1.7439	1.7314	3.5395	0.2509	0.2491	0.1612
26	Co	1.7439	5.2203	3.5395	0.2509	0.7509	0.1612
27	Co	5.2128	1.7414	3.5450	0.7499	0.2505	0.1615
28	Co	5.2128	5.2103	3.5450	0.7499	0.7495	0.1615
29	Co	1.7234	0.0000	5.2106	0.2479	0.0000	0.2374
30	Co	3.4754	3.4758	3.5141	0.4999	0.5000	0.1601
31	Co	5.2340	0.0000	5.2072	0.7529	0.0000	0.2372
32	Co	5.1843	3.4758	5.1980	0.7458	0.5000	0.2368

/db/jmorales/CoNi-alloy/Slabs/Segregation/Ni-CoNi(001)/Ni-CoNi(001)-1-Co-CO

a =	6.9516692132
b =	6.9516692132
c =	21.9516692133
alpha =	90.0
beta =	90.0
gamma =	90.0

	Atom	X	Y	Z	X	Y	Z
1	Co	0.0000	0.0000	0.0000	0.0000	0.0000	0.0000
2	Co	1.7379	1.7379	0.0000	0.2500	0.2500	0.0000
3	Co	0.0000	3.4758	0.0000	0.0000	0.5000	0.0000
4	Co	1.7379	5.2138	0.0000	0.2500	0.7500	0.0000
5	Co	3.4758	0.0000	0.0000	0.5000	0.0000	0.0000
6	Co	5.2138	1.7379	0.0000	0.7500	0.2500	0.0000
7	Co	3.4758	3.4758	0.0000	0.5000	0.5000	0.0000
8	Co	5.2138	5.2138	0.0000	0.7500	0.7500	0.0000
9	Co	0.0018	-0.0000	3.5631	0.0003	-0.0000	0.1623
10	Co	1.7337	1.7363	3.5542	0.2494	0.2498	0.1619
11	Co	-0.0034	3.4757	3.5390	-0.0005	0.5000	0.1612
12	Co	1.7335	5.2153	3.5542	0.2494	0.7502	0.1619
13	Co	3.4758	0.0001	3.5450	0.5000	0.0000	0.1615
14	Co	5.2264	1.7282	3.5424	0.7518	0.2486	0.1614
15	Co	1.7339	3.4758	5.2174	0.2494	0.5000	0.2377
16	Co	5.2264	5.2235	3.5422	0.7518	0.7514	0.1614
17	Ni	1.7379	0.0000	1.7379	0.2500	0.0000	0.0792
18	Ni	0.0000	1.7379	1.7379	0.0000	0.2500	0.0792
19	Ni	1.7379	3.4758	1.7379	0.2500	0.5000	0.0792
20	Ni	0.0000	5.2138	1.7379	0.0000	0.7500	0.0792
21	Ni	5.2138	0.0000	1.7379	0.7500	0.0000	0.0792
22	Ni	3.4758	1.7379	1.7379	0.5000	0.2500	0.0792
23	Ni	5.2138	3.4758	1.7379	0.7500	0.5000	0.0792
24	Ni	3.4758	5.2138	1.7379	0.5000	0.7500	0.0792
25	Ni	1.7495	-0.0000	5.2156	0.2517	-0.0000	0.2376
26	Ni	-0.0035	1.7474	5.2160	-0.0005	0.2514	0.2376
27	Ni	3.4742	3.4759	3.4837	0.4998	0.5000	0.1587
28	Ni	-0.0035	5.2043	5.2161	-0.0005	0.7486	0.2376
29	Ni	5.2022	0.0000	5.2146	0.7483	0.0000	0.2375
30	Ni	3.4783	1.7711	5.2555	0.5004	0.2548	0.2394
31	Ni	5.1958	3.4759	5.2650	0.7474	0.5000	0.2398
32	Ni	3.4782	5.1807	5.2555	0.5003	0.7453	0.2394
33	O	3.4266	3.4758	7.5675	0.4929	0.5000	0.3447
34	C	3.4929	3.4759	6.3551	0.5025	0.5000	0.2895

/db/jmorales/CoNi-alloy/Slabs/CoNi(001)

a =			6.951669213				
b =			6.951669213				
c =			21.951669213				
alpha =			90.0				
beta =			90.0				
gamma =			90.0				
	Atom	X	Y	Z	X	Y	Z
1	Ni	0.0000	0.0000	0.0000	0.0000	0.0000	0.0000
2	Ni	0.0000	3.4758	0.0000	0.0000	0.5000	0.0000
3	Ni	3.4758	0.0000	0.0000	0.5000	0.0000	0.0000
4	Ni	3.4758	3.4758	0.0000	0.5000	0.5000	0.0000
5	Ni	0.0000	1.7379	1.7379	0.0000	0.2500	0.0792
6	Ni	0.0000	5.2138	1.7379	0.0000	0.7500	0.0792
7	Ni	3.4758	1.7379	1.7379	0.5000	0.2500	0.0792
8	Ni	3.4758	5.2138	1.7379	0.5000	0.7500	0.0792
9	Ni	0.0000	0.0000	3.4758	0.0000	0.0000	0.1583
10	Ni	0.0000	3.4758	3.4758	0.0000	0.5000	0.1583
11	Ni	3.4758	0.0000	3.4758	0.5000	0.0000	0.1583
12	Ni	3.4758	3.4758	3.4758	0.5000	0.5000	0.1583
13	Ni	0.0000	1.7379	5.2138	0.0000	0.2500	0.2375
14	Ni	0.0000	5.2138	5.2138	0.0000	0.7500	0.2375
15	Ni	3.4758	1.7379	5.2138	0.5000	0.2500	0.2375
16	Ni	3.4758	5.2138	5.2138	0.5000	0.7500	0.2375
17	Co	1.7379	1.7379	0.0000	0.2500	0.2500	0.0000
18	Co	1.7379	5.2138	0.0000	0.2500	0.7500	0.0000
19	Co	5.2138	1.7379	0.0000	0.7500	0.2500	0.0000
20	Co	5.2138	5.2138	0.0000	0.7500	0.7500	0.0000
21	Co	1.7379	0.0000	1.7379	0.2500	0.0000	0.0792
22	Co	1.7379	3.4758	1.7379	0.2500	0.5000	0.0792
23	Co	5.2138	0.0000	1.7379	0.7500	0.0000	0.0792
24	Co	5.2138	3.4758	1.7379	0.7500	0.5000	0.0792
25	Co	1.7379	1.7379	3.4758	0.2500	0.2500	0.1583
26	Co	1.7379	5.2138	3.4758	0.2500	0.7500	0.1583
27	Co	5.2138	1.7379	3.4758	0.7500	0.2500	0.1583
28	Co	5.2138	5.2138	3.4758	0.7500	0.7500	0.1583
29	Co	1.7379	0.0000	5.2138	0.2500	0.0000	0.2375
30	Co	1.7379	3.4758	5.2138	0.2500	0.5000	0.2375
31	Co	5.2138	0.0000	5.2138	0.7500	0.0000	0.2375
32	Co	5.2138	3.4758	5.2138	0.7500	0.5000	0.2375

/db/jmorales/CoNi-alloy/Slabs/Segregation/CoNi(001)/CoNi(001)-2-Ni

a =			6.9516692132			
b =			6.9516692132			
c =			21.9516692133			
alpha =			90.0			
beta =			90.0			
gamma =			90.0			
	Atom	X	Y	Z	X	Y	Z
1 Ni		0.0000	0.0000	0.0000	0.0000	0.0000	0.0000

#	Atom	X	Y	Z	X	Y	Z
1	Ni	0.0000	0.0000	0.0000	0.0000	0.0000	0.0000
2	Ni	0.0000	3.4758	0.0000	0.0000	0.5000	0.0000
3	Ni	3.4758	0.0000	0.0000	0.5000	0.0000	0.0000
4	Ni	3.4758	3.4758	0.0000	0.5000	0.5000	0.0000
5	Ni	0.0000	1.7379	1.7379	0.0000	0.2500	0.0792
6	Ni	0.0000	5.2138	1.7379	0.0000	0.7500	0.0792
7	Ni	3.4758	1.7379	1.7379	0.5000	0.2500	0.0792
8	Ni	3.4758	5.2138	1.7379	0.5000	0.7500	0.0792
9	Ni	0.0004	0.0001	3.4951	0.0001	0.0000	0.1592
10	Ni	3.4764	0.0000	3.4961	0.5001	0.0000	0.1593
11	Ni	1.7385	3.4759	5.2121	0.2501	0.5000	0.2374
12	Ni	0.0005	1.7065	5.2074	0.0001	0.2455	0.2372
13	Ni	0.0006	5.2452	5.2073	0.0001	0.7545	0.2372
14	Ni	3.4766	1.7069	5.2082	0.5001	0.2455	0.2373
15	Ni	3.4764	5.2448	5.2083	0.5001	0.7545	0.2373
16	Ni	5.2140	3.4760	5.2135	0.7500	0.5000	0.2375
17	Co	1.7379	1.7379	0.0000	0.2500	0.2500	0.0000
18	Co	1.7379	5.2138	0.0000	0.2500	0.7500	0.0000
19	Co	5.2138	1.7379	0.0000	0.7500	0.2500	0.0000
20	Co	5.2138	5.2138	0.0000	0.7500	0.7500	0.0000
21	Co	1.7379	0.0000	1.7379	0.2500	0.0000	0.0792
22	Co	1.7379	3.4758	1.7379	0.2500	0.5000	0.0792
23	Co	5.2138	0.0000	1.7379	0.7500	0.0000	0.0792
24	Co	5.2138	3.4758	1.7379	0.7500	0.5000	0.0792
25	Co	1.7386	1.7394	3.5332	0.2501	0.2502	0.1610
26	Co	1.7385	5.2124	3.5332	0.2501	0.7498	0.1610
27	Co	5.2140	1.7403	3.5326	0.7500	0.2503	0.1609
28	Co	5.2140	5.2116	3.5326	0.7500	0.7497	0.1609
29	Co	1.7384	0.0002	5.2016	0.2501	0.0000	0.2370
30	Co	3.4768	3.4760	3.5189	0.5001	0.5000	0.1603
31	Co	5.2152	0.0001	5.2001	0.7502	0.0000	0.2369
32	Co	-0.0005	3.4759	3.5185	-0.0001	0.5000	0.1603

/db/jmorales/CoNi-alloy/Slabs/Islanding/CoNi(111)/CoNi(111)-3-Ni-CO

a =				9.831199646				
b =				9.831199646075708				
c =				21.0202999115				
alpha =				90.0				
beta =				90.0				
gamma =				120.0				
		Atom	X	Y	Z	X	Y	Z
1	Ni		0.0000	0.0000	0.0000	0.0000	0.0000	0.0000
2	Ni		-1.2289	2.1285	0.0000	0.0000	0.2500	0.0000
3	Ni		-2.4578	4.2570	0.0000	0.0000	0.5000	0.0000
4	Ni		-3.6867	6.3856	0.0000	0.0000	0.7500	0.0000
5	Ni		4.9156	0.0000	0.0000	0.5000	0.0000	0.0000
6	Ni		3.6867	2.1285	0.0000	0.5000	0.2500	0.0000
7	Ni		2.4578	4.2570	0.0000	0.5000	0.5000	0.0000
8	Ni		1.2289	6.3856	0.0000	0.5000	0.7500	0.0000
9	Ni		2.4578	1.4190	2.0068	0.3333	0.1667	0.0955
10	Ni		1.2289	3.5475	2.0068	0.3333	0.4167	0.0955
11	Ni		0.0000	5.6760	2.0068	0.3333	0.6667	0.0955
12	Ni		-1.2289	7.8045	2.0068	0.3333	0.9167	0.0955
13	Ni		7.3734	1.4190	2.0068	0.8333	0.1667	0.0955
14	Ni		6.1445	3.5475	2.0068	0.8333	0.4167	0.0955
15	Ni		4.9156	5.6760	2.0068	0.8333	0.6667	0.0955
16	Ni		3.6867	7.8045	2.0068	0.8333	0.9167	0.0955
17	Ni		0.0371	2.8581	4.0037	0.1716	0.3357	0.1905
18	Ni		1.2613	0.7296	4.0097	0.1711	0.0857	0.1908
19	Ni		-2.4306	7.1137	4.0142	0.1705	0.8355	0.1910
20	Ni		-1.1955	4.9806	4.0069	0.1709	0.5850	0.1906
21	Ni		4.9430	2.8513	4.0139	0.6702	0.3349	0.1910
22	Ni		6.1687	0.7290	4.0112	0.6703	0.0856	0.1908
23	Ni		2.4948	7.1138	4.0141	0.6715	0.8355	0.1910
24	Ni		3.7190	4.9833	4.0157	0.6709	0.5853	0.1910
25	Ni		-1.1811	2.1507	6.0341	0.0062	0.2526	0.2871
26	Ni		-2.4116	4.2958	6.0338	0.0070	0.5046	0.2870
27	Ni		4.9602	0.0327	6.0258	0.5065	0.0038	0.2867
28	Ni		3.7456	2.1572	6.0863	0.5077	0.2534	0.2895
29	Ni		2.5154	4.2941	6.0857	0.5080	0.5044	0.2895
30	Ni		1.2742	2.1496	6.0945	0.2558	0.2525	0.2899
31	Ni		0.0271	4.3165	6.0362	0.2563	0.5070	0.2872
32	Ni		4.9901	4.3092	6.0432	0.7606	0.5061	0.2875
33	Co		1.2289	2.1285	0.0000	0.2500	0.2500	0.0000
34	Co		2.4578	0.0000	0.0000	0.2500	0.0000	0.0000
35	Co		-1.2289	6.3856	0.0000	0.2500	0.7500	0.0000
36	Co		0.0000	4.2570	0.0000	0.2500	0.5000	0.0000
37	Co		6.1445	2.1285	0.0000	0.7500	0.2500	0.0000
38	Co		7.3734	0.0000	0.0000	0.7500	0.0000	0.0000
39	Co		3.6867	6.3856	0.0000	0.7500	0.7500	0.0000
40	Co		4.9156	4.2570	0.0000	0.7500	0.5000	0.0000
41	Co		-1.2289	3.5475	2.0068	0.0833	0.4167	0.0955

42	Co	0.0000	1.4190	2.0068	0.0833	0.1667	0.0955
43	Co	-3.6867	7.8045	2.0068	0.0833	0.9167	0.0955
44	Co	-2.4578	5.6760	2.0068	0.0833	0.6667	0.0955
45	Co	3.6867	3.5475	2.0068	0.5833	0.4167	0.0955
46	Co	4.9156	1.4190	2.0068	0.5833	0.1667	0.0955
47	Co	1.2289	7.8045	2.0068	0.5833	0.9167	0.0955
48	Co	2.4578	5.6760	2.0068	0.5833	0.6667	0.0955
49	Co	3.7036	0.7297	4.0559	0.4196	0.0857	0.1930
50	Co	2.4999	2.8635	4.0040	0.4224	0.3363	0.1905
51	Co	1.2654	4.9876	4.0605	0.4216	0.5858	0.1932
52	Co	0.0329	7.1109	4.0524	0.4209	0.8352	0.1928
53	Co	8.6360	0.7277	4.0515	0.9212	0.0855	0.1927
54	Co	7.4001	2.8544	4.0625	0.9203	0.3353	0.1933
55	Co	6.1754	4.9874	4.0486	0.9210	0.5858	0.1926
56	Co	4.9450	7.1166	4.0595	0.9209	0.8359	0.1931
57	Co	2.5077	-0.0080	6.0034	0.2546	-0.0009	0.2856
58	Co	-1.1755	6.4320	6.0103	0.2582	0.7555	0.2859
59	Co	6.2186	2.1478	6.0162	0.7587	0.2523	0.2862
60	Co	7.4368	0.0306	6.0115	0.7582	0.0036	0.2860
61	Co	3.7469	6.4316	6.0278	0.7588	0.7554	0.2868
62	Co	0.0277	0.0280	5.9928	0.0045	0.0033	0.2851
63	Co	1.2750	6.4133	5.9939	0.5063	0.7533	0.2851
64	Co	-3.6464	6.3984	5.9926	0.0049	0.7515	0.2851
65	O	2.5012	2.8587	8.6170	0.4223	0.3358	0.4099
66	C	2.4848	2.8415	7.4240	0.4196	0.3337	0.3532

/db/jmorales/CoNi-alloy/Slabs/Segregation/CoNi(111)/CoNi(111)-2-Co-CO

a = 9.831199646
b = 9.831199646075708
c = 21.0202999115
alpha = 90.0
beta = 90.0
gamma = 120.0

	Atom	X	Y	Z	X	Y	Z
1	Ni	0.0000	0.0000	0.0000	0.0000	0.0000	0.0000
2	Ni	-1.2289	2.1285	0.0000	0.0000	0.2500	0.0000
3	Ni	-2.4578	4.2570	0.0000	0.0000	0.5000	0.0000
4	Ni	-3.6867	6.3856	0.0000	0.0000	0.7500	0.0000
5	Ni	4.9156	0.0000	0.0000	0.5000	0.0000	0.0000
6	Ni	3.6867	2.1285	0.0000	0.5000	0.2500	0.0000
7	Ni	2.4578	4.2570	0.0000	0.5000	0.5000	0.0000
8	Ni	1.2289	6.3856	0.0000	0.5000	0.7500	0.0000
9	Ni	2.4578	1.4190	2.0068	0.3333	0.1667	0.0955
10	Ni	1.2289	3.5475	2.0068	0.3333	0.4167	0.0955
11	Ni	0.0000	5.6760	2.0068	0.3333	0.6667	0.0955
12	Ni	-1.2289	7.8045	2.0068	0.3333	0.9167	0.0955
13	Ni	7.3734	1.4190	2.0068	0.8333	0.1667	0.0955
14	Ni	6.1445	3.5475	2.0068	0.8333	0.4167	0.0955
15	Ni	4.9156	5.6760	2.0068	0.8333	0.6667	0.0955
16	Ni	3.6867	7.8045	2.0068	0.8333	0.9167	0.0955
17	Ni	0.0287	2.8498	4.0140	0.1703	0.3347	0.1910
18	Ni	1.2535	0.7296	4.0138	0.1703	0.0857	0.1909
19	Ni	-2.4340	7.1052	4.0183	0.1697	0.8345	0.1912
20	Ni	-1.2083	4.9824	4.0184	0.1697	0.5852	0.1912
21	Ni	4.9378	2.8603	4.0197	0.6702	0.3360	0.1912
22	Ni	6.1766	0.7230	4.0166	0.6707	0.0849	0.1911
23	Ni	2.4849	7.1161	4.0174	0.6707	0.8358	0.1911
24	Ni	3.7158	4.9740	4.0206	0.6701	0.5842	0.1913
25	Ni	0.0434	0.0233	6.0325	0.0058	0.0027	0.2870
26	Ni	-1.1872	2.1538	6.0327	0.0057	0.2530	0.2870
27	Ni	-2.4168	4.2812	6.0335	0.0056	0.5028	0.2870
28	Ni	-3.6465	6.4077	6.0336	0.0054	0.7526	0.2870
29	Ni	4.9650	0.0238	6.0193	0.5064	0.0028	0.2864
30	Ni	1.2753	6.4147	6.0208	0.5064	0.7534	0.2864
31	Ni	3.7066	0.7295	4.0375	0.4199	0.0857	0.1921
32	Ni	1.2562	4.9732	4.0390	0.4198	0.5841	0.1921
33	Co	1.2289	2.1285	0.0000	0.2500	0.2500	0.0000
34	Co	2.4578	0.0000	0.0000	0.2500	0.0000	0.0000
35	Co	-1.2289	6.3856	0.0000	0.2500	0.7500	0.0000
36	Co	0.0000	4.2570	0.0000	0.2500	0.5000	0.0000
37	Co	6.1445	2.1285	0.0000	0.7500	0.2500	0.0000
38	Co	7.3734	0.0000	0.0000	0.7500	0.0000	0.0000
39	Co	3.6867	6.3856	0.0000	0.7500	0.7500	0.0000
40	Co	4.9156	4.2570	0.0000	0.7500	0.5000	0.0000
41	Co	-1.2289	3.5475	2.0068	0.0833	0.4167	0.0955

42	Co	0.0000	1.4190	2.0068	0.0833	0.1667	0.0955
43	Co	-3.6867	7.8045	2.0068	0.0833	0.9167	0.0955
44	Co	-2.4578	5.6760	2.0068	0.0833	0.6667	0.0955
45	Co	3.6867	3.5475	2.0068	0.5833	0.4167	0.0955
46	Co	4.9156	1.4190	2.0068	0.5833	0.1667	0.0955
47	Co	1.2289	7.8045	2.0068	0.5833	0.9167	0.0955
48	Co	2.4578	5.6760	2.0068	0.5833	0.6667	0.0955
49	Co	2.4841	2.8525	4.0760	0.4202	0.3350	0.1939
50	Co	0.0237	7.1085	4.0539	0.4199	0.8349	0.1929
51	Co	8.6282	0.7255	4.0623	0.9202	0.0852	0.1933
52	Co	7.3969	2.8523	4.0578	0.9199	0.3350	0.1930
53	Co	6.1677	4.9799	4.0560	0.9198	0.5849	0.1930
54	Co	4.9395	7.1085	4.0573	0.9199	0.8349	0.1930
55	Co	1.2767	2.1560	6.0711	0.2565	0.2532	0.2888
56	Co	2.5142	0.0138	6.0231	0.2565	0.0016	0.2865
57	Co	-1.1637	6.4227	6.0136	0.2588	0.7544	0.2861
58	Co	0.0411	4.2986	6.0239	0.2566	0.5049	0.2866
59	Co	6.1954	2.1639	6.0101	0.7572	0.2542	0.2859
60	Co	7.4281	0.0327	6.0323	0.7575	0.0038	0.2870
61	Co	3.7415	6.4129	6.0106	0.7572	0.7532	0.2859
62	Co	4.9814	4.2943	6.0187	0.7589	0.5044	0.2863
63	Co	3.7343	2.1481	6.0415	0.5060	0.2523	0.2874
64	Co	2.4993	4.2845	6.0470	0.5058	0.5032	0.2877
65	O	2.5330	2.8607	8.6068	0.4256	0.3360	0.4095
66	C	2.5020	2.8575	7.4098	0.4223	0.3356	0.3525

/db/jmorales/CoNi-alloy/Slabs/Islanding/CoNi(001)/CoNi(001)-1-Co-CO

a =			6.9516692132				
b =			6.9516692132				
c =			21.9516692133				
alpha =			90.0				
beta =			90.0				
gamma =			90.0				
	Atom	X	Y	Z	X	Y	Z
1	Ni	0.0000	0.0000	0.0000	0.0000	0.0000	0.0000
2	Ni	0.0000	3.4758	0.0000	0.0000	0.5000	0.0000
3	Ni	3.4758	0.0000	0.0000	0.5000	0.0000	0.0000
4	Ni	3.4758	3.4758	0.0000	0.5000	0.5000	0.0000
5	Ni	0.0000	1.7379	1.7379	0.0000	0.2500	0.0792
6	Ni	0.0000	5.2138	1.7379	0.0000	0.7500	0.0792
7	Ni	3.4758	1.7379	1.7379	0.5000	0.2500	0.0792
8	Ni	3.4758	5.2138	1.7379	0.5000	0.7500	0.0792
9	Ni	6.9506	0.0009	3.5017	0.9998	0.0001	0.1595
10	Ni	6.9483	3.4745	3.4965	0.9995	0.4998	0.1593
11	Ni	3.4758	-0.0070	3.5081	0.5000	-0.0010	0.1598
12	Ni	3.4763	3.4793	3.4527	0.5001	0.5005	0.1573
13	Ni	6.9477	1.7524	5.2047	0.9994	0.2521	0.2371
14	Ni	6.9490	5.2035	5.2120	0.9996	0.7485	0.2374
15	Ni	3.4828	5.1886	5.2518	0.5010	0.7464	0.2392
16	Ni	1.7432	0.0008	5.2214	0.2508	0.0001	0.2379
17	Co	1.7379	1.7379	0.0000	0.2500	0.2500	0.0000
18	Co	1.7379	5.2138	0.0000	0.2500	0.7500	0.0000
19	Co	5.2138	1.7379	0.0000	0.7500	0.2500	0.0000
20	Co	5.2138	5.2138	0.0000	0.7500	0.7500	0.0000
21	Co	1.7379	0.0000	1.7379	0.2500	0.0000	0.0792
22	Co	1.7379	3.4758	1.7379	0.2500	0.5000	0.0792
23	Co	5.2138	0.0000	1.7379	0.7500	0.0000	0.0792
24	Co	5.2138	3.4758	1.7379	0.7500	0.5000	0.0792
25	Co	1.7425	1.7456	3.5487	0.2507	0.2511	0.1617
26	Co	1.7342	5.2030	3.5392	0.2495	0.7484	0.1612
27	Co	5.2088	1.7360	3.5598	0.7493	0.2497	0.1622
28	Co	5.2148	5.2150	3.5547	0.7502	0.7502	0.1619
29	Co	1.7566	3.4675	5.2375	0.2527	0.4988	0.2386
30	Co	5.2105	0.0036	5.2049	0.7495	0.0005	0.2371
31	Co	5.1899	3.4647	5.2400	0.7466	0.4984	0.2387
32	Co	3.4763	1.7527	5.2055	0.5001	0.2521	0.2371
33	O	3.4647	3.4632	7.5767	0.4984	0.4982	0.3452
34	C	3.4699	3.5043	6.3618	0.4991	0.5041	0.2898

/db/jmorales/CoNi-alloy/Slabs/Islanding/CoNi(111)/CoNi(111)-2-Co

a = 9.831199646
b = 9.831199646075708
c = 21.0202999115
alpha = 90.0
beta = 90.0
gamma = 120.0

	Atom	X	Y	Z	X	Y	Z
1	Ni	0.0000	0.0000	0.0000	0.0000	0.0000	0.0000
2	Ni	-1.2289	2.1285	0.0000	0.0000	0.2500	0.0000
3	Ni	-2.4578	4.2570	0.0000	0.0000	0.5000	0.0000
4	Ni	-3.6867	6.3856	0.0000	0.0000	0.7500	0.0000
5	Ni	4.9156	0.0000	0.0000	0.5000	0.0000	0.0000
6	Ni	3.6867	2.1285	0.0000	0.5000	0.2500	0.0000
7	Ni	2.4578	4.2570	0.0000	0.5000	0.5000	0.0000
8	Ni	1.2289	6.3856	0.0000	0.5000	0.7500	0.0000
9	Ni	2.4578	1.4190	2.0068	0.3333	0.1667	0.0955
10	Ni	1.2289	3.5475	2.0068	0.3333	0.4167	0.0955
11	Ni	0.0000	5.6760	2.0068	0.3333	0.6667	0.0955
12	Ni	-1.2289	7.8045	2.0068	0.3333	0.9167	0.0955
13	Ni	7.3734	1.4190	2.0068	0.8333	0.1667	0.0955
14	Ni	6.1445	3.5475	2.0068	0.8333	0.4167	0.0955
15	Ni	4.9156	5.6760	2.0068	0.8333	0.6667	0.0955
16	Ni	3.6867	7.8045	2.0068	0.8333	0.9167	0.0955
17	Ni	0.0378	2.8574	4.0180	0.1717	0.3356	0.1912
18	Ni	1.2656	0.7295	4.0081	0.1716	0.0857	0.1907
19	Ni	-2.4227	7.1123	4.0077	0.1713	0.8354	0.1907
20	Ni	-1.1989	4.9856	4.0134	0.1708	0.5856	0.1909
21	Ni	4.9484	2.8577	4.0125	0.6712	0.3356	0.1909
22	Ni	6.1777	0.7266	4.0161	0.6710	0.0853	0.1911
23	Ni	2.4900	7.1148	4.0031	0.6711	0.8357	0.1904
24	Ni	3.7159	4.9846	4.0029	0.6707	0.5855	0.1904
25	Ni	0.0559	0.0335	6.0286	0.0077	0.0039	0.2868
26	Ni	-1.1796	2.1653	6.0290	0.0072	0.2543	0.2868
27	Ni	-2.4088	4.2815	6.0273	0.0064	0.5029	0.2867
28	Ni	-3.6348	6.4089	6.0243	0.0066	0.7527	0.2866
29	Ni	4.9685	0.0337	6.0284	0.5074	0.0040	0.2868
30	Ni	1.2872	6.4188	6.0311	0.5079	0.7539	0.2869
31	Ni	2.5245	0.0416	6.0556	0.2592	0.0049	0.2881
32	Ni	3.7467	6.4164	6.0526	0.7579	0.7536	0.2879
33	Co	1.2289	2.1285	0.0000	0.2500	0.2500	0.0000
34	Co	2.4578	0.0000	0.0000	0.2500	0.0000	0.0000
35	Co	-1.2289	6.3856	0.0000	0.2500	0.7500	0.0000
36	Co	0.0000	4.2570	0.0000	0.2500	0.5000	0.0000
37	Co	6.1445	2.1285	0.0000	0.7500	0.2500	0.0000
38	Co	7.3734	0.0000	0.0000	0.7500	0.0000	0.0000
39	Co	3.6867	6.3856	0.0000	0.7500	0.7500	0.0000
40	Co	4.9156	4.2570	0.0000	0.7500	0.5000	0.0000
41	Co	-1.2289	3.5475	2.0068	0.0833	0.4167	0.0955

42	Co	0.0000	1.4190	2.0068	0.0833	0.1667	0.0955
43	Co	-3.6867	7.8045	2.0068	0.0833	0.9167	0.0955
44	Co	-2.4578	5.6760	2.0068	0.0833	0.6667	0.0955
45	Co	3.6867	3.5475	2.0068	0.5833	0.4167	0.0955
46	Co	4.9156	1.4190	2.0068	0.5833	0.1667	0.0955
47	Co	1.2289	7.8045	2.0068	0.5833	0.9167	0.0955
48	Co	2.4578	5.6760	2.0068	0.5833	0.6667	0.0955
49	Co	3.7213	0.7362	4.0460	0.4218	0.0865	0.1925
50	Co	2.4908	2.8552	4.0638	0.4210	0.3354	0.1933
51	Co	1.2605	4.9828	4.0558	0.4208	0.5852	0.1929
52	Co	0.0283	7.1099	4.0581	0.4204	0.8351	0.1931
53	Co	8.6338	0.7241	4.0610	0.9207	0.0850	0.1932
54	Co	7.4057	2.8566	4.0469	0.9210	0.3355	0.1925
55	Co	6.1688	4.9822	4.0536	0.9201	0.5852	0.1928
56	Co	4.9568	7.1186	4.0421	0.9222	0.8361	0.1923
57	Co	1.3041	2.1847	6.0280	0.2610	0.2566	0.2868
58	Co	-1.1474	6.4164	6.0159	0.2601	0.7536	0.2862
59	Co	0.0769	4.2910	6.0208	0.2598	0.5040	0.2864
60	Co	6.2115	2.1723	6.0150	0.7594	0.2551	0.2861
61	Co	7.4326	0.0485	6.0165	0.7589	0.0057	0.2862
62	Co	4.9679	4.2789	6.0111	0.7566	0.5026	0.2860
63	Co	3.7440	2.1332	5.9943	0.5061	0.2506	0.2852
64	Co	2.4978	4.2949	5.9923	0.5063	0.5044	0.2851

/db/jmorales/CoNi-alloy/Slabs/Segregation/CoNi(111)/CoNi(111)-1-Co

a =	9.831199646
b =	9.831199646075708
c =	21.0202999115
alpha =	90.0
beta =	90.0
gamma =	120.0

	Atom	X	Y	Z	X	Y	Z
1	Ni	0.0000	0.0000	0.0000	0.0000	0.0000	0.0000
2	Ni	-1.2289	2.1285	0.0000	0.0000	0.2500	0.0000
3	Ni	-2.4578	4.2570	0.0000	0.0000	0.5000	0.0000
4	Ni	-3.6867	6.3856	0.0000	0.0000	0.7500	0.0000
5	Ni	4.9156	0.0000	0.0000	0.5000	0.0000	0.0000
6	Ni	3.6867	2.1285	0.0000	0.5000	0.2500	0.0000
7	Ni	2.4578	4.2570	0.0000	0.5000	0.5000	0.0000
8	Ni	1.2289	6.3856	0.0000	0.5000	0.7500	0.0000
9	Ni	2.4578	1.4190	2.0068	0.3333	0.1667	0.0955
10	Ni	1.2289	3.5475	2.0068	0.3333	0.4167	0.0955
11	Ni	0.0000	5.6760	2.0068	0.3333	0.6667	0.0955
12	Ni	-1.2289	7.8045	2.0068	0.3333	0.9167	0.0955
13	Ni	7.3734	1.4190	2.0068	0.8333	0.1667	0.0955
14	Ni	6.1445	3.5475	2.0068	0.8333	0.4167	0.0955
15	Ni	4.9156	5.6760	2.0068	0.8333	0.6667	0.0955
16	Ni	3.6867	7.8045	2.0068	0.8333	0.9167	0.0955
17	Ni	0.0311	2.8567	4.0129	0.1709	0.3355	0.1909
18	Ni	1.2547	0.7314	4.0195	0.1706	0.0859	0.1912
19	Ni	-2.4285	7.1080	4.0156	0.1704	0.8349	0.1910
20	Ni	-1.1973	4.9859	4.0143	0.1710	0.5856	0.1910
21	Ni	4.9443	2.8604	4.0115	0.6709	0.3360	0.1908
22	Ni	6.1842	0.7238	4.0155	0.6716	0.0850	0.1910
23	Ni	2.4914	7.1121	4.0160	0.6711	0.8353	0.1911
24	Ni	3.7173	4.9868	4.0145	0.6710	0.5857	0.1910
25	Ni	0.0500	0.0271	6.0351	0.0067	0.0032	0.2871
26	Ni	-1.1797	2.1599	6.0304	0.0068	0.2537	0.2869
27	Ni	-2.4100	4.2855	6.0276	0.0065	0.5033	0.2868
28	Ni	-3.6370	6.4138	6.0349	0.0067	0.7533	0.2871
29	Ni	4.9705	0.0267	6.0225	0.5071	0.0031	0.2865
30	Ni	2.5074	4.2918	6.0346	0.5071	0.5041	0.2871
31	Ni	1.2789	6.4167	6.0319	0.5069	0.7537	0.2870
32	Ni	3.7157	0.7299	4.0378	0.4208	0.0857	0.1921
33	Co	1.2289	2.1285	0.0000	0.2500	0.2500	0.0000
34	Co	2.4578	0.0000	0.0000	0.2500	0.0000	0.0000
35	Co	-1.2289	6.3856	0.0000	0.2500	0.7500	0.0000
36	Co	0.0000	4.2570	0.0000	0.2500	0.5000	0.0000
37	Co	6.1445	2.1285	0.0000	0.7500	0.2500	0.0000
38	Co	7.3734	0.0000	0.0000	0.7500	0.0000	0.0000
39	Co	3.6867	6.3856	0.0000	0.7500	0.7500	0.0000
40	Co	4.9156	4.2570	0.0000	0.7500	0.5000	0.0000
41	Co	-1.2289	3.5475	2.0068	0.0833	0.4167	0.0955

42	Co	0.0000	1.4190	2.0068	0.0833	0.1667	0.0955
43	Co	-3.6867	7.8045	2.0068	0.0833	0.9167	0.0955
44	Co	-2.4578	5.6760	2.0068	0.0833	0.6667	0.0955
45	Co	3.6867	3.5475	2.0068	0.5833	0.4167	0.0955
46	Co	4.9156	1.4190	2.0068	0.5833	0.1667	0.0955
47	Co	1.2289	7.8045	2.0068	0.5833	0.9167	0.0955
48	Co	2.4578	5.6760	2.0068	0.5833	0.6667	0.0955
49	Co	2.4841	2.8616	4.0639	0.4207	0.3361	0.1933
50	Co	1.2530	4.9818	4.0615	0.4200	0.5851	0.1932
51	Co	0.0357	7.0994	4.0557	0.4206	0.8338	0.1929
52	Co	8.6292	0.7260	4.0614	0.9204	0.0853	0.1932
53	Co	7.3986	2.8529	4.0557	0.9201	0.3351	0.1929
54	Co	6.1700	4.9823	4.0576	0.9202	0.5852	0.1930
55	Co	4.9476	7.1124	4.0596	0.9209	0.8354	0.1931
56	Co	1.3060	2.1615	6.0242	0.2598	0.2539	0.2866
57	Co	2.5211	0.0495	6.0293	0.2593	0.0058	0.2868
58	Co	-1.1582	6.4291	6.0168	0.2597	0.7551	0.2862
59	Co	0.0710	4.2972	6.0198	0.2596	0.5047	0.2864
60	Co	6.2056	2.1684	6.0121	0.7586	0.2547	0.2860
61	Co	7.4381	0.0410	6.0260	0.7590	0.0048	0.2867
62	Co	3.7560	6.4225	6.0208	0.7592	0.7543	0.2864
63	Co	4.9790	4.2907	6.0238	0.7584	0.5040	0.2866
64	Co	3.7245	2.1536	5.9960	0.5053	0.2529	0.2852

/db/jmorales/CoNi-alloy/Slabs/Islanding/CoNi(111)/CoNi(111)-2-Ni

a =		9.831199646					
b =		9.831199646075708					
c =		21.0202999115					
alpha =		90.0					
beta =		90.0					
gamma =		120.0					
	Atom	X	Y	Z	X	Y	Z
1	Ni	0.0000	0.0000	0.0000	0.0000	0.0000	0.0000
2	Ni	-1.2289	2.1285	0.0000	0.0000	0.2500	0.0000
3	Ni	-2.4578	4.2570	0.0000	0.0000	0.5000	0.0000
4	Ni	-3.6867	6.3856	0.0000	0.0000	0.7500	0.0000
5	Ni	4.9156	0.0000	0.0000	0.5000	0.0000	0.0000
6	Ni	3.6867	2.1285	0.0000	0.5000	0.2500	0.0000
7	Ni	2.4578	4.2570	0.0000	0.5000	0.5000	0.0000
8	Ni	1.2289	6.3856	0.0000	0.5000	0.7500	0.0000
9	Ni	2.4578	1.4190	2.0068	0.3333	0.1667	0.0955
10	Ni	1.2289	3.5475	2.0068	0.3333	0.4167	0.0955
11	Ni	0.0000	5.6760	2.0068	0.3333	0.6667	0.0955
12	Ni	-1.2289	7.8045	2.0068	0.3333	0.9167	0.0955
13	Ni	7.3734	1.4190	2.0068	0.8333	0.1667	0.0955
14	Ni	6.1445	3.5475	2.0068	0.8333	0.4167	0.0955
15	Ni	4.9156	5.6760	2.0068	0.8333	0.6667	0.0955
16	Ni	3.6867	7.8045	2.0068	0.8333	0.9167	0.0955
17	Ni	0.0312	2.8576	3.9973	0.1710	0.3356	0.1902
18	Ni	1.2607	0.7297	4.0063	0.1711	0.0857	0.1906
19	Ni	-2.4245	7.1132	4.0176	0.1711	0.8355	0.1911
20	Ni	-1.1938	4.9849	4.0103	0.1713	0.5855	0.1908
21	Ni	4.9480	2.8575	4.0149	0.6711	0.3356	0.1910
22	Ni	6.1733	0.7298	4.0084	0.6708	0.0857	0.1907
23	Ni	2.4938	7.1174	4.0110	0.6716	0.8360	0.1908
24	Ni	3.7200	4.9825	4.0151	0.6710	0.5852	0.1910
25	Ni	-1.1815	2.1536	6.0350	0.0063	0.2529	0.2871
26	Ni	-2.4116	4.2917	6.0336	0.0067	0.5041	0.2870
27	Ni	-3.6396	6.4105	6.0320	0.0063	0.7529	0.2870
28	Ni	4.9682	0.0395	6.0282	0.5077	0.0046	0.2868
29	Ni	3.7402	2.1580	6.0250	0.5072	0.2535	0.2866
30	Ni	2.5224	4.2874	6.0267	0.5084	0.5036	0.2867
31	Ni	1.2875	2.1526	6.0463	0.2574	0.2528	0.2876
32	Ni	0.0584	4.2990	6.0512	0.2584	0.5049	0.2879
33	Co	1.2289	2.1285	0.0000	0.2500	0.2500	0.0000
34	Co	2.4578	0.0000	0.0000	0.2500	0.0000	0.0000
35	Co	-1.2289	6.3856	0.0000	0.2500	0.7500	0.0000
36	Co	0.0000	4.2570	0.0000	0.2500	0.5000	0.0000
37	Co	6.1445	2.1285	0.0000	0.7500	0.2500	0.0000
38	Co	7.3734	0.0000	0.0000	0.7500	0.0000	0.0000
39	Co	3.6867	6.3856	0.0000	0.7500	0.7500	0.0000
40	Co	4.9156	4.2570	0.0000	0.7500	0.5000	0.0000
41	Co	-1.2289	3.5475	2.0068	0.0833	0.4167	0.0955

42	Co	0.0000	1.4190	2.0068	0.0833	0.1667	0.0955
43	Co	-3.6867	7.8045	2.0068	0.0833	0.9167	0.0955
44	Co	-2.4578	5.6760	2.0068	0.0833	0.6667	0.0955
45	Co	3.6867	3.5475	2.0068	0.5833	0.4167	0.0955
46	Co	4.9156	1.4190	2.0068	0.5833	0.1667	0.0955
47	Co	1.2289	7.8045	2.0068	0.5833	0.9167	0.0955
48	Co	2.4578	5.6760	2.0068	0.5833	0.6667	0.0955
49	Co	3.7116	0.7264	4.0517	0.4202	0.0853	0.1928
50	Co	2.4903	2.8599	4.0380	0.4213	0.3359	0.1921
51	Co	1.2658	4.9913	4.0536	0.4219	0.5862	0.1928
52	Co	0.0327	7.1097	4.0605	0.4209	0.8351	0.1932
53	Co	8.6341	0.7260	4.0581	0.9209	0.0853	0.1931
54	Co	7.4048	2.8550	4.0634	0.9209	0.3353	0.1933
55	Co	6.1762	4.9835	4.0555	0.9209	0.5853	0.1929
56	Co	4.9451	7.1193	4.0545	0.9211	0.8362	0.1929
57	Co	2.5279	0.0263	6.0123	0.2587	0.0031	0.2860
58	Co	-1.1649	6.4357	6.0194	0.2595	0.7559	0.2864
59	Co	6.2146	2.1646	6.0185	0.7593	0.2542	0.2863
60	Co	7.4405	0.0264	6.0153	0.7584	0.0031	0.2862
61	Co	3.7517	6.4301	6.0160	0.7592	0.7552	0.2862
62	Co	4.9893	4.3043	6.0199	0.7603	0.5055	0.2864
63	Co	0.0333	0.0130	5.9941	0.0041	0.0015	0.2852
64	Co	1.2772	6.4076	5.9938	0.5062	0.7526	0.2851

/db/jmorales/CoNi-alloy/Slabs/Islanding/CoNi(111)/CoNi(111)-4-Co-CO

a =		9.831199646
b =		9.831199646075708
c =		21.0202999115
alpha =		90.0
beta =		90.0
gamma =		120.0

	Atom	X	Y	Z	X	Y	Z
1	Ni	0.0000	0.0000	0.0000	0.0000	0.0000	0.0000
2	Ni	-1.2289	2.1285	0.0000	0.0000	0.2500	0.0000
3	Ni	-2.4578	4.2570	0.0000	0.0000	0.5000	0.0000
4	Ni	-3.6867	6.3856	0.0000	0.0000	0.7500	0.0000
5	Ni	4.9156	0.0000	0.0000	0.5000	0.0000	0.0000
6	Ni	3.6867	2.1285	0.0000	0.5000	0.2500	0.0000
7	Ni	2.4578	4.2570	0.0000	0.5000	0.5000	0.0000
8	Ni	1.2289	6.3856	0.0000	0.5000	0.7500	0.0000
9	Ni	2.4578	1.4190	2.0068	0.3333	0.1667	0.0955
10	Ni	1.2289	3.5475	2.0068	0.3333	0.4167	0.0955
11	Ni	0.0000	5.6760	2.0068	0.3333	0.6667	0.0955
12	Ni	-1.2289	7.8045	2.0068	0.3333	0.9167	0.0955
13	Ni	7.3734	1.4190	2.0068	0.8333	0.1667	0.0955
14	Ni	6.1445	3.5475	2.0068	0.8333	0.4167	0.0955
15	Ni	4.9156	5.6760	2.0068	0.8333	0.6667	0.0955
16	Ni	3.6867	7.8045	2.0068	0.8333	0.9167	0.0955
17	Ni	0.0361	2.8596	4.0127	0.1716	0.3359	0.1909
18	Ni	1.2648	0.7278	4.0061	0.1714	0.0855	0.1906
19	Ni	-2.4271	7.1141	3.9962	0.1709	0.8356	0.1901
20	Ni	-1.2019	4.9825	4.0029	0.1703	0.5852	0.1904
21	Ni	4.9454	2.8593	4.0252	0.6710	0.3358	0.1915
22	Ni	6.1792	0.7273	4.0085	0.6712	0.0854	0.1907
23	Ni	2.4906	7.1120	3.9966	0.6710	0.8353	0.1901
24	Ni	3.7112	4.9807	4.0071	0.6700	0.5850	0.1906
25	Ni	0.0525	0.0377	6.0260	0.0076	0.0044	0.2867
26	Ni	-3.6356	6.4139	6.0262	0.0069	0.7533	0.2867
27	Ni	4.9716	0.0414	6.0203	0.5081	0.0049	0.2864
28	Ni	1.2830	6.4078	6.0195	0.5068	0.7526	0.2864
29	Ni	2.5127	0.0211	6.0316	0.2568	0.0025	0.2869
30	Ni	3.7468	6.4082	6.0403	0.7574	0.7527	0.2874
31	Ni	7.4293	0.0450	6.0478	0.7583	0.0053	0.2877
32	Ni	-1.1670	6.4089	6.0402	0.2577	0.7527	0.2873
33	Co	1.2289	2.1285	0.0000	0.2500	0.2500	0.0000
34	Co	2.4578	0.0000	0.0000	0.2500	0.0000	0.0000
35	Co	-1.2289	6.3856	0.0000	0.2500	0.7500	0.0000
36	Co	0.0000	4.2570	0.0000	0.2500	0.5000	0.0000
37	Co	6.1445	2.1285	0.0000	0.7500	0.2500	0.0000
38	Co	7.3734	0.0000	0.0000	0.7500	0.0000	0.0000
39	Co	3.6867	6.3856	0.0000	0.7500	0.7500	0.0000
40	Co	4.9156	4.2570	0.0000	0.7500	0.5000	0.0000
41	Co	-1.2289	3.5475	2.0068	0.0833	0.4167	0.0955

42	Co	0.0000	1.4190	2.0068	0.0833	0.1667	0.0955
43	Co	-3.6867	7.8045	2.0068	0.0833	0.9167	0.0955
44	Co	-2.4578	5.6760	2.0068	0.0833	0.6667	0.0955
45	Co	3.6867	3.5475	2.0068	0.5833	0.4167	0.0955
46	Co	4.9156	1.4190	2.0068	0.5833	0.1667	0.0955
47	Co	1.2289	7.8045	2.0068	0.5833	0.9167	0.0955
48	Co	2.4578	5.6760	2.0068	0.5833	0.6667	0.0955
49	Co	3.7230	0.7328	4.0424	0.4217	0.0861	0.1923
50	Co	2.4906	2.8578	4.0635	0.4212	0.3357	0.1933
51	Co	1.2564	4.9809	4.0456	0.4203	0.5850	0.1925
52	Co	0.0368	7.1147	4.0347	0.4216	0.8356	0.1919
53	Co	8.6423	0.7351	4.0495	0.9222	0.0863	0.1926
54	Co	7.4055	2.8585	4.0565	0.9211	0.3357	0.1930
55	Co	6.1702	4.9815	4.0552	0.9202	0.5851	0.1929
56	Co	4.9515	7.1145	4.0377	0.9215	0.8356	0.1921
57	Co	1.2768	2.1571	6.0668	0.2565	0.2534	0.2886
58	Co	0.0402	4.2873	5.9995	0.2559	0.5036	0.2854
59	Co	6.2093	2.1719	6.0195	0.7591	0.2551	0.2864
60	Co	5.0002	4.2830	6.0090	0.7601	0.5030	0.2859
61	Co	3.7533	2.1486	6.0415	0.5080	0.2524	0.2874
62	Co	2.5061	4.2943	6.0429	0.5071	0.5044	0.2875
63	Co	-1.1745	2.1433	5.9916	0.0064	0.2517	0.2850
64	Co	-2.4161	4.2828	5.9897	0.0058	0.5030	0.2849
65	O	2.5447	2.8857	8.5910	0.4283	0.3389	0.4087
66	C	2.4872	2.8666	7.3946	0.4213	0.3367	0.3518

/db/jmorales/CoNi-alloy/Slabs/Segregation/Co-CoNi(001)/Co-CoNi(001)-2-Ni-CO

a = 6.9516692132
b = 6.9516692132
c = 21.9516692133
alpha = 90.0
beta = 90.0
gamma = 90.0

	Atom	X	Y	Z	X	Y	Z
1	Ni	0.0000	0.0000	0.0000	0.0000	0.0000	0.0000
2	Ni	1.7379	1.7379	0.0000	0.2500	0.2500	0.0000
3	Ni	0.0000	3.4758	0.0000	0.0000	0.5000	0.0000
4	Ni	1.7379	5.2138	0.0000	0.2500	0.7500	0.0000
5	Ni	3.4758	0.0000	0.0000	0.5000	0.0000	0.0000
6	Ni	5.2138	1.7379	0.0000	0.7500	0.2500	0.0000
7	Ni	3.4758	3.4758	0.0000	0.5000	0.5000	0.0000
8	Ni	5.2138	5.2138	0.0000	0.7500	0.7500	0.0000
9	Ni	0.0018	0.0035	3.5178	0.0003	0.0005	0.1603
10	Ni	1.7264	1.7351	3.5204	0.2483	0.2496	0.1604
11	Ni	-0.0015	3.4758	3.5371	-0.0002	0.5000	0.1611
12	Ni	1.7286	5.2148	3.5187	0.2487	0.7502	0.1603
13	Ni	5.2258	1.7403	3.5211	0.7517	0.2503	0.1604
14	Ni	1.7624	3.4888	5.2964	0.2535	0.5019	0.2413
15	Ni	5.2262	5.2111	3.5105	0.7518	0.7496	0.1599
16	Ni	3.4862	1.7597	5.2826	0.5015	0.2531	0.2406
17	Co	1.7379	0.0000	1.7379	0.2500	0.0000	0.0792
18	Co	0.0000	1.7379	1.7379	0.0000	0.2500	0.0792
19	Co	1.7379	3.4758	1.7379	0.2500	0.5000	0.0792
20	Co	0.0000	5.2138	1.7379	0.0000	0.7500	0.0792
21	Co	5.2138	0.0000	1.7379	0.7500	0.0000	0.0792
22	Co	3.4758	1.7379	1.7379	0.5000	0.2500	0.0792
23	Co	5.2138	3.4758	1.7379	0.7500	0.5000	0.0792
24	Co	3.4758	5.2138	1.7379	0.5000	0.7500	0.0792
25	Co	1.7513	-0.0216	5.2280	0.2519	-0.0031	0.2382
26	Co	6.9308	1.7148	5.2511	0.9970	0.2467	0.2392
27	Co	3.4862	3.4872	3.4552	0.5015	0.5016	0.1574
28	Co	6.9240	5.2489	5.2480	0.9960	0.7551	0.2391
29	Co	5.2155	6.9262	5.2279	0.7502	0.9963	0.2382
30	Co	5.1954	3.4994	5.2685	0.7474	0.5034	0.2400
31	Co	3.4967	5.2097	5.2454	0.5030	0.7494	0.2390
32	Co	3.4775	-0.0073	3.5136	0.5002	-0.0011	0.1601
33	O	3.4560	3.4669	7.6169	0.4971	0.4987	0.3470
34	C	3.4468	3.4432	6.4040	0.4958	0.4953	0.2917

/db/jmorales/CoNi-alloy/Slabs/Segregation/CoNi(111)/CoNi(111)-2-Ni

a =		9.831199646					
b =		9.831199646075708					
c =		21.0202999115					
alpha =		90.0					
beta =		90.0					
gamma =		120.0					

	Atom	X	Y	Z	X	Y	Z
1	Ni	0.0000	0.0000	0.0000	0.0000	0.0000	0.0000
2	Ni	-1.2289	2.1285	0.0000	0.0000	0.2500	0.0000
3	Ni	-2.4578	4.2570	0.0000	0.0000	0.5000	0.0000
4	Ni	-3.6867	6.3856	0.0000	0.0000	0.7500	0.0000
5	Ni	4.9156	0.0000	0.0000	0.5000	0.0000	0.0000
6	Ni	3.6867	2.1285	0.0000	0.5000	0.2500	0.0000
7	Ni	2.4578	4.2570	0.0000	0.5000	0.5000	0.0000
8	Ni	1.2289	6.3856	0.0000	0.5000	0.7500	0.0000
9	Ni	2.4578	1.4190	2.0068	0.3333	0.1667	0.0955
10	Ni	1.2289	3.5475	2.0068	0.3333	0.4167	0.0955
11	Ni	0.0000	5.6760	2.0068	0.3333	0.6667	0.0955
12	Ni	-1.2289	7.8045	2.0068	0.3333	0.9167	0.0955
13	Ni	7.3734	1.4190	2.0068	0.8333	0.1667	0.0955
14	Ni	6.1445	3.5475	2.0068	0.8333	0.4167	0.0955
15	Ni	4.9156	5.6760	2.0068	0.8333	0.6667	0.0955
16	Ni	3.6867	7.8045	2.0068	0.8333	0.9167	0.0955
17	Ni	0.0315	2.8573	4.0045	0.1710	0.3356	0.1905
18	Ni	-1.1977	4.9813	4.0121	0.1707	0.5851	0.1909
19	Ni	4.9468	2.8565	4.0128	0.6709	0.3355	0.1909
20	Ni	6.1742	0.7265	4.0095	0.6707	0.0853	0.1907
21	Ni	2.4875	7.1122	4.0088	0.6707	0.8353	0.1907
22	Ni	3.7168	4.9840	4.0116	0.6708	0.5854	0.1908
23	Ni	0.0452	0.0269	6.0334	0.0062	0.0032	0.2870
24	Ni	-1.1867	2.1592	6.0290	0.0061	0.2536	0.2868
25	Ni	-2.4154	4.2831	6.0272	0.0058	0.5031	0.2867
26	Ni	-3.6409	6.4065	6.0405	0.0059	0.7525	0.2874
27	Ni	4.9637	0.0294	6.0214	0.5066	0.0034	0.2865
28	Ni	3.7415	2.1627	6.0165	0.5076	0.2540	0.2862
29	Ni	2.5097	4.2882	6.0212	0.5071	0.5037	0.2864
30	Ni	1.2802	6.4150	6.0213	0.5070	0.7535	0.2864
31	Ni	1.2797	2.1663	6.0346	0.2574	0.2544	0.2871
32	Ni	2.5115	0.0262	6.0288	0.2570	0.0031	0.2868
33	Co	1.2289	2.1285	0.0000	0.2500	0.2500	0.0000
34	Co	2.4578	0.0000	0.0000	0.2500	0.0000	0.0000
35	Co	-1.2289	6.3856	0.0000	0.2500	0.7500	0.0000
36	Co	0.0000	4.2570	0.0000	0.2500	0.5000	0.0000
37	Co	6.1445	2.1285	0.0000	0.7500	0.2500	0.0000
38	Co	7.3734	0.0000	0.0000	0.7500	0.0000	0.0000
39	Co	3.6867	6.3856	0.0000	0.7500	0.7500	0.0000
40	Co	4.9156	4.2570	0.0000	0.7500	0.5000	0.0000
41	Co	-1.2289	3.5475	2.0068	0.0833	0.4167	0.0955

42	Co	0.0000	1.4190	2.0068	0.0833	0.1667	0.0955
43	Co	-3.6867	7.8045	2.0068	0.0833	0.9167	0.0955
44	Co	-2.4578	5.6760	2.0068	0.0833	0.6667	0.0955
45	Co	3.6867	3.5475	2.0068	0.5833	0.4167	0.0955
46	Co	4.9156	1.4190	2.0068	0.5833	0.1667	0.0955
47	Co	1.2289	7.8045	2.0068	0.5833	0.9167	0.0955
48	Co	2.4578	5.6760	2.0068	0.5833	0.6667	0.0955
49	Co	3.7053	0.7236	4.0331	0.4194	0.0850	0.1919
50	Co	2.4874	2.8451	4.0380	0.4201	0.3342	0.1921
51	Co	1.2571	4.9810	4.0493	0.4204	0.5850	0.1926
52	Co	0.0189	7.1114	4.0494	0.4196	0.8353	0.1926
53	Co	8.6370	0.7302	4.0547	0.9214	0.0858	0.1929
54	Co	7.4053	2.8549	4.0499	0.9209	0.3353	0.1927
55	Co	6.1790	4.9928	4.0489	0.9217	0.5864	0.1926
56	Co	4.9546	7.1176	4.0593	0.9220	0.8360	0.1931
57	Co	-1.1685	6.4159	5.9996	0.2579	0.7536	0.2854
58	Co	0.0570	4.3077	6.0073	0.2588	0.5059	0.2858
59	Co	6.2124	2.1682	6.0086	0.7592	0.2547	0.2858
60	Co	7.4341	0.0364	6.0092	0.7583	0.0043	0.2859
61	Co	3.7474	6.4215	6.0112	0.7583	0.7542	0.2860
62	Co	4.9836	4.2963	6.0111	0.7592	0.5046	0.2860
63	Co	1.2436	0.7238	3.9905	0.1690	0.0850	0.1898
64	Co	-2.4256	7.1006	4.0133	0.1703	0.8340	0.1909

/db/jmorales/CoNi-alloy/Slabs/Segregation/CoNi(001)/CoNi(001)-4-Co-CO

a =	6.9516692132
b =	6.9516692132
c =	21.9516692133
alpha =	90.0
beta =	90.0
gamma =	90.0

	Atom	X	Y	Z	X	Y	Z
1	Ni	0.0000	0.0000	0.0000	0.0000	0.0000	0.0000
2	Ni	0.0000	3.4758	0.0000	0.0000	0.5000	0.0000
3	Ni	3.4758	0.0000	0.0000	0.5000	0.0000	0.0000
4	Ni	3.4758	3.4758	0.0000	0.5000	0.5000	0.0000
5	Ni	0.0000	1.7379	1.7379	0.0000	0.2500	0.0792
6	Ni	0.0000	5.2138	1.7379	0.0000	0.7500	0.0792
7	Ni	3.4758	1.7379	1.7379	0.5000	0.2500	0.0792
8	Ni	3.4758	5.2138	1.7379	0.5000	0.7500	0.0792
9	Ni	-0.0003	-0.0001	3.5221	-0.0000	-0.0000	0.1604
10	Ni	-0.0001	3.4757	3.5379	-0.0000	0.5000	0.1612
11	Ni	3.4755	6.9515	3.5427	0.4999	1.0000	0.1614
12	Ni	3.4754	3.4758	3.4869	0.4999	0.5000	0.1588
13	Ni	1.7364	1.7364	3.5463	0.2498	0.2498	0.1615
14	Ni	1.7364	5.2150	3.5463	0.2498	0.7502	0.1616
15	Ni	5.2154	1.7367	3.5465	0.7502	0.2498	0.1616
16	Ni	5.2155	5.2148	3.5465	0.7502	0.7502	0.1616
17	Co	1.7379	1.7379	0.0000	0.2500	0.2500	0.0000
18	Co	1.7379	5.2138	0.0000	0.2500	0.7500	0.0000
19	Co	5.2138	1.7379	0.0000	0.7500	0.2500	0.0000
20	Co	5.2138	5.2138	0.0000	0.7500	0.7500	0.0000
21	Co	1.7379	0.0000	1.7379	0.2500	0.0000	0.0792
22	Co	1.7379	3.4758	1.7379	0.2500	0.5000	0.0792
23	Co	5.2138	0.0000	1.7379	0.7500	0.0000	0.0792
24	Co	5.2138	3.4758	1.7379	0.7500	0.5000	0.0792
25	Co	3.4756	1.7647	5.2746	0.5000	0.2539	0.2403
26	Co	1.7392	6.9514	5.2591	0.2502	1.0000	0.2396
27	Co	1.7574	3.4758	5.2884	0.2528	0.5000	0.2409
28	Co	5.2117	-0.0001	5.2578	0.7497	-0.0000	0.2395
29	Co	5.1941	3.4756	5.2879	0.7472	0.5000	0.2409
30	Co	3.4756	5.1865	5.2746	0.5000	0.7461	0.2403
31	Co	-0.0003	1.7274	5.2495	-0.0000	0.2485	0.2391
32	Co	0.0000	5.2239	5.2495	0.0000	0.7515	0.2391
33	O	3.4765	3.4755	7.6614	0.5001	0.4999	0.3490
34	C	3.4763	3.4756	6.4483	0.5001	0.5000	0.2938

/db/jmorales/CoNi-alloy/Slabs/Islanding/CoNi(111)/CoNi(111)-4-Ni-CO

a = 9.831199646
b = 9.831199646075708
c = 21.0202999115
alpha = 90.0
beta = 90.0
gamma = 120.0

	Atom	X	Y	Z	X	Y	Z
1	Ni	0.0000	0.0000	0.0000	0.0000	0.0000	0.0000
2	Ni	-1.2289	2.1285	0.0000	0.0000	0.2500	0.0000
3	Ni	-2.4578	4.2570	0.0000	0.0000	0.5000	0.0000
4	Ni	-3.6867	6.3856	0.0000	0.0000	0.7500	0.0000
5	Ni	4.9156	0.0000	0.0000	0.5000	0.0000	0.0000
6	Ni	3.6867	2.1285	0.0000	0.5000	0.2500	0.0000
7	Ni	2.4578	4.2570	0.0000	0.5000	0.5000	0.0000
8	Ni	1.2289	6.3856	0.0000	0.5000	0.7500	0.0000
9	Ni	2.4578	1.4190	2.0068	0.3333	0.1667	0.0955
10	Ni	1.2289	3.5475	2.0068	0.3333	0.4167	0.0955
11	Ni	0.0000	5.6760	2.0068	0.3333	0.6667	0.0955
12	Ni	-1.2289	7.8045	2.0068	0.3333	0.9167	0.0955
13	Ni	7.3734	1.4190	2.0068	0.8333	0.1667	0.0955
14	Ni	6.1445	3.5475	2.0068	0.8333	0.4167	0.0955
15	Ni	4.9156	5.6760	2.0068	0.8333	0.6667	0.0955
16	Ni	3.6867	7.8045	2.0068	0.8333	0.9167	0.0955
17	Ni	0.0382	2.8579	4.0036	0.1717	0.3357	0.1905
18	Ni	1.2599	0.7294	4.0083	0.1710	0.0857	0.1907
19	Ni	-2.4267	7.1158	4.0171	0.1710	0.8358	0.1911
20	Ni	-1.1934	4.9823	4.0063	0.1712	0.5852	0.1906
21	Ni	4.9414	2.8535	4.0080	0.6702	0.3352	0.1907
22	Ni	6.1693	0.7306	4.0022	0.6704	0.0858	0.1904
23	Ni	2.4937	7.1116	4.0201	0.6713	0.8353	0.1912
24	Ni	3.7209	4.9809	4.0166	0.6710	0.5850	0.1911
25	Ni	-1.1871	2.1511	6.0302	0.0056	0.2527	0.2869
26	Ni	-2.4060	4.2975	6.0321	0.0076	0.5048	0.2870
27	Ni	3.7381	2.1562	6.0891	0.5069	0.2532	0.2897
28	Ni	2.5134	4.2940	6.0877	0.5078	0.5043	0.2896
29	Ni	1.2722	2.1472	6.0925	0.2555	0.2522	0.2898
30	Ni	0.0275	4.3128	6.0375	0.2561	0.5065	0.2872
31	Ni	4.9838	4.3153	6.0427	0.7604	0.5068	0.2875
32	Ni	6.2011	2.1487	6.0439	0.7569	0.2524	0.2875
33	Co	1.2289	2.1285	0.0000	0.2500	0.2500	0.0000
34	Co	2.4578	0.0000	0.0000	0.2500	0.0000	0.0000
35	Co	-1.2289	6.3856	0.0000	0.2500	0.7500	0.0000
36	Co	0.0000	4.2570	0.0000	0.2500	0.5000	0.0000
37	Co	6.1445	2.1285	0.0000	0.7500	0.2500	0.0000
38	Co	7.3734	0.0000	0.0000	0.7500	0.0000	0.0000
39	Co	3.6867	6.3856	0.0000	0.7500	0.7500	0.0000
40	Co	4.9156	4.2570	0.0000	0.7500	0.5000	0.0000
41	Co	-1.2289	3.5475	2.0068	0.0833	0.4167	0.0955

42	Co	0.0000	1.4190	2.0068	0.0833	0.1667	0.0955
43	Co	-3.6867	7.8045	2.0068	0.0833	0.9167	0.0955
44	Co	-2.4578	5.6760	2.0068	0.0833	0.6667	0.0955
45	Co	3.6867	3.5475	2.0068	0.5833	0.4167	0.0955
46	Co	4.9156	1.4190	2.0068	0.5833	0.1667	0.0955
47	Co	1.2289	7.8045	2.0068	0.5833	0.9167	0.0955
48	Co	2.4578	5.6760	2.0068	0.5833	0.6667	0.0955
49	Co	3.7067	0.7252	4.0569	0.4196	0.0852	0.1930
50	Co	2.4977	2.8625	4.0055	0.4222	0.3362	0.1906
51	Co	1.2656	4.9857	4.0567	0.4215	0.5856	0.1930
52	Co	0.0343	7.1184	4.0520	0.4215	0.8361	0.1928
53	Co	8.6342	0.7254	4.0493	0.9208	0.0852	0.1926
54	Co	7.4084	2.8597	4.0433	0.9215	0.3359	0.1924
55	Co	6.1780	4.9874	4.0495	0.9213	0.5858	0.1926
56	Co	4.9469	7.1149	4.0606	0.9210	0.8357	0.1932
57	Co	2.5039	-0.0068	6.0045	0.2543	-0.0008	0.2857
58	Co	-1.1692	6.4285	6.0164	0.2586	0.7550	0.2862
59	Co	7.4239	0.0226	6.0082	0.7565	0.0027	0.2858
60	Co	3.7385	6.4313	6.0292	0.7580	0.7554	0.2868
61	Co	0.0244	0.0240	5.9930	0.0039	0.0028	0.2851
62	Co	1.2782	6.3959	5.9993	0.5056	0.7512	0.2854
63	Co	-3.6405	6.4001	5.9963	0.0056	0.7517	0.2853
64	Co	4.9459	0.0284	5.9853	0.5047	0.0033	0.2847
65	O	2.5000	2.8570	8.6164	0.4221	0.3356	0.4099
66	C	2.4782	2.8465	7.4233	0.4192	0.3343	0.3532

/db/jmorales/CoNi-alloy/Slabs/Segregation/Co-CoNi(001)/Co-CoNi(001)-4-Ni

a =	6.9516692132
b =	6.9516692132
c =	21.9516692133
alpha =	90.0
beta =	90.0
gamma =	90.0

	Atom	X	Y	Z	X	Y	Z
1	Ni	0.0000	0.0000	0.0000	0.0000	0.0000	0.0000
2	Ni	1.7379	1.7379	0.0000	0.2500	0.2500	0.0000
3	Ni	0.0000	3.4758	0.0000	0.0000	0.5000	0.0000
4	Ni	1.7379	5.2138	0.0000	0.2500	0.7500	0.0000
5	Ni	3.4758	0.0000	0.0000	0.5000	0.0000	0.0000
6	Ni	5.2138	1.7379	0.0000	0.7500	0.2500	0.0000
7	Ni	3.4758	3.4758	0.0000	0.5000	0.5000	0.0000
8	Ni	5.2138	5.2138	0.0000	0.7500	0.7500	0.0000
9	Ni	-0.0025	0.0008	3.5031	-0.0004	0.0001	0.1596
10	Ni	1.7331	1.7302	3.5139	0.2493	0.2489	0.1601
11	Ni	0.0067	3.4760	3.5101	0.0010	0.5000	0.1599
12	Ni	1.7334	5.2225	3.5136	0.2494	0.7513	0.1601
13	Ni	1.7092	3.4766	5.2449	0.2459	0.5001	0.2389
14	Ni	3.4718	1.7226	5.2322	0.4994	0.2478	0.2384
15	Ni	5.2416	3.4775	5.2328	0.7540	0.5002	0.2384
16	Ni	3.4719	5.2307	5.2310	0.4994	0.7524	0.2383
17	Co	1.7379	0.0000	1.7379	0.2500	0.0000	0.0792
18	Co	0.0000	1.7379	1.7379	0.0000	0.2500	0.0792
19	Co	1.7379	3.4758	1.7379	0.2500	0.5000	0.0792
20	Co	0.0000	5.2138	1.7379	0.0000	0.7500	0.0792
21	Co	5.2138	0.0000	1.7379	0.7500	0.0000	0.0792
22	Co	3.4758	1.7379	1.7379	0.5000	0.2500	0.0792
23	Co	5.2138	3.4758	1.7379	0.7500	0.5000	0.0792
24	Co	3.4758	5.2138	1.7379	0.5000	0.7500	0.0792
25	Co	1.7144	0.0007	5.2339	0.2466	0.0001	0.2384
26	Co	6.9445	1.7017	5.2327	0.9990	0.2448	0.2384
27	Co	3.4738	3.4765	3.5195	0.4997	0.5001	0.1603
28	Co	6.9449	5.2520	5.2321	0.9990	0.7555	0.2383
29	Co	5.2353	-0.0001	5.2143	0.7531	-0.0000	0.2375
30	Co	3.4755	0.0016	3.5207	0.4999	0.0002	0.1604
31	Co	5.2225	1.7386	3.5247	0.7513	0.2501	0.1606
32	Co	5.2227	5.2145	3.5232	0.7513	0.7501	0.1605

/db/jmorales/CoNi-alloy/Slabs/Segregation/Ni-CoNi(001)/Ni-CoNi(001)-1-Co

a =	6.9516692132
b =	6.9516692132
c =	21.9516692133
alpha =	90.0
beta =	90.0
gamma =	90.0

	Atom	X	Y	Z	X	Y	Z
1	Co	0.0000	0.0000	0.0000	0.0000	0.0000	0.0000
2	Co	1.7379	1.7379	0.0000	0.2500	0.2500	0.0000
3	Co	0.0000	3.4758	0.0000	0.0000	0.5000	0.0000
4	Co	1.7379	5.2138	0.0000	0.2500	0.7500	0.0000
5	Co	3.4758	0.0000	0.0000	0.5000	0.0000	0.0000
6	Co	5.2138	1.7379	0.0000	0.7500	0.2500	0.0000
7	Co	3.4758	3.4758	0.0000	0.5000	0.5000	0.0000
8	Co	5.2138	5.2138	0.0000	0.7500	0.7500	0.0000
9	Co	-0.0023	0.0000	3.5614	-0.0003	0.0000	0.1622
10	Co	1.7349	1.7368	3.5422	0.2496	0.2498	0.1614
11	Co	0.0035	3.4758	3.5319	0.0005	0.5000	0.1609
12	Co	1.7349	5.2149	3.5422	0.2496	0.7502	0.1614
13	Co	3.4788	0.0000	3.5395	0.5004	0.0000	0.1612
14	Co	5.2235	1.7332	3.5432	0.7514	0.2493	0.1614
15	Co	1.7222	3.4758	5.1659	0.2477	0.5000	0.2353
16	Co	5.2235	5.2185	3.5432	0.7514	0.7507	0.1614
17	Ni	1.7379	0.0000	1.7379	0.2500	0.0000	0.0792
18	Ni	0.0000	1.7379	1.7379	0.0000	0.2500	0.0792
19	Ni	1.7379	3.4758	1.7379	0.2500	0.5000	0.0792
20	Ni	0.0000	5.2138	1.7379	0.0000	0.7500	0.0792
21	Ni	5.2138	0.0000	1.7379	0.7500	0.0000	0.0792
22	Ni	3.4758	1.7379	1.7379	0.5000	0.2500	0.0792
23	Ni	5.2138	3.4758	1.7379	0.7500	0.5000	0.0792
24	Ni	3.4758	5.2138	1.7379	0.5000	0.7500	0.0792
25	Ni	1.7477	0.0000	5.2136	0.2514	0.0000	0.2375
26	Ni	0.0018	1.7475	5.2198	0.0003	0.2514	0.2378
27	Ni	3.4768	3.4758	3.5189	0.5001	0.5000	0.1603
28	Ni	0.0018	5.2041	5.2198	0.0003	0.7486	0.2378
29	Ni	5.2028	0.0000	5.2210	0.7484	0.0000	0.2378
30	Ni	3.4757	1.7477	5.2118	0.5000	0.2514	0.2374
31	Ni	5.2218	3.4758	5.2136	0.7512	0.5000	0.2375
32	Ni	3.4757	5.2039	5.2118	0.5000	0.7486	0.2374

/db/jmorales/CoNi-alloy/Slabs/Segregation/Ni-CoNi(001)/Ni-CoNi(001)-2-Co-CO

a = 6.9516692132
b = 6.9516692132
c = 21.9516692133
alpha = 90.0
beta = 90.0
gamma = 90.0

	Atom	X	Y	Z	X	Y	Z
1	Co	0.0000	0.0000	0.0000	0.0000	0.0000	0.0000
2	Co	1.7379	1.7379	0.0000	0.2500	0.2500	0.0000
3	Co	0.0000	3.4758	0.0000	0.0000	0.5000	0.0000
4	Co	1.7379	5.2138	0.0000	0.2500	0.7500	0.0000
5	Co	3.4758	0.0000	0.0000	0.5000	0.0000	0.0000
6	Co	5.2138	1.7379	0.0000	0.7500	0.2500	0.0000
7	Co	3.4758	3.4758	0.0000	0.5000	0.5000	0.0000
8	Co	5.2138	5.2138	0.0000	0.7500	0.7500	0.0000
9	Co	-0.0010	0.0005	3.5568	-0.0001	0.0001	0.1620
10	Co	1.7371	1.7405	3.5594	0.2499	0.2504	0.1621
11	Co	6.9493	3.4777	3.5510	0.9997	0.5003	0.1618
12	Co	1.7305	5.2106	3.5510	0.2489	0.7495	0.1618
13	Co	5.2170	1.7385	3.5549	0.7505	0.2501	0.1619
14	Co	1.7404	3.4790	5.2150	0.2504	0.5005	0.2376
15	Co	5.2301	5.2163	3.5399	0.7524	0.7504	0.1613
16	Co	3.4690	1.7652	5.2237	0.4990	0.2539	0.2380
17	Ni	1.7379	0.0000	1.7379	0.2500	0.0000	0.0792
18	Ni	0.0000	1.7379	1.7379	0.0000	0.2500	0.0792
19	Ni	1.7379	3.4758	1.7379	0.2500	0.5000	0.0792
20	Ni	0.0000	5.2138	1.7379	0.0000	0.7500	0.0792
21	Ni	5.2138	0.0000	1.7379	0.7500	0.0000	0.0792
22	Ni	3.4758	1.7379	1.7379	0.5000	0.2500	0.0792
23	Ni	5.2138	3.4758	1.7379	0.7500	0.5000	0.0792
24	Ni	3.4758	5.2138	1.7379	0.5000	0.7500	0.0792
25	Ni	1.7536	-0.0012	5.2159	0.2523	-0.0002	0.2376
26	Ni	-0.0008	1.7423	5.2192	-0.0001	0.2506	0.2378
27	Ni	3.4743	3.4788	3.4795	0.4998	0.5004	0.1585
28	Ni	6.9486	5.2132	5.2197	0.9996	0.7499	0.2378
29	Ni	5.2009	-0.0015	5.2163	0.7481	-0.0002	0.2376
30	Ni	5.1885	3.4768	5.2639	0.7464	0.5001	0.2398
31	Ni	3.4758	5.1810	5.2720	0.5000	0.7453	0.2402
32	Ni	3.4765	-0.0095	3.5338	0.5001	-0.0014	0.1610
33	O	3.4219	3.4656	7.5683	0.4922	0.4985	0.3448
34	C	3.4983	3.4816	6.3553	0.5032	0.5008	0.2895

/db/jmorales/CoNi-alloy/Slabs/Segregation/CoNi(001)/CoNi(001)-4-Ni-CO

a =	6.9516692132
b =	6.9516692132
c =	21.9516692133
alpha =	90.0
beta =	90.0
gamma =	90.0

	Atom	X	Y	Z	X	Y	Z
1	Ni	0.0000	0.0000	0.0000	0.0000	0.0000	0.0000
2	Ni	0.0000	3.4758	0.0000	0.0000	0.5000	0.0000
3	Ni	3.4758	0.0000	0.0000	0.5000	0.0000	0.0000
4	Ni	3.4758	3.4758	0.0000	0.5000	0.5000	0.0000
5	Ni	0.0000	1.7379	1.7379	0.0000	0.2500	0.0792
6	Ni	0.0000	5.2138	1.7379	0.0000	0.7500	0.0792
7	Ni	3.4758	1.7379	1.7379	0.5000	0.2500	0.0792
8	Ni	3.4758	5.2138	1.7379	0.5000	0.7500	0.0792
9	Ni	1.7573	3.4760	5.2437	0.2528	0.5000	0.2389
10	Ni	-0.0001	1.7379	5.1960	-0.0000	0.2500	0.2367
11	Ni	6.9515	5.2138	5.1959	1.0000	0.7500	0.2367
12	Ni	3.4757	1.7640	5.2447	0.5000	0.2538	0.2389
13	Ni	3.4758	5.1879	5.2442	0.5000	0.7463	0.2389
14	Ni	5.1940	3.4759	5.2435	0.7472	0.5000	0.2389
15	Ni	1.7447	-0.0002	5.1929	0.2510	-0.0000	0.2366
16	Ni	5.2067	-0.0001	5.1928	0.7490	-0.0000	0.2366
17	Co	1.7379	1.7379	0.0000	0.2500	0.2500	0.0000
18	Co	1.7379	5.2138	0.0000	0.2500	0.7500	0.0000
19	Co	5.2138	1.7379	0.0000	0.7500	0.2500	0.0000
20	Co	5.2138	5.2138	0.0000	0.7500	0.7500	0.0000
21	Co	1.7379	0.0000	1.7379	0.2500	0.0000	0.0792
22	Co	1.7379	3.4758	1.7379	0.2500	0.5000	0.0792
23	Co	5.2138	0.0000	1.7379	0.7500	0.0000	0.0792
24	Co	5.2138	3.4758	1.7379	0.7500	0.5000	0.0792
25	Co	1.7323	1.7347	3.5219	0.2492	0.2495	0.1604
26	Co	1.7321	5.2171	3.5218	0.2492	0.7505	0.1604
27	Co	5.2192	1.7347	3.5219	0.7508	0.2495	0.1604
28	Co	5.2193	5.2171	3.5218	0.7508	0.7505	0.1604
29	Co	3.4758	3.4758	3.4510	0.5000	0.5000	0.1572
30	Co	-0.0000	3.4758	3.5005	-0.0000	0.5000	0.1595
31	Co	-0.0001	0.0007	3.5057	-0.0000	0.0001	0.1597
32	Co	3.4757	0.0001	3.5011	0.5000	0.0000	0.1595
33	O	3.4755	3.4769	7.5565	0.5000	0.5001	0.3442
34	C	3.4757	3.4761	6.3427	0.5000	0.5000	0.2889

/db/jmorales/CoNi-alloy/Slabs/Segregation/CoNi(111)/CoNi(111)-3-Co

a =			9.831199646				
b =			9.831199646075708				
c =			21.0202999115				
alpha =			90.0				
beta =			90.0				
gamma =			120.0				
	Atom	X	Y	Z	X	Y	Z
1	Ni	0.0000	0.0000	0.0000	0.0000	0.0000	0.0000
2	Ni	-1.2289	2.1285	0.0000	0.0000	0.2500	0.0000
3	Ni	-2.4578	4.2570	0.0000	0.0000	0.5000	0.0000
4	Ni	-3.6867	6.3856	0.0000	0.0000	0.7500	0.0000
5	Ni	4.9156	0.0000	0.0000	0.5000	0.0000	0.0000
6	Ni	3.6867	2.1285	0.0000	0.5000	0.2500	0.0000
7	Ni	2.4578	4.2570	0.0000	0.5000	0.5000	0.0000
8	Ni	1.2289	6.3856	0.0000	0.5000	0.7500	0.0000
9	Ni	2.4578	1.4190	2.0068	0.3333	0.1667	0.0955
10	Ni	1.2289	3.5475	2.0068	0.3333	0.4167	0.0955
11	Ni	0.0000	5.6760	2.0068	0.3333	0.6667	0.0955
12	Ni	-1.2289	7.8045	2.0068	0.3333	0.9167	0.0955
13	Ni	7.3734	1.4190	2.0068	0.8333	0.1667	0.0955
14	Ni	6.1445	3.5475	2.0068	0.8333	0.4167	0.0955
15	Ni	4.9156	5.6760	2.0068	0.8333	0.6667	0.0955
16	Ni	3.6867	7.8045	2.0068	0.8333	0.9167	0.0955
17	Ni	0.0261	2.8517	4.0190	0.1701	0.3349	0.1912
18	Ni	1.2530	0.7265	4.0217	0.1701	0.0853	0.1913
19	Ni	-2.4363	7.1056	4.0220	0.1695	0.8346	0.1913
20	Ni	-1.2131	4.9787	4.0251	0.1690	0.5848	0.1915
21	Ni	4.9426	2.8572	4.0210	0.6705	0.3356	0.1913
22	Ni	6.1825	0.7328	4.0197	0.6719	0.0861	0.1912
23	Ni	2.4884	7.1131	4.0200	0.6708	0.8355	0.1912
24	Ni	3.7212	4.9782	4.0204	0.6709	0.5847	0.1913
25	Ni	0.0377	0.0182	6.0406	0.0049	0.0021	0.2874
26	Ni	-1.1901	2.1531	6.0424	0.0054	0.2529	0.2875
27	Ni	-2.4251	4.2781	6.0419	0.0046	0.5025	0.2874
28	Ni	-3.6488	6.4066	6.0421	0.0051	0.7525	0.2874
29	Ni	4.9590	0.0214	6.0251	0.5057	0.0025	0.2866
30	Ni	3.7037	0.7358	4.0385	0.4199	0.0864	0.1921
31	Ni	1.2612	4.9724	4.0445	0.4203	0.5840	0.1924
32	Ni	0.0298	7.1078	4.0357	0.4205	0.8348	0.1920
33	Co	1.2289	2.1285	0.0000	0.2500	0.2500	0.0000
34	Co	2.4578	0.0000	0.0000	0.2500	0.0000	0.0000
35	Co	-1.2289	6.3856	0.0000	0.2500	0.7500	0.0000
36	Co	0.0000	4.2570	0.0000	0.2500	0.5000	0.0000
37	Co	6.1445	2.1285	0.0000	0.7500	0.2500	0.0000
38	Co	7.3734	0.0000	0.0000	0.7500	0.0000	0.0000
39	Co	3.6867	6.3856	0.0000	0.7500	0.7500	0.0000
40	Co	4.9156	4.2570	0.0000	0.7500	0.5000	0.0000
41	Co	-1.2289	3.5475	2.0068	0.0833	0.4167	0.0955

42	Co	0.0000	1.4190	2.0068	0.0833	0.1667	0.0955
43	Co	-3.6867	7.8045	2.0068	0.0833	0.9167	0.0955
44	Co	-2.4578	5.6760	2.0068	0.0833	0.6667	0.0955
45	Co	3.6867	3.5475	2.0068	0.5833	0.4167	0.0955
46	Co	4.9156	1.4190	2.0068	0.5833	0.1667	0.0955
47	Co	1.2289	7.8045	2.0068	0.5833	0.9167	0.0955
48	Co	2.4578	5.6760	2.0068	0.5833	0.6667	0.0955
49	Co	2.4828	2.8527	4.0727	0.4201	0.3351	0.1938
50	Co	8.6261	0.7224	4.0635	0.9199	0.0849	0.1933
51	Co	7.3980	2.8520	4.0632	0.9200	0.3350	0.1933
52	Co	6.1705	4.9826	4.0577	0.9202	0.5852	0.1930
53	Co	4.9393	7.1093	4.0593	0.9199	0.8350	0.1931
54	Co	1.2876	2.1671	6.0319	0.2582	0.2545	0.2870
55	Co	2.5112	0.0465	6.0319	0.2582	0.0055	0.2870
56	Co	-1.1585	6.4081	6.0306	0.2585	0.7527	0.2869
57	Co	0.0586	4.2864	6.0400	0.2577	0.5035	0.2873
58	Co	6.1868	2.1562	6.0275	0.7559	0.2533	0.2867
59	Co	7.4175	0.0245	6.0325	0.7559	0.0029	0.2870
60	Co	3.7362	6.4035	6.0291	0.7561	0.7521	0.2868
61	Co	4.9551	4.2884	6.0339	0.7559	0.5037	0.2871
62	Co	3.7237	2.1491	6.0107	0.5050	0.2524	0.2859
63	Co	2.4907	4.2806	6.0105	0.5047	0.5028	0.2859
64	Co	1.2634	6.4056	5.9977	0.5047	0.7524	0.2853

/db/jmorales/CoNi-alloy/Slabs/Segregation/CoNi(111)/CoNi(111)-4-Co

a =		9.831199646
b =		9.831199646075708
c =		21.0202999115
alpha =		90.0
beta =		90.0
gamma =		120.0

	Atom	X	Y	Z	X	Y	Z
1	Ni	0.0000	0.0000	0.0000	0.0000	0.0000	0.0000
2	Ni	-1.2289	2.1285	0.0000	0.0000	0.2500	0.0000
3	Ni	-2.4578	4.2570	0.0000	0.0000	0.5000	0.0000
4	Ni	-3.6867	6.3856	0.0000	0.0000	0.7500	0.0000
5	Ni	4.9156	0.0000	0.0000	0.5000	0.0000	0.0000
6	Ni	3.6867	2.1285	0.0000	0.5000	0.2500	0.0000
7	Ni	2.4578	4.2570	0.0000	0.5000	0.5000	0.0000
8	Ni	1.2289	6.3856	0.0000	0.5000	0.7500	0.0000
9	Ni	2.4578	1.4190	2.0068	0.3333	0.1667	0.0955
10	Ni	1.2289	3.5475	2.0068	0.3333	0.4167	0.0955
11	Ni	0.0000	5.6760	2.0068	0.3333	0.6667	0.0955
12	Ni	-1.2289	7.8045	2.0068	0.3333	0.9167	0.0955
13	Ni	7.3734	1.4190	2.0068	0.8333	0.1667	0.0955
14	Ni	6.1445	3.5475	2.0068	0.8333	0.4167	0.0955
15	Ni	4.9156	5.6760	2.0068	0.8333	0.6667	0.0955
16	Ni	3.6867	7.8045	2.0068	0.8333	0.9167	0.0955
17	Ni	0.0176	2.8484	4.0252	0.1691	0.3345	0.1915
18	Ni	1.2462	0.7189	4.0247	0.1690	0.0844	0.1915
19	Ni	-2.4402	7.1051	4.0259	0.1690	0.8345	0.1915
20	Ni	-1.2120	4.9761	4.0254	0.1689	0.5845	0.1915
21	Ni	4.9512	2.8582	4.0244	0.6715	0.3357	0.1915
22	Ni	6.1793	0.7295	4.0238	0.6714	0.0857	0.1914
23	Ni	2.4930	7.1153	4.0247	0.6714	0.8357	0.1915
24	Ni	3.7216	4.9866	4.0237	0.6714	0.5857	0.1914
25	Ni	0.0337	0.0193	6.0469	0.0046	0.0023	0.2877
26	Ni	-1.1940	2.1481	6.0468	0.0047	0.2523	0.2877
27	Ni	-2.4243	4.2759	6.0466	0.0045	0.5022	0.2877
28	Ni	-3.6526	6.4052	6.0466	0.0046	0.7523	0.2877
29	Ni	3.7151	0.7261	4.0427	0.4205	0.0853	0.1923
30	Ni	1.2573	4.9831	4.0422	0.4205	0.5853	0.1923
31	Ni	0.0292	7.1118	4.0428	0.4206	0.8353	0.1923
32	Ni	2.4872	2.8545	4.0416	0.4206	0.3353	0.1923
33	Co	1.2289	2.1285	0.0000	0.2500	0.2500	0.0000
34	Co	2.4578	0.0000	0.0000	0.2500	0.0000	0.0000
35	Co	-1.2289	6.3856	0.0000	0.2500	0.7500	0.0000
36	Co	0.0000	4.2570	0.0000	0.2500	0.5000	0.0000
37	Co	6.1445	2.1285	0.0000	0.7500	0.2500	0.0000
38	Co	7.3734	0.0000	0.0000	0.7500	0.0000	0.0000
39	Co	3.6867	6.3856	0.0000	0.7500	0.7500	0.0000
40	Co	4.9156	4.2570	0.0000	0.7500	0.5000	0.0000
41	Co	-1.2289	3.5475	2.0068	0.0833	0.4167	0.0955

42	Co	0.0000	1.4190	2.0068	0.0833	0.1667	0.0955
43	Co	-3.6867	7.8045	2.0068	0.0833	0.9167	0.0955
44	Co	-2.4578	5.6760	2.0068	0.0833	0.6667	0.0955
45	Co	3.6867	3.5475	2.0068	0.5833	0.4167	0.0955
46	Co	4.9156	1.4190	2.0068	0.5833	0.1667	0.0955
47	Co	1.2289	7.8045	2.0068	0.5833	0.9167	0.0955
48	Co	2.4578	5.6760	2.0068	0.5833	0.6667	0.0955
49	Co	8.6273	0.7247	4.0626	0.9201	0.0851	0.1933
50	Co	7.3987	2.8515	4.0629	0.9200	0.3349	0.1933
51	Co	6.1689	4.9813	4.0620	0.9200	0.5851	0.1932
52	Co	4.9406	7.1088	4.0629	0.9200	0.8349	0.1933
53	Co	1.2891	2.1616	6.0369	0.2581	0.2539	0.2872
54	Co	2.5168	0.0348	6.0393	0.2580	0.0041	0.2873
55	Co	-1.1688	6.4186	6.0384	0.2581	0.7539	0.2873
56	Co	0.0589	4.2922	6.0392	0.2581	0.5041	0.2873
57	Co	6.1803	2.1494	6.0414	0.7549	0.2525	0.2874
58	Co	7.4096	0.0206	6.0411	0.7549	0.0024	0.2874
59	Co	3.7223	6.4062	6.0415	0.7548	0.7524	0.2874
60	Co	4.9525	4.2780	6.0415	0.7550	0.5025	0.2874
61	Co	3.7230	2.1494	6.0042	0.5049	0.2525	0.2856
62	Co	2.4932	4.2773	6.0038	0.5048	0.5024	0.2856
63	Co	1.2654	6.4060	6.0053	0.5049	0.7524	0.2857
64	Co	4.9506	0.0208	6.0059	0.5048	0.0024	0.2857

/db/jmorales/CoNi-alloy/Slabs/Segregation/Co-CoNi(001)/Co-CoNi(001)-1-Ni-CO

a = 6.9516692132
b = 6.9516692132
c = 21.9516692133
alpha = 90.0
beta = 90.0
gamma = 90.0

	Atom	X	Y	Z	X	Y	Z
1	Ni	0.0000	0.0000	0.0000	0.0000	0.0000	0.0000
2	Ni	1.7379	1.7379	0.0000	0.2500	0.2500	0.0000
3	Ni	0.0000	3.4758	0.0000	0.0000	0.5000	0.0000
4	Ni	1.7379	5.2138	0.0000	0.2500	0.7500	0.0000
5	Ni	3.4758	0.0000	0.0000	0.5000	0.0000	0.0000
6	Ni	5.2138	1.7379	0.0000	0.7500	0.2500	0.0000
7	Ni	3.4758	3.4758	0.0000	0.5000	0.5000	0.0000
8	Ni	5.2138	5.2138	0.0000	0.7500	0.7500	0.0000
9	Ni	0.0023	0.0000	3.5230	0.0003	0.0000	0.1605
10	Ni	1.7336	1.7291	3.5301	0.2494	0.2487	0.1608
11	Ni	-0.0013	3.4758	3.5445	-0.0002	0.5000	0.1615
12	Ni	1.7336	5.2226	3.5301	0.2494	0.7513	0.1608
13	Ni	3.4755	0.0000	3.5177	0.5000	0.0000	0.1602
14	Ni	5.2216	1.7327	3.5200	0.7511	0.2492	0.1604
15	Ni	1.7655	3.4759	5.2998	0.2540	0.5000	0.2414
16	Ni	5.2216	5.2190	3.5200	0.7511	0.7508	0.1604
17	Co	1.7379	0.0000	1.7379	0.2500	0.0000	0.0792
18	Co	0.0000	1.7379	1.7379	0.0000	0.2500	0.0792
19	Co	1.7379	3.4758	1.7379	0.2500	0.5000	0.0792
20	Co	0.0000	5.2138	1.7379	0.0000	0.7500	0.0792
21	Co	5.2138	0.0000	1.7379	0.7500	0.0000	0.0792
22	Co	3.4758	1.7379	1.7379	0.5000	0.2500	0.0792
23	Co	5.2138	3.4758	1.7379	0.7500	0.5000	0.0792
24	Co	3.4758	5.2138	1.7379	0.5000	0.7500	0.0792
25	Co	1.7475	0.0000	5.2555	0.2514	0.0000	0.2394
26	Co	6.9266	1.7055	5.2660	0.9964	0.2453	0.2399
27	Co	3.4866	3.4759	3.4730	0.5015	0.5000	0.1582
28	Co	6.9266	5.2462	5.2660	0.9964	0.7547	0.2399
29	Co	5.2034	-0.0000	5.2443	0.7485	-0.0000	0.2389
30	Co	3.5008	1.7557	5.2788	0.5036	0.2526	0.2405
31	Co	5.1867	3.4759	5.2661	0.7461	0.5000	0.2399
32	Co	3.5008	5.1960	5.2788	0.5036	0.7474	0.2405
33	O	3.4607	3.4759	7.6441	0.4978	0.5000	0.3482
34	C	3.4519	3.4759	6.4302	0.4966	0.5000	0.2929

/db/jmorales/CoNi-alloy/Slabs/Ni-CoNi(001)-expanded

a =			7.044921511				
b =			7.044921511				
c =			22.044921511				
alpha =			90.0				
beta =			90.0				
gamma =			90.0				
	Atom	X	Y	Z	X	Y	Z
1	Co	0.0000	0.0000	0.0000	0.0000	0.0000	0.0000
2	Co	1.7612	1.7612	0.0000	0.2500	0.2500	0.0000
3	Co	0.0000	0.0000	3.5225	0.0000	0.0000	0.1598
4	Co	1.7612	1.7612	3.5225	0.2500	0.2500	0.1598
5	Co	0.0000	3.5225	0.0000	0.0000	0.5000	0.0000
6	Co	1.7612	5.2837	0.0000	0.2500	0.7500	0.0000
7	Co	0.0000	3.5225	3.5225	0.0000	0.5000	0.1598
8	Co	1.7612	5.2837	3.5225	0.2500	0.7500	0.1598
9	Co	3.5225	0.0000	0.0000	0.5000	0.0000	0.0000
10	Co	5.2837	1.7612	0.0000	0.7500	0.2500	0.0000
11	Co	3.5225	0.0000	3.5225	0.5000	0.0000	0.1598
12	Co	5.2837	1.7612	3.5225	0.7500	0.2500	0.1598
13	Co	3.5225	3.5225	0.0000	0.5000	0.5000	0.0000
14	Co	5.2837	5.2837	0.0000	0.7500	0.7500	0.0000
15	Co	3.5225	3.5225	3.5225	0.5000	0.5000	0.1598
16	Co	5.2837	5.2837	3.5225	0.7500	0.7500	0.1598
17	Ni	0.0000	1.7612	1.7612	0.0000	0.2500	0.0799
18	Ni	1.7612	0.0000	1.7612	0.2500	0.0000	0.0799
19	Ni	0.0000	1.7612	5.2837	0.0000	0.2500	0.2397
20	Ni	1.7612	0.0000	5.2837	0.2500	0.0000	0.2397
21	Ni	0.0000	5.2837	1.7612	0.0000	0.7500	0.0799
22	Ni	1.7612	3.5225	1.7612	0.2500	0.5000	0.0799
23	Ni	0.0000	5.2837	5.2837	0.0000	0.7500	0.2397
24	Ni	1.7612	3.5225	5.2837	0.2500	0.5000	0.2397
25	Ni	3.5225	1.7612	1.7612	0.5000	0.2500	0.0799
26	Ni	5.2837	0.0000	1.7612	0.7500	0.0000	0.0799
27	Ni	3.5225	1.7612	5.2837	0.5000	0.2500	0.2397
28	Ni	5.2837	0.0000	5.2837	0.7500	0.0000	0.2397
29	Ni	3.5225	5.2837	1.7612	0.5000	0.7500	0.0799
30	Ni	5.2837	3.5225	1.7612	0.7500	0.5000	0.0799
31	Ni	3.5225	5.2837	5.2837	0.5000	0.7500	0.2397
32	Ni	5.2837	3.5225	5.2837	0.7500	0.5000	0.2397

/db/jmorales/CoNi-alloy/Slabs/Islanding/CoNi(111)/CoNi(111)-1-Co

a =			9.831199646				
b =			9.831199646075708				
c =			21.0202999115				
alpha =			90.0				
beta =			90.0				
gamma =			120.0				
	Atom	X	Y	Z	X	Y	Z
1	Ni	0.0000	0.0000	0.0000	0.0000	0.0000	0.0000
2	Ni	-1.2289	2.1285	0.0000	0.0000	0.2500	0.0000
3	Ni	-2.4578	4.2570	0.0000	0.0000	0.5000	0.0000
4	Ni	-3.6867	6.3856	0.0000	0.0000	0.7500	0.0000
5	Ni	4.9156	0.0000	0.0000	0.5000	0.0000	0.0000
6	Ni	3.6867	2.1285	0.0000	0.5000	0.2500	0.0000
7	Ni	2.4578	4.2570	0.0000	0.5000	0.5000	0.0000
8	Ni	1.2289	6.3856	0.0000	0.5000	0.7500	0.0000
9	Ni	2.4578	1.4190	2.0068	0.3333	0.1667	0.0955
10	Ni	1.2289	3.5475	2.0068	0.3333	0.4167	0.0955
11	Ni	0.0000	5.6760	2.0068	0.3333	0.6667	0.0955
12	Ni	-1.2289	7.8045	2.0068	0.3333	0.9167	0.0955
13	Ni	7.3734	1.4190	2.0068	0.8333	0.1667	0.0955
14	Ni	6.1445	3.5475	2.0068	0.8333	0.4167	0.0955
15	Ni	4.9156	5.6760	2.0068	0.8333	0.6667	0.0955
16	Ni	3.6867	7.8045	2.0068	0.8333	0.9167	0.0955
17	Ni	0.0362	2.8587	4.0157	0.1716	0.3358	0.1910
18	Ni	1.2647	0.7298	4.0076	0.1715	0.0857	0.1907
19	Ni	-2.4227	7.1144	4.0055	0.1714	0.8356	0.1906
20	Ni	-1.1939	4.9869	4.0148	0.1714	0.5857	0.1910
21	Ni	4.9509	2.8605	4.0093	0.6716	0.3360	0.1907
22	Ni	6.1795	0.7279	4.0150	0.6713	0.0855	0.1910
23	Ni	2.4923	7.1156	4.0129	0.6714	0.8357	0.1909
24	Ni	3.7188	4.9865	4.0113	0.6711	0.5857	0.1908
25	Ni	0.0543	0.0334	6.0287	0.0075	0.0039	0.2868
26	Ni	-1.1771	2.1650	6.0277	0.0074	0.2543	0.2868
27	Ni	-2.4032	4.2845	6.0237	0.0072	0.5032	0.2866
28	Ni	-3.6306	6.4104	6.0284	0.0072	0.7529	0.2868
29	Ni	4.9776	0.0319	6.0282	0.5082	0.0038	0.2868
30	Ni	2.5140	4.2976	6.0248	0.5081	0.5048	0.2866
31	Ni	1.2902	6.4168	6.0274	0.5081	0.7537	0.2867
32	Ni	2.5262	0.0417	6.0567	0.2594	0.0049	0.2881
33	Co	1.2289	2.1285	0.0000	0.2500	0.2500	0.0000
34	Co	2.4578	0.0000	0.0000	0.2500	0.0000	0.0000
35	Co	-1.2289	6.3856	0.0000	0.2500	0.7500	0.0000
36	Co	0.0000	4.2570	0.0000	0.2500	0.5000	0.0000
37	Co	6.1445	2.1285	0.0000	0.7500	0.2500	0.0000
38	Co	7.3734	0.0000	0.0000	0.7500	0.0000	0.0000
39	Co	3.6867	6.3856	0.0000	0.7500	0.7500	0.0000
40	Co	4.9156	4.2570	0.0000	0.7500	0.5000	0.0000
41	Co	-1.2289	3.5475	2.0068	0.0833	0.4167	0.0955

42	Co	0.0000	1.4190	2.0068	0.0833	0.1667	0.0955
43	Co	-3.6867	7.8045	2.0068	0.0833	0.9167	0.0955
44	Co	-2.4578	5.6760	2.0068	0.0833	0.6667	0.0955
45	Co	3.6867	3.5475	2.0068	0.5833	0.4167	0.0955
46	Co	4.9156	1.4190	2.0068	0.5833	0.1667	0.0955
47	Co	1.2289	7.8045	2.0068	0.5833	0.9167	0.0955
48	Co	2.4578	5.6760	2.0068	0.5833	0.6667	0.0955
49	Co	3.7217	0.7373	4.0514	0.4219	0.0866	0.1927
50	Co	2.4925	2.8512	4.0592	0.4210	0.3349	0.1931
51	Co	1.2581	4.9838	4.0548	0.4207	0.5854	0.1929
52	Co	0.0300	7.1133	4.0543	0.4208	0.8355	0.1929
53	Co	8.6344	0.7272	4.0583	0.9210	0.0854	0.1931
54	Co	7.4068	2.8574	4.0471	0.9212	0.3356	0.1925
55	Co	6.1729	4.9849	4.0557	0.9206	0.5855	0.1929
56	Co	4.9502	7.1143	4.0576	0.9213	0.8356	0.1930
57	Co	1.3038	2.1763	6.0196	0.2604	0.2556	0.2864
58	Co	-1.1468	6.4198	6.0158	0.2604	0.7540	0.2862
59	Co	0.0810	4.3034	6.0171	0.2610	0.5054	0.2862
60	Co	6.2149	2.1741	6.0128	0.7598	0.2554	0.2860
61	Co	7.4466	0.0478	6.0181	0.7603	0.0056	0.2863
62	Co	3.7618	6.4240	6.0186	0.7599	0.7545	0.2863
63	Co	4.9876	4.2929	6.0172	0.7594	0.5042	0.2863
64	Co	3.7401	2.1505	5.9905	0.5067	0.2526	0.2850

/db/jmorales/CoNi-alloy/Slabs/Ni(111)

a = 9.829999924
b = 9.829999923668225
c = 21.019699097
alpha = 90.0
beta = 90.0
gamma = 120.0

	Atom	X	Y	Z	X	Y	Z
1	Ni	0.0000	0.0000	0.0000	0.0000	0.0000	0.0000
2	Ni	-1.2288	2.1283	0.0000	0.0000	0.2500	0.0000
3	Ni	-2.4575	4.2565	0.0000	-0.0000	0.5000	0.0000
4	Ni	-3.6862	6.3848	0.0000	-0.0000	0.7500	0.0000
5	Ni	2.4575	0.0000	0.0000	0.2500	0.0000	0.0000
6	Ni	1.2288	2.1283	0.0000	0.2500	0.2500	0.0000
7	Ni	-0.0000	4.2565	0.0000	0.2500	0.5000	0.0000
8	Ni	-1.2288	6.3848	0.0000	0.2500	0.7500	0.0000
9	Ni	4.9150	0.0000	0.0000	0.5000	0.0000	0.0000
10	Ni	3.6862	2.1283	0.0000	0.5000	0.2500	0.0000
11	Ni	2.4575	4.2565	0.0000	0.5000	0.5000	0.0000
12	Ni	1.2288	6.3848	0.0000	0.5000	0.7500	0.0000
13	Ni	7.3725	0.0000	0.0000	0.7500	0.0000	0.0000
14	Ni	6.1438	2.1283	0.0000	0.7500	0.2500	0.0000
15	Ni	4.9150	4.2565	0.0000	0.7500	0.5000	0.0000
16	Ni	3.6862	6.3848	0.0000	0.7500	0.7500	0.0000
17	Ni	-0.0000	1.4188	2.0065	0.0833	0.1667	0.0955
18	Ni	-1.2288	3.5471	2.0065	0.0833	0.4167	0.0955
19	Ni	-2.4575	5.6754	2.0065	0.0833	0.6667	0.0955
20	Ni	-3.6863	7.8036	2.0065	0.0833	0.9167	0.0955
21	Ni	2.4575	1.4188	2.0065	0.3333	0.1667	0.0955
22	Ni	1.2287	3.5471	2.0065	0.3333	0.4167	0.0955
23	Ni	-0.0000	5.6754	2.0065	0.3333	0.6667	0.0955
24	Ni	-1.2288	7.8036	2.0065	0.3333	0.9167	0.0955
25	Ni	4.9150	1.4188	2.0065	0.5833	0.1667	0.0955
26	Ni	3.6862	3.5471	2.0065	0.5833	0.4167	0.0955
27	Ni	2.4575	5.6754	2.0065	0.5833	0.6667	0.0955
28	Ni	1.2287	7.8036	2.0065	0.5833	0.9167	0.0955
29	Ni	7.3725	1.4188	2.0065	0.8333	0.1667	0.0955
30	Ni	6.1437	3.5471	2.0065	0.8333	0.4167	0.0955
31	Ni	4.9150	5.6754	2.0065	0.8333	0.6667	0.0955
32	Ni	3.6862	7.8036	2.0065	0.8333	0.9167	0.0955
33	Ni	1.2288	0.7094	4.0131	0.1667	0.0833	0.1909
34	Ni	0.0000	2.8377	4.0131	0.1667	0.3333	0.1909
35	Ni	-1.2287	4.9659	4.0131	0.1667	0.5833	0.1909
36	Ni	-2.4575	7.0942	4.0131	0.1667	0.8333	0.1909
37	Ni	3.6863	0.7094	4.0131	0.4167	0.0833	0.1909
38	Ni	2.4575	2.8377	4.0131	0.4167	0.3333	0.1909
39	Ni	1.2288	4.9659	4.0131	0.4167	0.5833	0.1909
40	Ni	0.0000	7.0942	4.0131	0.4167	0.8333	0.1909
41	Ni	6.1438	0.7094	4.0131	0.6667	0.0833	0.1909

42	Ni	4.9150	2.8377	4.0131	0.6667	0.3333	0.1909
43	Ni	3.6863	4.9659	4.0131	0.6667	0.5833	0.1909
44	Ni	2.4575	7.0942	4.0131	0.6667	0.8333	0.1909
45	Ni	8.6013	0.7094	4.0131	0.9167	0.0833	0.1909
46	Ni	7.3725	2.8377	4.0131	0.9167	0.3333	0.1909
47	Ni	6.1438	4.9659	4.0131	0.9167	0.5833	0.1909
48	Ni	4.9150	7.0942	4.0131	0.9167	0.8333	0.1909
49	Ni	0.0000	0.0000	6.0198	0.0000	0.0000	0.2864
50	Ni	-1.2288	2.1283	6.0198	0.0000	0.2500	0.2864
51	Ni	-2.4575	4.2565	6.0198	-0.0000	0.5000	0.2864
52	Ni	-3.6862	6.3848	6.0198	-0.0000	0.7500	0.2864
53	Ni	2.4575	0.0000	6.0198	0.2500	0.0000	0.2864
54	Ni	1.2288	2.1283	6.0198	0.2500	0.2500	0.2864
55	Ni	-0.0000	4.2565	6.0198	0.2500	0.5000	0.2864
56	Ni	-1.2288	6.3848	6.0198	0.2500	0.7500	0.2864
57	Ni	4.9150	0.0000	6.0198	0.5000	0.0000	0.2864
58	Ni	3.6862	2.1283	6.0198	0.5000	0.2500	0.2864
59	Ni	2.4575	4.2565	6.0198	0.5000	0.5000	0.2864
60	Ni	1.2288	6.3848	6.0198	0.5000	0.7500	0.2864
61	Ni	7.3725	0.0000	6.0198	0.7500	0.0000	0.2864
62	Ni	6.1438	2.1283	6.0198	0.7500	0.2500	0.2864
63	Ni	4.9150	4.2565	6.0198	0.7500	0.5000	0.2864
64	Ni	3.6862	6.3848	6.0198	0.7500	0.7500	0.2864

/db/jmorales/CoNi-alloy/Slabs/Islanding/CoNi(111)/CoNi(111)-4-Ni

a =			9.831199646				
b =			9.831199646075708				
c =			21.0202999115				
alpha =			90.0				
beta =			90.0				
gamma =			120.0				
	Atom	X	Y	Z	X	Y	Z
1	Ni	0.0000	0.0000	0.0000	0.0000	0.0000	0.0000
2	Ni	-1.2289	2.1285	0.0000	0.0000	0.2500	0.0000
3	Ni	-2.4578	4.2570	0.0000	0.0000	0.5000	0.0000
4	Ni	-3.6867	6.3856	0.0000	0.0000	0.7500	0.0000
5	Ni	4.9156	0.0000	0.0000	0.5000	0.0000	0.0000
6	Ni	3.6867	2.1285	0.0000	0.5000	0.2500	0.0000
7	Ni	2.4578	4.2570	0.0000	0.5000	0.5000	0.0000
8	Ni	1.2289	6.3856	0.0000	0.5000	0.7500	0.0000
9	Ni	2.4578	1.4190	2.0068	0.3333	0.1667	0.0955
10	Ni	1.2289	3.5475	2.0068	0.3333	0.4167	0.0955
11	Ni	0.0000	5.6760	2.0068	0.3333	0.6667	0.0955
12	Ni	-1.2289	7.8045	2.0068	0.3333	0.9167	0.0955
13	Ni	7.3734	1.4190	2.0068	0.8333	0.1667	0.0955
14	Ni	6.1445	3.5475	2.0068	0.8333	0.4167	0.0955
15	Ni	4.9156	5.6760	2.0068	0.8333	0.6667	0.0955
16	Ni	3.6867	7.8045	2.0068	0.8333	0.9167	0.0955
17	Ni	0.0316	2.8566	3.9993	0.1710	0.3355	0.1903
18	Ni	1.2537	0.7295	4.0033	0.1704	0.0857	0.1904
19	Ni	-2.4218	7.1155	4.0210	0.1715	0.8357	0.1913
20	Ni	-1.1939	4.9829	4.0113	0.1712	0.5853	0.1908
21	Ni	4.9470	2.8567	3.9998	0.6710	0.3355	0.1903
22	Ni	6.1700	0.7298	4.0032	0.6705	0.0857	0.1904
23	Ni	2.4935	7.1154	4.0211	0.6715	0.8357	0.1913
24	Ni	3.7213	4.9823	4.0109	0.6711	0.5852	0.1908
25	Ni	-1.1843	2.1528	6.0302	0.0060	0.2529	0.2869
26	Ni	-2.4068	4.2934	6.0347	0.0073	0.5043	0.2871
27	Ni	3.7310	2.1523	6.0306	0.5059	0.2528	0.2869
28	Ni	2.5092	4.2942	6.0336	0.5074	0.5044	0.2870
29	Ni	1.2823	2.1491	6.0472	0.2566	0.2524	0.2877
30	Ni	0.0514	4.3018	6.0526	0.2579	0.5053	0.2879
31	Ni	4.9671	4.3014	6.0524	0.7578	0.5052	0.2879
32	Ni	6.1985	2.1485	6.0470	0.7567	0.2523	0.2877
33	Co	1.2289	2.1285	0.0000	0.2500	0.2500	0.0000
34	Co	2.4578	0.0000	0.0000	0.2500	0.0000	0.0000
35	Co	-1.2289	6.3856	0.0000	0.2500	0.7500	0.0000
36	Co	0.0000	4.2570	0.0000	0.2500	0.5000	0.0000
37	Co	6.1445	2.1285	0.0000	0.7500	0.2500	0.0000
38	Co	7.3734	0.0000	0.0000	0.7500	0.0000	0.0000
39	Co	3.6867	6.3856	0.0000	0.7500	0.7500	0.0000
40	Co	4.9156	4.2570	0.0000	0.7500	0.5000	0.0000
41	Co	-1.2289	3.5475	2.0068	0.0833	0.4167	0.0955

42	Co	0.0000	1.4190	2.0068	0.0833	0.1667	0.0955
43	Co	-3.6867	7.8045	2.0068	0.0833	0.9167	0.0955
44	Co	-2.4578	5.6760	2.0068	0.0833	0.6667	0.0955
45	Co	3.6867	3.5475	2.0068	0.5833	0.4167	0.0955
46	Co	4.9156	1.4190	2.0068	0.5833	0.1667	0.0955
47	Co	1.2289	7.8045	2.0068	0.5833	0.9167	0.0955
48	Co	2.4578	5.6760	2.0068	0.5833	0.6667	0.0955
49	Co	3.7114	0.7243	4.0525	0.4201	0.0851	0.1928
50	Co	2.4922	2.8585	4.0416	0.4214	0.3357	0.1923
51	Co	1.2653	4.9904	4.0533	0.4218	0.5861	0.1928
52	Co	0.0331	7.1162	4.0585	0.4213	0.8358	0.1931
53	Co	8.6274	0.7236	4.0534	0.9200	0.0850	0.1928
54	Co	7.4084	2.8595	4.0421	0.9215	0.3359	0.1923
55	Co	6.1808	4.9910	4.0525	0.9218	0.5862	0.1928
56	Co	4.9487	7.1160	4.0575	0.9213	0.8358	0.1930
57	Co	2.5134	0.0208	6.0100	0.2569	0.0024	0.2859
58	Co	-1.1740	6.4284	6.0244	0.2581	0.7550	0.2866
59	Co	7.4288	0.0212	6.0113	0.7569	0.0025	0.2860
60	Co	3.7422	6.4292	6.0233	0.7582	0.7551	0.2865
61	Co	0.0322	0.0233	5.9932	0.0046	0.0027	0.2851
62	Co	1.2737	6.3995	6.0014	0.5054	0.7516	0.2855
63	Co	-3.6420	6.3991	6.0012	0.0053	0.7516	0.2855
64	Co	4.9476	0.0234	5.9918	0.5046	0.0028	0.2850

/db/jmorales/CoNi-alloy/Slabs/Segregation/CoNi(001)/CoNi(001)-1-Co

a =	6.9516692132
b =	6.9516692132
c =	21.9516692133
alpha =	90.0
beta =	90.0
gamma =	90.0

	Atom	X	Y	Z	X	Y	Z
1	Ni	0.0000	0.0000	0.0000	0.0000	0.0000	0.0000
2	Ni	0.0000	3.4758	0.0000	0.0000	0.5000	0.0000
3	Ni	3.4758	0.0000	0.0000	0.5000	0.0000	0.0000
4	Ni	3.4758	3.4758	0.0000	0.5000	0.5000	0.0000
5	Ni	0.0000	1.7379	1.7379	0.0000	0.2500	0.0792
6	Ni	0.0000	5.2138	1.7379	0.0000	0.7500	0.0792
7	Ni	3.4758	1.7379	1.7379	0.5000	0.2500	0.0792
8	Ni	3.4758	5.2138	1.7379	0.5000	0.7500	0.0792
9	Ni	-0.0027	-0.0029	3.5091	-0.0004	-0.0004	0.1599
10	Ni	-0.0027	3.4787	3.5091	-0.0004	0.5004	0.1599
11	Ni	3.4746	-0.0087	3.5051	0.4998	-0.0013	0.1597
12	Ni	3.4746	3.4845	3.5051	0.4998	0.5013	0.1597
13	Ni	0.0148	1.7379	5.2115	0.0021	0.2500	0.2374
14	Ni	0.0001	5.2138	5.2186	0.0000	0.7500	0.2377
15	Ni	1.7415	1.7379	3.5260	0.2505	0.2500	0.1606
16	Ni	3.4736	5.2138	5.2156	0.4997	0.7500	0.2376
17	Co	1.7379	1.7379	0.0000	0.2500	0.2500	0.0000
18	Co	1.7379	5.2138	0.0000	0.2500	0.7500	0.0000
19	Co	5.2138	1.7379	0.0000	0.7500	0.2500	0.0000
20	Co	5.2138	5.2138	0.0000	0.7500	0.7500	0.0000
21	Co	1.7379	0.0000	1.7379	0.2500	0.0000	0.0792
22	Co	1.7379	3.4758	1.7379	0.2500	0.5000	0.0792
23	Co	5.2138	0.0000	1.7379	0.7500	0.0000	0.0792
24	Co	5.2138	3.4758	1.7379	0.7500	0.5000	0.0792
25	Co	3.4637	1.7379	5.2011	0.4982	0.2500	0.2369
26	Co	1.7346	5.2138	3.5494	0.2495	0.7500	0.1617
27	Co	5.2100	1.7379	3.5518	0.7495	0.2500	0.1618
28	Co	5.2138	5.2138	3.5609	0.7500	0.7500	0.1622
29	Co	1.7519	-0.0077	5.2349	0.2520	-0.0011	0.2385
30	Co	1.7519	3.4836	5.2349	0.2520	0.5011	0.2385
31	Co	5.1960	0.0274	5.2292	0.7474	0.0039	0.2382
32	Co	5.1960	3.4484	5.2292	0.7474	0.4961	0.2382

/db/jmorales/CoNi-alloy/Slabs/Segregation/CoNi(001)/CoNi(001)-1-Co-CO

a =		6.9516692132					
b =		6.9516692132					
c =		21.9516692133					
alpha =		90.0					
beta =		90.0					
gamma =		90.0					
	Atom	X	Y	Z	X	Y	Z
1	Ni	0.0000	0.0000	0.0000	0.0000	0.0000	0.0000
2	Ni	0.0000	3.4758	0.0000	0.0000	0.5000	0.0000
3	Ni	3.4758	0.0000	0.0000	0.5000	0.0000	0.0000
4	Ni	3.4758	3.4758	0.0000	0.5000	0.5000	0.0000
5	Ni	0.0000	1.7379	1.7379	0.0000	0.2500	0.0792
6	Ni	0.0000	5.2138	1.7379	0.0000	0.7500	0.0792
7	Ni	3.4758	1.7379	1.7379	0.5000	0.2500	0.0792
8	Ni	3.4758	5.2138	1.7379	0.5000	0.7500	0.0792
9	Ni	6.9480	6.9482	3.5076	0.9995	0.9995	0.1598
10	Ni	6.9496	3.4781	3.5041	0.9997	0.5003	0.1596
11	Ni	3.4768	6.9384	3.5221	0.5001	0.9981	0.1604
12	Ni	3.4756	3.4851	3.4645	0.5000	0.5013	0.1578
13	Ni	0.0193	1.7381	5.2049	0.0028	0.2500	0.2371
14	Ni	-0.0031	5.2143	5.2190	-0.0005	0.7501	0.2378
15	Ni	1.7409	1.7371	3.5228	0.2504	0.2499	0.1605
16	Ni	3.4823	5.1928	5.2600	0.5009	0.7470	0.2396
17	Co	1.7379	1.7379	0.0000	0.2500	0.2500	0.0000
18	Co	1.7379	5.2138	0.0000	0.2500	0.7500	0.0000
19	Co	5.2138	1.7379	0.0000	0.7500	0.2500	0.0000
20	Co	5.2138	5.2138	0.0000	0.7500	0.7500	0.0000
21	Co	1.7379	0.0000	1.7379	0.2500	0.0000	0.0792
22	Co	1.7379	3.4758	1.7379	0.2500	0.5000	0.0792
23	Co	5.2138	0.0000	1.7379	0.7500	0.0000	0.0792
24	Co	5.2138	3.4758	1.7379	0.7500	0.5000	0.0792
25	Co	3.4512	1.7531	5.2132	0.4965	0.2522	0.2375
26	Co	1.7353	5.2131	3.5561	0.2496	0.7499	0.1620
27	Co	5.2093	1.7380	3.5570	0.7494	0.2500	0.1620
28	Co	5.2160	5.2141	3.5641	0.7503	0.7500	0.1624
29	Co	1.7436	6.9411	5.2260	0.2508	0.9985	0.2381
30	Co	1.7602	3.4785	5.2648	0.2532	0.5004	0.2398
31	Co	5.2040	0.0323	5.2246	0.7486	0.0046	0.2380
32	Co	5.1903	3.4438	5.2611	0.7466	0.4954	0.2397
33	O	3.4735	3.4690	7.6009	0.4997	0.4990	0.3463
34	C	3.4788	3.4942	6.3857	0.5004	0.5026	0.2909

/db/jmorales/CoNi-alloy/Slabs/Islanding/CoNi(111)/CoNi(111)-3-Ni

a = 9.831199646
b = 9.831199646075708
c = 21.0202999115
alpha = 90.0
beta = 90.0
gamma = 120.0

	Atom	X	Y	Z	X	Y	Z
1	Ni	0.0000	0.0000	0.0000	0.0000	0.0000	0.0000
2	Ni	-1.2289	2.1285	0.0000	0.0000	0.2500	0.0000
3	Ni	-2.4578	4.2570	0.0000	0.0000	0.5000	0.0000
4	Ni	-3.6867	6.3856	0.0000	0.0000	0.7500	0.0000
5	Ni	4.9156	0.0000	0.0000	0.5000	0.0000	0.0000
6	Ni	3.6867	2.1285	0.0000	0.5000	0.2500	0.0000
7	Ni	2.4578	4.2570	0.0000	0.5000	0.5000	0.0000
8	Ni	1.2289	6.3856	0.0000	0.5000	0.7500	0.0000
9	Ni	2.4578	1.4190	2.0068	0.3333	0.1667	0.0955
10	Ni	1.2289	3.5475	2.0068	0.3333	0.4167	0.0955
11	Ni	0.0000	5.6760	2.0068	0.3333	0.6667	0.0955
12	Ni	-1.2289	7.8045	2.0068	0.3333	0.9167	0.0955
13	Ni	7.3734	1.4190	2.0068	0.8333	0.1667	0.0955
14	Ni	6.1445	3.5475	2.0068	0.8333	0.4167	0.0955
15	Ni	4.9156	5.6760	2.0068	0.8333	0.6667	0.0955
16	Ni	3.6867	7.8045	2.0068	0.8333	0.9167	0.0955
17	Ni	0.0299	2.8567	3.9972	0.1708	0.3355	0.1902
18	Ni	1.2552	0.7291	4.0070	0.1705	0.0856	0.1906
19	Ni	-2.4255	7.1135	4.0164	0.1710	0.8355	0.1911
20	Ni	-1.1952	4.9818	4.0104	0.1710	0.5851	0.1908
21	Ni	4.9477	2.8550	4.0077	0.6709	0.3353	0.1907
22	Ni	6.1704	0.7288	4.0113	0.6704	0.0856	0.1908
23	Ni	2.4944	7.1168	4.0148	0.6717	0.8359	0.1910
24	Ni	3.7208	4.9831	4.0086	0.6711	0.5853	0.1907
25	Ni	-1.1789	2.1524	6.0341	0.0065	0.2528	0.2871
26	Ni	-2.4121	4.2940	6.0354	0.0068	0.5043	0.2871
27	Ni	4.9591	0.0321	6.0270	0.5063	0.0038	0.2867
28	Ni	3.7357	2.1502	6.0281	0.5063	0.2525	0.2868
29	Ni	2.5165	4.2922	6.0306	0.5080	0.5041	0.2869
30	Ni	1.2828	2.1516	6.0501	0.2568	0.2527	0.2878
31	Ni	0.0516	4.3022	6.0502	0.2579	0.5053	0.2878
32	Ni	4.9744	4.2977	6.0544	0.7584	0.5048	0.2880
33	Co	1.2289	2.1285	0.0000	0.2500	0.2500	0.0000
34	Co	2.4578	0.0000	0.0000	0.2500	0.0000	0.0000
35	Co	-1.2289	6.3856	0.0000	0.2500	0.7500	0.0000
36	Co	0.0000	4.2570	0.0000	0.2500	0.5000	0.0000
37	Co	6.1445	2.1285	0.0000	0.7500	0.2500	0.0000
38	Co	7.3734	0.0000	0.0000	0.7500	0.0000	0.0000
39	Co	3.6867	6.3856	0.0000	0.7500	0.7500	0.0000
40	Co	4.9156	4.2570	0.0000	0.7500	0.5000	0.0000
41	Co	-1.2289	3.5475	2.0068	0.0833	0.4167	0.0955

42	Co	0.0000	1.4190	2.0068	0.0833	0.1667	0.0955
43	Co	-3.6867	7.8045	2.0068	0.0833	0.9167	0.0955
44	Co	-2.4578	5.6760	2.0068	0.0833	0.6667	0.0955
45	Co	3.6867	3.5475	2.0068	0.5833	0.4167	0.0955
46	Co	4.9156	1.4190	2.0068	0.5833	0.1667	0.0955
47	Co	1.2289	7.8045	2.0068	0.5833	0.9167	0.0955
48	Co	2.4578	5.6760	2.0068	0.5833	0.6667	0.0955
49	Co	3.7078	0.7268	4.0515	0.4198	0.0854	0.1927
50	Co	2.4933	2.8606	4.0413	0.4216	0.3360	0.1923
51	Co	1.2657	4.9913	4.0570	0.4219	0.5862	0.1930
52	Co	0.0333	7.1088	4.0551	0.4209	0.8350	0.1929
53	Co	8.6302	0.7252	4.0562	0.9204	0.0852	0.1930
54	Co	7.4004	2.8533	4.0621	0.9203	0.3351	0.1932
55	Co	6.1794	4.9920	4.0494	0.9217	0.5863	0.1926
56	Co	4.9468	7.1159	4.0566	0.9211	0.8358	0.1930
57	Co	2.5132	0.0199	6.0092	0.2568	0.0023	0.2859
58	Co	-1.1775	6.4323	6.0178	0.2580	0.7555	0.2863
59	Co	6.2176	2.1508	6.0183	0.7587	0.2526	0.2863
60	Co	7.4388	0.0290	6.0152	0.7584	0.0034	0.2862
61	Co	3.7491	6.4286	6.0222	0.7589	0.7551	0.2865
62	Co	0.0316	0.0257	5.9949	0.0047	0.0030	0.2852
63	Co	1.2695	6.4143	5.9960	0.5058	0.7534	0.2852
64	Co	-3.6465	6.3966	5.9966	0.0047	0.7513	0.2853

/db/jmorales/CoNi-alloy/Slabs/Islanding/CoNi(001)/CoNi(001)-2-Ni

a =	6.9516692132
b =	6.9516692132
c =	21.9516692133
alpha =	90.0
beta =	90.0
gamma =	90.0

	Atom	X	Y	Z	X	Y	Z
1	Ni	0.0000	0.0000	0.0000	0.0000	0.0000	0.0000
2	Ni	0.0000	3.4758	0.0000	0.0000	0.5000	0.0000
3	Ni	3.4758	0.0000	0.0000	0.5000	0.0000	0.0000
4	Ni	3.4758	3.4758	0.0000	0.5000	0.5000	0.0000
5	Ni	0.0000	1.7379	1.7379	0.0000	0.2500	0.0792
6	Ni	0.0000	5.2138	1.7379	0.0000	0.7500	0.0792
7	Ni	3.4758	1.7379	1.7379	0.5000	0.2500	0.0792
8	Ni	3.4758	5.2138	1.7379	0.5000	0.7500	0.0792
9	Ni	6.9504	6.9505	3.4799	0.9998	0.9998	0.1585
10	Ni	0.0006	3.4758	3.5022	0.0001	0.5000	0.1595
11	Ni	3.4767	-0.0004	3.5032	0.5001	-0.0001	0.1596
12	Ni	3.4753	3.4755	3.5032	0.4999	0.5000	0.1596
13	Ni	3.4766	1.7200	5.2184	0.5001	0.2474	0.2377
14	Ni	3.4755	5.2314	5.2201	0.4999	0.7525	0.2378
15	Ni	1.7206	3.4743	5.2189	0.2475	0.4998	0.2377
16	Ni	5.2312	3.4752	5.2201	0.7525	0.4999	0.2378
17	Co	1.7379	1.7379	0.0000	0.2500	0.2500	0.0000
18	Co	1.7379	5.2138	0.0000	0.2500	0.7500	0.0000
19	Co	5.2138	1.7379	0.0000	0.7500	0.2500	0.0000
20	Co	5.2138	5.2138	0.0000	0.7500	0.7500	0.0000
21	Co	1.7379	0.0000	1.7379	0.2500	0.0000	0.0792
22	Co	1.7379	3.4758	1.7379	0.2500	0.5000	0.0792
23	Co	5.2138	0.0000	1.7379	0.7500	0.0000	0.0792
24	Co	5.2138	3.4758	1.7379	0.7500	0.5000	0.0792
25	Co	1.7308	1.7264	3.5446	0.2490	0.2483	0.1615
26	Co	1.7318	5.2241	3.5465	0.2491	0.7515	0.1616
27	Co	5.2202	1.7288	3.5455	0.7509	0.2487	0.1615
28	Co	5.2194	5.2214	3.5482	0.7508	0.7511	0.1616
29	Co	1.7068	0.0013	5.2044	0.2455	0.0002	0.2371
30	Co	5.2451	-0.0000	5.2048	0.7545	-0.0000	0.2371
31	Co	-0.0008	1.7087	5.1931	-0.0001	0.2458	0.2366
32	Co	0.0014	5.2422	5.1949	0.0002	0.7541	0.2367

/db/jmorales/CoNi-alloy/Slabs/Segregation/CoNi(111)/CoNi(111)-3-Ni

a =		9.831199646
b =		9.831199646075708
c =		21.0202999115
alpha =		90.0
beta =		90.0
gamma =		120.0

	Atom	X	Y	Z	X	Y	Z
1	Ni	0.0000	0.0000	0.0000	0.0000	0.0000	0.0000
2	Ni	-1.2289	2.1285	0.0000	0.0000	0.2500	0.0000
3	Ni	-2.4578	4.2570	0.0000	0.0000	0.5000	0.0000
4	Ni	-3.6867	6.3856	0.0000	0.0000	0.7500	0.0000
5	Ni	4.9156	0.0000	0.0000	0.5000	0.0000	0.0000
6	Ni	3.6867	2.1285	0.0000	0.5000	0.2500	0.0000
7	Ni	2.4578	4.2570	0.0000	0.5000	0.5000	0.0000
8	Ni	1.2289	6.3856	0.0000	0.5000	0.7500	0.0000
9	Ni	2.4578	1.4190	2.0068	0.3333	0.1667	0.0955
10	Ni	1.2289	3.5475	2.0068	0.3333	0.4167	0.0955
11	Ni	0.0000	5.6760	2.0068	0.3333	0.6667	0.0955
12	Ni	-1.2289	7.8045	2.0068	0.3333	0.9167	0.0955
13	Ni	7.3734	1.4190	2.0068	0.8333	0.1667	0.0955
14	Ni	6.1445	3.5475	2.0068	0.8333	0.4167	0.0955
15	Ni	4.9156	5.6760	2.0068	0.8333	0.6667	0.0955
16	Ni	3.6867	7.8045	2.0068	0.8333	0.9167	0.0955
17	Ni	-1.1987	4.9815	4.0020	0.1706	0.5851	0.1904
18	Ni	4.9464	2.8570	4.0111	0.6709	0.3356	0.1908
19	Ni	6.1761	0.7263	4.0082	0.6709	0.0853	0.1907
20	Ni	2.4858	7.1134	4.0109	0.6706	0.8355	0.1908
21	Ni	3.7174	4.9848	4.0109	0.6709	0.5855	0.1908
22	Ni	0.0418	0.0226	6.0408	0.0056	0.0027	0.2874
23	Ni	-1.1866	2.1568	6.0319	0.0060	0.2533	0.2870
24	Ni	-2.4198	4.2833	6.0314	0.0054	0.5031	0.2869
25	Ni	-3.6466	6.4083	6.0372	0.0054	0.7527	0.2872
26	Ni	4.9629	0.0308	6.0200	0.5066	0.0036	0.2864
27	Ni	3.7412	2.1611	6.0147	0.5075	0.2538	0.2861
28	Ni	2.5103	4.2903	6.0137	0.5073	0.5039	0.2861
29	Ni	1.2822	6.4126	6.0178	0.5070	0.7532	0.2863
30	Ni	1.2736	2.1580	6.0243	0.2563	0.2535	0.2866
31	Ni	2.5131	0.0241	6.0301	0.2570	0.0028	0.2869
32	Ni	0.0482	4.2954	6.0304	0.2572	0.5045	0.2869
33	Co	1.2289	2.1285	0.0000	0.2500	0.2500	0.0000
34	Co	2.4578	0.0000	0.0000	0.2500	0.0000	0.0000
35	Co	-1.2289	6.3856	0.0000	0.2500	0.7500	0.0000
36	Co	0.0000	4.2570	0.0000	0.2500	0.5000	0.0000
37	Co	6.1445	2.1285	0.0000	0.7500	0.2500	0.0000
38	Co	7.3734	0.0000	0.0000	0.7500	0.0000	0.0000
39	Co	3.6867	6.3856	0.0000	0.7500	0.7500	0.0000
40	Co	4.9156	4.2570	0.0000	0.7500	0.5000	0.0000
41	Co	-1.2289	3.5475	2.0068	0.0833	0.4167	0.0955

42	Co	0.0000	1.4190	2.0068	0.0833	0.1667	0.0955
43	Co	-3.6867	7.8045	2.0068	0.0833	0.9167	0.0955
44	Co	-2.4578	5.6760	2.0068	0.0833	0.6667	0.0955
45	Co	3.6867	3.5475	2.0068	0.5833	0.4167	0.0955
46	Co	4.9156	1.4190	2.0068	0.5833	0.1667	0.0955
47	Co	1.2289	7.8045	2.0068	0.5833	0.9167	0.0955
48	Co	2.4578	5.6760	2.0068	0.5833	0.6667	0.0955
49	Co	3.7089	0.7276	4.0315	0.4200	0.0855	0.1918
50	Co	2.4841	2.8491	4.0332	0.4200	0.3346	0.1919
51	Co	1.2592	4.9789	4.0344	0.4205	0.5848	0.1919
52	Co	0.0207	7.1098	4.0465	0.4196	0.8351	0.1925
53	Co	8.6415	0.7303	4.0596	0.9219	0.0858	0.1931
54	Co	7.4122	2.8585	4.0489	0.9218	0.3357	0.1926
55	Co	6.1797	4.9918	4.0488	0.9217	0.5863	0.1926
56	Co	4.9543	7.1205	4.0526	0.9221	0.8363	0.1928
57	Co	-1.1800	6.4266	5.9885	0.2574	0.7548	0.2849
58	Co	6.2068	2.1662	6.0060	0.7586	0.2544	0.2857
59	Co	7.4316	0.0332	6.0078	0.7579	0.0039	0.2858
60	Co	3.7475	6.4228	6.0077	0.7584	0.7544	0.2858
61	Co	4.9813	4.2943	6.0073	0.7589	0.5044	0.2858
62	Co	1.2517	0.7201	4.0068	0.1696	0.0846	0.1906
63	Co	-2.4277	7.1086	4.0166	0.1705	0.8349	0.1911
64	Co	0.0226	2.8506	3.9955	0.1697	0.3348	0.1901

/db/jmorales/CoNi-alloy/Slabs/Segregation/CoNi(001)/CoNi(001)-3-Co-CO

a =	6.9516692132
b =	6.9516692132
c =	21.9516692133
alpha =	90.0
beta =	90.0
gamma =	90.0

	Atom	X	Y	Z	X	Y	Z
1	Ni	0.0000	0.0000	0.0000	0.0000	0.0000	0.0000
2	Ni	0.0000	3.4758	0.0000	0.0000	0.5000	0.0000
3	Ni	3.4758	0.0000	0.0000	0.5000	0.0000	0.0000
4	Ni	3.4758	3.4758	0.0000	0.5000	0.5000	0.0000
5	Ni	0.0000	1.7379	1.7379	0.0000	0.2500	0.0792
6	Ni	0.0000	5.2138	1.7379	0.0000	0.7500	0.0792
7	Ni	3.4758	1.7379	1.7379	0.5000	0.2500	0.0792
8	Ni	3.4758	5.2138	1.7379	0.5000	0.7500	0.0792
9	Ni	6.9506	-0.0007	3.5210	0.9998	-0.0001	0.1604
10	Ni	6.9514	3.4736	3.5249	1.0000	0.4997	0.1606
11	Ni	3.4731	-0.0008	3.5270	0.4996	-0.0001	0.1607
12	Ni	3.4741	3.4742	3.4761	0.4997	0.4998	0.1584
13	Ni	0.0080	5.2177	5.2407	0.0012	0.7506	0.2387
14	Ni	1.7323	1.7384	3.5339	0.2492	0.2501	0.1610
15	Ni	1.7372	5.2147	3.5480	0.2499	0.7501	0.1616
16	Ni	5.2166	1.7368	3.5462	0.7504	0.2498	0.1615
17	Co	1.7379	1.7379	0.0000	0.2500	0.2500	0.0000
18	Co	1.7379	5.2138	0.0000	0.2500	0.7500	0.0000
19	Co	5.2138	1.7379	0.0000	0.7500	0.2500	0.0000
20	Co	5.2138	5.2138	0.0000	0.7500	0.7500	0.0000
21	Co	1.7379	0.0000	1.7379	0.2500	0.0000	0.0792
22	Co	1.7379	3.4758	1.7379	0.2500	0.5000	0.0792
23	Co	5.2138	0.0000	1.7379	0.7500	0.0000	0.0792
24	Co	5.2138	3.4758	1.7379	0.7500	0.5000	0.0792
25	Co	3.4731	1.7587	5.2467	0.4996	0.2530	0.2390
26	Co	5.2136	5.2121	3.5575	0.7500	0.7498	0.1621
27	Co	1.7585	0.0314	5.2512	0.2530	0.0045	0.2392
28	Co	1.7725	3.4477	5.2842	0.2550	0.4960	0.2407
29	Co	5.1905	-0.0120	5.2427	0.7467	-0.0017	0.2388
30	Co	5.1842	3.4772	5.2784	0.7457	0.5002	0.2405
31	Co	3.4690	5.1925	5.2550	0.4990	0.7469	0.2394
32	Co	0.0026	1.7323	5.2347	0.0004	0.2492	0.2385
33	O	3.4833	3.4791	7.6429	0.5011	0.5005	0.3482
34	C	3.4775	3.4810	6.4286	0.5002	0.5007	0.2929

/db/jmorales/CoNi-alloy/Slabs/CoNi(111)-expanded

a =	9.963000298
b =	9.963000298017379
c =	21.101100922
alpha =	90.0
beta =	90.0
gamma =	120.0

	Atom	X	Y	Z	X	Y	Z
1	Ni	0.0000	0.0000	0.0000	0.0000	0.0000	0.0000
2	Ni	-1.2454	2.1570	0.0000	-0.0000	0.2500	0.0000
3	Ni	-2.4908	4.3141	0.0000	0.0000	0.5000	0.0000
4	Ni	-3.7361	6.4712	0.0000	-0.0000	0.7500	0.0000
5	Ni	4.9815	0.0000	0.0000	0.5000	0.0000	0.0000
6	Ni	3.7361	2.1570	0.0000	0.5000	0.2500	0.0000
7	Ni	2.4908	4.3141	0.0000	0.5000	0.5000	0.0000
8	Ni	1.2454	6.4712	0.0000	0.5000	0.7500	0.0000
9	Ni	2.4908	1.4380	2.0337	0.3333	0.1667	0.0964
10	Ni	1.2454	3.5951	2.0337	0.3333	0.4167	0.0964
11	Ni	0.0000	5.7521	2.0337	0.3333	0.6667	0.0964
12	Ni	-1.2454	7.9092	2.0337	0.3333	0.9167	0.0964
13	Ni	7.4723	1.4380	2.0337	0.8333	0.1667	0.0964
14	Ni	6.2269	3.5951	2.0337	0.8333	0.4167	0.0964
15	Ni	4.9815	5.7521	2.0337	0.8333	0.6667	0.0964
16	Ni	3.7362	7.9092	2.0337	0.8333	0.9167	0.0964
17	Ni	-0.0000	2.8761	4.0674	0.1667	0.3333	0.1928
18	Ni	1.2454	0.7190	4.0674	0.1667	0.0833	0.1928
19	Ni	-2.4908	7.1902	4.0674	0.1667	0.8333	0.1928
20	Ni	-1.2454	5.0331	4.0674	0.1667	0.5833	0.1928
21	Ni	4.9815	2.8761	4.0674	0.6667	0.3333	0.1928
22	Ni	6.2268	0.7190	4.0674	0.6667	0.0833	0.1928
23	Ni	2.4907	7.1902	4.0674	0.6667	0.8333	0.1928
24	Ni	3.7361	5.0331	4.0674	0.6667	0.5833	0.1928
25	Ni	0.0000	0.0000	6.1012	0.0000	0.0000	0.2891
26	Ni	-1.2454	2.1570	6.1012	-0.0000	0.2500	0.2891
27	Ni	-2.4908	4.3141	6.1012	0.0000	0.5000	0.2891
28	Ni	-3.7361	6.4712	6.1012	-0.0000	0.7500	0.2891
29	Ni	4.9815	0.0000	6.1012	0.5000	0.0000	0.2891
30	Ni	3.7361	2.1570	6.1012	0.5000	0.2500	0.2891
31	Ni	2.4908	4.3141	6.1012	0.5000	0.5000	0.2891
32	Ni	1.2454	6.4712	6.1012	0.5000	0.7500	0.2891
33	Co	1.2454	2.1570	0.0000	0.2500	0.2500	0.0000
34	Co	2.4908	0.0000	0.0000	0.2500	0.0000	0.0000
35	Co	-1.2454	6.4712	0.0000	0.2500	0.7500	0.0000
36	Co	0.0000	4.3141	0.0000	0.2500	0.5000	0.0000
37	Co	6.2269	2.1570	0.0000	0.7500	0.2500	0.0000
38	Co	7.4722	0.0000	0.0000	0.7500	0.0000	0.0000
39	Co	3.7361	6.4712	0.0000	0.7500	0.7500	0.0000
40	Co	4.9815	4.3141	0.0000	0.7500	0.5000	0.0000
41	Co	-1.2454	3.5951	2.0337	0.0833	0.4167	0.0964

42	Co	0.0000	1.4380	2.0337	0.0833	0.1667	0.0964
43	Co	-3.7361	7.9092	2.0337	0.0833	0.9167	0.0964
44	Co	-2.4907	5.7521	2.0337	0.0833	0.6667	0.0964
45	Co	3.7362	3.5951	2.0337	0.5833	0.4167	0.0964
46	Co	4.9815	1.4380	2.0337	0.5833	0.1667	0.0964
47	Co	1.2454	7.9092	2.0337	0.5833	0.9167	0.0964
48	Co	2.4908	5.7521	2.0337	0.5833	0.6667	0.0964
49	Co	3.7361	0.7190	4.0674	0.4167	0.0833	0.1928
50	Co	2.4907	2.8761	4.0674	0.4167	0.3333	0.1928
51	Co	1.2454	5.0331	4.0674	0.4167	0.5833	0.1928
52	Co	-0.0000	7.1902	4.0674	0.4167	0.8333	0.1928
53	Co	8.7176	0.7190	4.0674	0.9167	0.0833	0.1928
54	Co	7.4722	2.8761	4.0674	0.9167	0.3333	0.1928
55	Co	6.2268	5.0331	4.0674	0.9167	0.5833	0.1928
56	Co	4.9815	7.1902	4.0674	0.9167	0.8333	0.1928
57	Co	1.2454	2.1570	6.1012	0.2500	0.2500	0.2891
58	Co	2.4908	0.0000	6.1012	0.2500	0.0000	0.2891
59	Co	-1.2454	6.4712	6.1012	0.2500	0.7500	0.2891
60	Co	0.0000	4.3141	6.1012	0.2500	0.5000	0.2891
61	Co	6.2269	2.1570	6.1012	0.7500	0.2500	0.2891
62	Co	7.4722	0.0000	6.1012	0.7500	0.0000	0.2891
63	Co	3.7361	6.4712	6.1012	0.7500	0.7500	0.2891
64	Co	4.9815	4.3141	6.1012	0.7500	0.5000	0.2891

/db/jmorales/CoNi-alloy/Slabs/Segregation/CoNi(111)/CoNi(111)-4-Co-CO

a = 9.831199646
b = 9.831199646075708
c = 21.0202999115
alpha = 90.0
beta = 90.0
gamma = 120.0

	Atom	X	Y	Z	X	Y	Z
1	Ni	0.0000	0.0000	0.0000	0.0000	0.0000	0.0000
2	Ni	-1.2289	2.1285	0.0000	0.0000	0.2500	0.0000
3	Ni	-2.4578	4.2570	0.0000	0.0000	0.5000	0.0000
4	Ni	-3.6867	6.3856	0.0000	0.0000	0.7500	0.0000
5	Ni	4.9156	0.0000	0.0000	0.5000	0.0000	0.0000
6	Ni	3.6867	2.1285	0.0000	0.5000	0.2500	0.0000
7	Ni	2.4578	4.2570	0.0000	0.5000	0.5000	0.0000
8	Ni	1.2289	6.3856	0.0000	0.5000	0.7500	0.0000
9	Ni	2.4578	1.4190	2.0068	0.3333	0.1667	0.0955
10	Ni	1.2289	3.5475	2.0068	0.3333	0.4167	0.0955
11	Ni	0.0000	5.6760	2.0068	0.3333	0.6667	0.0955
12	Ni	-1.2289	7.8045	2.0068	0.3333	0.9167	0.0955
13	Ni	7.3734	1.4190	2.0068	0.8333	0.1667	0.0955
14	Ni	6.1445	3.5475	2.0068	0.8333	0.4167	0.0955
15	Ni	4.9156	5.6760	2.0068	0.8333	0.6667	0.0955
16	Ni	3.6867	7.8045	2.0068	0.8333	0.9167	0.0955
17	Ni	0.0202	2.8491	4.0211	0.1694	0.3346	0.1913
18	Ni	1.2478	0.7201	4.0201	0.1692	0.0846	0.1912
19	Ni	-2.4427	7.1035	4.0259	0.1687	0.8343	0.1915
20	Ni	-1.2146	4.9748	4.0257	0.1686	0.5843	0.1915
21	Ni	4.9433	2.8591	4.0266	0.6707	0.3358	0.1916
22	Ni	6.1729	0.7285	4.0209	0.6707	0.0856	0.1913
23	Ni	2.4897	7.1102	4.0226	0.6708	0.8351	0.1914
24	Ni	3.7174	4.9782	4.0274	0.6705	0.5847	0.1916
25	Ni	0.0318	0.0199	6.0413	0.0044	0.0023	0.2874
26	Ni	-1.1946	2.1448	6.0412	0.0044	0.2519	0.2874
27	Ni	-2.4243	4.2745	6.0464	0.0044	0.5021	0.2876
28	Ni	-3.6529	6.4055	6.0456	0.0046	0.7523	0.2876
29	Ni	3.7088	0.7275	4.0441	0.4200	0.0855	0.1924
30	Ni	1.2551	4.9772	4.0462	0.4200	0.5846	0.1925
31	Ni	0.0260	7.1093	4.0413	0.4201	0.8350	0.1923
32	Ni	2.4817	2.8510	4.0442	0.4199	0.3349	0.1924
33	Co	1.2289	2.1285	0.0000	0.2500	0.2500	0.0000
34	Co	2.4578	0.0000	0.0000	0.2500	0.0000	0.0000
35	Co	-1.2289	6.3856	0.0000	0.2500	0.7500	0.0000
36	Co	0.0000	4.2570	0.0000	0.2500	0.5000	0.0000
37	Co	6.1445	2.1285	0.0000	0.7500	0.2500	0.0000
38	Co	7.3734	0.0000	0.0000	0.7500	0.0000	0.0000
39	Co	3.6867	6.3856	0.0000	0.7500	0.7500	0.0000
40	Co	4.9156	4.2570	0.0000	0.7500	0.5000	0.0000
41	Co	-1.2289	3.5475	2.0068	0.0833	0.4167	0.0955

42	Co	0.0000	1.4190	2.0068	0.0833	0.1667	0.0955
43	Co	-3.6867	7.8045	2.0068	0.0833	0.9167	0.0955
44	Co	-2.4578	5.6760	2.0068	0.0833	0.6667	0.0955
45	Co	3.6867	3.5475	2.0068	0.5833	0.4167	0.0955
46	Co	4.9156	1.4190	2.0068	0.5833	0.1667	0.0955
47	Co	1.2289	7.8045	2.0068	0.5833	0.9167	0.0955
48	Co	2.4578	5.6760	2.0068	0.5833	0.6667	0.0955
49	Co	8.6269	0.7245	4.0609	0.9201	0.0851	0.1932
50	Co	7.3956	2.8492	4.0598	0.9196	0.3346	0.1931
51	Co	6.1667	4.9809	4.0661	0.9198	0.5850	0.1934
52	Co	4.9361	7.1077	4.0600	0.9195	0.8348	0.1931
53	Co	1.2708	2.1501	6.0783	0.2555	0.2525	0.2892
54	Co	2.5221	0.0032	6.0288	0.2567	0.0004	0.2868
55	Co	-1.1715	6.4159	6.0356	0.2576	0.7536	0.2871
56	Co	0.0360	4.3055	6.0305	0.2565	0.5057	0.2869
57	Co	6.1824	2.1446	6.0316	0.7548	0.2519	0.2869
58	Co	7.4106	0.0198	6.0394	0.7550	0.0023	0.2873
59	Co	3.7209	6.4075	6.0329	0.7548	0.7526	0.2870
60	Co	4.9721	4.2889	6.0249	0.7576	0.5037	0.2866
61	Co	3.7334	2.1490	6.0419	0.5059	0.2524	0.2874
62	Co	2.4989	4.2845	6.0460	0.5058	0.5032	0.2876
63	Co	1.2662	6.4073	6.0035	0.5051	0.7525	0.2856
64	Co	4.9537	0.0193	6.0023	0.5050	0.0023	0.2855
65	O	2.5221	2.8654	8.6113	0.4248	0.3366	0.4097
66	C	2.4894	2.8485	7.4151	0.4205	0.3346	0.3528

/db/jmorales/CoNi-alloy/Slabs/Segregation/Co-CoNi(001)/Co-CoNi(001)-3-Ni

a =	6.9516692132
b =	6.9516692132
c =	21.9516692133
alpha =	90.0
beta =	90.0
gamma =	90.0

	Atom	X	Y	Z	X	Y	Z
1	Ni	0.0000	0.0000	0.0000	0.0000	0.0000	0.0000
2	Ni	1.7379	1.7379	0.0000	0.2500	0.2500	0.0000
3	Ni	0.0000	3.4758	0.0000	0.0000	0.5000	0.0000
4	Ni	1.7379	5.2138	0.0000	0.2500	0.7500	0.0000
5	Ni	3.4758	0.0000	0.0000	0.5000	0.0000	0.0000
6	Ni	5.2138	1.7379	0.0000	0.7500	0.2500	0.0000
7	Ni	3.4758	3.4758	0.0000	0.5000	0.5000	0.0000
8	Ni	5.2138	5.2138	0.0000	0.7500	0.7500	0.0000
9	Ni	0.0007	-0.0020	3.5084	0.0001	-0.0003	0.1598
10	Ni	1.7300	1.7340	3.5241	0.2489	0.2494	0.1605
11	Ni	0.0038	3.4805	3.5121	0.0006	0.5007	0.1600
12	Ni	1.7343	5.2168	3.5096	0.2495	0.7504	0.1599
13	Ni	1.7195	3.4930	5.2441	0.2473	0.5025	0.2389
14	Ni	5.2188	5.2155	3.5127	0.7507	0.7503	0.1600
15	Ni	3.4791	1.7213	5.2351	0.5005	0.2476	0.2385
16	Ni	5.2344	3.4943	5.2375	0.7530	0.5027	0.2386
17	Co	1.7379	0.0000	1.7379	0.2500	0.0000	0.0792
18	Co	0.0000	1.7379	1.7379	0.0000	0.2500	0.0792
19	Co	1.7379	3.4758	1.7379	0.2500	0.5000	0.0792
20	Co	0.0000	5.2138	1.7379	0.0000	0.7500	0.0792
21	Co	5.2138	0.0000	1.7379	0.7500	0.0000	0.0792
22	Co	3.4758	1.7379	1.7379	0.5000	0.2500	0.0792
23	Co	5.2138	3.4758	1.7379	0.7500	0.5000	0.0792
24	Co	3.4758	5.2138	1.7379	0.5000	0.7500	0.0792
25	Co	1.7291	6.9241	5.2334	0.2487	0.9960	0.2384
26	Co	6.9308	1.7097	5.2436	0.9970	0.2459	0.2389
27	Co	3.4729	3.4766	3.5161	0.4996	0.5001	0.1602
28	Co	0.0056	5.2638	5.2553	0.0008	0.7572	0.2394
29	Co	5.2241	6.9335	5.2267	0.7515	0.9974	0.2381
30	Co	3.4691	5.2342	5.2298	0.4990	0.7529	0.2382
31	Co	3.4737	6.9403	3.5229	0.4997	0.9984	0.1605
32	Co	5.2254	1.7394	3.5245	0.7517	0.2502	0.1606

/db/jmorales/CoNi-alloy/Slabs/CoNi(001)-expanded

a =	7.044921511
b =	7.044921511
c =	22.044921511
alpha =	90.0
beta =	90.0
gamma =	90.0

	Atom	X	Y	Z	X	Y	Z
1	Ni	0.0000	0.0000	0.0000	0.0000	0.0000	0.0000
2	Ni	0.0000	3.5225	0.0000	0.0000	0.5000	0.0000
3	Ni	3.5225	0.0000	0.0000	0.5000	0.0000	0.0000
4	Ni	3.5225	3.5225	0.0000	0.5000	0.5000	0.0000
5	Ni	0.0000	1.7612	1.7612	0.0000	0.2500	0.0799
6	Ni	0.0000	5.2837	1.7612	0.0000	0.7500	0.0799
7	Ni	3.5225	1.7612	1.7612	0.5000	0.2500	0.0799
8	Ni	3.5225	5.2837	1.7612	0.5000	0.7500	0.0799
9	Ni	0.0000	0.0000	3.5225	0.0000	0.0000	0.1598
10	Ni	0.0000	3.5225	3.5225	0.0000	0.5000	0.1598
11	Ni	3.5225	0.0000	3.5225	0.5000	0.0000	0.1598
12	Ni	3.5225	3.5225	3.5225	0.5000	0.5000	0.1598
13	Ni	0.0000	1.7612	5.2837	0.0000	0.2500	0.2397
14	Ni	0.0000	5.2837	5.2837	0.0000	0.7500	0.2397
15	Ni	3.5225	1.7612	5.2837	0.5000	0.2500	0.2397
16	Ni	3.5225	5.2837	5.2837	0.5000	0.7500	0.2397
17	Co	1.7612	1.7612	0.0000	0.2500	0.2500	0.0000
18	Co	1.7612	5.2837	0.0000	0.2500	0.7500	0.0000
19	Co	5.2837	1.7612	0.0000	0.7500	0.2500	0.0000
20	Co	5.2837	5.2837	0.0000	0.7500	0.7500	0.0000
21	Co	1.7612	0.0000	1.7612	0.2500	0.0000	0.0799
22	Co	1.7612	3.5225	1.7612	0.2500	0.5000	0.0799
23	Co	5.2837	0.0000	1.7612	0.7500	0.0000	0.0799
24	Co	5.2837	3.5225	1.7612	0.7500	0.5000	0.0799
25	Co	1.7612	1.7612	3.5225	0.2500	0.2500	0.1598
26	Co	1.7612	5.2837	3.5225	0.2500	0.7500	0.1598
27	Co	5.2837	1.7612	3.5225	0.7500	0.2500	0.1598
28	Co	5.2837	5.2837	3.5225	0.7500	0.7500	0.1598
29	Co	1.7612	0.0000	5.2837	0.2500	0.0000	0.2397
30	Co	1.7612	3.5225	5.2837	0.2500	0.5000	0.2397
31	Co	5.2837	0.0000	5.2837	0.7500	0.0000	0.2397
32	Co	5.2837	3.5225	5.2837	0.7500	0.5000	0.2397

/db/jmorales/CoNi-alloy/Slabs/Segregation/CoNi(111)/CoNi(111)-1-Co-CO

a =	9.831199646
b =	9.831199646075708
c =	21.0202999115
alpha =	90.0
beta =	90.0
gamma =	120.0

	Atom	X	Y	Z	X	Y	Z
1	Ni	0.0000	0.0000	0.0000	0.0000	0.0000	0.0000
2	Ni	-1.2289	2.1285	0.0000	0.0000	0.2500	0.0000
3	Ni	-2.4578	4.2570	0.0000	0.0000	0.5000	0.0000
4	Ni	-3.6867	6.3856	0.0000	0.0000	0.7500	0.0000
5	Ni	4.9156	0.0000	0.0000	0.5000	0.0000	0.0000
6	Ni	3.6867	2.1285	0.0000	0.5000	0.2500	0.0000
7	Ni	2.4578	4.2570	0.0000	0.5000	0.5000	0.0000
8	Ni	1.2289	6.3856	0.0000	0.5000	0.7500	0.0000
9	Ni	2.4578	1.4190	2.0068	0.3333	0.1667	0.0955
10	Ni	1.2289	3.5475	2.0068	0.3333	0.4167	0.0955
11	Ni	0.0000	5.6760	2.0068	0.3333	0.6667	0.0955
12	Ni	-1.2289	7.8045	2.0068	0.3333	0.9167	0.0955
13	Ni	7.3734	1.4190	2.0068	0.8333	0.1667	0.0955
14	Ni	6.1445	3.5475	2.0068	0.8333	0.4167	0.0955
15	Ni	4.9156	5.6760	2.0068	0.8333	0.6667	0.0955
16	Ni	3.6867	7.8045	2.0068	0.8333	0.9167	0.0955
17	Ni	0.0294	2.8556	4.0081	0.1707	0.3354	0.1907
18	Ni	1.2540	0.7285	4.0104	0.1703	0.0856	0.1908
19	Ni	-2.4341	7.1089	4.0177	0.1699	0.8350	0.1911
20	Ni	-1.2011	4.9838	4.0145	0.1705	0.5854	0.1910
21	Ni	4.9352	2.8635	4.0178	0.6701	0.3363	0.1911
22	Ni	6.1770	0.7249	4.0128	0.6709	0.0851	0.1909
23	Ni	2.4862	7.1091	4.0154	0.6704	0.8350	0.1910
24	Ni	3.7104	4.9826	4.0184	0.6700	0.5852	0.1912
25	Ni	0.0489	0.0323	6.0276	0.0069	0.0038	0.2868
26	Ni	-1.1834	2.1569	6.0262	0.0063	0.2533	0.2867
27	Ni	-2.4123	4.2855	6.0247	0.0063	0.5033	0.2866
28	Ni	-3.6398	6.4159	6.0327	0.0066	0.7536	0.2870
29	Ni	4.9715	0.0312	6.0171	0.5075	0.0037	0.2863
30	Ni	2.5138	4.2930	6.0782	0.5078	0.5042	0.2892
31	Ni	1.2771	6.4167	6.0266	0.5067	0.7537	0.2867
32	Ni	3.7082	0.7318	4.0337	0.4202	0.0860	0.1919
33	Co	1.2289	2.1285	0.0000	0.2500	0.2500	0.0000
34	Co	2.4578	0.0000	0.0000	0.2500	0.0000	0.0000
35	Co	-1.2289	6.3856	0.0000	0.2500	0.7500	0.0000
36	Co	0.0000	4.2570	0.0000	0.2500	0.5000	0.0000
37	Co	6.1445	2.1285	0.0000	0.7500	0.2500	0.0000
38	Co	7.3734	0.0000	0.0000	0.7500	0.0000	0.0000
39	Co	3.6867	6.3856	0.0000	0.7500	0.7500	0.0000
40	Co	4.9156	4.2570	0.0000	0.7500	0.5000	0.0000
41	Co	-1.2289	3.5475	2.0068	0.0833	0.4167	0.0955

42	Co	0.0000	1.4190	2.0068	0.0833	0.1667	0.0955
43	Co	-3.6867	7.8045	2.0068	0.0833	0.9167	0.0955
44	Co	-2.4578	5.6760	2.0068	0.0833	0.6667	0.0955
45	Co	3.6867	3.5475	2.0068	0.5833	0.4167	0.0955
46	Co	4.9156	1.4190	2.0068	0.5833	0.1667	0.0955
47	Co	1.2289	7.8045	2.0068	0.5833	0.9167	0.0955
48	Co	2.4578	5.6760	2.0068	0.5833	0.6667	0.0955
49	Co	2.4791	2.8596	4.0616	0.4201	0.3359	0.1932
50	Co	1.2503	4.9773	4.0601	0.4195	0.5846	0.1932
51	Co	0.0318	7.1001	4.0547	0.4202	0.8339	0.1929
52	Co	8.6292	0.7272	4.0578	0.9204	0.0854	0.1930
53	Co	7.3952	2.8526	4.0534	0.9197	0.3350	0.1928
54	Co	6.1662	4.9830	4.0569	0.9198	0.5853	0.1930
55	Co	4.9405	7.1100	4.0593	0.9201	0.8351	0.1931
56	Co	1.2811	2.1606	6.0619	0.2572	0.2538	0.2884
57	Co	2.5205	0.0218	6.0241	0.2577	0.0026	0.2866
58	Co	-1.1625	6.4318	6.0133	0.2595	0.7554	0.2861
59	Co	0.0430	4.3140	6.0110	0.2577	0.5067	0.2860
60	Co	6.2033	2.1662	6.0072	0.7582	0.2544	0.2858
61	Co	7.4351	0.0416	6.0254	0.7587	0.0049	0.2866
62	Co	3.7513	6.4285	6.0157	0.7591	0.7550	0.2862
63	Co	4.9941	4.3032	6.0146	0.7607	0.5054	0.2861
64	Co	3.7360	2.1585	6.0403	0.5068	0.2535	0.2874
65	O	2.5171	2.8526	8.6110	0.4236	0.3350	0.4097
66	C	2.4956	2.7998	7.4164	0.4183	0.3288	0.3528

/db/jmorales/CoNi-alloy/Slabs/Segregation/CoNi(111)/CoNi(111)-4-Ni

a =			9.831199646				
b =			9.831199646075708				
c =			21.0202999115				
alpha =			90.0				
beta =			90.0				
gamma =			120.0				
	Atom	X	Y	Z	X	Y	Z
1	Ni	0.0000	0.0000	0.0000	0.0000	0.0000	0.0000
2	Ni	-1.2289	2.1285	0.0000	0.0000	0.2500	0.0000
3	Ni	-2.4578	4.2570	0.0000	0.0000	0.5000	0.0000
4	Ni	-3.6867	6.3856	0.0000	0.0000	0.7500	0.0000
5	Ni	4.9156	0.0000	0.0000	0.5000	0.0000	0.0000
6	Ni	3.6867	2.1285	0.0000	0.5000	0.2500	0.0000
7	Ni	2.4578	4.2570	0.0000	0.5000	0.5000	0.0000
8	Ni	1.2289	6.3856	0.0000	0.5000	0.7500	0.0000
9	Ni	2.4578	1.4190	2.0068	0.3333	0.1667	0.0955
10	Ni	1.2289	3.5475	2.0068	0.3333	0.4167	0.0955
11	Ni	0.0000	5.6760	2.0068	0.3333	0.6667	0.0955
12	Ni	-1.2289	7.8045	2.0068	0.3333	0.9167	0.0955
13	Ni	7.3734	1.4190	2.0068	0.8333	0.1667	0.0955
14	Ni	6.1445	3.5475	2.0068	0.8333	0.4167	0.0955
15	Ni	4.9156	5.6760	2.0068	0.8333	0.6667	0.0955
16	Ni	3.6867	7.8045	2.0068	0.8333	0.9167	0.0955
17	Ni	4.9466	2.8562	4.0085	0.6709	0.3355	0.1907
18	Ni	6.1754	0.7274	4.0088	0.6709	0.0854	0.1907
19	Ni	2.4888	7.1133	4.0087	0.6709	0.8355	0.1907
20	Ni	3.7173	4.9847	4.0084	0.6708	0.5855	0.1907
21	Ni	0.0380	0.0228	6.0363	0.0052	0.0027	0.2872
22	Ni	-1.1904	2.1517	6.0363	0.0053	0.2527	0.2872
23	Ni	-2.4185	4.2804	6.0369	0.0054	0.5027	0.2872
24	Ni	-3.6483	6.4090	6.0353	0.0053	0.7527	0.2871
25	Ni	4.9688	0.0313	6.0140	0.5073	0.0037	0.2861
26	Ni	3.7395	2.1599	6.0131	0.5072	0.2537	0.2861
27	Ni	2.5109	4.2884	6.0126	0.5072	0.5037	0.2860
28	Ni	1.2820	6.4170	6.0141	0.5072	0.7537	0.2861
29	Ni	1.2750	2.1552	6.0227	0.2563	0.2531	0.2865
30	Ni	2.5037	0.0270	6.0230	0.2563	0.0032	0.2865
31	Ni	0.0461	4.2846	6.0230	0.2563	0.5032	0.2865
32	Ni	-1.1828	6.4127	6.0217	0.2563	0.7532	0.2865
33	Co	1.2289	2.1285	0.0000	0.2500	0.2500	0.0000
34	Co	2.4578	0.0000	0.0000	0.2500	0.0000	0.0000
35	Co	-1.2289	6.3856	0.0000	0.2500	0.7500	0.0000
36	Co	0.0000	4.2570	0.0000	0.2500	0.5000	0.0000
37	Co	6.1445	2.1285	0.0000	0.7500	0.2500	0.0000
38	Co	7.3734	0.0000	0.0000	0.7500	0.0000	0.0000
39	Co	3.6867	6.3856	0.0000	0.7500	0.7500	0.0000
40	Co	4.9156	4.2570	0.0000	0.7500	0.5000	0.0000
41	Co	-1.2289	3.5475	2.0068	0.0833	0.4167	0.0955

42	Co	0.0000	1.4190	2.0068	0.0833	0.1667	0.0955
43	Co	-3.6867	7.8045	2.0068	0.0833	0.9167	0.0955
44	Co	-2.4578	5.6760	2.0068	0.0833	0.6667	0.0955
45	Co	3.6867	3.5475	2.0068	0.5833	0.4167	0.0955
46	Co	4.9156	1.4190	2.0068	0.5833	0.1667	0.0955
47	Co	1.2289	7.8045	2.0068	0.5833	0.9167	0.0955
48	Co	2.4578	5.6760	2.0068	0.5833	0.6667	0.0955
49	Co	3.7131	0.7256	4.0303	0.4203	0.0852	0.1917
50	Co	2.4847	2.8533	4.0294	0.4203	0.3351	0.1917
51	Co	1.2554	4.9826	4.0299	0.4203	0.5852	0.1917
52	Co	0.0273	7.1107	4.0297	0.4204	0.8352	0.1917
53	Co	8.6435	0.7336	4.0521	0.9223	0.0862	0.1928
54	Co	7.4142	2.8623	4.0519	0.9222	0.3362	0.1928
55	Co	6.1855	4.9903	4.0517	0.9222	0.5861	0.1928
56	Co	4.9565	7.1193	4.0516	0.9223	0.8362	0.1927
57	Co	6.2038	2.1641	6.0043	0.7581	0.2542	0.2856
58	Co	7.4325	0.0348	6.0048	0.7581	0.0041	0.2857
59	Co	3.7459	6.4205	6.0045	0.7581	0.7541	0.2857
60	Co	4.9756	4.2918	6.0042	0.7581	0.5041	0.2856
61	Co	1.2532	0.7239	4.0044	0.1700	0.0850	0.1905
62	Co	-2.4329	7.1100	4.0046	0.1701	0.8351	0.1905
63	Co	0.0251	2.8533	4.0058	0.1701	0.3351	0.1906
64	Co	-1.2047	4.9808	4.0043	0.1700	0.5850	0.1905

/db/jmorales/CoNi-alloy/Slabs/Segregation/CoNi(111)/CoNi(111)-1-Ni-CO

a =				9.831199646			
b =				9.831199646075708			
c =				21.0202999115			
alpha =				90.0			
beta =				90.0			
gamma =				120.0			
	Atom	X	Y	Z	X	Y	Z
1	Ni	0.0000	0.0000	0.0000	0.0000	0.0000	0.0000
2	Ni	-1.2289	2.1285	0.0000	0.0000	0.2500	0.0000
3	Ni	-2.4578	4.2570	0.0000	0.0000	0.5000	0.0000
4	Ni	-3.6867	6.3856	0.0000	0.0000	0.7500	0.0000
5	Ni	4.9156	0.0000	0.0000	0.5000	0.0000	0.0000
6	Ni	3.6867	2.1285	0.0000	0.5000	0.2500	0.0000
7	Ni	2.4578	4.2570	0.0000	0.5000	0.5000	0.0000
8	Ni	1.2289	6.3856	0.0000	0.5000	0.7500	0.0000
9	Ni	2.4578	1.4190	2.0068	0.3333	0.1667	0.0955
10	Ni	1.2289	3.5475	2.0068	0.3333	0.4167	0.0955
11	Ni	0.0000	5.6760	2.0068	0.3333	0.6667	0.0955
12	Ni	-1.2289	7.8045	2.0068	0.3333	0.9167	0.0955
13	Ni	7.3734	1.4190	2.0068	0.8333	0.1667	0.0955
14	Ni	6.1445	3.5475	2.0068	0.8333	0.4167	0.0955
15	Ni	4.9156	5.6760	2.0068	0.8333	0.6667	0.0955
16	Ni	3.6867	7.8045	2.0068	0.8333	0.9167	0.0955
17	Ni	0.0357	2.8559	4.0065	0.1713	0.3354	0.1906
18	Ni	-2.4257	7.1054	4.0122	0.1705	0.8345	0.1909
19	Ni	-1.1996	4.9826	4.0138	0.1706	0.5852	0.1910
20	Ni	4.9425	2.8522	4.0188	0.6702	0.3350	0.1912
21	Ni	6.1705	0.7286	4.0099	0.6704	0.0856	0.1908
22	Ni	2.4883	7.1080	4.0106	0.6705	0.8349	0.1908
23	Ni	3.7119	4.9821	4.0211	0.6701	0.5852	0.1913
24	Ni	0.0474	0.0277	6.0327	0.0064	0.0033	0.2870
25	Ni	-1.1842	2.1551	6.0285	0.0061	0.2531	0.2868
26	Ni	-2.4109	4.2878	6.0212	0.0066	0.5036	0.2864
27	Ni	-3.6390	6.4132	6.0260	0.0065	0.7532	0.2867
28	Ni	4.9693	0.0289	6.0223	0.5072	0.0034	0.2865
29	Ni	3.7450	2.1660	6.0791	0.5081	0.2544	0.2892
30	Ni	2.5183	4.2905	6.0855	0.5081	0.5039	0.2895
31	Ni	1.2810	6.4213	6.0241	0.5074	0.7542	0.2866
32	Ni	1.2742	2.1616	6.0898	0.2565	0.2539	0.2897
33	Co	1.2289	2.1285	0.0000	0.2500	0.2500	0.0000
34	Co	2.4578	0.0000	0.0000	0.2500	0.0000	0.0000
35	Co	-1.2289	6.3856	0.0000	0.2500	0.7500	0.0000
36	Co	0.0000	4.2570	0.0000	0.2500	0.5000	0.0000
37	Co	6.1445	2.1285	0.0000	0.7500	0.2500	0.0000
38	Co	7.3734	0.0000	0.0000	0.7500	0.0000	0.0000
39	Co	3.6867	6.3856	0.0000	0.7500	0.7500	0.0000
40	Co	4.9156	4.2570	0.0000	0.7500	0.5000	0.0000
41	Co	-1.2289	3.5475	2.0068	0.0833	0.4167	0.0955

42	Co	0.0000	1.4190	2.0068	0.0833	0.1667	0.0955
43	Co	-3.6867	7.8045	2.0068	0.0833	0.9167	0.0955
44	Co	-2.4578	5.6760	2.0068	0.0833	0.6667	0.0955
45	Co	3.6867	3.5475	2.0068	0.5833	0.4167	0.0955
46	Co	4.9156	1.4190	2.0068	0.5833	0.1667	0.0955
47	Co	1.2289	7.8045	2.0068	0.5833	0.9167	0.0955
48	Co	2.4578	5.6760	2.0068	0.5833	0.6667	0.0955
49	Co	3.7002	0.7295	4.0539	0.4192	0.0857	0.1929
50	Co	2.4886	2.8515	4.0009	0.4206	0.3349	0.1903
51	Co	1.2555	4.9722	4.0610	0.4197	0.5840	0.1932
52	Co	0.0283	7.1114	4.0521	0.4205	0.8353	0.1928
53	Co	8.6418	0.7311	4.0499	0.9220	0.0859	0.1927
54	Co	7.4013	2.8534	4.0556	0.9204	0.3351	0.1929
55	Co	6.1706	4.9799	4.0500	0.9201	0.5849	0.1927
56	Co	4.9472	7.1170	4.0555	0.9212	0.8359	0.1929
57	Co	2.5185	0.0056	5.9887	0.2565	0.0007	0.2849
58	Co	-1.1594	6.4252	6.0068	0.2594	0.7547	0.2858
59	Co	0.0342	4.3235	6.0042	0.2574	0.5078	0.2856
60	Co	6.2149	2.1635	6.0093	0.7592	0.2541	0.2859
61	Co	7.4378	0.0394	6.0121	0.7589	0.0046	0.2860
62	Co	3.7525	6.4279	6.0118	0.7592	0.7550	0.2860
63	Co	5.0044	4.3103	6.0047	0.7622	0.5063	0.2857
64	Co	1.2553	0.7205	3.9998	0.1700	0.0846	0.1903
65	O	2.4941	2.8519	8.6178	0.4212	0.3350	0.4100
66	C	2.4902	2.8530	7.4246	0.4208	0.3351	0.3532

/db/jmorales/CoNi-alloy/Slabs/Co-CoNi(001)

a =	6.951669213		
b =	6.951669213		
c =	21.951669213		
alpha =	90.0		
beta =	90.0		
gamma =	90.0		

	Atom	X	Y	Z	X	Y	Z
1	Ni	0.0000	0.0000	0.0000	0.0000	0.0000	0.0000
2	Ni	1.7379	1.7379	0.0000	0.2500	0.2500	0.0000
3	Ni	0.0000	3.4758	0.0000	0.0000	0.5000	0.0000
4	Ni	1.7379	5.2138	0.0000	0.2500	0.7500	0.0000
5	Ni	3.4758	0.0000	0.0000	0.5000	0.0000	0.0000
6	Ni	5.2138	1.7379	0.0000	0.7500	0.2500	0.0000
7	Ni	3.4758	3.4758	0.0000	0.5000	0.5000	0.0000
8	Ni	5.2138	5.2138	0.0000	0.7500	0.7500	0.0000
9	Ni	0.0000	0.0000	3.5158	0.0000	0.0000	0.1602
10	Ni	1.7379	1.7379	3.4758	0.2500	0.2500	0.1583
11	Ni	0.0000	3.4758	3.5158	0.0000	0.5000	0.1602
12	Ni	1.7379	5.2138	3.4758	0.2500	0.7500	0.1583
13	Ni	3.4758	0.0000	3.5158	0.5000	0.0000	0.1602
14	Ni	5.2138	1.7379	3.4758	0.7500	0.2500	0.1583
15	Ni	3.4758	3.4758	3.5158	0.5000	0.5000	0.1602
16	Ni	5.2138	5.2138	3.4758	0.7500	0.7500	0.1583
17	Co	1.7379	0.0000	1.7379	0.2500	0.0000	0.0792
18	Co	0.0000	1.7379	1.7379	0.0000	0.2500	0.0792
19	Co	1.7379	3.4758	1.7379	0.2500	0.5000	0.0792
20	Co	0.0000	5.2138	1.7379	0.0000	0.7500	0.0792
21	Co	5.2138	0.0000	1.7379	0.7500	0.0000	0.0792
22	Co	3.4758	1.7379	1.7379	0.5000	0.2500	0.0792
23	Co	5.2138	3.4758	1.7379	0.7500	0.5000	0.0792
24	Co	3.4758	5.2138	1.7379	0.5000	0.7500	0.0792
25	Co	1.7379	0.0000	5.2498	0.2500	0.0000	0.2392
26	Co	0.0000	1.7379	5.2498	0.0000	0.2500	0.2392
27	Co	1.7379	3.4758	5.2498	0.2500	0.5000	0.2392
28	Co	0.0000	5.2138	5.2498	0.0000	0.7500	0.2392
29	Co	5.2138	0.0000	5.2498	0.7500	0.0000	0.2392
30	Co	3.4758	1.7379	5.2498	0.5000	0.2500	0.2392
31	Co	5.2138	3.4758	5.2498	0.7500	0.5000	0.2392
32	Co	3.4758	5.2138	5.2498	0.5000	0.7500	0.2392

/db/jmorales/CoNi-alloy/Slabs/Segregation/Co-CoNi(001)/Co-CoNi(001)-1-Ni

a =	6.9516692132
b =	6.9516692132
c =	21.9516692133
alpha =	90.0
beta =	90.0
gamma =	90.0

	Atom	X	Y	Z	X	Y	Z
1	Ni	0.0000	0.0000	0.0000	0.0000	0.0000	0.0000
2	Ni	1.7379	1.7379	0.0000	0.2500	0.2500	0.0000
3	Ni	0.0000	3.4758	0.0000	0.0000	0.5000	0.0000
4	Ni	1.7379	5.2138	0.0000	0.2500	0.7500	0.0000
5	Ni	3.4758	0.0000	0.0000	0.5000	0.0000	0.0000
6	Ni	5.2138	1.7379	0.0000	0.7500	0.2500	0.0000
7	Ni	3.4758	3.4758	0.0000	0.5000	0.5000	0.0000
8	Ni	5.2138	5.2138	0.0000	0.7500	0.7500	0.0000
9	Ni	-0.0009	0.0000	3.5238	-0.0001	0.0000	0.1605
10	Ni	1.7343	1.7281	3.5270	0.2495	0.2486	0.1607
11	Ni	-0.0082	3.4758	3.5285	-0.0012	0.5000	0.1607
12	Ni	1.7343	5.2235	3.5270	0.2495	0.7514	0.1607
13	Ni	3.4744	0.0000	3.5290	0.4998	0.0000	0.1608
14	Ni	5.2198	1.7334	3.5183	0.7509	0.2493	0.1603
15	Ni	1.7285	3.4758	5.2571	0.2486	0.5000	0.2395
16	Ni	5.2198	5.2183	3.5183	0.7509	0.7507	0.1603
17	Co	1.7379	0.0000	1.7379	0.2500	0.0000	0.0792
18	Co	0.0000	1.7379	1.7379	0.0000	0.2500	0.0792
19	Co	1.7379	3.4758	1.7379	0.2500	0.5000	0.0792
20	Co	0.0000	5.2138	1.7379	0.0000	0.7500	0.0792
21	Co	5.2138	0.0000	1.7379	0.7500	0.0000	0.0792
22	Co	3.4758	1.7379	1.7379	0.5000	0.2500	0.0792
23	Co	5.2138	3.4758	1.7379	0.7500	0.5000	0.0792
24	Co	3.4758	5.2138	1.7379	0.5000	0.7500	0.0792
25	Co	1.7370	0.0000	5.2642	0.2499	0.0000	0.2398
26	Co	-0.0215	1.7142	5.2701	-0.0031	0.2466	0.2401
27	Co	3.4781	3.4758	3.5267	0.5003	0.5000	0.1607
28	Co	-0.0215	5.2375	5.2701	-0.0031	0.7534	0.2401
29	Co	5.2151	0.0000	5.2560	0.7502	0.0000	0.2394
30	Co	3.4945	1.7445	5.2585	0.5027	0.2510	0.2395
31	Co	5.2020	3.4758	5.2523	0.7483	0.5000	0.2393
32	Co	3.4945	5.2071	5.2585	0.5027	0.7490	0.2395

/db/jmorales/CoNi-alloy/Slabs/Segregation/CoNi(001)/CoNi(001)-2-Co-CO

a =	6.9516692132
b =	6.9516692132
c =	21.9516692133
alpha =	90.0
beta =	90.0
gamma =	90.0

	Atom	X	Y	Z	X	Y	Z
1	Ni	0.0000	0.0000	0.0000	0.0000	0.0000	0.0000
2	Ni	0.0000	3.4758	0.0000	0.0000	0.5000	0.0000
3	Ni	3.4758	0.0000	0.0000	0.5000	0.0000	0.0000
4	Ni	3.4758	3.4758	0.0000	0.5000	0.5000	0.0000
5	Ni	0.0000	1.7379	1.7379	0.0000	0.2500	0.0792
6	Ni	0.0000	5.2138	1.7379	0.0000	0.7500	0.0792
7	Ni	3.4758	1.7379	1.7379	0.5000	0.2500	0.0792
8	Ni	3.4758	5.2138	1.7379	0.5000	0.7500	0.0792
9	Ni	6.9466	0.0004	3.5167	0.9993	0.0001	0.1602
10	Ni	6.9469	3.4756	3.5162	0.9993	0.5000	0.1602
11	Ni	3.4764	-0.0000	3.5177	0.5001	-0.0000	0.1602
12	Ni	3.4770	3.4757	3.4717	0.5002	0.5000	0.1582
13	Ni	0.0119	1.7351	5.2282	0.0017	0.2496	0.2382
14	Ni	0.0109	5.2167	5.2269	0.0016	0.7504	0.2381
15	Ni	1.7352	1.7387	3.5305	0.2496	0.2501	0.1608
16	Ni	1.7358	5.2127	3.5300	0.2497	0.7498	0.1608
17	Co	1.7379	1.7379	0.0000	0.2500	0.2500	0.0000
18	Co	1.7379	5.2138	0.0000	0.2500	0.7500	0.0000
19	Co	5.2138	1.7379	0.0000	0.7500	0.2500	0.0000
20	Co	5.2138	5.2138	0.0000	0.7500	0.7500	0.0000
21	Co	1.7379	0.0000	1.7379	0.2500	0.0000	0.0792
22	Co	1.7379	3.4758	1.7379	0.2500	0.5000	0.0792
23	Co	5.2138	0.0000	1.7379	0.7500	0.0000	0.0792
24	Co	5.2138	3.4758	1.7379	0.7500	0.5000	0.0792
25	Co	3.4571	1.7500	5.2298	0.4973	0.2517	0.2382
26	Co	5.2136	1.7386	3.5650	0.7500	0.2501	0.1624
27	Co	5.2134	5.2130	3.5647	0.7499	0.7499	0.1624
28	Co	1.7706	6.9503	5.2424	0.2547	0.9998	0.2388
29	Co	1.7850	3.4764	5.2769	0.2568	0.5001	0.2404
30	Co	5.1808	0.0003	5.2327	0.7453	0.0000	0.2384
31	Co	5.1757	3.4748	5.2701	0.7445	0.4998	0.2401
32	Co	3.4581	5.2012	5.2298	0.4974	0.7482	0.2382
33	O	3.4901	3.4744	7.6260	0.5021	0.4998	0.3474
34	C	3.4722	3.4740	6.4114	0.4995	0.4997	0.2921

/db/jmorales/CoNi-alloy/Slabs/Islanding/CoNi(111)/CoNi(111)-4-Co

a = 9.831199646
b = 9.831199646075708
c = 21.0202999115
alpha = 90.0
beta = 90.0
gamma = 120.0

	Atom	X	Y	Z	X	Y	Z
1	Ni	0.0000	0.0000	0.0000	0.0000	0.0000	0.0000
2	Ni	-1.2289	2.1285	0.0000	0.0000	0.2500	0.0000
3	Ni	-2.4578	4.2570	0.0000	0.0000	0.5000	0.0000
4	Ni	-3.6867	6.3856	0.0000	0.0000	0.7500	0.0000
5	Ni	4.9156	0.0000	0.0000	0.5000	0.0000	0.0000
6	Ni	3.6867	2.1285	0.0000	0.5000	0.2500	0.0000
7	Ni	2.4578	4.2570	0.0000	0.5000	0.5000	0.0000
8	Ni	1.2289	6.3856	0.0000	0.5000	0.7500	0.0000
9	Ni	2.4578	1.4190	2.0068	0.3333	0.1667	0.0955
10	Ni	1.2289	3.5475	2.0068	0.3333	0.4167	0.0955
11	Ni	0.0000	5.6760	2.0068	0.3333	0.6667	0.0955
12	Ni	-1.2289	7.8045	2.0068	0.3333	0.9167	0.0955
13	Ni	7.3734	1.4190	2.0068	0.8333	0.1667	0.0955
14	Ni	6.1445	3.5475	2.0068	0.8333	0.4167	0.0955
15	Ni	4.9156	5.6760	2.0068	0.8333	0.6667	0.0955
16	Ni	3.6867	7.8045	2.0068	0.8333	0.9167	0.0955
17	Ni	0.0372	2.8585	4.0203	0.1717	0.3357	0.1913
18	Ni	1.2654	0.7253	4.0110	0.1713	0.0852	0.1908
19	Ni	-2.4252	7.1133	3.9992	0.1711	0.8355	0.1903
20	Ni	-1.2030	4.9859	4.0030	0.1704	0.5856	0.1904
21	Ni	4.9529	2.8580	4.0199	0.6716	0.3357	0.1912
22	Ni	6.1810	0.7253	4.0112	0.6713	0.0852	0.1908
23	Ni	2.4906	7.1135	3.9995	0.6711	0.8355	0.1903
24	Ni	3.7127	4.9864	4.0023	0.6705	0.5857	0.1904
25	Ni	0.0532	0.0355	6.0337	0.0075	0.0042	0.2870
26	Ni	-3.6405	6.4089	6.0305	0.0061	0.7527	0.2869
27	Ni	4.9686	0.0356	6.0339	0.5075	0.0042	0.2871
28	Ni	1.2755	6.4081	6.0308	0.5061	0.7526	0.2869
29	Ni	2.5114	0.0437	6.0521	0.2580	0.0051	0.2879
30	Ni	3.7423	6.4039	6.0462	0.7567	0.7522	0.2876
31	Ni	7.4271	0.0430	6.0519	0.7580	0.0050	0.2879
32	Ni	-1.1728	6.4052	6.0459	0.2569	0.7523	0.2876
33	Co	1.2289	2.1285	0.0000	0.2500	0.2500	0.0000
34	Co	2.4578	0.0000	0.0000	0.2500	0.0000	0.0000
35	Co	-1.2289	6.3856	0.0000	0.2500	0.7500	0.0000
36	Co	0.0000	4.2570	0.0000	0.2500	0.5000	0.0000
37	Co	6.1445	2.1285	0.0000	0.7500	0.2500	0.0000
38	Co	7.3734	0.0000	0.0000	0.7500	0.0000	0.0000
39	Co	3.6867	6.3856	0.0000	0.7500	0.7500	0.0000
40	Co	4.9156	4.2570	0.0000	0.7500	0.5000	0.0000
41	Co	-1.2289	3.5475	2.0068	0.0833	0.4167	0.0955

42	Co	0.0000	1.4190	2.0068	0.0833	0.1667	0.0955
43	Co	-3.6867	7.8045	2.0068	0.0833	0.9167	0.0955
44	Co	-2.4578	5.6760	2.0068	0.0833	0.6667	0.0955
45	Co	3.6867	3.5475	2.0068	0.5833	0.4167	0.0955
46	Co	4.9156	1.4190	2.0068	0.5833	0.1667	0.0955
47	Co	1.2289	7.8045	2.0068	0.5833	0.9167	0.0955
48	Co	2.4578	5.6760	2.0068	0.5833	0.6667	0.0955
49	Co	3.7246	0.7332	4.0526	0.4219	0.0861	0.1928
50	Co	2.4923	2.8587	4.0563	0.4214	0.3358	0.1930
51	Co	1.2553	4.9809	4.0528	0.4202	0.5850	0.1928
52	Co	0.0364	7.1156	4.0419	0.4216	0.8357	0.1923
53	Co	8.6403	0.7331	4.0520	0.9219	0.0861	0.1928
54	Co	7.4075	2.8587	4.0573	0.9213	0.3358	0.1930
55	Co	6.1705	4.9805	4.0523	0.9201	0.5850	0.1928
56	Co	4.9520	7.1156	4.0421	0.9216	0.8357	0.1923
57	Co	1.2860	2.1711	6.0217	0.2583	0.2550	0.2865
58	Co	0.0567	4.2773	6.0103	0.2570	0.5024	0.2859
59	Co	6.2010	2.1707	6.0236	0.7582	0.2550	0.2866
60	Co	4.9730	4.2778	6.0101	0.7571	0.5024	0.2859
61	Co	3.7337	2.1417	5.9998	0.5055	0.2515	0.2854
62	Co	2.4922	4.2788	5.9909	0.5048	0.5026	0.2850
63	Co	-1.1818	2.1411	6.0004	0.0055	0.2515	0.2855
64	Co	-2.4235	4.2791	5.9918	0.0048	0.5026	0.2851

/db/jmorales/CoNi-alloy/Slabs/Islanding/CoNi(111)/CoNi(111)-1-Ni-CO

a = 9.831199646
b = 9.831199646075708
c = 21.0202999115
alpha = 90.0
beta = 90.0
gamma = 120.0

	Atom	X	Y	Z	X	Y	Z
1	Ni	0.0000	0.0000	0.0000	0.0000	0.0000	0.0000
2	Ni	-1.2289	2.1285	0.0000	0.0000	0.2500	0.0000
3	Ni	-2.4578	4.2570	0.0000	0.0000	0.5000	0.0000
4	Ni	-3.6867	6.3856	0.0000	0.0000	0.7500	0.0000
5	Ni	4.9156	0.0000	0.0000	0.5000	0.0000	0.0000
6	Ni	3.6867	2.1285	0.0000	0.5000	0.2500	0.0000
7	Ni	2.4578	4.2570	0.0000	0.5000	0.5000	0.0000
8	Ni	1.2289	6.3856	0.0000	0.5000	0.7500	0.0000
9	Ni	2.4578	1.4190	2.0068	0.3333	0.1667	0.0955
10	Ni	1.2289	3.5475	2.0068	0.3333	0.4167	0.0955
11	Ni	0.0000	5.6760	2.0068	0.3333	0.6667	0.0955
12	Ni	-1.2289	7.8045	2.0068	0.3333	0.9167	0.0955
13	Ni	7.3734	1.4190	2.0068	0.8333	0.1667	0.0955
14	Ni	6.1445	3.5475	2.0068	0.8333	0.4167	0.0955
15	Ni	4.9156	5.6760	2.0068	0.8333	0.6667	0.0955
16	Ni	3.6867	7.8045	2.0068	0.8333	0.9167	0.0955
17	Ni	0.0367	2.8583	4.0053	0.1716	0.3357	0.1905
18	Ni	1.2646	0.7271	4.0044	0.1713	0.0854	0.1905
19	Ni	-2.4251	7.1130	4.0138	0.1710	0.8354	0.1909
20	Ni	-1.1965	4.9837	4.0149	0.1710	0.5853	0.1910
21	Ni	4.9474	2.8557	4.0238	0.6709	0.3354	0.1914
22	Ni	6.1741	0.7300	4.0101	0.6709	0.0857	0.1908
23	Ni	2.4946	7.1113	4.0134	0.6714	0.8352	0.1909
24	Ni	3.7173	4.9821	4.0221	0.6707	0.5852	0.1913
25	Ni	-1.1782	2.1622	6.0310	0.0071	0.2540	0.2869
26	Ni	-2.4061	4.2907	6.0256	0.0072	0.5040	0.2867
27	Ni	-3.6272	6.4085	6.0234	0.0074	0.7527	0.2866
28	Ni	4.9740	0.0320	6.0288	0.5078	0.0038	0.2868
29	Ni	3.7472	2.1665	6.0853	0.5084	0.2545	0.2895
30	Ni	2.5232	4.2910	6.0865	0.5086	0.5040	0.2896
31	Ni	1.2851	6.4210	6.0285	0.5078	0.7542	0.2868
32	Ni	1.2770	2.1544	6.0949	0.2564	0.2530	0.2900
33	Co	1.2289	2.1285	0.0000	0.2500	0.2500	0.0000
34	Co	2.4578	0.0000	0.0000	0.2500	0.0000	0.0000
35	Co	-1.2289	6.3856	0.0000	0.2500	0.7500	0.0000
36	Co	0.0000	4.2570	0.0000	0.2500	0.5000	0.0000
37	Co	6.1445	2.1285	0.0000	0.7500	0.2500	0.0000
38	Co	7.3734	0.0000	0.0000	0.7500	0.0000	0.0000
39	Co	3.6867	6.3856	0.0000	0.7500	0.7500	0.0000
40	Co	4.9156	4.2570	0.0000	0.7500	0.5000	0.0000
41	Co	-1.2289	3.5475	2.0068	0.0833	0.4167	0.0955

42	Co	0.0000	1.4190	2.0068	0.0833	0.1667	0.0955
43	Co	-3.6867	7.8045	2.0068	0.0833	0.9167	0.0955
44	Co	-2.4578	5.6760	2.0068	0.0833	0.6667	0.0955
45	Co	3.6867	3.5475	2.0068	0.5833	0.4167	0.0955
46	Co	4.9156	1.4190	2.0068	0.5833	0.1667	0.0955
47	Co	1.2289	7.8045	2.0068	0.5833	0.9167	0.0955
48	Co	2.4578	5.6760	2.0068	0.5833	0.6667	0.0955
49	Co	3.7093	0.7319	4.0593	0.4203	0.0860	0.1931
50	Co	2.4991	2.8608	4.0031	0.4222	0.3360	0.1904
51	Co	1.2609	4.9732	4.0641	0.4203	0.5841	0.1933
52	Co	0.0323	7.1133	4.0559	0.4210	0.8355	0.1930
53	Co	8.6436	0.7278	4.0540	0.9219	0.0855	0.1929
54	Co	7.4032	2.8525	4.0601	0.9206	0.3350	0.1932
55	Co	6.1727	4.9816	4.0514	0.9204	0.5851	0.1927
56	Co	4.9443	7.1151	4.0611	0.9208	0.8357	0.1932
57	Co	2.5272	0.0041	6.0034	0.2573	0.0005	0.2856
58	Co	-1.1565	6.4285	6.0121	0.2599	0.7550	0.2860
59	Co	0.0389	4.3200	6.0063	0.2577	0.5074	0.2857
60	Co	6.2212	2.1685	6.0148	0.7602	0.2547	0.2861
61	Co	7.4530	0.0316	6.0183	0.7599	0.0037	0.2863
62	Co	3.7532	6.4417	6.0216	0.7601	0.7566	0.2865
63	Co	5.0102	4.3137	6.0108	0.7629	0.5067	0.2860
64	Co	0.0353	0.0226	5.9933	0.0049	0.0027	0.2851
65	O	2.4897	2.8556	8.6220	0.4209	0.3354	0.4102
66	C	2.4805	2.8513	7.4287	0.4198	0.3349	0.3534

/db/jmorales/CoNi-alloy/Slabs/Segregation/CoNi(001)/CoNi(001)-4-Ni

a = 6.9516692132
b = 6.9516692132
c = 21.9516692133
alpha = 90.0
beta = 90.0
gamma = 90.0

	Atom	X	Y	Z	X	Y	Z
1	Ni	0.0000	0.0000	0.0000	0.0000	0.0000	0.0000
2	Ni	0.0000	3.4758	0.0000	0.0000	0.5000	0.0000
3	Ni	3.4758	0.0000	0.0000	0.5000	0.0000	0.0000
4	Ni	3.4758	3.4758	0.0000	0.5000	0.5000	0.0000
5	Ni	0.0000	1.7379	1.7379	0.0000	0.2500	0.0792
6	Ni	0.0000	5.2138	1.7379	0.0000	0.7500	0.0792
7	Ni	3.4758	1.7379	1.7379	0.5000	0.2500	0.0792
8	Ni	3.4758	5.2138	1.7379	0.5000	0.7500	0.0792
9	Ni	1.7379	3.4758	5.1967	0.2500	0.5000	0.2367
10	Ni	6.9515	1.7378	5.2009	1.0000	0.2500	0.2369
11	Ni	6.9515	5.2140	5.2008	1.0000	0.7500	0.2369
12	Ni	3.4756	1.7389	5.1994	0.5000	0.2501	0.2369
13	Ni	3.4757	5.2129	5.1995	0.5000	0.7499	0.2369
14	Ni	5.2137	3.4759	5.1967	0.7500	0.5000	0.2367
15	Ni	1.7380	0.0001	5.1987	0.2500	0.0000	0.2368
16	Ni	5.2137	0.0000	5.1986	0.7500	0.0000	0.2368
17	Co	1.7379	1.7379	0.0000	0.2500	0.2500	0.0000
18	Co	1.7379	5.2138	0.0000	0.2500	0.7500	0.0000
19	Co	5.2138	1.7379	0.0000	0.7500	0.2500	0.0000
20	Co	5.2138	5.2138	0.0000	0.7500	0.7500	0.0000
21	Co	1.7379	0.0000	1.7379	0.2500	0.0000	0.0792
22	Co	1.7379	3.4758	1.7379	0.2500	0.5000	0.0792
23	Co	5.2138	0.0000	1.7379	0.7500	0.0000	0.0792
24	Co	5.2138	3.4758	1.7379	0.7500	0.5000	0.0792
25	Co	1.7370	1.7374	3.5252	0.2499	0.2499	0.1606
26	Co	1.7371	5.2144	3.5251	0.2499	0.7501	0.1606
27	Co	5.2143	1.7375	3.5251	0.7501	0.2499	0.1606
28	Co	5.2143	5.2144	3.5251	0.7501	0.7501	0.1606
29	Co	3.4756	3.4760	3.5025	0.5000	0.5000	0.1596
30	Co	6.9515	3.4759	3.5035	1.0000	0.5000	0.1596
31	Co	-0.0002	0.0001	3.5036	-0.0000	0.0000	0.1596
32	Co	3.4760	-0.0000	3.5034	0.5000	-0.0000	0.1596

/db/jmorales/CoNi-alloy/Slabs/Islanding/CoNi(111)/CoNi(111)-1-Ni

a =		9.831199646					
b =		9.831199646075708					
c =		21.0202999115					
alpha =		90.0					
beta =		90.0					
gamma =		120.0					
	Atom	X	Y	Z	X	Y	Z
1	Ni	0.0000	0.0000	0.0000	0.0000	0.0000	0.0000
2	Ni	-1.2289	2.1285	0.0000	0.0000	0.2500	0.0000
3	Ni	-2.4578	4.2570	0.0000	0.0000	0.5000	0.0000
4	Ni	-3.6867	6.3856	0.0000	0.0000	0.7500	0.0000
5	Ni	4.9156	0.0000	0.0000	0.5000	0.0000	0.0000
6	Ni	3.6867	2.1285	0.0000	0.5000	0.2500	0.0000
7	Ni	2.4578	4.2570	0.0000	0.5000	0.5000	0.0000
8	Ni	1.2289	6.3856	0.0000	0.5000	0.7500	0.0000
9	Ni	2.4578	1.4190	2.0068	0.3333	0.1667	0.0955
10	Ni	1.2289	3.5475	2.0068	0.3333	0.4167	0.0955
11	Ni	0.0000	5.6760	2.0068	0.3333	0.6667	0.0955
12	Ni	-1.2289	7.8045	2.0068	0.3333	0.9167	0.0955
13	Ni	7.3734	1.4190	2.0068	0.8333	0.1667	0.0955
14	Ni	6.1445	3.5475	2.0068	0.8333	0.4167	0.0955
15	Ni	4.9156	5.6760	2.0068	0.8333	0.6667	0.0955
16	Ni	3.6867	7.8045	2.0068	0.8333	0.9167	0.0955
17	Ni	0.0350	2.8584	4.0033	0.1714	0.3357	0.1905
18	Ni	1.2634	0.7280	4.0015	0.1713	0.0855	0.1904
19	Ni	-2.4213	7.1142	4.0134	0.1715	0.8356	0.1909
20	Ni	-1.1938	4.9856	4.0153	0.1714	0.5856	0.1910
21	Ni	4.9531	2.8589	4.0162	0.6717	0.3358	0.1911
22	Ni	6.1774	0.7305	4.0109	0.6712	0.0858	0.1908
23	Ni	2.4964	7.1161	4.0142	0.6718	0.8358	0.1910
24	Ni	3.7231	4.9861	4.0141	0.6715	0.5856	0.1910
25	Ni	-1.1745	2.1661	6.0291	0.0077	0.2544	0.2868
26	Ni	-2.4028	4.2931	6.0270	0.0077	0.5042	0.2867
27	Ni	-3.6251	6.4101	6.0261	0.0077	0.7529	0.2867
28	Ni	4.9728	0.0330	6.0293	0.5078	0.0039	0.2868
29	Ni	3.7409	2.1619	6.0217	0.5075	0.2539	0.2865
30	Ni	2.5199	4.2885	6.0260	0.5082	0.5037	0.2867
31	Ni	1.2849	6.4193	6.0287	0.5077	0.7540	0.2868
32	Ni	1.2936	2.1617	6.0522	0.2585	0.2539	0.2879
33	Co	1.2289	2.1285	0.0000	0.2500	0.2500	0.0000
34	Co	2.4578	0.0000	0.0000	0.2500	0.0000	0.0000
35	Co	-1.2289	6.3856	0.0000	0.2500	0.7500	0.0000
36	Co	0.0000	4.2570	0.0000	0.2500	0.5000	0.0000
37	Co	6.1445	2.1285	0.0000	0.7500	0.2500	0.0000
38	Co	7.3734	0.0000	0.0000	0.7500	0.0000	0.0000
39	Co	3.6867	6.3856	0.0000	0.7500	0.7500	0.0000
40	Co	4.9156	4.2570	0.0000	0.7500	0.5000	0.0000
41	Co	-1.2289	3.5475	2.0068	0.0833	0.4167	0.0955

42	Co	0.0000	1.4190	2.0068	0.0833	0.1667	0.0955
43	Co	-3.6867	7.8045	2.0068	0.0833	0.9167	0.0955
44	Co	-2.4578	5.6760	2.0068	0.0833	0.6667	0.0955
45	Co	3.6867	3.5475	2.0068	0.5833	0.4167	0.0955
46	Co	4.9156	1.4190	2.0068	0.5833	0.1667	0.0955
47	Co	1.2289	7.8045	2.0068	0.5833	0.9167	0.0955
48	Co	2.4578	5.6760	2.0068	0.5833	0.6667	0.0955
49	Co	3.7156	0.7277	4.0530	0.4207	0.0855	0.1928
50	Co	2.4955	2.8596	4.0388	0.4218	0.3359	0.1921
51	Co	1.2618	4.9832	4.0572	0.4210	0.5853	0.1930
52	Co	0.0328	7.1141	4.0552	0.4211	0.8356	0.1929
53	Co	8.6402	0.7272	4.0568	0.9216	0.0854	0.1930
54	Co	7.4055	2.8534	4.0592	0.9208	0.3351	0.1931
55	Co	6.1785	4.9857	4.0502	0.9212	0.5856	0.1927
56	Co	4.9465	7.1165	4.0611	0.9211	0.8359	0.1932
57	Co	2.5253	0.0312	6.0130	0.2587	0.0037	0.2861
58	Co	-1.1541	6.4318	6.0168	0.2603	0.7554	0.2862
59	Co	0.0680	4.3040	6.0165	0.2597	0.5055	0.2862
60	Co	6.2225	2.1746	6.0186	0.7606	0.2554	0.2863
61	Co	7.4544	0.0330	6.0190	0.7602	0.0039	0.2863
62	Co	3.7592	6.4390	6.0231	0.7605	0.7563	0.2865
63	Co	4.9975	4.3044	6.0175	0.7611	0.5056	0.2863
64	Co	0.0413	0.0245	5.9933	0.0056	0.0029	0.2851

/db/jmorales/CoNi-alloy/Slabs/Segregation/Ni-CoNi(001)/Ni-CoNi(001)-2-Co

a =			6.9516692132				
b =			6.9516692132				
c =			21.9516692133				
alpha =			90.0				
beta =			90.0				
gamma =			90.0				
	Atom	X	Y	Z	X	Y	Z
1	Co	0.0000	0.0000	0.0000	0.0000	0.0000	0.0000
2	Co	1.7379	1.7379	0.0000	0.2500	0.2500	0.0000
3	Co	0.0000	3.4758	0.0000	0.0000	0.5000	0.0000
4	Co	1.7379	5.2138	0.0000	0.2500	0.7500	0.0000
5	Co	3.4758	0.0000	0.0000	0.5000	0.0000	0.0000
6	Co	5.2138	1.7379	0.0000	0.7500	0.2500	0.0000
7	Co	3.4758	3.4758	0.0000	0.5000	0.5000	0.0000
8	Co	5.2138	5.2138	0.0000	0.7500	0.7500	0.0000
9	Co	-0.0034	6.9471	3.5576	-0.0005	0.9993	0.1621
10	Co	1.7315	1.7402	3.5474	0.2491	0.2503	0.1616
11	Co	-0.0003	3.4803	3.5517	-0.0000	0.5006	0.1618
12	Co	1.7325	5.2089	3.5481	0.2492	0.7493	0.1616
13	Co	5.2203	1.7361	3.5496	0.7509	0.2497	0.1617
14	Co	1.7520	3.4735	5.1942	0.2520	0.4997	0.2366
15	Co	5.2232	5.2170	3.5479	0.7514	0.7505	0.1616
16	Co	3.4641	1.7560	5.1877	0.4983	0.2526	0.2363
17	Ni	1.7379	0.0000	1.7379	0.2500	0.0000	0.0792
18	Ni	0.0000	1.7379	1.7379	0.0000	0.2500	0.0792
19	Ni	1.7379	3.4758	1.7379	0.2500	0.5000	0.0792
20	Ni	0.0000	5.2138	1.7379	0.0000	0.7500	0.0792
21	Ni	5.2138	0.0000	1.7379	0.7500	0.0000	0.0792
22	Ni	3.4758	1.7379	1.7379	0.5000	0.2500	0.0792
23	Ni	5.2138	3.4758	1.7379	0.7500	0.5000	0.0792
24	Ni	3.4758	5.2138	1.7379	0.5000	0.7500	0.0792
25	Ni	1.7568	0.0017	5.2200	0.2527	0.0002	0.2378
26	Ni	0.0048	1.7423	5.2259	0.0007	0.2506	0.2381
27	Ni	3.4773	3.4784	3.5091	0.5002	0.5004	0.1599
28	Ni	0.0011	5.2099	5.2298	0.0002	0.7495	0.2382
29	Ni	5.1940	0.0026	5.2262	0.7472	0.0004	0.2381
30	Ni	5.1996	3.4740	5.2213	0.7480	0.4997	0.2379
31	Ni	3.4717	5.1985	5.2263	0.4994	0.7478	0.2381
32	Ni	3.4760	-0.0017	3.5219	0.5000	-0.0002	0.1604

/db/jmorales/CoNi-alloy/Slabs/Islanding/CoNi(111)/CoNi(111)-3-Co-CO
a = 9.831199646
b = 9.831199646075708
c = 21.0202999115
alpha = 90.0
beta = 90.0
gamma = 120.0

	Atom	X	Y	Z	X	Y	Z
1	Ni	0.0000	0.0000	0.0000	0.0000	0.0000	0.0000
2	Ni	-1.2289	2.1285	0.0000	0.0000	0.2500	0.0000
3	Ni	-2.4578	4.2570	0.0000	0.0000	0.5000	0.0000
4	Ni	-3.6867	6.3856	0.0000	0.0000	0.7500	0.0000
5	Ni	4.9156	0.0000	0.0000	0.5000	0.0000	0.0000
6	Ni	3.6867	2.1285	0.0000	0.5000	0.2500	0.0000
7	Ni	2.4578	4.2570	0.0000	0.5000	0.5000	0.0000
8	Ni	1.2289	6.3856	0.0000	0.5000	0.7500	0.0000
9	Ni	2.4578	1.4190	2.0068	0.3333	0.1667	0.0955
10	Ni	1.2289	3.5475	2.0068	0.3333	0.4167	0.0955
11	Ni	0.0000	5.6760	2.0068	0.3333	0.6667	0.0955
12	Ni	-1.2289	7.8045	2.0068	0.3333	0.9167	0.0955
13	Ni	7.3734	1.4190	2.0068	0.8333	0.1667	0.0955
14	Ni	6.1445	3.5475	2.0068	0.8333	0.4167	0.0955
15	Ni	4.9156	5.6760	2.0068	0.8333	0.6667	0.0955
16	Ni	3.6867	7.8045	2.0068	0.8333	0.9167	0.0955
17	Ni	0.0370	2.8599	4.0085	0.1717	0.3359	0.1907
18	Ni	1.2630	0.7282	4.0041	0.1712	0.0855	0.1905
19	Ni	-2.4268	7.1126	4.0055	0.1708	0.8354	0.1906
20	Ni	-1.2016	4.9829	4.0100	0.1704	0.5853	0.1908
21	Ni	4.9411	2.8581	4.0225	0.6704	0.3357	0.1914
22	Ni	6.1770	0.7259	4.0062	0.6709	0.0853	0.1906
23	Ni	2.4883	7.1115	3.9934	0.6707	0.8353	0.1900
24	Ni	3.7110	4.9793	4.0096	0.6699	0.5848	0.1907
25	Ni	0.0588	0.0350	6.0236	0.0080	0.0041	0.2866
26	Ni	-2.4087	4.2886	6.0237	0.0068	0.5037	0.2866
27	Ni	-3.6330	6.4090	6.0245	0.0068	0.7527	0.2866
28	Ni	4.9680	0.0408	6.0219	0.5077	0.0048	0.2865
29	Ni	1.2898	6.4084	6.0210	0.5075	0.7527	0.2864
30	Ni	2.5224	0.0157	6.0360	0.2575	0.0018	0.2871
31	Ni	3.7464	6.4109	6.0410	0.7576	0.7530	0.2874
32	Ni	7.4297	0.0453	6.0441	0.7584	0.0053	0.2875
33	Co	1.2289	2.1285	0.0000	0.2500	0.2500	0.0000
34	Co	2.4578	0.0000	0.0000	0.2500	0.0000	0.0000
35	Co	-1.2289	6.3856	0.0000	0.2500	0.7500	0.0000
36	Co	0.0000	4.2570	0.0000	0.2500	0.5000	0.0000
37	Co	6.1445	2.1285	0.0000	0.7500	0.2500	0.0000
38	Co	7.3734	0.0000	0.0000	0.7500	0.0000	0.0000
39	Co	3.6867	6.3856	0.0000	0.7500	0.7500	0.0000
40	Co	4.9156	4.2570	0.0000	0.7500	0.5000	0.0000
41	Co	-1.2289	3.5475	2.0068	0.0833	0.4167	0.0955

42	Co	0.0000	1.4190	2.0068	0.0833	0.1667	0.0955
43	Co	-3.6867	7.8045	2.0068	0.0833	0.9167	0.0955
44	Co	-2.4578	5.6760	2.0068	0.0833	0.6667	0.0955
45	Co	3.6867	3.5475	2.0068	0.5833	0.4167	0.0955
46	Co	4.9156	1.4190	2.0068	0.5833	0.1667	0.0955
47	Co	1.2289	7.8045	2.0068	0.5833	0.9167	0.0955
48	Co	2.4578	5.6760	2.0068	0.5833	0.6667	0.0955
49	Co	3.7191	0.7353	4.0412	0.4215	0.0864	0.1923
50	Co	2.4879	2.8561	4.0628	0.4208	0.3355	0.1933
51	Co	1.2581	4.9802	4.0477	0.4204	0.5849	0.1926
52	Co	0.0272	7.1098	4.0553	0.4203	0.8351	0.1929
53	Co	8.6426	0.7341	4.0533	0.9222	0.0862	0.1928
54	Co	7.4059	2.8501	4.0522	0.9207	0.3348	0.1928
55	Co	6.1667	4.9841	4.0511	0.9200	0.5854	0.1927
56	Co	4.9503	7.1158	4.0370	0.9214	0.8358	0.1921
57	Co	1.2813	2.1549	6.0643	0.2569	0.2531	0.2885
58	Co	-1.1489	6.4064	6.0099	0.2594	0.7524	0.2859
59	Co	0.0524	4.3039	6.0033	0.2581	0.5055	0.2856
60	Co	6.2061	2.1720	6.0117	0.7588	0.2551	0.2860
61	Co	4.9983	4.2865	6.0053	0.7601	0.5035	0.2857
62	Co	3.7513	2.1454	6.0377	0.5076	0.2520	0.2872
63	Co	2.5059	4.2955	6.0424	0.5071	0.5045	0.2875
64	Co	-1.1775	2.1588	5.9879	0.0070	0.2536	0.2849
65	O	2.5452	2.8845	8.5905	0.4283	0.3388	0.4087
66	C	2.4858	2.8687	7.3942	0.4213	0.3369	0.3518

/db/jmorales/CoNi-alloy/Slabs/Islanding/CoNi(001)/CoNi(001)-2-Ni-CO

a =		6.9516692132					
b =		6.9516692132					
c =		21.9516692133					
alpha =		90.0					
beta =		90.0					
gamma =		90.0					
	Atom	X	Y	Z	X	Y	Z
1	Ni	0.0000	0.0000	0.0000	0.0000	0.0000	0.0000
2	Ni	0.0000	3.4758	0.0000	0.0000	0.5000	0.0000
3	Ni	3.4758	0.0000	0.0000	0.5000	0.0000	0.0000
4	Ni	3.4758	3.4758	0.0000	0.5000	0.5000	0.0000
5	Ni	0.0000	1.7379	1.7379	0.0000	0.2500	0.0792
6	Ni	0.0000	5.2138	1.7379	0.0000	0.7500	0.0792
7	Ni	3.4758	1.7379	1.7379	0.5000	0.2500	0.0792
8	Ni	3.4758	5.2138	1.7379	0.5000	0.7500	0.0792
9	Ni	6.9511	0.0004	3.4739	0.9999	0.0001	0.1583
10	Ni	-0.0010	3.4759	3.5132	-0.0001	0.5000	0.1600
11	Ni	3.4755	-0.0003	3.5164	0.4999	-0.0000	0.1602
12	Ni	3.4759	3.4761	3.4515	0.5000	0.5000	0.1572
13	Ni	3.4748	1.7457	5.2560	0.4999	0.2511	0.2394
14	Ni	3.4741	5.2060	5.2559	0.4998	0.7489	0.2394
15	Ni	1.7474	3.4752	5.2599	0.2514	0.4999	0.2396
16	Ni	5.2021	3.4765	5.2618	0.7483	0.5001	0.2397
17	Co	1.7379	1.7379	0.0000	0.2500	0.2500	0.0000
18	Co	1.7379	5.2138	0.0000	0.2500	0.7500	0.0000
19	Co	5.2138	1.7379	0.0000	0.7500	0.2500	0.0000
20	Co	5.2138	5.2138	0.0000	0.7500	0.7500	0.0000
21	Co	1.7379	0.0000	1.7379	0.2500	0.0000	0.0792
22	Co	1.7379	3.4758	1.7379	0.2500	0.5000	0.0792
23	Co	5.2138	0.0000	1.7379	0.7500	0.0000	0.0792
24	Co	5.2138	3.4758	1.7379	0.7500	0.5000	0.0792
25	Co	1.7276	1.7260	3.5392	0.2485	0.2483	0.1612
26	Co	1.7274	5.2261	3.5392	0.2485	0.7518	0.1612
27	Co	5.2222	1.7259	3.5405	0.7512	0.2483	0.1613
28	Co	5.2227	5.2261	3.5399	0.7513	0.7518	0.1613
29	Co	1.6979	0.0011	5.1978	0.2442	0.0002	0.2368
30	Co	5.2513	-0.0002	5.1980	0.7554	-0.0000	0.2368
31	Co	-0.0015	1.7006	5.1867	-0.0002	0.2446	0.2363
32	Co	-0.0001	5.2516	5.1862	-0.0000	0.7554	0.2363
33	O	3.4750	3.4780	7.5603	0.4999	0.5003	0.3444
34	C	3.4755	3.4759	6.3465	0.5000	0.5000	0.2891

/db/jmorales/CoNi-alloy/Slabs/Islanding/CoNi(001)/CoNi(001)-1-Ni-CO

a =		6.9516692132					
b =		6.9516692132					
c =		21.9516692133					
alpha =		90.0					
beta =		90.0					
gamma =		90.0					
	Atom	X	Y	Z	X	Y	Z
1	Ni	0.0000	0.0000	0.0000	0.0000	0.0000	0.0000
2	Ni	0.0000	3.4758	0.0000	0.0000	0.5000	0.0000
3	Ni	3.4758	0.0000	0.0000	0.5000	0.0000	0.0000
4	Ni	3.4758	3.4758	0.0000	0.5000	0.5000	0.0000
5	Ni	0.0000	1.7379	1.7379	0.0000	0.2500	0.0792
6	Ni	0.0000	5.2138	1.7379	0.0000	0.7500	0.0792
7	Ni	3.4758	1.7379	1.7379	0.5000	0.2500	0.0792
8	Ni	3.4758	5.2138	1.7379	0.5000	0.7500	0.0792
9	Ni	6.9443	-0.0116	3.4920	0.9989	-0.0017	0.1591
10	Ni	0.0067	3.4814	3.5027	0.0010	0.5008	0.1596
11	Ni	3.4813	0.0040	3.5186	0.5008	0.0006	0.1603
12	Ni	3.4746	3.4746	3.4508	0.4998	0.4998	0.1572
13	Ni	-0.0007	5.2251	5.2080	-0.0001	0.7516	0.2372
14	Ni	3.4772	1.7498	5.2432	0.5002	0.2517	0.2389
15	Ni	3.4724	5.1996	5.2478	0.4995	0.7480	0.2391
16	Ni	1.7526	3.4697	5.2743	0.2521	0.4991	0.2403
17	Co	1.7379	1.7379	0.0000	0.2500	0.2500	0.0000
18	Co	1.7379	5.2138	0.0000	0.2500	0.7500	0.0000
19	Co	5.2138	1.7379	0.0000	0.7500	0.2500	0.0000
20	Co	5.2138	5.2138	0.0000	0.7500	0.7500	0.0000
21	Co	1.7379	0.0000	1.7379	0.2500	0.0000	0.0792
22	Co	1.7379	3.4758	1.7379	0.2500	0.5000	0.0792
23	Co	5.2138	0.0000	1.7379	0.7500	0.0000	0.0792
24	Co	5.2138	3.4758	1.7379	0.7500	0.5000	0.0792
25	Co	1.7246	1.7204	3.5366	0.2481	0.2475	0.1611
26	Co	1.7361	5.2332	3.5297	0.2497	0.7528	0.1608
27	Co	5.2195	1.7413	3.5592	0.7508	0.2505	0.1621
28	Co	5.2132	5.2101	3.5571	0.7499	0.7495	0.1620
29	Co	1.7185	0.0063	5.2078	0.2472	0.0009	0.2372
30	Co	5.2353	0.0064	5.2080	0.7531	0.0009	0.2372
31	Co	5.2128	3.4760	5.2310	0.7499	0.5000	0.2383
32	Co	6.9457	1.7183	5.1829	0.9991	0.2472	0.2361
33	O	3.4787	3.4768	7.5668	0.5004	0.5001	0.3447
34	C	3.4469	3.4861	6.3530	0.4958	0.5015	0.2894

/db/jmorales/CoNi-alloy/Slabs/Co-CoNi(001)-expanded

a =			7.044921511				
b =			7.044921511				
c =			22.044921511				
alpha =			90.0				
beta =			90.0				
gamma =			90.0				
	Atom	X	Y	Z	X	Y	Z
1	Ni	0.0000	0.0000	0.0000	0.0000	0.0000	0.0000
2	Ni	1.7612	1.7612	0.0000	0.2500	0.2500	0.0000
3	Ni	0.0000	0.0000	3.5225	0.0000	0.0000	0.1598
4	Ni	1.7612	1.7612	3.5225	0.2500	0.2500	0.1598
5	Ni	0.0000	3.5225	0.0000	0.0000	0.5000	0.0000
6	Ni	1.7612	5.2837	0.0000	0.2500	0.7500	0.0000
7	Ni	0.0000	3.5225	3.5225	0.0000	0.5000	0.1598
8	Ni	1.7612	5.2837	3.5225	0.2500	0.7500	0.1598
9	Ni	3.5225	0.0000	0.0000	0.5000	0.0000	0.0000
10	Ni	5.2837	1.7612	0.0000	0.7500	0.2500	0.0000
11	Ni	3.5225	0.0000	3.5225	0.5000	0.0000	0.1598
12	Ni	5.2837	1.7612	3.5225	0.7500	0.2500	0.1598
13	Ni	3.5225	3.5225	0.0000	0.5000	0.5000	0.0000
14	Ni	5.2837	5.2837	0.0000	0.7500	0.7500	0.0000
15	Ni	3.5225	3.5225	3.5225	0.5000	0.5000	0.1598
16	Ni	5.2837	5.2837	3.5225	0.7500	0.7500	0.1598
17	Co	0.0000	1.7612	1.7612	0.0000	0.2500	0.0799
18	Co	1.7612	0.0000	1.7612	0.2500	0.0000	0.0799
19	Co	0.0000	1.7612	5.2837	0.0000	0.2500	0.2397
20	Co	1.7612	0.0000	5.2837	0.2500	0.0000	0.2397
21	Co	0.0000	5.2837	1.7612	0.0000	0.7500	0.0799
22	Co	1.7612	3.5225	1.7612	0.2500	0.5000	0.0799
23	Co	0.0000	5.2837	5.2837	0.0000	0.7500	0.2397
24	Co	1.7612	3.5225	5.2837	0.2500	0.5000	0.2397
25	Co	3.5225	1.7612	1.7612	0.5000	0.2500	0.0799
26	Co	5.2837	0.0000	1.7612	0.7500	0.0000	0.0799
27	Co	3.5225	1.7612	5.2837	0.5000	0.2500	0.2397
28	Co	5.2837	0.0000	5.2837	0.7500	0.0000	0.2397
29	Co	3.5225	5.2837	1.7612	0.5000	0.7500	0.0799
30	Co	5.2837	3.5225	1.7612	0.7500	0.5000	0.0799
31	Co	3.5225	5.2837	5.2837	0.5000	0.7500	0.2397
32	Co	5.2837	3.5225	5.2837	0.7500	0.5000	0.2397

/db/jmorales/CoNi-alloy/Slabs/Islanding/CoNi(001)/CoNi(001)-1-Ni

a =			6.9516692132				
b =			6.9516692132				
c =			21.9516692133				
alpha =			90.0				
beta =			90.0				
gamma =			90.0				
	Atom	X	Y	Z	X	Y	Z
1	Ni	0.0000	0.0000	0.0000	0.0000	0.0000	0.0000
2	Ni	0.0000	3.4758	0.0000	0.0000	0.5000	0.0000
3	Ni	3.4758	0.0000	0.0000	0.5000	0.0000	0.0000
4	Ni	3.4758	3.4758	0.0000	0.5000	0.5000	0.0000
5	Ni	0.0000	1.7379	1.7379	0.0000	0.2500	0.0792
6	Ni	0.0000	5.2138	1.7379	0.0000	0.7500	0.0792
7	Ni	3.4758	1.7379	1.7379	0.5000	0.2500	0.0792
8	Ni	3.4758	5.2138	1.7379	0.5000	0.7500	0.0792
9	Ni	-0.0020	-0.0075	3.4949	-0.0003	-0.0011	0.1592
10	Ni	6.9502	3.4818	3.5009	0.9998	0.5009	0.1595
11	Ni	3.4768	-0.0010	3.5034	0.5001	-0.0001	0.1596
12	Ni	3.4755	3.4760	3.5044	0.5000	0.5000	0.1596
13	Ni	6.9447	5.2279	5.2118	0.9990	0.7520	0.2374
14	Ni	3.4796	1.7277	5.2119	0.5005	0.2485	0.2374
15	Ni	3.4752	5.2217	5.2137	0.4999	0.7512	0.2375
16	Ni	1.7230	3.4729	5.2291	0.2479	0.4996	0.2382
17	Co	1.7379	1.7379	0.0000	0.2500	0.2500	0.0000
18	Co	1.7379	5.2138	0.0000	0.2500	0.7500	0.0000
19	Co	5.2138	1.7379	0.0000	0.7500	0.2500	0.0000
20	Co	5.2138	5.2138	0.0000	0.7500	0.7500	0.0000
21	Co	1.7379	0.0000	1.7379	0.2500	0.0000	0.0792
22	Co	1.7379	3.4758	1.7379	0.2500	0.5000	0.0792
23	Co	5.2138	0.0000	1.7379	0.7500	0.0000	0.0792
24	Co	5.2138	3.4758	1.7379	0.7500	0.5000	0.0792
25	Co	1.7304	1.7281	3.5491	0.2489	0.2486	0.1617
26	Co	1.7375	5.2253	3.5388	0.2499	0.7517	0.1612
27	Co	5.2184	1.7389	3.5575	0.7507	0.2501	0.1621
28	Co	5.2114	5.2144	3.5506	0.7497	0.7501	0.1617
29	Co	1.7251	0.0073	5.2143	0.2482	0.0011	0.2375
30	Co	5.2279	0.0091	5.2142	0.7520	0.0013	0.2375
31	Co	5.2223	3.4747	5.2131	0.7512	0.4998	0.2375
32	Co	6.9432	1.7241	5.1937	0.9988	0.2480	0.2366

/db/jmorales/CoNi-alloy/Slabs/Segregation/CoNi(111)/CoNi(111)-2-Ni-CO

a =	9.831199646
b =	9.831199646075708
c =	21.0202999115
alpha =	90.0
beta =	90.0
gamma =	120.0

	Atom	X	Y	Z	X	Y	Z
1	Ni	0.0000	0.0000	0.0000	0.0000	0.0000	0.0000
2	Ni	-1.2289	2.1285	0.0000	0.0000	0.2500	0.0000
3	Ni	-2.4578	4.2570	0.0000	0.0000	0.5000	0.0000
4	Ni	-3.6867	6.3856	0.0000	0.0000	0.7500	0.0000
5	Ni	4.9156	0.0000	0.0000	0.5000	0.0000	0.0000
6	Ni	3.6867	2.1285	0.0000	0.5000	0.2500	0.0000
7	Ni	2.4578	4.2570	0.0000	0.5000	0.5000	0.0000
8	Ni	1.2289	6.3856	0.0000	0.5000	0.7500	0.0000
9	Ni	2.4578	1.4190	2.0068	0.3333	0.1667	0.0955
10	Ni	1.2289	3.5475	2.0068	0.3333	0.4167	0.0955
11	Ni	0.0000	5.6760	2.0068	0.3333	0.6667	0.0955
12	Ni	-1.2289	7.8045	2.0068	0.3333	0.9167	0.0955
13	Ni	7.3734	1.4190	2.0068	0.8333	0.1667	0.0955
14	Ni	6.1445	3.5475	2.0068	0.8333	0.4167	0.0955
15	Ni	4.9156	5.6760	2.0068	0.8333	0.6667	0.0955
16	Ni	3.6867	7.8045	2.0068	0.8333	0.9167	0.0955
17	Ni	0.0366	2.8570	4.0086	0.1715	0.3356	0.1907
18	Ni	-1.2000	4.9779	4.0107	0.1703	0.5847	0.1908
19	Ni	4.9438	2.8553	4.0206	0.6705	0.3354	0.1913
20	Ni	6.1724	0.7286	4.0084	0.6706	0.0856	0.1907
21	Ni	2.4875	7.1092	4.0085	0.6705	0.8350	0.1907
22	Ni	3.7115	4.9814	4.0186	0.6701	0.5851	0.1912
23	Ni	0.0434	0.0269	6.0316	0.0060	0.0032	0.2869
24	Ni	-1.1859	2.1563	6.0266	0.0060	0.2533	0.2867
25	Ni	-2.4164	4.2782	6.0234	0.0055	0.5025	0.2865
26	Ni	-3.6406	6.4068	6.0392	0.0059	0.7525	0.2873
27	Ni	4.9668	0.0302	6.0184	0.5070	0.0035	0.2863
28	Ni	3.7487	2.1665	6.0744	0.5085	0.2545	0.2890
29	Ni	2.5180	4.2948	6.0852	0.5083	0.5044	0.2895
30	Ni	1.2849	6.4183	6.0215	0.5076	0.7538	0.2865
31	Ni	1.2703	2.1677	6.0875	0.2565	0.2546	0.2896
32	Ni	2.5145	0.0014	6.0152	0.2558	0.0002	0.2862
33	Co	1.2289	2.1285	0.0000	0.2500	0.2500	0.0000
34	Co	2.4578	0.0000	0.0000	0.2500	0.0000	0.0000
35	Co	-1.2289	6.3856	0.0000	0.2500	0.7500	0.0000
36	Co	0.0000	4.2570	0.0000	0.2500	0.5000	0.0000
37	Co	6.1445	2.1285	0.0000	0.7500	0.2500	0.0000
38	Co	7.3734	0.0000	0.0000	0.7500	0.0000	0.0000
39	Co	3.6867	6.3856	0.0000	0.7500	0.7500	0.0000
40	Co	4.9156	4.2570	0.0000	0.7500	0.5000	0.0000
41	Co	-1.2289	3.5475	2.0068	0.0833	0.4167	0.0955

42	Co	0.0000	1.4190	2.0068	0.0833	0.1667	0.0955
43	Co	-3.6867	7.8045	2.0068	0.0833	0.9167	0.0955
44	Co	-2.4578	5.6760	2.0068	0.0833	0.6667	0.0955
45	Co	3.6867	3.5475	2.0068	0.5833	0.4167	0.0955
46	Co	4.9156	1.4190	2.0068	0.5833	0.1667	0.0955
47	Co	1.2289	7.8045	2.0068	0.5833	0.9167	0.0955
48	Co	2.4578	5.6760	2.0068	0.5833	0.6667	0.0955
49	Co	3.7039	0.7281	4.0334	0.4195	0.0855	0.1919
50	Co	2.4892	2.8484	4.0050	0.4205	0.3346	0.1905
51	Co	1.2583	4.9736	4.0555	0.4201	0.5842	0.1929
52	Co	0.0193	7.1131	4.0506	0.4197	0.8354	0.1927
53	Co	8.6410	0.7317	4.0503	0.9219	0.0859	0.1927
54	Co	7.4040	2.8533	4.0494	0.9207	0.3351	0.1926
55	Co	6.1766	4.9911	4.0484	0.9214	0.5862	0.1926
56	Co	4.9553	7.1179	4.0589	0.9220	0.8360	0.1931
57	Co	-1.1671	6.4110	5.9950	0.2578	0.7530	0.2852
58	Co	0.0318	4.3268	5.9987	0.2573	0.5082	0.2854
59	Co	6.2156	2.1614	6.0065	0.7592	0.2539	0.2857
60	Co	7.4351	0.0373	6.0082	0.7585	0.0044	0.2858
61	Co	3.7455	6.4261	6.0080	0.7584	0.7548	0.2858
62	Co	5.0019	4.3097	6.0007	0.7619	0.5062	0.2855
63	Co	1.2482	0.7240	3.9896	0.1695	0.0850	0.1898
64	Co	-2.4248	7.1027	4.0168	0.1705	0.8342	0.1911
65	O	2.5002	2.8495	8.6123	0.4217	0.3347	0.4097
66	C	2.4868	2.8524	7.4190	0.4205	0.3350	0.3529

/db/jmorales/CoNi-alloy/Slabs/Segregation/Ni-CoNi(001)/Ni-CoNi(001)-4-Co-CO

a =	6.9516692132
b =	6.9516692132
c =	21.9516692133
alpha =	90.0
beta =	90.0
gamma =	90.0

	Atom	X	Y	Z	X	Y	Z
1	Co	0.0000	0.0000	0.0000	0.0000	0.0000	0.0000
2	Co	1.7379	1.7379	0.0000	0.2500	0.2500	0.0000
3	Co	0.0000	3.4758	0.0000	0.0000	0.5000	0.0000
4	Co	1.7379	5.2138	0.0000	0.2500	0.7500	0.0000
5	Co	3.4758	0.0000	0.0000	0.5000	0.0000	0.0000
6	Co	5.2138	1.7379	0.0000	0.7500	0.2500	0.0000
7	Co	3.4758	3.4758	0.0000	0.5000	0.5000	0.0000
8	Co	5.2138	5.2138	0.0000	0.7500	0.7500	0.0000
9	Co	0.0134	0.0002	3.5594	0.0019	0.0000	0.1621
10	Co	1.7304	1.7324	3.5603	0.2489	0.2492	0.1622
11	Co	0.0137	3.4748	3.5600	0.0020	0.4999	0.1622
12	Co	1.7307	5.2180	3.5602	0.2490	0.7506	0.1622
13	Co	1.7545	3.4752	5.2345	0.2524	0.4999	0.2385
14	Co	3.4825	1.7844	5.2512	0.5010	0.2567	0.2392
15	Co	5.1777	3.4763	5.2350	0.7448	0.5001	0.2385
16	Co	3.4819	5.1664	5.2531	0.5009	0.7432	0.2393
17	Ni	1.7379	0.0000	1.7379	0.2500	0.0000	0.0792
18	Ni	0.0000	1.7379	1.7379	0.0000	0.2500	0.0792
19	Ni	1.7379	3.4758	1.7379	0.2500	0.5000	0.0792
20	Ni	0.0000	5.2138	1.7379	0.0000	0.7500	0.0792
21	Ni	5.2138	0.0000	1.7379	0.7500	0.0000	0.0792
22	Ni	3.4758	1.7379	1.7379	0.5000	0.2500	0.0792
23	Ni	5.2138	3.4758	1.7379	0.7500	0.5000	0.0792
24	Ni	3.4758	5.2138	1.7379	0.5000	0.7500	0.0792
25	Ni	1.7625	-0.0008	5.2366	0.2535	-0.0001	0.2386
26	Ni	6.9392	1.7500	5.2337	0.9982	0.2517	0.2384
27	Ni	3.4657	3.4759	3.4896	0.4985	0.5000	0.1590
28	Ni	6.9395	5.2014	5.2339	0.9982	0.7482	0.2384
29	Ni	5.1965	-0.0010	5.2244	0.7475	-0.0002	0.2380
30	Ni	3.4727	-0.0003	3.5396	0.4996	-0.0000	0.1612
31	Ni	5.2147	1.7364	3.5331	0.7501	0.2498	0.1609
32	Ni	5.2138	5.2140	3.5326	0.7500	0.7500	0.1609
33	O	3.4557	3.4724	7.6111	0.4971	0.4995	0.3467
34	C	3.4726	3.4746	6.3952	0.4995	0.4998	0.2913

/db/jmorales/CoNi-alloy/Slabs/Ni(111)-expanded

a =			9.967200279				
b =			9.967200279058309				
c =			21.103700638				
alpha =			90.0				
beta =			90.0				
gamma =			120.0				
	Atom	X	Y	Z	X	Y	Z
1	Ni	0.0000	0.0000	0.0000	0.0000	0.0000	0.0000
2	Ni	-1.2459	2.1580	0.0000	-0.0000	0.2500	0.0000
3	Ni	-2.4918	4.3159	0.0000	-0.0000	0.5000	0.0000
4	Ni	-3.7377	6.4739	0.0000	0.0000	0.7500	0.0000
5	Ni	2.4918	0.0000	0.0000	0.2500	0.0000	0.0000
6	Ni	1.2459	2.1580	0.0000	0.2500	0.2500	0.0000
7	Ni	0.0000	4.3159	0.0000	0.2500	0.5000	0.0000
8	Ni	-1.2459	6.4739	0.0000	0.2500	0.7500	0.0000
9	Ni	4.9836	0.0000	0.0000	0.5000	0.0000	0.0000
10	Ni	3.7377	2.1580	0.0000	0.5000	0.2500	0.0000
11	Ni	2.4918	4.3159	0.0000	0.5000	0.5000	0.0000
12	Ni	1.2459	6.4739	0.0000	0.5000	0.7500	0.0000
13	Ni	7.4754	0.0000	0.0000	0.7500	0.0000	0.0000
14	Ni	6.2295	2.1580	0.0000	0.7500	0.2500	0.0000
15	Ni	4.9836	4.3159	0.0000	0.7500	0.5000	0.0000
16	Ni	3.7377	6.4739	0.0000	0.7500	0.7500	0.0000
17	Ni	-0.0000	1.4386	2.0346	0.0833	0.1667	0.0964
18	Ni	-1.2459	3.5966	2.0346	0.0833	0.4167	0.0964
19	Ni	-2.4918	5.7546	2.0346	0.0833	0.6667	0.0964
20	Ni	-3.7377	7.9125	2.0346	0.0833	0.9167	0.0964
21	Ni	2.4918	1.4386	2.0346	0.3333	0.1667	0.0964
22	Ni	1.2459	3.5966	2.0346	0.3333	0.4167	0.0964
23	Ni	-0.0000	5.7546	2.0346	0.3333	0.6667	0.0964
24	Ni	-1.2459	7.9125	2.0346	0.3333	0.9167	0.0964
25	Ni	4.9836	1.4386	2.0346	0.5833	0.1667	0.0964
26	Ni	3.7377	3.5966	2.0346	0.5833	0.4167	0.0964
27	Ni	2.4918	5.7546	2.0346	0.5833	0.6667	0.0964
28	Ni	1.2459	7.9125	2.0346	0.5833	0.9167	0.0964
29	Ni	7.4754	1.4386	2.0346	0.8333	0.1667	0.0964
30	Ni	6.2295	3.5966	2.0346	0.8333	0.4167	0.0964
31	Ni	4.9836	5.7546	2.0346	0.8333	0.6667	0.0964
32	Ni	3.7377	7.9125	2.0346	0.8333	0.9167	0.0964
33	Ni	1.2459	0.7193	4.0692	0.1667	0.0833	0.1928
34	Ni	0.0000	2.8773	4.0692	0.1667	0.3333	0.1928
35	Ni	-1.2459	5.0352	4.0692	0.1667	0.5833	0.1928
36	Ni	-2.4918	7.1932	4.0692	0.1667	0.8333	0.1928
37	Ni	3.7377	0.7193	4.0692	0.4167	0.0833	0.1928
38	Ni	2.4918	2.8773	4.0692	0.4167	0.3333	0.1928
39	Ni	1.2459	5.0352	4.0692	0.4167	0.5833	0.1928
40	Ni	0.0000	7.1932	4.0692	0.4167	0.8333	0.1928
41	Ni	6.2295	0.7193	4.0692	0.6667	0.0833	0.1928

42	Ni	4.9836	2.8773	4.0692	0.6667	0.3333	0.1928
43	Ni	3.7377	5.0352	4.0692	0.6667	0.5833	0.1928
44	Ni	2.4918	7.1932	4.0692	0.6667	0.8333	0.1928
45	Ni	8.7213	0.7193	4.0692	0.9167	0.0833	0.1928
46	Ni	7.4754	2.8773	4.0692	0.9167	0.3333	0.1928
47	Ni	6.2295	5.0352	4.0692	0.9167	0.5833	0.1928
48	Ni	4.9836	7.1932	4.0692	0.9167	0.8333	0.1928
49	Ni	0.0000	0.0000	6.1036	0.0000	0.0000	0.2892
50	Ni	-1.2459	2.1580	6.1036	-0.0000	0.2500	0.2892
51	Ni	-2.4918	4.3159	6.1036	-0.0000	0.5000	0.2892
52	Ni	-3.7377	6.4739	6.1036	0.0000	0.7500	0.2892
53	Ni	2.4918	0.0000	6.1036	0.2500	0.0000	0.2892
54	Ni	1.2459	2.1580	6.1036	0.2500	0.2500	0.2892
55	Ni	0.0000	4.3159	6.1036	0.2500	0.5000	0.2892
56	Ni	-1.2459	6.4739	6.1036	0.2500	0.7500	0.2892
57	Ni	4.9836	0.0000	6.1036	0.5000	0.0000	0.2892
58	Ni	3.7377	2.1580	6.1036	0.5000	0.2500	0.2892
59	Ni	2.4918	4.3159	6.1036	0.5000	0.5000	0.2892
60	Ni	1.2459	6.4739	6.1036	0.5000	0.7500	0.2892
61	Ni	7.4754	0.0000	6.1036	0.7500	0.0000	0.2892
62	Ni	6.2295	2.1580	6.1036	0.7500	0.2500	0.2892
63	Ni	4.9836	4.3159	6.1036	0.7500	0.5000	0.2892
64	Ni	3.7377	6.4739	6.1036	0.7500	0.7500	0.2892

/db/jmorales/CoNi-alloy/Slabs/Islanding/CoNi(001)/CoNi(001)-2-Co

a =			6.9516692132				
b =			6.9516692132				
c =			21.9516692133				
alpha =			90.0				
beta =			90.0				
gamma =			90.0				
	Atom	X	Y	Z	X	Y	Z
1	Ni	0.0000	0.0000	0.0000	0.0000	0.0000	0.0000
2	Ni	0.0000	3.4758	0.0000	0.0000	0.5000	0.0000
3	Ni	3.4758	0.0000	0.0000	0.5000	0.0000	0.0000
4	Ni	3.4758	3.4758	0.0000	0.5000	0.5000	0.0000
5	Ni	0.0000	1.7379	1.7379	0.0000	0.2500	0.0792
6	Ni	0.0000	5.2138	1.7379	0.0000	0.7500	0.0792
7	Ni	3.4758	1.7379	1.7379	0.5000	0.2500	0.0792
8	Ni	3.4758	5.2138	1.7379	0.5000	0.7500	0.0792
9	Ni	-0.0000	-0.0003	3.5045	-0.0000	-0.0000	0.1596
10	Ni	0.0002	3.4765	3.5032	0.0000	0.5001	0.1596
11	Ni	3.4755	-0.0009	3.5022	0.5000	-0.0001	0.1595
12	Ni	3.4757	3.4750	3.4792	0.5000	0.4999	0.1585
13	Ni	0.0000	1.7542	5.2201	0.0000	0.2523	0.2378
14	Ni	-0.0007	5.1938	5.2218	-0.0001	0.7471	0.2379
15	Ni	1.7546	-0.0004	5.2205	0.2524	-0.0001	0.2378
16	Ni	5.1963	-0.0002	5.2212	0.7475	-0.0000	0.2379
17	Co	1.7379	1.7379	0.0000	0.2500	0.2500	0.0000
18	Co	1.7379	5.2138	0.0000	0.2500	0.7500	0.0000
19	Co	5.2138	1.7379	0.0000	0.7500	0.2500	0.0000
20	Co	5.2138	5.2138	0.0000	0.7500	0.7500	0.0000
21	Co	1.7379	0.0000	1.7379	0.2500	0.0000	0.0792
22	Co	1.7379	3.4758	1.7379	0.2500	0.5000	0.0792
23	Co	5.2138	0.0000	1.7379	0.7500	0.0000	0.0792
24	Co	5.2138	3.4758	1.7379	0.7500	0.5000	0.0792
25	Co	1.7438	1.7462	3.5460	0.2508	0.2512	0.1615
26	Co	1.7429	5.2037	3.5478	0.2507	0.7485	0.1616
27	Co	5.2079	1.7457	3.5467	0.7492	0.2511	0.1616
28	Co	5.2080	5.2037	3.5486	0.7492	0.7486	0.1617
29	Co	1.7696	3.4736	5.2042	0.2546	0.4997	0.2371
30	Co	5.1818	3.4732	5.2040	0.7454	0.4996	0.2371
31	Co	3.4753	1.7655	5.1942	0.4999	0.2540	0.2366
32	Co	3.4763	5.1845	5.1930	0.5001	0.7458	0.2366

/db/jmorales/CoNi-alloy/Slabs/Ni(001)-expanded

a =	7.047964809
b =	7.047964809
c =	22.047964809
alpha =	90.0
beta =	90.0
gamma =	90.0

	Atom	X	Y	Z	X	Y	Z
1	Ni	0.0000	0.0000	0.0000	0.0000	0.0000	0.0000
2	Ni	1.7620	1.7620	0.0000	0.2500	0.2500	0.0000
3	Ni	0.0000	3.5240	0.0000	0.0000	0.5000	0.0000
4	Ni	1.7620	5.2860	0.0000	0.2500	0.7500	0.0000
5	Ni	3.5240	0.0000	0.0000	0.5000	0.0000	0.0000
6	Ni	5.2860	1.7620	0.0000	0.7500	0.2500	0.0000
7	Ni	3.5240	3.5240	0.0000	0.5000	0.5000	0.0000
8	Ni	5.2860	5.2860	0.0000	0.7500	0.7500	0.0000
9	Ni	0.0000	1.7620	1.7620	0.0000	0.2500	0.0799
10	Ni	1.7620	0.0000	1.7620	0.2500	0.0000	0.0799
11	Ni	0.0000	5.2860	1.7620	0.0000	0.7500	0.0799
12	Ni	1.7620	3.5240	1.7620	0.2500	0.5000	0.0799
13	Ni	3.5240	1.7620	1.7620	0.5000	0.2500	0.0799
14	Ni	5.2860	0.0000	1.7620	0.7500	0.0000	0.0799
15	Ni	3.5240	5.2860	1.7620	0.5000	0.7500	0.0799
16	Ni	5.2860	3.5240	1.7620	0.7500	0.5000	0.0799
17	Ni	0.0000	0.0000	3.5240	0.0000	0.0000	0.1598
18	Ni	1.7620	1.7620	3.5240	0.2500	0.2500	0.1598
19	Ni	0.0000	3.5240	3.5240	0.0000	0.5000	0.1598
20	Ni	1.7620	5.2860	3.5240	0.2500	0.7500	0.1598
21	Ni	3.5240	0.0000	3.5240	0.5000	0.0000	0.1598
22	Ni	5.2860	1.7620	3.5240	0.7500	0.2500	0.1598
23	Ni	3.5240	3.5240	3.5240	0.5000	0.5000	0.1598
24	Ni	5.2860	5.2860	3.5240	0.7500	0.7500	0.1598
25	Ni	0.0000	1.7620	5.2860	0.0000	0.2500	0.2397
26	Ni	1.7620	0.0000	5.2860	0.2500	0.0000	0.2397
27	Ni	0.0000	5.2860	5.2860	0.0000	0.7500	0.2397
28	Ni	1.7620	3.5240	5.2860	0.2500	0.5000	0.2397
29	Ni	3.5240	1.7620	5.2860	0.5000	0.2500	0.2397
30	Ni	5.2860	0.0000	5.2860	0.7500	0.0000	0.2397
31	Ni	3.5240	5.2860	5.2860	0.5000	0.7500	0.2397
32	Ni	5.2860	3.5240	5.2860	0.7500	0.5000	0.2397

/db/jmorales/CoNi-alloy/Slabs/Ni-CoNi(001)

a =	6.951669213
b =	6.951669213
c =	21.951669213
alpha =	90.0
beta =	90.0
gamma =	90.0

	Atom	X	Y	Z	X	Y	Z
1	Co	0.0000	0.0000	0.0000	0.0000	0.0000	0.0000
2	Co	1.7379	1.7379	0.0000	0.2500	0.2500	0.0000
3	Co	0.0000	3.4758	0.0000	0.0000	0.5000	0.0000
4	Co	1.7379	5.2138	0.0000	0.2500	0.7500	0.0000
5	Co	3.4758	0.0000	0.0000	0.5000	0.0000	0.0000
6	Co	5.2138	1.7379	0.0000	0.7500	0.2500	0.0000
7	Co	3.4758	3.4758	0.0000	0.5000	0.5000	0.0000
8	Co	5.2138	5.2138	0.0000	0.7500	0.7500	0.0000
9	Co	0.0000	0.0000	3.5200	0.0000	0.0000	0.1604
10	Co	1.7379	1.7379	3.4758	0.2500	0.2500	0.1583
11	Co	0.0000	3.4758	3.5200	0.0000	0.5000	0.1604
12	Co	1.7379	5.2138	3.4758	0.2500	0.7500	0.1583
13	Co	3.4758	0.0000	3.5200	0.5000	0.0000	0.1604
14	Co	5.2138	1.7379	3.4758	0.7500	0.2500	0.1583
15	Co	3.4758	3.4758	3.5200	0.5000	0.5000	0.1604
16	Co	5.2138	5.2138	3.4758	0.7500	0.7500	0.1583
17	Ni	1.7379	0.0000	1.7379	0.2500	0.0000	0.0792
18	Ni	0.0000	1.7379	1.7379	0.0000	0.2500	0.0792
19	Ni	1.7379	3.4758	1.7379	0.2500	0.5000	0.0792
20	Ni	0.0000	5.2138	1.7379	0.0000	0.7500	0.0792
21	Ni	5.2138	0.0000	1.7379	0.7500	0.0000	0.0792
22	Ni	3.4758	1.7379	1.7379	0.5000	0.2500	0.0792
23	Ni	5.2138	3.4758	1.7379	0.7500	0.5000	0.0792
24	Ni	3.4758	5.2138	1.7379	0.5000	0.7500	0.0792
25	Ni	1.7379	0.0000	5.1728	0.2500	0.0000	0.2356
26	Ni	0.0000	1.7379	5.1728	0.0000	0.2500	0.2356
27	Ni	1.7379	3.4758	5.1728	0.2500	0.5000	0.2356
28	Ni	0.0000	5.2138	5.1728	0.0000	0.7500	0.2356
29	Ni	5.2138	0.0000	5.1728	0.7500	0.0000	0.2356
30	Ni	3.4758	1.7379	5.1728	0.5000	0.2500	0.2356
31	Ni	5.2138	3.4758	5.1728	0.7500	0.5000	0.2356
32	Ni	3.4758	5.2138	5.1728	0.5000	0.7500	0.2356

/db/jmorales/CoNi-alloy/Slabs/Segregation/Co-CoNi(001)/Co-CoNi(001)-4-Ni-CO

a = 6.9516692132
b = 6.9516692132
c = 21.9516692133
alpha = 90.0
beta = 90.0
gamma = 90.0

	Atom	X	Y	Z	X	Y	Z
1	Ni	0.0000	0.0000	0.0000	0.0000	0.0000	0.0000
2	Ni	1.7379	1.7379	0.0000	0.2500	0.2500	0.0000
3	Ni	0.0000	3.4758	0.0000	0.0000	0.5000	0.0000
4	Ni	1.7379	5.2138	0.0000	0.2500	0.7500	0.0000
5	Ni	3.4758	0.0000	0.0000	0.5000	0.0000	0.0000
6	Ni	5.2138	1.7379	0.0000	0.7500	0.2500	0.0000
7	Ni	3.4758	3.4758	0.0000	0.5000	0.5000	0.0000
8	Ni	5.2138	5.2138	0.0000	0.7500	0.7500	0.0000
9	Ni	0.0003	-0.0013	3.5021	0.0000	-0.0002	0.1595
10	Ni	1.7287	1.7317	3.5064	0.2487	0.2491	0.1597
11	Ni	0.0022	3.4753	3.5278	0.0003	0.4999	0.1607
12	Ni	1.7282	5.2184	3.5069	0.2486	0.7507	0.1598
13	Ni	1.7462	3.4752	5.2761	0.2512	0.4999	0.2404
14	Ni	3.4740	1.7399	5.2676	0.4997	0.2503	0.2400
15	Ni	5.2025	3.4749	5.2713	0.7484	0.4999	0.2401
16	Ni	3.4744	5.2100	5.2680	0.4998	0.7495	0.2400
17	Co	1.7379	0.0000	1.7379	0.2500	0.0000	0.0792
18	Co	0.0000	1.7379	1.7379	0.0000	0.2500	0.0792
19	Co	1.7379	3.4758	1.7379	0.2500	0.5000	0.0792
20	Co	0.0000	5.2138	1.7379	0.0000	0.7500	0.0792
21	Co	5.2138	0.0000	1.7379	0.7500	0.0000	0.0792
22	Co	3.4758	1.7379	1.7379	0.5000	0.2500	0.0792
23	Co	5.2138	3.4758	1.7379	0.7500	0.5000	0.0792
24	Co	3.4758	5.2138	1.7379	0.5000	0.7500	0.0792
25	Co	1.7192	-0.0013	5.2158	0.2473	-0.0002	0.2376
26	Co	6.9422	1.6964	5.2224	0.9986	0.2440	0.2379
27	Co	3.4817	3.4745	3.4495	0.5008	0.4998	0.1571
28	Co	-0.0091	5.2543	5.2238	-0.0013	0.7558	0.2380
29	Co	5.2338	-0.0008	5.1975	0.7529	-0.0001	0.2368
30	Co	3.4733	-0.0012	3.5180	0.4996	-0.0002	0.1603
31	Co	5.2278	1.7283	3.5130	0.7520	0.2486	0.1600
32	Co	5.2281	5.2216	3.5128	0.7521	0.7511	0.1600
33	O	3.4737	3.4728	7.5508	0.4997	0.4996	0.3440
34	C	3.4625	3.4751	6.3347	0.4981	0.4999	0.2886

/db/jmorales/CoNi-alloy/Slabs/Islanding/CoNi(111)/CoNi(111)-3-Co

a = 9.831199646
b = 9.831199646075708
c = 21.0202999115
alpha = 90.0
beta = 90.0
gamma = 120.0

	Atom	X	Y	Z	X	Y	Z
1	Ni	0.0000	0.0000	0.0000	0.0000	0.0000	0.0000
2	Ni	-1.2289	2.1285	0.0000	0.0000	0.2500	0.0000
3	Ni	-2.4578	4.2570	0.0000	0.0000	0.5000	0.0000
4	Ni	-3.6867	6.3856	0.0000	0.0000	0.7500	0.0000
5	Ni	4.9156	0.0000	0.0000	0.5000	0.0000	0.0000
6	Ni	3.6867	2.1285	0.0000	0.5000	0.2500	0.0000
7	Ni	2.4578	4.2570	0.0000	0.5000	0.5000	0.0000
8	Ni	1.2289	6.3856	0.0000	0.5000	0.7500	0.0000
9	Ni	2.4578	1.4190	2.0068	0.3333	0.1667	0.0955
10	Ni	1.2289	3.5475	2.0068	0.3333	0.4167	0.0955
11	Ni	0.0000	5.6760	2.0068	0.3333	0.6667	0.0955
12	Ni	-1.2289	7.8045	2.0068	0.3333	0.9167	0.0955
13	Ni	7.3734	1.4190	2.0068	0.8333	0.1667	0.0955
14	Ni	6.1445	3.5475	2.0068	0.8333	0.4167	0.0955
15	Ni	4.9156	5.6760	2.0068	0.8333	0.6667	0.0955
16	Ni	3.6867	7.8045	2.0068	0.8333	0.9167	0.0955
17	Ni	0.0374	2.8598	4.0146	0.1717	0.3359	0.1910
18	Ni	1.2648	0.7265	4.0088	0.1713	0.0853	0.1907
19	Ni	-2.4246	7.1113	4.0070	0.1710	0.8352	0.1906
20	Ni	-1.2023	4.9852	4.0111	0.1705	0.5855	0.1908
21	Ni	4.9488	2.8563	4.0162	0.6711	0.3355	0.1911
22	Ni	6.1796	0.7247	4.0104	0.6711	0.0851	0.1908
23	Ni	2.4885	7.1135	3.9962	0.6709	0.8355	0.1901
24	Ni	3.7140	4.9859	4.0061	0.6706	0.5856	0.1906
25	Ni	0.0604	0.0335	6.0297	0.0081	0.0039	0.2869
26	Ni	-2.4122	4.2876	6.0270	0.0064	0.5036	0.2867
27	Ni	-3.6352	6.4061	6.0271	0.0064	0.7524	0.2867
28	Ni	4.9637	0.0357	6.0344	0.5070	0.0042	0.2871
29	Ni	1.2807	6.4080	6.0338	0.5066	0.7526	0.2870
30	Ni	2.5184	0.0394	6.0541	0.2585	0.0046	0.2880
31	Ni	3.7430	6.4069	6.0482	0.7570	0.7525	0.2877
32	Ni	7.4273	0.0437	6.0483	0.7581	0.0051	0.2877
33	Co	1.2289	2.1285	0.0000	0.2500	0.2500	0.0000
34	Co	2.4578	0.0000	0.0000	0.2500	0.0000	0.0000
35	Co	-1.2289	6.3856	0.0000	0.2500	0.7500	0.0000
36	Co	0.0000	4.2570	0.0000	0.2500	0.5000	0.0000
37	Co	6.1445	2.1285	0.0000	0.7500	0.2500	0.0000
38	Co	7.3734	0.0000	0.0000	0.7500	0.0000	0.0000
39	Co	3.6867	6.3856	0.0000	0.7500	0.7500	0.0000
40	Co	4.9156	4.2570	0.0000	0.7500	0.5000	0.0000
41	Co	-1.2289	3.5475	2.0068	0.0833	0.4167	0.0955

42	Co	0.0000	1.4190	2.0068	0.0833	0.1667	0.0955
43	Co	-3.6867	7.8045	2.0068	0.0833	0.9167	0.0955
44	Co	-2.4578	5.6760	2.0068	0.0833	0.6667	0.0955
45	Co	3.6867	3.5475	2.0068	0.5833	0.4167	0.0955
46	Co	4.9156	1.4190	2.0068	0.5833	0.1667	0.0955
47	Co	1.2289	7.8045	2.0068	0.5833	0.9167	0.0955
48	Co	2.4578	5.6760	2.0068	0.5833	0.6667	0.0955
49	Co	3.7226	0.7346	4.0493	0.4218	0.0863	0.1926
50	Co	2.4900	2.8587	4.0567	0.4212	0.3358	0.1930
51	Co	1.2576	4.9817	4.0557	0.4205	0.5851	0.1929
52	Co	0.0283	7.1099	4.0615	0.4204	0.8351	0.1932
53	Co	8.6409	0.7339	4.0561	0.9220	0.0862	0.1930
54	Co	7.4074	2.8518	4.0541	0.9209	0.3350	0.1929
55	Co	6.1668	4.9828	4.0511	0.9199	0.5852	0.1927
56	Co	4.9526	7.1177	4.0406	0.9218	0.8360	0.1922
57	Co	1.2935	2.1703	6.0212	0.2590	0.2549	0.2864
58	Co	-1.1536	6.4071	6.0173	0.2589	0.7525	0.2863
59	Co	0.0675	4.2852	6.0151	0.2585	0.5033	0.2862
60	Co	6.1978	2.1734	6.0171	0.7581	0.2553	0.2863
61	Co	4.9732	4.2767	6.0092	0.7570	0.5023	0.2859
62	Co	3.7291	2.1388	5.9956	0.5049	0.2512	0.2852
63	Co	2.4916	4.2817	5.9934	0.5049	0.5029	0.2851
64	Co	-1.1862	2.1562	5.9947	0.0060	0.2533	0.2852

/db/jmorales/CoNi-alloy/Slabs/Segregation/Ni-CoNi(001)/Ni-CoNi(001)-4-Co

a =	6.9516692132
b =	6.9516692132
c =	21.9516692133
alpha =	90.0
beta =	90.0
gamma =	90.0

	Atom	X	Y	Z	X	Y	Z
1	Co	0.0000	0.0000	0.0000	0.0000	0.0000	0.0000
2	Co	1.7379	1.7379	0.0000	0.2500	0.2500	0.0000
3	Co	0.0000	3.4758	0.0000	0.0000	0.5000	0.0000
4	Co	1.7379	5.2138	0.0000	0.2500	0.7500	0.0000
5	Co	3.4758	0.0000	0.0000	0.5000	0.0000	0.0000
6	Co	5.2138	1.7379	0.0000	0.7500	0.2500	0.0000
7	Co	3.4758	3.4758	0.0000	0.5000	0.5000	0.0000
8	Co	5.2138	5.2138	0.0000	0.7500	0.7500	0.0000
9	Co	0.0103	-0.0003	3.5585	0.0015	-0.0000	0.1621
10	Co	1.7297	1.7368	3.5561	0.2488	0.2498	0.1620
11	Co	0.0094	3.4761	3.5589	0.0013	0.5000	0.1621
12	Co	1.7298	5.2146	3.5561	0.2488	0.7501	0.1620
13	Co	1.7616	3.4759	5.2074	0.2534	0.5000	0.2372
14	Co	3.4738	1.7699	5.2112	0.4997	0.2546	0.2374
15	Co	5.1840	3.4748	5.2154	0.7457	0.4999	0.2376
16	Co	3.4742	5.1819	5.2104	0.4998	0.7454	0.2374
17	Ni	1.7379	0.0000	1.7379	0.2500	0.0000	0.0792
18	Ni	0.0000	1.7379	1.7379	0.0000	0.2500	0.0792
19	Ni	1.7379	3.4758	1.7379	0.2500	0.5000	0.0792
20	Ni	0.0000	5.2138	1.7379	0.0000	0.7500	0.0792
21	Ni	5.2138	0.0000	1.7379	0.7500	0.0000	0.0792
22	Ni	3.4758	1.7379	1.7379	0.5000	0.2500	0.0792
23	Ni	5.2138	3.4758	1.7379	0.7500	0.5000	0.0792
24	Ni	3.4758	5.2138	1.7379	0.5000	0.7500	0.0792
25	Ni	1.7634	-0.0000	5.2396	0.2537	-0.0000	0.2387
26	Ni	6.9453	1.7491	5.2431	0.9991	0.2516	0.2388
27	Ni	3.4702	3.4755	3.5126	0.4992	0.4999	0.1600
28	Ni	6.9445	5.2022	5.2439	0.9990	0.7483	0.2389
29	Ni	5.1892	0.0003	5.2380	0.7465	0.0000	0.2386
30	Ni	3.4763	0.0002	3.5335	0.5001	0.0000	0.1610
31	Ni	5.2180	1.7371	3.5335	0.7506	0.2499	0.1610
32	Ni	5.2177	5.2142	3.5339	0.7506	0.7501	0.1610

/db/jmorales/CoNi-alloy/Slabs/Segregation/CoNi(001)/CoNi(001)-4-Co

a =	6.9516692132
b =	6.9516692132
c =	21.9516692133
alpha =	90.0
beta =	90.0
gamma =	90.0

	Atom	X	Y	Z	X	Y	Z
1	Ni	0.0000	0.0000	0.0000	0.0000	0.0000	0.0000
2	Ni	0.0000	3.4758	0.0000	0.0000	0.5000	0.0000
3	Ni	3.4758	0.0000	0.0000	0.5000	0.0000	0.0000
4	Ni	3.4758	3.4758	0.0000	0.5000	0.5000	0.0000
5	Ni	0.0000	1.7379	1.7379	0.0000	0.2500	0.0792
6	Ni	0.0000	5.2138	1.7379	0.0000	0.7500	0.0792
7	Ni	3.4758	1.7379	1.7379	0.5000	0.2500	0.0792
8	Ni	3.4758	5.2138	1.7379	0.5000	0.7500	0.0792
9	Ni	-0.0005	-0.0000	3.5291	-0.0001	-0.0000	0.1608
10	Ni	-0.0008	3.4757	3.5288	-0.0001	0.5000	0.1608
11	Ni	3.4753	-0.0000	3.5277	0.4999	-0.0000	0.1607
12	Ni	3.4755	3.4758	3.5294	0.5000	0.5000	0.1608
13	Ni	1.7378	1.7376	3.5443	0.2500	0.2500	0.1615
14	Ni	1.7379	5.2142	3.5443	0.2500	0.7501	0.1615
15	Ni	5.2134	1.7377	3.5442	0.7499	0.2500	0.1615
16	Ni	5.2134	5.2139	3.5443	0.7500	0.7500	0.1615
17	Co	1.7379	1.7379	0.0000	0.2500	0.2500	0.0000
18	Co	1.7379	5.2138	0.0000	0.2500	0.7500	0.0000
19	Co	5.2138	1.7379	0.0000	0.7500	0.2500	0.0000
20	Co	5.2138	5.2138	0.0000	0.7500	0.7500	0.0000
21	Co	1.7379	0.0000	1.7379	0.2500	0.0000	0.0792
22	Co	1.7379	3.4758	1.7379	0.2500	0.5000	0.0792
23	Co	5.2138	0.0000	1.7379	0.7500	0.0000	0.0792
24	Co	5.2138	3.4758	1.7379	0.7500	0.5000	0.0792
25	Co	3.4745	1.7376	5.2583	0.4998	0.2500	0.2395
26	Co	1.7379	6.9512	5.2640	0.2500	0.9999	0.2398
27	Co	1.7355	3.4760	5.2630	0.2497	0.5000	0.2398
28	Co	5.2119	0.0002	5.2653	0.7497	0.0000	0.2399
29	Co	5.2140	3.4755	5.2645	0.7500	0.4999	0.2398
30	Co	3.4750	5.2139	5.2581	0.4999	0.7500	0.2395
31	Co	-0.0005	1.7369	5.2588	-0.0001	0.2499	0.2396
32	Co	-0.0010	5.2145	5.2588	-0.0001	0.7501	0.2396

/db/jmorales/CoNi-alloy/Slabs/Segregation/CoNi(001)/CoNi(001)-3-Ni-CO

a =	6.9516692132
b =	6.9516692132
c =	21.9516692133
alpha =	90.0
beta =	90.0
gamma =	90.0

	Atom	X	Y	Z	X	Y	Z
1	Ni	0.0000	0.0000	0.0000	0.0000	0.0000	0.0000
2	Ni	0.0000	3.4758	0.0000	0.0000	0.5000	0.0000
3	Ni	3.4758	0.0000	0.0000	0.5000	0.0000	0.0000
4	Ni	3.4758	3.4758	0.0000	0.5000	0.5000	0.0000
5	Ni	0.0000	1.7379	1.7379	0.0000	0.2500	0.0792
6	Ni	0.0000	5.2138	1.7379	0.0000	0.7500	0.0792
7	Ni	3.4758	1.7379	1.7379	0.5000	0.2500	0.0792
8	Ni	3.4758	5.2138	1.7379	0.5000	0.7500	0.0792
9	Ni	3.4821	0.0001	3.5078	0.5009	0.0000	0.1598
10	Ni	1.7538	3.4760	5.2534	0.2523	0.5000	0.2393
11	Ni	6.9500	1.7313	5.2030	0.9998	0.2491	0.2370
12	Ni	6.9500	5.2205	5.2030	0.9998	0.7510	0.2370
13	Ni	3.4794	1.7565	5.2514	0.5005	0.2527	0.2392
14	Ni	3.4794	5.1955	5.2512	0.5005	0.7474	0.2392
15	Ni	5.1970	3.4760	5.2506	0.7476	0.5000	0.2392
16	Ni	1.7359	0.0001	5.2000	0.2497	0.0000	0.2369
17	Co	1.7379	1.7379	0.0000	0.2500	0.2500	0.0000
18	Co	1.7379	5.2138	0.0000	0.2500	0.7500	0.0000
19	Co	5.2138	1.7379	0.0000	0.7500	0.2500	0.0000
20	Co	5.2138	5.2138	0.0000	0.7500	0.7500	0.0000
21	Co	1.7379	0.0000	1.7379	0.2500	0.0000	0.0792
22	Co	1.7379	3.4758	1.7379	0.2500	0.5000	0.0792
23	Co	5.2138	0.0000	1.7379	0.7500	0.0000	0.0792
24	Co	5.2138	3.4758	1.7379	0.7500	0.5000	0.0792
25	Co	1.7269	1.7391	3.5250	0.2484	0.2502	0.1606
26	Co	1.7269	5.2128	3.5250	0.2484	0.7499	0.1606
27	Co	5.2232	1.7368	3.5249	0.7514	0.2498	0.1606
28	Co	5.2232	5.2151	3.5248	0.7514	0.7502	0.1606
29	Co	3.4729	3.4759	3.4547	0.4996	0.5000	0.1574
30	Co	5.2283	0.0001	5.1673	0.7521	0.0000	0.2354
31	Co	0.0006	3.4759	3.5063	0.0001	0.5000	0.1597
32	Co	6.9439	0.0001	3.5076	0.9989	0.0000	0.1598
33	O	3.4746	3.4764	7.5508	0.4998	0.5001	0.3440
34	C	3.4780	3.4761	6.3356	0.5003	0.5000	0.2886

/db/jmorales/CoNi-alloy/Slabs/Segregation/CoNi(111)/CoNi(111)-1-Ni

a =	9.831199646
b =	9.831199646075708
c =	21.0202999115
alpha =	90.0
beta =	90.0
gamma =	120.0

	Atom	X	Y	Z	X	Y	Z
1	Ni	0.0000	0.0000	0.0000	0.0000	0.0000	0.0000
2	Ni	-1.2289	2.1285	0.0000	0.0000	0.2500	0.0000
3	Ni	-2.4578	4.2570	0.0000	0.0000	0.5000	0.0000
4	Ni	-3.6867	6.3856	0.0000	0.0000	0.7500	0.0000
5	Ni	4.9156	0.0000	0.0000	0.5000	0.0000	0.0000
6	Ni	3.6867	2.1285	0.0000	0.5000	0.2500	0.0000
7	Ni	2.4578	4.2570	0.0000	0.5000	0.5000	0.0000
8	Ni	1.2289	6.3856	0.0000	0.5000	0.7500	0.0000
9	Ni	2.4578	1.4190	2.0068	0.3333	0.1667	0.0955
10	Ni	1.2289	3.5475	2.0068	0.3333	0.4167	0.0955
11	Ni	0.0000	5.6760	2.0068	0.3333	0.6667	0.0955
12	Ni	-1.2289	7.8045	2.0068	0.3333	0.9167	0.0955
13	Ni	7.3734	1.4190	2.0068	0.8333	0.1667	0.0955
14	Ni	6.1445	3.5475	2.0068	0.8333	0.4167	0.0955
15	Ni	4.9156	5.6760	2.0068	0.8333	0.6667	0.0955
16	Ni	3.6867	7.8045	2.0068	0.8333	0.9167	0.0955
17	Ni	0.0313	2.8562	4.0018	0.1709	0.3355	0.1904
18	Ni	-2.4241	7.1071	4.0105	0.1708	0.8347	0.1908
19	Ni	-1.1979	4.9853	4.0142	0.1709	0.5855	0.1910
20	Ni	4.9497	2.8566	4.0136	0.6712	0.3355	0.1909
21	Ni	6.1738	0.7271	4.0116	0.6707	0.0854	0.1908
22	Ni	2.4899	7.1125	4.0112	0.6710	0.8354	0.1908
23	Ni	3.7182	4.9859	4.0138	0.6710	0.5856	0.1909
24	Ni	0.0505	0.0270	6.0355	0.0067	0.0032	0.2871
25	Ni	-1.1808	2.1582	6.0300	0.0066	0.2535	0.2869
26	Ni	-2.4092	4.2888	6.0256	0.0068	0.5037	0.2867
27	Ni	-3.6354	6.4127	6.0298	0.0068	0.7532	0.2869
28	Ni	4.9680	0.0267	6.0249	0.5069	0.0031	0.2866
29	Ni	3.7377	2.1615	6.0229	0.5071	0.2539	0.2865
30	Ni	2.5131	4.2882	6.0228	0.5075	0.5037	0.2865
31	Ni	1.2813	6.4179	6.0241	0.5072	0.7538	0.2866
32	Ni	1.2919	2.1633	6.0416	0.2585	0.2541	0.2874
33	Co	1.2289	2.1285	0.0000	0.2500	0.2500	0.0000
34	Co	2.4578	0.0000	0.0000	0.2500	0.0000	0.0000
35	Co	-1.2289	6.3856	0.0000	0.2500	0.7500	0.0000
36	Co	0.0000	4.2570	0.0000	0.2500	0.5000	0.0000
37	Co	6.1445	2.1285	0.0000	0.7500	0.2500	0.0000
38	Co	7.3734	0.0000	0.0000	0.7500	0.0000	0.0000
39	Co	3.6867	6.3856	0.0000	0.7500	0.7500	0.0000
40	Co	4.9156	4.2570	0.0000	0.7500	0.5000	0.0000
41	Co	-1.2289	3.5475	2.0068	0.0833	0.4167	0.0955

42	Co	0.0000	1.4190	2.0068	0.0833	0.1667	0.0955
43	Co	-3.6867	7.8045	2.0068	0.0833	0.9167	0.0955
44	Co	-2.4578	5.6760	2.0068	0.0833	0.6667	0.0955
45	Co	3.6867	3.5475	2.0068	0.5833	0.4167	0.0955
46	Co	4.9156	1.4190	2.0068	0.5833	0.1667	0.0955
47	Co	1.2289	7.8045	2.0068	0.5833	0.9167	0.0955
48	Co	2.4578	5.6760	2.0068	0.5833	0.6667	0.0955
49	Co	3.7050	0.7263	4.0532	0.4195	0.0853	0.1928
50	Co	2.4871	2.8498	4.0382	0.4203	0.3347	0.1921
51	Co	1.2557	4.9804	4.0544	0.4202	0.5850	0.1929
52	Co	0.0284	7.1107	4.0514	0.4205	0.8352	0.1927
53	Co	8.6386	0.7299	4.0555	0.9216	0.0857	0.1929
54	Co	7.4039	2.8543	4.0564	0.9207	0.3352	0.1930
55	Co	6.1762	4.9838	4.0498	0.9209	0.5854	0.1927
56	Co	4.9491	7.1178	4.0559	0.9214	0.8360	0.1930
57	Co	2.5174	0.0357	6.0006	0.2582	0.0042	0.2855
58	Co	-1.1563	6.4245	6.0116	0.2597	0.7546	0.2860
59	Co	0.0603	4.3075	6.0127	0.2591	0.5059	0.2860
60	Co	6.2146	2.1680	6.0131	0.7595	0.2546	0.2861
61	Co	7.4394	0.0395	6.0141	0.7590	0.0046	0.2861
62	Co	3.7579	6.4245	6.0164	0.7595	0.7546	0.2862
63	Co	4.9882	4.3004	6.0126	0.7599	0.5051	0.2860
64	Co	1.2548	0.7222	4.0068	0.1700	0.0848	0.1906

/db/jmorales/CoNi-alloy/Slabs/Segregation/CoNi(111)/CoNi(111)-4-Ni-CO
a = 9.831199646
b = 9.831199646075708
c = 21.0202999115
alpha = 90.0
beta = 90.0
gamma = 120.0

	Atom	X	Y	Z	X	Y	Z
1	Ni	0.0000	0.0000	0.0000	0.0000	0.0000	0.0000
2	Ni	-1.2289	2.1285	0.0000	0.0000	0.2500	0.0000
3	Ni	-2.4578	4.2570	0.0000	0.0000	0.5000	0.0000
4	Ni	-3.6867	6.3856	0.0000	0.0000	0.7500	0.0000
5	Ni	4.9156	0.0000	0.0000	0.5000	0.0000	0.0000
6	Ni	3.6867	2.1285	0.0000	0.5000	0.2500	0.0000
7	Ni	2.4578	4.2570	0.0000	0.5000	0.5000	0.0000
8	Ni	1.2289	6.3856	0.0000	0.5000	0.7500	0.0000
9	Ni	2.4578	1.4190	2.0068	0.3333	0.1667	0.0955
10	Ni	1.2289	3.5475	2.0068	0.3333	0.4167	0.0955
11	Ni	0.0000	5.6760	2.0068	0.3333	0.6667	0.0955
12	Ni	-1.2289	7.8045	2.0068	0.3333	0.9167	0.0955
13	Ni	7.3734	1.4190	2.0068	0.8333	0.1667	0.0955
14	Ni	6.1445	3.5475	2.0068	0.8333	0.4167	0.0955
15	Ni	4.9156	5.6760	2.0068	0.8333	0.6667	0.0955
16	Ni	3.6867	7.8045	2.0068	0.8333	0.9167	0.0955
17	Ni	4.9420	2.8541	4.0161	0.6703	0.3352	0.1911
18	Ni	6.1733	0.7289	4.0080	0.6707	0.0856	0.1907
19	Ni	2.4893	7.1098	4.0086	0.6707	0.8351	0.1907
20	Ni	3.7129	4.9811	4.0173	0.6702	0.5850	0.1911
21	Ni	0.0354	0.0247	6.0330	0.0050	0.0029	0.2870
22	Ni	-1.1903	2.1483	6.0319	0.0051	0.2523	0.2870
23	Ni	-2.4193	4.2766	6.0329	0.0051	0.5023	0.2870
24	Ni	-3.6518	6.4107	6.0330	0.0050	0.7530	0.2870
25	Ni	4.9717	0.0321	6.0118	0.5076	0.0038	0.2860
26	Ni	3.7491	2.1650	6.0746	0.5085	0.2543	0.2890
27	Ni	2.5174	4.2914	6.0783	0.5081	0.5040	0.2892
28	Ni	1.2839	6.4191	6.0128	0.5076	0.7539	0.2860
29	Ni	1.2725	2.1533	6.0814	0.2559	0.2529	0.2893
30	Ni	2.5042	0.0038	6.0096	0.2549	0.0005	0.2859
31	Ni	0.0253	4.2962	6.0099	0.2549	0.5046	0.2859
32	Ni	-1.1841	6.4118	6.0199	0.2561	0.7531	0.2864
33	Co	1.2289	2.1285	0.0000	0.2500	0.2500	0.0000
34	Co	2.4578	0.0000	0.0000	0.2500	0.0000	0.0000
35	Co	-1.2289	6.3856	0.0000	0.2500	0.7500	0.0000
36	Co	0.0000	4.2570	0.0000	0.2500	0.5000	0.0000
37	Co	6.1445	2.1285	0.0000	0.7500	0.2500	0.0000
38	Co	7.3734	0.0000	0.0000	0.7500	0.0000	0.0000
39	Co	3.6867	6.3856	0.0000	0.7500	0.7500	0.0000
40	Co	4.9156	4.2570	0.0000	0.7500	0.5000	0.0000
41	Co	-1.2289	3.5475	2.0068	0.0833	0.4167	0.0955

42	Co	0.0000	1.4190	2.0068	0.0833	0.1667	0.0955
43	Co	-3.6867	7.8045	2.0068	0.0833	0.9167	0.0955
44	Co	-2.4578	5.6760	2.0068	0.0833	0.6667	0.0955
45	Co	3.6867	3.5475	2.0068	0.5833	0.4167	0.0955
46	Co	4.9156	1.4190	2.0068	0.5833	0.1667	0.0955
47	Co	1.2289	7.8045	2.0068	0.5833	0.9167	0.0955
48	Co	2.4578	5.6760	2.0068	0.5833	0.6667	0.0955
49	Co	3.7105	0.7291	4.0314	0.4202	0.0856	0.1918
50	Co	2.4842	2.8528	4.0013	0.4202	0.3351	0.1904
51	Co	1.2568	4.9781	4.0326	0.4202	0.5847	0.1918
52	Co	0.0301	7.1123	4.0282	0.4207	0.8354	0.1916
53	Co	8.6451	0.7343	4.0482	0.9225	0.0862	0.1926
54	Co	7.4160	2.8620	4.0492	0.9224	0.3362	0.1926
55	Co	6.1824	4.9892	4.0476	0.9219	0.5860	0.1926
56	Co	4.9572	7.1206	4.0501	0.9224	0.8363	0.1927
57	Co	6.2067	2.1575	6.0008	0.7580	0.2534	0.2855
58	Co	7.4331	0.0350	6.0052	0.7581	0.0041	0.2857
59	Co	3.7420	6.4249	6.0023	0.7579	0.7546	0.2855
60	Co	4.9970	4.3050	5.9948	0.7611	0.5056	0.2852
61	Co	1.2551	0.7299	4.0004	0.1705	0.0857	0.1903
62	Co	-2.4316	7.1096	4.0112	0.1702	0.8350	0.1908
63	Co	0.0312	2.8514	4.0008	0.1706	0.3349	0.1903
64	Co	-1.2043	4.9824	4.0095	0.1701	0.5852	0.1907
65	O	2.4983	2.8514	8.6068	0.4216	0.3349	0.4095
66	C	2.4929	2.8535	7.4136	0.4211	0.3352	0.3527

/db/jmorales/CoNi-alloy/Slabs/Ni(001)

a =			6.950991869				
b =			6.950991869				
c =			21.950991869				
alpha =			90.0				
beta =			90.0				
gamma =			90.0				
	Atom	X	Y	Z	X	Y	Z
1	Ni	0.0000	0.0000	0.0000	0.0000	0.0000	0.0000
2	Ni	1.7378	1.7378	0.0000	0.2500	0.2500	0.0000
3	Ni	0.0000	3.4755	0.0000	0.0000	0.5000	0.0000
4	Ni	1.7378	5.2132	0.0000	0.2500	0.7500	0.0000
5	Ni	3.4755	0.0000	0.0000	0.5000	0.0000	0.0000
6	Ni	5.2132	1.7378	0.0000	0.7500	0.2500	0.0000
7	Ni	3.4755	3.4755	0.0000	0.5000	0.5000	0.0000
8	Ni	5.2132	5.2132	0.0000	0.7500	0.7500	0.0000
9	Ni	0.0000	1.7378	1.7378	0.0000	0.2500	0.0792
10	Ni	1.7378	0.0000	1.7378	0.2500	0.0000	0.0792
11	Ni	0.0000	5.2132	1.7378	0.0000	0.7500	0.0792
12	Ni	1.7378	3.4755	1.7378	0.2500	0.5000	0.0792
13	Ni	3.4755	1.7378	1.7378	0.5000	0.2500	0.0792
14	Ni	5.2132	0.0000	1.7378	0.7500	0.0000	0.0792
15	Ni	3.4755	5.2132	1.7378	0.5000	0.7500	0.0792
16	Ni	5.2132	3.4755	1.7378	0.7500	0.5000	0.0792
17	Ni	0.0000	0.0000	3.4755	0.0000	0.0000	0.1583
18	Ni	1.7378	1.7378	3.4755	0.2500	0.2500	0.1583
19	Ni	0.0000	3.4755	3.4755	0.0000	0.5000	0.1583
20	Ni	1.7378	5.2132	3.4755	0.2500	0.7500	0.1583
21	Ni	3.4755	0.0000	3.4755	0.5000	0.0000	0.1583
22	Ni	5.2132	1.7378	3.4755	0.7500	0.2500	0.1583
23	Ni	3.4755	3.4755	3.4755	0.5000	0.5000	0.1583
24	Ni	5.2132	5.2132	3.4755	0.7500	0.7500	0.1583
25	Ni	0.0000	1.7378	5.2132	0.0000	0.2500	0.2375
26	Ni	1.7378	0.0000	5.2132	0.2500	0.0000	0.2375
27	Ni	0.0000	5.2132	5.2132	0.0000	0.7500	0.2375
28	Ni	1.7378	3.4755	5.2132	0.2500	0.5000	0.2375
29	Ni	3.4755	1.7378	5.2132	0.5000	0.2500	0.2375
30	Ni	5.2132	0.0000	5.2132	0.7500	0.0000	0.2375
31	Ni	3.4755	5.2132	5.2132	0.5000	0.7500	0.2375
32	Ni	5.2132	3.4755	5.2132	0.7500	0.5000	0.2375

/db/jmorales/CoNi-alloy/Adsorptions/Functionals/PBEsol/Ni(111)-CO

a =	9.8299999236
b =	9.829999923841429
c =	21.0196990967
alpha =	90.0
beta =	90.0
gamma =	120.0

	Atom	X	Y	Z	X	Y	Z
1	Ni	0.0000	0.0000	0.0000	0.0000	0.0000	0.0000
2	Ni	-1.2287	2.1283	0.0000	0.0000	0.2500	0.0000
3	Ni	-2.4575	4.2565	0.0000	0.0000	0.5000	0.0000
4	Ni	-3.6862	6.3848	0.0000	-0.0000	0.7500	0.0000
5	Ni	2.4575	0.0000	0.0000	0.2500	0.0000	0.0000
6	Ni	1.2287	2.1283	0.0000	0.2500	0.2500	0.0000
7	Ni	0.0000	4.2565	0.0000	0.2500	0.5000	0.0000
8	Ni	-1.2287	6.3848	0.0000	0.2500	0.7500	0.0000
9	Ni	4.9150	0.0000	0.0000	0.5000	0.0000	0.0000
10	Ni	3.6862	2.1283	0.0000	0.5000	0.2500	0.0000
11	Ni	2.4575	4.2565	0.0000	0.5000	0.5000	0.0000
12	Ni	1.2287	6.3848	0.0000	0.5000	0.7500	0.0000
13	Ni	7.3725	0.0000	0.0000	0.7500	0.0000	0.0000
14	Ni	6.1437	2.1283	0.0000	0.7500	0.2500	0.0000
15	Ni	4.9150	4.2565	0.0000	0.7500	0.5000	0.0000
16	Ni	3.6862	6.3848	0.0000	0.7500	0.7500	0.0000
17	Ni	-0.0000	1.4188	2.0065	0.0833	0.1667	0.0955
18	Ni	-1.2288	3.5471	2.0065	0.0833	0.4167	0.0955
19	Ni	-2.4575	5.6754	2.0065	0.0833	0.6667	0.0955
20	Ni	-3.6863	7.8036	2.0065	0.0833	0.9167	0.0955
21	Ni	2.4575	1.4188	2.0065	0.3333	0.1667	0.0955
22	Ni	1.2287	3.5471	2.0065	0.3333	0.4167	0.0955
23	Ni	-0.0000	5.6754	2.0065	0.3333	0.6667	0.0955
24	Ni	-1.2288	7.8036	2.0065	0.3333	0.9167	0.0955
25	Ni	4.9150	1.4188	2.0065	0.5833	0.1667	0.0955
26	Ni	3.6862	3.5471	2.0065	0.5833	0.4167	0.0955
27	Ni	2.4575	5.6754	2.0065	0.5833	0.6667	0.0955
28	Ni	1.2287	7.8036	2.0065	0.5833	0.9167	0.0955
29	Ni	7.3725	1.4188	2.0065	0.8333	0.1667	0.0955
30	Ni	6.1437	3.5471	2.0065	0.8333	0.4167	0.0955
31	Ni	4.9150	5.6754	2.0065	0.8333	0.6667	0.0955
32	Ni	3.6862	7.8036	2.0065	0.8333	0.9167	0.0955
33	Ni	1.2286	0.7197	3.9693	0.1673	0.0845	0.1888
34	Ni	0.0019	2.8403	3.9643	0.1670	0.3336	0.1886
35	Ni	-1.2223	4.9637	3.9688	0.1672	0.5831	0.1888
36	Ni	-2.4582	7.0959	3.9637	0.1667	0.8335	0.1886
37	Ni	3.6810	0.7122	3.9595	0.4163	0.0837	0.1884
38	Ni	2.4551	2.8407	3.9648	0.4166	0.3337	0.1886
39	Ni	1.2289	4.9650	3.9647	0.4166	0.5832	0.1886
40	Ni	-0.0013	7.0908	3.9595	0.4163	0.8329	0.1884
41	Ni	6.1437	0.7108	3.9620	0.6667	0.0835	0.1885

42	Ni	4.9122	2.8413	3.9604	0.6666	0.3338	0.1884
43	Ni	3.6789	4.9633	3.9694	0.6658	0.5830	0.1888
44	Ni	2.4584	7.0922	3.9597	0.6666	0.8331	0.1884
45	Ni	8.6059	0.7127	3.9602	0.9173	0.0837	0.1884
46	Ni	7.3764	2.8421	3.9598	0.9173	0.3339	0.1884
47	Ni	6.1432	4.9673	3.9652	0.9167	0.5835	0.1886
48	Ni	4.9147	7.0955	3.9655	0.9167	0.8335	0.1887
49	Ni	0.0002	0.0046	5.9210	0.0003	0.0005	0.2817
50	Ni	8.5762	2.1186	5.9180	0.9969	0.2489	0.2815
51	Ni	-2.4580	4.2609	5.9206	0.0002	0.5005	0.2817
52	Ni	-3.6873	6.3883	5.9235	0.0001	0.7504	0.2818
53	Ni	2.4553	0.0033	5.9207	0.2500	0.0004	0.2817
54	Ni	1.2277	2.1332	5.9884	0.2502	0.2506	0.2849
55	Ni	-0.0009	4.2608	5.9859	0.2502	0.5005	0.2848
56	Ni	-1.2297	6.3881	5.9206	0.2501	0.7504	0.2817
57	Ni	4.9121	0.0024	5.9198	0.4998	0.0003	0.2816
58	Ni	3.7102	2.1153	5.9179	0.5017	0.2485	0.2815
59	Ni	2.4564	4.2587	5.9866	0.5000	0.5003	0.2848
60	Ni	1.2282	6.4177	5.9177	0.5019	0.7539	0.2815
61	Ni	7.3742	0.0026	5.9208	0.7503	0.0003	0.2817
62	Ni	6.1437	2.1337	5.9208	0.7503	0.2506	0.2817
63	Ni	4.9136	4.2595	5.9215	0.7500	0.5004	0.2817
64	Ni	3.6859	6.3874	5.9214	0.7501	0.7503	0.2817
65	O	1.2239	3.5609	8.4830	0.3337	0.4183	0.4036
66	C	1.2269	3.5547	7.2909	0.3336	0.4176	0.3469

/db/jmorales/CoNi-alloy/Adsorptions/Functionals/RPBE/CoNi(111)

a =		9.831199646					
b =		9.831199646075708					
c =		21.0202999115					
alpha =		90.0					
beta =		90.0					
gamma =		120.0					
	Atom	X	Y	Z	X	Y	Z
1	Ni	0.0000	0.0000	0.0000	0.0000	0.0000	0.0000
2	Ni	-1.2289	2.1285	0.0000	0.0000	0.2500	0.0000
3	Ni	-2.4578	4.2570	0.0000	0.0000	0.5000	0.0000
4	Ni	-3.6867	6.3856	0.0000	0.0000	0.7500	0.0000
5	Ni	4.9156	0.0000	0.0000	0.5000	0.0000	0.0000
6	Ni	3.6867	2.1285	0.0000	0.5000	0.2500	0.0000
7	Ni	2.4578	4.2570	0.0000	0.5000	0.5000	0.0000
8	Ni	1.2289	6.3856	0.0000	0.5000	0.7500	0.0000
9	Ni	2.4578	1.4190	2.0068	0.3333	0.1667	0.0955
10	Ni	1.2289	3.5475	2.0068	0.3333	0.4167	0.0955
11	Ni	0.0000	5.6760	2.0068	0.3333	0.6667	0.0955
12	Ni	-1.2289	7.8045	2.0068	0.3333	0.9167	0.0955
13	Ni	7.3734	1.4190	2.0068	0.8333	0.1667	0.0955
14	Ni	6.1445	3.5475	2.0068	0.8333	0.4167	0.0955
15	Ni	4.9156	5.6760	2.0068	0.8333	0.6667	0.0955
16	Ni	3.6867	7.8045	2.0068	0.8333	0.9167	0.0955
17	Ni	0.0340	2.8576	4.0236	0.1713	0.3356	0.1914
18	Ni	1.2629	0.7291	4.0236	0.1713	0.0856	0.1914
19	Ni	-2.4238	7.1146	4.0236	0.1713	0.8356	0.1914
20	Ni	-1.1949	4.9861	4.0236	0.1713	0.5856	0.1914
21	Ni	4.9496	2.8576	4.0236	0.6713	0.3356	0.1914
22	Ni	6.1785	0.7291	4.0236	0.6713	0.0856	0.1914
23	Ni	2.4918	7.1146	4.0236	0.6713	0.8356	0.1914
24	Ni	3.7207	4.9861	4.0236	0.6713	0.5856	0.1914
25	Ni	0.0515	0.0297	6.0556	0.0070	0.0035	0.2881
26	Ni	-1.1774	2.1583	6.0556	0.0070	0.2535	0.2881
27	Ni	-2.4063	4.2868	6.0556	0.0070	0.5035	0.2881
28	Ni	-3.6352	6.4153	6.0556	0.0070	0.7535	0.2881
29	Ni	4.9671	0.0297	6.0556	0.5070	0.0035	0.2881
30	Ni	3.7382	2.1583	6.0556	0.5070	0.2535	0.2881
31	Ni	2.5093	4.2868	6.0556	0.5070	0.5035	0.2881
32	Ni	1.2804	6.4153	6.0556	0.5070	0.7535	0.2881
33	Co	1.2289	2.1285	0.0000	0.2500	0.2500	0.0000
34	Co	2.4578	0.0000	0.0000	0.2500	0.0000	0.0000
35	Co	-1.2289	6.3856	0.0000	0.2500	0.7500	0.0000
36	Co	0.0000	4.2570	0.0000	0.2500	0.5000	0.0000
37	Co	6.1445	2.1285	0.0000	0.7500	0.2500	0.0000
38	Co	7.3734	0.0000	0.0000	0.7500	0.0000	0.0000
39	Co	3.6867	6.3856	0.0000	0.7500	0.7500	0.0000
40	Co	4.9156	4.2570	0.0000	0.7500	0.5000	0.0000
41	Co	-1.2289	3.5475	2.0068	0.0833	0.4167	0.0955

42	Co	0.0000	1.4190	2.0068	0.0833	0.1667	0.0955
43	Co	-3.6867	7.8045	2.0068	0.0833	0.9167	0.0955
44	Co	-2.4578	5.6760	2.0068	0.0833	0.6667	0.0955
45	Co	3.6867	3.5475	2.0068	0.5833	0.4167	0.0955
46	Co	4.9156	1.4190	2.0068	0.5833	0.1667	0.0955
47	Co	1.2289	7.8045	2.0068	0.5833	0.9167	0.0955
48	Co	2.4578	5.6760	2.0068	0.5833	0.6667	0.0955
49	Co	3.7145	0.7254	4.0674	0.4204	0.0852	0.1935
50	Co	2.4856	2.8539	4.0674	0.4204	0.3352	0.1935
51	Co	1.2567	4.9824	4.0674	0.4204	0.5852	0.1935
52	Co	0.0278	7.1110	4.0674	0.4204	0.8352	0.1935
53	Co	8.6301	0.7254	4.0674	0.9204	0.0852	0.1935
54	Co	7.4012	2.8539	4.0674	0.9204	0.3352	0.1935
55	Co	6.1723	4.9824	4.0674	0.9204	0.5852	0.1935
56	Co	4.9434	7.1110	4.0674	0.9204	0.8352	0.1935
57	Co	1.3004	2.1698	6.0512	0.2597	0.2549	0.2879
58	Co	2.5293	0.0413	6.0512	0.2597	0.0049	0.2879
59	Co	-1.1574	6.4269	6.0512	0.2597	0.7549	0.2879
60	Co	0.0715	4.2984	6.0512	0.2597	0.5049	0.2879
61	Co	6.2160	2.1698	6.0512	0.7597	0.2549	0.2879
62	Co	7.4449	0.0413	6.0512	0.7597	0.0049	0.2879
63	Co	3.7582	6.4269	6.0512	0.7597	0.7549	0.2879
64	Co	4.9871	4.2984	6.0512	0.7597	0.5049	0.2879

/db/jmorales/CoNi-alloy/Adsorptions/Expanded-models/CO-Ni(001)-expanded

a =	7.047964809
b =	7.047964809
c =	22.047964809
alpha =	90.0
beta =	90.0
gamma =	90.0

	Atom	X	Y	Z	X	Y	Z
1	Ni	0.0000	0.0000	0.0000	0.0000	0.0000	0.0000
2	Ni	1.7620	1.7620	0.0000	0.2500	0.2500	0.0000
3	Ni	0.0000	3.5240	0.0000	0.0000	0.5000	0.0000
4	Ni	1.7620	5.2860	0.0000	0.2500	0.7500	0.0000
5	Ni	3.5240	0.0000	0.0000	0.5000	0.0000	0.0000
6	Ni	5.2860	1.7620	0.0000	0.7500	0.2500	0.0000
7	Ni	3.5240	3.5240	0.0000	0.5000	0.5000	0.0000
8	Ni	5.2860	5.2860	0.0000	0.7500	0.7500	0.0000
9	Ni	0.0000	1.7620	1.7620	0.0000	0.2500	0.0799
10	Ni	1.7620	0.0000	1.7620	0.2500	0.0000	0.0799
11	Ni	0.0000	5.2860	1.7620	0.0000	0.7500	0.0799
12	Ni	1.7620	3.5240	1.7620	0.2500	0.5000	0.0799
13	Ni	3.5240	1.7620	1.7620	0.5000	0.2500	0.0799
14	Ni	5.2860	0.0000	1.7620	0.7500	0.0000	0.0799
15	Ni	3.5240	5.2860	1.7620	0.5000	0.7500	0.0799
16	Ni	5.2860	3.5240	1.7620	0.7500	0.5000	0.0799
17	Ni	0.0000	0.0000	3.5240	0.0000	0.0000	0.1598
18	Ni	1.7620	1.7620	3.5240	0.2500	0.2500	0.1598
19	Ni	0.0000	3.5240	3.5240	0.0000	0.5000	0.1598
20	Ni	1.7620	5.2860	3.5240	0.2500	0.7500	0.1598
21	Ni	3.5240	0.0000	3.5240	0.5000	0.0000	0.1598
22	Ni	5.2860	1.7620	3.5240	0.7500	0.2500	0.1598
23	Ni	3.5240	3.5240	3.5240	0.5000	0.5000	0.1598
24	Ni	5.2860	5.2860	3.5240	0.7500	0.7500	0.1598
25	Ni	0.0000	1.7620	5.2860	0.0000	0.2500	0.2397
26	Ni	1.7620	0.0000	5.2860	0.2500	0.0000	0.2397
27	Ni	0.0000	5.2860	5.2860	0.0000	0.7500	0.2397
28	Ni	1.7620	3.5240	5.2860	0.2500	0.5000	0.2397
29	Ni	3.5240	1.7620	5.2860	0.5000	0.2500	0.2397
30	Ni	5.2860	0.0000	5.2860	0.7500	0.0000	0.2397
31	Ni	3.5240	5.2860	5.2860	0.5000	0.7500	0.2397
32	Ni	5.2860	3.5240	5.2860	0.7500	0.5000	0.2397
33	O	3.4754	3.4748	7.5578	0.4931	0.4930	0.3428
34	C	3.4732	3.4786	6.3439	0.4928	0.4936	0.2877

/db/jmorales/CoNi-alloy/Adsorptions/Expanded-models/CO_2-Ni(111)-expanded

a =	9.967200279
b =	9.967200279058309
c =	21.103700638
alpha =	90.0
beta =	90.0
gamma =	120.0

	Atom	X	Y	Z	X	Y	Z
1	Ni	0.0000	0.0000	0.0000	0.0000	0.0000	0.0000
2	Ni	-1.2459	2.1580	0.0000	-0.0000	0.2500	0.0000
3	Ni	-2.4918	4.3159	0.0000	-0.0000	0.5000	0.0000
4	Ni	-3.7377	6.4739	0.0000	0.0000	0.7500	0.0000
5	Ni	2.4918	0.0000	0.0000	0.2500	0.0000	0.0000
6	Ni	1.2459	2.1580	0.0000	0.2500	0.2500	0.0000
7	Ni	0.0000	4.3159	0.0000	0.2500	0.5000	0.0000
8	Ni	-1.2459	6.4739	0.0000	0.2500	0.7500	0.0000
9	Ni	4.9836	0.0000	0.0000	0.5000	0.0000	0.0000
10	Ni	3.7377	2.1580	0.0000	0.5000	0.2500	0.0000
11	Ni	2.4918	4.3159	0.0000	0.5000	0.5000	0.0000
12	Ni	1.2459	6.4739	0.0000	0.5000	0.7500	0.0000
13	Ni	7.4754	0.0000	0.0000	0.7500	0.0000	0.0000
14	Ni	6.2295	2.1580	0.0000	0.7500	0.2500	0.0000
15	Ni	4.9836	4.3159	0.0000	0.7500	0.5000	0.0000
16	Ni	3.7377	6.4739	0.0000	0.7500	0.7500	0.0000
17	Ni	-0.0000	1.4386	2.0346	0.0833	0.1667	0.0964
18	Ni	-1.2459	3.5966	2.0346	0.0833	0.4167	0.0964
19	Ni	-2.4918	5.7546	2.0346	0.0833	0.6667	0.0964
20	Ni	-3.7377	7.9125	2.0346	0.0833	0.9167	0.0964
21	Ni	2.4918	1.4386	2.0346	0.3333	0.1667	0.0964
22	Ni	1.2459	3.5966	2.0346	0.3333	0.4167	0.0964
23	Ni	-0.0000	5.7546	2.0346	0.3333	0.6667	0.0964
24	Ni	-1.2459	7.9125	2.0346	0.3333	0.9167	0.0964
25	Ni	4.9836	1.4386	2.0346	0.5833	0.1667	0.0964
26	Ni	3.7377	3.5966	2.0346	0.5833	0.4167	0.0964
27	Ni	2.4918	5.7546	2.0346	0.5833	0.6667	0.0964
28	Ni	1.2459	7.9125	2.0346	0.5833	0.9167	0.0964
29	Ni	7.4754	1.4386	2.0346	0.8333	0.1667	0.0964
30	Ni	6.2295	3.5966	2.0346	0.8333	0.4167	0.0964
31	Ni	4.9836	5.7546	2.0346	0.8333	0.6667	0.0964
32	Ni	3.7377	7.9125	2.0346	0.8333	0.9167	0.0964
33	Ni	1.2459	0.7193	4.0692	0.1667	0.0833	0.1928
34	Ni	0.0000	2.8773	4.0692	0.1667	0.3333	0.1928
35	Ni	-1.2459	5.0352	4.0692	0.1667	0.5833	0.1928
36	Ni	-2.4918	7.1932	4.0692	0.1667	0.8333	0.1928
37	Ni	3.7377	0.7193	4.0692	0.4167	0.0833	0.1928
38	Ni	2.4918	2.8773	4.0692	0.4167	0.3333	0.1928
39	Ni	1.2459	5.0352	4.0692	0.4167	0.5833	0.1928
40	Ni	0.0000	7.1932	4.0692	0.4167	0.8333	0.1928

41	Ni	6.2295	0.7193	4.0692	0.6667	0.0833	0.1928
42	Ni	4.9836	2.8773	4.0692	0.6667	0.3333	0.1928
43	Ni	3.7377	5.0352	4.0692	0.6667	0.5833	0.1928
44	Ni	2.4918	7.1932	4.0692	0.6667	0.8333	0.1928
45	Ni	8.7213	0.7193	4.0692	0.9167	0.0833	0.1928
46	Ni	7.4754	2.8773	4.0692	0.9167	0.3333	0.1928
47	Ni	6.2295	5.0352	4.0692	0.9167	0.5833	0.1928
48	Ni	4.9836	7.1932	4.0692	0.9167	0.8333	0.1928
49	Ni	0.0000	0.0000	6.1036	0.0000	0.0000	0.2892
50	Ni	-1.2459	2.1580	6.1036	-0.0000	0.2500	0.2892
51	Ni	-2.4918	4.3159	6.1036	-0.0000	0.5000	0.2892
52	Ni	-3.7377	6.4739	6.1036	0.0000	0.7500	0.2892
53	Ni	2.4918	0.0000	6.1036	0.2500	0.0000	0.2892
54	Ni	1.2459	2.1580	6.1036	0.2500	0.2500	0.2892
55	Ni	0.0000	4.3159	6.1036	0.2500	0.5000	0.2892
56	Ni	-1.2459	6.4739	6.1036	0.2500	0.7500	0.2892
57	Ni	4.9836	0.0000	6.1036	0.5000	0.0000	0.2892
58	Ni	3.7377	2.1580	6.1036	0.5000	0.2500	0.2892
59	Ni	2.4918	4.3159	6.1036	0.5000	0.5000	0.2892
60	Ni	1.2459	6.4739	6.1036	0.5000	0.7500	0.2892
61	Ni	7.4754	0.0000	6.1036	0.7500	0.0000	0.2892
62	Ni	6.2295	2.1580	6.1036	0.7500	0.2500	0.2892
63	Ni	4.9836	4.3159	6.1036	0.7500	0.5000	0.2892
64	Ni	3.7377	6.4739	6.1036	0.7500	0.7500	0.2892
65	C	2.1535	3.7003	7.9418	0.4304	0.4287	0.3763
66	O	2.6238	4.4813	8.7467	0.5228	0.5192	0.4145
67	O	1.5054	2.6006	8.0005	0.3017	0.3013	0.3791

/db/jmorales/CoNi-alloy/Adsorptions/Functionals/PBEsol/Ni-CoNi(001)-CO

a = 6.9516692132
b = 6.9516692132
c = 21.9516692133
alpha = 90.0
beta = 90.0
gamma = 90.0

	Atom	X	Y	Z	X	Y	Z
1	Co	0.0000	0.0000	0.0000	0.0000	0.0000	0.0000
2	Co	1.7379	1.7379	0.0000	0.2500	0.2500	0.0000
3	Co	0.0000	3.4758	0.0000	0.0000	0.5000	0.0000
4	Co	1.7379	5.2138	0.0000	0.2500	0.7500	0.0000
5	Co	3.4758	0.0000	0.0000	0.5000	0.0000	0.0000
6	Co	5.2138	1.7379	0.0000	0.7500	0.2500	0.0000
7	Co	3.4758	3.4758	0.0000	0.5000	0.5000	0.0000
8	Co	5.2138	5.2138	0.0000	0.7500	0.7500	0.0000
9	Co	-0.0002	0.0001	3.4930	-0.0000	0.0000	0.1591
10	Co	1.7326	1.7330	3.4850	0.2492	0.2493	0.1588
11	Co	-0.0002	3.4759	3.4938	-0.0000	0.5000	0.1592
12	Co	1.7327	5.2189	3.4850	0.2493	0.7507	0.1588
13	Co	3.4758	0.0002	3.4938	0.5000	0.0000	0.1592
14	Co	5.2187	1.7328	3.4851	0.7507	0.2493	0.1588
15	Co	3.4756	3.4760	3.4377	0.5000	0.5000	0.1566
16	Co	5.2187	5.2190	3.4851	0.7507	0.7508	0.1588
17	Ni	1.7379	0.0000	1.7379	0.2500	0.0000	0.0792
18	Ni	0.0000	1.7379	1.7379	0.0000	0.2500	0.0792
19	Ni	1.7379	3.4758	1.7379	0.2500	0.5000	0.0792
20	Ni	0.0000	5.2138	1.7379	0.0000	0.7500	0.0792
21	Ni	5.2138	0.0000	1.7379	0.7500	0.0000	0.0792
22	Ni	3.4758	1.7379	1.7379	0.5000	0.2500	0.0792
23	Ni	5.2138	3.4758	1.7379	0.7500	0.5000	0.0792
24	Ni	3.4758	5.2138	1.7379	0.5000	0.7500	0.0792
25	Ni	1.7383	0.0001	5.0992	0.2501	0.0000	0.2323
26	Ni	-0.0002	1.7383	5.0993	-0.0000	0.2501	0.2323
27	Ni	1.7685	3.4759	5.1436	0.2544	0.5000	0.2343
28	Ni	-0.0000	5.2133	5.0992	-0.0000	0.7499	0.2323
29	Ni	5.2133	-0.0000	5.0993	0.7499	-0.0000	0.2323
30	Ni	3.4756	1.7689	5.1436	0.5000	0.2545	0.2343
31	Ni	5.1826	3.4760	5.1434	0.7455	0.5000	0.2343
32	Ni	3.4756	5.1831	5.1436	0.5000	0.7456	0.2343
33	O	3.4753	3.4761	7.4368	0.4999	0.5000	0.3388
34	C	3.4757	3.4759	6.2251	0.5000	0.5000	0.2836

/db/jmorales/CoNi-alloy/Adsorptions/CO_2-Sm(subsurf)$Ce_{26}O_{54}$

a =			11.6082000732				
b =			11.608200073110298				
c =			22.898399353				
alpha =			90.0				
beta =			90.0				
gamma =			120.0				
	Atom	X	Y	Z	X	Y	Z
1	Sm	1.9348	5.6027	3.9953	0.4453	0.5573	0.1745
2	Ce	1.9347	1.1170	0.7898	0.2222	0.1111	0.0345
3	Ce	0.0000	4.4680	0.7898	0.2222	0.4444	0.0345
4	Ce	-1.9347	7.8190	0.7898	0.2222	0.7778	0.0345
5	Ce	5.8041	1.1170	0.7898	0.5556	0.1111	0.0345
6	Ce	3.8694	4.4680	0.7898	0.5556	0.4444	0.0345
7	Ce	1.9347	7.8190	0.7898	0.5556	0.7778	0.0345
8	Ce	9.6735	1.1170	0.7898	0.8889	0.1111	0.0345
9	Ce	7.7388	4.4680	0.7898	0.8889	0.4444	0.0345
10	Ce	5.8041	7.8190	0.7898	0.8889	0.7778	0.0345
11	Ce	0.0021	2.2522	3.9541	0.1122	0.2240	0.1727
12	Ce	-1.9162	5.5837	3.9433	0.1126	0.5554	0.1722
13	Ce	-3.8695	8.9286	3.9542	0.1107	0.8882	0.1727
14	Ce	3.8672	2.2521	3.9541	0.4452	0.2240	0.1727
15	Ce	0.0130	8.9080	3.9688	0.4442	0.8861	0.1733
16	Ce	7.7388	2.2415	3.9252	0.7782	0.2230	0.1714
17	Ce	5.7852	5.5841	3.9434	0.7761	0.5555	0.1722
18	Ce	3.8563	8.9080	3.9688	0.7753	0.8861	0.1733
19	Ce	-0.0002	0.0013	7.0933	0.0000	0.0001	0.3098
20	Ce	-1.9410	3.3447	7.1023	-0.0009	0.3327	0.3102
21	Ce	-3.8701	6.6951	7.0978	-0.0004	0.6660	0.3100
22	Ce	3.8691	0.0011	7.0930	0.3334	0.0001	0.3098
23	Ce	1.9345	3.3466	7.1043	0.3331	0.3329	0.3103
24	Ce	-0.1178	6.6500	7.1414	0.3206	0.6615	0.3119
25	Ce	7.7387	0.1262	7.0768	0.6729	0.0125	0.3091
26	Ce	5.8099	3.3448	7.1023	0.6669	0.3327	0.3102
27	Ce	3.9860	6.6506	7.1419	0.6742	0.6616	0.3119
28	O	0.0000	0.0000	0.0000	0.0000	0.0000	0.0000
29	O	-1.9347	3.3510	0.0000	-0.0000	0.3333	0.0000
30	O	-3.8694	6.7020	0.0000	-0.0000	0.6667	0.0000
31	O	3.8694	0.0000	0.0000	0.3333	0.0000	0.0000
32	O	1.9347	3.3510	0.0000	0.3333	0.3333	0.0000
33	O	0.0000	6.7020	0.0000	0.3333	0.6667	0.0000
34	O	7.7388	0.0000	0.0000	0.6667	0.0000	0.0000
35	O	5.8041	3.3510	0.0000	0.6667	0.3333	0.0000
36	O	3.8694	6.7020	0.0000	0.6667	0.6667	0.0000
37	O	-0.0000	2.2340	1.5798	0.1111	0.2222	0.0690
38	O	-1.9347	5.5850	1.5798	0.1111	0.5556	0.0690
39	O	-3.8694	8.9360	1.5798	0.1111	0.8889	0.0690
40	O	3.8694	2.2340	1.5798	0.4444	0.2222	0.0690

41	O	1.9347	5.5850	1.5798	0.4444	0.5556	0.0690
42	O	-0.0000	8.9360	1.5798	0.4444	0.8889	0.0690
43	O	7.7388	2.2340	1.5798	0.7778	0.2222	0.0690
44	O	5.8041	5.5850	1.5798	0.7778	0.5556	0.0690
45	O	3.8694	8.9360	1.5798	0.7778	0.8889	0.0690
46	O	1.9347	1.1170	3.1593	0.2222	0.1111	0.1380
47	O	0.0000	4.4680	3.1593	0.2222	0.4444	0.1380
48	O	-1.9347	7.8190	3.1593	0.2222	0.7778	0.1380
49	O	5.8041	1.1170	3.1593	0.5556	0.1111	0.1380
50	O	3.8694	4.4680	3.1593	0.5556	0.4444	0.1380
51	O	1.9347	7.8190	3.1593	0.5556	0.7778	0.1380
52	O	9.6735	1.1170	3.1593	0.8889	0.1111	0.1380
53	O	7.7388	4.4680	3.1593	0.8889	0.4444	0.1380
54	O	5.8041	7.8190	3.1593	0.8889	0.7778	0.1380
55	O	-5.8290	10.0269	4.7749	-0.0034	0.9974	0.2085
56	O	-1.9296	3.3518	4.7590	0.0005	0.3334	0.2078
57	O	-3.8695	6.7070	4.7614	0.0002	0.6672	0.2079
58	O	3.8938	-0.0261	4.7746	0.3341	-0.0026	0.2085
59	O	1.9348	3.3483	4.8345	0.3332	0.3331	0.2111
60	O	-0.0625	6.7115	4.8017	0.3284	0.6676	0.2097
61	O	7.7389	0.0186	4.7651	0.6676	0.0018	0.2081
62	O	5.7988	3.3520	4.7592	0.6663	0.3334	0.2078
63	O	3.9316	6.7124	4.8016	0.6725	0.6677	0.2097
64	O	-0.0079	2.1900	6.2939	0.1082	0.2179	0.2749
65	O	-1.9653	5.5732	6.3179	0.1079	0.5544	0.2759
66	O	-3.8694	8.9527	6.2918	0.1119	0.8905	0.2748
67	O	3.8770	2.1889	6.2940	0.4429	0.2177	0.2749
68	O	1.9346	5.7371	6.5021	0.4520	0.5707	0.2840
69	O	0.0603	8.9054	6.3699	0.4481	0.8858	0.2782
70	O	7.7390	2.2588	6.2762	0.7790	0.2247	0.2741
71	O	5.8334	5.5746	6.3180	0.7798	0.5545	0.2759
72	O	3.8081	8.9051	6.3700	0.7710	0.8858	0.2782
73	O	1.9345	1.1155	7.8820	0.2221	0.1110	0.3442
74	O	-0.0494	4.3864	7.8199	0.2139	0.4363	0.3415
75	O	-1.9770	7.8172	7.8692	0.2185	0.7776	0.3437
76	O	5.8279	1.1373	7.8832	0.5586	0.1131	0.3443
77	O	3.9176	4.3859	7.8198	0.5556	0.4363	0.3415
78	O	1.9353	8.0648	8.2798	0.5678	0.8022	0.3616
79	O	9.6503	1.1371	7.8832	0.8879	0.1131	0.3443
80	O	7.7380	4.4562	7.8984	0.8882	0.4433	0.3449
81	O	5.8451	7.8189	7.8694	0.8924	0.7778	0.3437
82	C	1.9336	7.1131	9.2255	0.5203	0.7076	0.4029
83	O	0.7951	6.6609	9.5686	0.3998	0.6626	0.4179
84	O	3.0701	6.6589	9.5712	0.5957	0.6624	0.4180

/db/jmorales/CoNi-alloy/Adsorptions/CO-CoNi(111)

a =			9.831199646				
b =			9.831199645902503				
c =			21.020299912				
alpha =			90.0				
beta =			90.0				
gamma =			120.0				
	Atom	X	Y	Z	X	Y	Z
1	Ni	0.0000	0.0000	0.0000	0.0000	0.0000	0.0000
2	Ni	-1.2289	2.1285	0.0000	0.0000	0.2500	0.0000
3	Ni	-2.4578	4.2570	0.0000	-0.0000	0.5000	0.0000
4	Ni	-3.6867	6.3856	0.0000	-0.0000	0.7500	0.0000
5	Ni	4.9156	0.0000	0.0000	0.5000	0.0000	0.0000
6	Ni	3.6867	2.1285	0.0000	0.5000	0.2500	0.0000
7	Ni	2.4578	4.2570	0.0000	0.5000	0.5000	0.0000
8	Ni	1.2289	6.3856	0.0000	0.5000	0.7500	0.0000
9	Ni	2.4578	1.4190	2.0068	0.3333	0.1667	0.0955
10	Ni	1.2289	3.5475	2.0068	0.3333	0.4167	0.0955
11	Ni	0.0000	5.6760	2.0068	0.3333	0.6667	0.0955
12	Ni	-1.2289	7.8046	2.0068	0.3333	0.9167	0.0955
13	Ni	7.3734	1.4190	2.0068	0.8333	0.1667	0.0955
14	Ni	6.1445	3.5475	2.0068	0.8333	0.4167	0.0955
15	Ni	4.9156	5.6760	2.0068	0.8333	0.6667	0.0955
16	Ni	3.6867	7.8046	2.0068	0.8333	0.9167	0.0955
17	Ni	0.0316	2.8586	4.0088	0.1711	0.3358	0.1907
18	Ni	1.2559	0.7292	4.0036	0.1706	0.0856	0.1905
19	Ni	-2.4327	7.1124	4.0143	0.1702	0.8354	0.1910
20	Ni	-1.1999	4.9848	4.0034	0.1707	0.5855	0.1905
21	Ni	4.9420	2.8584	4.0126	0.6705	0.3357	0.1909
22	Ni	6.1725	0.7283	4.0090	0.6706	0.0855	0.1907
23	Ni	2.4856	7.1116	4.0134	0.6705	0.8353	0.1909
24	Ni	3.7079	4.9821	4.0200	0.6697	0.5852	0.1912
25	Ni	0.0517	0.0364	6.0221	0.0074	0.0043	0.2865
26	Ni	-1.1949	2.1526	6.0198	0.0049	0.2528	0.2864
27	Ni	-2.4040	4.2899	6.0219	0.0074	0.5039	0.2865
28	Ni	-3.6372	6.4185	6.0266	0.0070	0.7539	0.2867
29	Ni	4.9653	0.0331	6.0226	0.5070	0.0039	0.2865
30	Ni	3.7521	2.1520	6.0194	0.5080	0.2528	0.2864
31	Ni	2.5184	4.2961	6.0838	0.5085	0.5046	0.2894
32	Ni	1.2782	6.4372	6.0196	0.5080	0.7561	0.2864
33	Co	1.2289	2.1285	0.0000	0.2500	0.2500	0.0000
34	Co	2.4578	0.0000	0.0000	0.2500	0.0000	0.0000
35	Co	-1.2289	6.3856	0.0000	0.2500	0.7500	0.0000
36	Co	0.0000	4.2570	0.0000	0.2500	0.5000	0.0000
37	Co	6.1445	2.1285	0.0000	0.7500	0.2500	0.0000
38	Co	7.3734	0.0000	0.0000	0.7500	0.0000	0.0000
39	Co	3.6867	6.3856	0.0000	0.7500	0.7500	0.0000
40	Co	4.9156	4.2570	0.0000	0.7500	0.5000	0.0000
41	Co	-1.2289	3.5475	2.0068	0.0833	0.4167	0.0955

42	Co	0.0000	1.4190	2.0068	0.0833	0.1667	0.0955
43	Co	-3.6867	7.8046	2.0068	0.0833	0.9167	0.0955
44	Co	-2.4578	5.6760	2.0068	0.0833	0.6667	0.0955
45	Co	3.6867	3.5475	2.0068	0.5833	0.4167	0.0955
46	Co	4.9156	1.4190	2.0068	0.5833	0.1667	0.0955
47	Co	1.2289	7.8046	2.0068	0.5833	0.9167	0.0955
48	Co	2.4578	5.6760	2.0068	0.5833	0.6667	0.0955
49	Co	3.7064	0.7268	4.0528	0.4197	0.0854	0.1928
50	Co	2.4820	2.8530	4.0590	0.4200	0.3351	0.1931
51	Co	1.2526	4.9835	4.0583	0.4201	0.5853	0.1931
52	Co	0.0221	7.1073	4.0530	0.4196	0.8348	0.1928
53	Co	8.6336	0.7272	4.0511	0.9209	0.0854	0.1927
54	Co	7.4028	2.8603	4.0507	0.9210	0.3359	0.1927
55	Co	6.1668	4.9841	4.0571	0.9200	0.5854	0.1930
56	Co	4.9390	7.1095	4.0572	0.9199	0.8350	0.1930
57	Co	1.2926	2.1635	6.0679	0.2585	0.2541	0.2887
58	Co	2.5271	0.0440	6.0137	0.2596	0.0052	0.2861
59	Co	-1.1597	6.4304	6.0138	0.2597	0.7553	0.2861
60	Co	0.0588	4.3010	6.0671	0.2586	0.5052	0.2886
61	Co	6.2147	2.1728	6.0136	0.7597	0.2552	0.2861
62	Co	7.4426	0.0451	6.0142	0.7597	0.0053	0.2861
63	Co	3.7576	6.4325	6.0114	0.7600	0.7555	0.2860
64	Co	4.9877	4.3017	6.0114	0.7600	0.5052	0.2860
65	O	2.6789	3.0844	8.6269	0.4536	0.3623	0.4104
66	C	2.6373	3.0606	7.4323	0.4480	0.3595	0.3536

/db/jmorales/CoNi-alloy/Adsorptions/Functionals/PBEsol/CoNi(111)-CO

a =			9.831199646				
b =			9.831199646075708				
c =			21.0202999115				
alpha =			90.0				
beta =			90.0				
gamma =			120.0				
	Atom	X	Y	Z	X	Y	Z
1	Ni	0.0000	0.0000	0.0000	0.0000	0.0000	0.0000
2	Ni	-1.2289	2.1285	0.0000	0.0000	0.2500	0.0000
3	Ni	-2.4578	4.2570	0.0000	0.0000	0.5000	0.0000
4	Ni	-3.6867	6.3856	0.0000	0.0000	0.7500	0.0000
5	Ni	4.9156	0.0000	0.0000	0.5000	0.0000	0.0000
6	Ni	3.6867	2.1285	0.0000	0.5000	0.2500	0.0000
7	Ni	2.4578	4.2570	0.0000	0.5000	0.5000	0.0000
8	Ni	1.2289	6.3856	0.0000	0.5000	0.7500	0.0000
9	Ni	2.4578	1.4190	2.0068	0.3333	0.1667	0.0955
10	Ni	1.2289	3.5475	2.0068	0.3333	0.4167	0.0955
11	Ni	0.0000	5.6760	2.0068	0.3333	0.6667	0.0955
12	Ni	-1.2289	7.8045	2.0068	0.3333	0.9167	0.0955
13	Ni	7.3734	1.4190	2.0068	0.8333	0.1667	0.0955
14	Ni	6.1445	3.5475	2.0068	0.8333	0.4167	0.0955
15	Ni	4.9156	5.6760	2.0068	0.8333	0.6667	0.0955
16	Ni	3.6867	7.8045	2.0068	0.8333	0.9167	0.0955
17	Ni	0.0298	2.8593	3.9610	0.1709	0.3358	0.1884
18	Ni	1.2616	0.7249	3.9595	0.1709	0.0851	0.1884
19	Ni	-2.4288	7.1134	3.9728	0.1707	0.8355	0.1890
20	Ni	-1.1986	4.9827	3.9725	0.1707	0.5852	0.1890
21	Ni	4.9415	2.8543	3.9765	0.6703	0.3352	0.1892
22	Ni	6.1726	0.7275	3.9708	0.6706	0.0854	0.1889
23	Ni	2.4877	7.1096	3.9713	0.6706	0.8350	0.1889
24	Ni	3.7133	4.9809	3.9768	0.6702	0.5850	0.1892
25	Ni	0.0521	0.0337	5.9270	0.0073	0.0040	0.2820
26	Ni	-1.1715	2.1560	5.9286	0.0075	0.2532	0.2820
27	Ni	-2.4042	4.2853	5.9300	0.0071	0.5033	0.2821
28	Ni	-3.6342	6.4177	5.9307	0.0072	0.7538	0.2821
29	Ni	4.9695	0.0305	5.9301	0.5073	0.0036	0.2821
30	Ni	3.7510	2.1698	5.9821	0.5090	0.2549	0.2846
31	Ni	2.5260	4.2898	5.9828	0.5089	0.5038	0.2846
32	Ni	1.2839	6.4166	5.9313	0.5074	0.7537	0.2822
33	Co	1.2289	2.1285	0.0000	0.2500	0.2500	0.0000
34	Co	2.4578	0.0000	0.0000	0.2500	0.0000	0.0000
35	Co	-1.2289	6.3856	0.0000	0.2500	0.7500	0.0000
36	Co	0.0000	4.2570	0.0000	0.2500	0.5000	0.0000
37	Co	6.1445	2.1285	0.0000	0.7500	0.2500	0.0000
38	Co	7.3734	0.0000	0.0000	0.7500	0.0000	0.0000
39	Co	3.6867	6.3856	0.0000	0.7500	0.7500	0.0000
40	Co	4.9156	4.2570	0.0000	0.7500	0.5000	0.0000
41	Co	-1.2289	3.5475	2.0068	0.0833	0.4167	0.0955

42	Co	0.0000	1.4190	2.0068	0.0833	0.1667	0.0955
43	Co	-3.6867	7.8045	2.0068	0.0833	0.9167	0.0955
44	Co	-2.4578	5.6760	2.0068	0.0833	0.6667	0.0955
45	Co	3.6867	3.5475	2.0068	0.5833	0.4167	0.0955
46	Co	4.9156	1.4190	2.0068	0.5833	0.1667	0.0955
47	Co	1.2289	7.8045	2.0068	0.5833	0.9167	0.0955
48	Co	2.4578	5.6760	2.0068	0.5833	0.6667	0.0955
49	Co	3.7092	0.7262	4.0067	0.4199	0.0853	0.1906
50	Co	2.4845	2.8525	3.9935	0.4202	0.3350	0.1900
51	Co	1.2542	4.9786	4.0079	0.4199	0.5847	0.1907
52	Co	0.0276	7.1099	4.0066	0.4203	0.8351	0.1906
53	Co	8.6336	0.7281	4.0023	0.9209	0.0855	0.1904
54	Co	7.3984	2.8522	4.0063	0.9200	0.3350	0.1906
55	Co	6.1706	4.9825	4.0055	0.9203	0.5852	0.1906
56	Co	4.9413	7.1077	4.0060	0.9200	0.8348	0.1906
57	Co	1.2887	2.1607	5.9548	0.2580	0.2538	0.2833
58	Co	2.5344	0.0171	5.9212	0.2588	0.0020	0.2817
59	Co	-1.1550	6.4272	5.9225	0.2600	0.7549	0.2817
60	Co	0.0542	4.3138	5.9217	0.2588	0.5067	0.2817
61	Co	6.2241	2.1683	5.9239	0.7604	0.2547	0.2818
62	Co	7.4493	0.0437	5.9258	0.7603	0.0051	0.2819
63	Co	3.7608	6.4345	5.9241	0.7604	0.7558	0.2818
64	Co	5.0107	4.3118	5.9200	0.7629	0.5064	0.2816
65	O	2.5059	2.8617	8.4858	0.4230	0.3361	0.4037
66	C	2.4388	2.8247	7.2954	0.4139	0.3318	0.3471

/db/jmorales/CoNi-alloy/Adsorptions/CO-Ni(111)

a =	9.829999924
b =	9.829999923668225
c =	21.019699097
alpha =	90.0
beta =	90.0
gamma =	120.0

	Atom	X	Y	Z	X	Y	Z
1	Ni	0.0000	0.0000	0.0000	0.0000	0.0000	0.0000
2	Ni	-1.2288	2.1283	0.0000	0.0000	0.2500	0.0000
3	Ni	-2.4575	4.2565	0.0000	-0.0000	0.5000	0.0000
4	Ni	-3.6862	6.3848	0.0000	-0.0000	0.7500	0.0000
5	Ni	2.4575	0.0000	0.0000	0.2500	0.0000	0.0000
6	Ni	1.2288	2.1283	0.0000	0.2500	0.2500	0.0000
7	Ni	-0.0000	4.2565	0.0000	0.2500	0.5000	0.0000
8	Ni	-1.2288	6.3848	0.0000	0.2500	0.7500	0.0000
9	Ni	4.9150	0.0000	0.0000	0.5000	0.0000	0.0000
10	Ni	3.6862	2.1283	0.0000	0.5000	0.2500	0.0000
11	Ni	2.4575	4.2565	0.0000	0.5000	0.5000	0.0000
12	Ni	1.2288	6.3848	0.0000	0.5000	0.7500	0.0000
13	Ni	7.3725	0.0000	0.0000	0.7500	0.0000	0.0000
14	Ni	6.1438	2.1283	0.0000	0.7500	0.2500	0.0000
15	Ni	4.9150	4.2565	0.0000	0.7500	0.5000	0.0000
16	Ni	3.6862	6.3848	0.0000	0.7500	0.7500	0.0000
17	Ni	-0.0000	1.4188	2.0065	0.0833	0.1667	0.0955
18	Ni	-1.2288	3.5471	2.0065	0.0833	0.4167	0.0955
19	Ni	-2.4575	5.6754	2.0065	0.0833	0.6667	0.0955
20	Ni	-3.6863	7.8036	2.0065	0.0833	0.9167	0.0955
21	Ni	2.4575	1.4188	2.0065	0.3333	0.1667	0.0955
22	Ni	1.2287	3.5471	2.0065	0.3333	0.4167	0.0955
23	Ni	-0.0000	5.6754	2.0065	0.3333	0.6667	0.0955
24	Ni	-1.2288	7.8036	2.0065	0.3333	0.9167	0.0955
25	Ni	4.9150	1.4188	2.0065	0.5833	0.1667	0.0955
26	Ni	3.6862	3.5471	2.0065	0.5833	0.4167	0.0955
27	Ni	2.4575	5.6754	2.0065	0.5833	0.6667	0.0955
28	Ni	1.2287	7.8036	2.0065	0.5833	0.9167	0.0955
29	Ni	7.3725	1.4188	2.0065	0.8333	0.1667	0.0955
30	Ni	6.1437	3.5471	2.0065	0.8333	0.4167	0.0955
31	Ni	4.9150	5.6754	2.0065	0.8333	0.6667	0.0955
32	Ni	3.6862	7.8036	2.0065	0.8333	0.9167	0.0955
33	Ni	1.2288	0.7094	4.0131	0.1667	0.0833	0.1909
34	Ni	0.0000	2.8377	4.0131	0.1667	0.3333	0.1909
35	Ni	-1.2287	4.9659	4.0131	0.1667	0.5833	0.1909
36	Ni	-2.4575	7.0942	4.0131	0.1667	0.8333	0.1909
37	Ni	3.6863	0.7094	4.0131	0.4167	0.0833	0.1909
38	Ni	2.4575	2.8377	4.0131	0.4167	0.3333	0.1909
39	Ni	1.2288	4.9659	4.0131	0.4167	0.5833	0.1909
40	Ni	0.0000	7.0942	4.0131	0.4167	0.8333	0.1909
41	Ni	6.1438	0.7094	4.0131	0.6667	0.0833	0.1909

42	Ni	4.9150	2.8377	4.0131	0.6667	0.3333	0.1909
43	Ni	3.6863	4.9659	4.0131	0.6667	0.5833	0.1909
44	Ni	2.4575	7.0942	4.0131	0.6667	0.8333	0.1909
45	Ni	8.6013	0.7094	4.0131	0.9167	0.0833	0.1909
46	Ni	7.3725	2.8377	4.0131	0.9167	0.3333	0.1909
47	Ni	6.1438	4.9659	4.0131	0.9167	0.5833	0.1909
48	Ni	4.9150	7.0942	4.0131	0.9167	0.8333	0.1909
49	Ni	0.0000	0.0000	6.0198	0.0000	0.0000	0.2864
50	Ni	-1.2288	2.1283	6.0198	0.0000	0.2500	0.2864
51	Ni	-2.4575	4.2565	6.0198	-0.0000	0.5000	0.2864
52	Ni	-3.6862	6.3848	6.0198	-0.0000	0.7500	0.2864
53	Ni	2.4575	0.0000	6.0198	0.2500	0.0000	0.2864
54	Ni	1.2288	2.1283	6.0198	0.2500	0.2500	0.2864
55	Ni	-0.0000	4.2565	6.0198	0.2500	0.5000	0.2864
56	Ni	-1.2288	6.3848	6.0198	0.2500	0.7500	0.2864
57	Ni	4.9150	0.0000	6.0198	0.5000	0.0000	0.2864
58	Ni	3.6862	2.1283	6.0198	0.5000	0.2500	0.2864
59	Ni	2.4575	4.2565	6.0198	0.5000	0.5000	0.2864
60	Ni	1.2288	6.3848	6.0198	0.5000	0.7500	0.2864
61	Ni	7.3725	0.0000	6.0198	0.7500	0.0000	0.2864
62	Ni	6.1438	2.1283	6.0198	0.7500	0.2500	0.2864
63	Ni	4.9150	4.2565	6.0198	0.7500	0.5000	0.2864
64	Ni	3.6862	6.3848	6.0198	0.7500	0.7500	0.2864
65	O	1.2000	3.7000	8.2752	0.3394	0.4346	0.3937
66	C	1.2000	3.7000	7.1248	0.3394	0.4346	0.3390

/db/jmorales/CoNi-alloy/Adsorptions/Functionals/RPBE/Ni(001)-CO

a =			6.9509918686				
b =			6.9509918686				
c =			21.9509918687				
alpha =			90.0				
beta =			90.0				
gamma =			90.0				
	Atom	X	Y	Z	X	Y	Z
1	Ni	0.0000	0.0000	0.0000	0.0000	0.0000	0.0000
2	Ni	1.7377	1.7377	0.0000	0.2500	0.2500	0.0000
3	Ni	0.0000	3.4755	0.0000	0.0000	0.5000	0.0000
4	Ni	1.7377	5.2132	0.0000	0.2500	0.7500	0.0000
5	Ni	3.4755	0.0000	0.0000	0.5000	0.0000	0.0000
6	Ni	5.2132	1.7377	0.0000	0.7500	0.2500	0.0000
7	Ni	3.4755	3.4755	0.0000	0.5000	0.5000	0.0000
8	Ni	5.2132	5.2132	0.0000	0.7500	0.7500	0.0000
9	Ni	0.0000	1.7377	1.7377	0.0000	0.2500	0.0792
10	Ni	1.7377	0.0000	1.7377	0.2500	0.0000	0.0792
11	Ni	0.0000	5.2132	1.7377	0.0000	0.7500	0.0792
12	Ni	1.7377	3.4755	1.7377	0.2500	0.5000	0.0792
13	Ni	3.4755	1.7377	1.7377	0.5000	0.2500	0.0792
14	Ni	5.2132	0.0000	1.7377	0.7500	0.0000	0.0792
15	Ni	3.4755	5.2132	1.7377	0.5000	0.7500	0.0792
16	Ni	5.2132	3.4755	1.7377	0.7500	0.5000	0.0792
17	Ni	6.9508	0.0003	3.5183	1.0000	0.0000	0.1603
18	Ni	1.7369	1.7372	3.5125	0.2499	0.2499	0.1600
19	Ni	6.9508	3.4756	3.5227	1.0000	0.5000	0.1605
20	Ni	1.7373	5.2137	3.5124	0.2499	0.7501	0.1600
21	Ni	3.4754	0.0001	3.5227	0.5000	0.0000	0.1605
22	Ni	5.2140	1.7370	3.5125	0.7501	0.2499	0.1600
23	Ni	3.4755	3.4755	3.4723	0.5000	0.5000	0.1582
24	Ni	5.2135	5.2139	3.5126	0.7500	0.7501	0.1600
25	Ni	-0.0002	1.7386	5.2528	-0.0000	0.2501	0.2393
26	Ni	1.7383	0.0002	5.2527	0.2501	0.0000	0.2393
27	Ni	-0.0001	5.2126	5.2528	-0.0000	0.7499	0.2393
28	Ni	1.7559	3.4756	5.2948	0.2526	0.5000	0.2412
29	Ni	3.4753	1.7558	5.2954	0.5000	0.2526	0.2412
30	Ni	5.2123	0.0001	5.2529	0.7499	0.0000	0.2393
31	Ni	3.4753	5.1950	5.2948	0.5000	0.7474	0.2412
32	Ni	5.1950	3.4757	5.2952	0.7474	0.5000	0.2412
33	O	3.4750	3.4760	7.6272	0.4999	0.5001	0.3475
34	C	3.4754	3.4755	6.4094	0.5000	0.5000	0.2920

/db/jmorales/CoNi-alloy/Adsorptions/Coverage/CoNi(111)-2CO

a =	9.831199646
b =	9.831199646075708
c =	21.0202999115
alpha =	90.0
beta =	90.0
gamma =	120.0

	Atom	X	Y	Z	X	Y	Z
1	Ni	0.0000	0.0000	0.0000	0.0000	0.0000	0.0000
2	Ni	-1.2289	2.1285	0.0000	0.0000	0.2500	0.0000
3	Ni	-2.4578	4.2570	0.0000	0.0000	0.5000	0.0000
4	Ni	-3.6867	6.3856	0.0000	0.0000	0.7500	0.0000
5	Ni	4.9156	0.0000	0.0000	0.5000	0.0000	0.0000
6	Ni	3.6867	2.1285	0.0000	0.5000	0.2500	0.0000
7	Ni	2.4578	4.2570	0.0000	0.5000	0.5000	0.0000
8	Ni	1.2289	6.3856	0.0000	0.5000	0.7500	0.0000
9	Ni	2.4578	1.4190	2.0068	0.3333	0.1667	0.0955
10	Ni	1.2289	3.5475	2.0068	0.3333	0.4167	0.0955
11	Ni	0.0000	5.6760	2.0068	0.3333	0.6667	0.0955
12	Ni	-1.2289	7.8045	2.0068	0.3333	0.9167	0.0955
13	Ni	7.3734	1.4190	2.0068	0.8333	0.1667	0.0955
14	Ni	6.1445	3.5475	2.0068	0.8333	0.4167	0.0955
15	Ni	4.9156	5.6760	2.0068	0.8333	0.6667	0.0955
16	Ni	3.6867	7.8045	2.0068	0.8333	0.9167	0.0955
17	Ni	0.0398	2.8616	4.0010	0.1721	0.3361	0.1903
18	Ni	1.2640	0.7324	4.0050	0.1716	0.0860	0.1905
19	Ni	-2.4201	7.1170	4.0272	0.1718	0.8359	0.1916
20	Ni	-1.1929	4.9852	4.0045	0.1714	0.5855	0.1905
21	Ni	4.9439	2.8530	4.0186	0.6704	0.3351	0.1912
22	Ni	6.1770	0.7279	4.0096	0.6711	0.0855	0.1908
23	Ni	2.4861	7.1130	4.0176	0.6706	0.8354	0.1911
24	Ni	3.7166	4.9837	4.0219	0.6707	0.5854	0.1913
25	Ni	0.0519	0.0316	6.0170	0.0071	0.0037	0.2862
26	Ni	-1.1610	2.1679	6.0173	0.0092	0.2546	0.2863
27	Ni	-2.4019	4.2864	6.0192	0.0074	0.5035	0.2864
28	Ni	-3.6329	6.4164	6.0206	0.0073	0.7536	0.2864
29	Ni	4.9906	0.0393	6.0146	0.5099	0.0046	0.2861
30	Ni	3.7586	2.1731	6.0891	0.5099	0.2552	0.2897
31	Ni	2.5397	4.3075	6.0267	0.5113	0.5059	0.2867
32	Ni	1.3055	6.4253	6.0878	0.5101	0.7547	0.2896
33	Co	1.2289	2.1285	0.0000	0.2500	0.2500	0.0000
34	Co	2.4578	0.0000	0.0000	0.2500	0.0000	0.0000
35	Co	-1.2289	6.3856	0.0000	0.2500	0.7500	0.0000
36	Co	0.0000	4.2570	0.0000	0.2500	0.5000	0.0000
37	Co	6.1445	2.1285	0.0000	0.7500	0.2500	0.0000
38	Co	7.3734	0.0000	0.0000	0.7500	0.0000	0.0000
39	Co	3.6867	6.3856	0.0000	0.7500	0.7500	0.0000
40	Co	4.9156	4.2570	0.0000	0.7500	0.5000	0.0000
41	Co	-1.2289	3.5475	2.0068	0.0833	0.4167	0.0955

42	Co	0.0000	1.4190	2.0068	0.0833	0.1667	0.0955
43	Co	-3.6867	7.8045	2.0068	0.0833	0.9167	0.0955
44	Co	-2.4578	5.6760	2.0068	0.0833	0.6667	0.0955
45	Co	3.6867	3.5475	2.0068	0.5833	0.4167	0.0955
46	Co	4.9156	1.4190	2.0068	0.5833	0.1667	0.0955
47	Co	1.2289	7.8045	2.0068	0.5833	0.9167	0.0955
48	Co	2.4578	5.6760	2.0068	0.5833	0.6667	0.0955
49	Co	3.7178	0.7271	4.0524	0.4209	0.0854	0.1928
50	Co	2.4877	2.8596	4.0444	0.4210	0.3359	0.1924
51	Co	1.2619	4.9826	4.0418	0.4210	0.5852	0.1923
52	Co	0.0303	7.1120	4.0519	0.4207	0.8353	0.1928
53	Co	8.6366	0.7300	4.0503	0.9214	0.0857	0.1927
54	Co	7.4075	2.8579	4.0504	0.9213	0.3357	0.1927
55	Co	6.1777	4.9829	4.0511	0.9210	0.5853	0.1927
56	Co	4.9477	7.1156	4.0494	0.9211	0.8357	0.1926
57	Co	1.2944	2.1612	6.0643	0.2586	0.2538	0.2885
58	Co	2.5391	0.0195	6.0311	0.2594	0.0023	0.2869
59	Co	-1.1704	6.4454	6.0251	0.2595	0.7570	0.2866
60	Co	0.0636	4.2954	6.0613	0.2587	0.5045	0.2884
61	Co	6.2246	2.1735	6.0095	0.7608	0.2553	0.2859
62	Co	7.4527	0.0466	6.0104	0.7608	0.0055	0.2859
63	Co	3.7739	6.4396	6.0079	0.7620	0.7564	0.2858
64	Co	5.0038	4.3031	6.0092	0.7617	0.5054	0.2859
65	O	2.5373	1.8672	8.6873	0.3677	0.2193	0.4133
66	O	0.6497	5.4019	8.6962	0.3833	0.6345	0.4137
67	C	2.4469	1.9186	7.5050	0.3616	0.2253	0.3570
68	C	0.5865	5.3131	7.5154	0.3717	0.6240	0.3575

/db/jmorales/CoNi-alloy/Adsorptions/CO-Sm(surf)$Ce_{26}O_{53}$

a = 11.6082000732
b = 11.608200073110298
c = 22.898399353
alpha = 90.0
beta = 90.0
gamma = 120.0

	Atom	X	Y	Z	X	Y	Z
1	Sm	1.9655	3.1646	7.2219	0.3267	0.3148	0.3154
2	Ce	1.9347	1.1170	0.7898	0.2222	0.1111	0.0345
3	Ce	0.0000	4.4680	0.7898	0.2222	0.4444	0.0345
4	Ce	-1.9347	7.8190	0.7898	0.2222	0.7778	0.0345
5	Ce	5.8041	1.1170	0.7898	0.5556	0.1111	0.0345
6	Ce	3.8694	4.4680	0.7898	0.5556	0.4444	0.0345
7	Ce	1.9347	7.8190	0.7898	0.5556	0.7778	0.0345
8	Ce	9.6735	1.1170	0.7898	0.8889	0.1111	0.0345
9	Ce	7.7388	4.4680	0.7898	0.8889	0.4444	0.0345
10	Ce	5.8041	7.8190	0.7898	0.8889	0.7778	0.0345
11	Ce	0.0066	2.2405	3.9843	0.1120	0.2229	0.1740
12	Ce	-1.9368	5.5847	3.9602	0.1109	0.5555	0.1729
13	Ce	-3.8701	8.9351	3.9449	0.1110	0.8888	0.1723
14	Ce	3.8634	2.2361	3.9728	0.4440	0.2224	0.1735
15	Ce	1.9466	5.5752	3.8591	0.4450	0.5546	0.1685
16	Ce	-0.0005	8.9392	3.9568	0.4446	0.8892	0.1728
17	Ce	7.7354	2.2350	3.9379	0.7775	0.2223	0.1720
18	Ce	5.8000	5.5862	3.9767	0.7775	0.5557	0.1737
19	Ce	3.8662	8.9311	3.9833	0.7773	0.8884	0.1740
20	Ce	0.0080	-0.0001	7.1073	0.0007	-0.0000	0.3104
21	Ce	9.6557	3.3432	7.0860	0.9981	0.3326	0.3095
22	Ce	-3.8702	6.7069	7.1020	0.0002	0.6672	0.3102
23	Ce	3.8563	-0.0014	7.0902	0.3321	-0.0001	0.3096
24	Ce	-0.1288	6.7817	7.1858	0.3262	0.6746	0.3138
25	Ce	7.7294	0.0219	7.0930	0.6669	0.0022	0.3098
26	Ce	5.7908	3.3475	7.0981	0.6654	0.3330	0.3100
27	Ce	3.9998	6.7472	7.2750	0.6801	0.6712	0.3177
28	O	0.0000	0.0000	0.0000	0.0000	0.0000	0.0000
29	O	-1.9347	3.3510	0.0000	-0.0000	0.3333	0.0000
30	O	-3.8694	6.7020	0.0000	-0.0000	0.6667	0.0000
31	O	3.8694	0.0000	0.0000	0.3333	0.0000	0.0000
32	O	1.9347	3.3510	0.0000	0.3333	0.3333	0.0000
33	O	0.0000	6.7020	0.0000	0.3333	0.6667	0.0000
34	O	7.7388	0.0000	0.0000	0.6667	0.0000	0.0000
35	O	5.8041	3.3510	0.0000	0.6667	0.3333	0.0000
36	O	3.8694	6.7020	0.0000	0.6667	0.6667	0.0000
37	O	-0.0000	2.2340	1.5798	0.1111	0.2222	0.0690
38	O	-1.9347	5.5850	1.5798	0.1111	0.5556	0.0690
39	O	-3.8694	8.9360	1.5798	0.1111	0.8889	0.0690
40	O	3.8694	2.2340	1.5798	0.4444	0.2222	0.0690

41	O	1.9347	5.5850	1.5798	0.4444	0.5556	0.0690
42	O	-0.0000	8.9360	1.5798	0.4444	0.8889	0.0690
43	O	7.7388	2.2340	1.5798	0.7778	0.2222	0.0690
44	O	5.8041	5.5850	1.5798	0.7778	0.5556	0.0690
45	O	3.8694	8.9360	1.5798	0.7778	0.8889	0.0690
46	O	1.9347	1.1170	3.1593	0.2222	0.1111	0.1380
47	O	0.0000	4.4680	3.1593	0.2222	0.4444	0.1380
48	O	-1.9347	7.8190	3.1593	0.2222	0.7778	0.1380
49	O	5.8041	1.1170	3.1593	0.5556	0.1111	0.1380
50	O	3.8694	4.4680	3.1593	0.5556	0.4444	0.1380
51	O	1.9347	7.8190	3.1593	0.5556	0.7778	0.1380
52	O	9.6735	1.1170	3.1593	0.8889	0.1111	0.1380
53	O	7.7388	4.4680	3.1593	0.8889	0.4444	0.1380
54	O	5.8041	7.8190	3.1593	0.8889	0.7778	0.1380
55	O	0.0038	-0.0005	4.7405	0.0003	-0.0000	0.2070
56	O	-1.9281	3.3658	4.7569	0.0013	0.3348	0.2077
57	O	-3.8633	6.7021	4.7224	0.0005	0.6667	0.2062
58	O	-1.9375	10.0436	4.7137	0.3326	0.9991	0.2059
59	O	1.9382	3.4910	4.8535	0.3406	0.3473	0.2120
60	O	0.1315	6.6302	4.8717	0.3411	0.6595	0.2128
61	O	1.9240	10.0407	4.7560	0.6651	0.9988	0.2077
62	O	5.7913	3.3567	4.7507	0.6658	0.3339	0.2075
63	O	3.7493	6.6301	4.8516	0.6527	0.6595	0.2119
64	O	-0.0425	2.1882	6.3163	0.1052	0.2177	0.2758
65	O	-1.9847	5.6120	6.2915	0.1081	0.5582	0.2748
66	O	-3.8531	8.9234	6.2547	0.1119	0.8876	0.2731
67	O	3.8995	2.1451	6.2775	0.4426	0.2134	0.2741
68	O	-0.0587	8.9729	6.2817	0.4412	0.8926	0.2743
69	O	7.7439	2.2327	6.2651	0.7782	0.2221	0.2736
70	O	5.9025	5.5829	6.2935	0.7861	0.5553	0.2748
71	O	3.8887	9.0159	6.3152	0.7834	0.8968	0.2758
72	O	1.9389	0.9871	7.9464	0.2161	0.0982	0.3470
73	O	0.1390	4.6468	7.7184	0.2431	0.4622	0.3371
74	O	-1.9713	7.8375	7.9594	0.2200	0.7796	0.3476
75	O	5.8445	1.1135	7.9083	0.5589	0.1108	0.3454
76	O	3.8927	4.4134	7.8194	0.5549	0.4390	0.3415
77	O	1.8208	7.7152	7.7793	0.5406	0.7675	0.3397
78	O	9.6206	1.1595	7.9337	0.8864	0.1153	0.3465
79	O	7.7797	4.4276	7.9120	0.8904	0.4404	0.3455
80	O	5.9338	7.8886	7.9761	0.9035	0.7847	0.3483
81	O	1.8952	3.6609	11.1424	0.3453	0.3642	0.4866
82	C	1.8916	3.4498	10.0198	0.3345	0.3432	0.4376

/db/jmorales/CoNi-alloy/Adsorptions/CO_2-Ni(001)

a =			6.950991869				
b =			6.950991869				
c =			21.950991869				
alpha =			90.0				
beta =			90.0				
gamma =			90.0				
	Atom	X	Y	Z	X	Y	Z
1	Ni	0.0000	0.0000	0.0000	0.0000	0.0000	0.0000
2	Ni	1.7378	1.7378	0.0000	0.2500	0.2500	0.0000
3	Ni	0.0000	3.4755	0.0000	0.0000	0.5000	0.0000
4	Ni	1.7378	5.2132	0.0000	0.2500	0.7500	0.0000
5	Ni	3.4755	0.0000	0.0000	0.5000	0.0000	0.0000
6	Ni	5.2132	1.7378	0.0000	0.7500	0.2500	0.0000
7	Ni	3.4755	3.4755	0.0000	0.5000	0.5000	0.0000
8	Ni	5.2132	5.2132	0.0000	0.7500	0.7500	0.0000
9	Ni	0.0000	1.7378	1.7378	0.0000	0.2500	0.0792
10	Ni	1.7378	0.0000	1.7378	0.2500	0.0000	0.0792
11	Ni	0.0000	5.2132	1.7378	0.0000	0.7500	0.0792
12	Ni	1.7378	3.4755	1.7378	0.2500	0.5000	0.0792
13	Ni	3.4755	1.7378	1.7378	0.5000	0.2500	0.0792
14	Ni	5.2132	0.0000	1.7378	0.7500	0.0000	0.0792
15	Ni	3.4755	5.2132	1.7378	0.5000	0.7500	0.0792
16	Ni	5.2132	3.4755	1.7378	0.7500	0.5000	0.0792
17	Ni	0.0050	0.0001	3.4764	0.0007	0.0000	0.1584
18	Ni	1.7479	1.7571	3.5157	0.2515	0.2528	0.1602
19	Ni	6.9446	3.4723	3.5083	0.9991	0.4995	0.1598
20	Ni	1.7448	5.2009	3.5015	0.2510	0.7482	0.1595
21	Ni	3.4742	0.0093	3.5120	0.4998	0.0013	0.1600
22	Ni	5.2097	1.7424	3.5044	0.7495	0.2507	0.1596
23	Ni	3.4728	3.4676	3.5071	0.4996	0.4989	0.1598
24	Ni	5.2035	5.1991	3.4938	0.7486	0.7480	0.1592
25	Ni	0.0055	1.6718	5.1918	0.0008	0.2405	0.2365
26	Ni	1.7550	6.9455	5.1977	0.2525	0.9992	0.2368
27	Ni	6.9420	5.2754	5.1851	0.9987	0.7589	0.2362
28	Ni	1.6250	3.4866	5.3156	0.2338	0.5016	0.2422
29	Ni	3.4964	1.6893	5.2921	0.5030	0.2430	0.2411
30	Ni	5.2034	6.9146	5.1923	0.7486	0.9948	0.2365
31	Ni	3.4667	5.1756	5.2800	0.4987	0.7446	0.2405
32	Ni	5.3190	3.4923	5.3026	0.7652	0.5024	0.2416
33	C	3.0948	3.8408	6.6423	0.4452	0.5526	0.3026
34	O	2.1439	3.9476	7.4480	0.3084	0.5679	0.3393
35	O	4.1054	2.9593	6.7532	0.5906	0.4257	0.3076

/db/jmorales/CoNi-alloy/Adsorptions/Functionals/RPBE/Ni(111)-CO

a =		9.8299999236					
b =		9.829999923841429					
c =		21.0196990967					
alpha =		90.0					
beta =		90.0					
gamma =		120.0					
	Atom	X	Y	Z	X	Y	Z
1	Ni	0.0000	0.0000	0.0000	0.0000	0.0000	0.0000
2	Ni	-1.2287	2.1283	0.0000	0.0000	0.2500	0.0000
3	Ni	-2.4575	4.2565	0.0000	0.0000	0.5000	0.0000
4	Ni	-3.6862	6.3848	0.0000	-0.0000	0.7500	0.0000
5	Ni	2.4575	0.0000	0.0000	0.2500	0.0000	0.0000
6	Ni	1.2287	2.1283	0.0000	0.2500	0.2500	0.0000
7	Ni	0.0000	4.2565	0.0000	0.2500	0.5000	0.0000
8	Ni	-1.2287	6.3848	0.0000	0.2500	0.7500	0.0000
9	Ni	4.9150	0.0000	0.0000	0.5000	0.0000	0.0000
10	Ni	3.6862	2.1283	0.0000	0.5000	0.2500	0.0000
11	Ni	2.4575	4.2565	0.0000	0.5000	0.5000	0.0000
12	Ni	1.2287	6.3848	0.0000	0.5000	0.7500	0.0000
13	Ni	7.3725	0.0000	0.0000	0.7500	0.0000	0.0000
14	Ni	6.1437	2.1283	0.0000	0.7500	0.2500	0.0000
15	Ni	4.9150	4.2565	0.0000	0.7500	0.5000	0.0000
16	Ni	3.6862	6.3848	0.0000	0.7500	0.7500	0.0000
17	Ni	-0.0000	1.4188	2.0065	0.0833	0.1667	0.0955
18	Ni	-1.2288	3.5471	2.0065	0.0833	0.4167	0.0955
19	Ni	-2.4575	5.6754	2.0065	0.0833	0.6667	0.0955
20	Ni	-3.6863	7.8036	2.0065	0.0833	0.9167	0.0955
21	Ni	2.4575	1.4188	2.0065	0.3333	0.1667	0.0955
22	Ni	1.2287	3.5471	2.0065	0.3333	0.4167	0.0955
23	Ni	-0.0000	5.6754	2.0065	0.3333	0.6667	0.0955
24	Ni	-1.2288	7.8036	2.0065	0.3333	0.9167	0.0955
25	Ni	4.9150	1.4188	2.0065	0.5833	0.1667	0.0955
26	Ni	3.6862	3.5471	2.0065	0.5833	0.4167	0.0955
27	Ni	2.4575	5.6754	2.0065	0.5833	0.6667	0.0955
28	Ni	1.2287	7.8036	2.0065	0.5833	0.9167	0.0955
29	Ni	7.3725	1.4188	2.0065	0.8333	0.1667	0.0955
30	Ni	6.1437	3.5471	2.0065	0.8333	0.4167	0.0955
31	Ni	4.9150	5.6754	2.0065	0.8333	0.6667	0.0955
32	Ni	3.6862	7.8036	2.0065	0.8333	0.9167	0.0955
33	Ni	1.2288	0.7217	4.0269	0.1674	0.0848	0.1916
34	Ni	0.0048	2.8419	4.0240	0.1674	0.3338	0.1914
35	Ni	-1.2204	4.9629	4.0254	0.1673	0.5830	0.1915
36	Ni	-2.4579	7.0963	4.0212	0.1667	0.8336	0.1913
37	Ni	3.6823	0.7130	4.0143	0.4165	0.0838	0.1910
38	Ni	2.4532	2.8414	4.0224	0.4164	0.3338	0.1914
39	Ni	1.2285	4.9626	4.0213	0.4164	0.5829	0.1913
40	Ni	-0.0006	7.0913	4.0144	0.4164	0.8330	0.1910
41	Ni	6.1439	0.7109	4.0155	0.6668	0.0835	0.1910

42	Ni	4.9110	2.8423	4.0179	0.6665	0.3339	0.1912
43	Ni	3.6785	4.9626	4.0274	0.6657	0.5829	0.1916
44	Ni	2.4579	7.0898	4.0178	0.6665	0.8328	0.1911
45	Ni	8.6046	0.7129	4.0144	0.9172	0.0837	0.1910
46	Ni	7.3764	2.8404	4.0141	0.9172	0.3337	0.1910
47	Ni	6.1435	4.9676	4.0179	0.9167	0.5835	0.1912
48	Ni	4.9147	7.0953	4.0184	0.9167	0.8335	0.1912
49	Ni	0.0010	0.0056	6.0408	0.0004	0.0007	0.2874
50	Ni	8.5782	2.1177	6.0406	0.9970	0.2488	0.2874
51	Ni	-2.4555	4.2598	6.0392	0.0004	0.5004	0.2873
52	Ni	-3.6871	6.3875	6.0482	0.0001	0.7503	0.2877
53	Ni	2.4559	0.0047	6.0472	0.2501	0.0006	0.2877
54	Ni	1.2296	2.1320	6.1235	0.2503	0.2504	0.2913
55	Ni	-0.0012	4.2595	6.1196	0.2500	0.5003	0.2911
56	Ni	-1.2289	6.3855	6.0462	0.2500	0.7501	0.2876
57	Ni	4.9119	0.0029	6.0421	0.4999	0.0003	0.2874
58	Ni	3.7089	2.1176	6.0414	0.5017	0.2487	0.2874
59	Ni	2.4577	4.2618	6.1177	0.5003	0.5006	0.2910
60	Ni	1.2270	6.4157	6.0408	0.5016	0.7536	0.2874
61	Ni	7.3731	0.0025	6.0475	0.7502	0.0003	0.2877
62	Ni	6.1439	2.1324	6.0484	0.7503	0.2505	0.2877
63	Ni	4.9138	4.2598	6.0479	0.7501	0.5004	0.2877
64	Ni	3.6854	6.3864	6.0472	0.7500	0.7502	0.2877
65	O	1.2248	3.5607	8.6690	0.3337	0.4183	0.4124
66	C	1.2268	3.5565	7.4705	0.3337	0.4178	0.3554

/db/jmorales/CoNi-alloy/Adsorptions/CO-Co-CoNi(001)

a =				6.951669213			
b =				6.951669213			
c =				21.951669213			
alpha =				90.0			
beta =				90.0			
gamma =				90.0			
	Atom	X	Y	Z	X	Y	Z
1	Ni	0.0000	0.0000	0.0000	0.0000	0.0000	0.0000
2	Ni	1.7379	1.7379	0.0000	0.2500	0.2500	0.0000
3	Ni	0.0000	3.4758	0.0000	0.0000	0.5000	0.0000
4	Ni	1.7379	5.2138	0.0000	0.2500	0.7500	0.0000
5	Ni	3.4758	0.0000	0.0000	0.5000	0.0000	0.0000
6	Ni	5.2138	1.7379	0.0000	0.7500	0.2500	0.0000
7	Ni	3.4758	3.4758	0.0000	0.5000	0.5000	0.0000
8	Ni	5.2138	5.2138	0.0000	0.7500	0.7500	0.0000
9	Ni	6.9516	0.0002	3.5116	1.0000	0.0000	0.1600
10	Ni	1.7350	1.7357	3.4758	0.2496	0.2497	0.1583
11	Ni	6.9510	3.4759	3.5213	0.9999	0.5000	0.1604
12	Ni	1.7352	5.2163	3.4758	0.2496	0.7504	0.1583
13	Ni	3.4756	0.0002	3.5214	0.5000	0.0000	0.1604
14	Ni	5.2157	1.7357	3.4758	0.7503	0.2497	0.1583
15	Ni	3.4757	3.4760	3.4835	0.5000	0.5000	0.1587
16	Ni	5.2156	5.2163	3.4758	0.7503	0.7504	0.1583
17	Co	1.7379	0.0000	1.7379	0.2500	0.0000	0.0792
18	Co	0.0000	1.7379	1.7379	0.0000	0.2500	0.0792
19	Co	1.7379	3.4758	1.7379	0.2500	0.5000	0.0792
20	Co	0.0000	5.2138	1.7379	0.0000	0.7500	0.0792
21	Co	5.2138	0.0000	1.7379	0.7500	0.0000	0.0792
22	Co	3.4758	1.7379	1.7379	0.5000	0.2500	0.0792
23	Co	5.2138	3.4758	1.7379	0.7500	0.5000	0.0792
24	Co	3.4758	5.2138	1.7379	0.5000	0.7500	0.0792
25	Co	1.7374	0.0003	5.2419	0.2499	0.0000	0.2388
26	Co	6.9508	1.7384	5.2420	0.9999	0.2501	0.2388
27	Co	1.7540	3.4760	5.2694	0.2523	0.5000	0.2400
28	Co	6.9508	5.2138	5.2420	0.9999	0.7500	0.2388
29	Co	5.2128	0.0003	5.2421	0.7499	0.0000	0.2388
30	Co	3.4752	1.7551	5.2706	0.4999	0.2525	0.2401
31	Co	5.1959	3.4760	5.2708	0.7474	0.5000	0.2401
32	Co	3.4753	5.1973	5.2701	0.4999	0.7476	0.2401
33	O	3.4719	3.4771	7.6461	0.4994	0.5002	0.3483
34	C	3.4741	3.4764	6.4327	0.4998	0.5001	0.2930

/db/jmorales/CoNi-alloy/Adsorptions/Functionals/PBEsol/Ni(001)

a =				6.9509918686			
b =				6.9509918686			
c =				21.9509918687			
alpha =				90.0			
beta =				90.0			
gamma =				90.0			
		Atom	X	Y	Z	X	Y	Z

		Atom	X	Y	Z	X	Y	Z
1	Ni		0.0000	0.0000	0.0000	0.0000	0.0000	0.0000
2	Ni		1.7377	1.7377	0.0000	0.2500	0.2500	0.0000
3	Ni		0.0000	3.4755	0.0000	0.0000	0.5000	0.0000
4	Ni		1.7377	5.2132	0.0000	0.2500	0.7500	0.0000
5	Ni		3.4755	0.0000	0.0000	0.5000	0.0000	0.0000
6	Ni		5.2132	1.7377	0.0000	0.7500	0.2500	0.0000
7	Ni		3.4755	3.4755	0.0000	0.5000	0.5000	0.0000
8	Ni		5.2132	5.2132	0.0000	0.7500	0.7500	0.0000
9	Ni		0.0000	1.7377	1.7377	0.0000	0.2500	0.0792
10	Ni		1.7377	0.0000	1.7377	0.2500	0.0000	0.0792
11	Ni		0.0000	5.2132	1.7377	0.0000	0.7500	0.0792
12	Ni		1.7377	3.4755	1.7377	0.2500	0.5000	0.0792
13	Ni		3.4755	1.7377	1.7377	0.5000	0.2500	0.0792
14	Ni		5.2132	0.0000	1.7377	0.7500	0.0000	0.0792
15	Ni		3.4755	5.2132	1.7377	0.5000	0.7500	0.0792
16	Ni		5.2132	3.4755	1.7377	0.7500	0.5000	0.0792
17	Ni		0.0000	0.0000	3.4514	0.0000	0.0000	0.1572
18	Ni		1.7377	1.7377	3.4514	0.2500	0.2500	0.1572
19	Ni		0.0000	3.4755	3.4514	0.0000	0.5000	0.1572
20	Ni		1.7377	5.2132	3.4514	0.2500	0.7500	0.1572
21	Ni		3.4755	0.0000	3.4514	0.5000	0.0000	0.1572
22	Ni		5.2132	1.7377	3.4514	0.7500	0.2500	0.1572
23	Ni		3.4755	3.4755	3.4514	0.5000	0.5000	0.1572
24	Ni		5.2132	5.2132	3.4514	0.7500	0.7500	0.1572
25	Ni		0.0000	1.7377	5.1042	0.0000	0.2500	0.2325
26	Ni		1.7377	0.0000	5.1042	0.2500	0.0000	0.2325
27	Ni		0.0000	5.2132	5.1042	0.0000	0.7500	0.2325
28	Ni		1.7377	3.4755	5.1042	0.2500	0.5000	0.2325
29	Ni		3.4755	1.7377	5.1042	0.5000	0.2500	0.2325
30	Ni		5.2132	0.0000	5.1042	0.7500	0.0000	0.2325
31	Ni		3.4755	5.2132	5.1042	0.5000	0.7500	0.2325
32	Ni		5.2132	3.4755	5.1042	0.7500	0.5000	0.2325

/db/jmorales/CoNi-alloy/Adsorptions/CO-Ni-CoNi(001)

a =		6.951669213					
b =		6.951669213					
c =		21.951669213					
alpha =		90.0					
beta =		90.0					
gamma =		90.0					
	Atom	X	Y	Z	X	Y	Z
1	Co	0.0000	0.0000	0.0000	0.0000	0.0000	0.0000
2	Co	1.7379	1.7379	0.0000	0.2500	0.2500	0.0000
3	Co	0.0000	3.4758	0.0000	0.0000	0.5000	0.0000
4	Co	1.7379	5.2138	0.0000	0.2500	0.7500	0.0000
5	Co	3.4758	0.0000	0.0000	0.5000	0.0000	0.0000
6	Co	5.2138	1.7379	0.0000	0.7500	0.2500	0.0000
7	Co	3.4758	3.4758	0.0000	0.5000	0.5000	0.0000
8	Co	5.2138	5.2138	0.0000	0.7500	0.7500	0.0000
9	Co	6.9516	6.9516	3.5271	1.0000	1.0000	0.1607
10	Co	1.7345	1.7347	3.4758	0.2495	0.2495	0.1583
11	Co	6.9506	3.4759	3.5283	0.9998	0.5000	0.1607
12	Co	1.7344	5.2171	3.4758	0.2495	0.7505	0.1583
13	Co	3.4755	0.0004	3.5283	0.5000	0.0001	0.1607
14	Co	5.2168	1.7348	3.4758	0.7504	0.2495	0.1583
15	Co	3.4757	3.4759	3.4742	0.5000	0.5000	0.1583
16	Co	5.2168	5.2170	3.4758	0.7504	0.7505	0.1583
17	Ni	1.7379	0.0000	1.7379	0.2500	0.0000	0.0792
18	Ni	0.0000	1.7379	1.7379	0.0000	0.2500	0.0792
19	Ni	1.7379	3.4758	1.7379	0.2500	0.5000	0.0792
20	Ni	0.0000	5.2138	1.7379	0.0000	0.7500	0.0792
21	Ni	5.2138	0.0000	1.7379	0.7500	0.0000	0.0792
22	Ni	3.4758	1.7379	1.7379	0.5000	0.2500	0.0792
23	Ni	5.2138	3.4758	1.7379	0.7500	0.5000	0.0792
24	Ni	3.4758	5.2138	1.7379	0.5000	0.7500	0.0792
25	Ni	1.7391	0.0002	5.1707	0.2502	0.0000	0.2355
26	Ni	6.9512	1.7394	5.1707	0.9999	0.2502	0.2356
27	Ni	1.7626	3.4761	5.2190	0.2535	0.5000	0.2377
28	Ni	6.9512	5.2124	5.1706	0.9999	0.7498	0.2355
29	Ni	5.2120	0.0002	5.1708	0.7498	0.0000	0.2356
30	Ni	3.4754	1.7641	5.2207	0.4999	0.2538	0.2378
31	Ni	5.1870	3.4760	5.2210	0.7461	0.5000	0.2378
32	Ni	3.4754	5.1886	5.2197	0.4999	0.7464	0.2378
33	O	3.4677	3.4793	7.5516	0.4988	0.5005	0.3440
34	C	3.4731	3.4770	6.3396	0.4996	0.5002	0.2888

/db/jmorales/CoNi-alloy/Adsorptions/CO_2-Sm(surf)$Ce_{26}O_{54}$

a = 11.6082000732
b = 11.608200073110298
c = 22.898399353
alpha = 90.0
beta = 90.0
gamma = 120.0

	Atom	X	Y	Z	X	Y	Z
1	Sm	2.0475	3.2620	7.1597	0.3386	0.3245	0.3127
2	Ce	1.9347	1.1170	0.7898	0.2222	0.1111	0.0345
3	Ce	0.0000	4.4680	0.7898	0.2222	0.4444	0.0345
4	Ce	-1.9347	7.8190	0.7898	0.2222	0.7778	0.0345
5	Ce	5.8041	1.1170	0.7898	0.5556	0.1111	0.0345
6	Ce	3.8694	4.4680	0.7898	0.5556	0.4444	0.0345
7	Ce	1.9347	7.8190	0.7898	0.5556	0.7778	0.0345
8	Ce	9.6735	1.1170	0.7898	0.8889	0.1111	0.0345
9	Ce	7.7388	4.4680	0.7898	0.8889	0.4444	0.0345
10	Ce	5.8041	7.8190	0.7898	0.8889	0.7778	0.0345
11	Ce	0.0143	2.2449	3.9804	0.1129	0.2233	0.1738
12	Ce	-1.9211	5.5868	3.9633	0.1124	0.5557	0.1731
13	Ce	-3.8631	8.9375	3.9475	0.1117	0.8890	0.1724
14	Ce	3.8596	2.2414	3.9559	0.4440	0.2230	0.1728
15	Ce	1.9187	5.5718	3.9987	0.4424	0.5542	0.1746
16	Ce	0.0011	8.9363	3.9401	0.4446	0.8889	0.1721
17	Ce	7.7307	2.2294	3.9248	0.7768	0.2218	0.1714
18	Ce	5.8045	5.5784	3.9476	0.7775	0.5549	0.1724
19	Ce	3.8660	8.9394	3.9551	0.7777	0.8892	0.1727
20	Ce	0.0151	-0.0032	7.1013	0.0011	-0.0003	0.3101
21	Ce	-2.0312	3.2735	7.0614	-0.0122	0.3256	0.3084
22	Ce	-3.8623	6.6976	7.0875	0.0004	0.6662	0.3095
23	Ce	3.8568	0.0034	7.1024	0.3324	0.0003	0.3102
24	Ce	-0.0022	6.8329	7.1395	0.3397	0.6797	0.3118
25	Ce	7.7389	0.0141	7.1035	0.6674	0.0014	0.3102
26	Ce	5.8047	3.3552	7.0961	0.6669	0.3338	0.3099
27	Ce	3.8806	6.7034	7.1144	0.6677	0.6668	0.3107
28	O	0.0000	0.0000	0.0000	0.0000	0.0000	0.0000
29	O	-1.9347	3.3510	0.0000	-0.0000	0.3333	0.0000
30	O	-3.8694	6.7020	0.0000	-0.0000	0.6667	0.0000
31	O	3.8694	0.0000	0.0000	0.3333	0.0000	0.0000
32	O	1.9347	3.3510	0.0000	0.3333	0.3333	0.0000
33	O	0.0000	6.7020	0.0000	0.3333	0.6667	0.0000
34	O	7.7388	0.0000	0.0000	0.6667	0.0000	0.0000
35	O	5.8041	3.3510	0.0000	0.6667	0.3333	0.0000
36	O	3.8694	6.7020	0.0000	0.6667	0.6667	0.0000
37	O	-0.0000	2.2340	1.5798	0.1111	0.2222	0.0690
38	O	-1.9347	5.5850	1.5798	0.1111	0.5556	0.0690
39	O	-3.8694	8.9360	1.5798	0.1111	0.8889	0.0690
40	O	3.8694	2.2340	1.5798	0.4444	0.2222	0.0690

41	O	1.9347	5.5850	1.5798	0.4444	0.5556	0.0690
42	O	-0.0000	8.9360	1.5798	0.4444	0.8889	0.0690
43	O	7.7388	2.2340	1.5798	0.7778	0.2222	0.0690
44	O	5.8041	5.5850	1.5798	0.7778	0.5556	0.0690
45	O	3.8694	8.9360	1.5798	0.7778	0.8889	0.0690
46	O	1.9347	1.1170	3.1593	0.2222	0.1111	0.1380
47	O	0.0000	4.4680	3.1593	0.2222	0.4444	0.1380
48	O	-1.9347	7.8190	3.1593	0.2222	0.7778	0.1380
49	O	5.8041	1.1170	3.1593	0.5556	0.1111	0.1380
50	O	3.8694	4.4680	3.1593	0.5556	0.4444	0.1380
51	O	1.9347	7.8190	3.1593	0.5556	0.7778	0.1380
52	O	9.6735	1.1170	3.1593	0.8889	0.1111	0.1380
53	O	7.7388	4.4680	3.1593	0.8889	0.4444	0.1380
54	O	5.8041	7.8190	3.1593	0.8889	0.7778	0.1380
55	O	0.0124	0.0278	4.7631	0.0024	0.0028	0.2080
56	O	-1.9555	3.3464	4.7474	-0.0020	0.3329	0.2073
57	O	-3.8304	6.6826	4.7560	0.0024	0.6647	0.2077
58	O	-1.9397	10.0495	4.7526	0.3327	0.9997	0.2076
59	O	1.9511	3.3546	4.7469	0.3349	0.3337	0.2073
60	O	0.0004	6.7289	4.7505	0.3347	0.6693	0.2075
61	O	7.7412	-0.0081	4.7497	0.6665	-0.0008	0.2074
62	O	5.7989	3.3596	4.7476	0.6666	0.3342	0.2073
63	O	3.8314	6.6763	4.7936	0.6621	0.6641	0.2093
64	O	-0.0311	2.2836	6.3466	0.1109	0.2272	0.2772
65	O	-1.8573	5.5597	6.3417	0.1165	0.5530	0.2770
66	O	-3.8795	8.8965	6.2643	0.1083	0.8850	0.2736
67	O	3.9021	2.2060	6.3081	0.4459	0.2194	0.2755
68	O	1.8103	5.5778	6.4295	0.4334	0.5548	0.2808
69	O	-0.0068	8.9546	6.2997	0.4448	0.8907	0.2751
70	O	7.7169	2.2200	6.2632	0.7752	0.2208	0.2735
71	O	5.8491	5.6199	6.2594	0.7834	0.5590	0.2734
72	O	3.9088	8.9480	6.2783	0.7818	0.8901	0.2742
73	O	1.9411	0.9724	7.8789	0.2156	0.0967	0.3441
74	O	-0.2587	4.3810	8.3012	0.1956	0.4358	0.3625
75	O	-1.9468	7.8444	7.8693	0.2224	0.7803	0.3437
76	O	5.8196	1.1211	7.8956	0.5571	0.1115	0.3448
77	O	4.0255	4.5319	7.8334	0.5722	0.4508	0.3421
78	O	1.9851	7.9215	7.8378	0.5650	0.7880	0.3423
79	O	9.6308	1.1231	7.8836	0.8855	0.1117	0.3443
80	O	7.7400	4.4438	7.8563	0.8878	0.4420	0.3431
81	O	5.8129	7.8094	7.8861	0.8892	0.7768	0.3444
82	C	0.6184	4.8261	9.2056	0.2933	0.4801	0.4020
83	O	1.5691	4.0450	9.5242	0.3364	0.4024	0.4159
84	O	0.4964	6.0484	9.5470	0.3436	0.6017	0.4169

/db/jmorales/CoNi-alloy/Adsorptions/Functionals/PBEsol/CoNi(001)

a =			6.9516692132				
b =			6.9516692132				
c =			21.9516692133				
alpha =			90.0				
beta =			90.0				
gamma =			90.0				
	Atom	X	Y	Z	X	Y	Z
1	Ni	0.0000	0.0000	0.0000	0.0000	0.0000	0.0000
2	Ni	0.0000	3.4758	0.0000	0.0000	0.5000	0.0000
3	Ni	3.4758	0.0000	0.0000	0.5000	0.0000	0.0000
4	Ni	3.4758	3.4758	0.0000	0.5000	0.5000	0.0000
5	Ni	0.0000	1.7379	1.7379	0.0000	0.2500	0.0792
6	Ni	0.0000	5.2138	1.7379	0.0000	0.7500	0.0792
7	Ni	3.4758	1.7379	1.7379	0.5000	0.2500	0.0792
8	Ni	3.4758	5.2138	1.7379	0.5000	0.7500	0.0792
9	Ni	0.0000	0.0000	3.4505	0.0000	0.0000	0.1572
10	Ni	0.0000	3.4758	3.4505	0.0000	0.5000	0.1572
11	Ni	3.4758	0.0000	3.4505	0.5000	0.0000	0.1572
12	Ni	3.4758	3.4758	3.4505	0.5000	0.5000	0.1572
13	Ni	0.0000	1.7379	5.0943	0.0000	0.2500	0.2321
14	Ni	0.0000	5.2138	5.0943	0.0000	0.7500	0.2321
15	Ni	3.4758	1.7379	5.0943	0.5000	0.2500	0.2321
16	Ni	3.4758	5.2138	5.0943	0.5000	0.7500	0.2321
17	Co	1.7379	1.7379	0.0000	0.2500	0.2500	0.0000
18	Co	1.7379	5.2138	0.0000	0.2500	0.7500	0.0000
19	Co	5.2138	1.7379	0.0000	0.7500	0.2500	0.0000
20	Co	5.2138	5.2138	0.0000	0.7500	0.7500	0.0000
21	Co	1.7379	0.0000	1.7379	0.2500	0.0000	0.0792
22	Co	1.7379	3.4758	1.7379	0.2500	0.5000	0.0792
23	Co	5.2138	0.0000	1.7379	0.7500	0.0000	0.0792
24	Co	5.2138	3.4758	1.7379	0.7500	0.5000	0.0792
25	Co	1.7379	1.7379	3.4959	0.2500	0.2500	0.1593
26	Co	1.7379	5.2138	3.4959	0.2500	0.7500	0.1593
27	Co	5.2138	1.7379	3.4959	0.7500	0.2500	0.1593
28	Co	5.2138	5.2138	3.4959	0.7500	0.7500	0.1593
29	Co	1.7379	0.0000	5.1053	0.2500	0.0000	0.2326
30	Co	1.7379	3.4758	5.1053	0.2500	0.5000	0.2326
31	Co	5.2138	0.0000	5.1053	0.7500	0.0000	0.2326
32	Co	5.2138	3.4758	5.1053	0.7500	0.5000	0.2326

/db/jmorales/CoNi-alloy/Adsorptions/CO_2-Sm(subsurf)$Ce_{26}O_{53}$

a = 11.6082000732
b = 11.608200073110298
c = 22.898399353
alpha = 90.0
beta = 90.0
gamma = 120.0

	Atom	X	Y	Z	X	Y	Z
1	Sm	1.9457	5.5935	3.8487	0.4458	0.5564	0.1681
2	Ce	1.9347	1.1170	0.7898	0.2222	0.1111	0.0345
3	Ce	0.0000	4.4680	0.7898	0.2222	0.4444	0.0345
4	Ce	-1.9347	7.8190	0.7898	0.2222	0.7778	0.0345
5	Ce	5.8041	1.1170	0.7898	0.5556	0.1111	0.0345
6	Ce	3.8694	4.4680	0.7898	0.5556	0.4444	0.0345
7	Ce	1.9347	7.8190	0.7898	0.5556	0.7778	0.0345
8	Ce	9.6735	1.1170	0.7898	0.8889	0.1111	0.0345
9	Ce	7.7388	4.4680	0.7898	0.8889	0.4444	0.0345
10	Ce	5.8041	7.8190	0.7898	0.8889	0.7778	0.0345
11	Ce	0.0029	2.2480	3.9523	0.1121	0.2236	0.1726
12	Ce	-1.9132	5.5857	3.9476	0.1130	0.5556	0.1724
13	Ce	-3.8707	8.9319	3.9420	0.1108	0.8885	0.1722
14	Ce	3.8690	2.2468	3.9401	0.4450	0.2235	0.1721
15	Ce	0.0146	8.9099	3.9664	0.4444	0.8863	0.1732
16	Ce	7.7372	2.2426	3.9063	0.7781	0.2231	0.1706
17	Ce	5.7627	5.5962	3.9707	0.7748	0.5567	0.1734
18	Ce	3.8556	8.8950	3.9902	0.7746	0.8848	0.1743
19	Ce	0.0078	-0.0019	7.0944	0.0006	-0.0002	0.3098
20	Ce	9.6483	3.3479	7.0816	0.9977	0.3330	0.3093
21	Ce	7.7203	6.7073	7.0942	0.9987	0.6672	0.3098
22	Ce	3.8685	-0.0021	7.0755	0.3332	-0.0002	0.3090
23	Ce	1.9650	3.1778	7.1348	0.3273	0.3161	0.3116
24	Ce	-0.2522	6.7655	7.2140	0.3148	0.6730	0.3150
25	Ce	7.7477	0.1360	7.0744	0.6742	0.0135	0.3089
26	Ce	5.8123	3.3468	7.0829	0.6672	0.3329	0.3093
27	Ce	4.0827	6.7517	7.3342	0.6875	0.6716	0.3203
28	O	0.0000	0.0000	0.0000	0.0000	0.0000	0.0000
29	O	-1.9347	3.3510	0.0000	-0.0000	0.3333	0.0000
30	O	-3.8694	6.7020	0.0000	-0.0000	0.6667	0.0000
31	O	3.8694	0.0000	0.0000	0.3333	0.0000	0.0000
32	O	1.9347	3.3510	0.0000	0.3333	0.3333	0.0000
33	O	0.0000	6.7020	0.0000	0.3333	0.6667	0.0000
34	O	7.7388	0.0000	0.0000	0.6667	0.0000	0.0000
35	O	5.8041	3.3510	0.0000	0.6667	0.3333	0.0000
36	O	3.8694	6.7020	0.0000	0.6667	0.6667	0.0000
37	O	-0.0000	2.2340	1.5798	0.1111	0.2222	0.0690
38	O	-1.9347	5.5850	1.5798	0.1111	0.5556	0.0690
39	O	-3.8694	8.9360	1.5798	0.1111	0.8889	0.0690
40	O	3.8694	2.2340	1.5798	0.4444	0.2222	0.0690

41	O	1.9347	5.5850	1.5798	0.4444	0.5556	0.0690
42	O	-0.0000	8.9360	1.5798	0.4444	0.8889	0.0690
43	O	7.7388	2.2340	1.5798	0.7778	0.2222	0.0690
44	O	5.8041	5.5850	1.5798	0.7778	0.5556	0.0690
45	O	3.8694	8.9360	1.5798	0.7778	0.8889	0.0690
46	O	1.9347	1.1170	3.1593	0.2222	0.1111	0.1380
47	O	0.0000	4.4680	3.1593	0.2222	0.4444	0.1380
48	O	-1.9347	7.8190	3.1593	0.2222	0.7778	0.1380
49	O	5.8041	1.1170	3.1593	0.5556	0.1111	0.1380
50	O	3.8694	4.4680	3.1593	0.5556	0.4444	0.1380
51	O	1.9347	7.8190	3.1593	0.5556	0.7778	0.1380
52	O	9.6735	1.1170	3.1593	0.8889	0.1111	0.1380
53	O	7.7388	4.4680	3.1593	0.8889	0.4444	0.1380
54	O	5.8041	7.8190	3.1593	0.8889	0.7778	0.1380
55	O	-0.0136	-0.0129	4.7550	-0.0018	-0.0013	0.2077
56	O	-1.9174	3.3639	4.7670	0.0021	0.3346	0.2082
57	O	-3.8675	6.7086	4.7443	0.0005	0.6673	0.2072
58	O	-1.9111	10.0296	4.7362	0.3342	0.9977	0.2068
59	O	1.9423	3.4077	4.8819	0.3368	0.3390	0.2132
60	O	0.0432	6.6739	4.9075	0.3357	0.6639	0.2143
61	O	7.7258	0.0049	4.7732	0.6658	0.0005	0.2084
62	O	5.7859	3.3536	4.7606	0.6652	0.3336	0.2079
63	O	3.8346	6.6900	4.8714	0.6631	0.6655	0.2127
64	O	-0.0200	2.1674	6.3248	0.1061	0.2156	0.2762
65	O	-2.0111	5.5880	6.3388	0.1047	0.5559	0.2768
66	O	-3.8542	8.9698	6.2565	0.1141	0.8922	0.2732
67	O	3.8939	2.1335	6.2827	0.4416	0.2122	0.2744
68	O	-0.0133	8.9604	6.3644	0.4445	0.8913	0.2779
69	O	7.7464	2.2635	6.2461	0.7799	0.2252	0.2728
70	O	5.9030	5.5559	6.3395	0.7848	0.5527	0.2769
71	O	3.8552	9.0303	6.3887	0.7812	0.8983	0.2790
72	O	1.9490	1.0741	7.9357	0.2213	0.1068	0.3466
73	O	0.2227	4.5803	7.5640	0.2470	0.4556	0.3303
74	O	-2.0382	7.8534	7.9258	0.2150	0.7812	0.3461
75	O	5.8640	1.1697	7.8914	0.5633	0.1164	0.3446
76	O	3.7531	4.3815	7.7055	0.5412	0.4358	0.3365
77	O	1.8542	7.7658	7.9112	0.5460	0.7725	0.3455
78	O	9.6056	1.2184	7.9251	0.8881	0.1212	0.3461
79	O	7.7622	4.3976	7.9205	0.8874	0.4374	0.3459
80	O	5.9851	7.9127	7.9609	0.9091	0.7871	0.3477
81	C	1.8153	7.1383	9.1757	0.5114	0.7101	0.4007
82	O	0.6408	6.8333	9.5465	0.3951	0.6797	0.4169
83	O	2.9359	6.8915	9.6845	0.5957	0.6855	0.4229

/db/jmorales/CoNi-alloy/Adsorptions/Coverage/CoNi(001)-2CO

a =			6.9516692132				
b =			6.9516692132				
c =			21.9516692133				
alpha =			90.0				
beta =			90.0				
gamma =			90.0				
	Atom	X	Y	Z	X	Y	Z
1	Ni	0.0000	0.0000	0.0000	0.0000	0.0000	0.0000
2	Ni	0.0000	3.4758	0.0000	0.0000	0.5000	0.0000
3	Ni	3.4758	0.0000	0.0000	0.5000	0.0000	0.0000
4	Ni	3.4758	3.4758	0.0000	0.5000	0.5000	0.0000
5	Ni	0.0000	1.7379	1.7379	0.0000	0.2500	0.0792
6	Ni	0.0000	5.2138	1.7379	0.0000	0.7500	0.0792
7	Ni	3.4758	1.7379	1.7379	0.5000	0.2500	0.0792
8	Ni	3.4758	5.2138	1.7379	0.5000	0.7500	0.0792
9	Ni	6.9504	-0.0006	3.4980	0.9998	-0.0001	0.1594
10	Ni	6.9504	3.4746	3.4981	0.9998	0.4998	0.1594
11	Ni	3.4747	-0.0009	3.4658	0.4998	-0.0001	0.1579
12	Ni	3.4747	3.4752	3.4650	0.4998	0.4999	0.1578
13	Ni	6.9498	1.7369	5.2078	0.9997	0.2499	0.2372
14	Ni	6.9498	5.2129	5.2065	0.9997	0.7499	0.2372
15	Ni	3.4740	1.7369	5.2702	0.4997	0.2499	0.2401
16	Ni	3.4736	5.2129	5.2697	0.4997	0.7499	0.2401
17	Co	1.7379	1.7379	0.0000	0.2500	0.2500	0.0000
18	Co	1.7379	5.2138	0.0000	0.2500	0.7500	0.0000
19	Co	5.2138	1.7379	0.0000	0.7500	0.2500	0.0000
20	Co	5.2138	5.2138	0.0000	0.7500	0.7500	0.0000
21	Co	1.7379	0.0000	1.7379	0.2500	0.0000	0.0792
22	Co	1.7379	3.4758	1.7379	0.2500	0.5000	0.0792
23	Co	5.2138	0.0000	1.7379	0.7500	0.0000	0.0792
24	Co	5.2138	3.4758	1.7379	0.7500	0.5000	0.0792
25	Co	1.7316	1.7370	3.5519	0.2491	0.2499	0.1618
26	Co	1.7314	5.2126	3.5512	0.2491	0.7498	0.1618
27	Co	5.2182	1.7373	3.5520	0.7506	0.2499	0.1618
28	Co	5.2176	5.2125	3.5518	0.7506	0.7498	0.1618
29	Co	1.7476	-0.0009	5.2386	0.2514	-0.0001	0.2386
30	Co	1.7464	3.4746	5.2390	0.2512	0.4998	0.2387
31	Co	5.1988	-0.0013	5.2401	0.7478	-0.0002	0.2387
32	Co	5.1997	3.4750	5.2410	0.7480	0.4999	0.2388
33	O	3.4646	3.4762	7.5827	0.4984	0.5000	0.3454
34	O	3.4685	6.9486	7.5846	0.4989	0.9996	0.3455
35	C	3.4749	3.4748	6.3733	0.4999	0.4999	0.2903
36	C	3.4745	0.0003	6.3752	0.4998	0.0000	0.2904

/db/jmorales/CoNi-alloy/Adsorptions/CO_2-CoNi-Ce_3SmO_7

a =				13.9033384264			
b =				13.9033384264			
c =				21.9516692133			
alpha =				90.0			
beta =				90.0			
gamma =				90.0			
	Atom	X	Y	Z	X	Y	Z
1	Ni	0.0000	0.0000	0.0000	0.0000	0.0000	0.0000
2	Ni	0.0000	3.4758	0.0000	0.0000	0.2500	0.0000
3	Ni	3.4758	0.0000	0.0000	0.2500	0.0000	0.0000
4	Ni	3.4758	3.4758	0.0000	0.2500	0.2500	0.0000
5	Ni	0.0000	6.9517	0.0000	0.0000	0.5000	0.0000
6	Ni	0.0000	10.4275	0.0000	0.0000	0.7500	0.0000
7	Ni	3.4758	6.9517	0.0000	0.2500	0.5000	0.0000
8	Ni	3.4758	10.4275	0.0000	0.2500	0.7500	0.0000
9	Ni	6.9517	0.0000	0.0000	0.5000	0.0000	0.0000
10	Ni	6.9517	3.4758	0.0000	0.5000	0.2500	0.0000
11	Ni	10.4275	0.0000	0.0000	0.7500	0.0000	0.0000
12	Ni	10.4275	3.4758	0.0000	0.7500	0.2500	0.0000
13	Ni	6.9517	6.9517	0.0000	0.5000	0.5000	0.0000
14	Ni	6.9517	10.4275	0.0000	0.5000	0.7500	0.0000
15	Ni	10.4275	6.9517	0.0000	0.7500	0.5000	0.0000
16	Ni	10.4275	10.4275	0.0000	0.7500	0.7500	0.0000
17	Ni	0.0000	1.7379	1.7379	0.0000	0.1250	0.0792
18	Ni	0.0000	5.2138	1.7379	0.0000	0.3750	0.0792
19	Ni	3.4758	1.7379	1.7379	0.2500	0.1250	0.0792
20	Ni	3.4758	5.2138	1.7379	0.2500	0.3750	0.0792
21	Ni	0.0000	8.6896	1.7379	0.0000	0.6250	0.0792
22	Ni	0.0000	12.1654	1.7379	0.0000	0.8750	0.0792
23	Ni	3.4758	8.6896	1.7379	0.2500	0.6250	0.0792
24	Ni	3.4758	12.1654	1.7379	0.2500	0.8750	0.0792
25	Ni	6.9517	1.7379	1.7379	0.5000	0.1250	0.0792
26	Ni	6.9517	5.2138	1.7379	0.5000	0.3750	0.0792
27	Ni	10.4275	1.7379	1.7379	0.7500	0.1250	0.0792
28	Ni	10.4275	5.2138	1.7379	0.7500	0.3750	0.0792
29	Ni	6.9517	8.6896	1.7379	0.5000	0.6250	0.0792
30	Ni	6.9517	12.1654	1.7379	0.5000	0.8750	0.0792
31	Ni	10.4275	8.6896	1.7379	0.7500	0.6250	0.0792
32	Ni	10.4275	12.1654	1.7379	0.7500	0.8750	0.0792
33	Ni	13.8976	13.9005	3.5027	0.9996	0.9998	0.1596
34	Ni	0.0009	3.4780	3.5007	0.0001	0.2502	0.1595
35	Ni	3.4966	13.8822	3.5404	0.2515	0.9985	0.1613
36	Ni	3.4671	3.4584	3.4666	0.2494	0.2487	0.1579
37	Ni	0.0014	6.9571	3.5114	0.0001	0.5004	0.1600
38	Ni	13.8768	10.4332	3.4790	0.9981	0.7504	0.1585
39	Ni	3.4819	6.9556	3.4999	0.2504	0.5003	0.1594
40	Ni	3.5037	10.4253	3.5574	0.2520	0.7498	0.1621

41	Ni	6.9366	0.0119	3.5069	0.4989	0.0009	0.1598
42	Ni	6.9507	3.4976	3.5247	0.4999	0.2516	0.1606
43	Ni	10.4228	0.0032	3.5059	0.7497	0.0002	0.1597
44	Ni	10.4283	3.4775	3.5018	0.7501	0.2501	0.1595
45	Ni	6.9702	6.9277	3.4624	0.5013	0.4983	0.1577
46	Ni	6.9566	10.4446	3.4626	0.5004	0.7512	0.1577
47	Ni	10.4377	6.9377	3.4904	0.7507	0.4990	0.1590
48	Ni	10.4321	10.4419	3.5021	0.7503	0.7510	0.1595
49	Ni	13.8998	1.7542	5.2038	0.9997	0.1262	0.2371
50	Ni	0.0004	5.2110	5.2171	0.0000	0.3748	0.2377
51	Ni	3.4499	1.7543	5.1980	0.2481	0.1262	0.2368
52	Ni	3.4627	5.1840	5.1027	0.2491	0.3729	0.2324
53	Ni	13.8630	8.7118	5.1863	0.9971	0.6266	0.2363
54	Ni	0.0028	12.1615	5.1999	0.0002	0.8747	0.2369
55	Ni	3.4635	8.6684	5.2480	0.2491	0.6235	0.2391
56	Ni	3.3878	12.1421	5.3256	0.2437	0.8733	0.2426
57	Ni	6.9707	1.7640	5.2011	0.5014	0.1269	0.2369
58	Ni	6.9467	5.1600	5.3956	0.4996	0.3711	0.2458
59	Ni	10.4267	1.7353	5.2032	0.7499	0.1248	0.2370
60	Ni	10.4295	5.2159	5.2130	0.7501	0.3752	0.2375
61	Ni	7.0372	8.6814	5.0648	0.5061	0.6244	0.2307
62	Ni	6.9861	12.1726	5.1223	0.5025	0.8755	0.2333
63	Ni	10.4210	8.6955	5.2047	0.7495	0.6254	0.2371
64	Ni	10.4295	12.1806	5.2663	0.7501	0.8761	0.2399
65	Co	1.7379	1.7379	0.0000	0.1250	0.1250	0.0000
66	Co	1.7379	5.2138	0.0000	0.1250	0.3750	0.0000
67	Co	5.2138	1.7379	0.0000	0.3750	0.1250	0.0000
68	Co	5.2138	5.2138	0.0000	0.3750	0.3750	0.0000
69	Co	1.7379	8.6896	0.0000	0.1250	0.6250	0.0000
70	Co	1.7379	12.1654	0.0000	0.1250	0.8750	0.0000
71	Co	5.2138	8.6896	0.0000	0.3750	0.6250	0.0000
72	Co	5.2138	12.1654	0.0000	0.3750	0.8750	0.0000
73	Co	8.6896	1.7379	0.0000	0.6250	0.1250	0.0000
74	Co	8.6896	5.2138	0.0000	0.6250	0.3750	0.0000
75	Co	12.1654	1.7379	0.0000	0.8750	0.1250	0.0000
76	Co	12.1654	5.2138	0.0000	0.8750	0.3750	0.0000
77	Co	8.6896	8.6896	0.0000	0.6250	0.6250	0.0000
78	Co	8.6896	12.1654	0.0000	0.6250	0.8750	0.0000
79	Co	12.1654	8.6896	0.0000	0.8750	0.6250	0.0000
80	Co	12.1654	12.1654	0.0000	0.8750	0.8750	0.0000
81	Co	1.7379	0.0000	1.7379	0.1250	0.0000	0.0792
82	Co	1.7379	3.4758	1.7379	0.1250	0.2500	0.0792
83	Co	5.2138	0.0000	1.7379	0.3750	0.0000	0.0792
84	Co	5.2138	3.4758	1.7379	0.3750	0.2500	0.0792
85	Co	1.7379	6.9517	1.7379	0.1250	0.5000	0.0792
86	Co	1.7379	10.4275	1.7379	0.1250	0.7500	0.0792
87	Co	5.2138	6.9517	1.7379	0.3750	0.5000	0.0792
88	Co	5.2138	10.4275	1.7379	0.3750	0.7500	0.0792
89	Co	8.6896	0.0000	1.7379	0.6250	0.0000	0.0792

90	Co	8.6896	3.4758	1.7379	0.6250	0.2500	0.0792
91	Co	12.1654	0.0000	1.7379	0.8750	0.0000	0.0792
92	Co	12.1654	3.4758	1.7379	0.8750	0.2500	0.0792
93	Co	8.6896	6.9517	1.7379	0.6250	0.5000	0.0792
94	Co	8.6896	10.4275	1.7379	0.6250	0.7500	0.0792
95	Co	12.1654	6.9517	1.7379	0.8750	0.5000	0.0792
96	Co	12.1654	10.4275	1.7379	0.8750	0.7500	0.0792
97	Co	1.7340	1.7366	3.5382	0.1247	0.1249	0.1612
98	Co	1.7097	5.2396	3.5361	0.1230	0.3769	0.1611
99	Co	5.2132	1.7245	3.5634	0.3750	0.1240	0.1623
100	Co	5.2415	5.2005	3.5347	0.3770	0.3740	0.1610
101	Co	1.7342	8.6780	3.5227	0.1247	0.6242	0.1605
102	Co	1.7486	12.1723	3.5395	0.1258	0.8755	0.1612
103	Co	5.1979	8.6836	3.5525	0.3739	0.6246	0.1618
104	Co	5.1887	12.1634	3.6229	0.3732	0.8749	0.1650
105	Co	8.6880	1.7346	3.5422	0.6249	0.1248	0.1614
106	Co	8.6680	5.2029	3.5810	0.6234	0.3742	0.1631
107	Co	12.1635	1.7401	3.5491	0.8749	0.1252	0.1617
108	Co	12.1709	5.2112	3.5596	0.8754	0.3748	0.1622
109	Co	8.7307	8.7018	3.4951	0.6280	0.6259	0.1592
110	Co	8.7146	12.1742	3.4567	0.6268	0.8756	0.1575
111	Co	12.1492	8.6886	3.5419	0.8738	0.6249	0.1613
112	Co	12.1450	12.1765	3.5776	0.8735	0.8758	0.1630
113	Co	1.7009	0.0356	5.2114	0.1223	0.0026	0.2374
114	Co	1.7477	3.4903	5.2161	0.1257	0.2510	0.2376
115	Co	5.2258	0.0202	5.3380	0.3759	0.0015	0.2432
116	Co	5.1681	3.4420	5.2058	0.3717	0.2476	0.2371
117	Co	1.6936	6.9682	5.3196	0.1218	0.5012	0.2423
118	Co	1.6843	10.4114	5.1446	0.1211	0.7488	0.2344
119	Co	5.2901	6.9324	5.1889	0.3805	0.4986	0.2364
120	Co	5.3060	10.3618	5.2730	0.3816	0.7453	0.2402
121	Co	8.6870	0.0081	5.2757	0.6248	0.0006	0.2403
122	Co	8.7134	3.4618	5.2281	0.6267	0.2490	0.2382
123	Co	12.1751	0.0153	5.2228	0.8757	0.0011	0.2379
124	Co	12.1593	3.4740	5.2249	0.8746	0.2499	0.2380
125	Co	8.7215	6.9750	5.1724	0.6273	0.5017	0.2356
126	Co	8.6970	10.4404	5.2526	0.6255	0.7509	0.2393
127	Co	12.1590	6.9538	5.2215	0.8745	0.5002	0.2379
128	Co	12.1625	10.4286	5.2159	0.8748	0.7501	0.2376
129	O	5.9954	6.1718	6.8507	0.4312	0.4439	0.3121
130	O	2.5472	7.8723	6.8677	0.1832	0.5662	0.3129
131	O	4.6693	8.0057	8.7512	0.3358	0.5758	0.3987
132	O	5.6238	10.2374	7.1831	0.4045	0.7363	0.3272
133	O	7.8208	9.9935	8.9133	0.5625	0.7188	0.4060
134	O	4.7919	12.1346	9.0457	0.3447	0.8728	0.4121
135	O	8.6257	12.2080	6.3597	0.6204	0.8781	0.2897
136	Ce	3.4990	9.8297	7.9189	0.2517	0.7070	0.3607
137	Ce	3.8546	6.1705	7.6768	0.2772	0.4438	0.3497
138	Ce	6.8678	8.2906	7.9013	0.4940	0.5963	0.3599

139 Sm	7.0868	11.7498	8.0424	0.5097	0.8451	0.3664
140 C	4.4401	12.7065	7.9331	0.3194	0.9139	0.3614
141 O	3.3290	12.3201	7.3562	0.2394	0.8861	0.3351
142 O	5.2639	13.5348	7.3600	0.3786	0.9735	0.3353

/db/jmorales/CoNi-alloy/Adsorptions/Expanded-models/CO-Ni(111)-expanded

a =	9.967200279
b =	9.967200279058309
c =	21.103700638
alpha =	90.0
beta =	90.0
gamma =	120.0

	Atom	X	Y	Z	X	Y	Z
1	Ni	0.0000	0.0000	0.0000	0.0000	0.0000	0.0000
2	Ni	-1.2459	2.1580	0.0000	-0.0000	0.2500	0.0000
3	Ni	-2.4918	4.3159	0.0000	-0.0000	0.5000	0.0000
4	Ni	-3.7377	6.4739	0.0000	0.0000	0.7500	0.0000
5	Ni	2.4918	0.0000	0.0000	0.2500	0.0000	0.0000
6	Ni	1.2459	2.1580	0.0000	0.2500	0.2500	0.0000
7	Ni	0.0000	4.3159	0.0000	0.2500	0.5000	0.0000
8	Ni	-1.2459	6.4739	0.0000	0.2500	0.7500	0.0000
9	Ni	4.9836	0.0000	0.0000	0.5000	0.0000	0.0000
10	Ni	3.7377	2.1580	0.0000	0.5000	0.2500	0.0000
11	Ni	2.4918	4.3159	0.0000	0.5000	0.5000	0.0000
12	Ni	1.2459	6.4739	0.0000	0.5000	0.7500	0.0000
13	Ni	7.4754	0.0000	0.0000	0.7500	0.0000	0.0000
14	Ni	6.2295	2.1580	0.0000	0.7500	0.2500	0.0000
15	Ni	4.9836	4.3159	0.0000	0.7500	0.5000	0.0000
16	Ni	3.7377	6.4739	0.0000	0.7500	0.7500	0.0000
17	Ni	-0.0000	1.4386	2.0346	0.0833	0.1667	0.0964
18	Ni	-1.2459	3.5966	2.0346	0.0833	0.4167	0.0964
19	Ni	-2.4918	5.7546	2.0346	0.0833	0.6667	0.0964
20	Ni	-3.7377	7.9125	2.0346	0.0833	0.9167	0.0964
21	Ni	2.4918	1.4386	2.0346	0.3333	0.1667	0.0964
22	Ni	1.2459	3.5966	2.0346	0.3333	0.4167	0.0964
23	Ni	-0.0000	5.7546	2.0346	0.3333	0.6667	0.0964
24	Ni	-1.2459	7.9125	2.0346	0.3333	0.9167	0.0964
25	Ni	4.9836	1.4386	2.0346	0.5833	0.1667	0.0964
26	Ni	3.7377	3.5966	2.0346	0.5833	0.4167	0.0964
27	Ni	2.4918	5.7546	2.0346	0.5833	0.6667	0.0964
28	Ni	1.2459	7.9125	2.0346	0.5833	0.9167	0.0964
29	Ni	7.4754	1.4386	2.0346	0.8333	0.1667	0.0964
30	Ni	6.2295	3.5966	2.0346	0.8333	0.4167	0.0964
31	Ni	4.9836	5.7546	2.0346	0.8333	0.6667	0.0964
32	Ni	3.7377	7.9125	2.0346	0.8333	0.9167	0.0964
33	Ni	1.2459	0.7193	4.0692	0.1667	0.0833	0.1928
34	Ni	0.0000	2.8773	4.0692	0.1667	0.3333	0.1928
35	Ni	-1.2459	5.0352	4.0692	0.1667	0.5833	0.1928
36	Ni	-2.4918	7.1932	4.0692	0.1667	0.8333	0.1928
37	Ni	3.7377	0.7193	4.0692	0.4167	0.0833	0.1928
38	Ni	2.4918	2.8773	4.0692	0.4167	0.3333	0.1928
39	Ni	1.2459	5.0352	4.0692	0.4167	0.5833	0.1928
40	Ni	0.0000	7.1932	4.0692	0.4167	0.8333	0.1928
41	Ni	6.2295	0.7193	4.0692	0.6667	0.0833	0.1928

42	Ni	4.9836	2.8773	4.0692	0.6667	0.3333	0.1928
43	Ni	3.7377	5.0352	4.0692	0.6667	0.5833	0.1928
44	Ni	2.4918	7.1932	4.0692	0.6667	0.8333	0.1928
45	Ni	8.7213	0.7193	4.0692	0.9167	0.0833	0.1928
46	Ni	7.4754	2.8773	4.0692	0.9167	0.3333	0.1928
47	Ni	6.2295	5.0352	4.0692	0.9167	0.5833	0.1928
48	Ni	4.9836	7.1932	4.0692	0.9167	0.8333	0.1928
49	Ni	0.0000	0.0000	6.1036	0.0000	0.0000	0.2892
50	Ni	-1.2459	2.1580	6.1036	-0.0000	0.2500	0.2892
51	Ni	-2.4918	4.3159	6.1036	-0.0000	0.5000	0.2892
52	Ni	-3.7377	6.4739	6.1036	0.0000	0.7500	0.2892
53	Ni	2.4918	0.0000	6.1036	0.2500	0.0000	0.2892
54	Ni	1.2459	2.1580	6.1036	0.2500	0.2500	0.2892
55	Ni	0.0000	4.3159	6.1036	0.2500	0.5000	0.2892
56	Ni	-1.2459	6.4739	6.1036	0.2500	0.7500	0.2892
57	Ni	4.9836	0.0000	6.1036	0.5000	0.0000	0.2892
58	Ni	3.7377	2.1580	6.1036	0.5000	0.2500	0.2892
59	Ni	2.4918	4.3159	6.1036	0.5000	0.5000	0.2892
60	Ni	1.2459	6.4739	6.1036	0.5000	0.7500	0.2892
61	Ni	7.4754	0.0000	6.1036	0.7500	0.0000	0.2892
62	Ni	6.2295	2.1580	6.1036	0.7500	0.2500	0.2892
63	Ni	4.9836	4.3159	6.1036	0.7500	0.5000	0.2892
64	Ni	3.7377	6.4739	6.1036	0.7500	0.7500	0.2892
65	O	1.2302	3.5766	8.6014	0.3306	0.4143	0.4076
66	C	1.2357	3.5787	7.4074	0.3313	0.4146	0.3510

/db/jmorales/CoNi-alloy/Adsorptions/Functionals/PBEsol/Ni-CoNi(001)

a = 6.9516692132
b = 6.9516692132
c = 21.9516692133
alpha = 90.0
beta = 90.0
gamma = 90.0

	Atom	X	Y	Z	X	Y	Z
1	Co	0.0000	0.0000	0.0000	0.0000	0.0000	0.0000
2	Co	1.7379	1.7379	0.0000	0.2500	0.2500	0.0000
3	Co	0.0000	3.4758	0.0000	0.0000	0.5000	0.0000
4	Co	1.7379	5.2138	0.0000	0.2500	0.7500	0.0000
5	Co	3.4758	0.0000	0.0000	0.5000	0.0000	0.0000
6	Co	5.2138	1.7379	0.0000	0.7500	0.2500	0.0000
7	Co	3.4758	3.4758	0.0000	0.5000	0.5000	0.0000
8	Co	5.2138	5.2138	0.0000	0.7500	0.7500	0.0000
9	Co	0.0000	0.0000	3.4823	0.0000	0.0000	0.1586
10	Co	1.7379	1.7379	3.4801	0.2500	0.2500	0.1585
11	Co	0.0000	3.4758	3.4823	0.0000	0.5000	0.1586
12	Co	1.7379	5.2138	3.4801	0.2500	0.7500	0.1585
13	Co	3.4758	0.0000	3.4823	0.5000	0.0000	0.1586
14	Co	5.2138	1.7379	3.4801	0.7500	0.2500	0.1585
15	Co	3.4758	3.4758	3.4823	0.5000	0.5000	0.1586
16	Co	5.2138	5.2138	3.4801	0.7500	0.7500	0.1585
17	Ni	1.7379	0.0000	1.7379	0.2500	0.0000	0.0792
18	Ni	0.0000	1.7379	1.7379	0.0000	0.2500	0.0792
19	Ni	1.7379	3.4758	1.7379	0.2500	0.5000	0.0792
20	Ni	0.0000	5.2138	1.7379	0.0000	0.7500	0.0792
21	Ni	5.2138	0.0000	1.7379	0.7500	0.0000	0.0792
22	Ni	3.4758	1.7379	1.7379	0.5000	0.2500	0.0792
23	Ni	5.2138	3.4758	1.7379	0.7500	0.5000	0.0792
24	Ni	3.4758	5.2138	1.7379	0.5000	0.7500	0.0792
25	Ni	1.7379	0.0000	5.0965	0.2500	0.0000	0.2322
26	Ni	0.0000	1.7379	5.0965	0.0000	0.2500	0.2322
27	Ni	1.7379	3.4758	5.0965	0.2500	0.5000	0.2322
28	Ni	0.0000	5.2138	5.0965	0.0000	0.7500	0.2322
29	Ni	5.2138	0.0000	5.0965	0.7500	0.0000	0.2322
30	Ni	3.4758	1.7379	5.0965	0.5000	0.2500	0.2322
31	Ni	5.2138	3.4758	5.0965	0.7500	0.5000	0.2322
32	Ni	3.4758	5.2138	5.0965	0.5000	0.7500	0.2322

/db/jmorales/CoNi-alloy/Adsorptions/Functionals/PBEsol/Co-CoNi(001)

a =			6.9516692132				
b =			6.9516692132				
c =			21.9516692133				
alpha =			90.0				
beta =			90.0				
gamma =			90.0				
	Atom	X	Y	Z	X	Y	Z
1	Ni	0.0000	0.0000	0.0000	0.0000	0.0000	0.0000
2	Ni	1.7379	1.7379	0.0000	0.2500	0.2500	0.0000
3	Ni	0.0000	3.4758	0.0000	0.0000	0.5000	0.0000
4	Ni	1.7379	5.2138	0.0000	0.2500	0.7500	0.0000
5	Ni	3.4758	0.0000	0.0000	0.5000	0.0000	0.0000
6	Ni	5.2138	1.7379	0.0000	0.7500	0.2500	0.0000
7	Ni	3.4758	3.4758	0.0000	0.5000	0.5000	0.0000
8	Ni	5.2138	5.2138	0.0000	0.7500	0.7500	0.0000
9	Ni	0.0000	0.0000	3.4833	0.0000	0.0000	0.1587
10	Ni	1.7379	1.7379	3.4822	0.2500	0.2500	0.1586
11	Ni	0.0000	3.4758	3.4833	0.0000	0.5000	0.1587
12	Ni	1.7379	5.2138	3.4822	0.2500	0.7500	0.1586
13	Ni	3.4758	0.0000	3.4833	0.5000	0.0000	0.1587
14	Ni	5.2138	1.7379	3.4822	0.7500	0.2500	0.1586
15	Ni	3.4758	3.4758	3.4833	0.5000	0.5000	0.1587
16	Ni	5.2138	5.2138	3.4822	0.7500	0.7500	0.1586
17	Co	1.7379	0.0000	1.7379	0.2500	0.0000	0.0792
18	Co	0.0000	1.7379	1.7379	0.0000	0.2500	0.0792
19	Co	1.7379	3.4758	1.7379	0.2500	0.5000	0.0792
20	Co	0.0000	5.2138	1.7379	0.0000	0.7500	0.0792
21	Co	5.2138	0.0000	1.7379	0.7500	0.0000	0.0792
22	Co	3.4758	1.7379	1.7379	0.5000	0.2500	0.0792
23	Co	5.2138	3.4758	1.7379	0.7500	0.5000	0.0792
24	Co	3.4758	5.2138	1.7379	0.5000	0.7500	0.0792
25	Co	1.7379	0.0000	5.1695	0.2500	0.0000	0.2355
26	Co	0.0000	1.7379	5.1695	0.0000	0.2500	0.2355
27	Co	1.7379	3.4758	5.1695	0.2500	0.5000	0.2355
28	Co	0.0000	5.2138	5.1695	0.0000	0.7500	0.2355
29	Co	5.2138	0.0000	5.1695	0.7500	0.0000	0.2355
30	Co	3.4758	1.7379	5.1695	0.5000	0.2500	0.2355
31	Co	5.2138	3.4758	5.1695	0.7500	0.5000	0.2355
32	Co	3.4758	5.2138	5.1695	0.5000	0.7500	0.2355

/db/jmorales/CoNi-alloy/Adsorptions/CO_2-Co-CoNi(001)

a =			6.951669213				
b =			6.951669213				
c =			21.951669213				
alpha =			90.0				
beta =			90.0				
gamma =			90.0				
	Atom	X	Y	Z	X	Y	Z
1	Ni	0.0000	0.0000	0.0000	0.0000	0.0000	0.0000
2	Ni	1.7379	1.7379	0.0000	0.2500	0.2500	0.0000
3	Ni	0.0000	3.4758	0.0000	0.0000	0.5000	0.0000
4	Ni	1.7379	5.2138	0.0000	0.2500	0.7500	0.0000
5	Ni	3.4758	0.0000	0.0000	0.5000	0.0000	0.0000
6	Ni	5.2138	1.7379	0.0000	0.7500	0.2500	0.0000
7	Ni	3.4758	3.4758	0.0000	0.5000	0.5000	0.0000
8	Ni	5.2138	5.2138	0.0000	0.7500	0.7500	0.0000
9	Ni	6.9507	0.0006	3.5041	0.9999	0.0001	0.1596
10	Ni	1.7464	1.7493	3.5339	0.2512	0.2516	0.1610
11	Ni	6.9394	3.4740	3.5233	0.9982	0.4997	0.1605
12	Ni	1.7375	5.2136	3.5229	0.2499	0.7500	0.1605
13	Ni	3.4778	0.0096	3.5246	0.5003	0.0014	0.1606
14	Ni	5.2106	1.7409	3.5442	0.7495	0.2504	0.1615
15	Ni	3.4782	3.4767	3.5415	0.5003	0.5001	0.1613
16	Ni	5.2037	5.2062	3.5377	0.7485	0.7489	0.1612
17	Co	1.7379	0.0000	1.7379	0.2500	0.0000	0.0792
18	Co	0.0000	1.7379	1.7379	0.0000	0.2500	0.0792
19	Co	1.7379	3.4758	1.7379	0.2500	0.5000	0.0792
20	Co	0.0000	5.2138	1.7379	0.0000	0.7500	0.0792
21	Co	5.2138	0.0000	1.7379	0.7500	0.0000	0.0792
22	Co	3.4758	1.7379	1.7379	0.5000	0.2500	0.0792
23	Co	5.2138	3.4758	1.7379	0.7500	0.5000	0.0792
24	Co	3.4758	5.2138	1.7379	0.5000	0.7500	0.0792
25	Co	1.7079	0.0076	5.2543	0.2457	0.0011	0.2394
26	Co	0.0261	1.7175	5.2601	0.0038	0.2471	0.2396
27	Co	1.7354	3.5110	5.2868	0.2496	0.5051	0.2408
28	Co	6.9428	5.2158	5.2585	0.9987	0.7503	0.2396
29	Co	5.2578	6.9306	5.2602	0.7563	0.9970	0.2396
30	Co	3.4588	1.6680	5.3321	0.4975	0.2399	0.2429
31	Co	5.2633	3.4830	5.3342	0.7571	0.5010	0.2430
32	Co	3.4439	5.2528	5.2868	0.4954	0.7556	0.2408
33	C	3.0550	3.8254	6.6388	0.4395	0.5503	0.3024
34	O	2.9652	4.8033	7.4028	0.4265	0.6910	0.3372
35	O	3.9505	2.8514	6.7822	0.5683	0.4102	0.3090

/db/jmorales/CoNi-alloy/Adsorptions/CO-Ni-Ce_3SmO_7

a =				13.9019837372			
b =				13.9019837372			
c =				21.9509918687			
alpha =				90.0			
beta =				90.0			
gamma =				90.0			
	Atom	X	Y	Z	X	Y	Z
1	Ni	0.0000	0.0000	0.0000	0.0000	0.0000	0.0000
2	Ni	1.7377	1.7377	0.0000	0.1250	0.1250	0.0000
3	Ni	0.0000	3.4755	0.0000	0.0000	0.2500	0.0000
4	Ni	1.7377	5.2132	0.0000	0.1250	0.3750	0.0000
5	Ni	3.4755	0.0000	0.0000	0.2500	0.0000	0.0000
6	Ni	5.2132	1.7377	0.0000	0.3750	0.1250	0.0000
7	Ni	3.4755	3.4755	0.0000	0.2500	0.2500	0.0000
8	Ni	5.2132	5.2132	0.0000	0.3750	0.3750	0.0000
9	Ni	0.0000	6.9510	0.0000	0.0000	0.5000	0.0000
10	Ni	1.7377	8.6887	0.0000	0.1250	0.6250	0.0000
11	Ni	0.0000	10.4265	0.0000	0.0000	0.7500	0.0000
12	Ni	1.7377	12.1642	0.0000	0.1250	0.8750	0.0000
13	Ni	3.4755	6.9510	0.0000	0.2500	0.5000	0.0000
14	Ni	5.2132	8.6887	0.0000	0.3750	0.6250	0.0000
15	Ni	3.4755	10.4265	0.0000	0.2500	0.7500	0.0000
16	Ni	5.2132	12.1642	0.0000	0.3750	0.8750	0.0000
17	Ni	6.9510	0.0000	0.0000	0.5000	0.0000	0.0000
18	Ni	8.6887	1.7377	0.0000	0.6250	0.1250	0.0000
19	Ni	6.9510	3.4755	0.0000	0.5000	0.2500	0.0000
20	Ni	8.6887	5.2132	0.0000	0.6250	0.3750	0.0000
21	Ni	10.4265	0.0000	0.0000	0.7500	0.0000	0.0000
22	Ni	12.1642	1.7377	0.0000	0.8750	0.1250	0.0000
23	Ni	10.4265	3.4755	0.0000	0.7500	0.2500	0.0000
24	Ni	12.1642	5.2132	0.0000	0.8750	0.3750	0.0000
25	Ni	6.9510	6.9510	0.0000	0.5000	0.5000	0.0000
26	Ni	8.6887	8.6887	0.0000	0.6250	0.6250	0.0000
27	Ni	6.9510	10.4265	0.0000	0.5000	0.7500	0.0000
28	Ni	8.6887	12.1642	0.0000	0.6250	0.8750	0.0000
29	Ni	10.4265	6.9510	0.0000	0.7500	0.5000	0.0000
30	Ni	12.1642	8.6887	0.0000	0.8750	0.6250	0.0000
31	Ni	10.4265	10.4265	0.0000	0.7500	0.7500	0.0000
32	Ni	12.1642	12.1642	0.0000	0.8750	0.8750	0.0000
33	Ni	0.0000	1.7377	1.7377	0.0000	0.1250	0.0792
34	Ni	1.7377	0.0000	1.7377	0.1250	0.0000	0.0792
35	Ni	0.0000	5.2132	1.7377	0.0000	0.3750	0.0792
36	Ni	1.7377	3.4755	1.7377	0.1250	0.2500	0.0792
37	Ni	3.4755	1.7377	1.7377	0.2500	0.1250	0.0792
38	Ni	5.2132	0.0000	1.7377	0.3750	0.0000	0.0792
39	Ni	3.4755	5.2132	1.7377	0.2500	0.3750	0.0792
40	Ni	5.2132	3.4755	1.7377	0.3750	0.2500	0.0792

41	Ni	0.0000	8.6887	1.7377	0.0000	0.6250	0.0792
42	Ni	1.7377	6.9510	1.7377	0.1250	0.5000	0.0792
43	Ni	0.0000	12.1642	1.7377	0.0000	0.8750	0.0792
44	Ni	1.7377	10.4265	1.7377	0.1250	0.7500	0.0792
45	Ni	3.4755	8.6887	1.7377	0.2500	0.6250	0.0792
46	Ni	5.2132	6.9510	1.7377	0.3750	0.5000	0.0792
47	Ni	3.4755	12.1642	1.7377	0.2500	0.8750	0.0792
48	Ni	5.2132	10.4265	1.7377	0.3750	0.7500	0.0792
49	Ni	6.9510	1.7377	1.7377	0.5000	0.1250	0.0792
50	Ni	8.6887	0.0000	1.7377	0.6250	0.0000	0.0792
51	Ni	6.9510	5.2132	1.7377	0.5000	0.3750	0.0792
52	Ni	8.6887	3.4755	1.7377	0.6250	0.2500	0.0792
53	Ni	10.4265	1.7377	1.7377	0.7500	0.1250	0.0792
54	Ni	12.1642	0.0000	1.7377	0.8750	0.0000	0.0792
55	Ni	10.4265	5.2132	1.7377	0.7500	0.3750	0.0792
56	Ni	12.1642	3.4755	1.7377	0.8750	0.2500	0.0792
57	Ni	6.9510	8.6887	1.7377	0.5000	0.6250	0.0792
58	Ni	8.6887	6.9510	1.7377	0.6250	0.5000	0.0792
59	Ni	6.9510	12.1642	1.7377	0.5000	0.8750	0.0792
60	Ni	8.6887	10.4265	1.7377	0.6250	0.7500	0.0792
61	Ni	10.4265	8.6887	1.7377	0.7500	0.6250	0.0792
62	Ni	12.1642	6.9510	1.7377	0.8750	0.5000	0.0792
63	Ni	10.4265	12.1642	1.7377	0.7500	0.8750	0.0792
64	Ni	12.1642	10.4265	1.7377	0.8750	0.7500	0.0792
65	Ni	0.0050	0.0024	3.5138	0.0004	0.0002	0.1601
66	Ni	1.7047	1.7576	3.4424	0.1226	0.1264	0.1568
67	Ni	13.8991	3.4798	3.5574	0.9998	0.2503	0.1621
68	Ni	1.7264	5.1985	3.5232	0.1242	0.3739	0.1605
69	Ni	3.4600	13.8634	3.4700	0.2489	0.9972	0.1581
70	Ni	5.2129	1.7214	3.4914	0.3750	0.1238	0.1591
71	Ni	3.4743	3.4881	3.4831	0.2499	0.2509	0.1587
72	Ni	5.2103	5.2043	3.5093	0.3748	0.3744	0.1599
73	Ni	13.8908	6.9535	3.5051	0.9992	0.5002	0.1597
74	Ni	1.7387	8.6887	3.5033	0.1251	0.6250	0.1596
75	Ni	13.9001	10.4255	3.5080	0.9999	0.7499	0.1598
76	Ni	1.7283	12.1666	3.5251	0.1243	0.8752	0.1606
77	Ni	3.4909	6.9320	3.5217	0.2511	0.4986	0.1604
78	Ni	5.2060	8.6797	3.5087	0.3745	0.6243	0.1598
79	Ni	3.4743	10.4217	3.5077	0.2499	0.7497	0.1598
80	Ni	5.2179	12.1490	3.5019	0.3753	0.8739	0.1595
81	Ni	6.9498	0.0055	3.5210	0.4999	0.0004	0.1604
82	Ni	8.6611	1.7245	3.5122	0.6230	0.1240	0.1600
83	Ni	6.9568	3.4792	3.4937	0.5004	0.2503	0.1592
84	Ni	8.6805	5.2279	3.4908	0.6244	0.3761	0.1590
85	Ni	10.4312	0.0190	3.5155	0.7503	0.0014	0.1602
86	Ni	12.1946	1.7465	3.5467	0.8772	0.1256	0.1616
87	Ni	10.4344	3.4673	3.4600	0.7506	0.2494	0.1576
88	Ni	12.1554	5.2049	3.5124	0.8744	0.3744	0.1600
89	Ni	6.9511	6.9640	3.4784	0.5000	0.5009	0.1585

90	Ni	8.7117	8.7057	3.4825	0.6267	0.6262	0.1586
91	Ni	6.9557	10.4472	3.4904	0.5003	0.7515	0.1590
92	Ni	8.6943	12.1680	3.5087	0.6254	0.8753	0.1598
93	Ni	10.4414	6.9444	3.5008	0.7511	0.4995	0.1595
94	Ni	12.1596	8.6933	3.5091	0.8747	0.6253	0.1599
95	Ni	10.4317	10.4353	3.5098	0.7504	0.7506	0.1599
96	Ni	12.1626	12.1685	3.5084	0.8749	0.8753	0.1598
97	Ni	0.0411	1.7442	5.3168	0.0030	0.1255	0.2422
98	Ni	1.7436	13.8607	5.3043	0.1254	0.9970	0.2416
99	Ni	13.8581	5.2369	5.2193	0.9968	0.3767	0.2378
100	Ni	1.7136	3.5330	5.4173	0.1233	0.2541	0.2468
101	Ni	3.4333	1.6959	5.0977	0.2470	0.1220	0.2322
102	Ni	5.1827	13.8635	5.1863	0.3728	0.9972	0.2363
103	Ni	3.4798	5.2189	5.2654	0.2503	0.3754	0.2399
104	Ni	5.2347	3.4379	5.2117	0.3765	0.2473	0.2374
105	Ni	13.8971	8.6876	5.2240	0.9997	0.6249	0.2380
106	Ni	1.7285	6.9734	5.1994	0.1243	0.5016	0.2369
107	Ni	13.8899	12.1565	5.2243	0.9991	0.8744	0.2380
108	Ni	1.7336	10.4230	5.2252	0.1247	0.7498	0.2380
109	Ni	3.4688	8.6825	5.2260	0.2495	0.6246	0.2381
110	Ni	5.2201	6.9939	5.2855	0.3755	0.5031	0.2408
111	Ni	3.4804	12.1352	5.2210	0.2504	0.8729	0.2379
112	Ni	5.2234	10.4087	5.2280	0.3757	0.7487	0.2382
113	Ni	6.9723	1.6955	5.3620	0.5015	0.1220	0.2443
114	Ni	8.6944	13.8903	5.2235	0.6254	0.9992	0.2380
115	Ni	7.0050	5.2468	5.2341	0.5039	0.3774	0.2384
116	Ni	8.7011	3.4874	5.1882	0.6259	0.2509	0.2364
117	Ni	10.3993	1.7672	5.2775	0.7480	0.1271	0.2404
118	Ni	12.1667	0.0101	5.2257	0.8752	0.0007	0.2381
119	Ni	10.4020	5.1905	5.2790	0.7482	0.3734	0.2405
120	Ni	12.0933	3.4722	5.3318	0.8699	0.2498	0.2429
121	Ni	6.9859	8.7257	5.1378	0.5025	0.6277	0.2341
122	Ni	8.7164	6.9920	5.1670	0.6270	0.5030	0.2354
123	Ni	6.9495	12.1585	5.2259	0.4999	0.8746	0.2381
124	Ni	8.6945	10.4325	5.2321	0.6254	0.7504	0.2384
125	Ni	10.4248	8.6937	5.2247	0.7499	0.6254	0.2380
126	Ni	12.1469	6.9536	5.2248	0.8738	0.5002	0.2380
127	Ni	10.4352	12.1741	5.2228	0.7506	0.8757	0.2379
128	Ni	12.1618	10.4263	5.2271	0.8748	0.7500	0.2381
129	O	1.8458	1.6261	6.3098	0.1328	0.1170	0.2874
130	O	5.9397	2.4974	6.7696	0.4273	0.1796	0.3084
131	O	2.7806	4.4474	6.8801	0.2000	0.3199	0.3134
132	O	4.9841	4.2351	8.8398	0.3585	0.3046	0.4027
133	O	6.0973	6.0564	6.7841	0.4386	0.4356	0.3091
134	O	8.0453	6.0765	8.8324	0.5787	0.4371	0.4024
135	O	5.3019	7.8624	8.8277	0.3814	0.5656	0.4022
136	Ce	4.0712	6.2513	8.1089	0.2928	0.4497	0.3694
137	Ce	3.8363	2.6143	7.7348	0.2760	0.1881	0.3524
138	Ce	7.2783	4.2372	8.0389	0.5235	0.3048	0.3662

139	Sm	7.2061	7.7779	7.8996	0.5183	0.5595	0.3599
140	C	10.3123	3.4761	6.3504	0.7418	0.2500	0.2893
141	O	10.0864	3.4745	7.5743	0.7255	0.2499	0.3451

/db/jmorales/CoNi-alloy/Adsorptions/Functionals/PBEsol/Ni(001)-CO

a =		6.9509918686					
b =		6.9509918686					
c =		21.9509918687					
alpha =		90.0					
beta =		90.0					
gamma =		90.0					
	Atom	X	Y	Z	X	Y	Z
1	Ni	0.0000	0.0000	0.0000	0.0000	0.0000	0.0000
2	Ni	1.7377	1.7377	0.0000	0.2500	0.2500	0.0000
3	Ni	0.0000	3.4755	0.0000	0.0000	0.5000	0.0000
4	Ni	1.7377	5.2132	0.0000	0.2500	0.7500	0.0000
5	Ni	3.4755	0.0000	0.0000	0.5000	0.0000	0.0000
6	Ni	5.2132	1.7377	0.0000	0.7500	0.2500	0.0000
7	Ni	3.4755	3.4755	0.0000	0.5000	0.5000	0.0000
8	Ni	5.2132	5.2132	0.0000	0.7500	0.7500	0.0000
9	Ni	0.0000	1.7377	1.7377	0.0000	0.2500	0.0792
10	Ni	1.7377	0.0000	1.7377	0.2500	0.0000	0.0792
11	Ni	0.0000	5.2132	1.7377	0.0000	0.7500	0.0792
12	Ni	1.7377	3.4755	1.7377	0.2500	0.5000	0.0792
13	Ni	3.4755	1.7377	1.7377	0.5000	0.2500	0.0792
14	Ni	5.2132	0.0000	1.7377	0.7500	0.0000	0.0792
15	Ni	3.4755	5.2132	1.7377	0.5000	0.7500	0.0792
16	Ni	5.2132	3.4755	1.7377	0.7500	0.5000	0.0792
17	Ni	-0.0001	0.0001	3.4512	-0.0000	0.0000	0.1572
18	Ni	1.7371	1.7371	3.4474	0.2499	0.2499	0.1571
19	Ni	-0.0000	3.4755	3.4545	-0.0000	0.5000	0.1574
20	Ni	1.7370	5.2139	3.4473	0.2499	0.7501	0.1570
21	Ni	3.4754	0.0000	3.4545	0.5000	0.0000	0.1574
22	Ni	5.2137	1.7372	3.4474	0.7501	0.2499	0.1570
23	Ni	3.4754	3.4755	3.4068	0.5000	0.5000	0.1552
24	Ni	5.2138	5.2139	3.4473	0.7501	0.7501	0.1570
25	Ni	-0.0000	1.7385	5.0968	-0.0000	0.2501	0.2322
26	Ni	1.7383	-0.0000	5.0969	0.2501	-0.0000	0.2322
27	Ni	6.9509	5.2126	5.0970	1.0000	0.7499	0.2322
28	Ni	1.7617	3.4757	5.1432	0.2534	0.5000	0.2343
29	Ni	3.4753	1.7616	5.1433	0.5000	0.2534	0.2343
30	Ni	5.2124	-0.0001	5.0967	0.7499	-0.0000	0.2322
31	Ni	3.4753	5.1892	5.1432	0.5000	0.7465	0.2343
32	Ni	5.1894	3.4756	5.1431	0.7466	0.5000	0.2343
33	O	3.4752	3.4757	7.3990	0.5000	0.5000	0.3371
34	C	3.4754	3.4755	6.1853	0.5000	0.5000	0.2818

/db/jmorales/CoNi-alloy/Adsorptions/Functionals/RPBE/CoNi(001)

a =			6.9516692132				
b =			6.9516692132				
c =			21.9516692133				
alpha =			90.0				
beta =			90.0				
gamma =			90.0				
	Atom	X	Y	Z	X	Y	Z
1	Ni	0.0000	0.0000	0.0000	0.0000	0.0000	0.0000
2	Ni	0.0000	3.4758	0.0000	0.0000	0.5000	0.0000
3	Ni	3.4758	0.0000	0.0000	0.5000	0.0000	0.0000
4	Ni	3.4758	3.4758	0.0000	0.5000	0.5000	0.0000
5	Ni	0.0000	1.7379	1.7379	0.0000	0.2500	0.0792
6	Ni	0.0000	5.2138	1.7379	0.0000	0.7500	0.0792
7	Ni	3.4758	1.7379	1.7379	0.5000	0.2500	0.0792
8	Ni	3.4758	5.2138	1.7379	0.5000	0.7500	0.0792
9	Ni	0.0000	0.0000	3.5085	0.0000	0.0000	0.1598
10	Ni	0.0000	3.4758	3.5085	0.0000	0.5000	0.1598
11	Ni	3.4758	0.0000	3.5085	0.5000	0.0000	0.1598
12	Ni	3.4758	3.4758	3.5085	0.5000	0.5000	0.1598
13	Ni	0.0000	1.7379	5.2381	0.0000	0.2500	0.2386
14	Ni	0.0000	5.2138	5.2381	0.0000	0.7500	0.2386
15	Ni	3.4758	1.7379	5.2381	0.5000	0.2500	0.2386
16	Ni	3.4758	5.2138	5.2381	0.5000	0.7500	0.2386
17	Co	1.7379	1.7379	0.0000	0.2500	0.2500	0.0000
18	Co	1.7379	5.2138	0.0000	0.2500	0.7500	0.0000
19	Co	5.2138	1.7379	0.0000	0.7500	0.2500	0.0000
20	Co	5.2138	5.2138	0.0000	0.7500	0.7500	0.0000
21	Co	1.7379	0.0000	1.7379	0.2500	0.0000	0.0792
22	Co	1.7379	3.4758	1.7379	0.2500	0.5000	0.0792
23	Co	5.2138	0.0000	1.7379	0.7500	0.0000	0.0792
24	Co	5.2138	3.4758	1.7379	0.7500	0.5000	0.0792
25	Co	1.7379	1.7379	3.5647	0.2500	0.2500	0.1624
26	Co	1.7379	5.2138	3.5647	0.2500	0.7500	0.1624
27	Co	5.2138	1.7379	3.5647	0.7500	0.2500	0.1624
28	Co	5.2138	5.2138	3.5647	0.7500	0.7500	0.1624
29	Co	1.7379	0.0000	5.2600	0.2500	0.0000	0.2396
30	Co	1.7379	3.4758	5.2600	0.2500	0.5000	0.2396
31	Co	5.2138	0.0000	5.2600	0.7500	0.0000	0.2396
32	Co	5.2138	3.4758	5.2600	0.7500	0.5000	0.2396

/db/jmorales/CoNi-alloy/Adsorptions/Expanded-models/CO_2-Ni(001)-expanded

a = 7.047964809
b = 7.047964809
c = 22.047964809
alpha = 90.0
beta = 90.0
gamma = 90.0

	Atom	X	Y	Z	X	Y	Z
1	Ni	0.0000	0.0000	0.0000	0.0000	0.0000	0.0000
2	Ni	1.7620	1.7620	0.0000	0.2500	0.2500	0.0000
3	Ni	0.0000	3.5240	0.0000	0.0000	0.5000	0.0000
4	Ni	1.7620	5.2860	0.0000	0.2500	0.7500	0.0000
5	Ni	3.5240	0.0000	0.0000	0.5000	0.0000	0.0000
6	Ni	5.2860	1.7620	0.0000	0.7500	0.2500	0.0000
7	Ni	3.5240	3.5240	0.0000	0.5000	0.5000	0.0000
8	Ni	5.2860	5.2860	0.0000	0.7500	0.7500	0.0000
9	Ni	0.0000	1.7620	1.7620	0.0000	0.2500	0.0799
10	Ni	1.7620	0.0000	1.7620	0.2500	0.0000	0.0799
11	Ni	0.0000	5.2860	1.7620	0.0000	0.7500	0.0799
12	Ni	1.7620	3.5240	1.7620	0.2500	0.5000	0.0799
13	Ni	3.5240	1.7620	1.7620	0.5000	0.2500	0.0799
14	Ni	5.2860	0.0000	1.7620	0.7500	0.0000	0.0799
15	Ni	3.5240	5.2860	1.7620	0.5000	0.7500	0.0799
16	Ni	5.2860	3.5240	1.7620	0.7500	0.5000	0.0799
17	Ni	0.0000	0.0000	3.5240	0.0000	0.0000	0.1598
18	Ni	1.7620	1.7620	3.5240	0.2500	0.2500	0.1598
19	Ni	0.0000	3.5240	3.5240	0.0000	0.5000	0.1598
20	Ni	1.7620	5.2860	3.5240	0.2500	0.7500	0.1598
21	Ni	3.5240	0.0000	3.5240	0.5000	0.0000	0.1598
22	Ni	5.2860	1.7620	3.5240	0.7500	0.2500	0.1598
23	Ni	3.5240	3.5240	3.5240	0.5000	0.5000	0.1598
24	Ni	5.2860	5.2860	3.5240	0.7500	0.7500	0.1598
25	Ni	0.0000	1.7620	5.2860	0.0000	0.2500	0.2397
26	Ni	1.7620	0.0000	5.2860	0.2500	0.0000	0.2397
27	Ni	0.0000	5.2860	5.2860	0.0000	0.7500	0.2397
28	Ni	1.7620	3.5240	5.2860	0.2500	0.5000	0.2397
29	Ni	3.5240	1.7620	5.2860	0.5000	0.2500	0.2397
30	Ni	5.2860	0.0000	5.2860	0.7500	0.0000	0.2397
31	Ni	3.5240	5.2860	5.2860	0.5000	0.7500	0.2397
32	Ni	5.2860	3.5240	5.2860	0.7500	0.5000	0.2397
33	C	3.0944	3.8411	6.6418	0.4391	0.5450	0.3012
34	O	2.1446	3.9476	7.4476	0.3043	0.5601	0.3378
35	O	4.1052	2.9593	6.7530	0.5825	0.4199	0.3063

/db/jmorales/CoNi-alloy/Adsorptions/Coverage/Ni(001)-2CO

a =	6.9509918686
b =	6.9509918686
c =	21.9509918687
alpha =	90.0
beta =	90.0
gamma =	90.0

	Atom	X	Y	Z	X	Y	Z
1	Ni	0.0000	0.0000	0.0000	0.0000	0.0000	0.0000
2	Ni	1.7377	1.7377	0.0000	0.2500	0.2500	0.0000
3	Ni	0.0000	3.4755	0.0000	0.0000	0.5000	0.0000
4	Ni	1.7377	5.2132	0.0000	0.2500	0.7500	0.0000
5	Ni	3.4755	0.0000	0.0000	0.5000	0.0000	0.0000
6	Ni	5.2132	1.7377	0.0000	0.7500	0.2500	0.0000
7	Ni	3.4755	3.4755	0.0000	0.5000	0.5000	0.0000
8	Ni	5.2132	5.2132	0.0000	0.7500	0.7500	0.0000
9	Ni	0.0000	1.7377	1.7377	0.0000	0.2500	0.0792
10	Ni	1.7377	0.0000	1.7377	0.2500	0.0000	0.0792
11	Ni	0.0000	5.2132	1.7377	0.0000	0.7500	0.0792
12	Ni	1.7377	3.4755	1.7377	0.2500	0.5000	0.0792
13	Ni	3.4755	1.7377	1.7377	0.5000	0.2500	0.0792
14	Ni	5.2132	0.0000	1.7377	0.7500	0.0000	0.0792
15	Ni	3.4755	5.2132	1.7377	0.5000	0.7500	0.0792
16	Ni	5.2132	3.4755	1.7377	0.7500	0.5000	0.0792
17	Ni	-0.0038	-0.0022	3.4959	-0.0005	-0.0003	0.1593
18	Ni	1.7430	1.7445	3.4685	0.2507	0.2510	0.1580
19	Ni	0.0070	3.4768	3.5090	0.0010	0.5002	0.1599
20	Ni	1.7371	5.2067	3.5100	0.2499	0.7491	0.1599
21	Ni	3.4749	0.0084	3.5106	0.4999	0.0012	0.1599
22	Ni	5.2039	1.7379	3.5094	0.7487	0.2500	0.1599
23	Ni	3.4679	3.4704	3.4685	0.4989	0.4993	0.1580
24	Ni	5.2148	5.2168	3.4961	0.7502	0.7505	0.1593
25	Ni	0.0074	1.7402	5.2366	0.0011	0.2504	0.2386
26	Ni	1.7388	0.0192	5.2404	0.2502	0.0028	0.2387
27	Ni	0.0018	5.2135	5.2036	0.0003	0.7500	0.2371
28	Ni	1.7561	3.4505	5.3320	0.2526	0.4964	0.2429
29	Ni	3.4552	1.7626	5.3317	0.4971	0.2536	0.2429
30	Ni	5.2092	0.0004	5.2040	0.7494	0.0001	0.2371
31	Ni	3.4685	5.1994	5.2401	0.4990	0.7480	0.2387
32	Ni	5.1998	3.4773	5.2381	0.7481	0.5003	0.2386
33	O	3.6492	3.6467	7.6018	0.5250	0.5246	0.3463
34	O	1.5557	1.5709	7.6014	0.2238	0.2260	0.3463
35	C	3.6317	3.6295	6.3976	0.5225	0.5222	0.2914
36	C	1.5727	1.5896	6.3971	0.2263	0.2287	0.2914

/db/jmorales/CoNi-alloy/Adsorptions/Expanded-models/CO-Co-CoNi(001)-expanded

a = 7.044921511
b = 7.044921511
c = 22.044921511
alpha = 90.0
beta = 90.0
gamma = 90.0

	Atom	X	Y	Z	X	Y	Z
1	Ni	0.0000	0.0000	0.0000	0.0000	0.0000	0.0000
2	Ni	1.7612	1.7612	0.0000	0.2500	0.2500	0.0000
3	Ni	0.0000	0.0000	3.5225	0.0000	0.0000	0.1598
4	Ni	1.7612	1.7612	3.5225	0.2500	0.2500	0.1598
5	Ni	0.0000	3.5225	0.0000	0.0000	0.5000	0.0000
6	Ni	1.7612	5.2837	0.0000	0.2500	0.7500	0.0000
7	Ni	0.0000	3.5225	3.5225	0.0000	0.5000	0.1598
8	Ni	1.7612	5.2837	3.5225	0.2500	0.7500	0.1598
9	Ni	3.5225	0.0000	0.0000	0.5000	0.0000	0.0000
10	Ni	5.2837	1.7612	0.0000	0.7500	0.2500	0.0000
11	Ni	3.5225	0.0000	3.5225	0.5000	0.0000	0.1598
12	Ni	5.2837	1.7612	3.5225	0.7500	0.2500	0.1598
13	Ni	3.5225	3.5225	0.0000	0.5000	0.5000	0.0000
14	Ni	5.2837	5.2837	0.0000	0.7500	0.7500	0.0000
15	Ni	3.5225	3.5225	3.5225	0.5000	0.5000	0.1598
16	Ni	5.2837	5.2837	3.5225	0.7500	0.7500	0.1598
17	Co	0.0000	1.7612	1.7612	0.0000	0.2500	0.0799
18	Co	1.7612	0.0000	1.7612	0.2500	0.0000	0.0799
19	Co	0.0000	1.7612	5.2837	0.0000	0.2500	0.2397
20	Co	1.7612	0.0000	5.2837	0.2500	0.0000	0.2397
21	Co	0.0000	5.2837	1.7612	0.0000	0.7500	0.0799
22	Co	1.7612	3.5225	1.7612	0.2500	0.5000	0.0799
23	Co	0.0000	5.2837	5.2837	0.0000	0.7500	0.2397
24	Co	1.7612	3.5225	5.2837	0.2500	0.5000	0.2397
25	Co	3.5225	1.7612	1.7612	0.5000	0.2500	0.0799
26	Co	5.2837	0.0000	1.7612	0.7500	0.0000	0.0799
27	Co	3.5225	1.7612	5.2837	0.5000	0.2500	0.2397
28	Co	5.2837	0.0000	5.2837	0.7500	0.0000	0.2397
29	Co	3.5225	5.2837	1.7612	0.5000	0.7500	0.0799
30	Co	5.2837	3.5225	1.7612	0.7500	0.5000	0.0799
31	Co	3.5225	5.2837	5.2837	0.5000	0.7500	0.2397
32	Co	5.2837	3.5225	5.2837	0.7500	0.5000	0.2397
33	O	3.4725	3.4762	7.6442	0.4929	0.4934	0.3468
34	C	3.4751	3.4784	6.4323	0.4933	0.4937	0.2918

/db/jmorales/CoNi-alloy/Adsorptions/Expanded-models/CO-Ni-CoNi(001)-expanded

a = 7.044921511
b = 7.044921511
c = 22.044921511
alpha = 90.0
beta = 90.0
gamma = 90.0

	Atom	X	Y	Z	X	Y	Z
1	Co	0.0000	0.0000	0.0000	0.0000	0.0000	0.0000
2	Co	1.7612	1.7612	0.0000	0.2500	0.2500	0.0000
3	Co	0.0000	0.0000	3.5225	0.0000	0.0000	0.1598
4	Co	1.7612	1.7612	3.5225	0.2500	0.2500	0.1598
5	Co	0.0000	3.5225	0.0000	0.0000	0.5000	0.0000
6	Co	1.7612	5.2837	0.0000	0.2500	0.7500	0.0000
7	Co	0.0000	3.5225	3.5225	0.0000	0.5000	0.1598
8	Co	1.7612	5.2837	3.5225	0.2500	0.7500	0.1598
9	Co	3.5225	0.0000	0.0000	0.5000	0.0000	0.0000
10	Co	5.2837	1.7612	0.0000	0.7500	0.2500	0.0000
11	Co	3.5225	0.0000	3.5225	0.5000	0.0000	0.1598
12	Co	5.2837	1.7612	3.5225	0.7500	0.2500	0.1598
13	Co	3.5225	3.5225	0.0000	0.5000	0.5000	0.0000
14	Co	5.2837	5.2837	0.0000	0.7500	0.7500	0.0000
15	Co	3.5225	3.5225	3.5225	0.5000	0.5000	0.1598
16	Co	5.2837	5.2837	3.5225	0.7500	0.7500	0.1598
17	Ni	0.0000	1.7612	1.7612	0.0000	0.2500	0.0799
18	Ni	1.7612	0.0000	1.7612	0.2500	0.0000	0.0799
19	Ni	0.0000	1.7612	5.2837	0.0000	0.2500	0.2397
20	Ni	1.7612	0.0000	5.2837	0.2500	0.0000	0.2397
21	Ni	0.0000	5.2837	1.7612	0.0000	0.7500	0.0799
22	Ni	1.7612	3.5225	1.7612	0.2500	0.5000	0.0799
23	Ni	0.0000	5.2837	5.2837	0.0000	0.7500	0.2397
24	Ni	1.7612	3.5225	5.2837	0.2500	0.5000	0.2397
25	Ni	3.5225	1.7612	1.7612	0.5000	0.2500	0.0799
26	Ni	5.2837	0.0000	1.7612	0.7500	0.0000	0.0799
27	Ni	3.5225	1.7612	5.2837	0.5000	0.2500	0.2397
28	Ni	5.2837	0.0000	5.2837	0.7500	0.0000	0.2397
29	Ni	3.5225	5.2837	1.7612	0.5000	0.7500	0.0799
30	Ni	5.2837	3.5225	1.7612	0.7500	0.5000	0.0799
31	Ni	3.5225	5.2837	5.2837	0.5000	0.7500	0.2397
32	Ni	5.2837	3.5225	5.2837	0.7500	0.5000	0.2397
33	O	3.4688	3.4788	7.5487	0.4924	0.4938	0.3424
34	C	3.4730	3.4771	6.3353	0.4930	0.4936	0.2874

/db/jmorales/CoNi-alloy/Adsorptions/Expanded-models/CO_2-Co-CoNi(001)-expanded

a =				7.044921511			
b =				7.044921511			
c =				22.044921511			
alpha =				90.0			
beta =				90.0			
gamma =				90.0			
	Atom	X	Y	Z	X	Y	Z
1	Ni	0.0000	0.0000	0.0000	0.0000	0.0000	0.0000
2	Ni	1.7612	1.7612	0.0000	0.2500	0.2500	0.0000
3	Ni	7.0420	7.0392	3.4964	0.9996	0.9992	0.1586
4	Ni	1.7768	1.7673	3.5200	0.2522	0.2509	0.1597
5	Ni	0.0000	3.5225	0.0000	0.0000	0.5000	0.0000
6	Ni	1.7612	5.2837	0.0000	0.2500	0.7500	0.0000
7	Ni	7.0263	3.5203	3.5074	0.9974	0.4997	0.1591
8	Ni	1.7716	5.2771	3.5368	0.2515	0.7491	0.1604
9	Ni	3.5225	0.0000	0.0000	0.5000	0.0000	0.0000
10	Ni	5.2837	1.7612	0.0000	0.7500	0.2500	0.0000
11	Ni	3.5269	7.0428	3.5202	0.5006	0.9997	0.1597
12	Ni	5.2763	1.7592	3.5364	0.7489	0.2497	0.1604
13	Ni	3.5225	3.5225	0.0000	0.5000	0.5000	0.0000
14	Ni	5.2837	5.2837	0.0000	0.7500	0.7500	0.0000
15	Ni	3.5322	3.5238	3.5509	0.5014	0.5002	0.1611
16	Ni	5.2587	5.2714	3.5594	0.7465	0.7483	0.1615
17	Co	0.0000	1.7612	1.7612	0.0000	0.2500	0.0799
18	Co	1.7612	0.0000	1.7612	0.2500	0.0000	0.0799
19	Co	0.0320	1.7857	5.2267	0.0045	0.2535	0.2371
20	Co	1.6703	0.0254	5.2290	0.2371	0.0036	0.2372
21	Co	0.0000	5.2837	1.7612	0.0000	0.7500	0.0799
22	Co	1.7612	3.5225	1.7612	0.2500	0.5000	0.0799
23	Co	7.0393	5.2402	5.2241	0.9992	0.7438	0.2370
24	Co	1.7730	3.5232	5.2645	0.2517	0.5001	0.2388
25	Co	3.5225	1.7612	1.7612	0.5000	0.2500	0.0799
26	Co	5.2837	0.0000	1.7612	0.7500	0.0000	0.0799
27	Co	3.4915	1.6315	5.3030	0.4956	0.2316	0.2406
28	Co	5.3789	7.0391	5.2424	0.7635	0.9992	0.2378
29	Co	3.5225	5.2837	1.7612	0.5000	0.7500	0.0799
30	Co	5.2837	3.5225	1.7612	0.7500	0.5000	0.0799
31	Co	3.4894	5.4758	5.3469	0.4953	0.7773	0.2425
32	Co	5.3512	3.4780	5.3148	0.7596	0.4937	0.2411
33	C	3.1201	3.8652	6.6075	0.4429	0.5487	0.2997
34	O	3.1336	4.9297	7.3032	0.4448	0.6998	0.3313
35	O	4.0303	2.8882	6.7746	0.5721	0.4100	0.3073

/db/jmorales/CoNi-alloy/Adsorptions/CO_2-CoNi(111)

a =				9.831199646			
b =				9.831199645902503			
c =				21.020299912			
alpha =				90.0			
beta =				90.0			
gamma =				120.0			
	Atom	X	Y	Z	X	Y	Z
1	Ni	0.0000	0.0000	0.0000	0.0000	0.0000	0.0000
2	Ni	-1.2289	2.1285	0.0000	0.0000	0.2500	0.0000
3	Ni	-2.4578	4.2570	0.0000	-0.0000	0.5000	0.0000
4	Ni	-3.6867	6.3856	0.0000	-0.0000	0.7500	0.0000
5	Ni	4.9156	0.0000	0.0000	0.5000	0.0000	0.0000
6	Ni	3.6867	2.1285	0.0000	0.5000	0.2500	0.0000
7	Ni	2.4578	4.2570	0.0000	0.5000	0.5000	0.0000
8	Ni	1.2289	6.3856	0.0000	0.5000	0.7500	0.0000
9	Ni	2.4578	1.4190	2.0068	0.3333	0.1667	0.0955
10	Ni	1.2289	3.5475	2.0068	0.3333	0.4167	0.0955
11	Ni	0.0000	5.6760	2.0068	0.3333	0.6667	0.0955
12	Ni	-1.2289	7.8046	2.0068	0.3333	0.9167	0.0955
13	Ni	7.3734	1.4190	2.0068	0.8333	0.1667	0.0955
14	Ni	6.1445	3.5475	2.0068	0.8333	0.4167	0.0955
15	Ni	4.9156	5.6760	2.0068	0.8333	0.6667	0.0955
16	Ni	3.6867	7.8046	2.0068	0.8333	0.9167	0.0955
17	Ni	0.0452	2.8520	4.0233	0.1721	0.3350	0.1914
18	Ni	1.2638	0.7408	4.0224	0.1721	0.0870	0.1914
19	Ni	-2.4272	7.1188	4.0093	0.1712	0.8361	0.1907
20	Ni	-1.1909	4.9798	4.0153	0.1713	0.5849	0.1910
21	Ni	4.9465	2.8598	4.0084	0.6711	0.3359	0.1907
22	Ni	6.1779	0.7290	4.0093	0.6712	0.0856	0.1907
23	Ni	2.4910	7.1124	4.0113	0.6711	0.8354	0.1908
24	Ni	3.7155	4.9822	4.0128	0.6705	0.5852	0.1909
25	Ni	0.0448	0.0245	6.0154	0.0060	0.0029	0.2862
26	Ni	-1.1896	2.1548	6.0144	0.0055	0.2531	0.2861
27	Ni	-2.4062	4.2907	6.0213	0.0072	0.5040	0.2865
28	Ni	-3.6296	6.4182	6.0219	0.0077	0.7538	0.2865
29	Ni	4.9747	0.0284	6.0224	0.5077	0.0033	0.2865
30	Ni	3.7765	2.1517	6.0097	0.5105	0.2527	0.2859
31	Ni	2.5363	4.2974	6.1049	0.5104	0.5047	0.2904
32	Ni	1.2854	6.4291	6.0204	0.5083	0.7551	0.2864
33	Co	1.2289	2.1285	0.0000	0.2500	0.2500	0.0000
34	Co	2.4578	0.0000	0.0000	0.2500	0.0000	0.0000
35	Co	-1.2289	6.3856	0.0000	0.2500	0.7500	0.0000
36	Co	0.0000	4.2570	0.0000	0.2500	0.5000	0.0000
37	Co	6.1445	2.1285	0.0000	0.7500	0.2500	0.0000
38	Co	7.3734	0.0000	0.0000	0.7500	0.0000	0.0000
39	Co	3.6867	6.3856	0.0000	0.7500	0.7500	0.0000
40	Co	4.9156	4.2570	0.0000	0.7500	0.5000	0.0000

41	Co	-1.2289	3.5475	2.0068	0.0833	0.4167	0.0955
42	Co	0.0000	1.4190	2.0068	0.0833	0.1667	0.0955
43	Co	-3.6867	7.8046	2.0068	0.0833	0.9167	0.0955
44	Co	-2.4578	5.6760	2.0068	0.0833	0.6667	0.0955
45	Co	3.6867	3.5475	2.0068	0.5833	0.4167	0.0955
46	Co	4.9156	1.4190	2.0068	0.5833	0.1667	0.0955
47	Co	1.2289	7.8046	2.0068	0.5833	0.9167	0.0955
48	Co	2.4578	5.6760	2.0068	0.5833	0.6667	0.0955
49	Co	3.7149	0.7298	4.0491	0.4207	0.0857	0.1926
50	Co	2.4787	2.8566	4.0796	0.4199	0.3355	0.1941
51	Co	1.2650	4.9794	4.0550	0.4211	0.5848	0.1929
52	Co	0.0302	7.1112	4.0537	0.4207	0.8352	0.1928
53	Co	8.6360	0.7267	4.0496	0.9211	0.0854	0.1927
54	Co	7.4062	2.8558	4.0503	0.9211	0.3354	0.1927
55	Co	6.1733	4.9873	4.0509	0.9208	0.5858	0.1927
56	Co	4.9459	7.1080	4.0534	0.9205	0.8349	0.1928
57	Co	1.2959	2.1381	6.1176	0.2574	0.2511	0.2910
58	Co	2.5369	0.0367	6.0098	0.2602	0.0043	0.2859
59	Co	-1.1561	6.4334	6.0122	0.2602	0.7556	0.2860
60	Co	0.0515	4.3281	5.9985	0.2594	0.5083	0.2854
61	Co	6.2254	2.1690	6.0171	0.7606	0.2548	0.2862
62	Co	7.4437	0.0445	6.0142	0.7598	0.0052	0.2861
63	Co	3.7640	6.4315	6.0099	0.7606	0.7554	0.2859
64	Co	5.0018	4.3016	6.0068	0.7614	0.5052	0.2858
65	C	2.1246	3.7757	7.9644	0.4378	0.4435	0.3789
66	O	2.6329	4.5174	8.7807	0.5331	0.5306	0.4177
67	O	1.3947	2.7250	8.0173	0.3019	0.3201	0.3814

/db/jmorales/CoNi-alloy/Adsorptions/CO-Sm(surf)$Ce_{26}O_{54}$

a = 11.6082000732
b = 11.608200073110298
c = 22.898399353
alpha = 90.0
beta = 90.0
gamma = 120.0

	Atom	X	Y	Z	X	Y	Z
1	Sm	1.9356	3.3468	7.1309	0.3332	0.3329	0.3114
2	Ce	1.9347	1.1170	0.7898	0.2222	0.1111	0.0345
3	Ce	0.0000	4.4680	0.7898	0.2222	0.4444	0.0345
4	Ce	-1.9347	7.8190	0.7898	0.2222	0.7778	0.0345
5	Ce	5.8041	1.1170	0.7898	0.5556	0.1111	0.0345
6	Ce	3.8694	4.4680	0.7898	0.5556	0.4444	0.0345
7	Ce	1.9347	7.8190	0.7898	0.5556	0.7778	0.0345
8	Ce	9.6735	1.1170	0.7898	0.8889	0.1111	0.0345
9	Ce	7.7388	4.4680	0.7898	0.8889	0.4444	0.0345
10	Ce	5.8041	7.8190	0.7898	0.8889	0.7778	0.0345
11	Ce	0.0071	2.2382	3.9697	0.1119	0.2226	0.1734
12	Ce	-1.9339	5.5844	3.9482	0.1111	0.5555	0.1724
13	Ce	-3.8694	8.9366	3.9482	0.1111	0.8889	0.1724
14	Ce	3.8622	2.2382	3.9696	0.4440	0.2226	0.1734
15	Ce	1.9347	5.5765	3.9700	0.4440	0.5547	0.1734
16	Ce	0.0005	8.9363	3.9566	0.4445	0.8889	0.1728
17	Ce	7.7388	2.2334	3.9565	0.7777	0.2222	0.1728
18	Ce	5.8035	5.5845	3.9483	0.7777	0.5555	0.1724
19	Ce	3.8688	8.9363	3.9567	0.7777	0.8889	0.1728
20	Ce	0.0044	-0.0035	7.1054	0.0002	-0.0004	0.3103
21	Ce	-1.9356	3.3558	7.1056	0.0002	0.3338	0.3103
22	Ce	-3.8692	6.7018	7.0913	0.0000	0.6666	0.3097
23	Ce	3.8649	-0.0034	7.1054	0.3328	-0.0003	0.3103
24	Ce	-0.0045	6.6999	7.1053	0.3328	0.6665	0.3103
25	Ce	7.7387	0.0002	7.1162	0.6667	0.0000	0.3108
26	Ce	5.8050	3.3561	7.1056	0.6670	0.3338	0.3103
27	Ce	3.8743	6.6999	7.1054	0.6670	0.6665	0.3103
28	O	0.0000	0.0000	0.0000	0.0000	0.0000	0.0000
29	O	-1.9347	3.3510	0.0000	-0.0000	0.3333	0.0000
30	O	-3.8694	6.7020	0.0000	-0.0000	0.6667	0.0000
31	O	3.8694	0.0000	0.0000	0.3333	0.0000	0.0000
32	O	1.9347	3.3510	0.0000	0.3333	0.3333	0.0000
33	O	0.0000	6.7020	0.0000	0.3333	0.6667	0.0000
34	O	7.7388	0.0000	0.0000	0.6667	0.0000	0.0000
35	O	5.8041	3.3510	0.0000	0.6667	0.3333	0.0000
36	O	3.8694	6.7020	0.0000	0.6667	0.6667	0.0000
37	O	-0.0000	2.2340	1.5798	0.1111	0.2222	0.0690
38	O	-1.9347	5.5850	1.5798	0.1111	0.5556	0.0690
39	O	-3.8694	8.9360	1.5798	0.1111	0.8889	0.0690
40	O	3.8694	2.2340	1.5798	0.4444	0.2222	0.0690

41	O	1.9347	5.5850	1.5798	0.4444	0.5556	0.0690
42	O	-0.0000	8.9360	1.5798	0.4444	0.8889	0.0690
43	O	7.7388	2.2340	1.5798	0.7778	0.2222	0.0690
44	O	5.8041	5.5850	1.5798	0.7778	0.5556	0.0690
45	O	3.8694	8.9360	1.5798	0.7778	0.8889	0.0690
46	O	1.9347	1.1170	3.1593	0.2222	0.1111	0.1380
47	O	0.0000	4.4680	3.1593	0.2222	0.4444	0.1380
48	O	-1.9347	7.8190	3.1593	0.2222	0.7778	0.1380
49	O	5.8041	1.1170	3.1593	0.5556	0.1111	0.1380
50	O	3.8694	4.4680	3.1593	0.5556	0.4444	0.1380
51	O	1.9347	7.8190	3.1593	0.5556	0.7778	0.1380
52	O	9.6735	1.1170	3.1593	0.8889	0.1111	0.1380
53	O	7.7388	4.4680	3.1593	0.8889	0.4444	0.1380
54	O	5.8041	7.8190	3.1593	0.8889	0.7778	0.1380
55	O	0.0015	-0.0021	4.7604	0.0000	-0.0002	0.2079
56	O	-1.9361	3.3534	4.7601	-0.0000	0.3336	0.2079
57	O	-3.8690	6.7012	4.7451	-0.0000	0.6666	0.2072
58	O	3.8678	-0.0022	4.7604	0.3331	-0.0002	0.2079
59	O	1.9347	3.3506	4.7395	0.3333	0.3333	0.2070
60	O	-0.0026	6.7019	4.7603	0.3331	0.6667	0.2079
61	O	7.7388	-0.0000	4.7621	0.6667	-0.0000	0.2080
62	O	5.8055	3.3536	4.7602	0.6669	0.3336	0.2079
63	O	3.8721	6.7017	4.7603	0.6669	0.6666	0.2079
64	O	-0.0263	2.2196	6.3156	0.1081	0.2208	0.2758
65	O	-1.9524	5.5950	6.2787	0.1101	0.5565	0.2742
66	O	-3.8696	8.9127	6.2779	0.1099	0.8866	0.2742
67	O	3.8956	2.2197	6.3154	0.4460	0.2208	0.2758
68	O	1.9347	5.6137	6.3154	0.4459	0.5584	0.2758
69	O	0.0019	8.9371	6.3015	0.4447	0.8890	0.2752
70	O	7.7388	2.2319	6.3014	0.7777	0.2220	0.2752
71	O	5.8231	5.5959	6.2786	0.7800	0.5566	0.2742
72	O	3.8674	8.9371	6.3013	0.7777	0.8890	0.2752
73	O	1.9347	0.9921	7.9086	0.2160	0.0987	0.3454
74	O	-0.1085	4.5312	7.9153	0.2160	0.4507	0.3457
75	O	-1.9387	7.8164	7.8924	0.2217	0.7775	0.3447
76	O	5.8152	1.1110	7.9075	0.5562	0.1105	0.3453
77	O	3.9781	4.5313	7.9138	0.5681	0.4507	0.3456
78	O	1.9348	7.8308	7.9078	0.5561	0.7790	0.3453
79	O	9.6621	1.1108	7.9077	0.8876	0.1105	0.3453
80	O	7.7390	4.4724	7.8924	0.8891	0.4449	0.3447
81	O	5.8079	7.8167	7.8927	0.8891	0.7776	0.3447
82	O	1.8804	3.6039	11.0467	0.3412	0.3585	0.4824
83	C	1.9079	3.4772	9.9122	0.3373	0.3459	0.4329

/db/jmorales/CoNi-alloy/Adsorptions/Expanded-models/CO_2-CoNi(001)-expanded

a =			7.044921511				
b =			7.044921511				
c =			22.044921511				
alpha =			90.0				
beta =			90.0				
gamma =			90.0				
	Atom	X	Y	Z	X	Y	Z
1	Ni	0.0000	0.0000	0.0000	0.0000	0.0000	0.0000
2	Ni	0.0000	3.5225	0.0000	0.0000	0.5000	0.0000
3	Ni	3.5225	0.0000	0.0000	0.5000	0.0000	0.0000
4	Ni	3.5225	3.5225	0.0000	0.5000	0.5000	0.0000
5	Ni	0.0000	1.7612	1.7612	0.0000	0.2500	0.0799
6	Ni	0.0000	5.2837	1.7612	0.0000	0.7500	0.0799
7	Ni	3.5225	1.7612	1.7612	0.5000	0.2500	0.0799
8	Ni	3.5225	5.2837	1.7612	0.5000	0.7500	0.0799
9	Ni	0.0000	0.0000	3.5225	0.0000	0.0000	0.1598
10	Ni	0.0000	3.5225	3.5225	0.0000	0.5000	0.1598
11	Ni	3.5225	0.0000	3.5225	0.5000	0.0000	0.1598
12	Ni	3.5225	3.5225	3.5225	0.5000	0.5000	0.1598
13	Ni	0.0000	1.7612	5.2837	0.0000	0.2500	0.2397
14	Ni	0.0000	5.2837	5.2837	0.0000	0.7500	0.2397
15	Ni	3.5225	1.7612	5.2837	0.5000	0.2500	0.2397
16	Ni	3.5225	5.2837	5.2837	0.5000	0.7500	0.2397
17	Co	1.7612	1.7612	0.0000	0.2500	0.2500	0.0000
18	Co	1.7612	5.2837	0.0000	0.2500	0.7500	0.0000
19	Co	5.2837	1.7612	0.0000	0.7500	0.2500	0.0000
20	Co	5.2837	5.2837	0.0000	0.7500	0.7500	0.0000
21	Co	1.7612	0.0000	1.7612	0.2500	0.0000	0.0799
22	Co	1.7612	3.5225	1.7612	0.2500	0.5000	0.0799
23	Co	5.2837	0.0000	1.7612	0.7500	0.0000	0.0799
24	Co	5.2837	3.5225	1.7612	0.7500	0.5000	0.0799
25	Co	1.7612	1.7612	3.5225	0.2500	0.2500	0.1598
26	Co	1.7612	5.2837	3.5225	0.2500	0.7500	0.1598
27	Co	5.2837	1.7612	3.5225	0.7500	0.2500	0.1598
28	Co	5.2837	5.2837	3.5225	0.7500	0.7500	0.1598
29	Co	1.7612	0.0000	5.2837	0.2500	0.0000	0.2397
30	Co	1.7612	3.5225	5.2837	0.2500	0.5000	0.2397
31	Co	5.2837	0.0000	5.2837	0.7500	0.0000	0.2397
32	Co	5.2837	3.5225	5.2837	0.7500	0.5000	0.2397
33	C	3.4788	4.0384	6.7746	0.4938	0.5732	0.3073
34	O	4.6280	3.7235	7.2197	0.6569	0.5285	0.3275
35	O	2.3288	3.7146	7.2128	0.3306	0.5273	0.3272

/db/jmorales/CoNi-alloy/Adsorptions/CO_2-Ni-CoNi(001)

a =			6.951669213				
b =			6.951669213				
c =			21.951669213				
alpha =			90.0				
beta =			90.0				
gamma =			90.0				
	Atom	X	Y	Z	X	Y	Z
1	Co	0.0000	0.0000	0.0000	0.0000	0.0000	0.0000
2	Co	1.7379	1.7379	0.0000	0.2500	0.2500	0.0000
3	Co	0.0000	3.4758	0.0000	0.0000	0.5000	0.0000
4	Co	1.7379	5.2138	0.0000	0.2500	0.7500	0.0000
5	Co	3.4758	0.0000	0.0000	0.5000	0.0000	0.0000
6	Co	5.2138	1.7379	0.0000	0.7500	0.2500	0.0000
7	Co	3.4758	3.4758	0.0000	0.5000	0.5000	0.0000
8	Co	5.2138	5.2138	0.0000	0.7500	0.7500	0.0000
9	Co	6.9496	6.9509	3.5032	0.9997	0.9999	0.1596
10	Co	1.7435	1.7482	3.4758	0.2508	0.2515	0.1583
11	Co	6.9184	3.4722	3.5201	0.9952	0.4995	0.1604
12	Co	1.7401	5.2095	3.4758	0.2503	0.7494	0.1583
13	Co	3.4708	0.0121	3.5315	0.4993	0.0017	0.1609
14	Co	5.2123	1.7362	3.4758	0.7498	0.2498	0.1583
15	Co	3.4672	3.4637	3.5067	0.4988	0.4982	0.1597
16	Co	5.2024	5.2055	3.4758	0.7484	0.7488	0.1583
17	Ni	1.7379	0.0000	1.7379	0.2500	0.0000	0.0792
18	Ni	0.0000	1.7379	1.7379	0.0000	0.2500	0.0792
19	Ni	1.7379	3.4758	1.7379	0.2500	0.5000	0.0792
20	Ni	0.0000	5.2138	1.7379	0.0000	0.7500	0.0792
21	Ni	5.2138	0.0000	1.7379	0.7500	0.0000	0.0792
22	Ni	3.4758	1.7379	1.7379	0.5000	0.2500	0.0792
23	Ni	5.2138	3.4758	1.7379	0.7500	0.5000	0.0792
24	Ni	3.4758	5.2138	1.7379	0.5000	0.7500	0.0792
25	Ni	1.7345	6.9514	5.1703	0.2495	1.0000	0.2355
26	Ni	0.0019	1.7025	5.1562	0.0003	0.2449	0.2349
27	Ni	1.6729	3.4749	5.2160	0.2406	0.4999	0.2376
28	Ni	6.9401	5.2466	5.1554	0.9983	0.7547	0.2349
29	Ni	5.2085	6.9252	5.1684	0.7492	0.9962	0.2354
30	Ni	3.4877	1.7189	5.2302	0.5017	0.2473	0.2383
31	Ni	5.2400	3.4770	5.2474	0.7538	0.5002	0.2390
32	Ni	3.4515	5.1621	5.2258	0.4965	0.7426	0.2381
33	C	3.0285	3.8311	6.6646	0.4357	0.5511	0.3036
34	O	2.0561	3.9811	7.4250	0.2958	0.5727	0.3382
35	O	4.0749	3.0344	6.8277	0.5862	0.4365	0.3110

/db/jmorales/CoNi-alloy/Adsorptions/Functionals/RPBE/Co-CoNi(001)

a =	6.9516692132
b =	6.9516692132
c =	21.9516692133
alpha =	90.0
beta =	90.0
gamma =	90.0

	Atom	X	Y	Z	X	Y	Z
1	Ni	0.0000	0.0000	0.0000	0.0000	0.0000	0.0000
2	Ni	1.7379	1.7379	0.0000	0.2500	0.2500	0.0000
3	Ni	0.0000	3.4758	0.0000	0.0000	0.5000	0.0000
4	Ni	1.7379	5.2138	0.0000	0.2500	0.7500	0.0000
5	Ni	3.4758	0.0000	0.0000	0.5000	0.0000	0.0000
6	Ni	5.2138	1.7379	0.0000	0.7500	0.2500	0.0000
7	Ni	3.4758	3.4758	0.0000	0.5000	0.5000	0.0000
8	Ni	5.2138	5.2138	0.0000	0.7500	0.7500	0.0000
9	Ni	0.0000	0.0000	3.5385	0.0000	0.0000	0.1612
10	Ni	1.7379	1.7379	3.5366	0.2500	0.2500	0.1611
11	Ni	0.0000	3.4758	3.5385	0.0000	0.5000	0.1612
12	Ni	1.7379	5.2138	3.5366	0.2500	0.7500	0.1611
13	Ni	3.4758	0.0000	3.5385	0.5000	0.0000	0.1612
14	Ni	5.2138	1.7379	3.5366	0.7500	0.2500	0.1611
15	Ni	3.4758	3.4758	3.5385	0.5000	0.5000	0.1612
16	Ni	5.2138	5.2138	3.5366	0.7500	0.7500	0.1611
17	Co	1.7379	0.0000	1.7379	0.2500	0.0000	0.0792
18	Co	0.0000	1.7379	1.7379	0.0000	0.2500	0.0792
19	Co	1.7379	3.4758	1.7379	0.2500	0.5000	0.0792
20	Co	0.0000	5.2138	1.7379	0.0000	0.7500	0.0792
21	Co	5.2138	0.0000	1.7379	0.7500	0.0000	0.0792
22	Co	3.4758	1.7379	1.7379	0.5000	0.2500	0.0792
23	Co	5.2138	3.4758	1.7379	0.7500	0.5000	0.0792
24	Co	3.4758	5.2138	1.7379	0.5000	0.7500	0.0792
25	Co	1.7379	0.0000	5.3089	0.2500	0.0000	0.2418
26	Co	0.0000	1.7379	5.3089	0.0000	0.2500	0.2418
27	Co	1.7379	3.4758	5.3089	0.2500	0.5000	0.2418
28	Co	0.0000	5.2138	5.3089	0.0000	0.7500	0.2418
29	Co	5.2138	0.0000	5.3089	0.7500	0.0000	0.2418
30	Co	3.4758	1.7379	5.3089	0.5000	0.2500	0.2418
31	Co	5.2138	3.4758	5.3089	0.7500	0.5000	0.2418
32	Co	3.4758	5.2138	5.3089	0.5000	0.7500	0.2418

/db/jmorales/CoNi-alloy/Adsorptions/Functionals/PBEsol/CoNi(111)

a = 9.831199646
b = 9.831199646075708
c = 21.0202999115
alpha = 90.0
beta = 90.0
gamma = 120.0

	Atom	X	Y	Z	X	Y	Z
1	Ni	0.0000	0.0000	0.0000	0.0000	0.0000	0.0000
2	Ni	-1.2289	2.1285	0.0000	0.0000	0.2500	0.0000
3	Ni	-2.4578	4.2570	0.0000	0.0000	0.5000	0.0000
4	Ni	-3.6867	6.3856	0.0000	0.0000	0.7500	0.0000
5	Ni	4.9156	0.0000	0.0000	0.5000	0.0000	0.0000
6	Ni	3.6867	2.1285	0.0000	0.5000	0.2500	0.0000
7	Ni	2.4578	4.2570	0.0000	0.5000	0.5000	0.0000
8	Ni	1.2289	6.3856	0.0000	0.5000	0.7500	0.0000
9	Ni	2.4578	1.4190	2.0068	0.3333	0.1667	0.0955
10	Ni	1.2289	3.5475	2.0068	0.3333	0.4167	0.0955
11	Ni	0.0000	5.6760	2.0068	0.3333	0.6667	0.0955
12	Ni	-1.2289	7.8045	2.0068	0.3333	0.9167	0.0955
13	Ni	7.3734	1.4190	2.0068	0.8333	0.1667	0.0955
14	Ni	6.1445	3.5475	2.0068	0.8333	0.4167	0.0955
15	Ni	4.9156	5.6760	2.0068	0.8333	0.6667	0.0955
16	Ni	3.6867	7.8045	2.0068	0.8333	0.9167	0.0955
17	Ni	0.0305	2.8555	3.9710	0.1708	0.3354	0.1889
18	Ni	1.2594	0.7270	3.9710	0.1708	0.0854	0.1889
19	Ni	-2.4273	7.1125	3.9710	0.1708	0.8354	0.1889
20	Ni	-1.1984	4.9840	3.9710	0.1708	0.5854	0.1889
21	Ni	4.9461	2.8555	3.9710	0.6708	0.3354	0.1889
22	Ni	6.1750	0.7270	3.9710	0.6708	0.0854	0.1889
23	Ni	2.4883	7.1125	3.9710	0.6708	0.8354	0.1889
24	Ni	3.7172	4.9840	3.9710	0.6708	0.5854	0.1889
25	Ni	0.0497	0.0288	5.9347	0.0067	0.0034	0.2823
26	Ni	-1.1792	2.1573	5.9347	0.0067	0.2534	0.2823
27	Ni	-2.4081	4.2858	5.9347	0.0067	0.5034	0.2823
28	Ni	-3.6370	6.4143	5.9347	0.0067	0.7534	0.2823
29	Ni	4.9653	0.0288	5.9347	0.5067	0.0034	0.2823
30	Ni	3.7364	2.1573	5.9347	0.5067	0.2534	0.2823
31	Ni	2.5075	4.2858	5.9347	0.5067	0.5034	0.2823
32	Ni	1.2786	6.4143	5.9347	0.5067	0.7534	0.2823
33	Co	1.2289	2.1285	0.0000	0.2500	0.2500	0.0000
34	Co	2.4578	0.0000	0.0000	0.2500	0.0000	0.0000
35	Co	-1.2289	6.3856	0.0000	0.2500	0.7500	0.0000
36	Co	0.0000	4.2570	0.0000	0.2500	0.5000	0.0000
37	Co	6.1445	2.1285	0.0000	0.7500	0.2500	0.0000
38	Co	7.3734	0.0000	0.0000	0.7500	0.0000	0.0000
39	Co	3.6867	6.3856	0.0000	0.7500	0.7500	0.0000
40	Co	4.9156	4.2570	0.0000	0.7500	0.5000	0.0000
41	Co	-1.2289	3.5475	2.0068	0.0833	0.4167	0.0955

42	Co	0.0000	1.4190	2.0068	0.0833	0.1667	0.0955
43	Co	-3.6867	7.8045	2.0068	0.0833	0.9167	0.0955
44	Co	-2.4578	5.6760	2.0068	0.0833	0.6667	0.0955
45	Co	3.6867	3.5475	2.0068	0.5833	0.4167	0.0955
46	Co	4.9156	1.4190	2.0068	0.5833	0.1667	0.0955
47	Co	1.2289	7.8045	2.0068	0.5833	0.9167	0.0955
48	Co	2.4578	5.6760	2.0068	0.5833	0.6667	0.0955
49	Co	3.7137	0.7251	4.0076	0.4203	0.0852	0.1907
50	Co	2.4848	2.8536	4.0076	0.4203	0.3352	0.1907
51	Co	1.2559	4.9821	4.0076	0.4203	0.5852	0.1907
52	Co	0.0270	7.1107	4.0076	0.4203	0.8352	0.1907
53	Co	8.6293	0.7251	4.0076	0.9203	0.0852	0.1907
54	Co	7.4004	2.8536	4.0076	0.9203	0.3352	0.1907
55	Co	6.1715	4.9821	4.0076	0.9203	0.5852	0.1907
56	Co	4.9426	7.1107	4.0076	0.9203	0.8352	0.1907
57	Co	1.3010	2.1701	5.9255	0.2598	0.2549	0.2819
58	Co	2.5299	0.0416	5.9255	0.2598	0.0049	0.2819
59	Co	-1.1568	6.4272	5.9255	0.2598	0.7549	0.2819
60	Co	0.0721	4.2986	5.9255	0.2598	0.5049	0.2819
61	Co	6.2166	2.1701	5.9255	0.7598	0.2549	0.2819
62	Co	7.4455	0.0416	5.9255	0.7598	0.0049	0.2819
63	Co	3.7588	6.4272	5.9255	0.7598	0.7549	0.2819
64	Co	4.9877	4.2986	5.9255	0.7598	0.5049	0.2819

/db/jmorales/CoNi-alloy/Adsorptions/Functionals/RPBE/Ni(111)

a =			9.8299999236				
b =			9.829999923841429				
c =			21.0196990967				
alpha =			90.0				
beta =			90.0				
gamma =			120.0				
	Atom	X	Y	Z	X	Y	Z
1	Ni	0.0000	0.0000	0.0000	0.0000	0.0000	0.0000
2	Ni	-1.2287	2.1283	0.0000	0.0000	0.2500	0.0000
3	Ni	-2.4575	4.2565	0.0000	0.0000	0.5000	0.0000
4	Ni	-3.6862	6.3848	0.0000	-0.0000	0.7500	0.0000
5	Ni	2.4575	0.0000	0.0000	0.2500	0.0000	0.0000
6	Ni	1.2287	2.1283	0.0000	0.2500	0.2500	0.0000
7	Ni	0.0000	4.2565	0.0000	0.2500	0.5000	0.0000
8	Ni	-1.2287	6.3848	0.0000	0.2500	0.7500	0.0000
9	Ni	4.9150	0.0000	0.0000	0.5000	0.0000	0.0000
10	Ni	3.6862	2.1283	0.0000	0.5000	0.2500	0.0000
11	Ni	2.4575	4.2565	0.0000	0.5000	0.5000	0.0000
12	Ni	1.2287	6.3848	0.0000	0.5000	0.7500	0.0000
13	Ni	7.3725	0.0000	0.0000	0.7500	0.0000	0.0000
14	Ni	6.1437	2.1283	0.0000	0.7500	0.2500	0.0000
15	Ni	4.9150	4.2565	0.0000	0.7500	0.5000	0.0000
16	Ni	3.6862	6.3848	0.0000	0.7500	0.7500	0.0000
17	Ni	-0.0000	1.4188	2.0065	0.0833	0.1667	0.0955
18	Ni	-1.2288	3.5471	2.0065	0.0833	0.4167	0.0955
19	Ni	-2.4575	5.6754	2.0065	0.0833	0.6667	0.0955
20	Ni	-3.6863	7.8036	2.0065	0.0833	0.9167	0.0955
21	Ni	2.4575	1.4188	2.0065	0.3333	0.1667	0.0955
22	Ni	1.2287	3.5471	2.0065	0.3333	0.4167	0.0955
23	Ni	-0.0000	5.6754	2.0065	0.3333	0.6667	0.0955
24	Ni	-1.2288	7.8036	2.0065	0.3333	0.9167	0.0955
25	Ni	4.9150	1.4188	2.0065	0.5833	0.1667	0.0955
26	Ni	3.6862	3.5471	2.0065	0.5833	0.4167	0.0955
27	Ni	2.4575	5.6754	2.0065	0.5833	0.6667	0.0955
28	Ni	1.2287	7.8036	2.0065	0.5833	0.9167	0.0955
29	Ni	7.3725	1.4188	2.0065	0.8333	0.1667	0.0955
30	Ni	6.1437	3.5471	2.0065	0.8333	0.4167	0.0955
31	Ni	4.9150	5.6754	2.0065	0.8333	0.6667	0.0955
32	Ni	3.6862	7.8036	2.0065	0.8333	0.9167	0.0955
33	Ni	1.2288	0.7094	4.0160	0.1667	0.0833	0.1911
34	Ni	0.0000	2.8377	4.0160	0.1667	0.3333	0.1911
35	Ni	-1.2287	4.9659	4.0160	0.1667	0.5833	0.1911
36	Ni	-2.4575	7.0942	4.0160	0.1667	0.8333	0.1911
37	Ni	3.6863	0.7094	4.0160	0.4167	0.0833	0.1911
38	Ni	2.4575	2.8377	4.0160	0.4167	0.3333	0.1911
39	Ni	1.2288	4.9659	4.0160	0.4167	0.5833	0.1911
40	Ni	0.0000	7.0942	4.0160	0.4167	0.8333	0.1911
41	Ni	6.1438	0.7094	4.0160	0.6667	0.0833	0.1911

42	Ni	4.9150	2.8377	4.0160	0.6667	0.3333	0.1911
43	Ni	3.6863	4.9659	4.0160	0.6667	0.5833	0.1911
44	Ni	2.4575	7.0942	4.0160	0.6667	0.8333	0.1911
45	Ni	8.6013	0.7094	4.0160	0.9167	0.0833	0.1911
46	Ni	7.3725	2.8377	4.0160	0.9167	0.3333	0.1911
47	Ni	6.1438	4.9659	4.0160	0.9167	0.5833	0.1911
48	Ni	4.9150	7.0942	4.0160	0.9167	0.8333	0.1911
49	Ni	0.0000	0.0000	6.0466	0.0000	0.0000	0.2877
50	Ni	-1.2287	2.1283	6.0466	0.0000	0.2500	0.2877
51	Ni	-2.4575	4.2565	6.0466	0.0000	0.5000	0.2877
52	Ni	-3.6862	6.3848	6.0466	-0.0000	0.7500	0.2877
53	Ni	2.4575	0.0000	6.0466	0.2500	0.0000	0.2877
54	Ni	1.2287	2.1283	6.0466	0.2500	0.2500	0.2877
55	Ni	0.0000	4.2565	6.0466	0.2500	0.5000	0.2877
56	Ni	-1.2287	6.3848	6.0466	0.2500	0.7500	0.2877
57	Ni	4.9150	0.0000	6.0466	0.5000	0.0000	0.2877
58	Ni	3.6862	2.1283	6.0466	0.5000	0.2500	0.2877
59	Ni	2.4575	4.2565	6.0466	0.5000	0.5000	0.2877
60	Ni	1.2287	6.3848	6.0466	0.5000	0.7500	0.2877
61	Ni	7.3725	0.0000	6.0466	0.7500	0.0000	0.2877
62	Ni	6.1437	2.1283	6.0466	0.7500	0.2500	0.2877
63	Ni	4.9150	4.2565	6.0466	0.7500	0.5000	0.2877
64	Ni	3.6862	6.3848	6.0466	0.7500	0.7500	0.2877

/db/jmorales/CoNi-alloy/Adsorptions/Coverage/Co-CoNi(001)-2CO

a = 6.9516692132
b = 6.9516692132
c = 21.9516692133
alpha = 90.0
beta = 90.0
gamma = 90.0

	Atom	X	Y	Z	X	Y	Z
1	Ni	0.0000	0.0000	0.0000	0.0000	0.0000	0.0000
2	Ni	1.7379	1.7379	0.0000	0.2500	0.2500	0.0000
3	Ni	0.0000	3.4758	0.0000	0.0000	0.5000	0.0000
4	Ni	1.7379	5.2138	0.0000	0.2500	0.7500	0.0000
5	Ni	3.4758	0.0000	0.0000	0.5000	0.0000	0.0000
6	Ni	5.2138	1.7379	0.0000	0.7500	0.2500	0.0000
7	Ni	3.4758	3.4758	0.0000	0.5000	0.5000	0.0000
8	Ni	5.2138	5.2138	0.0000	0.7500	0.7500	0.0000
9	Ni	6.9470	-0.0031	3.5232	0.9993	-0.0004	0.1605
10	Ni	1.7382	1.7387	3.5012	0.2500	0.2501	0.1595
11	Ni	0.0083	3.4779	3.5391	0.0012	0.5003	0.1612
12	Ni	1.7350	5.2056	3.5378	0.2496	0.7488	0.1612
13	Ni	3.4769	0.0096	3.5400	0.5002	0.0014	0.1613
14	Ni	5.2040	1.7369	3.5408	0.7486	0.2499	0.1613
15	Ni	3.4744	3.4758	3.5018	0.4998	0.5000	0.1595
16	Ni	5.2167	5.2186	3.5233	0.7504	0.7507	0.1605
17	Co	1.7379	0.0000	1.7379	0.2500	0.0000	0.0792
18	Co	0.0000	1.7379	1.7379	0.0000	0.2500	0.0792
19	Co	1.7379	3.4758	1.7379	0.2500	0.5000	0.0792
20	Co	0.0000	5.2138	1.7379	0.0000	0.7500	0.0792
21	Co	5.2138	0.0000	1.7379	0.7500	0.0000	0.0792
22	Co	3.4758	1.7379	1.7379	0.5000	0.2500	0.0792
23	Co	5.2138	3.4758	1.7379	0.7500	0.5000	0.0792
24	Co	3.4758	5.2138	1.7379	0.5000	0.7500	0.0792
25	Co	1.7354	0.0189	5.2811	0.2496	0.0027	0.2406
26	Co	0.0092	1.7392	5.2796	0.0013	0.2502	0.2405
27	Co	1.7685	3.4423	5.3425	0.2544	0.4952	0.2434
28	Co	0.0059	5.2113	5.2676	0.0009	0.7497	0.2400
29	Co	5.2106	-0.0014	5.2689	0.7495	-0.0002	0.2400
30	Co	3.4478	1.7701	5.3416	0.4960	0.2546	0.2433
31	Co	5.1981	3.4824	5.2798	0.7478	0.5009	0.2405
32	Co	3.4703	5.2019	5.2792	0.4992	0.7483	0.2405
33	O	3.6564	3.6584	7.7258	0.5260	0.5263	0.3519
34	O	1.5519	1.5559	7.7292	0.2232	0.2238	0.3521
35	C	3.6659	3.6715	6.5227	0.5273	0.5281	0.2971
36	C	1.5347	1.5423	6.5266	0.2208	0.2219	0.2973

/db/jmorales/CoNi-alloy/Adsorptions/CO-Ni(001)

a =	6.950991869
b =	6.950991869
c =	21.950991869
alpha =	90.0
beta =	90.0
gamma =	90.0

	Atom	X	Y	Z	X	Y	Z
1	Ni	0.0000	0.0000	0.0000	0.0000	0.0000	0.0000
2	Ni	1.7378	1.7378	0.0000	0.2500	0.2500	0.0000
3	Ni	0.0000	3.4755	0.0000	0.0000	0.5000	0.0000
4	Ni	1.7378	5.2132	0.0000	0.2500	0.7500	0.0000
5	Ni	3.4755	0.0000	0.0000	0.5000	0.0000	0.0000
6	Ni	5.2132	1.7378	0.0000	0.7500	0.2500	0.0000
7	Ni	3.4755	3.4755	0.0000	0.5000	0.5000	0.0000
8	Ni	5.2132	5.2132	0.0000	0.7500	0.7500	0.0000
9	Ni	0.0000	1.7378	1.7378	0.0000	0.2500	0.0792
10	Ni	1.7378	0.0000	1.7378	0.2500	0.0000	0.0792
11	Ni	0.0000	5.2132	1.7378	0.0000	0.7500	0.0792
12	Ni	1.7378	3.4755	1.7378	0.2500	0.5000	0.0792
13	Ni	3.4755	1.7378	1.7378	0.5000	0.2500	0.0792
14	Ni	5.2132	0.0000	1.7378	0.7500	0.0000	0.0792
15	Ni	3.4755	5.2132	1.7378	0.5000	0.7500	0.0792
16	Ni	5.2132	3.4755	1.7378	0.7500	0.5000	0.0792
17	Ni	0.0000	0.0000	3.4755	0.0000	0.0000	0.1583
18	Ni	1.7378	1.7378	3.4755	0.2500	0.2500	0.1583
19	Ni	0.0000	3.4755	3.4755	0.0000	0.5000	0.1583
20	Ni	1.7378	5.2132	3.4755	0.2500	0.7500	0.1583
21	Ni	3.4755	0.0000	3.4755	0.5000	0.0000	0.1583
22	Ni	5.2132	1.7378	3.4755	0.7500	0.2500	0.1583
23	Ni	3.4755	3.4755	3.4755	0.5000	0.5000	0.1583
24	Ni	5.2132	5.2132	3.4755	0.7500	0.7500	0.1583
25	Ni	0.0000	1.7378	5.2132	0.0000	0.2500	0.2375
26	Ni	1.7378	0.0000	5.2132	0.2500	0.0000	0.2375
27	Ni	0.0000	5.2132	5.2132	0.0000	0.7500	0.2375
28	Ni	1.7378	3.4755	5.2132	0.2500	0.5000	0.2375
29	Ni	3.4755	1.7378	5.2132	0.5000	0.2500	0.2375
30	Ni	5.2132	0.0000	5.2132	0.7500	0.0000	0.2375
31	Ni	3.4755	5.2132	5.2132	0.5000	0.7500	0.2375
32	Ni	5.2132	3.4755	5.2132	0.7500	0.5000	0.2375
33	O	3.4000	3.6000	7.6752	0.4891	0.5179	0.3497
34	C	3.4000	3.6000	6.5248	0.4891	0.5179	0.2972

/db/jmorales/CoNi-alloy/Adsorptions/Functionals/PBEsol/Co-CoNi(001)-CO

a =	6.9516692132
b =	6.9516692132
c =	21.9516692133
alpha =	90.0
beta =	90.0
gamma =	90.0

	Atom	X	Y	Z	X	Y	Z
1	Ni	0.0000	0.0000	0.0000	0.0000	0.0000	0.0000
2	Ni	1.7379	1.7379	0.0000	0.2500	0.2500	0.0000
3	Ni	0.0000	3.4758	0.0000	0.0000	0.5000	0.0000
4	Ni	1.7379	5.2138	0.0000	0.2500	0.7500	0.0000
5	Ni	3.4758	0.0000	0.0000	0.5000	0.0000	0.0000
6	Ni	5.2138	1.7379	0.0000	0.7500	0.2500	0.0000
7	Ni	3.4758	3.4758	0.0000	0.5000	0.5000	0.0000
8	Ni	5.2138	5.2138	0.0000	0.7500	0.7500	0.0000
9	Ni	-0.0001	0.0001	3.4807	-0.0000	0.0000	0.1586
10	Ni	1.7339	1.7340	3.4879	0.2494	0.2494	0.1589
11	Ni	-0.0001	3.4759	3.4897	-0.0000	0.5000	0.1590
12	Ni	1.7339	5.2177	3.4881	0.2494	0.7506	0.1589
13	Ni	3.4757	-0.0000	3.4898	0.5000	-0.0000	0.1590
14	Ni	5.2176	1.7341	3.4877	0.7506	0.2494	0.1589
15	Ni	3.4762	3.4756	3.4407	0.5001	0.5000	0.1567
16	Ni	5.2176	5.2177	3.4879	0.7505	0.7506	0.1589
17	Co	1.7379	0.0000	1.7379	0.2500	0.0000	0.0792
18	Co	0.0000	1.7379	1.7379	0.0000	0.2500	0.0792
19	Co	1.7379	3.4758	1.7379	0.2500	0.5000	0.0792
20	Co	0.0000	5.2138	1.7379	0.0000	0.7500	0.0792
21	Co	5.2138	0.0000	1.7379	0.7500	0.0000	0.0792
22	Co	3.4758	1.7379	1.7379	0.5000	0.2500	0.0792
23	Co	5.2138	3.4758	1.7379	0.7500	0.5000	0.0792
24	Co	3.4758	5.2138	1.7379	0.5000	0.7500	0.0792
25	Co	1.7370	0.0001	5.1671	0.2499	0.0000	0.2354
26	Co	-0.0002	1.7376	5.1669	-0.0000	0.2499	0.2354
27	Co	1.7617	3.4759	5.1910	0.2534	0.5000	0.2365
28	Co	-0.0003	5.2142	5.1671	-0.0000	0.7501	0.2354
29	Co	5.2144	0.0002	5.1669	0.7501	0.0000	0.2354
30	Co	3.4757	1.7617	5.1909	0.5000	0.2534	0.2365
31	Co	5.1898	3.4757	5.1908	0.7465	0.5000	0.2365
32	Co	3.4759	5.1901	5.1909	0.5000	0.7466	0.2365
33	O	3.4757	3.4759	7.5211	0.5000	0.5000	0.3426
34	C	3.4758	3.4758	6.3066	0.5000	0.5000	0.2873

/db/jmorales/CoNi-alloy/Adsorptions/Functionals/RPBE/CoNi(001)-CO

a =			6.9516692132			
b =			6.9516692132			
c =			21.9516692133			
alpha =			90.0			
beta =			90.0			
gamma =			90.0			
	Atom	X	Y	Z	X	Y	Z

	Atom	X	Y	Z	X	Y	Z
1	Ni	0.0000	0.0000	0.0000	0.0000	0.0000	0.0000
2	Ni	0.0000	3.4758	0.0000	0.0000	0.5000	0.0000
3	Ni	3.4758	0.0000	0.0000	0.5000	0.0000	0.0000
4	Ni	3.4758	3.4758	0.0000	0.5000	0.5000	0.0000
5	Ni	0.0000	1.7379	1.7379	0.0000	0.2500	0.0792
6	Ni	0.0000	5.2138	1.7379	0.0000	0.7500	0.0792
7	Ni	3.4758	1.7379	1.7379	0.5000	0.2500	0.0792
8	Ni	3.4758	5.2138	1.7379	0.5000	0.7500	0.0792
9	Ni	6.9506	0.0005	3.5083	0.9998	0.0001	0.1598
10	Ni	6.9512	3.4762	3.5010	0.9999	0.5001	0.1595
11	Ni	3.4749	0.0005	3.5334	0.4999	0.0001	0.1610
12	Ni	3.4752	3.4763	3.4586	0.4999	0.5001	0.1576
13	Ni	6.9513	1.7361	5.2369	1.0000	0.2497	0.2386
14	Ni	6.9509	5.2168	5.2367	0.9999	0.7504	0.2386
15	Ni	3.4750	1.7583	5.2713	0.4999	0.2529	0.2401
16	Ni	3.4754	5.1949	5.2712	0.4999	0.7473	0.2401
17	Co	1.7379	1.7379	0.0000	0.2500	0.2500	0.0000
18	Co	1.7379	5.2138	0.0000	0.2500	0.7500	0.0000
19	Co	5.2138	1.7379	0.0000	0.7500	0.2500	0.0000
20	Co	5.2138	5.2138	0.0000	0.7500	0.7500	0.0000
21	Co	1.7379	0.0000	1.7379	0.2500	0.0000	0.0792
22	Co	1.7379	3.4758	1.7379	0.2500	0.5000	0.0792
23	Co	5.2138	0.0000	1.7379	0.7500	0.0000	0.0792
24	Co	5.2138	3.4758	1.7379	0.7500	0.5000	0.0792
25	Co	1.7369	1.7387	3.5649	0.2498	0.2501	0.1624
26	Co	1.7373	5.2136	3.5650	0.2499	0.7500	0.1624
27	Co	5.2137	1.7386	3.5651	0.7500	0.2501	0.1624
28	Co	5.2134	5.2137	3.5652	0.7499	0.7500	0.1624
29	Co	1.7320	0.0006	5.2541	0.2492	0.0001	0.2394
30	Co	1.7403	3.4763	5.2809	0.2503	0.5001	0.2406
31	Co	5.2165	0.0007	5.2543	0.7504	0.0001	0.2394
32	Co	5.2096	3.4762	5.2815	0.7494	0.5001	0.2406
33	O	3.4710	3.4775	7.6441	0.4993	0.5002	0.3482
34	C	3.4735	3.4768	6.4247	0.4997	0.5001	0.2927

/db/jmorales/CoNi-alloy/Adsorptions/Functionals/RPBE/CoNi(111)-CO

a = 9.831199646

b = 9.831199646075708

c = 21.0202999115

alpha = 90.0

beta = 90.0

gamma = 120.0

	Atom	X	Y	Z	X	Y	Z
1	Ni	0.0000	0.0000	0.0000	0.0000	0.0000	0.0000
2	Ni	-1.2289	2.1285	0.0000	0.0000	0.2500	0.0000
3	Ni	-2.4578	4.2570	0.0000	0.0000	0.5000	0.0000
4	Ni	-3.6867	6.3856	0.0000	0.0000	0.7500	0.0000
5	Ni	4.9156	0.0000	0.0000	0.5000	0.0000	0.0000
6	Ni	3.6867	2.1285	0.0000	0.5000	0.2500	0.0000
7	Ni	2.4578	4.2570	0.0000	0.5000	0.5000	0.0000
8	Ni	1.2289	6.3856	0.0000	0.5000	0.7500	0.0000
9	Ni	2.4578	1.4190	2.0068	0.3333	0.1667	0.0955
10	Ni	1.2289	3.5475	2.0068	0.3333	0.4167	0.0955
11	Ni	0.0000	5.6760	2.0068	0.3333	0.6667	0.0955
12	Ni	-1.2289	7.8045	2.0068	0.3333	0.9167	0.0955
13	Ni	7.3734	1.4190	2.0068	0.8333	0.1667	0.0955
14	Ni	6.1445	3.5475	2.0068	0.8333	0.4167	0.0955
15	Ni	4.9156	5.6760	2.0068	0.8333	0.6667	0.0955
16	Ni	3.6867	7.8045	2.0068	0.8333	0.9167	0.0955
17	Ni	0.0356	2.8593	4.0122	0.1715	0.3358	0.1909
18	Ni	1.2642	0.7282	4.0112	0.1714	0.0855	0.1908
19	Ni	-2.4243	7.1153	4.0242	0.1713	0.8357	0.1914
20	Ni	-1.1949	4.9841	4.0247	0.1712	0.5854	0.1915
21	Ni	4.9453	2.8561	4.0305	0.6708	0.3355	0.1917
22	Ni	6.1754	0.7290	4.0218	0.6710	0.0856	0.1913
23	Ni	2.4916	7.1121	4.0230	0.6711	0.8353	0.1914
24	Ni	3.7162	4.9816	4.0302	0.6706	0.5851	0.1917
25	Ni	0.0575	0.0375	6.0500	0.0081	0.0044	0.2878
26	Ni	-1.1658	2.1594	6.0502	0.0082	0.2536	0.2878
27	Ni	-2.3980	4.2880	6.0539	0.0079	0.5036	0.2880
28	Ni	-3.6264	6.4212	6.0532	0.0082	0.7542	0.2880
29	Ni	4.9762	0.0344	6.0531	0.5082	0.0040	0.2880
30	Ni	3.7591	2.1730	6.1082	0.5100	0.2552	0.2906
31	Ni	2.5307	4.2952	6.1073	0.5097	0.5045	0.2905
32	Ni	1.2913	6.4208	6.0535	0.5084	0.7541	0.2880
33	Co	1.2289	2.1285	0.0000	0.2500	0.2500	0.0000
34	Co	2.4578	0.0000	0.0000	0.2500	0.0000	0.0000
35	Co	-1.2289	6.3856	0.0000	0.2500	0.7500	0.0000
36	Co	0.0000	4.2570	0.0000	0.2500	0.5000	0.0000
37	Co	6.1445	2.1285	0.0000	0.7500	0.2500	0.0000
38	Co	7.3734	0.0000	0.0000	0.7500	0.0000	0.0000
39	Co	3.6867	6.3856	0.0000	0.7500	0.7500	0.0000
40	Co	4.9156	4.2570	0.0000	0.7500	0.5000	0.0000
41	Co	-1.2289	3.5475	2.0068	0.0833	0.4167	0.0955

42	Co	0.0000	1.4190	2.0068	0.0833	0.1667	0.0955
43	Co	-3.6867	7.8045	2.0068	0.0833	0.9167	0.0955
44	Co	-2.4578	5.6760	2.0068	0.0833	0.6667	0.0955
45	Co	3.6867	3.5475	2.0068	0.5833	0.4167	0.0955
46	Co	4.9156	1.4190	2.0068	0.5833	0.1667	0.0955
47	Co	1.2289	7.8045	2.0068	0.5833	0.9167	0.0955
48	Co	2.4578	5.6760	2.0068	0.5833	0.6667	0.0955
49	Co	3.7112	0.7300	4.0687	0.4204	0.0857	0.1936
50	Co	2.4879	2.8544	4.0583	0.4207	0.3353	0.1931
51	Co	1.2583	4.9793	4.0692	0.4204	0.5848	0.1936
52	Co	0.0306	7.1117	4.0671	0.4208	0.8353	0.1935
53	Co	8.6349	0.7287	4.0638	0.9211	0.0856	0.1933
54	Co	7.4024	2.8536	4.0664	0.9205	0.3352	0.1935
55	Co	6.1739	4.9840	4.0657	0.9207	0.5854	0.1934
56	Co	4.9445	7.1104	4.0664	0.9205	0.8351	0.1935
57	Co	1.2925	2.1627	6.0831	0.2585	0.2540	0.2894
58	Co	2.5386	0.0194	6.0412	0.2594	0.0023	0.2874
59	Co	-1.1518	6.4271	6.0465	0.2603	0.7549	0.2877
60	Co	0.0586	4.3145	6.0433	0.2593	0.5068	0.2875
61	Co	6.2259	2.1681	6.0462	0.7606	0.2547	0.2876
62	Co	7.4511	0.0452	6.0504	0.7606	0.0053	0.2878
63	Co	3.7644	6.4339	6.0463	0.7607	0.7557	0.2876
64	Co	5.0140	4.3130	6.0401	0.7633	0.5066	0.2873
65	O	2.4947	2.8567	8.6688	0.4215	0.3355	0.4124
66	C	2.4206	2.8159	7.4731	0.4116	0.3307	0.3555

/db/jmorales/CoNi-alloy/Adsorptions/CO-Sm(subsurf)$Ce_{26}O_{53}$

a =			11.6082000732				
b =			11.608200073110298				
c =			22.898399353				
alpha =			90.0				
beta =			90.0				
gamma =			120.0				
	Atom	X	Y	Z	X	Y	Z
1	Sm	1.9437	5.5928	3.8380	0.4456	0.5563	0.1676
2	Ce	1.9347	1.1170	0.7898	0.2222	0.1111	0.0345
3	Ce	0.0000	4.4680	0.7898	0.2222	0.4444	0.0345
4	Ce	-1.9347	7.8190	0.7898	0.2222	0.7778	0.0345
5	Ce	5.8041	1.1170	0.7898	0.5556	0.1111	0.0345
6	Ce	3.8694	4.4680	0.7898	0.5556	0.4444	0.0345
7	Ce	1.9347	7.8190	0.7898	0.5556	0.7778	0.0345
8	Ce	9.6735	1.1170	0.7898	0.8889	0.1111	0.0345
9	Ce	7.7388	4.4680	0.7898	0.8889	0.4444	0.0345
10	Ce	5.8041	7.8190	0.7898	0.8889	0.7778	0.0345
11	Ce	0.0056	2.2515	3.9577	0.1125	0.2240	0.1728
12	Ce	-1.9121	5.5838	3.9619	0.1130	0.5554	0.1730
13	Ce	-3.8698	8.9361	3.9453	0.1111	0.8889	0.1723
14	Ce	3.8643	2.2488	3.9432	0.4447	0.2237	0.1722
15	Ce	0.0093	8.9186	3.9489	0.4444	0.8872	0.1725
16	Ce	7.7351	2.2331	3.9212	0.7774	0.2221	0.1712
17	Ce	5.7748	5.5875	3.9713	0.7754	0.5558	0.1734
18	Ce	3.8570	8.9091	3.9722	0.7754	0.8862	0.1735
19	Ce	5.8039	10.0518	7.1003	0.9999	0.9999	0.3101
20	Ce	9.6240	3.3217	7.0844	0.9943	0.3304	0.3094
21	Ce	7.7340	6.7036	7.0992	0.9997	0.6668	0.3100
22	Ce	-1.9413	10.0518	7.0809	0.3327	0.9999	0.3092
23	Ce	1.9683	3.1909	7.1492	0.3283	0.3174	0.3122
24	Ce	-0.1238	6.8062	7.1753	0.3278	0.6770	0.3134
25	Ce	7.7276	0.0206	7.0863	0.6667	0.0021	0.3095
26	Ce	5.8146	3.3327	7.0893	0.6667	0.3315	0.3096
27	Ce	3.9937	6.7726	7.2338	0.6809	0.6737	0.3159
28	O	0.0000	0.0000	0.0000	0.0000	0.0000	0.0000
29	O	-1.9347	3.3510	0.0000	-0.0000	0.3333	0.0000
30	O	-3.8694	6.7020	0.0000	-0.0000	0.6667	0.0000
31	O	3.8694	0.0000	0.0000	0.3333	0.0000	0.0000
32	O	1.9347	3.3510	0.0000	0.3333	0.3333	0.0000
33	O	0.0000	6.7020	0.0000	0.3333	0.6667	0.0000
34	O	7.7388	0.0000	0.0000	0.6667	0.0000	0.0000
35	O	5.8041	3.3510	0.0000	0.6667	0.3333	0.0000
36	O	3.8694	6.7020	0.0000	0.6667	0.6667	0.0000
37	O	-0.0000	2.2340	1.5798	0.1111	0.2222	0.0690
38	O	-1.9347	5.5850	1.5798	0.1111	0.5556	0.0690
39	O	-3.8694	8.9360	1.5798	0.1111	0.8889	0.0690
40	O	3.8694	2.2340	1.5798	0.4444	0.2222	0.0690

41	O	1.9347	5.5850	1.5798	0.4444	0.5556	0.0690
42	O	-0.0000	8.9360	1.5798	0.4444	0.8889	0.0690
43	O	7.7388	2.2340	1.5798	0.7778	0.2222	0.0690
44	O	5.8041	5.5850	1.5798	0.7778	0.5556	0.0690
45	O	3.8694	8.9360	1.5798	0.7778	0.8889	0.0690
46	O	1.9347	1.1170	3.1593	0.2222	0.1111	0.1380
47	O	0.0000	4.4680	3.1593	0.2222	0.4444	0.1380
48	O	-1.9347	7.8190	3.1593	0.2222	0.7778	0.1380
49	O	5.8041	1.1170	3.1593	0.5556	0.1111	0.1380
50	O	3.8694	4.4680	3.1593	0.5556	0.4444	0.1380
51	O	1.9347	7.8190	3.1593	0.5556	0.7778	0.1380
52	O	9.6735	1.1170	3.1593	0.8889	0.1111	0.1380
53	O	7.7388	4.4680	3.1593	0.8889	0.4444	0.1380
54	O	5.8041	7.8190	3.1593	0.8889	0.7778	0.1380
55	O	0.0062	0.0082	4.7534	0.0009	0.0008	0.2076
56	O	-1.9297	3.3554	4.7727	0.0006	0.3338	0.2084
57	O	-3.8567	6.7038	4.7568	0.0012	0.6668	0.2077
58	O	3.8641	-0.0043	4.7278	0.3327	-0.0004	0.2065
59	O	1.9404	3.4165	4.8911	0.3371	0.3399	0.2136
60	O	0.0546	6.6744	4.8897	0.3367	0.6639	0.2135
61	O	1.9225	10.0438	4.7658	0.6652	0.9991	0.2081
62	O	5.7907	3.3465	4.7654	0.6653	0.3329	0.2081
63	O	3.8312	6.6799	4.8637	0.6623	0.6645	0.2124
64	O	-0.0278	2.1989	6.3439	0.1070	0.2187	0.2770
65	O	-1.9753	5.5769	6.3562	0.1072	0.5547	0.2776
66	O	-3.8582	8.9484	6.2792	0.1127	0.8901	0.2742
67	O	3.8959	2.1541	6.2956	0.4428	0.2143	0.2749
68	O	-0.0560	8.9955	6.3042	0.4426	0.8948	0.2753
69	O	7.7389	2.2346	6.2708	0.7778	0.2223	0.2739
70	O	5.9022	5.5494	6.3358	0.7845	0.5520	0.2767
71	O	3.8881	9.0409	6.3358	0.7846	0.8993	0.2767
72	O	1.9409	1.0785	7.9513	0.2208	0.1073	0.3472
73	O	0.3066	4.6331	7.6747	0.2568	0.4609	0.3352
74	O	-1.9854	7.8574	7.9479	0.2198	0.7816	0.3471
75	O	5.8366	1.1365	7.9147	0.5593	0.1131	0.3456
76	O	3.7521	4.4030	7.7686	0.5422	0.4380	0.3393
77	O	1.8290	7.7548	7.7709	0.5433	0.7714	0.3394
78	O	9.6222	1.1787	7.9390	0.8875	0.1173	0.3467
79	O	7.7618	4.3919	7.9399	0.8871	0.4369	0.3467
80	O	5.9314	7.8927	7.9686	0.9035	0.7851	0.3480
81	O	-0.0030	7.1311	11.1656	0.3544	0.7094	0.4876
82	C	-0.0503	6.9128	10.0452	0.3395	0.6876	0.4387

/db/jmorales/CoNi-alloy/Adsorptions/Expanded-models/CO_2-Ni-CoNi(001)-expanded

a =	7.044921511
b =	7.044921511
c =	22.044921511
alpha =	90.0
beta =	90.0
gamma =	90.0

	Atom	X	Y	Z	X	Y	Z
1	Co	0.0000	0.0000	0.0000	0.0000	0.0000	0.0000
2	Co	1.7612	1.7612	0.0000	0.2500	0.2500	0.0000
3	Co	0.0022	0.0005	3.5279	0.0003	0.0001	0.1600
4	Co	1.7711	1.7640	3.5413	0.2514	0.2504	0.1606
5	Co	0.0000	3.5225	0.0000	0.0000	0.5000	0.0000
6	Co	1.7612	5.2837	0.0000	0.2500	0.7500	0.0000
7	Co	7.0256	3.5231	3.5486	0.9973	0.5001	0.1610
8	Co	1.7619	5.2851	3.5304	0.2501	0.7502	0.1601
9	Co	3.5225	0.0000	0.0000	0.5000	0.0000	0.0000
10	Co	5.2837	1.7612	0.0000	0.7500	0.2500	0.0000
11	Co	3.5238	0.0239	3.5460	0.5002	0.0034	0.1609
12	Co	5.2904	1.7564	3.5406	0.7509	0.2493	0.1606
13	Co	3.5225	3.5225	0.0000	0.5000	0.5000	0.0000
14	Co	5.2837	5.2837	0.0000	0.7500	0.7500	0.0000
15	Co	3.5275	3.5196	3.5084	0.5007	0.4996	0.1591
16	Co	5.2844	5.2756	3.5405	0.7501	0.7489	0.1606
17	Ni	0.0000	1.7612	1.7612	0.0000	0.2500	0.0799
18	Ni	1.7612	0.0000	1.7612	0.2500	0.0000	0.0799
19	Ni	0.0231	1.7491	5.1760	0.0033	0.2483	0.2348
20	Ni	1.7485	7.0445	5.1754	0.2482	0.9999	0.2348
21	Ni	0.0000	5.2837	1.7612	0.0000	0.7500	0.0799
22	Ni	1.7612	3.5225	1.7612	0.2500	0.5000	0.0799
23	Ni	0.0047	5.2976	5.1766	0.0007	0.7520	0.2348
24	Ni	1.7892	3.5380	5.2132	0.2540	0.5022	0.2365
25	Ni	3.5225	1.7612	1.7612	0.5000	0.2500	0.0799
26	Ni	5.2837	0.0000	1.7612	0.7500	0.0000	0.0799
27	Ni	3.5264	1.7404	5.2375	0.5006	0.2471	0.2376
28	Ni	5.3010	7.0245	5.1746	0.7524	0.9971	0.2347
29	Ni	3.5225	5.2837	1.7612	0.5000	0.7500	0.0799
30	Ni	5.2837	3.5225	1.7612	0.7500	0.5000	0.0799
31	Ni	3.5137	5.2660	5.2083	0.4988	0.7475	0.2363
32	Ni	5.3060	3.5201	5.2366	0.7532	0.4997	0.2375
33	C	3.1285	3.9493	6.7157	0.4441	0.5606	0.3046
34	O	2.6252	4.5222	7.6778	0.3726	0.6419	0.3483
35	O	4.0591	2.9774	6.7523	0.5762	0.4226	0.3063

/db/jmorales/CoNi-alloy/Adsorptions/CO-CoNi(001)

a = 6.951669213
b = 6.951669213
c = 21.951669213
alpha = 90.0
beta = 90.0
gamma = 90.0

	Atom	X	Y	Z	X	Y	Z
1	Ni	0.0000	0.0000	0.0000	0.0000	0.0000	0.0000
2	Ni	0.0000	3.4758	0.0000	0.0000	0.5000	0.0000
3	Ni	3.4758	0.0000	0.0000	0.5000	0.0000	0.0000
4	Ni	3.4758	3.4758	0.0000	0.5000	0.5000	0.0000
5	Ni	0.0000	1.7379	1.7379	0.0000	0.2500	0.0792
6	Ni	0.0000	5.2138	1.7379	0.0000	0.7500	0.0792
7	Ni	3.4758	1.7379	1.7379	0.5000	0.2500	0.0792
8	Ni	3.4758	5.2138	1.7379	0.5000	0.7500	0.0792
9	Ni	0.0000	0.0000	3.4758	0.0000	0.0000	0.1583
10	Ni	0.0000	3.4758	3.4758	0.0000	0.5000	0.1583
11	Ni	3.4758	0.0000	3.4758	0.5000	0.0000	0.1583
12	Ni	3.4758	3.4758	3.4758	0.5000	0.5000	0.1583
13	Ni	0.0000	1.7379	5.2138	0.0000	0.2500	0.2375
14	Ni	0.0000	5.2138	5.2138	0.0000	0.7500	0.2375
15	Ni	3.4758	1.7379	5.2138	0.5000	0.2500	0.2375
16	Ni	3.4758	5.2138	5.2138	0.5000	0.7500	0.2375
17	Co	1.7379	1.7379	0.0000	0.2500	0.2500	0.0000
18	Co	1.7379	5.2138	0.0000	0.2500	0.7500	0.0000
19	Co	5.2138	1.7379	0.0000	0.7500	0.2500	0.0000
20	Co	5.2138	5.2138	0.0000	0.7500	0.7500	0.0000
21	Co	1.7379	0.0000	1.7379	0.2500	0.0000	0.0792
22	Co	1.7379	3.4758	1.7379	0.2500	0.5000	0.0792
23	Co	5.2138	0.0000	1.7379	0.7500	0.0000	0.0792
24	Co	5.2138	3.4758	1.7379	0.7500	0.5000	0.0792
25	Co	1.7379	1.7379	3.4758	0.2500	0.2500	0.1583
26	Co	1.7379	5.2138	3.4758	0.2500	0.7500	0.1583
27	Co	5.2138	1.7379	3.4758	0.7500	0.2500	0.1583
28	Co	5.2138	5.2138	3.4758	0.7500	0.7500	0.1583
29	Co	1.7379	0.0000	5.2138	0.2500	0.0000	0.2375
30	Co	1.7379	3.4758	5.2138	0.2500	0.5000	0.2375
31	Co	5.2138	0.0000	5.2138	0.7500	0.0000	0.2375
32	Co	5.2138	3.4758	5.2138	0.7500	0.5000	0.2375
33	O	3.4000	3.5000	7.4752	0.4891	0.5035	0.3405
34	C	3.4000	3.5000	6.3248	0.4891	0.5035	0.2881

/db/jmorales/CoNi-alloy/Adsorptions/Expanded-models/CO-CoNi(111)-expanded

a =	9.963000298
b =	9.963000298017379
c =	21.101100922
alpha =	90.0
beta =	90.0
gamma =	120.0

	Atom	X	Y	Z	X	Y	Z
1	Ni	0.0000	0.0000	0.0000	0.0000	0.0000	0.0000
2	Ni	-1.2454	2.1570	0.0000	-0.0000	0.2500	0.0000
3	Ni	-2.4908	4.3141	0.0000	0.0000	0.5000	0.0000
4	Ni	-3.7361	6.4712	0.0000	-0.0000	0.7500	0.0000
5	Ni	4.9815	0.0000	0.0000	0.5000	0.0000	0.0000
6	Ni	3.7361	2.1570	0.0000	0.5000	0.2500	0.0000
7	Ni	2.4908	4.3141	0.0000	0.5000	0.5000	0.0000
8	Ni	1.2454	6.4712	0.0000	0.5000	0.7500	0.0000
9	Ni	2.4908	1.4380	2.0337	0.3333	0.1667	0.0964
10	Ni	1.2454	3.5951	2.0337	0.3333	0.4167	0.0964
11	Ni	0.0000	5.7521	2.0337	0.3333	0.6667	0.0964
12	Ni	-1.2454	7.9092	2.0337	0.3333	0.9167	0.0964
13	Ni	7.4723	1.4380	2.0337	0.8333	0.1667	0.0964
14	Ni	6.2269	3.5951	2.0337	0.8333	0.4167	0.0964
15	Ni	4.9815	5.7521	2.0337	0.8333	0.6667	0.0964
16	Ni	3.7362	7.9092	2.0337	0.8333	0.9167	0.0964
17	Ni	-0.0000	2.8761	4.0674	0.1667	0.3333	0.1928
18	Ni	1.2454	0.7190	4.0674	0.1667	0.0833	0.1928
19	Ni	-2.4908	7.1902	4.0674	0.1667	0.8333	0.1928
20	Ni	-1.2454	5.0331	4.0674	0.1667	0.5833	0.1928
21	Ni	4.9815	2.8761	4.0674	0.6667	0.3333	0.1928
22	Ni	6.2268	0.7190	4.0674	0.6667	0.0833	0.1928
23	Ni	2.4907	7.1902	4.0674	0.6667	0.8333	0.1928
24	Ni	3.7361	5.0331	4.0674	0.6667	0.5833	0.1928
25	Ni	0.0000	0.0000	6.1012	0.0000	0.0000	0.2891
26	Ni	-1.2454	2.1570	6.1012	-0.0000	0.2500	0.2891
27	Ni	-2.4908	4.3141	6.1012	0.0000	0.5000	0.2891
28	Ni	-3.7361	6.4712	6.1012	-0.0000	0.7500	0.2891
29	Ni	4.9815	0.0000	6.1012	0.5000	0.0000	0.2891
30	Ni	3.7361	2.1570	6.1012	0.5000	0.2500	0.2891
31	Ni	2.4908	4.3141	6.1012	0.5000	0.5000	0.2891
32	Ni	1.2454	6.4712	6.1012	0.5000	0.7500	0.2891
33	Co	1.2454	2.1570	0.0000	0.2500	0.2500	0.0000
34	Co	2.4908	0.0000	0.0000	0.2500	0.0000	0.0000
35	Co	-1.2454	6.4712	0.0000	0.2500	0.7500	0.0000
36	Co	0.0000	4.3141	0.0000	0.2500	0.5000	0.0000
37	Co	6.2269	2.1570	0.0000	0.7500	0.2500	0.0000
38	Co	7.4722	0.0000	0.0000	0.7500	0.0000	0.0000
39	Co	3.7361	6.4712	0.0000	0.7500	0.7500	0.0000
40	Co	4.9815	4.3141	0.0000	0.7500	0.5000	0.0000
41	Co	-1.2454	3.5951	2.0337	0.0833	0.4167	0.0964

42	Co	0.0000	1.4380	2.0337	0.0833	0.1667	0.0964
43	Co	-3.7361	7.9092	2.0337	0.0833	0.9167	0.0964
44	Co	-2.4907	5.7521	2.0337	0.0833	0.6667	0.0964
45	Co	3.7362	3.5951	2.0337	0.5833	0.4167	0.0964
46	Co	4.9815	1.4380	2.0337	0.5833	0.1667	0.0964
47	Co	1.2454	7.9092	2.0337	0.5833	0.9167	0.0964
48	Co	2.4908	5.7521	2.0337	0.5833	0.6667	0.0964
49	Co	3.7361	0.7190	4.0674	0.4167	0.0833	0.1928
50	Co	2.4907	2.8761	4.0674	0.4167	0.3333	0.1928
51	Co	1.2454	5.0331	4.0674	0.4167	0.5833	0.1928
52	Co	-0.0000	7.1902	4.0674	0.4167	0.8333	0.1928
53	Co	8.7176	0.7190	4.0674	0.9167	0.0833	0.1928
54	Co	7.4722	2.8761	4.0674	0.9167	0.3333	0.1928
55	Co	6.2268	5.0331	4.0674	0.9167	0.5833	0.1928
56	Co	4.9815	7.1902	4.0674	0.9167	0.8333	0.1928
57	Co	1.2454	2.1570	6.1012	0.2500	0.2500	0.2891
58	Co	2.4908	0.0000	6.1012	0.2500	0.0000	0.2891
59	Co	-1.2454	6.4712	6.1012	0.2500	0.7500	0.2891
60	Co	0.0000	4.3141	6.1012	0.2500	0.5000	0.2891
61	Co	6.2269	2.1570	6.1012	0.7500	0.2500	0.2891
62	Co	7.4722	0.0000	6.1012	0.7500	0.0000	0.2891
63	Co	3.7361	6.4712	6.1012	0.7500	0.7500	0.2891
64	Co	4.9815	4.3141	6.1012	0.7500	0.5000	0.2891
65	O	1.2789	3.5844	8.6269	0.3361	0.4154	0.4088
66	C	1.2373	3.5606	7.4323	0.3305	0.4127	0.3522

/db/jmorales/CoNi-alloy/Adsorptions/Functionals/PBEsol/CoNi(001)-CO

a =			6.9516692132				
b =			6.9516692132				
c =			21.9516692133				
alpha =			90.0				
beta =			90.0				
gamma =			90.0				
	Atom	X	Y	Z	X	Y	Z
1	Ni	0.0000	0.0000	0.0000	0.0000	0.0000	0.0000
2	Ni	0.0000	3.4758	0.0000	0.0000	0.5000	0.0000
3	Ni	3.4758	0.0000	0.0000	0.5000	0.0000	0.0000
4	Ni	3.4758	3.4758	0.0000	0.5000	0.5000	0.0000
5	Ni	0.0000	1.7379	1.7379	0.0000	0.2500	0.0792
6	Ni	0.0000	5.2138	1.7379	0.0000	0.7500	0.0792
7	Ni	3.4758	1.7379	1.7379	0.5000	0.2500	0.0792
8	Ni	3.4758	5.2138	1.7379	0.5000	0.7500	0.0792
9	Ni	-0.0010	0.0002	3.4500	-0.0001	0.0000	0.1572
10	Ni	6.9513	3.4761	3.4405	0.9999	0.5000	0.1567
11	Ni	3.4748	0.0003	3.4683	0.4999	0.0000	0.1580
12	Ni	3.4757	3.4759	3.4011	0.5000	0.5000	0.1549
13	Ni	6.9514	1.7401	5.0912	1.0000	0.2503	0.2319
14	Ni	6.9513	5.2121	5.0912	0.9999	0.7498	0.2319
15	Ni	3.4751	1.7613	5.1209	0.4999	0.2534	0.2333
16	Ni	3.4753	5.1911	5.1207	0.4999	0.7467	0.2333
17	Co	1.7379	1.7379	0.0000	0.2500	0.2500	0.0000
18	Co	1.7379	5.2138	0.0000	0.2500	0.7500	0.0000
19	Co	5.2138	1.7379	0.0000	0.7500	0.2500	0.0000
20	Co	5.2138	5.2138	0.0000	0.7500	0.7500	0.0000
21	Co	1.7379	0.0000	1.7379	0.2500	0.0000	0.0792
22	Co	1.7379	3.4758	1.7379	0.2500	0.5000	0.0792
23	Co	5.2138	0.0000	1.7379	0.7500	0.0000	0.0792
24	Co	5.2138	3.4758	1.7379	0.7500	0.5000	0.0792
25	Co	1.7335	1.7357	3.4932	0.2494	0.2497	0.1591
26	Co	1.7336	5.2167	3.4932	0.2494	0.7504	0.1591
27	Co	5.2169	1.7355	3.4933	0.7504	0.2497	0.1591
28	Co	5.2168	5.2169	3.4933	0.7504	0.7505	0.1591
29	Co	1.7282	0.0003	5.1015	0.2486	0.0000	0.2324
30	Co	1.7506	3.4762	5.1320	0.2518	0.5001	0.2338
31	Co	5.2213	0.0005	5.1014	0.7511	0.0001	0.2324
32	Co	5.1995	3.4759	5.1316	0.7480	0.5000	0.2338
33	O	3.4717	3.4767	7.4079	0.4994	0.5001	0.3375
34	C	3.4751	3.4763	6.1915	0.4999	0.5001	0.2821

/db/jmorales/CoNi-alloy/Adsorptions/Functionals/RPBE/Ni-CoNi(001)

a =	6.9516692132	
b =	6.9516692132	
c =	21.9516692133	
alpha =	90.0	
beta =	90.0	
gamma =	90.0	

	Atom	X	Y	Z	X	Y	Z
1	Co	0.0000	0.0000	0.0000	0.0000	0.0000	0.0000
2	Co	1.7379	1.7379	0.0000	0.2500	0.2500	0.0000
3	Co	0.0000	3.4758	0.0000	0.0000	0.5000	0.0000
4	Co	1.7379	5.2138	0.0000	0.2500	0.7500	0.0000
5	Co	3.4758	0.0000	0.0000	0.5000	0.0000	0.0000
6	Co	5.2138	1.7379	0.0000	0.7500	0.2500	0.0000
7	Co	3.4758	3.4758	0.0000	0.5000	0.5000	0.0000
8	Co	5.2138	5.2138	0.0000	0.7500	0.7500	0.0000
9	Co	0.0000	0.0000	3.5511	0.0000	0.0000	0.1618
10	Co	1.7379	1.7379	3.5504	0.2500	0.2500	0.1617
11	Co	0.0000	3.4758	3.5511	0.0000	0.5000	0.1618
12	Co	1.7379	5.2138	3.5504	0.2500	0.7500	0.1617
13	Co	3.4758	0.0000	3.5511	0.5000	0.0000	0.1618
14	Co	5.2138	1.7379	3.5504	0.7500	0.2500	0.1617
15	Co	3.4758	3.4758	3.5511	0.5000	0.5000	0.1618
16	Co	5.2138	5.2138	3.5504	0.7500	0.7500	0.1617
17	Ni	1.7379	0.0000	1.7379	0.2500	0.0000	0.0792
18	Ni	0.0000	1.7379	1.7379	0.0000	0.2500	0.0792
19	Ni	1.7379	3.4758	1.7379	0.2500	0.5000	0.0792
20	Ni	0.0000	5.2138	1.7379	0.0000	0.7500	0.0792
21	Ni	5.2138	0.0000	1.7379	0.7500	0.0000	0.0792
22	Ni	3.4758	1.7379	1.7379	0.5000	0.2500	0.0792
23	Ni	5.2138	3.4758	1.7379	0.7500	0.5000	0.0792
24	Ni	3.4758	5.2138	1.7379	0.5000	0.7500	0.0792
25	Ni	1.7379	0.0000	5.2476	0.2500	0.0000	0.2391
26	Ni	0.0000	1.7379	5.2476	0.0000	0.2500	0.2391
27	Ni	1.7379	3.4758	5.2476	0.2500	0.5000	0.2391
28	Ni	0.0000	5.2138	5.2476	0.0000	0.7500	0.2391
29	Ni	5.2138	0.0000	5.2476	0.7500	0.0000	0.2391
30	Ni	3.4758	1.7379	5.2476	0.5000	0.2500	0.2391
31	Ni	5.2138	3.4758	5.2476	0.7500	0.5000	0.2391
32	Ni	3.4758	5.2138	5.2476	0.5000	0.7500	0.2391

/db/jmorales/CoNi-alloy/Adsorptions/CO_2-Ni(111)

a =				9.829999924			
b =				9.829999923668225			
c =				21.019699097			
alpha =				90.0			
beta =				90.0			
gamma =				120.0			
	Atom	X	Y	Z	X	Y	Z
1	Ni	0.0000	0.0000	0.0000	0.0000	0.0000	0.0000
2	Ni	-1.2288	2.1283	0.0000	0.0000	0.2500	0.0000
3	Ni	-2.4575	4.2565	0.0000	-0.0000	0.5000	0.0000
4	Ni	-3.6862	6.3848	0.0000	-0.0000	0.7500	0.0000
5	Ni	2.4575	0.0000	0.0000	0.2500	0.0000	0.0000
6	Ni	1.2288	2.1283	0.0000	0.2500	0.2500	0.0000
7	Ni	-0.0000	4.2565	0.0000	0.2500	0.5000	0.0000
8	Ni	-1.2288	6.3848	0.0000	0.2500	0.7500	0.0000
9	Ni	4.9150	0.0000	0.0000	0.5000	0.0000	0.0000
10	Ni	3.6862	2.1283	0.0000	0.5000	0.2500	0.0000
11	Ni	2.4575	4.2565	0.0000	0.5000	0.5000	0.0000
12	Ni	1.2288	6.3848	0.0000	0.5000	0.7500	0.0000
13	Ni	7.3725	0.0000	0.0000	0.7500	0.0000	0.0000
14	Ni	6.1438	2.1283	0.0000	0.7500	0.2500	0.0000
15	Ni	4.9150	4.2565	0.0000	0.7500	0.5000	0.0000
16	Ni	3.6862	6.3848	0.0000	0.7500	0.7500	0.0000
17	Ni	-0.0000	1.4188	2.0065	0.0833	0.1667	0.0955
18	Ni	-1.2288	3.5471	2.0065	0.0833	0.4167	0.0955
19	Ni	-2.4575	5.6754	2.0065	0.0833	0.6667	0.0955
20	Ni	-3.6863	7.8036	2.0065	0.0833	0.9167	0.0955
21	Ni	2.4575	1.4188	2.0065	0.3333	0.1667	0.0955
22	Ni	1.2287	3.5471	2.0065	0.3333	0.4167	0.0955
23	Ni	-0.0000	5.6754	2.0065	0.3333	0.6667	0.0955
24	Ni	-1.2288	7.8036	2.0065	0.3333	0.9167	0.0955
25	Ni	4.9150	1.4188	2.0065	0.5833	0.1667	0.0955
26	Ni	3.6862	3.5471	2.0065	0.5833	0.4167	0.0955
27	Ni	2.4575	5.6754	2.0065	0.5833	0.6667	0.0955
28	Ni	1.2287	7.8036	2.0065	0.5833	0.9167	0.0955
29	Ni	7.3725	1.4188	2.0065	0.8333	0.1667	0.0955
30	Ni	6.1437	3.5471	2.0065	0.8333	0.4167	0.0955
31	Ni	4.9150	5.6754	2.0065	0.8333	0.6667	0.0955
32	Ni	3.6862	7.8036	2.0065	0.8333	0.9167	0.0955
33	Ni	1.2288	0.7094	4.0131	0.1667	0.0833	0.1909
34	Ni	0.0000	2.8377	4.0131	0.1667	0.3333	0.1909
35	Ni	-1.2287	4.9659	4.0131	0.1667	0.5833	0.1909
36	Ni	-2.4575	7.0942	4.0131	0.1667	0.8333	0.1909
37	Ni	3.6863	0.7094	4.0131	0.4167	0.0833	0.1909
38	Ni	2.4575	2.8377	4.0131	0.4167	0.3333	0.1909
39	Ni	1.2288	4.9659	4.0131	0.4167	0.5833	0.1909
40	Ni	0.0000	7.0942	4.0131	0.4167	0.8333	0.1909

41	Ni	6.1438	0.7094	4.0131	0.6667	0.0833	0.1909
42	Ni	4.9150	2.8377	4.0131	0.6667	0.3333	0.1909
43	Ni	3.6863	4.9659	4.0131	0.6667	0.5833	0.1909
44	Ni	2.4575	7.0942	4.0131	0.6667	0.8333	0.1909
45	Ni	8.6013	0.7094	4.0131	0.9167	0.0833	0.1909
46	Ni	7.3725	2.8377	4.0131	0.9167	0.3333	0.1909
47	Ni	6.1438	4.9659	4.0131	0.9167	0.5833	0.1909
48	Ni	4.9150	7.0942	4.0131	0.9167	0.8333	0.1909
49	Ni	0.0000	0.0000	6.0198	0.0000	0.0000	0.2864
50	Ni	-1.2288	2.1283	6.0198	0.0000	0.2500	0.2864
51	Ni	-2.4575	4.2565	6.0198	-0.0000	0.5000	0.2864
52	Ni	-3.6862	6.3848	6.0198	-0.0000	0.7500	0.2864
53	Ni	2.4575	0.0000	6.0198	0.2500	0.0000	0.2864
54	Ni	1.2288	2.1283	6.0198	0.2500	0.2500	0.2864
55	Ni	-0.0000	4.2565	6.0198	0.2500	0.5000	0.2864
56	Ni	-1.2288	6.3848	6.0198	0.2500	0.7500	0.2864
57	Ni	4.9150	0.0000	6.0198	0.5000	0.0000	0.2864
58	Ni	3.6862	2.1283	6.0198	0.5000	0.2500	0.2864
59	Ni	2.4575	4.2565	6.0198	0.5000	0.5000	0.2864
60	Ni	1.2288	6.3848	6.0198	0.5000	0.7500	0.2864
61	Ni	7.3725	0.0000	6.0198	0.7500	0.0000	0.2864
62	Ni	6.1438	2.1283	6.0198	0.7500	0.2500	0.2864
63	Ni	4.9150	4.2565	6.0198	0.7500	0.5000	0.2864
64	Ni	3.6862	6.3848	6.0198	0.7500	0.7500	0.2864
65	C	2.0000	3.5000	7.9000	0.4090	0.4111	0.3758
66	O	2.5455	4.1120	8.8000	0.5005	0.4830	0.4187
67	O	1.2424	2.5971	7.9000	0.2789	0.3051	0.3758

/db/jmorales/CoNi-alloy/Adsorptions/CO-Sm(subsurf)$Ce_{26}O_{54}$

a = 11.6082000732
b = 11.608200073110298
c = 22.898399353
alpha = 90.0
beta = 90.0
gamma = 120.0

	Atom	X	Y	Z	X	Y	Z
1	Sm	1.9358	5.5842	3.9659	0.4445	0.5555	0.1732
2	Ce	1.9347	1.1170	0.7898	0.2222	0.1111	0.0345
3	Ce	0.0000	4.4680	0.7898	0.2222	0.4444	0.0345
4	Ce	-1.9347	7.8190	0.7898	0.2222	0.7778	0.0345
5	Ce	5.8041	1.1170	0.7898	0.5556	0.1111	0.0345
6	Ce	3.8694	4.4680	0.7898	0.5556	0.4444	0.0345
7	Ce	1.9347	7.8190	0.7898	0.5556	0.7778	0.0345
8	Ce	9.6735	1.1170	0.7898	0.8889	0.1111	0.0345
9	Ce	7.7388	4.4680	0.7898	0.8889	0.4444	0.0345
10	Ce	5.8041	7.8190	0.7898	0.8889	0.7778	0.0345
11	Ce	0.0073	2.2491	3.9569	0.1125	0.2237	0.1728
12	Ce	-1.9191	5.5825	3.9570	0.1123	0.5553	0.1728
13	Ce	-3.8696	8.9363	3.9576	0.1111	0.8889	0.1728
14	Ce	3.8653	2.2510	3.9614	0.4449	0.2239	0.1730
15	Ce	0.0100	8.9238	3.9570	0.4447	0.8877	0.1728
16	Ce	7.7359	2.2358	3.9582	0.7776	0.2224	0.1729
17	Ce	5.7873	5.5799	3.9618	0.7761	0.5550	0.1730
18	Ce	3.8598	8.9220	3.9569	0.7763	0.8875	0.1728
19	Ce	-0.0029	0.0016	7.1017	-0.0002	0.0002	0.3101
20	Ce	-1.9302	3.3482	7.1139	0.0003	0.3331	0.3107
21	Ce	-3.8687	6.7024	7.1012	0.0001	0.6667	0.3101
22	Ce	3.8695	-0.0009	7.1017	0.3333	-0.0001	0.3101
23	Ce	1.9304	3.3531	7.1032	0.3331	0.3335	0.3102
24	Ce	0.0041	6.6998	7.1011	0.3336	0.6665	0.3101
25	Ce	7.7429	-0.0022	7.1139	0.6669	-0.0002	0.3107
26	Ce	5.8049	3.3485	7.1434	0.6666	0.3331	0.3120
27	Ce	3.8658	6.7042	7.1031	0.6665	0.6669	0.3102
28	O	0.0000	0.0000	0.0000	0.0000	0.0000	0.0000
29	O	-1.9347	3.3510	0.0000	-0.0000	0.3333	0.0000
30	O	-3.8694	6.7020	0.0000	-0.0000	0.6667	0.0000
31	O	3.8694	0.0000	0.0000	0.3333	0.0000	0.0000
32	O	1.9347	3.3510	0.0000	0.3333	0.3333	0.0000
33	O	0.0000	6.7020	0.0000	0.3333	0.6667	0.0000
34	O	7.7388	0.0000	0.0000	0.6667	0.0000	0.0000
35	O	5.8041	3.3510	0.0000	0.6667	0.3333	0.0000
36	O	3.8694	6.7020	0.0000	0.6667	0.6667	0.0000
37	O	-0.0000	2.2340	1.5798	0.1111	0.2222	0.0690
38	O	-1.9347	5.5850	1.5798	0.1111	0.5556	0.0690
39	O	-3.8694	8.9360	1.5798	0.1111	0.8889	0.0690
40	O	3.8694	2.2340	1.5798	0.4444	0.2222	0.0690

41	O	1.9347	5.5850	1.5798	0.4444	0.5556	0.0690
42	O	-0.0000	8.9360	1.5798	0.4444	0.8889	0.0690
43	O	7.7388	2.2340	1.5798	0.7778	0.2222	0.0690
44	O	5.8041	5.5850	1.5798	0.7778	0.5556	0.0690
45	O	3.8694	8.9360	1.5798	0.7778	0.8889	0.0690
46	O	1.9347	1.1170	3.1593	0.2222	0.1111	0.1380
47	O	0.0000	4.4680	3.1593	0.2222	0.4444	0.1380
48	O	-1.9347	7.8190	3.1593	0.2222	0.7778	0.1380
49	O	5.8041	1.1170	3.1593	0.5556	0.1111	0.1380
50	O	3.8694	4.4680	3.1593	0.5556	0.4444	0.1380
51	O	1.9347	7.8190	3.1593	0.5556	0.7778	0.1380
52	O	9.6735	1.1170	3.1593	0.8889	0.1111	0.1380
53	O	7.7388	4.4680	3.1593	0.8889	0.4444	0.1380
54	O	5.8041	7.8190	3.1593	0.8889	0.7778	0.1380
55	O	0.0016	-0.0010	4.7664	0.0001	-0.0001	0.2082
56	O	-1.9383	3.3480	4.7715	-0.0005	0.3330	0.2084
57	O	-3.8718	6.7027	4.7694	-0.0002	0.6667	0.2083
58	O	3.8676	0.0019	4.7696	0.3333	0.0002	0.2083
59	O	1.9368	3.2997	4.8039	0.3310	0.3282	0.2098
60	O	-0.0424	6.7264	4.8021	0.3309	0.6691	0.2097
61	O	7.7395	0.0050	4.7718	0.6670	0.0005	0.2084
62	O	5.8062	3.3493	4.7665	0.6668	0.3332	0.2082
63	O	3.9151	6.7255	4.8041	0.6718	0.6690	0.2098
64	O	-0.0144	2.2280	6.3160	0.1096	0.2216	0.2758
65	O	-1.9441	5.5751	6.3146	0.1098	0.5546	0.2758
66	O	-3.8698	8.9361	6.3048	0.1111	0.8889	0.2753
67	O	3.8886	2.2335	6.3302	0.4461	0.2222	0.2764
68	O	1.9335	5.5852	6.3741	0.4443	0.5556	0.2784
69	O	0.0036	8.9482	6.3143	0.4454	0.8901	0.2758
70	O	7.7346	2.2372	6.3219	0.7776	0.2225	0.2761
71	O	5.8149	5.5671	6.3300	0.7778	0.5538	0.2764
72	O	3.8675	8.9514	6.3159	0.7784	0.8904	0.2758
73	O	1.9344	1.1130	7.8924	0.2220	0.1107	0.3447
74	O	-0.0174	4.4550	7.9031	0.2201	0.4432	0.3451
75	O	-1.9384	7.8212	7.8915	0.2220	0.7780	0.3446
76	O	5.8059	1.0944	7.9129	0.5546	0.1089	0.3456
77	O	3.8652	4.4716	7.9134	0.5554	0.4448	0.3456
78	O	1.9365	7.8403	7.9031	0.5568	0.7799	0.3451
79	O	9.6713	1.1185	7.9102	0.8888	0.1113	0.3454
80	O	7.7604	4.4792	7.9168	0.8913	0.4456	0.3457
81	O	5.8071	7.8200	7.8927	0.8892	0.7779	0.3447
82	O	5.7940	3.5975	11.1342	0.6781	0.3578	0.4862
83	C	5.8026	3.4689	9.9990	0.6724	0.3451	0.4367

/db/jmorales/CoNi-alloy/Adsorptions/Functionals/PBEsol/Ni(111)

a =	9.8299999236		
b =	9.829999923841429		
c =	21.0196990967		
alpha =	90.0		
beta =	90.0		
gamma =	120.0		

	Atom	X	Y	Z	X	Y	Z
1	Ni	0.0000	0.0000	0.0000	0.0000	0.0000	0.0000
2	Ni	-1.2287	2.1283	0.0000	0.0000	0.2500	0.0000
3	Ni	-2.4575	4.2565	0.0000	0.0000	0.5000	0.0000
4	Ni	-3.6862	6.3848	0.0000	-0.0000	0.7500	0.0000
5	Ni	2.4575	0.0000	0.0000	0.2500	0.0000	0.0000
6	Ni	1.2287	2.1283	0.0000	0.2500	0.2500	0.0000
7	Ni	0.0000	4.2565	0.0000	0.2500	0.5000	0.0000
8	Ni	-1.2287	6.3848	0.0000	0.2500	0.7500	0.0000
9	Ni	4.9150	0.0000	0.0000	0.5000	0.0000	0.0000
10	Ni	3.6862	2.1283	0.0000	0.5000	0.2500	0.0000
11	Ni	2.4575	4.2565	0.0000	0.5000	0.5000	0.0000
12	Ni	1.2287	6.3848	0.0000	0.5000	0.7500	0.0000
13	Ni	7.3725	0.0000	0.0000	0.7500	0.0000	0.0000
14	Ni	6.1437	2.1283	0.0000	0.7500	0.2500	0.0000
15	Ni	4.9150	4.2565	0.0000	0.7500	0.5000	0.0000
16	Ni	3.6862	6.3848	0.0000	0.7500	0.7500	0.0000
17	Ni	-0.0000	1.4188	2.0065	0.0833	0.1667	0.0955
18	Ni	-1.2288	3.5471	2.0065	0.0833	0.4167	0.0955
19	Ni	-2.4575	5.6754	2.0065	0.0833	0.6667	0.0955
20	Ni	-3.6863	7.8036	2.0065	0.0833	0.9167	0.0955
21	Ni	2.4575	1.4188	2.0065	0.3333	0.1667	0.0955
22	Ni	1.2287	3.5471	2.0065	0.3333	0.4167	0.0955
23	Ni	-0.0000	5.6754	2.0065	0.3333	0.6667	0.0955
24	Ni	-1.2288	7.8036	2.0065	0.3333	0.9167	0.0955
25	Ni	4.9150	1.4188	2.0065	0.5833	0.1667	0.0955
26	Ni	3.6862	3.5471	2.0065	0.5833	0.4167	0.0955
27	Ni	2.4575	5.6754	2.0065	0.5833	0.6667	0.0955
28	Ni	1.2287	7.8036	2.0065	0.5833	0.9167	0.0955
29	Ni	7.3725	1.4188	2.0065	0.8333	0.1667	0.0955
30	Ni	6.1437	3.5471	2.0065	0.8333	0.4167	0.0955
31	Ni	4.9150	5.6754	2.0065	0.8333	0.6667	0.0955
32	Ni	3.6862	7.8036	2.0065	0.8333	0.9167	0.0955
33	Ni	1.2288	0.7094	3.9642	0.1667	0.0833	0.1886
34	Ni	0.0000	2.8377	3.9642	0.1667	0.3333	0.1886
35	Ni	-1.2287	4.9659	3.9642	0.1667	0.5833	0.1886
36	Ni	-2.4575	7.0942	3.9642	0.1667	0.8333	0.1886
37	Ni	3.6863	0.7094	3.9642	0.4167	0.0833	0.1886
38	Ni	2.4575	2.8377	3.9642	0.4167	0.3333	0.1886
39	Ni	1.2288	4.9659	3.9642	0.4167	0.5833	0.1886
40	Ni	0.0000	7.0942	3.9642	0.4167	0.8333	0.1886
41	Ni	6.1438	0.7094	3.9642	0.6667	0.0833	0.1886

42	Ni	4.9150	2.8377	3.9642	0.6667	0.3333	0.1886
43	Ni	3.6863	4.9659	3.9642	0.6667	0.5833	0.1886
44	Ni	2.4575	7.0942	3.9642	0.6667	0.8333	0.1886
45	Ni	8.6013	0.7094	3.9642	0.9167	0.0833	0.1886
46	Ni	7.3725	2.8377	3.9642	0.9167	0.3333	0.1886
47	Ni	6.1438	4.9659	3.9642	0.9167	0.5833	0.1886
48	Ni	4.9150	7.0942	3.9642	0.9167	0.8333	0.1886
49	Ni	0.0000	0.0000	5.9249	0.0000	0.0000	0.2819
50	Ni	-1.2287	2.1283	5.9249	0.0000	0.2500	0.2819
51	Ni	-2.4575	4.2565	5.9249	0.0000	0.5000	0.2819
52	Ni	-3.6862	6.3848	5.9249	-0.0000	0.7500	0.2819
53	Ni	2.4575	0.0000	5.9249	0.2500	0.0000	0.2819
54	Ni	1.2287	2.1283	5.9249	0.2500	0.2500	0.2819
55	Ni	0.0000	4.2565	5.9249	0.2500	0.5000	0.2819
56	Ni	-1.2287	6.3848	5.9249	0.2500	0.7500	0.2819
57	Ni	4.9150	0.0000	5.9249	0.5000	0.0000	0.2819
58	Ni	3.6862	2.1283	5.9249	0.5000	0.2500	0.2819
59	Ni	2.4575	4.2565	5.9249	0.5000	0.5000	0.2819
60	Ni	1.2287	6.3848	5.9249	0.5000	0.7500	0.2819
61	Ni	7.3725	0.0000	5.9249	0.7500	0.0000	0.2819
62	Ni	6.1437	2.1283	5.9249	0.7500	0.2500	0.2819
63	Ni	4.9150	4.2565	5.9249	0.7500	0.5000	0.2819
64	Ni	3.6862	6.3848	5.9249	0.7500	0.7500	0.2819

/db/jmorales/CoNi-alloy/Adsorptions/Expanded-models/CO_2-CoNi(111)-expanded

a =		9.963000298					
b =		9.963000298017379					
c =		21.101100922					
alpha =		90.0					
beta =		90.0					
gamma =		120.0					
	Atom	X	Y	Z	X	Y	Z
1	Ni	0.0000	0.0000	0.0000	0.0000	0.0000	0.0000
2	Ni	-1.2454	2.1570	0.0000	-0.0000	0.2500	0.0000
3	Ni	-2.4908	4.3141	0.0000	0.0000	0.5000	0.0000
4	Ni	-3.7361	6.4712	0.0000	-0.0000	0.7500	0.0000
5	Ni	4.9815	0.0000	0.0000	0.5000	0.0000	0.0000
6	Ni	3.7361	2.1570	0.0000	0.5000	0.2500	0.0000
7	Ni	2.4908	4.3141	0.0000	0.5000	0.5000	0.0000
8	Ni	1.2454	6.4712	0.0000	0.5000	0.7500	0.0000
9	Ni	2.4908	1.4380	2.0337	0.3333	0.1667	0.0964
10	Ni	1.2454	3.5951	2.0337	0.3333	0.4167	0.0964
11	Ni	0.0000	5.7521	2.0337	0.3333	0.6667	0.0964
12	Ni	-1.2454	7.9092	2.0337	0.3333	0.9167	0.0964
13	Ni	7.4723	1.4380	2.0337	0.8333	0.1667	0.0964
14	Ni	6.2269	3.5951	2.0337	0.8333	0.4167	0.0964
15	Ni	4.9815	5.7521	2.0337	0.8333	0.6667	0.0964
16	Ni	3.7362	7.9092	2.0337	0.8333	0.9167	0.0964
17	Ni	-0.0000	2.8761	4.0674	0.1667	0.3333	0.1928
18	Ni	1.2454	0.7190	4.0674	0.1667	0.0833	0.1928
19	Ni	-2.4908	7.1902	4.0674	0.1667	0.8333	0.1928
20	Ni	-1.2454	5.0331	4.0674	0.1667	0.5833	0.1928
21	Ni	4.9815	2.8761	4.0674	0.6667	0.3333	0.1928
22	Ni	6.2268	0.7190	4.0674	0.6667	0.0833	0.1928
23	Ni	2.4907	7.1902	4.0674	0.6667	0.8333	0.1928
24	Ni	3.7361	5.0331	4.0674	0.6667	0.5833	0.1928
25	Ni	0.0000	0.0000	6.1012	0.0000	0.0000	0.2891
26	Ni	-1.2454	2.1570	6.1012	-0.0000	0.2500	0.2891
27	Ni	-2.4908	4.3141	6.1012	0.0000	0.5000	0.2891
28	Ni	-3.7361	6.4712	6.1012	-0.0000	0.7500	0.2891
29	Ni	4.9815	0.0000	6.1012	0.5000	0.0000	0.2891
30	Ni	3.7361	2.1570	6.1012	0.5000	0.2500	0.2891
31	Ni	2.4908	4.3141	6.1012	0.5000	0.5000	0.2891
32	Ni	1.2454	6.4712	6.1012	0.5000	0.7500	0.2891
33	Co	1.2454	2.1570	0.0000	0.2500	0.2500	0.0000
34	Co	2.4908	0.0000	0.0000	0.2500	0.0000	0.0000
35	Co	-1.2454	6.4712	0.0000	0.2500	0.7500	0.0000
36	Co	0.0000	4.3141	0.0000	0.2500	0.5000	0.0000
37	Co	6.2269	2.1570	0.0000	0.7500	0.2500	0.0000
38	Co	7.4722	0.0000	0.0000	0.7500	0.0000	0.0000
39	Co	3.7361	6.4712	0.0000	0.7500	0.7500	0.0000
40	Co	4.9815	4.3141	0.0000	0.7500	0.5000	0.0000

41	Co	-1.2454	3.5951	2.0337	0.0833	0.4167	0.0964
42	Co	0.0000	1.4380	2.0337	0.0833	0.1667	0.0964
43	Co	-3.7361	7.9092	2.0337	0.0833	0.9167	0.0964
44	Co	-2.4907	5.7521	2.0337	0.0833	0.6667	0.0964
45	Co	3.7362	3.5951	2.0337	0.5833	0.4167	0.0964
46	Co	4.9815	1.4380	2.0337	0.5833	0.1667	0.0964
47	Co	1.2454	7.9092	2.0337	0.5833	0.9167	0.0964
48	Co	2.4908	5.7521	2.0337	0.5833	0.6667	0.0964
49	Co	3.7361	0.7190	4.0674	0.4167	0.0833	0.1928
50	Co	2.4907	2.8761	4.0674	0.4167	0.3333	0.1928
51	Co	1.2454	5.0331	4.0674	0.4167	0.5833	0.1928
52	Co	-0.0000	7.1902	4.0674	0.4167	0.8333	0.1928
53	Co	8.7176	0.7190	4.0674	0.9167	0.0833	0.1928
54	Co	7.4722	2.8761	4.0674	0.9167	0.3333	0.1928
55	Co	6.2268	5.0331	4.0674	0.9167	0.5833	0.1928
56	Co	4.9815	7.1902	4.0674	0.9167	0.8333	0.1928
57	Co	1.2454	2.1570	6.1012	0.2500	0.2500	0.2891
58	Co	2.4908	0.0000	6.1012	0.2500	0.0000	0.2891
59	Co	-1.2454	6.4712	6.1012	0.2500	0.7500	0.2891
60	Co	0.0000	4.3141	6.1012	0.2500	0.5000	0.2891
61	Co	6.2269	2.1570	6.1012	0.7500	0.2500	0.2891
62	Co	7.4722	0.0000	6.1012	0.7500	0.0000	0.2891
63	Co	3.7361	6.4712	6.1012	0.7500	0.7500	0.2891
64	Co	4.9815	4.3141	6.1012	0.7500	0.5000	0.2891
65	C	2.1246	3.7757	7.9644	0.4321	0.4376	0.3774
66	O	2.6329	4.5174	8.7807	0.5260	0.5236	0.4161
67	O	1.3947	2.7250	8.0173	0.2979	0.3158	0.3799

/db/jmorales/CoNi-alloy/Adsorptions/Expanded-models/CO-CoNi(001)-expanded

a = 7.044921511
b = 7.044921511
c = 22.044921511
alpha = 90.0
beta = 90.0
gamma = 90.0

	Atom	X	Y	Z	X	Y	Z
1	Ni	0.0000	0.0000	0.0000	0.0000	0.0000	0.0000
2	Ni	0.0000	3.5225	0.0000	0.0000	0.5000	0.0000
3	Ni	3.5225	0.0000	0.0000	0.5000	0.0000	0.0000
4	Ni	3.5225	3.5225	0.0000	0.5000	0.5000	0.0000
5	Ni	0.0000	1.7612	1.7612	0.0000	0.2500	0.0799
6	Ni	0.0000	5.2837	1.7612	0.0000	0.7500	0.0799
7	Ni	3.5225	1.7612	1.7612	0.5000	0.2500	0.0799
8	Ni	3.5225	5.2837	1.7612	0.5000	0.7500	0.0799
9	Ni	0.0000	0.0000	3.5225	0.0000	0.0000	0.1598
10	Ni	0.0000	3.5225	3.5225	0.0000	0.5000	0.1598
11	Ni	3.5225	0.0000	3.5225	0.5000	0.0000	0.1598
12	Ni	3.5225	3.5225	3.5225	0.5000	0.5000	0.1598
13	Ni	0.0000	1.7612	5.2837	0.0000	0.2500	0.2397
14	Ni	0.0000	5.2837	5.2837	0.0000	0.7500	0.2397
15	Ni	3.5225	1.7612	5.2837	0.5000	0.2500	0.2397
16	Ni	3.5225	5.2837	5.2837	0.5000	0.7500	0.2397
17	Co	1.7612	1.7612	0.0000	0.2500	0.2500	0.0000
18	Co	1.7612	5.2837	0.0000	0.2500	0.7500	0.0000
19	Co	5.2837	1.7612	0.0000	0.7500	0.2500	0.0000
20	Co	5.2837	5.2837	0.0000	0.7500	0.7500	0.0000
21	Co	1.7612	0.0000	1.7612	0.2500	0.0000	0.0799
22	Co	1.7612	3.5225	1.7612	0.2500	0.5000	0.0799
23	Co	5.2837	0.0000	1.7612	0.7500	0.0000	0.0799
24	Co	5.2837	3.5225	1.7612	0.7500	0.5000	0.0799
25	Co	1.7612	1.7612	3.5225	0.2500	0.2500	0.1598
26	Co	1.7612	5.2837	3.5225	0.2500	0.7500	0.1598
27	Co	5.2837	1.7612	3.5225	0.7500	0.2500	0.1598
28	Co	5.2837	5.2837	3.5225	0.7500	0.7500	0.1598
29	Co	1.7612	0.0000	5.2837	0.2500	0.0000	0.2397
30	Co	1.7612	3.5225	5.2837	0.2500	0.5000	0.2397
31	Co	5.2837	0.0000	5.2837	0.7500	0.0000	0.2397
32	Co	5.2837	3.5225	5.2837	0.7500	0.5000	0.2397
33	O	3.4598	3.4815	7.5730	0.4911	0.4942	0.3435
34	C	3.4727	3.4773	6.3578	0.4929	0.4936	0.2884

/db/jmorales/CoNi-alloy/Adsorptions/Coverage/Ni-CoNi(001)-2CO

a =	6.9516692132
b =	6.9516692132
c =	21.9516692133
alpha =	90.0
beta =	90.0
gamma =	90.0

	Atom	X	Y	Z	X	Y	Z
1	Co	0.0000	0.0000	0.0000	0.0000	0.0000	0.0000
2	Co	1.7379	1.7379	0.0000	0.2500	0.2500	0.0000
3	Co	0.0000	3.4758	0.0000	0.0000	0.5000	0.0000
4	Co	1.7379	5.2138	0.0000	0.2500	0.7500	0.0000
5	Co	3.4758	0.0000	0.0000	0.5000	0.0000	0.0000
6	Co	5.2138	1.7379	0.0000	0.7500	0.2500	0.0000
7	Co	3.4758	3.4758	0.0000	0.5000	0.5000	0.0000
8	Co	5.2138	5.2138	0.0000	0.7500	0.7500	0.0000
9	Co	6.9431	-0.0085	3.5337	0.9988	-0.0012	0.1610
10	Co	1.7432	1.7434	3.5028	0.2508	0.2508	0.1596
11	Co	-0.0001	3.4776	3.5566	-0.0000	0.5003	0.1620
12	Co	1.7345	5.2109	3.5558	0.2495	0.7496	0.1620
13	Co	3.4776	-0.0001	3.5566	0.5003	-0.0000	0.1620
14	Co	5.2109	1.7347	3.5558	0.7496	0.2495	0.1620
15	Co	3.4689	3.4690	3.5047	0.4990	0.4990	0.1597
16	Co	5.2183	5.2184	3.5374	0.7507	0.7507	0.1611
17	Ni	1.7379	0.0000	1.7379	0.2500	0.0000	0.0792
18	Ni	0.0000	1.7379	1.7379	0.0000	0.2500	0.0792
19	Ni	1.7379	3.4758	1.7379	0.2500	0.5000	0.0792
20	Ni	0.0000	5.2138	1.7379	0.0000	0.7500	0.0792
21	Ni	5.2138	0.0000	1.7379	0.7500	0.0000	0.0792
22	Ni	3.4758	1.7379	1.7379	0.5000	0.2500	0.0792
23	Ni	5.2138	3.4758	1.7379	0.7500	0.5000	0.0792
24	Ni	3.4758	5.2138	1.7379	0.5000	0.7500	0.0792
25	Ni	1.7356	0.0260	5.2448	0.2497	0.0037	0.2389
26	Ni	0.0258	1.7357	5.2446	0.0037	0.2497	0.2389
27	Ni	1.7597	3.4507	5.3253	0.2531	0.4964	0.2426
28	Ni	-0.0022	5.2145	5.2152	-0.0003	0.7501	0.2376
29	Ni	5.2146	-0.0022	5.2151	0.7501	-0.0003	0.2376
30	Ni	3.4509	1.7597	5.3253	0.4964	0.2531	0.2426
31	Ni	5.1853	3.4742	5.2479	0.7459	0.4998	0.2391
32	Ni	3.4741	5.1854	5.2479	0.4997	0.7459	0.2391
33	O	3.6377	3.6379	7.6530	0.5233	0.5233	0.3486
34	O	1.5537	1.5539	7.6650	0.2235	0.2235	0.3492
35	C	3.6334	3.6334	6.4518	0.5227	0.5227	0.2939
36	C	1.5495	1.5497	6.4656	0.2229	0.2229	0.2945

/db/jmorales/CoNi-alloy/Adsorptions/CO-CoNi-Ce_3SmO_7

a = 13.9033384264
b = 13.9033384264
c = 21.9516692133
alpha = 90.0
beta = 90.0
gamma = 90.0

	Atom	X	Y	Z	X	Y	Z
1	Ni	0.0000	0.0000	0.0000	0.0000	0.0000	0.0000
2	Ni	0.0000	3.4758	0.0000	0.0000	0.2500	0.0000
3	Ni	3.4758	0.0000	0.0000	0.2500	0.0000	0.0000
4	Ni	3.4758	3.4758	0.0000	0.2500	0.2500	0.0000
5	Ni	0.0000	6.9517	0.0000	0.0000	0.5000	0.0000
6	Ni	0.0000	10.4275	0.0000	0.0000	0.7500	0.0000
7	Ni	3.4758	6.9517	0.0000	0.2500	0.5000	0.0000
8	Ni	3.4758	10.4275	0.0000	0.2500	0.7500	0.0000
9	Ni	6.9517	0.0000	0.0000	0.5000	0.0000	0.0000
10	Ni	6.9517	3.4758	0.0000	0.5000	0.2500	0.0000
11	Ni	10.4275	0.0000	0.0000	0.7500	0.0000	0.0000
12	Ni	10.4275	3.4758	0.0000	0.7500	0.2500	0.0000
13	Ni	6.9517	6.9517	0.0000	0.5000	0.5000	0.0000
14	Ni	6.9517	10.4275	0.0000	0.5000	0.7500	0.0000
15	Ni	10.4275	6.9517	0.0000	0.7500	0.5000	0.0000
16	Ni	10.4275	10.4275	0.0000	0.7500	0.7500	0.0000
17	Ni	0.0000	1.7379	1.7379	0.0000	0.1250	0.0792
18	Ni	0.0000	5.2138	1.7379	0.0000	0.3750	0.0792
19	Ni	3.4758	1.7379	1.7379	0.2500	0.1250	0.0792
20	Ni	3.4758	5.2138	1.7379	0.2500	0.3750	0.0792
21	Ni	0.0000	8.6896	1.7379	0.0000	0.6250	0.0792
22	Ni	0.0000	12.1654	1.7379	0.0000	0.8750	0.0792
23	Ni	3.4758	8.6896	1.7379	0.2500	0.6250	0.0792
24	Ni	3.4758	12.1654	1.7379	0.2500	0.8750	0.0792
25	Ni	6.9517	1.7379	1.7379	0.5000	0.1250	0.0792
26	Ni	6.9517	5.2138	1.7379	0.5000	0.3750	0.0792
27	Ni	10.4275	1.7379	1.7379	0.7500	0.1250	0.0792
28	Ni	10.4275	5.2138	1.7379	0.7500	0.3750	0.0792
29	Ni	6.9517	8.6896	1.7379	0.5000	0.6250	0.0792
30	Ni	6.9517	12.1654	1.7379	0.5000	0.8750	0.0792
31	Ni	10.4275	8.6896	1.7379	0.7500	0.6250	0.0792
32	Ni	10.4275	12.1654	1.7379	0.7500	0.8750	0.0792
33	Ni	0.0007	13.8987	3.5061	0.0001	0.9997	0.1597
34	Ni	0.0004	3.4738	3.5021	0.0000	0.2499	0.1595
35	Ni	3.4724	13.9019	3.4990	0.2498	0.9999	0.1594
36	Ni	3.4632	3.4472	3.4622	0.2491	0.2479	0.1577
37	Ni	0.0033	6.9579	3.5152	0.0002	0.5004	0.1601
38	Ni	13.8938	10.4340	3.4958	0.9993	0.7505	0.1592
39	Ni	3.4810	6.9519	3.4931	0.2504	0.5000	0.1591
40	Ni	3.4935	10.4033	3.5191	0.2513	0.7483	0.1603

41	Ni	6.9578	0.0139	3.4494	0.5004	0.0010	0.1571
42	Ni	6.9599	3.4776	3.5256	0.5006	0.2501	0.1606
43	Ni	10.4272	0.0026	3.5020	0.7500	0.0002	0.1595
44	Ni	10.4289	3.4754	3.5035	0.7501	0.2500	0.1596
45	Ni	6.9657	6.9639	3.4923	0.5010	0.5009	0.1591
46	Ni	6.9615	10.4509	3.4862	0.5007	0.7517	0.1588
47	Ni	10.4323	6.9457	3.4911	0.7503	0.4996	0.1590
48	Ni	10.4270	10.4359	3.4978	0.7500	0.7506	0.1593
49	Ni	0.0021	1.7362	5.2109	0.0002	0.1249	0.2374
50	Ni	0.0020	5.2090	5.2157	0.0001	0.3747	0.2376
51	Ni	3.4628	1.7369	5.2057	0.2491	0.1249	0.2371
52	Ni	3.4555	5.1654	5.0817	0.2485	0.3715	0.2315
53	Ni	13.8821	8.7192	5.1955	0.9985	0.6271	0.2367
54	Ni	0.0019	12.1629	5.2129	0.0001	0.8748	0.2375
55	Ni	3.4511	8.6605	5.2346	0.2482	0.6229	0.2385
56	Ni	3.4813	12.1600	5.2166	0.2504	0.8746	0.2376
57	Ni	6.9477	1.7409	5.2510	0.4997	0.1252	0.2392
58	Ni	6.9799	5.1405	5.3645	0.5020	0.3697	0.2444
59	Ni	10.4384	1.7311	5.2053	0.7508	0.1245	0.2371
60	Ni	10.4252	5.2186	5.2115	0.7498	0.3753	0.2374
61	Ni	6.9921	8.7184	5.2181	0.5029	0.6271	0.2377
62	Ni	6.9645	12.2375	5.1894	0.5009	0.8802	0.2364
63	Ni	10.4263	8.6863	5.1988	0.7499	0.6248	0.2368
64	Ni	10.4293	12.1951	5.2762	0.7501	0.8771	0.2404
65	Co	1.7379	1.7379	0.0000	0.1250	0.1250	0.0000
66	Co	1.7379	5.2138	0.0000	0.1250	0.3750	0.0000
67	Co	5.2138	1.7379	0.0000	0.3750	0.1250	0.0000
68	Co	5.2138	5.2138	0.0000	0.3750	0.3750	0.0000
69	Co	1.7379	8.6896	0.0000	0.1250	0.6250	0.0000
70	Co	1.7379	12.1654	0.0000	0.1250	0.8750	0.0000
71	Co	5.2138	8.6896	0.0000	0.3750	0.6250	0.0000
72	Co	5.2138	12.1654	0.0000	0.3750	0.8750	0.0000
73	Co	8.6896	1.7379	0.0000	0.6250	0.1250	0.0000
74	Co	8.6896	5.2138	0.0000	0.6250	0.3750	0.0000
75	Co	12.1654	1.7379	0.0000	0.8750	0.1250	0.0000
76	Co	12.1654	5.2138	0.0000	0.8750	0.3750	0.0000
77	Co	8.6896	8.6896	0.0000	0.6250	0.6250	0.0000
78	Co	8.6896	12.1654	0.0000	0.6250	0.8750	0.0000
79	Co	12.1654	8.6896	0.0000	0.8750	0.6250	0.0000
80	Co	12.1654	12.1654	0.0000	0.8750	0.8750	0.0000
81	Co	1.7379	0.0000	1.7379	0.1250	0.0000	0.0792
82	Co	1.7379	3.4758	1.7379	0.1250	0.2500	0.0792
83	Co	5.2138	0.0000	1.7379	0.3750	0.0000	0.0792
84	Co	5.2138	3.4758	1.7379	0.3750	0.2500	0.0792
85	Co	1.7379	6.9517	1.7379	0.1250	0.5000	0.0792
86	Co	1.7379	10.4275	1.7379	0.1250	0.7500	0.0792
87	Co	5.2138	6.9517	1.7379	0.3750	0.5000	0.0792
88	Co	5.2138	10.4275	1.7379	0.3750	0.7500	0.0792
89	Co	8.6896	0.0000	1.7379	0.6250	0.0000	0.0792

90	Co	8.6896	3.4758	1.7379	0.6250	0.2500	0.0792
91	Co	12.1654	0.0000	1.7379	0.8750	0.0000	0.0792
92	Co	12.1654	3.4758	1.7379	0.8750	0.2500	0.0792
93	Co	8.6896	6.9517	1.7379	0.6250	0.5000	0.0792
94	Co	8.6896	10.4275	1.7379	0.6250	0.7500	0.0792
95	Co	12.1654	6.9517	1.7379	0.8750	0.5000	0.0792
96	Co	12.1654	10.4275	1.7379	0.8750	0.7500	0.0792
97	Co	1.7310	1.7333	3.5531	0.1245	0.1247	0.1619
98	Co	1.7046	5.2345	3.5255	0.1226	0.3765	0.1606
99	Co	5.2124	1.7304	3.5329	0.3749	0.1245	0.1609
100	Co	5.2480	5.1838	3.5205	0.3775	0.3728	0.1604
101	Co	1.7306	8.6830	3.5333	0.1245	0.6245	0.1610
102	Co	1.7472	12.1657	3.5486	0.1257	0.8750	0.1617
103	Co	5.2060	8.6806	3.5608	0.3744	0.6244	0.1622
104	Co	5.2137	12.1663	3.5598	0.3750	0.8751	0.1622
105	Co	8.6899	1.7281	3.5592	0.6250	0.1243	0.1621
106	Co	8.6694	5.2022	3.5692	0.6235	0.3742	0.1626
107	Co	12.1693	1.7401	3.5533	0.8753	0.1252	0.1619
108	Co	12.1681	5.2132	3.5589	0.8752	0.3750	0.1621
109	Co	8.6876	8.6949	3.5283	0.6249	0.6254	0.1607
110	Co	8.7057	12.1894	3.4661	0.6262	0.8767	0.1579
111	Co	12.1595	8.6938	3.5472	0.8746	0.6253	0.1616
112	Co	12.1509	12.1680	3.5713	0.8740	0.8752	0.1627
113	Co	1.7514	13.8970	5.2250	0.1260	0.9995	0.2380
114	Co	1.7432	3.4820	5.2176	0.1254	0.2504	0.2377
115	Co	5.2300	0.0208	5.2539	0.3762	0.0015	0.2393
116	Co	5.1601	3.4374	5.1946	0.3711	0.2472	0.2366
117	Co	1.7139	6.9498	5.3282	0.1233	0.4999	0.2427
118	Co	1.7362	10.4590	5.1881	0.1249	0.7523	0.2363
119	Co	5.2651	6.8731	5.1728	0.3787	0.4943	0.2356
120	Co	5.2140	10.4390	5.2290	0.3750	0.7508	0.2382
121	Co	8.6662	0.0095	5.3184	0.6233	0.0007	0.2423
122	Co	8.7253	3.4539	5.2231	0.6276	0.2484	0.2379
123	Co	12.1687	0.0025	5.2134	0.8752	0.0002	0.2375
124	Co	12.1653	3.4744	5.2249	0.8750	0.2499	0.2380
125	Co	8.7151	6.9739	5.1747	0.6268	0.5016	0.2357
126	Co	8.7413	10.4342	5.2177	0.6287	0.7505	0.2377
127	Co	12.1667	6.9551	5.2183	0.8751	0.5002	0.2377
128	Co	12.1627	10.4273	5.2164	0.8748	0.7500	0.2376
129	O	5.9705	6.0482	6.7895	0.4294	0.4350	0.3093
130	O	2.6394	7.7813	6.8349	0.1898	0.5597	0.3114
131	O	4.8489	7.7788	8.7708	0.3488	0.5595	0.3996
132	O	6.0656	9.6667	6.8034	0.4363	0.6953	0.3099
133	O	7.9016	9.6706	8.9706	0.5683	0.6956	0.4087
134	O	4.9927	11.3334	8.8562	0.3591	0.8152	0.4034
135	O	8.7041	12.0038	6.3091	0.6260	0.8634	0.2874
136	Ce	3.8679	9.7694	8.0121	0.2782	0.7027	0.3650
137	Ce	3.8594	6.0653	7.6943	0.2776	0.4362	0.3505
138	Ce	7.1126	7.8947	8.0784	0.5116	0.5678	0.3680

139 Sm	7.0191	11.4196	8.0817	0.5048	0.8214	0.3682
140 C	6.7659	0.1800	6.4059	0.4866	0.0129	0.2918
141 O	6.9328	0.0477	7.6340	0.4986	0.0034	0.3478

/db/jmorales/CoNi-alloy/Adsorptions/CO_2-Sm(surf)$Ce_{26}O_{53}$

a =			11.6082000732				
b =			11.608200073110298				
c =			22.898399353				
alpha =			90.0				
beta =			90.0				
gamma =			120.0				
	Atom	X	Y	Z	X	Y	Z
1	Sm	2.0168	3.0873	7.1856	0.3273	0.3071	0.3138
2	Ce	1.9347	1.1170	0.7898	0.2222	0.1111	0.0345
3	Ce	0.0000	4.4680	0.7898	0.2222	0.4444	0.0345
4	Ce	-1.9347	7.8190	0.7898	0.2222	0.7778	0.0345
5	Ce	5.8041	1.1170	0.7898	0.5556	0.1111	0.0345
6	Ce	3.8694	4.4680	0.7898	0.5556	0.4444	0.0345
7	Ce	1.9347	7.8190	0.7898	0.5556	0.7778	0.0345
8	Ce	9.6735	1.1170	0.7898	0.8889	0.1111	0.0345
9	Ce	7.7388	4.4680	0.7898	0.8889	0.4444	0.0345
10	Ce	5.8041	7.8190	0.7898	0.8889	0.7778	0.0345
11	Ce	-0.0055	2.2422	3.9936	0.1110	0.2230	0.1744
12	Ce	-1.9121	5.5719	4.0005	0.1124	0.5543	0.1747
13	Ce	-3.8678	8.9339	3.9445	0.1111	0.8887	0.1723
14	Ce	3.8595	2.2370	3.9495	0.4437	0.2225	0.1725
15	Ce	1.9432	5.5810	3.8657	0.4450	0.5552	0.1688
16	Ce	-0.0024	8.9459	3.9303	0.4447	0.8899	0.1716
17	Ce	7.7343	2.2396	3.9256	0.7777	0.2228	0.1714
18	Ce	5.8036	5.5770	3.9845	0.7773	0.5548	0.1740
19	Ce	3.8630	8.9328	3.9900	0.7771	0.8886	0.1742
20	Ce	-0.0002	-0.0235	7.1036	-0.0012	-0.0023	0.3102
21	Ce	9.5506	3.2418	7.1430	0.9840	0.3225	0.3119
22	Ce	-3.8748	6.6912	7.1229	-0.0010	0.6656	0.3111
23	Ce	3.8241	0.0352	7.0820	0.3312	0.0035	0.3093
24	Ce	-0.1439	7.0218	7.2143	0.3368	0.6985	0.3151
25	Ce	7.7142	0.0379	7.0964	0.6664	0.0038	0.3099
26	Ce	5.7725	3.3465	7.1089	0.6637	0.3329	0.3105
27	Ce	3.9659	6.7292	7.3028	0.6763	0.6694	0.3189
28	O	0.0000	0.0000	0.0000	0.0000	0.0000	0.0000
29	O	-1.9347	3.3510	0.0000	-0.0000	0.3333	0.0000
30	O	-3.8694	6.7020	0.0000	-0.0000	0.6667	0.0000
31	O	3.8694	0.0000	0.0000	0.3333	0.0000	0.0000
32	O	1.9347	3.3510	0.0000	0.3333	0.3333	0.0000
33	O	0.0000	6.7020	0.0000	0.3333	0.6667	0.0000
34	O	7.7388	0.0000	0.0000	0.6667	0.0000	0.0000
35	O	5.8041	3.3510	0.0000	0.6667	0.3333	0.0000
36	O	3.8694	6.7020	0.0000	0.6667	0.6667	0.0000
37	O	-0.0000	2.2340	1.5798	0.1111	0.2222	0.0690
38	O	-1.9347	5.5850	1.5798	0.1111	0.5556	0.0690
39	O	-3.8694	8.9360	1.5798	0.1111	0.8889	0.0690
40	O	3.8694	2.2340	1.5798	0.4444	0.2222	0.0690

41	O	1.9347	5.5850	1.5798	0.4444	0.5556	0.0690
42	O	-0.0000	8.9360	1.5798	0.4444	0.8889	0.0690
43	O	7.7388	2.2340	1.5798	0.7778	0.2222	0.0690
44	O	5.8041	5.5850	1.5798	0.7778	0.5556	0.0690
45	O	3.8694	8.9360	1.5798	0.7778	0.8889	0.0690
46	O	1.9347	1.1170	3.1593	0.2222	0.1111	0.1380
47	O	0.0000	4.4680	3.1593	0.2222	0.4444	0.1380
48	O	-1.9347	7.8190	3.1593	0.2222	0.7778	0.1380
49	O	5.8041	1.1170	3.1593	0.5556	0.1111	0.1380
50	O	3.8694	4.4680	3.1593	0.5556	0.4444	0.1380
51	O	1.9347	7.8190	3.1593	0.5556	0.7778	0.1380
52	O	9.6735	1.1170	3.1593	0.8889	0.1111	0.1380
53	O	7.7388	4.4680	3.1593	0.8889	0.4444	0.1380
54	O	5.8041	7.8190	3.1593	0.8889	0.7778	0.1380
55	O	0.0023	0.0188	4.7400	0.0011	0.0019	0.2070
56	O	-1.9574	3.3688	4.7680	-0.0011	0.3351	0.2082
57	O	-3.7964	6.6594	4.7732	0.0042	0.6624	0.2085
58	O	3.8472	-0.0093	4.6930	0.3310	-0.0009	0.2049
59	O	1.9305	3.4892	4.8573	0.3398	0.3471	0.2121
60	O	0.1261	6.6642	4.8836	0.3423	0.6629	0.2133
61	O	1.9311	10.0418	4.7402	0.6658	0.9989	0.2070
62	O	5.7883	3.3645	4.7393	0.6660	0.3347	0.2070
63	O	3.7152	6.6111	4.8735	0.6489	0.6576	0.2128
64	O	-0.0622	2.2311	6.3146	0.1056	0.2219	0.2758
65	O	-1.8201	5.5481	6.4417	0.1192	0.5519	0.2813
66	O	-3.8994	8.9075	6.2347	0.1071	0.8861	0.2723
67	O	3.8873	2.1421	6.2436	0.4414	0.2131	0.2727
68	O	-0.0783	9.0409	6.2501	0.4429	0.8993	0.2729
69	O	7.7214	2.2277	6.2607	0.7760	0.2216	0.2734
70	O	5.8868	5.6219	6.2834	0.7867	0.5592	0.2744
71	O	3.9186	8.9896	6.2996	0.7847	0.8942	0.2751
72	O	1.9323	0.9476	7.9314	0.2136	0.0943	0.3464
73	O	-0.0553	3.4895	8.8225	0.1688	0.3471	0.3853
74	O	-2.0239	7.9512	7.9209	0.2211	0.7909	0.3459
75	O	5.8051	1.1318	7.9063	0.5564	0.1126	0.3453
76	O	3.9614	4.3612	7.8241	0.5582	0.4338	0.3417
77	O	1.8211	7.9451	7.8082	0.5520	0.7903	0.3410
78	O	9.5798	1.1198	7.9165	0.8810	0.1114	0.3457
79	O	7.7319	4.4176	7.8922	0.8858	0.4394	0.3447
80	O	5.9231	7.8568	7.9812	0.9010	0.7815	0.3485
81	C	0.1539	4.7572	8.7543	0.2499	0.4732	0.3823
82	O	1.1598	5.1554	7.9332	0.3563	0.5128	0.3465
83	O	-0.5661	5.6689	9.2627	0.2332	0.5639	0.4045

/db/jmorales/CoNi-alloy/Adsorptions/Coverage/Ni(111)-2CO

a =		9.8299999236
b =		9.829999923841429
c =		21.0196990967
alpha =		90.0
beta =		90.0
gamma =		120.0

	Atom	X	Y	Z	X	Y	Z
1	Ni	0.0000	0.0000	0.0000	0.0000	0.0000	0.0000
2	Ni	-1.2287	2.1283	0.0000	0.0000	0.2500	0.0000
3	Ni	-2.4575	4.2565	0.0000	0.0000	0.5000	0.0000
4	Ni	-3.6862	6.3848	0.0000	-0.0000	0.7500	0.0000
5	Ni	2.4575	0.0000	0.0000	0.2500	0.0000	0.0000
6	Ni	1.2287	2.1283	0.0000	0.2500	0.2500	0.0000
7	Ni	0.0000	4.2565	0.0000	0.2500	0.5000	0.0000
8	Ni	-1.2287	6.3848	0.0000	0.2500	0.7500	0.0000
9	Ni	4.9150	0.0000	0.0000	0.5000	0.0000	0.0000
10	Ni	3.6862	2.1283	0.0000	0.5000	0.2500	0.0000
11	Ni	2.4575	4.2565	0.0000	0.5000	0.5000	0.0000
12	Ni	1.2287	6.3848	0.0000	0.5000	0.7500	0.0000
13	Ni	7.3725	0.0000	0.0000	0.7500	0.0000	0.0000
14	Ni	6.1437	2.1283	0.0000	0.7500	0.2500	0.0000
15	Ni	4.9150	4.2565	0.0000	0.7500	0.5000	0.0000
16	Ni	3.6862	6.3848	0.0000	0.7500	0.7500	0.0000
17	Ni	-0.0000	1.4188	2.0065	0.0833	0.1667	0.0955
18	Ni	-1.2288	3.5471	2.0065	0.0833	0.4167	0.0955
19	Ni	-2.4575	5.6754	2.0065	0.0833	0.6667	0.0955
20	Ni	-3.6863	7.8036	2.0065	0.0833	0.9167	0.0955
21	Ni	2.4575	1.4188	2.0065	0.3333	0.1667	0.0955
22	Ni	1.2287	3.5471	2.0065	0.3333	0.4167	0.0955
23	Ni	-0.0000	5.6754	2.0065	0.3333	0.6667	0.0955
24	Ni	-1.2288	7.8036	2.0065	0.3333	0.9167	0.0955
25	Ni	4.9150	1.4188	2.0065	0.5833	0.1667	0.0955
26	Ni	3.6862	3.5471	2.0065	0.5833	0.4167	0.0955
27	Ni	2.4575	5.6754	2.0065	0.5833	0.6667	0.0955
28	Ni	1.2287	7.8036	2.0065	0.5833	0.9167	0.0955
29	Ni	7.3725	1.4188	2.0065	0.8333	0.1667	0.0955
30	Ni	6.1437	3.5471	2.0065	0.8333	0.4167	0.0955
31	Ni	4.9150	5.6754	2.0065	0.8333	0.6667	0.0955
32	Ni	3.6862	7.8036	2.0065	0.8333	0.9167	0.0955
33	Ni	1.2265	0.7142	4.0118	0.1667	0.0839	0.1909
34	Ni	-0.0011	2.8526	4.0268	0.1674	0.3351	0.1916
35	Ni	-1.2158	4.9569	4.0259	0.1674	0.5823	0.1915
36	Ni	-2.4555	7.0887	4.0140	0.1665	0.8327	0.1910
37	Ni	3.6796	0.7057	4.0020	0.4158	0.0829	0.1904
38	Ni	2.4493	2.8407	4.0020	0.4160	0.3337	0.1904
39	Ni	1.2228	4.9631	4.0290	0.4159	0.5830	0.1917
40	Ni	-0.0021	7.0863	4.0023	0.4160	0.8324	0.1904
41	Ni	6.1422	0.7051	4.0051	0.6663	0.0828	0.1905

42	Ni	4.9077	2.8398	4.0061	0.6660	0.3336	0.1906
43	Ni	3.6729	4.9641	4.0149	0.6652	0.5831	0.1910
44	Ni	2.4464	7.0855	4.0165	0.6650	0.8323	0.1911
45	Ni	8.6021	0.7112	4.0074	0.9169	0.0835	0.1907
46	Ni	7.3756	2.8397	4.0041	0.9171	0.3336	0.1905
47	Ni	6.1444	4.9670	4.0072	0.9168	0.5835	0.1906
48	Ni	4.9100	7.0920	4.0108	0.9160	0.8331	0.1908
49	Ni	-0.0069	-0.0043	6.0107	-0.0010	-0.0005	0.2860
50	Ni	8.5747	2.1213	6.0119	0.9969	0.2492	0.2860
51	Ni	-2.4773	4.2385	6.0115	-0.0031	0.4979	0.2860
52	Ni	-3.6974	6.3803	6.0125	-0.0014	0.7495	0.2860
53	Ni	2.4467	-0.0080	6.0045	0.2484	-0.0009	0.2857
54	Ni	1.2134	2.1133	6.0586	0.2476	0.2482	0.2882
55	Ni	0.0200	4.2664	6.2382	0.2526	0.5012	0.2968
56	Ni	-1.2497	6.3776	6.0584	0.2474	0.7492	0.2882
57	Ni	4.9084	0.0222	6.0056	0.5006	0.0026	0.2857
58	Ni	3.7032	2.1096	6.0061	0.5006	0.2478	0.2857
59	Ni	2.4762	4.2350	6.0832	0.5006	0.4975	0.2894
60	Ni	1.2187	6.4124	6.0847	0.5006	0.7532	0.2895
61	Ni	7.3704	0.0007	6.0134	0.7498	0.0001	0.2861
62	Ni	6.1391	2.1272	6.0145	0.7495	0.2499	0.2861
63	Ni	4.9127	4.2560	6.0140	0.7497	0.4999	0.2861
64	Ni	3.6714	6.3785	6.0139	0.7481	0.7493	0.2861
65	O	1.4668	3.3316	8.6406	0.3449	0.3914	0.4111
66	O	-0.0731	5.9883	8.6411	0.3443	0.7034	0.4111
67	C	1.3813	3.3688	7.4541	0.3384	0.3957	0.3546
68	C	-0.0802	5.8947	7.4549	0.3381	0.6924	0.3547

/db/jmorales/CoNi-alloy/Adsorptions/Functionals/RPBE/Ni(001)
a = 6.9509918686
b = 6.9509918686
c = 21.9509918687
alpha = 90.0
beta = 90.0
gamma = 90.0

	Atom	X	Y	Z	X	Y	Z
1	Ni	0.0000	0.0000	0.0000	0.0000	0.0000	0.0000
2	Ni	1.7377	1.7377	0.0000	0.2500	0.2500	0.0000
3	Ni	0.0000	3.4755	0.0000	0.0000	0.5000	0.0000
4	Ni	1.7377	5.2132	0.0000	0.2500	0.7500	0.0000
5	Ni	3.4755	0.0000	0.0000	0.5000	0.0000	0.0000
6	Ni	5.2132	1.7377	0.0000	0.7500	0.2500	0.0000
7	Ni	3.4755	3.4755	0.0000	0.5000	0.5000	0.0000
8	Ni	5.2132	5.2132	0.0000	0.7500	0.7500	0.0000
9	Ni	0.0000	1.7377	1.7377	0.0000	0.2500	0.0792
10	Ni	1.7377	0.0000	1.7377	0.2500	0.0000	0.0792
11	Ni	0.0000	5.2132	1.7377	0.0000	0.7500	0.0792
12	Ni	1.7377	3.4755	1.7377	0.2500	0.5000	0.0792
13	Ni	3.4755	1.7377	1.7377	0.5000	0.2500	0.0792
14	Ni	5.2132	0.0000	1.7377	0.7500	0.0000	0.0792
15	Ni	3.4755	5.2132	1.7377	0.5000	0.7500	0.0792
16	Ni	5.2132	3.4755	1.7377	0.7500	0.5000	0.0792
17	Ni	0.0000	0.0000	3.5182	0.0000	0.0000	0.1603
18	Ni	1.7377	1.7377	3.5182	0.2500	0.2500	0.1603
19	Ni	0.0000	3.4755	3.5182	0.0000	0.5000	0.1603
20	Ni	1.7377	5.2132	3.5182	0.2500	0.7500	0.1603
21	Ni	3.4755	0.0000	3.5182	0.5000	0.0000	0.1603
22	Ni	5.2132	1.7377	3.5182	0.7500	0.2500	0.1603
23	Ni	3.4755	3.4755	3.5182	0.5000	0.5000	0.1603
24	Ni	5.2132	5.2132	3.5182	0.7500	0.7500	0.1603
25	Ni	0.0000	1.7377	5.2613	0.0000	0.2500	0.2397
26	Ni	1.7377	0.0000	5.2613	0.2500	0.0000	0.2397
27	Ni	0.0000	5.2132	5.2613	0.0000	0.7500	0.2397
28	Ni	1.7377	3.4755	5.2613	0.2500	0.5000	0.2397
29	Ni	3.4755	1.7377	5.2613	0.5000	0.2500	0.2397
30	Ni	5.2132	0.0000	5.2613	0.7500	0.0000	0.2397
31	Ni	3.4755	5.2132	5.2613	0.5000	0.7500	0.2397
32	Ni	5.2132	3.4755	5.2613	0.7500	0.5000	0.2397

/db/jmorales/CoNi-alloy/Adsorptions/CO_2-CoNi(001)

a =		6.951669213					
b =		6.951669213					
c =		21.951669213					
alpha =		90.0					
beta =		90.0					
gamma =		90.0					
	Atom	X	Y	Z	X	Y	Z
1	Ni	0.0000	0.0000	0.0000	0.0000	0.0000	0.0000
2	Ni	0.0000	3.4758	0.0000	0.0000	0.5000	0.0000
3	Ni	3.4758	0.0000	0.0000	0.5000	0.0000	0.0000
4	Ni	3.4758	3.4758	0.0000	0.5000	0.5000	0.0000
5	Ni	0.0000	1.7379	1.7379	0.0000	0.2500	0.0792
6	Ni	0.0000	5.2138	1.7379	0.0000	0.7500	0.0792
7	Ni	3.4758	1.7379	1.7379	0.5000	0.2500	0.0792
8	Ni	3.4758	5.2138	1.7379	0.5000	0.7500	0.0792
9	Ni	0.0019	6.9513	3.5051	0.0003	0.9999	0.1597
10	Ni	6.9515	3.4750	3.5015	1.0000	0.4999	0.1595
11	Ni	3.4736	0.0017	3.5039	0.4997	0.0002	0.1596
12	Ni	3.4775	3.4743	3.4928	0.5002	0.4998	0.1591
13	Ni	6.9290	1.7197	5.1737	0.9967	0.2474	0.2357
14	Ni	6.9389	5.2259	5.1747	0.9982	0.7518	0.2357
15	Ni	3.4484	1.7599	5.2570	0.4960	0.2532	0.2395
16	Ni	3.4966	5.2364	5.1730	0.5030	0.7533	0.2357
17	Co	1.7379	1.7379	0.0000	0.2500	0.2500	0.0000
18	Co	1.7379	5.2138	0.0000	0.2500	0.7500	0.0000
19	Co	5.2138	1.7379	0.0000	0.7500	0.2500	0.0000
20	Co	5.2138	5.2138	0.0000	0.7500	0.7500	0.0000
21	Co	1.7379	0.0000	1.7379	0.2500	0.0000	0.0792
22	Co	1.7379	3.4758	1.7379	0.2500	0.5000	0.0792
23	Co	5.2138	0.0000	1.7379	0.7500	0.0000	0.0792
24	Co	5.2138	3.4758	1.7379	0.7500	0.5000	0.0792
25	Co	1.7397	1.7316	3.5230	0.2503	0.2491	0.1605
26	Co	1.7368	5.2202	3.5313	0.2498	0.7509	0.1609
27	Co	5.2200	1.7405	3.5334	0.7509	0.2504	0.1610
28	Co	5.2146	5.2124	3.5386	0.7501	0.7498	0.1612
29	Co	1.7336	6.9480	5.1904	0.2494	0.9995	0.2364
30	Co	1.7304	3.4844	5.1619	0.2489	0.5012	0.2351
31	Co	5.2154	6.9509	5.1971	0.7502	0.9999	0.2368
32	Co	5.2194	3.4807	5.1901	0.7508	0.5007	0.2364
33	C	3.5112	4.1099	6.7524	0.5051	0.5912	0.3076
34	O	4.6045	3.9090	7.2203	0.6624	0.5623	0.3289
35	O	2.2793	3.9097	7.0774	0.3279	0.5624	0.3224

/db/jmorales/CoNi-alloy/Adsorptions/Functionals/RPBE/Ni-CoNi(001)-CO

a = 6.9516692132
b = 6.9516692132
c = 21.9516692133
alpha = 90.0
beta = 90.0
gamma = 90.0

	Atom	X	Y	Z	X	Y	Z
1	Co	0.0000	0.0000	0.0000	0.0000	0.0000	0.0000
2	Co	1.7379	1.7379	0.0000	0.2500	0.2500	0.0000
3	Co	0.0000	3.4758	0.0000	0.0000	0.5000	0.0000
4	Co	1.7379	5.2138	0.0000	0.2500	0.7500	0.0000
5	Co	3.4758	0.0000	0.0000	0.5000	0.0000	0.0000
6	Co	5.2138	1.7379	0.0000	0.7500	0.2500	0.0000
7	Co	3.4758	3.4758	0.0000	0.5000	0.5000	0.0000
8	Co	5.2138	5.2138	0.0000	0.7500	0.7500	0.0000
9	Co	0.0003	-0.0001	3.5593	0.0000	-0.0000	0.1621
10	Co	1.7337	1.7343	3.5560	0.2494	0.2495	0.1620
11	Co	-0.0004	3.4760	3.5610	-0.0001	0.5000	0.1622
12	Co	1.7340	5.2175	3.5556	0.2494	0.7505	0.1620
13	Co	3.4755	-0.0000	3.5613	0.5000	-0.0000	0.1622
14	Co	5.2175	1.7340	3.5562	0.7505	0.2494	0.1620
15	Co	3.4759	3.4758	3.5045	0.5000	0.5000	0.1596
16	Co	5.2173	5.2179	3.5560	0.7505	0.7506	0.1620
17	Ni	1.7379	0.0000	1.7379	0.2500	0.0000	0.0792
18	Ni	0.0000	1.7379	1.7379	0.0000	0.2500	0.0792
19	Ni	1.7379	3.4758	1.7379	0.2500	0.5000	0.0792
20	Ni	0.0000	5.2138	1.7379	0.0000	0.7500	0.0792
21	Ni	5.2138	0.0000	1.7379	0.7500	0.0000	0.0792
22	Ni	3.4758	1.7379	1.7379	0.5000	0.2500	0.0792
23	Ni	5.2138	3.4758	1.7379	0.7500	0.5000	0.0792
24	Ni	3.4758	5.2138	1.7379	0.5000	0.7500	0.0792
25	Ni	1.7405	-0.0001	5.2504	0.2504	-0.0000	0.2392
26	Ni	0.0001	1.7414	5.2506	0.0000	0.2505	0.2392
27	Ni	1.7619	3.4759	5.2915	0.2534	0.5000	0.2411
28	Ni	0.0002	5.2106	5.2509	0.0000	0.7495	0.2392
29	Ni	5.2108	0.0001	5.2500	0.7496	0.0000	0.2392
30	Ni	3.4756	1.7622	5.2917	0.5000	0.2535	0.2411
31	Ni	5.1897	3.4761	5.2919	0.7465	0.5000	0.2411
32	Ni	3.4759	5.1893	5.2922	0.5000	0.7465	0.2411
33	O	3.4756	3.4760	7.6562	0.5000	0.5000	0.3488
34	C	3.4756	3.4758	6.4399	0.5000	0.5000	0.2934

/db/jmorales/CoNi-alloy/Adsorptions/CO_2-Ni-Ce_3SmO_7

a = 13.9019837372
b = 13.9019837372
c = 21.9509918687
alpha = 90.0
beta = 90.0
gamma = 90.0

	Atom	X	Y	Z	X	Y	Z
1	Ni	0.0000	0.0000	0.0000	0.0000	0.0000	0.0000
2	Ni	1.7377	1.7377	0.0000	0.1250	0.1250	0.0000
3	Ni	0.0000	3.4755	0.0000	0.0000	0.2500	0.0000
4	Ni	1.7377	5.2132	0.0000	0.1250	0.3750	0.0000
5	Ni	3.4755	0.0000	0.0000	0.2500	0.0000	0.0000
6	Ni	5.2132	1.7377	0.0000	0.3750	0.1250	0.0000
7	Ni	3.4755	3.4755	0.0000	0.2500	0.2500	0.0000
8	Ni	5.2132	5.2132	0.0000	0.3750	0.3750	0.0000
9	Ni	0.0000	6.9510	0.0000	0.0000	0.5000	0.0000
10	Ni	1.7377	8.6887	0.0000	0.1250	0.6250	0.0000
11	Ni	0.0000	10.4265	0.0000	0.0000	0.7500	0.0000
12	Ni	1.7377	12.1642	0.0000	0.1250	0.8750	0.0000
13	Ni	3.4755	6.9510	0.0000	0.2500	0.5000	0.0000
14	Ni	5.2132	8.6887	0.0000	0.3750	0.6250	0.0000
15	Ni	3.4755	10.4265	0.0000	0.2500	0.7500	0.0000
16	Ni	5.2132	12.1642	0.0000	0.3750	0.8750	0.0000
17	Ni	6.9510	0.0000	0.0000	0.5000	0.0000	0.0000
18	Ni	8.6887	1.7377	0.0000	0.6250	0.1250	0.0000
19	Ni	6.9510	3.4755	0.0000	0.5000	0.2500	0.0000
20	Ni	8.6887	5.2132	0.0000	0.6250	0.3750	0.0000
21	Ni	10.4265	0.0000	0.0000	0.7500	0.0000	0.0000
22	Ni	12.1642	1.7377	0.0000	0.8750	0.1250	0.0000
23	Ni	10.4265	3.4755	0.0000	0.7500	0.2500	0.0000
24	Ni	12.1642	5.2132	0.0000	0.8750	0.3750	0.0000
25	Ni	6.9510	6.9510	0.0000	0.5000	0.5000	0.0000
26	Ni	8.6887	8.6887	0.0000	0.6250	0.6250	0.0000
27	Ni	6.9510	10.4265	0.0000	0.5000	0.7500	0.0000
28	Ni	8.6887	12.1642	0.0000	0.6250	0.8750	0.0000
29	Ni	10.4265	6.9510	0.0000	0.7500	0.5000	0.0000
30	Ni	12.1642	8.6887	0.0000	0.8750	0.6250	0.0000
31	Ni	10.4265	10.4265	0.0000	0.7500	0.7500	0.0000
32	Ni	12.1642	12.1642	0.0000	0.8750	0.8750	0.0000
33	Ni	0.0000	1.7377	1.7377	0.0000	0.1250	0.0792
34	Ni	1.7377	0.0000	1.7377	0.1250	0.0000	0.0792
35	Ni	0.0000	5.2132	1.7377	0.0000	0.3750	0.0792
36	Ni	1.7377	3.4755	1.7377	0.1250	0.2500	0.0792
37	Ni	3.4755	1.7377	1.7377	0.2500	0.1250	0.0792
38	Ni	5.2132	0.0000	1.7377	0.3750	0.0000	0.0792
39	Ni	3.4755	5.2132	1.7377	0.2500	0.3750	0.0792
40	Ni	5.2132	3.4755	1.7377	0.3750	0.2500	0.0792

41	Ni	0.0000	8.6887	1.7377	0.0000	0.6250	0.0792
42	Ni	1.7377	6.9510	1.7377	0.1250	0.5000	0.0792
43	Ni	0.0000	12.1642	1.7377	0.0000	0.8750	0.0792
44	Ni	1.7377	10.4265	1.7377	0.1250	0.7500	0.0792
45	Ni	3.4755	8.6887	1.7377	0.2500	0.6250	0.0792
46	Ni	5.2132	6.9510	1.7377	0.3750	0.5000	0.0792
47	Ni	3.4755	12.1642	1.7377	0.2500	0.8750	0.0792
48	Ni	5.2132	10.4265	1.7377	0.3750	0.7500	0.0792
49	Ni	6.9510	1.7377	1.7377	0.5000	0.1250	0.0792
50	Ni	8.6887	0.0000	1.7377	0.6250	0.0000	0.0792
51	Ni	6.9510	5.2132	1.7377	0.5000	0.3750	0.0792
52	Ni	8.6887	3.4755	1.7377	0.6250	0.2500	0.0792
53	Ni	10.4265	1.7377	1.7377	0.7500	0.1250	0.0792
54	Ni	12.1642	0.0000	1.7377	0.8750	0.0000	0.0792
55	Ni	10.4265	5.2132	1.7377	0.7500	0.3750	0.0792
56	Ni	12.1642	3.4755	1.7377	0.8750	0.2500	0.0792
57	Ni	6.9510	8.6887	1.7377	0.5000	0.6250	0.0792
58	Ni	8.6887	6.9510	1.7377	0.6250	0.5000	0.0792
59	Ni	6.9510	12.1642	1.7377	0.5000	0.8750	0.0792
60	Ni	8.6887	10.4265	1.7377	0.6250	0.7500	0.0792
61	Ni	10.4265	8.6887	1.7377	0.7500	0.6250	0.0792
62	Ni	12.1642	6.9510	1.7377	0.8750	0.5000	0.0792
63	Ni	10.4265	12.1642	1.7377	0.7500	0.8750	0.0792
64	Ni	12.1642	10.4265	1.7377	0.8750	0.7500	0.0792
65	Ni	0.0068	13.8999	3.5102	0.0005	0.9999	0.1599
66	Ni	1.7067	1.7516	3.4389	0.1228	0.1260	0.1567
67	Ni	0.0148	3.4836	3.5261	0.0011	0.2506	0.1606
68	Ni	1.7337	5.2034	3.5146	0.1247	0.3743	0.1601
69	Ni	3.4611	13.8606	3.4680	0.2490	0.9970	0.1580
70	Ni	5.2144	1.7171	3.4951	0.3751	0.1235	0.1592
71	Ni	3.4777	3.4845	3.4830	0.2502	0.2506	0.1587
72	Ni	5.2052	5.2033	3.5139	0.3744	0.3743	0.1601
73	Ni	13.8956	6.9510	3.4997	0.9995	0.5000	0.1594
74	Ni	1.7430	8.6885	3.5054	0.1254	0.6250	0.1597
75	Ni	0.0037	10.4263	3.5060	0.0003	0.7500	0.1597
76	Ni	1.7308	12.1651	3.5234	0.1245	0.8751	0.1605
77	Ni	3.4917	6.9307	3.5269	0.2512	0.4985	0.1607
78	Ni	5.1904	8.6774	3.4960	0.3734	0.6242	0.1593
79	Ni	3.4723	10.4230	3.5077	0.2498	0.7497	0.1598
80	Ni	5.2130	12.1521	3.5019	0.3750	0.8741	0.1595
81	Ni	6.9480	0.0021	3.5242	0.4998	0.0002	0.1605
82	Ni	8.6628	1.7212	3.5241	0.6231	0.1238	0.1605
83	Ni	6.9516	3.4792	3.5000	0.5000	0.2503	0.1594
84	Ni	8.7049	5.2319	3.5233	0.6262	0.3763	0.1605
85	Ni	10.4327	0.0068	3.5089	0.7504	0.0005	0.1598
86	Ni	12.1945	1.7381	3.5338	0.8772	0.1250	0.1610
87	Ni	10.4301	3.4799	3.5201	0.7503	0.2503	0.1604
88	Ni	12.1279	5.2062	3.5359	0.8724	0.3745	0.1611
89	Ni	6.9705	6.9615	3.4877	0.5014	0.5008	0.1589

90	Ni	8.7197	8.7079	3.4759	0.6272	0.6264	0.1583
91	Ni	6.9520	10.4620	3.4757	0.5001	0.7526	0.1583
92	Ni	8.6933	12.1751	3.5072	0.6253	0.8758	0.1598
93	Ni	10.4364	6.9394	3.5187	0.7507	0.4992	0.1603
94	Ni	12.1650	8.6924	3.5033	0.8751	0.6253	0.1596
95	Ni	10.4326	10.4370	3.5082	0.7504	0.7508	0.1598
96	Ni	12.1662	12.1683	3.5056	0.8751	0.8753	0.1597
97	Ni	0.0455	1.7247	5.3081	0.0033	0.1241	0.2418
98	Ni	1.7493	13.8482	5.3015	0.1258	0.9961	0.2415
99	Ni	13.8681	5.2433	5.2090	0.9976	0.3772	0.2373
100	Ni	1.6986	3.5293	5.3878	0.1222	0.2539	0.2454
101	Ni	3.4291	1.6933	5.0901	0.2467	0.1218	0.2319
102	Ni	5.1755	13.8560	5.1910	0.3723	0.9967	0.2365
103	Ni	3.4802	5.2089	5.2678	0.2503	0.3747	0.2400
104	Ni	5.2220	3.4279	5.2340	0.3756	0.2466	0.2384
105	Ni	13.8991	8.6869	5.2241	0.9998	0.6249	0.2380
106	Ni	1.7332	6.9743	5.2026	0.1247	0.5017	0.2370
107	Ni	13.8956	12.1596	5.2226	0.9995	0.8747	0.2379
108	Ni	1.7330	10.4213	5.2295	0.1247	0.7496	0.2382
109	Ni	3.4694	8.6868	5.2365	0.2496	0.6249	0.2386
110	Ni	5.2166	6.9976	5.2923	0.3752	0.5034	0.2411
111	Ni	3.4789	12.1300	5.2248	0.2502	0.8725	0.2380
112	Ni	5.2202	10.4133	5.2278	0.3755	0.7490	0.2382
113	Ni	6.9694	1.6708	5.3793	0.5013	0.1202	0.2451
114	Ni	8.7059	13.8869	5.2332	0.6262	0.9989	0.2384
115	Ni	6.9818	5.2336	5.2182	0.5022	0.3765	0.2377
116	Ni	8.6642	3.4552	5.1660	0.6232	0.2485	0.2353
117	Ni	10.4214	1.7415	5.2228	0.7496	0.1253	0.2379
118	Ni	12.1656	0.0015	5.2254	0.8751	0.0001	0.2380
119	Ni	10.4326	5.1438	5.4264	0.7504	0.3700	0.2472
120	Ni	12.1903	3.4477	5.2295	0.8769	0.2480	0.2382
121	Ni	6.9531	8.7525	5.0899	0.5002	0.6296	0.2319
122	Ni	8.7120	7.0390	5.2249	0.6267	0.5063	0.2380
123	Ni	6.9490	12.1593	5.2320	0.4999	0.8746	0.2384
124	Ni	8.6812	10.4358	5.2152	0.6245	0.7507	0.2376
125	Ni	10.4748	8.7390	5.2222	0.7535	0.6286	0.2379
126	Ni	12.1686	6.9563	5.2129	0.8753	0.5004	0.2375
127	Ni	10.4349	12.1610	5.2263	0.7506	0.8748	0.2381
128	Ni	12.1841	10.4414	5.2244	0.8764	0.7511	0.2380
129	O	1.8210	1.6099	6.3211	0.1310	0.1158	0.2880
130	O	5.8754	2.4096	6.8232	0.4226	0.1733	0.3108
131	O	2.7018	4.4662	6.8843	0.1943	0.3213	0.3136
132	O	4.9319	4.2617	8.7651	0.3548	0.3066	0.3993
133	O	6.1656	6.1319	6.8569	0.4435	0.4411	0.3124
134	O	8.3064	6.1924	8.7888	0.5975	0.4454	0.4004
135	O	5.1998	7.8848	8.8615	0.3740	0.5672	0.4037
136	Ce	3.9841	6.2818	8.0332	0.2866	0.4519	0.3660
137	Ce	3.6983	2.5975	7.6897	0.2660	0.1868	0.3503
138	Ce	7.0394	4.1368	7.8403	0.5064	0.2976	0.3572

139 Sm	6.9717	8.0859	7.7342	0.5015	0.5816	0.3523
140 C	9.1769	6.2266	7.8033	0.6601	0.4479	0.3555
141 O	9.2857	7.3801	7.1789	0.6679	0.5309	0.3270
142 O	9.7646	5.1551	7.4078	0.7024	0.3708	0.3375

/db/jmorales/CoNi-alloy/Adsorptions/Functionals/RPBE/Co-CoNi(001)-CO

a = 6.9516692132
b = 6.9516692132
c = 21.9516692133
alpha = 90.0
beta = 90.0
gamma = 90.0

	Atom	X	Y	Z	X	Y	Z
1	Ni	0.0000	0.0000	0.0000	0.0000	0.0000	0.0000
2	Ni	1.7379	1.7379	0.0000	0.2500	0.2500	0.0000
3	Ni	0.0000	3.4758	0.0000	0.0000	0.5000	0.0000
4	Ni	1.7379	5.2138	0.0000	0.2500	0.7500	0.0000
5	Ni	3.4758	0.0000	0.0000	0.5000	0.0000	0.0000
6	Ni	5.2138	1.7379	0.0000	0.7500	0.2500	0.0000
7	Ni	3.4758	3.4758	0.0000	0.5000	0.5000	0.0000
8	Ni	5.2138	5.2138	0.0000	0.7500	0.7500	0.0000
9	Ni	6.9515	0.0001	3.5368	1.0000	0.0000	0.1611
10	Ni	1.7371	1.7362	3.5460	0.2499	0.2498	0.1615
11	Ni	-0.0001	3.4758	3.5436	-0.0000	0.5000	0.1614
12	Ni	1.7367	5.2152	3.5462	0.2498	0.7502	0.1615
13	Ni	3.4759	0.0001	3.5440	0.5000	0.0000	0.1614
14	Ni	5.2152	1.7372	3.5460	0.7502	0.2499	0.1615
15	Ni	3.4756	3.4759	3.5020	0.5000	0.5000	0.1595
16	Ni	5.2154	5.2145	3.5460	0.7502	0.7501	0.1615
17	Co	1.7379	0.0000	1.7379	0.2500	0.0000	0.0792
18	Co	0.0000	1.7379	1.7379	0.0000	0.2500	0.0792
19	Co	1.7379	3.4758	1.7379	0.2500	0.5000	0.0792
20	Co	0.0000	5.2138	1.7379	0.0000	0.7500	0.0792
21	Co	5.2138	0.0000	1.7379	0.7500	0.0000	0.0792
22	Co	3.4758	1.7379	1.7379	0.5000	0.2500	0.0792
23	Co	5.2138	3.4758	1.7379	0.7500	0.5000	0.0792
24	Co	3.4758	5.2138	1.7379	0.5000	0.7500	0.0792
25	Co	1.7401	0.0002	5.3063	0.2503	0.0000	0.2417
26	Co	6.9512	1.7410	5.3066	0.9999	0.2504	0.2417
27	Co	1.7530	3.4759	5.3297	0.2522	0.5000	0.2428
28	Co	6.9511	5.2109	5.3064	0.9999	0.7496	0.2417
29	Co	5.2109	0.0002	5.3068	0.7496	0.0000	0.2417
30	Co	3.4755	1.7535	5.3301	0.5000	0.2522	0.2428
31	Co	5.1976	3.4759	5.3299	0.7477	0.5000	0.2428
32	Co	3.4755	5.1988	5.3298	0.5000	0.7478	0.2428
33	O	3.4750	3.4766	7.7388	0.4999	0.5001	0.3525
34	C	3.4756	3.4760	6.5211	0.5000	0.5000	0.2971

/db/jmorales/CoNi-alloy/Profiles/Ni-Ce_3SmO_7/O-coverage/CO_2-Ni-Ce_3SmO_7-3O

a =			13.9019837372				
b =			13.9019837372				
c =			21.9509918687				
alpha =			90.0				
beta =			90.0				
gamma =			90.0				
	Atom	X	Y	Z	X	Y	Z
1	Ni	0.0000	0.0000	0.0000	0.0000	0.0000	0.0000
2	Ni	1.7377	1.7377	0.0000	0.1250	0.1250	0.0000
3	Ni	0.0000	3.4755	0.0000	0.0000	0.2500	0.0000
4	Ni	1.7377	5.2132	0.0000	0.1250	0.3750	0.0000
5	Ni	3.4755	0.0000	0.0000	0.2500	0.0000	0.0000
6	Ni	5.2132	1.7377	0.0000	0.3750	0.1250	0.0000
7	Ni	3.4755	3.4755	0.0000	0.2500	0.2500	0.0000
8	Ni	5.2132	5.2132	0.0000	0.3750	0.3750	0.0000
9	Ni	0.0000	6.9510	0.0000	0.0000	0.5000	0.0000
10	Ni	1.7377	8.6887	0.0000	0.1250	0.6250	0.0000
11	Ni	0.0000	10.4265	0.0000	0.0000	0.7500	0.0000
12	Ni	1.7377	12.1642	0.0000	0.1250	0.8750	0.0000
13	Ni	3.4755	6.9510	0.0000	0.2500	0.5000	0.0000
14	Ni	5.2132	8.6887	0.0000	0.3750	0.6250	0.0000
15	Ni	3.4755	10.4265	0.0000	0.2500	0.7500	0.0000
16	Ni	5.2132	12.1642	0.0000	0.3750	0.8750	0.0000
17	Ni	6.9510	0.0000	0.0000	0.5000	0.0000	0.0000
18	Ni	8.6887	1.7377	0.0000	0.6250	0.1250	0.0000
19	Ni	6.9510	3.4755	0.0000	0.5000	0.2500	0.0000
20	Ni	8.6887	5.2132	0.0000	0.6250	0.3750	0.0000
21	Ni	10.4265	0.0000	0.0000	0.7500	0.0000	0.0000
22	Ni	12.1642	1.7377	0.0000	0.8750	0.1250	0.0000
23	Ni	10.4265	3.4755	0.0000	0.7500	0.2500	0.0000
24	Ni	12.1642	5.2132	0.0000	0.8750	0.3750	0.0000
25	Ni	6.9510	6.9510	0.0000	0.5000	0.5000	0.0000
26	Ni	8.6887	8.6887	0.0000	0.6250	0.6250	0.0000
27	Ni	6.9510	10.4265	0.0000	0.5000	0.7500	0.0000
28	Ni	8.6887	12.1642	0.0000	0.6250	0.8750	0.0000
29	Ni	10.4265	6.9510	0.0000	0.7500	0.5000	0.0000
30	Ni	12.1642	8.6887	0.0000	0.8750	0.6250	0.0000
31	Ni	10.4265	10.4265	0.0000	0.7500	0.7500	0.0000
32	Ni	12.1642	12.1642	0.0000	0.8750	0.8750	0.0000
33	Ni	0.0000	1.7377	1.7377	0.0000	0.1250	0.0792
34	Ni	1.7377	0.0000	1.7377	0.1250	0.0000	0.0792
35	Ni	0.0000	5.2132	1.7377	0.0000	0.3750	0.0792
36	Ni	1.7377	3.4755	1.7377	0.1250	0.2500	0.0792
37	Ni	3.4755	1.7377	1.7377	0.2500	0.1250	0.0792
38	Ni	5.2132	0.0000	1.7377	0.3750	0.0000	0.0792
39	Ni	3.4755	5.2132	1.7377	0.2500	0.3750	0.0792
40	Ni	5.2132	3.4755	1.7377	0.3750	0.2500	0.0792

41	Ni	0.0000	8.6887	1.7377	0.0000	0.6250	0.0792
42	Ni	1.7377	6.9510	1.7377	0.1250	0.5000	0.0792
43	Ni	0.0000	12.1642	1.7377	0.0000	0.8750	0.0792
44	Ni	1.7377	10.4265	1.7377	0.1250	0.7500	0.0792
45	Ni	3.4755	8.6887	1.7377	0.2500	0.6250	0.0792
46	Ni	5.2132	6.9510	1.7377	0.3750	0.5000	0.0792
47	Ni	3.4755	12.1642	1.7377	0.2500	0.8750	0.0792
48	Ni	5.2132	10.4265	1.7377	0.3750	0.7500	0.0792
49	Ni	6.9510	1.7377	1.7377	0.5000	0.1250	0.0792
50	Ni	8.6887	0.0000	1.7377	0.6250	0.0000	0.0792
51	Ni	6.9510	5.2132	1.7377	0.5000	0.3750	0.0792
52	Ni	8.6887	3.4755	1.7377	0.6250	0.2500	0.0792
53	Ni	10.4265	1.7377	1.7377	0.7500	0.1250	0.0792
54	Ni	12.1642	0.0000	1.7377	0.8750	0.0000	0.0792
55	Ni	10.4265	5.2132	1.7377	0.7500	0.3750	0.0792
56	Ni	12.1642	3.4755	1.7377	0.8750	0.2500	0.0792
57	Ni	6.9510	8.6887	1.7377	0.5000	0.6250	0.0792
58	Ni	8.6887	6.9510	1.7377	0.6250	0.5000	0.0792
59	Ni	6.9510	12.1642	1.7377	0.5000	0.8750	0.0792
60	Ni	8.6887	10.4265	1.7377	0.6250	0.7500	0.0792
61	Ni	10.4265	8.6887	1.7377	0.7500	0.6250	0.0792
62	Ni	12.1642	6.9510	1.7377	0.8750	0.5000	0.0792
63	Ni	10.4265	12.1642	1.7377	0.7500	0.8750	0.0792
64	Ni	12.1642	10.4265	1.7377	0.8750	0.7500	0.0792
65	Ni	0.0107	0.0014	3.5023	0.0008	0.0001	0.1596
66	Ni	1.7404	1.7482	3.4530	0.1252	0.1258	0.1573
67	Ni	0.0028	3.4710	3.5071	0.0002	0.2497	0.1598
68	Ni	1.6990	5.2037	3.4270	0.1222	0.3743	0.1561
69	Ni	3.4756	13.8965	3.5076	0.2500	0.9996	0.1598
70	Ni	5.2117	1.7432	3.5563	0.3749	0.1254	0.1620
71	Ni	3.4931	3.4356	3.4814	0.2513	0.2471	0.1586
72	Ni	5.1917	5.2155	3.4791	0.3734	0.3752	0.1585
73	Ni	13.8781	6.9509	3.5343	0.9983	0.5000	0.1610
74	Ni	1.7444	8.6782	3.5301	0.1255	0.6242	0.1608
75	Ni	13.8873	10.4281	3.5143	0.9989	0.7501	0.1601
76	Ni	1.7443	12.1695	3.5121	0.1255	0.8754	0.1600
77	Ni	3.4671	6.9916	3.4668	0.2494	0.5029	0.1579
78	Ni	5.2341	8.7086	3.5036	0.3765	0.6264	0.1596
79	Ni	3.4740	10.4010	3.5229	0.2499	0.7482	0.1605
80	Ni	5.2150	12.1598	3.4975	0.3751	0.8747	0.1593
81	Ni	6.9406	0.0013	3.5031	0.4992	0.0001	0.1596
82	Ni	8.6949	1.7268	3.4300	0.6254	0.1242	0.1563
83	Ni	6.9302	3.4619	3.4888	0.4985	0.2490	0.1589
84	Ni	8.6854	5.2073	3.5344	0.6248	0.3746	0.1610
85	Ni	10.4367	13.8888	3.5200	0.7507	0.9991	0.1604
86	Ni	12.1668	1.7297	3.5247	0.8752	0.1244	0.1606
87	Ni	10.4426	3.4914	3.5056	0.7512	0.2511	0.1597
88	Ni	12.1776	5.2341	3.5695	0.8760	0.3765	0.1626
89	Ni	6.9299	6.9347	3.5035	0.4985	0.4988	0.1596

90	Ni	8.6904	8.6787	3.4589	0.6251	0.6243	0.1576
91	Ni	6.9874	10.4250	3.5154	0.5026	0.7499	0.1601
92	Ni	8.6926	12.2019	3.5021	0.6253	0.8777	0.1595
93	Ni	10.4327	6.9270	3.5433	0.7504	0.4983	0.1614
94	Ni	12.1798	8.6710	3.4953	0.8761	0.6237	0.1592
95	Ni	10.4331	10.4522	3.4224	0.7505	0.7518	0.1559
96	Ni	12.1709	12.1690	3.4989	0.8755	0.8753	0.1594
97	Ni	0.0302	1.7304	5.2850	0.0022	0.1245	0.2408
98	Ni	1.7448	13.8587	5.2951	0.1255	0.9969	0.2412
99	Ni	0.0367	5.1973	5.2821	0.0026	0.3739	0.2406
100	Ni	1.7586	3.4751	5.3382	0.1265	0.2500	0.2432
101	Ni	3.4620	1.7137	5.2892	0.2490	0.1233	0.2410
102	Ni	5.2044	13.8822	5.2177	0.3744	0.9986	0.2377
103	Ni	3.4360	5.1959	5.0502	0.2472	0.3737	0.2301
104	Ni	5.1825	3.4396	5.3446	0.3728	0.2474	0.2435
105	Ni	0.0153	8.7087	5.2092	0.0011	0.6264	0.2373
106	Ni	1.7192	6.9501	5.2635	0.1237	0.4999	0.2398
107	Ni	13.8975	12.1639	5.2109	0.9997	0.8750	0.2374
108	Ni	1.7223	10.4485	5.2170	0.1239	0.7516	0.2377
109	Ni	3.4565	8.7630	5.3664	0.2486	0.6303	0.2445
110	Ni	5.1826	6.9821	5.2197	0.3728	0.5022	0.2378
111	Ni	3.4932	12.1335	5.2209	0.2513	0.8728	0.2378
112	Ni	5.2553	10.4415	5.1997	0.3780	0.7511	0.2369
113	Ni	6.9494	1.6969	5.2699	0.4999	0.1221	0.2401
114	Ni	8.6975	0.0039	5.2778	0.6256	0.0003	0.2404
115	Ni	6.9027	5.1889	5.2207	0.4965	0.3732	0.2378
116	Ni	8.6692	3.4368	5.2149	0.6236	0.2472	0.2376
117	Ni	10.4371	1.7405	5.2813	0.7508	0.1252	0.2406
118	Ni	12.1823	0.0079	5.2105	0.8763	0.0006	0.2374
119	Ni	10.4155	5.1305	5.3455	0.7492	0.3690	0.2435
120	Ni	12.2003	3.4482	5.2024	0.8776	0.2480	0.2370
121	Ni	6.9967	8.7230	5.2428	0.5033	0.6275	0.2388
122	Ni	8.6872	6.9293	5.2387	0.6249	0.4984	0.2387
123	Ni	6.9515	12.1668	5.2159	0.5000	0.8752	0.2376
124	Ni	8.7094	10.4752	5.2147	0.6265	0.7535	0.2376
125	Ni	10.4205	8.7235	5.1115	0.7496	0.6275	0.2329
126	Ni	12.1793	6.9807	5.4012	0.8761	0.5021	0.2461
127	Ni	10.4492	12.1876	5.2696	0.7516	0.8767	0.2401
128	Ni	12.1757	10.4312	5.2683	0.8758	0.7503	0.2400
129	O	6.0058	4.4169	6.8479	0.4320	0.3177	0.3120
130	O	4.1832	7.6777	6.7774	0.3009	0.5523	0.3088
131	O	5.9437	6.5869	8.7608	0.4275	0.4738	0.3991
132	O	7.9157	7.7474	6.8877	0.5694	0.5573	0.3138
133	O	10.4324	10.4396	6.2767	0.7504	0.7509	0.2859
134	O	9.9147	6.8746	8.8311	0.7132	0.4945	0.4023
135	O	7.7859	9.7295	8.9044	0.5601	0.6999	0.4057
136	Ce	6.0059	8.8213	7.9877	0.4320	0.6345	0.3639
137	Ce	4.1480	5.5688	7.6582	0.2984	0.4006	0.3489
138	Ce	7.8022	5.5652	7.8735	0.5612	0.4003	0.3587

139	Sm	9.5029	9.0705	7.9068	0.6836	0.6525	0.3602
140	C	10.6730	6.4275	7.8440	0.7677	0.4623	0.3573
141	O	11.5644	7.2109	7.3425	0.8319	0.5187	0.3345
142	O	10.3682	5.2563	7.3603	0.7458	0.3781	0.3353
143	O	1.9325	5.2992	6.3297	0.1390	0.3812	0.2884
144	O	1.7419	1.6591	6.1918	0.1253	0.1193	0.2821
145	O	8.6870	1.7562	6.1664	0.6249	0.1263	0.2809

/db/jmorales/CoNi-alloy/Profiles/Sm(surf)$Ce_{26}O_{54}$/CO2

a =				11.6082000732			
b =				11.608200073110298			
c =				22.898399353			
alpha =				90.0			
beta =				90.0			
gamma =				120.0			
	Atom	X	Y	Z	X	Y	Z
1	Sm	2.0475	3.2620	7.1597	0.3386	0.3245	0.3127
2	Ce	1.9347	1.1170	0.7898	0.2222	0.1111	0.0345
3	Ce	0.0000	4.4680	0.7898	0.2222	0.4444	0.0345
4	Ce	-1.9347	7.8190	0.7898	0.2222	0.7778	0.0345
5	Ce	5.8041	1.1170	0.7898	0.5556	0.1111	0.0345
6	Ce	3.8694	4.4680	0.7898	0.5556	0.4444	0.0345
7	Ce	1.9347	7.8190	0.7898	0.5556	0.7778	0.0345
8	Ce	9.6735	1.1170	0.7898	0.8889	0.1111	0.0345
9	Ce	7.7388	4.4680	0.7898	0.8889	0.4444	0.0345
10	Ce	5.8041	7.8190	0.7898	0.8889	0.7778	0.0345
11	Ce	0.0143	2.2449	3.9804	0.1129	0.2233	0.1738
12	Ce	-1.9211	5.5868	3.9633	0.1124	0.5557	0.1731
13	Ce	-3.8631	8.9375	3.9475	0.1117	0.8890	0.1724
14	Ce	3.8596	2.2414	3.9559	0.4440	0.2230	0.1728
15	Ce	1.9187	5.5718	3.9987	0.4424	0.5542	0.1746
16	Ce	0.0011	8.9363	3.9401	0.4446	0.8889	0.1721
17	Ce	7.7307	2.2294	3.9248	0.7768	0.2218	0.1714
18	Ce	5.8045	5.5784	3.9476	0.7775	0.5549	0.1724
19	Ce	3.8660	8.9394	3.9551	0.7777	0.8892	0.1727
20	Ce	0.0151	-0.0032	7.1013	0.0011	-0.0003	0.3101
21	Ce	-2.0312	3.2735	7.0614	-0.0122	0.3256	0.3084
22	Ce	-3.8623	6.6976	7.0875	0.0004	0.6662	0.3095
23	Ce	3.8568	0.0034	7.1024	0.3324	0.0003	0.3102
24	Ce	-0.0022	6.8329	7.1395	0.3397	0.6797	0.3118
25	Ce	7.7389	0.0141	7.1035	0.6674	0.0014	0.3102
26	Ce	5.8047	3.3552	7.0961	0.6669	0.3338	0.3099
27	Ce	3.8806	6.7034	7.1144	0.6677	0.6668	0.3107
28	O	0.0000	0.0000	0.0000	0.0000	0.0000	0.0000
29	O	-1.9347	3.3510	0.0000	-0.0000	0.3333	0.0000
30	O	-3.8694	6.7020	0.0000	-0.0000	0.6667	0.0000
31	O	3.8694	0.0000	0.0000	0.3333	0.0000	0.0000
32	O	1.9347	3.3510	0.0000	0.3333	0.3333	0.0000
33	O	0.0000	6.7020	0.0000	0.3333	0.6667	0.0000
34	O	7.7388	0.0000	0.0000	0.6667	0.0000	0.0000
35	O	5.8041	3.3510	0.0000	0.6667	0.3333	0.0000
36	O	3.8694	6.7020	0.0000	0.6667	0.6667	0.0000
37	O	-0.0000	2.2340	1.5798	0.1111	0.2222	0.0690
38	O	-1.9347	5.5850	1.5798	0.1111	0.5556	0.0690
39	O	-3.8694	8.9360	1.5798	0.1111	0.8889	0.0690
40	O	3.8694	2.2340	1.5798	0.4444	0.2222	0.0690

41	O	1.9347	5.5850	1.5798	0.4444	0.5556	0.0690
42	O	-0.0000	8.9360	1.5798	0.4444	0.8889	0.0690
43	O	7.7388	2.2340	1.5798	0.7778	0.2222	0.0690
44	O	5.8041	5.5850	1.5798	0.7778	0.5556	0.0690
45	O	3.8694	8.9360	1.5798	0.7778	0.8889	0.0690
46	O	1.9347	1.1170	3.1593	0.2222	0.1111	0.1380
47	O	0.0000	4.4680	3.1593	0.2222	0.4444	0.1380
48	O	-1.9347	7.8190	3.1593	0.2222	0.7778	0.1380
49	O	5.8041	1.1170	3.1593	0.5556	0.1111	0.1380
50	O	3.8694	4.4680	3.1593	0.5556	0.4444	0.1380
51	O	1.9347	7.8190	3.1593	0.5556	0.7778	0.1380
52	O	9.6735	1.1170	3.1593	0.8889	0.1111	0.1380
53	O	7.7388	4.4680	3.1593	0.8889	0.4444	0.1380
54	O	5.8041	7.8190	3.1593	0.8889	0.7778	0.1380
55	O	0.0124	0.0278	4.7631	0.0024	0.0028	0.2080
56	O	-1.9555	3.3464	4.7474	-0.0020	0.3329	0.2073
57	O	-3.8304	6.6826	4.7560	0.0024	0.6647	0.2077
58	O	-1.9397	10.0495	4.7526	0.3327	0.9997	0.2076
59	O	1.9511	3.3546	4.7469	0.3349	0.3337	0.2073
60	O	0.0004	6.7289	4.7505	0.3347	0.6693	0.2075
61	O	7.7412	-0.0081	4.7497	0.6665	-0.0008	0.2074
62	O	5.7989	3.3596	4.7476	0.6666	0.3342	0.2073
63	O	3.8314	6.6763	4.7936	0.6621	0.6641	0.2093
64	O	-0.0311	2.2836	6.3466	0.1109	0.2272	0.2772
65	O	-1.8573	5.5597	6.3417	0.1165	0.5530	0.2770
66	O	-3.8795	8.8965	6.2643	0.1083	0.8850	0.2736
67	O	3.9021	2.2060	6.3081	0.4459	0.2194	0.2755
68	O	1.8103	5.5778	6.4295	0.4334	0.5548	0.2808
69	O	-0.0068	8.9546	6.2997	0.4448	0.8907	0.2751
70	O	7.7169	2.2200	6.2632	0.7752	0.2208	0.2735
71	O	5.8491	5.6199	6.2594	0.7834	0.5590	0.2734
72	O	3.9088	8.9480	6.2783	0.7818	0.8901	0.2742
73	O	1.9411	0.9724	7.8789	0.2156	0.0967	0.3441
74	O	-0.2587	4.3810	8.3012	0.1956	0.4358	0.3625
75	O	-1.9468	7.8444	7.8693	0.2224	0.7803	0.3437
76	O	5.8196	1.1211	7.8956	0.5571	0.1115	0.3448
77	O	4.0255	4.5319	7.8334	0.5722	0.4508	0.3421
78	O	1.9851	7.9215	7.8378	0.5650	0.7880	0.3423
79	O	9.6308	1.1231	7.8836	0.8855	0.1117	0.3443
80	O	7.7400	4.4438	7.8563	0.8878	0.4420	0.3431
81	O	5.8129	7.8094	7.8861	0.8892	0.7768	0.3444
82	C	0.6184	4.8261	9.2056	0.2933	0.4801	0.4020
83	O	1.5691	4.0450	9.5242	0.3364	0.4024	0.4159
84	O	0.4964	6.0484	9.5470	0.3436	0.6017	0.4169

/db/jmorales/CoNi-alloy/Profiles/CoNi-Ce_3SmO_7/O-coverage/TS-O-coverage/TS-CoNi-Ce_3SmO_7-3O

a =			13.9033384264				
b =			13.9033384264				
c =			21.9516692133				
alpha =			90.0				
beta =			90.0				
gamma =			90.0				

	Atom	X	Y	Z	X	Y	Z
1	Ni	0.0000	0.0000	0.0000	0.0000	0.0000	0.0000
2	Ni	0.0000	3.4758	0.0000	0.0000	0.2500	0.0000
3	Ni	3.4758	0.0000	0.0000	0.2500	0.0000	0.0000
4	Ni	3.4758	3.4758	0.0000	0.2500	0.2500	0.0000
5	Ni	0.0000	6.9517	0.0000	0.0000	0.5000	0.0000
6	Ni	0.0000	10.4275	0.0000	0.0000	0.7500	0.0000
7	Ni	3.4758	6.9517	0.0000	0.2500	0.5000	0.0000
8	Ni	3.4758	10.4275	0.0000	0.2500	0.7500	0.0000
9	Ni	6.9517	0.0000	0.0000	0.5000	0.0000	0.0000
10	Ni	6.9517	3.4758	0.0000	0.5000	0.2500	0.0000
11	Ni	10.4275	0.0000	0.0000	0.7500	0.0000	0.0000
12	Ni	10.4275	3.4758	0.0000	0.7500	0.2500	0.0000
13	Ni	6.9517	6.9517	0.0000	0.5000	0.5000	0.0000
14	Ni	6.9517	10.4275	0.0000	0.5000	0.7500	0.0000
15	Ni	10.4275	6.9517	0.0000	0.7500	0.5000	0.0000
16	Ni	10.4275	10.4275	0.0000	0.7500	0.7500	0.0000
17	Ni	0.0000	1.7379	1.7379	0.0000	0.1250	0.0792
18	Ni	0.0000	5.2138	1.7379	0.0000	0.3750	0.0792
19	Ni	3.4758	1.7379	1.7379	0.2500	0.1250	0.0792
20	Ni	3.4758	5.2138	1.7379	0.2500	0.3750	0.0792
21	Ni	0.0000	8.6896	1.7379	0.0000	0.6250	0.0792
22	Ni	0.0000	12.1654	1.7379	0.0000	0.8750	0.0792
23	Ni	3.4758	8.6896	1.7379	0.2500	0.6250	0.0792
24	Ni	3.4758	12.1654	1.7379	0.2500	0.8750	0.0792
25	Ni	6.9517	1.7379	1.7379	0.5000	0.1250	0.0792
26	Ni	6.9517	5.2138	1.7379	0.5000	0.3750	0.0792
27	Ni	10.4275	1.7379	1.7379	0.7500	0.1250	0.0792
28	Ni	10.4275	5.2138	1.7379	0.7500	0.3750	0.0792
29	Ni	6.9517	8.6896	1.7379	0.5000	0.6250	0.0792
30	Ni	6.9517	12.1654	1.7379	0.5000	0.8750	0.0792
31	Ni	10.4275	8.6896	1.7379	0.7500	0.6250	0.0792
32	Ni	10.4275	12.1654	1.7379	0.7500	0.8750	0.0792
33	Ni	13.9018	13.8996	3.4456	0.9999	0.9997	0.1570
34	Ni	0.0189	3.4573	3.5276	0.0014	0.2487	0.1607
35	Ni	3.4655	0.0108	3.5134	0.2493	0.0008	0.1601
36	Ni	3.4697	3.4441	3.4271	0.2496	0.2477	0.1561
37	Ni	13.9004	6.9532	3.5087	0.9998	0.5001	0.1598
38	Ni	13.8836	10.4473	3.5054	0.9986	0.7514	0.1597
39	Ni	3.4774	6.9476	3.4877	0.2501	0.4997	0.1589

40	Ni	3.4802	10.3844	3.5100	0.2503	0.7469	0.1599
41	Ni	6.9510	0.0122	3.4943	0.5000	0.0009	0.1592
42	Ni	6.9458	3.4666	3.5434	0.4996	0.2493	0.1614
43	Ni	10.4473	0.0052	3.4539	0.7514	0.0004	0.1573
44	Ni	10.4342	3.4653	3.5081	0.7505	0.2492	0.1598
45	Ni	6.9648	6.9622	3.4932	0.5009	0.5008	0.1591
46	Ni	6.9667	10.4422	3.4832	0.5011	0.7511	0.1587
47	Ni	10.4383	6.9375	3.4873	0.7508	0.4990	0.1589
48	Ni	10.4277	10.4467	3.5051	0.7500	0.7514	0.1597
49	Ni	0.0213	1.7558	5.2653	0.0015	0.1263	0.2399
50	Ni	0.0070	5.2113	5.2140	0.0005	0.3748	0.2375
51	Ni	3.4604	1.7111	5.2309	0.2489	0.1231	0.2383
52	Ni	3.4603	5.2053	5.0549	0.2489	0.3744	0.2303
53	Ni	13.8661	8.7165	5.1947	0.9973	0.6269	0.2366
54	Ni	0.0140	12.1324	5.2600	0.0010	0.8726	0.2396
55	Ni	3.4377	8.6482	5.2399	0.2473	0.6220	0.2387
56	Ni	3.4357	12.1156	5.1890	0.2471	0.8714	0.2364
57	Ni	6.9644	1.7460	5.3283	0.5009	0.1256	0.2427
58	Ni	6.9969	5.1779	5.3451	0.5033	0.3724	0.2435
59	Ni	10.4692	1.7707	5.2474	0.7530	0.1274	0.2390
60	Ni	10.4196	5.2081	5.2070	0.7494	0.3746	0.2372
61	Ni	6.9858	8.7129	5.2204	0.5025	0.6267	0.2378
62	Ni	7.0005	12.1535	5.2113	0.5035	0.8741	0.2374
63	Ni	10.4444	8.6735	5.1870	0.7512	0.6238	0.2363
64	Ni	10.3875	12.1761	5.3516	0.7471	0.8758	0.2438
65	Co	1.7379	1.7379	0.0000	0.1250	0.1250	0.0000
66	Co	1.7379	5.2138	0.0000	0.1250	0.3750	0.0000
67	Co	5.2138	1.7379	0.0000	0.3750	0.1250	0.0000
68	Co	5.2138	5.2138	0.0000	0.3750	0.3750	0.0000
69	Co	1.7379	8.6896	0.0000	0.1250	0.6250	0.0000
70	Co	1.7379	12.1654	0.0000	0.1250	0.8750	0.0000
71	Co	5.2138	8.6896	0.0000	0.3750	0.6250	0.0000
72	Co	5.2138	12.1654	0.0000	0.3750	0.8750	0.0000
73	Co	8.6896	1.7379	0.0000	0.6250	0.1250	0.0000
74	Co	8.6896	5.2138	0.0000	0.6250	0.3750	0.0000
75	Co	12.1654	1.7379	0.0000	0.8750	0.1250	0.0000
76	Co	12.1654	5.2138	0.0000	0.8750	0.3750	0.0000
77	Co	8.6896	8.6896	0.0000	0.6250	0.6250	0.0000
78	Co	8.6896	12.1654	0.0000	0.6250	0.8750	0.0000
79	Co	12.1654	8.6896	0.0000	0.8750	0.6250	0.0000
80	Co	12.1654	12.1654	0.0000	0.8750	0.8750	0.0000
81	Co	1.7379	0.0000	1.7379	0.1250	0.0000	0.0792
82	Co	1.7379	3.4758	1.7379	0.1250	0.2500	0.0792
83	Co	5.2138	0.0000	1.7379	0.3750	0.0000	0.0792
84	Co	5.2138	3.4758	1.7379	0.3750	0.2500	0.0792
85	Co	1.7379	6.9517	1.7379	0.1250	0.5000	0.0792
86	Co	1.7379	10.4275	1.7379	0.1250	0.7500	0.0792
87	Co	5.2138	6.9517	1.7379	0.3750	0.5000	0.0792
88	Co	5.2138	10.4275	1.7379	0.3750	0.7500	0.0792

89	Co	8.6896	0.0000	1.7379	0.6250	0.0000	0.0792
90	Co	8.6896	3.4758	1.7379	0.6250	0.2500	0.0792
91	Co	12.1654	0.0000	1.7379	0.8750	0.0000	0.0792
92	Co	12.1654	3.4758	1.7379	0.8750	0.2500	0.0792
93	Co	8.6896	6.9517	1.7379	0.6250	0.5000	0.0792
94	Co	8.6896	10.4275	1.7379	0.6250	0.7500	0.0792
95	Co	12.1654	6.9517	1.7379	0.8750	0.5000	0.0792
96	Co	12.1654	10.4275	1.7379	0.8750	0.7500	0.0792
97	Co	1.7370	1.7436	3.5569	0.1249	0.1254	0.1620
98	Co	1.7016	5.2310	3.5183	0.1224	0.3762	0.1603
99	Co	5.2209	1.7361	3.5767	0.3755	0.1249	0.1629
100	Co	5.2540	5.1794	3.5065	0.3779	0.3725	0.1597
101	Co	1.7191	8.6736	3.5269	0.1236	0.6238	0.1607
102	Co	1.7376	12.1617	3.5226	0.1250	0.8747	0.1605
103	Co	5.2040	8.6764	3.5512	0.3743	0.6241	0.1618
104	Co	5.2092	12.1439	3.5517	0.3747	0.8735	0.1618
105	Co	8.6882	1.7294	3.5576	0.6249	0.1244	0.1621
106	Co	8.6719	5.1971	3.5638	0.6237	0.3738	0.1623
107	Co	12.1851	1.7374	3.5478	0.8764	0.1250	0.1616
108	Co	12.1713	5.2043	3.5544	0.8754	0.3743	0.1619
109	Co	8.6933	8.6749	3.5153	0.6253	0.6239	0.1601
110	Co	8.7065	12.1978	3.4857	0.6262	0.8773	0.1588
111	Co	12.1582	8.6923	3.5386	0.8745	0.6252	0.1612
112	Co	12.1459	12.1796	3.5813	0.8736	0.8760	0.1631
113	Co	1.7549	13.8810	5.2697	0.1262	0.9984	0.2401
114	Co	1.7855	3.4978	5.2831	0.1284	0.2516	0.2407
115	Co	5.1488	13.8689	5.2476	0.3703	0.9975	0.2391
116	Co	5.1447	3.4472	5.2616	0.3700	0.2479	0.2397
117	Co	1.6968	6.9624	5.3251	0.1220	0.5008	0.2426
118	Co	1.7294	10.4280	5.1774	0.1244	0.7500	0.2359
119	Co	5.2647	6.8898	5.1655	0.3787	0.4955	0.2353
120	Co	5.1897	10.3911	5.2174	0.3733	0.7474	0.2377
121	Co	8.7035	0.0727	5.3477	0.6260	0.0052	0.2436
122	Co	8.7074	3.4678	5.2117	0.6263	0.2494	0.2374
123	Co	12.1970	13.8893	5.3684	0.8773	0.9990	0.2446
124	Co	12.1848	3.4951	5.2122	0.8764	0.2514	0.2374
125	Co	8.7235	6.9740	5.1801	0.6274	0.5016	0.2360
126	Co	8.7518	10.3753	5.2002	0.6295	0.7462	0.2369
127	Co	12.1707	6.9371	5.2172	0.8754	0.4990	0.2377
128	Co	12.1577	10.4252	5.2135	0.8744	0.7498	0.2375
129	O	5.9428	6.0316	6.7646	0.4274	0.4338	0.3082
130	O	2.6675	7.6974	6.8181	0.1919	0.5536	0.3106
131	O	4.8436	7.6967	8.7782	0.3484	0.5536	0.3999
132	O	6.0397	9.7150	6.8151	0.4344	0.6987	0.3105
133	O	7.8551	9.6249	9.0052	0.5650	0.6923	0.4102
134	O	4.9687	11.2273	8.9939	0.3574	0.8075	0.4097
135	O	8.6287	11.7528	6.4055	0.6206	0.8453	0.2918
136	Ce	3.8608	9.6943	8.0662	0.2777	0.6973	0.3675
137	Ce	3.8551	5.9386	7.7227	0.2773	0.4271	0.3518

138	Ce	7.0900	7.8856	8.0710	0.5099	0.5672	0.3677
139	Sm	6.9824	11.4511	8.2169	0.5022	0.8236	0.3743
140	C	7.4686	0.5865	6.8294	0.5372	0.0422	0.3111
141	O	6.1662	13.2184	6.6268	0.4435	0.9507	0.3019
142	O	7.7306	0.5947	8.0136	0.5560	0.0428	0.3651
143	O	10.5095	0.1036	6.2232	0.7559	0.0075	0.2835
144	O	0.0735	13.8912	6.1940	0.0053	0.9991	0.2822
145	O	3.4970	3.7412	6.3730	0.2515	0.2691	0.2903

/db/jmorales/CoNi-alloy/Profiles/Co-CoNi(001)/TS

a =			6.951669213				
b =			6.951669213				
c =			21.951669213				
alpha =			90.0				
beta =			90.0				
gamma =			90.0				
	Atom	X	Y	Z	X	Y	Z
1	Ni	0.0000	0.0000	0.0000	0.0000	0.0000	0.0000
2	Ni	1.7379	1.7379	0.0000	0.2500	0.2500	0.0000
3	Ni	0.0000	3.4758	0.0000	0.0000	0.5000	0.0000
4	Ni	1.7379	5.2138	0.0000	0.2500	0.7500	0.0000
5	Ni	3.4758	0.0000	0.0000	0.5000	0.0000	0.0000
6	Ni	5.2138	1.7379	0.0000	0.7500	0.2500	0.0000
7	Ni	3.4758	3.4758	0.0000	0.5000	0.5000	0.0000
8	Ni	5.2138	5.2138	0.0000	0.7500	0.7500	0.0000
9	Ni	0.0103	0.0052	3.4978	0.0015	0.0007	0.1593
10	Ni	1.7672	1.7583	3.5531	0.2542	0.2529	0.1619
11	Ni	6.9492	3.4607	3.5325	0.9996	0.4978	0.1609
12	Ni	1.7486	5.1966	3.5215	0.2515	0.7475	0.1604
13	Ni	3.4838	6.9488	3.5121	0.5011	0.9996	0.1600
14	Ni	5.2132	1.7382	3.5651	0.7499	0.2500	0.1624
15	Ni	3.4979	3.4775	3.5798	0.5032	0.5002	0.1631
16	Ni	5.2198	5.2023	3.5410	0.7509	0.7484	0.1613
17	Co	1.7379	0.0000	1.7379	0.2500	0.0000	0.0792
18	Co	0.0000	1.7379	1.7379	0.0000	0.2500	0.0792
19	Co	1.7379	3.4758	1.7379	0.2500	0.5000	0.0792
20	Co	0.0000	5.2138	1.7379	0.0000	0.7500	0.0792
21	Co	5.2138	0.0000	1.7379	0.7500	0.0000	0.0792
22	Co	3.4758	1.7379	1.7379	0.5000	0.2500	0.0792
23	Co	5.2138	3.4758	1.7379	0.7500	0.5000	0.0792
24	Co	3.4758	5.2138	1.7379	0.5000	0.7500	0.0792
25	Co	1.6815	0.0029	5.2413	0.2419	0.0004	0.2388
26	Co	0.0473	1.7346	5.2821	0.0068	0.2495	0.2406
27	Co	1.7405	3.5325	5.3472	0.2504	0.5081	0.2436
28	Co	0.0100	5.1752	5.2586	0.0014	0.7445	0.2396
29	Co	5.3186	6.9120	5.2650	0.7651	0.9943	0.2398
30	Co	3.4860	1.5464	5.4057	0.5015	0.2224	0.2463
31	Co	5.2699	3.4947	5.3906	0.7581	0.5027	0.2456
32	Co	3.4638	5.3380	5.2979	0.4983	0.7679	0.2413
33	C	2.7919	3.9725	6.8827	0.4016	0.5714	0.3135
34	O	2.8866	4.5248	7.9307	0.4152	0.6509	0.3613
35	O	4.0956	2.7891	6.6451	0.5891	0.4012	0.3027

/db/jmorales/CoNi-alloy/Profiles/CoNi-Ce_3SmO_7/CO-O

a =	13.9033384264
b =	13.9033384264
c =	21.9516692133
alpha =	90.0
beta =	90.0
gamma =	90.0

	Atom	X	Y	Z	X	Y	Z
1	Ni	0.0000	0.0000	0.0000	0.0000	0.0000	0.0000
2	Ni	0.0000	3.4758	0.0000	0.0000	0.2500	0.0000
3	Ni	3.4758	0.0000	0.0000	0.2500	0.0000	0.0000
4	Ni	3.4758	3.4758	0.0000	0.2500	0.2500	0.0000
5	Ni	0.0000	6.9517	0.0000	0.0000	0.5000	0.0000
6	Ni	0.0000	10.4275	0.0000	0.0000	0.7500	0.0000
7	Ni	3.4758	6.9517	0.0000	0.2500	0.5000	0.0000
8	Ni	3.4758	10.4275	0.0000	0.2500	0.7500	0.0000
9	Ni	6.9517	0.0000	0.0000	0.5000	0.0000	0.0000
10	Ni	6.9517	3.4758	0.0000	0.5000	0.2500	0.0000
11	Ni	10.4275	0.0000	0.0000	0.7500	0.0000	0.0000
12	Ni	10.4275	3.4758	0.0000	0.7500	0.2500	0.0000
13	Ni	6.9517	6.9517	0.0000	0.5000	0.5000	0.0000
14	Ni	6.9517	10.4275	0.0000	0.5000	0.7500	0.0000
15	Ni	10.4275	6.9517	0.0000	0.7500	0.5000	0.0000
16	Ni	10.4275	10.4275	0.0000	0.7500	0.7500	0.0000
17	Ni	0.0000	1.7379	1.7379	0.0000	0.1250	0.0792
18	Ni	0.0000	5.2138	1.7379	0.0000	0.3750	0.0792
19	Ni	3.4758	1.7379	1.7379	0.2500	0.1250	0.0792
20	Ni	3.4758	5.2138	1.7379	0.2500	0.3750	0.0792
21	Ni	0.0000	8.6896	1.7379	0.0000	0.6250	0.0792
22	Ni	0.0000	12.1654	1.7379	0.0000	0.8750	0.0792
23	Ni	3.4758	8.6896	1.7379	0.2500	0.6250	0.0792
24	Ni	3.4758	12.1654	1.7379	0.2500	0.8750	0.0792
25	Ni	6.9517	1.7379	1.7379	0.5000	0.1250	0.0792
26	Ni	6.9517	5.2138	1.7379	0.5000	0.3750	0.0792
27	Ni	10.4275	1.7379	1.7379	0.7500	0.1250	0.0792
28	Ni	10.4275	5.2138	1.7379	0.7500	0.3750	0.0792
29	Ni	6.9517	8.6896	1.7379	0.5000	0.6250	0.0792
30	Ni	6.9517	12.1654	1.7379	0.5000	0.8750	0.0792
31	Ni	10.4275	8.6896	1.7379	0.7500	0.6250	0.0792
32	Ni	10.4275	12.1654	1.7379	0.7500	0.8750	0.0792
33	Ni	0.0062	13.8982	3.5074	0.0004	0.9996	0.1598
34	Ni	0.0017	3.4741	3.5018	0.0001	0.2499	0.1595
35	Ni	3.4749	13.8992	3.4401	0.2499	0.9997	0.1567
36	Ni	3.4619	3.4329	3.4830	0.2490	0.2469	0.1587
37	Ni	0.0045	6.9577	3.5149	0.0003	0.5004	0.1601
38	Ni	13.8906	10.4332	3.4924	0.9991	0.7504	0.1591
39	Ni	3.4787	6.9550	3.4960	0.2502	0.5002	0.1593
40	Ni	3.4964	10.4232	3.5415	0.2515	0.7497	0.1613

41	Ni	6.9414	0.0140	3.4590	0.4993	0.0010	0.1576
42	Ni	6.9580	3.4792	3.5256	0.5005	0.2502	0.1606
43	Ni	10.4293	0.0042	3.5006	0.7501	0.0003	0.1595
44	Ni	10.4283	3.4746	3.5056	0.7501	0.2499	0.1597
45	Ni	6.9625	6.9622	3.4940	0.5008	0.5008	0.1592
46	Ni	6.9671	10.4461	3.4836	0.5011	0.7513	0.1587
47	Ni	10.4362	6.9421	3.4891	0.7506	0.4993	0.1589
48	Ni	10.4264	10.4360	3.4976	0.7499	0.7506	0.1593
49	Ni	0.0033	1.7389	5.2079	0.0002	0.1251	0.2372
50	Ni	0.0039	5.2122	5.2150	0.0003	0.3749	0.2376
51	Ni	3.4719	1.7481	5.2586	0.2497	0.1257	0.2396
52	Ni	3.4538	5.1654	5.0868	0.2484	0.3715	0.2317
53	Ni	13.8763	8.7183	5.1930	0.9981	0.6271	0.2366
54	Ni	0.0004	12.1594	5.2091	0.0000	0.8746	0.2373
55	Ni	3.4529	8.6764	5.2435	0.2484	0.6241	0.2389
56	Ni	3.4871	12.1363	5.2781	0.2508	0.8729	0.2404
57	Ni	6.9370	1.7403	5.2483	0.4989	0.1252	0.2391
58	Ni	6.9785	5.1483	5.3608	0.5019	0.3703	0.2442
59	Ni	10.4420	1.7330	5.2049	0.7510	0.1246	0.2371
60	Ni	10.4259	5.2154	5.2124	0.7499	0.3751	0.2375
61	Ni	6.9968	8.7109	5.2135	0.5032	0.6265	0.2375
62	Ni	6.9750	12.2393	5.2038	0.5017	0.8803	0.2371
63	Ni	10.4282	8.6827	5.1951	0.7500	0.6245	0.2367
64	Ni	10.4204	12.2006	5.2799	0.7495	0.8775	0.2405
65	Co	1.7379	1.7379	0.0000	0.1250	0.1250	0.0000
66	Co	1.7379	5.2138	0.0000	0.1250	0.3750	0.0000
67	Co	5.2138	1.7379	0.0000	0.3750	0.1250	0.0000
68	Co	5.2138	5.2138	0.0000	0.3750	0.3750	0.0000
69	Co	1.7379	8.6896	0.0000	0.1250	0.6250	0.0000
70	Co	1.7379	12.1654	0.0000	0.1250	0.8750	0.0000
71	Co	5.2138	8.6896	0.0000	0.3750	0.6250	0.0000
72	Co	5.2138	12.1654	0.0000	0.3750	0.8750	0.0000
73	Co	8.6896	1.7379	0.0000	0.6250	0.1250	0.0000
74	Co	8.6896	5.2138	0.0000	0.6250	0.3750	0.0000
75	Co	12.1654	1.7379	0.0000	0.8750	0.1250	0.0000
76	Co	12.1654	5.2138	0.0000	0.8750	0.3750	0.0000
77	Co	8.6896	8.6896	0.0000	0.6250	0.6250	0.0000
78	Co	8.6896	12.1654	0.0000	0.6250	0.8750	0.0000
79	Co	12.1654	8.6896	0.0000	0.8750	0.6250	0.0000
80	Co	12.1654	12.1654	0.0000	0.8750	0.8750	0.0000
81	Co	1.7379	0.0000	1.7379	0.1250	0.0000	0.0792
82	Co	1.7379	3.4758	1.7379	0.1250	0.2500	0.0792
83	Co	5.2138	0.0000	1.7379	0.3750	0.0000	0.0792
84	Co	5.2138	3.4758	1.7379	0.3750	0.2500	0.0792
85	Co	1.7379	6.9517	1.7379	0.1250	0.5000	0.0792
86	Co	1.7379	10.4275	1.7379	0.1250	0.7500	0.0792
87	Co	5.2138	6.9517	1.7379	0.3750	0.5000	0.0792
88	Co	5.2138	10.4275	1.7379	0.3750	0.7500	0.0792
89	Co	8.6896	0.0000	1.7379	0.6250	0.0000	0.0792

90	Co	8.6896	3.4758	1.7379	0.6250	0.2500	0.0792
91	Co	12.1654	0.0000	1.7379	0.8750	0.0000	0.0792
92	Co	12.1654	3.4758	1.7379	0.8750	0.2500	0.0792
93	Co	8.6896	6.9517	1.7379	0.6250	0.5000	0.0792
94	Co	8.6896	10.4275	1.7379	0.6250	0.7500	0.0792
95	Co	12.1654	6.9517	1.7379	0.8750	0.5000	0.0792
96	Co	12.1654	10.4275	1.7379	0.8750	0.7500	0.0792
97	Co	1.7327	1.7275	3.5516	0.1246	0.1242	0.1618
98	Co	1.7076	5.2354	3.5263	0.1228	0.3766	0.1606
99	Co	5.2082	1.7283	3.5339	0.3746	0.1243	0.1610
100	Co	5.2445	5.1847	3.5187	0.3772	0.3729	0.1603
101	Co	1.7334	8.6843	3.5342	0.1247	0.6246	0.1610
102	Co	1.7482	12.1691	3.5447	0.1257	0.8753	0.1615
103	Co	5.2051	8.6821	3.5610	0.3744	0.6245	0.1622
104	Co	5.2175	12.1641	3.5654	0.3753	0.8749	0.1624
105	Co	8.6863	1.7290	3.5597	0.6248	0.1244	0.1622
106	Co	8.6689	5.1995	3.5670	0.6235	0.3740	0.1625
107	Co	12.1718	1.7409	3.5537	0.8755	0.1252	0.1619
108	Co	12.1688	5.2098	3.5569	0.8752	0.3747	0.1620
109	Co	8.6910	8.6858	3.5225	0.6251	0.6247	0.1605
110	Co	8.7048	12.1930	3.4685	0.6261	0.8770	0.1580
111	Co	12.1579	8.6925	3.5434	0.8745	0.6252	0.1614
112	Co	12.1483	12.1691	3.5727	0.8738	0.8753	0.1628
113	Co	1.7459	13.8907	5.2751	0.1256	0.9991	0.2403
114	Co	1.7331	3.4892	5.2118	0.1247	0.2510	0.2374
115	Co	5.2113	0.0078	5.3569	0.3748	0.0006	0.2440
116	Co	5.1708	3.4488	5.1867	0.3719	0.2481	0.2363
117	Co	1.7179	6.9566	5.3278	0.1236	0.5004	0.2427
118	Co	1.7156	10.4535	5.1805	0.1234	0.7519	0.2360
119	Co	5.2593	6.8760	5.1720	0.3783	0.4946	0.2356
120	Co	5.2342	10.4277	5.2278	0.3765	0.7500	0.2382
121	Co	8.6599	0.0251	5.3025	0.6229	0.0018	0.2416
122	Co	8.7201	3.4521	5.2249	0.6272	0.2483	0.2380
123	Co	12.1754	0.0013	5.2076	0.8757	0.0001	0.2372
124	Co	12.1713	3.4759	5.2248	0.8754	0.2500	0.2380
125	Co	8.7205	6.9666	5.1717	0.6272	0.5011	0.2356
126	Co	8.7451	10.4224	5.2115	0.6290	0.7496	0.2374
127	Co	12.1670	6.9513	5.2171	0.8751	0.5000	0.2377
128	Co	12.1622	10.4294	5.2169	0.8748	0.7501	0.2377
129	O	5.9533	6.0470	6.7855	0.4282	0.4349	0.3091
130	O	2.6490	7.7822	6.8305	0.1905	0.5597	0.3112
131	O	4.8701	7.7744	8.7916	0.3503	0.5592	0.4005
132	O	6.0998	9.6662	6.8078	0.4387	0.6952	0.3101
133	O	7.9602	9.6229	8.9730	0.5725	0.6921	0.4088
134	O	5.0794	11.3013	8.9151	0.3653	0.8128	0.4061
135	O	8.7131	11.9653	6.3128	0.6267	0.8606	0.2876
136	Ce	3.9182	9.7824	8.0311	0.2818	0.7036	0.3659
137	Ce	3.8547	6.0805	7.7168	0.2772	0.4373	0.3515
138	Ce	7.1336	7.8704	8.0758	0.5131	0.5661	0.3679

139 Sm	7.0846	11.3929	8.1214	0.5096	0.8194	0.3700
140 C	6.8431	0.1577	6.3864	0.4922	0.0113	0.2909
141 O	6.9879	0.0251	7.6199	0.5026	0.0018	0.3471
142 O	3.4545	13.8983	6.1886	0.2485	0.9996	0.2819

/db/jmorales/CoNi-alloy/Profiles/Ni-CoNi(001)/CO_2

a =	6.951669213
b =	6.951669213
c =	21.951669213
alpha =	90.0
beta =	90.0
gamma =	90.0

	Atom	X	Y	Z	X	Y	Z
1	Co	0.0000	0.0000	0.0000	0.0000	0.0000	0.0000
2	Co	1.7379	1.7379	0.0000	0.2500	0.2500	0.0000
3	Co	0.0000	3.4758	0.0000	0.0000	0.5000	0.0000
4	Co	1.7379	5.2138	0.0000	0.2500	0.7500	0.0000
5	Co	3.4758	0.0000	0.0000	0.5000	0.0000	0.0000
6	Co	5.2138	1.7379	0.0000	0.7500	0.2500	0.0000
7	Co	3.4758	3.4758	0.0000	0.5000	0.5000	0.0000
8	Co	5.2138	5.2138	0.0000	0.7500	0.7500	0.0000
9	Co	6.9496	6.9509	3.5032	0.9997	0.9999	0.1596
10	Co	1.7435	1.7482	3.4758	0.2508	0.2515	0.1583
11	Co	6.9184	3.4722	3.5201	0.9952	0.4995	0.1604
12	Co	1.7401	5.2095	3.4758	0.2503	0.7494	0.1583
13	Co	3.4708	0.0121	3.5315	0.4993	0.0017	0.1609
14	Co	5.2123	1.7362	3.4758	0.7498	0.2498	0.1583
15	Co	3.4672	3.4637	3.5067	0.4988	0.4982	0.1597
16	Co	5.2024	5.2055	3.4758	0.7484	0.7488	0.1583
17	Ni	1.7379	0.0000	1.7379	0.2500	0.0000	0.0792
18	Ni	0.0000	1.7379	1.7379	0.0000	0.2500	0.0792
19	Ni	1.7379	3.4758	1.7379	0.2500	0.5000	0.0792
20	Ni	0.0000	5.2138	1.7379	0.0000	0.7500	0.0792
21	Ni	5.2138	0.0000	1.7379	0.7500	0.0000	0.0792
22	Ni	3.4758	1.7379	1.7379	0.5000	0.2500	0.0792
23	Ni	5.2138	3.4758	1.7379	0.7500	0.5000	0.0792
24	Ni	3.4758	5.2138	1.7379	0.5000	0.7500	0.0792
25	Ni	1.7345	6.9514	5.1703	0.2495	1.0000	0.2355
26	Ni	0.0019	1.7025	5.1562	0.0003	0.2449	0.2349
27	Ni	1.6729	3.4749	5.2160	0.2406	0.4999	0.2376
28	Ni	6.9401	5.2466	5.1554	0.9983	0.7547	0.2349
29	Ni	5.2085	6.9252	5.1684	0.7492	0.9962	0.2354
30	Ni	3.4877	1.7189	5.2302	0.5017	0.2473	0.2383
31	Ni	5.2400	3.4770	5.2474	0.7538	0.5002	0.2390
32	Ni	3.4515	5.1621	5.2258	0.4965	0.7426	0.2381
33	C	3.0285	3.8311	6.6646	0.4357	0.5511	0.3036
34	O	2.0561	3.9811	7.4250	0.2958	0.5727	0.3382
35	O	4.0749	3.0344	6.8277	0.5862	0.4365	0.3110

/db/jmorales/CoNi-alloy/Profiles/Sm(surf)$Ce_{26}O_{53}$/CO2

a =				11.6082000732			
b =				11.608200073110298			
c =				22.898399353			
alpha =				90.0			
beta =				90.0			
gamma =				120.0			
	Atom	X	Y	Z	X	Y	Z
1	Sm	2.0168	3.0873	7.1856	0.3273	0.3071	0.3138
2	Ce	1.9347	1.1170	0.7898	0.2222	0.1111	0.0345
3	Ce	0.0000	4.4680	0.7898	0.2222	0.4444	0.0345
4	Ce	-1.9347	7.8190	0.7898	0.2222	0.7778	0.0345
5	Ce	5.8041	1.1170	0.7898	0.5556	0.1111	0.0345
6	Ce	3.8694	4.4680	0.7898	0.5556	0.4444	0.0345
7	Ce	1.9347	7.8190	0.7898	0.5556	0.7778	0.0345
8	Ce	9.6735	1.1170	0.7898	0.8889	0.1111	0.0345
9	Ce	7.7388	4.4680	0.7898	0.8889	0.4444	0.0345
10	Ce	5.8041	7.8190	0.7898	0.8889	0.7778	0.0345
11	Ce	-0.0055	2.2422	3.9936	0.1110	0.2230	0.1744
12	Ce	-1.9121	5.5719	4.0005	0.1124	0.5543	0.1747
13	Ce	-3.8678	8.9339	3.9445	0.1111	0.8887	0.1723
14	Ce	3.8595	2.2370	3.9495	0.4437	0.2225	0.1725
15	Ce	1.9432	5.5810	3.8657	0.4450	0.5552	0.1688
16	Ce	-0.0024	8.9459	3.9303	0.4447	0.8899	0.1716
17	Ce	7.7343	2.2396	3.9256	0.7777	0.2228	0.1714
18	Ce	5.8036	5.5770	3.9845	0.7773	0.5548	0.1740
19	Ce	3.8630	8.9328	3.9900	0.7771	0.8886	0.1742
20	Ce	-0.0002	-0.0235	7.1036	-0.0012	-0.0023	0.3102
21	Ce	9.5506	3.2418	7.1430	0.9840	0.3225	0.3119
22	Ce	-3.8748	6.6912	7.1229	-0.0010	0.6656	0.3111
23	Ce	3.8241	0.0352	7.0820	0.3312	0.0035	0.3093
24	Ce	-0.1439	7.0218	7.2143	0.3368	0.6985	0.3151
25	Ce	7.7142	0.0379	7.0964	0.6664	0.0038	0.3099
26	Ce	5.7725	3.3465	7.1089	0.6637	0.3329	0.3105
27	Ce	3.9659	6.7292	7.3028	0.6763	0.6694	0.3189
28	O	0.0000	0.0000	0.0000	0.0000	0.0000	0.0000
29	O	-1.9347	3.3510	0.0000	-0.0000	0.3333	0.0000
30	O	-3.8694	6.7020	0.0000	-0.0000	0.6667	0.0000
31	O	3.8694	0.0000	0.0000	0.3333	0.0000	0.0000
32	O	1.9347	3.3510	0.0000	0.3333	0.3333	0.0000
33	O	0.0000	6.7020	0.0000	0.3333	0.6667	0.0000
34	O	7.7388	0.0000	0.0000	0.6667	0.0000	0.0000
35	O	5.8041	3.3510	0.0000	0.6667	0.3333	0.0000
36	O	3.8694	6.7020	0.0000	0.6667	0.6667	0.0000
37	O	-0.0000	2.2340	1.5798	0.1111	0.2222	0.0690
38	O	-1.9347	5.5850	1.5798	0.1111	0.5556	0.0690
39	O	-3.8694	8.9360	1.5798	0.1111	0.8889	0.0690
40	O	3.8694	2.2340	1.5798	0.4444	0.2222	0.0690

41	O	1.9347	5.5850	1.5798	0.4444	0.5556	0.0690
42	O	-0.0000	8.9360	1.5798	0.4444	0.8889	0.0690
43	O	7.7388	2.2340	1.5798	0.7778	0.2222	0.0690
44	O	5.8041	5.5850	1.5798	0.7778	0.5556	0.0690
45	O	3.8694	8.9360	1.5798	0.7778	0.8889	0.0690
46	O	1.9347	1.1170	3.1593	0.2222	0.1111	0.1380
47	O	0.0000	4.4680	3.1593	0.2222	0.4444	0.1380
48	O	-1.9347	7.8190	3.1593	0.2222	0.7778	0.1380
49	O	5.8041	1.1170	3.1593	0.5556	0.1111	0.1380
50	O	3.8694	4.4680	3.1593	0.5556	0.4444	0.1380
51	O	1.9347	7.8190	3.1593	0.5556	0.7778	0.1380
52	O	9.6735	1.1170	3.1593	0.8889	0.1111	0.1380
53	O	7.7388	4.4680	3.1593	0.8889	0.4444	0.1380
54	O	5.8041	7.8190	3.1593	0.8889	0.7778	0.1380
55	O	0.0023	0.0188	4.7400	0.0011	0.0019	0.2070
56	O	-1.9574	3.3688	4.7680	-0.0011	0.3351	0.2082
57	O	-3.7964	6.6594	4.7732	0.0042	0.6624	0.2085
58	O	3.8472	-0.0093	4.6930	0.3310	-0.0009	0.2049
59	O	1.9305	3.4892	4.8573	0.3398	0.3471	0.2121
60	O	0.1261	6.6642	4.8836	0.3423	0.6629	0.2133
61	O	1.9311	10.0418	4.7402	0.6658	0.9989	0.2070
62	O	5.7883	3.3645	4.7393	0.6660	0.3347	0.2070
63	O	3.7152	6.6111	4.8735	0.6489	0.6576	0.2128
64	O	-0.0622	2.2311	6.3146	0.1056	0.2219	0.2758
65	O	-1.8201	5.5481	6.4417	0.1192	0.5519	0.2813
66	O	-3.8994	8.9075	6.2347	0.1071	0.8861	0.2723
67	O	3.8873	2.1421	6.2436	0.4414	0.2131	0.2727
68	O	-0.0783	9.0409	6.2501	0.4429	0.8993	0.2729
69	O	7.7214	2.2277	6.2607	0.7760	0.2216	0.2734
70	O	5.8868	5.6219	6.2834	0.7867	0.5592	0.2744
71	O	3.9186	8.9896	6.2996	0.7847	0.8942	0.2751
72	O	1.9323	0.9476	7.9314	0.2136	0.0943	0.3464
73	O	-0.0553	3.4895	8.8225	0.1688	0.3471	0.3853
74	O	-2.0239	7.9512	7.9209	0.2211	0.7909	0.3459
75	O	5.8051	1.1318	7.9063	0.5564	0.1126	0.3453
76	O	3.9614	4.3612	7.8241	0.5582	0.4338	0.3417
77	O	1.8211	7.9451	7.8082	0.5520	0.7903	0.3410
78	O	9.5798	1.1198	7.9165	0.8810	0.1114	0.3457
79	O	7.7319	4.4176	7.8922	0.8858	0.4394	0.3447
80	O	5.9231	7.8568	7.9812	0.9010	0.7815	0.3485
81	C	0.1539	4.7572	8.7543	0.2499	0.4732	0.3823
82	O	1.1598	5.1554	7.9332	0.3563	0.5128	0.3465
83	O	-0.5661	5.6689	9.2627	0.2332	0.5639	0.4045

/db/jmorales/CoNi-alloy/Profiles/Sm(surf)$Ce_{26}O_{53}$/O

a =				11.6082000732			
b =				11.608200073110298			
c =				22.898399353			
alpha =				90.0			
beta =				90.0			
gamma =				120.0			
	Atom	X	Y	Z	X	Y	Z
1	Sm	1.9390	3.3524	7.1077	0.3338	0.3335	0.3104
2	Ce	1.9347	1.1170	0.7898	0.2222	0.1111	0.0345
3	Ce	0.0000	4.4680	0.7898	0.2222	0.4444	0.0345
4	Ce	-1.9347	7.8190	0.7898	0.2222	0.7778	0.0345
5	Ce	5.8041	1.1170	0.7898	0.5556	0.1111	0.0345
6	Ce	3.8694	4.4680	0.7898	0.5556	0.4444	0.0345
7	Ce	1.9347	7.8190	0.7898	0.5556	0.7778	0.0345
8	Ce	9.6735	1.1170	0.7898	0.8889	0.1111	0.0345
9	Ce	7.7388	4.4680	0.7898	0.8889	0.4444	0.0345
10	Ce	5.8041	7.8190	0.7898	0.8889	0.7778	0.0345
11	Ce	0.0053	2.2362	3.9668	0.1117	0.2224	0.1732
12	Ce	-1.9330	5.5846	3.9483	0.1112	0.5555	0.1724
13	Ce	-3.8683	8.9361	3.9492	0.1112	0.8889	0.1725
14	Ce	3.8664	2.2364	3.9667	0.4443	0.2225	0.1732
15	Ce	1.9360	5.5807	3.9671	0.4443	0.5551	0.1732
16	Ce	0.0011	8.9358	3.9571	0.4445	0.8889	0.1728
17	Ce	7.7398	2.2338	3.9563	0.7779	0.2222	0.1728
18	Ce	5.8057	5.5854	3.9483	0.7779	0.5556	0.1724
19	Ce	3.8705	8.9364	3.9570	0.7779	0.8889	0.1728
20	Ce	0.0098	-0.0011	7.1079	0.0008	-0.0001	0.3104
21	Ce	-1.9291	3.3566	7.1066	0.0008	0.3339	0.3104
22	Ce	-3.8648	6.7024	7.0927	0.0004	0.6667	0.3097
23	Ce	3.8666	-0.0009	7.1072	0.3330	-0.0001	0.3104
24	Ce	-0.0004	6.6968	7.1068	0.3330	0.6662	0.3104
25	Ce	7.7427	-0.0004	7.1161	0.6670	-0.0000	0.3108
26	Ce	5.8053	3.3574	7.1064	0.6671	0.3340	0.3103
27	Ce	3.8787	6.6982	7.1070	0.6673	0.6663	0.3104
28	O	0.0000	0.0000	0.0000	0.0000	0.0000	0.0000
29	O	-1.9347	3.3510	0.0000	-0.0000	0.3333	0.0000
30	O	-3.8694	6.7020	0.0000	-0.0000	0.6667	0.0000
31	O	3.8694	0.0000	0.0000	0.3333	0.0000	0.0000
32	O	1.9347	3.3510	0.0000	0.3333	0.3333	0.0000
33	O	0.0000	6.7020	0.0000	0.3333	0.6667	0.0000
34	O	7.7388	0.0000	0.0000	0.6667	0.0000	0.0000
35	O	5.8041	3.3510	0.0000	0.6667	0.3333	0.0000
36	O	3.8694	6.7020	0.0000	0.6667	0.6667	0.0000
37	O	-0.0000	2.2340	1.5798	0.1111	0.2222	0.0690
38	O	-1.9347	5.5850	1.5798	0.1111	0.5556	0.0690
39	O	-3.8694	8.9360	1.5798	0.1111	0.8889	0.0690
40	O	3.8694	2.2340	1.5798	0.4444	0.2222	0.0690

41	O	1.9347	5.5850	1.5798	0.4444	0.5556	0.0690
42	O	-0.0000	8.9360	1.5798	0.4444	0.8889	0.0690
43	O	7.7388	2.2340	1.5798	0.7778	0.2222	0.0690
44	O	5.8041	5.5850	1.5798	0.7778	0.5556	0.0690
45	O	3.8694	8.9360	1.5798	0.7778	0.8889	0.0690
46	O	1.9347	1.1170	3.1593	0.2222	0.1111	0.1380
47	O	0.0000	4.4680	3.1593	0.2222	0.4444	0.1380
48	O	-1.9347	7.8190	3.1593	0.2222	0.7778	0.1380
49	O	5.8041	1.1170	3.1593	0.5556	0.1111	0.1380
50	O	3.8694	4.4680	3.1593	0.5556	0.4444	0.1380
51	O	1.9347	7.8190	3.1593	0.5556	0.7778	0.1380
52	O	9.6735	1.1170	3.1593	0.8889	0.1111	0.1380
53	O	7.7388	4.4680	3.1593	0.8889	0.4444	0.1380
54	O	5.8041	7.8190	3.1593	0.8889	0.7778	0.1380
55	O	0.0028	-0.0035	4.7605	0.0001	-0.0004	0.2079
56	O	-1.9352	3.3534	4.7596	0.0001	0.3336	0.2079
57	O	-3.8670	6.7024	4.7479	0.0002	0.6667	0.2073
58	O	3.8700	-0.0039	4.7610	0.3332	-0.0004	0.2079
59	O	1.9364	3.3527	4.7584	0.3336	0.3335	0.2078
60	O	-0.0012	6.7016	4.7602	0.3332	0.6666	0.2079
61	O	7.7395	0.0001	4.7634	0.6667	0.0000	0.2080
62	O	5.8072	3.3543	4.7593	0.6671	0.3337	0.2078
63	O	3.8755	6.7028	4.7597	0.6672	0.6667	0.2079
64	O	-0.0303	2.2139	6.3095	0.1075	0.2202	0.2755
65	O	-1.9461	5.5932	6.2822	0.1105	0.5564	0.2744
66	O	-3.8667	8.9187	6.2831	0.1105	0.8872	0.2744
67	O	3.9067	2.2139	6.3085	0.4467	0.2202	0.2755
68	O	0.0042	8.9345	6.3034	0.4447	0.8887	0.2753
69	O	7.7412	2.2338	6.3026	0.7780	0.2222	0.2752
70	O	5.8244	5.5955	6.2823	0.7800	0.5566	0.2744
71	O	3.8716	8.9362	6.3026	0.7780	0.8889	0.2752
72	O	1.9393	1.0219	7.9038	0.2179	0.1017	0.3452
73	O	-0.0790	4.5152	7.9022	0.2178	0.4491	0.3451
74	O	-1.9365	7.8160	7.8935	0.2219	0.7775	0.3447
75	O	5.8194	1.1087	7.9048	0.5565	0.1103	0.3452
76	O	3.9590	4.5135	7.9023	0.5655	0.4490	0.3451
77	O	1.9406	7.8330	7.9065	0.5568	0.7792	0.3453
78	O	9.6656	1.1097	7.9059	0.8878	0.1104	0.3453
79	O	7.7436	4.4747	7.8925	0.8896	0.4451	0.3447
80	O	5.8136	7.8178	7.8939	0.8896	0.7777	0.3447
81	O	1.9396	5.6261	6.3106	0.4469	0.5596	0.2756

/db/jmorales/CoNi-alloy/Profiles/Ni-Ce_3SmO_7/O-coverage/M-CO2-O-coverage/M-CO_2-Ni-Ce_3SmO_7-2O

a =			13.9019837372			
b =			13.9019837372			
c =			21.9509918687			
alpha =			90.0			
beta =			90.0			
gamma =			90.0			
Atom	X	Y	Z	X	Y	Z
1 Ni	0.0000	0.0000	0.0000	0.0000	0.0000	0.0000
2 Ni	1.7377	1.7377	0.0000	0.1250	0.1250	0.0000
3 Ni	0.0000	3.4755	0.0000	0.0000	0.2500	0.0000
4 Ni	1.7377	5.2132	0.0000	0.1250	0.3750	0.0000
5 Ni	3.4755	0.0000	0.0000	0.2500	0.0000	0.0000
6 Ni	5.2132	1.7377	0.0000	0.3750	0.1250	0.0000
7 Ni	3.4755	3.4755	0.0000	0.2500	0.2500	0.0000
8 Ni	5.2132	5.2132	0.0000	0.3750	0.3750	0.0000
9 Ni	0.0000	6.9510	0.0000	0.0000	0.5000	0.0000
10 Ni	1.7377	8.6887	0.0000	0.1250	0.6250	0.0000
11 Ni	0.0000	10.4265	0.0000	0.0000	0.7500	0.0000
12 Ni	1.7377	12.1642	0.0000	0.1250	0.8750	0.0000
13 Ni	3.4755	6.9510	0.0000	0.2500	0.5000	0.0000
14 Ni	5.2132	8.6887	0.0000	0.3750	0.6250	0.0000
15 Ni	3.4755	10.4265	0.0000	0.2500	0.7500	0.0000
16 Ni	5.2132	12.1642	0.0000	0.3750	0.8750	0.0000
17 Ni	6.9510	0.0000	0.0000	0.5000	0.0000	0.0000
18 Ni	8.6887	1.7377	0.0000	0.6250	0.1250	0.0000
19 Ni	6.9510	3.4755	0.0000	0.5000	0.2500	0.0000
20 Ni	8.6887	5.2132	0.0000	0.6250	0.3750	0.0000
21 Ni	10.4265	0.0000	0.0000	0.7500	0.0000	0.0000
22 Ni	12.1642	1.7377	0.0000	0.8750	0.1250	0.0000
23 Ni	10.4265	3.4755	0.0000	0.7500	0.2500	0.0000
24 Ni	12.1642	5.2132	0.0000	0.8750	0.3750	0.0000
25 Ni	6.9510	6.9510	0.0000	0.5000	0.5000	0.0000
26 Ni	8.6887	8.6887	0.0000	0.6250	0.6250	0.0000
27 Ni	6.9510	10.4265	0.0000	0.5000	0.7500	0.0000
28 Ni	8.6887	12.1642	0.0000	0.6250	0.8750	0.0000
29 Ni	10.4265	6.9510	0.0000	0.7500	0.5000	0.0000
30 Ni	12.1642	8.6887	0.0000	0.8750	0.6250	0.0000
31 Ni	10.4265	10.4265	0.0000	0.7500	0.7500	0.0000
32 Ni	12.1642	12.1642	0.0000	0.8750	0.8750	0.0000
33 Ni	0.0000	1.7377	1.7377	0.0000	0.1250	0.0792
34 Ni	1.7377	0.0000	1.7377	0.1250	0.0000	0.0792
35 Ni	0.0000	5.2132	1.7377	0.0000	0.3750	0.0792
36 Ni	1.7377	3.4755	1.7377	0.1250	0.2500	0.0792
37 Ni	3.4755	1.7377	1.7377	0.2500	0.1250	0.0792
38 Ni	5.2132	0.0000	1.7377	0.3750	0.0000	0.0792
39 Ni	3.4755	5.2132	1.7377	0.2500	0.3750	0.0792

40	Ni	5.2132	3.4755	1.7377	0.3750	0.2500	0.0792
41	Ni	0.0000	8.6887	1.7377	0.0000	0.6250	0.0792
42	Ni	1.7377	6.9510	1.7377	0.1250	0.5000	0.0792
43	Ni	0.0000	12.1642	1.7377	0.0000	0.8750	0.0792
44	Ni	1.7377	10.4265	1.7377	0.1250	0.7500	0.0792
45	Ni	3.4755	8.6887	1.7377	0.2500	0.6250	0.0792
46	Ni	5.2132	6.9510	1.7377	0.3750	0.5000	0.0792
47	Ni	3.4755	12.1642	1.7377	0.2500	0.8750	0.0792
48	Ni	5.2132	10.4265	1.7377	0.3750	0.7500	0.0792
49	Ni	6.9510	1.7377	1.7377	0.5000	0.1250	0.0792
50	Ni	8.6887	0.0000	1.7377	0.6250	0.0000	0.0792
51	Ni	6.9510	5.2132	1.7377	0.5000	0.3750	0.0792
52	Ni	8.6887	3.4755	1.7377	0.6250	0.2500	0.0792
53	Ni	10.4265	1.7377	1.7377	0.7500	0.1250	0.0792
54	Ni	12.1642	0.0000	1.7377	0.8750	0.0000	0.0792
55	Ni	10.4265	5.2132	1.7377	0.7500	0.3750	0.0792
56	Ni	12.1642	3.4755	1.7377	0.8750	0.2500	0.0792
57	Ni	6.9510	8.6887	1.7377	0.5000	0.6250	0.0792
58	Ni	8.6887	6.9510	1.7377	0.6250	0.5000	0.0792
59	Ni	6.9510	12.1642	1.7377	0.5000	0.8750	0.0792
60	Ni	8.6887	10.4265	1.7377	0.6250	0.7500	0.0792
61	Ni	10.4265	8.6887	1.7377	0.7500	0.6250	0.0792
62	Ni	12.1642	6.9510	1.7377	0.8750	0.5000	0.0792
63	Ni	10.4265	12.1642	1.7377	0.7500	0.8750	0.0792
64	Ni	12.1642	10.4265	1.7377	0.8750	0.7500	0.0792
65	Ni	0.0111	0.0128	3.5067	0.0008	0.0009	0.1598
66	Ni	1.7332	1.7755	3.4605	0.1247	0.1277	0.1576
67	Ni	13.8768	3.4809	3.5226	0.9982	0.2504	0.1605
68	Ni	1.6808	5.1845	3.4714	0.1209	0.3729	0.1581
69	Ni	3.4663	0.0060	3.5104	0.2493	0.0004	0.1599
70	Ni	5.2018	1.7449	3.5423	0.3742	0.1255	0.1614
71	Ni	3.4999	3.4485	3.4811	0.2518	0.2481	0.1586
72	Ni	5.2365	5.2090	3.4762	0.3767	0.3747	0.1584
73	Ni	0.0008	6.9361	3.5183	0.0001	0.4989	0.1603
74	Ni	1.7528	8.6530	3.5469	0.1261	0.6224	0.1616
75	Ni	13.8977	10.4178	3.5203	0.9997	0.7494	0.1604
76	Ni	1.7400	12.1747	3.5145	0.1252	0.8757	0.1601
77	Ni	3.4734	6.9714	3.4687	0.2498	0.5015	0.1580
78	Ni	5.2286	8.7085	3.5013	0.3761	0.6264	0.1595
79	Ni	3.4647	10.3972	3.5261	0.2492	0.7479	0.1606
80	Ni	5.2046	12.1657	3.4999	0.3744	0.8751	0.1594
81	Ni	6.9381	13.8926	3.5001	0.4991	0.9993	0.1595
82	Ni	8.6922	1.7067	3.4892	0.6252	0.1228	0.1590
83	Ni	6.9491	3.4593	3.4979	0.4999	0.2488	0.1593
84	Ni	8.6541	5.2382	3.5053	0.6225	0.3768	0.1597
85	Ni	10.4443	13.8783	3.5188	0.7513	0.9983	0.1603
86	Ni	12.1792	1.7472	3.5298	0.8761	0.1257	0.1608
87	Ni	10.4174	3.4641	3.4860	0.7493	0.2492	0.1588
88	Ni	12.1536	5.2179	3.5022	0.8742	0.3753	0.1595

89	Ni	6.9405	6.9492	3.5084	0.4992	0.4999	0.1598
90	Ni	8.6900	8.7017	3.4647	0.6251	0.6259	0.1578
91	Ni	6.9724	10.4356	3.5086	0.5015	0.7507	0.1598
92	Ni	8.6952	12.1794	3.4891	0.6255	0.8761	0.1590
93	Ni	10.4081	6.9705	3.5097	0.7487	0.5014	0.1599
94	Ni	12.1853	8.6613	3.5089	0.8765	0.6230	0.1599
95	Ni	10.4495	10.4446	3.4295	0.7517	0.7513	0.1562
96	Ni	12.1806	12.1662	3.5025	0.8762	0.8751	0.1596
97	Ni	0.0247	1.7950	5.2897	0.0018	0.1291	0.2410
98	Ni	1.7299	13.8926	5.2966	0.1244	0.9993	0.2413
99	Ni	13.8149	5.1765	5.3226	0.9937	0.3724	0.2425
100	Ni	1.7499	3.5721	5.4108	0.1259	0.2569	0.2465
101	Ni	3.4485	1.7573	5.2859	0.2481	0.1264	0.2408
102	Ni	5.2014	13.9005	5.2212	0.3741	0.9999	0.2379
103	Ni	3.5196	5.1801	5.0585	0.2532	0.3726	0.2304
104	Ni	5.2548	3.4292	5.3553	0.3780	0.2467	0.2440
105	Ni	0.0357	8.6753	5.2159	0.0026	0.6240	0.2376
106	Ni	1.7235	6.8537	5.3019	0.1240	0.4930	0.2415
107	Ni	0.0035	12.1651	5.2123	0.0003	0.8751	0.2375
108	Ni	1.7313	10.4342	5.2201	0.1245	0.7506	0.2378
109	Ni	3.4544	8.7180	5.3772	0.2485	0.6271	0.2450
110	Ni	5.2125	6.9732	5.2067	0.3749	0.5016	0.2372
111	Ni	3.4760	12.1546	5.2179	0.2500	0.8743	0.2377
112	Ni	5.2236	10.4439	5.1931	0.3757	0.7513	0.2366
113	Ni	6.9785	1.6857	5.2035	0.5020	0.1213	0.2370
114	Ni	8.6891	13.8773	5.2165	0.6250	0.9982	0.2376
115	Ni	6.9599	5.2036	5.2790	0.5006	0.3743	0.2405
116	Ni	8.6984	3.4417	5.1619	0.6257	0.2476	0.2352
117	Ni	10.4110	1.6982	5.2091	0.7489	0.1222	0.2373
118	Ni	12.1961	0.0186	5.2150	0.8773	0.0013	0.2376
119	Ni	10.3661	5.2135	5.2012	0.7457	0.3750	0.2369
120	Ni	12.0465	3.4787	5.3075	0.8665	0.2502	0.2418
121	Ni	6.9804	8.7553	5.2398	0.5021	0.6298	0.2387
122	Ni	8.7016	6.9750	5.2700	0.6259	0.5017	0.2401
123	Ni	6.9369	12.1644	5.2204	0.4990	0.8750	0.2378
124	Ni	8.7193	10.4874	5.2008	0.6272	0.7544	0.2369
125	Ni	10.4465	8.7246	5.1724	0.7514	0.6276	0.2356
126	Ni	12.1272	6.9984	5.3132	0.8723	0.5034	0.2420
127	Ni	10.4682	12.1807	5.2810	0.7530	0.8762	0.2406
128	Ni	12.1974	10.4185	5.2803	0.8774	0.7494	0.2405
129	O	6.1404	4.3733	6.7946	0.4417	0.3146	0.3095
130	O	4.2334	7.6149	6.7576	0.3045	0.5478	0.3078
131	O	6.0970	6.6168	8.7669	0.4386	0.4760	0.3994
132	O	7.8957	7.9465	6.7968	0.5680	0.5716	0.3096
133	O	10.4349	10.4136	6.2582	0.7506	0.7491	0.2851
134	O	9.5186	7.2546	9.0012	0.6847	0.5218	0.4101
135	O	7.6130	9.9547	8.8660	0.5476	0.7161	0.4039
136	Ce	5.9617	8.8313	8.0384	0.4288	0.6353	0.3662
137	Ce	4.4035	5.5050	7.7371	0.3168	0.3960	0.3525

138	Ce	8.2795	5.8211	8.0774	0.5956	0.4187	0.3680
139	Sm	9.4156	9.1903	8.0925	0.6773	0.6611	0.3687
140	C	11.4738	4.8933	6.7489	0.8253	0.3520	0.3075
141	O	12.5762	5.6962	6.7953	0.9046	0.4097	0.3096
142	O	10.8003	4.6254	7.7753	0.7769	0.3327	0.3542
143	O	2.2012	5.2810	6.4086	0.1583	0.3799	0.2919
144	O	1.7297	1.6975	6.1836	0.1244	0.1221	0.2817

/db/jmorales/CoNi-alloy/Profiles/CoNi-Ce_3SmO_7/CO2

a =			13.9033384264				
b =			13.9033384264				
c =			21.9516692133				
alpha =			90.0				
beta =			90.0				
gamma =			90.0				
	Atom	X	Y	Z	X	Y	Z
1	Ni	0.0000	0.0000	0.0000	0.0000	0.0000	0.0000
2	Ni	0.0000	3.4758	0.0000	0.0000	0.2500	0.0000
3	Ni	3.4758	0.0000	0.0000	0.2500	0.0000	0.0000
4	Ni	3.4758	3.4758	0.0000	0.2500	0.2500	0.0000
5	Ni	0.0000	6.9517	0.0000	0.0000	0.5000	0.0000
6	Ni	0.0000	10.4275	0.0000	0.0000	0.7500	0.0000
7	Ni	3.4758	6.9517	0.0000	0.2500	0.5000	0.0000
8	Ni	3.4758	10.4275	0.0000	0.2500	0.7500	0.0000
9	Ni	6.9517	0.0000	0.0000	0.5000	0.0000	0.0000
10	Ni	6.9517	3.4758	0.0000	0.5000	0.2500	0.0000
11	Ni	10.4275	0.0000	0.0000	0.7500	0.0000	0.0000
12	Ni	10.4275	3.4758	0.0000	0.7500	0.2500	0.0000
13	Ni	6.9517	6.9517	0.0000	0.5000	0.5000	0.0000
14	Ni	6.9517	10.4275	0.0000	0.5000	0.7500	0.0000
15	Ni	10.4275	6.9517	0.0000	0.7500	0.5000	0.0000
16	Ni	10.4275	10.4275	0.0000	0.7500	0.7500	0.0000
17	Ni	0.0000	1.7379	1.7379	0.0000	0.1250	0.0792
18	Ni	0.0000	5.2138	1.7379	0.0000	0.3750	0.0792
19	Ni	3.4758	1.7379	1.7379	0.2500	0.1250	0.0792
20	Ni	3.4758	5.2138	1.7379	0.2500	0.3750	0.0792
21	Ni	0.0000	8.6896	1.7379	0.0000	0.6250	0.0792
22	Ni	0.0000	12.1654	1.7379	0.0000	0.8750	0.0792
23	Ni	3.4758	8.6896	1.7379	0.2500	0.6250	0.0792
24	Ni	3.4758	12.1654	1.7379	0.2500	0.8750	0.0792
25	Ni	6.9517	1.7379	1.7379	0.5000	0.1250	0.0792
26	Ni	6.9517	5.2138	1.7379	0.5000	0.3750	0.0792
27	Ni	10.4275	1.7379	1.7379	0.7500	0.1250	0.0792
28	Ni	10.4275	5.2138	1.7379	0.7500	0.3750	0.0792
29	Ni	6.9517	8.6896	1.7379	0.5000	0.6250	0.0792
30	Ni	6.9517	12.1654	1.7379	0.5000	0.8750	0.0792
31	Ni	10.4275	8.6896	1.7379	0.7500	0.6250	0.0792
32	Ni	10.4275	12.1654	1.7379	0.7500	0.8750	0.0792
33	Ni	13.8976	13.9005	3.5027	0.9996	0.9998	0.1596
34	Ni	0.0009	3.4780	3.5007	0.0001	0.2502	0.1595
35	Ni	3.4966	13.8822	3.5404	0.2515	0.9985	0.1613
36	Ni	3.4671	3.4584	3.4666	0.2494	0.2487	0.1579
37	Ni	0.0014	6.9571	3.5114	0.0001	0.5004	0.1600
38	Ni	13.8768	10.4332	3.4790	0.9981	0.7504	0.1585
39	Ni	3.4819	6.9556	3.4999	0.2504	0.5003	0.1594
40	Ni	3.5037	10.4253	3.5574	0.2520	0.7498	0.1621

41	Ni	6.9366	0.0119	3.5069	0.4989	0.0009	0.1598
42	Ni	6.9507	3.4976	3.5247	0.4999	0.2516	0.1606
43	Ni	10.4228	0.0032	3.5059	0.7497	0.0002	0.1597
44	Ni	10.4283	3.4775	3.5018	0.7501	0.2501	0.1595
45	Ni	6.9702	6.9277	3.4624	0.5013	0.4983	0.1577
46	Ni	6.9566	10.4446	3.4626	0.5004	0.7512	0.1577
47	Ni	10.4377	6.9377	3.4904	0.7507	0.4990	0.1590
48	Ni	10.4321	10.4419	3.5021	0.7503	0.7510	0.1595
49	Ni	13.8998	1.7542	5.2038	0.9997	0.1262	0.2371
50	Ni	0.0004	5.2110	5.2171	0.0000	0.3748	0.2377
51	Ni	3.4499	1.7543	5.1980	0.2481	0.1262	0.2368
52	Ni	3.4627	5.1840	5.1027	0.2491	0.3729	0.2324
53	Ni	13.8630	8.7118	5.1863	0.9971	0.6266	0.2363
54	Ni	0.0028	12.1615	5.1999	0.0002	0.8747	0.2369
55	Ni	3.4635	8.6684	5.2480	0.2491	0.6235	0.2391
56	Ni	3.3878	12.1421	5.3256	0.2437	0.8733	0.2426
57	Ni	6.9707	1.7640	5.2011	0.5014	0.1269	0.2369
58	Ni	6.9467	5.1600	5.3956	0.4996	0.3711	0.2458
59	Ni	10.4267	1.7353	5.2032	0.7499	0.1248	0.2370
60	Ni	10.4295	5.2159	5.2130	0.7501	0.3752	0.2375
61	Ni	7.0372	8.6814	5.0648	0.5061	0.6244	0.2307
62	Ni	6.9861	12.1726	5.1223	0.5025	0.8755	0.2333
63	Ni	10.4210	8.6955	5.2047	0.7495	0.6254	0.2371
64	Ni	10.4295	12.1806	5.2663	0.7501	0.8761	0.2399
65	Co	1.7379	1.7379	0.0000	0.1250	0.1250	0.0000
66	Co	1.7379	5.2138	0.0000	0.1250	0.3750	0.0000
67	Co	5.2138	1.7379	0.0000	0.3750	0.1250	0.0000
68	Co	5.2138	5.2138	0.0000	0.3750	0.3750	0.0000
69	Co	1.7379	8.6896	0.0000	0.1250	0.6250	0.0000
70	Co	1.7379	12.1654	0.0000	0.1250	0.8750	0.0000
71	Co	5.2138	8.6896	0.0000	0.3750	0.6250	0.0000
72	Co	5.2138	12.1654	0.0000	0.3750	0.8750	0.0000
73	Co	8.6896	1.7379	0.0000	0.6250	0.1250	0.0000
74	Co	8.6896	5.2138	0.0000	0.6250	0.3750	0.0000
75	Co	12.1654	1.7379	0.0000	0.8750	0.1250	0.0000
76	Co	12.1654	5.2138	0.0000	0.8750	0.3750	0.0000
77	Co	8.6896	8.6896	0.0000	0.6250	0.6250	0.0000
78	Co	8.6896	12.1654	0.0000	0.6250	0.8750	0.0000
79	Co	12.1654	8.6896	0.0000	0.8750	0.6250	0.0000
80	Co	12.1654	12.1654	0.0000	0.8750	0.8750	0.0000
81	Co	1.7379	0.0000	1.7379	0.1250	0.0000	0.0792
82	Co	1.7379	3.4758	1.7379	0.1250	0.2500	0.0792
83	Co	5.2138	0.0000	1.7379	0.3750	0.0000	0.0792
84	Co	5.2138	3.4758	1.7379	0.3750	0.2500	0.0792
85	Co	1.7379	6.9517	1.7379	0.1250	0.5000	0.0792
86	Co	1.7379	10.4275	1.7379	0.1250	0.7500	0.0792
87	Co	5.2138	6.9517	1.7379	0.3750	0.5000	0.0792
88	Co	5.2138	10.4275	1.7379	0.3750	0.7500	0.0792
89	Co	8.6896	0.0000	1.7379	0.6250	0.0000	0.0792

90	Co	8.6896	3.4758	1.7379	0.6250	0.2500	0.0792
91	Co	12.1654	0.0000	1.7379	0.8750	0.0000	0.0792
92	Co	12.1654	3.4758	1.7379	0.8750	0.2500	0.0792
93	Co	8.6896	6.9517	1.7379	0.6250	0.5000	0.0792
94	Co	8.6896	10.4275	1.7379	0.6250	0.7500	0.0792
95	Co	12.1654	6.9517	1.7379	0.8750	0.5000	0.0792
96	Co	12.1654	10.4275	1.7379	0.8750	0.7500	0.0792
97	Co	1.7340	1.7366	3.5382	0.1247	0.1249	0.1612
98	Co	1.7097	5.2396	3.5361	0.1230	0.3769	0.1611
99	Co	5.2132	1.7245	3.5634	0.3750	0.1240	0.1623
100	Co	5.2415	5.2005	3.5347	0.3770	0.3740	0.1610
101	Co	1.7342	8.6780	3.5227	0.1247	0.6242	0.1605
102	Co	1.7486	12.1723	3.5395	0.1258	0.8755	0.1612
103	Co	5.1979	8.6836	3.5525	0.3739	0.6246	0.1618
104	Co	5.1887	12.1634	3.6229	0.3732	0.8749	0.1650
105	Co	8.6880	1.7346	3.5422	0.6249	0.1248	0.1614
106	Co	8.6680	5.2029	3.5810	0.6234	0.3742	0.1631
107	Co	12.1635	1.7401	3.5491	0.8749	0.1252	0.1617
108	Co	12.1709	5.2112	3.5596	0.8754	0.3748	0.1622
109	Co	8.7307	8.7018	3.4951	0.6280	0.6259	0.1592
110	Co	8.7146	12.1742	3.4567	0.6268	0.8756	0.1575
111	Co	12.1492	8.6886	3.5419	0.8738	0.6249	0.1613
112	Co	12.1450	12.1765	3.5776	0.8735	0.8758	0.1630
113	Co	1.7009	0.0356	5.2114	0.1223	0.0026	0.2374
114	Co	1.7477	3.4903	5.2161	0.1257	0.2510	0.2376
115	Co	5.2258	0.0202	5.3380	0.3759	0.0015	0.2432
116	Co	5.1681	3.4420	5.2058	0.3717	0.2476	0.2371
117	Co	1.6936	6.9682	5.3196	0.1218	0.5012	0.2423
118	Co	1.6843	10.4114	5.1446	0.1211	0.7488	0.2344
119	Co	5.2901	6.9324	5.1889	0.3805	0.4986	0.2364
120	Co	5.3060	10.3618	5.2730	0.3816	0.7453	0.2402
121	Co	8.6870	0.0081	5.2757	0.6248	0.0006	0.2403
122	Co	8.7134	3.4618	5.2281	0.6267	0.2490	0.2382
123	Co	12.1751	0.0153	5.2228	0.8757	0.0011	0.2379
124	Co	12.1593	3.4740	5.2249	0.8746	0.2499	0.2380
125	Co	8.7215	6.9750	5.1724	0.6273	0.5017	0.2356
126	Co	8.6970	10.4404	5.2526	0.6255	0.7509	0.2393
127	Co	12.1590	6.9538	5.2215	0.8745	0.5002	0.2379
128	Co	12.1625	10.4286	5.2159	0.8748	0.7501	0.2376
129	O	5.9954	6.1718	6.8507	0.4312	0.4439	0.3121
130	O	2.5472	7.8723	6.8677	0.1832	0.5662	0.3129
131	O	4.6693	8.0057	8.7512	0.3358	0.5758	0.3987
132	O	5.6238	10.2374	7.1831	0.4045	0.7363	0.3272
133	O	7.8208	9.9935	8.9133	0.5625	0.7188	0.4060
134	O	4.7919	12.1346	9.0457	0.3447	0.8728	0.4121
135	O	8.6257	12.2080	6.3597	0.6204	0.8781	0.2897
136	Ce	3.4990	9.8297	7.9189	0.2517	0.7070	0.3607
137	Ce	3.8546	6.1705	7.6768	0.2772	0.4438	0.3497
138	Ce	6.8678	8.2906	7.9013	0.4940	0.5963	0.3599

139 Sm	7.0868	11.7498	8.0424	0.5097	0.8451	0.3664
140 C	4.4401	12.7065	7.9331	0.3194	0.9139	0.3614
141 O	3.3290	12.3201	7.3562	0.2394	0.8861	0.3351
142 O	5.2639	13.5348	7.3600	0.3786	0.9735	0.3353

/db/jmorales/CoNi-alloy/Profiles/Ni(001)/O

a =			6.950991869				
b =			6.950991869				
c =			21.950991869				
alpha =			90.0				
beta =			90.0				
gamma =			90.0				
	Atom	X	Y	Z	X	Y	Z
1	Ni	0.0000	0.0000	0.0000	0.0000	0.0000	0.0000
2	Ni	1.7378	1.7378	0.0000	0.2500	0.2500	0.0000
3	Ni	0.0000	3.4755	0.0000	0.0000	0.5000	0.0000
4	Ni	1.7378	5.2132	0.0000	0.2500	0.7500	0.0000
5	Ni	3.4755	0.0000	0.0000	0.5000	0.0000	0.0000
6	Ni	5.2132	1.7378	0.0000	0.7500	0.2500	0.0000
7	Ni	3.4755	3.4755	0.0000	0.5000	0.5000	0.0000
8	Ni	5.2132	5.2132	0.0000	0.7500	0.7500	0.0000
9	Ni	0.0000	1.7378	1.7378	0.0000	0.2500	0.0792
10	Ni	1.7378	0.0000	1.7378	0.2500	0.0000	0.0792
11	Ni	0.0000	5.2132	1.7378	0.0000	0.7500	0.0792
12	Ni	1.7378	3.4755	1.7378	0.2500	0.5000	0.0792
13	Ni	3.4755	1.7378	1.7378	0.5000	0.2500	0.0792
14	Ni	5.2132	0.0000	1.7378	0.7500	0.0000	0.0792
15	Ni	3.4755	5.2132	1.7378	0.5000	0.7500	0.0792
16	Ni	5.2132	3.4755	1.7378	0.7500	0.5000	0.0792
17	Ni	0.0003	0.0025	3.5343	0.0000	0.0004	0.1610
18	Ni	1.7369	1.7369	3.5021	0.2499	0.2499	0.1595
19	Ni	6.9505	3.4769	3.5084	0.9999	0.5002	0.1598
20	Ni	1.7366	5.2170	3.5029	0.2498	0.7505	0.1596
21	Ni	3.4756	0.0015	3.4372	0.5000	0.0002	0.1566
22	Ni	5.2131	1.7368	3.5020	0.7500	0.2499	0.1595
23	Ni	3.4751	3.4766	3.4837	0.4999	0.5002	0.1587
24	Ni	5.2136	5.2166	3.5023	0.7501	0.7505	0.1596
25	Ni	6.9506	1.7499	5.2059	0.9999	0.2517	0.2372
26	Ni	1.7511	0.0012	5.2722	0.2519	0.0002	0.2402
27	Ni	0.0003	5.2054	5.2064	0.0000	0.7489	0.2372
28	Ni	1.7660	3.4758	5.2496	0.2541	0.5000	0.2392
29	Ni	3.4759	1.7657	5.3444	0.5001	0.2540	0.2435
30	Ni	5.2009	0.0009	5.2718	0.7482	0.0001	0.2402
31	Ni	3.4752	5.1872	5.3432	0.5000	0.7463	0.2434
32	Ni	5.1844	3.4764	5.2494	0.7458	0.5001	0.2391
33	O	3.4770	0.0010	6.1344	0.5002	0.0001	0.2795

/db/jmorales/CoNi-alloy/Profiles/Ni-Ce_3SmO_7/O-coverage/CO_2-Ni-Ce_3SmO_7-O

a =	13.9019837372
b =	13.9019837372
c =	21.9509918687
alpha =	90.0
beta =	90.0
gamma =	90.0

	Atom	X	Y	Z	X	Y	Z
1	Ni	0.0000	0.0000	0.0000	0.0000	0.0000	0.0000
2	Ni	1.7377	1.7377	0.0000	0.1250	0.1250	0.0000
3	Ni	0.0000	3.4755	0.0000	0.0000	0.2500	0.0000
4	Ni	1.7377	5.2132	0.0000	0.1250	0.3750	0.0000
5	Ni	3.4755	0.0000	0.0000	0.2500	0.0000	0.0000
6	Ni	5.2132	1.7377	0.0000	0.3750	0.1250	0.0000
7	Ni	3.4755	3.4755	0.0000	0.2500	0.2500	0.0000
8	Ni	5.2132	5.2132	0.0000	0.3750	0.3750	0.0000
9	Ni	0.0000	6.9510	0.0000	0.0000	0.5000	0.0000
10	Ni	1.7377	8.6887	0.0000	0.1250	0.6250	0.0000
11	Ni	0.0000	10.4265	0.0000	0.0000	0.7500	0.0000
12	Ni	1.7377	12.1642	0.0000	0.1250	0.8750	0.0000
13	Ni	3.4755	6.9510	0.0000	0.2500	0.5000	0.0000
14	Ni	5.2132	8.6887	0.0000	0.3750	0.6250	0.0000
15	Ni	3.4755	10.4265	0.0000	0.2500	0.7500	0.0000
16	Ni	5.2132	12.1642	0.0000	0.3750	0.8750	0.0000
17	Ni	6.9510	0.0000	0.0000	0.5000	0.0000	0.0000
18	Ni	8.6887	1.7377	0.0000	0.6250	0.1250	0.0000
19	Ni	6.9510	3.4755	0.0000	0.5000	0.2500	0.0000
20	Ni	8.6887	5.2132	0.0000	0.6250	0.3750	0.0000
21	Ni	10.4265	0.0000	0.0000	0.7500	0.0000	0.0000
22	Ni	12.1642	1.7377	0.0000	0.8750	0.1250	0.0000
23	Ni	10.4265	3.4755	0.0000	0.7500	0.2500	0.0000
24	Ni	12.1642	5.2132	0.0000	0.8750	0.3750	0.0000
25	Ni	6.9510	6.9510	0.0000	0.5000	0.5000	0.0000
26	Ni	8.6887	8.6887	0.0000	0.6250	0.6250	0.0000
27	Ni	6.9510	10.4265	0.0000	0.5000	0.7500	0.0000
28	Ni	8.6887	12.1642	0.0000	0.6250	0.8750	0.0000
29	Ni	10.4265	6.9510	0.0000	0.7500	0.5000	0.0000
30	Ni	12.1642	8.6887	0.0000	0.8750	0.6250	0.0000
31	Ni	10.4265	10.4265	0.0000	0.7500	0.7500	0.0000
32	Ni	12.1642	12.1642	0.0000	0.8750	0.8750	0.0000
33	Ni	0.0000	1.7377	1.7377	0.0000	0.1250	0.0792
34	Ni	1.7377	0.0000	1.7377	0.1250	0.0000	0.0792
35	Ni	0.0000	5.2132	1.7377	0.0000	0.3750	0.0792
36	Ni	1.7377	3.4755	1.7377	0.1250	0.2500	0.0792
37	Ni	3.4755	1.7377	1.7377	0.2500	0.1250	0.0792
38	Ni	5.2132	0.0000	1.7377	0.3750	0.0000	0.0792
39	Ni	3.4755	5.2132	1.7377	0.2500	0.3750	0.0792
40	Ni	5.2132	3.4755	1.7377	0.3750	0.2500	0.0792

41	Ni	0.0000	8.6887	1.7377	0.0000	0.6250	0.0792
42	Ni	1.7377	6.9510	1.7377	0.1250	0.5000	0.0792
43	Ni	0.0000	12.1642	1.7377	0.0000	0.8750	0.0792
44	Ni	1.7377	10.4265	1.7377	0.1250	0.7500	0.0792
45	Ni	3.4755	8.6887	1.7377	0.2500	0.6250	0.0792
46	Ni	5.2132	6.9510	1.7377	0.3750	0.5000	0.0792
47	Ni	3.4755	12.1642	1.7377	0.2500	0.8750	0.0792
48	Ni	5.2132	10.4265	1.7377	0.3750	0.7500	0.0792
49	Ni	6.9510	1.7377	1.7377	0.5000	0.1250	0.0792
50	Ni	8.6887	0.0000	1.7377	0.6250	0.0000	0.0792
51	Ni	6.9510	5.2132	1.7377	0.5000	0.3750	0.0792
52	Ni	8.6887	3.4755	1.7377	0.6250	0.2500	0.0792
53	Ni	10.4265	1.7377	1.7377	0.7500	0.1250	0.0792
54	Ni	12.1642	0.0000	1.7377	0.8750	0.0000	0.0792
55	Ni	10.4265	5.2132	1.7377	0.7500	0.3750	0.0792
56	Ni	12.1642	3.4755	1.7377	0.8750	0.2500	0.0792
57	Ni	6.9510	8.6887	1.7377	0.5000	0.6250	0.0792
58	Ni	8.6887	6.9510	1.7377	0.6250	0.5000	0.0792
59	Ni	6.9510	12.1642	1.7377	0.5000	0.8750	0.0792
60	Ni	8.6887	10.4265	1.7377	0.6250	0.7500	0.0792
61	Ni	10.4265	8.6887	1.7377	0.7500	0.6250	0.0792
62	Ni	12.1642	6.9510	1.7377	0.8750	0.5000	0.0792
63	Ni	10.4265	12.1642	1.7377	0.7500	0.8750	0.0792
64	Ni	12.1642	10.4265	1.7377	0.8750	0.7500	0.0792
65	Ni	0.0105	0.0047	3.5047	0.0008	0.0003	0.1597
66	Ni	1.7389	1.7534	3.5254	0.1251	0.1261	0.1606
67	Ni	0.0004	3.4732	3.5076	0.0000	0.2498	0.1598
68	Ni	1.7007	5.2095	3.4180	0.1223	0.3747	0.1557
69	Ni	3.4763	13.8993	3.5090	0.2501	0.9998	0.1599
70	Ni	5.2169	1.7424	3.5280	0.3753	0.1253	0.1607
71	Ni	3.4931	3.4341	3.4830	0.2513	0.2470	0.1587
72	Ni	5.1978	5.2089	3.4799	0.3739	0.3747	0.1585
73	Ni	13.8708	6.9445	3.5340	0.9978	0.4995	0.1610
74	Ni	1.7423	8.6747	3.5324	0.1253	0.6240	0.1609
75	Ni	13.8859	10.4283	3.5187	0.9988	0.7501	0.1603
76	Ni	1.7436	12.1612	3.5067	0.1254	0.8748	0.1598
77	Ni	3.4701	6.9893	3.4652	0.2496	0.5028	0.1579
78	Ni	5.2303	8.7092	3.5051	0.3762	0.6265	0.1597
79	Ni	3.4724	10.4044	3.5272	0.2498	0.7484	0.1607
80	Ni	5.2120	12.1646	3.5027	0.3749	0.8750	0.1596
81	Ni	6.9427	13.8979	3.5033	0.4994	0.9997	0.1596
82	Ni	8.6972	1.7211	3.4902	0.6256	0.1238	0.1590
83	Ni	6.9343	3.4609	3.4936	0.4988	0.2490	0.1592
84	Ni	8.6828	5.2220	3.5263	0.6246	0.3756	0.1606
85	Ni	10.4350	13.8840	3.5236	0.7506	0.9987	0.1605
86	Ni	12.1630	1.7331	3.5027	0.8749	0.1247	0.1596
87	Ni	10.4366	3.4936	3.5086	0.7507	0.2513	0.1598
88	Ni	12.1701	5.2255	3.5711	0.8754	0.3759	0.1627
89	Ni	6.9256	6.9377	3.5073	0.4982	0.4990	0.1598

90	Ni	8.6886	8.6803	3.4621	0.6250	0.6244	0.1577
91	Ni	6.9803	10.4254	3.5184	0.5021	0.7499	0.1603
92	Ni	8.6919	12.1868	3.4919	0.6252	0.8766	0.1591
93	Ni	10.4340	6.9288	3.5454	0.7505	0.4984	0.1615
94	Ni	12.1811	8.6718	3.4961	0.8762	0.6238	0.1593
95	Ni	10.4389	10.4528	3.4256	0.7509	0.7519	0.1561
96	Ni	12.1721	12.1746	3.5051	0.8756	0.8757	0.1597
97	Ni	0.0166	1.7307	5.2249	0.0012	0.1245	0.2380
98	Ni	1.7408	13.8951	5.2244	0.1252	0.9995	0.2380
99	Ni	0.0209	5.1867	5.2890	0.0015	0.3731	0.2409
100	Ni	1.7615	3.4720	5.2976	0.1267	0.2497	0.2413
101	Ni	3.4710	1.7137	5.2298	0.2497	0.1233	0.2382
102	Ni	5.2104	13.8968	5.2283	0.3748	0.9996	0.2382
103	Ni	3.4511	5.1936	5.0499	0.2482	0.3736	0.2301
104	Ni	5.1888	3.4190	5.3639	0.3732	0.2459	0.2444
105	Ni	0.0168	8.7005	5.2125	0.0012	0.6258	0.2375
106	Ni	1.7166	6.9249	5.2540	0.1235	0.4981	0.2394
107	Ni	0.0006	12.1755	5.2195	0.0000	0.8758	0.2378
108	Ni	1.7204	10.4445	5.2220	0.1238	0.7513	0.2379
109	Ni	3.4465	8.7545	5.3656	0.2479	0.6297	0.2444
110	Ni	5.1815	6.9801	5.2230	0.3727	0.5021	0.2379
111	Ni	3.4834	12.1507	5.2300	0.2506	0.8740	0.2383
112	Ni	5.2439	10.4427	5.2055	0.3772	0.7512	0.2371
113	Ni	6.9681	1.7028	5.2077	0.5012	0.1225	0.2372
114	Ni	8.6894	0.0016	5.2187	0.6251	0.0001	0.2377
115	Ni	6.9049	5.1815	5.2305	0.4967	0.3727	0.2383
116	Ni	8.6705	3.4367	5.1635	0.6237	0.2472	0.2352
117	Ni	10.4241	1.7354	5.2166	0.7498	0.1248	0.2376
118	Ni	12.1793	0.0199	5.2223	0.8761	0.0014	0.2379
119	Ni	10.4030	5.1199	5.3594	0.7483	0.3683	0.2442
120	Ni	12.1892	3.4354	5.2130	0.8768	0.2471	0.2375
121	Ni	6.9903	8.7275	5.2508	0.5028	0.6278	0.2392
122	Ni	8.6843	6.9364	5.2425	0.6247	0.4989	0.2388
123	Ni	6.9495	12.1680	5.2230	0.4999	0.8753	0.2379
124	Ni	8.7092	10.4713	5.2130	0.6265	0.7532	0.2375
125	Ni	10.4229	8.7249	5.1147	0.7497	0.6276	0.2330
126	Ni	12.1755	6.9807	5.4009	0.8758	0.5021	0.2460
127	Ni	10.4474	12.1945	5.2842	0.7515	0.8772	0.2407
128	Ni	12.1814	10.4316	5.2707	0.8762	0.7504	0.2401
129	O	6.0026	4.4067	6.8587	0.4318	0.3170	0.3125
130	O	4.1608	7.6646	6.7791	0.2993	0.5513	0.3088
131	O	5.9260	6.5823	8.7603	0.4263	0.4735	0.3991
132	O	7.8970	7.7538	6.8886	0.5680	0.5577	0.3138
133	O	10.4295	10.4510	6.2800	0.7502	0.7518	0.2861
134	O	9.9026	6.8994	8.8297	0.7123	0.4963	0.4022
135	O	7.7536	9.7388	8.9002	0.5577	0.7005	0.4055
136	Ce	5.9782	8.8193	7.9869	0.4300	0.6344	0.3639
137	Ce	4.1427	5.5555	7.6536	0.2980	0.3996	0.3487
138	Ce	7.7956	5.5752	7.8750	0.5608	0.4010	0.3588

139 Sm	9.4749	9.0883	7.9026	0.6816	0.6537	0.3600
140 C	10.6634	6.4465	7.8465	0.7670	0.4637	0.3575
141 O	11.5561	7.2249	7.3422	0.8313	0.5197	0.3345
142 O	10.3586	5.2716	7.3712	0.7451	0.3792	0.3358
143 O	1.8981	5.2199	6.2922	0.1365	0.3755	0.2866

/db/jmorales/CoNi-alloy/Profiles/Ni-Ce_3SmO_7/O-coverage/CO-O-Ni-Ce_3SmO_7-O

a =			13.9019837372				
b =			13.9019837372				
c =			21.9509918687				
alpha =			90.0				
beta =			90.0				
gamma =			90.0				
	Atom	X	Y	Z	X	Y	Z
1	Ni	0.0000	0.0000	0.0000	0.0000	0.0000	0.0000
2	Ni	1.7377	1.7377	0.0000	0.1250	0.1250	0.0000
3	Ni	0.0000	3.4755	0.0000	0.0000	0.2500	0.0000
4	Ni	1.7377	5.2132	0.0000	0.1250	0.3750	0.0000
5	Ni	3.4755	0.0000	0.0000	0.2500	0.0000	0.0000
6	Ni	5.2132	1.7377	0.0000	0.3750	0.1250	0.0000
7	Ni	3.4755	3.4755	0.0000	0.2500	0.2500	0.0000
8	Ni	5.2132	5.2132	0.0000	0.3750	0.3750	0.0000
9	Ni	0.0000	6.9510	0.0000	0.0000	0.5000	0.0000
10	Ni	1.7377	8.6887	0.0000	0.1250	0.6250	0.0000
11	Ni	0.0000	10.4265	0.0000	0.0000	0.7500	0.0000
12	Ni	1.7377	12.1642	0.0000	0.1250	0.8750	0.0000
13	Ni	3.4755	6.9510	0.0000	0.2500	0.5000	0.0000
14	Ni	5.2132	8.6887	0.0000	0.3750	0.6250	0.0000
15	Ni	3.4755	10.4265	0.0000	0.2500	0.7500	0.0000
16	Ni	5.2132	12.1642	0.0000	0.3750	0.8750	0.0000
17	Ni	6.9510	0.0000	0.0000	0.5000	0.0000	0.0000
18	Ni	8.6887	1.7377	0.0000	0.6250	0.1250	0.0000
19	Ni	6.9510	3.4755	0.0000	0.5000	0.2500	0.0000
20	Ni	8.6887	5.2132	0.0000	0.6250	0.3750	0.0000
21	Ni	10.4265	0.0000	0.0000	0.7500	0.0000	0.0000
22	Ni	12.1642	1.7377	0.0000	0.8750	0.1250	0.0000
23	Ni	10.4265	3.4755	0.0000	0.7500	0.2500	0.0000
24	Ni	12.1642	5.2132	0.0000	0.8750	0.3750	0.0000
25	Ni	6.9510	6.9510	0.0000	0.5000	0.5000	0.0000
26	Ni	8.6887	8.6887	0.0000	0.6250	0.6250	0.0000
27	Ni	6.9510	10.4265	0.0000	0.5000	0.7500	0.0000
28	Ni	8.6887	12.1642	0.0000	0.6250	0.8750	0.0000
29	Ni	10.4265	6.9510	0.0000	0.7500	0.5000	0.0000
30	Ni	12.1642	8.6887	0.0000	0.8750	0.6250	0.0000
31	Ni	10.4265	10.4265	0.0000	0.7500	0.7500	0.0000
32	Ni	12.1642	12.1642	0.0000	0.8750	0.8750	0.0000
33	Ni	0.0000	1.7377	1.7377	0.0000	0.1250	0.0792
34	Ni	1.7377	0.0000	1.7377	0.1250	0.0000	0.0792
35	Ni	0.0000	5.2132	1.7377	0.0000	0.3750	0.0792
36	Ni	1.7377	3.4755	1.7377	0.1250	0.2500	0.0792
37	Ni	3.4755	1.7377	1.7377	0.2500	0.1250	0.0792
38	Ni	5.2132	0.0000	1.7377	0.3750	0.0000	0.0792
39	Ni	3.4755	5.2132	1.7377	0.2500	0.3750	0.0792
40	Ni	5.2132	3.4755	1.7377	0.3750	0.2500	0.0792

41	Ni	0.0000	8.6887	1.7377	0.0000	0.6250	0.0792
42	Ni	1.7377	6.9510	1.7377	0.1250	0.5000	0.0792
43	Ni	0.0000	12.1642	1.7377	0.0000	0.8750	0.0792
44	Ni	1.7377	10.4265	1.7377	0.1250	0.7500	0.0792
45	Ni	3.4755	8.6887	1.7377	0.2500	0.6250	0.0792
46	Ni	5.2132	6.9510	1.7377	0.3750	0.5000	0.0792
47	Ni	3.4755	12.1642	1.7377	0.2500	0.8750	0.0792
48	Ni	5.2132	10.4265	1.7377	0.3750	0.7500	0.0792
49	Ni	6.9510	1.7377	1.7377	0.5000	0.1250	0.0792
50	Ni	8.6887	0.0000	1.7377	0.6250	0.0000	0.0792
51	Ni	6.9510	5.2132	1.7377	0.5000	0.3750	0.0792
52	Ni	8.6887	3.4755	1.7377	0.6250	0.2500	0.0792
53	Ni	10.4265	1.7377	1.7377	0.7500	0.1250	0.0792
54	Ni	12.1642	0.0000	1.7377	0.8750	0.0000	0.0792
55	Ni	10.4265	5.2132	1.7377	0.7500	0.3750	0.0792
56	Ni	12.1642	3.4755	1.7377	0.8750	0.2500	0.0792
57	Ni	6.9510	8.6887	1.7377	0.5000	0.6250	0.0792
58	Ni	8.6887	6.9510	1.7377	0.6250	0.5000	0.0792
59	Ni	6.9510	12.1642	1.7377	0.5000	0.8750	0.0792
60	Ni	8.6887	10.4265	1.7377	0.6250	0.7500	0.0792
61	Ni	10.4265	8.6887	1.7377	0.7500	0.6250	0.0792
62	Ni	12.1642	6.9510	1.7377	0.8750	0.5000	0.0792
63	Ni	10.4265	12.1642	1.7377	0.7500	0.8750	0.0792
64	Ni	12.1642	10.4265	1.7377	0.8750	0.7500	0.0792
65	Ni	0.0061	0.0055	3.5062	0.0004	0.0004	0.1597
66	Ni	1.7169	1.7642	3.5476	0.1235	0.1269	0.1616
67	Ni	13.8955	3.4735	3.5078	0.9995	0.2499	0.1598
68	Ni	1.6951	5.2045	3.4216	0.1219	0.3744	0.1559
69	Ni	3.4719	0.0087	3.5101	0.2497	0.0006	0.1599
70	Ni	5.2234	1.7488	3.5285	0.3757	0.1258	0.1607
71	Ni	3.4926	3.4352	3.4781	0.2512	0.2471	0.1584
72	Ni	5.2194	5.2032	3.4821	0.3754	0.3743	0.1586
73	Ni	13.8995	6.9468	3.5019	0.9998	0.4997	0.1595
74	Ni	1.7414	8.6665	3.5422	0.1253	0.6234	0.1614
75	Ni	13.8857	10.4211	3.5237	0.9988	0.7496	0.1605
76	Ni	1.7425	12.1607	3.5047	0.1253	0.8747	0.1597
77	Ni	3.4685	6.9806	3.4620	0.2495	0.5021	0.1577
78	Ni	5.2244	8.7085	3.4961	0.3758	0.6264	0.1593
79	Ni	3.4653	10.4034	3.5235	0.2493	0.7483	0.1605
80	Ni	5.2097	12.1693	3.5010	0.3747	0.8754	0.1595
81	Ni	6.9496	-0.0004	3.5037	0.4999	-0.0000	0.1596
82	Ni	8.7167	1.7270	3.5143	0.6270	0.1242	0.1601
83	Ni	6.9555	3.4659	3.5048	0.5003	0.2493	0.1597
84	Ni	8.6709	5.2356	3.5295	0.6237	0.3766	0.1608
85	Ni	10.4355	13.8778	3.5226	0.7506	0.9983	0.1605
86	Ni	12.1583	1.7434	3.4399	0.8746	0.1254	0.1567
87	Ni	10.4249	3.4835	3.5001	0.7499	0.2506	0.1595
88	Ni	12.1699	5.2002	3.4914	0.8754	0.3741	0.1591
89	Ni	6.9330	6.9393	3.5138	0.4987	0.4992	0.1601

90	Ni	8.6888	8.6842	3.4763	0.6250	0.6247	0.1584
91	Ni	6.9744	10.4259	3.5133	0.5017	0.7500	0.1601
92	Ni	8.6916	12.1736	3.4950	0.6252	0.8757	0.1592
93	Ni	10.4079	6.9561	3.5162	0.7487	0.5004	0.1602
94	Ni	12.1779	8.6759	3.4901	0.8760	0.6241	0.1590
95	Ni	10.4432	10.4384	3.4338	0.7512	0.7509	0.1564
96	Ni	12.1723	12.1759	3.5167	0.8756	0.8758	0.1602
97	Ni	13.8877	1.7631	5.2848	0.9990	0.1268	0.2408
98	Ni	1.7348	-0.0007	5.2183	0.1248	-0.0001	0.2377
99	Ni	13.8368	5.2058	5.3138	0.9953	0.3745	0.2421
100	Ni	1.7351	3.5179	5.2962	0.1248	0.2530	0.2413
101	Ni	3.4683	1.7343	5.2212	0.2495	0.1248	0.2379
102	Ni	5.2111	0.0017	5.2229	0.3748	0.0001	0.2379
103	Ni	3.4702	5.1842	5.0424	0.2496	0.3729	0.2297
104	Ni	5.2105	3.4288	5.3652	0.3748	0.2466	0.2444
105	Ni	0.0067	8.6697	5.2158	0.0005	0.6236	0.2376
106	Ni	1.7037	6.8799	5.2647	0.1226	0.4949	0.2398
107	Ni	0.0044	12.1676	5.2132	0.0003	0.8752	0.2375
108	Ni	1.7220	10.4371	5.2222	0.1239	0.7508	0.2379
109	Ni	3.4428	8.7427	5.3554	0.2476	0.6289	0.2440
110	Ni	5.1783	6.9690	5.2037	0.3725	0.5013	0.2371
111	Ni	3.4773	12.1575	5.2221	0.2501	0.8745	0.2379
112	Ni	5.2279	10.4470	5.1915	0.3761	0.7515	0.2365
113	Ni	6.9873	1.7104	5.2065	0.5026	0.1230	0.2372
114	Ni	8.6936	13.8902	5.2157	0.6253	0.9992	0.2376
115	Ni	6.9315	5.1840	5.2713	0.4986	0.3729	0.2401
116	Ni	8.7150	3.4760	5.1950	0.6269	0.2500	0.2367
117	Ni	10.4351	1.7371	5.2847	0.7506	0.1250	0.2407
118	Ni	12.1740	13.8992	5.2805	0.8757	0.9998	0.2406
119	Ni	10.4395	5.2202	5.2744	0.7509	0.3755	0.2403
120	Ni	12.1407	3.5001	5.3570	0.8733	0.2518	0.2440
121	Ni	6.9651	8.7363	5.2398	0.5010	0.6284	0.2387
122	Ni	8.7005	6.9419	5.2874	0.6258	0.4993	0.2409
123	Ni	6.9476	12.1624	5.2191	0.4998	0.8749	0.2378
124	Ni	8.7167	10.4571	5.2149	0.6270	0.7522	0.2376
125	Ni	10.4566	8.6922	5.1802	0.7522	0.6252	0.2360
126	Ni	12.1513	6.9339	5.2565	0.8741	0.4988	0.2395
127	Ni	10.4463	12.1588	5.2763	0.7514	0.8746	0.2404
128	Ni	12.1810	10.4189	5.2812	0.8762	0.7495	0.2406
129	O	6.0031	4.4406	6.8150	0.4318	0.3194	0.3105
130	O	4.1527	7.6306	6.7390	0.2987	0.5489	0.3070
131	O	5.8761	6.6405	8.7985	0.4227	0.4777	0.4008
132	O	7.7643	7.8411	6.8033	0.5585	0.5640	0.3099
133	O	10.4105	10.3518	6.2707	0.7489	0.7446	0.2857
134	O	9.4020	7.0222	8.9440	0.6763	0.5051	0.4075
135	O	7.6205	9.8187	8.8654	0.5482	0.7063	0.4039
136	Ce	5.8816	8.8681	8.0220	0.4231	0.6379	0.3655
137	Ce	4.1949	5.5408	7.7009	0.3017	0.3986	0.3508
138	Ce	7.9320	5.7551	8.0878	0.5706	0.4140	0.3685

139	Sm	9.3794	8.9746	8.0464	0.6747	0.6456	0.3666
140	C	12.1218	5.2638	6.3822	0.8719	0.3786	0.2907
141	O	12.1431	5.2521	7.6010	0.8735	0.3778	0.3463
142	O	12.1661	1.7132	6.1560	0.8751	0.1232	0.2804
143	O	1.9925	5.2135	6.3081	0.1433	0.3750	0.2874

/db/jmorales/CoNi-alloy/Profiles/Sm(surf)$Ce_{26}O_{53}$/CO-O

a = 11.6082000732
b = 11.608200073110298
c = 22.898399353
alpha = 90.0
beta = 90.0
gamma = 120.0

	Atom	X	Y	Z	X	Y	Z
1	Sm	1.9374	3.3408	7.1443	0.3331	0.3323	0.3120
2	Ce	1.9347	1.1170	0.7898	0.2222	0.1111	0.0345
3	Ce	0.0000	4.4680	0.7898	0.2222	0.4444	0.0345
4	Ce	-1.9347	7.8190	0.7898	0.2222	0.7778	0.0345
5	Ce	5.8041	1.1170	0.7898	0.5556	0.1111	0.0345
6	Ce	3.8694	4.4680	0.7898	0.5556	0.4444	0.0345
7	Ce	1.9347	7.8190	0.7898	0.5556	0.7778	0.0345
8	Ce	9.6735	1.1170	0.7898	0.8889	0.1111	0.0345
9	Ce	7.7388	4.4680	0.7898	0.8889	0.4444	0.0345
10	Ce	5.8041	7.8190	0.7898	0.8889	0.7778	0.0345
11	Ce	0.0078	2.2390	3.9681	0.1120	0.2227	0.1733
12	Ce	-1.9334	5.5840	3.9467	0.1112	0.5555	0.1724
13	Ce	-3.8691	8.9372	3.9479	0.1112	0.8890	0.1724
14	Ce	3.8625	2.2389	3.9689	0.4441	0.2227	0.1733
15	Ce	1.9354	5.5757	3.9697	0.4440	0.5546	0.1734
16	Ce	0.0014	8.9368	3.9548	0.4446	0.8890	0.1727
17	Ce	7.7394	2.2331	3.9530	0.7778	0.2221	0.1726
18	Ce	5.8038	5.5841	3.9472	0.7777	0.5555	0.1724
19	Ce	3.8692	8.9373	3.9546	0.7778	0.8890	0.1727
20	Ce	0.0059	-0.0023	7.1058	0.0004	-0.0002	0.3103
21	Ce	-1.9367	3.3529	7.1031	-0.0001	0.3335	0.3102
22	Ce	-3.8675	6.7025	7.0905	0.0002	0.6667	0.3097
23	Ce	3.8680	-0.0027	7.1056	0.3331	-0.0003	0.3103
24	Ce	-0.0018	6.7055	7.1029	0.3334	0.6670	0.3102
25	Ce	7.7417	0.0007	7.1150	0.6669	0.0001	0.3107
26	Ce	5.8125	3.3523	7.1035	0.6675	0.3335	0.3102
27	Ce	3.8769	6.7075	7.1035	0.6676	0.6672	0.3102
28	O	0.0000	0.0000	0.0000	0.0000	0.0000	0.0000
29	O	-1.9347	3.3510	0.0000	-0.0000	0.3333	0.0000
30	O	-3.8694	6.7020	0.0000	-0.0000	0.6667	0.0000
31	O	3.8694	0.0000	0.0000	0.3333	0.0000	0.0000
32	O	1.9347	3.3510	0.0000	0.3333	0.3333	0.0000
33	O	0.0000	6.7020	0.0000	0.3333	0.6667	0.0000
34	O	7.7388	0.0000	0.0000	0.6667	0.0000	0.0000
35	O	5.8041	3.3510	0.0000	0.6667	0.3333	0.0000
36	O	3.8694	6.7020	0.0000	0.6667	0.6667	0.0000
37	O	-0.0000	2.2340	1.5798	0.1111	0.2222	0.0690
38	O	-1.9347	5.5850	1.5798	0.1111	0.5556	0.0690
39	O	-3.8694	8.9360	1.5798	0.1111	0.8889	0.0690
40	O	3.8694	2.2340	1.5798	0.4444	0.2222	0.0690

41	O	1.9347	5.5850	1.5798	0.4444	0.5556	0.0690
42	O	-0.0000	8.9360	1.5798	0.4444	0.8889	0.0690
43	O	7.7388	2.2340	1.5798	0.7778	0.2222	0.0690
44	O	5.8041	5.5850	1.5798	0.7778	0.5556	0.0690
45	O	3.8694	8.9360	1.5798	0.7778	0.8889	0.0690
46	O	1.9347	1.1170	3.1593	0.2222	0.1111	0.1380
47	O	0.0000	4.4680	3.1593	0.2222	0.4444	0.1380
48	O	-1.9347	7.8190	3.1593	0.2222	0.7778	0.1380
49	O	5.8041	1.1170	3.1593	0.5556	0.1111	0.1380
50	O	3.8694	4.4680	3.1593	0.5556	0.4444	0.1380
51	O	1.9347	7.8190	3.1593	0.5556	0.7778	0.1380
52	O	9.6735	1.1170	3.1593	0.8889	0.1111	0.1380
53	O	7.7388	4.4680	3.1593	0.8889	0.4444	0.1380
54	O	5.8041	7.8190	3.1593	0.8889	0.7778	0.1380
55	O	0.0026	-0.0005	4.7615	0.0002	-0.0001	0.2079
56	O	-1.9355	3.3535	4.7588	0.0001	0.3336	0.2078
57	O	-3.8692	6.7008	4.7496	-0.0000	0.6665	0.2074
58	O	3.8679	-0.0013	4.7623	0.3331	-0.0001	0.2080
59	O	1.9356	3.3518	4.7483	0.3334	0.3334	0.2074
60	O	-0.0006	6.7019	4.7605	0.3333	0.6667	0.2079
61	O	7.7395	0.0001	4.7615	0.6667	0.0000	0.2079
62	O	5.8067	3.3535	4.7596	0.6670	0.3336	0.2079
63	O	3.8717	6.7016	4.7614	0.6668	0.6666	0.2079
64	O	-0.0278	2.2204	6.3191	0.1080	0.2209	0.2760
65	O	-1.9472	5.5933	6.2807	0.1104	0.5564	0.2743
66	O	-3.8693	8.9152	6.2817	0.1101	0.8868	0.2743
67	O	3.9014	2.2213	6.3192	0.4466	0.2210	0.2760
68	O	0.0048	8.9369	6.3017	0.4449	0.8890	0.2752
69	O	7.7407	2.2312	6.2997	0.7778	0.2219	0.2751
70	O	5.8202	5.5937	6.2815	0.7796	0.5564	0.2743
71	O	3.8696	8.9382	6.3004	0.7779	0.8891	0.2751
72	O	1.9398	1.0017	7.9097	0.2169	0.0996	0.3454
73	O	-0.0999	4.5306	7.9174	0.2167	0.4507	0.3458
74	O	-1.9357	7.8168	7.8915	0.2220	0.7776	0.3446
75	O	5.8191	1.1111	7.9078	0.5566	0.1105	0.3453
76	O	3.9804	4.5322	7.9247	0.5683	0.4508	0.3461
77	O	1.9389	7.8312	7.9093	0.5565	0.7790	0.3454
78	O	9.6631	1.1113	7.9080	0.8877	0.1105	0.3453
79	O	7.7418	4.4709	7.8906	0.8893	0.4447	0.3446
80	O	5.8081	7.8171	7.8922	0.8891	0.7776	0.3447
81	C	2.0508	3.7900	9.9189	0.3652	0.3770	0.4332
82	O	2.1472	4.1871	10.9860	0.3932	0.4165	0.4798
83	O	1.9368	5.6167	6.3217	0.4462	0.5587	0.2761

/db/jmorales/CoNi-alloy/Profiles/CoNi-Ce_3SmO_7/TS-2

a =	13.903338426
b =	13.903338426
c =	21.951669213
alpha =	90.0
beta =	90.0
gamma =	90.0

	Atom	X	Y	Z	X	Y	Z
1	Ni	0.0000	0.0000	0.0000	0.0000	0.0000	0.0000
2	Ni	0.0000	3.4758	0.0000	0.0000	0.2500	0.0000
3	Ni	3.4758	0.0000	0.0000	0.2500	0.0000	0.0000
4	Ni	3.4758	3.4758	0.0000	0.2500	0.2500	0.0000
5	Ni	0.0000	6.9517	0.0000	0.0000	0.5000	0.0000
6	Ni	0.0000	10.4275	0.0000	0.0000	0.7500	0.0000
7	Ni	3.4758	6.9517	0.0000	0.2500	0.5000	0.0000
8	Ni	3.4758	10.4275	0.0000	0.2500	0.7500	0.0000
9	Ni	6.9517	0.0000	0.0000	0.5000	0.0000	0.0000
10	Ni	6.9517	3.4758	0.0000	0.5000	0.2500	0.0000
11	Ni	10.4275	0.0000	0.0000	0.7500	0.0000	0.0000
12	Ni	10.4275	3.4758	0.0000	0.7500	0.2500	0.0000
13	Ni	6.9517	6.9517	0.0000	0.5000	0.5000	0.0000
14	Ni	6.9517	10.4275	0.0000	0.5000	0.7500	0.0000
15	Ni	10.4275	6.9517	0.0000	0.7500	0.5000	0.0000
16	Ni	10.4275	10.4275	0.0000	0.7500	0.7500	0.0000
17	Ni	0.0000	1.7379	1.7379	0.0000	0.1250	0.0792
18	Ni	0.0000	5.2138	1.7379	0.0000	0.3750	0.0792
19	Ni	3.4758	1.7379	1.7379	0.2500	0.1250	0.0792
20	Ni	3.4758	5.2138	1.7379	0.2500	0.3750	0.0792
21	Ni	0.0000	8.6896	1.7379	0.0000	0.6250	0.0792
22	Ni	0.0000	12.1654	1.7379	0.0000	0.8750	0.0792
23	Ni	3.4758	8.6896	1.7379	0.2500	0.6250	0.0792
24	Ni	3.4758	12.1654	1.7379	0.2500	0.8750	0.0792
25	Ni	6.9517	1.7379	1.7379	0.5000	0.1250	0.0792
26	Ni	6.9517	5.2138	1.7379	0.5000	0.3750	0.0792
27	Ni	10.4275	1.7379	1.7379	0.7500	0.1250	0.0792
28	Ni	10.4275	5.2138	1.7379	0.7500	0.3750	0.0792
29	Ni	6.9517	8.6896	1.7379	0.5000	0.6250	0.0792
30	Ni	6.9517	12.1654	1.7379	0.5000	0.8750	0.0792
31	Ni	10.4275	8.6896	1.7379	0.7500	0.6250	0.0792
32	Ni	10.4275	12.1654	1.7379	0.7500	0.8750	0.0792
33	Ni	13.8923	0.0031	3.5130	0.9992	0.0002	0.1600
34	Ni	0.0022	3.4769	3.5145	0.0002	0.2501	0.1601
35	Ni	3.4937	13.9032	3.5196	0.2513	1.0000	0.1603
36	Ni	3.4704	3.4503	3.4868	0.2496	0.2482	0.1588
37	Ni	0.0103	6.9677	3.5332	0.0007	0.5012	0.1610
38	Ni	13.9018	10.4410	3.5229	0.9999	0.7510	0.1605
39	Ni	3.4759	6.9589	3.5117	0.2500	0.5005	0.1600
40	Ni	3.4981	10.3931	3.5573	0.2516	0.7475	0.1621

41	Ni	6.9418	0.0185	3.5224	0.4993	0.0013	0.1605
42	Ni	6.9522	3.4974	3.5431	0.5000	0.2516	0.1614
43	Ni	10.4176	0.0035	3.5176	0.7493	0.0003	0.1602
44	Ni	10.4329	3.4812	3.5159	0.7504	0.2504	0.1602
45	Ni	6.9756	6.9239	3.4783	0.5017	0.4980	0.1585
46	Ni	6.9638	10.4409	3.4806	0.5009	0.7510	0.1586
47	Ni	10.4469	6.9420	3.5028	0.7514	0.4993	0.1596
48	Ni	10.4295	10.4414	3.5155	0.7501	0.7510	0.1601
49	Ni	13.8994	1.7498	5.2292	0.9997	0.1259	0.2382
50	Ni	13.9008	5.2105	5.2382	0.9998	0.3748	0.2386
51	Ni	3.4505	1.7563	5.2225	0.2482	0.1263	0.2379
52	Ni	3.4637	5.1783	5.1149	0.2491	0.3725	0.2330
53	Ni	13.8755	8.7297	5.2322	0.9980	0.6279	0.2383
54	Ni	13.8662	12.1865	5.2132	0.9973	0.8765	0.2375
55	Ni	3.4686	8.6564	5.2798	0.2495	0.6226	0.2405
56	Ni	3.4585	12.1847	5.1923	0.2488	0.8764	0.2365
57	Ni	6.9736	1.7629	5.2353	0.5016	0.1268	0.2385
58	Ni	6.9540	5.1566	5.4120	0.5002	0.3709	0.2465
59	Ni	10.4205	1.7406	5.2227	0.7495	0.1252	0.2379
60	Ni	10.4364	5.2168	5.2323	0.7506	0.3752	0.2384
61	Ni	7.0477	8.6743	5.0875	0.5069	0.6239	0.2318
62	Ni	6.9738	12.1775	5.1416	0.5016	0.8759	0.2342
63	Ni	10.4195	8.6950	5.2232	0.7494	0.6254	0.2379
64	Ni	10.4313	12.1867	5.2834	0.7503	0.8765	0.2407
65	Co	1.7379	1.7379	0.0000	0.1250	0.1250	0.0000
66	Co	1.7379	5.2138	0.0000	0.1250	0.3750	0.0000
67	Co	5.2138	1.7379	0.0000	0.3750	0.1250	0.0000
68	Co	5.2138	5.2138	0.0000	0.3750	0.3750	0.0000
69	Co	1.7379	8.6896	0.0000	0.1250	0.6250	0.0000
70	Co	1.7379	12.1654	0.0000	0.1250	0.8750	0.0000
71	Co	5.2138	8.6896	0.0000	0.3750	0.6250	0.0000
72	Co	5.2138	12.1654	0.0000	0.3750	0.8750	0.0000
73	Co	8.6896	1.7379	0.0000	0.6250	0.1250	0.0000
74	Co	8.6896	5.2138	0.0000	0.6250	0.3750	0.0000
75	Co	12.1654	1.7379	0.0000	0.8750	0.1250	0.0000
76	Co	12.1654	5.2138	0.0000	0.8750	0.3750	0.0000
77	Co	8.6896	8.6896	0.0000	0.6250	0.6250	0.0000
78	Co	8.6896	12.1654	0.0000	0.6250	0.8750	0.0000
79	Co	12.1654	8.6896	0.0000	0.8750	0.6250	0.0000
80	Co	12.1654	12.1654	0.0000	0.8750	0.8750	0.0000
81	Co	1.7379	0.0000	1.7379	0.1250	0.0000	0.0792
82	Co	1.7379	3.4758	1.7379	0.1250	0.2500	0.0792
83	Co	5.2138	0.0000	1.7379	0.3750	0.0000	0.0792
84	Co	5.2138	3.4758	1.7379	0.3750	0.2500	0.0792
85	Co	1.7379	6.9517	1.7379	0.1250	0.5000	0.0792
86	Co	1.7379	10.4275	1.7379	0.1250	0.7500	0.0792
87	Co	5.2138	6.9517	1.7379	0.3750	0.5000	0.0792
88	Co	5.2138	10.4275	1.7379	0.3750	0.7500	0.0792
89	Co	8.6896	0.0000	1.7379	0.6250	0.0000	0.0792

90	Co	8.6896	3.4758	1.7379	0.6250	0.2500	0.0792
91	Co	12.1654	0.0000	1.7379	0.8750	0.0000	0.0792
92	Co	12.1654	3.4758	1.7379	0.8750	0.2500	0.0792
93	Co	8.6896	6.9517	1.7379	0.6250	0.5000	0.0792
94	Co	8.6896	10.4275	1.7379	0.6250	0.7500	0.0792
95	Co	12.1654	6.9517	1.7379	0.8750	0.5000	0.0792
96	Co	12.1654	10.4275	1.7379	0.8750	0.7500	0.0792
97	Co	1.7327	1.7307	3.5640	0.1246	0.1245	0.1624
98	Co	1.7096	5.2358	3.5464	0.1230	0.3766	0.1616
99	Co	5.2168	1.7236	3.5748	0.3752	0.1240	0.1628
100	Co	5.2432	5.2019	3.5482	0.3771	0.3741	0.1616
101	Co	1.7323	8.7216	3.5904	0.1246	0.6273	0.1636
102	Co	1.7302	12.1757	3.5194	0.1244	0.8757	0.1603
103	Co	5.2051	8.6770	3.5637	0.3744	0.6241	0.1623
104	Co	5.2172	12.1648	3.5879	0.3752	0.8750	0.1634
105	Co	8.6880	1.7415	3.5555	0.6249	0.1253	0.1620
106	Co	8.6750	5.2009	3.5869	0.6240	0.3741	0.1634
107	Co	12.1628	1.7421	3.5668	0.8748	0.1253	0.1625
108	Co	12.1741	5.2145	3.5748	0.8756	0.3751	0.1628
109	Co	8.7354	8.6953	3.5056	0.6283	0.6254	0.1597
110	Co	8.7164	12.1743	3.4721	0.6269	0.8756	0.1582
111	Co	12.1627	8.6940	3.5675	0.8748	0.6253	0.1625
112	Co	12.1364	12.1770	3.5816	0.8729	0.8758	0.1632
113	Co	1.7096	0.0203	5.2282	0.1230	0.0015	0.2382
114	Co	1.7401	3.4866	5.2455	0.1252	0.2508	0.2390
115	Co	5.2219	0.0218	5.3409	0.3756	0.0016	0.2433
116	Co	5.1733	3.4316	5.2262	0.3721	0.2468	0.2381
117	Co	1.6909	6.9522	5.3420	0.1216	0.5000	0.2434
118	Co	1.7092	10.5171	5.2634	0.1229	0.7564	0.2398
119	Co	5.2887	6.9364	5.2002	0.3804	0.4989	0.2369
120	Co	5.3124	10.3498	5.2884	0.3821	0.7444	0.2409
121	Co	8.6855	0.0196	5.3002	0.6247	0.0014	0.2414
122	Co	8.7195	3.4646	5.2612	0.6272	0.2492	0.2397
123	Co	12.1685	0.0270	5.2516	0.8752	0.0019	0.2392
124	Co	12.1628	3.4676	5.2580	0.8748	0.2494	0.2395
125	Co	8.7385	6.9682	5.1992	0.6285	0.5012	0.2368
126	Co	8.6976	10.4391	5.2783	0.6256	0.7508	0.2405
127	Co	12.1680	6.9611	5.2599	0.8752	0.5007	0.2396
128	Co	12.1582	10.4322	5.2476	0.8745	0.7503	0.2391
129	O	6.0154	6.1633	6.8412	0.4327	0.4433	0.3116
130	O	2.5567	7.8194	6.8894	0.1839	0.5624	0.3138
131	O	4.6964	7.9131	8.7867	0.3378	0.5692	0.4003
132	O	5.5670	10.1993	7.2254	0.4004	0.7336	0.3291
133	O	7.7916	10.0450	8.8895	0.5604	0.7225	0.4050
134	O	4.4645	12.0776	9.0342	0.3211	0.8687	0.4115
135	O	8.6109	12.2008	6.3744	0.6193	0.8775	0.2904
136	Ce	3.4402	9.7518	7.9694	0.2474	0.7014	0.3630
137	Ce	3.8772	6.1145	7.6941	0.2789	0.4398	0.3505
138	Ce	6.8562	8.2764	7.9288	0.4931	0.5953	0.3612

139 Sm	7.0007	11.7391	7.9818	0.5035	0.8443	0.3636
140 C	4.1297	12.6618	7.9488	0.2970	0.9107	0.3621
141 O	3.0088	11.9264	7.0782	0.2164	0.8578	0.3224
142 O	4.8477	13.5258	7.3654	0.3487	0.9728	0.3355

/db/jmorales/CoNi-alloy/Profiles/Ni-Ce_3SmO_7/O-coverage/M-CO-O-O-coverage/CO-O-Ni-Ce_3SmO_7-6O

a =				13.9019837372			
b =				13.9019837372			
c =				21.9509918687			
alpha =				90.0			
beta =				90.0			
gamma =				90.0			
	Atom	X	Y	Z	X	Y	Z
1	Ni	0.0000	0.0000	0.0000	0.0000	0.0000	0.0000
2	Ni	1.7377	1.7377	0.0000	0.1250	0.1250	0.0000
3	Ni	0.0000	3.4755	0.0000	0.0000	0.2500	0.0000
4	Ni	1.7377	5.2132	0.0000	0.1250	0.3750	0.0000
5	Ni	3.4755	0.0000	0.0000	0.2500	0.0000	0.0000
6	Ni	5.2132	1.7377	0.0000	0.3750	0.1250	0.0000
7	Ni	3.4755	3.4755	0.0000	0.2500	0.2500	0.0000
8	Ni	5.2132	5.2132	0.0000	0.3750	0.3750	0.0000
9	Ni	0.0000	6.9510	0.0000	0.0000	0.5000	0.0000
10	Ni	1.7377	8.6887	0.0000	0.1250	0.6250	0.0000
11	Ni	0.0000	10.4265	0.0000	0.0000	0.7500	0.0000
12	Ni	1.7377	12.1642	0.0000	0.1250	0.8750	0.0000
13	Ni	3.4755	6.9510	0.0000	0.2500	0.5000	0.0000
14	Ni	5.2132	8.6887	0.0000	0.3750	0.6250	0.0000
15	Ni	3.4755	10.4265	0.0000	0.2500	0.7500	0.0000
16	Ni	5.2132	12.1642	0.0000	0.3750	0.8750	0.0000
17	Ni	6.9510	0.0000	0.0000	0.5000	0.0000	0.0000
18	Ni	8.6887	1.7377	0.0000	0.6250	0.1250	0.0000
19	Ni	6.9510	3.4755	0.0000	0.5000	0.2500	0.0000
20	Ni	8.6887	5.2132	0.0000	0.6250	0.3750	0.0000
21	Ni	10.4265	0.0000	0.0000	0.7500	0.0000	0.0000
22	Ni	12.1642	1.7377	0.0000	0.8750	0.1250	0.0000
23	Ni	10.4265	3.4755	0.0000	0.7500	0.2500	0.0000
24	Ni	12.1642	5.2132	0.0000	0.8750	0.3750	0.0000
25	Ni	6.9510	6.9510	0.0000	0.5000	0.5000	0.0000
26	Ni	8.6887	8.6887	0.0000	0.6250	0.6250	0.0000
27	Ni	6.9510	10.4265	0.0000	0.5000	0.7500	0.0000
28	Ni	8.6887	12.1642	0.0000	0.6250	0.8750	0.0000
29	Ni	10.4265	6.9510	0.0000	0.7500	0.5000	0.0000
30	Ni	12.1642	8.6887	0.0000	0.8750	0.6250	0.0000
31	Ni	10.4265	10.4265	0.0000	0.7500	0.7500	0.0000
32	Ni	12.1642	12.1642	0.0000	0.8750	0.8750	0.0000
33	Ni	0.0000	1.7377	1.7377	0.0000	0.1250	0.0792
34	Ni	1.7377	0.0000	1.7377	0.1250	0.0000	0.0792
35	Ni	0.0000	5.2132	1.7377	0.0000	0.3750	0.0792
36	Ni	1.7377	3.4755	1.7377	0.1250	0.2500	0.0792
37	Ni	3.4755	1.7377	1.7377	0.2500	0.1250	0.0792
38	Ni	5.2132	0.0000	1.7377	0.3750	0.0000	0.0792
39	Ni	3.4755	5.2132	1.7377	0.2500	0.3750	0.0792

40	Ni	5.2132	3.4755	1.7377	0.3750	0.2500	0.0792
41	Ni	0.0000	8.6887	1.7377	0.0000	0.6250	0.0792
42	Ni	1.7377	6.9510	1.7377	0.1250	0.5000	0.0792
43	Ni	0.0000	12.1642	1.7377	0.0000	0.8750	0.0792
44	Ni	1.7377	10.4265	1.7377	0.1250	0.7500	0.0792
45	Ni	3.4755	8.6887	1.7377	0.2500	0.6250	0.0792
46	Ni	5.2132	6.9510	1.7377	0.3750	0.5000	0.0792
47	Ni	3.4755	12.1642	1.7377	0.2500	0.8750	0.0792
48	Ni	5.2132	10.4265	1.7377	0.3750	0.7500	0.0792
49	Ni	6.9510	1.7377	1.7377	0.5000	0.1250	0.0792
50	Ni	8.6887	0.0000	1.7377	0.6250	0.0000	0.0792
51	Ni	6.9510	5.2132	1.7377	0.5000	0.3750	0.0792
52	Ni	8.6887	3.4755	1.7377	0.6250	0.2500	0.0792
53	Ni	10.4265	1.7377	1.7377	0.7500	0.1250	0.0792
54	Ni	12.1642	0.0000	1.7377	0.8750	0.0000	0.0792
55	Ni	10.4265	5.2132	1.7377	0.7500	0.3750	0.0792
56	Ni	12.1642	3.4755	1.7377	0.8750	0.2500	0.0792
57	Ni	6.9510	8.6887	1.7377	0.5000	0.6250	0.0792
58	Ni	8.6887	6.9510	1.7377	0.6250	0.5000	0.0792
59	Ni	6.9510	12.1642	1.7377	0.5000	0.8750	0.0792
60	Ni	8.6887	10.4265	1.7377	0.6250	0.7500	0.0792
61	Ni	10.4265	8.6887	1.7377	0.7500	0.6250	0.0792
62	Ni	12.1642	6.9510	1.7377	0.8750	0.5000	0.0792
63	Ni	10.4265	12.1642	1.7377	0.7500	0.8750	0.0792
64	Ni	12.1642	10.4265	1.7377	0.8750	0.7500	0.0792
65	Ni	0.0094	0.0142	3.5020	0.0007	0.0010	0.1595
66	Ni	1.7410	1.7692	3.4823	0.1252	0.1273	0.1586
67	Ni	0.0060	3.4745	3.5010	0.0004	0.2499	0.1595
68	Ni	1.6967	5.2147	3.4518	0.1220	0.3751	0.1573
69	Ni	3.4597	13.8942	3.5055	0.2489	0.9994	0.1597
70	Ni	5.2159	1.7427	3.4779	0.3752	0.1254	0.1584
71	Ni	3.4900	3.4438	3.4784	0.2510	0.2477	0.1585
72	Ni	5.2218	5.2031	3.4746	0.3756	0.3743	0.1583
73	Ni	13.8998	6.9494	3.4931	0.9998	0.4999	0.1591
74	Ni	1.7423	8.6654	3.4690	0.1253	0.6233	0.1580
75	Ni	13.8702	10.4172	3.5184	0.9977	0.7493	0.1603
76	Ni	1.7122	12.1475	3.5395	0.1232	0.8738	0.1612
77	Ni	3.4710	6.9843	3.4616	0.2497	0.5024	0.1577
78	Ni	5.2294	8.7119	3.5073	0.3762	0.6267	0.1598
79	Ni	3.4617	10.4041	3.5200	0.2490	0.7484	0.1604
80	Ni	5.2172	12.1669	3.5008	0.3753	0.8752	0.1595
81	Ni	6.9503	0.0000	3.5046	0.4999	0.0000	0.1597
82	Ni	8.6869	1.7362	3.4485	0.6249	0.1249	0.1571
83	Ni	6.9477	3.4592	3.4966	0.4998	0.2488	0.1593
84	Ni	8.6699	5.2137	3.5392	0.6236	0.3750	0.1612
85	Ni	10.4446	0.0232	3.5060	0.7513	0.0017	0.1597
86	Ni	12.1718	1.7454	3.4693	0.8755	0.1256	0.1580
87	Ni	10.4191	3.4730	3.4964	0.7495	0.2498	0.1593
88	Ni	12.1614	5.1962	3.4665	0.8748	0.3738	0.1579

89	Ni	6.9429	6.9435	3.5076	0.4994	0.4995	0.1598
90	Ni	8.6910	8.6847	3.4750	0.6252	0.6247	0.1583
91	Ni	6.9984	10.4297	3.5231	0.5034	0.7502	0.1605
92	Ni	8.7218	12.1934	3.5218	0.6274	0.8771	0.1604
93	Ni	10.3954	6.9404	3.4983	0.7478	0.4992	0.1594
94	Ni	12.1750	8.6818	3.5061	0.8758	0.6245	0.1597
95	Ni	10.4446	10.4379	3.4490	0.7513	0.7508	0.1571
96	Ni	12.1669	12.1820	3.4448	0.8752	0.8763	0.1569
97	Ni	0.0228	1.8149	5.3621	0.0016	0.1306	0.2443
98	Ni	1.7200	13.8967	5.2738	0.1237	0.9996	0.2403
99	Ni	13.8289	5.2098	5.2556	0.9947	0.3748	0.2394
100	Ni	1.7611	3.5460	5.3575	0.1267	0.2551	0.2441
101	Ni	3.4712	1.7239	5.3633	0.2497	0.1240	0.2443
102	Ni	5.2027	13.8547	5.2719	0.3742	0.9966	0.2402
103	Ni	3.4774	5.2094	5.0434	0.2501	0.3747	0.2298
104	Ni	5.1934	3.4749	5.3554	0.3736	0.2500	0.2440
105	Ni	13.8834	8.6577	5.2612	0.9987	0.6228	0.2397
106	Ni	1.6960	6.8994	5.3387	0.1220	0.4963	0.2432
107	Ni	13.8769	12.1428	5.2589	0.9982	0.8735	0.2396
108	Ni	1.7143	10.4208	5.2863	0.1233	0.7496	0.2408
109	Ni	3.4632	8.7234	5.3957	0.2491	0.6275	0.2458
110	Ni	5.2086	6.9725	5.1938	0.3747	0.5015	0.2366
111	Ni	3.4628	12.1378	5.1960	0.2491	0.8731	0.2367
112	Ni	5.2331	10.4525	5.1837	0.3764	0.7519	0.2361
113	Ni	6.9242	1.7065	5.3778	0.4981	0.1228	0.2450
114	Ni	8.6909	0.0286	5.2568	0.6252	0.0021	0.2395
115	Ni	6.9315	5.1874	5.2540	0.4986	0.3731	0.2394
116	Ni	8.7012	3.4482	5.2415	0.6259	0.2480	0.2388
117	Ni	10.4479	1.7498	5.3793	0.7515	0.1259	0.2451
118	Ni	12.2218	0.0583	5.3569	0.8791	0.0042	0.2440
119	Ni	10.4353	5.2187	5.2594	0.7506	0.3754	0.2396
120	Ni	12.1421	3.5200	5.3185	0.8734	0.2532	0.2423
121	Ni	6.9941	8.7530	5.2591	0.5031	0.6296	0.2396
122	Ni	8.6776	6.9267	5.2907	0.6242	0.4982	0.2410
123	Ni	6.9744	12.1501	5.2049	0.5017	0.8740	0.2371
124	Ni	8.7829	10.4874	5.2548	0.6318	0.7544	0.2394
125	Ni	10.4482	8.6038	5.1827	0.7516	0.6189	0.2361
126	Ni	12.1494	6.9146	5.2253	0.8739	0.4974	0.2380
127	Ni	10.4843	12.2611	5.2677	0.7542	0.8820	0.2400
128	Ni	12.1002	10.4000	5.3485	0.8704	0.7481	0.2437
129	O	6.0676	4.5112	6.8296	0.4365	0.3245	0.3111
130	O	4.2891	7.5915	6.7610	0.3085	0.5461	0.3080
131	O	5.9304	6.6410	8.8660	0.4266	0.4777	0.4039
132	O	7.8141	7.8644	6.8444	0.5621	0.5657	0.3118
133	O	10.3130	10.0468	6.3610	0.7418	0.7227	0.2898
134	O	9.4347	6.9564	9.0160	0.6787	0.5004	0.4107
135	O	7.6224	9.7878	8.9950	0.5483	0.7041	0.4098
136	Ce	5.9120	8.8572	8.0795	0.4253	0.6371	0.3681
137	Ce	4.2515	5.5311	7.7586	0.3058	0.3979	0.3534

138	Ce	7.9527	5.7366	8.1068	0.5721	0.4126	0.3693
139	Sm	9.4050	8.9469	8.2301	0.6765	0.6436	0.3749
140	C	12.1228	5.2845	6.3672	0.8720	0.3801	0.2901
141	O	12.1571	5.2705	7.5839	0.8745	0.3791	0.3455
142	O	12.1814	1.8187	6.1991	0.8762	0.1308	0.2824
143	O	2.1475	5.2522	6.3990	0.1545	0.3778	0.2915
144	O	1.7421	1.6765	6.2034	0.1253	0.1206	0.2826
145	O	8.6771	1.7535	6.1822	0.6242	0.1261	0.2816
146	O	1.5961	8.7607	6.2153	0.1148	0.6302	0.2831
147	O	12.2295	12.2116	6.1899	0.8797	0.8784	0.2820
148	O	5.1950	1.5994	6.2105	0.3737	0.1150	0.2829

/db/jmorales/CoNi-alloy/Profiles/CoNi(001)/CO_2

a =			6.951669213				
b =			6.951669213				
c =			21.951669213				
alpha =			90.0				
beta =			90.0				
gamma =			90.0				
	Atom	X	Y	Z	X	Y	Z
1	Ni	0.0000	0.0000	0.0000	0.0000	0.0000	0.0000
2	Ni	0.0000	3.4758	0.0000	0.0000	0.5000	0.0000
3	Ni	3.4758	0.0000	0.0000	0.5000	0.0000	0.0000
4	Ni	3.4758	3.4758	0.0000	0.5000	0.5000	0.0000
5	Ni	0.0000	1.7379	1.7379	0.0000	0.2500	0.0792
6	Ni	0.0000	5.2138	1.7379	0.0000	0.7500	0.0792
7	Ni	3.4758	1.7379	1.7379	0.5000	0.2500	0.0792
8	Ni	3.4758	5.2138	1.7379	0.5000	0.7500	0.0792
9	Ni	0.0019	6.9513	3.5051	0.0003	0.9999	0.1597
10	Ni	6.9515	3.4750	3.5015	1.0000	0.4999	0.1595
11	Ni	3.4736	0.0017	3.5039	0.4997	0.0002	0.1596
12	Ni	3.4775	3.4743	3.4928	0.5002	0.4998	0.1591
13	Ni	6.9290	1.7197	5.1737	0.9967	0.2474	0.2357
14	Ni	6.9389	5.2259	5.1747	0.9982	0.7518	0.2357
15	Ni	3.4484	1.7599	5.2570	0.4960	0.2532	0.2395
16	Ni	3.4966	5.2364	5.1730	0.5030	0.7533	0.2357
17	Co	1.7379	1.7379	0.0000	0.2500	0.2500	0.0000
18	Co	1.7379	5.2138	0.0000	0.2500	0.7500	0.0000
19	Co	5.2138	1.7379	0.0000	0.7500	0.2500	0.0000
20	Co	5.2138	5.2138	0.0000	0.7500	0.7500	0.0000
21	Co	1.7379	0.0000	1.7379	0.2500	0.0000	0.0792
22	Co	1.7379	3.4758	1.7379	0.2500	0.5000	0.0792
23	Co	5.2138	0.0000	1.7379	0.7500	0.0000	0.0792
24	Co	5.2138	3.4758	1.7379	0.7500	0.5000	0.0792
25	Co	1.7397	1.7316	3.5230	0.2503	0.2491	0.1605
26	Co	1.7368	5.2202	3.5313	0.2498	0.7509	0.1609
27	Co	5.2200	1.7405	3.5334	0.7509	0.2504	0.1610
28	Co	5.2146	5.2124	3.5386	0.7501	0.7498	0.1612
29	Co	1.7336	6.9480	5.1904	0.2494	0.9995	0.2364
30	Co	1.7304	3.4844	5.1619	0.2489	0.5012	0.2351
31	Co	5.2154	6.9509	5.1971	0.7502	0.9999	0.2368
32	Co	5.2194	3.4807	5.1901	0.7508	0.5007	0.2364
33	C	3.5112	4.1099	6.7524	0.5051	0.5912	0.3076
34	O	4.6045	3.9090	7.2203	0.6624	0.5623	0.3289
35	O	2.2793	3.9097	7.0774	0.3279	0.5624	0.3224

/db/jmorales/CoNi-alloy/Profiles/CoNi(001)/CO-O

a =			6.951669213				
b =			6.951669213				
c =			21.951669213				
alpha =			90.0				
beta =			90.0				
gamma =			90.0				
	Atom	X	Y	Z	X	Y	Z
1	Ni	0.0000	0.0000	0.0000	0.0000	0.0000	0.0000
2	Ni	0.0000	3.4758	0.0000	0.0000	0.5000	0.0000
3	Ni	3.4758	0.0000	0.0000	0.5000	0.0000	0.0000
4	Ni	3.4758	3.4758	0.0000	0.5000	0.5000	0.0000
5	Ni	0.0000	1.7379	1.7379	0.0000	0.2500	0.0792
6	Ni	0.0000	5.2138	1.7379	0.0000	0.7500	0.0792
7	Ni	3.4758	1.7379	1.7379	0.5000	0.2500	0.0792
8	Ni	3.4758	5.2138	1.7379	0.5000	0.7500	0.0792
9	Ni	6.9504	0.0005	3.5030	0.9998	0.0001	0.1596
10	Ni	6.9486	3.4759	3.4943	0.9996	0.5000	0.1592
11	Ni	3.4742	0.0013	3.5227	0.4998	0.0002	0.1605
12	Ni	3.4747	3.4760	3.4514	0.4998	0.5000	0.1572
13	Ni	6.9500	1.7358	5.2028	0.9998	0.2497	0.2370
14	Ni	6.9498	5.2171	5.2019	0.9997	0.7505	0.2370
15	Ni	3.4726	1.7625	5.2361	0.4995	0.2535	0.2385
16	Ni	3.4729	5.1928	5.2339	0.4996	0.7470	0.2384
17	Co	1.7379	1.7379	0.0000	0.2500	0.2500	0.0000
18	Co	1.7379	5.2138	0.0000	0.2500	0.7500	0.0000
19	Co	5.2138	1.7379	0.0000	0.7500	0.2500	0.0000
20	Co	5.2138	5.2138	0.0000	0.7500	0.7500	0.0000
21	Co	1.7379	0.0000	1.7379	0.2500	0.0000	0.0792
22	Co	1.7379	3.4758	1.7379	0.2500	0.5000	0.0792
23	Co	5.2138	0.0000	1.7379	0.7500	0.0000	0.0792
24	Co	5.2138	3.4758	1.7379	0.7500	0.5000	0.0792
25	Co	1.7341	1.7378	3.5545	0.2494	0.2500	0.1619
26	Co	1.7340	5.2157	3.5528	0.2494	0.7503	0.1618
27	Co	5.2138	1.7381	3.5555	0.7500	0.2500	0.1620
28	Co	5.2139	5.2142	3.5544	0.7500	0.7501	0.1619
29	Co	1.7332	0.0005	5.2163	0.2493	0.0001	0.2376
30	Co	1.7379	3.4785	5.2450	0.2500	0.5004	0.2389
31	Co	5.2149	0.0004	5.2152	0.7502	0.0001	0.2376
32	Co	5.2066	3.4773	5.2503	0.7490	0.5002	0.2392
33	O	3.4598	3.4815	7.5730	0.4977	0.5008	0.3450
34	C	3.4727	3.4773	6.3578	0.4995	0.5002	0.2896
35	O	5.2000	3.5000	7.3000	0.7480	0.5035	0.3325

/db/jmorales/CoNi-alloy/Profiles/Sm(subsurf)$Ce_{26}O_{54}$/surface

a =	11.6082000732
b =	11.608200073110298
c =	22.898399353
alpha =	90.0
beta =	90.0
gamma =	120.0

	Atom	X	Y	Z	X	Y	Z
1	Sm	1.9347	5.5850	3.9681	0.4444	0.5556	0.1733
2	Ce	1.9347	1.1170	0.7898	0.2222	0.1111	0.0345
3	Ce	0.0000	4.4680	0.7898	0.2222	0.4444	0.0345
4	Ce	-1.9347	7.8190	0.7898	0.2222	0.7778	0.0345
5	Ce	5.8041	1.1170	0.7898	0.5556	0.1111	0.0345
6	Ce	3.8694	4.4680	0.7898	0.5556	0.4444	0.0345
7	Ce	1.9347	7.8190	0.7898	0.5556	0.7778	0.0345
8	Ce	9.6735	1.1170	0.7898	0.8889	0.1111	0.0345
9	Ce	7.7388	4.4680	0.7898	0.8889	0.4444	0.0345
10	Ce	5.8041	7.8190	0.7898	0.8889	0.7778	0.0345
11	Ce	0.0068	2.2492	3.9581	0.1125	0.2237	0.1729
12	Ce	-1.9181	5.5833	3.9581	0.1125	0.5554	0.1729
13	Ce	-3.8694	8.9360	3.9588	0.1111	0.8889	0.1729
14	Ce	3.8626	2.2492	3.9581	0.4446	0.2237	0.1729
15	Ce	0.0098	8.9225	3.9581	0.4446	0.8875	0.1729
16	Ce	7.7388	2.2340	3.9538	0.7778	0.2222	0.1727
17	Ce	5.7875	5.5833	3.9581	0.7763	0.5554	0.1729
18	Ce	3.8596	8.9225	3.9581	0.7763	0.8875	0.1729
19	Ce	-0.0033	0.0019	7.1001	-0.0002	0.0002	0.3101
20	Ce	-1.9359	3.3503	7.1130	-0.0001	0.3333	0.3106
21	Ce	-3.8694	6.6981	7.1001	-0.0002	0.6663	0.3101
22	Ce	3.8727	0.0019	7.1001	0.3337	0.0002	0.3101
23	Ce	1.9347	3.3547	7.1029	0.3335	0.3337	0.3102
24	Ce	0.0033	6.7002	7.1029	0.3335	0.6665	0.3102
25	Ce	7.7388	0.0016	7.1130	0.6667	0.0002	0.3106
26	Ce	5.8053	3.3503	7.1130	0.6667	0.3333	0.3106
27	Ce	3.8661	6.7002	7.1029	0.6663	0.6665	0.3102
28	O	0.0000	0.0000	0.0000	0.0000	0.0000	0.0000
29	O	-1.9347	3.3510	0.0000	-0.0000	0.3333	0.0000
30	O	-3.8694	6.7020	0.0000	-0.0000	0.6667	0.0000
31	O	3.8694	0.0000	0.0000	0.3333	0.0000	0.0000
32	O	1.9347	3.3510	0.0000	0.3333	0.3333	0.0000
33	O	0.0000	6.7020	0.0000	0.3333	0.6667	0.0000
34	O	7.7388	0.0000	0.0000	0.6667	0.0000	0.0000
35	O	5.8041	3.3510	0.0000	0.6667	0.3333	0.0000
36	O	3.8694	6.7020	0.0000	0.6667	0.6667	0.0000
37	O	-0.0000	2.2340	1.5798	0.1111	0.2222	0.0690
38	O	-1.9347	5.5850	1.5798	0.1111	0.5556	0.0690
39	O	-3.8694	8.9360	1.5798	0.1111	0.8889	0.0690
40	O	3.8694	2.2340	1.5798	0.4444	0.2222	0.0690

41	O	1.9347	5.5850	1.5798	0.4444	0.5556	0.0690
42	O	-0.0000	8.9360	1.5798	0.4444	0.8889	0.0690
43	O	7.7388	2.2340	1.5798	0.7778	0.2222	0.0690
44	O	5.8041	5.5850	1.5798	0.7778	0.5556	0.0690
45	O	3.8694	8.9360	1.5798	0.7778	0.8889	0.0690
46	O	1.9347	1.1170	3.1593	0.2222	0.1111	0.1380
47	O	0.0000	4.4680	3.1593	0.2222	0.4444	0.1380
48	O	-1.9347	7.8190	3.1593	0.2222	0.7778	0.1380
49	O	5.8041	1.1170	3.1593	0.5556	0.1111	0.1380
50	O	3.8694	4.4680	3.1593	0.5556	0.4444	0.1380
51	O	1.9347	7.8190	3.1593	0.5556	0.7778	0.1380
52	O	9.6735	1.1170	3.1593	0.8889	0.1111	0.1380
53	O	7.7388	4.4680	3.1593	0.8889	0.4444	0.1380
54	O	5.8041	7.8190	3.1593	0.8889	0.7778	0.1380
55	O	0.0009	-0.0005	4.7665	0.0001	-0.0001	0.2082
56	O	-1.9365	3.3499	4.7689	-0.0002	0.3332	0.2083
57	O	-3.8694	6.7030	4.7666	0.0000	0.6668	0.2082
58	O	3.8685	-0.0005	4.7665	0.3332	-0.0001	0.2082
59	O	1.9347	3.3019	4.8036	0.3309	0.3285	0.2098
60	O	-0.0423	6.7266	4.8037	0.3309	0.6691	0.2098
61	O	7.7388	0.0022	4.7689	0.6668	0.0002	0.2083
62	O	5.8059	3.3499	4.7689	0.6668	0.3332	0.2083
63	O	3.9117	6.7266	4.8037	0.6715	0.6691	0.2098
64	O	-0.0147	2.2288	6.3157	0.1096	0.2217	0.2758
65	O	-1.9464	5.5748	6.3157	0.1096	0.5545	0.2758
66	O	-3.8694	8.9360	6.3030	0.1111	0.8889	0.2753
67	O	3.8841	2.2288	6.3157	0.4455	0.2217	0.2758
68	O	1.9347	5.5850	6.3765	0.4444	0.5556	0.2785
69	O	0.0029	8.9516	6.3157	0.4455	0.8904	0.2758
70	O	7.7388	2.2340	6.3074	0.7778	0.2222	0.2755
71	O	5.8158	5.5748	6.3157	0.7783	0.5545	0.2758
72	O	3.8665	8.9516	6.3157	0.7783	0.8904	0.2758
73	O	1.9347	1.1130	7.8864	0.2220	0.1107	0.3444
74	O	-0.0179	4.4575	7.8988	0.2202	0.4434	0.3449
75	O	-1.9381	7.8210	7.8863	0.2220	0.7780	0.3444
76	O	5.8068	1.1187	7.9044	0.5559	0.1113	0.3452
77	O	3.8874	4.4575	7.8988	0.5566	0.4434	0.3449
78	O	1.9347	7.8401	7.8988	0.5566	0.7799	0.3449
79	O	9.6709	1.1187	7.9044	0.8887	0.1113	0.3452
80	O	7.7388	4.4650	7.9043	0.8887	0.4441	0.3452
81	O	5.8075	7.8210	7.8863	0.8893	0.7780	0.3444

/db/jmorales/CoNi-alloy/Profiles/Sm(surf)$Ce_{26}O_{53}$/surface

a =				11.6082000732			
b =				11.608200073110298			
c =				22.898399353			
alpha =				90.0			
beta =				90.0			
gamma =				120.0			
	Atom	X	Y	Z	X	Y	Z
1	Sm	1.9652	3.1749	7.1862	0.3272	0.3158	0.3138
2	Ce	1.9347	1.1170	0.7898	0.2222	0.1111	0.0345
3	Ce	0.0000	4.4680	0.7898	0.2222	0.4444	0.0345
4	Ce	-1.9347	7.8190	0.7898	0.2222	0.7778	0.0345
5	Ce	5.8041	1.1170	0.7898	0.5556	0.1111	0.0345
6	Ce	3.8694	4.4680	0.7898	0.5556	0.4444	0.0345
7	Ce	1.9347	7.8190	0.7898	0.5556	0.7778	0.0345
8	Ce	9.6735	1.1170	0.7898	0.8889	0.1111	0.0345
9	Ce	7.7388	4.4680	0.7898	0.8889	0.4444	0.0345
10	Ce	5.8041	7.8190	0.7898	0.8889	0.7778	0.0345
11	Ce	0.0008	2.2379	3.9821	0.1114	0.2226	0.1739
12	Ce	-1.9376	5.5848	3.9626	0.1109	0.5555	0.1730
13	Ce	-3.8716	8.9355	3.9470	0.1109	0.8888	0.1724
14	Ce	3.8663	2.2334	3.9692	0.4441	0.2222	0.1733
15	Ce	1.9460	5.5788	3.8575	0.4451	0.5549	0.1685
16	Ce	-0.0026	8.9380	3.9593	0.4443	0.8891	0.1729
17	Ce	7.7340	2.2358	3.9403	0.7775	0.2224	0.1721
18	Ce	5.7990	5.5865	3.9787	0.7774	0.5557	0.1738
19	Ce	3.8652	8.9303	3.9869	0.7771	0.8883	0.1741
20	Ce	0.0057	0.0057	7.1140	0.0008	0.0006	0.3107
21	Ce	9.6517	3.3428	7.0885	0.9977	0.3325	0.3096
22	Ce	-3.8724	6.7090	7.1062	0.0001	0.6674	0.3103
23	Ce	3.8509	0.0016	7.0958	0.3318	0.0002	0.3099
24	Ce	-0.1297	6.7803	7.1917	0.3261	0.6745	0.3141
25	Ce	7.7247	0.0207	7.0946	0.6665	0.0021	0.3098
26	Ce	5.7850	3.3481	7.1015	0.6649	0.3330	0.3101
27	Ce	3.9976	6.7471	7.2851	0.6800	0.6712	0.3181
28	O	0.0000	0.0000	0.0000	0.0000	0.0000	0.0000
29	O	-1.9347	3.3510	0.0000	-0.0000	0.3333	0.0000
30	O	-3.8694	6.7020	0.0000	-0.0000	0.6667	0.0000
31	O	3.8694	0.0000	0.0000	0.3333	0.0000	0.0000
32	O	1.9347	3.3510	0.0000	0.3333	0.3333	0.0000
33	O	0.0000	6.7020	0.0000	0.3333	0.6667	0.0000
34	O	7.7388	0.0000	0.0000	0.6667	0.0000	0.0000
35	O	5.8041	3.3510	0.0000	0.6667	0.3333	0.0000
36	O	3.8694	6.7020	0.0000	0.6667	0.6667	0.0000
37	O	-0.0000	2.2340	1.5798	0.1111	0.2222	0.0690
38	O	-1.9347	5.5850	1.5798	0.1111	0.5556	0.0690
39	O	-3.8694	8.9360	1.5798	0.1111	0.8889	0.0690
40	O	3.8694	2.2340	1.5798	0.4444	0.2222	0.0690

41	O	1.9347	5.5850	1.5798	0.4444	0.5556	0.0690
42	O	-0.0000	8.9360	1.5798	0.4444	0.8889	0.0690
43	O	7.7388	2.2340	1.5798	0.7778	0.2222	0.0690
44	O	5.8041	5.5850	1.5798	0.7778	0.5556	0.0690
45	O	3.8694	8.9360	1.5798	0.7778	0.8889	0.0690
46	O	1.9347	1.1170	3.1593	0.2222	0.1111	0.1380
47	O	0.0000	4.4680	3.1593	0.2222	0.4444	0.1380
48	O	-1.9347	7.8190	3.1593	0.2222	0.7778	0.1380
49	O	5.8041	1.1170	3.1593	0.5556	0.1111	0.1380
50	O	3.8694	4.4680	3.1593	0.5556	0.4444	0.1380
51	O	1.9347	7.8190	3.1593	0.5556	0.7778	0.1380
52	O	9.6735	1.1170	3.1593	0.8889	0.1111	0.1380
53	O	7.7388	4.4680	3.1593	0.8889	0.4444	0.1380
54	O	5.8041	7.8190	3.1593	0.8889	0.7778	0.1380
55	O	-5.8025	10.0520	4.7414	0.0001	0.9999	0.2071
56	O	-1.9319	3.3672	4.7571	0.0010	0.3349	0.2077
57	O	-3.8636	6.7037	4.7252	0.0006	0.6668	0.2064
58	O	-1.9399	10.0417	4.7132	0.3323	0.9989	0.2058
59	O	1.9378	3.4913	4.8604	0.3406	0.3473	0.2123
60	O	0.1314	6.6300	4.8738	0.3411	0.6595	0.2128
61	O	1.9220	10.0403	4.7584	0.6649	0.9987	0.2078
62	O	5.7906	3.3581	4.7510	0.6659	0.3340	0.2075
63	O	3.7438	6.6286	4.8526	0.6522	0.6594	0.2119
64	O	-0.0501	2.1880	6.3097	0.1045	0.2176	0.2756
65	O	-1.9853	5.6117	6.2962	0.1081	0.5582	0.2750
66	O	-3.8574	8.9306	6.2615	0.1119	0.8883	0.2734
67	O	3.9005	2.1421	6.2702	0.4426	0.2131	0.2738
68	O	-0.0644	8.9718	6.2835	0.4407	0.8924	0.2744
69	O	7.7401	2.2331	6.2675	0.7778	0.2221	0.2737
70	O	5.8975	5.5871	6.2944	0.7859	0.5558	0.2749
71	O	3.8874	9.0142	6.3175	0.7832	0.8967	0.2759
72	O	1.9317	1.0160	7.9468	0.2169	0.1011	0.3470
73	O	0.1593	4.6448	7.7106	0.2447	0.4620	0.3367
74	O	-1.9760	7.8375	7.9605	0.2196	0.7796	0.3476
75	O	5.8380	1.1126	7.9065	0.5583	0.1107	0.3453
76	O	3.8825	4.4052	7.8114	0.5536	0.4382	0.3411
77	O	1.8168	7.7212	7.7801	0.5405	0.7681	0.3398
78	O	9.6189	1.1624	7.9327	0.8864	0.1156	0.3464
79	O	7.7761	4.4283	7.9136	0.8901	0.4405	0.3456
80	O	5.9342	7.8924	7.9808	0.9037	0.7851	0.3485

/db/jmorales/CoNi-alloy/Profiles/Ni-Ce_3SmO_7/CO2

a =	13.9019837372
b =	13.9019837372
c =	21.9509918687
alpha =	90.0
beta =	90.0
gamma =	90.0

	Atom	X	Y	Z	X	Y	Z
1	Ni	0.0000	0.0000	0.0000	0.0000	0.0000	0.0000
2	Ni	1.7377	1.7377	0.0000	0.1250	0.1250	0.0000
3	Ni	0.0000	3.4755	0.0000	0.0000	0.2500	0.0000
4	Ni	1.7377	5.2132	0.0000	0.1250	0.3750	0.0000
5	Ni	3.4755	0.0000	0.0000	0.2500	0.0000	0.0000
6	Ni	5.2132	1.7377	0.0000	0.3750	0.1250	0.0000
7	Ni	3.4755	3.4755	0.0000	0.2500	0.2500	0.0000
8	Ni	5.2132	5.2132	0.0000	0.3750	0.3750	0.0000
9	Ni	0.0000	6.9510	0.0000	0.0000	0.5000	0.0000
10	Ni	1.7377	8.6887	0.0000	0.1250	0.6250	0.0000
11	Ni	0.0000	10.4265	0.0000	0.0000	0.7500	0.0000
12	Ni	1.7377	12.1642	0.0000	0.1250	0.8750	0.0000
13	Ni	3.4755	6.9510	0.0000	0.2500	0.5000	0.0000
14	Ni	5.2132	8.6887	0.0000	0.3750	0.6250	0.0000
15	Ni	3.4755	10.4265	0.0000	0.2500	0.7500	0.0000
16	Ni	5.2132	12.1642	0.0000	0.3750	0.8750	0.0000
17	Ni	6.9510	0.0000	0.0000	0.5000	0.0000	0.0000
18	Ni	8.6887	1.7377	0.0000	0.6250	0.1250	0.0000
19	Ni	6.9510	3.4755	0.0000	0.5000	0.2500	0.0000
20	Ni	8.6887	5.2132	0.0000	0.6250	0.3750	0.0000
21	Ni	10.4265	0.0000	0.0000	0.7500	0.0000	0.0000
22	Ni	12.1642	1.7377	0.0000	0.8750	0.1250	0.0000
23	Ni	10.4265	3.4755	0.0000	0.7500	0.2500	0.0000
24	Ni	12.1642	5.2132	0.0000	0.8750	0.3750	0.0000
25	Ni	6.9510	6.9510	0.0000	0.5000	0.5000	0.0000
26	Ni	8.6887	8.6887	0.0000	0.6250	0.6250	0.0000
27	Ni	6.9510	10.4265	0.0000	0.5000	0.7500	0.0000
28	Ni	8.6887	12.1642	0.0000	0.6250	0.8750	0.0000
29	Ni	10.4265	6.9510	0.0000	0.7500	0.5000	0.0000
30	Ni	12.1642	8.6887	0.0000	0.8750	0.6250	0.0000
31	Ni	10.4265	10.4265	0.0000	0.7500	0.7500	0.0000
32	Ni	12.1642	12.1642	0.0000	0.8750	0.8750	0.0000
33	Ni	0.0000	1.7377	1.7377	0.0000	0.1250	0.0792
34	Ni	1.7377	0.0000	1.7377	0.1250	0.0000	0.0792
35	Ni	0.0000	5.2132	1.7377	0.0000	0.3750	0.0792
36	Ni	1.7377	3.4755	1.7377	0.1250	0.2500	0.0792
37	Ni	3.4755	1.7377	1.7377	0.2500	0.1250	0.0792
38	Ni	5.2132	0.0000	1.7377	0.3750	0.0000	0.0792
39	Ni	3.4755	5.2132	1.7377	0.2500	0.3750	0.0792
40	Ni	5.2132	3.4755	1.7377	0.3750	0.2500	0.0792

41	Ni	0.0000	8.6887	1.7377	0.0000	0.6250	0.0792
42	Ni	1.7377	6.9510	1.7377	0.1250	0.5000	0.0792
43	Ni	0.0000	12.1642	1.7377	0.0000	0.8750	0.0792
44	Ni	1.7377	10.4265	1.7377	0.1250	0.7500	0.0792
45	Ni	3.4755	8.6887	1.7377	0.2500	0.6250	0.0792
46	Ni	5.2132	6.9510	1.7377	0.3750	0.5000	0.0792
47	Ni	3.4755	12.1642	1.7377	0.2500	0.8750	0.0792
48	Ni	5.2132	10.4265	1.7377	0.3750	0.7500	0.0792
49	Ni	6.9510	1.7377	1.7377	0.5000	0.1250	0.0792
50	Ni	8.6887	0.0000	1.7377	0.6250	0.0000	0.0792
51	Ni	6.9510	5.2132	1.7377	0.5000	0.3750	0.0792
52	Ni	8.6887	3.4755	1.7377	0.6250	0.2500	0.0792
53	Ni	10.4265	1.7377	1.7377	0.7500	0.1250	0.0792
54	Ni	12.1642	0.0000	1.7377	0.8750	0.0000	0.0792
55	Ni	10.4265	5.2132	1.7377	0.7500	0.3750	0.0792
56	Ni	12.1642	3.4755	1.7377	0.8750	0.2500	0.0792
57	Ni	6.9510	8.6887	1.7377	0.5000	0.6250	0.0792
58	Ni	8.6887	6.9510	1.7377	0.6250	0.5000	0.0792
59	Ni	6.9510	12.1642	1.7377	0.5000	0.8750	0.0792
60	Ni	8.6887	10.4265	1.7377	0.6250	0.7500	0.0792
61	Ni	10.4265	8.6887	1.7377	0.7500	0.6250	0.0792
62	Ni	12.1642	6.9510	1.7377	0.8750	0.5000	0.0792
63	Ni	10.4265	12.1642	1.7377	0.7500	0.8750	0.0792
64	Ni	12.1642	10.4265	1.7377	0.8750	0.7500	0.0792
65	Ni	0.0080	0.0025	3.5059	0.0006	0.0002	0.1597
66	Ni	1.7396	1.7336	3.5075	0.1251	0.1247	0.1598
67	Ni	13.8977	3.4672	3.5033	0.9997	0.2494	0.1596
68	Ni	1.7021	5.2089	3.4697	0.1224	0.3747	0.1581
69	Ni	3.4785	13.8997	3.5113	0.2502	0.9998	0.1600
70	Ni	5.2144	1.7450	3.5301	0.3751	0.1255	0.1608
71	Ni	3.4919	3.4408	3.4989	0.2512	0.2475	0.1594
72	Ni	5.2009	5.2126	3.4857	0.3741	0.3750	0.1588
73	Ni	13.8666	6.9481	3.5297	0.9975	0.4998	0.1608
74	Ni	1.7439	8.6920	3.5160	0.1254	0.6252	0.1602
75	Ni	13.8855	10.4317	3.5195	0.9988	0.7504	0.1603
76	Ni	1.7446	12.1618	3.5073	0.1255	0.8748	0.1598
77	Ni	3.4635	6.9824	3.4856	0.2491	0.5023	0.1588
78	Ni	5.2259	8.7039	3.5077	0.3759	0.6261	0.1598
79	Ni	3.4753	10.4023	3.5308	0.2500	0.7483	0.1609
80	Ni	5.2103	12.1634	3.5040	0.3748	0.8749	0.1596
81	Ni	6.9408	13.8986	3.5036	0.4993	0.9998	0.1596
82	Ni	8.6950	1.7213	3.4906	0.6255	0.1238	0.1590
83	Ni	6.9326	3.4628	3.4948	0.4987	0.2491	0.1592
84	Ni	8.6890	5.2245	3.5275	0.6250	0.3758	0.1607
85	Ni	10.4348	13.8844	3.5228	0.7506	0.9987	0.1605
86	Ni	12.1637	1.7322	3.5050	0.8750	0.1246	0.1597
87	Ni	10.4353	3.4883	3.5091	0.7506	0.2509	0.1599
88	Ni	12.1493	5.2249	3.5491	0.8739	0.3758	0.1617
89	Ni	6.9271	6.9380	3.5070	0.4983	0.4991	0.1598

90	Ni	8.6918	8.6819	3.4603	0.6252	0.6245	0.1576
91	Ni	6.9775	10.4253	3.5167	0.5019	0.7499	0.1602
92	Ni	8.6906	12.1860	3.4927	0.6251	0.8766	0.1591
93	Ni	10.4375	6.9348	3.5450	0.7508	0.4988	0.1615
94	Ni	12.1833	8.6752	3.4972	0.8764	0.6240	0.1593
95	Ni	10.4391	10.4541	3.4273	0.7509	0.7520	0.1561
96	Ni	12.1720	12.1748	3.5049	0.8756	0.8758	0.1597
97	Ni	0.0205	1.7271	5.2274	0.0015	0.1242	0.2381
98	Ni	1.7432	13.8908	5.2267	0.1254	0.9992	0.2381
99	Ni	0.0125	5.1911	5.2243	0.0009	0.3734	0.2380
100	Ni	1.7429	3.4763	5.2194	0.1254	0.2501	0.2378
101	Ni	3.4609	1.7257	5.2354	0.2490	0.1241	0.2385
102	Ni	5.2081	-0.0028	5.2300	0.3746	-0.0002	0.2383
103	Ni	3.4282	5.1903	5.0788	0.2466	0.3733	0.2314
104	Ni	5.1838	3.4291	5.3699	0.3729	0.2467	0.2446
105	Ni	0.0284	8.7047	5.2147	0.0020	0.6261	0.2376
106	Ni	1.7095	6.9192	5.1952	0.1230	0.4977	0.2367
107	Ni	0.0012	12.1761	5.2215	0.0001	0.8759	0.2379
108	Ni	1.7225	10.4483	5.2255	0.1239	0.7516	0.2381
109	Ni	3.4294	8.7374	5.3843	0.2467	0.6285	0.2453
110	Ni	5.1770	6.9802	5.2317	0.3724	0.5021	0.2383
111	Ni	3.4864	12.1505	5.2320	0.2508	0.8740	0.2384
112	Ni	5.2412	10.4369	5.2079	0.3770	0.7507	0.2373
113	Ni	6.9687	1.7033	5.2094	0.5013	0.1225	0.2373
114	Ni	8.6878	13.9017	5.2185	0.6249	1.0000	0.2377
115	Ni	6.9122	5.1919	5.2277	0.4972	0.3735	0.2382
116	Ni	8.6724	3.4393	5.1636	0.6238	0.2474	0.2352
117	Ni	10.4227	1.7356	5.2175	0.7497	0.1248	0.2377
118	Ni	12.1779	0.0177	5.2241	0.8760	0.0013	0.2380
119	Ni	10.4065	5.1224	5.3604	0.7486	0.3685	0.2442
120	Ni	12.1946	3.4399	5.2144	0.8772	0.2474	0.2375
121	Ni	6.9941	8.7257	5.2437	0.5031	0.6277	0.2389
122	Ni	8.6937	6.9399	5.2428	0.6254	0.4992	0.2388
123	Ni	6.9457	12.1669	5.2238	0.4996	0.8752	0.2380
124	Ni	8.7085	10.4719	5.2127	0.6264	0.7533	0.2375
125	Ni	10.4272	8.7255	5.1158	0.7501	0.6276	0.2331
126	Ni	12.1939	6.9802	5.4023	0.8771	0.5021	0.2461
127	Ni	10.4458	12.1961	5.2839	0.7514	0.8773	0.2407
128	Ni	12.1837	10.4331	5.2719	0.8764	0.7505	0.2402
129	O	6.0733	4.3823	6.8593	0.4369	0.3152	0.3125
130	O	4.1925	7.6824	6.8034	0.3016	0.5526	0.3099
131	O	5.9918	6.5720	8.7425	0.4310	0.4727	0.3983
132	O	7.9456	7.7691	6.8937	0.5715	0.5588	0.3140
133	O	10.4343	10.4544	6.2870	0.7506	0.7520	0.2864
134	O	9.9496	6.8947	8.8344	0.7157	0.4960	0.4025
135	O	7.7975	9.7477	8.9091	0.5609	0.7012	0.4059
136	Ce	6.0291	8.8248	7.9837	0.4337	0.6348	0.3637
137	Ce	4.2504	5.5562	7.6422	0.3057	0.3997	0.3481
138	Ce	7.8756	5.5647	7.8475	0.5665	0.4003	0.3575

139	Sm	9.5216	9.0914	7.9188	0.6849	0.6540	0.3608
140	C	10.7139	6.4438	7.8536	0.7707	0.4635	0.3578
141	O	11.5990	7.2270	7.3458	0.8343	0.5199	0.3346
142	O	10.4197	5.2631	7.3830	0.7495	0.3786	0.3363

/db/jmorales/CoNi-alloy/Profiles/Ni(111)/CO_2

a =			9.829999924				
b =			9.82999992366825				
c =			21.019699097				
alpha =			90.0				
beta =			90.0				
gamma =			120.0				
	Atom	X	Y	Z	X	Y	Z
1	Ni	0.0000	0.0000	0.0000	0.0000	0.0000	0.0000
2	Ni	-1.2288	2.1283	0.0000	0.0000	0.2500	0.0000
3	Ni	-2.4575	4.2565	0.0000	-0.0000	0.5000	0.0000
4	Ni	-3.6862	6.3848	0.0000	-0.0000	0.7500	0.0000
5	Ni	2.4575	0.0000	0.0000	0.2500	0.0000	0.0000
6	Ni	1.2288	2.1283	0.0000	0.2500	0.2500	0.0000
7	Ni	-0.0000	4.2565	0.0000	0.2500	0.5000	0.0000
8	Ni	-1.2288	6.3848	0.0000	0.2500	0.7500	0.0000
9	Ni	4.9150	0.0000	0.0000	0.5000	0.0000	0.0000
10	Ni	3.6862	2.1283	0.0000	0.5000	0.2500	0.0000
11	Ni	2.4575	4.2565	0.0000	0.5000	0.5000	0.0000
12	Ni	1.2288	6.3848	0.0000	0.5000	0.7500	0.0000
13	Ni	7.3725	0.0000	0.0000	0.7500	0.0000	0.0000
14	Ni	6.1438	2.1283	0.0000	0.7500	0.2500	0.0000
15	Ni	4.9150	4.2565	0.0000	0.7500	0.5000	0.0000
16	Ni	3.6862	6.3848	0.0000	0.7500	0.7500	0.0000
17	Ni	-0.0000	1.4188	2.0065	0.0833	0.1667	0.0955
18	Ni	-1.2288	3.5471	2.0065	0.0833	0.4167	0.0955
19	Ni	-2.4575	5.6754	2.0065	0.0833	0.6667	0.0955
20	Ni	-3.6863	7.8036	2.0065	0.0833	0.9167	0.0955
21	Ni	2.4575	1.4188	2.0065	0.3333	0.1667	0.0955
22	Ni	1.2287	3.5471	2.0065	0.3333	0.4167	0.0955
23	Ni	-0.0000	5.6754	2.0065	0.3333	0.6667	0.0955
24	Ni	-1.2288	7.8036	2.0065	0.3333	0.9167	0.0955
25	Ni	4.9150	1.4188	2.0065	0.5833	0.1667	0.0955
26	Ni	3.6862	3.5471	2.0065	0.5833	0.4167	0.0955
27	Ni	2.4575	5.6754	2.0065	0.5833	0.6667	0.0955
28	Ni	1.2287	7.8036	2.0065	0.5833	0.9167	0.0955
29	Ni	7.3725	1.4188	2.0065	0.8333	0.1667	0.0955
30	Ni	6.1437	3.5471	2.0065	0.8333	0.4167	0.0955
31	Ni	4.9150	5.6754	2.0065	0.8333	0.6667	0.0955
32	Ni	3.6862	7.8036	2.0065	0.8333	0.9167	0.0955
33	Ni	1.2288	0.7094	4.0131	0.1667	0.0833	0.1909
34	Ni	0.0000	2.8377	4.0131	0.1667	0.3333	0.1909
35	Ni	-1.2287	4.9659	4.0131	0.1667	0.5833	0.1909
36	Ni	-2.4575	7.0942	4.0131	0.1667	0.8333	0.1909
37	Ni	3.6863	0.7094	4.0131	0.4167	0.0833	0.1909
38	Ni	2.4575	2.8377	4.0131	0.4167	0.3333	0.1909
39	Ni	1.2288	4.9659	4.0131	0.4167	0.5833	0.1909
40	Ni	0.0000	7.0942	4.0131	0.4167	0.8333	0.1909

41	Ni	6.1438	0.7094	4.0131	0.6667	0.0833	0.1909
42	Ni	4.9150	2.8377	4.0131	0.6667	0.3333	0.1909
43	Ni	3.6863	4.9659	4.0131	0.6667	0.5833	0.1909
44	Ni	2.4575	7.0942	4.0131	0.6667	0.8333	0.1909
45	Ni	8.6013	0.7094	4.0131	0.9167	0.0833	0.1909
46	Ni	7.3725	2.8377	4.0131	0.9167	0.3333	0.1909
47	Ni	6.1438	4.9659	4.0131	0.9167	0.5833	0.1909
48	Ni	4.9150	7.0942	4.0131	0.9167	0.8333	0.1909
49	Ni	0.0000	0.0000	6.0198	0.0000	0.0000	0.2864
50	Ni	-1.2288	2.1283	6.0198	0.0000	0.2500	0.2864
51	Ni	-2.4575	4.2565	6.0198	-0.0000	0.5000	0.2864
52	Ni	-3.6862	6.3848	6.0198	-0.0000	0.7500	0.2864
53	Ni	2.4575	0.0000	6.0198	0.2500	0.0000	0.2864
54	Ni	1.2288	2.1283	6.0198	0.2500	0.2500	0.2864
55	Ni	-0.0000	4.2565	6.0198	0.2500	0.5000	0.2864
56	Ni	-1.2288	6.3848	6.0198	0.2500	0.7500	0.2864
57	Ni	4.9150	0.0000	6.0198	0.5000	0.0000	0.2864
58	Ni	3.6862	2.1283	6.0198	0.5000	0.2500	0.2864
59	Ni	2.4575	4.2565	6.0198	0.5000	0.5000	0.2864
60	Ni	1.2288	6.3848	6.0198	0.5000	0.7500	0.2864
61	Ni	7.3725	0.0000	6.0198	0.7500	0.0000	0.2864
62	Ni	6.1438	2.1283	6.0198	0.7500	0.2500	0.2864
63	Ni	4.9150	4.2565	6.0198	0.7500	0.5000	0.2864
64	Ni	3.6862	6.3848	6.0198	0.7500	0.7500	0.2864
65	C	2.0000	3.5000	7.9000	0.4090	0.4111	0.3758
66	O	2.5455	4.1120	8.8000	0.5005	0.4830	0.4187
67	O	1.2424	2.5971	7.9000	0.2789	0.3051	0.3758

/db/jmorales/CoNi-alloy/Profiles/Ni-CoNi(001)/O

a =			6.951669213				
b =			6.951669213				
c =			21.951669213				
alpha =			90.0				
beta =			90.0				
gamma =			90.0				
	Atom	X	Y	Z	X	Y	Z
1	Co	0.0000	0.0000	0.0000	0.0000	0.0000	0.0000
2	Co	1.7379	1.7379	0.0000	0.2500	0.2500	0.0000
3	Co	0.0000	3.4758	0.0000	0.0000	0.5000	0.0000
4	Co	1.7379	5.2138	0.0000	0.2500	0.7500	0.0000
5	Co	3.4758	0.0000	0.0000	0.5000	0.0000	0.0000
6	Co	5.2138	1.7379	0.0000	0.7500	0.2500	0.0000
7	Co	3.4758	3.4758	0.0000	0.5000	0.5000	0.0000
8	Co	5.2138	5.2138	0.0000	0.7500	0.7500	0.0000
9	Co	6.9452	0.0073	3.5196	0.9991	0.0010	0.1603
10	Co	1.7366	1.7499	3.4758	0.2498	0.2517	0.1583
11	Co	0.0122	3.4645	3.5330	0.0018	0.4984	0.1609
12	Co	1.7342	5.2174	3.4758	0.2495	0.7505	0.1583
13	Co	3.4856	6.9383	3.5322	0.5014	0.9981	0.1609
14	Co	5.2176	1.7335	3.4758	0.7506	0.2494	0.1583
15	Co	3.4845	3.4660	3.5066	0.5012	0.4986	0.1597
16	Co	5.2019	5.2144	3.4758	0.7483	0.7501	0.1583
17	Ni	1.7379	0.0000	1.7379	0.2500	0.0000	0.0792
18	Ni	0.0000	1.7379	1.7379	0.0000	0.2500	0.0792
19	Ni	1.7379	3.4758	1.7379	0.2500	0.5000	0.0792
20	Ni	0.0000	5.2138	1.7379	0.0000	0.7500	0.0792
21	Ni	5.2138	0.0000	1.7379	0.7500	0.0000	0.0792
22	Ni	3.4758	1.7379	1.7379	0.5000	0.2500	0.0792
23	Ni	5.2138	3.4758	1.7379	0.7500	0.5000	0.0792
24	Ni	3.4758	5.2138	1.7379	0.5000	0.7500	0.0792
25	Ni	1.7295	0.0098	5.1611	0.2488	0.0014	0.2351
26	Ni	0.0084	1.7376	5.2686	0.0012	0.2500	0.2400
27	Ni	1.7661	3.4971	5.2443	0.2541	0.5031	0.2389
28	Ni	6.9404	5.2200	5.1616	0.9984	0.7509	0.2351
29	Ni	5.2135	6.9433	5.2680	0.7500	0.9988	0.2400
30	Ni	3.4815	1.7182	5.2498	0.5008	0.2472	0.2392
31	Ni	5.2321	3.4694	5.2499	0.7526	0.4991	0.2392
32	Ni	3.4540	5.1862	5.2452	0.4969	0.7460	0.2389
33	O	5.2278	1.7219	6.1511	0.7520	0.2477	0.2802

/db/jmorales/CoNi-alloy/Profiles/CoNi(001)/O

a =		6.951669213
b =		6.951669213
c =		21.951669213
alpha =		90.0
beta =		90.0
gamma =		90.0

	Atom	X	Y	Z	X	Y	Z
1	Ni	0.0000	0.0000	0.0000	0.0000	0.0000	0.0000
2	Ni	0.0000	3.4758	0.0000	0.0000	0.5000	0.0000
3	Ni	3.4758	0.0000	0.0000	0.5000	0.0000	0.0000
4	Ni	3.4758	3.4758	0.0000	0.5000	0.5000	0.0000
5	Ni	0.0000	1.7379	1.7379	0.0000	0.2500	0.0792
6	Ni	0.0000	5.2138	1.7379	0.0000	0.7500	0.0792
7	Ni	3.4758	1.7379	1.7379	0.5000	0.2500	0.0792
8	Ni	3.4758	5.2138	1.7379	0.5000	0.7500	0.0792
9	Ni	6.9494	0.0000	3.5371	0.9997	0.0000	0.1611
10	Ni	6.9501	3.4757	3.4251	0.9998	0.5000	0.1560
11	Ni	3.4746	0.0001	3.5075	0.4998	0.0000	0.1598
12	Ni	3.4747	3.4758	3.4657	0.4998	0.5000	0.1579
13	Ni	6.9490	1.7030	5.2573	0.9996	0.2450	0.2395
14	Ni	6.9489	5.2489	5.2572	0.9996	0.7551	0.2395
15	Ni	3.4740	1.7585	5.2266	0.4997	0.2530	0.2381
16	Ni	3.4737	5.1936	5.2268	0.4997	0.7471	0.2381
17	Co	1.7379	1.7379	0.0000	0.2500	0.2500	0.0000
18	Co	1.7379	5.2138	0.0000	0.2500	0.7500	0.0000
19	Co	5.2138	1.7379	0.0000	0.7500	0.2500	0.0000
20	Co	5.2138	5.2138	0.0000	0.7500	0.7500	0.0000
21	Co	1.7379	0.0000	1.7379	0.2500	0.0000	0.0792
22	Co	1.7379	3.4758	1.7379	0.2500	0.5000	0.0792
23	Co	5.2138	0.0000	1.7379	0.7500	0.0000	0.0792
24	Co	5.2138	3.4758	1.7379	0.7500	0.5000	0.0792
25	Co	1.7346	1.7374	3.5444	0.2495	0.2499	0.1615
26	Co	1.7345	5.2144	3.5444	0.2495	0.7501	0.1615
27	Co	5.2139	1.7370	3.5448	0.7500	0.2499	0.1615
28	Co	5.2139	5.2138	3.5441	0.7500	0.7500	0.1615
29	Co	1.7631	0.0000	5.1986	0.2536	0.0000	0.2368
30	Co	1.7344	3.4758	5.3248	0.2495	0.5000	0.2426
31	Co	5.1853	6.9513	5.1974	0.7459	1.0000	0.2368
32	Co	5.2127	3.4757	5.3279	0.7498	0.5000	0.2427
33	O	6.9491	3.4758	6.1178	0.9996	0.5000	0.2787

/db/jmorales/CoNi-alloy/Profiles/CoNi-Ce_3SmO_7/TS-3

a =			13.903338426				
b =			13.903338426				
c =			21.951669213				
alpha =			90.0				
beta =			90.0				
gamma =			90.0				
	Atom	X	Y	Z	X	Y	Z
1	Ni	0.0000	0.0000	0.0000	0.0000	0.0000	0.0000
2	Ni	0.0000	3.4758	0.0000	0.0000	0.2500	0.0000
3	Ni	3.4758	0.0000	0.0000	0.2500	0.0000	0.0000
4	Ni	3.4758	3.4758	0.0000	0.2500	0.2500	0.0000
5	Ni	0.0000	6.9517	0.0000	0.0000	0.5000	0.0000
6	Ni	0.0000	10.4275	0.0000	0.0000	0.7500	0.0000
7	Ni	3.4758	6.9517	0.0000	0.2500	0.5000	0.0000
8	Ni	3.4758	10.4275	0.0000	0.2500	0.7500	0.0000
9	Ni	6.9517	0.0000	0.0000	0.5000	0.0000	0.0000
10	Ni	6.9517	3.4758	0.0000	0.5000	0.2500	0.0000
11	Ni	10.4275	0.0000	0.0000	0.7500	0.0000	0.0000
12	Ni	10.4275	3.4758	0.0000	0.7500	0.2500	0.0000
13	Ni	6.9517	6.9517	0.0000	0.5000	0.5000	0.0000
14	Ni	6.9517	10.4275	0.0000	0.5000	0.7500	0.0000
15	Ni	10.4275	6.9517	0.0000	0.7500	0.5000	0.0000
16	Ni	10.4275	10.4275	0.0000	0.7500	0.7500	0.0000
17	Ni	0.0000	1.7379	1.7379	0.0000	0.1250	0.0792
18	Ni	0.0000	5.2138	1.7379	0.0000	0.3750	0.0792
19	Ni	3.4758	1.7379	1.7379	0.2500	0.1250	0.0792
20	Ni	3.4758	5.2138	1.7379	0.2500	0.3750	0.0792
21	Ni	0.0000	8.6896	1.7379	0.0000	0.6250	0.0792
22	Ni	0.0000	12.1654	1.7379	0.0000	0.8750	0.0792
23	Ni	3.4758	8.6896	1.7379	0.2500	0.6250	0.0792
24	Ni	3.4758	12.1654	1.7379	0.2500	0.8750	0.0792
25	Ni	6.9517	1.7379	1.7379	0.5000	0.1250	0.0792
26	Ni	6.9517	5.2138	1.7379	0.5000	0.3750	0.0792
27	Ni	10.4275	1.7379	1.7379	0.7500	0.1250	0.0792
28	Ni	10.4275	5.2138	1.7379	0.7500	0.3750	0.0792
29	Ni	6.9517	8.6896	1.7379	0.5000	0.6250	0.0792
30	Ni	6.9517	12.1654	1.7379	0.5000	0.8750	0.0792
31	Ni	10.4275	8.6896	1.7379	0.7500	0.6250	0.0792
32	Ni	10.4275	12.1654	1.7379	0.7500	0.8750	0.0792
33	Ni	13.8955	0.0001	3.5112	0.9994	0.0000	0.1599
34	Ni	13.9026	3.4800	3.5123	0.9999	0.2503	0.1600
35	Ni	3.4890	13.8869	3.5636	0.2509	0.9988	0.1623
36	Ni	3.4635	3.4556	3.4851	0.2491	0.2485	0.1588
37	Ni	0.0080	6.9680	3.5299	0.0006	0.5012	0.1608
38	Ni	0.0092	10.4475	3.5129	0.0007	0.7514	0.1600
39	Ni	3.4794	6.9555	3.5072	0.2503	0.5003	0.1598
40	Ni	3.4925	10.4254	3.5655	0.2512	0.7499	0.1624

41	Ni	6.9523	0.0148	3.5046	0.5000	0.0011	0.1597
42	Ni	6.9572	3.5024	3.5364	0.5004	0.2519	0.1611
43	Ni	10.4158	0.0023	3.5196	0.7492	0.0002	0.1603
44	Ni	10.4298	3.4835	3.5144	0.7502	0.2506	0.1601
45	Ni	6.9690	6.9317	3.4802	0.5012	0.4986	0.1585
46	Ni	6.9588	10.4510	3.4767	0.5005	0.7517	0.1584
47	Ni	10.4449	6.9462	3.5000	0.7513	0.4996	0.1594
48	Ni	10.4263	10.4421	3.5119	0.7499	0.7511	0.1600
49	Ni	13.8894	1.7540	5.2218	0.9990	0.1262	0.2379
50	Ni	0.0036	5.2174	5.2406	0.0003	0.3753	0.2387
51	Ni	3.4420	1.7634	5.2108	0.2476	0.1268	0.2374
52	Ni	3.4541	5.1771	5.1233	0.2484	0.3724	0.2334
53	Ni	13.8737	8.7368	5.2077	0.9979	0.6284	0.2372
54	Ni	13.8516	12.1909	5.2240	0.9963	0.8768	0.2380
55	Ni	3.4899	8.6487	5.2670	0.2510	0.6221	0.2399
56	Ni	3.5504	12.3381	5.8206	0.2554	0.8874	0.2652
57	Ni	6.9907	1.7778	5.2112	0.5028	0.1279	0.2374
58	Ni	6.9552	5.1628	5.4013	0.5003	0.3713	0.2461
59	Ni	10.4188	1.7371	5.2216	0.7494	0.1249	0.2379
60	Ni	10.4391	5.2251	5.2336	0.7508	0.3758	0.2384
61	Ni	7.0450	8.6883	5.0983	0.5067	0.6249	0.2322
62	Ni	6.9572	12.1875	5.1489	0.5004	0.8766	0.2346
63	Ni	10.4131	8.6928	5.2197	0.7490	0.6252	0.2378
64	Ni	10.4390	12.1892	5.2782	0.7508	0.8767	0.2404
65	Co	1.7379	1.7379	0.0000	0.1250	0.1250	0.0000
66	Co	1.7379	5.2138	0.0000	0.1250	0.3750	0.0000
67	Co	5.2138	1.7379	0.0000	0.3750	0.1250	0.0000
68	Co	5.2138	5.2138	0.0000	0.3750	0.3750	0.0000
69	Co	1.7379	8.6896	0.0000	0.1250	0.6250	0.0000
70	Co	1.7379	12.1654	0.0000	0.1250	0.8750	0.0000
71	Co	5.2138	8.6896	0.0000	0.3750	0.6250	0.0000
72	Co	5.2138	12.1654	0.0000	0.3750	0.8750	0.0000
73	Co	8.6896	1.7379	0.0000	0.6250	0.1250	0.0000
74	Co	8.6896	5.2138	0.0000	0.6250	0.3750	0.0000
75	Co	12.1654	1.7379	0.0000	0.8750	0.1250	0.0000
76	Co	12.1654	5.2138	0.0000	0.8750	0.3750	0.0000
77	Co	8.6896	8.6896	0.0000	0.6250	0.6250	0.0000
78	Co	8.6896	12.1654	0.0000	0.6250	0.8750	0.0000
79	Co	12.1654	8.6896	0.0000	0.8750	0.6250	0.0000
80	Co	12.1654	12.1654	0.0000	0.8750	0.8750	0.0000
81	Co	1.7379	0.0000	1.7379	0.1250	0.0000	0.0792
82	Co	1.7379	3.4758	1.7379	0.1250	0.2500	0.0792
83	Co	5.2138	0.0000	1.7379	0.3750	0.0000	0.0792
84	Co	5.2138	3.4758	1.7379	0.3750	0.2500	0.0792
85	Co	1.7379	6.9517	1.7379	0.1250	0.5000	0.0792
86	Co	1.7379	10.4275	1.7379	0.1250	0.7500	0.0792
87	Co	5.2138	6.9517	1.7379	0.3750	0.5000	0.0792
88	Co	5.2138	10.4275	1.7379	0.3750	0.7500	0.0792
89	Co	8.6896	0.0000	1.7379	0.6250	0.0000	0.0792

90	Co	8.6896	3.4758	1.7379	0.6250	0.2500	0.0792
91	Co	12.1654	0.0000	1.7379	0.8750	0.0000	0.0792
92	Co	12.1654	3.4758	1.7379	0.8750	0.2500	0.0792
93	Co	8.6896	6.9517	1.7379	0.6250	0.5000	0.0792
94	Co	8.6896	10.4275	1.7379	0.6250	0.7500	0.0792
95	Co	12.1654	6.9517	1.7379	0.8750	0.5000	0.0792
96	Co	12.1654	10.4275	1.7379	0.8750	0.7500	0.0792
97	Co	1.7282	1.7330	3.5527	0.1243	0.1246	0.1618
98	Co	1.7074	5.2421	3.5448	0.1228	0.3770	0.1615
99	Co	5.2193	1.7378	3.5533	0.3754	0.1250	0.1619
100	Co	5.2378	5.1949	3.5475	0.3767	0.3736	0.1616
101	Co	1.7373	8.7024	3.5604	0.1250	0.6259	0.1622
102	Co	1.7756	12.1939	3.5844	0.1277	0.8770	0.1633
103	Co	5.2059	8.6863	3.5538	0.3744	0.6248	0.1619
104	Co	5.1580	12.1637	3.6374	0.3710	0.8749	0.1657
105	Co	8.6931	1.7394	3.5542	0.6253	0.1251	0.1619
106	Co	8.6728	5.2069	3.5868	0.6238	0.3745	0.1634
107	Co	12.1582	1.7467	3.5618	0.8745	0.1256	0.1623
108	Co	12.1740	5.2149	3.5759	0.8756	0.3751	0.1629
109	Co	8.7232	8.6985	3.5064	0.6274	0.6256	0.1597
110	Co	8.7118	12.1759	3.4722	0.6266	0.8758	0.1582
111	Co	12.1627	8.7007	3.5623	0.8748	0.6258	0.1623
112	Co	12.1444	12.1729	3.5832	0.8735	0.8755	0.1632
113	Co	1.6884	0.0518	5.2372	0.1214	0.0037	0.2386
114	Co	1.7303	3.4919	5.2446	0.1245	0.2512	0.2389
115	Co	5.2672	0.0777	5.2819	0.3788	0.0056	0.2406
116	Co	5.1742	3.4342	5.2147	0.3722	0.2470	0.2376
117	Co	1.6981	6.9782	5.3390	0.1221	0.5019	0.2432
118	Co	1.7390	10.5048	5.2456	0.1251	0.7556	0.2390
119	Co	5.2806	6.9230	5.1907	0.3798	0.4979	0.2365
120	Co	5.2824	10.3633	5.2461	0.3799	0.7454	0.2390
121	Co	8.6790	0.0010	5.3152	0.6242	0.0001	0.2421
122	Co	8.7304	3.4785	5.2601	0.6279	0.2502	0.2396
123	Co	12.1645	0.0289	5.2475	0.8749	0.0021	0.2390
124	Co	12.1597	3.4738	5.2556	0.8746	0.2499	0.2394
125	Co	8.7353	6.9731	5.1999	0.6283	0.5015	0.2369
126	Co	8.7149	10.4572	5.2732	0.6268	0.7521	0.2402
127	Co	12.1722	6.9639	5.2496	0.8755	0.5009	0.2391
128	Co	12.1419	10.4276	5.2404	0.8733	0.7500	0.2387
129	O	5.9757	6.1586	6.8223	0.4298	0.4430	0.3108
130	O	2.5920	7.8800	6.8354	0.1864	0.5668	0.3114
131	O	4.7611	7.9377	8.8079	0.3424	0.5709	0.4012
132	O	5.8084	9.9894	7.0472	0.4178	0.7185	0.3210
133	O	7.7720	9.9294	9.0092	0.5590	0.7142	0.4104
134	O	4.7591	11.9876	8.5891	0.3423	0.8622	0.3913
135	O	8.5500	12.1720	6.3658	0.6150	0.8755	0.2900
136	Ce	3.6711	9.9594	8.0549	0.2640	0.7163	0.3669
137	Ce	3.8406	6.2069	7.7207	0.2762	0.4464	0.3517
138	Ce	6.9620	8.1534	7.9889	0.5007	0.5864	0.3639

139	Sm	6.9963	11.6369	8.0886	0.5032	0.8370	0.3685
140	C	4.5466	13.0050	7.3359	0.3270	0.9354	0.3342
141	O	2.2183	11.2543	6.7966	0.1596	0.8095	0.3096
142	O	5.1311	0.1603	7.5824	0.3691	0.0115	0.3454

/db/jmorales/CoNi-alloy/Profiles/Ni-Ce_3SmO_7/TS-2

a =			13.901983737				
b =			13.901983737				
c =			21.950991869				
alpha =			90.0				
beta =			90.0				
gamma =			90.0				
	Atom	X	Y	Z	X	Y	Z
1	Ni	0.0000	0.0000	0.0000	0.0000	0.0000	0.0000
2	Ni	1.7378	1.7378	0.0000	0.1250	0.1250	0.0000
3	Ni	0.0000	3.4755	0.0000	0.0000	0.2500	0.0000
4	Ni	1.7378	5.2132	0.0000	0.1250	0.3750	0.0000
5	Ni	3.4755	0.0000	0.0000	0.2500	0.0000	0.0000
6	Ni	5.2132	1.7378	0.0000	0.3750	0.1250	0.0000
7	Ni	3.4755	3.4755	0.0000	0.2500	0.2500	0.0000
8	Ni	5.2132	5.2132	0.0000	0.3750	0.3750	0.0000
9	Ni	0.0000	6.9510	0.0000	0.0000	0.5000	0.0000
10	Ni	1.7378	8.6887	0.0000	0.1250	0.6250	0.0000
11	Ni	0.0000	10.4265	0.0000	0.0000	0.7500	0.0000
12	Ni	1.7378	12.1642	0.0000	0.1250	0.8750	0.0000
13	Ni	3.4755	6.9510	0.0000	0.2500	0.5000	0.0000
14	Ni	5.2132	8.6887	0.0000	0.3750	0.6250	0.0000
15	Ni	3.4755	10.4265	0.0000	0.2500	0.7500	0.0000
16	Ni	5.2132	12.1642	0.0000	0.3750	0.8750	0.0000
17	Ni	6.9510	0.0000	0.0000	0.5000	0.0000	0.0000
18	Ni	8.6887	1.7378	0.0000	0.6250	0.1250	0.0000
19	Ni	6.9510	3.4755	0.0000	0.5000	0.2500	0.0000
20	Ni	8.6887	5.2132	0.0000	0.6250	0.3750	0.0000
21	Ni	10.4265	0.0000	0.0000	0.7500	0.0000	0.0000
22	Ni	12.1642	1.7378	0.0000	0.8750	0.1250	0.0000
23	Ni	10.4265	3.4755	0.0000	0.7500	0.2500	0.0000
24	Ni	12.1642	5.2132	0.0000	0.8750	0.3750	0.0000
25	Ni	6.9510	6.9510	0.0000	0.5000	0.5000	0.0000
26	Ni	8.6887	8.6887	0.0000	0.6250	0.6250	0.0000
27	Ni	6.9510	10.4265	0.0000	0.5000	0.7500	0.0000
28	Ni	8.6887	12.1642	0.0000	0.6250	0.8750	0.0000
29	Ni	10.4265	6.9510	0.0000	0.7500	0.5000	0.0000
30	Ni	12.1642	8.6887	0.0000	0.8750	0.6250	0.0000
31	Ni	10.4265	10.4265	0.0000	0.7500	0.7500	0.0000
32	Ni	12.1642	12.1642	0.0000	0.8750	0.8750	0.0000
33	Ni	0.0000	1.7378	1.7378	0.0000	0.1250	0.0792
34	Ni	1.7378	0.0000	1.7378	0.1250	0.0000	0.0792
35	Ni	0.0000	5.2132	1.7378	0.0000	0.3750	0.0792
36	Ni	1.7378	3.4755	1.7378	0.1250	0.2500	0.0792
37	Ni	3.4755	1.7378	1.7378	0.2500	0.1250	0.0792
38	Ni	5.2132	0.0000	1.7378	0.3750	0.0000	0.0792
39	Ni	3.4755	5.2132	1.7378	0.2500	0.3750	0.0792
40	Ni	5.2132	3.4755	1.7378	0.3750	0.2500	0.0792

41	Ni	0.0000	8.6887	1.7378	0.0000	0.6250	0.0792
42	Ni	1.7378	6.9510	1.7378	0.1250	0.5000	0.0792
43	Ni	0.0000	12.1642	1.7378	0.0000	0.8750	0.0792
44	Ni	1.7378	10.4265	1.7378	0.1250	0.7500	0.0792
45	Ni	3.4755	8.6887	1.7378	0.2500	0.6250	0.0792
46	Ni	5.2132	6.9510	1.7378	0.3750	0.5000	0.0792
47	Ni	3.4755	12.1642	1.7378	0.2500	0.8750	0.0792
48	Ni	5.2132	10.4265	1.7378	0.3750	0.7500	0.0792
49	Ni	6.9510	1.7378	1.7378	0.5000	0.1250	0.0792
50	Ni	8.6887	0.0000	1.7378	0.6250	0.0000	0.0792
51	Ni	6.9510	5.2132	1.7378	0.5000	0.3750	0.0792
52	Ni	8.6887	3.4755	1.7378	0.6250	0.2500	0.0792
53	Ni	10.4265	1.7378	1.7378	0.7500	0.1250	0.0792
54	Ni	12.1642	0.0000	1.7378	0.8750	0.0000	0.0792
55	Ni	10.4265	5.2132	1.7378	0.7500	0.3750	0.0792
56	Ni	12.1642	3.4755	1.7378	0.8750	0.2500	0.0792
57	Ni	6.9510	8.6887	1.7378	0.5000	0.6250	0.0792
58	Ni	8.6887	6.9510	1.7378	0.6250	0.5000	0.0792
59	Ni	6.9510	12.1642	1.7378	0.5000	0.8750	0.0792
60	Ni	8.6887	10.4265	1.7378	0.6250	0.7500	0.0792
61	Ni	10.4265	8.6887	1.7378	0.7500	0.6250	0.0792
62	Ni	12.1642	6.9510	1.7378	0.8750	0.5000	0.0792
63	Ni	10.4265	12.1642	1.7378	0.7500	0.8750	0.0792
64	Ni	12.1642	10.4265	1.7378	0.8750	0.7500	0.0792
65	Ni	0.0060	0.0066	3.5149	0.0004	0.0005	0.1601
66	Ni	1.7347	1.7339	3.5146	0.1248	0.1247	0.1601
67	Ni	13.8830	3.4695	3.5159	0.9986	0.2496	0.1602
68	Ni	1.7036	5.2060	3.4739	0.1225	0.3745	0.1583
69	Ni	3.4844	0.0023	3.5265	0.2506	0.0002	0.1607
70	Ni	5.2278	1.7546	3.5433	0.3760	0.1262	0.1614
71	Ni	3.4902	3.4333	3.5018	0.2511	0.2470	0.1595
72	Ni	5.1958	5.2071	3.4795	0.3737	0.3746	0.1585
73	Ni	13.8904	6.9506	3.5148	0.9992	0.5000	0.1601
74	Ni	1.7520	8.6996	3.5199	0.1260	0.6258	0.1604
75	Ni	13.8891	10.4386	3.5292	0.9991	0.7509	0.1608
76	Ni	1.7507	12.1720	3.5170	0.1259	0.8756	0.1602
77	Ni	3.4617	6.9800	3.4872	0.2490	0.5021	0.1589
78	Ni	5.2190	8.7132	3.5114	0.3754	0.6268	0.1600
79	Ni	3.4767	10.4134	3.5405	0.2501	0.7491	0.1613
80	Ni	5.2146	12.1766	3.5129	0.3751	0.8759	0.1600
81	Ni	6.9524	0.0092	3.5150	0.5001	0.0007	0.1601
82	Ni	8.7112	1.7565	3.5239	0.6266	0.1264	0.1605
83	Ni	6.9724	3.4698	3.5196	0.5015	0.2496	0.1603
84	Ni	8.6840	5.2369	3.5403	0.6247	0.3767	0.1613
85	Ni	10.4349	13.8813	3.5313	0.7506	0.9985	0.1609
86	Ni	12.1564	1.7422	3.5155	0.8744	0.1253	0.1602
87	Ni	10.4332	3.5141	3.5316	0.7505	0.2528	0.1609
88	Ni	12.1400	5.2293	3.5596	0.8733	0.3762	0.1622
89	Ni	6.9125	6.9486	3.5033	0.4972	0.4998	0.1596

90	Ni	8.6788	8.6888	3.4590	0.6243	0.6250	0.1576
91	Ni	6.9671	10.4306	3.5238	0.5012	0.7503	0.1605
92	Ni	8.6883	12.1893	3.4970	0.6250	0.8768	0.1593
93	Ni	10.4238	6.9386	3.5495	0.7498	0.4991	0.1617
94	Ni	12.1960	8.6681	3.5052	0.8773	0.6235	0.1597
95	Ni	10.4431	10.4501	3.4305	0.7512	0.7517	0.1563
96	Ni	12.1763	12.1726	3.5080	0.8759	0.8756	0.1598
97	Ni	0.0025	1.7472	5.2406	0.0002	0.1257	0.2387
98	Ni	1.7316	13.9019	5.2396	0.1246	1.0000	0.2387
99	Ni	0.0300	5.1743	5.2221	0.0022	0.3722	0.2379
100	Ni	1.7517	3.4687	5.2313	0.1260	0.2495	0.2383
101	Ni	3.4579	1.7245	5.2422	0.2487	0.1240	0.2388
102	Ni	5.2320	0.0196	5.2477	0.3764	0.0014	0.2391
103	Ni	3.4243	5.1871	5.0807	0.2463	0.3731	0.2315
104	Ni	5.1768	3.4184	5.3694	0.3724	0.2459	0.2446
105	Ni	0.0586	8.7263	5.2249	0.0042	0.6277	0.2380
106	Ni	1.7074	6.9240	5.2039	0.1228	0.4981	0.2371
107	Ni	0.0019	12.1779	5.2319	0.0001	0.8760	0.2383
108	Ni	1.7349	10.4662	5.2403	0.1248	0.7529	0.2387
109	Ni	3.4293	8.7497	5.3936	0.2467	0.6294	0.2457
110	Ni	5.1586	6.9803	5.2299	0.3711	0.5021	0.2383
111	Ni	3.4903	12.1735	5.2507	0.2511	0.8757	0.2392
112	Ni	5.2252	10.4561	5.2231	0.3759	0.7521	0.2379
113	Ni	7.0047	1.7332	5.2328	0.5039	0.1247	0.2384
114	Ni	8.6852	0.0010	5.2309	0.6247	0.0001	0.2383
115	Ni	6.8859	5.1932	5.2238	0.4953	0.3736	0.2380
116	Ni	8.7711	3.5010	5.2740	0.6309	0.2518	0.2403
117	Ni	10.4337	1.7080	5.2332	0.7505	0.1229	0.2384
118	Ni	12.1846	0.0155	5.2322	0.8765	0.0011	0.2384
119	Ni	10.3729	5.4070	5.4784	0.7461	0.3889	0.2496
120	Ni	12.1528	3.4672	5.2348	0.8742	0.2494	0.2385
121	Ni	6.9581	8.7644	5.2685	0.5005	0.6304	0.2400
122	Ni	8.6259	7.0033	5.2407	0.6205	0.5038	0.2387
123	Ni	6.9423	12.1681	5.2411	0.4994	0.8753	0.2388
124	Ni	8.7144	10.4717	5.2045	0.6268	0.7533	0.2371
125	Ni	10.4404	8.7086	5.1261	0.7510	0.6264	0.2335
126	Ni	12.2678	6.9970	5.3779	0.8824	0.5033	0.2450
127	Ni	10.4514	12.2009	5.2869	0.7518	0.8776	0.2408
128	Ni	12.1848	10.4255	5.2793	0.8765	0.7499	0.2405
129	O	6.0417	4.3827	6.8406	0.4346	0.3153	0.3116
130	O	4.1651	7.6593	6.7958	0.2996	0.5509	0.3096
131	O	5.9196	6.5545	8.7747	0.4258	0.4715	0.3997
132	O	7.7698	7.7921	6.8524	0.5589	0.5605	0.3122
133	O	10.4146	10.4528	6.2680	0.7491	0.7519	0.2855
134	O	9.9818	6.8187	8.2487	0.7180	0.4905	0.3758
135	O	7.7458	9.6799	8.9417	0.5572	0.6963	0.4074
136	Ce	5.9322	8.8056	8.0440	0.4267	0.6334	0.3665
137	Ce	4.2124	5.5369	7.6532	0.3030	0.3983	0.3486
138	Ce	7.8386	5.5435	7.8258	0.5638	0.3988	0.3565

139	Sm	9.4072	9.0730	7.8212	0.6767	0.6526	0.3563
140	C	10.7412	6.1242	7.4132	0.7726	0.4405	0.3377
141	O	11.9841	6.4631	7.2767	0.8620	0.4649	0.3315
142	O	10.1362	4.7376	7.2196	0.7291	0.3408	0.3289

/db/jmorales/CoNi-alloy/Profiles/Sm(subsurf)$Ce_{26}O_{54}$/CO2

a =			11.6082000732				
b =			11.608200073110298				
c =			22.898399353				
alpha =			90.0				
beta =			90.0				
gamma =			120.0				
	Atom	X	Y	Z	X	Y	Z
1	Sm	1.9348	5.6027	3.9953	0.4453	0.5573	0.1745
2	Ce	1.9347	1.1170	0.7898	0.2222	0.1111	0.0345
3	Ce	0.0000	4.4680	0.7898	0.2222	0.4444	0.0345
4	Ce	-1.9347	7.8190	0.7898	0.2222	0.7778	0.0345
5	Ce	5.8041	1.1170	0.7898	0.5556	0.1111	0.0345
6	Ce	3.8694	4.4680	0.7898	0.5556	0.4444	0.0345
7	Ce	1.9347	7.8190	0.7898	0.5556	0.7778	0.0345
8	Ce	9.6735	1.1170	0.7898	0.8889	0.1111	0.0345
9	Ce	7.7388	4.4680	0.7898	0.8889	0.4444	0.0345
10	Ce	5.8041	7.8190	0.7898	0.8889	0.7778	0.0345
11	Ce	0.0021	2.2522	3.9541	0.1122	0.2240	0.1727
12	Ce	-1.9162	5.5837	3.9433	0.1126	0.5554	0.1722
13	Ce	-3.8695	8.9286	3.9542	0.1107	0.8882	0.1727
14	Ce	3.8672	2.2521	3.9541	0.4452	0.2240	0.1727
15	Ce	0.0130	8.9080	3.9688	0.4442	0.8861	0.1733
16	Ce	7.7388	2.2415	3.9252	0.7782	0.2230	0.1714
17	Ce	5.7852	5.5841	3.9434	0.7761	0.5555	0.1722
18	Ce	3.8563	8.9080	3.9688	0.7753	0.8861	0.1733
19	Ce	-0.0002	0.0013	7.0933	0.0000	0.0001	0.3098
20	Ce	-1.9410	3.3447	7.1023	-0.0009	0.3327	0.3102
21	Ce	-3.8701	6.6951	7.0978	-0.0004	0.6660	0.3100
22	Ce	3.8691	0.0011	7.0930	0.3334	0.0001	0.3098
23	Ce	1.9345	3.3466	7.1043	0.3331	0.3329	0.3103
24	Ce	-0.1178	6.6500	7.1414	0.3206	0.6615	0.3119
25	Ce	7.7387	0.1262	7.0768	0.6729	0.0125	0.3091
26	Ce	5.8099	3.3448	7.1023	0.6669	0.3327	0.3102
27	Ce	3.9860	6.6506	7.1419	0.6742	0.6616	0.3119
28	O	0.0000	0.0000	0.0000	0.0000	0.0000	0.0000
29	O	-1.9347	3.3510	0.0000	-0.0000	0.3333	0.0000
30	O	-3.8694	6.7020	0.0000	-0.0000	0.6667	0.0000
31	O	3.8694	0.0000	0.0000	0.3333	0.0000	0.0000
32	O	1.9347	3.3510	0.0000	0.3333	0.3333	0.0000
33	O	0.0000	6.7020	0.0000	0.3333	0.6667	0.0000
34	O	7.7388	0.0000	0.0000	0.6667	0.0000	0.0000
35	O	5.8041	3.3510	0.0000	0.6667	0.3333	0.0000
36	O	3.8694	6.7020	0.0000	0.6667	0.6667	0.0000
37	O	-0.0000	2.2340	1.5798	0.1111	0.2222	0.0690
38	O	-1.9347	5.5850	1.5798	0.1111	0.5556	0.0690
39	O	-3.8694	8.9360	1.5798	0.1111	0.8889	0.0690
40	O	3.8694	2.2340	1.5798	0.4444	0.2222	0.0690

41	O	1.9347	5.5850	1.5798	0.4444	0.5556	0.0690
42	O	-0.0000	8.9360	1.5798	0.4444	0.8889	0.0690
43	O	7.7388	2.2340	1.5798	0.7778	0.2222	0.0690
44	O	5.8041	5.5850	1.5798	0.7778	0.5556	0.0690
45	O	3.8694	8.9360	1.5798	0.7778	0.8889	0.0690
46	O	1.9347	1.1170	3.1593	0.2222	0.1111	0.1380
47	O	0.0000	4.4680	3.1593	0.2222	0.4444	0.1380
48	O	-1.9347	7.8190	3.1593	0.2222	0.7778	0.1380
49	O	5.8041	1.1170	3.1593	0.5556	0.1111	0.1380
50	O	3.8694	4.4680	3.1593	0.5556	0.4444	0.1380
51	O	1.9347	7.8190	3.1593	0.5556	0.7778	0.1380
52	O	9.6735	1.1170	3.1593	0.8889	0.1111	0.1380
53	O	7.7388	4.4680	3.1593	0.8889	0.4444	0.1380
54	O	5.8041	7.8190	3.1593	0.8889	0.7778	0.1380
55	O	-5.8290	10.0269	4.7749	-0.0034	0.9974	0.2085
56	O	-1.9296	3.3518	4.7590	0.0005	0.3334	0.2078
57	O	-3.8695	6.7070	4.7614	0.0002	0.6672	0.2079
58	O	3.8938	-0.0261	4.7746	0.3341	-0.0026	0.2085
59	O	1.9348	3.3483	4.8345	0.3332	0.3331	0.2111
60	O	-0.0625	6.7115	4.8017	0.3284	0.6676	0.2097
61	O	7.7389	0.0186	4.7651	0.6676	0.0018	0.2081
62	O	5.7988	3.3520	4.7592	0.6663	0.3334	0.2078
63	O	3.9316	6.7124	4.8016	0.6725	0.6677	0.2097
64	O	-0.0079	2.1900	6.2939	0.1082	0.2179	0.2749
65	O	-1.9653	5.5732	6.3179	0.1079	0.5544	0.2759
66	O	-3.8694	8.9527	6.2918	0.1119	0.8905	0.2748
67	O	3.8770	2.1889	6.2940	0.4429	0.2177	0.2749
68	O	1.9346	5.7371	6.5021	0.4520	0.5707	0.2840
69	O	0.0603	8.9054	6.3699	0.4481	0.8858	0.2782
70	O	7.7390	2.2588	6.2762	0.7790	0.2247	0.2741
71	O	5.8334	5.5746	6.3180	0.7798	0.5545	0.2759
72	O	3.8081	8.9051	6.3700	0.7710	0.8858	0.2782
73	O	1.9345	1.1155	7.8820	0.2221	0.1110	0.3442
74	O	-0.0494	4.3864	7.8199	0.2139	0.4363	0.3415
75	O	-1.9770	7.8172	7.8692	0.2185	0.7776	0.3437
76	O	5.8279	1.1373	7.8832	0.5586	0.1131	0.3443
77	O	3.9176	4.3859	7.8198	0.5556	0.4363	0.3415
78	O	1.9353	8.0648	8.2798	0.5678	0.8022	0.3616
79	O	9.6503	1.1371	7.8832	0.8879	0.1131	0.3443
80	O	7.7380	4.4562	7.8984	0.8882	0.4433	0.3449
81	O	5.8451	7.8189	7.8694	0.8924	0.7778	0.3437
82	C	1.9336	7.1131	9.2255	0.5203	0.7076	0.4029
83	O	0.7951	6.6609	9.5686	0.3998	0.6626	0.4179
84	O	3.0701	6.6589	9.5712	0.5957	0.6624	0.4180

/db/jmorales/CoNi-alloy/Profiles/Co-CoNi(001)/O

a =	6.951669213
b =	6.951669213
c =	21.951669213
alpha =	90.0
beta =	90.0
gamma =	90.0

	Atom	X	Y	Z	X	Y	Z
1	Ni	0.0000	0.0000	0.0000	0.0000	0.0000	0.0000
2	Ni	1.7379	1.7379	0.0000	0.2500	0.2500	0.0000
3	Ni	0.0000	3.4758	0.0000	0.0000	0.5000	0.0000
4	Ni	1.7379	5.2138	0.0000	0.2500	0.7500	0.0000
5	Ni	3.4758	0.0000	0.0000	0.5000	0.0000	0.0000
6	Ni	5.2138	1.7379	0.0000	0.7500	0.2500	0.0000
7	Ni	3.4758	3.4758	0.0000	0.5000	0.5000	0.0000
8	Ni	5.2138	5.2138	0.0000	0.7500	0.7500	0.0000
9	Ni	0.0002	6.9515	3.5074	0.0000	1.0000	0.1598
10	Ni	1.7376	1.7401	3.4758	0.2500	0.2503	0.1583
11	Ni	6.9456	3.4786	3.5183	0.9991	0.5004	0.1603
12	Ni	1.7380	5.2140	3.4758	0.2500	0.7500	0.1583
13	Ni	3.4739	0.0063	3.5183	0.4997	0.0009	0.1603
14	Ni	5.2173	1.7345	3.4758	0.7505	0.2495	0.1583
15	Ni	3.4820	3.4700	3.5104	0.5009	0.4992	0.1599
16	Ni	5.2118	5.2145	3.4758	0.7497	0.7501	0.1583
17	Co	1.7379	0.0000	1.7379	0.2500	0.0000	0.0792
18	Co	0.0000	1.7379	1.7379	0.0000	0.2500	0.0792
19	Co	1.7379	3.4758	1.7379	0.2500	0.5000	0.0792
20	Co	0.0000	5.2138	1.7379	0.0000	0.7500	0.0792
21	Co	5.2138	0.0000	1.7379	0.7500	0.0000	0.0792
22	Co	3.4758	1.7379	1.7379	0.5000	0.2500	0.0792
23	Co	5.2138	3.4758	1.7379	0.7500	0.5000	0.0792
24	Co	3.4758	5.2138	1.7379	0.5000	0.7500	0.0792
25	Co	1.7247	0.0193	5.2297	0.2481	0.0028	0.2382
26	Co	0.0467	1.7291	5.3015	0.0067	0.2487	0.2415
27	Co	1.7561	3.4975	5.2680	0.2526	0.5031	0.2400
28	Co	6.9334	5.2280	5.2298	0.9974	0.7521	0.2382
29	Co	5.2234	6.9047	5.3015	0.7514	0.9932	0.2415
30	Co	3.4506	1.7066	5.2970	0.4964	0.2455	0.2413
31	Co	5.2457	3.5014	5.2971	0.7546	0.5037	0.2413
32	Co	3.4540	5.1958	5.2682	0.4969	0.7474	0.2400
33	O	5.2249	1.7284	6.1673	0.7516	0.2486	0.2809

/db/jmorales/CoNi-alloy/Profiles/Ni(111)/CO-O

a =	9.829999924
b =	9.829999923668225
c =	21.019699097
alpha =	90.0
beta =	90.0
gamma =	120.0

	Atom	X	Y	Z	X	Y	Z
1	Ni	0.0000	0.0000	0.0000	0.0000	0.0000	0.0000
2	Ni	-1.2288	2.1283	0.0000	0.0000	0.2500	0.0000
3	Ni	-2.4575	4.2565	0.0000	-0.0000	0.5000	0.0000
4	Ni	-3.6862	6.3848	0.0000	-0.0000	0.7500	0.0000
5	Ni	2.4575	0.0000	0.0000	0.2500	0.0000	0.0000
6	Ni	1.2288	2.1283	0.0000	0.2500	0.2500	0.0000
7	Ni	-0.0000	4.2565	0.0000	0.2500	0.5000	0.0000
8	Ni	-1.2288	6.3848	0.0000	0.2500	0.7500	0.0000
9	Ni	4.9150	0.0000	0.0000	0.5000	0.0000	0.0000
10	Ni	3.6862	2.1283	0.0000	0.5000	0.2500	0.0000
11	Ni	2.4575	4.2565	0.0000	0.5000	0.5000	0.0000
12	Ni	1.2288	6.3848	0.0000	0.5000	0.7500	0.0000
13	Ni	7.3725	0.0000	0.0000	0.7500	0.0000	0.0000
14	Ni	6.1438	2.1283	0.0000	0.7500	0.2500	0.0000
15	Ni	4.9150	4.2565	0.0000	0.7500	0.5000	0.0000
16	Ni	3.6862	6.3848	0.0000	0.7500	0.7500	0.0000
17	Ni	-0.0000	1.4188	2.0065	0.0833	0.1667	0.0955
18	Ni	-1.2288	3.5471	2.0065	0.0833	0.4167	0.0955
19	Ni	-2.4575	5.6754	2.0065	0.0833	0.6667	0.0955
20	Ni	-3.6863	7.8036	2.0065	0.0833	0.9167	0.0955
21	Ni	2.4575	1.4188	2.0065	0.3333	0.1667	0.0955
22	Ni	1.2287	3.5471	2.0065	0.3333	0.4167	0.0955
23	Ni	-0.0000	5.6754	2.0065	0.3333	0.6667	0.0955
24	Ni	-1.2288	7.8036	2.0065	0.3333	0.9167	0.0955
25	Ni	4.9150	1.4188	2.0065	0.5833	0.1667	0.0955
26	Ni	3.6862	3.5471	2.0065	0.5833	0.4167	0.0955
27	Ni	2.4575	5.6754	2.0065	0.5833	0.6667	0.0955
28	Ni	1.2287	7.8036	2.0065	0.5833	0.9167	0.0955
29	Ni	7.3725	1.4188	2.0065	0.8333	0.1667	0.0955
30	Ni	6.1437	3.5471	2.0065	0.8333	0.4167	0.0955
31	Ni	4.9150	5.6754	2.0065	0.8333	0.6667	0.0955
32	Ni	3.6862	7.8036	2.0065	0.8333	0.9167	0.0955
33	Ni	1.2476	0.6861	4.0122	0.1672	0.0806	0.1909
34	Ni	0.0221	2.8226	4.0403	0.1680	0.3316	0.1922
35	Ni	-1.2050	4.9424	4.0188	0.1677	0.5806	0.1912
36	Ni	-2.4380	7.0771	4.0388	0.1677	0.8313	0.1921
37	Ni	3.6976	0.6955	4.0245	0.4170	0.0817	0.1915
38	Ni	2.4707	2.8350	4.0406	0.4178	0.3330	0.1922
39	Ni	1.2396	4.9397	4.0207	0.4162	0.5803	0.1913
40	Ni	0.0133	7.0774	4.0212	0.4170	0.8314	0.1913
41	Ni	6.1560	0.6886	4.0129	0.6667	0.0809	0.1909

42	Ni	4.9154	2.8136	4.0304	0.6653	0.3305	0.1917
43	Ni	3.6835	4.9283	4.0262	0.6642	0.5789	0.1915
44	Ni	2.4583	7.0532	4.0292	0.6643	0.8285	0.1917
45	Ni	8.6138	0.7017	4.0038	0.9175	0.0824	0.1905
46	Ni	7.3930	2.8254	4.0064	0.9180	0.3319	0.1906
47	Ni	6.1477	4.9457	4.0064	0.9159	0.5810	0.1906
48	Ni	4.9261	7.0719	4.0024	0.9165	0.8307	0.1904
49	Ni	4.8842	8.4458	5.9998	0.9929	0.9921	0.2854
50	Ni	8.5930	2.0987	6.0106	0.9974	0.2465	0.2860
51	Ni	7.3794	4.2140	6.0245	0.9982	0.4950	0.2866
52	Ni	6.1519	6.3407	6.0027	0.9982	0.7448	0.2856
53	Ni	-2.4179	8.4437	6.1404	0.2500	0.9919	0.2921
54	Ni	1.1825	2.0724	6.0674	0.2420	0.2434	0.2887
55	Ni	0.0134	4.2155	6.1137	0.2489	0.4952	0.2909
56	Ni	-1.2232	6.3509	6.0204	0.2486	0.7460	0.2864
57	Ni	0.0609	8.4522	6.0043	0.5026	0.9928	0.2857
58	Ni	3.7308	2.1204	6.1385	0.5041	0.2491	0.2920
59	Ni	2.4571	4.2639	6.1517	0.5004	0.5009	0.2927
60	Ni	1.2478	6.3485	6.1259	0.4998	0.7457	0.2914
61	Ni	2.4772	8.4720	6.0262	0.7496	0.9952	0.2867
62	Ni	6.1774	2.0986	6.0170	0.7517	0.2465	0.2863
63	Ni	4.9336	4.2130	6.0188	0.7493	0.4949	0.2863
64	Ni	3.7299	6.3488	6.0221	0.7523	0.7458	0.2865
65	O	1.1179	4.8999	8.7333	0.4015	0.5756	0.4155
66	C	1.1970	4.8419	7.5508	0.4062	0.5688	0.3592
67	O	2.4658	1.3844	7.2160	0.3322	0.1626	0.3433

/db/jmorales/CoNi-alloy/Profiles/Sm(subsurf)$Ce_{26}O_{53}$/CO-O

a =				11.6082000732			
b =				11.608200073110298			
c =				22.898399353			
alpha =				90.0			
beta =				90.0			
gamma =				120.0			
	Atom	X	Y	Z	X	Y	Z
1	Sm	1.9324	5.5866	3.9706	0.4443	0.5557	0.1734
2	Ce	1.9347	1.1170	0.7898	0.2222	0.1111	0.0345
3	Ce	0.0000	4.4680	0.7898	0.2222	0.4444	0.0345
4	Ce	-1.9347	7.8190	0.7898	0.2222	0.7778	0.0345
5	Ce	5.8041	1.1170	0.7898	0.5556	0.1111	0.0345
6	Ce	3.8694	4.4680	0.7898	0.5556	0.4444	0.0345
7	Ce	1.9347	7.8190	0.7898	0.5556	0.7778	0.0345
8	Ce	9.6735	1.1170	0.7898	0.8889	0.1111	0.0345
9	Ce	7.7388	4.4680	0.7898	0.8889	0.4444	0.0345
10	Ce	5.8041	7.8190	0.7898	0.8889	0.7778	0.0345
11	Ce	0.0073	2.2498	3.9581	0.1125	0.2238	0.1729
12	Ce	-1.9144	5.5851	3.9615	0.1129	0.5556	0.1730
13	Ce	-3.8681	8.9355	3.9585	0.1112	0.8888	0.1729
14	Ce	3.8641	2.2496	3.9581	0.4448	0.2238	0.1729
15	Ce	0.0105	8.9184	3.9621	0.4445	0.8871	0.1730
16	Ce	7.7389	2.2345	3.9547	0.7778	0.2223	0.1727
17	Ce	5.7882	5.5825	3.9579	0.7763	0.5553	0.1728
18	Ce	3.8596	8.9218	3.9580	0.7762	0.8875	0.1728
19	Ce	-0.0007	0.0015	7.1013	0.0000	0.0002	0.3101
20	Ce	9.6710	3.3469	7.1155	0.9996	0.3329	0.3107
21	Ce	7.7365	6.6965	7.1027	0.9995	0.6661	0.3102
22	Ce	3.8741	0.0063	7.1023	0.3340	0.0006	0.3102
23	Ce	1.9398	3.3517	7.1038	0.3338	0.3334	0.3102
24	Ce	0.0044	6.6975	7.1296	0.3335	0.6662	0.3114
25	Ce	7.7426	0.0067	7.1148	0.6673	0.0007	0.3107
26	Ce	5.8069	3.3509	7.1185	0.6669	0.3333	0.3109
27	Ce	3.8734	6.6984	7.1038	0.6668	0.6663	0.3102
28	O	0.0000	0.0000	0.0000	0.0000	0.0000	0.0000
29	O	-1.9347	3.3510	0.0000	-0.0000	0.3333	0.0000
30	O	-3.8694	6.7020	0.0000	-0.0000	0.6667	0.0000
31	O	3.8694	0.0000	0.0000	0.3333	0.0000	0.0000
32	O	1.9347	3.3510	0.0000	0.3333	0.3333	0.0000
33	O	0.0000	6.7020	0.0000	0.3333	0.6667	0.0000
34	O	7.7388	0.0000	0.0000	0.6667	0.0000	0.0000
35	O	5.8041	3.3510	0.0000	0.6667	0.3333	0.0000
36	O	3.8694	6.7020	0.0000	0.6667	0.6667	0.0000
37	O	-0.0000	2.2340	1.5798	0.1111	0.2222	0.0690
38	O	-1.9347	5.5850	1.5798	0.1111	0.5556	0.0690
39	O	-3.8694	8.9360	1.5798	0.1111	0.8889	0.0690
40	O	3.8694	2.2340	1.5798	0.4444	0.2222	0.0690

41	O	1.9347	5.5850	1.5798	0.4444	0.5556	0.0690
42	O	-0.0000	8.9360	1.5798	0.4444	0.8889	0.0690
43	O	7.7388	2.2340	1.5798	0.7778	0.2222	0.0690
44	O	5.8041	5.5850	1.5798	0.7778	0.5556	0.0690
45	O	3.8694	8.9360	1.5798	0.7778	0.8889	0.0690
46	O	1.9347	1.1170	3.1593	0.2222	0.1111	0.1380
47	O	0.0000	4.4680	3.1593	0.2222	0.4444	0.1380
48	O	-1.9347	7.8190	3.1593	0.2222	0.7778	0.1380
49	O	5.8041	1.1170	3.1593	0.5556	0.1111	0.1380
50	O	3.8694	4.4680	3.1593	0.5556	0.4444	0.1380
51	O	1.9347	7.8190	3.1593	0.5556	0.7778	0.1380
52	O	9.6735	1.1170	3.1593	0.8889	0.1111	0.1380
53	O	7.7388	4.4680	3.1593	0.8889	0.4444	0.1380
54	O	5.8041	7.8190	3.1593	0.8889	0.7778	0.1380
55	O	-5.8011	10.0517	4.7679	0.0002	0.9999	0.2082
56	O	9.6721	3.3516	4.7725	0.9999	0.3334	0.2084
57	O	-3.8672	6.7027	4.7695	0.0002	0.6667	0.2083
58	O	-1.9331	10.0516	4.7697	0.3334	0.9999	0.2083
59	O	1.9352	3.3029	4.8054	0.3310	0.3286	0.2099
60	O	-0.0443	6.7266	4.8002	0.3307	0.6691	0.2096
61	O	7.7377	0.0022	4.7724	0.6667	0.0002	0.2084
62	O	5.8072	3.3494	4.7706	0.6669	0.3332	0.2083
63	O	3.9120	6.7259	4.8050	0.6715	0.6690	0.2098
64	O	-0.0143	2.2248	6.3169	0.1094	0.2213	0.2759
65	O	-1.9421	5.5768	6.3306	0.1101	0.5547	0.2765
66	O	-3.8684	8.9366	6.3049	0.1112	0.8889	0.2753
67	O	3.8871	2.2300	6.3184	0.4458	0.2218	0.2759
68	O	0.0055	8.9453	6.3308	0.4454	0.8898	0.2765
69	O	7.7405	2.2355	6.3111	0.7780	0.2224	0.2756
70	O	5.8183	5.5729	6.3175	0.7784	0.5543	0.2759
71	O	3.8702	8.9536	6.3158	0.7787	0.8906	0.2758
72	O	1.9382	1.1138	7.8932	0.2224	0.1108	0.3447
73	O	-0.0146	4.4355	7.9067	0.2193	0.4412	0.3453
74	O	-1.9537	7.8319	7.9002	0.2212	0.7791	0.3450
75	O	5.8095	1.1189	7.9131	0.5561	0.1113	0.3456
76	O	3.8900	4.4580	7.9059	0.5568	0.4434	0.3453
77	O	1.9600	7.8528	7.9139	0.5594	0.7811	0.3456
78	O	9.6706	1.1191	7.9124	0.8887	0.1113	0.3455
79	O	7.7392	4.4638	7.9148	0.8887	0.4440	0.3456
80	O	5.8100	7.8190	7.8920	0.8894	0.7778	0.3447
81	C	0.0561	6.8462	9.9835	0.3453	0.6810	0.4360
82	O	0.1065	6.9989	11.1145	0.3573	0.6962	0.4854
83	O	1.9333	5.5891	6.3889	0.4445	0.5560	0.2790

/db/jmorales/CoNi-alloy/Profiles/CoNi-Ce_3SmO_7/O-coverage/CO-O-O-coverage/CO-O-CoNi-Ce_3SmO_7-3O

a =				13.9033384264			
b =				13.9033384264			
c =				21.9516692133			
alpha =				90.0			
beta =				90.0			
gamma =				90.0			
	Atom	X	Y	Z	X	Y	Z
1	Ni	0.0000	0.0000	0.0000	0.0000	0.0000	0.0000
2	Ni	0.0000	3.4758	0.0000	0.0000	0.2500	0.0000
3	Ni	3.4758	0.0000	0.0000	0.2500	0.0000	0.0000
4	Ni	3.4758	3.4758	0.0000	0.2500	0.2500	0.0000
5	Ni	0.0000	6.9517	0.0000	0.0000	0.5000	0.0000
6	Ni	0.0000	10.4275	0.0000	0.0000	0.7500	0.0000
7	Ni	3.4758	6.9517	0.0000	0.2500	0.5000	0.0000
8	Ni	3.4758	10.4275	0.0000	0.2500	0.7500	0.0000
9	Ni	6.9517	0.0000	0.0000	0.5000	0.0000	0.0000
10	Ni	6.9517	3.4758	0.0000	0.5000	0.2500	0.0000
11	Ni	10.4275	0.0000	0.0000	0.7500	0.0000	0.0000
12	Ni	10.4275	3.4758	0.0000	0.7500	0.2500	0.0000
13	Ni	6.9517	6.9517	0.0000	0.5000	0.5000	0.0000
14	Ni	6.9517	10.4275	0.0000	0.5000	0.7500	0.0000
15	Ni	10.4275	6.9517	0.0000	0.7500	0.5000	0.0000
16	Ni	10.4275	10.4275	0.0000	0.7500	0.7500	0.0000
17	Ni	0.0000	1.7379	1.7379	0.0000	0.1250	0.0792
18	Ni	0.0000	5.2138	1.7379	0.0000	0.3750	0.0792
19	Ni	3.4758	1.7379	1.7379	0.2500	0.1250	0.0792
20	Ni	3.4758	5.2138	1.7379	0.2500	0.3750	0.0792
21	Ni	0.0000	8.6896	1.7379	0.0000	0.6250	0.0792
22	Ni	0.0000	12.1654	1.7379	0.0000	0.8750	0.0792
23	Ni	3.4758	8.6896	1.7379	0.2500	0.6250	0.0792
24	Ni	3.4758	12.1654	1.7379	0.2500	0.8750	0.0792
25	Ni	6.9517	1.7379	1.7379	0.5000	0.1250	0.0792
26	Ni	6.9517	5.2138	1.7379	0.5000	0.3750	0.0792
27	Ni	10.4275	1.7379	1.7379	0.7500	0.1250	0.0792
28	Ni	10.4275	5.2138	1.7379	0.7500	0.3750	0.0792
29	Ni	6.9517	8.6896	1.7379	0.5000	0.6250	0.0792
30	Ni	6.9517	12.1654	1.7379	0.5000	0.8750	0.0792
31	Ni	10.4275	8.6896	1.7379	0.7500	0.6250	0.0792
32	Ni	10.4275	12.1654	1.7379	0.7500	0.8750	0.0792
33	Ni	13.9032	13.8958	3.4524	1.0000	0.9995	0.1573
34	Ni	0.0307	3.4519	3.5314	0.0022	0.2483	0.1609
35	Ni	3.4771	13.8921	3.4453	0.2501	0.9992	0.1569
36	Ni	3.4619	3.4355	3.4360	0.2490	0.2471	0.1565
37	Ni	0.0056	6.9612	3.5090	0.0004	0.5007	0.1598
38	Ni	13.8768	10.4572	3.5106	0.9981	0.7521	0.1599
39	Ni	3.4720	6.9637	3.4853	0.2497	0.5009	0.1588

40	Ni	3.5028	10.4197	3.5397	0.2519	0.7494	0.1613
41	Ni	6.9508	0.0135	3.4689	0.4999	0.0010	0.1580
42	Ni	6.9309	3.4861	3.5359	0.4985	0.2507	0.1611
43	Ni	10.4313	0.0224	3.4499	0.7503	0.0016	0.1572
44	Ni	10.4329	3.4742	3.5113	0.7504	0.2499	0.1600
45	Ni	6.9558	6.9591	3.4953	0.5003	0.5005	0.1592
46	Ni	6.9746	10.4339	3.4891	0.5016	0.7505	0.1589
47	Ni	10.4414	6.9301	3.4865	0.7510	0.4984	0.1588
48	Ni	10.4225	10.4396	3.5006	0.7496	0.7509	0.1595
49	Ni	0.0203	1.7474	5.2656	0.0015	0.1257	0.2399
50	Ni	0.0283	5.2097	5.2106	0.0020	0.3747	0.2374
51	Ni	3.4874	1.6685	5.2428	0.2508	0.1200	0.2388
52	Ni	3.4463	5.2270	5.0513	0.2479	0.3760	0.2301
53	Ni	13.8649	8.7312	5.1893	0.9972	0.6280	0.2364
54	Ni	13.8882	12.1448	5.2607	0.9989	0.8735	0.2396
55	Ni	3.4462	8.6748	5.2376	0.2479	0.6239	0.2386
56	Ni	3.5011	12.1222	5.2654	0.2518	0.8719	0.2399
57	Ni	6.9027	1.7595	5.2564	0.4965	0.1266	0.2395
58	Ni	6.9825	5.1742	5.3470	0.5022	0.3722	0.2436
59	Ni	10.4517	1.8131	5.2568	0.7517	0.1304	0.2395
60	Ni	10.4259	5.2067	5.2096	0.7499	0.3745	0.2373
61	Ni	6.9854	8.6920	5.2253	0.5024	0.6252	0.2380
62	Ni	7.0475	12.2404	5.2477	0.5069	0.8804	0.2391
63	Ni	10.4453	8.6577	5.1819	0.7513	0.6227	0.2361
64	Ni	10.3432	12.1806	5.3561	0.7439	0.8761	0.2440
65	Co	1.7379	1.7379	0.0000	0.1250	0.1250	0.0000
66	Co	1.7379	5.2138	0.0000	0.1250	0.3750	0.0000
67	Co	5.2138	1.7379	0.0000	0.3750	0.1250	0.0000
68	Co	5.2138	5.2138	0.0000	0.3750	0.3750	0.0000
69	Co	1.7379	8.6896	0.0000	0.1250	0.6250	0.0000
70	Co	1.7379	12.1654	0.0000	0.1250	0.8750	0.0000
71	Co	5.2138	8.6896	0.0000	0.3750	0.6250	0.0000
72	Co	5.2138	12.1654	0.0000	0.3750	0.8750	0.0000
73	Co	8.6896	1.7379	0.0000	0.6250	0.1250	0.0000
74	Co	8.6896	5.2138	0.0000	0.6250	0.3750	0.0000
75	Co	12.1654	1.7379	0.0000	0.8750	0.1250	0.0000
76	Co	12.1654	5.2138	0.0000	0.8750	0.3750	0.0000
77	Co	8.6896	8.6896	0.0000	0.6250	0.6250	0.0000
78	Co	8.6896	12.1654	0.0000	0.6250	0.8750	0.0000
79	Co	12.1654	8.6896	0.0000	0.8750	0.6250	0.0000
80	Co	12.1654	12.1654	0.0000	0.8750	0.8750	0.0000
81	Co	1.7379	0.0000	1.7379	0.1250	0.0000	0.0792
82	Co	1.7379	3.4758	1.7379	0.1250	0.2500	0.0792
83	Co	5.2138	0.0000	1.7379	0.3750	0.0000	0.0792
84	Co	5.2138	3.4758	1.7379	0.3750	0.2500	0.0792
85	Co	1.7379	6.9517	1.7379	0.1250	0.5000	0.0792
86	Co	1.7379	10.4275	1.7379	0.1250	0.7500	0.0792
87	Co	5.2138	6.9517	1.7379	0.3750	0.5000	0.0792
88	Co	5.2138	10.4275	1.7379	0.3750	0.7500	0.0792

89	Co	8.6896	0.0000	1.7379	0.6250	0.0000	0.0792
90	Co	8.6896	3.4758	1.7379	0.6250	0.2500	0.0792
91	Co	12.1654	0.0000	1.7379	0.8750	0.0000	0.0792
92	Co	12.1654	3.4758	1.7379	0.8750	0.2500	0.0792
93	Co	8.6896	6.9517	1.7379	0.6250	0.5000	0.0792
94	Co	8.6896	10.4275	1.7379	0.6250	0.7500	0.0792
95	Co	12.1654	6.9517	1.7379	0.8750	0.5000	0.0792
96	Co	12.1654	10.4275	1.7379	0.8750	0.7500	0.0792
97	Co	1.7405	1.7278	3.5609	0.1252	0.1243	0.1622
98	Co	1.7036	5.2315	3.5106	0.1225	0.3763	0.1599
99	Co	5.2076	1.7448	3.5408	0.3746	0.1255	0.1613
100	Co	5.2398	5.1885	3.5059	0.3769	0.3732	0.1597
101	Co	1.7259	8.7020	3.5282	0.1241	0.6259	0.1607
102	Co	1.7439	12.1644	3.5464	0.1254	0.8749	0.1616
103	Co	5.2048	8.6865	3.5601	0.3744	0.6248	0.1622
104	Co	5.2444	12.1458	3.5678	0.3772	0.8736	0.1625
105	Co	8.6792	1.7503	3.5540	0.6243	0.1259	0.1619
106	Co	8.6672	5.2007	3.5644	0.6234	0.3741	0.1624
107	Co	12.1814	1.7447	3.5508	0.8762	0.1255	0.1618
108	Co	12.1826	5.2049	3.5523	0.8762	0.3744	0.1618
109	Co	8.6918	8.6592	3.5127	0.6252	0.6228	0.1600
110	Co	8.7137	12.1944	3.4781	0.6267	0.8771	0.1584
111	Co	12.1593	8.6894	3.5343	0.8746	0.6250	0.1610
112	Co	12.1249	12.1859	3.5875	0.8721	0.8765	0.1634
113	Co	1.7539	13.8611	5.3636	0.1261	0.9970	0.2443
114	Co	1.8104	3.4832	5.2863	0.1302	0.2505	0.2408
115	Co	5.2525	13.8698	5.3309	0.3778	0.9976	0.2428
116	Co	5.1076	3.4576	5.2548	0.3674	0.2487	0.2394
117	Co	1.6960	6.9872	5.3149	0.1220	0.5026	0.2421
118	Co	1.7218	10.4499	5.1788	0.1238	0.7516	0.2359
119	Co	5.2530	6.8925	5.1698	0.3778	0.4957	0.2355
120	Co	5.2423	10.4225	5.2491	0.3771	0.7496	0.2391
121	Co	8.6685	0.1296	5.3494	0.6235	0.0093	0.2437
122	Co	8.6850	3.4705	5.2117	0.6247	0.2496	0.2374
123	Co	12.1396	13.9029	5.3528	0.8731	1.0000	0.2438
124	Co	12.1991	3.5032	5.2113	0.8774	0.2520	0.2374
125	Co	8.7229	6.9595	5.1836	0.6274	0.5006	0.2361
126	Co	8.7510	10.3500	5.1864	0.6294	0.7444	0.2363
127	Co	12.1813	6.9332	5.2152	0.8761	0.4987	0.2376
128	Co	12.1379	10.4271	5.2079	0.8730	0.7500	0.2372
129	O	5.9146	6.0095	6.7710	0.4254	0.4322	0.3085
130	O	2.6588	7.7186	6.8224	0.1912	0.5552	0.3108
131	O	4.8425	7.6869	8.7911	0.3483	0.5529	0.4005
132	O	6.0706	9.6358	6.8253	0.4366	0.6931	0.3109
133	O	7.8303	9.5469	9.0709	0.5632	0.6867	0.4132
134	O	4.9566	11.2608	8.8952	0.3565	0.8099	0.4052
135	O	8.5756	11.6731	6.4203	0.6168	0.8396	0.2925
136	Ce	3.8693	9.6781	8.0427	0.2783	0.6961	0.3664
137	Ce	3.8135	5.9482	7.7257	0.2743	0.4278	0.3519

138	Ce	7.0829	7.8081	8.0917	0.5094	0.5616	0.3686
139	Sm	7.0141	11.3427	8.2021	0.5045	0.8158	0.3736
140	C	6.8809	0.1115	6.3699	0.4949	0.0080	0.2902
141	O	6.9942	13.8893	7.6101	0.5031	0.9990	0.3467
142	O	3.5239	13.8429	6.1909	0.2535	0.9957	0.2820
143	O	10.4075	0.1281	6.1986	0.7486	0.0092	0.2824
144	O	0.0009	13.8993	6.1861	0.0001	0.9997	0.2818
145	O	3.4758	3.8003	6.3879	0.2500	0.2733	0.2910

/db/jmorales/CoNi-alloy/Profiles/Ni-Ce_3SmO_7/O-coverage/M-CO2-O-coverage/M-CO_2-Ni-Ce_3SmO_7-3O

a =			13.9019837372				
b =			13.9019837372				
c =			21.9509918687				
alpha =			90.0				
beta =			90.0				
gamma =			90.0				
	Atom	X	Y	Z	X	Y	Z
1	Ni	0.0000	0.0000	0.0000	0.0000	0.0000	0.0000
2	Ni	1.7377	1.7377	0.0000	0.1250	0.1250	0.0000
3	Ni	0.0000	3.4755	0.0000	0.0000	0.2500	0.0000
4	Ni	1.7377	5.2132	0.0000	0.1250	0.3750	0.0000
5	Ni	3.4755	0.0000	0.0000	0.2500	0.0000	0.0000
6	Ni	5.2132	1.7377	0.0000	0.3750	0.1250	0.0000
7	Ni	3.4755	3.4755	0.0000	0.2500	0.2500	0.0000
8	Ni	5.2132	5.2132	0.0000	0.3750	0.3750	0.0000
9	Ni	0.0000	6.9510	0.0000	0.0000	0.5000	0.0000
10	Ni	1.7377	8.6887	0.0000	0.1250	0.6250	0.0000
11	Ni	0.0000	10.4265	0.0000	0.0000	0.7500	0.0000
12	Ni	1.7377	12.1642	0.0000	0.1250	0.8750	0.0000
13	Ni	3.4755	6.9510	0.0000	0.2500	0.5000	0.0000
14	Ni	5.2132	8.6887	0.0000	0.3750	0.6250	0.0000
15	Ni	3.4755	10.4265	0.0000	0.2500	0.7500	0.0000
16	Ni	5.2132	12.1642	0.0000	0.3750	0.8750	0.0000
17	Ni	6.9510	0.0000	0.0000	0.5000	0.0000	0.0000
18	Ni	8.6887	1.7377	0.0000	0.6250	0.1250	0.0000
19	Ni	6.9510	3.4755	0.0000	0.5000	0.2500	0.0000
20	Ni	8.6887	5.2132	0.0000	0.6250	0.3750	0.0000
21	Ni	10.4265	0.0000	0.0000	0.7500	0.0000	0.0000
22	Ni	12.1642	1.7377	0.0000	0.8750	0.1250	0.0000
23	Ni	10.4265	3.4755	0.0000	0.7500	0.2500	0.0000
24	Ni	12.1642	5.2132	0.0000	0.8750	0.3750	0.0000
25	Ni	6.9510	6.9510	0.0000	0.5000	0.5000	0.0000
26	Ni	8.6887	8.6887	0.0000	0.6250	0.6250	0.0000
27	Ni	6.9510	10.4265	0.0000	0.5000	0.7500	0.0000
28	Ni	8.6887	12.1642	0.0000	0.6250	0.8750	0.0000
29	Ni	10.4265	6.9510	0.0000	0.7500	0.5000	0.0000
30	Ni	12.1642	8.6887	0.0000	0.8750	0.6250	0.0000
31	Ni	10.4265	10.4265	0.0000	0.7500	0.7500	0.0000
32	Ni	12.1642	12.1642	0.0000	0.8750	0.8750	0.0000
33	Ni	0.0000	1.7377	1.7377	0.0000	0.1250	0.0792
34	Ni	1.7377	0.0000	1.7377	0.1250	0.0000	0.0792
35	Ni	0.0000	5.2132	1.7377	0.0000	0.3750	0.0792
36	Ni	1.7377	3.4755	1.7377	0.1250	0.2500	0.0792
37	Ni	3.4755	1.7377	1.7377	0.2500	0.1250	0.0792
38	Ni	5.2132	0.0000	1.7377	0.3750	0.0000	0.0792
39	Ni	3.4755	5.2132	1.7377	0.2500	0.3750	0.0792

40	Ni	5.2132	3.4755	1.7377	0.3750	0.2500	0.0792
41	Ni	0.0000	8.6887	1.7377	0.0000	0.6250	0.0792
42	Ni	1.7377	6.9510	1.7377	0.1250	0.5000	0.0792
43	Ni	0.0000	12.1642	1.7377	0.0000	0.8750	0.0792
44	Ni	1.7377	10.4265	1.7377	0.1250	0.7500	0.0792
45	Ni	3.4755	8.6887	1.7377	0.2500	0.6250	0.0792
46	Ni	5.2132	6.9510	1.7377	0.3750	0.5000	0.0792
47	Ni	3.4755	12.1642	1.7377	0.2500	0.8750	0.0792
48	Ni	5.2132	10.4265	1.7377	0.3750	0.7500	0.0792
49	Ni	6.9510	1.7377	1.7377	0.5000	0.1250	0.0792
50	Ni	8.6887	0.0000	1.7377	0.6250	0.0000	0.0792
51	Ni	6.9510	5.2132	1.7377	0.5000	0.3750	0.0792
52	Ni	8.6887	3.4755	1.7377	0.6250	0.2500	0.0792
53	Ni	10.4265	1.7377	1.7377	0.7500	0.1250	0.0792
54	Ni	12.1642	0.0000	1.7377	0.8750	0.0000	0.0792
55	Ni	10.4265	5.2132	1.7377	0.7500	0.3750	0.0792
56	Ni	12.1642	3.4755	1.7377	0.8750	0.2500	0.0792
57	Ni	6.9510	8.6887	1.7377	0.5000	0.6250	0.0792
58	Ni	8.6887	6.9510	1.7377	0.6250	0.5000	0.0792
59	Ni	6.9510	12.1642	1.7377	0.5000	0.8750	0.0792
60	Ni	8.6887	10.4265	1.7377	0.6250	0.7500	0.0792
61	Ni	10.4265	8.6887	1.7377	0.7500	0.6250	0.0792
62	Ni	12.1642	6.9510	1.7377	0.8750	0.5000	0.0792
63	Ni	10.4265	12.1642	1.7377	0.7500	0.8750	0.0792
64	Ni	12.1642	10.4265	1.7377	0.8750	0.7500	0.0792
65	Ni	0.0103	0.0154	3.5057	0.0007	0.0011	0.1597
66	Ni	1.7334	1.7753	3.4606	0.1247	0.1277	0.1577
67	Ni	13.8773	3.4780	3.5212	0.9982	0.2502	0.1604
68	Ni	1.6807	5.1859	3.4693	0.1209	0.3730	0.1580
69	Ni	3.4661	0.0072	3.5096	0.2493	0.0005	0.1599
70	Ni	5.2144	1.7443	3.5537	0.3751	0.1255	0.1619
71	Ni	3.5011	3.4451	3.4789	0.2518	0.2478	0.1585
72	Ni	5.2337	5.2116	3.4732	0.3765	0.3749	0.1582
73	Ni	0.0020	6.9379	3.5184	0.0001	0.4991	0.1603
74	Ni	1.7529	8.6540	3.5466	0.1261	0.6225	0.1616
75	Ni	13.8993	10.4162	3.5191	0.9998	0.7493	0.1603
76	Ni	1.7410	12.1733	3.5141	0.1252	0.8756	0.1601
77	Ni	3.4734	6.9744	3.4681	0.2498	0.5017	0.1580
78	Ni	5.2307	8.7087	3.5011	0.3763	0.6264	0.1595
79	Ni	3.4651	10.3944	3.5267	0.2493	0.7477	0.1607
80	Ni	5.2016	12.1629	3.4973	0.3742	0.8749	0.1593
81	Ni	6.9355	13.8955	3.5021	0.4989	0.9995	0.1595
82	Ni	8.6917	1.7104	3.4283	0.6252	0.1230	0.1562
83	Ni	6.9483	3.4603	3.4921	0.4998	0.2489	0.1591
84	Ni	8.6544	5.2230	3.5108	0.6225	0.3757	0.1599
85	Ni	10.4461	13.8799	3.5176	0.7514	0.9984	0.1602
86	Ni	12.1636	1.7470	3.5434	0.8750	0.1257	0.1614
87	Ni	10.4166	3.4653	3.4825	0.7493	0.2493	0.1586
88	Ni	12.1578	5.2214	3.4965	0.8745	0.3756	0.1593

89	Ni	6.9438	6.9478	3.5071	0.4995	0.4998	0.1598
90	Ni	8.6908	8.7009	3.4624	0.6251	0.6259	0.1577
91	Ni	6.9760	10.4362	3.5084	0.5018	0.7507	0.1598
92	Ni	8.6939	12.1964	3.4998	0.6254	0.8773	0.1594
93	Ni	10.4071	6.9691	3.5062	0.7486	0.5013	0.1597
94	Ni	12.1865	8.6616	3.5091	0.8766	0.6230	0.1599
95	Ni	10.4468	10.4456	3.4269	0.7515	0.7514	0.1561
96	Ni	12.1845	12.1630	3.4985	0.8765	0.8749	0.1594
97	Ni	0.0179	1.7905	5.2887	0.0013	0.1288	0.2409
98	Ni	1.7324	13.8926	5.2950	0.1246	0.9993	0.2412
99	Ni	13.8189	5.1800	5.3211	0.9940	0.3726	0.2424
100	Ni	1.7461	3.5677	5.4055	0.1256	0.2566	0.2463
101	Ni	3.4525	1.7580	5.2851	0.2483	0.1265	0.2408
102	Ni	5.1923	13.8925	5.2157	0.3735	0.9993	0.2376
103	Ni	3.5154	5.1810	5.0540	0.2529	0.3727	0.2302
104	Ni	5.2442	3.4421	5.3392	0.3772	0.2476	0.2432
105	Ni	0.0344	8.6753	5.2156	0.0025	0.6240	0.2376
106	Ni	1.7261	6.8562	5.2990	0.1242	0.4932	0.2414
107	Ni	0.0061	12.1626	5.2096	0.0004	0.8749	0.2373
108	Ni	1.7320	10.4329	5.2200	0.1246	0.7505	0.2378
109	Ni	3.4598	8.7168	5.3788	0.2489	0.6270	0.2450
110	Ni	5.2135	6.9720	5.2041	0.3750	0.5015	0.2371
111	Ni	3.4759	12.1499	5.2173	0.2500	0.8740	0.2377
112	Ni	5.2260	10.4420	5.1923	0.3759	0.7511	0.2365
113	Ni	6.9649	1.6828	5.2676	0.5010	0.1211	0.2400
114	Ni	8.6938	13.8694	5.2825	0.6254	0.9977	0.2406
115	Ni	6.9570	5.2069	5.2692	0.5004	0.3745	0.2400
116	Ni	8.6998	3.4351	5.2199	0.6258	0.2471	0.2378
117	Ni	10.4234	1.6957	5.2790	0.7498	0.1220	0.2405
118	Ni	12.1996	0.0102	5.2092	0.8775	0.0007	0.2373
119	Ni	10.3729	5.2163	5.1869	0.7461	0.3752	0.2363
120	Ni	12.0598	3.4871	5.2920	0.8675	0.2508	0.2411
121	Ni	6.9855	8.7527	5.2379	0.5025	0.6296	0.2386
122	Ni	8.7036	6.9699	5.2626	0.6261	0.5014	0.2397
123	Ni	6.9338	12.1634	5.2158	0.4988	0.8749	0.2376
124	Ni	8.7190	10.4914	5.1996	0.6272	0.7547	0.2369
125	Ni	10.4445	8.7252	5.1666	0.7513	0.6276	0.2354
126	Ni	12.1253	6.9987	5.3125	0.8722	0.5034	0.2420
127	Ni	10.4716	12.1739	5.2674	0.7532	0.8757	0.2400
128	Ni	12.1976	10.4184	5.2803	0.8774	0.7494	0.2406
129	O	6.1400	4.3780	6.7830	0.4417	0.3149	0.3090
130	O	4.2350	7.6086	6.7568	0.3046	0.5473	0.3078
131	O	6.1008	6.6059	8.7667	0.4388	0.4752	0.3994
132	O	7.9004	7.9327	6.7933	0.5683	0.5706	0.3095
133	O	10.4405	10.4068	6.2540	0.7510	0.7486	0.2849
134	O	9.5225	7.2405	8.9956	0.6850	0.5208	0.4098
135	O	7.6213	9.9462	8.8570	0.5482	0.7155	0.4035
136	Ce	5.9686	8.8203	8.0377	0.4293	0.6345	0.3662
137	Ce	4.4038	5.4995	7.7350	0.3168	0.3956	0.3524

138 Ce	8.2813	5.8073	8.0728	0.5957	0.4177	0.3678
139 Sm	9.4227	9.1760	8.0864	0.6778	0.6600	0.3684
140 C	11.4865	4.8857	6.7319	0.8263	0.3514	0.3067
141 O	12.5808	5.7004	6.7897	0.9050	0.4100	0.3093
142 O	10.8024	4.6134	7.7501	0.7770	0.3318	0.3531
143 O	2.1968	5.2788	6.4018	0.1580	0.3797	0.2916
144 O	1.7276	1.6972	6.1833	0.1243	0.1221	0.2817
145 O	8.6838	1.7199	6.1488	0.6246	0.1237	0.2801

/db/jmorales/CoNi-alloy/Profiles/CoNi(111)/TS

a =			9.831199646				
b =			9.831199645902503				
c =			21.020299912				
alpha =			90.0				
beta =			90.0				
gamma =			120.0				
	Atom	X	Y	Z	X	Y	Z
1	Ni	0.0000	0.0000	0.0000	0.0000	0.0000	0.0000
2	Ni	-1.2289	2.1285	0.0000	0.0000	0.2500	0.0000
3	Ni	-2.4578	4.2570	0.0000	-0.0000	0.5000	0.0000
4	Ni	-3.6867	6.3856	0.0000	-0.0000	0.7500	0.0000
5	Ni	4.9156	0.0000	0.0000	0.5000	0.0000	0.0000
6	Ni	3.6867	2.1285	0.0000	0.5000	0.2500	0.0000
7	Ni	2.4578	4.2570	0.0000	0.5000	0.5000	0.0000
8	Ni	1.2289	6.3856	0.0000	0.5000	0.7500	0.0000
9	Ni	2.4578	1.4190	2.0068	0.3333	0.1667	0.0955
10	Ni	1.2289	3.5475	2.0068	0.3333	0.4167	0.0955
11	Ni	0.0000	5.6760	2.0068	0.3333	0.6667	0.0955
12	Ni	-1.2289	7.8046	2.0068	0.3333	0.9167	0.0955
13	Ni	7.3734	1.4190	2.0068	0.8333	0.1667	0.0955
14	Ni	6.1445	3.5475	2.0068	0.8333	0.4167	0.0955
15	Ni	4.9156	5.6760	2.0068	0.8333	0.6667	0.0955
16	Ni	3.6867	7.8046	2.0068	0.8333	0.9167	0.0955
17	Ni	0.0508	2.8572	4.0318	0.1730	0.3356	0.1918
18	Ni	1.2773	0.7316	4.0310	0.1729	0.0859	0.1918
19	Ni	-2.4113	7.1248	4.0354	0.1731	0.8368	0.1920
20	Ni	-1.1754	4.9814	4.0340	0.1730	0.5851	0.1919
21	Ni	4.9478	2.8468	4.0485	0.6705	0.3344	0.1926
22	Ni	6.1931	0.7286	4.0275	0.6727	0.0856	0.1916
23	Ni	2.5051	7.1113	4.0252	0.6724	0.8352	0.1915
24	Ni	3.7304	4.9781	4.0317	0.6718	0.5847	0.1918
25	Ni	0.0586	0.0240	6.0494	0.0074	0.0028	0.2878
26	Ni	-1.1736	2.1612	6.0527	0.0075	0.2538	0.2879
27	Ni	-2.3855	4.2903	6.0514	0.0093	0.5039	0.2879
28	Ni	-3.6097	6.4157	6.0522	0.0096	0.7535	0.2879
29	Ni	5.0024	0.0321	6.0454	0.5107	0.0038	0.2876
30	Ni	3.7450	2.1820	6.1402	0.5091	0.2563	0.2921
31	Ni	2.5481	4.3744	6.0981	0.5161	0.5138	0.2901
32	Ni	1.2955	6.4421	6.0464	0.5101	0.7566	0.2876
33	Co	1.2289	2.1285	0.0000	0.2500	0.2500	0.0000
34	Co	2.4578	0.0000	0.0000	0.2500	0.0000	0.0000
35	Co	-1.2289	6.3856	0.0000	0.2500	0.7500	0.0000
36	Co	0.0000	4.2570	0.0000	0.2500	0.5000	0.0000
37	Co	6.1445	2.1285	0.0000	0.7500	0.2500	0.0000
38	Co	7.3734	0.0000	0.0000	0.7500	0.0000	0.0000
39	Co	3.6867	6.3856	0.0000	0.7500	0.7500	0.0000
40	Co	4.9156	4.2570	0.0000	0.7500	0.5000	0.0000
41	Co	-1.2289	3.5475	2.0068	0.0833	0.4167	0.0955

42	Co	0.0000	1.4190	2.0068	0.0833	0.1667	0.0955
43	Co	-3.6867	7.8046	2.0068	0.0833	0.9167	0.0955
44	Co	-2.4578	5.6760	2.0068	0.0833	0.6667	0.0955
45	Co	3.6867	3.5475	2.0068	0.5833	0.4167	0.0955
46	Co	4.9156	1.4190	2.0068	0.5833	0.1667	0.0955
47	Co	1.2289	7.8046	2.0068	0.5833	0.9167	0.0955
48	Co	2.4578	5.6760	2.0068	0.5833	0.6667	0.0955
49	Co	3.7292	0.7352	4.0862	0.4225	0.0864	0.1944
50	Co	2.4957	2.8561	4.0519	0.4216	0.3355	0.1928
51	Co	1.2708	4.9786	4.0655	0.4216	0.5847	0.1934
52	Co	0.0437	7.1110	4.0744	0.4220	0.8352	0.1938
53	Co	8.6492	0.7308	4.0723	0.9227	0.0858	0.1937
54	Co	7.4162	2.8584	4.0728	0.9222	0.3357	0.1938
55	Co	6.1864	4.9843	4.0704	0.9220	0.5854	0.1936
56	Co	4.9561	7.1120	4.0787	0.9218	0.8353	0.1940
57	Co	1.2535	2.1317	6.0985	0.2527	0.2504	0.2901
58	Co	2.5610	0.0026	6.0458	0.2606	0.0003	0.2876
59	Co	-1.1372	6.4296	6.0432	0.2619	0.7552	0.2875
60	Co	0.0701	4.3207	6.0449	0.2609	0.5075	0.2876
61	Co	6.2276	2.1717	6.0379	0.7610	0.2551	0.2872
62	Co	7.4646	0.0464	6.0440	0.7620	0.0055	0.2875
63	Co	3.7914	6.4429	6.0409	0.7640	0.7567	0.2874
64	Co	5.0311	4.3118	6.0339	0.7650	0.5064	0.2871
65	C	2.1700	4.0767	7.8633	0.4601	0.4788	0.3741
66	O	1.9570	4.3926	8.9754	0.4570	0.5159	0.4270
67	O	2.3601	2.3373	7.5475	0.3773	0.2745	0.3591

/db/jmorales/CoNi-alloy/Profiles/CoNi-Ce_3SmO_7/O-coverage/CO-O-O-coverage/CO-O-CoNi-Ce_3SmO_7-5O

a =				13.9033384264			
b =				13.9033384264			
c =				21.9516692133			
alpha =				90.0			
beta =				90.0			
gamma =				90.0			
	Atom	X	Y	Z	X	Y	Z
1	Ni	0.0000	0.0000	0.0000	0.0000	0.0000	0.0000
2	Ni	0.0000	3.4758	0.0000	0.0000	0.2500	0.0000
3	Ni	3.4758	0.0000	0.0000	0.2500	0.0000	0.0000
4	Ni	3.4758	3.4758	0.0000	0.2500	0.2500	0.0000
5	Ni	0.0000	6.9517	0.0000	0.0000	0.5000	0.0000
6	Ni	0.0000	10.4275	0.0000	0.0000	0.7500	0.0000
7	Ni	3.4758	6.9517	0.0000	0.2500	0.5000	0.0000
8	Ni	3.4758	10.4275	0.0000	0.2500	0.7500	0.0000
9	Ni	6.9517	0.0000	0.0000	0.5000	0.0000	0.0000
10	Ni	6.9517	3.4758	0.0000	0.5000	0.2500	0.0000
11	Ni	10.4275	0.0000	0.0000	0.7500	0.0000	0.0000
12	Ni	10.4275	3.4758	0.0000	0.7500	0.2500	0.0000
13	Ni	6.9517	6.9517	0.0000	0.5000	0.5000	0.0000
14	Ni	6.9517	10.4275	0.0000	0.5000	0.7500	0.0000
15	Ni	10.4275	6.9517	0.0000	0.7500	0.5000	0.0000
16	Ni	10.4275	10.4275	0.0000	0.7500	0.7500	0.0000
17	Ni	0.0000	1.7379	1.7379	0.0000	0.1250	0.0792
18	Ni	0.0000	5.2138	1.7379	0.0000	0.3750	0.0792
19	Ni	3.4758	1.7379	1.7379	0.2500	0.1250	0.0792
20	Ni	3.4758	5.2138	1.7379	0.2500	0.3750	0.0792
21	Ni	0.0000	8.6896	1.7379	0.0000	0.6250	0.0792
22	Ni	0.0000	12.1654	1.7379	0.0000	0.8750	0.0792
23	Ni	3.4758	8.6896	1.7379	0.2500	0.6250	0.0792
24	Ni	3.4758	12.1654	1.7379	0.2500	0.8750	0.0792
25	Ni	6.9517	1.7379	1.7379	0.5000	0.1250	0.0792
26	Ni	6.9517	5.2138	1.7379	0.5000	0.3750	0.0792
27	Ni	10.4275	1.7379	1.7379	0.7500	0.1250	0.0792
28	Ni	10.4275	5.2138	1.7379	0.7500	0.3750	0.0792
29	Ni	6.9517	8.6896	1.7379	0.5000	0.6250	0.0792
30	Ni	6.9517	12.1654	1.7379	0.5000	0.8750	0.0792
31	Ni	10.4275	8.6896	1.7379	0.7500	0.6250	0.0792
32	Ni	10.4275	12.1654	1.7379	0.7500	0.8750	0.0792
33	Ni	0.0005	13.8934	3.4648	0.0000	0.9993	0.1578
34	Ni	0.0246	3.4577	3.5274	0.0018	0.2487	0.1607
35	Ni	3.4717	13.8976	3.4460	0.2497	0.9996	0.1570
36	Ni	3.4830	3.4329	3.4429	0.2505	0.2469	0.1568
37	Ni	0.0026	6.9636	3.5186	0.0002	0.5009	0.1603
38	Ni	13.8780	10.4514	3.4478	0.9982	0.7517	0.1571
39	Ni	3.4661	6.9616	3.4745	0.2493	0.5007	0.1583

40	Ni	3.4775	10.4246	3.5413	0.2501	0.7498	0.1613
41	Ni	6.9496	0.0136	3.4775	0.4999	0.0010	0.1584
42	Ni	6.9351	3.4785	3.4552	0.4988	0.2502	0.1574
43	Ni	10.4324	0.0193	3.4486	0.7504	0.0014	0.1571
44	Ni	10.4115	3.4730	3.5127	0.7488	0.2498	0.1600
45	Ni	6.9568	6.9606	3.5084	0.5004	0.5006	0.1598
46	Ni	6.9693	10.4364	3.4888	0.5013	0.7506	0.1589
47	Ni	10.4355	6.9296	3.4868	0.7506	0.4984	0.1588
48	Ni	10.4452	10.4444	3.5166	0.7513	0.7512	0.1602
49	Ni	0.0210	1.7779	5.2666	0.0015	0.1279	0.2399
50	Ni	0.0184	5.2049	5.2094	0.0013	0.3744	0.2373
51	Ni	3.5082	1.6909	5.2409	0.2523	0.1216	0.2387
52	Ni	3.4705	5.2185	5.0476	0.2496	0.3753	0.2299
53	Ni	13.8631	8.6665	5.2269	0.9971	0.6233	0.2381
54	Ni	13.8848	12.1877	5.2868	0.9987	0.8766	0.2408
55	Ni	3.4285	8.6799	5.2261	0.2466	0.6243	0.2381
56	Ni	3.4801	12.1220	5.2654	0.2503	0.8719	0.2399
57	Ni	6.8948	1.7110	5.3053	0.4959	0.1231	0.2417
58	Ni	6.9774	5.2639	5.3721	0.5019	0.3786	0.2447
59	Ni	10.4319	1.8078	5.2517	0.7503	0.1300	0.2392
60	Ni	10.3998	5.2017	5.2077	0.7480	0.3741	0.2372
61	Ni	6.9806	8.6987	5.2320	0.5021	0.6257	0.2383
62	Ni	7.0332	12.2319	5.2531	0.5059	0.8798	0.2393
63	Ni	10.4536	8.6652	5.1816	0.7519	0.6232	0.2360
64	Ni	10.3673	12.1704	5.3483	0.7457	0.8754	0.2436
65	Co	1.7379	1.7379	0.0000	0.1250	0.1250	0.0000
66	Co	1.7379	5.2138	0.0000	0.1250	0.3750	0.0000
67	Co	5.2138	1.7379	0.0000	0.3750	0.1250	0.0000
68	Co	5.2138	5.2138	0.0000	0.3750	0.3750	0.0000
69	Co	1.7379	8.6896	0.0000	0.1250	0.6250	0.0000
70	Co	1.7379	12.1654	0.0000	0.1250	0.8750	0.0000
71	Co	5.2138	8.6896	0.0000	0.3750	0.6250	0.0000
72	Co	5.2138	12.1654	0.0000	0.3750	0.8750	0.0000
73	Co	8.6896	1.7379	0.0000	0.6250	0.1250	0.0000
74	Co	8.6896	5.2138	0.0000	0.6250	0.3750	0.0000
75	Co	12.1654	1.7379	0.0000	0.8750	0.1250	0.0000
76	Co	12.1654	5.2138	0.0000	0.8750	0.3750	0.0000
77	Co	8.6896	8.6896	0.0000	0.6250	0.6250	0.0000
78	Co	8.6896	12.1654	0.0000	0.6250	0.8750	0.0000
79	Co	12.1654	8.6896	0.0000	0.8750	0.6250	0.0000
80	Co	12.1654	12.1654	0.0000	0.8750	0.8750	0.0000
81	Co	1.7379	0.0000	1.7379	0.1250	0.0000	0.0792
82	Co	1.7379	3.4758	1.7379	0.1250	0.2500	0.0792
83	Co	5.2138	0.0000	1.7379	0.3750	0.0000	0.0792
84	Co	5.2138	3.4758	1.7379	0.3750	0.2500	0.0792
85	Co	1.7379	6.9517	1.7379	0.1250	0.5000	0.0792
86	Co	1.7379	10.4275	1.7379	0.1250	0.7500	0.0792
87	Co	5.2138	6.9517	1.7379	0.3750	0.5000	0.0792
88	Co	5.2138	10.4275	1.7379	0.3750	0.7500	0.0792

89	Co	8.6896	0.0000	1.7379	0.6250	0.0000	0.0792
90	Co	8.6896	3.4758	1.7379	0.6250	0.2500	0.0792
91	Co	12.1654	0.0000	1.7379	0.8750	0.0000	0.0792
92	Co	12.1654	3.4758	1.7379	0.8750	0.2500	0.0792
93	Co	8.6896	6.9517	1.7379	0.6250	0.5000	0.0792
94	Co	8.6896	10.4275	1.7379	0.6250	0.7500	0.0792
95	Co	12.1654	6.9517	1.7379	0.8750	0.5000	0.0792
96	Co	12.1654	10.4275	1.7379	0.8750	0.7500	0.0792
97	Co	1.7433	1.7398	3.5617	0.1254	0.1251	0.1623
98	Co	1.7079	5.2204	3.5109	0.1228	0.3755	0.1599
99	Co	5.2125	1.7404	3.5299	0.3749	0.1252	0.1608
100	Co	5.2438	5.1919	3.4929	0.3772	0.3734	0.1591
101	Co	1.7202	8.6981	3.5200	0.1237	0.6256	0.1604
102	Co	1.7399	12.1672	3.5486	0.1251	0.8751	0.1617
103	Co	5.1952	8.7003	3.5540	0.3737	0.6258	0.1619
104	Co	5.2307	12.1426	3.5653	0.3762	0.8734	0.1624
105	Co	8.6760	1.7505	3.5494	0.6240	0.1259	0.1617
106	Co	8.6645	5.2002	3.5615	0.6232	0.3740	0.1622
107	Co	12.1755	1.7478	3.5513	0.8757	0.1257	0.1618
108	Co	12.1716	5.1992	3.5519	0.8754	0.3740	0.1618
109	Co	8.6942	8.6697	3.5159	0.6253	0.6236	0.1602
110	Co	8.7157	12.1895	3.4887	0.6269	0.8767	0.1589
111	Co	12.1622	8.6846	3.5351	0.8748	0.6246	0.1610
112	Co	12.1309	12.1859	3.5886	0.8725	0.8765	0.1635
113	Co	1.7535	13.8967	5.3566	0.1261	0.9995	0.2440
114	Co	1.8189	3.4968	5.2861	0.1308	0.2515	0.2408
115	Co	5.2353	13.8535	5.3233	0.3766	0.9964	0.2425
116	Co	5.1734	3.4735	5.3122	0.3721	0.2498	0.2420
117	Co	1.7129	6.9534	5.2869	0.1232	0.5001	0.2408
118	Co	1.6737	10.4509	5.2241	0.1204	0.7517	0.2380
119	Co	5.2255	6.9197	5.1484	0.3758	0.4977	0.2345
120	Co	5.2248	10.4170	5.2430	0.3758	0.7492	0.2388
121	Co	8.6788	0.0914	5.3275	0.6242	0.0066	0.2427
122	Co	8.6277	3.4648	5.2641	0.6206	0.2492	0.2398
123	Co	12.1474	0.0266	5.3479	0.8737	0.0019	0.2436
124	Co	12.1761	3.5044	5.2023	0.8758	0.2521	0.2370
125	Co	8.7350	6.9823	5.1896	0.6283	0.5022	0.2364
126	Co	8.7529	10.3502	5.1884	0.6296	0.7444	0.2364
127	Co	12.1530	6.9116	5.2095	0.8741	0.4971	0.2373
128	Co	12.1880	10.4237	5.2678	0.8766	0.7497	0.2400
129	O	5.8211	6.1495	6.7778	0.4187	0.4423	0.3088
130	O	2.6176	7.7370	6.7956	0.1883	0.5565	0.3096
131	O	4.7452	7.7410	8.8572	0.3413	0.5568	0.4035
132	O	6.0275	9.6247	6.8355	0.4335	0.6923	0.3114
133	O	7.7607	9.5914	9.1311	0.5582	0.6899	0.4160
134	O	4.9141	11.2947	8.8882	0.3534	0.8124	0.4049
135	O	8.5509	11.6601	6.4245	0.6150	0.8387	0.2927
136	Ce	3.7988	9.7221	8.0509	0.2732	0.6993	0.3668
137	Ce	3.7510	5.9932	7.7484	0.2698	0.4311	0.3530

138	Ce	6.9948	7.8476	8.1756	0.5031	0.5644	0.3724
139	Sm	6.9680	11.3627	8.2138	0.5012	0.8173	0.3742
140	C	6.8544	0.0620	6.3761	0.4930	0.0045	0.2905
141	O	6.9863	13.8811	7.6154	0.5025	0.9984	0.3469
142	O	3.5213	13.8436	6.1845	0.2533	0.9957	0.2817
143	O	10.4092	0.1150	6.1925	0.7487	0.0083	0.2821
144	O	0.0041	0.0679	6.2039	0.0003	0.0049	0.2826
145	O	3.4501	3.8346	6.4025	0.2481	0.2758	0.2917
146	O	6.9697	3.4248	6.2272	0.5013	0.2463	0.2837
147	O	0.0281	10.3664	6.2220	0.0020	0.7456	0.2834

/db/jmorales/CoNi-alloy/Profiles/CoNi-Ce_3SmO_7/O-coverage/M-CO2-O-coverage/M-CO_2-CoNi-Ce_3SmO_7-O

a =			13.9033384264				
b =			13.9033384264				
c =			21.9516692133				
alpha =			90.0				
beta =			90.0				
gamma =			90.0				
	Atom	X	Y	Z	X	Y	Z
1	Ni	0.0000	0.0000	0.0000	0.0000	0.0000	0.0000
2	Ni	0.0000	3.4758	0.0000	0.0000	0.2500	0.0000
3	Ni	3.4758	0.0000	0.0000	0.2500	0.0000	0.0000
4	Ni	3.4758	3.4758	0.0000	0.2500	0.2500	0.0000
5	Ni	0.0000	6.9517	0.0000	0.0000	0.5000	0.0000
6	Ni	0.0000	10.4275	0.0000	0.0000	0.7500	0.0000
7	Ni	3.4758	6.9517	0.0000	0.2500	0.5000	0.0000
8	Ni	3.4758	10.4275	0.0000	0.2500	0.7500	0.0000
9	Ni	6.9517	0.0000	0.0000	0.5000	0.0000	0.0000
10	Ni	6.9517	3.4758	0.0000	0.5000	0.2500	0.0000
11	Ni	10.4275	0.0000	0.0000	0.7500	0.0000	0.0000
12	Ni	10.4275	3.4758	0.0000	0.7500	0.2500	0.0000
13	Ni	6.9517	6.9517	0.0000	0.5000	0.5000	0.0000
14	Ni	6.9517	10.4275	0.0000	0.5000	0.7500	0.0000
15	Ni	10.4275	6.9517	0.0000	0.7500	0.5000	0.0000
16	Ni	10.4275	10.4275	0.0000	0.7500	0.7500	0.0000
17	Ni	0.0000	1.7379	1.7379	0.0000	0.1250	0.0792
18	Ni	0.0000	5.2138	1.7379	0.0000	0.3750	0.0792
19	Ni	3.4758	1.7379	1.7379	0.2500	0.1250	0.0792
20	Ni	3.4758	5.2138	1.7379	0.2500	0.3750	0.0792
21	Ni	0.0000	8.6896	1.7379	0.0000	0.6250	0.0792
22	Ni	0.0000	12.1654	1.7379	0.0000	0.8750	0.0792
23	Ni	3.4758	8.6896	1.7379	0.2500	0.6250	0.0792
24	Ni	3.4758	12.1654	1.7379	0.2500	0.8750	0.0792
25	Ni	6.9517	1.7379	1.7379	0.5000	0.1250	0.0792
26	Ni	6.9517	5.2138	1.7379	0.5000	0.3750	0.0792
27	Ni	10.4275	1.7379	1.7379	0.7500	0.1250	0.0792
28	Ni	10.4275	5.2138	1.7379	0.7500	0.3750	0.0792
29	Ni	6.9517	8.6896	1.7379	0.5000	0.6250	0.0792
30	Ni	6.9517	12.1654	1.7379	0.5000	0.8750	0.0792
31	Ni	10.4275	8.6896	1.7379	0.7500	0.6250	0.0792
32	Ni	10.4275	12.1654	1.7379	0.7500	0.8750	0.0792
33	Ni	13.8913	13.8996	3.5019	0.9991	0.9997	0.1595
34	Ni	0.0059	3.4755	3.4998	0.0004	0.2500	0.1594
35	Ni	3.4738	-0.0009	3.4992	0.2499	-0.0001	0.1594
36	Ni	3.4617	3.4231	3.5006	0.2490	0.2462	0.1595
37	Ni	13.9017	6.9562	3.5079	0.9999	0.5003	0.1598
38	Ni	13.8787	10.4237	3.4782	0.9982	0.7497	0.1584
39	Ni	3.4739	6.9657	3.4981	0.2499	0.5010	0.1594

40	Ni	3.4982	10.3930	3.5087	0.2516	0.7475	0.1598
41	Ni	6.9527	0.0172	3.5032	0.5001	0.0012	0.1596
42	Ni	6.9521	3.4923	3.5185	0.5000	0.2512	0.1603
43	Ni	10.4294	0.0149	3.4493	0.7501	0.0011	0.1571
44	Ni	10.4321	3.4659	3.5159	0.7503	0.2493	0.1602
45	Ni	6.9574	6.9631	3.5067	0.5004	0.5008	0.1597
46	Ni	6.9740	10.4402	3.4747	0.5016	0.7509	0.1583
47	Ni	10.4327	6.9424	3.4887	0.7504	0.4993	0.1589
48	Ni	10.4272	10.4464	3.5056	0.7500	0.7514	0.1597
49	Ni	0.0215	1.7361	5.2067	0.0015	0.1249	0.2372
50	Ni	0.0107	5.2205	5.2138	0.0008	0.3755	0.2375
51	Ni	3.4364	1.7547	5.2987	0.2472	0.1262	0.2414
52	Ni	3.4356	5.1758	5.0981	0.2471	0.3723	0.2322
53	Ni	13.8584	8.7095	5.1823	0.9968	0.6264	0.2361
54	Ni	13.8767	12.1424	5.1904	0.9981	0.8733	0.2364
55	Ni	3.4625	8.7015	5.2571	0.2490	0.6259	0.2395
56	Ni	3.4704	12.1081	5.1835	0.2496	0.8709	0.2361
57	Ni	6.9781	1.7500	5.1999	0.5019	0.1259	0.2369
58	Ni	6.9744	5.1883	5.3661	0.5016	0.3732	0.2445
59	Ni	10.4274	1.7740	5.2570	0.7500	0.1276	0.2395
60	Ni	10.4257	5.2107	5.2122	0.7499	0.3748	0.2374
61	Ni	6.9989	8.7128	5.2239	0.5034	0.6267	0.2380
62	Ni	6.9458	12.2289	5.1778	0.4996	0.8796	0.2359
63	Ni	10.4198	8.6917	5.2027	0.7494	0.6252	0.2370
64	Ni	10.4765	12.1747	5.2817	0.7535	0.8757	0.2406
65	Co	1.7379	1.7379	0.0000	0.1250	0.1250	0.0000
66	Co	1.7379	5.2138	0.0000	0.1250	0.3750	0.0000
67	Co	5.2138	1.7379	0.0000	0.3750	0.1250	0.0000
68	Co	5.2138	5.2138	0.0000	0.3750	0.3750	0.0000
69	Co	1.7379	8.6896	0.0000	0.1250	0.6250	0.0000
70	Co	1.7379	12.1654	0.0000	0.1250	0.8750	0.0000
71	Co	5.2138	8.6896	0.0000	0.3750	0.6250	0.0000
72	Co	5.2138	12.1654	0.0000	0.3750	0.8750	0.0000
73	Co	8.6896	1.7379	0.0000	0.6250	0.1250	0.0000
74	Co	8.6896	5.2138	0.0000	0.6250	0.3750	0.0000
75	Co	12.1654	1.7379	0.0000	0.8750	0.1250	0.0000
76	Co	12.1654	5.2138	0.0000	0.8750	0.3750	0.0000
77	Co	8.6896	8.6896	0.0000	0.6250	0.6250	0.0000
78	Co	8.6896	12.1654	0.0000	0.6250	0.8750	0.0000
79	Co	12.1654	8.6896	0.0000	0.8750	0.6250	0.0000
80	Co	12.1654	12.1654	0.0000	0.8750	0.8750	0.0000
81	Co	1.7379	0.0000	1.7379	0.1250	0.0000	0.0792
82	Co	1.7379	3.4758	1.7379	0.1250	0.2500	0.0792
83	Co	5.2138	0.0000	1.7379	0.3750	0.0000	0.0792
84	Co	5.2138	3.4758	1.7379	0.3750	0.2500	0.0792
85	Co	1.7379	6.9517	1.7379	0.1250	0.5000	0.0792
86	Co	1.7379	10.4275	1.7379	0.1250	0.7500	0.0792
87	Co	5.2138	6.9517	1.7379	0.3750	0.5000	0.0792
88	Co	5.2138	10.4275	1.7379	0.3750	0.7500	0.0792

89	Co	8.6896	0.0000	1.7379	0.6250	0.0000	0.0792
90	Co	8.6896	3.4758	1.7379	0.6250	0.2500	0.0792
91	Co	12.1654	0.0000	1.7379	0.8750	0.0000	0.0792
92	Co	12.1654	3.4758	1.7379	0.8750	0.2500	0.0792
93	Co	8.6896	6.9517	1.7379	0.6250	0.5000	0.0792
94	Co	8.6896	10.4275	1.7379	0.6250	0.7500	0.0792
95	Co	12.1654	6.9517	1.7379	0.8750	0.5000	0.0792
96	Co	12.1654	10.4275	1.7379	0.8750	0.7500	0.0792
97	Co	1.7367	1.7151	3.5604	0.1249	0.1234	0.1622
98	Co	1.7114	5.2381	3.5240	0.1231	0.3768	0.1605
99	Co	5.2075	1.7257	3.5687	0.3745	0.1241	0.1626
100	Co	5.2282	5.1910	3.5215	0.3760	0.3734	0.1604
101	Co	1.7294	8.6870	3.5230	0.1244	0.6248	0.1605
102	Co	1.7304	12.1562	3.5200	0.1245	0.8743	0.1604
103	Co	5.2086	8.6821	3.5651	0.3746	0.6245	0.1624
104	Co	5.2192	12.1659	3.5539	0.3754	0.8750	0.1619
105	Co	8.6959	1.7369	3.5442	0.6255	0.1249	0.1615
106	Co	8.6698	5.2067	3.5663	0.6236	0.3745	0.1625
107	Co	12.1762	1.7461	3.5506	0.8758	0.1256	0.1617
108	Co	12.1696	5.2099	3.5591	0.8753	0.3747	0.1621
109	Co	8.6925	8.6950	3.5336	0.6252	0.6254	0.1610
110	Co	8.7095	12.1989	3.4751	0.6264	0.8774	0.1583
111	Co	12.1462	8.6873	3.5390	0.8736	0.6248	0.1612
112	Co	12.1496	12.1773	3.5554	0.8739	0.8759	0.1620
113	Co	1.7280	13.8039	5.2671	0.1243	0.9928	0.2399
114	Co	1.7196	3.4937	5.2187	0.1237	0.2513	0.2377
115	Co	5.2179	0.0098	5.2894	0.3753	0.0007	0.2410
116	Co	5.1983	3.4575	5.1960	0.3739	0.2487	0.2367
117	Co	1.7059	6.9914	5.3079	0.1227	0.5029	0.2418
118	Co	1.6908	10.4320	5.1542	0.1216	0.7503	0.2348
119	Co	5.2336	6.8775	5.1821	0.3764	0.4947	0.2361
120	Co	5.2531	10.4280	5.2267	0.3778	0.7500	0.2381
121	Co	8.7206	-0.0043	5.3557	0.6272	-0.0003	0.2440
122	Co	8.6941	3.4748	5.2180	0.6253	0.2499	0.2377
123	Co	12.1931	0.0284	5.2770	0.8770	0.0020	0.2404
124	Co	12.1921	3.4877	5.2224	0.8769	0.2509	0.2379
125	Co	8.7260	6.9650	5.1794	0.6276	0.5010	0.2359
126	Co	8.7386	10.4774	5.2076	0.6285	0.7536	0.2372
127	Co	12.1547	6.9514	5.2198	0.8742	0.5000	0.2378
128	Co	12.1644	10.4158	5.2131	0.8749	0.7492	0.2375
129	O	5.7982	5.9748	6.8016	0.4170	0.4297	0.3098
130	O	2.5284	7.9502	6.8013	0.1819	0.5718	0.3098
131	O	4.7899	7.9262	8.7355	0.3445	0.5701	0.3979
132	O	6.2107	9.7307	6.8213	0.4467	0.6999	0.3107
133	O	8.1464	9.3330	8.9623	0.5859	0.6713	0.4083
134	O	5.4468	11.3101	9.0650	0.3918	0.8135	0.4130
135	O	8.4881	12.0416	6.3501	0.6105	0.8661	0.2893
136	Ce	4.0265	10.1347	8.0519	0.2896	0.7289	0.3668
137	Ce	3.6858	6.2766	7.7390	0.2651	0.4514	0.3525

138	Ce	7.0282	7.7487	8.0132	0.5055	0.5573	0.3650
139	Sm	7.4246	11.1835	8.2570	0.5340	0.8044	0.3761
140	C	3.0624	13.2910	6.7253	0.2203	0.9560	0.3064
141	O	3.9265	0.4457	6.7887	0.2824	0.0321	0.3093
142	O	2.8403	12.5659	7.7359	0.2043	0.9038	0.3524
143	O	10.5447	0.1047	6.2227	0.7584	0.0075	0.2835

/db/jmorales/CoNi-alloy/Profiles/CoNi-Ce_3SmO_7/O-coverage/TS-O-coverage/TS-CoNi-Ce_3SmO_7-5O

a =			13.9033384264				
b =			13.9033384264				
c =			21.9516692133				
alpha =			90.0				
beta =			90.0				
gamma =			90.0				
	Atom	X	Y	Z	X	Y	Z
1	Ni	0.0000	0.0000	0.0000	0.0000	0.0000	0.0000
2	Ni	0.0000	3.4758	0.0000	0.0000	0.2500	0.0000
3	Ni	3.4758	0.0000	0.0000	0.2500	0.0000	0.0000
4	Ni	3.4758	3.4758	0.0000	0.2500	0.2500	0.0000
5	Ni	0.0000	6.9517	0.0000	0.0000	0.5000	0.0000
6	Ni	0.0000	10.4275	0.0000	0.0000	0.7500	0.0000
7	Ni	3.4758	6.9517	0.0000	0.2500	0.5000	0.0000
8	Ni	3.4758	10.4275	0.0000	0.2500	0.7500	0.0000
9	Ni	6.9517	0.0000	0.0000	0.5000	0.0000	0.0000
10	Ni	6.9517	3.4758	0.0000	0.5000	0.2500	0.0000
11	Ni	10.4275	0.0000	0.0000	0.7500	0.0000	0.0000
12	Ni	10.4275	3.4758	0.0000	0.7500	0.2500	0.0000
13	Ni	6.9517	6.9517	0.0000	0.5000	0.5000	0.0000
14	Ni	6.9517	10.4275	0.0000	0.5000	0.7500	0.0000
15	Ni	10.4275	6.9517	0.0000	0.7500	0.5000	0.0000
16	Ni	10.4275	10.4275	0.0000	0.7500	0.7500	0.0000
17	Ni	0.0000	1.7379	1.7379	0.0000	0.1250	0.0792
18	Ni	0.0000	5.2138	1.7379	0.0000	0.3750	0.0792
19	Ni	3.4758	1.7379	1.7379	0.2500	0.1250	0.0792
20	Ni	3.4758	5.2138	1.7379	0.2500	0.3750	0.0792
21	Ni	0.0000	8.6896	1.7379	0.0000	0.6250	0.0792
22	Ni	0.0000	12.1654	1.7379	0.0000	0.8750	0.0792
23	Ni	3.4758	8.6896	1.7379	0.2500	0.6250	0.0792
24	Ni	3.4758	12.1654	1.7379	0.2500	0.8750	0.0792
25	Ni	6.9517	1.7379	1.7379	0.5000	0.1250	0.0792
26	Ni	6.9517	5.2138	1.7379	0.5000	0.3750	0.0792
27	Ni	10.4275	1.7379	1.7379	0.7500	0.1250	0.0792
28	Ni	10.4275	5.2138	1.7379	0.7500	0.3750	0.0792
29	Ni	6.9517	8.6896	1.7379	0.5000	0.6250	0.0792
30	Ni	6.9517	12.1654	1.7379	0.5000	0.8750	0.0792
31	Ni	10.4275	8.6896	1.7379	0.7500	0.6250	0.0792
32	Ni	10.4275	12.1654	1.7379	0.7500	0.8750	0.0792
33	Ni	0.0002	13.8971	3.4565	0.0000	0.9996	0.1575
34	Ni	0.0132	3.4672	3.5181	0.0009	0.2494	0.1603
35	Ni	3.4607	0.0237	3.5192	0.2489	0.0017	0.1603
36	Ni	3.4883	3.4494	3.4257	0.2509	0.2481	0.1561
37	Ni	0.0010	6.9627	3.5202	0.0001	0.5008	0.1604
38	Ni	13.8886	10.4494	3.4479	0.9989	0.7516	0.1571
39	Ni	3.4695	6.9473	3.4852	0.2495	0.4997	0.1588

40	Ni	3.4618	10.3946	3.5124	0.2490	0.7476	0.1600
41	Ni	6.9525	0.0190	3.5080	0.5001	0.0014	0.1598
42	Ni	6.9496	3.4680	3.4590	0.4998	0.2494	0.1576
43	Ni	10.4463	0.0074	3.4528	0.7513	0.0005	0.1573
44	Ni	10.4143	3.4731	3.5112	0.7491	0.2498	0.1600
45	Ni	6.9655	6.9721	3.5042	0.5010	0.5015	0.1596
46	Ni	6.9626	10.4458	3.4816	0.5008	0.7513	0.1586
47	Ni	10.4390	6.9436	3.4858	0.7508	0.4994	0.1588
48	Ni	10.4523	10.4517	3.5191	0.7518	0.7517	0.1603
49	Ni	0.0199	1.7906	5.2626	0.0014	0.1288	0.2397
50	Ni	0.0043	5.2135	5.2100	0.0003	0.3750	0.2373
51	Ni	3.4843	1.7539	5.2448	0.2506	0.1262	0.2389
52	Ni	3.4829	5.1920	5.0654	0.2505	0.3734	0.2308
53	Ni	13.8710	8.6620	5.2343	0.9977	0.6230	0.2384
54	Ni	0.0073	12.1819	5.2746	0.0005	0.8762	0.2403
55	Ni	3.4258	8.6561	5.2319	0.2464	0.6226	0.2383
56	Ni	3.4275	12.1250	5.1840	0.2465	0.8721	0.2362
57	Ni	6.9670	1.7253	5.3499	0.5011	0.1241	0.2437
58	Ni	6.9983	5.2861	5.3619	0.5034	0.3802	0.2443
59	Ni	10.4475	1.7852	5.2461	0.7514	0.1284	0.2390
60	Ni	10.4072	5.2169	5.2019	0.7485	0.3752	0.2370
61	Ni	6.9847	8.7274	5.2219	0.5024	0.6277	0.2379
62	Ni	6.9944	12.1480	5.2083	0.5031	0.8737	0.2373
63	Ni	10.4555	8.6841	5.1866	0.7520	0.6246	0.2363
64	Ni	10.4054	12.1775	5.3374	0.7484	0.8759	0.2431
65	Co	1.7379	1.7379	0.0000	0.1250	0.1250	0.0000
66	Co	1.7379	5.2138	0.0000	0.1250	0.3750	0.0000
67	Co	5.2138	1.7379	0.0000	0.3750	0.1250	0.0000
68	Co	5.2138	5.2138	0.0000	0.3750	0.3750	0.0000
69	Co	1.7379	8.6896	0.0000	0.1250	0.6250	0.0000
70	Co	1.7379	12.1654	0.0000	0.1250	0.8750	0.0000
71	Co	5.2138	8.6896	0.0000	0.3750	0.6250	0.0000
72	Co	5.2138	12.1654	0.0000	0.3750	0.8750	0.0000
73	Co	8.6896	1.7379	0.0000	0.6250	0.1250	0.0000
74	Co	8.6896	5.2138	0.0000	0.6250	0.3750	0.0000
75	Co	12.1654	1.7379	0.0000	0.8750	0.1250	0.0000
76	Co	12.1654	5.2138	0.0000	0.8750	0.3750	0.0000
77	Co	8.6896	8.6896	0.0000	0.6250	0.6250	0.0000
78	Co	8.6896	12.1654	0.0000	0.6250	0.8750	0.0000
79	Co	12.1654	8.6896	0.0000	0.8750	0.6250	0.0000
80	Co	12.1654	12.1654	0.0000	0.8750	0.8750	0.0000
81	Co	1.7379	0.0000	1.7379	0.1250	0.0000	0.0792
82	Co	1.7379	3.4758	1.7379	0.1250	0.2500	0.0792
83	Co	5.2138	0.0000	1.7379	0.3750	0.0000	0.0792
84	Co	5.2138	3.4758	1.7379	0.3750	0.2500	0.0792
85	Co	1.7379	6.9517	1.7379	0.1250	0.5000	0.0792
86	Co	1.7379	10.4275	1.7379	0.1250	0.7500	0.0792
87	Co	5.2138	6.9517	1.7379	0.3750	0.5000	0.0792
88	Co	5.2138	10.4275	1.7379	0.3750	0.7500	0.0792

89	Co	8.6896	0.0000	1.7379	0.6250	0.0000	0.0792
90	Co	8.6896	3.4758	1.7379	0.6250	0.2500	0.0792
91	Co	12.1654	0.0000	1.7379	0.8750	0.0000	0.0792
92	Co	12.1654	3.4758	1.7379	0.8750	0.2500	0.0792
93	Co	8.6896	6.9517	1.7379	0.6250	0.5000	0.0792
94	Co	8.6896	10.4275	1.7379	0.6250	0.7500	0.0792
95	Co	12.1654	6.9517	1.7379	0.8750	0.5000	0.0792
96	Co	12.1654	10.4275	1.7379	0.8750	0.7500	0.0792
97	Co	1.7422	1.7589	3.5548	0.1253	0.1265	0.1619
98	Co	1.7093	5.2236	3.5176	0.1229	0.3757	0.1602
99	Co	5.2186	1.7345	3.5662	0.3753	0.1248	0.1625
100	Co	5.2541	5.1919	3.4957	0.3779	0.3734	0.1592
101	Co	1.7183	8.6791	3.5229	0.1236	0.6242	0.1605
102	Co	1.7373	12.1698	3.5267	0.1250	0.8753	0.1607
103	Co	5.1945	8.6944	3.5478	0.3736	0.6253	0.1616
104	Co	5.2016	12.1462	3.5498	0.3741	0.8736	0.1617
105	Co	8.6893	1.7401	3.5526	0.6250	0.1252	0.1618
106	Co	8.6718	5.2089	3.5540	0.6237	0.3746	0.1619
107	Co	12.1780	1.7477	3.5468	0.8759	0.1257	0.1616
108	Co	12.1668	5.2070	3.5490	0.8751	0.3745	0.1617
109	Co	8.6987	8.6873	3.5175	0.6257	0.6248	0.1602
110	Co	8.7107	12.1914	3.4874	0.6265	0.8769	0.1589
111	Co	12.1671	8.6921	3.5387	0.8751	0.6252	0.1612
112	Co	12.1499	12.1834	3.5802	0.8739	0.8763	0.1631
113	Co	1.7522	0.0151	5.2666	0.1260	0.0011	0.2399
114	Co	1.7804	3.5113	5.2777	0.1281	0.2525	0.2404
115	Co	5.1351	13.8621	5.2508	0.3693	0.9970	0.2392
116	Co	5.2217	3.4706	5.3052	0.3756	0.2496	0.2417
117	Co	1.7162	6.9359	5.3075	0.1234	0.4989	0.2418
118	Co	1.6925	10.4369	5.2307	0.1217	0.7507	0.2383
119	Co	5.2383	6.9237	5.1463	0.3768	0.4980	0.2344
120	Co	5.1753	10.3973	5.2217	0.3722	0.7478	0.2379
121	Co	8.7084	0.0562	5.3416	0.6263	0.0040	0.2433
122	Co	8.6629	3.5025	5.2633	0.6231	0.2519	0.2398
123	Co	12.2018	0.0258	5.3648	0.8776	0.0019	0.2444
124	Co	12.1698	3.5069	5.2032	0.8753	0.2522	0.2370
125	Co	8.7416	7.0014	5.1872	0.6287	0.5036	0.2363
126	Co	8.7597	10.3799	5.2027	0.6300	0.7466	0.2370
127	Co	12.1520	6.9233	5.2132	0.8740	0.4980	0.2375
128	Co	12.2022	10.4240	5.2722	0.8776	0.7497	0.2402
129	O	5.8744	6.1944	6.7749	0.4225	0.4455	0.3086
130	O	2.6354	7.7322	6.8155	0.1895	0.5561	0.3105
131	O	4.7848	7.7684	8.8287	0.3441	0.5587	0.4022
132	O	6.0182	9.7253	6.8289	0.4329	0.6995	0.3111
133	O	7.8054	9.6978	9.0651	0.5614	0.6975	0.4130
134	O	4.9273	11.2937	8.9615	0.3544	0.8123	0.4082
135	O	8.6130	11.7535	6.4059	0.6195	0.8454	0.2918
136	Ce	3.8278	9.7487	8.0621	0.2753	0.7012	0.3673
137	Ce	3.8192	6.0081	7.7403	0.2747	0.4321	0.3526

138	Ce	7.0444	7.9482	8.1331	0.5067	0.5717	0.3705
139	Sm	6.9648	11.5045	8.2342	0.5009	0.8275	0.3751
140	C	7.4686	0.5865	6.8294	0.5372	0.0422	0.3111
141	O	6.1662	13.2184	6.6268	0.4435	0.9507	0.3019
142	O	7.7306	0.5947	8.0136	0.5560	0.0428	0.3651
143	O	10.5049	0.1049	6.2038	0.7556	0.0075	0.2826
144	O	0.0758	0.0739	6.2098	0.0054	0.0053	0.2829
145	O	3.4646	3.7155	6.3563	0.2492	0.2672	0.2896
146	O	6.9712	3.4901	6.2311	0.5014	0.2510	0.2839
147	O	0.0372	10.3629	6.2216	0.0027	0.7454	0.2834

/db/jmorales/CoNi-alloy/Profiles/CoNi(001)/TS

a =			6.951669213				
b =			6.951669213				
c =			21.951669213				
alpha =			90.0				
beta =			90.0				
gamma =			90.0				
	Atom	X	Y	Z	X	Y	Z
1	Ni	0.0000	0.0000	0.0000	0.0000	0.0000	0.0000
2	Ni	0.0000	3.4758	0.0000	0.0000	0.5000	0.0000
3	Ni	3.4758	0.0000	0.0000	0.5000	0.0000	0.0000
4	Ni	3.4758	3.4758	0.0000	0.5000	0.5000	0.0000
5	Ni	0.0000	1.7379	1.7379	0.0000	0.2500	0.0792
6	Ni	0.0000	5.2138	1.7379	0.0000	0.7500	0.0792
7	Ni	3.4758	1.7379	1.7379	0.5000	0.2500	0.0792
8	Ni	3.4758	5.2138	1.7379	0.5000	0.7500	0.0792
9	Ni	0.0023	0.0087	3.4849	0.0003	0.0012	0.1588
10	Ni	6.9431	3.4819	3.5015	0.9988	0.5009	0.1595
11	Ni	3.4738	6.9466	3.4910	0.4997	0.9993	0.1590
12	Ni	3.4744	3.4786	3.4840	0.4998	0.5004	0.1587
13	Ni	0.0167	1.7001	5.2006	0.0024	0.2446	0.2369
14	Ni	6.9440	5.2534	5.1922	0.9989	0.7557	0.2365
15	Ni	3.4758	1.7012	5.2427	0.5000	0.2447	0.2388
16	Ni	3.4477	5.2003	5.2756	0.4960	0.7481	0.2403
17	Co	1.7379	1.7379	0.0000	0.2500	0.2500	0.0000
18	Co	1.7379	5.2138	0.0000	0.2500	0.7500	0.0000
19	Co	5.2138	1.7379	0.0000	0.7500	0.2500	0.0000
20	Co	5.2138	5.2138	0.0000	0.7500	0.7500	0.0000
21	Co	1.7379	0.0000	1.7379	0.2500	0.0000	0.0792
22	Co	1.7379	3.4758	1.7379	0.2500	0.5000	0.0792
23	Co	5.2138	0.0000	1.7379	0.7500	0.0000	0.0792
24	Co	5.2138	3.4758	1.7379	0.7500	0.5000	0.0792
25	Co	1.7351	1.7440	3.5460	0.2496	0.2509	0.1615
26	Co	1.7394	5.2048	3.5374	0.2502	0.7487	0.1611
27	Co	5.2237	1.7463	3.5290	0.7514	0.2512	0.1608
28	Co	5.2140	5.2124	3.5466	0.7500	0.7498	0.1616
29	Co	1.7402	0.0015	5.1904	0.2503	0.0002	0.2364
30	Co	1.6986	3.4975	5.2341	0.2443	0.5031	0.2384
31	Co	5.2177	6.9297	5.1876	0.7506	0.9968	0.2363
32	Co	5.2760	3.4707	5.3042	0.7590	0.4993	0.2416
33	C	2.7454	4.1011	6.7557	0.3949	0.5899	0.3078
34	O	2.4709	4.0952	7.9021	0.3554	0.5891	0.3600
35	O	4.2519	2.7362	6.5384	0.6116	0.3936	0.2979

/db/jmorales/CoNi-alloy/Profiles/Ni-Ce_3SmO_7/TS-3

a =			13.901983737				
b =			13.901983737				
c =			21.950991869				
alpha =			90.0				
beta =			90.0				
gamma =			90.0				
	Atom	X	Y	Z	X	Y	Z
1	Ni	0.0000	0.0000	0.0000	0.0000	0.0000	0.0000
2	Ni	1.7378	1.7378	0.0000	0.1250	0.1250	0.0000
3	Ni	0.0000	3.4755	0.0000	0.0000	0.2500	0.0000
4	Ni	1.7378	5.2132	0.0000	0.1250	0.3750	0.0000
5	Ni	3.4755	0.0000	0.0000	0.2500	0.0000	0.0000
6	Ni	5.2132	1.7378	0.0000	0.3750	0.1250	0.0000
7	Ni	3.4755	3.4755	0.0000	0.2500	0.2500	0.0000
8	Ni	5.2132	5.2132	0.0000	0.3750	0.3750	0.0000
9	Ni	0.0000	6.9510	0.0000	0.0000	0.5000	0.0000
10	Ni	1.7378	8.6887	0.0000	0.1250	0.6250	0.0000
11	Ni	0.0000	10.4265	0.0000	0.0000	0.7500	0.0000
12	Ni	1.7378	12.1642	0.0000	0.1250	0.8750	0.0000
13	Ni	3.4755	6.9510	0.0000	0.2500	0.5000	0.0000
14	Ni	5.2132	8.6887	0.0000	0.3750	0.6250	0.0000
15	Ni	3.4755	10.4265	0.0000	0.2500	0.7500	0.0000
16	Ni	5.2132	12.1642	0.0000	0.3750	0.8750	0.0000
17	Ni	6.9510	0.0000	0.0000	0.5000	0.0000	0.0000
18	Ni	8.6887	1.7378	0.0000	0.6250	0.1250	0.0000
19	Ni	6.9510	3.4755	0.0000	0.5000	0.2500	0.0000
20	Ni	8.6887	5.2132	0.0000	0.6250	0.3750	0.0000
21	Ni	10.4265	0.0000	0.0000	0.7500	0.0000	0.0000
22	Ni	12.1642	1.7378	0.0000	0.8750	0.1250	0.0000
23	Ni	10.4265	3.4755	0.0000	0.7500	0.2500	0.0000
24	Ni	12.1642	5.2132	0.0000	0.8750	0.3750	0.0000
25	Ni	6.9510	6.9510	0.0000	0.5000	0.5000	0.0000
26	Ni	8.6887	8.6887	0.0000	0.6250	0.6250	0.0000
27	Ni	6.9510	10.4265	0.0000	0.5000	0.7500	0.0000
28	Ni	8.6887	12.1642	0.0000	0.6250	0.8750	0.0000
29	Ni	10.4265	6.9510	0.0000	0.7500	0.5000	0.0000
30	Ni	12.1642	8.6887	0.0000	0.8750	0.6250	0.0000
31	Ni	10.4265	10.4265	0.0000	0.7500	0.7500	0.0000
32	Ni	12.1642	12.1642	0.0000	0.8750	0.8750	0.0000
33	Ni	0.0000	1.7378	1.7378	0.0000	0.1250	0.0792
34	Ni	1.7378	0.0000	1.7378	0.1250	0.0000	0.0792
35	Ni	0.0000	5.2132	1.7378	0.0000	0.3750	0.0792
36	Ni	1.7378	3.4755	1.7378	0.1250	0.2500	0.0792
37	Ni	3.4755	1.7378	1.7378	0.2500	0.1250	0.0792
38	Ni	5.2132	0.0000	1.7378	0.3750	0.0000	0.0792
39	Ni	3.4755	5.2132	1.7378	0.2500	0.3750	0.0792
40	Ni	5.2132	3.4755	1.7378	0.3750	0.2500	0.0792

41	Ni	0.0000	8.6887	1.7378	0.0000	0.6250	0.0792
42	Ni	1.7378	6.9510	1.7378	0.1250	0.5000	0.0792
43	Ni	0.0000	12.1642	1.7378	0.0000	0.8750	0.0792
44	Ni	1.7378	10.4265	1.7378	0.1250	0.7500	0.0792
45	Ni	3.4755	8.6887	1.7378	0.2500	0.6250	0.0792
46	Ni	5.2132	6.9510	1.7378	0.3750	0.5000	0.0792
47	Ni	3.4755	12.1642	1.7378	0.2500	0.8750	0.0792
48	Ni	5.2132	10.4265	1.7378	0.3750	0.7500	0.0792
49	Ni	6.9510	1.7378	1.7378	0.5000	0.1250	0.0792
50	Ni	8.6887	0.0000	1.7378	0.6250	0.0000	0.0792
51	Ni	6.9510	5.2132	1.7378	0.5000	0.3750	0.0792
52	Ni	8.6887	3.4755	1.7378	0.6250	0.2500	0.0792
53	Ni	10.4265	1.7378	1.7378	0.7500	0.1250	0.0792
54	Ni	12.1642	0.0000	1.7378	0.8750	0.0000	0.0792
55	Ni	10.4265	5.2132	1.7378	0.7500	0.3750	0.0792
56	Ni	12.1642	3.4755	1.7378	0.8750	0.2500	0.0792
57	Ni	6.9510	8.6887	1.7378	0.5000	0.6250	0.0792
58	Ni	8.6887	6.9510	1.7378	0.6250	0.5000	0.0792
59	Ni	6.9510	12.1642	1.7378	0.5000	0.8750	0.0792
60	Ni	8.6887	10.4265	1.7378	0.6250	0.7500	0.0792
61	Ni	10.4265	8.6887	1.7378	0.7500	0.6250	0.0792
62	Ni	12.1642	6.9510	1.7378	0.8750	0.5000	0.0792
63	Ni	10.4265	12.1642	1.7378	0.7500	0.8750	0.0792
64	Ni	12.1642	10.4265	1.7378	0.8750	0.7500	0.0792
65	Ni	0.0074	0.0049	3.5148	0.0005	0.0004	0.1601
66	Ni	1.7372	1.7329	3.5150	0.1250	0.1247	0.1601
67	Ni	13.8881	3.4686	3.5139	0.9990	0.2495	0.1601
68	Ni	1.7031	5.2029	3.4768	0.1225	0.3743	0.1584
69	Ni	3.4826	0.0027	3.5290	0.2505	0.0002	0.1608
70	Ni	5.2224	1.7568	3.5420	0.3757	0.1264	0.1614
71	Ni	3.4885	3.4372	3.5036	0.2509	0.2472	0.1596
72	Ni	5.1990	5.2077	3.4800	0.3740	0.3746	0.1585
73	Ni	13.8934	6.9528	3.5086	0.9994	0.5001	0.1598
74	Ni	1.7514	8.6990	3.5222	0.1260	0.6257	0.1605
75	Ni	13.8923	10.4382	3.5292	0.9993	0.7508	0.1608
76	Ni	1.7528	12.1701	3.5175	0.1261	0.8754	0.1602
77	Ni	3.4593	6.9769	3.4842	0.2488	0.5019	0.1587
78	Ni	5.2192	8.7096	3.5052	0.3754	0.6265	0.1597
79	Ni	3.4745	10.4122	3.5385	0.2499	0.7490	0.1612
80	Ni	5.2119	12.1753	3.5103	0.3749	0.8758	0.1599
81	Ni	6.9501	0.0027	3.5100	0.4999	0.0002	0.1599
82	Ni	8.7060	1.7523	3.5160	0.6262	0.1260	0.1602
83	Ni	6.9668	3.4735	3.5143	0.5011	0.2499	0.1601
84	Ni	8.6924	5.2334	3.5456	0.6253	0.3765	0.1615
85	Ni	10.4353	13.8786	3.5291	0.7506	0.9983	0.1608
86	Ni	12.1601	1.7427	3.5140	0.8747	0.1254	0.1601
87	Ni	10.4352	3.5143	3.5410	0.7506	0.2528	0.1613
88	Ni	12.1391	5.2219	3.5580	0.8732	0.3756	0.1621
89	Ni	6.9227	6.9468	3.5032	0.4980	0.4997	0.1596

90	Ni	8.6804	8.6837	3.4610	0.6244	0.6246	0.1577
91	Ni	6.9671	10.4310	3.5174	0.5012	0.7503	0.1602
92	Ni	8.6880	12.1847	3.4967	0.6249	0.8765	0.1593
93	Ni	10.4197	6.9352	3.5503	0.7495	0.4989	0.1617
94	Ni	12.1887	8.6867	3.4880	0.8768	0.6249	0.1589
95	Ni	10.4382	10.4498	3.4300	0.7508	0.7517	0.1563
96	Ni	12.1770	12.1711	3.5088	0.8759	0.8755	0.1598
97	Ni	0.0040	1.7392	5.2420	0.0003	0.1251	0.2388
98	Ni	1.7340	0.0025	5.2425	0.1247	0.0002	0.2388
99	Ni	0.0132	5.1952	5.2237	0.0009	0.3737	0.2380
100	Ni	1.7432	3.4736	5.2316	0.1254	0.2499	0.2383
101	Ni	3.4626	1.7349	5.2439	0.2491	0.1248	0.2389
102	Ni	5.2221	0.0128	5.2493	0.3756	0.0009	0.2391
103	Ni	3.4246	5.1831	5.0846	0.2463	0.3728	0.2316
104	Ni	5.1940	3.4339	5.3754	0.3736	0.2470	0.2449
105	Ni	0.0440	8.7089	5.2217	0.0032	0.6265	0.2379
106	Ni	1.7081	6.9220	5.2033	0.1229	0.4979	0.2370
107	Ni	0.0041	12.1786	5.2314	0.0003	0.8760	0.2383
108	Ni	1.7325	10.4600	5.2410	0.1246	0.7524	0.2388
109	Ni	3.4356	8.7496	5.3843	0.2471	0.6294	0.2453
110	Ni	5.1560	6.9755	5.2194	0.3709	0.5018	0.2378
111	Ni	3.4918	12.1675	5.2536	0.2512	0.8752	0.2393
112	Ni	5.2290	10.4516	5.2143	0.3761	0.7518	0.2375
113	Ni	6.9856	1.7140	5.2149	0.5025	0.1233	0.2376
114	Ni	8.6876	13.8949	5.2227	0.6249	0.9995	0.2379
115	Ni	6.8841	5.2185	5.2321	0.4952	0.3754	0.2384
116	Ni	8.7216	3.4665	5.3010	0.6274	0.2494	0.2415
117	Ni	10.4444	1.7069	5.2236	0.7513	0.1228	0.2380
118	Ni	12.1831	0.0097	5.2305	0.8764	0.0007	0.2383
119	Ni	10.4801	5.3681	5.8684	0.7539	0.3861	0.2673
120	Ni	12.1784	3.4717	5.2381	0.8760	0.2497	0.2386
121	Ni	6.9585	8.7541	5.2525	0.5005	0.6297	0.2393
122	Ni	8.6582	6.9796	5.2530	0.6228	0.5021	0.2393
123	Ni	6.9392	12.1689	5.2375	0.4992	0.8753	0.2386
124	Ni	8.7074	10.4713	5.2084	0.6263	0.7532	0.2373
125	Ni	10.4370	8.7162	5.1413	0.7508	0.6270	0.2342
126	Ni	12.1761	6.9934	5.2658	0.8759	0.5030	0.2399
127	Ni	10.4503	12.1864	5.2903	0.7517	0.8766	0.2410
128	Ni	12.1874	10.4251	5.2800	0.8767	0.7499	0.2405
129	O	6.0609	4.4463	6.8124	0.4360	0.3198	0.3103
130	O	4.1587	7.6400	6.7717	0.2991	0.5496	0.3085
131	O	5.9018	6.6059	8.8026	0.4245	0.4752	0.4010
132	O	7.7501	7.8383	6.8129	0.5575	0.5638	0.3104
133	O	10.4076	10.4014	6.2594	0.7486	0.7482	0.2852
134	O	9.9051	6.8746	8.3608	0.7125	0.4945	0.3809
135	O	7.6985	9.6844	8.9554	0.5538	0.6966	0.4080
136	Ce	5.9078	8.8335	8.0462	0.4250	0.6354	0.3666
137	Ce	4.2636	5.5395	7.6943	0.3067	0.3985	0.3505
138	Ce	7.9719	5.6284	7.9516	0.5734	0.4049	0.3622

139 Sm	9.4061	9.0318	7.9069	0.6766	0.6497	0.3602
140 C	11.1412	6.5076	7.2457	0.8014	0.4681	0.3301
141 O	12.1893	6.9271	7.6973	0.8768	0.4983	0.3507
142 O	9.3926	4.1478	6.8407	0.6756	0.2984	0.3116

/db/jmorales/CoNi-alloy/Profiles/Co-CoNi(001)/CO_2

a =			6.951669213				
b =			6.951669213				
c =			21.951669213				
alpha =			90.0				
beta =			90.0				
gamma =			90.0				
	Atom	X	Y	Z	X	Y	Z
1	Ni	0.0000	0.0000	0.0000	0.0000	0.0000	0.0000
2	Ni	1.7379	1.7379	0.0000	0.2500	0.2500	0.0000
3	Ni	0.0000	3.4758	0.0000	0.0000	0.5000	0.0000
4	Ni	1.7379	5.2138	0.0000	0.2500	0.7500	0.0000
5	Ni	3.4758	0.0000	0.0000	0.5000	0.0000	0.0000
6	Ni	5.2138	1.7379	0.0000	0.7500	0.2500	0.0000
7	Ni	3.4758	3.4758	0.0000	0.5000	0.5000	0.0000
8	Ni	5.2138	5.2138	0.0000	0.7500	0.7500	0.0000
9	Ni	6.9507	0.0006	3.5041	0.9999	0.0001	0.1596
10	Ni	1.7464	1.7493	3.5339	0.2512	0.2516	0.1610
11	Ni	6.9394	3.4740	3.5233	0.9982	0.4997	0.1605
12	Ni	1.7375	5.2136	3.5229	0.2499	0.7500	0.1605
13	Ni	3.4778	0.0096	3.5246	0.5003	0.0014	0.1606
14	Ni	5.2106	1.7409	3.5442	0.7495	0.2504	0.1615
15	Ni	3.4782	3.4767	3.5415	0.5003	0.5001	0.1613
16	Ni	5.2037	5.2062	3.5377	0.7485	0.7489	0.1612
17	Co	1.7379	0.0000	1.7379	0.2500	0.0000	0.0792
18	Co	0.0000	1.7379	1.7379	0.0000	0.2500	0.0792
19	Co	1.7379	3.4758	1.7379	0.2500	0.5000	0.0792
20	Co	0.0000	5.2138	1.7379	0.0000	0.7500	0.0792
21	Co	5.2138	0.0000	1.7379	0.7500	0.0000	0.0792
22	Co	3.4758	1.7379	1.7379	0.5000	0.2500	0.0792
23	Co	5.2138	3.4758	1.7379	0.7500	0.5000	0.0792
24	Co	3.4758	5.2138	1.7379	0.5000	0.7500	0.0792
25	Co	1.7079	0.0076	5.2543	0.2457	0.0011	0.2394
26	Co	0.0261	1.7175	5.2601	0.0038	0.2471	0.2396
27	Co	1.7354	3.5110	5.2868	0.2496	0.5051	0.2408
28	Co	6.9428	5.2158	5.2585	0.9987	0.7503	0.2396
29	Co	5.2578	6.9306	5.2602	0.7563	0.9970	0.2396
30	Co	3.4588	1.6680	5.3321	0.4975	0.2399	0.2429
31	Co	5.2633	3.4830	5.3342	0.7571	0.5010	0.2430
32	Co	3.4439	5.2528	5.2868	0.4954	0.7556	0.2408
33	C	3.0550	3.8254	6.6388	0.4395	0.5503	0.3024
34	O	2.9652	4.8033	7.4028	0.4265	0.6910	0.3372
35	O	3.9505	2.8514	6.7822	0.5683	0.4102	0.3090

/db/jmorales/CoNi-alloy/Profiles/Ni-Ce_3SmO_7/O

a =				13.9019837372			
b =				13.9019837372			
c =				21.9509918687			
alpha =				90.0			
beta =				90.0			
gamma =				90.0			
	Atom	X	Y	Z	X	Y	Z
1	Ni	0.0000	0.0000	0.0000	0.0000	0.0000	0.0000
2	Ni	1.7377	1.7377	0.0000	0.1250	0.1250	0.0000
3	Ni	0.0000	3.4755	0.0000	0.0000	0.2500	0.0000
4	Ni	1.7377	5.2132	0.0000	0.1250	0.3750	0.0000
5	Ni	3.4755	0.0000	0.0000	0.2500	0.0000	0.0000
6	Ni	5.2132	1.7377	0.0000	0.3750	0.1250	0.0000
7	Ni	3.4755	3.4755	0.0000	0.2500	0.2500	0.0000
8	Ni	5.2132	5.2132	0.0000	0.3750	0.3750	0.0000
9	Ni	0.0000	6.9510	0.0000	0.0000	0.5000	0.0000
10	Ni	1.7377	8.6887	0.0000	0.1250	0.6250	0.0000
11	Ni	0.0000	10.4265	0.0000	0.0000	0.7500	0.0000
12	Ni	1.7377	12.1642	0.0000	0.1250	0.8750	0.0000
13	Ni	3.4755	6.9510	0.0000	0.2500	0.5000	0.0000
14	Ni	5.2132	8.6887	0.0000	0.3750	0.6250	0.0000
15	Ni	3.4755	10.4265	0.0000	0.2500	0.7500	0.0000
16	Ni	5.2132	12.1642	0.0000	0.3750	0.8750	0.0000
17	Ni	6.9510	0.0000	0.0000	0.5000	0.0000	0.0000
18	Ni	8.6887	1.7377	0.0000	0.6250	0.1250	0.0000
19	Ni	6.9510	3.4755	0.0000	0.5000	0.2500	0.0000
20	Ni	8.6887	5.2132	0.0000	0.6250	0.3750	0.0000
21	Ni	10.4265	0.0000	0.0000	0.7500	0.0000	0.0000
22	Ni	12.1642	1.7377	0.0000	0.8750	0.1250	0.0000
23	Ni	10.4265	3.4755	0.0000	0.7500	0.2500	0.0000
24	Ni	12.1642	5.2132	0.0000	0.8750	0.3750	0.0000
25	Ni	6.9510	6.9510	0.0000	0.5000	0.5000	0.0000
26	Ni	8.6887	8.6887	0.0000	0.6250	0.6250	0.0000
27	Ni	6.9510	10.4265	0.0000	0.5000	0.7500	0.0000
28	Ni	8.6887	12.1642	0.0000	0.6250	0.8750	0.0000
29	Ni	10.4265	6.9510	0.0000	0.7500	0.5000	0.0000
30	Ni	12.1642	8.6887	0.0000	0.8750	0.6250	0.0000
31	Ni	10.4265	10.4265	0.0000	0.7500	0.7500	0.0000
32	Ni	12.1642	12.1642	0.0000	0.8750	0.8750	0.0000
33	Ni	0.0000	1.7377	1.7377	0.0000	0.1250	0.0792
34	Ni	1.7377	0.0000	1.7377	0.1250	0.0000	0.0792
35	Ni	0.0000	5.2132	1.7377	0.0000	0.3750	0.0792
36	Ni	1.7377	3.4755	1.7377	0.1250	0.2500	0.0792
37	Ni	3.4755	1.7377	1.7377	0.2500	0.1250	0.0792
38	Ni	5.2132	0.0000	1.7377	0.3750	0.0000	0.0792
39	Ni	3.4755	5.2132	1.7377	0.2500	0.3750	0.0792
40	Ni	5.2132	3.4755	1.7377	0.3750	0.2500	0.0792

41	Ni	0.0000	8.6887	1.7377	0.0000	0.6250	0.0792
42	Ni	1.7377	6.9510	1.7377	0.1250	0.5000	0.0792
43	Ni	0.0000	12.1642	1.7377	0.0000	0.8750	0.0792
44	Ni	1.7377	10.4265	1.7377	0.1250	0.7500	0.0792
45	Ni	3.4755	8.6887	1.7377	0.2500	0.6250	0.0792
46	Ni	5.2132	6.9510	1.7377	0.3750	0.5000	0.0792
47	Ni	3.4755	12.1642	1.7377	0.2500	0.8750	0.0792
48	Ni	5.2132	10.4265	1.7377	0.3750	0.7500	0.0792
49	Ni	6.9510	1.7377	1.7377	0.5000	0.1250	0.0792
50	Ni	8.6887	0.0000	1.7377	0.6250	0.0000	0.0792
51	Ni	6.9510	5.2132	1.7377	0.5000	0.3750	0.0792
52	Ni	8.6887	3.4755	1.7377	0.6250	0.2500	0.0792
53	Ni	10.4265	1.7377	1.7377	0.7500	0.1250	0.0792
54	Ni	12.1642	0.0000	1.7377	0.8750	0.0000	0.0792
55	Ni	10.4265	5.2132	1.7377	0.7500	0.3750	0.0792
56	Ni	12.1642	3.4755	1.7377	0.8750	0.2500	0.0792
57	Ni	6.9510	8.6887	1.7377	0.5000	0.6250	0.0792
58	Ni	8.6887	6.9510	1.7377	0.6250	0.5000	0.0792
59	Ni	6.9510	12.1642	1.7377	0.5000	0.8750	0.0792
60	Ni	8.6887	10.4265	1.7377	0.6250	0.7500	0.0792
61	Ni	10.4265	8.6887	1.7377	0.7500	0.6250	0.0792
62	Ni	12.1642	6.9510	1.7377	0.8750	0.5000	0.0792
63	Ni	10.4265	12.1642	1.7377	0.7500	0.8750	0.0792
64	Ni	12.1642	10.4265	1.7377	0.8750	0.7500	0.0792
65	Ni	0.0051	0.0016	3.5060	0.0004	0.0001	0.1597
66	Ni	1.7225	1.7346	3.5195	0.1239	0.1248	0.1603
67	Ni	13.8962	3.4674	3.5063	0.9996	0.2494	0.1597
68	Ni	1.7095	5.2048	3.4707	0.1230	0.3744	0.1581
69	Ni	3.4792	0.0039	3.5124	0.2503	0.0003	0.1600
70	Ni	5.2214	1.7501	3.5348	0.3756	0.1259	0.1610
71	Ni	3.4937	3.4401	3.4989	0.2513	0.2475	0.1594
72	Ni	5.2138	5.2087	3.4940	0.3750	0.3747	0.1592
73	Ni	13.8979	6.9496	3.5013	0.9997	0.4999	0.1595
74	Ni	1.7436	8.6933	3.5220	0.1254	0.6253	0.1604
75	Ni	13.8845	10.4294	3.5246	0.9987	0.7502	0.1606
76	Ni	1.7448	12.1598	3.5070	0.1255	0.8747	0.1598
77	Ni	3.4632	6.9778	3.4840	0.2491	0.5019	0.1587
78	Ni	5.2206	8.7029	3.5030	0.3755	0.6260	0.1596
79	Ni	3.4714	10.4020	3.5316	0.2497	0.7482	0.1609
80	Ni	5.2108	12.1667	3.5030	0.3748	0.8752	0.1596
81	Ni	6.9474	0.0008	3.5054	0.4997	0.0001	0.1597
82	Ni	8.7133	1.7285	3.5147	0.6268	0.1243	0.1601
83	Ni	6.9468	3.4657	3.5064	0.4997	0.2493	0.1597
84	Ni	8.6645	5.2343	3.5221	0.6233	0.3765	0.1605
85	Ni	10.4321	13.8790	3.5232	0.7504	0.9983	0.1605
86	Ni	12.1601	1.7352	3.4405	0.8747	0.1248	0.1567
87	Ni	10.4267	3.4801	3.5013	0.7500	0.2503	0.1595
88	Ni	12.1627	5.1985	3.5201	0.8749	0.3739	0.1604
89	Ni	6.9285	6.9418	3.5148	0.4984	0.4993	0.1601

90	Ni	8.6927	8.6837	3.4741	0.6253	0.6246	0.1583
91	Ni	6.9746	10.4232	3.5146	0.5017	0.7498	0.1601
92	Ni	8.6908	12.1727	3.4958	0.6251	0.8756	0.1593
93	Ni	10.4183	6.9560	3.5179	0.7494	0.5004	0.1603
94	Ni	12.1839	8.6856	3.4890	0.8764	0.6248	0.1589
95	Ni	10.4423	10.4413	3.4338	0.7511	0.7511	0.1564
96	Ni	12.1710	12.1806	3.5175	0.8755	0.8762	0.1602
97	Ni	0.0081	1.7370	5.2928	0.0006	0.1249	0.2411
98	Ni	1.7428	13.8942	5.2208	0.1254	0.9994	0.2378
99	Ni	0.0013	5.2056	5.2184	0.0001	0.3745	0.2377
100	Ni	1.7428	3.4795	5.2135	0.1254	0.2503	0.2375
101	Ni	3.4623	1.7337	5.2309	0.2491	0.1247	0.2383
102	Ni	5.2130	0.0006	5.2286	0.3750	0.0000	0.2382
103	Ni	3.4322	5.1856	5.0829	0.2469	0.3730	0.2316
104	Ni	5.1986	3.4361	5.3771	0.3739	0.2472	0.2450
105	Ni	0.0069	8.6784	5.2217	0.0005	0.6243	0.2379
106	Ni	1.7144	6.9160	5.1992	0.1233	0.4975	0.2369
107	Ni	0.0018	12.1733	5.2177	0.0001	0.8757	0.2377
108	Ni	1.7189	10.4430	5.2288	0.1236	0.7512	0.2382
109	Ni	3.4306	8.7401	5.3873	0.2468	0.6287	0.2454
110	Ni	5.1654	6.9738	5.2240	0.3716	0.5016	0.2380
111	Ni	3.4830	12.1526	5.2285	0.2505	0.8742	0.2382
112	Ni	5.2331	10.4422	5.1996	0.3764	0.7511	0.2369
113	Ni	6.9878	1.7114	5.2124	0.5027	0.1231	0.2375
114	Ni	8.6901	13.8881	5.2171	0.6251	0.9990	0.2377
115	Ni	6.9273	5.1940	5.2650	0.4983	0.3736	0.2399
116	Ni	8.7093	3.4804	5.1960	0.6265	0.2504	0.2367
117	Ni	10.4188	1.7357	5.2942	0.7494	0.1249	0.2412
118	Ni	12.1663	0.0082	5.2829	0.8752	0.0006	0.2407
119	Ni	10.4169	5.2136	5.2184	0.7493	0.3750	0.2377
120	Ni	12.1585	3.4769	5.2879	0.8746	0.2501	0.2409
121	Ni	6.9722	8.7315	5.2381	0.5015	0.6281	0.2386
122	Ni	8.7084	6.9419	5.2793	0.6264	0.4993	0.2405
123	Ni	6.9474	12.1598	5.2209	0.4997	0.8747	0.2378
124	Ni	8.7168	10.4573	5.2188	0.6270	0.7522	0.2377
125	Ni	10.4651	8.6956	5.1755	0.7528	0.6255	0.2358
126	Ni	12.1644	6.9422	5.2158	0.8750	0.4994	0.2376
127	Ni	10.4448	12.1625	5.2787	0.7513	0.8749	0.2405
128	Ni	12.1839	10.4289	5.2841	0.8764	0.7502	0.2407
129	O	6.0801	4.4048	6.8251	0.4374	0.3169	0.3109
130	O	4.1801	7.6529	6.7815	0.3007	0.5505	0.3089
131	O	5.9698	6.6157	8.7823	0.4294	0.4759	0.4001
132	O	7.8187	7.8477	6.8051	0.5624	0.5645	0.3100
133	O	10.4213	10.3553	6.2777	0.7496	0.7449	0.2860
134	O	9.5582	6.9457	8.8547	0.6875	0.4996	0.4034
135	O	7.7132	9.7548	8.9195	0.5548	0.7017	0.4063
136	Ce	5.9644	8.8648	8.0271	0.4290	0.6377	0.3657
137	Ce	4.3160	5.5422	7.6896	0.3105	0.3987	0.3503
138	Ce	8.0229	5.7234	8.0292	0.5771	0.4117	0.3658

139 Sm	9.4623	8.9420	8.0402	0.6806	0.6432	0.3663
140 O	12.1694	1.7417	6.1542	0.8754	0.1253	0.2804

/db/jmorales/CoNi-alloy/Profiles/Ni-Ce_3SmO_7/O-coverage/M-CO2-O-coverage/M-CO_2-Ni-Ce_3SmO_7-O

a =	13.9019837372
b =	13.9019837372
c =	21.9509918687
alpha =	90.0
beta =	90.0
gamma =	90.0

	Atom	X	Y	Z	X	Y	Z
1	Ni	0.0000	0.0000	0.0000	0.0000	0.0000	0.0000
2	Ni	1.7377	1.7377	0.0000	0.1250	0.1250	0.0000
3	Ni	0.0000	3.4755	0.0000	0.0000	0.2500	0.0000
4	Ni	1.7377	5.2132	0.0000	0.1250	0.3750	0.0000
5	Ni	3.4755	0.0000	0.0000	0.2500	0.0000	0.0000
6	Ni	5.2132	1.7377	0.0000	0.3750	0.1250	0.0000
7	Ni	3.4755	3.4755	0.0000	0.2500	0.2500	0.0000
8	Ni	5.2132	5.2132	0.0000	0.3750	0.3750	0.0000
9	Ni	0.0000	6.9510	0.0000	0.0000	0.5000	0.0000
10	Ni	1.7377	8.6887	0.0000	0.1250	0.6250	0.0000
11	Ni	0.0000	10.4265	0.0000	0.0000	0.7500	0.0000
12	Ni	1.7377	12.1642	0.0000	0.1250	0.8750	0.0000
13	Ni	3.4755	6.9510	0.0000	0.2500	0.5000	0.0000
14	Ni	5.2132	8.6887	0.0000	0.3750	0.6250	0.0000
15	Ni	3.4755	10.4265	0.0000	0.2500	0.7500	0.0000
16	Ni	5.2132	12.1642	0.0000	0.3750	0.8750	0.0000
17	Ni	6.9510	0.0000	0.0000	0.5000	0.0000	0.0000
18	Ni	8.6887	1.7377	0.0000	0.6250	0.1250	0.0000
19	Ni	6.9510	3.4755	0.0000	0.5000	0.2500	0.0000
20	Ni	8.6887	5.2132	0.0000	0.6250	0.3750	0.0000
21	Ni	10.4265	0.0000	0.0000	0.7500	0.0000	0.0000
22	Ni	12.1642	1.7377	0.0000	0.8750	0.1250	0.0000
23	Ni	10.4265	3.4755	0.0000	0.7500	0.2500	0.0000
24	Ni	12.1642	5.2132	0.0000	0.8750	0.3750	0.0000
25	Ni	6.9510	6.9510	0.0000	0.5000	0.5000	0.0000
26	Ni	8.6887	8.6887	0.0000	0.6250	0.6250	0.0000
27	Ni	6.9510	10.4265	0.0000	0.5000	0.7500	0.0000
28	Ni	8.6887	12.1642	0.0000	0.6250	0.8750	0.0000
29	Ni	10.4265	6.9510	0.0000	0.7500	0.5000	0.0000
30	Ni	12.1642	8.6887	0.0000	0.8750	0.6250	0.0000
31	Ni	10.4265	10.4265	0.0000	0.7500	0.7500	0.0000
32	Ni	12.1642	12.1642	0.0000	0.8750	0.8750	0.0000
33	Ni	0.0000	1.7377	1.7377	0.0000	0.1250	0.0792
34	Ni	1.7377	0.0000	1.7377	0.1250	0.0000	0.0792
35	Ni	0.0000	5.2132	1.7377	0.0000	0.3750	0.0792
36	Ni	1.7377	3.4755	1.7377	0.1250	0.2500	0.0792
37	Ni	3.4755	1.7377	1.7377	0.2500	0.1250	0.0792
38	Ni	5.2132	0.0000	1.7377	0.3750	0.0000	0.0792
39	Ni	3.4755	5.2132	1.7377	0.2500	0.3750	0.0792

40	Ni	5.2132	3.4755	1.7377	0.3750	0.2500	0.0792
41	Ni	0.0000	8.6887	1.7377	0.0000	0.6250	0.0792
42	Ni	1.7377	6.9510	1.7377	0.1250	0.5000	0.0792
43	Ni	0.0000	12.1642	1.7377	0.0000	0.8750	0.0792
44	Ni	1.7377	10.4265	1.7377	0.1250	0.7500	0.0792
45	Ni	3.4755	8.6887	1.7377	0.2500	0.6250	0.0792
46	Ni	5.2132	6.9510	1.7377	0.3750	0.5000	0.0792
47	Ni	3.4755	12.1642	1.7377	0.2500	0.8750	0.0792
48	Ni	5.2132	10.4265	1.7377	0.3750	0.7500	0.0792
49	Ni	6.9510	1.7377	1.7377	0.5000	0.1250	0.0792
50	Ni	8.6887	0.0000	1.7377	0.6250	0.0000	0.0792
51	Ni	6.9510	5.2132	1.7377	0.5000	0.3750	0.0792
52	Ni	8.6887	3.4755	1.7377	0.6250	0.2500	0.0792
53	Ni	10.4265	1.7377	1.7377	0.7500	0.1250	0.0792
54	Ni	12.1642	0.0000	1.7377	0.8750	0.0000	0.0792
55	Ni	10.4265	5.2132	1.7377	0.7500	0.3750	0.0792
56	Ni	12.1642	3.4755	1.7377	0.8750	0.2500	0.0792
57	Ni	6.9510	8.6887	1.7377	0.5000	0.6250	0.0792
58	Ni	8.6887	6.9510	1.7377	0.6250	0.5000	0.0792
59	Ni	6.9510	12.1642	1.7377	0.5000	0.8750	0.0792
60	Ni	8.6887	10.4265	1.7377	0.6250	0.7500	0.0792
61	Ni	10.4265	8.6887	1.7377	0.7500	0.6250	0.0792
62	Ni	12.1642	6.9510	1.7377	0.8750	0.5000	0.0792
63	Ni	10.4265	12.1642	1.7377	0.7500	0.8750	0.0792
64	Ni	12.1642	10.4265	1.7377	0.8750	0.7500	0.0792
65	Ni	0.0091	0.0177	3.5104	0.0007	0.0013	0.1599
66	Ni	1.7276	1.7774	3.5411	0.1243	0.1279	0.1613
67	Ni	13.8761	3.4800	3.5263	0.9981	0.2503	0.1606
68	Ni	1.6833	5.1983	3.4495	0.1211	0.3739	0.1571
69	Ni	3.4687	0.0078	3.5095	0.2495	0.0006	0.1599
70	Ni	5.2222	1.7417	3.5228	0.3756	0.1253	0.1605
71	Ni	3.4991	3.4425	3.4809	0.2517	0.2476	0.1586
72	Ni	5.2318	5.2045	3.4806	0.3763	0.3744	0.1586
73	Ni	13.8982	6.9350	3.5179	0.9997	0.4989	0.1603
74	Ni	1.7479	8.6567	3.5428	0.1257	0.6227	0.1614
75	Ni	13.8925	10.4199	3.5237	0.9993	0.7495	0.1605
76	Ni	1.7406	12.1637	3.5069	0.1252	0.8750	0.1598
77	Ni	3.4755	6.9726	3.4664	0.2500	0.5016	0.1579
78	Ni	5.2274	8.7084	3.5034	0.3760	0.6264	0.1596
79	Ni	3.4653	10.3975	3.5279	0.2493	0.7479	0.1607
80	Ni	5.2021	12.1664	3.5027	0.3742	0.8752	0.1596
81	Ni	6.9417	13.8889	3.5012	0.4993	0.9991	0.1595
82	Ni	8.6920	1.7083	3.4914	0.6252	0.1229	0.1591
83	Ni	6.9513	3.4614	3.4992	0.5000	0.2490	0.1594
84	Ni	8.6529	5.2381	3.5089	0.6224	0.3768	0.1599
85	Ni	10.4390	13.8739	3.5206	0.7509	0.9980	0.1604
86	Ni	12.1591	1.7442	3.5141	0.8746	0.1255	0.1601
87	Ni	10.4163	3.4677	3.4875	0.7493	0.2494	0.1589
88	Ni	12.1601	5.2153	3.5091	0.8747	0.3751	0.1599

89	Ni	6.9354	6.9480	3.5120	0.4989	0.4998	0.1600
90	Ni	8.6878	8.6987	3.4702	0.6249	0.6257	0.1581
91	Ni	6.9694	10.4347	3.5113	0.5013	0.7506	0.1600
92	Ni	8.6933	12.1777	3.4908	0.6253	0.8760	0.1590
93	Ni	10.4066	6.9692	3.5118	0.7486	0.5013	0.1600
94	Ni	12.1844	8.6608	3.5098	0.8765	0.6230	0.1599
95	Ni	10.4496	10.4433	3.4324	0.7517	0.7512	0.1564
96	Ni	12.1798	12.1680	3.5063	0.8761	0.8753	0.1597
97	Ni	13.8947	1.7742	5.2355	0.9995	0.1276	0.2385
98	Ni	1.7338	0.0117	5.2237	0.1247	0.0008	0.2380
99	Ni	13.8334	5.1671	5.3480	0.9951	0.3717	0.2436
100	Ni	1.7500	3.5304	5.3230	0.1259	0.2540	0.2425
101	Ni	3.4759	1.7389	5.2225	0.2500	0.1251	0.2379
102	Ni	5.2047	13.9015	5.2241	0.3744	1.0000	0.2380
103	Ni	3.5049	5.1791	5.0579	0.2521	0.3725	0.2304
104	Ni	5.2412	3.4248	5.3542	0.3770	0.2464	0.2439
105	Ni	0.0319	8.6755	5.2163	0.0023	0.6240	0.2376
106	Ni	1.7247	6.8579	5.2795	0.1241	0.4933	0.2405
107	Ni	0.0082	12.1736	5.2173	0.0006	0.8757	0.2377
108	Ni	1.7292	10.4310	5.2234	0.1244	0.7503	0.2380
109	Ni	3.4539	8.7185	5.3723	0.2484	0.6271	0.2447
110	Ni	5.2039	6.9681	5.2115	0.3743	0.5012	0.2374
111	Ni	3.4707	12.1607	5.2241	0.2497	0.8747	0.2380
112	Ni	5.2179	10.4452	5.1988	0.3753	0.7513	0.2368
113	Ni	6.9793	1.6870	5.2049	0.5020	0.1214	0.2371
114	Ni	8.6883	13.8791	5.2199	0.6250	0.9984	0.2378
115	Ni	6.9539	5.1961	5.2820	0.5002	0.3738	0.2406
116	Ni	8.6996	3.4412	5.1675	0.6258	0.2475	0.2354
117	Ni	10.4088	1.6976	5.2150	0.7487	0.1221	0.2376
118	Ni	12.1866	0.0190	5.2211	0.8766	0.0014	0.2379
119	Ni	10.3655	5.2138	5.2087	0.7456	0.3750	0.2373
120	Ni	12.0500	3.4806	5.3023	0.8668	0.2504	0.2415
121	Ni	6.9701	8.7549	5.2456	0.5014	0.6298	0.2390
122	Ni	8.6927	6.9704	5.2760	0.6253	0.5014	0.2404
123	Ni	6.9389	12.1629	5.2250	0.4991	0.8749	0.2380
124	Ni	8.7181	10.4781	5.2011	0.6271	0.7537	0.2369
125	Ni	10.4464	8.7189	5.1786	0.7514	0.6272	0.2359
126	Ni	12.1314	6.9924	5.3124	0.8726	0.5030	0.2420
127	Ni	10.4613	12.1769	5.2861	0.7525	0.8759	0.2408
128	Ni	12.1952	10.4198	5.2843	0.8772	0.7495	0.2407
129	O	6.1181	4.3710	6.7943	0.4401	0.3144	0.3095
130	O	4.1842	7.5887	6.7547	0.3010	0.5459	0.3077
131	O	6.0462	6.5911	8.7715	0.4349	0.4741	0.3996
132	O	7.8553	7.9250	6.8056	0.5651	0.5701	0.3100
133	O	10.4309	10.4064	6.2668	0.7503	0.7486	0.2855
134	O	9.4632	7.2628	9.0199	0.6807	0.5224	0.4109
135	O	7.5535	9.9557	8.8488	0.5433	0.7161	0.4031
136	Ce	5.9122	8.8096	8.0361	0.4253	0.6337	0.3661
137	Ce	4.3666	5.4819	7.7258	0.3141	0.3943	0.3520

138 Ce	8.2428	5.8170	8.0912	0.5929	0.4184	0.3686
139 Sm	9.3619	9.1893	8.0847	0.6734	0.6610	0.3683
140 C	11.4562	4.9240	6.7649	0.8241	0.3542	0.3082
141 O	12.5714	5.7003	6.8126	0.9043	0.4100	0.3104
142 O	10.7830	4.6507	7.7891	0.7756	0.3345	0.3548
143 O	2.1000	5.2047	6.3473	0.1511	0.3744	0.2892

/db/jmorales/CoNi-alloy/Profiles/CoNi-Ce_3SmO_7/O-CO-O

a =		13.9033384264					
b =		13.9033384264					
c =		21.9516692133					
alpha =		90.0					
beta =		90.0					
gamma =		90.0					
	Atom	X	Y	Z	X	Y	Z
1	Ni	0.0000	0.0000	0.0000	0.0000	0.0000	0.0000
2	Ni	0.0000	3.4758	0.0000	0.0000	0.2500	0.0000
3	Ni	3.4758	0.0000	0.0000	0.2500	0.0000	0.0000
4	Ni	3.4758	3.4758	0.0000	0.2500	0.2500	0.0000
5	Ni	0.0000	6.9517	0.0000	0.0000	0.5000	0.0000
6	Ni	0.0000	10.4275	0.0000	0.0000	0.7500	0.0000
7	Ni	3.4758	6.9517	0.0000	0.2500	0.5000	0.0000
8	Ni	3.4758	10.4275	0.0000	0.2500	0.7500	0.0000
9	Ni	6.9517	0.0000	0.0000	0.5000	0.0000	0.0000
10	Ni	6.9517	3.4758	0.0000	0.5000	0.2500	0.0000
11	Ni	10.4275	0.0000	0.0000	0.7500	0.0000	0.0000
12	Ni	10.4275	3.4758	0.0000	0.7500	0.2500	0.0000
13	Ni	6.9517	6.9517	0.0000	0.5000	0.5000	0.0000
14	Ni	6.9517	10.4275	0.0000	0.5000	0.7500	0.0000
15	Ni	10.4275	6.9517	0.0000	0.7500	0.5000	0.0000
16	Ni	10.4275	10.4275	0.0000	0.7500	0.7500	0.0000
17	Ni	0.0000	1.7379	1.7379	0.0000	0.1250	0.0792
18	Ni	0.0000	5.2138	1.7379	0.0000	0.3750	0.0792
19	Ni	3.4758	1.7379	1.7379	0.2500	0.1250	0.0792
20	Ni	3.4758	5.2138	1.7379	0.2500	0.3750	0.0792
21	Ni	0.0000	8.6896	1.7379	0.0000	0.6250	0.0792
22	Ni	0.0000	12.1654	1.7379	0.0000	0.8750	0.0792
23	Ni	3.4758	8.6896	1.7379	0.2500	0.6250	0.0792
24	Ni	3.4758	12.1654	1.7379	0.2500	0.8750	0.0792
25	Ni	6.9517	1.7379	1.7379	0.5000	0.1250	0.0792
26	Ni	6.9517	5.2138	1.7379	0.5000	0.3750	0.0792
27	Ni	10.4275	1.7379	1.7379	0.7500	0.1250	0.0792
28	Ni	10.4275	5.2138	1.7379	0.7500	0.3750	0.0792
29	Ni	6.9517	8.6896	1.7379	0.5000	0.6250	0.0792
30	Ni	6.9517	12.1654	1.7379	0.5000	0.8750	0.0792
31	Ni	10.4275	8.6896	1.7379	0.7500	0.6250	0.0792
32	Ni	10.4275	12.1654	1.7379	0.7500	0.8750	0.0792
33	Ni	13.8981	13.9006	3.5026	0.9996	0.9998	0.1596
34	Ni	-0.0035	3.4807	3.4969	-0.0003	0.2503	0.1593
35	Ni	3.4885	13.8857	3.5439	0.2509	0.9987	0.1614
36	Ni	3.4671	3.4575	3.4636	0.2494	0.2487	0.1578
37	Ni	0.0004	6.9552	3.5101	0.0000	0.5003	0.1599
38	Ni	-0.0009	10.4413	3.4966	-0.0001	0.7510	0.1593
39	Ni	3.4846	6.9449	3.4890	0.2506	0.4995	0.1589
40	Ni	3.5005	10.4136	3.5590	0.2518	0.7490	0.1621

41	Ni	6.9394	0.0132	3.4958	0.4991	0.0009	0.1593
42	Ni	6.9556	3.4973	3.5205	0.5003	0.2515	0.1604
43	Ni	10.4208	0.0006	3.5057	0.7495	0.0000	0.1597
44	Ni	10.4272	3.4780	3.5019	0.7500	0.2502	0.1595
45	Ni	6.9739	6.9326	3.4611	0.5016	0.4986	0.1577
46	Ni	6.9482	10.4420	3.4632	0.4997	0.7510	0.1578
47	Ni	10.4372	6.9425	3.4923	0.7507	0.4993	0.1591
48	Ni	10.4291	10.4433	3.4989	0.7501	0.7511	0.1594
49	Ni	13.8909	1.7583	5.1996	0.9991	0.1265	0.2369
50	Ni	13.8995	5.2084	5.2162	0.9997	0.3746	0.2376
51	Ni	3.4487	1.7687	5.1916	0.2480	0.1272	0.2365
52	Ni	3.4674	5.1758	5.0975	0.2494	0.3723	0.2322
53	Ni	13.8680	8.7166	5.1782	0.9975	0.6269	0.2359
54	Ni	13.8764	12.1835	5.2160	0.9981	0.8763	0.2376
55	Ni	3.4803	8.6240	5.2304	0.2503	0.6203	0.2383
56	Ni	3.4303	12.2868	5.7432	0.2467	0.8837	0.2616
57	Ni	6.9773	1.7672	5.1923	0.5018	0.1271	0.2365
58	Ni	6.9538	5.1522	5.3962	0.5002	0.3706	0.2458
59	Ni	10.4201	1.7337	5.2015	0.7495	0.1247	0.2370
60	Ni	10.4290	5.2176	5.2138	0.7501	0.3753	0.2375
61	Ni	7.0354	8.6844	5.0767	0.5060	0.6246	0.2313
62	Ni	6.9714	12.1696	5.0985	0.5014	0.8753	0.2323
63	Ni	10.4177	8.6970	5.2026	0.7493	0.6255	0.2370
64	Ni	10.4323	12.1779	5.2669	0.7503	0.8759	0.2399
65	Co	1.7379	1.7379	0.0000	0.1250	0.1250	0.0000
66	Co	1.7379	5.2138	0.0000	0.1250	0.3750	0.0000
67	Co	5.2138	1.7379	0.0000	0.3750	0.1250	0.0000
68	Co	5.2138	5.2138	0.0000	0.3750	0.3750	0.0000
69	Co	1.7379	8.6896	0.0000	0.1250	0.6250	0.0000
70	Co	1.7379	12.1654	0.0000	0.1250	0.8750	0.0000
71	Co	5.2138	8.6896	0.0000	0.3750	0.6250	0.0000
72	Co	5.2138	12.1654	0.0000	0.3750	0.8750	0.0000
73	Co	8.6896	1.7379	0.0000	0.6250	0.1250	0.0000
74	Co	8.6896	5.2138	0.0000	0.6250	0.3750	0.0000
75	Co	12.1654	1.7379	0.0000	0.8750	0.1250	0.0000
76	Co	12.1654	5.2138	0.0000	0.8750	0.3750	0.0000
77	Co	8.6896	8.6896	0.0000	0.6250	0.6250	0.0000
78	Co	8.6896	12.1654	0.0000	0.6250	0.8750	0.0000
79	Co	12.1654	8.6896	0.0000	0.8750	0.6250	0.0000
80	Co	12.1654	12.1654	0.0000	0.8750	0.8750	0.0000
81	Co	1.7379	0.0000	1.7379	0.1250	0.0000	0.0792
82	Co	1.7379	3.4758	1.7379	0.1250	0.2500	0.0792
83	Co	5.2138	0.0000	1.7379	0.3750	0.0000	0.0792
84	Co	5.2138	3.4758	1.7379	0.3750	0.2500	0.0792
85	Co	1.7379	6.9517	1.7379	0.1250	0.5000	0.0792
86	Co	1.7379	10.4275	1.7379	0.1250	0.7500	0.0792
87	Co	5.2138	6.9517	1.7379	0.3750	0.5000	0.0792
88	Co	5.2138	10.4275	1.7379	0.3750	0.7500	0.0792
89	Co	8.6896	0.0000	1.7379	0.6250	0.0000	0.0792

90	Co	8.6896	3.4758	1.7379	0.6250	0.2500	0.0792
91	Co	12.1654	0.0000	1.7379	0.8750	0.0000	0.0792
92	Co	12.1654	3.4758	1.7379	0.8750	0.2500	0.0792
93	Co	8.6896	6.9517	1.7379	0.6250	0.5000	0.0792
94	Co	8.6896	10.4275	1.7379	0.6250	0.7500	0.0792
95	Co	12.1654	6.9517	1.7379	0.8750	0.5000	0.0792
96	Co	12.1654	10.4275	1.7379	0.8750	0.7500	0.0792
97	Co	1.7291	1.7398	3.5336	0.1244	0.1251	0.1610
98	Co	1.7082	5.2375	3.5319	0.1229	0.3767	0.1609
99	Co	5.2155	1.7285	3.5529	0.3751	0.1243	0.1619
100	Co	5.2477	5.1983	3.5317	0.3774	0.3739	0.1609
101	Co	1.7335	8.6768	3.5308	0.1247	0.6241	0.1608
102	Co	1.7766	12.1930	3.5665	0.1278	0.8770	0.1625
103	Co	5.2027	8.6836	3.5505	0.3742	0.6246	0.1617
104	Co	5.1462	12.1535	3.6378	0.3701	0.8741	0.1657
105	Co	8.6876	1.7301	3.5396	0.6249	0.1244	0.1612
106	Co	8.6695	5.2060	3.5795	0.6236	0.3744	0.1631
107	Co	12.1597	1.7448	3.5425	0.8746	0.1255	0.1614
108	Co	12.1661	5.2114	3.5616	0.8750	0.3748	0.1622
109	Co	8.7257	8.7050	3.4975	0.6276	0.6261	0.1593
110	Co	8.7153	12.1704	3.4544	0.6268	0.8754	0.1574
111	Co	12.1569	8.6940	3.5394	0.8744	0.6253	0.1612
112	Co	12.1523	12.1758	3.5822	0.8741	0.8757	0.1632
113	Co	1.6993	0.0691	5.2109	0.1222	0.0050	0.2374
114	Co	1.7407	3.4914	5.2152	0.1252	0.2511	0.2376
115	Co	5.2311	0.0351	5.2806	0.3763	0.0025	0.2406
116	Co	5.1756	3.4432	5.1975	0.3723	0.2477	0.2368
117	Co	1.6917	6.9513	5.3137	0.1217	0.5000	0.2421
118	Co	1.7160	10.4481	5.1781	0.1234	0.7515	0.2359
119	Co	5.2966	6.9257	5.1854	0.3810	0.4981	0.2362
120	Co	5.2891	10.3557	5.2802	0.3804	0.7448	0.2405
121	Co	8.6806	-0.0071	5.2718	0.6244	-0.0005	0.2402
122	Co	8.7182	3.4654	5.2260	0.6271	0.2493	0.2381
123	Co	12.1654	0.0280	5.2209	0.8750	0.0020	0.2378
124	Co	12.1546	3.4720	5.2288	0.8742	0.2497	0.2382
125	Co	8.7209	6.9783	5.1764	0.6273	0.5019	0.2358
126	Co	8.6923	10.4496	5.2585	0.6252	0.7516	0.2395
127	Co	12.1636	6.9600	5.2197	0.8749	0.5006	0.2378
128	Co	12.1507	10.4216	5.2094	0.8739	0.7496	0.2373
129	O	6.0260	6.1801	6.8372	0.4334	0.4445	0.3115
130	O	2.5749	7.8052	6.8464	0.1852	0.5614	0.3119
131	O	4.6880	7.8970	8.7975	0.3372	0.5680	0.4008
132	O	5.5973	10.1119	7.1716	0.4026	0.7273	0.3267
133	O	7.6941	10.0858	8.9537	0.5534	0.7254	0.4079
134	O	4.2847	12.1403	8.4453	0.3082	0.8732	0.3847
135	O	8.5834	12.1948	6.3679	0.6174	0.8771	0.2901
136	Ce	3.4324	9.7870	7.9341	0.2469	0.7039	0.3614
137	Ce	3.8900	6.1155	7.6875	0.2798	0.4399	0.3502
138	Ce	6.8581	8.2785	7.9767	0.4933	0.5954	0.3634

139 Sm	6.9412	11.7537	7.9398	0.4992	0.8454	0.3617
140 C	4.3441	12.8264	7.3474	0.3125	0.9225	0.3347
141 O	2.0728	11.2648	6.7380	0.1491	0.8102	0.3069
142 O	5.2559	13.7602	7.2985	0.3780	0.9897	0.3325

/db/jmorales/CoNi-alloy/Profiles/CoNi-Ce_3SmO_7/O-coverage/M-CO2-O-coverage/M-CO_2-CoNi-Ce_3SmO_7-6O

a =			13.9033384264				
b =			13.9033384264				
c =			21.9516692133				
alpha =			90.0				
beta =			90.0				
gamma =			90.0				
	Atom	X	Y	Z	X	Y	Z
1	Ni	0.0000	0.0000	0.0000	0.0000	0.0000	0.0000
2	Ni	0.0000	3.4758	0.0000	0.0000	0.2500	0.0000
3	Ni	3.4758	0.0000	0.0000	0.2500	0.0000	0.0000
4	Ni	3.4758	3.4758	0.0000	0.2500	0.2500	0.0000
5	Ni	0.0000	6.9517	0.0000	0.0000	0.5000	0.0000
6	Ni	0.0000	10.4275	0.0000	0.0000	0.7500	0.0000
7	Ni	3.4758	6.9517	0.0000	0.2500	0.5000	0.0000
8	Ni	3.4758	10.4275	0.0000	0.2500	0.7500	0.0000
9	Ni	6.9517	0.0000	0.0000	0.5000	0.0000	0.0000
10	Ni	6.9517	3.4758	0.0000	0.5000	0.2500	0.0000
11	Ni	10.4275	0.0000	0.0000	0.7500	0.0000	0.0000
12	Ni	10.4275	3.4758	0.0000	0.7500	0.2500	0.0000
13	Ni	6.9517	6.9517	0.0000	0.5000	0.5000	0.0000
14	Ni	6.9517	10.4275	0.0000	0.5000	0.7500	0.0000
15	Ni	10.4275	6.9517	0.0000	0.7500	0.5000	0.0000
16	Ni	10.4275	10.4275	0.0000	0.7500	0.7500	0.0000
17	Ni	0.0000	1.7379	1.7379	0.0000	0.1250	0.0792
18	Ni	0.0000	5.2138	1.7379	0.0000	0.3750	0.0792
19	Ni	3.4758	1.7379	1.7379	0.2500	0.1250	0.0792
20	Ni	3.4758	5.2138	1.7379	0.2500	0.3750	0.0792
21	Ni	0.0000	8.6896	1.7379	0.0000	0.6250	0.0792
22	Ni	0.0000	12.1654	1.7379	0.0000	0.8750	0.0792
23	Ni	3.4758	8.6896	1.7379	0.2500	0.6250	0.0792
24	Ni	3.4758	12.1654	1.7379	0.2500	0.8750	0.0792
25	Ni	6.9517	1.7379	1.7379	0.5000	0.1250	0.0792
26	Ni	6.9517	5.2138	1.7379	0.5000	0.3750	0.0792
27	Ni	10.4275	1.7379	1.7379	0.7500	0.1250	0.0792
28	Ni	10.4275	5.2138	1.7379	0.7500	0.3750	0.0792
29	Ni	6.9517	8.6896	1.7379	0.5000	0.6250	0.0792
30	Ni	6.9517	12.1654	1.7379	0.5000	0.8750	0.0792
31	Ni	10.4275	8.6896	1.7379	0.7500	0.6250	0.0792
32	Ni	10.4275	12.1654	1.7379	0.7500	0.8750	0.0792
33	Ni	13.8988	13.8962	3.4560	0.9997	0.9995	0.1574
34	Ni	0.0083	3.4679	3.5273	0.0006	0.2494	0.1607
35	Ni	3.4622	0.0172	3.5150	0.2490	0.0012	0.1601
36	Ni	3.4833	3.4210	3.4559	0.2505	0.2461	0.1574
37	Ni	0.0003	6.9644	3.5150	0.0000	0.5009	0.1601
38	Ni	13.8812	10.4424	3.4418	0.9984	0.7511	0.1568
39	Ni	3.4722	6.9647	3.4842	0.2497	0.5009	0.1587

40	Ni	3.4807	10.3922	3.5104	0.2504	0.7475	0.1599
41	Ni	6.9512	0.0158	3.5102	0.5000	0.0011	0.1599
42	Ni	6.9532	3.4847	3.4629	0.5001	0.2506	0.1577
43	Ni	10.4388	0.0307	3.4679	0.7508	0.0022	0.1580
44	Ni	10.4208	3.4690	3.4565	0.7495	0.2495	0.1575
45	Ni	6.9630	6.9677	3.5074	0.5008	0.5012	0.1598
46	Ni	6.9695	10.4361	3.4746	0.5013	0.7506	0.1583
47	Ni	10.4283	6.9291	3.5010	0.7501	0.4984	0.1595
48	Ni	10.4482	10.4439	3.5178	0.7515	0.7512	0.1603
49	Ni	0.0511	1.7796	5.2690	0.0037	0.1280	0.2400
50	Ni	0.0097	5.2202	5.2065	0.0007	0.3755	0.2372
51	Ni	3.4460	1.7278	5.3219	0.2479	0.1243	0.2424
52	Ni	3.4670	5.2204	5.0587	0.2494	0.3755	0.2304
53	Ni	13.8622	8.6566	5.2254	0.9970	0.6226	0.2380
54	Ni	13.8718	12.1523	5.2605	0.9977	0.8741	0.2396
55	Ni	3.4447	8.6910	5.2473	0.2478	0.6251	0.2390
56	Ni	3.4624	12.1074	5.1812	0.2490	0.8708	0.2360
57	Ni	6.9759	1.7229	5.2420	0.5017	0.1239	0.2388
58	Ni	6.9756	5.2399	5.3701	0.5017	0.3769	0.2446
59	Ni	10.4105	1.7414	5.2735	0.7488	0.1253	0.2402
60	Ni	10.4120	5.2533	5.2594	0.7489	0.3778	0.2396
61	Ni	7.0019	8.7214	5.2369	0.5036	0.6273	0.2386
62	Ni	6.9198	12.2126	5.1743	0.4977	0.8784	0.2357
63	Ni	10.4178	8.6878	5.2024	0.7493	0.6249	0.2370
64	Ni	10.5132	12.1674	5.2573	0.7562	0.8751	0.2395
65	Co	1.7379	1.7379	0.0000	0.1250	0.1250	0.0000
66	Co	1.7379	5.2138	0.0000	0.1250	0.3750	0.0000
67	Co	5.2138	1.7379	0.0000	0.3750	0.1250	0.0000
68	Co	5.2138	5.2138	0.0000	0.3750	0.3750	0.0000
69	Co	1.7379	8.6896	0.0000	0.1250	0.6250	0.0000
70	Co	1.7379	12.1654	0.0000	0.1250	0.8750	0.0000
71	Co	5.2138	8.6896	0.0000	0.3750	0.6250	0.0000
72	Co	5.2138	12.1654	0.0000	0.3750	0.8750	0.0000
73	Co	8.6896	1.7379	0.0000	0.6250	0.1250	0.0000
74	Co	8.6896	5.2138	0.0000	0.6250	0.3750	0.0000
75	Co	12.1654	1.7379	0.0000	0.8750	0.1250	0.0000
76	Co	12.1654	5.2138	0.0000	0.8750	0.3750	0.0000
77	Co	8.6896	8.6896	0.0000	0.6250	0.6250	0.0000
78	Co	8.6896	12.1654	0.0000	0.6250	0.8750	0.0000
79	Co	12.1654	8.6896	0.0000	0.8750	0.6250	0.0000
80	Co	12.1654	12.1654	0.0000	0.8750	0.8750	0.0000
81	Co	1.7379	0.0000	1.7379	0.1250	0.0000	0.0792
82	Co	1.7379	3.4758	1.7379	0.1250	0.2500	0.0792
83	Co	5.2138	0.0000	1.7379	0.3750	0.0000	0.0792
84	Co	5.2138	3.4758	1.7379	0.3750	0.2500	0.0792
85	Co	1.7379	6.9517	1.7379	0.1250	0.5000	0.0792
86	Co	1.7379	10.4275	1.7379	0.1250	0.7500	0.0792
87	Co	5.2138	6.9517	1.7379	0.3750	0.5000	0.0792
88	Co	5.2138	10.4275	1.7379	0.3750	0.7500	0.0792

89	Co	8.6896	0.0000	1.7379	0.6250	0.0000	0.0792
90	Co	8.6896	3.4758	1.7379	0.6250	0.2500	0.0792
91	Co	12.1654	0.0000	1.7379	0.8750	0.0000	0.0792
92	Co	12.1654	3.4758	1.7379	0.8750	0.2500	0.0792
93	Co	8.6896	6.9517	1.7379	0.6250	0.5000	0.0792
94	Co	8.6896	10.4275	1.7379	0.6250	0.7500	0.0792
95	Co	12.1654	6.9517	1.7379	0.8750	0.5000	0.0792
96	Co	12.1654	10.4275	1.7379	0.8750	0.7500	0.0792
97	Co	1.7415	1.7421	3.5628	0.1253	0.1253	0.1623
98	Co	1.7088	5.2299	3.5071	0.1229	0.3762	0.1598
99	Co	5.2134	1.7354	3.5671	0.3750	0.1248	0.1625
100	Co	5.2441	5.1916	3.4976	0.3772	0.3734	0.1593
101	Co	1.7274	8.6888	3.5160	0.1242	0.6249	0.1602
102	Co	1.7306	12.1645	3.5270	0.1245	0.8749	0.1607
103	Co	5.2040	8.6941	3.5581	0.3743	0.6253	0.1621
104	Co	5.2050	12.1527	3.5455	0.3744	0.8741	0.1615
105	Co	8.6888	1.7400	3.5414	0.6249	0.1252	0.1613
106	Co	8.6721	5.2016	3.5598	0.6237	0.3741	0.1622
107	Co	12.1834	1.7527	3.5559	0.8763	0.1261	0.1620
108	Co	12.1678	5.2051	3.5515	0.8752	0.3744	0.1618
109	Co	8.6984	8.7058	3.5368	0.6256	0.6262	0.1611
110	Co	8.7171	12.1903	3.4805	0.6270	0.8768	0.1586
111	Co	12.1479	8.6817	3.5366	0.8737	0.6244	0.1611
112	Co	12.1562	12.1799	3.5451	0.8743	0.8760	0.1615
113	Co	1.7337	13.8197	5.3276	0.1247	0.9940	0.2427
114	Co	1.7792	3.5291	5.2762	0.1280	0.2538	0.2404
115	Co	5.2137	13.8827	5.2599	0.3750	0.9985	0.2396
116	Co	5.1835	3.4711	5.3224	0.3728	0.2497	0.2425
117	Co	1.7091	6.9717	5.2916	0.1229	0.5014	0.2411
118	Co	1.6685	10.4231	5.2033	0.1200	0.7497	0.2370
119	Co	5.2376	6.9145	5.1529	0.3767	0.4973	0.2347
120	Co	5.2293	10.4085	5.2328	0.3761	0.7486	0.2384
121	Co	8.7164	13.8433	5.3227	0.6269	0.9957	0.2425
122	Co	8.7036	3.4892	5.3566	0.6260	0.2510	0.2440
123	Co	12.2087	0.0495	5.3590	0.8781	0.0036	0.2441
124	Co	12.1569	3.4940	5.2671	0.8744	0.2513	0.2399
125	Co	8.7128	6.9847	5.1767	0.6267	0.5024	0.2358
126	Co	8.7489	10.4900	5.2197	0.6293	0.7545	0.2378
127	Co	12.1597	6.9436	5.2072	0.8746	0.4994	0.2372
128	Co	12.1854	10.3920	5.2701	0.8764	0.7474	0.2401
129	O	5.7536	6.0678	6.7752	0.4138	0.4364	0.3086
130	O	2.5720	7.8410	6.7813	0.1850	0.5640	0.3089
131	O	4.7370	7.8219	8.7919	0.3407	0.5626	0.4005
132	O	6.1453	9.6682	6.8367	0.4420	0.6954	0.3114
133	O	8.0883	9.3344	8.9871	0.5818	0.6714	0.4094
134	O	5.3477	11.1953	9.0982	0.3846	0.8052	0.4145
135	O	8.3968	12.0038	6.3735	0.6039	0.8634	0.2903
136	Ce	3.9502	9.9903	8.0882	0.2841	0.7186	0.3685
137	Ce	3.6665	6.1143	7.7485	0.2637	0.4398	0.3530

138	Ce	6.9712	7.7195	8.0887	0.5014	0.5552	0.3685
139	Sm	7.3232	11.1605	8.2781	0.5267	0.8027	0.3771
140	C	3.1566	13.2484	6.7410	0.2270	0.9529	0.3071
141	O	3.9792	0.4249	6.8109	0.2862	0.0306	0.3103
142	O	2.8987	12.5279	7.7381	0.2085	0.9011	0.3525
143	O	10.4921	0.0211	6.2198	0.7546	0.0015	0.2833
144	O	0.0393	0.0970	6.2133	0.0028	0.0070	0.2830
145	O	0.0159	10.3557	6.2062	0.0011	0.7448	0.2827
146	O	6.9453	3.3666	6.2172	0.4995	0.2421	0.2832
147	O	3.4259	3.8028	6.3683	0.2464	0.2735	0.2901
148	O	10.4865	3.5560	6.2184	0.7542	0.2558	0.2833

/db/jmorales/CoNi-alloy/Profiles/Ni-Ce_3SmO_7/M-CO2

a =				13.9019837372			
b =				13.9019837372			
c =				21.9509918687			
alpha =				90.0			
beta =				90.0			
gamma =				90.0			
	Atom	X	Y	Z	X	Y	Z
1	Ni	0.0000	0.0000	0.0000	0.0000	0.0000	0.0000
2	Ni	1.7377	1.7377	0.0000	0.1250	0.1250	0.0000
3	Ni	0.0000	3.4755	0.0000	0.0000	0.2500	0.0000
4	Ni	1.7377	5.2132	0.0000	0.1250	0.3750	0.0000
5	Ni	3.4755	0.0000	0.0000	0.2500	0.0000	0.0000
6	Ni	5.2132	1.7377	0.0000	0.3750	0.1250	0.0000
7	Ni	3.4755	3.4755	0.0000	0.2500	0.2500	0.0000
8	Ni	5.2132	5.2132	0.0000	0.3750	0.3750	0.0000
9	Ni	0.0000	6.9510	0.0000	0.0000	0.5000	0.0000
10	Ni	1.7377	8.6887	0.0000	0.1250	0.6250	0.0000
11	Ni	0.0000	10.4265	0.0000	0.0000	0.7500	0.0000
12	Ni	1.7377	12.1642	0.0000	0.1250	0.8750	0.0000
13	Ni	3.4755	6.9510	0.0000	0.2500	0.5000	0.0000
14	Ni	5.2132	8.6887	0.0000	0.3750	0.6250	0.0000
15	Ni	3.4755	10.4265	0.0000	0.2500	0.7500	0.0000
16	Ni	5.2132	12.1642	0.0000	0.3750	0.8750	0.0000
17	Ni	6.9510	0.0000	0.0000	0.5000	0.0000	0.0000
18	Ni	8.6887	1.7377	0.0000	0.6250	0.1250	0.0000
19	Ni	6.9510	3.4755	0.0000	0.5000	0.2500	0.0000
20	Ni	8.6887	5.2132	0.0000	0.6250	0.3750	0.0000
21	Ni	10.4265	0.0000	0.0000	0.7500	0.0000	0.0000
22	Ni	12.1642	1.7377	0.0000	0.8750	0.1250	0.0000
23	Ni	10.4265	3.4755	0.0000	0.7500	0.2500	0.0000
24	Ni	12.1642	5.2132	0.0000	0.8750	0.3750	0.0000
25	Ni	6.9510	6.9510	0.0000	0.5000	0.5000	0.0000
26	Ni	8.6887	8.6887	0.0000	0.6250	0.6250	0.0000
27	Ni	6.9510	10.4265	0.0000	0.5000	0.7500	0.0000
28	Ni	8.6887	12.1642	0.0000	0.6250	0.8750	0.0000
29	Ni	10.4265	6.9510	0.0000	0.7500	0.5000	0.0000
30	Ni	12.1642	8.6887	0.0000	0.8750	0.6250	0.0000
31	Ni	10.4265	10.4265	0.0000	0.7500	0.7500	0.0000
32	Ni	12.1642	12.1642	0.0000	0.8750	0.8750	0.0000
33	Ni	0.0000	1.7377	1.7377	0.0000	0.1250	0.0792
34	Ni	1.7377	0.0000	1.7377	0.1250	0.0000	0.0792
35	Ni	0.0000	5.2132	1.7377	0.0000	0.3750	0.0792
36	Ni	1.7377	3.4755	1.7377	0.1250	0.2500	0.0792
37	Ni	3.4755	1.7377	1.7377	0.2500	0.1250	0.0792
38	Ni	5.2132	0.0000	1.7377	0.3750	0.0000	0.0792
39	Ni	3.4755	5.2132	1.7377	0.2500	0.3750	0.0792
40	Ni	5.2132	3.4755	1.7377	0.3750	0.2500	0.0792

41	Ni	0.0000	8.6887	1.7377	0.0000	0.6250	0.0792
42	Ni	1.7377	6.9510	1.7377	0.1250	0.5000	0.0792
43	Ni	0.0000	12.1642	1.7377	0.0000	0.8750	0.0792
44	Ni	1.7377	10.4265	1.7377	0.1250	0.7500	0.0792
45	Ni	3.4755	8.6887	1.7377	0.2500	0.6250	0.0792
46	Ni	5.2132	6.9510	1.7377	0.3750	0.5000	0.0792
47	Ni	3.4755	12.1642	1.7377	0.2500	0.8750	0.0792
48	Ni	5.2132	10.4265	1.7377	0.3750	0.7500	0.0792
49	Ni	6.9510	1.7377	1.7377	0.5000	0.1250	0.0792
50	Ni	8.6887	0.0000	1.7377	0.6250	0.0000	0.0792
51	Ni	6.9510	5.2132	1.7377	0.5000	0.3750	0.0792
52	Ni	8.6887	3.4755	1.7377	0.6250	0.2500	0.0792
53	Ni	10.4265	1.7377	1.7377	0.7500	0.1250	0.0792
54	Ni	12.1642	0.0000	1.7377	0.8750	0.0000	0.0792
55	Ni	10.4265	5.2132	1.7377	0.7500	0.3750	0.0792
56	Ni	12.1642	3.4755	1.7377	0.8750	0.2500	0.0792
57	Ni	6.9510	8.6887	1.7377	0.5000	0.6250	0.0792
58	Ni	8.6887	6.9510	1.7377	0.6250	0.5000	0.0792
59	Ni	6.9510	12.1642	1.7377	0.5000	0.8750	0.0792
60	Ni	8.6887	10.4265	1.7377	0.6250	0.7500	0.0792
61	Ni	10.4265	8.6887	1.7377	0.7500	0.6250	0.0792
62	Ni	12.1642	6.9510	1.7377	0.8750	0.5000	0.0792
63	Ni	10.4265	12.1642	1.7377	0.7500	0.8750	0.0792
64	Ni	12.1642	10.4265	1.7377	0.8750	0.7500	0.0792
65	Ni	0.0049	0.0090	3.5093	0.0004	0.0006	0.1599
66	Ni	1.7336	1.7405	3.5083	0.1247	0.1252	0.1598
67	Ni	13.8782	3.4729	3.5216	0.9983	0.2498	0.1604
68	Ni	1.6839	5.1996	3.5032	0.1211	0.3740	0.1596
69	Ni	3.4756	0.0039	3.5104	0.2500	0.0003	0.1599
70	Ni	5.2204	1.7462	3.5246	0.3755	0.1256	0.1606
71	Ni	3.4968	3.4499	3.5029	0.2515	0.2482	0.1596
72	Ni	5.2202	5.2074	3.4962	0.3755	0.3746	0.1593
73	Ni	13.8979	6.9422	3.5131	0.9997	0.4994	0.1600
74	Ni	1.7522	8.6911	3.5172	0.1260	0.6252	0.1602
75	Ni	13.8897	10.4294	3.5207	0.9991	0.7502	0.1604
76	Ni	1.7437	12.1629	3.5076	0.1254	0.8749	0.1598
77	Ni	3.4685	6.9682	3.4870	0.2495	0.5012	0.1589
78	Ni	5.2239	8.6992	3.5096	0.3758	0.6258	0.1599
79	Ni	3.4757	10.3978	3.5346	0.2500	0.7479	0.1610
80	Ni	5.2059	12.1645	3.5035	0.3745	0.8750	0.1596
81	Ni	6.9417	13.8938	3.5028	0.4993	0.9994	0.1596
82	Ni	8.6907	1.7132	3.4926	0.6251	0.1232	0.1591
83	Ni	6.9473	3.4663	3.5005	0.4997	0.2493	0.1595
84	Ni	8.6615	5.2387	3.5020	0.6230	0.3768	0.1595
85	Ni	10.4373	13.8779	3.5191	0.7508	0.9983	0.1603
86	Ni	12.1600	1.7422	3.5132	0.8747	0.1253	0.1600
87	Ni	10.4223	3.4681	3.4879	0.7497	0.2495	0.1589
88	Ni	12.1652	5.2147	3.5013	0.8751	0.3751	0.1595
89	Ni	6.9320	6.9446	3.5122	0.4986	0.4995	0.1600

90	Ni	8.6933	8.6963	3.4633	0.6253	0.6255	0.1578
91	Ni	6.9685	10.4305	3.5097	0.5013	0.7503	0.1599
92	Ni	8.6917	12.1787	3.4906	0.6252	0.8760	0.1590
93	Ni	10.4203	6.9670	3.5066	0.7496	0.5012	0.1597
94	Ni	12.1863	8.6651	3.5081	0.8766	0.6233	0.1598
95	Ni	10.4485	10.4439	3.4308	0.7516	0.7513	0.1563
96	Ni	12.1770	12.1694	3.5060	0.8759	0.8754	0.1597
97	Ni	13.8911	1.7543	5.2285	0.9992	0.1262	0.2382
98	Ni	1.7388	0.0072	5.2237	0.1251	0.0005	0.2380
99	Ni	0.0176	5.1768	5.3409	0.0013	0.3724	0.2433
100	Ni	1.7455	3.4649	5.2158	0.1256	0.2492	0.2376
101	Ni	3.4782	1.7395	5.2299	0.2502	0.1251	0.2383
102	Ni	5.2068	13.8999	5.2247	0.3745	0.9999	0.2380
103	Ni	3.4383	5.1787	5.1001	0.2473	0.3725	0.2323
104	Ni	5.2301	3.4468	5.3580	0.3762	0.2479	0.2441
105	Ni	0.0273	8.6977	5.2115	0.0020	0.6256	0.2374
106	Ni	1.7274	6.9318	5.1920	0.1243	0.4986	0.2365
107	Ni	0.0097	12.1740	5.2212	0.0007	0.8757	0.2379
108	Ni	1.7342	10.4350	5.2274	0.1247	0.7506	0.2381
109	Ni	3.4576	8.7119	5.4040	0.2487	0.6267	0.2462
110	Ni	5.1902	6.9558	5.2288	0.3733	0.5003	0.2382
111	Ni	3.4766	12.1588	5.2272	0.2501	0.8746	0.2381
112	Ni	5.2280	10.4396	5.2035	0.3761	0.7509	0.2371
113	Ni	6.9761	1.6934	5.2079	0.5018	0.1218	0.2373
114	Ni	8.6897	13.8871	5.2189	0.6251	0.9989	0.2378
115	Ni	6.9690	5.2122	5.2718	0.5013	0.3749	0.2402
116	Ni	8.7020	3.4479	5.1624	0.6260	0.2480	0.2352
117	Ni	10.4159	1.7098	5.2139	0.7492	0.1230	0.2375
118	Ni	12.1791	0.0122	5.2216	0.8761	0.0009	0.2379
119	Ni	10.3840	5.2143	5.2000	0.7469	0.3751	0.2369
120	Ni	12.0775	3.4889	5.3077	0.8688	0.2510	0.2418
121	Ni	6.9782	8.7402	5.2395	0.5020	0.6287	0.2387
122	Ni	8.7118	6.9722	5.2574	0.6267	0.5015	0.2395
123	Ni	6.9434	12.1632	5.2230	0.4995	0.8749	0.2379
124	Ni	8.7163	10.4717	5.1950	0.6270	0.7533	0.2367
125	Ni	10.4573	8.7163	5.1748	0.7522	0.6270	0.2357
126	Ni	12.1494	6.9825	5.3105	0.8739	0.5023	0.2419
127	Ni	10.4512	12.1803	5.2828	0.7518	0.8762	0.2407
128	Ni	12.1905	10.4292	5.2822	0.8769	0.7502	0.2406
129	O	6.2376	4.3185	6.7899	0.4487	0.3106	0.3093
130	O	4.1939	7.5642	6.7915	0.3017	0.5441	0.3094
131	O	6.1683	6.5634	8.7386	0.4437	0.4721	0.3981
132	O	7.9329	7.9501	6.7960	0.5706	0.5719	0.3096
133	O	10.4313	10.4082	6.2666	0.7503	0.7487	0.2855
134	O	9.5724	7.2722	8.9967	0.6886	0.5231	0.4099
135	O	7.5930	9.9407	8.8749	0.5462	0.7151	0.4043
136	Ce	5.9777	8.7987	8.0131	0.4300	0.6329	0.3650
137	Ce	4.5340	5.4503	7.7228	0.3261	0.3920	0.3518
138	Ce	8.3891	5.8133	8.0453	0.6034	0.4182	0.3665

139 Sm	9.4172	9.2136	8.1042	0.6774	0.6628	0.3692
140 C	11.5337	4.8800	6.7266	0.8296	0.3510	0.3064
141 O	12.6391	5.6844	6.7754	0.9092	0.4089	0.3087
142 O	10.8536	4.6329	7.7586	0.7807	0.3333	0.3534

/db/jmorales/CoNi-alloy/Profiles/Sm(subsurf)$Ce_{26}O_{54}$/CO-O

a =				11.6082000732			
b =				11.608200073110298			
c =				22.898399353			
alpha =				90.0			
beta =				90.0			
gamma =				120.0			
	Atom	X	Y	Z	X	Y	Z
1	Sm	1.9328	5.5972	3.9650	0.4449	0.5568	0.1732
2	Ce	1.9347	1.1170	0.7898	0.2222	0.1111	0.0345
3	Ce	0.0000	4.4680	0.7898	0.2222	0.4444	0.0345
4	Ce	-1.9347	7.8190	0.7898	0.2222	0.7778	0.0345
5	Ce	5.8041	1.1170	0.7898	0.5556	0.1111	0.0345
6	Ce	3.8694	4.4680	0.7898	0.5556	0.4444	0.0345
7	Ce	1.9347	7.8190	0.7898	0.5556	0.7778	0.0345
8	Ce	9.6735	1.1170	0.7898	0.8889	0.1111	0.0345
9	Ce	7.7388	4.4680	0.7898	0.8889	0.4444	0.0345
10	Ce	5.8041	7.8190	0.7898	0.8889	0.7778	0.0345
11	Ce	0.0012	2.2543	3.9467	0.1122	0.2242	0.1724
12	Ce	-1.9171	5.5808	3.9271	0.1124	0.5551	0.1715
13	Ce	-3.8692	8.9290	3.9469	0.1108	0.8882	0.1724
14	Ce	3.8688	2.2554	3.9454	0.4455	0.2244	0.1723
15	Ce	0.0165	8.9126	3.9575	0.4447	0.8866	0.1728
16	Ce	7.7384	2.2404	3.9169	0.7781	0.2229	0.1711
17	Ce	5.7905	5.5783	3.9241	0.7763	0.5549	0.1714
18	Ce	3.8507	8.9121	3.9570	0.7750	0.8865	0.1728
19	Ce	0.0011	-0.0025	7.0931	-0.0000	-0.0002	0.3098
20	Ce	9.6711	3.3434	7.0989	0.9994	0.3326	0.3100
21	Ce	-3.8658	6.6974	7.0854	0.0001	0.6662	0.3094
22	Ce	3.8693	0.0034	7.0887	0.3335	0.0003	0.3096
23	Ce	1.9374	3.3408	7.0952	0.3331	0.3323	0.3099
24	Ce	-0.1131	6.6376	7.0978	0.3204	0.6603	0.3100
25	Ce	7.7389	0.1394	7.0737	0.6736	0.0139	0.3089
26	Ce	5.8091	3.3453	7.1022	0.6668	0.3328	0.3102
27	Ce	3.9907	6.6257	7.0879	0.6733	0.6591	0.3095
28	O	0.0000	0.0000	0.0000	0.0000	0.0000	0.0000
29	O	-1.9347	3.3510	0.0000	-0.0000	0.3333	0.0000
30	O	-3.8694	6.7020	0.0000	-0.0000	0.6667	0.0000
31	O	3.8694	0.0000	0.0000	0.3333	0.0000	0.0000
32	O	1.9347	3.3510	0.0000	0.3333	0.3333	0.0000
33	O	0.0000	6.7020	0.0000	0.3333	0.6667	0.0000
34	O	7.7388	0.0000	0.0000	0.6667	0.0000	0.0000
35	O	5.8041	3.3510	0.0000	0.6667	0.3333	0.0000
36	O	3.8694	6.7020	0.0000	0.6667	0.6667	0.0000
37	O	-0.0000	2.2340	1.5798	0.1111	0.2222	0.0690
38	O	-1.9347	5.5850	1.5798	0.1111	0.5556	0.0690
39	O	-3.8694	8.9360	1.5798	0.1111	0.8889	0.0690
40	O	3.8694	2.2340	1.5798	0.4444	0.2222	0.0690

41	O	1.9347	5.5850	1.5798	0.4444	0.5556	0.0690
42	O	-0.0000	8.9360	1.5798	0.4444	0.8889	0.0690
43	O	7.7388	2.2340	1.5798	0.7778	0.2222	0.0690
44	O	5.8041	5.5850	1.5798	0.7778	0.5556	0.0690
45	O	3.8694	8.9360	1.5798	0.7778	0.8889	0.0690
46	O	1.9347	1.1170	3.1593	0.2222	0.1111	0.1380
47	O	0.0000	4.4680	3.1593	0.2222	0.4444	0.1380
48	O	-1.9347	7.8190	3.1593	0.2222	0.7778	0.1380
49	O	5.8041	1.1170	3.1593	0.5556	0.1111	0.1380
50	O	3.8694	4.4680	3.1593	0.5556	0.4444	0.1380
51	O	1.9347	7.8190	3.1593	0.5556	0.7778	0.1380
52	O	9.6735	1.1170	3.1593	0.8889	0.1111	0.1380
53	O	7.7388	4.4680	3.1593	0.8889	0.4444	0.1380
54	O	5.8041	7.8190	3.1593	0.8889	0.7778	0.1380
55	O	5.7654	10.0237	4.7906	0.9952	0.9971	0.2092
56	O	-1.9314	3.3481	4.7607	0.0001	0.3330	0.2079
57	O	-3.8662	6.7075	4.7598	0.0005	0.6672	0.2079
58	O	-1.9000	10.0301	4.7843	0.3352	0.9977	0.2089
59	O	1.9350	3.3459	4.8329	0.3331	0.3328	0.2111
60	O	-0.0622	6.7164	4.7971	0.3287	0.6681	0.2095
61	O	7.7399	0.0238	4.7621	0.6679	0.0024	0.2080
62	O	5.8008	3.3456	4.7593	0.6661	0.3328	0.2078
63	O	3.9326	6.7142	4.7920	0.6727	0.6679	0.2093
64	O	-0.0034	2.2076	6.3050	0.1095	0.2196	0.2753
65	O	-1.9535	5.5647	6.3101	0.1085	0.5535	0.2756
66	O	-3.8646	8.9575	6.2901	0.1126	0.8910	0.2747
67	O	3.8752	2.2099	6.3069	0.4437	0.2198	0.2754
68	O	1.9299	5.7289	6.4853	0.4512	0.5699	0.2832
69	O	0.0897	8.9004	6.3945	0.4504	0.8853	0.2793
70	O	7.7386	2.2566	6.2836	0.7789	0.2245	0.2744
71	O	5.8384	5.5575	6.3034	0.7794	0.5528	0.2753
72	O	3.7636	8.8891	6.4069	0.7663	0.8842	0.2798
73	O	1.9342	1.1137	7.8894	0.2220	0.1108	0.3445
74	O	-0.0359	4.4124	7.8796	0.2164	0.4389	0.3441
75	O	-1.9718	7.8125	7.8714	0.2187	0.7771	0.3438
76	O	5.8191	1.1474	7.8934	0.5584	0.1141	0.3447
77	O	3.9142	4.4249	7.8716	0.5573	0.4402	0.3438
78	O	1.7227	8.0281	8.3898	0.5477	0.7986	0.3664
79	O	9.6595	1.1445	7.8891	0.8890	0.1138	0.3445
80	O	7.7460	4.4578	7.9026	0.8890	0.4434	0.3451
81	O	5.8561	7.8066	7.8592	0.8928	0.7765	0.3432
82	C	-0.1160	6.3490	10.0047	0.3058	0.6316	0.4369
83	O	-0.1288	6.0826	11.1151	0.2914	0.6051	0.4854
84	O	2.7339	7.4522	9.0797	0.6062	0.7413	0.3965

/db/jmorales/CoNi-alloy/Profiles/CoNi-Ce_3SmO_7/O-coverage/CO-O-O-coverage/CO-O-CoNi-Ce_3SmO_7-6O

a =			13.9033384264				
b =			13.9033384264				
c =			21.9516692133				
alpha =			90.0				
beta =			90.0				
gamma =			90.0				
	Atom	X	Y	Z	X	Y	Z
1	Ni	0.0000	0.0000	0.0000	0.0000	0.0000	0.0000
2	Ni	0.0000	3.4758	0.0000	0.0000	0.2500	0.0000
3	Ni	3.4758	0.0000	0.0000	0.2500	0.0000	0.0000
4	Ni	3.4758	3.4758	0.0000	0.2500	0.2500	0.0000
5	Ni	0.0000	6.9517	0.0000	0.0000	0.5000	0.0000
6	Ni	0.0000	10.4275	0.0000	0.0000	0.7500	0.0000
7	Ni	3.4758	6.9517	0.0000	0.2500	0.5000	0.0000
8	Ni	3.4758	10.4275	0.0000	0.2500	0.7500	0.0000
9	Ni	6.9517	0.0000	0.0000	0.5000	0.0000	0.0000
10	Ni	6.9517	3.4758	0.0000	0.5000	0.2500	0.0000
11	Ni	10.4275	0.0000	0.0000	0.7500	0.0000	0.0000
12	Ni	10.4275	3.4758	0.0000	0.7500	0.2500	0.0000
13	Ni	6.9517	6.9517	0.0000	0.5000	0.5000	0.0000
14	Ni	6.9517	10.4275	0.0000	0.5000	0.7500	0.0000
15	Ni	10.4275	6.9517	0.0000	0.7500	0.5000	0.0000
16	Ni	10.4275	10.4275	0.0000	0.7500	0.7500	0.0000
17	Ni	0.0000	1.7379	1.7379	0.0000	0.1250	0.0792
18	Ni	0.0000	5.2138	1.7379	0.0000	0.3750	0.0792
19	Ni	3.4758	1.7379	1.7379	0.2500	0.1250	0.0792
20	Ni	3.4758	5.2138	1.7379	0.2500	0.3750	0.0792
21	Ni	0.0000	8.6896	1.7379	0.0000	0.6250	0.0792
22	Ni	0.0000	12.1654	1.7379	0.0000	0.8750	0.0792
23	Ni	3.4758	8.6896	1.7379	0.2500	0.6250	0.0792
24	Ni	3.4758	12.1654	1.7379	0.2500	0.8750	0.0792
25	Ni	6.9517	1.7379	1.7379	0.5000	0.1250	0.0792
26	Ni	6.9517	5.2138	1.7379	0.5000	0.3750	0.0792
27	Ni	10.4275	1.7379	1.7379	0.7500	0.1250	0.0792
28	Ni	10.4275	5.2138	1.7379	0.7500	0.3750	0.0792
29	Ni	6.9517	8.6896	1.7379	0.5000	0.6250	0.0792
30	Ni	6.9517	12.1654	1.7379	0.5000	0.8750	0.0792
31	Ni	10.4275	8.6896	1.7379	0.7500	0.6250	0.0792
32	Ni	10.4275	12.1654	1.7379	0.7500	0.8750	0.0792
33	Ni	13.9014	13.8908	3.4640	0.9999	0.9991	0.1578
34	Ni	0.0083	3.4593	3.5306	0.0006	0.2488	0.1608
35	Ni	3.4729	-0.0025	3.4464	0.2498	-0.0002	0.1570
36	Ni	3.4776	3.4291	3.4395	0.2501	0.2466	0.1567
37	Ni	0.0090	6.9641	3.5158	0.0006	0.5009	0.1602
38	Ni	13.8828	10.4516	3.4470	0.9985	0.7517	0.1570
39	Ni	3.4657	6.9568	3.4764	0.2493	0.5004	0.1584

40	Ni	3.4770	10.4246	3.5444	0.2501	0.7498	0.1615
41	Ni	6.9506	0.0099	3.4759	0.4999	0.0007	0.1583
42	Ni	6.9537	3.4832	3.4670	0.5001	0.2505	0.1579
43	Ni	10.4340	0.0275	3.4560	0.7505	0.0020	0.1574
44	Ni	10.4168	3.4738	3.4532	0.7492	0.2499	0.1573
45	Ni	6.9528	6.9607	3.5044	0.5001	0.5006	0.1596
46	Ni	6.9638	10.4359	3.4881	0.5009	0.7506	0.1589
47	Ni	10.4342	6.9225	3.4991	0.7505	0.4979	0.1594
48	Ni	10.4447	10.4378	3.5161	0.7512	0.7507	0.1602
49	Ni	0.0055	1.7741	5.2679	0.0004	0.1276	0.2400
50	Ni	0.0169	5.2074	5.2051	0.0012	0.3745	0.2371
51	Ni	3.5038	1.6945	5.2387	0.2520	0.1219	0.2386
52	Ni	3.4691	5.2101	5.0456	0.2495	0.3747	0.2298
53	Ni	13.8766	8.6689	5.2245	0.9981	0.6235	0.2380
54	Ni	13.8904	12.1827	5.2794	0.9991	0.8762	0.2405
55	Ni	3.4276	8.6734	5.2320	0.2465	0.6238	0.2383
56	Ni	3.4811	12.1293	5.2676	0.2504	0.8724	0.2400
57	Ni	6.9202	1.7113	5.3046	0.4977	0.1231	0.2416
58	Ni	6.9712	5.2501	5.3573	0.5014	0.3776	0.2440
59	Ni	10.4313	1.7840	5.2829	0.7503	0.1283	0.2407
60	Ni	10.4022	5.2554	5.2559	0.7482	0.3780	0.2394
61	Ni	6.9662	8.6987	5.2280	0.5010	0.6257	0.2382
62	Ni	7.0281	12.2269	5.2515	0.5055	0.8794	0.2392
63	Ni	10.4547	8.6611	5.1850	0.7520	0.6229	0.2362
64	Ni	10.3755	12.1421	5.3392	0.7463	0.8733	0.2432
65	Co	1.7379	1.7379	0.0000	0.1250	0.1250	0.0000
66	Co	1.7379	5.2138	0.0000	0.1250	0.3750	0.0000
67	Co	5.2138	1.7379	0.0000	0.3750	0.1250	0.0000
68	Co	5.2138	5.2138	0.0000	0.3750	0.3750	0.0000
69	Co	1.7379	8.6896	0.0000	0.1250	0.6250	0.0000
70	Co	1.7379	12.1654	0.0000	0.1250	0.8750	0.0000
71	Co	5.2138	8.6896	0.0000	0.3750	0.6250	0.0000
72	Co	5.2138	12.1654	0.0000	0.3750	0.8750	0.0000
73	Co	8.6896	1.7379	0.0000	0.6250	0.1250	0.0000
74	Co	8.6896	5.2138	0.0000	0.6250	0.3750	0.0000
75	Co	12.1654	1.7379	0.0000	0.8750	0.1250	0.0000
76	Co	12.1654	5.2138	0.0000	0.8750	0.3750	0.0000
77	Co	8.6896	8.6896	0.0000	0.6250	0.6250	0.0000
78	Co	8.6896	12.1654	0.0000	0.6250	0.8750	0.0000
79	Co	12.1654	8.6896	0.0000	0.8750	0.6250	0.0000
80	Co	12.1654	12.1654	0.0000	0.8750	0.8750	0.0000
81	Co	1.7379	0.0000	1.7379	0.1250	0.0000	0.0792
82	Co	1.7379	3.4758	1.7379	0.1250	0.2500	0.0792
83	Co	5.2138	0.0000	1.7379	0.3750	0.0000	0.0792
84	Co	5.2138	3.4758	1.7379	0.3750	0.2500	0.0792
85	Co	1.7379	6.9517	1.7379	0.1250	0.5000	0.0792
86	Co	1.7379	10.4275	1.7379	0.1250	0.7500	0.0792
87	Co	5.2138	6.9517	1.7379	0.3750	0.5000	0.0792
88	Co	5.2138	10.4275	1.7379	0.3750	0.7500	0.0792

89	Co	8.6896	0.0000	1.7379	0.6250	0.0000	0.0792
90	Co	8.6896	3.4758	1.7379	0.6250	0.2500	0.0792
91	Co	12.1654	0.0000	1.7379	0.8750	0.0000	0.0792
92	Co	12.1654	3.4758	1.7379	0.8750	0.2500	0.0792
93	Co	8.6896	6.9517	1.7379	0.6250	0.5000	0.0792
94	Co	8.6896	10.4275	1.7379	0.6250	0.7500	0.0792
95	Co	12.1654	6.9517	1.7379	0.8750	0.5000	0.0792
96	Co	12.1654	10.4275	1.7379	0.8750	0.7500	0.0792
97	Co	1.7352	1.7384	3.5630	0.1248	0.1250	0.1623
98	Co	1.7053	5.2170	3.5079	0.1227	0.3752	0.1598
99	Co	5.2199	1.7408	3.5313	0.3754	0.1252	0.1609
100	Co	5.2441	5.1870	3.4910	0.3772	0.3731	0.1590
101	Co	1.7268	8.6948	3.5193	0.1242	0.6254	0.1603
102	Co	1.7440	12.1685	3.5474	0.1254	0.8752	0.1616
103	Co	5.1905	8.6993	3.5534	0.3733	0.6257	0.1619
104	Co	5.2285	12.1422	3.5664	0.3761	0.8733	0.1625
105	Co	8.6805	1.7523	3.5518	0.6243	0.1260	0.1618
106	Co	8.6707	5.2035	3.5543	0.6236	0.3743	0.1619
107	Co	12.1747	1.7493	3.5522	0.8757	0.1258	0.1618
108	Co	12.1718	5.2039	3.5450	0.8755	0.3743	0.1615
109	Co	8.6934	8.6694	3.5118	0.6253	0.6235	0.1600
110	Co	8.7172	12.1844	3.4884	0.6270	0.8764	0.1589
111	Co	12.1644	8.6840	3.5338	0.8749	0.6246	0.1610
112	Co	12.1314	12.1775	3.5806	0.8726	0.8759	0.1631
113	Co	1.7478	-0.0013	5.3501	0.1257	-0.0001	0.2437
114	Co	1.8047	3.4951	5.2780	0.1298	0.2514	0.2404
115	Co	5.2395	13.8617	5.3203	0.3769	0.9970	0.2424
116	Co	5.1701	3.4631	5.3009	0.3719	0.2491	0.2415
117	Co	1.7138	6.9419	5.2903	0.1233	0.4993	0.2410
118	Co	1.6841	10.4527	5.2269	0.1211	0.7518	0.2381
119	Co	5.2256	6.9121	5.1458	0.3758	0.4972	0.2344
120	Co	5.2141	10.4171	5.2504	0.3750	0.7493	0.2392
121	Co	8.6889	0.0692	5.3123	0.6249	0.0050	0.2420
122	Co	8.6732	3.4858	5.3616	0.6238	0.2507	0.2442
123	Co	12.1417	0.0058	5.3416	0.8733	0.0004	0.2433
124	Co	12.1594	3.5160	5.2652	0.8746	0.2529	0.2399
125	Co	8.7131	6.9937	5.1798	0.6267	0.5030	0.2360
126	Co	8.7492	10.3347	5.1885	0.6293	0.7433	0.2364
127	Co	12.1793	6.9281	5.2027	0.8760	0.4983	0.2370
128	Co	12.1952	10.4161	5.2678	0.8771	0.7492	0.2400
129	O	5.8300	6.1369	6.7725	0.4193	0.4414	0.3085
130	O	2.6313	7.7045	6.7991	0.1893	0.5541	0.3097
131	O	4.7606	7.7135	8.8584	0.3424	0.5548	0.4035
132	O	6.0194	9.6158	6.8367	0.4329	0.6916	0.3114
133	O	7.7652	9.5820	9.1229	0.5585	0.6892	0.4156
134	O	4.9026	11.2681	8.9027	0.3526	0.8105	0.4056
135	O	8.5197	11.6309	6.4264	0.6128	0.8366	0.2928
136	Ce	3.7974	9.6896	8.0650	0.2731	0.6969	0.3674
137	Ce	3.7715	5.9636	7.7519	0.2713	0.4289	0.3531

138	Ce	7.0098	7.8378	8.1628	0.5042	0.5637	0.3719
139	Sm	6.9555	11.3532	8.2216	0.5003	0.8166	0.3745
140	C	6.8640	0.0561	6.3721	0.4937	0.0040	0.2903
141	O	6.9875	13.8711	7.6117	0.5026	0.9977	0.3467
142	O	3.5216	13.8489	6.1837	0.2533	0.9961	0.2817
143	O	10.4093	0.0378	6.1961	0.7487	0.0027	0.2823
144	O	13.9018	0.0691	6.1980	0.9999	0.0050	0.2823
145	O	3.4388	3.8168	6.3914	0.2473	0.2745	0.2912
146	O	6.9300	3.4194	6.2216	0.4984	0.2459	0.2834
147	O	0.0368	10.3615	6.2210	0.0026	0.7453	0.2834
148	O	10.4760	3.5613	6.2136	0.7535	0.2561	0.2831

/db/jmorales/CoNi-alloy/Profiles/CoNi(111)/CO-O

a =	9.831199646
b =	9.831199645902503
c =	21.020299912
alpha =	90.0
beta =	90.0
gamma =	120.0

	Atom	X	Y	Z	X	Y	Z
1	Ni	0.0000	0.0000	0.0000	0.0000	0.0000	0.0000
2	Ni	-1.2289	2.1285	0.0000	0.0000	0.2500	0.0000
3	Ni	-2.4578	4.2570	0.0000	-0.0000	0.5000	0.0000
4	Ni	-3.6867	6.3856	0.0000	-0.0000	0.7500	0.0000
5	Ni	4.9156	0.0000	0.0000	0.5000	0.0000	0.0000
6	Ni	3.6867	2.1285	0.0000	0.5000	0.2500	0.0000
7	Ni	2.4578	4.2570	0.0000	0.5000	0.5000	0.0000
8	Ni	1.2289	6.3856	0.0000	0.5000	0.7500	0.0000
9	Ni	2.4578	1.4190	2.0068	0.3333	0.1667	0.0955
10	Ni	1.2289	3.5475	2.0068	0.3333	0.4167	0.0955
11	Ni	0.0000	5.6760	2.0068	0.3333	0.6667	0.0955
12	Ni	-1.2289	7.8046	2.0068	0.3333	0.9167	0.0955
13	Ni	7.3734	1.4190	2.0068	0.8333	0.1667	0.0955
14	Ni	6.1445	3.5475	2.0068	0.8333	0.4167	0.0955
15	Ni	4.9156	5.6760	2.0068	0.8333	0.6667	0.0955
16	Ni	3.6867	7.8046	2.0068	0.8333	0.9167	0.0955
17	Ni	0.0361	2.8452	4.0075	0.1708	0.3342	0.1906
18	Ni	1.2632	0.7242	4.0316	0.1710	0.0851	0.1918
19	Ni	-2.4268	7.0981	4.0100	0.1700	0.8337	0.1908
20	Ni	-1.2000	4.9729	4.0046	0.1700	0.5841	0.1905
21	Ni	4.9286	2.8378	4.0330	0.6680	0.3333	0.1919
22	Ni	6.1753	0.7187	4.0057	0.6703	0.0844	0.1906
23	Ni	2.4859	7.0994	4.0200	0.6698	0.8338	0.1912
24	Ni	3.7096	4.9665	4.0163	0.6690	0.5833	0.1911
25	Ni	-4.9004	8.5076	6.0130	0.0012	0.9992	0.2861
26	Ni	-1.1845	2.1427	6.0167	0.0053	0.2517	0.2862
27	Ni	-2.4031	4.2660	6.0177	0.0061	0.5011	0.2863
28	Ni	-3.6385	6.3890	6.0135	0.0051	0.7504	0.2861
29	Ni	0.0878	8.5102	6.0084	0.5087	0.9995	0.2858
30	Ni	3.7779	2.1572	6.1123	0.5110	0.2534	0.2908
31	Ni	2.5089	4.3212	6.0818	0.5090	0.5075	0.2893
32	Ni	1.2870	6.4135	6.0358	0.5076	0.7533	0.2871
33	Co	1.2289	2.1285	0.0000	0.2500	0.2500	0.0000
34	Co	2.4578	0.0000	0.0000	0.2500	0.0000	0.0000
35	Co	-1.2289	6.3856	0.0000	0.2500	0.7500	0.0000
36	Co	0.0000	4.2570	0.0000	0.2500	0.5000	0.0000
37	Co	6.1445	2.1285	0.0000	0.7500	0.2500	0.0000
38	Co	7.3734	0.0000	0.0000	0.7500	0.0000	0.0000
39	Co	3.6867	6.3856	0.0000	0.7500	0.7500	0.0000
40	Co	4.9156	4.2570	0.0000	0.7500	0.5000	0.0000
41	Co	-1.2289	3.5475	2.0068	0.0833	0.4167	0.0955

42	Co	0.0000	1.4190	2.0068	0.0833	0.1667	0.0955
43	Co	-3.6867	7.8046	2.0068	0.0833	0.9167	0.0955
44	Co	-2.4578	5.6760	2.0068	0.0833	0.6667	0.0955
45	Co	3.6867	3.5475	2.0068	0.5833	0.4167	0.0955
46	Co	4.9156	1.4190	2.0068	0.5833	0.1667	0.0955
47	Co	1.2289	7.8046	2.0068	0.5833	0.9167	0.0955
48	Co	2.4578	5.6760	2.0068	0.5833	0.6667	0.0955
49	Co	3.7088	0.7114	4.0626	0.4190	0.0836	0.1933
50	Co	2.4738	2.8442	4.0747	0.4187	0.3341	0.1938
51	Co	1.2545	4.9685	4.0383	0.4194	0.5836	0.1921
52	Co	0.0203	7.0933	4.0508	0.4186	0.8331	0.1927
53	Co	8.6368	0.7234	4.0468	0.9210	0.0850	0.1925
54	Co	7.4026	2.8474	4.0462	0.9202	0.3344	0.1925
55	Co	6.1635	4.9676	4.0496	0.9187	0.5835	0.1927
56	Co	4.9503	7.0988	4.0440	0.9204	0.8338	0.1924
57	Co	1.2705	2.1400	6.1155	0.2549	0.2514	0.2909
58	Co	-2.3812	8.4894	6.0999	0.2563	0.9971	0.2902
59	Co	-1.1728	6.4120	6.0109	0.2573	0.7531	0.2860
60	Co	0.0484	4.2808	6.0574	0.2563	0.5028	0.2882
61	Co	6.2231	2.1499	6.0041	0.7592	0.2525	0.2856
62	Co	7.4438	0.0262	6.0129	0.7587	0.0031	0.2861
63	Co	3.7640	6.4140	6.0082	0.7595	0.7533	0.2858
64	Co	4.9848	4.2853	6.0055	0.7587	0.5033	0.2857
65	O	1.2895	4.3970	8.7038	0.3894	0.5164	0.4141
66	C	1.1251	4.3779	7.5326	0.3715	0.5142	0.3583
67	O	2.5144	1.3822	7.2684	0.3369	0.1623	0.3458

/db/jmorales/CoNi-alloy/Profiles/CoNi-Ce_3SmO_7/O-coverage/M-CO2-O-coverage/M-CO_2-CoNi-Ce_3SmO_7-5O

a =			13.9033384264			
b =			13.9033384264			
c =			21.9516692133			
alpha =			90.0			
beta =			90.0			
gamma =			90.0			
Atom	X	Y	Z	X	Y	Z
1 Ni	0.0000	0.0000	0.0000	0.0000	0.0000	0.0000
2 Ni	0.0000	3.4758	0.0000	0.0000	0.2500	0.0000
3 Ni	3.4758	0.0000	0.0000	0.2500	0.0000	0.0000
4 Ni	3.4758	3.4758	0.0000	0.2500	0.2500	0.0000
5 Ni	0.0000	6.9517	0.0000	0.0000	0.5000	0.0000
6 Ni	0.0000	10.4275	0.0000	0.0000	0.7500	0.0000
7 Ni	3.4758	6.9517	0.0000	0.2500	0.5000	0.0000
8 Ni	3.4758	10.4275	0.0000	0.2500	0.7500	0.0000
9 Ni	6.9517	0.0000	0.0000	0.5000	0.0000	0.0000
10 Ni	6.9517	3.4758	0.0000	0.5000	0.2500	0.0000
11 Ni	10.4275	0.0000	0.0000	0.7500	0.0000	0.0000
12 Ni	10.4275	3.4758	0.0000	0.7500	0.2500	0.0000
13 Ni	6.9517	6.9517	0.0000	0.5000	0.5000	0.0000
14 Ni	6.9517	10.4275	0.0000	0.5000	0.7500	0.0000
15 Ni	10.4275	6.9517	0.0000	0.7500	0.5000	0.0000
16 Ni	10.4275	10.4275	0.0000	0.7500	0.7500	0.0000
17 Ni	0.0000	1.7379	1.7379	0.0000	0.1250	0.0792
18 Ni	0.0000	5.2138	1.7379	0.0000	0.3750	0.0792
19 Ni	3.4758	1.7379	1.7379	0.2500	0.1250	0.0792
20 Ni	3.4758	5.2138	1.7379	0.2500	0.3750	0.0792
21 Ni	0.0000	8.6896	1.7379	0.0000	0.6250	0.0792
22 Ni	0.0000	12.1654	1.7379	0.0000	0.8750	0.0792
23 Ni	3.4758	8.6896	1.7379	0.2500	0.6250	0.0792
24 Ni	3.4758	12.1654	1.7379	0.2500	0.8750	0.0792
25 Ni	6.9517	1.7379	1.7379	0.5000	0.1250	0.0792
26 Ni	6.9517	5.2138	1.7379	0.5000	0.3750	0.0792
27 Ni	10.4275	1.7379	1.7379	0.7500	0.1250	0.0792
28 Ni	10.4275	5.2138	1.7379	0.7500	0.3750	0.0792
29 Ni	6.9517	8.6896	1.7379	0.5000	0.6250	0.0792
30 Ni	6.9517	12.1654	1.7379	0.5000	0.8750	0.0792
31 Ni	10.4275	8.6896	1.7379	0.7500	0.6250	0.0792
32 Ni	10.4275	12.1654	1.7379	0.7500	0.8750	0.0792
33 Ni	13.8987	13.8978	3.4575	0.9997	0.9996	0.1575
34 Ni	0.0205	3.4626	3.5248	0.0015	0.2490	0.1606
35 Ni	3.4622	0.0136	3.5149	0.2490	0.0010	0.1601
36 Ni	3.4868	3.4208	3.4577	0.2508	0.2460	0.1575
37 Ni	13.8973	6.9600	3.5174	0.9996	0.5006	0.1602
38 Ni	13.8776	10.4408	3.4418	0.9982	0.7510	0.1568
39 Ni	3.4706	6.9642	3.4813	0.2496	0.5009	0.1586

40	Ni	3.4781	10.3892	3.5079	0.2502	0.7472	0.1598
41	Ni	6.9476	0.0142	3.5088	0.4997	0.0010	0.1598
42	Ni	6.9384	3.4764	3.4521	0.4990	0.2500	0.1573
43	Ni	10.4371	0.0167	3.4543	0.7507	0.0012	0.1574
44	Ni	10.4142	3.4644	3.5160	0.7490	0.2492	0.1602
45	Ni	6.9675	6.9684	3.5114	0.5011	0.5012	0.1600
46	Ni	6.9692	10.4348	3.4785	0.5013	0.7505	0.1585
47	Ni	10.4265	6.9383	3.4892	0.7499	0.4990	0.1589
48	Ni	10.4492	10.4473	3.5212	0.7516	0.7514	0.1604
49	Ni	0.0628	1.7779	5.2720	0.0045	0.1279	0.2402
50	Ni	0.0072	5.2125	5.2073	0.0005	0.3749	0.2372
51	Ni	3.4514	1.7297	5.3252	0.2482	0.1244	0.2426
52	Ni	3.4713	5.2177	5.0558	0.2497	0.3753	0.2303
53	Ni	13.8533	8.6486	5.2292	0.9964	0.6221	0.2382
54	Ni	13.8709	12.1588	5.2674	0.9977	0.8745	0.2400
55	Ni	3.4390	8.6871	5.2437	0.2474	0.6248	0.2389
56	Ni	3.4587	12.1024	5.1803	0.2488	0.8705	0.2360
57	Ni	6.9530	1.7159	5.2396	0.5001	0.1234	0.2387
58	Ni	6.9888	5.2576	5.3811	0.5027	0.3782	0.2451
59	Ni	10.4063	1.7600	5.2493	0.7485	0.1266	0.2391
60	Ni	10.4020	5.2021	5.2051	0.7482	0.3742	0.2371
61	Ni	7.0051	8.7194	5.2432	0.5038	0.6271	0.2389
62	Ni	6.9181	12.2098	5.1761	0.4976	0.8782	0.2358
63	Ni	10.4213	8.6924	5.2023	0.7496	0.6252	0.2370
64	Ni	10.5054	12.1846	5.2605	0.7556	0.8764	0.2396
65	Co	1.7379	1.7379	0.0000	0.1250	0.1250	0.0000
66	Co	1.7379	5.2138	0.0000	0.1250	0.3750	0.0000
67	Co	5.2138	1.7379	0.0000	0.3750	0.1250	0.0000
68	Co	5.2138	5.2138	0.0000	0.3750	0.3750	0.0000
69	Co	1.7379	8.6896	0.0000	0.1250	0.6250	0.0000
70	Co	1.7379	12.1654	0.0000	0.1250	0.8750	0.0000
71	Co	5.2138	8.6896	0.0000	0.3750	0.6250	0.0000
72	Co	5.2138	12.1654	0.0000	0.3750	0.8750	0.0000
73	Co	8.6896	1.7379	0.0000	0.6250	0.1250	0.0000
74	Co	8.6896	5.2138	0.0000	0.6250	0.3750	0.0000
75	Co	12.1654	1.7379	0.0000	0.8750	0.1250	0.0000
76	Co	12.1654	5.2138	0.0000	0.8750	0.3750	0.0000
77	Co	8.6896	8.6896	0.0000	0.6250	0.6250	0.0000
78	Co	8.6896	12.1654	0.0000	0.6250	0.8750	0.0000
79	Co	12.1654	8.6896	0.0000	0.8750	0.6250	0.0000
80	Co	12.1654	12.1654	0.0000	0.8750	0.8750	0.0000
81	Co	1.7379	0.0000	1.7379	0.1250	0.0000	0.0792
82	Co	1.7379	3.4758	1.7379	0.1250	0.2500	0.0792
83	Co	5.2138	0.0000	1.7379	0.3750	0.0000	0.0792
84	Co	5.2138	3.4758	1.7379	0.3750	0.2500	0.0792
85	Co	1.7379	6.9517	1.7379	0.1250	0.5000	0.0792
86	Co	1.7379	10.4275	1.7379	0.1250	0.7500	0.0792
87	Co	5.2138	6.9517	1.7379	0.3750	0.5000	0.0792
88	Co	5.2138	10.4275	1.7379	0.3750	0.7500	0.0792

89	Co	8.6896	0.0000	1.7379	0.6250	0.0000	0.0792
90	Co	8.6896	3.4758	1.7379	0.6250	0.2500	0.0792
91	Co	12.1654	0.0000	1.7379	0.8750	0.0000	0.0792
92	Co	12.1654	3.4758	1.7379	0.8750	0.2500	0.0792
93	Co	8.6896	6.9517	1.7379	0.6250	0.5000	0.0792
94	Co	8.6896	10.4275	1.7379	0.6250	0.7500	0.0792
95	Co	12.1654	6.9517	1.7379	0.8750	0.5000	0.0792
96	Co	12.1654	10.4275	1.7379	0.8750	0.7500	0.0792
97	Co	1.7462	1.7411	3.5610	0.1256	0.1252	0.1622
98	Co	1.7101	5.2276	3.5096	0.1230	0.3760	0.1599
99	Co	5.2071	1.7316	3.5659	0.3745	0.1245	0.1624
100	Co	5.2467	5.1928	3.4968	0.3774	0.3735	0.1593
101	Co	1.7211	8.6866	3.5174	0.1238	0.6248	0.1602
102	Co	1.7280	12.1627	3.5266	0.1243	0.8748	0.1607
103	Co	5.2054	8.6945	3.5590	0.3744	0.6254	0.1621
104	Co	5.2029	12.1499	3.5457	0.3742	0.8739	0.1615
105	Co	8.6853	1.7351	3.5409	0.6247	0.1248	0.1613
106	Co	8.6644	5.1982	3.5634	0.6232	0.3739	0.1623
107	Co	12.1803	1.7480	3.5572	0.8761	0.1257	0.1620
108	Co	12.1639	5.1977	3.5562	0.8749	0.3738	0.1620
109	Co	8.6981	8.7063	3.5434	0.6256	0.6262	0.1614
110	Co	8.7138	12.1900	3.4809	0.6267	0.8768	0.1586
111	Co	12.1484	8.6797	3.5410	0.8738	0.6243	0.1613
112	Co	12.1575	12.1832	3.5518	0.8744	0.8763	0.1618
113	Co	1.7380	13.8186	5.3266	0.1250	0.9939	0.2427
114	Co	1.7863	3.5272	5.2833	0.1285	0.2537	0.2407
115	Co	5.2086	13.8748	5.2633	0.3746	0.9979	0.2398
116	Co	5.1974	3.4763	5.3256	0.3738	0.2500	0.2426
117	Co	1.7111	6.9661	5.2888	0.1231	0.5010	0.2409
118	Co	1.6613	10.4185	5.1998	0.1195	0.7493	0.2369
119	Co	5.2336	6.9163	5.1506	0.3764	0.4975	0.2346
120	Co	5.2244	10.4061	5.2332	0.3758	0.7485	0.2384
121	Co	8.6989	13.8511	5.3258	0.6257	0.9962	0.2426
122	Co	8.6579	3.4675	5.2637	0.6227	0.2494	0.2398
123	Co	12.2064	0.0633	5.3685	0.8779	0.0046	0.2446
124	Co	12.1691	3.4857	5.2057	0.8753	0.2507	0.2371
125	Co	8.7330	6.9774	5.1887	0.6281	0.5019	0.2364
126	Co	8.7491	10.4949	5.2226	0.6293	0.7548	0.2379
127	Co	12.1335	6.9221	5.2121	0.8727	0.4979	0.2374
128	Co	12.1854	10.4005	5.2709	0.8764	0.7481	0.2401
129	O	5.7480	6.0759	6.7740	0.4134	0.4370	0.3086
130	O	2.5728	7.8323	6.7833	0.1850	0.5633	0.3090
131	O	4.7228	7.8063	8.8039	0.3397	0.5615	0.4011
132	O	6.1150	9.6564	6.8406	0.4398	0.6945	0.3116
133	O	8.0552	9.3548	8.9891	0.5794	0.6728	0.4095
134	O	5.3092	11.1860	9.0965	0.3819	0.8046	0.4144
135	O	8.4049	12.0162	6.3749	0.6045	0.8643	0.2904
136	Ce	3.9233	9.9685	8.0850	0.2822	0.7170	0.3683
137	Ce	3.6605	6.0980	7.7479	0.2633	0.4386	0.3530

138	Ce	6.9545	7.7230	8.1023	0.5002	0.5555	0.3691
139	Sm	7.2792	11.1682	8.2618	0.5236	0.8033	0.3764
140	C	3.1378	13.2525	6.7384	0.2257	0.9532	0.3070
141	O	3.9768	0.4172	6.8125	0.2860	0.0300	0.3103
142	O	2.8782	12.5241	7.7288	0.2070	0.9008	0.3521
143	O	10.4773	0.0940	6.2061	0.7536	0.0068	0.2827
144	O	0.0399	0.0873	6.2186	0.0029	0.0063	0.2833
145	O	0.0155	10.3557	6.2071	0.0011	0.7448	0.2828
146	O	6.9832	3.3702	6.2217	0.5023	0.2424	0.2834
147	O	3.4432	3.8025	6.3756	0.2477	0.2735	0.2904

/db/jmorales/CoNi-alloy/Profiles/Ni-Ce_3SmO_7/O-coverage/TS-O-coverage/TS-Ni-Ce_3SmO_7-4O

a =			13.901983737			
b =			13.901983737			
c =			21.950991869			
alpha =			90.0			
beta =			90.0			
gamma =			90.0			
Atom	X	Y	Z	X	Y	Z
1 Ni	0.0000	0.0000	0.0000	0.0000	0.0000	0.0000
2 Ni	1.7378	1.7378	0.0000	0.1250	0.1250	0.0000
3 Ni	0.0000	3.4755	0.0000	0.0000	0.2500	0.0000
4 Ni	1.7378	5.2132	0.0000	0.1250	0.3750	0.0000
5 Ni	3.4755	0.0000	0.0000	0.2500	0.0000	0.0000
6 Ni	5.2132	1.7378	0.0000	0.3750	0.1250	0.0000
7 Ni	3.4755	3.4755	0.0000	0.2500	0.2500	0.0000
8 Ni	5.2132	5.2132	0.0000	0.3750	0.3750	0.0000
9 Ni	0.0000	6.9510	0.0000	0.0000	0.5000	0.0000
10 Ni	1.7378	8.6887	0.0000	0.1250	0.6250	0.0000
11 Ni	0.0000	10.4265	0.0000	0.0000	0.7500	0.0000
12 Ni	1.7378	12.1642	0.0000	0.1250	0.8750	0.0000
13 Ni	3.4755	6.9510	0.0000	0.2500	0.5000	0.0000
14 Ni	5.2132	8.6887	0.0000	0.3750	0.6250	0.0000
15 Ni	3.4755	10.4265	0.0000	0.2500	0.7500	0.0000
16 Ni	5.2132	12.1642	0.0000	0.3750	0.8750	0.0000
17 Ni	6.9510	0.0000	0.0000	0.5000	0.0000	0.0000
18 Ni	8.6887	1.7378	0.0000	0.6250	0.1250	0.0000
19 Ni	6.9510	3.4755	0.0000	0.5000	0.2500	0.0000
20 Ni	8.6887	5.2132	0.0000	0.6250	0.3750	0.0000
21 Ni	10.4265	0.0000	0.0000	0.7500	0.0000	0.0000
22 Ni	12.1642	1.7378	0.0000	0.8750	0.1250	0.0000
23 Ni	10.4265	3.4755	0.0000	0.7500	0.2500	0.0000
24 Ni	12.1642	5.2132	0.0000	0.8750	0.3750	0.0000
25 Ni	6.9510	6.9510	0.0000	0.5000	0.5000	0.0000
26 Ni	8.6887	8.6887	0.0000	0.6250	0.6250	0.0000
27 Ni	6.9510	10.4265	0.0000	0.5000	0.7500	0.0000
28 Ni	8.6887	12.1642	0.0000	0.6250	0.8750	0.0000
29 Ni	10.4265	6.9510	0.0000	0.7500	0.5000	0.0000
30 Ni	12.1642	8.6887	0.0000	0.8750	0.6250	0.0000
31 Ni	10.4265	10.4265	0.0000	0.7500	0.7500	0.0000
32 Ni	12.1642	12.1642	0.0000	0.8750	0.8750	0.0000
33 Ni	0.0000	1.7378	1.7378	0.0000	0.1250	0.0792
34 Ni	1.7378	0.0000	1.7378	0.1250	0.0000	0.0792
35 Ni	0.0000	5.2132	1.7378	0.0000	0.3750	0.0792
36 Ni	1.7378	3.4755	1.7378	0.1250	0.2500	0.0792
37 Ni	3.4755	1.7378	1.7378	0.2500	0.1250	0.0792
38 Ni	5.2132	0.0000	1.7378	0.3750	0.0000	0.0792
39 Ni	3.4755	5.2132	1.7378	0.2500	0.3750	0.0792

40	Ni	5.2132	3.4755	1.7378	0.3750	0.2500	0.0792
41	Ni	0.0000	8.6887	1.7378	0.0000	0.6250	0.0792
42	Ni	1.7378	6.9510	1.7378	0.1250	0.5000	0.0792
43	Ni	0.0000	12.1642	1.7378	0.0000	0.8750	0.0792
44	Ni	1.7378	10.4265	1.7378	0.1250	0.7500	0.0792
45	Ni	3.4755	8.6887	1.7378	0.2500	0.6250	0.0792
46	Ni	5.2132	6.9510	1.7378	0.3750	0.5000	0.0792
47	Ni	3.4755	12.1642	1.7378	0.2500	0.8750	0.0792
48	Ni	5.2132	10.4265	1.7378	0.3750	0.7500	0.0792
49	Ni	6.9510	1.7378	1.7378	0.5000	0.1250	0.0792
50	Ni	8.6887	0.0000	1.7378	0.6250	0.0000	0.0792
51	Ni	6.9510	5.2132	1.7378	0.5000	0.3750	0.0792
52	Ni	8.6887	3.4755	1.7378	0.6250	0.2500	0.0792
53	Ni	10.4265	1.7378	1.7378	0.7500	0.1250	0.0792
54	Ni	12.1642	0.0000	1.7378	0.8750	0.0000	0.0792
55	Ni	10.4265	5.2132	1.7378	0.7500	0.3750	0.0792
56	Ni	12.1642	3.4755	1.7378	0.8750	0.2500	0.0792
57	Ni	6.9510	8.6887	1.7378	0.5000	0.6250	0.0792
58	Ni	8.6887	6.9510	1.7378	0.6250	0.5000	0.0792
59	Ni	6.9510	12.1642	1.7378	0.5000	0.8750	0.0792
60	Ni	8.6887	10.4265	1.7378	0.6250	0.7500	0.0792
61	Ni	10.4265	8.6887	1.7378	0.7500	0.6250	0.0792
62	Ni	12.1642	6.9510	1.7378	0.8750	0.5000	0.0792
63	Ni	10.4265	12.1642	1.7378	0.7500	0.8750	0.0792
64	Ni	12.1642	10.4265	1.7378	0.8750	0.7500	0.0792
65	Ni	0.0056	0.0140	3.5011	0.0004	0.0010	0.1595
66	Ni	1.7348	1.7744	3.4565	0.1248	0.1276	0.1575
67	Ni	13.8772	3.4859	3.5120	0.9982	0.2508	0.1600
68	Ni	1.6852	5.1988	3.4564	0.1212	0.3740	0.1575
69	Ni	3.4626	13.8963	3.5065	0.2491	0.9996	0.1597
70	Ni	5.2164	1.7430	3.5470	0.3752	0.1254	0.1616
71	Ni	3.4990	3.4511	3.4767	0.2517	0.2482	0.1584
72	Ni	5.2265	5.2265	3.4692	0.3760	0.3760	0.1580
73	Ni	13.8996	6.9372	3.5106	0.9998	0.4990	0.1599
74	Ni	1.7492	8.6548	3.5368	0.1258	0.6226	0.1611
75	Ni	13.8857	10.4160	3.5179	0.9988	0.7492	0.1603
76	Ni	1.7196	12.1650	3.5243	0.1237	0.8751	0.1606
77	Ni	3.4719	6.9883	3.4655	0.2497	0.5027	0.1579
78	Ni	5.2287	8.7034	3.5005	0.3761	0.6261	0.1595
79	Ni	3.4616	10.3897	3.5174	0.2490	0.7474	0.1602
80	Ni	5.2079	12.1486	3.4892	0.3746	0.8739	0.1590
81	Ni	6.9442	13.8928	3.4966	0.4995	0.9993	0.1593
82	Ni	8.6824	1.7245	3.4209	0.6245	0.1240	0.1558
83	Ni	6.9428	3.4705	3.4886	0.4994	0.2496	0.1589
84	Ni	8.6543	5.2224	3.5199	0.6225	0.3757	0.1604
85	Ni	10.4464	0.0077	3.5029	0.7514	0.0006	0.1596
86	Ni	12.1689	1.7483	3.5420	0.8753	0.1258	0.1614
87	Ni	10.4182	3.4702	3.4832	0.7494	0.2496	0.1587
88	Ni	12.1589	5.2147	3.4930	0.8746	0.3751	0.1591

89	Ni	6.9378	6.9448	3.5117	0.4990	0.4996	0.1600
90	Ni	8.6959	8.6743	3.4730	0.6255	0.6240	0.1582
91	Ni	6.9915	10.4184	3.5201	0.5029	0.7494	0.1604
92	Ni	8.7174	12.1905	3.5210	0.6271	0.8769	0.1604
93	Ni	10.4135	6.9349	3.5087	0.7491	0.4988	0.1598
94	Ni	12.1770	8.6631	3.5104	0.8759	0.6232	0.1599
95	Ni	10.4513	10.4346	3.4453	0.7518	0.7506	0.1570
96	Ni	12.1746	12.1630	3.4378	0.8757	0.8749	0.1566
97	Ni	0.0447	1.8017	5.2790	0.0032	0.1296	0.2405
98	Ni	1.7214	13.8828	5.2963	0.1238	0.9986	0.2413
99	Ni	13.8449	5.1554	5.3060	0.9959	0.3708	0.2417
100	Ni	1.7586	3.5746	5.3770	0.1265	0.2571	0.2450
101	Ni	3.4466	1.7425	5.2851	0.2479	0.1253	0.2408
102	Ni	5.2032	13.8808	5.2034	0.3743	0.9985	0.2370
103	Ni	3.5101	5.2093	5.0377	0.2525	0.3747	0.2295
104	Ni	5.2150	3.4421	5.3350	0.3751	0.2476	0.2430
105	Ni	0.0193	8.6853	5.2026	0.0014	0.6247	0.2370
106	Ni	1.7181	6.8763	5.2760	0.1236	0.4946	0.2404
107	Ni	13.8874	12.1412	5.2753	0.9989	0.8733	0.2403
108	Ni	1.7253	10.4250	5.2054	0.1241	0.7499	0.2371
109	Ni	3.4438	8.7246	5.3614	0.2477	0.6276	0.2442
110	Ni	5.2080	6.9811	5.2070	0.3746	0.5022	0.2372
111	Ni	3.4680	12.1359	5.2072	0.2495	0.8730	0.2372
112	Ni	5.2298	10.4281	5.1914	0.3762	0.7501	0.2365
113	Ni	6.9586	1.7004	5.2673	0.5005	0.1223	0.2400
114	Ni	8.6792	13.8870	5.2870	0.6243	0.9989	0.2409
115	Ni	6.9251	5.2047	5.2698	0.4981	0.3744	0.2401
116	Ni	8.6838	3.4517	5.2232	0.6246	0.2483	0.2380
117	Ni	10.4030	1.7060	5.2666	0.7483	0.1227	0.2399
118	Ni	12.2182	0.0592	5.2868	0.8789	0.0043	0.2408
119	Ni	10.3767	5.2004	5.2398	0.7464	0.3741	0.2387
120	Ni	12.0811	3.4537	5.3040	0.8690	0.2484	0.2416
121	Ni	6.9904	8.7387	5.2545	0.5028	0.6286	0.2394
122	Ni	8.6853	6.9212	5.2839	0.6248	0.4979	0.2407
123	Ni	6.9519	12.1494	5.2173	0.5001	0.8739	0.2377
124	Ni	8.7723	10.4840	5.2424	0.6310	0.7541	0.2388
125	Ni	10.4553	8.6171	5.1802	0.7521	0.6198	0.2360
126	Ni	12.1600	6.9535	5.3375	0.8747	0.5002	0.2432
127	Ni	10.4914	12.2539	5.2947	0.7547	0.8815	0.2412
128	Ni	12.1234	10.4115	5.3621	0.8721	0.7489	0.2443
129	O	6.0550	4.4117	6.8217	0.4356	0.3173	0.3108
130	O	4.1975	7.6226	6.7798	0.3019	0.5483	0.3089
131	O	5.9991	6.5978	8.7756	0.4315	0.4746	0.3998
132	O	7.8491	7.8482	6.8417	0.5646	0.5645	0.3117
133	O	10.3339	10.1110	6.3553	0.7433	0.7273	0.2895
134	O	9.4180	6.9525	9.0065	0.6775	0.5001	0.4103
135	O	7.6057	9.7764	8.9534	0.5471	0.7032	0.4079
136	Ce	5.9657	8.7992	8.0106	0.4291	0.6329	0.3649
137	Ce	4.2908	5.5361	7.6652	0.3086	0.3982	0.3492

138	Ce	8.0634	5.7197	8.0554	0.5800	0.4114	0.3670
139	Sm	9.4247	8.9707	8.1823	0.6779	0.6453	0.3728
140	C	11.3515	4.5325	6.7430	0.8165	0.3260	0.3072
141	O	12.7243	5.7689	6.6384	0.9153	0.4150	0.3024
142	O	10.9757	4.3667	7.8817	0.7895	0.3141	0.3591
143	O	2.1916	5.2862	6.4066	0.1576	0.3802	0.2919
144	O	1.7347	1.6880	6.1904	0.1248	0.1214	0.2820
145	O	8.6702	1.7443	6.1592	0.6237	0.1255	0.2806
146	O	12.2566	12.3030	6.1943	0.8816	0.8850	0.2822

/db/jmorales/CoNi-alloy/Profiles/Co-CoNi(001)/CO-O

a =			6.951669213				
b =			6.951669213				
c =			21.951669213				
alpha =			90.0				
beta =			90.0				
gamma =			90.0				
	Atom	X	Y	Z	X	Y	Z
1	Ni	0.0000	0.0000	0.0000	0.0000	0.0000	0.0000
2	Ni	1.7379	1.7379	0.0000	0.2500	0.2500	0.0000
3	Ni	0.0000	3.4758	0.0000	0.0000	0.5000	0.0000
4	Ni	1.7379	5.2138	0.0000	0.2500	0.7500	0.0000
5	Ni	3.4758	0.0000	0.0000	0.5000	0.0000	0.0000
6	Ni	5.2138	1.7379	0.0000	0.7500	0.2500	0.0000
7	Ni	3.4758	3.4758	0.0000	0.5000	0.5000	0.0000
8	Ni	5.2138	5.2138	0.0000	0.7500	0.7500	0.0000
9	Ni	0.0007	6.9504	3.5197	0.0001	0.9998	0.1603
10	Ni	1.7398	1.7384	3.5496	0.2503	0.2501	0.1617
11	Ni	6.9477	3.4770	3.5331	0.9994	0.5002	0.1609
12	Ni	1.7375	5.2140	3.5093	0.2499	0.7500	0.1599
13	Ni	3.4748	0.0042	3.5331	0.4998	0.0006	0.1610
14	Ni	5.2166	1.7346	3.4694	0.7504	0.2495	0.1580
15	Ni	3.4796	3.4714	3.5224	0.5005	0.4994	0.1605
16	Ni	5.2134	5.2122	3.5498	0.7500	0.7498	0.1617
17	Co	1.7379	0.0000	1.7379	0.2500	0.0000	0.0792
18	Co	0.0000	1.7379	1.7379	0.0000	0.2500	0.0792
19	Co	1.7379	3.4758	1.7379	0.2500	0.5000	0.0792
20	Co	0.0000	5.2138	1.7379	0.0000	0.7500	0.0792
21	Co	5.2138	0.0000	1.7379	0.7500	0.0000	0.0792
22	Co	3.4758	1.7379	1.7379	0.5000	0.2500	0.0792
23	Co	5.2138	3.4758	1.7379	0.7500	0.5000	0.0792
24	Co	3.4758	5.2138	1.7379	0.5000	0.7500	0.0792
25	Co	1.7244	0.0131	5.2569	0.2481	0.0019	0.2395
26	Co	0.0395	1.7262	5.3214	0.0057	0.2483	0.2424
27	Co	1.7538	3.5040	5.2926	0.2523	0.5040	0.2411
28	Co	6.9387	5.2279	5.2575	0.9981	0.7520	0.2395
29	Co	5.2252	6.9125	5.3220	0.7516	0.9944	0.2424
30	Co	3.4598	1.7061	5.3164	0.4977	0.2454	0.2422
31	Co	5.2454	3.4921	5.3167	0.7545	0.5023	0.2422
32	Co	3.4477	5.1977	5.2931	0.4960	0.7477	0.2411
33	O	2.6382	4.3138	7.9694	0.3795	0.6205	0.3630
34	C	2.6175	4.3346	6.7861	0.3765	0.6235	0.3091
35	O	5.2225	1.7292	6.1916	0.7513	0.2487	0.2821

/db/jmorales/CoNi-alloy/Profiles/Ni(111)/O

a =	9.829999924
b =	9.82999923668225
c =	21.019699097
alpha =	90.0
beta =	90.0
gamma =	120.0

	Atom	X	Y	Z	X	Y	Z
1	Ni	0.0000	0.0000	0.0000	0.0000	0.0000	0.0000
2	Ni	-1.2288	2.1283	0.0000	0.0000	0.2500	0.0000
3	Ni	-2.4575	4.2565	0.0000	-0.0000	0.5000	0.0000
4	Ni	-3.6862	6.3848	0.0000	-0.0000	0.7500	0.0000
5	Ni	2.4575	0.0000	0.0000	0.2500	0.0000	0.0000
6	Ni	1.2288	2.1283	0.0000	0.2500	0.2500	0.0000
7	Ni	-0.0000	4.2565	0.0000	0.2500	0.5000	0.0000
8	Ni	-1.2288	6.3848	0.0000	0.2500	0.7500	0.0000
9	Ni	4.9150	0.0000	0.0000	0.5000	0.0000	0.0000
10	Ni	3.6862	2.1283	0.0000	0.5000	0.2500	0.0000
11	Ni	2.4575	4.2565	0.0000	0.5000	0.5000	0.0000
12	Ni	1.2288	6.3848	0.0000	0.5000	0.7500	0.0000
13	Ni	7.3725	0.0000	0.0000	0.7500	0.0000	0.0000
14	Ni	6.1438	2.1283	0.0000	0.7500	0.2500	0.0000
15	Ni	4.9150	4.2565	0.0000	0.7500	0.5000	0.0000
16	Ni	3.6862	6.3848	0.0000	0.7500	0.7500	0.0000
17	Ni	-0.0000	1.4188	2.0065	0.0833	0.1667	0.0955
18	Ni	-1.2288	3.5471	2.0065	0.0833	0.4167	0.0955
19	Ni	-2.4575	5.6754	2.0065	0.0833	0.6667	0.0955
20	Ni	-3.6863	7.8036	2.0065	0.0833	0.9167	0.0955
21	Ni	2.4575	1.4188	2.0065	0.3333	0.1667	0.0955
22	Ni	1.2287	3.5471	2.0065	0.3333	0.4167	0.0955
23	Ni	-0.0000	5.6754	2.0065	0.3333	0.6667	0.0955
24	Ni	-1.2288	7.8036	2.0065	0.3333	0.9167	0.0955
25	Ni	4.9150	1.4188	2.0065	0.5833	0.1667	0.0955
26	Ni	3.6862	3.5471	2.0065	0.5833	0.4167	0.0955
27	Ni	2.4575	5.6754	2.0065	0.5833	0.6667	0.0955
28	Ni	1.2287	7.8036	2.0065	0.5833	0.9167	0.0955
29	Ni	7.3725	1.4188	2.0065	0.8333	0.1667	0.0955
30	Ni	6.1437	3.5471	2.0065	0.8333	0.4167	0.0955
31	Ni	4.9150	5.6754	2.0065	0.8333	0.6667	0.0955
32	Ni	3.6862	7.8036	2.0065	0.8333	0.9167	0.0955
33	Ni	1.2276	0.7229	4.0194	0.1673	0.0849	0.1912
34	Ni	0.0037	2.8427	4.0152	0.1673	0.3339	0.1910
35	Ni	-1.2219	4.9634	4.0173	0.1672	0.5830	0.1911
36	Ni	-2.4579	7.0977	4.0124	0.1668	0.8337	0.1909
37	Ni	3.6824	0.7130	4.0062	0.4165	0.0837	0.1906
38	Ni	2.4534	2.8417	4.0124	0.4165	0.3338	0.1909
39	Ni	1.2287	4.9647	4.0109	0.4166	0.5832	0.1908
40	Ni	-0.0014	7.0923	4.0065	0.4164	0.8331	0.1906
41	Ni	6.1430	0.7109	4.0087	0.6667	0.0835	0.1907

42	Ni	4.9105	2.8428	4.0065	0.6665	0.3339	0.1906
43	Ni	3.6786	4.9645	4.0168	0.6658	0.5832	0.1911
44	Ni	2.4578	7.0918	4.0062	0.6666	0.8331	0.1906
45	Ni	8.6048	0.7124	4.0060	0.9172	0.0837	0.1906
46	Ni	7.3754	2.8410	4.0050	0.9172	0.3337	0.1905
47	Ni	6.1439	4.9678	4.0102	0.9168	0.5836	0.1908
48	Ni	4.9153	7.0959	4.0101	0.9168	0.8335	0.1908
49	Ni	0.0003	0.0042	6.0158	0.0003	0.0005	0.2862
50	Ni	8.5776	2.1179	6.0090	0.9970	0.2488	0.2859
51	Ni	-2.4580	4.2588	6.0144	0.0001	0.5003	0.2861
52	Ni	-3.6864	6.3889	6.0176	0.0002	0.7505	0.2863
53	Ni	2.4551	0.0077	6.0164	0.2502	0.0009	0.2862
54	Ni	1.2292	2.1364	6.0967	0.2505	0.2510	0.2900
55	Ni	-0.0038	4.2629	6.0884	0.2500	0.5007	0.2896
56	Ni	-1.2305	6.3889	6.0165	0.2501	0.7505	0.2862
57	Ni	4.9133	0.0023	6.0178	0.5000	0.0003	0.2863
58	Ni	3.7088	2.1201	6.0090	0.5018	0.2490	0.2859
59	Ni	2.4582	4.2658	6.0921	0.5006	0.5011	0.2898
60	Ni	1.2281	6.4164	6.0109	0.5018	0.7537	0.2860
61	Ni	7.3742	0.0026	6.0164	0.7503	0.0003	0.2862
62	Ni	6.1431	2.1336	6.0160	0.7502	0.2506	0.2862
63	Ni	4.9131	4.2612	6.0150	0.7501	0.5006	0.2862
64	Ni	3.6865	6.3886	6.0157	0.7503	0.7505	0.2862
65	O	2.4000	1.5000	7.1000	0.3323	0.1762	0.3378

/db/jmorales/CoNi-alloy/Profiles/Ni-Ce_3SmO_7/CO-O

a =			13.9019837372				
b =			13.9019837372				
c =			21.9509918687				
alpha =			90.0				
beta =			90.0				
gamma =			90.0				
	Atom	X	Y	Z	X	Y	Z
1	Ni	0.0000	0.0000	0.0000	0.0000	0.0000	0.0000
2	Ni	1.7377	1.7377	0.0000	0.1250	0.1250	0.0000
3	Ni	0.0000	3.4755	0.0000	0.0000	0.2500	0.0000
4	Ni	1.7377	5.2132	0.0000	0.1250	0.3750	0.0000
5	Ni	3.4755	0.0000	0.0000	0.2500	0.0000	0.0000
6	Ni	5.2132	1.7377	0.0000	0.3750	0.1250	0.0000
7	Ni	3.4755	3.4755	0.0000	0.2500	0.2500	0.0000
8	Ni	5.2132	5.2132	0.0000	0.3750	0.3750	0.0000
9	Ni	0.0000	6.9510	0.0000	0.0000	0.5000	0.0000
10	Ni	1.7377	8.6887	0.0000	0.1250	0.6250	0.0000
11	Ni	0.0000	10.4265	0.0000	0.0000	0.7500	0.0000
12	Ni	1.7377	12.1642	0.0000	0.1250	0.8750	0.0000
13	Ni	3.4755	6.9510	0.0000	0.2500	0.5000	0.0000
14	Ni	5.2132	8.6887	0.0000	0.3750	0.6250	0.0000
15	Ni	3.4755	10.4265	0.0000	0.2500	0.7500	0.0000
16	Ni	5.2132	12.1642	0.0000	0.3750	0.8750	0.0000
17	Ni	6.9510	0.0000	0.0000	0.5000	0.0000	0.0000
18	Ni	8.6887	1.7377	0.0000	0.6250	0.1250	0.0000
19	Ni	6.9510	3.4755	0.0000	0.5000	0.2500	0.0000
20	Ni	8.6887	5.2132	0.0000	0.6250	0.3750	0.0000
21	Ni	10.4265	0.0000	0.0000	0.7500	0.0000	0.0000
22	Ni	12.1642	1.7377	0.0000	0.8750	0.1250	0.0000
23	Ni	10.4265	3.4755	0.0000	0.7500	0.2500	0.0000
24	Ni	12.1642	5.2132	0.0000	0.8750	0.3750	0.0000
25	Ni	6.9510	6.9510	0.0000	0.5000	0.5000	0.0000
26	Ni	8.6887	8.6887	0.0000	0.6250	0.6250	0.0000
27	Ni	6.9510	10.4265	0.0000	0.5000	0.7500	0.0000
28	Ni	8.6887	12.1642	0.0000	0.6250	0.8750	0.0000
29	Ni	10.4265	6.9510	0.0000	0.7500	0.5000	0.0000
30	Ni	12.1642	8.6887	0.0000	0.8750	0.6250	0.0000
31	Ni	10.4265	10.4265	0.0000	0.7500	0.7500	0.0000
32	Ni	12.1642	12.1642	0.0000	0.8750	0.8750	0.0000
33	Ni	0.0000	1.7377	1.7377	0.0000	0.1250	0.0792
34	Ni	1.7377	0.0000	1.7377	0.1250	0.0000	0.0792
35	Ni	0.0000	5.2132	1.7377	0.0000	0.3750	0.0792
36	Ni	1.7377	3.4755	1.7377	0.1250	0.2500	0.0792
37	Ni	3.4755	1.7377	1.7377	0.2500	0.1250	0.0792
38	Ni	5.2132	0.0000	1.7377	0.3750	0.0000	0.0792
39	Ni	3.4755	5.2132	1.7377	0.2500	0.3750	0.0792
40	Ni	5.2132	3.4755	1.7377	0.3750	0.2500	0.0792

41	Ni	0.0000	8.6887	1.7377	0.0000	0.6250	0.0792
42	Ni	1.7377	6.9510	1.7377	0.1250	0.5000	0.0792
43	Ni	0.0000	12.1642	1.7377	0.0000	0.8750	0.0792
44	Ni	1.7377	10.4265	1.7377	0.1250	0.7500	0.0792
45	Ni	3.4755	8.6887	1.7377	0.2500	0.6250	0.0792
46	Ni	5.2132	6.9510	1.7377	0.3750	0.5000	0.0792
47	Ni	3.4755	12.1642	1.7377	0.2500	0.8750	0.0792
48	Ni	5.2132	10.4265	1.7377	0.3750	0.7500	0.0792
49	Ni	6.9510	1.7377	1.7377	0.5000	0.1250	0.0792
50	Ni	8.6887	0.0000	1.7377	0.6250	0.0000	0.0792
51	Ni	6.9510	5.2132	1.7377	0.5000	0.3750	0.0792
52	Ni	8.6887	3.4755	1.7377	0.6250	0.2500	0.0792
53	Ni	10.4265	1.7377	1.7377	0.7500	0.1250	0.0792
54	Ni	12.1642	0.0000	1.7377	0.8750	0.0000	0.0792
55	Ni	10.4265	5.2132	1.7377	0.7500	0.3750	0.0792
56	Ni	12.1642	3.4755	1.7377	0.8750	0.2500	0.0792
57	Ni	6.9510	8.6887	1.7377	0.5000	0.6250	0.0792
58	Ni	8.6887	6.9510	1.7377	0.6250	0.5000	0.0792
59	Ni	6.9510	12.1642	1.7377	0.5000	0.8750	0.0792
60	Ni	8.6887	10.4265	1.7377	0.6250	0.7500	0.0792
61	Ni	10.4265	8.6887	1.7377	0.7500	0.6250	0.0792
62	Ni	12.1642	6.9510	1.7377	0.8750	0.5000	0.0792
63	Ni	10.4265	12.1642	1.7377	0.7500	0.8750	0.0792
64	Ni	12.1642	10.4265	1.7377	0.8750	0.7500	0.0792
65	Ni	0.0030	0.0009	3.5052	0.0002	0.0001	0.1597
66	Ni	1.7194	1.7358	3.5198	0.1237	0.1249	0.1603
67	Ni	13.8928	3.4661	3.5056	0.9993	0.2493	0.1597
68	Ni	1.6962	5.2041	3.4759	0.1220	0.3743	0.1583
69	Ni	3.4786	0.0059	3.5113	0.2502	0.0004	0.1600
70	Ni	5.2229	1.7531	3.5317	0.3757	0.1261	0.1609
71	Ni	3.4910	3.4404	3.4971	0.2511	0.2475	0.1593
72	Ni	5.2153	5.2075	3.4940	0.3751	0.3746	0.1592
73	Ni	13.8980	6.9515	3.4996	0.9997	0.5000	0.1594
74	Ni	1.7434	8.6935	3.5188	0.1254	0.6253	0.1603
75	Ni	13.8829	10.4264	3.5230	0.9986	0.7500	0.1605
76	Ni	1.7451	12.1602	3.5054	0.1255	0.8747	0.1597
77	Ni	3.4609	6.9741	3.4821	0.2490	0.5017	0.1586
78	Ni	5.2202	8.7004	3.5031	0.3755	0.6258	0.1596
79	Ni	3.4725	10.4021	3.5288	0.2498	0.7482	0.1608
80	Ni	5.2106	12.1674	3.5018	0.3748	0.8752	0.1595
81	Ni	6.9480	0.0020	3.5041	0.4998	0.0001	0.1596
82	Ni	8.7160	1.7294	3.5144	0.6270	0.1244	0.1601
83	Ni	6.9531	3.4697	3.5072	0.5002	0.2496	0.1598
84	Ni	8.6777	5.2373	3.5272	0.6242	0.3767	0.1607
85	Ni	10.4338	13.8774	3.5212	0.7505	0.9982	0.1604
86	Ni	12.1592	1.7411	3.4392	0.8746	0.1252	0.1567
87	Ni	10.4285	3.4804	3.5005	0.7501	0.2504	0.1595
88	Ni	12.1607	5.1995	3.4791	0.8747	0.3740	0.1585
89	Ni	6.9310	6.9384	3.5159	0.4986	0.4991	0.1602

90	Ni	8.6916	8.6824	3.4741	0.6252	0.6245	0.1583
91	Ni	6.9718	10.4233	3.5125	0.5015	0.7498	0.1600
92	Ni	8.6896	12.1726	3.4944	0.6251	0.8756	0.1592
93	Ni	10.4151	6.9587	3.5166	0.7492	0.5006	0.1602
94	Ni	12.1802	8.6773	3.4913	0.8761	0.6242	0.1590
95	Ni	10.4427	10.4390	3.4330	0.7512	0.7509	0.1564
96	Ni	12.1709	12.1771	3.5156	0.8755	0.8759	0.1602
97	Ni	13.8872	1.7464	5.2841	0.9989	0.1256	0.2407
98	Ni	1.7388	13.8984	5.2183	0.1251	0.9997	0.2377
99	Ni	13.8637	5.2050	5.2685	0.9972	0.3744	0.2400
100	Ni	1.7352	3.4788	5.2107	0.1248	0.2502	0.2374
101	Ni	3.4649	1.7368	5.2270	0.2492	0.1249	0.2381
102	Ni	5.2126	0.0029	5.2251	0.3750	0.0002	0.2380
103	Ni	3.4246	5.1817	5.0772	0.2463	0.3727	0.2313
104	Ni	5.2036	3.4445	5.3727	0.3743	0.2478	0.2448
105	Ni	0.0083	8.6800	5.2159	0.0006	0.6244	0.2376
106	Ni	1.7078	6.9133	5.1932	0.1228	0.4973	0.2366
107	Ni	0.0045	12.1681	5.2140	0.0003	0.8753	0.2375
108	Ni	1.7232	10.4417	5.2246	0.1240	0.7511	0.2380
109	Ni	3.4328	8.7363	5.3797	0.2469	0.6284	0.2451
110	Ni	5.1636	6.9639	5.2196	0.3714	0.5009	0.2378
111	Ni	3.4800	12.1563	5.2252	0.2503	0.8744	0.2380
112	Ni	5.2306	10.4427	5.1959	0.3763	0.7512	0.2367
113	Ni	6.9875	1.7135	5.2083	0.5026	0.1233	0.2373
114	Ni	8.6927	13.8920	5.2142	0.6253	0.9993	0.2375
115	Ni	6.9429	5.1960	5.2663	0.4994	0.3738	0.2399
116	Ni	8.7195	3.4788	5.1922	0.6272	0.2502	0.2365
117	Ni	10.4396	1.7398	5.2860	0.7509	0.1251	0.2408
118	Ni	12.1676	13.8919	5.2820	0.8752	0.9993	0.2406
119	Ni	10.4511	5.2166	5.2731	0.7518	0.3752	0.2402
120	Ni	12.1540	3.4993	5.3576	0.8743	0.2517	0.2441
121	Ni	6.9673	8.7281	5.2367	0.5012	0.6278	0.2386
122	Ni	8.7135	6.9458	5.2816	0.6268	0.4996	0.2406
123	Ni	6.9478	12.1617	5.2192	0.4998	0.8748	0.2378
124	Ni	8.7124	10.4553	5.2121	0.6267	0.7521	0.2374
125	Ni	10.4633	8.6963	5.1789	0.7526	0.6255	0.2359
126	Ni	12.1624	6.9288	5.2613	0.8749	0.4984	0.2397
127	Ni	10.4405	12.1588	5.2752	0.7510	0.8746	0.2403
128	Ni	12.1819	10.4240	5.2810	0.8763	0.7498	0.2406
129	O	6.0930	4.4108	6.8206	0.4383	0.3173	0.3107
130	O	4.1584	7.6302	6.7706	0.2991	0.5489	0.3084
131	O	5.9606	6.6196	8.7781	0.4288	0.4762	0.3999
132	O	7.8133	7.8629	6.8036	0.5620	0.5656	0.3099
133	O	10.4143	10.3583	6.2739	0.7491	0.7451	0.2858
134	O	9.4778	7.0402	8.9302	0.6818	0.5064	0.4068
135	O	7.6462	9.8218	8.8857	0.5500	0.7065	0.4048
136	Ce	5.9247	8.8662	8.0134	0.4262	0.6378	0.3651
137	Ce	4.3207	5.5258	7.6935	0.3108	0.3975	0.3505
138	Ce	8.0375	5.7573	8.0557	0.5782	0.4141	0.3670

139 Sm	9.4158	9.0051	8.0590	0.6773	0.6478	0.3671
140 C	12.1609	5.2551	6.3688	0.8748	0.3780	0.2901
141 O	12.1746	5.2374	7.5889	0.8757	0.3767	0.3457
142 O	12.1672	1.7050	6.1563	0.8752	0.1226	0.2805

/db/jmorales/CoNi-alloy/Profiles/CoNi-Ce_3SmO_7/surface

a =				13.9033384264			
b =				13.9033384264			
c =				21.9516692133			
alpha =				90.0			
beta =				90.0			
gamma =				90.0			
	Atom	X	Y	Z	X	Y	Z
1	Ni	0.0000	0.0000	0.0000	0.0000	0.0000	0.0000
2	Ni	0.0000	3.4758	0.0000	0.0000	0.2500	0.0000
3	Ni	3.4758	0.0000	0.0000	0.2500	0.0000	0.0000
4	Ni	3.4758	3.4758	0.0000	0.2500	0.2500	0.0000
5	Ni	0.0000	6.9517	0.0000	0.0000	0.5000	0.0000
6	Ni	0.0000	10.4275	0.0000	0.0000	0.7500	0.0000
7	Ni	3.4758	6.9517	0.0000	0.2500	0.5000	0.0000
8	Ni	3.4758	10.4275	0.0000	0.2500	0.7500	0.0000
9	Ni	6.9517	0.0000	0.0000	0.5000	0.0000	0.0000
10	Ni	6.9517	3.4758	0.0000	0.5000	0.2500	0.0000
11	Ni	10.4275	0.0000	0.0000	0.7500	0.0000	0.0000
12	Ni	10.4275	3.4758	0.0000	0.7500	0.2500	0.0000
13	Ni	6.9517	6.9517	0.0000	0.5000	0.5000	0.0000
14	Ni	6.9517	10.4275	0.0000	0.5000	0.7500	0.0000
15	Ni	10.4275	6.9517	0.0000	0.7500	0.5000	0.0000
16	Ni	10.4275	10.4275	0.0000	0.7500	0.7500	0.0000
17	Ni	0.0000	1.7379	1.7379	0.0000	0.1250	0.0792
18	Ni	0.0000	5.2138	1.7379	0.0000	0.3750	0.0792
19	Ni	3.4758	1.7379	1.7379	0.2500	0.1250	0.0792
20	Ni	3.4758	5.2138	1.7379	0.2500	0.3750	0.0792
21	Ni	0.0000	8.6896	1.7379	0.0000	0.6250	0.0792
22	Ni	0.0000	12.1654	1.7379	0.0000	0.8750	0.0792
23	Ni	3.4758	8.6896	1.7379	0.2500	0.6250	0.0792
24	Ni	3.4758	12.1654	1.7379	0.2500	0.8750	0.0792
25	Ni	6.9517	1.7379	1.7379	0.5000	0.1250	0.0792
26	Ni	6.9517	5.2138	1.7379	0.5000	0.3750	0.0792
27	Ni	10.4275	1.7379	1.7379	0.7500	0.1250	0.0792
28	Ni	10.4275	5.2138	1.7379	0.7500	0.3750	0.0792
29	Ni	6.9517	8.6896	1.7379	0.5000	0.6250	0.0792
30	Ni	6.9517	12.1654	1.7379	0.5000	0.8750	0.0792
31	Ni	10.4275	8.6896	1.7379	0.7500	0.6250	0.0792
32	Ni	10.4275	12.1654	1.7379	0.7500	0.8750	0.0792
33	Ni	0.0017	13.8993	3.5056	0.0001	0.9997	0.1597
34	Ni	-0.0005	3.4745	3.5013	-0.0000	0.2499	0.1595
35	Ni	3.4776	13.8987	3.5048	0.2501	0.9997	0.1597
36	Ni	3.4635	3.4475	3.4659	0.2491	0.2480	0.1579
37	Ni	0.0014	6.9589	3.5153	0.0001	0.5005	0.1601
38	Ni	13.8915	10.4336	3.4942	0.9992	0.7504	0.1592
39	Ni	3.4751	6.9563	3.4948	0.2499	0.5003	0.1592
40	Ni	3.4924	10.4008	3.5239	0.2512	0.7481	0.1605

41	Ni	6.9554	0.0134	3.4919	0.5003	0.0010	0.1591
42	Ni	6.9569	3.4883	3.5182	0.5004	0.2509	0.1603
43	Ni	10.4250	0.0026	3.5064	0.7498	0.0002	0.1597
44	Ni	10.4298	3.4758	3.5031	0.7502	0.2500	0.1596
45	Ni	6.9667	6.9624	3.4984	0.5011	0.5008	0.1594
46	Ni	6.9659	10.4362	3.4798	0.5010	0.7506	0.1585
47	Ni	10.4360	6.9465	3.4903	0.7506	0.4996	0.1590
48	Ni	10.4309	10.4375	3.4982	0.7502	0.7507	0.1594
49	Ni	0.0077	1.7384	5.2082	0.0006	0.1250	0.2373
50	Ni	0.0027	5.2100	5.2171	0.0002	0.3747	0.2377
51	Ni	3.4599	1.7359	5.2077	0.2489	0.1249	0.2372
52	Ni	3.4482	5.1668	5.0822	0.2480	0.3716	0.2315
53	Ni	13.8791	8.7186	5.1948	0.9983	0.6271	0.2366
54	Ni	0.0012	12.1644	5.2140	0.0001	0.8749	0.2375
55	Ni	3.4505	8.6667	5.2446	0.2482	0.6234	0.2389
56	Ni	3.4809	12.1510	5.2208	0.2504	0.8740	0.2378
57	Ni	6.9545	1.7392	5.2063	0.5002	0.1251	0.2372
58	Ni	6.9886	5.1606	5.3555	0.5027	0.3712	0.2440
59	Ni	10.4302	1.7302	5.2055	0.7502	0.1244	0.2371
60	Ni	10.4260	5.2148	5.2125	0.7499	0.3751	0.2375
61	Ni	7.0058	8.7054	5.2230	0.5039	0.6261	0.2379
62	Ni	6.9450	12.2020	5.1637	0.4995	0.8776	0.2352
63	Ni	10.4220	8.6954	5.2058	0.7496	0.6254	0.2372
64	Ni	10.4493	12.1908	5.2537	0.7516	0.8768	0.2393
65	Co	1.7379	1.7379	0.0000	0.1250	0.1250	0.0000
66	Co	1.7379	5.2138	0.0000	0.1250	0.3750	0.0000
67	Co	5.2138	1.7379	0.0000	0.3750	0.1250	0.0000
68	Co	5.2138	5.2138	0.0000	0.3750	0.3750	0.0000
69	Co	1.7379	8.6896	0.0000	0.1250	0.6250	0.0000
70	Co	1.7379	12.1654	0.0000	0.1250	0.8750	0.0000
71	Co	5.2138	8.6896	0.0000	0.3750	0.6250	0.0000
72	Co	5.2138	12.1654	0.0000	0.3750	0.8750	0.0000
73	Co	8.6896	1.7379	0.0000	0.6250	0.1250	0.0000
74	Co	8.6896	5.2138	0.0000	0.6250	0.3750	0.0000
75	Co	12.1654	1.7379	0.0000	0.8750	0.1250	0.0000
76	Co	12.1654	5.2138	0.0000	0.8750	0.3750	0.0000
77	Co	8.6896	8.6896	0.0000	0.6250	0.6250	0.0000
78	Co	8.6896	12.1654	0.0000	0.6250	0.8750	0.0000
79	Co	12.1654	8.6896	0.0000	0.8750	0.6250	0.0000
80	Co	12.1654	12.1654	0.0000	0.8750	0.8750	0.0000
81	Co	1.7379	0.0000	1.7379	0.1250	0.0000	0.0792
82	Co	1.7379	3.4758	1.7379	0.1250	0.2500	0.0792
83	Co	5.2138	0.0000	1.7379	0.3750	0.0000	0.0792
84	Co	5.2138	3.4758	1.7379	0.3750	0.2500	0.0792
85	Co	1.7379	6.9517	1.7379	0.1250	0.5000	0.0792
86	Co	1.7379	10.4275	1.7379	0.1250	0.7500	0.0792
87	Co	5.2138	6.9517	1.7379	0.3750	0.5000	0.0792
88	Co	5.2138	10.4275	1.7379	0.3750	0.7500	0.0792
89	Co	8.6896	0.0000	1.7379	0.6250	0.0000	0.0792

90	Co	8.6896	3.4758	1.7379	0.6250	0.2500	0.0792
91	Co	12.1654	0.0000	1.7379	0.8750	0.0000	0.0792
92	Co	12.1654	3.4758	1.7379	0.8750	0.2500	0.0792
93	Co	8.6896	6.9517	1.7379	0.6250	0.5000	0.0792
94	Co	8.6896	10.4275	1.7379	0.6250	0.7500	0.0792
95	Co	12.1654	6.9517	1.7379	0.8750	0.5000	0.0792
96	Co	12.1654	10.4275	1.7379	0.8750	0.7500	0.0792
97	Co	1.7328	1.7309	3.5476	0.1246	0.1245	0.1616
98	Co	1.7031	5.2357	3.5268	0.1225	0.3766	0.1607
99	Co	5.2157	1.7301	3.5528	0.3751	0.1244	0.1618
100	Co	5.2409	5.1880	3.5164	0.3770	0.3731	0.1602
101	Co	1.7291	8.6845	3.5336	0.1244	0.6246	0.1610
102	Co	1.7446	12.1655	3.5477	0.1255	0.8750	0.1616
103	Co	5.2074	8.6765	3.5622	0.3745	0.6241	0.1623
104	Co	5.2128	12.1594	3.5620	0.3749	0.8746	0.1623
105	Co	8.6909	1.7310	3.5514	0.6251	0.1245	0.1618
106	Co	8.6747	5.2035	3.5661	0.6239	0.3743	0.1625
107	Co	12.1680	1.7377	3.5511	0.8752	0.1250	0.1618
108	Co	12.1690	5.2129	3.5618	0.8753	0.3749	0.1623
109	Co	8.6935	8.7013	3.5402	0.6253	0.6258	0.1613
110	Co	8.7051	12.1834	3.4584	0.6261	0.8763	0.1575
111	Co	12.1564	8.6937	3.5500	0.8744	0.6253	0.1617
112	Co	12.1549	12.1734	3.5716	0.8742	0.8756	0.1627
113	Co	1.7444	13.8927	5.2326	0.1255	0.9992	0.2384
114	Co	1.7407	3.4826	5.2170	0.1252	0.2505	0.2377
115	Co	5.2037	0.0064	5.2225	0.3743	0.0005	0.2379
116	Co	5.1734	3.4346	5.1985	0.3721	0.2470	0.2368
117	Co	1.7089	6.9628	5.3287	0.1229	0.5008	0.2427
118	Co	1.7295	10.4585	5.1917	0.1244	0.7522	0.2365
119	Co	5.2513	6.8679	5.1714	0.3777	0.4940	0.2356
120	Co	5.2270	10.4214	5.2300	0.3760	0.7496	0.2383
121	Co	8.6849	-0.0052	5.2973	0.6247	-0.0004	0.2413
122	Co	8.7140	3.4534	5.2249	0.6268	0.2484	0.2380
123	Co	12.1810	0.0152	5.2235	0.8761	0.0011	0.2380
124	Co	12.1650	3.4690	5.2241	0.8750	0.2495	0.2380
125	Co	8.7278	6.9672	5.1775	0.6277	0.5011	0.2359
126	Co	8.7342	10.4855	5.2136	0.6282	0.7542	0.2375
127	Co	12.1622	6.9574	5.2213	0.8748	0.5004	0.2379
128	Co	12.1624	10.4282	5.2173	0.8748	0.7501	0.2377
129	O	5.9010	5.9827	6.7864	0.4244	0.4303	0.3092
130	O	2.6347	7.8126	6.8415	0.1895	0.5619	0.3117
131	O	4.8654	7.7407	8.7933	0.3499	0.5568	0.4006
132	O	6.1055	9.5876	6.8193	0.4391	0.6896	0.3107
133	O	8.0125	9.4781	8.9366	0.5763	0.6817	0.4071
134	O	5.1879	11.3128	8.8828	0.3731	0.8137	0.4047
135	O	8.6072	12.1672	6.3321	0.6191	0.8751	0.2885
136	Ce	3.9735	9.7827	8.0340	0.2858	0.7036	0.3660
137	Ce	3.7933	6.0782	7.7072	0.2728	0.4372	0.3511
138	Ce	7.1162	7.7326	8.0438	0.5118	0.5562	0.3664

139 Sm	7.1884	11.2290	8.0710	0.5170	0.8076	0.3677

/db/jmorales/CoNi-alloy/Profiles/Sm(surf)$Ce_{26}O_{54}$/CO-O

a =				11.6082000732			
b =				11.608200073110298			
c =				22.898399353			
alpha =				90.0			
beta =				90.0			
gamma =				120.0			
	Atom	X	Y	Z	X	Y	Z
1	Sm	1.8053	3.2753	7.0948	0.3184	0.3258	0.3098
2	Ce	1.9347	1.1170	0.7898	0.2222	0.1111	0.0345
3	Ce	0.0000	4.4680	0.7898	0.2222	0.4444	0.0345
4	Ce	-1.9347	7.8190	0.7898	0.2222	0.7778	0.0345
5	Ce	5.8041	1.1170	0.7898	0.5556	0.1111	0.0345
6	Ce	3.8694	4.4680	0.7898	0.5556	0.4444	0.0345
7	Ce	1.9347	7.8190	0.7898	0.5556	0.7778	0.0345
8	Ce	9.6735	1.1170	0.7898	0.8889	0.1111	0.0345
9	Ce	7.7388	4.4680	0.7898	0.8889	0.4444	0.0345
10	Ce	5.8041	7.8190	0.7898	0.8889	0.7778	0.0345
11	Ce	0.0029	2.2374	3.9452	0.1115	0.2226	0.1723
12	Ce	-1.9364	5.5774	3.9463	0.1106	0.5548	0.1723
13	Ce	-3.8759	8.9374	3.9467	0.1106	0.8890	0.1724
14	Ce	3.8609	2.2461	3.9784	0.4443	0.2234	0.1737
15	Ce	1.9461	5.5750	3.9831	0.4449	0.5546	0.1739
16	Ce	0.0043	8.9393	3.9575	0.4450	0.8892	0.1728
17	Ce	7.7473	2.2297	3.9243	0.7783	0.2218	0.1714
18	Ce	5.7922	5.5829	3.9629	0.7766	0.5553	0.1731
19	Ce	3.8687	8.9390	3.9319	0.7779	0.8892	0.1717
20	Ce	0.0115	0.0049	7.0967	0.0012	0.0005	0.3099
21	Ce	-1.9265	3.3592	7.1004	0.0011	0.3341	0.3101
22	Ce	-3.8730	6.6977	7.0876	-0.0005	0.6662	0.3095
23	Ce	3.8500	-0.0032	7.1068	0.3315	-0.0003	0.3104
24	Ce	-0.0161	6.6910	7.1100	0.3314	0.6656	0.3105
25	Ce	7.7395	0.0039	7.1070	0.6669	0.0004	0.3104
26	Ce	5.9133	3.2661	7.0645	0.6719	0.3249	0.3085
27	Ce	3.8531	6.8319	7.1035	0.6717	0.6796	0.3102
28	O	0.0000	0.0000	0.0000	0.0000	0.0000	0.0000
29	O	-1.9347	3.3510	0.0000	-0.0000	0.3333	0.0000
30	O	-3.8694	6.7020	0.0000	-0.0000	0.6667	0.0000
31	O	3.8694	0.0000	0.0000	0.3333	0.0000	0.0000
32	O	1.9347	3.3510	0.0000	0.3333	0.3333	0.0000
33	O	0.0000	6.7020	0.0000	0.3333	0.6667	0.0000
34	O	7.7388	0.0000	0.0000	0.6667	0.0000	0.0000
35	O	5.8041	3.3510	0.0000	0.6667	0.3333	0.0000
36	O	3.8694	6.7020	0.0000	0.6667	0.6667	0.0000
37	O	-0.0000	2.2340	1.5798	0.1111	0.2222	0.0690
38	O	-1.9347	5.5850	1.5798	0.1111	0.5556	0.0690
39	O	-3.8694	8.9360	1.5798	0.1111	0.8889	0.0690
40	O	3.8694	2.2340	1.5798	0.4444	0.2222	0.0690

41	O	1.9347	5.5850	1.5798	0.4444	0.5556	0.0690
42	O	-0.0000	8.9360	1.5798	0.4444	0.8889	0.0690
43	O	7.7388	2.2340	1.5798	0.7778	0.2222	0.0690
44	O	5.8041	5.5850	1.5798	0.7778	0.5556	0.0690
45	O	3.8694	8.9360	1.5798	0.7778	0.8889	0.0690
46	O	1.9347	1.1170	3.1593	0.2222	0.1111	0.1380
47	O	0.0000	4.4680	3.1593	0.2222	0.4444	0.1380
48	O	-1.9347	7.8190	3.1593	0.2222	0.7778	0.1380
49	O	5.8041	1.1170	3.1593	0.5556	0.1111	0.1380
50	O	3.8694	4.4680	3.1593	0.5556	0.4444	0.1380
51	O	1.9347	7.8190	3.1593	0.5556	0.7778	0.1380
52	O	9.6735	1.1170	3.1593	0.8889	0.1111	0.1380
53	O	7.7388	4.4680	3.1593	0.8889	0.4444	0.1380
54	O	5.8041	7.8190	3.1593	0.8889	0.7778	0.1380
55	O	0.0037	-0.0064	4.7481	-0.0000	-0.0006	0.2074
56	O	-1.9356	3.3603	4.7453	0.0004	0.3343	0.2072
57	O	-3.9131	6.6755	4.7634	-0.0051	0.6640	0.2080
58	O	3.8631	0.0361	4.7789	0.3346	0.0036	0.2087
59	O	1.9211	3.3473	4.7380	0.3320	0.3330	0.2069
60	O	0.0327	6.6780	4.7863	0.3350	0.6643	0.2090
61	O	7.7345	-0.0066	4.7520	0.6660	-0.0007	0.2075
62	O	5.8308	3.3440	4.7455	0.6686	0.3326	0.2072
63	O	3.8742	6.7280	4.7464	0.6684	0.6693	0.2073
64	O	-0.0482	2.2076	6.2935	0.1056	0.2196	0.2748
65	O	-1.9661	5.6129	6.2658	0.1098	0.5583	0.2736
66	O	-3.8570	8.8951	6.2632	0.1101	0.8848	0.2735
67	O	3.9133	2.3200	6.3746	0.4525	0.2308	0.2784
68	O	2.0501	5.5790	6.3989	0.4541	0.5550	0.2794
69	O	-0.0223	8.9325	6.2928	0.4424	0.8885	0.2748
70	O	7.7611	2.2190	6.2745	0.7790	0.2207	0.2740
71	O	5.7099	5.5363	6.3658	0.7672	0.5507	0.2780
72	O	3.8679	8.9482	6.2912	0.7783	0.8901	0.2747
73	O	1.9163	0.9825	7.8695	0.2139	0.0977	0.3437
74	O	-0.1239	4.5207	7.8719	0.2142	0.4497	0.3438
75	O	-1.9479	7.8097	7.8919	0.2206	0.7769	0.3446
76	O	5.8519	1.1070	7.8936	0.5592	0.1101	0.3447
77	O	4.1657	4.5596	8.3730	0.5856	0.4536	0.3657
78	O	1.9171	7.8804	7.8923	0.5571	0.7839	0.3447
79	O	9.6574	1.1068	7.9039	0.8870	0.1101	0.3452
80	O	7.7512	4.4489	7.8690	0.8890	0.4425	0.3436
81	O	5.8166	7.8461	7.8753	0.8913	0.7805	0.3439
82	C	3.9256	6.9990	9.9961	0.6863	0.6962	0.4365
83	O	4.0364	7.1612	11.1210	0.7039	0.7123	0.4857
84	O	3.1554	3.9877	9.0657	0.4702	0.3967	0.3959

/db/jmorales/CoNi-alloy/Profiles/Ni-Ce_3SmO_7/O-coverage/M-CO2-O-coverage/M-CO_2-Ni-Ce_3SmO_7-4O

a =			13.9019837372				
b =			13.9019837372				
c =			21.9509918687				
alpha =			90.0				
beta =			90.0				
gamma =			90.0				
	Atom	X	Y	Z	X	Y	Z
1	Ni	0.0000	0.0000	0.0000	0.0000	0.0000	0.0000
2	Ni	1.7377	1.7377	0.0000	0.1250	0.1250	0.0000
3	Ni	0.0000	3.4755	0.0000	0.0000	0.2500	0.0000
4	Ni	1.7377	5.2132	0.0000	0.1250	0.3750	0.0000
5	Ni	3.4755	0.0000	0.0000	0.2500	0.0000	0.0000
6	Ni	5.2132	1.7377	0.0000	0.3750	0.1250	0.0000
7	Ni	3.4755	3.4755	0.0000	0.2500	0.2500	0.0000
8	Ni	5.2132	5.2132	0.0000	0.3750	0.3750	0.0000
9	Ni	0.0000	6.9510	0.0000	0.0000	0.5000	0.0000
10	Ni	1.7377	8.6887	0.0000	0.1250	0.6250	0.0000
11	Ni	0.0000	10.4265	0.0000	0.0000	0.7500	0.0000
12	Ni	1.7377	12.1642	0.0000	0.1250	0.8750	0.0000
13	Ni	3.4755	6.9510	0.0000	0.2500	0.5000	0.0000
14	Ni	5.2132	8.6887	0.0000	0.3750	0.6250	0.0000
15	Ni	3.4755	10.4265	0.0000	0.2500	0.7500	0.0000
16	Ni	5.2132	12.1642	0.0000	0.3750	0.8750	0.0000
17	Ni	6.9510	0.0000	0.0000	0.5000	0.0000	0.0000
18	Ni	8.6887	1.7377	0.0000	0.6250	0.1250	0.0000
19	Ni	6.9510	3.4755	0.0000	0.5000	0.2500	0.0000
20	Ni	8.6887	5.2132	0.0000	0.6250	0.3750	0.0000
21	Ni	10.4265	0.0000	0.0000	0.7500	0.0000	0.0000
22	Ni	12.1642	1.7377	0.0000	0.8750	0.1250	0.0000
23	Ni	10.4265	3.4755	0.0000	0.7500	0.2500	0.0000
24	Ni	12.1642	5.2132	0.0000	0.8750	0.3750	0.0000
25	Ni	6.9510	6.9510	0.0000	0.5000	0.5000	0.0000
26	Ni	8.6887	8.6887	0.0000	0.6250	0.6250	0.0000
27	Ni	6.9510	10.4265	0.0000	0.5000	0.7500	0.0000
28	Ni	8.6887	12.1642	0.0000	0.6250	0.8750	0.0000
29	Ni	10.4265	6.9510	0.0000	0.7500	0.5000	0.0000
30	Ni	12.1642	8.6887	0.0000	0.8750	0.6250	0.0000
31	Ni	10.4265	10.4265	0.0000	0.7500	0.7500	0.0000
32	Ni	12.1642	12.1642	0.0000	0.8750	0.8750	0.0000
33	Ni	0.0000	1.7377	1.7377	0.0000	0.1250	0.0792
34	Ni	1.7377	0.0000	1.7377	0.1250	0.0000	0.0792
35	Ni	0.0000	5.2132	1.7377	0.0000	0.3750	0.0792
36	Ni	1.7377	3.4755	1.7377	0.1250	0.2500	0.0792
37	Ni	3.4755	1.7377	1.7377	0.2500	0.1250	0.0792
38	Ni	5.2132	0.0000	1.7377	0.3750	0.0000	0.0792
39	Ni	3.4755	5.2132	1.7377	0.2500	0.3750	0.0792

40	Ni	5.2132	3.4755	1.7377	0.3750	0.2500	0.0792
41	Ni	0.0000	8.6887	1.7377	0.0000	0.6250	0.0792
42	Ni	1.7377	6.9510	1.7377	0.1250	0.5000	0.0792
43	Ni	0.0000	12.1642	1.7377	0.0000	0.8750	0.0792
44	Ni	1.7377	10.4265	1.7377	0.1250	0.7500	0.0792
45	Ni	3.4755	8.6887	1.7377	0.2500	0.6250	0.0792
46	Ni	5.2132	6.9510	1.7377	0.3750	0.5000	0.0792
47	Ni	3.4755	12.1642	1.7377	0.2500	0.8750	0.0792
48	Ni	5.2132	10.4265	1.7377	0.3750	0.7500	0.0792
49	Ni	6.9510	1.7377	1.7377	0.5000	0.1250	0.0792
50	Ni	8.6887	0.0000	1.7377	0.6250	0.0000	0.0792
51	Ni	6.9510	5.2132	1.7377	0.5000	0.3750	0.0792
52	Ni	8.6887	3.4755	1.7377	0.6250	0.2500	0.0792
53	Ni	10.4265	1.7377	1.7377	0.7500	0.1250	0.0792
54	Ni	12.1642	0.0000	1.7377	0.8750	0.0000	0.0792
55	Ni	10.4265	5.2132	1.7377	0.7500	0.3750	0.0792
56	Ni	12.1642	3.4755	1.7377	0.8750	0.2500	0.0792
57	Ni	6.9510	8.6887	1.7377	0.5000	0.6250	0.0792
58	Ni	8.6887	6.9510	1.7377	0.6250	0.5000	0.0792
59	Ni	6.9510	12.1642	1.7377	0.5000	0.8750	0.0792
60	Ni	8.6887	10.4265	1.7377	0.6250	0.7500	0.0792
61	Ni	10.4265	8.6887	1.7377	0.7500	0.6250	0.0792
62	Ni	12.1642	6.9510	1.7377	0.8750	0.5000	0.0792
63	Ni	10.4265	12.1642	1.7377	0.7500	0.8750	0.0792
64	Ni	12.1642	10.4265	1.7377	0.8750	0.7500	0.0792
65	Ni	0.0106	0.0196	3.5003	0.0008	0.0014	0.1595
66	Ni	1.7382	1.7763	3.4567	0.1250	0.1278	0.1575
67	Ni	13.8788	3.4778	3.5161	0.9983	0.2502	0.1602
68	Ni	1.6829	5.1888	3.4646	0.1211	0.3732	0.1578
69	Ni	3.4665	0.0028	3.5083	0.2494	0.0002	0.1598
70	Ni	5.2154	1.7410	3.5505	0.3752	0.1252	0.1617
71	Ni	3.5046	3.4453	3.4775	0.2521	0.2478	0.1584
72	Ni	5.2312	5.2138	3.4702	0.3763	0.3750	0.1581
73	Ni	0.0015	6.9397	3.5143	0.0001	0.4992	0.1601
74	Ni	1.7518	8.6542	3.5417	0.1260	0.6225	0.1613
75	Ni	13.8931	10.4176	3.5192	0.9994	0.7494	0.1603
76	Ni	1.7271	12.1741	3.5233	0.1242	0.8757	0.1605
77	Ni	3.4741	6.9778	3.4652	0.2499	0.5019	0.1579
78	Ni	5.2330	8.7114	3.5035	0.3764	0.6266	0.1596
79	Ni	3.4639	10.3977	3.5253	0.2492	0.7479	0.1606
80	Ni	5.2048	12.1598	3.4959	0.3744	0.8747	0.1593
81	Ni	6.9388	13.8922	3.5014	0.4991	0.9993	0.1595
82	Ni	8.6863	1.7161	3.4258	0.6248	0.1234	0.1561
83	Ni	6.9435	3.4620	3.4898	0.4995	0.2490	0.1590
84	Ni	8.6489	5.2233	3.5130	0.6221	0.3757	0.1600
85	Ni	10.4484	13.8953	3.5099	0.7516	0.9995	0.1599
86	Ni	12.1684	1.7415	3.5497	0.8753	0.1253	0.1617
87	Ni	10.4184	3.4658	3.4829	0.7494	0.2493	0.1587
88	Ni	12.1609	5.2188	3.4952	0.8748	0.3754	0.1592

89	Ni	6.9387	6.9481	3.5095	0.4991	0.4998	0.1599
90	Ni	8.6889	8.6949	3.4626	0.6250	0.6254	0.1577
91	Ni	6.9835	10.4362	3.5122	0.5023	0.7507	0.1600
92	Ni	8.7122	12.1938	3.5155	0.6267	0.8771	0.1602
93	Ni	10.4051	6.9598	3.5006	0.7485	0.5006	0.1595
94	Ni	12.1812	8.6703	3.5189	0.8762	0.6237	0.1603
95	Ni	10.4476	10.4433	3.4423	0.7515	0.7512	0.1568
96	Ni	12.1776	12.1613	3.4411	0.8760	0.8748	0.1568
97	Ni	0.0311	1.8040	5.2792	0.0022	0.1298	0.2405
98	Ni	1.7312	13.8989	5.2854	0.1245	0.9998	0.2408
99	Ni	13.8333	5.1707	5.3186	0.9951	0.3719	0.2423
100	Ni	1.7626	3.5626	5.3898	0.1268	0.2563	0.2455
101	Ni	3.4580	1.7489	5.2817	0.2487	0.1258	0.2406
102	Ni	5.1967	13.8888	5.2124	0.3738	0.9990	0.2375
103	Ni	3.5108	5.1869	5.0476	0.2525	0.3731	0.2299
104	Ni	5.2348	3.4381	5.3347	0.3765	0.2473	0.2430
105	Ni	0.0284	8.6804	5.2108	0.0020	0.6244	0.2374
106	Ni	1.7234	6.8650	5.2909	0.1240	0.4938	0.2410
107	Ni	0.0122	12.1524	5.2736	0.0009	0.8741	0.2402
108	Ni	1.7347	10.4286	5.2123	0.1248	0.7501	0.2375
109	Ni	3.4565	8.7208	5.3748	0.2486	0.6273	0.2449
110	Ni	5.2076	6.9767	5.2097	0.3746	0.5019	0.2373
111	Ni	3.4727	12.1511	5.2142	0.2498	0.8741	0.2375
112	Ni	5.2267	10.4439	5.1913	0.3760	0.7513	0.2365
113	Ni	6.9602	1.6889	5.2643	0.5007	0.1215	0.2398
114	Ni	8.6879	13.8812	5.2766	0.6249	0.9985	0.2404
115	Ni	6.9414	5.2042	5.2682	0.4993	0.3743	0.2400
116	Ni	8.6919	3.4427	5.2180	0.6252	0.2476	0.2377
117	Ni	10.4142	1.7089	5.2708	0.7491	0.1229	0.2401
118	Ni	12.2124	0.0437	5.2809	0.8785	0.0031	0.2406
119	Ni	10.3755	5.2149	5.1864	0.7463	0.3751	0.2363
120	Ni	12.0653	3.4856	5.2879	0.8679	0.2507	0.2409
121	Ni	6.9880	8.7529	5.2439	0.5027	0.6296	0.2389
122	Ni	8.6883	6.9578	5.2709	0.6250	0.5005	0.2401
123	Ni	6.9411	12.1610	5.2137	0.4993	0.8748	0.2375
124	Ni	8.7323	10.4965	5.2200	0.6281	0.7550	0.2378
125	Ni	10.4464	8.6741	5.1766	0.7514	0.6239	0.2358
126	Ni	12.1395	6.9847	5.3011	0.8732	0.5024	0.2415
127	Ni	10.4872	12.2135	5.3043	0.7544	0.8785	0.2416
128	Ni	12.1659	10.4155	5.3472	0.8751	0.7492	0.2436
129	O	6.1353	4.3563	6.7830	0.4413	0.3134	0.3090
130	O	4.1978	7.5980	6.7504	0.3020	0.5465	0.3075
131	O	6.0716	6.5801	8.7378	0.4367	0.4733	0.3981
132	O	7.9032	7.9407	6.8091	0.5685	0.5712	0.3102
133	O	10.3135	10.2002	6.3074	0.7419	0.7337	0.2873
134	O	9.4768	7.1681	9.0312	0.6817	0.5156	0.4114
135	O	7.5627	9.9043	8.9285	0.5440	0.7124	0.4067
136	Ce	5.9410	8.8039	8.0194	0.4274	0.6333	0.3653
137	Ce	4.3795	5.4772	7.7222	0.3150	0.3940	0.3518

138	Ce	8.2465	5.7632	8.0568	0.5932	0.4146	0.3670
139	Sm	9.3866	9.1420	8.2047	0.6752	0.6576	0.3738
140	C	11.4919	4.8831	6.7305	0.8266	0.3513	0.3066
141	O	12.5902	5.6925	6.7856	0.9056	0.4095	0.3091
142	O	10.8100	4.6100	7.7479	0.7776	0.3316	0.3530
143	O	2.1724	5.2787	6.3863	0.1563	0.3797	0.2909
144	O	1.7419	1.7031	6.1825	0.1253	0.1225	0.2816
145	O	8.6776	1.7298	6.1501	0.6242	0.1244	0.2802
146	O	12.2751	12.2884	6.1927	0.8830	0.8839	0.2821

/db/jmorales/CoNi-alloy/Profiles/CoNi-Ce_3SmO_7/O-coverage/TS-O-coverage/TS-CoNi-Ce_3SmO_7-2O

a =			13.9033384264				
b =			13.9033384264				
c =			21.9516692133				
alpha =			90.0				
beta =			90.0				
gamma =			90.0				
	Atom	X	Y	Z	X	Y	Z
1	Ni	0.0000	0.0000	0.0000	0.0000	0.0000	0.0000
2	Ni	0.0000	3.4758	0.0000	0.0000	0.2500	0.0000
3	Ni	3.4758	0.0000	0.0000	0.2500	0.0000	0.0000
4	Ni	3.4758	3.4758	0.0000	0.2500	0.2500	0.0000
5	Ni	0.0000	6.9517	0.0000	0.0000	0.5000	0.0000
6	Ni	0.0000	10.4275	0.0000	0.0000	0.7500	0.0000
7	Ni	3.4758	6.9517	0.0000	0.2500	0.5000	0.0000
8	Ni	3.4758	10.4275	0.0000	0.2500	0.7500	0.0000
9	Ni	6.9517	0.0000	0.0000	0.5000	0.0000	0.0000
10	Ni	6.9517	3.4758	0.0000	0.5000	0.2500	0.0000
11	Ni	10.4275	0.0000	0.0000	0.7500	0.0000	0.0000
12	Ni	10.4275	3.4758	0.0000	0.7500	0.2500	0.0000
13	Ni	6.9517	6.9517	0.0000	0.5000	0.5000	0.0000
14	Ni	6.9517	10.4275	0.0000	0.5000	0.7500	0.0000
15	Ni	10.4275	6.9517	0.0000	0.7500	0.5000	0.0000
16	Ni	10.4275	10.4275	0.0000	0.7500	0.7500	0.0000
17	Ni	0.0000	1.7379	1.7379	0.0000	0.1250	0.0792
18	Ni	0.0000	5.2138	1.7379	0.0000	0.3750	0.0792
19	Ni	3.4758	1.7379	1.7379	0.2500	0.1250	0.0792
20	Ni	3.4758	5.2138	1.7379	0.2500	0.3750	0.0792
21	Ni	0.0000	8.6896	1.7379	0.0000	0.6250	0.0792
22	Ni	0.0000	12.1654	1.7379	0.0000	0.8750	0.0792
23	Ni	3.4758	8.6896	1.7379	0.2500	0.6250	0.0792
24	Ni	3.4758	12.1654	1.7379	0.2500	0.8750	0.0792
25	Ni	6.9517	1.7379	1.7379	0.5000	0.1250	0.0792
26	Ni	6.9517	5.2138	1.7379	0.5000	0.3750	0.0792
27	Ni	10.4275	1.7379	1.7379	0.7500	0.1250	0.0792
28	Ni	10.4275	5.2138	1.7379	0.7500	0.3750	0.0792
29	Ni	6.9517	8.6896	1.7379	0.5000	0.6250	0.0792
30	Ni	6.9517	12.1654	1.7379	0.5000	0.8750	0.0792
31	Ni	10.4275	8.6896	1.7379	0.7500	0.6250	0.0792
32	Ni	10.4275	12.1654	1.7379	0.7500	0.8750	0.0792
33	Ni	13.9018	13.8972	3.4443	0.9999	0.9996	0.1569
34	Ni	0.0055	3.4608	3.5162	0.0004	0.2489	0.1602
35	Ni	3.4630	13.8960	3.4958	0.2491	0.9995	0.1592
36	Ni	3.4712	3.4506	3.4642	0.2497	0.2482	0.1578
37	Ni	0.0028	6.9544	3.5091	0.0002	0.5002	0.1599
38	Ni	13.8860	10.4439	3.5057	0.9988	0.7512	0.1597
39	Ni	3.4830	6.9492	3.4929	0.2505	0.4998	0.1591

40	Ni	3.4830	10.3872	3.5062	0.2505	0.7471	0.1597
41	Ni	6.9532	0.0122	3.4943	0.5001	0.0009	0.1592
42	Ni	6.9572	3.4670	3.5348	0.5004	0.2494	0.1610
43	Ni	10.4493	0.0033	3.4543	0.7516	0.0002	0.1574
44	Ni	10.4345	3.4672	3.5095	0.7505	0.2494	0.1599
45	Ni	6.9660	6.9603	3.4922	0.5010	0.5006	0.1591
46	Ni	6.9686	10.4458	3.4771	0.5012	0.7513	0.1584
47	Ni	10.4351	6.9383	3.4879	0.7505	0.4990	0.1589
48	Ni	10.4284	10.4449	3.5044	0.7501	0.7512	0.1596
49	Ni	0.0196	1.7557	5.2523	0.0014	0.1263	0.2393
50	Ni	0.0098	5.2109	5.2133	0.0007	0.3748	0.2375
51	Ni	3.4543	1.7384	5.1890	0.2485	0.1250	0.2364
52	Ni	3.4656	5.1700	5.0902	0.2493	0.3719	0.2319
53	Ni	13.8760	8.7124	5.1921	0.9980	0.6266	0.2365
54	Ni	0.0201	12.1336	5.2579	0.0014	0.8727	0.2395
55	Ni	3.4515	8.6539	5.2425	0.2482	0.6224	0.2388
56	Ni	3.4366	12.1135	5.1864	0.2472	0.8713	0.2363
57	Ni	6.9739	1.7424	5.3271	0.5016	0.1253	0.2427
58	Ni	6.9675	5.1436	5.3661	0.5011	0.3700	0.2445
59	Ni	10.4697	1.7677	5.2453	0.7530	0.1271	0.2389
60	Ni	10.4216	5.2123	5.2071	0.7496	0.3749	0.2372
61	Ni	6.9874	8.7218	5.2051	0.5026	0.6273	0.2371
62	Ni	6.9922	12.1586	5.2005	0.5029	0.8745	0.2369
63	Ni	10.4375	8.6762	5.1848	0.7507	0.6240	0.2362
64	Ni	10.3998	12.1755	5.3402	0.7480	0.8757	0.2433
65	Co	1.7379	1.7379	0.0000	0.1250	0.1250	0.0000
66	Co	1.7379	5.2138	0.0000	0.1250	0.3750	0.0000
67	Co	5.2138	1.7379	0.0000	0.3750	0.1250	0.0000
68	Co	5.2138	5.2138	0.0000	0.3750	0.3750	0.0000
69	Co	1.7379	8.6896	0.0000	0.1250	0.6250	0.0000
70	Co	1.7379	12.1654	0.0000	0.1250	0.8750	0.0000
71	Co	5.2138	8.6896	0.0000	0.3750	0.6250	0.0000
72	Co	5.2138	12.1654	0.0000	0.3750	0.8750	0.0000
73	Co	8.6896	1.7379	0.0000	0.6250	0.1250	0.0000
74	Co	8.6896	5.2138	0.0000	0.6250	0.3750	0.0000
75	Co	12.1654	1.7379	0.0000	0.8750	0.1250	0.0000
76	Co	12.1654	5.2138	0.0000	0.8750	0.3750	0.0000
77	Co	8.6896	8.6896	0.0000	0.6250	0.6250	0.0000
78	Co	8.6896	12.1654	0.0000	0.6250	0.8750	0.0000
79	Co	12.1654	8.6896	0.0000	0.8750	0.6250	0.0000
80	Co	12.1654	12.1654	0.0000	0.8750	0.8750	0.0000
81	Co	1.7379	0.0000	1.7379	0.1250	0.0000	0.0792
82	Co	1.7379	3.4758	1.7379	0.1250	0.2500	0.0792
83	Co	5.2138	0.0000	1.7379	0.3750	0.0000	0.0792
84	Co	5.2138	3.4758	1.7379	0.3750	0.2500	0.0792
85	Co	1.7379	6.9517	1.7379	0.1250	0.5000	0.0792
86	Co	1.7379	10.4275	1.7379	0.1250	0.7500	0.0792
87	Co	5.2138	6.9517	1.7379	0.3750	0.5000	0.0792
88	Co	5.2138	10.4275	1.7379	0.3750	0.7500	0.0792

89	Co	8.6896	0.0000	1.7379	0.6250	0.0000	0.0792
90	Co	8.6896	3.4758	1.7379	0.6250	0.2500	0.0792
91	Co	12.1654	0.0000	1.7379	0.8750	0.0000	0.0792
92	Co	12.1654	3.4758	1.7379	0.8750	0.2500	0.0792
93	Co	8.6896	6.9517	1.7379	0.6250	0.5000	0.0792
94	Co	8.6896	10.4275	1.7379	0.6250	0.7500	0.0792
95	Co	12.1654	6.9517	1.7379	0.8750	0.5000	0.0792
96	Co	12.1654	10.4275	1.7379	0.8750	0.7500	0.0792
97	Co	1.7318	1.7336	3.5377	0.1246	0.1247	0.1612
98	Co	1.7112	5.2361	3.5263	0.1231	0.3766	0.1606
99	Co	5.2238	1.7159	3.5658	0.3757	0.1234	0.1624
100	Co	5.2499	5.1842	3.5217	0.3776	0.3729	0.1604
101	Co	1.7272	8.6722	3.5289	0.1242	0.6237	0.1608
102	Co	1.7358	12.1573	3.5183	0.1249	0.8744	0.1603
103	Co	5.2057	8.6747	3.5509	0.3744	0.6239	0.1618
104	Co	5.2083	12.1480	3.5491	0.3746	0.8737	0.1617
105	Co	8.6917	1.7278	3.5551	0.6252	0.1243	0.1620
106	Co	8.6667	5.1985	3.5703	0.6234	0.3739	0.1626
107	Co	12.1830	1.7362	3.5431	0.8763	0.1249	0.1614
108	Co	12.1711	5.2118	3.5550	0.8754	0.3749	0.1619
109	Co	8.6923	8.6787	3.5119	0.6252	0.6242	0.1600
110	Co	8.7077	12.1953	3.4830	0.6263	0.8772	0.1587
111	Co	12.1603	8.6914	3.5385	0.8746	0.6251	0.1612
112	Co	12.1496	12.1752	3.5797	0.8739	0.8757	0.1631
113	Co	1.7693	13.8878	5.2709	0.1273	0.9989	0.2401
114	Co	1.7570	3.4967	5.2109	0.1264	0.2515	0.2374
115	Co	5.1314	13.8790	5.2551	0.3691	0.9982	0.2394
116	Co	5.1610	3.4302	5.2043	0.3712	0.2467	0.2371
117	Co	1.7148	6.9524	5.3319	0.1233	0.5001	0.2429
118	Co	1.7329	10.4292	5.1759	0.1246	0.7501	0.2358
119	Co	5.2717	6.8834	5.1786	0.3792	0.4951	0.2359
120	Co	5.1974	10.4022	5.2115	0.3738	0.7482	0.2374
121	Co	8.7079	0.0595	5.3525	0.6263	0.0043	0.2438
122	Co	8.7169	3.4680	5.2185	0.6270	0.2494	0.2377
123	Co	12.2028	13.8870	5.3664	0.8777	0.9988	0.2445
124	Co	12.1804	3.4968	5.2132	0.8761	0.2515	0.2375
125	Co	8.7196	6.9734	5.1773	0.6272	0.5016	0.2358
126	Co	8.7522	10.3834	5.2061	0.6295	0.7468	0.2372
127	Co	12.1691	6.9473	5.2172	0.8753	0.4997	0.2377
128	Co	12.1609	10.4193	5.2126	0.8747	0.7494	0.2375
129	O	6.0003	6.0891	6.7962	0.4316	0.4380	0.3096
130	O	2.6489	7.7812	6.8323	0.1905	0.5597	0.3112
131	O	4.8634	7.8270	8.7583	0.3498	0.5630	0.3990
132	O	6.0684	9.7938	6.8179	0.4365	0.7044	0.3106
133	O	7.9149	9.7195	9.0009	0.5693	0.6991	0.4100
134	O	5.0171	11.3091	9.0084	0.3609	0.8134	0.4104
135	O	8.6327	11.7944	6.3876	0.6209	0.8483	0.2910
136	Ce	3.8937	9.8268	8.0366	0.2801	0.7068	0.3661
137	Ce	3.8892	6.1025	7.7150	0.2797	0.4389	0.3515

138	Ce	7.1326	7.9941	8.0544	0.5130	0.5750	0.3669
139	Sm	7.0364	11.5508	8.2378	0.5061	0.8308	0.3753
140	C	7.4686	0.5865	6.8294	0.5372	0.0422	0.3111
141	O	6.1662	13.2184	6.6268	0.4435	0.9507	0.3019
142	O	7.7306	0.5947	8.0136	0.5560	0.0428	0.3651
143	O	10.5189	0.1034	6.2235	0.7566	0.0074	0.2835
144	O	0.0839	13.8916	6.1876	0.0060	0.9992	0.2819

/db/jmorales/CoNi-alloy/Profiles/Sm(subsurf)$Ce_{26}O_{54}$/O

a =				11.6082000732			
b =				11.608200073110298			
c =				22.898399353			
alpha =				90.0			
beta =				90.0			
gamma =				120.0			
	Atom	X	Y	Z	X	Y	Z
1	Sm	1.9368	5.5947	3.9627	0.4451	0.5565	0.1731
2	Ce	1.9347	1.1170	0.7898	0.2222	0.1111	0.0345
3	Ce	0.0000	4.4680	0.7898	0.2222	0.4444	0.0345
4	Ce	-1.9347	7.8190	0.7898	0.2222	0.7778	0.0345
5	Ce	5.8041	1.1170	0.7898	0.5556	0.1111	0.0345
6	Ce	3.8694	4.4680	0.7898	0.5556	0.4444	0.0345
7	Ce	1.9347	7.8190	0.7898	0.5556	0.7778	0.0345
8	Ce	9.6735	1.1170	0.7898	0.8889	0.1111	0.0345
9	Ce	7.7388	4.4680	0.7898	0.8889	0.4444	0.0345
10	Ce	5.8041	7.8190	0.7898	0.8889	0.7778	0.0345
11	Ce	-0.0001	2.2544	3.9492	0.1121	0.2243	0.1725
12	Ce	-1.9219	5.5777	3.9219	0.1119	0.5548	0.1713
13	Ce	-3.8707	8.9284	3.9482	0.1106	0.8881	0.1724
14	Ce	3.8669	2.2546	3.9475	0.4453	0.2243	0.1724
15	Ce	0.0153	8.9174	3.9543	0.4448	0.8870	0.1727
16	Ce	7.7392	2.2378	3.9198	0.7780	0.2226	0.1712
17	Ce	5.7914	5.5785	3.9240	0.7764	0.5549	0.1714
18	Ce	3.8482	8.9106	3.9598	0.7747	0.8864	0.1729
19	Ce	-0.0005	-0.0025	7.1019	-0.0002	-0.0002	0.3101
20	Ce	-1.9365	3.3451	7.1041	-0.0005	0.3327	0.3102
21	Ce	-3.8624	6.6955	7.0884	0.0003	0.6660	0.3096
22	Ce	3.8669	0.0004	7.0891	0.3331	0.0000	0.3096
23	Ce	1.9354	3.3458	7.0999	0.3331	0.3328	0.3101
24	Ce	-0.1143	6.6318	7.0671	0.3200	0.6597	0.3086
25	Ce	7.7333	0.1313	7.0814	0.6727	0.0131	0.3093
26	Ce	5.8106	3.3398	7.1023	0.6667	0.3322	0.3102
27	Ce	3.9975	6.6175	7.0895	0.6735	0.6583	0.3096
28	O	0.0000	0.0000	0.0000	0.0000	0.0000	0.0000
29	O	-1.9347	3.3510	0.0000	-0.0000	0.3333	0.0000
30	O	-3.8694	6.7020	0.0000	-0.0000	0.6667	0.0000
31	O	3.8694	0.0000	0.0000	0.3333	0.0000	0.0000
32	O	1.9347	3.3510	0.0000	0.3333	0.3333	0.0000
33	O	0.0000	6.7020	0.0000	0.3333	0.6667	0.0000
34	O	7.7388	0.0000	0.0000	0.6667	0.0000	0.0000
35	O	5.8041	3.3510	0.0000	0.6667	0.3333	0.0000
36	O	3.8694	6.7020	0.0000	0.6667	0.6667	0.0000
37	O	-0.0000	2.2340	1.5798	0.1111	0.2222	0.0690
38	O	-1.9347	5.5850	1.5798	0.1111	0.5556	0.0690
39	O	-3.8694	8.9360	1.5798	0.1111	0.8889	0.0690
40	O	3.8694	2.2340	1.5798	0.4444	0.2222	0.0690

41	O	1.9347	5.5850	1.5798	0.4444	0.5556	0.0690
42	O	-0.0000	8.9360	1.5798	0.4444	0.8889	0.0690
43	O	7.7388	2.2340	1.5798	0.7778	0.2222	0.0690
44	O	5.8041	5.5850	1.5798	0.7778	0.5556	0.0690
45	O	3.8694	8.9360	1.5798	0.7778	0.8889	0.0690
46	O	1.9347	1.1170	3.1593	0.2222	0.1111	0.1380
47	O	0.0000	4.4680	3.1593	0.2222	0.4444	0.1380
48	O	-1.9347	7.8190	3.1593	0.2222	0.7778	0.1380
49	O	5.8041	1.1170	3.1593	0.5556	0.1111	0.1380
50	O	3.8694	4.4680	3.1593	0.5556	0.4444	0.1380
51	O	1.9347	7.8190	3.1593	0.5556	0.7778	0.1380
52	O	9.6735	1.1170	3.1593	0.8889	0.1111	0.1380
53	O	7.7388	4.4680	3.1593	0.8889	0.4444	0.1380
54	O	5.8041	7.8190	3.1593	0.8889	0.7778	0.1380
55	O	-0.0456	-0.0308	4.8017	-0.0055	-0.0031	0.2097
56	O	-1.9317	3.3431	4.7607	-0.0001	0.3325	0.2079
57	O	-3.8691	6.7100	4.7584	0.0004	0.6675	0.2078
58	O	-1.9056	10.0319	4.7817	0.3348	0.9979	0.2088
59	O	1.9334	3.3444	4.8351	0.3329	0.3327	0.2112
60	O	-0.0621	6.7126	4.7940	0.3285	0.6677	0.2094
61	O	7.7413	0.0229	4.7630	0.6680	0.0023	0.2080
62	O	5.8011	3.3433	4.7606	0.6660	0.3326	0.2079
63	O	3.9374	6.7147	4.7916	0.6732	0.6679	0.2093
64	O	-0.0043	2.2144	6.3142	0.1098	0.2203	0.2757
65	O	-1.9631	5.5584	6.2965	0.1073	0.5529	0.2750
66	O	-3.8628	8.9555	6.2947	0.1126	0.8908	0.2749
67	O	3.8728	2.2073	6.3100	0.4434	0.2196	0.2756
68	O	1.9379	5.7298	6.4758	0.4519	0.5700	0.2828
69	O	0.0822	8.9046	6.3785	0.4500	0.8858	0.2786
70	O	7.7394	2.2500	6.2886	0.7786	0.2238	0.2746
71	O	5.8424	5.5537	6.3044	0.7795	0.5524	0.2753
72	O	3.7437	8.8748	6.4228	0.7639	0.8828	0.2805
73	O	1.9298	1.1118	7.8898	0.2215	0.1106	0.3446
74	O	-0.0387	4.4464	7.8762	0.2178	0.4423	0.3440
75	O	-1.9587	7.7985	7.8693	0.2191	0.7757	0.3437
76	O	5.8174	1.1406	7.8935	0.5579	0.1135	0.3447
77	O	3.9158	4.4198	7.8714	0.5572	0.4397	0.3438
78	O	1.6078	8.0129	8.4456	0.5370	0.7971	0.3688
79	O	9.6604	1.1401	7.8910	0.8889	0.1134	0.3446
80	O	7.7465	4.4543	7.9018	0.8889	0.4431	0.3451
81	O	5.8570	7.8050	7.8611	0.8928	0.7764	0.3433
82	O	2.6710	7.4304	9.0416	0.5997	0.7391	0.3949

/db/jmorales/CoNi-alloy/Profiles/Ni-Ce_3SmO_7/O-coverage/M-CO2-O-coverage/M-CO_2-Ni-Ce_3SmO_7-5O

a = 13.9019837372
b = 13.9019837372
c = 21.9509918687
alpha = 90.0
beta = 90.0
gamma = 90.0

	Atom	X	Y	Z	X	Y	Z
1	Ni	0.0000	0.0000	0.0000	0.0000	0.0000	0.0000
2	Ni	1.7377	1.7377	0.0000	0.1250	0.1250	0.0000
3	Ni	0.0000	3.4755	0.0000	0.0000	0.2500	0.0000
4	Ni	1.7377	5.2132	0.0000	0.1250	0.3750	0.0000
5	Ni	3.4755	0.0000	0.0000	0.2500	0.0000	0.0000
6	Ni	5.2132	1.7377	0.0000	0.3750	0.1250	0.0000
7	Ni	3.4755	3.4755	0.0000	0.2500	0.2500	0.0000
8	Ni	5.2132	5.2132	0.0000	0.3750	0.3750	0.0000
9	Ni	0.0000	6.9510	0.0000	0.0000	0.5000	0.0000
10	Ni	1.7377	8.6887	0.0000	0.1250	0.6250	0.0000
11	Ni	0.0000	10.4265	0.0000	0.0000	0.7500	0.0000
12	Ni	1.7377	12.1642	0.0000	0.1250	0.8750	0.0000
13	Ni	3.4755	6.9510	0.0000	0.2500	0.5000	0.0000
14	Ni	5.2132	8.6887	0.0000	0.3750	0.6250	0.0000
15	Ni	3.4755	10.4265	0.0000	0.2500	0.7500	0.0000
16	Ni	5.2132	12.1642	0.0000	0.3750	0.8750	0.0000
17	Ni	6.9510	0.0000	0.0000	0.5000	0.0000	0.0000
18	Ni	8.6887	1.7377	0.0000	0.6250	0.1250	0.0000
19	Ni	6.9510	3.4755	0.0000	0.5000	0.2500	0.0000
20	Ni	8.6887	5.2132	0.0000	0.6250	0.3750	0.0000
21	Ni	10.4265	0.0000	0.0000	0.7500	0.0000	0.0000
22	Ni	12.1642	1.7377	0.0000	0.8750	0.1250	0.0000
23	Ni	10.4265	3.4755	0.0000	0.7500	0.2500	0.0000
24	Ni	12.1642	5.2132	0.0000	0.8750	0.3750	0.0000
25	Ni	6.9510	6.9510	0.0000	0.5000	0.5000	0.0000
26	Ni	8.6887	8.6887	0.0000	0.6250	0.6250	0.0000
27	Ni	6.9510	10.4265	0.0000	0.5000	0.7500	0.0000
28	Ni	8.6887	12.1642	0.0000	0.6250	0.8750	0.0000
29	Ni	10.4265	6.9510	0.0000	0.7500	0.5000	0.0000
30	Ni	12.1642	8.6887	0.0000	0.8750	0.6250	0.0000
31	Ni	10.4265	10.4265	0.0000	0.7500	0.7500	0.0000
32	Ni	12.1642	12.1642	0.0000	0.8750	0.8750	0.0000
33	Ni	0.0000	1.7377	1.7377	0.0000	0.1250	0.0792
34	Ni	1.7377	0.0000	1.7377	0.1250	0.0000	0.0792
35	Ni	0.0000	5.2132	1.7377	0.0000	0.3750	0.0792
36	Ni	1.7377	3.4755	1.7377	0.1250	0.2500	0.0792
37	Ni	3.4755	1.7377	1.7377	0.2500	0.1250	0.0792
38	Ni	5.2132	0.0000	1.7377	0.3750	0.0000	0.0792
39	Ni	3.4755	5.2132	1.7377	0.2500	0.3750	0.0792

40	Ni	5.2132	3.4755	1.7377	0.3750	0.2500	0.0792
41	Ni	0.0000	8.6887	1.7377	0.0000	0.6250	0.0792
42	Ni	1.7377	6.9510	1.7377	0.1250	0.5000	0.0792
43	Ni	0.0000	12.1642	1.7377	0.0000	0.8750	0.0792
44	Ni	1.7377	10.4265	1.7377	0.1250	0.7500	0.0792
45	Ni	3.4755	8.6887	1.7377	0.2500	0.6250	0.0792
46	Ni	5.2132	6.9510	1.7377	0.3750	0.5000	0.0792
47	Ni	3.4755	12.1642	1.7377	0.2500	0.8750	0.0792
48	Ni	5.2132	10.4265	1.7377	0.3750	0.7500	0.0792
49	Ni	6.9510	1.7377	1.7377	0.5000	0.1250	0.0792
50	Ni	8.6887	0.0000	1.7377	0.6250	0.0000	0.0792
51	Ni	6.9510	5.2132	1.7377	0.5000	0.3750	0.0792
52	Ni	8.6887	3.4755	1.7377	0.6250	0.2500	0.0792
53	Ni	10.4265	1.7377	1.7377	0.7500	0.1250	0.0792
54	Ni	12.1642	0.0000	1.7377	0.8750	0.0000	0.0792
55	Ni	10.4265	5.2132	1.7377	0.7500	0.3750	0.0792
56	Ni	12.1642	3.4755	1.7377	0.8750	0.2500	0.0792
57	Ni	6.9510	8.6887	1.7377	0.5000	0.6250	0.0792
58	Ni	8.6887	6.9510	1.7377	0.6250	0.5000	0.0792
59	Ni	6.9510	12.1642	1.7377	0.5000	0.8750	0.0792
60	Ni	8.6887	10.4265	1.7377	0.6250	0.7500	0.0792
61	Ni	10.4265	8.6887	1.7377	0.7500	0.6250	0.0792
62	Ni	12.1642	6.9510	1.7377	0.8750	0.5000	0.0792
63	Ni	10.4265	12.1642	1.7377	0.7500	0.8750	0.0792
64	Ni	12.1642	10.4265	1.7377	0.8750	0.7500	0.0792
65	Ni	0.0098	0.0210	3.4962	0.0007	0.0015	0.1593
66	Ni	1.7493	1.7765	3.4687	0.1258	0.1278	0.1580
67	Ni	13.8772	3.4796	3.5114	0.9982	0.2503	0.1600
68	Ni	1.6839	5.1887	3.4648	0.1211	0.3732	0.1578
69	Ni	3.4603	0.0042	3.5075	0.2489	0.0003	0.1598
70	Ni	5.2211	1.7442	3.4828	0.3756	0.1255	0.1587
71	Ni	3.4991	3.4419	3.4793	0.2517	0.2476	0.1585
72	Ni	5.2335	5.2010	3.4803	0.3765	0.3741	0.1585
73	Ni	0.0030	6.9395	3.5149	0.0002	0.4992	0.1601
74	Ni	1.7533	8.6518	3.5414	0.1261	0.6223	0.1613
75	Ni	13.8949	10.4157	3.5172	0.9995	0.7492	0.1602
76	Ni	1.7222	12.1746	3.5201	0.1239	0.8757	0.1604
77	Ni	3.4753	6.9710	3.4660	0.2500	0.5014	0.1579
78	Ni	5.2311	8.7117	3.5003	0.3763	0.6267	0.1595
79	Ni	3.4638	10.4009	3.5188	0.2492	0.7482	0.1603
80	Ni	5.2044	12.1739	3.5000	0.3744	0.8757	0.1594
81	Ni	6.9441	13.8968	3.5067	0.4995	0.9996	0.1598
82	Ni	8.6739	1.7139	3.4378	0.6239	0.1233	0.1566
83	Ni	6.9510	3.4549	3.4963	0.5000	0.2485	0.1593
84	Ni	8.6517	5.2232	3.5105	0.6223	0.3757	0.1599
85	Ni	10.4493	13.8939	3.5060	0.7516	0.9994	0.1597
86	Ni	12.1671	1.7422	3.5395	0.8752	0.1253	0.1612
87	Ni	10.4182	3.4646	3.4798	0.7494	0.2492	0.1585
88	Ni	12.1587	5.2227	3.4950	0.8746	0.3757	0.1592

89	Ni	6.9394	6.9492	3.5050	0.4992	0.4999	0.1597
90	Ni	8.6885	8.6968	3.4618	0.6250	0.6256	0.1577
91	Ni	6.9792	10.4390	3.5087	0.5020	0.7509	0.1598
92	Ni	8.7143	12.1927	3.5113	0.6268	0.8770	0.1600
93	Ni	10.4060	6.9621	3.5025	0.7485	0.5008	0.1596
94	Ni	12.1828	8.6706	3.5186	0.8763	0.6237	0.1603
95	Ni	10.4494	10.4436	3.4399	0.7516	0.7512	0.1567
96	Ni	12.1794	12.1597	3.4397	0.8761	0.8747	0.1567
97	Ni	13.8954	1.8068	5.2753	0.9995	0.1300	0.2403
98	Ni	1.7203	0.0156	5.2785	0.1237	0.0011	0.2405
99	Ni	13.8355	5.1730	5.3191	0.9952	0.3721	0.2423
100	Ni	1.7513	3.5492	5.3798	0.1260	0.2553	0.2451
101	Ni	3.4571	1.7459	5.3782	0.2487	0.1256	0.2450
102	Ni	5.1845	13.8686	5.2707	0.3729	0.9976	0.2401
103	Ni	3.5117	5.1764	5.0575	0.2526	0.3723	0.2304
104	Ni	5.2465	3.4617	5.3649	0.3774	0.2490	0.2444
105	Ni	0.0311	8.6785	5.2099	0.0022	0.6243	0.2373
106	Ni	1.7291	6.8628	5.2902	0.1244	0.4937	0.2410
107	Ni	0.0209	12.1541	5.2737	0.0015	0.8743	0.2402
108	Ni	1.7338	10.4255	5.2092	0.1247	0.7499	0.2373
109	Ni	3.4618	8.7249	5.3662	0.2490	0.6276	0.2445
110	Ni	5.2114	6.9707	5.1998	0.3749	0.5014	0.2369
111	Ni	3.4564	12.1453	5.2043	0.2486	0.8736	0.2371
112	Ni	5.2240	10.4553	5.1830	0.3758	0.7521	0.2361
113	Ni	6.9546	1.6688	5.4169	0.5003	0.1200	0.2468
114	Ni	8.7029	13.8893	5.2639	0.6260	0.9991	0.2398
115	Ni	6.9621	5.2087	5.2611	0.5008	0.3747	0.2397
116	Ni	8.6930	3.4222	5.2140	0.6253	0.2462	0.2375
117	Ni	10.4473	1.7088	5.2695	0.7515	0.1229	0.2401
118	Ni	12.2094	0.0300	5.2694	0.8782	0.0022	0.2401
119	Ni	10.3660	5.2157	5.1871	0.7456	0.3752	0.2363
120	Ni	12.0635	3.4973	5.2831	0.8678	0.2516	0.2407
121	Ni	6.9830	8.7566	5.2410	0.5023	0.6299	0.2388
122	Ni	8.6889	6.9680	5.2667	0.6250	0.5012	0.2399
123	Ni	6.9478	12.1608	5.2064	0.4998	0.8748	0.2372
124	Ni	8.7255	10.4943	5.2124	0.6276	0.7549	0.2375
125	Ni	10.4480	8.6838	5.1763	0.7515	0.6246	0.2358
126	Ni	12.1371	6.9855	5.3004	0.8730	0.5025	0.2415
127	Ni	10.4843	12.2021	5.3044	0.7542	0.8777	0.2416
128	Ni	12.1756	10.4195	5.3419	0.8758	0.7495	0.2434
129	O	6.1741	4.4876	6.8293	0.4441	0.3228	0.3111
130	O	4.2118	7.6117	6.7395	0.3030	0.5475	0.3070
131	O	6.0450	6.6521	8.7911	0.4348	0.4785	0.4005
132	O	7.8876	7.9436	6.8122	0.5674	0.5714	0.3103
133	O	10.3081	10.2188	6.2959	0.7415	0.7351	0.2868
134	O	9.4611	7.2111	9.0562	0.6806	0.5187	0.4126
135	O	7.5556	9.9467	8.9128	0.5435	0.7155	0.4060
136	Ce	5.9259	8.8500	8.0262	0.4263	0.6366	0.3656
137	Ce	4.3807	5.5207	7.7483	0.3151	0.3971	0.3530

138	Ce	8.2066	5.8109	8.0862	0.5903	0.4180	0.3684
139	Sm	9.3792	9.1661	8.2028	0.6747	0.6593	0.3737
140	C	11.4888	4.8896	6.7290	0.8264	0.3517	0.3065
141	O	12.5882	5.6932	6.7883	0.9055	0.4095	0.3092
142	O	10.7919	4.6234	7.7374	0.7763	0.3326	0.3525
143	O	2.1721	5.2820	6.3870	0.1562	0.3799	0.2910
144	O	1.6602	1.7171	6.1916	0.1194	0.1235	0.2821
145	O	8.7495	1.7257	6.1588	0.6294	0.1241	0.2806
146	O	12.2828	12.2816	6.1908	0.8835	0.8834	0.2820
147	O	5.1979	1.6106	6.2095	0.3739	0.1159	0.2829

/db/jmorales/CoNi-alloy/Profiles/CoNi-Ce_3SmO_7/M-CO2

a =				13.9033384264			
b =				13.9033384264			
c =				21.9516692133			
alpha =				90.0			
beta =				90.0			
gamma =				90.0			
	Atom	X	Y	Z	X	Y	Z
1	Ni	0.0000	0.0000	0.0000	0.0000	0.0000	0.0000
2	Ni	0.0000	3.4758	0.0000	0.0000	0.2500	0.0000
3	Ni	3.4758	0.0000	0.0000	0.2500	0.0000	0.0000
4	Ni	3.4758	3.4758	0.0000	0.2500	0.2500	0.0000
5	Ni	0.0000	6.9517	0.0000	0.0000	0.5000	0.0000
6	Ni	0.0000	10.4275	0.0000	0.0000	0.7500	0.0000
7	Ni	3.4758	6.9517	0.0000	0.2500	0.5000	0.0000
8	Ni	3.4758	10.4275	0.0000	0.2500	0.7500	0.0000
9	Ni	6.9517	0.0000	0.0000	0.5000	0.0000	0.0000
10	Ni	6.9517	3.4758	0.0000	0.5000	0.2500	0.0000
11	Ni	10.4275	0.0000	0.0000	0.7500	0.0000	0.0000
12	Ni	10.4275	3.4758	0.0000	0.7500	0.2500	0.0000
13	Ni	6.9517	6.9517	0.0000	0.5000	0.5000	0.0000
14	Ni	6.9517	10.4275	0.0000	0.5000	0.7500	0.0000
15	Ni	10.4275	6.9517	0.0000	0.7500	0.5000	0.0000
16	Ni	10.4275	10.4275	0.0000	0.7500	0.7500	0.0000
17	Ni	0.0000	1.7379	1.7379	0.0000	0.1250	0.0792
18	Ni	0.0000	5.2138	1.7379	0.0000	0.3750	0.0792
19	Ni	3.4758	1.7379	1.7379	0.2500	0.1250	0.0792
20	Ni	3.4758	5.2138	1.7379	0.2500	0.3750	0.0792
21	Ni	0.0000	8.6896	1.7379	0.0000	0.6250	0.0792
22	Ni	0.0000	12.1654	1.7379	0.0000	0.8750	0.0792
23	Ni	3.4758	8.6896	1.7379	0.2500	0.6250	0.0792
24	Ni	3.4758	12.1654	1.7379	0.2500	0.8750	0.0792
25	Ni	6.9517	1.7379	1.7379	0.5000	0.1250	0.0792
26	Ni	6.9517	5.2138	1.7379	0.5000	0.3750	0.0792
27	Ni	10.4275	1.7379	1.7379	0.7500	0.1250	0.0792
28	Ni	10.4275	5.2138	1.7379	0.7500	0.3750	0.0792
29	Ni	6.9517	8.6896	1.7379	0.5000	0.6250	0.0792
30	Ni	6.9517	12.1654	1.7379	0.5000	0.8750	0.0792
31	Ni	10.4275	8.6896	1.7379	0.7500	0.6250	0.0792
32	Ni	10.4275	12.1654	1.7379	0.7500	0.8750	0.0792
33	Ni	-0.0006	13.8999	3.5022	-0.0000	0.9998	0.1595
34	Ni	0.0053	3.4721	3.5009	0.0004	0.2497	0.1595
35	Ni	3.4774	13.9009	3.5002	0.2501	0.9998	0.1595
36	Ni	3.4627	3.4279	3.5016	0.2491	0.2466	0.1595
37	Ni	-0.0003	6.9573	3.5107	-0.0000	0.5004	0.1599
38	Ni	13.8781	10.4262	3.4802	0.9982	0.7499	0.1585
39	Ni	3.4758	6.9661	3.5029	0.2500	0.5010	0.1596
40	Ni	3.5005	10.3927	3.5112	0.2518	0.7475	0.1600

41	Ni	6.9469	0.0193	3.4990	0.4997	0.0014	0.1594
42	Ni	6.9524	3.4958	3.5177	0.5001	0.2514	0.1602
43	Ni	10.4264	0.0100	3.5024	0.7499	0.0007	0.1596
44	Ni	10.4316	3.4775	3.5037	0.7503	0.2501	0.1596
45	Ni	6.9582	6.9648	3.5055	0.5005	0.5009	0.1597
46	Ni	6.9749	10.4396	3.4700	0.5017	0.7509	0.1581
47	Ni	10.4337	6.9401	3.4899	0.7504	0.4992	0.1590
48	Ni	10.4298	10.4382	3.4923	0.7502	0.7508	0.1591
49	Ni	0.0205	1.7299	5.2130	0.0015	0.1244	0.2375
50	Ni	0.0089	5.2174	5.2157	0.0006	0.3753	0.2376
51	Ni	3.4452	1.7553	5.3002	0.2478	0.1262	0.2414
52	Ni	3.4372	5.1783	5.1059	0.2472	0.3725	0.2326
53	Ni	13.8527	8.7182	5.1853	0.9964	0.6271	0.2362
54	Ni	13.8802	12.1450	5.1969	0.9983	0.8735	0.2367
55	Ni	3.4662	8.7068	5.2649	0.2493	0.6262	0.2398
56	Ni	3.4770	12.1077	5.1891	0.2501	0.8708	0.2364
57	Ni	6.9723	1.7572	5.1963	0.5015	0.1264	0.2367
58	Ni	6.9743	5.1869	5.3699	0.5016	0.3731	0.2446
59	Ni	10.4327	1.7396	5.2048	0.7504	0.1251	0.2371
60	Ni	10.4272	5.2125	5.2127	0.7500	0.3749	0.2375
61	Ni	7.0090	8.7178	5.2211	0.5041	0.6270	0.2378
62	Ni	6.9694	12.2115	5.1658	0.5013	0.8783	0.2353
63	Ni	10.4235	8.6905	5.2044	0.7497	0.6251	0.2371
64	Ni	10.4503	12.2037	5.2516	0.7516	0.8778	0.2392
65	Co	1.7379	1.7379	0.0000	0.1250	0.1250	0.0000
66	Co	1.7379	5.2138	0.0000	0.1250	0.3750	0.0000
67	Co	5.2138	1.7379	0.0000	0.3750	0.1250	0.0000
68	Co	5.2138	5.2138	0.0000	0.3750	0.3750	0.0000
69	Co	1.7379	8.6896	0.0000	0.1250	0.6250	0.0000
70	Co	1.7379	12.1654	0.0000	0.1250	0.8750	0.0000
71	Co	5.2138	8.6896	0.0000	0.3750	0.6250	0.0000
72	Co	5.2138	12.1654	0.0000	0.3750	0.8750	0.0000
73	Co	8.6896	1.7379	0.0000	0.6250	0.1250	0.0000
74	Co	8.6896	5.2138	0.0000	0.6250	0.3750	0.0000
75	Co	12.1654	1.7379	0.0000	0.8750	0.1250	0.0000
76	Co	12.1654	5.2138	0.0000	0.8750	0.3750	0.0000
77	Co	8.6896	8.6896	0.0000	0.6250	0.6250	0.0000
78	Co	8.6896	12.1654	0.0000	0.6250	0.8750	0.0000
79	Co	12.1654	8.6896	0.0000	0.8750	0.6250	0.0000
80	Co	12.1654	12.1654	0.0000	0.8750	0.8750	0.0000
81	Co	1.7379	0.0000	1.7379	0.1250	0.0000	0.0792
82	Co	1.7379	3.4758	1.7379	0.1250	0.2500	0.0792
83	Co	5.2138	0.0000	1.7379	0.3750	0.0000	0.0792
84	Co	5.2138	3.4758	1.7379	0.3750	0.2500	0.0792
85	Co	1.7379	6.9517	1.7379	0.1250	0.5000	0.0792
86	Co	1.7379	10.4275	1.7379	0.1250	0.7500	0.0792
87	Co	5.2138	6.9517	1.7379	0.3750	0.5000	0.0792
88	Co	5.2138	10.4275	1.7379	0.3750	0.7500	0.0792
89	Co	8.6896	0.0000	1.7379	0.6250	0.0000	0.0792

90	Co	8.6896	3.4758	1.7379	0.6250	0.2500	0.0792
91	Co	12.1654	0.0000	1.7379	0.8750	0.0000	0.0792
92	Co	12.1654	3.4758	1.7379	0.8750	0.2500	0.0792
93	Co	8.6896	6.9517	1.7379	0.6250	0.5000	0.0792
94	Co	8.6896	10.4275	1.7379	0.6250	0.7500	0.0792
95	Co	12.1654	6.9517	1.7379	0.8750	0.5000	0.0792
96	Co	12.1654	10.4275	1.7379	0.8750	0.7500	0.0792
97	Co	1.7418	1.7132	3.5662	0.1253	0.1232	0.1625
98	Co	1.7107	5.2375	3.5287	0.1230	0.3767	0.1607
99	Co	5.2053	1.7272	3.5673	0.3744	0.1242	0.1625
100	Co	5.2305	5.1948	3.5278	0.3762	0.3736	0.1607
101	Co	1.7300	8.6898	3.5281	0.1244	0.6250	0.1607
102	Co	1.7351	12.1548	3.5217	0.1248	0.8742	0.1604
103	Co	5.2101	8.6823	3.5678	0.3747	0.6245	0.1625
104	Co	5.2246	12.1669	3.5569	0.3758	0.8751	0.1620
105	Co	8.6928	1.7387	3.5412	0.6252	0.1251	0.1613
106	Co	8.6687	5.2050	3.5703	0.6235	0.3744	0.1626
107	Co	12.1741	1.7416	3.5574	0.8756	0.1253	0.1621
108	Co	12.1721	5.2087	3.5600	0.8755	0.3746	0.1622
109	Co	8.6965	8.6958	3.5346	0.6255	0.6254	0.1610
110	Co	8.7062	12.1905	3.4537	0.6262	0.8768	0.1573
111	Co	12.1477	8.6892	3.5407	0.8737	0.6250	0.1613
112	Co	12.1471	12.1754	3.5599	0.8737	0.8757	0.1622
113	Co	1.7417	13.8014	5.2743	0.1253	0.9927	0.2403
114	Co	1.7242	3.4923	5.2190	0.1240	0.2512	0.2378
115	Co	5.2257	0.0019	5.2932	0.3759	0.0001	0.2411
116	Co	5.1984	3.4613	5.1980	0.3739	0.2490	0.2368
117	Co	1.7067	6.9931	5.3098	0.1228	0.5030	0.2419
118	Co	1.6953	10.4365	5.1579	0.1219	0.7506	0.2350
119	Co	5.2423	6.8846	5.1887	0.3771	0.4952	0.2364
120	Co	5.2614	10.4336	5.2218	0.3784	0.7504	0.2379
121	Co	8.6851	0.0205	5.2870	0.6247	0.0015	0.2408
122	Co	8.7093	3.4593	5.2271	0.6264	0.2488	0.2381
123	Co	12.1918	0.0171	5.2131	0.8769	0.0012	0.2375
124	Co	12.1751	3.4715	5.2251	0.8757	0.2497	0.2380
125	Co	8.7254	6.9659	5.1785	0.6276	0.5010	0.2359
126	Co	8.7452	10.4788	5.1940	0.6290	0.7537	0.2366
127	Co	12.1566	6.9514	5.2194	0.8744	0.5000	0.2378
128	Co	12.1490	10.4334	5.2158	0.8738	0.7504	0.2376
129	O	5.8256	6.0111	6.8124	0.4190	0.4324	0.3103
130	O	2.5169	7.9621	6.7980	0.1810	0.5727	0.3097
131	O	4.8166	7.9823	8.7382	0.3464	0.5741	0.3981
132	O	6.2422	9.7513	6.8081	0.4490	0.7014	0.3101
133	O	8.2008	9.3864	8.9166	0.5898	0.6751	0.4062
134	O	5.5353	11.3798	9.0268	0.3981	0.8185	0.4112
135	O	8.6545	12.1817	6.3119	0.6225	0.8762	0.2875
136	Ce	4.0831	10.2039	8.0568	0.2937	0.7339	0.3670
137	Ce	3.7103	6.3253	7.7455	0.2669	0.4549	0.3528
138	Ce	7.0570	7.7857	8.0241	0.5076	0.5600	0.3655

139 Sm	7.4882	11.2241	8.1656	0.5386	0.8073	0.3720
140 C	3.0850	13.2906	6.7335	0.2219	0.9559	0.3067
141 O	3.9388	0.4532	6.7945	0.2833	0.0326	0.3095
142 O	2.8701	12.5718	7.7526	0.2064	0.9042	0.3532

/db/jmorales/CoNi-alloy/Profiles/Ni-Ce_3SmO_7/TS-1

a =			13.901983737				
b =			13.901983737				
c =			21.950991869				
alpha =			90.0				
beta =			90.0				
gamma =			90.0				
	Atom	X	Y	Z	X	Y	Z
1	Ni	0.0000	0.0000	0.0000	0.0000	0.0000	0.0000
2	Ni	1.7378	1.7378	0.0000	0.1250	0.1250	0.0000
3	Ni	0.0000	3.4755	0.0000	0.0000	0.2500	0.0000
4	Ni	1.7378	5.2132	0.0000	0.1250	0.3750	0.0000
5	Ni	3.4755	0.0000	0.0000	0.2500	0.0000	0.0000
6	Ni	5.2132	1.7378	0.0000	0.3750	0.1250	0.0000
7	Ni	3.4755	3.4755	0.0000	0.2500	0.2500	0.0000
8	Ni	5.2132	5.2132	0.0000	0.3750	0.3750	0.0000
9	Ni	0.0000	6.9510	0.0000	0.0000	0.5000	0.0000
10	Ni	1.7378	8.6887	0.0000	0.1250	0.6250	0.0000
11	Ni	0.0000	10.4265	0.0000	0.0000	0.7500	0.0000
12	Ni	1.7378	12.1642	0.0000	0.1250	0.8750	0.0000
13	Ni	3.4755	6.9510	0.0000	0.2500	0.5000	0.0000
14	Ni	5.2132	8.6887	0.0000	0.3750	0.6250	0.0000
15	Ni	3.4755	10.4265	0.0000	0.2500	0.7500	0.0000
16	Ni	5.2132	12.1642	0.0000	0.3750	0.8750	0.0000
17	Ni	6.9510	0.0000	0.0000	0.5000	0.0000	0.0000
18	Ni	8.6887	1.7378	0.0000	0.6250	0.1250	0.0000
19	Ni	6.9510	3.4755	0.0000	0.5000	0.2500	0.0000
20	Ni	8.6887	5.2132	0.0000	0.6250	0.3750	0.0000
21	Ni	10.4265	0.0000	0.0000	0.7500	0.0000	0.0000
22	Ni	12.1642	1.7378	0.0000	0.8750	0.1250	0.0000
23	Ni	10.4265	3.4755	0.0000	0.7500	0.2500	0.0000
24	Ni	12.1642	5.2132	0.0000	0.8750	0.3750	0.0000
25	Ni	6.9510	6.9510	0.0000	0.5000	0.5000	0.0000
26	Ni	8.6887	8.6887	0.0000	0.6250	0.6250	0.0000
27	Ni	6.9510	10.4265	0.0000	0.5000	0.7500	0.0000
28	Ni	8.6887	12.1642	0.0000	0.6250	0.8750	0.0000
29	Ni	10.4265	6.9510	0.0000	0.7500	0.5000	0.0000
30	Ni	12.1642	8.6887	0.0000	0.8750	0.6250	0.0000
31	Ni	10.4265	10.4265	0.0000	0.7500	0.7500	0.0000
32	Ni	12.1642	12.1642	0.0000	0.8750	0.8750	0.0000
33	Ni	0.0000	1.7378	1.7378	0.0000	0.1250	0.0792
34	Ni	1.7378	0.0000	1.7378	0.1250	0.0000	0.0792
35	Ni	0.0000	5.2132	1.7378	0.0000	0.3750	0.0792
36	Ni	1.7378	3.4755	1.7378	0.1250	0.2500	0.0792
37	Ni	3.4755	1.7378	1.7378	0.2500	0.1250	0.0792
38	Ni	5.2132	0.0000	1.7378	0.3750	0.0000	0.0792
39	Ni	3.4755	5.2132	1.7378	0.2500	0.3750	0.0792
40	Ni	5.2132	3.4755	1.7378	0.3750	0.2500	0.0792

41	Ni	0.0000	8.6887	1.7378	0.0000	0.6250	0.0792
42	Ni	1.7378	6.9510	1.7378	0.1250	0.5000	0.0792
43	Ni	0.0000	12.1642	1.7378	0.0000	0.8750	0.0792
44	Ni	1.7378	10.4265	1.7378	0.1250	0.7500	0.0792
45	Ni	3.4755	8.6887	1.7378	0.2500	0.6250	0.0792
46	Ni	5.2132	6.9510	1.7378	0.3750	0.5000	0.0792
47	Ni	3.4755	12.1642	1.7378	0.2500	0.8750	0.0792
48	Ni	5.2132	10.4265	1.7378	0.3750	0.7500	0.0792
49	Ni	6.9510	1.7378	1.7378	0.5000	0.1250	0.0792
50	Ni	8.6887	0.0000	1.7378	0.6250	0.0000	0.0792
51	Ni	6.9510	5.2132	1.7378	0.5000	0.3750	0.0792
52	Ni	8.6887	3.4755	1.7378	0.6250	0.2500	0.0792
53	Ni	10.4265	1.7378	1.7378	0.7500	0.1250	0.0792
54	Ni	12.1642	0.0000	1.7378	0.8750	0.0000	0.0792
55	Ni	10.4265	5.2132	1.7378	0.7500	0.3750	0.0792
56	Ni	12.1642	3.4755	1.7378	0.8750	0.2500	0.0792
57	Ni	6.9510	8.6887	1.7378	0.5000	0.6250	0.0792
58	Ni	8.6887	6.9510	1.7378	0.6250	0.5000	0.0792
59	Ni	6.9510	12.1642	1.7378	0.5000	0.8750	0.0792
60	Ni	8.6887	10.4265	1.7378	0.6250	0.7500	0.0792
61	Ni	10.4265	8.6887	1.7378	0.7500	0.6250	0.0792
62	Ni	12.1642	6.9510	1.7378	0.8750	0.5000	0.0792
63	Ni	10.4265	12.1642	1.7378	0.7500	0.8750	0.0792
64	Ni	12.1642	10.4265	1.7378	0.8750	0.7500	0.0792
65	Ni	0.0112	0.0052	3.5173	0.0008	0.0004	0.1602
66	Ni	1.7340	1.7368	3.5188	0.1247	0.1249	0.1603
67	Ni	13.8803	3.4718	3.5286	0.9984	0.2497	0.1607
68	Ni	1.6876	5.2005	3.4983	0.1214	0.3741	0.1594
69	Ni	3.4780	13.9008	3.5268	0.2502	0.9999	0.1607
70	Ni	5.2194	1.7438	3.5403	0.3754	0.1254	0.1613
71	Ni	3.4985	3.4461	3.5094	0.2517	0.2479	0.1599
72	Ni	5.2212	5.2108	3.4955	0.3756	0.3748	0.1592
73	Ni	13.8984	6.9436	3.5177	0.9997	0.4995	0.1603
74	Ni	1.7526	8.6927	3.5228	0.1261	0.6253	0.1605
75	Ni	13.8881	10.4334	3.5312	0.9990	0.7505	0.1609
76	Ni	1.7508	12.1649	3.5203	0.1259	0.8750	0.1604
77	Ni	3.4684	6.9697	3.4868	0.2495	0.5013	0.1588
78	Ni	5.2245	8.7001	3.5110	0.3758	0.6258	0.1599
79	Ni	3.4749	10.3983	3.5422	0.2500	0.7480	0.1614
80	Ni	5.2060	12.1650	3.5102	0.3745	0.8751	0.1599
81	Ni	6.9424	13.8921	3.5130	0.4994	0.9993	0.1600
82	Ni	8.6887	1.7135	3.4972	0.6250	0.1233	0.1593
83	Ni	6.9457	3.4616	3.5061	0.4996	0.2490	0.1597
84	Ni	8.6638	5.2412	3.5080	0.6232	0.3770	0.1598
85	Ni	10.4389	13.8750	3.5293	0.7509	0.9981	0.1608
86	Ni	12.1631	1.7400	3.5142	0.8749	0.1252	0.1601
87	Ni	10.4222	3.4664	3.4914	0.7497	0.2493	0.1591
88	Ni	12.1674	5.2107	3.5051	0.8752	0.3748	0.1597
89	Ni	6.9333	6.9458	3.5140	0.4987	0.4996	0.1601

90	Ni	8.6931	8.6845	3.4708	0.6253	0.6247	0.1581
91	Ni	6.9703	10.4290	3.5168	0.5014	0.7502	0.1602
92	Ni	8.6913	12.1789	3.4999	0.6252	0.8761	0.1594
93	Ni	10.4252	6.9567	3.5173	0.7499	0.5004	0.1602
94	Ni	12.1888	8.6670	3.5047	0.8768	0.6234	0.1597
95	Ni	10.4465	10.4419	3.4368	0.7514	0.7511	0.1566
96	Ni	12.1790	12.1696	3.5110	0.8761	0.8754	0.1599
97	Ni	0.0011	1.7427	5.2357	0.0001	0.1254	0.2385
98	Ni	1.7452	0.0013	5.2461	0.1255	0.0001	0.2390
99	Ni	0.0325	5.1912	5.3792	0.0023	0.3734	0.2451
100	Ni	1.7449	3.4731	5.2222	0.1255	0.2498	0.2379
101	Ni	3.4762	1.7420	5.2438	0.2501	0.1253	0.2389
102	Ni	5.2077	13.8984	5.2442	0.3746	0.9997	0.2389
103	Ni	3.4363	5.1780	5.0966	0.2472	0.3725	0.2322
104	Ni	5.2313	3.4422	5.3713	0.3763	0.2476	0.2447
105	Ni	0.0294	8.6950	5.2187	0.0021	0.6255	0.2377
106	Ni	1.7354	6.9343	5.1964	0.1248	0.4988	0.2367
107	Ni	0.0104	12.1777	5.2340	0.0008	0.8760	0.2384
108	Ni	1.7260	10.4361	5.2392	0.1242	0.7507	0.2387
109	Ni	3.4497	8.7302	5.4123	0.2481	0.6280	0.2466
110	Ni	5.1770	6.9617	5.2330	0.3724	0.5008	0.2384
111	Ni	3.4822	12.1474	5.2473	0.2505	0.8738	0.2390
112	Ni	5.2325	10.4363	5.2115	0.3764	0.7507	0.2374
113	Ni	6.9830	1.6877	5.2262	0.5023	0.1214	0.2381
114	Ni	8.6906	13.8848	5.2318	0.6251	0.9988	0.2383
115	Ni	6.9547	5.2109	5.2729	0.5003	0.3748	0.2402
116	Ni	8.6968	3.4522	5.1668	0.6256	0.2483	0.2354
117	Ni	10.4161	1.7036	5.2234	0.7493	0.1225	0.2380
118	Ni	12.1805	0.0127	5.2305	0.8762	0.0009	0.2383
119	Ni	10.3735	5.1925	5.2461	0.7462	0.3735	0.2390
120	Ni	12.1070	3.4592	5.3215	0.8709	0.2488	0.2424
121	Ni	6.9786	8.7336	5.2449	0.5020	0.6282	0.2389
122	Ni	8.7172	6.9582	5.2782	0.6270	0.5005	0.2405
123	Ni	6.9391	12.1615	5.2394	0.4991	0.8748	0.2387
124	Ni	8.7142	10.4669	5.2049	0.6268	0.7529	0.2371
125	Ni	10.4682	8.7077	5.1722	0.7530	0.6264	0.2356
126	Ni	12.1494	6.9565	5.3479	0.8739	0.5004	0.2436
127	Ni	10.4519	12.1777	5.2905	0.7518	0.8760	0.2410
128	Ni	12.1927	10.4294	5.2886	0.8770	0.7502	0.2409
129	O	6.2164	4.3502	6.8059	0.4472	0.3129	0.3101
130	O	4.1862	7.5824	6.7897	0.3011	0.5454	0.3093
131	O	6.1179	6.5778	8.7394	0.4401	0.4732	0.3981
132	O	7.8992	7.9150	6.7924	0.5682	0.5693	0.3094
133	O	10.4229	10.3808	6.2594	0.7497	0.7467	0.2852
134	O	9.5556	7.1748	8.9596	0.6874	0.5161	0.4082
135	O	7.6272	9.8901	8.8789	0.5486	0.7114	0.4045
136	Ce	5.9667	8.8248	8.0097	0.4292	0.6348	0.3649
137	Ce	4.4740	5.4692	7.7104	0.3218	0.3934	0.3513
138	Ce	8.2736	5.7710	8.0482	0.5951	0.4151	0.3666

139 Sm	9.4298	9.1287	8.0851	0.6783	0.6566	0.3683
140 C	11.3515	4.5325	6.7430	0.8165	0.3260	0.3072
141 O	12.7243	5.7689	6.6384	0.9153	0.4150	0.3024
142 O	10.9757	4.3667	7.8817	0.7895	0.3141	0.3591

/db/jmorales/CoNi-alloy/Profiles/Ni-CoNi(001)/CO-O

a = 6.951669213
b = 6.951669213
c = 21.951669213
alpha = 90.0
beta = 90.0
gamma = 90.0

	Atom	X	Y	Z	X	Y	Z
1	Co	0.0000	0.0000	0.0000	0.0000	0.0000	0.0000
2	Co	1.7379	1.7379	0.0000	0.2500	0.2500	0.0000
3	Co	0.0000	3.4758	0.0000	0.0000	0.5000	0.0000
4	Co	1.7379	5.2138	0.0000	0.2500	0.7500	0.0000
5	Co	3.4758	0.0000	0.0000	0.5000	0.0000	0.0000
6	Co	5.2138	1.7379	0.0000	0.7500	0.2500	0.0000
7	Co	3.4758	3.4758	0.0000	0.5000	0.5000	0.0000
8	Co	5.2138	5.2138	0.0000	0.7500	0.7500	0.0000
9	Co	6.9452	0.0073	3.5196	0.9991	0.0010	0.1603
10	Co	1.7366	1.7499	3.4758	0.2498	0.2517	0.1583
11	Co	0.0122	3.4645	3.5330	0.0018	0.4984	0.1609
12	Co	1.7342	5.2174	3.4758	0.2495	0.7505	0.1583
13	Co	3.4856	6.9383	3.5322	0.5014	0.9981	0.1609
14	Co	5.2176	1.7335	3.4758	0.7506	0.2494	0.1583
15	Co	3.4845	3.4660	3.5066	0.5012	0.4986	0.1597
16	Co	5.2019	5.2144	3.4758	0.7483	0.7501	0.1583
17	Ni	1.7379	0.0000	1.7379	0.2500	0.0000	0.0792
18	Ni	0.0000	1.7379	1.7379	0.0000	0.2500	0.0792
19	Ni	1.7379	3.4758	1.7379	0.2500	0.5000	0.0792
20	Ni	0.0000	5.2138	1.7379	0.0000	0.7500	0.0792
21	Ni	5.2138	0.0000	1.7379	0.7500	0.0000	0.0792
22	Ni	3.4758	1.7379	1.7379	0.5000	0.2500	0.0792
23	Ni	5.2138	3.4758	1.7379	0.7500	0.5000	0.0792
24	Ni	3.4758	5.2138	1.7379	0.5000	0.7500	0.0792
25	Ni	1.7295	0.0098	5.1611	0.2488	0.0014	0.2351
26	Ni	0.0084	1.7376	5.2686	0.0012	0.2500	0.2400
27	Ni	1.7661	3.4971	5.2443	0.2541	0.5031	0.2389
28	Ni	6.9404	5.2200	5.1616	0.9984	0.7509	0.2351
29	Ni	5.2135	6.9433	5.2680	0.7500	0.9988	0.2400
30	Ni	3.4815	1.7182	5.2498	0.5008	0.2472	0.2392
31	Ni	5.2321	3.4694	5.2499	0.7526	0.4991	0.2392
32	Ni	3.4540	5.1862	5.2452	0.4969	0.7460	0.2389
33	O	2.6806	4.2718	7.9018	0.3856	0.6145	0.3600
34	C	2.6454	4.3060	6.7249	0.3805	0.6194	0.3063
35	O	5.2278	1.7219	6.1511	0.7520	0.2477	0.2802

/db/jmorales/CoNi-alloy/Profiles/Sm(surf)$Ce_{26}O_{54}$/surface

a =			11.6082000732				
b =			11.608200073110298				
c =			22.898399353				
alpha =			90.0				
beta =			90.0				
gamma =			120.0				
	Atom	X	Y	Z	X	Y	Z
1	Sm	1.9347	3.3510	7.1020	0.3333	0.3333	0.3102
2	Ce	1.9347	1.1170	0.7898	0.2222	0.1111	0.0345
3	Ce	0.0000	4.4680	0.7898	0.2222	0.4444	0.0345
4	Ce	-1.9347	7.8190	0.7898	0.2222	0.7778	0.0345
5	Ce	5.8041	1.1170	0.7898	0.5556	0.1111	0.0345
6	Ce	3.8694	4.4680	0.7898	0.5556	0.4444	0.0345
7	Ce	1.9347	7.8190	0.7898	0.5556	0.7778	0.0345
8	Ce	9.6735	1.1170	0.7898	0.8889	0.1111	0.0345
9	Ce	7.7388	4.4680	0.7898	0.8889	0.4444	0.0345
10	Ce	5.8041	7.8190	0.7898	0.8889	0.7778	0.0345
11	Ce	0.0040	2.2363	3.9679	0.1116	0.2225	0.1733
12	Ce	-1.9346	5.5850	3.9498	0.1111	0.5556	0.1725
13	Ce	-3.8694	8.9361	3.9498	0.1111	0.8889	0.1725
14	Ce	3.8654	2.2363	3.9679	0.4442	0.2225	0.1733
15	Ce	1.9347	5.5804	3.9679	0.4442	0.5551	0.1733
16	Ce	-0.0001	8.9360	3.9593	0.4444	0.8889	0.1729
17	Ce	7.7388	2.2341	3.9593	0.7778	0.2222	0.1729
18	Ce	5.8040	5.5850	3.9498	0.7778	0.5556	0.1725
19	Ce	3.8695	8.9360	3.9593	0.7778	0.8889	0.1729
20	Ce	0.0083	0.0005	7.1082	0.0007	0.0001	0.3104
21	Ce	-1.9301	3.3580	7.1082	0.0007	0.3340	0.3104
22	Ce	7.7388	6.7020	7.0965	1.0000	0.6667	0.3099
23	Ce	3.8610	0.0005	7.1082	0.3326	0.0001	0.3104
24	Ce	-0.0037	6.6945	7.1082	0.3326	0.6659	0.3104
25	Ce	1.9347	10.0530	7.1179	0.6667	1.0000	0.3108
26	Ce	5.7995	3.3580	7.1082	0.6666	0.3340	0.3104
27	Ce	3.8731	6.6945	7.1082	0.6666	0.6659	0.3104
28	O	0.0000	0.0000	0.0000	0.0000	0.0000	0.0000
29	O	-1.9347	3.3510	0.0000	-0.0000	0.3333	0.0000
30	O	-3.8694	6.7020	0.0000	-0.0000	0.6667	0.0000
31	O	3.8694	0.0000	0.0000	0.3333	0.0000	0.0000
32	O	1.9347	3.3510	0.0000	0.3333	0.3333	0.0000
33	O	0.0000	6.7020	0.0000	0.3333	0.6667	0.0000
34	O	7.7388	0.0000	0.0000	0.6667	0.0000	0.0000
35	O	5.8041	3.3510	0.0000	0.6667	0.3333	0.0000
36	O	3.8694	6.7020	0.0000	0.6667	0.6667	0.0000
37	O	-0.0000	2.2340	1.5798	0.1111	0.2222	0.0690
38	O	-1.9347	5.5850	1.5798	0.1111	0.5556	0.0690
39	O	-3.8694	8.9360	1.5798	0.1111	0.8889	0.0690
40	O	3.8694	2.2340	1.5798	0.4444	0.2222	0.0690

41	O	1.9347	5.5850	1.5798	0.4444	0.5556	0.0690
42	O	-0.0000	8.9360	1.5798	0.4444	0.8889	0.0690
43	O	7.7388	2.2340	1.5798	0.7778	0.2222	0.0690
44	O	5.8041	5.5850	1.5798	0.7778	0.5556	0.0690
45	O	3.8694	8.9360	1.5798	0.7778	0.8889	0.0690
46	O	1.9347	1.1170	3.1593	0.2222	0.1111	0.1380
47	O	0.0000	4.4680	3.1593	0.2222	0.4444	0.1380
48	O	-1.9347	7.8190	3.1593	0.2222	0.7778	0.1380
49	O	5.8041	1.1170	3.1593	0.5556	0.1111	0.1380
50	O	3.8694	4.4680	3.1593	0.5556	0.4444	0.1380
51	O	1.9347	7.8190	3.1593	0.5556	0.7778	0.1380
52	O	9.6735	1.1170	3.1593	0.8889	0.1111	0.1380
53	O	7.7388	4.4680	3.1593	0.8889	0.4444	0.1380
54	O	5.8041	7.8190	3.1593	0.8889	0.7778	0.1380
55	O	0.0016	-0.0042	4.7595	-0.0001	-0.0004	0.2079
56	O	-1.9375	3.3545	4.7595	-0.0001	0.3337	0.2079
57	O	-3.8694	6.7020	4.7484	0.0000	0.6667	0.2074
58	O	3.8678	-0.0042	4.7595	0.3330	-0.0004	0.2079
59	O	1.9347	3.3510	4.7534	0.3333	0.3333	0.2076
60	O	-0.0044	6.7027	4.7595	0.3330	0.6667	0.2079
61	O	7.7388	0.0000	4.7639	0.6667	0.0000	0.2080
62	O	5.8069	3.3545	4.7595	0.6671	0.3337	0.2079
63	O	3.8738	6.7027	4.7595	0.6671	0.6667	0.2079
64	O	-0.0338	2.2145	6.3058	0.1072	0.2203	0.2754
65	O	-1.9509	5.5944	6.2828	0.1102	0.5565	0.2744
66	O	-3.8694	8.9173	6.2828	0.1102	0.8870	0.2744
67	O	3.9032	2.2145	6.3058	0.4464	0.2203	0.2754
68	O	1.9347	5.6240	6.3058	0.4464	0.5594	0.2754
69	O	0.0004	8.9363	6.3032	0.4445	0.8889	0.2753
70	O	7.7388	2.2335	6.3032	0.7778	0.2222	0.2753
71	O	5.8203	5.5944	6.2828	0.7796	0.5565	0.2744
72	O	3.8690	8.9363	6.3032	0.7778	0.8889	0.2753
73	O	1.9347	1.0203	7.8989	0.2174	0.1015	0.3450
74	O	-0.0837	4.5163	7.8989	0.2174	0.4493	0.3450
75	O	-1.9405	7.8156	7.8936	0.2216	0.7774	0.3447
76	O	5.8157	1.1103	7.9062	0.5562	0.1104	0.3453
77	O	3.9531	4.5163	7.8989	0.5652	0.4493	0.3450
78	O	1.9347	7.8324	7.9062	0.5562	0.7791	0.3453
79	O	9.6619	1.1103	7.9062	0.8876	0.1104	0.3453
80	O	7.7388	4.4747	7.8936	0.8892	0.4451	0.3447
81	O	5.8099	7.8156	7.8936	0.8892	0.7774	0.3447

/db/jmorales/CoNi-alloy/Profiles/Ni-Ce_3SmO_7/surface

a =			13.9019837372				
b =			13.9019837372				
c =			21.9509918687				
alpha =			90.0				
beta =			90.0				
gamma =			90.0				
	Atom	X	Y	Z	X	Y	Z
1	Ni	0.0000	0.0000	0.0000	0.0000	0.0000	0.0000
2	Ni	1.7377	1.7377	0.0000	0.1250	0.1250	0.0000
3	Ni	0.0000	3.4755	0.0000	0.0000	0.2500	0.0000
4	Ni	1.7377	5.2132	0.0000	0.1250	0.3750	0.0000
5	Ni	3.4755	0.0000	0.0000	0.2500	0.0000	0.0000
6	Ni	5.2132	1.7377	0.0000	0.3750	0.1250	0.0000
7	Ni	3.4755	3.4755	0.0000	0.2500	0.2500	0.0000
8	Ni	5.2132	5.2132	0.0000	0.3750	0.3750	0.0000
9	Ni	0.0000	6.9510	0.0000	0.0000	0.5000	0.0000
10	Ni	1.7377	8.6887	0.0000	0.1250	0.6250	0.0000
11	Ni	0.0000	10.4265	0.0000	0.0000	0.7500	0.0000
12	Ni	1.7377	12.1642	0.0000	0.1250	0.8750	0.0000
13	Ni	3.4755	6.9510	0.0000	0.2500	0.5000	0.0000
14	Ni	5.2132	8.6887	0.0000	0.3750	0.6250	0.0000
15	Ni	3.4755	10.4265	0.0000	0.2500	0.7500	0.0000
16	Ni	5.2132	12.1642	0.0000	0.3750	0.8750	0.0000
17	Ni	6.9510	0.0000	0.0000	0.5000	0.0000	0.0000
18	Ni	8.6887	1.7377	0.0000	0.6250	0.1250	0.0000
19	Ni	6.9510	3.4755	0.0000	0.5000	0.2500	0.0000
20	Ni	8.6887	5.2132	0.0000	0.6250	0.3750	0.0000
21	Ni	10.4265	0.0000	0.0000	0.7500	0.0000	0.0000
22	Ni	12.1642	1.7377	0.0000	0.8750	0.1250	0.0000
23	Ni	10.4265	3.4755	0.0000	0.7500	0.2500	0.0000
24	Ni	12.1642	5.2132	0.0000	0.8750	0.3750	0.0000
25	Ni	6.9510	6.9510	0.0000	0.5000	0.5000	0.0000
26	Ni	8.6887	8.6887	0.0000	0.6250	0.6250	0.0000
27	Ni	6.9510	10.4265	0.0000	0.5000	0.7500	0.0000
28	Ni	8.6887	12.1642	0.0000	0.6250	0.8750	0.0000
29	Ni	10.4265	6.9510	0.0000	0.7500	0.5000	0.0000
30	Ni	12.1642	8.6887	0.0000	0.8750	0.6250	0.0000
31	Ni	10.4265	10.4265	0.0000	0.7500	0.7500	0.0000
32	Ni	12.1642	12.1642	0.0000	0.8750	0.8750	0.0000
33	Ni	0.0000	1.7377	1.7377	0.0000	0.1250	0.0792
34	Ni	1.7377	0.0000	1.7377	0.1250	0.0000	0.0792
35	Ni	0.0000	5.2132	1.7377	0.0000	0.3750	0.0792
36	Ni	1.7377	3.4755	1.7377	0.1250	0.2500	0.0792
37	Ni	3.4755	1.7377	1.7377	0.2500	0.1250	0.0792
38	Ni	5.2132	0.0000	1.7377	0.3750	0.0000	0.0792
39	Ni	3.4755	5.2132	1.7377	0.2500	0.3750	0.0792
40	Ni	5.2132	3.4755	1.7377	0.3750	0.2500	0.0792

41	Ni	0.0000	8.6887	1.7377	0.0000	0.6250	0.0792
42	Ni	1.7377	6.9510	1.7377	0.1250	0.5000	0.0792
43	Ni	0.0000	12.1642	1.7377	0.0000	0.8750	0.0792
44	Ni	1.7377	10.4265	1.7377	0.1250	0.7500	0.0792
45	Ni	3.4755	8.6887	1.7377	0.2500	0.6250	0.0792
46	Ni	5.2132	6.9510	1.7377	0.3750	0.5000	0.0792
47	Ni	3.4755	12.1642	1.7377	0.2500	0.8750	0.0792
48	Ni	5.2132	10.4265	1.7377	0.3750	0.7500	0.0792
49	Ni	6.9510	1.7377	1.7377	0.5000	0.1250	0.0792
50	Ni	8.6887	0.0000	1.7377	0.6250	0.0000	0.0792
51	Ni	6.9510	5.2132	1.7377	0.5000	0.3750	0.0792
52	Ni	8.6887	3.4755	1.7377	0.6250	0.2500	0.0792
53	Ni	10.4265	1.7377	1.7377	0.7500	0.1250	0.0792
54	Ni	12.1642	0.0000	1.7377	0.8750	0.0000	0.0792
55	Ni	10.4265	5.2132	1.7377	0.7500	0.3750	0.0792
56	Ni	12.1642	3.4755	1.7377	0.8750	0.2500	0.0792
57	Ni	6.9510	8.6887	1.7377	0.5000	0.6250	0.0792
58	Ni	8.6887	6.9510	1.7377	0.6250	0.5000	0.0792
59	Ni	6.9510	12.1642	1.7377	0.5000	0.8750	0.0792
60	Ni	8.6887	10.4265	1.7377	0.6250	0.7500	0.0792
61	Ni	10.4265	8.6887	1.7377	0.7500	0.6250	0.0792
62	Ni	12.1642	6.9510	1.7377	0.8750	0.5000	0.0792
63	Ni	10.4265	12.1642	1.7377	0.7500	0.8750	0.0792
64	Ni	12.1642	10.4265	1.7377	0.8750	0.7500	0.0792
65	Ni	0.0027	0.0027	3.5083	0.0002	0.0002	0.1598
66	Ni	1.7353	1.7351	3.5085	0.1248	0.1248	0.1598
67	Ni	13.8930	3.4655	3.5086	0.9994	0.2493	0.1598
68	Ni	1.7052	5.1996	3.4730	0.1227	0.3740	0.1582
69	Ni	3.4785	0.0018	3.5119	0.2502	0.0001	0.1600
70	Ni	5.2217	1.7491	3.5330	0.3756	0.1258	0.1610
71	Ni	3.4927	3.4400	3.4981	0.2512	0.2474	0.1594
72	Ni	5.2140	5.2055	3.4923	0.3751	0.3744	0.1591
73	Ni	13.8992	6.9495	3.5022	0.9998	0.4999	0.1595
74	Ni	1.7432	8.6938	3.5214	0.1254	0.6254	0.1604
75	Ni	13.8868	10.4292	3.5251	0.9989	0.7502	0.1606
76	Ni	1.7411	12.1631	3.5086	0.1252	0.8749	0.1598
77	Ni	3.4614	6.9753	3.4832	0.2490	0.5017	0.1587
78	Ni	5.2205	8.7015	3.5040	0.3755	0.6259	0.1596
79	Ni	3.4703	10.4036	3.5308	0.2496	0.7484	0.1609
80	Ni	5.2105	12.1663	3.5030	0.3748	0.8751	0.1596
81	Ni	6.9486	13.9002	3.5058	0.4998	0.9999	0.1597
82	Ni	8.6982	1.7293	3.5026	0.6257	0.1244	0.1596
83	Ni	6.9472	3.4668	3.5075	0.4997	0.2494	0.1598
84	Ni	8.6680	5.2301	3.5248	0.6235	0.3762	0.1606
85	Ni	10.4348	13.8808	3.5280	0.7506	0.9985	0.1607
86	Ni	12.1612	1.7348	3.5088	0.8748	0.1248	0.1598
87	Ni	10.4281	3.4785	3.5033	0.7501	0.2502	0.1596
88	Ni	12.1617	5.2126	3.5088	0.8748	0.3750	0.1598
89	Ni	6.9289	6.9394	3.5157	0.4984	0.4992	0.1602

90	Ni	8.6923	8.6831	3.4762	0.6253	0.6246	0.1584
91	Ni	6.9756	10.4244	3.5147	0.5018	0.7498	0.1601
92	Ni	8.6947	12.1754	3.4989	0.6254	0.8758	0.1594
93	Ni	10.4155	6.9562	3.5206	0.7492	0.5004	0.1604
94	Ni	12.1825	8.6868	3.4910	0.8763	0.6249	0.1590
95	Ni	10.4394	10.4401	3.4368	0.7509	0.7510	0.1566
96	Ni	12.1692	12.1660	3.5089	0.8754	0.8751	0.1599
97	Ni	0.0025	1.7356	5.2282	0.0002	0.1248	0.2382
98	Ni	1.7380	13.8988	5.2256	0.1250	0.9998	0.2381
99	Ni	13.8984	5.2009	5.2249	0.9997	0.3741	0.2380
100	Ni	1.7390	3.4740	5.2190	0.1251	0.2499	0.2378
101	Ni	3.4666	1.7310	5.2335	0.2494	0.1245	0.2384
102	Ni	5.2131	-0.0002	5.2275	0.3750	-0.0000	0.2381
103	Ni	3.4303	5.1810	5.0813	0.2468	0.3727	0.2315
104	Ni	5.1978	3.4355	5.3750	0.3739	0.2471	0.2449
105	Ni	0.0077	8.6797	5.2222	0.0006	0.6243	0.2379
106	Ni	1.7105	6.9148	5.1995	0.1230	0.4974	0.2369
107	Ni	0.0001	12.1752	5.2236	0.0000	0.8758	0.2380
108	Ni	1.7173	10.4426	5.2305	0.1235	0.7512	0.2383
109	Ni	3.4349	8.7376	5.3800	0.2471	0.6285	0.2451
110	Ni	5.1660	6.9660	5.2221	0.3716	0.5011	0.2379
111	Ni	3.4805	12.1560	5.2300	0.2504	0.8744	0.2383
112	Ni	5.2316	10.4427	5.1994	0.3763	0.7512	0.2369
113	Ni	6.9816	1.7096	5.2144	0.5022	0.1230	0.2375
114	Ni	8.6929	13.8944	5.2229	0.6253	0.9995	0.2379
115	Ni	6.9280	5.1900	5.2683	0.4983	0.3733	0.2400
116	Ni	8.7144	3.4734	5.2027	0.6268	0.2499	0.2370
117	Ni	10.4248	1.7332	5.2270	0.7499	0.1247	0.2381
118	Ni	12.1715	0.0075	5.2261	0.8755	0.0005	0.2381
119	Ni	10.4205	5.2112	5.2246	0.7496	0.3749	0.2380
120	Ni	12.1591	3.4735	5.2260	0.8746	0.2499	0.2381
121	Ni	6.9678	8.7303	5.2396	0.5012	0.6280	0.2387
122	Ni	8.7072	6.9414	5.2814	0.6263	0.4993	0.2406
123	Ni	6.9486	12.1626	5.2219	0.4998	0.8749	0.2379
124	Ni	8.7156	10.4559	5.2177	0.6269	0.7521	0.2377
125	Ni	10.4611	8.6955	5.1815	0.7525	0.6255	0.2360
126	Ni	12.1648	6.9460	5.2195	0.8750	0.4996	0.2378
127	Ni	10.4457	12.1664	5.2916	0.7514	0.8752	0.2411
128	Ni	12.1846	10.4248	5.2900	0.8765	0.7499	0.2410
129	O	6.0852	4.3903	6.8265	0.4377	0.3158	0.3110
130	O	4.1629	7.6327	6.7758	0.2994	0.5490	0.3087
131	O	5.9596	6.6067	8.7799	0.4287	0.4752	0.4000
132	O	7.8078	7.8466	6.8076	0.5616	0.5644	0.3101
133	O	10.4249	10.3605	6.2835	0.7499	0.7453	0.2863
134	O	9.5368	6.9696	8.8726	0.6860	0.5013	0.4042
135	O	7.6736	9.7625	8.9154	0.5520	0.7022	0.4061
136	Ce	5.9357	8.8538	8.0210	0.4270	0.6369	0.3654
137	Ce	4.3158	5.5215	7.6861	0.3104	0.3972	0.3501
138	Ce	8.0203	5.7308	8.0348	0.5769	0.4122	0.3660

139 Sm	9.4319	8.9601	8.0432	0.6785	0.6445	0.3664

/db/jmorales/CoNi-alloy/Profiles/CoNi-Ce_3SmO_7/O-coverage/M-CO2-O-coverage/M-CO_2-CoNi-Ce_3SmO_7-2O

a =	13.9033384264
b =	13.9033384264
c =	21.9516692133
alpha =	90.0
beta =	90.0
gamma =	90.0

	Atom	X	Y	Z	X	Y	Z
1	Ni	0.0000	0.0000	0.0000	0.0000	0.0000	0.0000
2	Ni	0.0000	3.4758	0.0000	0.0000	0.2500	0.0000
3	Ni	3.4758	0.0000	0.0000	0.2500	0.0000	0.0000
4	Ni	3.4758	3.4758	0.0000	0.2500	0.2500	0.0000
5	Ni	0.0000	6.9517	0.0000	0.0000	0.5000	0.0000
6	Ni	0.0000	10.4275	0.0000	0.0000	0.7500	0.0000
7	Ni	3.4758	6.9517	0.0000	0.2500	0.5000	0.0000
8	Ni	3.4758	10.4275	0.0000	0.2500	0.7500	0.0000
9	Ni	6.9517	0.0000	0.0000	0.5000	0.0000	0.0000
10	Ni	6.9517	3.4758	0.0000	0.5000	0.2500	0.0000
11	Ni	10.4275	0.0000	0.0000	0.7500	0.0000	0.0000
12	Ni	10.4275	3.4758	0.0000	0.7500	0.2500	0.0000
13	Ni	6.9517	6.9517	0.0000	0.5000	0.5000	0.0000
14	Ni	6.9517	10.4275	0.0000	0.5000	0.7500	0.0000
15	Ni	10.4275	6.9517	0.0000	0.7500	0.5000	0.0000
16	Ni	10.4275	10.4275	0.0000	0.7500	0.7500	0.0000
17	Ni	0.0000	1.7379	1.7379	0.0000	0.1250	0.0792
18	Ni	0.0000	5.2138	1.7379	0.0000	0.3750	0.0792
19	Ni	3.4758	1.7379	1.7379	0.2500	0.1250	0.0792
20	Ni	3.4758	5.2138	1.7379	0.2500	0.3750	0.0792
21	Ni	0.0000	8.6896	1.7379	0.0000	0.6250	0.0792
22	Ni	0.0000	12.1654	1.7379	0.0000	0.8750	0.0792
23	Ni	3.4758	8.6896	1.7379	0.2500	0.6250	0.0792
24	Ni	3.4758	12.1654	1.7379	0.2500	0.8750	0.0792
25	Ni	6.9517	1.7379	1.7379	0.5000	0.1250	0.0792
26	Ni	6.9517	5.2138	1.7379	0.5000	0.3750	0.0792
27	Ni	10.4275	1.7379	1.7379	0.7500	0.1250	0.0792
28	Ni	10.4275	5.2138	1.7379	0.7500	0.3750	0.0792
29	Ni	6.9517	8.6896	1.7379	0.5000	0.6250	0.0792
30	Ni	6.9517	12.1654	1.7379	0.5000	0.8750	0.0792
31	Ni	10.4275	8.6896	1.7379	0.7500	0.6250	0.0792
32	Ni	10.4275	12.1654	1.7379	0.7500	0.8750	0.0792
33	Ni	13.8965	13.8978	3.4447	0.9995	0.9996	0.1569
34	Ni	0.0062	3.4566	3.5153	0.0004	0.2486	0.1601
35	Ni	3.4711	13.9026	3.5010	0.2497	0.9999	0.1595
36	Ni	3.4654	3.4273	3.5001	0.2492	0.2465	0.1594
37	Ni	13.9019	6.9595	3.5089	0.9999	0.5006	0.1598
38	Ni	13.8775	10.4475	3.5012	0.9981	0.7514	0.1595
39	Ni	3.4715	6.9687	3.4979	0.2497	0.5012	0.1593

40	Ni	3.5030	10.3918	3.5108	0.2520	0.7474	0.1599
41	Ni	6.9513	0.0174	3.5005	0.5000	0.0013	0.1595
42	Ni	6.9508	3.4965	3.5179	0.4999	0.2515	0.1603
43	Ni	10.4349	0.0176	3.4547	0.7505	0.0013	0.1574
44	Ni	10.4335	3.4691	3.5148	0.7504	0.2495	0.1601
45	Ni	6.9584	6.9623	3.5055	0.5005	0.5008	0.1597
46	Ni	6.9752	10.4369	3.4722	0.5017	0.7507	0.1582
47	Ni	10.4375	6.9407	3.4877	0.7507	0.4992	0.1589
48	Ni	10.4261	10.4461	3.5049	0.7499	0.7513	0.1597
49	Ni	0.0430	1.7387	5.2597	0.0031	0.1251	0.2396
50	Ni	0.0119	5.2159	5.2146	0.0009	0.3752	0.2376
51	Ni	3.4456	1.7591	5.2986	0.2478	0.1265	0.2414
52	Ni	3.4366	5.1833	5.0990	0.2472	0.3728	0.2323
53	Ni	13.8573	8.7188	5.1855	0.9967	0.6271	0.2362
54	Ni	13.8767	12.1320	5.2502	0.9981	0.8726	0.2392
55	Ni	3.4656	8.6997	5.2601	0.2493	0.6257	0.2396
56	Ni	3.4859	12.1133	5.1855	0.2507	0.8713	0.2362
57	Ni	6.9745	1.7589	5.1954	0.5016	0.1265	0.2367
58	Ni	6.9782	5.1924	5.3654	0.5019	0.3735	0.2444
59	Ni	10.4212	1.7748	5.2544	0.7496	0.1277	0.2394
60	Ni	10.4267	5.2097	5.2113	0.7499	0.3747	0.2374
61	Ni	7.0042	8.7080	5.2208	0.5038	0.6263	0.2378
62	Ni	6.9462	12.2210	5.1733	0.4996	0.8790	0.2357
63	Ni	10.4192	8.6887	5.2007	0.7494	0.6249	0.2369
64	Ni	10.4608	12.1843	5.2770	0.7524	0.8764	0.2404
65	Co	1.7379	1.7379	0.0000	0.1250	0.1250	0.0000
66	Co	1.7379	5.2138	0.0000	0.1250	0.3750	0.0000
67	Co	5.2138	1.7379	0.0000	0.3750	0.1250	0.0000
68	Co	5.2138	5.2138	0.0000	0.3750	0.3750	0.0000
69	Co	1.7379	8.6896	0.0000	0.1250	0.6250	0.0000
70	Co	1.7379	12.1654	0.0000	0.1250	0.8750	0.0000
71	Co	5.2138	8.6896	0.0000	0.3750	0.6250	0.0000
72	Co	5.2138	12.1654	0.0000	0.3750	0.8750	0.0000
73	Co	8.6896	1.7379	0.0000	0.6250	0.1250	0.0000
74	Co	8.6896	5.2138	0.0000	0.6250	0.3750	0.0000
75	Co	12.1654	1.7379	0.0000	0.8750	0.1250	0.0000
76	Co	12.1654	5.2138	0.0000	0.8750	0.3750	0.0000
77	Co	8.6896	8.6896	0.0000	0.6250	0.6250	0.0000
78	Co	8.6896	12.1654	0.0000	0.6250	0.8750	0.0000
79	Co	12.1654	8.6896	0.0000	0.8750	0.6250	0.0000
80	Co	12.1654	12.1654	0.0000	0.8750	0.8750	0.0000
81	Co	1.7379	0.0000	1.7379	0.1250	0.0000	0.0792
82	Co	1.7379	3.4758	1.7379	0.1250	0.2500	0.0792
83	Co	5.2138	0.0000	1.7379	0.3750	0.0000	0.0792
84	Co	5.2138	3.4758	1.7379	0.3750	0.2500	0.0792
85	Co	1.7379	6.9517	1.7379	0.1250	0.5000	0.0792
86	Co	1.7379	10.4275	1.7379	0.1250	0.7500	0.0792
87	Co	5.2138	6.9517	1.7379	0.3750	0.5000	0.0792
88	Co	5.2138	10.4275	1.7379	0.3750	0.7500	0.0792

89	Co	8.6896	0.0000	1.7379	0.6250	0.0000	0.0792
90	Co	8.6896	3.4758	1.7379	0.6250	0.2500	0.0792
91	Co	12.1654	0.0000	1.7379	0.8750	0.0000	0.0792
92	Co	12.1654	3.4758	1.7379	0.8750	0.2500	0.0792
93	Co	8.6896	6.9517	1.7379	0.6250	0.5000	0.0792
94	Co	8.6896	10.4275	1.7379	0.6250	0.7500	0.0792
95	Co	12.1654	6.9517	1.7379	0.8750	0.5000	0.0792
96	Co	12.1654	10.4275	1.7379	0.8750	0.7500	0.0792
97	Co	1.7440	1.7203	3.5529	0.1254	0.1237	0.1618
98	Co	1.7091	5.2382	3.5249	0.1229	0.3768	0.1606
99	Co	5.2066	1.7301	3.5679	0.3745	0.1244	0.1625
100	Co	5.2283	5.1933	3.5211	0.3760	0.3735	0.1604
101	Co	1.7277	8.6907	3.5242	0.1243	0.6251	0.1605
102	Co	1.7340	12.1630	3.5298	0.1247	0.8748	0.1608
103	Co	5.2106	8.6813	3.5634	0.3748	0.6244	0.1623
104	Co	5.2208	12.1619	3.5496	0.3755	0.8747	0.1617
105	Co	8.6941	1.7406	3.5397	0.6253	0.1252	0.1612
106	Co	8.6728	5.2080	3.5637	0.6238	0.3746	0.1623
107	Co	12.1781	1.7391	3.5549	0.8759	0.1251	0.1619
108	Co	12.1734	5.2086	3.5596	0.8756	0.3746	0.1622
109	Co	8.6955	8.6905	3.5273	0.6254	0.6251	0.1607
110	Co	8.7095	12.1980	3.4720	0.6264	0.8773	0.1582
111	Co	12.1491	8.6916	3.5403	0.8738	0.6251	0.1613
112	Co	12.1478	12.1833	3.5615	0.8737	0.8763	0.1622
113	Co	1.7548	13.8048	5.3385	0.1262	0.9929	0.2432
114	Co	1.7331	3.5024	5.2147	0.1247	0.2519	0.2376
115	Co	5.2308	0.0098	5.2791	0.3762	0.0007	0.2405
116	Co	5.1963	3.4663	5.1992	0.3737	0.2493	0.2368
117	Co	1.7035	6.9928	5.3127	0.1225	0.5030	0.2420
118	Co	1.7080	10.4300	5.1512	0.1228	0.7502	0.2347
119	Co	5.2367	6.8797	5.1828	0.3767	0.4948	0.2361
120	Co	5.2590	10.4200	5.2270	0.3783	0.7495	0.2381
121	Co	8.6903	0.0013	5.3379	0.6250	0.0001	0.2432
122	Co	8.6930	3.4773	5.2168	0.6252	0.2501	0.2377
123	Co	12.1888	0.0303	5.3701	0.8767	0.0022	0.2446
124	Co	12.1838	3.4864	5.2166	0.8763	0.2508	0.2376
125	Co	8.7277	6.9636	5.1786	0.6277	0.5009	0.2359
126	Co	8.7413	10.4683	5.2027	0.6287	0.7529	0.2370
127	Co	12.1588	6.9507	5.2194	0.8745	0.4999	0.2378
128	Co	12.1456	10.4157	5.2110	0.8736	0.7492	0.2374
129	O	5.8073	5.9776	6.8002	0.4177	0.4299	0.3098
130	O	2.5301	7.9499	6.8019	0.1820	0.5718	0.3099
131	O	4.8123	7.9270	8.7345	0.3461	0.5702	0.3979
132	O	6.2403	9.7369	6.8250	0.4488	0.7003	0.3109
133	O	8.1827	9.3137	8.9634	0.5885	0.6699	0.4083
134	O	5.5007	11.3031	9.0860	0.3956	0.8130	0.4139
135	O	8.4837	12.0220	6.3450	0.6102	0.8647	0.2890
136	Ce	4.0734	10.1459	8.0569	0.2930	0.7297	0.3670
137	Ce	3.6961	6.2851	7.7430	0.2658	0.4521	0.3527

138	Ce	7.0491	7.7431	8.0092	0.5070	0.5569	0.3649
139	Sm	7.4740	11.1747	8.2758	0.5376	0.8037	0.3770
140	C	3.1569	13.2832	6.7334	0.2271	0.9554	0.3067
141	O	3.9681	0.4710	6.8059	0.2854	0.0339	0.3100
142	O	2.9365	12.5515	7.7403	0.2112	0.9028	0.3526
143	O	10.4758	0.1146	6.2210	0.7535	0.0082	0.2834
144	O	0.0355	0.0097	6.1990	0.0026	0.0007	0.2824

/db/jmorales/CoNi-alloy/Profiles/Ni-Ce_3SmO_7/O-coverage/M-CO2-O-coverage/M-CO_2-Ni-Ce_3SmO_7-6O

a =			13.901983737				
b =			13.901983737				
c =			21.950991869				
alpha =			90.0				
beta =			90.0				
gamma =			90.0				
	Atom	X	Y	Z	X	Y	Z
1	Ni	0.0000	0.0000	0.0000	0.0000	0.0000	0.0000
2	Ni	1.7378	1.7378	0.0000	0.1250	0.1250	0.0000
3	Ni	0.0000	3.4755	0.0000	0.0000	0.2500	0.0000
4	Ni	1.7378	5.2132	0.0000	0.1250	0.3750	0.0000
5	Ni	3.4755	0.0000	0.0000	0.2500	0.0000	0.0000
6	Ni	5.2132	1.7378	0.0000	0.3750	0.1250	0.0000
7	Ni	3.4755	3.4755	0.0000	0.2500	0.2500	0.0000
8	Ni	5.2132	5.2132	0.0000	0.3750	0.3750	0.0000
9	Ni	0.0000	6.9510	0.0000	0.0000	0.5000	0.0000
10	Ni	1.7378	8.6887	0.0000	0.1250	0.6250	0.0000
11	Ni	0.0000	10.4265	0.0000	0.0000	0.7500	0.0000
12	Ni	1.7378	12.1642	0.0000	0.1250	0.8750	0.0000
13	Ni	3.4755	6.9510	0.0000	0.2500	0.5000	0.0000
14	Ni	5.2132	8.6887	0.0000	0.3750	0.6250	0.0000
15	Ni	3.4755	10.4265	0.0000	0.2500	0.7500	0.0000
16	Ni	5.2132	12.1642	0.0000	0.3750	0.8750	0.0000
17	Ni	6.9510	0.0000	0.0000	0.5000	0.0000	0.0000
18	Ni	8.6887	1.7378	0.0000	0.6250	0.1250	0.0000
19	Ni	6.9510	3.4755	0.0000	0.5000	0.2500	0.0000
20	Ni	8.6887	5.2132	0.0000	0.6250	0.3750	0.0000
21	Ni	10.4265	0.0000	0.0000	0.7500	0.0000	0.0000
22	Ni	12.1642	1.7378	0.0000	0.8750	0.1250	0.0000
23	Ni	10.4265	3.4755	0.0000	0.7500	0.2500	0.0000
24	Ni	12.1642	5.2132	0.0000	0.8750	0.3750	0.0000
25	Ni	6.9510	6.9510	0.0000	0.5000	0.5000	0.0000
26	Ni	8.6887	8.6887	0.0000	0.6250	0.6250	0.0000
27	Ni	6.9510	10.4265	0.0000	0.5000	0.7500	0.0000
28	Ni	8.6887	12.1642	0.0000	0.6250	0.8750	0.0000
29	Ni	10.4265	6.9510	0.0000	0.7500	0.5000	0.0000
30	Ni	12.1642	8.6887	0.0000	0.8750	0.6250	0.0000
31	Ni	10.4265	10.4265	0.0000	0.7500	0.7500	0.0000
32	Ni	12.1642	12.1642	0.0000	0.8750	0.8750	0.0000
33	Ni	0.0000	1.7378	1.7378	0.0000	0.1250	0.0792
34	Ni	1.7378	0.0000	1.7378	0.1250	0.0000	0.0792
35	Ni	0.0000	5.2132	1.7378	0.0000	0.3750	0.0792
36	Ni	1.7378	3.4755	1.7378	0.1250	0.2500	0.0792
37	Ni	3.4755	1.7378	1.7378	0.2500	0.1250	0.0792
38	Ni	5.2132	0.0000	1.7378	0.3750	0.0000	0.0792
39	Ni	3.4755	5.2132	1.7378	0.2500	0.3750	0.0792

40	Ni	5.2132	3.4755	1.7378	0.3750	0.2500	0.0792
41	Ni	0.0000	8.6887	1.7378	0.0000	0.6250	0.0792
42	Ni	1.7378	6.9510	1.7378	0.1250	0.5000	0.0792
43	Ni	0.0000	12.1642	1.7378	0.0000	0.8750	0.0792
44	Ni	1.7378	10.4265	1.7378	0.1250	0.7500	0.0792
45	Ni	3.4755	8.6887	1.7378	0.2500	0.6250	0.0792
46	Ni	5.2132	6.9510	1.7378	0.3750	0.5000	0.0792
47	Ni	3.4755	12.1642	1.7378	0.2500	0.8750	0.0792
48	Ni	5.2132	10.4265	1.7378	0.3750	0.7500	0.0792
49	Ni	6.9510	1.7378	1.7378	0.5000	0.1250	0.0792
50	Ni	8.6887	0.0000	1.7378	0.6250	0.0000	0.0792
51	Ni	6.9510	5.2132	1.7378	0.5000	0.3750	0.0792
52	Ni	8.6887	3.4755	1.7378	0.6250	0.2500	0.0792
53	Ni	10.4265	1.7378	1.7378	0.7500	0.1250	0.0792
54	Ni	12.1642	0.0000	1.7378	0.8750	0.0000	0.0792
55	Ni	10.4265	5.2132	1.7378	0.7500	0.3750	0.0792
56	Ni	12.1642	3.4755	1.7378	0.8750	0.2500	0.0792
57	Ni	6.9510	8.6887	1.7378	0.5000	0.6250	0.0792
58	Ni	8.6887	6.9510	1.7378	0.6250	0.5000	0.0792
59	Ni	6.9510	12.1642	1.7378	0.5000	0.8750	0.0792
60	Ni	8.6887	10.4265	1.7378	0.6250	0.7500	0.0792
61	Ni	10.4265	8.6887	1.7378	0.7500	0.6250	0.0792
62	Ni	12.1642	6.9510	1.7378	0.8750	0.5000	0.0792
63	Ni	10.4265	12.1642	1.7378	0.7500	0.8750	0.0792
64	Ni	12.1642	10.4265	1.7378	0.8750	0.7500	0.0792
65	Ni	0.0196	0.0239	3.4974	0.0014	0.0017	0.1593
66	Ni	1.7538	1.7857	3.4712	0.1262	0.1284	0.1581
67	Ni	13.8839	3.4864	3.5158	0.9987	0.2508	0.1602
68	Ni	1.6867	5.2077	3.4898	0.1213	0.3746	0.1590
69	Ni	3.4596	0.0056	3.5062	0.2489	0.0004	0.1597
70	Ni	5.2215	1.7485	3.4814	0.3756	0.1258	0.1586
71	Ni	3.5008	3.4542	3.4869	0.2518	0.2485	0.1588
72	Ni	5.2444	5.2025	3.4814	0.3772	0.3742	0.1586
73	Ni	-0.0015	6.9368	3.5119	-0.0001	0.4990	0.1600
74	Ni	1.7572	8.6503	3.4737	0.1264	0.6222	0.1582
75	Ni	0.0015	10.4189	3.5124	0.0001	0.7495	0.1600
76	Ni	1.7279	12.1568	3.5335	0.1243	0.8745	0.1610
77	Ni	3.4805	6.9676	3.4693	0.2504	0.5012	0.1580
78	Ni	5.2244	8.7084	3.5110	0.3758	0.6264	0.1599
79	Ni	3.4632	10.4012	3.5211	0.2491	0.7482	0.1604
80	Ni	5.2045	12.1777	3.5002	0.3744	0.8760	0.1595
81	Ni	6.9432	-0.0018	3.5074	0.4994	-0.0001	0.1598
82	Ni	8.6754	1.7114	3.4377	0.6240	0.1231	0.1566
83	Ni	6.9572	3.4548	3.4957	0.5004	0.2485	0.1592
84	Ni	8.6579	5.2266	3.5084	0.6228	0.3760	0.1598
85	Ni	10.4526	13.8913	3.5087	0.7519	0.9992	0.1598
86	Ni	12.1710	1.7460	3.5424	0.8755	0.1256	0.1614
87	Ni	10.4175	3.4618	3.4817	0.7494	0.2490	0.1586
88	Ni	12.1487	5.2211	3.4890	0.8739	0.3756	0.1589

89	Ni	6.9479	6.9551	3.5038	0.4998	0.5003	0.1596
90	Ni	8.6867	8.7057	3.4607	0.6249	0.6262	0.1577
91	Ni	6.9698	10.4417	3.5052	0.5014	0.7511	0.1597
92	Ni	8.7127	12.1974	3.5076	0.6267	0.8774	0.1598
93	Ni	10.4052	6.9750	3.5038	0.7485	0.5017	0.1596
94	Ni	12.1985	8.6734	3.5245	0.8775	0.6239	0.1606
95	Ni	10.4534	10.4468	3.4355	0.7519	0.7515	0.1565
96	Ni	12.1831	12.1652	3.4373	0.8764	0.8751	0.1566
97	Ni	0.0046	1.8177	5.2758	0.0003	0.1308	0.2403
98	Ni	1.7337	0.0250	5.2772	0.1247	0.0018	0.2404
99	Ni	13.8206	5.1890	5.2986	0.9941	0.3733	0.2414
100	Ni	1.7510	3.5835	5.4268	0.1260	0.2578	0.2472
101	Ni	3.4630	1.7672	5.3683	0.2491	0.1271	0.2446
102	Ni	5.1809	-0.0259	5.2723	0.3727	-0.0019	0.2402
103	Ni	3.5292	5.1892	5.0698	0.2539	0.3733	0.2310
104	Ni	5.2742	3.4698	5.3645	0.3794	0.2496	0.2444
105	Ni	0.0311	8.6516	5.2573	0.0022	0.6223	0.2395
106	Ni	1.7406	6.8580	5.3788	0.1252	0.4933	0.2450
107	Ni	0.0373	12.1665	5.2682	0.0027	0.8752	0.2400
108	Ni	1.7473	10.4137	5.2894	0.1257	0.7491	0.2410
109	Ni	3.4896	8.6995	5.4182	0.2510	0.6258	0.2468
110	Ni	5.2353	6.9653	5.2053	0.3766	0.5010	0.2371
111	Ni	3.4599	12.1522	5.2009	0.2489	0.8741	0.2369
112	Ni	5.2183	10.4568	5.1847	0.3754	0.7522	0.2362
113	Ni	6.9584	1.6599	5.4208	0.5005	0.1194	0.2470
114	Ni	8.7061	13.8821	5.2636	0.6263	0.9986	0.2398
115	Ni	6.9893	5.2224	5.2641	0.5028	0.3757	0.2398
116	Ni	8.6972	3.4149	5.2138	0.6256	0.2456	0.2375
117	Ni	10.4484	1.7022	5.2692	0.7516	0.1224	0.2400
118	Ni	12.2148	0.0310	5.2662	0.8786	0.0022	0.2399
119	Ni	10.3628	5.2198	5.1863	0.7454	0.3755	0.2363
120	Ni	12.0571	3.4993	5.2911	0.8673	0.2517	0.2410
121	Ni	6.9810	8.7706	5.2500	0.5022	0.6309	0.2392
122	Ni	8.6969	6.9931	5.2678	0.6256	0.5030	0.2400
123	Ni	6.9428	12.1659	5.2055	0.4994	0.8751	0.2371
124	Ni	8.7126	10.5038	5.1995	0.6267	0.7556	0.2369
125	Ni	10.4571	8.7161	5.1746	0.7522	0.6270	0.2357
126	Ni	12.1168	6.9880	5.2964	0.8716	0.5027	0.2413
127	Ni	10.4917	12.1887	5.3143	0.7547	0.8768	0.2421
128	Ni	12.2043	10.4337	5.3109	0.8779	0.7505	0.2419
129	O	6.2549	4.4560	6.8248	0.4499	0.3205	0.3109
130	O	4.3424	7.5622	6.7878	0.3124	0.5440	0.3092
131	O	6.1488	6.6414	8.8275	0.4423	0.4777	0.4021
132	O	7.9254	7.9996	6.8161	0.5701	0.5754	0.3105
133	O	10.3032	10.2789	6.2765	0.7411	0.7394	0.2859
134	O	9.5206	7.3095	9.0522	0.6848	0.5258	0.4124
135	O	7.5747	10.0180	8.9131	0.5449	0.7206	0.4060
136	Ce	5.9634	8.8418	8.0789	0.4290	0.6360	0.3680
137	Ce	4.4858	5.4867	7.7842	0.3227	0.3947	0.3546

138	Ce	8.3164	5.8642	8.0916	0.5982	0.4218	0.3686
139	Sm	9.4035	9.2608	8.2054	0.6764	0.6662	0.3738
140	C	11.4846	4.9002	6.7269	0.8261	0.3525	0.3065
141	O	12.5902	5.6952	6.7830	0.9056	0.4097	0.3090
142	O	10.7860	4.6582	7.7424	0.7759	0.3351	0.3527
143	O	2.2862	5.2370	6.4614	0.1644	0.3767	0.2944
144	O	1.6670	1.7269	6.1913	0.1199	0.1242	0.2820
145	O	8.7511	1.7172	6.1631	0.6295	0.1235	0.2808
146	O	12.3029	12.2893	6.1947	0.8850	0.8840	0.2822
147	O	5.1975	1.6163	6.2106	0.3739	0.1163	0.2829
148	O	1.6341	8.7449	6.2204	0.1175	0.6290	0.2834

/db/jmorales/CoNi-alloy/Profiles/Sm(subsurf)$Ce_{26}O_{53}$/surface

a =			11.6082000732				
b =			11.608200073110298				
c =			22.898399353				
alpha =			90.0				
beta =			90.0				
gamma =			120.0				
	Atom	X	Y	Z	X	Y	Z
1	Sm	1.9460	5.5916	3.8366	0.4457	0.5562	0.1675
2	Ce	1.9347	1.1170	0.7898	0.2222	0.1111	0.0345
3	Ce	0.0000	4.4680	0.7898	0.2222	0.4444	0.0345
4	Ce	-1.9347	7.8190	0.7898	0.2222	0.7778	0.0345
5	Ce	5.8041	1.1170	0.7898	0.5556	0.1111	0.0345
6	Ce	3.8694	4.4680	0.7898	0.5556	0.4444	0.0345
7	Ce	1.9347	7.8190	0.7898	0.5556	0.7778	0.0345
8	Ce	9.6735	1.1170	0.7898	0.8889	0.1111	0.0345
9	Ce	7.7388	4.4680	0.7898	0.8889	0.4444	0.0345
10	Ce	5.8041	7.8190	0.7898	0.8889	0.7778	0.0345
11	Ce	0.0051	2.2512	3.9589	0.1124	0.2239	0.1729
12	Ce	-1.9173	5.5808	3.9588	0.1124	0.5551	0.1729
13	Ce	-3.8706	8.9353	3.9466	0.1110	0.8888	0.1724
14	Ce	3.8625	2.2483	3.9451	0.4446	0.2236	0.1723
15	Ce	0.0088	8.9231	3.9452	0.4446	0.8876	0.1723
16	Ce	7.7353	2.2320	3.9227	0.7774	0.2220	0.1713
17	Ce	5.7728	5.5881	3.9744	0.7752	0.5559	0.1736
18	Ce	3.8563	8.9074	3.9743	0.7752	0.8860	0.1736
19	Ce	5.8019	10.0506	7.1032	0.9997	0.9998	0.3102
20	Ce	9.6227	3.3215	7.0855	0.9942	0.3304	0.3094
21	Ce	7.7354	6.7019	7.1031	0.9997	0.6667	0.3102
22	Ce	-1.9430	10.0485	7.0850	0.3324	0.9995	0.3094
23	Ce	1.9646	3.1911	7.1538	0.3280	0.3174	0.3124
24	Ce	-0.1234	6.8075	7.1538	0.3279	0.6772	0.3124
25	Ce	7.7247	0.0168	7.0887	0.6663	0.0017	0.3096
26	Ce	5.8115	3.3308	7.0892	0.6663	0.3313	0.3096
27	Ce	3.9895	6.7715	7.2431	0.6805	0.6736	0.3163
28	O	0.0000	0.0000	0.0000	0.0000	0.0000	0.0000
29	O	-1.9347	3.3510	0.0000	-0.0000	0.3333	0.0000
30	O	-3.8694	6.7020	0.0000	-0.0000	0.6667	0.0000
31	O	3.8694	0.0000	0.0000	0.3333	0.0000	0.0000
32	O	1.9347	3.3510	0.0000	0.3333	0.3333	0.0000
33	O	0.0000	6.7020	0.0000	0.3333	0.6667	0.0000
34	O	7.7388	0.0000	0.0000	0.6667	0.0000	0.0000
35	O	5.8041	3.3510	0.0000	0.6667	0.3333	0.0000
36	O	3.8694	6.7020	0.0000	0.6667	0.6667	0.0000
37	O	-0.0000	2.2340	1.5798	0.1111	0.2222	0.0690
38	O	-1.9347	5.5850	1.5798	0.1111	0.5556	0.0690
39	O	-3.8694	8.9360	1.5798	0.1111	0.8889	0.0690
40	O	3.8694	2.2340	1.5798	0.4444	0.2222	0.0690

41	O	1.9347	5.5850	1.5798	0.4444	0.5556	0.0690
42	O	-0.0000	8.9360	1.5798	0.4444	0.8889	0.0690
43	O	7.7388	2.2340	1.5798	0.7778	0.2222	0.0690
44	O	5.8041	5.5850	1.5798	0.7778	0.5556	0.0690
45	O	3.8694	8.9360	1.5798	0.7778	0.8889	0.0690
46	O	1.9347	1.1170	3.1593	0.2222	0.1111	0.1380
47	O	0.0000	4.4680	3.1593	0.2222	0.4444	0.1380
48	O	-1.9347	7.8190	3.1593	0.2222	0.7778	0.1380
49	O	5.8041	1.1170	3.1593	0.5556	0.1111	0.1380
50	O	3.8694	4.4680	3.1593	0.5556	0.4444	0.1380
51	O	1.9347	7.8190	3.1593	0.5556	0.7778	0.1380
52	O	9.6735	1.1170	3.1593	0.8889	0.1111	0.1380
53	O	7.7388	4.4680	3.1593	0.8889	0.4444	0.1380
54	O	5.8041	7.8190	3.1593	0.8889	0.7778	0.1380
55	O	0.0053	0.0061	4.7571	0.0008	0.0006	0.2077
56	O	-1.9305	3.3536	4.7717	0.0005	0.3336	0.2084
57	O	-3.8607	6.7038	4.7573	0.0008	0.6669	0.2078
58	O	-1.9422	10.0482	4.7272	0.3325	0.9995	0.2064
59	O	1.9388	3.4183	4.8940	0.3370	0.3400	0.2137
60	O	0.0591	6.6726	4.8938	0.3370	0.6637	0.2137
61	O	1.9224	10.0430	4.7654	0.6651	0.9990	0.2081
62	O	5.7893	3.3456	4.7662	0.6651	0.3328	0.2081
63	O	3.8307	6.6791	4.8639	0.6622	0.6644	0.2124
64	O	-0.0285	2.1989	6.3454	0.1069	0.2187	0.2771
65	O	-1.9791	5.5771	6.3462	0.1069	0.5548	0.2771
66	O	-3.8557	8.9443	6.2834	0.1127	0.8897	0.2744
67	O	3.8916	2.1522	6.2970	0.4423	0.2141	0.2750
68	O	-0.0576	8.9975	6.2978	0.4425	0.8950	0.2750
69	O	7.7372	2.2324	6.2715	0.7776	0.2221	0.2739
70	O	5.8980	5.5494	6.3387	0.7841	0.5520	0.2768
71	O	3.8833	9.0356	6.3386	0.7839	0.8988	0.2768
72	O	1.9382	1.0768	7.9506	0.2205	0.1071	0.3472
73	O	0.2992	4.6403	7.6788	0.2566	0.4616	0.3353
74	O	-1.9678	7.8424	7.9498	0.2205	0.7801	0.3472
75	O	5.8332	1.1350	7.9111	0.5590	0.1129	0.3455
76	O	3.7492	4.3994	7.7694	0.5418	0.4376	0.3393
77	O	1.8149	7.7462	7.7701	0.5416	0.7705	0.3393
78	O	9.6212	1.1774	7.9369	0.8874	0.1171	0.3466
79	O	7.7631	4.3920	7.9366	0.8872	0.4369	0.3466
80	O	5.9318	7.8944	7.9703	0.9036	0.7853	0.3481

/db/jmorales/CoNi-alloy/Profiles/Ni-Ce_3SmO_7/O-coverage/M-CO-O-O-coverage/CO-O-Ni-Ce_3SmO_7-O

a = 13.9019837372
b = 13.9019837372
c = 21.9509918687
alpha = 90.0
beta = 90.0
gamma = 90.0

	Atom	X	Y	Z	X	Y	Z
1	Ni	0.0000	0.0000	0.0000	0.0000	0.0000	0.0000
2	Ni	1.7377	1.7377	0.0000	0.1250	0.1250	0.0000
3	Ni	0.0000	3.4755	0.0000	0.0000	0.2500	0.0000
4	Ni	1.7377	5.2132	0.0000	0.1250	0.3750	0.0000
5	Ni	3.4755	0.0000	0.0000	0.2500	0.0000	0.0000
6	Ni	5.2132	1.7377	0.0000	0.3750	0.1250	0.0000
7	Ni	3.4755	3.4755	0.0000	0.2500	0.2500	0.0000
8	Ni	5.2132	5.2132	0.0000	0.3750	0.3750	0.0000
9	Ni	0.0000	6.9510	0.0000	0.0000	0.5000	0.0000
10	Ni	1.7377	8.6887	0.0000	0.1250	0.6250	0.0000
11	Ni	0.0000	10.4265	0.0000	0.0000	0.7500	0.0000
12	Ni	1.7377	12.1642	0.0000	0.1250	0.8750	0.0000
13	Ni	3.4755	6.9510	0.0000	0.2500	0.5000	0.0000
14	Ni	5.2132	8.6887	0.0000	0.3750	0.6250	0.0000
15	Ni	3.4755	10.4265	0.0000	0.2500	0.7500	0.0000
16	Ni	5.2132	12.1642	0.0000	0.3750	0.8750	0.0000
17	Ni	6.9510	0.0000	0.0000	0.5000	0.0000	0.0000
18	Ni	8.6887	1.7377	0.0000	0.6250	0.1250	0.0000
19	Ni	6.9510	3.4755	0.0000	0.5000	0.2500	0.0000
20	Ni	8.6887	5.2132	0.0000	0.6250	0.3750	0.0000
21	Ni	10.4265	0.0000	0.0000	0.7500	0.0000	0.0000
22	Ni	12.1642	1.7377	0.0000	0.8750	0.1250	0.0000
23	Ni	10.4265	3.4755	0.0000	0.7500	0.2500	0.0000
24	Ni	12.1642	5.2132	0.0000	0.8750	0.3750	0.0000
25	Ni	6.9510	6.9510	0.0000	0.5000	0.5000	0.0000
26	Ni	8.6887	8.6887	0.0000	0.6250	0.6250	0.0000
27	Ni	6.9510	10.4265	0.0000	0.5000	0.7500	0.0000
28	Ni	8.6887	12.1642	0.0000	0.6250	0.8750	0.0000
29	Ni	10.4265	6.9510	0.0000	0.7500	0.5000	0.0000
30	Ni	12.1642	8.6887	0.0000	0.8750	0.6250	0.0000
31	Ni	10.4265	10.4265	0.0000	0.7500	0.7500	0.0000
32	Ni	12.1642	12.1642	0.0000	0.8750	0.8750	0.0000
33	Ni	0.0000	1.7377	1.7377	0.0000	0.1250	0.0792
34	Ni	1.7377	0.0000	1.7377	0.1250	0.0000	0.0792
35	Ni	0.0000	5.2132	1.7377	0.0000	0.3750	0.0792
36	Ni	1.7377	3.4755	1.7377	0.1250	0.2500	0.0792
37	Ni	3.4755	1.7377	1.7377	0.2500	0.1250	0.0792
38	Ni	5.2132	0.0000	1.7377	0.3750	0.0000	0.0792
39	Ni	3.4755	5.2132	1.7377	0.2500	0.3750	0.0792

40	Ni	5.2132	3.4755	1.7377	0.3750	0.2500	0.0792
41	Ni	0.0000	8.6887	1.7377	0.0000	0.6250	0.0792
42	Ni	1.7377	6.9510	1.7377	0.1250	0.5000	0.0792
43	Ni	0.0000	12.1642	1.7377	0.0000	0.8750	0.0792
44	Ni	1.7377	10.4265	1.7377	0.1250	0.7500	0.0792
45	Ni	3.4755	8.6887	1.7377	0.2500	0.6250	0.0792
46	Ni	5.2132	6.9510	1.7377	0.3750	0.5000	0.0792
47	Ni	3.4755	12.1642	1.7377	0.2500	0.8750	0.0792
48	Ni	5.2132	10.4265	1.7377	0.3750	0.7500	0.0792
49	Ni	6.9510	1.7377	1.7377	0.5000	0.1250	0.0792
50	Ni	8.6887	0.0000	1.7377	0.6250	0.0000	0.0792
51	Ni	6.9510	5.2132	1.7377	0.5000	0.3750	0.0792
52	Ni	8.6887	3.4755	1.7377	0.6250	0.2500	0.0792
53	Ni	10.4265	1.7377	1.7377	0.7500	0.1250	0.0792
54	Ni	12.1642	0.0000	1.7377	0.8750	0.0000	0.0792
55	Ni	10.4265	5.2132	1.7377	0.7500	0.3750	0.0792
56	Ni	12.1642	3.4755	1.7377	0.8750	0.2500	0.0792
57	Ni	6.9510	8.6887	1.7377	0.5000	0.6250	0.0792
58	Ni	8.6887	6.9510	1.7377	0.6250	0.5000	0.0792
59	Ni	6.9510	12.1642	1.7377	0.5000	0.8750	0.0792
60	Ni	8.6887	10.4265	1.7377	0.6250	0.7500	0.0792
61	Ni	10.4265	8.6887	1.7377	0.7500	0.6250	0.0792
62	Ni	12.1642	6.9510	1.7377	0.8750	0.5000	0.0792
63	Ni	10.4265	12.1642	1.7377	0.7500	0.8750	0.0792
64	Ni	12.1642	10.4265	1.7377	0.8750	0.7500	0.0792
65	Ni	0.0061	0.0055	3.5062	0.0004	0.0004	0.1597
66	Ni	1.7169	1.7642	3.5476	0.1235	0.1269	0.1616
67	Ni	13.8955	3.4735	3.5078	0.9995	0.2499	0.1598
68	Ni	1.6951	5.2045	3.4216	0.1219	0.3744	0.1559
69	Ni	3.4719	0.0087	3.5101	0.2497	0.0006	0.1599
70	Ni	5.2234	1.7488	3.5285	0.3757	0.1258	0.1607
71	Ni	3.4926	3.4352	3.4781	0.2512	0.2471	0.1584
72	Ni	5.2194	5.2032	3.4821	0.3754	0.3743	0.1586
73	Ni	13.8995	6.9468	3.5019	0.9998	0.4997	0.1595
74	Ni	1.7414	8.6665	3.5422	0.1253	0.6234	0.1614
75	Ni	13.8857	10.4211	3.5237	0.9988	0.7496	0.1605
76	Ni	1.7425	12.1607	3.5047	0.1253	0.8747	0.1597
77	Ni	3.4685	6.9806	3.4620	0.2495	0.5021	0.1577
78	Ni	5.2244	8.7085	3.4961	0.3758	0.6264	0.1593
79	Ni	3.4653	10.4034	3.5235	0.2493	0.7483	0.1605
80	Ni	5.2097	12.1693	3.5010	0.3747	0.8754	0.1595
81	Ni	6.9496	-0.0004	3.5037	0.4999	-0.0000	0.1596
82	Ni	8.7167	1.7270	3.5143	0.6270	0.1242	0.1601
83	Ni	6.9555	3.4659	3.5048	0.5003	0.2493	0.1597
84	Ni	8.6709	5.2356	3.5295	0.6237	0.3766	0.1608
85	Ni	10.4355	13.8778	3.5226	0.7506	0.9983	0.1605
86	Ni	12.1583	1.7434	3.4399	0.8746	0.1254	0.1567
87	Ni	10.4249	3.4835	3.5001	0.7499	0.2506	0.1595
88	Ni	12.1699	5.2002	3.4914	0.8754	0.3741	0.1591

89	Ni	6.9330	6.9393	3.5138	0.4987	0.4992	0.1601
90	Ni	8.6888	8.6842	3.4763	0.6250	0.6247	0.1584
91	Ni	6.9744	10.4259	3.5133	0.5017	0.7500	0.1601
92	Ni	8.6916	12.1736	3.4950	0.6252	0.8757	0.1592
93	Ni	10.4079	6.9561	3.5162	0.7487	0.5004	0.1602
94	Ni	12.1779	8.6759	3.4901	0.8760	0.6241	0.1590
95	Ni	10.4432	10.4384	3.4338	0.7512	0.7509	0.1564
96	Ni	12.1723	12.1759	3.5167	0.8756	0.8758	0.1602
97	Ni	13.8877	1.7631	5.2848	0.9990	0.1268	0.2408
98	Ni	1.7348	-0.0007	5.2183	0.1248	-0.0001	0.2377
99	Ni	13.8368	5.2058	5.3138	0.9953	0.3745	0.2421
100	Ni	1.7351	3.5179	5.2962	0.1248	0.2530	0.2413
101	Ni	3.4683	1.7343	5.2212	0.2495	0.1248	0.2379
102	Ni	5.2111	0.0017	5.2229	0.3748	0.0001	0.2379
103	Ni	3.4702	5.1842	5.0424	0.2496	0.3729	0.2297
104	Ni	5.2105	3.4288	5.3652	0.3748	0.2466	0.2444
105	Ni	0.0067	8.6697	5.2158	0.0005	0.6236	0.2376
106	Ni	1.7037	6.8799	5.2647	0.1226	0.4949	0.2398
107	Ni	0.0044	12.1676	5.2132	0.0003	0.8752	0.2375
108	Ni	1.7220	10.4371	5.2222	0.1239	0.7508	0.2379
109	Ni	3.4428	8.7427	5.3554	0.2476	0.6289	0.2440
110	Ni	5.1783	6.9690	5.2037	0.3725	0.5013	0.2371
111	Ni	3.4773	12.1575	5.2221	0.2501	0.8745	0.2379
112	Ni	5.2279	10.4470	5.1915	0.3761	0.7515	0.2365
113	Ni	6.9873	1.7104	5.2065	0.5026	0.1230	0.2372
114	Ni	8.6936	13.8902	5.2157	0.6253	0.9992	0.2376
115	Ni	6.9315	5.1840	5.2713	0.4986	0.3729	0.2401
116	Ni	8.7150	3.4760	5.1950	0.6269	0.2500	0.2367
117	Ni	10.4351	1.7371	5.2847	0.7506	0.1250	0.2407
118	Ni	12.1740	13.8992	5.2805	0.8757	0.9998	0.2406
119	Ni	10.4395	5.2202	5.2744	0.7509	0.3755	0.2403
120	Ni	12.1407	3.5001	5.3570	0.8733	0.2518	0.2440
121	Ni	6.9651	8.7363	5.2398	0.5010	0.6284	0.2387
122	Ni	8.7005	6.9419	5.2874	0.6258	0.4993	0.2409
123	Ni	6.9476	12.1624	5.2191	0.4998	0.8749	0.2378
124	Ni	8.7167	10.4571	5.2149	0.6270	0.7522	0.2376
125	Ni	10.4566	8.6922	5.1802	0.7522	0.6252	0.2360
126	Ni	12.1513	6.9339	5.2565	0.8741	0.4988	0.2395
127	Ni	10.4463	12.1588	5.2763	0.7514	0.8746	0.2404
128	Ni	12.1810	10.4189	5.2812	0.8762	0.7495	0.2406
129	O	6.0031	4.4406	6.8150	0.4318	0.3194	0.3105
130	O	4.1527	7.6306	6.7390	0.2987	0.5489	0.3070
131	O	5.8761	6.6405	8.7985	0.4227	0.4777	0.4008
132	O	7.7643	7.8411	6.8033	0.5585	0.5640	0.3099
133	O	10.4105	10.3518	6.2707	0.7489	0.7446	0.2857
134	O	9.4020	7.0222	8.9440	0.6763	0.5051	0.4075
135	O	7.6205	9.8187	8.8654	0.5482	0.7063	0.4039
136	Ce	5.8816	8.8681	8.0220	0.4231	0.6379	0.3655
137	Ce	4.1949	5.5408	7.7009	0.3017	0.3986	0.3508

138	Ce	7.9320	5.7551	8.0878	0.5706	0.4140	0.3685
139	Sm	9.3794	8.9746	8.0464	0.6747	0.6456	0.3666
140	C	12.1218	5.2638	6.3822	0.8719	0.3786	0.2907
141	O	12.1431	5.2521	7.6010	0.8735	0.3778	0.3463
142	O	12.1661	1.7132	6.1560	0.8751	0.1232	0.2804
143	O	1.9925	5.2135	6.3081	0.1433	0.3750	0.2874

/db/jmorales/CoNi-alloy/Profiles/CoNi(111)/CO_2

a =			9.831199646				
b =			9.831199645902503				
c =			21.020299912				
alpha =			90.0				
beta =			90.0				
gamma =			120.0				
	Atom	X	Y	Z	X	Y	Z
1	Ni	0.0000	0.0000	0.0000	0.0000	0.0000	0.0000
2	Ni	-1.2289	2.1285	0.0000	0.0000	0.2500	0.0000
3	Ni	-2.4578	4.2570	0.0000	-0.0000	0.5000	0.0000
4	Ni	-3.6867	6.3856	0.0000	-0.0000	0.7500	0.0000
5	Ni	4.9156	0.0000	0.0000	0.5000	0.0000	0.0000
6	Ni	3.6867	2.1285	0.0000	0.5000	0.2500	0.0000
7	Ni	2.4578	4.2570	0.0000	0.5000	0.5000	0.0000
8	Ni	1.2289	6.3856	0.0000	0.5000	0.7500	0.0000
9	Ni	2.4578	1.4190	2.0068	0.3333	0.1667	0.0955
10	Ni	1.2289	3.5475	2.0068	0.3333	0.4167	0.0955
11	Ni	0.0000	5.6760	2.0068	0.3333	0.6667	0.0955
12	Ni	-1.2289	7.8046	2.0068	0.3333	0.9167	0.0955
13	Ni	7.3734	1.4190	2.0068	0.8333	0.1667	0.0955
14	Ni	6.1445	3.5475	2.0068	0.8333	0.4167	0.0955
15	Ni	4.9156	5.6760	2.0068	0.8333	0.6667	0.0955
16	Ni	3.6867	7.8046	2.0068	0.8333	0.9167	0.0955
17	Ni	0.0452	2.8520	4.0233	0.1721	0.3350	0.1914
18	Ni	1.2638	0.7408	4.0224	0.1721	0.0870	0.1914
19	Ni	-2.4272	7.1188	4.0093	0.1712	0.8361	0.1907
20	Ni	-1.1909	4.9798	4.0153	0.1713	0.5849	0.1910
21	Ni	4.9465	2.8598	4.0084	0.6711	0.3359	0.1907
22	Ni	6.1779	0.7290	4.0093	0.6712	0.0856	0.1907
23	Ni	2.4910	7.1124	4.0113	0.6711	0.8354	0.1908
24	Ni	3.7155	4.9822	4.0128	0.6705	0.5852	0.1909
25	Ni	0.0448	0.0245	6.0154	0.0060	0.0029	0.2862
26	Ni	-1.1896	2.1548	6.0144	0.0055	0.2531	0.2861
27	Ni	-2.4062	4.2907	6.0213	0.0072	0.5040	0.2865
28	Ni	-3.6296	6.4182	6.0219	0.0077	0.7538	0.2865
29	Ni	4.9747	0.0284	6.0224	0.5077	0.0033	0.2865
30	Ni	3.7765	2.1517	6.0097	0.5105	0.2527	0.2859
31	Ni	2.5363	4.2974	6.1049	0.5104	0.5047	0.2904
32	Ni	1.2854	6.4291	6.0204	0.5083	0.7551	0.2864
33	Co	1.2289	2.1285	0.0000	0.2500	0.2500	0.0000
34	Co	2.4578	0.0000	0.0000	0.2500	0.0000	0.0000
35	Co	-1.2289	6.3856	0.0000	0.2500	0.7500	0.0000
36	Co	0.0000	4.2570	0.0000	0.2500	0.5000	0.0000
37	Co	6.1445	2.1285	0.0000	0.7500	0.2500	0.0000
38	Co	7.3734	0.0000	0.0000	0.7500	0.0000	0.0000
39	Co	3.6867	6.3856	0.0000	0.7500	0.7500	0.0000
40	Co	4.9156	4.2570	0.0000	0.7500	0.5000	0.0000

41	Co	-1.2289	3.5475	2.0068	0.0833	0.4167	0.0955
42	Co	0.0000	1.4190	2.0068	0.0833	0.1667	0.0955
43	Co	-3.6867	7.8046	2.0068	0.0833	0.9167	0.0955
44	Co	-2.4578	5.6760	2.0068	0.0833	0.6667	0.0955
45	Co	3.6867	3.5475	2.0068	0.5833	0.4167	0.0955
46	Co	4.9156	1.4190	2.0068	0.5833	0.1667	0.0955
47	Co	1.2289	7.8046	2.0068	0.5833	0.9167	0.0955
48	Co	2.4578	5.6760	2.0068	0.5833	0.6667	0.0955
49	Co	3.7149	0.7298	4.0491	0.4207	0.0857	0.1926
50	Co	2.4787	2.8566	4.0796	0.4199	0.3355	0.1941
51	Co	1.2650	4.9794	4.0550	0.4211	0.5848	0.1929
52	Co	0.0302	7.1112	4.0537	0.4207	0.8352	0.1928
53	Co	8.6360	0.7267	4.0496	0.9211	0.0854	0.1927
54	Co	7.4062	2.8558	4.0503	0.9211	0.3354	0.1927
55	Co	6.1733	4.9873	4.0509	0.9208	0.5858	0.1927
56	Co	4.9459	7.1080	4.0534	0.9205	0.8349	0.1928
57	Co	1.2959	2.1381	6.1176	0.2574	0.2511	0.2910
58	Co	2.5369	0.0367	6.0098	0.2602	0.0043	0.2859
59	Co	-1.1561	6.4334	6.0122	0.2602	0.7556	0.2860
60	Co	0.0515	4.3281	5.9985	0.2594	0.5083	0.2854
61	Co	6.2254	2.1690	6.0171	0.7606	0.2548	0.2862
62	Co	7.4437	0.0445	6.0142	0.7598	0.0052	0.2861
63	Co	3.7640	6.4315	6.0099	0.7606	0.7554	0.2859
64	Co	5.0018	4.3016	6.0068	0.7614	0.5052	0.2858
65	C	2.1246	3.7757	7.9644	0.4378	0.4435	0.3789
66	O	2.6329	4.5174	8.7807	0.5331	0.5306	0.4177
67	O	1.3947	2.7250	8.0173	0.3019	0.3201	0.3814

/db/jmorales/CoNi-alloy/Profiles/CoNi-Ce_3SmO_7/O-coverage/TS-O-coverage/TS-CoNi-Ce_3SmO_7-4O

a =			13.9033384264				
b =			13.9033384264				
c =			21.9516692133				
alpha =			90.0				
beta =			90.0				
gamma =			90.0				
	Atom	X	Y	Z	X	Y	Z
1	Ni	0.0000	0.0000	0.0000	0.0000	0.0000	0.0000
2	Ni	0.0000	3.4758	0.0000	0.0000	0.2500	0.0000
3	Ni	3.4758	0.0000	0.0000	0.2500	0.0000	0.0000
4	Ni	3.4758	3.4758	0.0000	0.2500	0.2500	0.0000
5	Ni	0.0000	6.9517	0.0000	0.0000	0.5000	0.0000
6	Ni	0.0000	10.4275	0.0000	0.0000	0.7500	0.0000
7	Ni	3.4758	6.9517	0.0000	0.2500	0.5000	0.0000
8	Ni	3.4758	10.4275	0.0000	0.2500	0.7500	0.0000
9	Ni	6.9517	0.0000	0.0000	0.5000	0.0000	0.0000
10	Ni	6.9517	3.4758	0.0000	0.5000	0.2500	0.0000
11	Ni	10.4275	0.0000	0.0000	0.7500	0.0000	0.0000
12	Ni	10.4275	3.4758	0.0000	0.7500	0.2500	0.0000
13	Ni	6.9517	6.9517	0.0000	0.5000	0.5000	0.0000
14	Ni	6.9517	10.4275	0.0000	0.5000	0.7500	0.0000
15	Ni	10.4275	6.9517	0.0000	0.7500	0.5000	0.0000
16	Ni	10.4275	10.4275	0.0000	0.7500	0.7500	0.0000
17	Ni	0.0000	1.7379	1.7379	0.0000	0.1250	0.0792
18	Ni	0.0000	5.2138	1.7379	0.0000	0.3750	0.0792
19	Ni	3.4758	1.7379	1.7379	0.2500	0.1250	0.0792
20	Ni	3.4758	5.2138	1.7379	0.2500	0.3750	0.0792
21	Ni	0.0000	8.6896	1.7379	0.0000	0.6250	0.0792
22	Ni	0.0000	12.1654	1.7379	0.0000	0.8750	0.0792
23	Ni	3.4758	8.6896	1.7379	0.2500	0.6250	0.0792
24	Ni	3.4758	12.1654	1.7379	0.2500	0.8750	0.0792
25	Ni	6.9517	1.7379	1.7379	0.5000	0.1250	0.0792
26	Ni	6.9517	5.2138	1.7379	0.5000	0.3750	0.0792
27	Ni	10.4275	1.7379	1.7379	0.7500	0.1250	0.0792
28	Ni	10.4275	5.2138	1.7379	0.7500	0.3750	0.0792
29	Ni	6.9517	8.6896	1.7379	0.5000	0.6250	0.0792
30	Ni	6.9517	12.1654	1.7379	0.5000	0.8750	0.0792
31	Ni	10.4275	8.6896	1.7379	0.7500	0.6250	0.0792
32	Ni	10.4275	12.1654	1.7379	0.7500	0.8750	0.0792
33	Ni	13.9013	0.0038	3.4464	0.9999	0.0003	0.1570
34	Ni	0.0163	3.4603	3.5244	0.0012	0.2489	0.1606
35	Ni	3.4638	0.0172	3.5154	0.2491	0.0012	0.1601
36	Ni	3.4852	3.4473	3.4317	0.2507	0.2480	0.1563
37	Ni	0.0008	6.9528	3.5077	0.0001	0.5001	0.1598
38	Ni	13.8828	10.4542	3.5046	0.9985	0.7519	0.1597
39	Ni	3.4660	6.9549	3.4878	0.2493	0.5002	0.1589

40	Ni	3.4778	10.3934	3.5078	0.2501	0.7475	0.1598
41	Ni	6.9526	0.0235	3.5067	0.5001	0.0017	0.1597
42	Ni	6.9447	3.4717	3.4623	0.4995	0.2497	0.1577
43	Ni	10.4449	0.0082	3.4540	0.7513	0.0006	0.1573
44	Ni	10.4161	3.4741	3.5152	0.7492	0.2499	0.1601
45	Ni	6.9633	6.9664	3.5041	0.5008	0.5011	0.1596
46	Ni	6.9653	10.4444	3.4822	0.5010	0.7512	0.1586
47	Ni	10.4406	6.9414	3.4855	0.7509	0.4993	0.1588
48	Ni	10.4315	10.4503	3.5045	0.7503	0.7516	0.1596
49	Ni	0.0214	1.7642	5.2649	0.0015	0.1269	0.2398
50	Ni	0.0052	5.2144	5.2125	0.0004	0.3750	0.2375
51	Ni	3.4785	1.7351	5.2345	0.2502	0.1248	0.2385
52	Ni	3.4736	5.2025	5.0646	0.2498	0.3742	0.2307
53	Ni	13.8713	8.7186	5.1887	0.9977	0.6271	0.2364
54	Ni	0.0097	12.1483	5.2594	0.0007	0.8738	0.2396
55	Ni	3.4242	8.6676	5.2404	0.2463	0.6234	0.2387
56	Ni	3.4331	12.1188	5.1876	0.2469	0.8716	0.2363
57	Ni	6.9579	1.7305	5.3510	0.5004	0.1245	0.2438
58	Ni	6.9905	5.2800	5.3626	0.5028	0.3798	0.2443
59	Ni	10.4524	1.7859	5.2512	0.7518	0.1284	0.2392
60	Ni	10.4178	5.2170	5.2068	0.7493	0.3752	0.2372
61	Ni	6.9880	8.7254	5.2274	0.5026	0.6276	0.2381
62	Ni	7.0032	12.1532	5.2063	0.5037	0.8741	0.2372
63	Ni	10.4555	8.6828	5.1843	0.7520	0.6245	0.2362
64	Ni	10.3874	12.1766	5.3449	0.7471	0.8758	0.2435
65	Co	1.7379	1.7379	0.0000	0.1250	0.1250	0.0000
66	Co	1.7379	5.2138	0.0000	0.1250	0.3750	0.0000
67	Co	5.2138	1.7379	0.0000	0.3750	0.1250	0.0000
68	Co	5.2138	5.2138	0.0000	0.3750	0.3750	0.0000
69	Co	1.7379	8.6896	0.0000	0.1250	0.6250	0.0000
70	Co	1.7379	12.1654	0.0000	0.1250	0.8750	0.0000
71	Co	5.2138	8.6896	0.0000	0.3750	0.6250	0.0000
72	Co	5.2138	12.1654	0.0000	0.3750	0.8750	0.0000
73	Co	8.6896	1.7379	0.0000	0.6250	0.1250	0.0000
74	Co	8.6896	5.2138	0.0000	0.6250	0.3750	0.0000
75	Co	12.1654	1.7379	0.0000	0.8750	0.1250	0.0000
76	Co	12.1654	5.2138	0.0000	0.8750	0.3750	0.0000
77	Co	8.6896	8.6896	0.0000	0.6250	0.6250	0.0000
78	Co	8.6896	12.1654	0.0000	0.6250	0.8750	0.0000
79	Co	12.1654	8.6896	0.0000	0.8750	0.6250	0.0000
80	Co	12.1654	12.1654	0.0000	0.8750	0.8750	0.0000
81	Co	1.7379	0.0000	1.7379	0.1250	0.0000	0.0792
82	Co	1.7379	3.4758	1.7379	0.1250	0.2500	0.0792
83	Co	5.2138	0.0000	1.7379	0.3750	0.0000	0.0792
84	Co	5.2138	3.4758	1.7379	0.3750	0.2500	0.0792
85	Co	1.7379	6.9517	1.7379	0.1250	0.5000	0.0792
86	Co	1.7379	10.4275	1.7379	0.1250	0.7500	0.0792
87	Co	5.2138	6.9517	1.7379	0.3750	0.5000	0.0792
88	Co	5.2138	10.4275	1.7379	0.3750	0.7500	0.0792

89	Co	8.6896	0.0000	1.7379	0.6250	0.0000	0.0792
90	Co	8.6896	3.4758	1.7379	0.6250	0.2500	0.0792
91	Co	12.1654	0.0000	1.7379	0.8750	0.0000	0.0792
92	Co	12.1654	3.4758	1.7379	0.8750	0.2500	0.0792
93	Co	8.6896	6.9517	1.7379	0.6250	0.5000	0.0792
94	Co	8.6896	10.4275	1.7379	0.6250	0.7500	0.0792
95	Co	12.1654	6.9517	1.7379	0.8750	0.5000	0.0792
96	Co	12.1654	10.4275	1.7379	0.8750	0.7500	0.0792
97	Co	1.7410	1.7507	3.5603	0.1252	0.1259	0.1622
98	Co	1.7064	5.2269	3.5198	0.1227	0.3759	0.1603
99	Co	5.2173	1.7363	3.5659	0.3753	0.1249	0.1624
100	Co	5.2481	5.1938	3.4969	0.3775	0.3736	0.1593
101	Co	1.7166	8.6839	3.5208	0.1235	0.6246	0.1604
102	Co	1.7339	12.1726	3.5223	0.1247	0.8755	0.1605
103	Co	5.1999	8.6896	3.5494	0.3740	0.6250	0.1617
104	Co	5.2085	12.1450	3.5511	0.3746	0.8735	0.1618
105	Co	8.6891	1.7445	3.5540	0.6250	0.1255	0.1619
106	Co	8.6756	5.2087	3.5583	0.6240	0.3746	0.1621
107	Co	12.1772	1.7435	3.5517	0.8758	0.1254	0.1618
108	Co	12.1689	5.2070	3.5542	0.8753	0.3745	0.1619
109	Co	8.6942	8.6797	3.5152	0.6253	0.6243	0.1601
110	Co	8.7076	12.1968	3.4848	0.6263	0.8773	0.1587
111	Co	12.1635	8.6954	3.5332	0.8749	0.6254	0.1610
112	Co	12.1463	12.1867	3.5820	0.8736	0.8765	0.1632
113	Co	1.7596	13.8935	5.2709	0.1266	0.9993	0.2401
114	Co	1.7860	3.5057	5.2816	0.1285	0.2521	0.2406
115	Co	5.1409	13.8596	5.2468	0.3698	0.9969	0.2390
116	Co	5.2021	3.4694	5.3147	0.3742	0.2495	0.2421
117	Co	1.6994	6.9622	5.3159	0.1222	0.5008	0.2422
118	Co	1.7215	10.4443	5.1752	0.1238	0.7512	0.2358
119	Co	5.2363	6.9258	5.1509	0.3766	0.4981	0.2346
120	Co	5.1888	10.3965	5.2238	0.3732	0.7478	0.2380
121	Co	8.7054	0.0720	5.3382	0.6261	0.0052	0.2432
122	Co	8.6601	3.5055	5.2657	0.6229	0.2521	0.2399
123	Co	12.1922	0.0005	5.3707	0.8769	0.0000	0.2447
124	Co	12.1774	3.5014	5.2049	0.8759	0.2518	0.2371
125	Co	8.7395	6.9944	5.1865	0.6286	0.5031	0.2363
126	Co	8.7549	10.3735	5.2007	0.6297	0.7461	0.2369
127	Co	12.1714	6.9433	5.2161	0.8754	0.4994	0.2376
128	Co	12.1636	10.4289	5.2128	0.8749	0.7501	0.2375
129	O	5.8914	6.2020	6.7814	0.4237	0.4461	0.3089
130	O	2.6368	7.7349	6.8185	0.1897	0.5563	0.3106
131	O	4.8114	7.7780	8.8281	0.3461	0.5594	0.4022
132	O	6.0359	9.7354	6.8231	0.4341	0.7002	0.3108
133	O	7.8447	9.6884	9.0418	0.5642	0.6968	0.4119
134	O	4.9722	11.2789	8.9907	0.3576	0.8112	0.4096
135	O	8.6286	11.7481	6.4049	0.6206	0.8450	0.2918
136	Ce	3.8503	9.7526	8.0691	0.2769	0.7015	0.3676
137	Ce	3.8291	6.0122	7.7332	0.2754	0.4324	0.3523

138 Ce	7.0684	7.9393	8.1348	0.5084	0.5710	0.3706
139 Sm	6.9853	11.5067	8.2322	0.5024	0.8276	0.3750
140 C	7.4686	0.5865	6.8294	0.5372	0.0422	0.3111
141 O	6.1662	13.2184	6.6268	0.4435	0.9507	0.3019
142 O	7.7306	0.5947	8.0136	0.5560	0.0428	0.3651
143 O	10.4983	0.1112	6.2128	0.7551	0.0080	0.2830
144 O	0.0712	0.0057	6.1935	0.0051	0.0004	0.2821
145 O	3.4598	3.7497	6.3690	0.2488	0.2697	0.2901
146 O	6.9614	3.4892	6.2342	0.5007	0.2510	0.2840

/db/jmorales/CoNi-alloy/Profiles/Ni-Ce_3SmO_7/O-coverage/CO_2-Ni-Ce_3SmO_7-2O

a =			13.9019837372				
b =			13.9019837372				
c =			21.9509918687				
alpha =			90.0				
beta =			90.0				
gamma =			90.0				
	Atom	X	Y	Z	X	Y	Z
1	Ni	0.0000	0.0000	0.0000	0.0000	0.0000	0.0000
2	Ni	1.7377	1.7377	0.0000	0.1250	0.1250	0.0000
3	Ni	0.0000	3.4755	0.0000	0.0000	0.2500	0.0000
4	Ni	1.7377	5.2132	0.0000	0.1250	0.3750	0.0000
5	Ni	3.4755	0.0000	0.0000	0.2500	0.0000	0.0000
6	Ni	5.2132	1.7377	0.0000	0.3750	0.1250	0.0000
7	Ni	3.4755	3.4755	0.0000	0.2500	0.2500	0.0000
8	Ni	5.2132	5.2132	0.0000	0.3750	0.3750	0.0000
9	Ni	0.0000	6.9510	0.0000	0.0000	0.5000	0.0000
10	Ni	1.7377	8.6887	0.0000	0.1250	0.6250	0.0000
11	Ni	0.0000	10.4265	0.0000	0.0000	0.7500	0.0000
12	Ni	1.7377	12.1642	0.0000	0.1250	0.8750	0.0000
13	Ni	3.4755	6.9510	0.0000	0.2500	0.5000	0.0000
14	Ni	5.2132	8.6887	0.0000	0.3750	0.6250	0.0000
15	Ni	3.4755	10.4265	0.0000	0.2500	0.7500	0.0000
16	Ni	5.2132	12.1642	0.0000	0.3750	0.8750	0.0000
17	Ni	6.9510	0.0000	0.0000	0.5000	0.0000	0.0000
18	Ni	8.6887	1.7377	0.0000	0.6250	0.1250	0.0000
19	Ni	6.9510	3.4755	0.0000	0.5000	0.2500	0.0000
20	Ni	8.6887	5.2132	0.0000	0.6250	0.3750	0.0000
21	Ni	10.4265	0.0000	0.0000	0.7500	0.0000	0.0000
22	Ni	12.1642	1.7377	0.0000	0.8750	0.1250	0.0000
23	Ni	10.4265	3.4755	0.0000	0.7500	0.2500	0.0000
24	Ni	12.1642	5.2132	0.0000	0.8750	0.3750	0.0000
25	Ni	6.9510	6.9510	0.0000	0.5000	0.5000	0.0000
26	Ni	8.6887	8.6887	0.0000	0.6250	0.6250	0.0000
27	Ni	6.9510	10.4265	0.0000	0.5000	0.7500	0.0000
28	Ni	8.6887	12.1642	0.0000	0.6250	0.8750	0.0000
29	Ni	10.4265	6.9510	0.0000	0.7500	0.5000	0.0000
30	Ni	12.1642	8.6887	0.0000	0.8750	0.6250	0.0000
31	Ni	10.4265	10.4265	0.0000	0.7500	0.7500	0.0000
32	Ni	12.1642	12.1642	0.0000	0.8750	0.8750	0.0000
33	Ni	0.0000	1.7377	1.7377	0.0000	0.1250	0.0792
34	Ni	1.7377	0.0000	1.7377	0.1250	0.0000	0.0792
35	Ni	0.0000	5.2132	1.7377	0.0000	0.3750	0.0792
36	Ni	1.7377	3.4755	1.7377	0.1250	0.2500	0.0792
37	Ni	3.4755	1.7377	1.7377	0.2500	0.1250	0.0792
38	Ni	5.2132	0.0000	1.7377	0.3750	0.0000	0.0792
39	Ni	3.4755	5.2132	1.7377	0.2500	0.3750	0.0792
40	Ni	5.2132	3.4755	1.7377	0.3750	0.2500	0.0792

41	Ni	0.0000	8.6887	1.7377	0.0000	0.6250	0.0792
42	Ni	1.7377	6.9510	1.7377	0.1250	0.5000	0.0792
43	Ni	0.0000	12.1642	1.7377	0.0000	0.8750	0.0792
44	Ni	1.7377	10.4265	1.7377	0.1250	0.7500	0.0792
45	Ni	3.4755	8.6887	1.7377	0.2500	0.6250	0.0792
46	Ni	5.2132	6.9510	1.7377	0.3750	0.5000	0.0792
47	Ni	3.4755	12.1642	1.7377	0.2500	0.8750	0.0792
48	Ni	5.2132	10.4265	1.7377	0.3750	0.7500	0.0792
49	Ni	6.9510	1.7377	1.7377	0.5000	0.1250	0.0792
50	Ni	8.6887	0.0000	1.7377	0.6250	0.0000	0.0792
51	Ni	6.9510	5.2132	1.7377	0.5000	0.3750	0.0792
52	Ni	8.6887	3.4755	1.7377	0.6250	0.2500	0.0792
53	Ni	10.4265	1.7377	1.7377	0.7500	0.1250	0.0792
54	Ni	12.1642	0.0000	1.7377	0.8750	0.0000	0.0792
55	Ni	10.4265	5.2132	1.7377	0.7500	0.3750	0.0792
56	Ni	12.1642	3.4755	1.7377	0.8750	0.2500	0.0792
57	Ni	6.9510	8.6887	1.7377	0.5000	0.6250	0.0792
58	Ni	8.6887	6.9510	1.7377	0.6250	0.5000	0.0792
59	Ni	6.9510	12.1642	1.7377	0.5000	0.8750	0.0792
60	Ni	8.6887	10.4265	1.7377	0.6250	0.7500	0.0792
61	Ni	10.4265	8.6887	1.7377	0.7500	0.6250	0.0792
62	Ni	12.1642	6.9510	1.7377	0.8750	0.5000	0.0792
63	Ni	10.4265	12.1642	1.7377	0.7500	0.8750	0.0792
64	Ni	12.1642	10.4265	1.7377	0.8750	0.7500	0.0792
65	Ni	0.0103	13.9006	3.5032	0.0007	0.9999	0.1596
66	Ni	1.7393	1.7489	3.4518	0.1251	0.1258	0.1573
67	Ni	0.0008	3.4738	3.5077	0.0001	0.2499	0.1598
68	Ni	1.6981	5.2022	3.4277	0.1221	0.3742	0.1562
69	Ni	3.4747	13.8944	3.5084	0.2499	0.9995	0.1598
70	Ni	5.1976	1.7447	3.5439	0.3739	0.1255	0.1614
71	Ni	3.4911	3.4386	3.4830	0.2511	0.2473	0.1587
72	Ni	5.1941	5.2120	3.4826	0.3736	0.3749	0.1587
73	Ni	13.8737	6.9492	3.5335	0.9980	0.4999	0.1610
74	Ni	1.7427	8.6777	3.5308	0.1254	0.6242	0.1609
75	Ni	13.8861	10.4290	3.5160	0.9989	0.7502	0.1602
76	Ni	1.7435	12.1707	3.5127	0.1254	0.8755	0.1600
77	Ni	3.4674	6.9905	3.4658	0.2494	0.5028	0.1579
78	Ni	5.2332	8.7085	3.5043	0.3764	0.6264	0.1596
79	Ni	3.4738	10.4040	3.5222	0.2499	0.7484	0.1605
80	Ni	5.2160	12.1633	3.5002	0.3752	0.8749	0.1595
81	Ni	6.9403	-0.0000	3.5025	0.4992	-0.0000	0.1596
82	Ni	8.6963	1.7209	3.4887	0.6255	0.1238	0.1589
83	Ni	6.9312	3.4610	3.4927	0.4986	0.2490	0.1591
84	Ni	8.6840	5.2225	3.5256	0.6247	0.3757	0.1606
85	Ni	10.4351	13.8864	3.5220	0.7506	0.9989	0.1605
86	Ni	12.1792	1.7309	3.5136	0.8761	0.1245	0.1601
87	Ni	10.4385	3.4911	3.5084	0.7509	0.2511	0.1598
88	Ni	12.1708	5.2286	3.5723	0.8755	0.3761	0.1627
89	Ni	6.9269	6.9362	3.5053	0.4983	0.4989	0.1597

90	Ni	8.6888	8.6798	3.4597	0.6250	0.6244	0.1576
91	Ni	6.9840	10.4244	3.5168	0.5024	0.7498	0.1602
92	Ni	8.6911	12.1881	3.4925	0.6252	0.8767	0.1591
93	Ni	10.4342	6.9282	3.5450	0.7506	0.4984	0.1615
94	Ni	12.1790	8.6716	3.4951	0.8761	0.6238	0.1592
95	Ni	10.4358	10.4527	3.4243	0.7507	0.7519	0.1560
96	Ni	12.1678	12.1724	3.5021	0.8753	0.8756	0.1595
97	Ni	0.0353	1.7316	5.2830	0.0025	0.1246	0.2407
98	Ni	1.7419	13.8564	5.2974	0.1253	0.9967	0.2413
99	Ni	0.0305	5.1955	5.2814	0.0022	0.3737	0.2406
100	Ni	1.7597	3.4783	5.3388	0.1266	0.2502	0.2432
101	Ni	3.4553	1.7125	5.2883	0.2485	0.1232	0.2409
102	Ni	5.2114	13.8929	5.2230	0.3749	0.9993	0.2379
103	Ni	3.4368	5.1968	5.0533	0.2472	0.3738	0.2302
104	Ni	5.1873	3.4270	5.3598	0.3731	0.2465	0.2442
105	Ni	0.0140	8.7091	5.2111	0.0010	0.6265	0.2374
106	Ni	1.7154	6.9493	5.2626	0.1234	0.4999	0.2397
107	Ni	13.8954	12.1645	5.2117	0.9995	0.8750	0.2374
108	Ni	1.7216	10.4506	5.2177	0.1238	0.7517	0.2377
109	Ni	3.4506	8.7639	5.3621	0.2482	0.6304	0.2443
110	Ni	5.1816	6.9822	5.2211	0.3727	0.5022	0.2379
111	Ni	3.4943	12.1381	5.2220	0.2514	0.8731	0.2379
112	Ni	5.2535	10.4418	5.2018	0.3779	0.7511	0.2370
113	Ni	6.9621	1.7082	5.2052	0.5008	0.1229	0.2371
114	Ni	8.6872	0.0042	5.2184	0.6249	0.0003	0.2377
115	Ni	6.9052	5.1813	5.2301	0.4967	0.3727	0.2383
116	Ni	8.6692	3.4363	5.1606	0.6236	0.2472	0.2351
117	Ni	10.4279	1.7356	5.2132	0.7501	0.1248	0.2375
118	Ni	12.1791	0.0145	5.2151	0.8761	0.0010	0.2376
119	Ni	10.4020	5.1182	5.3586	0.7482	0.3682	0.2441
120	Ni	12.1916	3.4389	5.2102	0.8770	0.2474	0.2374
121	Ni	6.9939	8.7245	5.2480	0.5031	0.6276	0.2391
122	Ni	8.6853	6.9338	5.2424	0.6248	0.4988	0.2388
123	Ni	6.9508	12.1686	5.2210	0.5000	0.8753	0.2378
124	Ni	8.7091	10.4721	5.2139	0.6265	0.7533	0.2375
125	Ni	10.4217	8.7236	5.1118	0.7497	0.6275	0.2329
126	Ni	12.1771	6.9829	5.4011	0.8759	0.5023	0.2461
127	Ni	10.4437	12.1972	5.2810	0.7512	0.8774	0.2406
128	Ni	12.1734	10.4325	5.2682	0.8757	0.7504	0.2400
129	O	5.9914	4.4241	6.8576	0.4310	0.3182	0.3124
130	O	4.1796	7.6841	6.7761	0.3006	0.5527	0.3087
131	O	5.9331	6.6007	8.7659	0.4268	0.4748	0.3993
132	O	7.9042	7.7572	6.8871	0.5686	0.5580	0.3137
133	O	10.4254	10.4503	6.2779	0.7499	0.7517	0.2860
134	O	9.9069	6.8885	8.8305	0.7126	0.4955	0.4023
135	O	7.7767	9.7399	8.9052	0.5594	0.7006	0.4057
136	Ce	5.9942	8.8340	7.9898	0.4312	0.6354	0.3640
137	Ce	4.1371	5.5802	7.6639	0.2976	0.4014	0.3491
138	Ce	7.7914	5.5792	7.8772	0.5604	0.4013	0.3589

139	Sm	9.4927	9.0813	7.9039	0.6828	0.6532	0.3601
140	C	10.6663	6.4355	7.8465	0.7673	0.4629	0.3575
141	O	11.5599	7.2144	7.3425	0.8315	0.5189	0.3345
142	O	10.3605	5.2622	7.3692	0.7453	0.3785	0.3357
143	O	1.9300	5.3007	6.3300	0.1388	0.3813	0.2884
144	O	1.7391	1.6580	6.1906	0.1251	0.1193	0.2820

/db/jmorales/CoNi-alloy/Profiles/Ni(001)/CO_2

a =		6.950991869					
b =		6.950991869					
c =		21.950991869					
alpha =		90.0					
beta =		90.0					
gamma =		90.0					
	Atom	X	Y	Z	X	Y	Z
1	Ni	0.0000	0.0000	0.0000	0.0000	0.0000	0.0000
2	Ni	1.7378	1.7378	0.0000	0.2500	0.2500	0.0000
3	Ni	0.0000	3.4755	0.0000	0.0000	0.5000	0.0000
4	Ni	1.7378	5.2132	0.0000	0.2500	0.7500	0.0000
5	Ni	3.4755	0.0000	0.0000	0.5000	0.0000	0.0000
6	Ni	5.2132	1.7378	0.0000	0.7500	0.2500	0.0000
7	Ni	3.4755	3.4755	0.0000	0.5000	0.5000	0.0000
8	Ni	5.2132	5.2132	0.0000	0.7500	0.7500	0.0000
9	Ni	0.0000	1.7378	1.7378	0.0000	0.2500	0.0792
10	Ni	1.7378	0.0000	1.7378	0.2500	0.0000	0.0792
11	Ni	0.0000	5.2132	1.7378	0.0000	0.7500	0.0792
12	Ni	1.7378	3.4755	1.7378	0.2500	0.5000	0.0792
13	Ni	3.4755	1.7378	1.7378	0.5000	0.2500	0.0792
14	Ni	5.2132	0.0000	1.7378	0.7500	0.0000	0.0792
15	Ni	3.4755	5.2132	1.7378	0.5000	0.7500	0.0792
16	Ni	5.2132	3.4755	1.7378	0.7500	0.5000	0.0792
17	Ni	0.0050	0.0001	3.4764	0.0007	0.0000	0.1584
18	Ni	1.7479	1.7571	3.5157	0.2515	0.2528	0.1602
19	Ni	6.9446	3.4723	3.5083	0.9991	0.4995	0.1598
20	Ni	1.7448	5.2009	3.5015	0.2510	0.7482	0.1595
21	Ni	3.4742	0.0093	3.5120	0.4998	0.0013	0.1600
22	Ni	5.2097	1.7424	3.5044	0.7495	0.2507	0.1596
23	Ni	3.4728	3.4676	3.5071	0.4996	0.4989	0.1598
24	Ni	5.2035	5.1991	3.4938	0.7486	0.7480	0.1592
25	Ni	0.0055	1.6718	5.1918	0.0008	0.2405	0.2365
26	Ni	1.7550	6.9455	5.1977	0.2525	0.9992	0.2368
27	Ni	6.9420	5.2754	5.1851	0.9987	0.7589	0.2362
28	Ni	1.6250	3.4866	5.3156	0.2338	0.5016	0.2422
29	Ni	3.4964	1.6893	5.2921	0.5030	0.2430	0.2411
30	Ni	5.2034	6.9146	5.1923	0.7486	0.9948	0.2365
31	Ni	3.4667	5.1756	5.2800	0.4987	0.7446	0.2405
32	Ni	5.3190	3.4923	5.3026	0.7652	0.5024	0.2416
33	C	3.0948	3.8408	6.6423	0.4452	0.5526	0.3026
34	O	2.1439	3.9476	7.4480	0.3084	0.5679	0.3393
35	O	4.1054	2.9593	6.7532	0.5906	0.4257	0.3076

/db/jmorales/CoNi-alloy/Profiles/Ni(111)/TS

a =			9.829999924				
b =			9.82999923668225				
c =			21.019699097				
alpha =			90.0				
beta =			90.0				
gamma =			120.0				
	Atom	X	Y	Z	X	Y	Z
1	Ni	0.0000	0.0000	0.0000	0.0000	0.0000	0.0000
2	Ni	-1.2288	2.1283	0.0000	0.0000	0.2500	0.0000
3	Ni	-2.4575	4.2565	0.0000	-0.0000	0.5000	0.0000
4	Ni	-3.6862	6.3848	0.0000	-0.0000	0.7500	0.0000
5	Ni	2.4575	0.0000	0.0000	0.2500	0.0000	0.0000
6	Ni	1.2288	2.1283	0.0000	0.2500	0.2500	0.0000
7	Ni	-0.0000	4.2565	0.0000	0.2500	0.5000	0.0000
8	Ni	-1.2288	6.3848	0.0000	0.2500	0.7500	0.0000
9	Ni	4.9150	0.0000	0.0000	0.5000	0.0000	0.0000
10	Ni	3.6862	2.1283	0.0000	0.5000	0.2500	0.0000
11	Ni	2.4575	4.2565	0.0000	0.5000	0.5000	0.0000
12	Ni	1.2288	6.3848	0.0000	0.5000	0.7500	0.0000
13	Ni	7.3725	0.0000	0.0000	0.7500	0.0000	0.0000
14	Ni	6.1438	2.1283	0.0000	0.7500	0.2500	0.0000
15	Ni	4.9150	4.2565	0.0000	0.7500	0.5000	0.0000
16	Ni	3.6862	6.3848	0.0000	0.7500	0.7500	0.0000
17	Ni	-0.0000	1.4188	2.0065	0.0833	0.1667	0.0955
18	Ni	-1.2288	3.5471	2.0065	0.0833	0.4167	0.0955
19	Ni	-2.4575	5.6754	2.0065	0.0833	0.6667	0.0955
20	Ni	-3.6863	7.8036	2.0065	0.0833	0.9167	0.0955
21	Ni	2.4575	1.4188	2.0065	0.3333	0.1667	0.0955
22	Ni	1.2287	3.5471	2.0065	0.3333	0.4167	0.0955
23	Ni	-0.0000	5.6754	2.0065	0.3333	0.6667	0.0955
24	Ni	-1.2288	7.8036	2.0065	0.3333	0.9167	0.0955
25	Ni	4.9150	1.4188	2.0065	0.5833	0.1667	0.0955
26	Ni	3.6862	3.5471	2.0065	0.5833	0.4167	0.0955
27	Ni	2.4575	5.6754	2.0065	0.5833	0.6667	0.0955
28	Ni	1.2287	7.8036	2.0065	0.5833	0.9167	0.0955
29	Ni	7.3725	1.4188	2.0065	0.8333	0.1667	0.0955
30	Ni	6.1437	3.5471	2.0065	0.8333	0.4167	0.0955
31	Ni	4.9150	5.6754	2.0065	0.8333	0.6667	0.0955
32	Ni	3.6862	7.8036	2.0065	0.8333	0.9167	0.0955
33	Ni	1.2184	0.7011	4.0263	0.1651	0.0824	0.1915
34	Ni	-0.0114	2.8252	4.0291	0.1648	0.3319	0.1917
35	Ni	-1.2386	4.9596	4.0256	0.1653	0.5826	0.1915
36	Ni	-2.4656	7.0910	4.0283	0.1657	0.8330	0.1916
37	Ni	3.6767	0.7122	4.0518	0.4159	0.0837	0.1928
38	Ni	2.4466	2.8296	4.0179	0.4151	0.3324	0.1911
39	Ni	1.2112	4.9574	4.0065	0.4144	0.5823	0.1906
40	Ni	-0.0141	7.0928	4.0273	0.4151	0.8332	0.1916
41	Ni	6.1343	0.7049	4.0285	0.6654	0.0828	0.1917

42	Ni	4.8907	2.8222	4.0551	0.6633	0.3315	0.1929
43	Ni	3.6795	4.9529	4.0145	0.6652	0.5818	0.1910
44	Ni	2.4496	7.0872	4.0259	0.6655	0.8325	0.1915
45	Ni	8.5931	0.7008	4.0277	0.9153	0.0823	0.1916
46	Ni	7.3578	2.8318	4.0307	0.9148	0.3326	0.1918
47	Ni	6.1340	4.9558	4.0275	0.9151	0.5821	0.1916
48	Ni	4.9097	7.0847	4.0279	0.9156	0.8322	0.1916
49	Ni	4.8859	8.4903	6.0672	0.9957	0.9973	0.2886
50	Ni	8.5626	2.1249	6.0592	0.9959	0.2496	0.2883
51	Ni	7.3557	4.2463	6.0689	0.9977	0.4988	0.2887
52	Ni	6.1310	6.3677	6.0646	0.9977	0.7480	0.2885
53	Ni	-2.4815	8.4635	6.0662	0.2447	0.9942	0.2886
54	Ni	1.1732	2.1166	6.0735	0.2437	0.2486	0.2889
55	Ni	-0.0321	4.2734	6.0596	0.2477	0.5020	0.2883
56	Ni	-1.2456	6.3758	6.0632	0.2478	0.7490	0.2885
57	Ni	-0.0205	8.5128	6.0721	0.4979	1.0000	0.2889
58	Ni	3.6143	2.1446	6.1986	0.4936	0.2519	0.2949
59	Ni	2.4327	4.3460	6.0626	0.5027	0.5105	0.2884
60	Ni	1.2006	6.4169	6.0499	0.4990	0.7538	0.2878
61	Ni	7.3521	0.0066	6.0748	0.7483	0.0008	0.2890
62	Ni	6.1097	2.1255	6.0702	0.7464	0.2497	0.2888
63	Ni	4.9098	4.2687	6.0614	0.7502	0.5014	0.2884
64	Ni	3.6832	6.4024	6.0572	0.7507	0.7521	0.2882
65	C	2.1322	4.0748	7.8259	0.4562	0.4787	0.3723
66	O	1.9802	4.4277	8.9397	0.4615	0.5201	0.4253
67	O	2.2700	2.3256	7.5566	0.3675	0.2732	0.3595

/db/jmorales/CoNi-alloy/Profiles/Ni-Ce_3SmO_7/O-coverage/M-CO-O-O-coverage/CO-O-Ni-Ce_3SmO_7-4O

a =			13.9019837372				
b =			13.9019837372				
c =			21.9509918687				
alpha =			90.0				
beta =			90.0				
gamma =			90.0				
	Atom	X	Y	Z	X	Y	Z
1	Ni	0.0000	0.0000	0.0000	0.0000	0.0000	0.0000
2	Ni	1.7377	1.7377	0.0000	0.1250	0.1250	0.0000
3	Ni	0.0000	3.4755	0.0000	0.0000	0.2500	0.0000
4	Ni	1.7377	5.2132	0.0000	0.1250	0.3750	0.0000
5	Ni	3.4755	0.0000	0.0000	0.2500	0.0000	0.0000
6	Ni	5.2132	1.7377	0.0000	0.3750	0.1250	0.0000
7	Ni	3.4755	3.4755	0.0000	0.2500	0.2500	0.0000
8	Ni	5.2132	5.2132	0.0000	0.3750	0.3750	0.0000
9	Ni	0.0000	6.9510	0.0000	0.0000	0.5000	0.0000
10	Ni	1.7377	8.6887	0.0000	0.1250	0.6250	0.0000
11	Ni	0.0000	10.4265	0.0000	0.0000	0.7500	0.0000
12	Ni	1.7377	12.1642	0.0000	0.1250	0.8750	0.0000
13	Ni	3.4755	6.9510	0.0000	0.2500	0.5000	0.0000
14	Ni	5.2132	8.6887	0.0000	0.3750	0.6250	0.0000
15	Ni	3.4755	10.4265	0.0000	0.2500	0.7500	0.0000
16	Ni	5.2132	12.1642	0.0000	0.3750	0.8750	0.0000
17	Ni	6.9510	0.0000	0.0000	0.5000	0.0000	0.0000
18	Ni	8.6887	1.7377	0.0000	0.6250	0.1250	0.0000
19	Ni	6.9510	3.4755	0.0000	0.5000	0.2500	0.0000
20	Ni	8.6887	5.2132	0.0000	0.6250	0.3750	0.0000
21	Ni	10.4265	0.0000	0.0000	0.7500	0.0000	0.0000
22	Ni	12.1642	1.7377	0.0000	0.8750	0.1250	0.0000
23	Ni	10.4265	3.4755	0.0000	0.7500	0.2500	0.0000
24	Ni	12.1642	5.2132	0.0000	0.8750	0.3750	0.0000
25	Ni	6.9510	6.9510	0.0000	0.5000	0.5000	0.0000
26	Ni	8.6887	8.6887	0.0000	0.6250	0.6250	0.0000
27	Ni	6.9510	10.4265	0.0000	0.5000	0.7500	0.0000
28	Ni	8.6887	12.1642	0.0000	0.6250	0.8750	0.0000
29	Ni	10.4265	6.9510	0.0000	0.7500	0.5000	0.0000
30	Ni	12.1642	8.6887	0.0000	0.8750	0.6250	0.0000
31	Ni	10.4265	10.4265	0.0000	0.7500	0.7500	0.0000
32	Ni	12.1642	12.1642	0.0000	0.8750	0.8750	0.0000
33	Ni	0.0000	1.7377	1.7377	0.0000	0.1250	0.0792
34	Ni	1.7377	0.0000	1.7377	0.1250	0.0000	0.0792
35	Ni	0.0000	5.2132	1.7377	0.0000	0.3750	0.0792
36	Ni	1.7377	3.4755	1.7377	0.1250	0.2500	0.0792
37	Ni	3.4755	1.7377	1.7377	0.2500	0.1250	0.0792
38	Ni	5.2132	0.0000	1.7377	0.3750	0.0000	0.0792
39	Ni	3.4755	5.2132	1.7377	0.2500	0.3750	0.0792

40	Ni	5.2132	3.4755	1.7377	0.3750	0.2500	0.0792
41	Ni	0.0000	8.6887	1.7377	0.0000	0.6250	0.0792
42	Ni	1.7377	6.9510	1.7377	0.1250	0.5000	0.0792
43	Ni	0.0000	12.1642	1.7377	0.0000	0.8750	0.0792
44	Ni	1.7377	10.4265	1.7377	0.1250	0.7500	0.0792
45	Ni	3.4755	8.6887	1.7377	0.2500	0.6250	0.0792
46	Ni	5.2132	6.9510	1.7377	0.3750	0.5000	0.0792
47	Ni	3.4755	12.1642	1.7377	0.2500	0.8750	0.0792
48	Ni	5.2132	10.4265	1.7377	0.3750	0.7500	0.0792
49	Ni	6.9510	1.7377	1.7377	0.5000	0.1250	0.0792
50	Ni	8.6887	0.0000	1.7377	0.6250	0.0000	0.0792
51	Ni	6.9510	5.2132	1.7377	0.5000	0.3750	0.0792
52	Ni	8.6887	3.4755	1.7377	0.6250	0.2500	0.0792
53	Ni	10.4265	1.7377	1.7377	0.7500	0.1250	0.0792
54	Ni	12.1642	0.0000	1.7377	0.8750	0.0000	0.0792
55	Ni	10.4265	5.2132	1.7377	0.7500	0.3750	0.0792
56	Ni	12.1642	3.4755	1.7377	0.8750	0.2500	0.0792
57	Ni	6.9510	8.6887	1.7377	0.5000	0.6250	0.0792
58	Ni	8.6887	6.9510	1.7377	0.6250	0.5000	0.0792
59	Ni	6.9510	12.1642	1.7377	0.5000	0.8750	0.0792
60	Ni	8.6887	10.4265	1.7377	0.6250	0.7500	0.0792
61	Ni	10.4265	8.6887	1.7377	0.7500	0.6250	0.0792
62	Ni	12.1642	6.9510	1.7377	0.8750	0.5000	0.0792
63	Ni	10.4265	12.1642	1.7377	0.7500	0.8750	0.0792
64	Ni	12.1642	10.4265	1.7377	0.8750	0.7500	0.0792
65	Ni	0.0015	0.0083	3.5070	0.0001	0.0006	0.1598
66	Ni	1.7257	1.7541	3.4637	0.1241	0.1262	0.1578
67	Ni	0.0049	3.4687	3.5039	0.0004	0.2495	0.1596
68	Ni	1.6942	5.1965	3.4241	0.1219	0.3738	0.1560
69	Ni	3.4728	13.8937	3.5046	0.2498	0.9994	0.1597
70	Ni	5.2138	1.7394	3.5464	0.3750	0.1251	0.1616
71	Ni	3.4962	3.4337	3.4719	0.2515	0.2470	0.1582
72	Ni	5.2018	5.2144	3.4717	0.3742	0.3751	0.1582
73	Ni	0.0061	6.9534	3.4960	0.0004	0.5002	0.1593
74	Ni	1.7394	8.6803	3.5282	0.1251	0.6244	0.1607
75	Ni	13.8676	10.4191	3.5229	0.9975	0.7495	0.1605
76	Ni	1.7201	12.1693	3.5250	0.1237	0.8754	0.1606
77	Ni	3.4642	6.9929	3.4601	0.2492	0.5030	0.1576
78	Ni	5.2367	8.7129	3.4985	0.3767	0.6267	0.1594
79	Ni	3.4671	10.4053	3.5191	0.2494	0.7485	0.1603
80	Ni	5.2226	12.1533	3.4935	0.3757	0.8742	0.1592
81	Ni	6.9501	13.9007	3.5028	0.4999	0.9999	0.1596
82	Ni	8.7015	1.7424	3.4437	0.6259	0.1253	0.1569
83	Ni	6.9351	3.4671	3.4933	0.4989	0.2494	0.1591
84	Ni	8.6656	5.2121	3.5405	0.6233	0.3749	0.1613
85	Ni	10.4418	0.0235	3.5091	0.7511	0.0017	0.1599
86	Ni	12.1716	1.7438	3.4774	0.8755	0.1254	0.1584
87	Ni	10.4262	3.4829	3.5020	0.7500	0.2505	0.1595
88	Ni	12.1885	5.1961	3.4882	0.8767	0.3738	0.1589

89	Ni	6.9328	6.9375	3.5122	0.4987	0.4990	0.1600
90	Ni	8.6958	8.6713	3.4778	0.6255	0.6237	0.1584
91	Ni	7.0102	10.4240	3.5274	0.5043	0.7498	0.1607
92	Ni	8.7195	12.1916	3.5272	0.6272	0.8770	0.1607
93	Ni	10.4051	6.9280	3.5022	0.7485	0.4983	0.1595
94	Ni	12.1630	8.6806	3.5024	0.8749	0.6244	0.1596
95	Ni	10.4410	10.4347	3.4538	0.7510	0.7506	0.1573
96	Ni	12.1640	12.1787	3.4476	0.8750	0.8760	0.1571
97	Ni	0.0374	1.7847	5.3744	0.0027	0.1284	0.2448
98	Ni	1.7301	13.8772	5.2818	0.1244	0.9982	0.2406
99	Ni	13.8977	5.2023	5.3312	0.9997	0.3742	0.2429
100	Ni	1.7723	3.4925	5.3187	0.1275	0.2512	0.2423
101	Ni	3.4825	1.7103	5.2813	0.2505	0.1230	0.2406
102	Ni	5.2153	13.8702	5.2050	0.3751	0.9977	0.2371
103	Ni	3.4416	5.1993	5.0296	0.2476	0.3740	0.2291
104	Ni	5.1730	3.4442	5.3357	0.3721	0.2477	0.2431
105	Ni	13.8942	8.6874	5.2063	0.9994	0.6249	0.2372
106	Ni	1.7022	6.9332	5.2533	0.1224	0.4987	0.2393
107	Ni	13.8699	12.1361	5.2679	0.9977	0.8730	0.2400
108	Ni	1.7166	10.4399	5.2061	0.1235	0.7510	0.2372
109	Ni	3.4491	8.7615	5.3488	0.2481	0.6302	0.2437
110	Ni	5.1749	6.9791	5.2013	0.3722	0.5020	0.2369
111	Ni	3.4815	12.1398	5.2098	0.2504	0.8732	0.2373
112	Ni	5.2455	10.4480	5.1862	0.3773	0.7515	0.2363
113	Ni	6.9313	1.7192	5.2640	0.4986	0.1237	0.2398
114	Ni	8.6855	0.0310	5.2707	0.6248	0.0022	0.2401
115	Ni	6.8981	5.1799	5.2519	0.4962	0.3726	0.2393
116	Ni	8.6967	3.4730	5.2510	0.6256	0.2498	0.2392
117	Ni	10.4315	1.7661	5.3800	0.7504	0.1270	0.2451
118	Ni	12.2064	0.0572	5.3806	0.8780	0.0041	0.2451
119	Ni	10.4533	5.2219	5.2648	0.7519	0.3756	0.2398
120	Ni	12.1691	3.5258	5.3289	0.8754	0.2536	0.2428
121	Ni	6.9886	8.7345	5.2500	0.5027	0.6283	0.2392
122	Ni	8.6722	6.9028	5.2922	0.6238	0.4965	0.2411
123	Ni	6.9762	12.1516	5.2147	0.5018	0.8741	0.2376
124	Ni	8.7904	10.4680	5.2659	0.6323	0.7530	0.2399
125	Ni	10.4523	8.5754	5.1924	0.7519	0.6168	0.2365
126	Ni	12.1902	6.9068	5.2399	0.8769	0.4968	0.2387
127	Ni	10.4740	12.2596	5.2643	0.7534	0.8819	0.2398
128	Ni	12.0862	10.3847	5.3823	0.8694	0.7470	0.2452
129	O	5.9582	4.4342	6.7922	0.4286	0.3190	0.3094
130	O	4.1309	7.6296	6.7256	0.2971	0.5488	0.3064
131	O	5.8310	6.6070	8.7805	0.4194	0.4753	0.4000
132	O	7.7889	7.8226	6.8293	0.5603	0.5627	0.3111
133	O	10.3106	10.0010	6.3823	0.7417	0.7194	0.2908
134	O	9.3539	6.8671	9.0057	0.6728	0.4940	0.4103
135	O	7.5830	9.7247	8.9788	0.5455	0.6995	0.4090
136	Ce	5.8745	8.8383	8.0176	0.4226	0.6358	0.3653
137	Ce	4.1420	5.5370	7.6863	0.2979	0.3983	0.3502

138	Ce	7.8672	5.6775	8.0728	0.5659	0.4084	0.3678
139	Sm	9.3652	8.8661	8.2257	0.6737	0.6378	0.3747
140	C	12.1461	5.2782	6.3824	0.8737	0.3797	0.2908
141	O	12.1582	5.2696	7.5998	0.8746	0.3791	0.3462
142	O	12.1808	1.8160	6.1945	0.8762	0.1306	0.2822
143	O	1.9410	5.2857	6.3031	0.1396	0.3802	0.2871
144	O	1.8173	1.6593	6.1981	0.1307	0.1194	0.2824
145	O	8.6092	1.7699	6.1801	0.6193	0.1273	0.2815
146	O	12.2176	12.2026	6.1855	0.8788	0.8778	0.2818

/db/jmorales/CoNi-alloy/Profiles/Ni(001)/TS

a = 6.950991869
b = 6.950991869
c = 21.950991869
alpha = 90.0
beta = 90.0
gamma = 90.0

	Atom	X	Y	Z	X	Y	Z
1	Ni	0.0000	0.0000	0.0000	0.0000	0.0000	0.0000
2	Ni	1.7378	1.7378	0.0000	0.2500	0.2500	0.0000
3	Ni	0.0000	3.4755	0.0000	0.0000	0.5000	0.0000
4	Ni	1.7378	5.2132	0.0000	0.2500	0.7500	0.0000
5	Ni	3.4755	0.0000	0.0000	0.5000	0.0000	0.0000
6	Ni	5.2132	1.7378	0.0000	0.7500	0.2500	0.0000
7	Ni	3.4755	3.4755	0.0000	0.5000	0.5000	0.0000
8	Ni	5.2132	5.2132	0.0000	0.7500	0.7500	0.0000
9	Ni	0.0000	1.7378	1.7378	0.0000	0.2500	0.0792
10	Ni	1.7378	0.0000	1.7378	0.2500	0.0000	0.0792
11	Ni	0.0000	5.2132	1.7378	0.0000	0.7500	0.0792
12	Ni	1.7378	3.4755	1.7378	0.2500	0.5000	0.0792
13	Ni	3.4755	1.7378	1.7378	0.5000	0.2500	0.0792
14	Ni	5.2132	0.0000	1.7378	0.7500	0.0000	0.0792
15	Ni	3.4755	5.2132	1.7378	0.5000	0.7500	0.0792
16	Ni	5.2132	3.4755	1.7378	0.7500	0.5000	0.0792
17	Ni	0.0015	6.9450	3.4944	0.0002	0.9991	0.1592
18	Ni	1.7520	1.7309	3.5102	0.2520	0.2490	0.1599
19	Ni	6.9453	3.4626	3.5229	0.9992	0.4981	0.1605
20	Ni	1.7479	5.1985	3.5054	0.2515	0.7479	0.1597
21	Ni	3.4850	6.9357	3.5388	0.5014	0.9978	0.1612
22	Ni	5.2160	1.7288	3.5064	0.7504	0.2487	0.1597
23	Ni	3.4838	3.4613	3.5103	0.5012	0.4980	0.1599
24	Ni	5.2081	5.1948	3.5179	0.7493	0.7473	0.1603
25	Ni	0.0270	1.6767	5.2104	0.0039	0.2412	0.2374
26	Ni	1.7364	6.9429	5.2127	0.2498	0.9988	0.2375
27	Ni	0.0102	5.2422	5.2037	0.0015	0.7542	0.2371
28	Ni	1.7042	3.4513	5.2534	0.2452	0.4965	0.2393
29	Ni	3.5000	1.6634	5.3205	0.5035	0.2393	0.2424
30	Ni	5.2355	6.9000	5.2216	0.7532	0.9927	0.2379
31	Ni	3.4626	5.1885	5.3152	0.4982	0.7464	0.2421
32	Ni	5.3214	3.4678	5.3325	0.7656	0.4989	0.2429
33	C	2.8052	4.0681	6.7502	0.4036	0.5853	0.3075
34	O	2.4412	4.0620	7.8747	0.3512	0.5844	0.3587
35	O	4.2232	2.7584	6.5572	0.6076	0.3968	0.2987

/db/jmorales/CoNi-alloy/Profiles/Ni-CoNi(001)/TS

a =		6.951669213					
b =		6.951669213					
c =		21.951669213					
alpha =		90.0					
beta =		90.0					
gamma =		90.0					
	Atom	X	Y	Z	X	Y	Z
1	Co	0.0000	0.0000	0.0000	0.0000	0.0000	0.0000
2	Co	1.7379	1.7379	0.0000	0.2500	0.2500	0.0000
3	Co	0.0000	3.4758	0.0000	0.0000	0.5000	0.0000
4	Co	1.7379	5.2138	0.0000	0.2500	0.7500	0.0000
5	Co	3.4758	0.0000	0.0000	0.5000	0.0000	0.0000
6	Co	5.2138	1.7379	0.0000	0.7500	0.2500	0.0000
7	Co	3.4758	3.4758	0.0000	0.5000	0.5000	0.0000
8	Co	5.2138	5.2138	0.0000	0.7500	0.7500	0.0000
9	Co	0.0021	6.9497	3.5685	0.0003	0.9997	0.1626
10	Co	1.7542	1.7319	3.5777	0.2523	0.2491	0.1630
11	Co	0.0064	3.4655	3.5793	0.0009	0.4985	0.1631
12	Co	1.7477	5.2041	3.5808	0.2514	0.7486	0.1631
13	Co	3.4861	6.9450	3.5793	0.5015	0.9990	0.1631
14	Co	5.2109	1.7407	3.5779	0.7496	0.2504	0.1630
15	Co	3.4770	3.4747	3.5723	0.5002	0.4998	0.1627
16	Co	5.2194	5.1976	3.5780	0.7508	0.7477	0.1630
17	Ni	1.7379	0.0000	1.7379	0.2500	0.0000	0.0792
18	Ni	0.0000	1.7379	1.7379	0.0000	0.2500	0.0792
19	Ni	1.7379	3.4758	1.7379	0.2500	0.5000	0.0792
20	Ni	0.0000	5.2138	1.7379	0.0000	0.7500	0.0792
21	Ni	5.2138	0.0000	1.7379	0.7500	0.0000	0.0792
22	Ni	3.4758	1.7379	1.7379	0.5000	0.2500	0.0792
23	Ni	5.2138	3.4758	1.7379	0.7500	0.5000	0.0792
24	Ni	3.4758	5.2138	1.7379	0.5000	0.7500	0.0792
25	Ni	1.7260	0.0074	5.2213	0.2483	0.0011	0.2379
26	Ni	0.0273	1.7076	5.2318	0.0039	0.2456	0.2383
27	Ni	1.7388	3.4842	5.3000	0.2501	0.5012	0.2414
28	Ni	6.9444	5.2251	5.2207	0.9990	0.7516	0.2378
29	Ni	5.2444	6.9240	5.2321	0.7544	0.9960	0.2383
30	Ni	3.4883	1.6895	5.3374	0.5018	0.2430	0.2431
31	Ni	5.2614	3.4638	5.3374	0.7569	0.4983	0.2431
32	Ni	3.4667	5.2129	5.3007	0.4987	0.7499	0.2415
33	C	2.8270	4.1239	6.8583	0.4067	0.5932	0.3124
34	O	2.5919	4.3583	7.9977	0.3728	0.6269	0.3643
35	O	4.0773	2.8740	6.6392	0.5865	0.4134	0.3024

/db/jmorales/CoNi-alloy/Profiles/Sm(subsurf)$Ce_{26}O_{53}$/O

a =			11.6082000732				
b =			11.608200073110298				
c =			22.898399353				
alpha =			90.0				
beta =			90.0				
gamma =			120.0				
	Atom	X	Y	Z	X	Y	Z
1	Sm	1.9354	5.5853	3.9680	0.4445	0.5556	0.1733
2	Ce	1.9347	1.1170	0.7898	0.2222	0.1111	0.0345
3	Ce	0.0000	4.4680	0.7898	0.2222	0.4444	0.0345
4	Ce	-1.9347	7.8190	0.7898	0.2222	0.7778	0.0345
5	Ce	5.8041	1.1170	0.7898	0.5556	0.1111	0.0345
6	Ce	3.8694	4.4680	0.7898	0.5556	0.4444	0.0345
7	Ce	1.9347	7.8190	0.7898	0.5556	0.7778	0.0345
8	Ce	9.6735	1.1170	0.7898	0.8889	0.1111	0.0345
9	Ce	7.7388	4.4680	0.7898	0.8889	0.4444	0.0345
10	Ce	5.8041	7.8190	0.7898	0.8889	0.7778	0.0345
11	Ce	0.0072	2.2490	3.9594	0.1125	0.2237	0.1729
12	Ce	-1.9179	5.5835	3.9594	0.1125	0.5554	0.1729
13	Ce	-3.8684	8.9365	3.9596	0.1112	0.8889	0.1729
14	Ce	3.8641	2.2493	3.9594	0.4448	0.2237	0.1729
15	Ce	0.0110	8.9232	3.9597	0.4448	0.8876	0.1729
16	Ce	7.7400	2.2343	3.9569	0.7779	0.2222	0.1728
17	Ce	5.7883	5.5834	3.9594	0.7763	0.5554	0.1729
18	Ce	3.8602	8.9225	3.9596	0.7763	0.8875	0.1729
19	Ce	-0.0007	0.0023	7.1036	0.0001	0.0002	0.3102
20	Ce	-1.9332	3.3510	7.1176	0.0001	0.3333	0.3108
21	Ce	-3.8669	6.6988	7.1036	0.0001	0.6664	0.3102
22	Ce	3.8759	0.0029	7.1042	0.3340	0.0003	0.3103
23	Ce	1.9376	3.3550	7.1057	0.3338	0.3337	0.3103
24	Ce	0.0059	6.7013	7.1058	0.3338	0.6666	0.3103
25	Ce	7.7422	0.0025	7.1184	0.6671	0.0002	0.3109
26	Ce	5.8086	3.3514	7.1180	0.6671	0.3334	0.3109
27	Ce	3.8689	6.7008	7.1061	0.6666	0.6665	0.3103
28	O	0.0000	0.0000	0.0000	0.0000	0.0000	0.0000
29	O	-1.9347	3.3510	0.0000	-0.0000	0.3333	0.0000
30	O	-3.8694	6.7020	0.0000	-0.0000	0.6667	0.0000
31	O	3.8694	0.0000	0.0000	0.3333	0.0000	0.0000
32	O	1.9347	3.3510	0.0000	0.3333	0.3333	0.0000
33	O	0.0000	6.7020	0.0000	0.3333	0.6667	0.0000
34	O	7.7388	0.0000	0.0000	0.6667	0.0000	0.0000
35	O	5.8041	3.3510	0.0000	0.6667	0.3333	0.0000
36	O	3.8694	6.7020	0.0000	0.6667	0.6667	0.0000
37	O	-0.0000	2.2340	1.5798	0.1111	0.2222	0.0690
38	O	-1.9347	5.5850	1.5798	0.1111	0.5556	0.0690
39	O	-3.8694	8.9360	1.5798	0.1111	0.8889	0.0690
40	O	3.8694	2.2340	1.5798	0.4444	0.2222	0.0690

41	O	1.9347	5.5850	1.5798	0.4444	0.5556	0.0690
42	O	-0.0000	8.9360	1.5798	0.4444	0.8889	0.0690
43	O	7.7388	2.2340	1.5798	0.7778	0.2222	0.0690
44	O	5.8041	5.5850	1.5798	0.7778	0.5556	0.0690
45	O	3.8694	8.9360	1.5798	0.7778	0.8889	0.0690
46	O	1.9347	1.1170	3.1593	0.2222	0.1111	0.1380
47	O	0.0000	4.4680	3.1593	0.2222	0.4444	0.1380
48	O	-1.9347	7.8190	3.1593	0.2222	0.7778	0.1380
49	O	5.8041	1.1170	3.1593	0.5556	0.1111	0.1380
50	O	3.8694	4.4680	3.1593	0.5556	0.4444	0.1380
51	O	1.9347	7.8190	3.1593	0.5556	0.7778	0.1380
52	O	9.6735	1.1170	3.1593	0.8889	0.1111	0.1380
53	O	7.7388	4.4680	3.1593	0.8889	0.4444	0.1380
54	O	5.8041	7.8190	3.1593	0.8889	0.7778	0.1380
55	O	-5.8019	10.0524	4.7690	0.0002	0.9999	0.2083
56	O	9.6726	3.3500	4.7718	0.9999	0.3332	0.2084
57	O	-3.8684	6.7037	4.7688	0.0002	0.6668	0.2083
58	O	-1.9340	10.0528	4.7692	0.3334	1.0000	0.2083
59	O	1.9354	3.3013	4.8053	0.3309	0.3284	0.2099
60	O	-0.0420	6.7273	4.8057	0.3310	0.6692	0.2099
61	O	7.7401	0.0035	4.7722	0.6670	0.0003	0.2084
62	O	5.8081	3.3497	4.7723	0.6669	0.3332	0.2084
63	O	3.9136	6.7274	4.8050	0.6717	0.6692	0.2098
64	O	-0.0131	2.2288	6.3196	0.1097	0.2217	0.2760
65	O	-1.9450	5.5748	6.3193	0.1097	0.5545	0.2760
66	O	-3.8658	8.9365	6.3063	0.1114	0.8889	0.2754
67	O	3.8868	2.2301	6.3197	0.4458	0.2218	0.2760
68	O	0.0065	8.9526	6.3203	0.4458	0.8905	0.2760
69	O	7.7428	2.2355	6.3135	0.7782	0.2224	0.2757
70	O	5.8189	5.5749	6.3187	0.7786	0.5546	0.2759
71	O	3.8685	8.9526	6.3192	0.7785	0.8905	0.2760
72	O	1.9381	1.1137	7.8915	0.2223	0.1108	0.3446
73	O	-0.0139	4.4589	7.9014	0.2206	0.4435	0.3451
74	O	-1.9344	7.8217	7.8915	0.2224	0.7780	0.3446
75	O	5.8106	1.1204	7.9108	0.5563	0.1114	0.3455
76	O	3.8901	4.4588	7.9038	0.5569	0.4435	0.3452
77	O	1.9383	7.8402	7.9044	0.5569	0.7799	0.3452
78	O	9.6734	1.1192	7.9126	0.8890	0.1113	0.3456
79	O	7.7412	4.4655	7.9124	0.8890	0.4442	0.3455
80	O	5.8111	7.8223	7.8896	0.8897	0.7781	0.3445
81	O	1.9383	5.5866	6.3780	0.4448	0.5557	0.2785

/db/jmorales/CoNi-alloy/Profiles/CoNi-Ce_3SmO_7/TS-1

a =	13.903338426
b =	13.903338426
c =	21.951669213
alpha =	90.0
beta =	90.0
gamma =	90.0

	Atom	X	Y	Z	X	Y	Z
1	Ni	0.0000	0.0000	0.0000	0.0000	0.0000	0.0000
2	Ni	0.0000	3.4758	0.0000	0.0000	0.2500	0.0000
3	Ni	3.4758	0.0000	0.0000	0.2500	0.0000	0.0000
4	Ni	3.4758	3.4758	0.0000	0.2500	0.2500	0.0000
5	Ni	0.0000	6.9517	0.0000	0.0000	0.5000	0.0000
6	Ni	0.0000	10.4275	0.0000	0.0000	0.7500	0.0000
7	Ni	3.4758	6.9517	0.0000	0.2500	0.5000	0.0000
8	Ni	3.4758	10.4275	0.0000	0.2500	0.7500	0.0000
9	Ni	6.9517	0.0000	0.0000	0.5000	0.0000	0.0000
10	Ni	6.9517	3.4758	0.0000	0.5000	0.2500	0.0000
11	Ni	10.4275	0.0000	0.0000	0.7500	0.0000	0.0000
12	Ni	10.4275	3.4758	0.0000	0.7500	0.2500	0.0000
13	Ni	6.9517	6.9517	0.0000	0.5000	0.5000	0.0000
14	Ni	6.9517	10.4275	0.0000	0.5000	0.7500	0.0000
15	Ni	10.4275	6.9517	0.0000	0.7500	0.5000	0.0000
16	Ni	10.4275	10.4275	0.0000	0.7500	0.7500	0.0000
17	Ni	0.0000	1.7379	1.7379	0.0000	0.1250	0.0792
18	Ni	0.0000	5.2138	1.7379	0.0000	0.3750	0.0792
19	Ni	3.4758	1.7379	1.7379	0.2500	0.1250	0.0792
20	Ni	3.4758	5.2138	1.7379	0.2500	0.3750	0.0792
21	Ni	0.0000	8.6896	1.7379	0.0000	0.6250	0.0792
22	Ni	0.0000	12.1654	1.7379	0.0000	0.8750	0.0792
23	Ni	3.4758	8.6896	1.7379	0.2500	0.6250	0.0792
24	Ni	3.4758	12.1654	1.7379	0.2500	0.8750	0.0792
25	Ni	6.9517	1.7379	1.7379	0.5000	0.1250	0.0792
26	Ni	6.9517	5.2138	1.7379	0.5000	0.3750	0.0792
27	Ni	10.4275	1.7379	1.7379	0.7500	0.1250	0.0792
28	Ni	10.4275	5.2138	1.7379	0.7500	0.3750	0.0792
29	Ni	6.9517	8.6896	1.7379	0.5000	0.6250	0.0792
30	Ni	6.9517	12.1654	1.7379	0.5000	0.8750	0.0792
31	Ni	10.4275	8.6896	1.7379	0.7500	0.6250	0.0792
32	Ni	10.4275	12.1654	1.7379	0.7500	0.8750	0.0792
33	Ni	0.0012	13.9017	3.5144	0.0001	0.9999	0.1601
34	Ni	0.0008	3.4764	3.5170	0.0001	0.2500	0.1602
35	Ni	3.4665	13.8995	3.5094	0.2493	0.9997	0.1599
36	Ni	3.4635	3.4523	3.4867	0.2491	0.2483	0.1588
37	Ni	13.9029	6.9563	3.5314	1.0000	0.5003	0.1609
38	Ni	13.8907	10.4243	3.5017	0.9991	0.7498	0.1595
39	Ni	3.4839	6.9471	3.5085	0.2506	0.4997	0.1598
40	Ni	3.4726	10.3924	3.5163	0.2498	0.7475	0.1602

41	Ni	6.9480	0.0194	3.4992	0.4997	0.0014	0.1594
42	Ni	6.9575	3.4648	3.5525	0.5004	0.2492	0.1618
43	Ni	10.4342	13.8978	3.5107	0.7505	0.9996	0.1599
44	Ni	10.4302	3.4773	3.5129	0.7502	0.2501	0.1600
45	Ni	6.9693	6.9659	3.5047	0.5013	0.5010	0.1597
46	Ni	6.9632	10.4420	3.4827	0.5008	0.7510	0.1587
47	Ni	10.4318	6.9450	3.5041	0.7503	0.4995	0.1596
48	Ni	10.4312	10.4362	3.5069	0.7503	0.7506	0.1598
49	Ni	0.0049	1.7369	5.2269	0.0004	0.1249	0.2381
50	Ni	13.9015	5.2141	5.2453	0.9999	0.3750	0.2389
51	Ni	3.4334	1.7383	5.2213	0.2469	0.1250	0.2379
52	Ni	3.4611	5.1697	5.1134	0.2489	0.3718	0.2329
53	Ni	13.8734	8.7086	5.2181	0.9978	0.6264	0.2377
54	Ni	13.9017	12.1601	5.2250	0.9999	0.8746	0.2380
55	Ni	3.4460	8.6639	5.2646	0.2479	0.6232	0.2398
56	Ni	3.4317	12.1211	5.2158	0.2468	0.8718	0.2376
57	Ni	6.9956	1.7491	5.3440	0.5032	0.1258	0.2434
58	Ni	6.9628	5.1300	5.3866	0.5008	0.3690	0.2454
59	Ni	10.4448	1.7341	5.2183	0.7512	0.1247	0.2377
60	Ni	10.4260	5.2161	5.2282	0.7499	0.3752	0.2382
61	Ni	7.0026	8.7360	5.2313	0.5037	0.6283	0.2383
62	Ni	6.9430	12.1586	5.1769	0.4994	0.8745	0.2358
63	Ni	10.4242	8.6827	5.2206	0.7498	0.6245	0.2378
64	Ni	10.4526	12.1905	5.2948	0.7518	0.8768	0.2412
65	Co	1.7379	1.7379	0.0000	0.1250	0.1250	0.0000
66	Co	1.7379	5.2138	0.0000	0.1250	0.3750	0.0000
67	Co	5.2138	1.7379	0.0000	0.3750	0.1250	0.0000
68	Co	5.2138	5.2138	0.0000	0.3750	0.3750	0.0000
69	Co	1.7379	8.6896	0.0000	0.1250	0.6250	0.0000
70	Co	1.7379	12.1654	0.0000	0.1250	0.8750	0.0000
71	Co	5.2138	8.6896	0.0000	0.3750	0.6250	0.0000
72	Co	5.2138	12.1654	0.0000	0.3750	0.8750	0.0000
73	Co	8.6896	1.7379	0.0000	0.6250	0.1250	0.0000
74	Co	8.6896	5.2138	0.0000	0.6250	0.3750	0.0000
75	Co	12.1654	1.7379	0.0000	0.8750	0.1250	0.0000
76	Co	12.1654	5.2138	0.0000	0.8750	0.3750	0.0000
77	Co	8.6896	8.6896	0.0000	0.6250	0.6250	0.0000
78	Co	8.6896	12.1654	0.0000	0.6250	0.8750	0.0000
79	Co	12.1654	8.6896	0.0000	0.8750	0.6250	0.0000
80	Co	12.1654	12.1654	0.0000	0.8750	0.8750	0.0000
81	Co	1.7379	0.0000	1.7379	0.1250	0.0000	0.0792
82	Co	1.7379	3.4758	1.7379	0.1250	0.2500	0.0792
83	Co	5.2138	0.0000	1.7379	0.3750	0.0000	0.0792
84	Co	5.2138	3.4758	1.7379	0.3750	0.2500	0.0792
85	Co	1.7379	6.9517	1.7379	0.1250	0.5000	0.0792
86	Co	1.7379	10.4275	1.7379	0.1250	0.7500	0.0792
87	Co	5.2138	6.9517	1.7379	0.3750	0.5000	0.0792
88	Co	5.2138	10.4275	1.7379	0.3750	0.7500	0.0792
89	Co	8.6896	0.0000	1.7379	0.6250	0.0000	0.0792

90	Co	8.6896	3.4758	1.7379	0.6250	0.2500	0.0792
91	Co	12.1654	0.0000	1.7379	0.8750	0.0000	0.0792
92	Co	12.1654	3.4758	1.7379	0.8750	0.2500	0.0792
93	Co	8.6896	6.9517	1.7379	0.6250	0.5000	0.0792
94	Co	8.6896	10.4275	1.7379	0.6250	0.7500	0.0792
95	Co	12.1654	6.9517	1.7379	0.8750	0.5000	0.0792
96	Co	12.1654	10.4275	1.7379	0.8750	0.7500	0.0792
97	Co	1.7254	1.7332	3.5600	0.1241	0.1247	0.1622
98	Co	1.7058	5.2368	3.5455	0.1227	0.3767	0.1615
99	Co	5.2139	1.7210	3.5737	0.3750	0.1238	0.1628
100	Co	5.2516	5.1777	3.5406	0.3777	0.3724	0.1613
101	Co	1.7286	8.6720	3.5540	0.1243	0.6237	0.1619
102	Co	1.7336	12.1632	3.5433	0.1247	0.8748	0.1614
103	Co	5.2088	8.6786	3.5599	0.3746	0.6242	0.1622
104	Co	5.1940	12.1521	3.5515	0.3736	0.8740	0.1618
105	Co	8.6894	1.7248	3.5627	0.6250	0.1241	0.1623
106	Co	8.6671	5.1944	3.5830	0.6234	0.3736	0.1632
107	Co	12.1690	1.7402	3.5661	0.8753	0.1252	0.1625
108	Co	12.1676	5.2130	3.5743	0.8752	0.3749	0.1628
109	Co	8.6944	8.6893	3.5344	0.6253	0.6250	0.1610
110	Co	8.6968	12.1898	3.4792	0.6255	0.8768	0.1585
111	Co	12.1558	8.6918	3.5621	0.8743	0.6252	0.1623
112	Co	12.1555	12.1645	3.5766	0.8743	0.8749	0.1629
113	Co	1.7386	13.8868	5.2568	0.1250	0.9988	0.2395
114	Co	1.7355	3.4935	5.2484	0.1248	0.2513	0.2391
115	Co	5.1247	0.0088	5.2706	0.3686	0.0006	0.2401
116	Co	5.1580	3.4164	5.2319	0.3710	0.2457	0.2383
117	Co	1.7095	6.9527	5.3624	0.1230	0.5001	0.2443
118	Co	1.7104	10.4560	5.2116	0.1230	0.7520	0.2374
119	Co	5.2675	6.8908	5.1850	0.3789	0.4956	0.2362
120	Co	5.1896	10.3997	5.2147	0.3733	0.7480	0.2376
121	Co	8.6930	0.0015	5.3540	0.6252	0.0001	0.2439
122	Co	8.7353	3.4646	5.2519	0.6283	0.2492	0.2392
123	Co	12.1740	0.0002	5.2314	0.8756	0.0000	0.2383
124	Co	12.1681	3.4691	5.2521	0.8752	0.2495	0.2393
125	Co	8.7225	6.9685	5.1931	0.6274	0.5012	0.2366
126	Co	8.7504	10.4440	5.2311	0.6294	0.7512	0.2383
127	Co	12.1656	6.9545	5.2534	0.8750	0.5002	0.2393
128	Co	12.1697	10.4233	5.2470	0.8753	0.7497	0.2390
129	O	6.0094	6.1101	6.7763	0.4322	0.4395	0.3087
130	O	2.6626	7.7998	6.8492	0.1915	0.5610	0.3120
131	O	4.9090	7.8342	8.7854	0.3531	0.5635	0.4002
132	O	6.0966	9.7775	6.8038	0.4385	0.7032	0.3099
133	O	7.9956	9.7400	8.9434	0.5751	0.7005	0.4074
134	O	5.1112	11.3022	9.0291	0.3676	0.8129	0.4113
135	O	8.7687	12.0091	6.3183	0.6307	0.8638	0.2878
136	Ce	3.9438	9.8402	8.0553	0.2837	0.7078	0.3670
137	Ce	3.9049	6.1232	7.7280	0.2809	0.4404	0.3520
138	Ce	7.1723	7.9878	8.0773	0.5159	0.5745	0.3680

139 Sm	7.0839	11.5583	8.2030	0.5095	0.8313	0.3737
140 C	7.4686	0.5865	6.8294	0.5372	0.0422	0.3111
141 O	6.1662	13.2184	6.6268	0.4435	0.9507	0.3019
142 O	7.7306	0.5947	8.0136	0.5560	0.0428	0.3651

/db/jmorales/CoNi-alloy/Profiles/CoNi-Ce_3SmO_7/O-coverage/TS-O-coverage/TS-CoNi-Ce_3SmO_7-O

a = 13.9033384264
b = 13.9033384264
c = 21.9516692133
alpha = 90.0
beta = 90.0
gamma = 90.0

	Atom	X	Y	Z	X	Y	Z
1	Ni	0.0000	0.0000	0.0000	0.0000	0.0000	0.0000
2	Ni	0.0000	3.4758	0.0000	0.0000	0.2500	0.0000
3	Ni	3.4758	0.0000	0.0000	0.2500	0.0000	0.0000
4	Ni	3.4758	3.4758	0.0000	0.2500	0.2500	0.0000
5	Ni	0.0000	6.9517	0.0000	0.0000	0.5000	0.0000
6	Ni	0.0000	10.4275	0.0000	0.0000	0.7500	0.0000
7	Ni	3.4758	6.9517	0.0000	0.2500	0.5000	0.0000
8	Ni	3.4758	10.4275	0.0000	0.2500	0.7500	0.0000
9	Ni	6.9517	0.0000	0.0000	0.5000	0.0000	0.0000
10	Ni	6.9517	3.4758	0.0000	0.5000	0.2500	0.0000
11	Ni	10.4275	0.0000	0.0000	0.7500	0.0000	0.0000
12	Ni	10.4275	3.4758	0.0000	0.7500	0.2500	0.0000
13	Ni	6.9517	6.9517	0.0000	0.5000	0.5000	0.0000
14	Ni	6.9517	10.4275	0.0000	0.5000	0.7500	0.0000
15	Ni	10.4275	6.9517	0.0000	0.7500	0.5000	0.0000
16	Ni	10.4275	10.4275	0.0000	0.7500	0.7500	0.0000
17	Ni	0.0000	1.7379	1.7379	0.0000	0.1250	0.0792
18	Ni	0.0000	5.2138	1.7379	0.0000	0.3750	0.0792
19	Ni	3.4758	1.7379	1.7379	0.2500	0.1250	0.0792
20	Ni	3.4758	5.2138	1.7379	0.2500	0.3750	0.0792
21	Ni	0.0000	8.6896	1.7379	0.0000	0.6250	0.0792
22	Ni	0.0000	12.1654	1.7379	0.0000	0.8750	0.0792
23	Ni	3.4758	8.6896	1.7379	0.2500	0.6250	0.0792
24	Ni	3.4758	12.1654	1.7379	0.2500	0.8750	0.0792
25	Ni	6.9517	1.7379	1.7379	0.5000	0.1250	0.0792
26	Ni	6.9517	5.2138	1.7379	0.5000	0.3750	0.0792
27	Ni	10.4275	1.7379	1.7379	0.7500	0.1250	0.0792
28	Ni	10.4275	5.2138	1.7379	0.7500	0.3750	0.0792
29	Ni	6.9517	8.6896	1.7379	0.5000	0.6250	0.0792
30	Ni	6.9517	12.1654	1.7379	0.5000	0.8750	0.0792
31	Ni	10.4275	8.6896	1.7379	0.7500	0.6250	0.0792
32	Ni	10.4275	12.1654	1.7379	0.7500	0.8750	0.0792
33	Ni	13.8966	13.8977	3.5022	0.9995	0.9996	0.1595
34	Ni	0.0062	3.4780	3.5006	0.0004	0.2502	0.1595
35	Ni	3.4682	13.8979	3.4959	0.2495	0.9996	0.1593
36	Ni	3.4695	3.4474	3.4681	0.2495	0.2480	0.1580
37	Ni	0.0062	6.9535	3.5126	0.0004	0.5001	0.1600
38	Ni	13.8868	10.4255	3.4902	0.9988	0.7499	0.1590
39	Ni	3.4837	6.9503	3.4971	0.2506	0.4999	0.1593

40	Ni	3.4788	10.3913	3.5086	0.2502	0.7474	0.1598
41	Ni	6.9600	0.0118	3.4960	0.5006	0.0008	0.1593
42	Ni	6.9580	3.4671	3.5350	0.5005	0.2494	0.1610
43	Ni	10.4395	0.0031	3.4479	0.7509	0.0002	0.1571
44	Ni	10.4336	3.4680	3.5097	0.7504	0.2494	0.1599
45	Ni	6.9650	6.9613	3.4938	0.5010	0.5007	0.1592
46	Ni	6.9705	10.4446	3.4764	0.5014	0.7512	0.1584
47	Ni	10.4348	6.9393	3.4888	0.7505	0.4991	0.1589
48	Ni	10.4304	10.4436	3.5067	0.7502	0.7512	0.1597
49	Ni	0.0049	1.7427	5.2019	0.0004	0.1253	0.2370
50	Ni	0.0117	5.2130	5.2159	0.0008	0.3749	0.2376
51	Ni	3.4511	1.7294	5.1955	0.2482	0.1244	0.2367
52	Ni	3.4644	5.1650	5.0966	0.2492	0.3715	0.2322
53	Ni	13.8806	8.7074	5.1936	0.9984	0.6263	0.2366
54	Ni	0.0043	12.1535	5.2040	0.0003	0.8741	0.2371
55	Ni	3.4513	8.6617	5.2470	0.2482	0.6230	0.2390
56	Ni	3.4325	12.1216	5.1901	0.2469	0.8719	0.2364
57	Ni	6.9858	1.7395	5.3268	0.5025	0.1251	0.2427
58	Ni	6.9651	5.1412	5.3690	0.5010	0.3698	0.2446
59	Ni	10.4653	1.7746	5.2502	0.7527	0.1276	0.2392
60	Ni	10.4235	5.2133	5.2113	0.7497	0.3750	0.2374
61	Ni	6.9884	8.7226	5.2098	0.5026	0.6274	0.2373
62	Ni	6.9900	12.1564	5.1977	0.5028	0.8744	0.2368
63	Ni	10.4363	8.6748	5.1929	0.7506	0.6239	0.2366
64	Ni	10.4176	12.1683	5.3390	0.7493	0.8752	0.2432
65	Co	1.7379	1.7379	0.0000	0.1250	0.1250	0.0000
66	Co	1.7379	5.2138	0.0000	0.1250	0.3750	0.0000
67	Co	5.2138	1.7379	0.0000	0.3750	0.1250	0.0000
68	Co	5.2138	5.2138	0.0000	0.3750	0.3750	0.0000
69	Co	1.7379	8.6896	0.0000	0.1250	0.6250	0.0000
70	Co	1.7379	12.1654	0.0000	0.1250	0.8750	0.0000
71	Co	5.2138	8.6896	0.0000	0.3750	0.6250	0.0000
72	Co	5.2138	12.1654	0.0000	0.3750	0.8750	0.0000
73	Co	8.6896	1.7379	0.0000	0.6250	0.1250	0.0000
74	Co	8.6896	5.2138	0.0000	0.6250	0.3750	0.0000
75	Co	12.1654	1.7379	0.0000	0.8750	0.1250	0.0000
76	Co	12.1654	5.2138	0.0000	0.8750	0.3750	0.0000
77	Co	8.6896	8.6896	0.0000	0.6250	0.6250	0.0000
78	Co	8.6896	12.1654	0.0000	0.6250	0.8750	0.0000
79	Co	12.1654	8.6896	0.0000	0.8750	0.6250	0.0000
80	Co	12.1654	12.1654	0.0000	0.8750	0.8750	0.0000
81	Co	1.7379	0.0000	1.7379	0.1250	0.0000	0.0792
82	Co	1.7379	3.4758	1.7379	0.1250	0.2500	0.0792
83	Co	5.2138	0.0000	1.7379	0.3750	0.0000	0.0792
84	Co	5.2138	3.4758	1.7379	0.3750	0.2500	0.0792
85	Co	1.7379	6.9517	1.7379	0.1250	0.5000	0.0792
86	Co	1.7379	10.4275	1.7379	0.1250	0.7500	0.0792
87	Co	5.2138	6.9517	1.7379	0.3750	0.5000	0.0792
88	Co	5.2138	10.4275	1.7379	0.3750	0.7500	0.0792

89	Co	8.6896	0.0000	1.7379	0.6250	0.0000	0.0792
90	Co	8.6896	3.4758	1.7379	0.6250	0.2500	0.0792
91	Co	12.1654	0.0000	1.7379	0.8750	0.0000	0.0792
92	Co	12.1654	3.4758	1.7379	0.8750	0.2500	0.0792
93	Co	8.6896	6.9517	1.7379	0.6250	0.5000	0.0792
94	Co	8.6896	10.4275	1.7379	0.6250	0.7500	0.0792
95	Co	12.1654	6.9517	1.7379	0.8750	0.5000	0.0792
96	Co	12.1654	10.4275	1.7379	0.8750	0.7500	0.0792
97	Co	1.7281	1.7339	3.5449	0.1243	0.1247	0.1615
98	Co	1.7148	5.2346	3.5283	0.1233	0.3765	0.1607
99	Co	5.2268	1.7147	3.5674	0.3759	0.1233	0.1625
100	Co	5.2479	5.1827	3.5256	0.3775	0.3728	0.1606
101	Co	1.7304	8.6721	3.5340	0.1245	0.6237	0.1610
102	Co	1.7314	12.1601	3.5261	0.1245	0.8746	0.1606
103	Co	5.2054	8.6772	3.5533	0.3744	0.6241	0.1619
104	Co	5.2084	12.1488	3.5498	0.3746	0.8738	0.1617
105	Co	8.6941	1.7267	3.5564	0.6253	0.1242	0.1620
106	Co	8.6665	5.1991	3.5705	0.6233	0.3739	0.1627
107	Co	12.1756	1.7429	3.5382	0.8757	0.1254	0.1612
108	Co	12.1708	5.2128	3.5576	0.8754	0.3749	0.1621
109	Co	8.6927	8.6786	3.5159	0.6252	0.6242	0.1602
110	Co	8.7089	12.1928	3.4845	0.6264	0.8770	0.1587
111	Co	12.1594	8.6871	3.5429	0.8746	0.6248	0.1614
112	Co	12.1495	12.1688	3.5732	0.8739	0.8752	0.1628
113	Co	1.7343	13.8904	5.2142	0.1247	0.9991	0.2375
114	Co	1.7439	3.4838	5.2184	0.1254	0.2506	0.2377
115	Co	5.1421	13.8799	5.2502	0.3698	0.9983	0.2392
116	Co	5.1652	3.4250	5.2058	0.3715	0.2463	0.2371
117	Co	1.7229	6.9520	5.3357	0.1239	0.5000	0.2431
118	Co	1.7158	10.4454	5.1852	0.1234	0.7513	0.2362
119	Co	5.2704	6.8836	5.1797	0.3791	0.4951	0.2360
120	Co	5.2002	10.4040	5.2077	0.3740	0.7483	0.2372
121	Co	8.7290	0.0471	5.3717	0.6278	0.0034	0.2447
122	Co	8.7151	3.4728	5.2184	0.6268	0.2498	0.2377
123	Co	12.1831	13.8990	5.2692	0.8763	0.9997	0.2400
124	Co	12.1892	3.4956	5.2206	0.8767	0.2514	0.2378
125	Co	8.7191	6.9711	5.1769	0.6271	0.5014	0.2358
126	Co	8.7533	10.3866	5.2107	0.6296	0.7471	0.2374
127	Co	12.1686	6.9465	5.2200	0.8752	0.4996	0.2378
128	Co	12.1731	10.4165	5.2201	0.8756	0.7492	0.2378
129	O	5.9938	6.0907	6.7935	0.4311	0.4381	0.3095
130	O	2.6574	7.7740	6.8359	0.1911	0.5591	0.3114
131	O	4.8823	7.8303	8.7861	0.3512	0.5632	0.4002
132	O	6.0733	9.7818	6.8216	0.4368	0.7036	0.3108
133	O	7.9162	9.7289	9.0186	0.5694	0.6998	0.4108
134	O	5.0334	11.3167	9.0148	0.3620	0.8140	0.4107
135	O	8.6440	11.8258	6.3719	0.6217	0.8506	0.2903
136	Ce	3.9057	9.8307	8.0467	0.2809	0.7071	0.3666
137	Ce	3.9018	6.1102	7.7354	0.2806	0.4395	0.3524

138	Ce	7.1424	7.9973	8.0713	0.5137	0.5752	0.3677
139	Sm	7.0433	11.5515	8.2433	0.5066	0.8308	0.3755
140	C	7.4686	0.5865	6.8294	0.5372	0.0422	0.3111
141	O	6.1662	13.2184	6.6268	0.4435	0.9507	0.3019
142	O	7.7306	0.5947	8.0136	0.5560	0.0428	0.3651
143	O	10.5763	0.1067	6.2335	0.7607	0.0077	0.2840

/db/jmorales/CoNi-alloy/Profiles/Ni-Ce_3SmO_7/O-coverage/M-CO-O-O-coverage/CO-O-Ni-Ce_3SmO_7-3O

a =			13.9019837372				
b =			13.9019837372				
c =			21.9509918687				
alpha =			90.0				
beta =			90.0				
gamma =			90.0				
	Atom	X	Y	Z	X	Y	Z
1	Ni	0.0000	0.0000	0.0000	0.0000	0.0000	0.0000
2	Ni	1.7377	1.7377	0.0000	0.1250	0.1250	0.0000
3	Ni	0.0000	3.4755	0.0000	0.0000	0.2500	0.0000
4	Ni	1.7377	5.2132	0.0000	0.1250	0.3750	0.0000
5	Ni	3.4755	0.0000	0.0000	0.2500	0.0000	0.0000
6	Ni	5.2132	1.7377	0.0000	0.3750	0.1250	0.0000
7	Ni	3.4755	3.4755	0.0000	0.2500	0.2500	0.0000
8	Ni	5.2132	5.2132	0.0000	0.3750	0.3750	0.0000
9	Ni	0.0000	6.9510	0.0000	0.0000	0.5000	0.0000
10	Ni	1.7377	8.6887	0.0000	0.1250	0.6250	0.0000
11	Ni	0.0000	10.4265	0.0000	0.0000	0.7500	0.0000
12	Ni	1.7377	12.1642	0.0000	0.1250	0.8750	0.0000
13	Ni	3.4755	6.9510	0.0000	0.2500	0.5000	0.0000
14	Ni	5.2132	8.6887	0.0000	0.3750	0.6250	0.0000
15	Ni	3.4755	10.4265	0.0000	0.2500	0.7500	0.0000
16	Ni	5.2132	12.1642	0.0000	0.3750	0.8750	0.0000
17	Ni	6.9510	0.0000	0.0000	0.5000	0.0000	0.0000
18	Ni	8.6887	1.7377	0.0000	0.6250	0.1250	0.0000
19	Ni	6.9510	3.4755	0.0000	0.5000	0.2500	0.0000
20	Ni	8.6887	5.2132	0.0000	0.6250	0.3750	0.0000
21	Ni	10.4265	0.0000	0.0000	0.7500	0.0000	0.0000
22	Ni	12.1642	1.7377	0.0000	0.8750	0.1250	0.0000
23	Ni	10.4265	3.4755	0.0000	0.7500	0.2500	0.0000
24	Ni	12.1642	5.2132	0.0000	0.8750	0.3750	0.0000
25	Ni	6.9510	6.9510	0.0000	0.5000	0.5000	0.0000
26	Ni	8.6887	8.6887	0.0000	0.6250	0.6250	0.0000
27	Ni	6.9510	10.4265	0.0000	0.5000	0.7500	0.0000
28	Ni	8.6887	12.1642	0.0000	0.6250	0.8750	0.0000
29	Ni	10.4265	6.9510	0.0000	0.7500	0.5000	0.0000
30	Ni	12.1642	8.6887	0.0000	0.8750	0.6250	0.0000
31	Ni	10.4265	10.4265	0.0000	0.7500	0.7500	0.0000
32	Ni	12.1642	12.1642	0.0000	0.8750	0.8750	0.0000
33	Ni	0.0000	1.7377	1.7377	0.0000	0.1250	0.0792
34	Ni	1.7377	0.0000	1.7377	0.1250	0.0000	0.0792
35	Ni	0.0000	5.2132	1.7377	0.0000	0.3750	0.0792
36	Ni	1.7377	3.4755	1.7377	0.1250	0.2500	0.0792
37	Ni	3.4755	1.7377	1.7377	0.2500	0.1250	0.0792
38	Ni	5.2132	0.0000	1.7377	0.3750	0.0000	0.0792
39	Ni	3.4755	5.2132	1.7377	0.2500	0.3750	0.0792

40	Ni	5.2132	3.4755	1.7377	0.3750	0.2500	0.0792
41	Ni	0.0000	8.6887	1.7377	0.0000	0.6250	0.0792
42	Ni	1.7377	6.9510	1.7377	0.1250	0.5000	0.0792
43	Ni	0.0000	12.1642	1.7377	0.0000	0.8750	0.0792
44	Ni	1.7377	10.4265	1.7377	0.1250	0.7500	0.0792
45	Ni	3.4755	8.6887	1.7377	0.2500	0.6250	0.0792
46	Ni	5.2132	6.9510	1.7377	0.3750	0.5000	0.0792
47	Ni	3.4755	12.1642	1.7377	0.2500	0.8750	0.0792
48	Ni	5.2132	10.4265	1.7377	0.3750	0.7500	0.0792
49	Ni	6.9510	1.7377	1.7377	0.5000	0.1250	0.0792
50	Ni	8.6887	0.0000	1.7377	0.6250	0.0000	0.0792
51	Ni	6.9510	5.2132	1.7377	0.5000	0.3750	0.0792
52	Ni	8.6887	3.4755	1.7377	0.6250	0.2500	0.0792
53	Ni	10.4265	1.7377	1.7377	0.7500	0.1250	0.0792
54	Ni	12.1642	0.0000	1.7377	0.8750	0.0000	0.0792
55	Ni	10.4265	5.2132	1.7377	0.7500	0.3750	0.0792
56	Ni	12.1642	3.4755	1.7377	0.8750	0.2500	0.0792
57	Ni	6.9510	8.6887	1.7377	0.5000	0.6250	0.0792
58	Ni	8.6887	6.9510	1.7377	0.6250	0.5000	0.0792
59	Ni	6.9510	12.1642	1.7377	0.5000	0.8750	0.0792
60	Ni	8.6887	10.4265	1.7377	0.6250	0.7500	0.0792
61	Ni	10.4265	8.6887	1.7377	0.7500	0.6250	0.0792
62	Ni	12.1642	6.9510	1.7377	0.8750	0.5000	0.0792
63	Ni	10.4265	12.1642	1.7377	0.7500	0.8750	0.0792
64	Ni	12.1642	10.4265	1.7377	0.8750	0.7500	0.0792
65	Ni	0.0147	0.0111	3.5075	0.0011	0.0008	0.1598
66	Ni	1.7276	1.7641	3.4719	0.1243	0.1269	0.1582
67	Ni	13.8995	3.4615	3.5093	0.9998	0.2490	0.1599
68	Ni	1.6911	5.1944	3.4391	0.1216	0.3736	0.1567
69	Ni	3.4754	0.0029	3.5067	0.2500	0.0002	0.1597
70	Ni	5.2166	1.7503	3.5495	0.3752	0.1259	0.1617
71	Ni	3.4985	3.4424	3.4732	0.2517	0.2476	0.1582
72	Ni	5.2187	5.2154	3.4737	0.3754	0.3752	0.1582
73	Ni	13.9004	6.9483	3.5020	0.9999	0.4998	0.1595
74	Ni	1.7379	8.6637	3.5445	0.1250	0.6232	0.1615
75	Ni	13.8862	10.4233	3.5220	0.9989	0.7498	0.1604
76	Ni	1.7449	12.1690	3.5120	0.1255	0.8753	0.1600
77	Ni	3.4629	6.9835	3.4645	0.2491	0.5023	0.1578
78	Ni	5.2292	8.7049	3.4950	0.3761	0.6262	0.1592
79	Ni	3.4637	10.3940	3.5215	0.2491	0.7477	0.1604
80	Ni	5.2097	12.1579	3.4945	0.3747	0.8745	0.1592
81	Ni	6.9392	0.0016	3.5023	0.4992	0.0001	0.1596
82	Ni	8.7052	1.7279	3.4463	0.6262	0.1243	0.1570
83	Ni	6.9475	3.4694	3.4953	0.4998	0.2496	0.1592
84	Ni	8.6782	5.2161	3.5416	0.6242	0.3752	0.1613
85	Ni	10.4339	13.8901	3.5269	0.7505	0.9991	0.1607
86	Ni	12.1621	1.7450	3.4605	0.8748	0.1255	0.1576
87	Ni	10.4204	3.4698	3.5029	0.7496	0.2496	0.1596
88	Ni	12.1651	5.1957	3.4810	0.8751	0.3737	0.1586

89	Ni	6.9449	6.9388	3.5103	0.4996	0.4991	0.1599
90	Ni	8.6936	8.6857	3.4724	0.6254	0.6248	0.1582
91	Ni	6.9831	10.4254	3.5110	0.5023	0.7499	0.1599
92	Ni	8.6918	12.1938	3.5051	0.6252	0.8771	0.1597
93	Ni	10.4055	6.9546	3.5147	0.7485	0.5003	0.1601
94	Ni	12.1784	8.6773	3.4900	0.8760	0.6242	0.1590
95	Ni	10.4416	10.4446	3.4312	0.7511	0.7513	0.1563
96	Ni	12.1731	12.1804	3.5128	0.8756	0.8762	0.1600
97	Ni	0.0492	1.7649	5.3996	0.0035	0.1270	0.2460
98	Ni	1.7558	13.8871	5.2872	0.1263	0.9989	0.2409
99	Ni	13.8090	5.1998	5.2797	0.9933	0.3740	0.2405
100	Ni	1.7460	3.5427	5.3490	0.1256	0.2548	0.2437
101	Ni	3.4857	1.7520	5.2839	0.2507	0.1260	0.2407
102	Ni	5.2008	13.8831	5.2109	0.3741	0.9986	0.2374
103	Ni	3.4751	5.1931	5.0399	0.2500	0.3736	0.2296
104	Ni	5.2155	3.4617	5.3431	0.3752	0.2490	0.2434
105	Ni	0.0028	8.6733	5.2157	0.0002	0.6239	0.2376
106	Ni	1.6868	6.8760	5.2865	0.1213	0.4946	0.2408
107	Ni	0.0046	12.1679	5.2039	0.0003	0.8753	0.2371
108	Ni	1.7198	10.4394	5.2183	0.1237	0.7509	0.2377
109	Ni	3.4416	8.7369	5.3569	0.2476	0.6285	0.2440
110	Ni	5.1885	6.9731	5.1979	0.3732	0.5016	0.2368
111	Ni	3.4853	12.1374	5.2147	0.2507	0.8731	0.2376
112	Ni	5.2325	10.4382	5.1855	0.3764	0.7508	0.2362
113	Ni	6.9365	1.7061	5.2664	0.4990	0.1227	0.2399
114	Ni	8.6832	13.9008	5.2740	0.6246	0.9999	0.2403
115	Ni	6.9356	5.1972	5.2596	0.4989	0.3738	0.2396
116	Ni	8.7032	3.4589	5.2515	0.6260	0.2488	0.2392
117	Ni	10.4145	1.7245	5.4122	0.7491	0.1240	0.2466
118	Ni	12.1885	0.0239	5.2613	0.8767	0.0017	0.2397
119	Ni	10.4518	5.2065	5.2641	0.7518	0.3745	0.2398
120	Ni	12.1449	3.4683	5.3283	0.8736	0.2495	0.2427
121	Ni	6.9800	8.7363	5.2341	0.5021	0.6284	0.2384
122	Ni	8.7141	6.9387	5.2863	0.6268	0.4991	0.2408
123	Ni	6.9439	12.1586	5.2117	0.4995	0.8746	0.2374
124	Ni	8.7242	10.4693	5.2116	0.6275	0.7531	0.2374
125	Ni	10.4563	8.6982	5.1740	0.7521	0.6257	0.2357
126	Ni	12.1456	6.9355	5.2527	0.8737	0.4989	0.2393
127	Ni	10.4520	12.1704	5.2654	0.7518	0.8754	0.2399
128	Ni	12.1778	10.4251	5.2786	0.8760	0.7499	0.2405
129	O	6.0315	4.4534	6.8070	0.4339	0.3203	0.3101
130	O	4.1740	7.6290	6.7347	0.3002	0.5488	0.3068
131	O	5.9037	6.6580	8.8031	0.4247	0.4789	0.4010
132	O	7.7810	7.8445	6.7974	0.5597	0.5643	0.3097
133	O	10.4261	10.3579	6.2681	0.7500	0.7451	0.2855
134	O	9.4132	7.0436	8.9474	0.6771	0.5067	0.4076
135	O	7.6281	9.8474	8.8406	0.5487	0.7083	0.4027
136	Ce	5.8909	8.8788	8.0140	0.4237	0.6387	0.3651
137	Ce	4.2225	5.5408	7.7158	0.3037	0.3986	0.3515

138	Ce	7.9520	5.7670	8.0908	0.5720	0.4148	0.3686
139	Sm	9.3886	8.9922	8.0412	0.6753	0.6468	0.3663
140	C	12.1215	5.2608	6.3718	0.8719	0.3784	0.2903
141	O	12.1417	5.2312	7.5899	0.8734	0.3763	0.3458
142	O	12.1813	1.7134	6.1751	0.8762	0.1233	0.2813
143	O	2.0848	5.2821	6.3641	0.1500	0.3800	0.2899
144	O	1.8211	1.6736	6.2024	0.1310	0.1204	0.2826
145	O	8.6104	1.7505	6.1799	0.6194	0.1259	0.2815

/db/jmorales/CoNi-alloy/Profiles/CoNi(111)/O

a =	9.831199646
b =	9.831199645902503
c =	21.020299912
alpha =	90.0
beta =	90.0
gamma =	120.0

	Atom	X	Y	Z	X	Y	Z
1	Ni	0.0000	0.0000	0.0000	0.0000	0.0000	0.0000
2	Ni	-1.2289	2.1285	0.0000	0.0000	0.2500	0.0000
3	Ni	-2.4578	4.2570	0.0000	-0.0000	0.5000	0.0000
4	Ni	-3.6867	6.3856	0.0000	-0.0000	0.7500	0.0000
5	Ni	4.9156	0.0000	0.0000	0.5000	0.0000	0.0000
6	Ni	3.6867	2.1285	0.0000	0.5000	0.2500	0.0000
7	Ni	2.4578	4.2570	0.0000	0.5000	0.5000	0.0000
8	Ni	1.2289	6.3856	0.0000	0.5000	0.7500	0.0000
9	Ni	2.4578	1.4190	2.0068	0.3333	0.1667	0.0955
10	Ni	1.2289	3.5475	2.0068	0.3333	0.4167	0.0955
11	Ni	0.0000	5.6760	2.0068	0.3333	0.6667	0.0955
12	Ni	-1.2289	7.8046	2.0068	0.3333	0.9167	0.0955
13	Ni	7.3734	1.4190	2.0068	0.8333	0.1667	0.0955
14	Ni	6.1445	3.5475	2.0068	0.8333	0.4167	0.0955
15	Ni	4.9156	5.6760	2.0068	0.8333	0.6667	0.0955
16	Ni	3.6867	7.8046	2.0068	0.8333	0.9167	0.0955
17	Ni	0.0516	2.8674	4.0014	0.1736	0.3368	0.1904
18	Ni	1.2831	0.7410	4.0203	0.1740	0.0870	0.1913
19	Ni	-2.4088	7.1166	4.0096	0.1729	0.8359	0.1908
20	Ni	-1.1791	4.9959	3.9988	0.1735	0.5868	0.1902
21	Ni	4.9524	2.8611	4.0288	0.6718	0.3360	0.1917
22	Ni	6.1932	0.7414	4.0080	0.6735	0.0871	0.1907
23	Ni	2.5048	7.1240	4.0140	0.6731	0.8367	0.1910
24	Ni	3.7368	4.9937	4.0079	0.6734	0.5865	0.1907
25	Ni	0.0513	0.0260	6.0063	0.0067	0.0031	0.2857
26	Ni	-1.1492	2.1767	6.0094	0.0109	0.2557	0.2859
27	Ni	-2.3710	4.3014	6.0146	0.0114	0.5052	0.2861
28	Ni	-3.6016	6.4278	6.0087	0.0111	0.7550	0.2859
29	Ni	5.0398	0.0277	6.0054	0.5143	0.0033	0.2857
30	Ni	3.8220	2.1895	6.0961	0.5173	0.2572	0.2900
31	Ni	2.5530	4.3573	6.0629	0.5156	0.5118	0.2884
32	Ni	1.3191	6.4470	6.0232	0.5128	0.7572	0.2865
33	Co	1.2289	2.1285	0.0000	0.2500	0.2500	0.0000
34	Co	2.4578	0.0000	0.0000	0.2500	0.0000	0.0000
35	Co	-1.2289	6.3856	0.0000	0.2500	0.7500	0.0000
36	Co	0.0000	4.2570	0.0000	0.2500	0.5000	0.0000
37	Co	6.1445	2.1285	0.0000	0.7500	0.2500	0.0000
38	Co	7.3734	0.0000	0.0000	0.7500	0.0000	0.0000
39	Co	3.6867	6.3856	0.0000	0.7500	0.7500	0.0000
40	Co	4.9156	4.2570	0.0000	0.7500	0.5000	0.0000
41	Co	-1.2289	3.5475	2.0068	0.0833	0.4167	0.0955

42	Co	0.0000	1.4190	2.0068	0.0833	0.1667	0.0955
43	Co	-3.6867	7.8046	2.0068	0.0833	0.9167	0.0955
44	Co	-2.4578	5.6760	2.0068	0.0833	0.6667	0.0955
45	Co	3.6867	3.5475	2.0068	0.5833	0.4167	0.0955
46	Co	4.9156	1.4190	2.0068	0.5833	0.1667	0.0955
47	Co	1.2289	7.8046	2.0068	0.5833	0.9167	0.0955
48	Co	2.4578	5.6760	2.0068	0.5833	0.6667	0.0955
49	Co	3.7325	0.7316	4.0507	0.4226	0.0859	0.1927
50	Co	2.4984	2.8670	4.0539	0.4225	0.3367	0.1929
51	Co	1.2712	4.9942	4.0324	0.4226	0.5866	0.1918
52	Co	0.0402	7.1167	4.0470	0.4220	0.8359	0.1925
53	Co	8.6534	0.7430	4.0428	0.9238	0.0873	0.1923
54	Co	7.4204	2.8719	4.0438	0.9234	0.3373	0.1924
55	Co	6.1883	4.9909	4.0439	0.9226	0.5862	0.1924
56	Co	4.9690	7.1228	4.0387	0.9237	0.8366	0.1921
57	Co	1.3114	2.1678	6.0949	0.2607	0.2546	0.2900
58	Co	-2.3466	8.5118	6.0838	0.2612	0.9997	0.2894
59	Co	-1.1348	6.4417	6.0060	0.2629	0.7566	0.2857
60	Co	0.0954	4.3098	6.0544	0.2628	0.5062	0.2880
61	Co	6.2593	2.1856	6.0014	0.7650	0.2567	0.2855
62	Co	7.4770	0.0638	6.0129	0.7643	0.0075	0.2861
63	Co	3.7941	6.4510	6.0052	0.7648	0.7577	0.2857
64	Co	5.0220	4.3188	5.9997	0.7644	0.5073	0.2854
65	O	2.7667	1.5060	7.3476	0.3699	0.1769	0.3495

/db/jmorales/CoNi-alloy/Profiles/CoNi-Ce_3SmO_7/O-coverage/CO-O-O-coverage/CO-O-CoNi-Ce_3SmO_7-4O

a =				13.9033384264			
b =				13.9033384264			
c =				21.9516692133			
alpha =				90.0			
beta =				90.0			
gamma =				90.0			
	Atom	X	Y	Z	X	Y	Z
1	Ni	0.0000	0.0000	0.0000	0.0000	0.0000	0.0000
2	Ni	0.0000	3.4758	0.0000	0.0000	0.2500	0.0000
3	Ni	3.4758	0.0000	0.0000	0.2500	0.0000	0.0000
4	Ni	3.4758	3.4758	0.0000	0.2500	0.2500	0.0000
5	Ni	0.0000	6.9517	0.0000	0.0000	0.5000	0.0000
6	Ni	0.0000	10.4275	0.0000	0.0000	0.7500	0.0000
7	Ni	3.4758	6.9517	0.0000	0.2500	0.5000	0.0000
8	Ni	3.4758	10.4275	0.0000	0.2500	0.7500	0.0000
9	Ni	6.9517	0.0000	0.0000	0.5000	0.0000	0.0000
10	Ni	6.9517	3.4758	0.0000	0.5000	0.2500	0.0000
11	Ni	10.4275	0.0000	0.0000	0.7500	0.0000	0.0000
12	Ni	10.4275	3.4758	0.0000	0.7500	0.2500	0.0000
13	Ni	6.9517	6.9517	0.0000	0.5000	0.5000	0.0000
14	Ni	6.9517	10.4275	0.0000	0.5000	0.7500	0.0000
15	Ni	10.4275	6.9517	0.0000	0.7500	0.5000	0.0000
16	Ni	10.4275	10.4275	0.0000	0.7500	0.7500	0.0000
17	Ni	0.0000	1.7379	1.7379	0.0000	0.1250	0.0792
18	Ni	0.0000	5.2138	1.7379	0.0000	0.3750	0.0792
19	Ni	3.4758	1.7379	1.7379	0.2500	0.1250	0.0792
20	Ni	3.4758	5.2138	1.7379	0.2500	0.3750	0.0792
21	Ni	0.0000	8.6896	1.7379	0.0000	0.6250	0.0792
22	Ni	0.0000	12.1654	1.7379	0.0000	0.8750	0.0792
23	Ni	3.4758	8.6896	1.7379	0.2500	0.6250	0.0792
24	Ni	3.4758	12.1654	1.7379	0.2500	0.8750	0.0792
25	Ni	6.9517	1.7379	1.7379	0.5000	0.1250	0.0792
26	Ni	6.9517	5.2138	1.7379	0.5000	0.3750	0.0792
27	Ni	10.4275	1.7379	1.7379	0.7500	0.1250	0.0792
28	Ni	10.4275	5.2138	1.7379	0.7500	0.3750	0.0792
29	Ni	6.9517	8.6896	1.7379	0.5000	0.6250	0.0792
30	Ni	6.9517	12.1654	1.7379	0.5000	0.8750	0.0792
31	Ni	10.4275	8.6896	1.7379	0.7500	0.6250	0.0792
32	Ni	10.4275	12.1654	1.7379	0.7500	0.8750	0.0792
33	Ni	0.0009	13.9025	3.4519	0.0001	0.9999	0.1573
34	Ni	0.0283	3.4516	3.5322	0.0020	0.2483	0.1609
35	Ni	3.4747	13.8927	3.4455	0.2499	0.9992	0.1570
36	Ni	3.4813	3.4337	3.4450	0.2504	0.2470	0.1569
37	Ni	0.0037	6.9543	3.5078	0.0003	0.5002	0.1598
38	Ni	13.8746	10.4593	3.5071	0.9979	0.7523	0.1598
39	Ni	3.4636	6.9662	3.4796	0.2491	0.5010	0.1585

40	Ni	3.4979	10.4224	3.5370	0.2516	0.7496	0.1611
41	Ni	6.9518	0.0162	3.4768	0.5000	0.0012	0.1584
42	Ni	6.9333	3.4808	3.4558	0.4987	0.2504	0.1574
43	Ni	10.4319	0.0197	3.4492	0.7503	0.0014	0.1571
44	Ni	10.4156	3.4739	3.5139	0.7491	0.2499	0.1601
45	Ni	6.9574	6.9560	3.5067	0.5004	0.5003	0.1597
46	Ni	6.9738	10.4349	3.4881	0.5016	0.7505	0.1589
47	Ni	10.4400	6.9294	3.4851	0.7509	0.4984	0.1588
48	Ni	10.4270	10.4414	3.5032	0.7500	0.7510	0.1596
49	Ni	0.0229	1.7536	5.2697	0.0016	0.1261	0.2401
50	Ni	0.0238	5.2056	5.2119	0.0017	0.3744	0.2374
51	Ni	3.5077	1.6798	5.2404	0.2523	0.1208	0.2387
52	Ni	3.4622	5.2241	5.0516	0.2490	0.3757	0.2301
53	Ni	13.8631	8.7249	5.1863	0.9971	0.6275	0.2363
54	Ni	13.8904	12.1544	5.2612	0.9991	0.8742	0.2397
55	Ni	3.4323	8.6857	5.2336	0.2469	0.6247	0.2384
56	Ni	3.4925	12.1153	5.2672	0.2512	0.8714	0.2399
57	Ni	6.8937	1.7147	5.3048	0.4958	0.1233	0.2417
58	Ni	6.9779	5.2571	5.3681	0.5019	0.3781	0.2445
59	Ni	10.4382	1.8106	5.2543	0.7508	0.1302	0.2394
60	Ni	10.4127	5.2026	5.2081	0.7489	0.3742	0.2373
61	Ni	6.9857	8.6931	5.2279	0.5024	0.6253	0.2382
62	Ni	7.0435	12.2309	5.2540	0.5066	0.8797	0.2393
63	Ni	10.4568	8.6634	5.1825	0.7521	0.6231	0.2361
64	Ni	10.3507	12.1679	5.3551	0.7445	0.8752	0.2439
65	Co	1.7379	1.7379	0.0000	0.1250	0.1250	0.0000
66	Co	1.7379	5.2138	0.0000	0.1250	0.3750	0.0000
67	Co	5.2138	1.7379	0.0000	0.3750	0.1250	0.0000
68	Co	5.2138	5.2138	0.0000	0.3750	0.3750	0.0000
69	Co	1.7379	8.6896	0.0000	0.1250	0.6250	0.0000
70	Co	1.7379	12.1654	0.0000	0.1250	0.8750	0.0000
71	Co	5.2138	8.6896	0.0000	0.3750	0.6250	0.0000
72	Co	5.2138	12.1654	0.0000	0.3750	0.8750	0.0000
73	Co	8.6896	1.7379	0.0000	0.6250	0.1250	0.0000
74	Co	8.6896	5.2138	0.0000	0.6250	0.3750	0.0000
75	Co	12.1654	1.7379	0.0000	0.8750	0.1250	0.0000
76	Co	12.1654	5.2138	0.0000	0.8750	0.3750	0.0000
77	Co	8.6896	8.6896	0.0000	0.6250	0.6250	0.0000
78	Co	8.6896	12.1654	0.0000	0.6250	0.8750	0.0000
79	Co	12.1654	8.6896	0.0000	0.8750	0.6250	0.0000
80	Co	12.1654	12.1654	0.0000	0.8750	0.8750	0.0000
81	Co	1.7379	0.0000	1.7379	0.1250	0.0000	0.0792
82	Co	1.7379	3.4758	1.7379	0.1250	0.2500	0.0792
83	Co	5.2138	0.0000	1.7379	0.3750	0.0000	0.0792
84	Co	5.2138	3.4758	1.7379	0.3750	0.2500	0.0792
85	Co	1.7379	6.9517	1.7379	0.1250	0.5000	0.0792
86	Co	1.7379	10.4275	1.7379	0.1250	0.7500	0.0792
87	Co	5.2138	6.9517	1.7379	0.3750	0.5000	0.0792
88	Co	5.2138	10.4275	1.7379	0.3750	0.7500	0.0792

89	Co	8.6896	0.0000	1.7379	0.6250	0.0000	0.0792
90	Co	8.6896	3.4758	1.7379	0.6250	0.2500	0.0792
91	Co	12.1654	0.0000	1.7379	0.8750	0.0000	0.0792
92	Co	12.1654	3.4758	1.7379	0.8750	0.2500	0.0792
93	Co	8.6896	6.9517	1.7379	0.6250	0.5000	0.0792
94	Co	8.6896	10.4275	1.7379	0.6250	0.7500	0.0792
95	Co	12.1654	6.9517	1.7379	0.8750	0.5000	0.0792
96	Co	12.1654	10.4275	1.7379	0.8750	0.7500	0.0792
97	Co	1.7450	1.7345	3.5656	0.1255	0.1248	0.1624
98	Co	1.7075	5.2247	3.5134	0.1228	0.3758	0.1601
99	Co	5.2115	1.7405	3.5306	0.3748	0.1252	0.1608
100	Co	5.2389	5.1920	3.4945	0.3768	0.3734	0.1592
101	Co	1.7186	8.7022	3.5223	0.1236	0.6259	0.1605
102	Co	1.7407	12.1685	3.5441	0.1252	0.8752	0.1614
103	Co	5.2014	8.6956	3.5535	0.3741	0.6254	0.1619
104	Co	5.2406	12.1432	3.5658	0.3769	0.8734	0.1624
105	Co	8.6782	1.7532	3.5486	0.6242	0.1261	0.1617
106	Co	8.6695	5.1987	3.5618	0.6236	0.3739	0.1623
107	Co	12.1762	1.7437	3.5552	0.8758	0.1254	0.1620
108	Co	12.1752	5.1999	3.5541	0.8757	0.3740	0.1619
109	Co	8.6949	8.6636	3.5126	0.6254	0.6231	0.1600
110	Co	8.7135	12.1926	3.4843	0.6267	0.8770	0.1587
111	Co	12.1615	8.6878	3.5325	0.8747	0.6249	0.1609
112	Co	12.1281	12.1901	3.5869	0.8723	0.8768	0.1634
113	Co	1.7621	13.8720	5.3620	0.1267	0.9977	0.2443
114	Co	1.8210	3.4885	5.2878	0.1310	0.2509	0.2409
115	Co	5.2450	13.8521	5.3252	0.3772	0.9963	0.2426
116	Co	5.1667	3.4734	5.3129	0.3716	0.2498	0.2420
117	Co	1.6972	6.9788	5.3027	0.1221	0.5020	0.2416
118	Co	1.7110	10.4533	5.1727	0.1231	0.7519	0.2356
119	Co	5.2285	6.9175	5.1496	0.3761	0.4975	0.2346
120	Co	5.2409	10.4158	5.2469	0.3769	0.7492	0.2390
121	Co	8.6783	0.1031	5.3280	0.6242	0.0074	0.2427
122	Co	8.6310	3.4656	5.2643	0.6208	0.2493	0.2398
123	Co	12.1390	0.0044	5.3569	0.8731	0.0003	0.2440
124	Co	12.1844	3.5011	5.2038	0.8764	0.2518	0.2371
125	Co	8.7369	6.9738	5.1847	0.6284	0.5016	0.2362
126	Co	8.7510	10.3433	5.1889	0.6294	0.7439	0.2364
127	Co	12.1767	6.9257	5.2155	0.8758	0.4981	0.2376
128	Co	12.1508	10.4297	5.2098	0.8739	0.7502	0.2373
129	O	5.8368	6.1423	6.7734	0.4198	0.4418	0.3086
130	O	2.6380	7.7270	6.8052	0.1897	0.5558	0.3100
131	O	4.7859	7.7278	8.8580	0.3442	0.5558	0.4035
132	O	6.0513	9.6231	6.8378	0.4352	0.6921	0.3115
133	O	7.8029	9.5805	9.1159	0.5612	0.6891	0.4153
134	O	4.9458	11.2825	8.9047	0.3557	0.8115	0.4057
135	O	8.5717	11.6602	6.4246	0.6165	0.8387	0.2927
136	Ce	3.8390	9.7099	8.0536	0.2761	0.6984	0.3669
137	Ce	3.7774	5.9842	7.7577	0.2717	0.4304	0.3534

138 Ce	7.0329	7.8379	8.1589	0.5058	0.5637	0.3717
139 Sm	6.9954	11.3554	8.2174	0.5031	0.8167	0.3743
140 C	6.8639	0.0582	6.3766	0.4937	0.0042	0.2905
141 O	6.9975	13.8745	7.6160	0.5033	0.9979	0.3469
142 O	3.5314	13.8444	6.1861	0.2540	0.9958	0.2818
143 O	10.4068	0.1235	6.1976	0.7485	0.0089	0.2823
144 O	0.0036	0.0007	6.1848	0.0003	0.0000	0.2817
145 O	3.4508	3.8452	6.4084	0.2482	0.2766	0.2919
146 O	6.9699	3.4223	6.2287	0.5013	0.2461	0.2837

/db/jmorales/CoNi-alloy/Profiles/CoNi-Ce_3SmO_7/O-coverage/TS-O-coverage/TS-CoNi-Ce_3SmO_7-6O

a =			13.9033384264				
b =			13.9033384264				
c =			21.9516692133				
alpha =			90.0				
beta =			90.0				
gamma =			90.0				
	Atom	X	Y	Z	X	Y	Z
1	Ni	0.0000	0.0000	0.0000	0.0000	0.0000	0.0000
2	Ni	0.0000	3.4758	0.0000	0.0000	0.2500	0.0000
3	Ni	3.4758	0.0000	0.0000	0.2500	0.0000	0.0000
4	Ni	3.4758	3.4758	0.0000	0.2500	0.2500	0.0000
5	Ni	0.0000	6.9517	0.0000	0.0000	0.5000	0.0000
6	Ni	0.0000	10.4275	0.0000	0.0000	0.7500	0.0000
7	Ni	3.4758	6.9517	0.0000	0.2500	0.5000	0.0000
8	Ni	3.4758	10.4275	0.0000	0.2500	0.7500	0.0000
9	Ni	6.9517	0.0000	0.0000	0.5000	0.0000	0.0000
10	Ni	6.9517	3.4758	0.0000	0.5000	0.2500	0.0000
11	Ni	10.4275	0.0000	0.0000	0.7500	0.0000	0.0000
12	Ni	10.4275	3.4758	0.0000	0.7500	0.2500	0.0000
13	Ni	6.9517	6.9517	0.0000	0.5000	0.5000	0.0000
14	Ni	6.9517	10.4275	0.0000	0.5000	0.7500	0.0000
15	Ni	10.4275	6.9517	0.0000	0.7500	0.5000	0.0000
16	Ni	10.4275	10.4275	0.0000	0.7500	0.7500	0.0000
17	Ni	0.0000	1.7379	1.7379	0.0000	0.1250	0.0792
18	Ni	0.0000	5.2138	1.7379	0.0000	0.3750	0.0792
19	Ni	3.4758	1.7379	1.7379	0.2500	0.1250	0.0792
20	Ni	3.4758	5.2138	1.7379	0.2500	0.3750	0.0792
21	Ni	0.0000	8.6896	1.7379	0.0000	0.6250	0.0792
22	Ni	0.0000	12.1654	1.7379	0.0000	0.8750	0.0792
23	Ni	3.4758	8.6896	1.7379	0.2500	0.6250	0.0792
24	Ni	3.4758	12.1654	1.7379	0.2500	0.8750	0.0792
25	Ni	6.9517	1.7379	1.7379	0.5000	0.1250	0.0792
26	Ni	6.9517	5.2138	1.7379	0.5000	0.3750	0.0792
27	Ni	10.4275	1.7379	1.7379	0.7500	0.1250	0.0792
28	Ni	10.4275	5.2138	1.7379	0.7500	0.3750	0.0792
29	Ni	6.9517	8.6896	1.7379	0.5000	0.6250	0.0792
30	Ni	6.9517	12.1654	1.7379	0.5000	0.8750	0.0792
31	Ni	10.4275	8.6896	1.7379	0.7500	0.6250	0.0792
32	Ni	10.4275	12.1654	1.7379	0.7500	0.8750	0.0792
33	Ni	13.8965	13.8939	3.4563	0.9995	0.9993	0.1574
34	Ni	-0.0015	3.4666	3.5216	-0.0001	0.2493	0.1604
35	Ni	3.4603	0.0249	3.5185	0.2489	0.0018	0.1603
36	Ni	3.4821	3.4428	3.4242	0.2505	0.2476	0.1560
37	Ni	0.0063	6.9638	3.5165	0.0005	0.5009	0.1602
38	Ni	13.8893	10.4506	3.4474	0.9990	0.7517	0.1570
39	Ni	3.4674	6.9455	3.4838	0.2494	0.4996	0.1587

40	Ni	3.4614	10.3961	3.5133	0.2490	0.7477	0.1600
41	Ni	6.9549	0.0177	3.5097	0.5002	0.0013	0.1599
42	Ni	6.9666	3.4714	3.4752	0.5011	0.2497	0.1583
43	Ni	10.4462	0.0197	3.4669	0.7513	0.0014	0.1579
44	Ni	10.4178	3.4758	3.4534	0.7493	0.2500	0.1573
45	Ni	6.9574	6.9708	3.5010	0.5004	0.5014	0.1595
46	Ni	6.9576	10.4447	3.4818	0.5004	0.7512	0.1586
47	Ni	10.4362	6.9345	3.4984	0.7506	0.4988	0.1594
48	Ni	10.4492	10.4456	3.5179	0.7516	0.7513	0.1603
49	Ni	0.0036	1.7873	5.2653	0.0003	0.1286	0.2399
50	Ni	0.0043	5.2174	5.2059	0.0003	0.3753	0.2372
51	Ni	3.4747	1.7432	5.2379	0.2499	0.1254	0.2386
52	Ni	3.4768	5.1892	5.0593	0.2501	0.3732	0.2305
53	Ni	13.8833	8.6672	5.2304	0.9986	0.6234	0.2383
54	Ni	0.0054	12.1778	5.2702	0.0004	0.8759	0.2401
55	Ni	3.4218	8.6565	5.2338	0.2461	0.6226	0.2384
56	Ni	3.4257	12.1317	5.1848	0.2464	0.8726	0.2362
57	Ni	6.9829	1.7311	5.3512	0.5022	0.1245	0.2438
58	Ni	6.9810	5.2645	5.3501	0.5021	0.3786	0.2437
59	Ni	10.4420	1.7711	5.2798	0.7510	0.1274	0.2405
60	Ni	10.4141	5.2728	5.2548	0.7490	0.3792	0.2394
61	Ni	6.9694	8.7234	5.2223	0.5013	0.6274	0.2379
62	Ni	6.9937	12.1444	5.2120	0.5030	0.8735	0.2374
63	Ni	10.4510	8.6758	5.1888	0.7517	0.6240	0.2364
64	Ni	10.4030	12.1530	5.3349	0.7482	0.8741	0.2430
65	Co	1.7379	1.7379	0.0000	0.1250	0.1250	0.0000
66	Co	1.7379	5.2138	0.0000	0.1250	0.3750	0.0000
67	Co	5.2138	1.7379	0.0000	0.3750	0.1250	0.0000
68	Co	5.2138	5.2138	0.0000	0.3750	0.3750	0.0000
69	Co	1.7379	8.6896	0.0000	0.1250	0.6250	0.0000
70	Co	1.7379	12.1654	0.0000	0.1250	0.8750	0.0000
71	Co	5.2138	8.6896	0.0000	0.3750	0.6250	0.0000
72	Co	5.2138	12.1654	0.0000	0.3750	0.8750	0.0000
73	Co	8.6896	1.7379	0.0000	0.6250	0.1250	0.0000
74	Co	8.6896	5.2138	0.0000	0.6250	0.3750	0.0000
75	Co	12.1654	1.7379	0.0000	0.8750	0.1250	0.0000
76	Co	12.1654	5.2138	0.0000	0.8750	0.3750	0.0000
77	Co	8.6896	8.6896	0.0000	0.6250	0.6250	0.0000
78	Co	8.6896	12.1654	0.0000	0.6250	0.8750	0.0000
79	Co	12.1654	8.6896	0.0000	0.8750	0.6250	0.0000
80	Co	12.1654	12.1654	0.0000	0.8750	0.8750	0.0000
81	Co	1.7379	0.0000	1.7379	0.1250	0.0000	0.0792
82	Co	1.7379	3.4758	1.7379	0.1250	0.2500	0.0792
83	Co	5.2138	0.0000	1.7379	0.3750	0.0000	0.0792
84	Co	5.2138	3.4758	1.7379	0.3750	0.2500	0.0792
85	Co	1.7379	6.9517	1.7379	0.1250	0.5000	0.0792
86	Co	1.7379	10.4275	1.7379	0.1250	0.7500	0.0792
87	Co	5.2138	6.9517	1.7379	0.3750	0.5000	0.0792
88	Co	5.2138	10.4275	1.7379	0.3750	0.7500	0.0792

89	Co	8.6896	0.0000	1.7379	0.6250	0.0000	0.0792
90	Co	8.6896	3.4758	1.7379	0.6250	0.2500	0.0792
91	Co	12.1654	0.0000	1.7379	0.8750	0.0000	0.0792
92	Co	12.1654	3.4758	1.7379	0.8750	0.2500	0.0792
93	Co	8.6896	6.9517	1.7379	0.6250	0.5000	0.0792
94	Co	8.6896	10.4275	1.7379	0.6250	0.7500	0.0792
95	Co	12.1654	6.9517	1.7379	0.8750	0.5000	0.0792
96	Co	12.1654	10.4275	1.7379	0.8750	0.7500	0.0792
97	Co	1.7314	1.7556	3.5566	0.1245	0.1263	0.1620
98	Co	1.7052	5.2199	3.5127	0.1226	0.3754	0.1600
99	Co	5.2227	1.7338	3.5663	0.3756	0.1247	0.1625
100	Co	5.2517	5.1851	3.4938	0.3777	0.3729	0.1592
101	Co	1.7230	8.6808	3.5197	0.1239	0.6244	0.1603
102	Co	1.7379	12.1736	3.5244	0.1250	0.8756	0.1606
103	Co	5.1890	8.6915	3.5442	0.3732	0.6251	0.1615
104	Co	5.2015	12.1461	3.5513	0.3741	0.8736	0.1618
105	Co	8.6909	1.7405	3.5556	0.6251	0.1252	0.1620
106	Co	8.6759	5.2115	3.5521	0.6240	0.3748	0.1618
107	Co	12.1756	1.7500	3.5506	0.8757	0.1259	0.1617
108	Co	12.1671	5.2116	3.5428	0.8751	0.3748	0.1614
109	Co	8.6934	8.6844	3.5137	0.6253	0.6246	0.1601
110	Co	8.7107	12.1902	3.4881	0.6265	0.8768	0.1589
111	Co	12.1666	8.6927	3.5382	0.8751	0.6252	0.1612
112	Co	12.1451	12.1765	3.5764	0.8735	0.8758	0.1629
113	Co	1.7381	0.0164	5.2611	0.1250	0.0012	0.2397
114	Co	1.7721	3.5090	5.2712	0.1275	0.2524	0.2401
115	Co	5.1433	13.8658	5.2457	0.3699	0.9973	0.2390
116	Co	5.2010	3.4589	5.2994	0.3741	0.2488	0.2414
117	Co	1.7143	6.9282	5.3033	0.1233	0.4983	0.2416
118	Co	1.6959	10.4443	5.2291	0.1220	0.7512	0.2382
119	Co	5.2328	6.9186	5.1436	0.3764	0.4976	0.2343
120	Co	5.1670	10.3940	5.2215	0.3716	0.7476	0.2379
121	Co	8.7112	0.0455	5.3360	0.6266	0.0033	0.2431
122	Co	8.7039	3.5202	5.3590	0.6260	0.2532	0.2441
123	Co	12.1912	0.0098	5.3626	0.8769	0.0007	0.2443
124	Co	12.1583	3.5170	5.2663	0.8745	0.2530	0.2399
125	Co	8.7149	7.0081	5.1767	0.6268	0.5041	0.2358
126	Co	8.7519	10.3665	5.1966	0.6295	0.7456	0.2367
127	Co	12.1771	6.9422	5.2058	0.8758	0.4993	0.2371
128	Co	12.2041	10.4148	5.2722	0.8778	0.7491	0.2402
129	O	5.8651	6.1813	6.7705	0.4218	0.4446	0.3084
130	O	2.6451	7.7022	6.8072	0.1902	0.5540	0.3101
131	O	4.7880	7.7390	8.8490	0.3444	0.5566	0.4031
132	O	6.0031	9.7013	6.8292	0.4318	0.6978	0.3111
133	O	7.8115	9.6731	9.0540	0.5618	0.6957	0.4125
134	O	4.9364	11.2586	8.9876	0.3551	0.8098	0.4094
135	O	8.5935	11.7229	6.4058	0.6181	0.8432	0.2918
136	Ce	3.8247	9.7179	8.0853	0.2751	0.6990	0.3683
137	Ce	3.8203	5.9839	7.7475	0.2748	0.4304	0.3529

138	Ce	7.0419	7.9240	8.1362	0.5065	0.5699	0.3706
139	Sm	6.9547	11.4802	8.2378	0.5002	0.8257	0.3753
140	C	7.4686	0.5865	6.8294	0.5372	0.0422	0.3111
141	O	6.1662	13.2184	6.6268	0.4435	0.9507	0.3019
142	O	7.7306	0.5947	8.0136	0.5560	0.0428	0.3651
143	O	10.5028	0.0329	6.2127	0.7554	0.0024	0.2830
144	O	0.0662	0.0774	6.2073	0.0048	0.0056	0.2828
145	O	3.4452	3.7244	6.3541	0.2478	0.2679	0.2895
146	O	6.9255	3.4861	6.2318	0.4981	0.2507	0.2839
147	O	0.0414	10.3603	6.2220	0.0030	0.7452	0.2834
148	O	10.4879	3.5710	6.2128	0.7543	0.2568	0.2830

/db/jmorales/CoNi-alloy/Profiles/Ni(001)/CO-O

a =			6.950991869				
b =			6.950991869				
c =			21.950991869				
alpha =			90.0				
beta =			90.0				
gamma =			90.0				
	Atom	X	Y	Z	X	Y	Z
1	Ni	0.0000	0.0000	0.0000	0.0000	0.0000	0.0000
2	Ni	1.7378	1.7378	0.0000	0.2500	0.2500	0.0000
3	Ni	0.0000	3.4755	0.0000	0.0000	0.5000	0.0000
4	Ni	1.7378	5.2132	0.0000	0.2500	0.7500	0.0000
5	Ni	3.4755	0.0000	0.0000	0.5000	0.0000	0.0000
6	Ni	5.2132	1.7378	0.0000	0.7500	0.2500	0.0000
7	Ni	3.4755	3.4755	0.0000	0.5000	0.5000	0.0000
8	Ni	5.2132	5.2132	0.0000	0.7500	0.7500	0.0000
9	Ni	0.0000	1.7378	1.7378	0.0000	0.2500	0.0792
10	Ni	1.7378	0.0000	1.7378	0.2500	0.0000	0.0792
11	Ni	0.0000	5.2132	1.7378	0.0000	0.7500	0.0792
12	Ni	1.7378	3.4755	1.7378	0.2500	0.5000	0.0792
13	Ni	3.4755	1.7378	1.7378	0.5000	0.2500	0.0792
14	Ni	5.2132	0.0000	1.7378	0.7500	0.0000	0.0792
15	Ni	3.4755	5.2132	1.7378	0.5000	0.7500	0.0792
16	Ni	5.2132	3.4755	1.7378	0.7500	0.5000	0.0792
17	Ni	6.9509	0.0002	3.5079	1.0000	0.0000	0.1598
18	Ni	1.7375	1.7378	3.5046	0.2500	0.2500	0.1597
19	Ni	6.9507	3.4754	3.5110	1.0000	0.5000	0.1599
20	Ni	1.7374	5.2134	3.5052	0.2500	0.7500	0.1597
21	Ni	3.4754	0.0001	3.5114	0.5000	0.0000	0.1600
22	Ni	5.2132	1.7375	3.5055	0.7500	0.2500	0.1597
23	Ni	3.4752	3.4752	3.4661	0.5000	0.5000	0.1579
24	Ni	5.2135	5.2135	3.5051	0.7500	0.7500	0.1597
25	Ni	0.0000	1.7384	5.2140	0.0000	0.2501	0.2375
26	Ni	1.7372	0.0001	5.2142	0.2499	0.0000	0.2375
27	Ni	6.9506	5.2136	5.2139	0.9999	0.7500	0.2375
28	Ni	1.7550	3.4763	5.2639	0.2525	0.5001	0.2398
29	Ni	3.4748	1.7562	5.2637	0.4999	0.2527	0.2398
30	Ni	5.2129	0.0003	5.2137	0.7499	0.0000	0.2375
31	Ni	3.4750	5.1961	5.2640	0.4999	0.7475	0.2398
32	Ni	5.1942	3.4756	5.2633	0.7473	0.5000	0.2398
33	O	3.4754	3.4748	7.5578	0.5000	0.4999	0.3443
34	C	3.4732	3.4786	6.3439	0.4997	0.5004	0.2890
35	O	3.4000	1.8000	7.3000	0.4891	0.2590	0.3326

/db/jmorales/CoNi-alloy/Profiles/Ni-Ce_3SmO_7/O-coverage/TS-O-coverage/TS-Ni-Ce_3SmO_7-5O

a = 13.9019837372
b = 13.9019837372
c = 21.9509918687
alpha = 90.0
beta = 90.0
gamma = 90.0

	Atom	X	Y	Z	X	Y	Z
1	Ni	0.0000	0.0000	0.0000	0.0000	0.0000	0.0000
2	Ni	1.7377	1.7377	0.0000	0.1250	0.1250	0.0000
3	Ni	0.0000	3.4755	0.0000	0.0000	0.2500	0.0000
4	Ni	1.7377	5.2132	0.0000	0.1250	0.3750	0.0000
5	Ni	3.4755	0.0000	0.0000	0.2500	0.0000	0.0000
6	Ni	5.2132	1.7377	0.0000	0.3750	0.1250	0.0000
7	Ni	3.4755	3.4755	0.0000	0.2500	0.2500	0.0000
8	Ni	5.2132	5.2132	0.0000	0.3750	0.3750	0.0000
9	Ni	0.0000	6.9510	0.0000	0.0000	0.5000	0.0000
10	Ni	1.7377	8.6887	0.0000	0.1250	0.6250	0.0000
11	Ni	0.0000	10.4265	0.0000	0.0000	0.7500	0.0000
12	Ni	1.7377	12.1642	0.0000	0.1250	0.8750	0.0000
13	Ni	3.4755	6.9510	0.0000	0.2500	0.5000	0.0000
14	Ni	5.2132	8.6887	0.0000	0.3750	0.6250	0.0000
15	Ni	3.4755	10.4265	0.0000	0.2500	0.7500	0.0000
16	Ni	5.2132	12.1642	0.0000	0.3750	0.8750	0.0000
17	Ni	6.9510	0.0000	0.0000	0.5000	0.0000	0.0000
18	Ni	8.6887	1.7377	0.0000	0.6250	0.1250	0.0000
19	Ni	6.9510	3.4755	0.0000	0.5000	0.2500	0.0000
20	Ni	8.6887	5.2132	0.0000	0.6250	0.3750	0.0000
21	Ni	10.4265	0.0000	0.0000	0.7500	0.0000	0.0000
22	Ni	12.1642	1.7377	0.0000	0.8750	0.1250	0.0000
23	Ni	10.4265	3.4755	0.0000	0.7500	0.2500	0.0000
24	Ni	12.1642	5.2132	0.0000	0.8750	0.3750	0.0000
25	Ni	6.9510	6.9510	0.0000	0.5000	0.5000	0.0000
26	Ni	8.6887	8.6887	0.0000	0.6250	0.6250	0.0000
27	Ni	6.9510	10.4265	0.0000	0.5000	0.7500	0.0000
28	Ni	8.6887	12.1642	0.0000	0.6250	0.8750	0.0000
29	Ni	10.4265	6.9510	0.0000	0.7500	0.5000	0.0000
30	Ni	12.1642	8.6887	0.0000	0.8750	0.6250	0.0000
31	Ni	10.4265	10.4265	0.0000	0.7500	0.7500	0.0000
32	Ni	12.1642	12.1642	0.0000	0.8750	0.8750	0.0000
33	Ni	0.0000	1.7377	1.7377	0.0000	0.1250	0.0792
34	Ni	1.7377	0.0000	1.7377	0.1250	0.0000	0.0792
35	Ni	0.0000	5.2132	1.7377	0.0000	0.3750	0.0792
36	Ni	1.7377	3.4755	1.7377	0.1250	0.2500	0.0792
37	Ni	3.4755	1.7377	1.7377	0.2500	0.1250	0.0792
38	Ni	5.2132	0.0000	1.7377	0.3750	0.0000	0.0792
39	Ni	3.4755	5.2132	1.7377	0.2500	0.3750	0.0792

40	Ni	5.2132	3.4755	1.7377	0.3750	0.2500	0.0792
41	Ni	0.0000	8.6887	1.7377	0.0000	0.6250	0.0792
42	Ni	1.7377	6.9510	1.7377	0.1250	0.5000	0.0792
43	Ni	0.0000	12.1642	1.7377	0.0000	0.8750	0.0792
44	Ni	1.7377	10.4265	1.7377	0.1250	0.7500	0.0792
45	Ni	3.4755	8.6887	1.7377	0.2500	0.6250	0.0792
46	Ni	5.2132	6.9510	1.7377	0.3750	0.5000	0.0792
47	Ni	3.4755	12.1642	1.7377	0.2500	0.8750	0.0792
48	Ni	5.2132	10.4265	1.7377	0.3750	0.7500	0.0792
49	Ni	6.9510	1.7377	1.7377	0.5000	0.1250	0.0792
50	Ni	8.6887	0.0000	1.7377	0.6250	0.0000	0.0792
51	Ni	6.9510	5.2132	1.7377	0.5000	0.3750	0.0792
52	Ni	8.6887	3.4755	1.7377	0.6250	0.2500	0.0792
53	Ni	10.4265	1.7377	1.7377	0.7500	0.1250	0.0792
54	Ni	12.1642	0.0000	1.7377	0.8750	0.0000	0.0792
55	Ni	10.4265	5.2132	1.7377	0.7500	0.3750	0.0792
56	Ni	12.1642	3.4755	1.7377	0.8750	0.2500	0.0792
57	Ni	6.9510	8.6887	1.7377	0.5000	0.6250	0.0792
58	Ni	8.6887	6.9510	1.7377	0.6250	0.5000	0.0792
59	Ni	6.9510	12.1642	1.7377	0.5000	0.8750	0.0792
60	Ni	8.6887	10.4265	1.7377	0.6250	0.7500	0.0792
61	Ni	10.4265	8.6887	1.7377	0.7500	0.6250	0.0792
62	Ni	12.1642	6.9510	1.7377	0.8750	0.5000	0.0792
63	Ni	10.4265	12.1642	1.7377	0.7500	0.8750	0.0792
64	Ni	12.1642	10.4265	1.7377	0.8750	0.7500	0.0792
65	Ni	0.0109	0.0199	3.4962	0.0008	0.0014	0.1593
66	Ni	1.7542	1.7747	3.4692	0.1262	0.1277	0.1580
67	Ni	13.8809	3.4787	3.5035	0.9985	0.2502	0.1596
68	Ni	1.6934	5.1883	3.4557	0.1218	0.3732	0.1574
69	Ni	3.4626	-0.0001	3.5091	0.2491	-0.0000	0.1599
70	Ni	5.2205	1.7434	3.4847	0.3755	0.1254	0.1587
71	Ni	3.5008	3.4409	3.4787	0.2518	0.2475	0.1585
72	Ni	5.2371	5.2040	3.4764	0.3767	0.3743	0.1584
73	Ni	0.0002	6.9368	3.5121	0.0000	0.4990	0.1600
74	Ni	1.7510	8.6534	3.5407	0.1260	0.6225	0.1613
75	Ni	13.8886	10.4171	3.5166	0.9990	0.7493	0.1602
76	Ni	1.7200	12.1735	3.5216	0.1237	0.8757	0.1604
77	Ni	3.4765	6.9752	3.4639	0.2501	0.5017	0.1578
78	Ni	5.2341	8.7110	3.4994	0.3765	0.6266	0.1594
79	Ni	3.4639	10.3997	3.5172	0.2492	0.7481	0.1602
80	Ni	5.2078	12.1696	3.5016	0.3746	0.8754	0.1595
81	Ni	6.9440	13.8929	3.5048	0.4995	0.9993	0.1597
82	Ni	8.6700	1.7163	3.4375	0.6237	0.1235	0.1566
83	Ni	6.9507	3.4575	3.4967	0.5000	0.2487	0.1593
84	Ni	8.6590	5.2205	3.5204	0.6229	0.3755	0.1604
85	Ni	10.4472	13.8977	3.5029	0.7515	0.9997	0.1596
86	Ni	12.1693	1.7416	3.5337	0.8754	0.1253	0.1610
87	Ni	10.4134	3.4634	3.4833	0.7491	0.2491	0.1587
88	Ni	12.1524	5.2148	3.4896	0.8741	0.3751	0.1590

89	Ni	6.9440	6.9483	3.5064	0.4995	0.4998	0.1597
90	Ni	8.6960	8.6886	3.4671	0.6255	0.6250	0.1579
91	Ni	6.9895	10.4346	3.5127	0.5028	0.7506	0.1600
92	Ni	8.7185	12.1894	3.5143	0.6271	0.8768	0.1601
93	Ni	10.4147	6.9466	3.5113	0.7492	0.4997	0.1600
94	Ni	12.1813	8.6706	3.5110	0.8762	0.6237	0.1599
95	Ni	10.4505	10.4400	3.4428	0.7517	0.7510	0.1568
96	Ni	12.1775	12.1615	3.4396	0.8760	0.8748	0.1567
97	Ni	0.0096	1.8101	5.2683	0.0007	0.1302	0.2400
98	Ni	1.7205	0.0073	5.2798	0.1238	0.0005	0.2405
99	Ni	13.8346	5.1576	5.3047	0.9952	0.3710	0.2417
100	Ni	1.7551	3.5648	5.3773	0.1262	0.2564	0.2450
101	Ni	3.4545	1.7424	5.3792	0.2485	0.1253	0.2451
102	Ni	5.1908	13.8661	5.2730	0.3734	0.9974	0.2402
103	Ni	3.5203	5.1809	5.0482	0.2532	0.3727	0.2300
104	Ni	5.2377	3.4637	5.3689	0.3768	0.2491	0.2446
105	Ni	0.0260	8.6813	5.2051	0.0019	0.6245	0.2371
106	Ni	1.7291	6.8555	5.2863	0.1244	0.4931	0.2408
107	Ni	0.0086	12.1483	5.2719	0.0006	0.8739	0.2402
108	Ni	1.7294	10.4258	5.2069	0.1244	0.7500	0.2372
109	Ni	3.4512	8.7254	5.3642	0.2483	0.6276	0.2444
110	Ni	5.2119	6.9781	5.2005	0.3749	0.5019	0.2369
111	Ni	3.4592	12.1402	5.2042	0.2488	0.8733	0.2371
112	Ni	5.2272	10.4505	5.1814	0.3760	0.7517	0.2360
113	Ni	6.9506	1.6781	5.3967	0.5000	0.1207	0.2459
114	Ni	8.6899	13.8879	5.2660	0.6251	0.9990	0.2399
115	Ni	6.9526	5.2031	5.2647	0.5001	0.3743	0.2398
116	Ni	8.6897	3.4260	5.2236	0.6251	0.2464	0.2380
117	Ni	10.4351	1.6968	5.2641	0.7506	0.1221	0.2398
118	Ni	12.2182	0.0422	5.2718	0.8789	0.0030	0.2402
119	Ni	10.3675	5.2024	5.2418	0.7458	0.3742	0.2388
120	Ni	12.0742	3.4584	5.2960	0.8685	0.2488	0.2413
121	Ni	6.9900	8.7560	5.2448	0.5028	0.6298	0.2389
122	Ni	8.6905	6.9534	5.2774	0.6251	0.5002	0.2404
123	Ni	6.9549	12.1518	5.2070	0.5003	0.8741	0.2372
124	Ni	8.7526	10.4873	5.2246	0.6296	0.7544	0.2380
125	Ni	10.4615	8.6504	5.1778	0.7525	0.6222	0.2359
126	Ni	12.1540	6.9576	5.3449	0.8743	0.5005	0.2435
127	Ni	10.4874	12.2251	5.2983	0.7544	0.8794	0.2414
128	Ni	12.1540	10.4158	5.3455	0.8743	0.7492	0.2435
129	O	6.1369	4.5062	6.8397	0.4414	0.3241	0.3116
130	O	4.2182	7.6310	6.7409	0.3034	0.5489	0.3071
131	O	6.0065	6.6745	8.8117	0.4321	0.4801	0.4014
132	O	7.8604	7.9160	6.8180	0.5654	0.5694	0.3106
133	O	10.3320	10.1641	6.3114	0.7432	0.7311	0.2875
134	O	9.4560	7.1213	9.0314	0.6802	0.5122	0.4114
135	O	7.5939	9.9077	8.9199	0.5462	0.7127	0.4064
136	Ce	5.9241	8.8775	8.0320	0.4261	0.6386	0.3659
137	Ce	4.3308	5.5423	7.7515	0.3115	0.3987	0.3531

138 Ce	8.0967	5.7916	8.1020	0.5824	0.4166	0.3691
139 Sm	9.3977	9.0863	8.1957	0.6760	0.6536	0.3734
140 C	11.3515	4.5325	6.7430	0.8165	0.3260	0.3072
141 O	12.7243	5.7689	6.6384	0.9153	0.4150	0.3024
142 O	10.9757	4.3667	7.8817	0.7895	0.3141	0.3591
143 O	2.2127	5.2864	6.3995	0.1592	0.3803	0.2915
144 O	1.6669	1.7106	6.1906	0.1199	0.1230	0.2820
145 O	8.7370	1.7195	6.1651	0.6285	0.1237	0.2809
146 O	12.2688	12.2915	6.1915	0.8825	0.8842	0.2821
147 O	5.1964	1.6109	6.2082	0.3738	0.1159	0.2828

/db/jmorales/CoNi-alloy/Profiles/Ni-Ce_3SmO_7/O-coverage/TS-O-coverage/TS-Ni-Ce_3SmO_7-O

a = 13.9019837372
b = 13.9019837372
c = 21.9509918687
alpha = 90.0
beta = 90.0
gamma = 90.0

	Atom	X	Y	Z	X	Y	Z
1	Ni	0.0000	0.0000	0.0000	0.0000	0.0000	0.0000
2	Ni	1.7377	1.7377	0.0000	0.1250	0.1250	0.0000
3	Ni	0.0000	3.4755	0.0000	0.0000	0.2500	0.0000
4	Ni	1.7377	5.2132	0.0000	0.1250	0.3750	0.0000
5	Ni	3.4755	0.0000	0.0000	0.2500	0.0000	0.0000
6	Ni	5.2132	1.7377	0.0000	0.3750	0.1250	0.0000
7	Ni	3.4755	3.4755	0.0000	0.2500	0.2500	0.0000
8	Ni	5.2132	5.2132	0.0000	0.3750	0.3750	0.0000
9	Ni	0.0000	6.9510	0.0000	0.0000	0.5000	0.0000
10	Ni	1.7377	8.6887	0.0000	0.1250	0.6250	0.0000
11	Ni	0.0000	10.4265	0.0000	0.0000	0.7500	0.0000
12	Ni	1.7377	12.1642	0.0000	0.1250	0.8750	0.0000
13	Ni	3.4755	6.9510	0.0000	0.2500	0.5000	0.0000
14	Ni	5.2132	8.6887	0.0000	0.3750	0.6250	0.0000
15	Ni	3.4755	10.4265	0.0000	0.2500	0.7500	0.0000
16	Ni	5.2132	12.1642	0.0000	0.3750	0.8750	0.0000
17	Ni	6.9510	0.0000	0.0000	0.5000	0.0000	0.0000
18	Ni	8.6887	1.7377	0.0000	0.6250	0.1250	0.0000
19	Ni	6.9510	3.4755	0.0000	0.5000	0.2500	0.0000
20	Ni	8.6887	5.2132	0.0000	0.6250	0.3750	0.0000
21	Ni	10.4265	0.0000	0.0000	0.7500	0.0000	0.0000
22	Ni	12.1642	1.7377	0.0000	0.8750	0.1250	0.0000
23	Ni	10.4265	3.4755	0.0000	0.7500	0.2500	0.0000
24	Ni	12.1642	5.2132	0.0000	0.8750	0.3750	0.0000
25	Ni	6.9510	6.9510	0.0000	0.5000	0.5000	0.0000
26	Ni	8.6887	8.6887	0.0000	0.6250	0.6250	0.0000
27	Ni	6.9510	10.4265	0.0000	0.5000	0.7500	0.0000
28	Ni	8.6887	12.1642	0.0000	0.6250	0.8750	0.0000
29	Ni	10.4265	6.9510	0.0000	0.7500	0.5000	0.0000
30	Ni	12.1642	8.6887	0.0000	0.8750	0.6250	0.0000
31	Ni	10.4265	10.4265	0.0000	0.7500	0.7500	0.0000
32	Ni	12.1642	12.1642	0.0000	0.8750	0.8750	0.0000
33	Ni	0.0000	1.7377	1.7377	0.0000	0.1250	0.0792
34	Ni	1.7377	0.0000	1.7377	0.1250	0.0000	0.0792
35	Ni	0.0000	5.2132	1.7377	0.0000	0.3750	0.0792
36	Ni	1.7377	3.4755	1.7377	0.1250	0.2500	0.0792
37	Ni	3.4755	1.7377	1.7377	0.2500	0.1250	0.0792
38	Ni	5.2132	0.0000	1.7377	0.3750	0.0000	0.0792
39	Ni	3.4755	5.2132	1.7377	0.2500	0.3750	0.0792

40	Ni	5.2132	3.4755	1.7377	0.3750	0.2500	0.0792
41	Ni	0.0000	8.6887	1.7377	0.0000	0.6250	0.0792
42	Ni	1.7377	6.9510	1.7377	0.1250	0.5000	0.0792
43	Ni	0.0000	12.1642	1.7377	0.0000	0.8750	0.0792
44	Ni	1.7377	10.4265	1.7377	0.1250	0.7500	0.0792
45	Ni	3.4755	8.6887	1.7377	0.2500	0.6250	0.0792
46	Ni	5.2132	6.9510	1.7377	0.3750	0.5000	0.0792
47	Ni	3.4755	12.1642	1.7377	0.2500	0.8750	0.0792
48	Ni	5.2132	10.4265	1.7377	0.3750	0.7500	0.0792
49	Ni	6.9510	1.7377	1.7377	0.5000	0.1250	0.0792
50	Ni	8.6887	0.0000	1.7377	0.6250	0.0000	0.0792
51	Ni	6.9510	5.2132	1.7377	0.5000	0.3750	0.0792
52	Ni	8.6887	3.4755	1.7377	0.6250	0.2500	0.0792
53	Ni	10.4265	1.7377	1.7377	0.7500	0.1250	0.0792
54	Ni	12.1642	0.0000	1.7377	0.8750	0.0000	0.0792
55	Ni	10.4265	5.2132	1.7377	0.7500	0.3750	0.0792
56	Ni	12.1642	3.4755	1.7377	0.8750	0.2500	0.0792
57	Ni	6.9510	8.6887	1.7377	0.5000	0.6250	0.0792
58	Ni	8.6887	6.9510	1.7377	0.6250	0.5000	0.0792
59	Ni	6.9510	12.1642	1.7377	0.5000	0.8750	0.0792
60	Ni	8.6887	10.4265	1.7377	0.6250	0.7500	0.0792
61	Ni	10.4265	8.6887	1.7377	0.7500	0.6250	0.0792
62	Ni	12.1642	6.9510	1.7377	0.8750	0.5000	0.0792
63	Ni	10.4265	12.1642	1.7377	0.7500	0.8750	0.0792
64	Ni	12.1642	10.4265	1.7377	0.8750	0.7500	0.0792
65	Ni	0.0128	0.0165	3.5100	0.0009	0.0012	0.1599
66	Ni	1.7348	1.7782	3.5428	0.1248	0.1279	0.1614
67	Ni	13.8841	3.4751	3.5143	0.9987	0.2500	0.1601
68	Ni	1.6953	5.1970	3.4414	0.1219	0.3738	0.1568
69	Ni	3.4708	0.0064	3.5098	0.2497	0.0005	0.1599
70	Ni	5.2245	1.7397	3.5240	0.3758	0.1251	0.1605
71	Ni	3.5055	3.4398	3.4787	0.2522	0.2474	0.1585
72	Ni	5.2411	5.2060	3.4702	0.3770	0.3745	0.1581
73	Ni	13.9010	6.9338	3.5137	0.9999	0.4988	0.1601
74	Ni	1.7511	8.6551	3.5448	0.1260	0.6226	0.1615
75	Ni	13.8955	10.4198	3.5218	0.9995	0.7495	0.1604
76	Ni	1.7421	12.1636	3.5071	0.1253	0.8750	0.1598
77	Ni	3.4789	6.9745	3.4622	0.2502	0.5017	0.1577
78	Ni	5.2289	8.7092	3.5003	0.3761	0.6265	0.1595
79	Ni	3.4664	10.3981	3.5278	0.2493	0.7480	0.1607
80	Ni	5.2034	12.1643	3.5036	0.3743	0.8750	0.1596
81	Ni	6.9427	13.8856	3.5021	0.4994	0.9988	0.1595
82	Ni	8.6906	1.7047	3.4905	0.6251	0.1226	0.1590
83	Ni	6.9543	3.4606	3.4993	0.5002	0.2489	0.1594
84	Ni	8.6617	5.2379	3.5099	0.6231	0.3768	0.1599
85	Ni	10.4402	13.8722	3.5217	0.7510	0.9979	0.1604
86	Ni	12.1622	1.7395	3.5099	0.8749	0.1251	0.1599
87	Ni	10.4130	3.4644	3.4856	0.7490	0.2492	0.1588
88	Ni	12.1553	5.2100	3.4955	0.8744	0.3748	0.1592

89	Ni	6.9422	6.9482	3.5095	0.4994	0.4998	0.1599
90	Ni	8.6964	8.6944	3.4713	0.6255	0.6254	0.1581
91	Ni	6.9731	10.4320	3.5120	0.5016	0.7504	0.1600
92	Ni	8.6956	12.1740	3.4926	0.6255	0.8757	0.1591
93	Ni	10.4200	6.9587	3.5203	0.7495	0.5006	0.1604
94	Ni	12.1876	8.6628	3.5046	0.8767	0.6231	0.1597
95	Ni	10.4525	10.4407	3.4345	0.7519	0.7510	0.1565
96	Ni	12.1822	12.1678	3.5069	0.8763	0.8753	0.1598
97	Ni	0.0071	1.7729	5.2302	0.0005	0.1275	0.2383
98	Ni	1.7382	0.0093	5.2241	0.1250	0.0007	0.2380
99	Ni	13.8381	5.1537	5.3129	0.9954	0.3707	0.2420
100	Ni	1.7615	3.5531	5.3253	0.1267	0.2556	0.2426
101	Ni	3.4809	1.7386	5.2241	0.2504	0.1251	0.2380
102	Ni	5.2058	13.8987	5.2263	0.3745	0.9998	0.2381
103	Ni	3.5338	5.1801	5.0441	0.2542	0.3726	0.2298
104	Ni	5.2536	3.4224	5.3586	0.3779	0.2462	0.2441
105	Ni	0.0340	8.6741	5.2134	0.0024	0.6239	0.2375
106	Ni	1.7378	6.8394	5.2809	0.1250	0.4920	0.2406
107	Ni	0.0093	12.1731	5.2188	0.0007	0.8756	0.2377
108	Ni	1.7295	10.4300	5.2234	0.1244	0.7503	0.2380
109	Ni	3.4510	8.7152	5.3705	0.2482	0.6269	0.2447
110	Ni	5.2097	6.9735	5.2081	0.3747	0.5016	0.2373
111	Ni	3.4726	12.1577	5.2264	0.2498	0.8745	0.2381
112	Ni	5.2208	10.4402	5.1983	0.3755	0.7510	0.2368
113	Ni	6.9837	1.6832	5.2061	0.5024	0.1211	0.2372
114	Ni	8.6879	13.8720	5.2221	0.6249	0.9978	0.2379
115	Ni	6.9522	5.2015	5.2751	0.5001	0.3742	0.2403
116	Ni	8.6957	3.4439	5.1676	0.6255	0.2477	0.2354
117	Ni	10.4059	1.6925	5.2109	0.7485	0.1217	0.2374
118	Ni	12.1920	0.0195	5.2204	0.8770	0.0014	0.2378
119	Ni	10.3664	5.1997	5.2485	0.7457	0.3740	0.2391
120	Ni	12.0725	3.4452	5.3109	0.8684	0.2478	0.2419
121	Ni	6.9785	8.7515	5.2421	0.5020	0.6295	0.2388
122	Ni	8.7051	6.9637	5.2779	0.6262	0.5009	0.2404
123	Ni	6.9399	12.1577	5.2250	0.4992	0.8745	0.2380
124	Ni	8.7256	10.4730	5.2050	0.6277	0.7533	0.2371
125	Ni	10.4642	8.7059	5.1763	0.7527	0.6262	0.2358
126	Ni	12.1476	6.9655	5.3548	0.8738	0.5010	0.2439
127	Ni	10.4659	12.1751	5.2869	0.7528	0.8758	0.2409
128	Ni	12.1977	10.4184	5.2812	0.8774	0.7494	0.2406
129	O	6.1191	4.3811	6.8030	0.4402	0.3151	0.3099
130	O	4.2014	7.6044	6.7554	0.3022	0.5470	0.3077
131	O	6.0453	6.6113	8.7681	0.4349	0.4756	0.3994
132	O	7.8568	7.9070	6.7974	0.5652	0.5688	0.3097
133	O	10.4349	10.3951	6.2717	0.7506	0.7477	0.2857
134	O	9.4914	7.1693	8.9695	0.6827	0.5157	0.4086
135	O	7.6126	9.9077	8.8589	0.5476	0.7127	0.4036
136	Ce	5.9321	8.8403	8.0248	0.4267	0.6359	0.3656
137	Ce	4.3509	5.4955	7.7254	0.3130	0.3953	0.3519

138	Ce	8.1722	5.7867	8.0767	0.5878	0.4163	0.3679
139	Sm	9.4031	9.1162	8.0708	0.6764	0.6558	0.3677
140	C	11.3515	4.5325	6.7430	0.8165	0.3260	0.3072
141	O	12.7243	5.7689	6.6384	0.9153	0.4150	0.3024
142	O	10.9757	4.3667	7.8817	0.7895	0.3141	0.3591
143	O	2.1867	5.1979	6.3668	0.1573	0.3739	0.2900

/db/jmorales/CoNi-alloy/Profiles/CoNi-Ce_3SmO_7/O-coverage/M-CO2-O-coverage/M-CO_2-CoNi-Ce_3SmO_7-4O

a =			13.9033384264				
b =			13.9033384264				
c =			21.9516692133				
alpha =			90.0				
beta =			90.0				
gamma =			90.0				
	Atom	X	Y	Z	X	Y	Z
1	Ni	0.0000	0.0000	0.0000	0.0000	0.0000	0.0000
2	Ni	0.0000	3.4758	0.0000	0.0000	0.2500	0.0000
3	Ni	3.4758	0.0000	0.0000	0.2500	0.0000	0.0000
4	Ni	3.4758	3.4758	0.0000	0.2500	0.2500	0.0000
5	Ni	0.0000	6.9517	0.0000	0.0000	0.5000	0.0000
6	Ni	0.0000	10.4275	0.0000	0.0000	0.7500	0.0000
7	Ni	3.4758	6.9517	0.0000	0.2500	0.5000	0.0000
8	Ni	3.4758	10.4275	0.0000	0.2500	0.7500	0.0000
9	Ni	6.9517	0.0000	0.0000	0.5000	0.0000	0.0000
10	Ni	6.9517	3.4758	0.0000	0.5000	0.2500	0.0000
11	Ni	10.4275	0.0000	0.0000	0.7500	0.0000	0.0000
12	Ni	10.4275	3.4758	0.0000	0.7500	0.2500	0.0000
13	Ni	6.9517	6.9517	0.0000	0.5000	0.5000	0.0000
14	Ni	6.9517	10.4275	0.0000	0.5000	0.7500	0.0000
15	Ni	10.4275	6.9517	0.0000	0.7500	0.5000	0.0000
16	Ni	10.4275	10.4275	0.0000	0.7500	0.7500	0.0000
17	Ni	0.0000	1.7379	1.7379	0.0000	0.1250	0.0792
18	Ni	0.0000	5.2138	1.7379	0.0000	0.3750	0.0792
19	Ni	3.4758	1.7379	1.7379	0.2500	0.1250	0.0792
20	Ni	3.4758	5.2138	1.7379	0.2500	0.3750	0.0792
21	Ni	0.0000	8.6896	1.7379	0.0000	0.6250	0.0792
22	Ni	0.0000	12.1654	1.7379	0.0000	0.8750	0.0792
23	Ni	3.4758	8.6896	1.7379	0.2500	0.6250	0.0792
24	Ni	3.4758	12.1654	1.7379	0.2500	0.8750	0.0792
25	Ni	6.9517	1.7379	1.7379	0.5000	0.1250	0.0792
26	Ni	6.9517	5.2138	1.7379	0.5000	0.3750	0.0792
27	Ni	10.4275	1.7379	1.7379	0.7500	0.1250	0.0792
28	Ni	10.4275	5.2138	1.7379	0.7500	0.3750	0.0792
29	Ni	6.9517	8.6896	1.7379	0.5000	0.6250	0.0792
30	Ni	6.9517	12.1654	1.7379	0.5000	0.8750	0.0792
31	Ni	10.4275	8.6896	1.7379	0.7500	0.6250	0.0792
32	Ni	10.4275	12.1654	1.7379	0.7500	0.8750	0.0792
33	Ni	13.9015	0.0014	3.4481	0.9999	0.0001	0.1571
34	Ni	0.0258	3.4568	3.5305	0.0019	0.2486	0.1608
35	Ni	3.4684	0.0087	3.5147	0.2495	0.0006	0.1601
36	Ni	3.4838	3.4235	3.4638	0.2506	0.2462	0.1578
37	Ni	13.8995	6.9550	3.5072	0.9997	0.5002	0.1598
38	Ni	13.8743	10.4503	3.4975	0.9979	0.7516	0.1593
39	Ni	3.4668	6.9714	3.4866	0.2494	0.5014	0.1588

40	Ni	3.4981	10.3912	3.5080	0.2516	0.7474	0.1598
41	Ni	6.9483	0.0216	3.5085	0.4998	0.0016	0.1598
42	Ni	6.9357	3.4808	3.4529	0.4989	0.2504	0.1573
43	Ni	10.4359	0.0190	3.4543	0.7506	0.0014	0.1574
44	Ni	10.4185	3.4688	3.5175	0.7494	0.2495	0.1602
45	Ni	6.9658	6.9628	3.5097	0.5010	0.5008	0.1599
46	Ni	6.9750	10.4327	3.4753	0.5017	0.7504	0.1583
47	Ni	10.4345	6.9374	3.4872	0.7505	0.4990	0.1589
48	Ni	10.4298	10.4482	3.5052	0.7502	0.7515	0.1597
49	Ni	0.0647	1.7501	5.2721	0.0047	0.1259	0.2402
50	Ni	0.0127	5.2164	5.2109	0.0009	0.3752	0.2374
51	Ni	3.4516	1.7148	5.3259	0.2483	0.1233	0.2426
52	Ni	3.4579	5.2303	5.0647	0.2487	0.3762	0.2307
53	Ni	13.8555	8.7180	5.1823	0.9966	0.6270	0.2361
54	Ni	13.8679	12.1344	5.2464	0.9975	0.8728	0.2390
55	Ni	3.4447	8.7035	5.2573	0.2478	0.6260	0.2395
56	Ni	3.4767	12.0954	5.1831	0.2501	0.8700	0.2361
57	Ni	6.9477	1.7259	5.2396	0.4997	0.1241	0.2387
58	Ni	6.9862	5.2594	5.3806	0.5025	0.3783	0.2451
59	Ni	10.4159	1.7715	5.2516	0.7492	0.1274	0.2392
60	Ni	10.4198	5.2062	5.2078	0.7494	0.3745	0.2372
61	Ni	7.0165	8.7077	5.2361	0.5047	0.6263	0.2385
62	Ni	6.9410	12.2126	5.1776	0.4992	0.8784	0.2359
63	Ni	10.4268	8.6904	5.1981	0.7500	0.6251	0.2368
64	Ni	10.4767	12.1818	5.2665	0.7535	0.8762	0.2399
65	Co	1.7379	1.7379	0.0000	0.1250	0.1250	0.0000
66	Co	1.7379	5.2138	0.0000	0.1250	0.3750	0.0000
67	Co	5.2138	1.7379	0.0000	0.3750	0.1250	0.0000
68	Co	5.2138	5.2138	0.0000	0.3750	0.3750	0.0000
69	Co	1.7379	8.6896	0.0000	0.1250	0.6250	0.0000
70	Co	1.7379	12.1654	0.0000	0.1250	0.8750	0.0000
71	Co	5.2138	8.6896	0.0000	0.3750	0.6250	0.0000
72	Co	5.2138	12.1654	0.0000	0.3750	0.8750	0.0000
73	Co	8.6896	1.7379	0.0000	0.6250	0.1250	0.0000
74	Co	8.6896	5.2138	0.0000	0.6250	0.3750	0.0000
75	Co	12.1654	1.7379	0.0000	0.8750	0.1250	0.0000
76	Co	12.1654	5.2138	0.0000	0.8750	0.3750	0.0000
77	Co	8.6896	8.6896	0.0000	0.6250	0.6250	0.0000
78	Co	8.6896	12.1654	0.0000	0.6250	0.8750	0.0000
79	Co	12.1654	8.6896	0.0000	0.8750	0.6250	0.0000
80	Co	12.1654	12.1654	0.0000	0.8750	0.8750	0.0000
81	Co	1.7379	0.0000	1.7379	0.1250	0.0000	0.0792
82	Co	1.7379	3.4758	1.7379	0.1250	0.2500	0.0792
83	Co	5.2138	0.0000	1.7379	0.3750	0.0000	0.0792
84	Co	5.2138	3.4758	1.7379	0.3750	0.2500	0.0792
85	Co	1.7379	6.9517	1.7379	0.1250	0.5000	0.0792
86	Co	1.7379	10.4275	1.7379	0.1250	0.7500	0.0792
87	Co	5.2138	6.9517	1.7379	0.3750	0.5000	0.0792
88	Co	5.2138	10.4275	1.7379	0.3750	0.7500	0.0792

89	Co	8.6896	0.0000	1.7379	0.6250	0.0000	0.0792
90	Co	8.6896	3.4758	1.7379	0.6250	0.2500	0.0792
91	Co	12.1654	0.0000	1.7379	0.8750	0.0000	0.0792
92	Co	12.1654	3.4758	1.7379	0.8750	0.2500	0.0792
93	Co	8.6896	6.9517	1.7379	0.6250	0.5000	0.0792
94	Co	8.6896	10.4275	1.7379	0.6250	0.7500	0.0792
95	Co	12.1654	6.9517	1.7379	0.8750	0.5000	0.0792
96	Co	12.1654	10.4275	1.7379	0.8750	0.7500	0.0792
97	Co	1.7502	1.7368	3.5645	0.1259	0.1249	0.1624
98	Co	1.7080	5.2339	3.5131	0.1228	0.3764	0.1600
99	Co	5.2044	1.7335	3.5675	0.3743	0.1247	0.1625
100	Co	5.2388	5.1938	3.5017	0.3768	0.3736	0.1595
101	Co	1.7231	8.6947	3.5179	0.1239	0.6254	0.1603
102	Co	1.7302	12.1650	3.5246	0.1244	0.8750	0.1606
103	Co	5.2095	8.6915	3.5600	0.3747	0.6251	0.1622
104	Co	5.2200	12.1496	3.5477	0.3754	0.8739	0.1616
105	Co	8.6874	1.7410	3.5436	0.6248	0.1252	0.1614
106	Co	8.6696	5.1990	3.5649	0.6236	0.3739	0.1624
107	Co	12.1817	1.7444	3.5590	0.8762	0.1255	0.1621
108	Co	12.1706	5.2009	3.5601	0.8754	0.3741	0.1622
109	Co	8.6991	8.6949	3.5320	0.6257	0.6254	0.1609
110	Co	8.7092	12.1979	3.4743	0.6264	0.8773	0.1583
111	Co	12.1493	8.6878	3.5350	0.8738	0.6249	0.1610
112	Co	12.1503	12.1883	3.5535	0.8739	0.8766	0.1619
113	Co	1.7512	13.7924	5.3386	0.1260	0.9920	0.2432
114	Co	1.7889	3.5204	5.2894	0.1287	0.2532	0.2410
115	Co	5.2222	13.8732	5.2678	0.3756	0.9978	0.2400
116	Co	5.1837	3.4844	5.3356	0.3728	0.2506	0.2431
117	Co	1.6925	6.9984	5.3001	0.1217	0.5034	0.2414
118	Co	1.6965	10.4318	5.1480	0.1220	0.7503	0.2345
119	Co	5.2338	6.9171	5.1528	0.3764	0.4975	0.2347
120	Co	5.2532	10.4055	5.2373	0.3778	0.7484	0.2386
121	Co	8.7018	13.8790	5.3203	0.6259	0.9983	0.2424
122	Co	8.6583	3.4756	5.2644	0.6227	0.2500	0.2398
123	Co	12.1928	0.0460	5.3693	0.8770	0.0033	0.2446
124	Co	12.1796	3.4841	5.2056	0.8760	0.2506	0.2371
125	Co	8.7379	6.9688	5.1831	0.6285	0.5012	0.2361
126	Co	8.7451	10.4751	5.2070	0.6290	0.7534	0.2372
127	Co	12.1591	6.9438	5.2168	0.8745	0.4994	0.2376
128	Co	12.1534	10.4142	5.2101	0.8741	0.7490	0.2373
129	O	5.7497	6.0754	6.7758	0.4136	0.4370	0.3087
130	O	2.5607	7.8652	6.7888	0.1842	0.5657	0.3093
131	O	4.7581	7.8373	8.7912	0.3422	0.5637	0.4005
132	O	6.1824	9.6690	6.8370	0.4447	0.6954	0.3115
133	O	8.1386	9.3129	8.9703	0.5854	0.6698	0.4086
134	O	5.4234	11.2145	9.0947	0.3901	0.8066	0.4143
135	O	8.4596	12.0085	6.3572	0.6085	0.8637	0.2896
136	Ce	4.0089	10.0230	8.0894	0.2883	0.7209	0.3685
137	Ce	3.6657	6.1390	7.7503	0.2637	0.4415	0.3531

138 Ce	6.9894	7.7110	8.0843	0.5027	0.5546	0.3683
139 Sm	7.3926	11.1477	8.2620	0.5317	0.8018	0.3764
140 C	3.1706	13.2323	6.7464	0.2280	0.9517	0.3073
141 O	3.9891	0.4116	6.8178	0.2869	0.0296	0.3106
142 O	2.9199	12.5110	7.7482	0.2100	0.8999	0.3530
143 O	10.4718	0.1142	6.2151	0.7532	0.0082	0.2831
144 O	0.0370	0.0205	6.1987	0.0027	0.0015	0.2824
145 O	3.4384	3.8223	6.3844	0.2473	0.2749	0.2908
146 O	6.9806	3.3761	6.2208	0.5021	0.2428	0.2834

/db/jmorales/CoNi-alloy/Profiles/CoNi-Ce_3SmO_7/O-coverage/CO-O-O-coverage/CO-O-CoNi-Ce_3SmO_7-O

a = 13.9033384264
b = 13.9033384264
c = 21.9516692133
alpha = 90.0
beta = 90.0
gamma = 90.0

	Atom	X	Y	Z	X	Y	Z
1	Ni	0.0000	0.0000	0.0000	0.0000	0.0000	0.0000
2	Ni	0.0000	3.4758	0.0000	0.0000	0.2500	0.0000
3	Ni	3.4758	0.0000	0.0000	0.2500	0.0000	0.0000
4	Ni	3.4758	3.4758	0.0000	0.2500	0.2500	0.0000
5	Ni	0.0000	6.9517	0.0000	0.0000	0.5000	0.0000
6	Ni	0.0000	10.4275	0.0000	0.0000	0.7500	0.0000
7	Ni	3.4758	6.9517	0.0000	0.2500	0.5000	0.0000
8	Ni	3.4758	10.4275	0.0000	0.2500	0.7500	0.0000
9	Ni	6.9517	0.0000	0.0000	0.5000	0.0000	0.0000
10	Ni	6.9517	3.4758	0.0000	0.5000	0.2500	0.0000
11	Ni	10.4275	0.0000	0.0000	0.7500	0.0000	0.0000
12	Ni	10.4275	3.4758	0.0000	0.7500	0.2500	0.0000
13	Ni	6.9517	6.9517	0.0000	0.5000	0.5000	0.0000
14	Ni	6.9517	10.4275	0.0000	0.5000	0.7500	0.0000
15	Ni	10.4275	6.9517	0.0000	0.7500	0.5000	0.0000
16	Ni	10.4275	10.4275	0.0000	0.7500	0.7500	0.0000
17	Ni	0.0000	1.7379	1.7379	0.0000	0.1250	0.0792
18	Ni	0.0000	5.2138	1.7379	0.0000	0.3750	0.0792
19	Ni	3.4758	1.7379	1.7379	0.2500	0.1250	0.0792
20	Ni	3.4758	5.2138	1.7379	0.2500	0.3750	0.0792
21	Ni	0.0000	8.6896	1.7379	0.0000	0.6250	0.0792
22	Ni	0.0000	12.1654	1.7379	0.0000	0.8750	0.0792
23	Ni	3.4758	8.6896	1.7379	0.2500	0.6250	0.0792
24	Ni	3.4758	12.1654	1.7379	0.2500	0.8750	0.0792
25	Ni	6.9517	1.7379	1.7379	0.5000	0.1250	0.0792
26	Ni	6.9517	5.2138	1.7379	0.5000	0.3750	0.0792
27	Ni	10.4275	1.7379	1.7379	0.7500	0.1250	0.0792
28	Ni	10.4275	5.2138	1.7379	0.7500	0.3750	0.0792
29	Ni	6.9517	8.6896	1.7379	0.5000	0.6250	0.0792
30	Ni	6.9517	12.1654	1.7379	0.5000	0.8750	0.0792
31	Ni	10.4275	8.6896	1.7379	0.7500	0.6250	0.0792
32	Ni	10.4275	12.1654	1.7379	0.7500	0.8750	0.0792
33	Ni	13.8982	13.8962	3.5094	0.9996	0.9995	0.1599
34	Ni	0.0041	3.4743	3.5003	0.0003	0.2499	0.1595
35	Ni	3.4765	13.8958	3.4371	0.2500	0.9995	0.1566
36	Ni	3.4606	3.4303	3.4804	0.2489	0.2467	0.1585
37	Ni	0.0048	6.9590	3.5095	0.0003	0.5005	0.1599
38	Ni	13.8836	10.4369	3.4919	0.9986	0.7507	0.1591
39	Ni	3.4742	6.9548	3.4923	0.2499	0.5002	0.1591

40	Ni	3.4992	10.4283	3.5392	0.2517	0.7501	0.1612
41	Ni	6.9546	0.0151	3.4677	0.5002	0.0011	0.1580
42	Ni	6.9528	3.4885	3.5214	0.5001	0.2509	0.1604
43	Ni	10.4261	0.0157	3.4435	0.7499	0.0011	0.1569
44	Ni	10.4320	3.4723	3.5127	0.7503	0.2497	0.1600
45	Ni	6.9562	6.9595	3.4976	0.5003	0.5006	0.1593
46	Ni	6.9678	10.4433	3.4838	0.5012	0.7511	0.1587
47	Ni	10.4372	6.9388	3.4874	0.7507	0.4991	0.1589
48	Ni	10.4237	10.4414	3.5051	0.7497	0.7510	0.1597
49	Ni	13.9015	1.7364	5.2033	0.9999	0.1249	0.2370
50	Ni	0.0130	5.2159	5.2111	0.0009	0.3752	0.2374
51	Ni	3.4745	1.7482	5.2567	0.2499	0.1257	0.2395
52	Ni	3.4482	5.1640	5.0833	0.2480	0.3714	0.2316
53	Ni	13.8764	8.7177	5.1867	0.9981	0.6270	0.2363
54	Ni	13.8999	12.1629	5.2092	0.9998	0.8748	0.2373
55	Ni	3.4514	8.6784	5.2330	0.2482	0.6242	0.2384
56	Ni	3.4801	12.1332	5.2728	0.2503	0.8727	0.2402
57	Ni	6.9227	1.7440	5.2449	0.4979	0.1254	0.2389
58	Ni	6.9715	5.1590	5.3640	0.5014	0.3711	0.2444
59	Ni	10.4586	1.8074	5.2576	0.7522	0.1300	0.2395
60	Ni	10.4250	5.2124	5.2128	0.7498	0.3749	0.2375
61	Ni	6.9736	8.7007	5.2156	0.5016	0.6258	0.2376
62	Ni	7.0238	12.2659	5.2473	0.5052	0.8822	0.2390
63	Ni	10.4374	8.6723	5.1876	0.7507	0.6238	0.2363
64	Ni	10.3735	12.1611	5.3487	0.7461	0.8747	0.2437
65	Co	1.7379	1.7379	0.0000	0.1250	0.1250	0.0000
66	Co	1.7379	5.2138	0.0000	0.1250	0.3750	0.0000
67	Co	5.2138	1.7379	0.0000	0.3750	0.1250	0.0000
68	Co	5.2138	5.2138	0.0000	0.3750	0.3750	0.0000
69	Co	1.7379	8.6896	0.0000	0.1250	0.6250	0.0000
70	Co	1.7379	12.1654	0.0000	0.1250	0.8750	0.0000
71	Co	5.2138	8.6896	0.0000	0.3750	0.6250	0.0000
72	Co	5.2138	12.1654	0.0000	0.3750	0.8750	0.0000
73	Co	8.6896	1.7379	0.0000	0.6250	0.1250	0.0000
74	Co	8.6896	5.2138	0.0000	0.6250	0.3750	0.0000
75	Co	12.1654	1.7379	0.0000	0.8750	0.1250	0.0000
76	Co	12.1654	5.2138	0.0000	0.8750	0.3750	0.0000
77	Co	8.6896	8.6896	0.0000	0.6250	0.6250	0.0000
78	Co	8.6896	12.1654	0.0000	0.6250	0.8750	0.0000
79	Co	12.1654	8.6896	0.0000	0.8750	0.6250	0.0000
80	Co	12.1654	12.1654	0.0000	0.8750	0.8750	0.0000
81	Co	1.7379	0.0000	1.7379	0.1250	0.0000	0.0792
82	Co	1.7379	3.4758	1.7379	0.1250	0.2500	0.0792
83	Co	5.2138	0.0000	1.7379	0.3750	0.0000	0.0792
84	Co	5.2138	3.4758	1.7379	0.3750	0.2500	0.0792
85	Co	1.7379	6.9517	1.7379	0.1250	0.5000	0.0792
86	Co	1.7379	10.4275	1.7379	0.1250	0.7500	0.0792
87	Co	5.2138	6.9517	1.7379	0.3750	0.5000	0.0792
88	Co	5.2138	10.4275	1.7379	0.3750	0.7500	0.0792

89	Co	8.6896	0.0000	1.7379	0.6250	0.0000	0.0792
90	Co	8.6896	3.4758	1.7379	0.6250	0.2500	0.0792
91	Co	12.1654	0.0000	1.7379	0.8750	0.0000	0.0792
92	Co	12.1654	3.4758	1.7379	0.8750	0.2500	0.0792
93	Co	8.6896	6.9517	1.7379	0.6250	0.5000	0.0792
94	Co	8.6896	10.4275	1.7379	0.6250	0.7500	0.0792
95	Co	12.1654	6.9517	1.7379	0.8750	0.5000	0.0792
96	Co	12.1654	10.4275	1.7379	0.8750	0.7500	0.0792
97	Co	1.7299	1.7249	3.5503	0.1244	0.1241	0.1617
98	Co	1.7077	5.2333	3.5195	0.1228	0.3764	0.1603
99	Co	5.2086	1.7295	3.5300	0.3746	0.1244	0.1608
100	Co	5.2382	5.1866	3.5140	0.3768	0.3730	0.1601
101	Co	1.7310	8.6889	3.5271	0.1245	0.6249	0.1607
102	Co	1.7419	12.1685	3.5439	0.1253	0.8752	0.1614
103	Co	5.2011	8.6828	3.5576	0.3741	0.6245	0.1621
104	Co	5.2327	12.1628	3.5719	0.3764	0.8748	0.1627
105	Co	8.6887	1.7433	3.5570	0.6249	0.1254	0.1620
106	Co	8.6694	5.2063	3.5670	0.6235	0.3745	0.1625
107	Co	12.1736	1.7441	3.5425	0.8756	0.1254	0.1614
108	Co	12.1701	5.2137	3.5530	0.8753	0.3750	0.1619
109	Co	8.6875	8.6722	3.5153	0.6249	0.6237	0.1601
110	Co	8.7112	12.1933	3.4850	0.6266	0.8770	0.1588
111	Co	12.1587	8.6904	3.5388	0.8745	0.6251	0.1612
112	Co	12.1340	12.1762	3.5861	0.8727	0.8758	0.1634
113	Co	1.7376	13.8923	5.2680	0.1250	0.9992	0.2400
114	Co	1.7290	3.4843	5.2093	0.1244	0.2506	0.2373
115	Co	5.2139	13.8976	5.3422	0.3750	0.9996	0.2434
116	Co	5.1687	3.4529	5.1830	0.3718	0.2483	0.2361
117	Co	1.7152	6.9596	5.3185	0.1234	0.5006	0.2423
118	Co	1.7094	10.4540	5.1752	0.1230	0.7519	0.2358
119	Co	5.2466	6.8719	5.1725	0.3774	0.4943	0.2356
120	Co	5.2387	10.4354	5.2312	0.3768	0.7506	0.2383
121	Co	8.7059	0.1047	5.3706	0.6262	0.0075	0.2447
122	Co	8.6943	3.4726	5.2163	0.6253	0.2498	0.2376
123	Co	12.1497	13.9006	5.2643	0.8739	0.9998	0.2398
124	Co	12.1996	3.5007	5.2212	0.8775	0.2518	0.2378
125	Co	8.7205	6.9691	5.1797	0.6272	0.5013	0.2360
126	Co	8.7379	10.3659	5.1941	0.6285	0.7456	0.2366
127	Co	12.1698	6.9471	5.2180	0.8753	0.4997	0.2377
128	Co	12.1655	10.4269	5.2151	0.8750	0.7500	0.2376
129	O	5.9293	6.0488	6.7923	0.4265	0.4351	0.3094
130	O	2.6351	7.7782	6.8170	0.1895	0.5594	0.3105
131	O	4.8172	7.7722	8.7922	0.3465	0.5590	0.4005
132	O	6.0728	9.6824	6.8275	0.4368	0.6964	0.3110
133	O	7.8313	9.6150	9.0936	0.5633	0.6916	0.4143
134	O	4.9703	11.3275	8.9081	0.3575	0.8147	0.4058
135	O	8.5698	11.7220	6.4030	0.6164	0.8431	0.2917
136	Ce	3.8605	9.7798	8.0153	0.2777	0.7034	0.3651
137	Ce	3.8271	6.0719	7.7168	0.2753	0.4367	0.3515

138	Ce	7.0808	7.8855	8.0988	0.5093	0.5672	0.3689
139	Sm	7.0188	11.4004	8.2140	0.5048	0.8200	0.3742
140	C	6.8725	0.1268	6.3583	0.4943	0.0091	0.2897
141	O	6.9895	0.0227	7.6013	0.5027	0.0016	0.3463
142	O	3.4580	13.8974	6.1633	0.2487	0.9996	0.2808
143	O	10.4832	0.1139	6.1970	0.7540	0.0082	0.2823

/db/jmorales/CoNi-alloy/Profiles/Ni-Ce_3SmO_7/O-coverage/CO_2-Ni-Ce_3SmO_7-5O

a =	13.9019837372
b =	13.9019837372
c =	21.9509918687
alpha =	90.0
beta =	90.0
gamma =	90.0

	Atom	X	Y	Z	X	Y	Z
1	Ni	0.0000	0.0000	0.0000	0.0000	0.0000	0.0000
2	Ni	1.7377	1.7377	0.0000	0.1250	0.1250	0.0000
3	Ni	0.0000	3.4755	0.0000	0.0000	0.2500	0.0000
4	Ni	1.7377	5.2132	0.0000	0.1250	0.3750	0.0000
5	Ni	3.4755	0.0000	0.0000	0.2500	0.0000	0.0000
6	Ni	5.2132	1.7377	0.0000	0.3750	0.1250	0.0000
7	Ni	3.4755	3.4755	0.0000	0.2500	0.2500	0.0000
8	Ni	5.2132	5.2132	0.0000	0.3750	0.3750	0.0000
9	Ni	0.0000	6.9510	0.0000	0.0000	0.5000	0.0000
10	Ni	1.7377	8.6887	0.0000	0.1250	0.6250	0.0000
11	Ni	0.0000	10.4265	0.0000	0.0000	0.7500	0.0000
12	Ni	1.7377	12.1642	0.0000	0.1250	0.8750	0.0000
13	Ni	3.4755	6.9510	0.0000	0.2500	0.5000	0.0000
14	Ni	5.2132	8.6887	0.0000	0.3750	0.6250	0.0000
15	Ni	3.4755	10.4265	0.0000	0.2500	0.7500	0.0000
16	Ni	5.2132	12.1642	0.0000	0.3750	0.8750	0.0000
17	Ni	6.9510	0.0000	0.0000	0.5000	0.0000	0.0000
18	Ni	8.6887	1.7377	0.0000	0.6250	0.1250	0.0000
19	Ni	6.9510	3.4755	0.0000	0.5000	0.2500	0.0000
20	Ni	8.6887	5.2132	0.0000	0.6250	0.3750	0.0000
21	Ni	10.4265	0.0000	0.0000	0.7500	0.0000	0.0000
22	Ni	12.1642	1.7377	0.0000	0.8750	0.1250	0.0000
23	Ni	10.4265	3.4755	0.0000	0.7500	0.2500	0.0000
24	Ni	12.1642	5.2132	0.0000	0.8750	0.3750	0.0000
25	Ni	6.9510	6.9510	0.0000	0.5000	0.5000	0.0000
26	Ni	8.6887	8.6887	0.0000	0.6250	0.6250	0.0000
27	Ni	6.9510	10.4265	0.0000	0.5000	0.7500	0.0000
28	Ni	8.6887	12.1642	0.0000	0.6250	0.8750	0.0000
29	Ni	10.4265	6.9510	0.0000	0.7500	0.5000	0.0000
30	Ni	12.1642	8.6887	0.0000	0.8750	0.6250	0.0000
31	Ni	10.4265	10.4265	0.0000	0.7500	0.7500	0.0000
32	Ni	12.1642	12.1642	0.0000	0.8750	0.8750	0.0000
33	Ni	0.0000	1.7377	1.7377	0.0000	0.1250	0.0792
34	Ni	1.7377	0.0000	1.7377	0.1250	0.0000	0.0792
35	Ni	0.0000	5.2132	1.7377	0.0000	0.3750	0.0792
36	Ni	1.7377	3.4755	1.7377	0.1250	0.2500	0.0792
37	Ni	3.4755	1.7377	1.7377	0.2500	0.1250	0.0792
38	Ni	5.2132	0.0000	1.7377	0.3750	0.0000	0.0792
39	Ni	3.4755	5.2132	1.7377	0.2500	0.3750	0.0792
40	Ni	5.2132	3.4755	1.7377	0.3750	0.2500	0.0792

41	Ni	0.0000	8.6887	1.7377	0.0000	0.6250	0.0792
42	Ni	1.7377	6.9510	1.7377	0.1250	0.5000	0.0792
43	Ni	0.0000	12.1642	1.7377	0.0000	0.8750	0.0792
44	Ni	1.7377	10.4265	1.7377	0.1250	0.7500	0.0792
45	Ni	3.4755	8.6887	1.7377	0.2500	0.6250	0.0792
46	Ni	5.2132	6.9510	1.7377	0.3750	0.5000	0.0792
47	Ni	3.4755	12.1642	1.7377	0.2500	0.8750	0.0792
48	Ni	5.2132	10.4265	1.7377	0.3750	0.7500	0.0792
49	Ni	6.9510	1.7377	1.7377	0.5000	0.1250	0.0792
50	Ni	8.6887	0.0000	1.7377	0.6250	0.0000	0.0792
51	Ni	6.9510	5.2132	1.7377	0.5000	0.3750	0.0792
52	Ni	8.6887	3.4755	1.7377	0.6250	0.2500	0.0792
53	Ni	10.4265	1.7377	1.7377	0.7500	0.1250	0.0792
54	Ni	12.1642	0.0000	1.7377	0.8750	0.0000	0.0792
55	Ni	10.4265	5.2132	1.7377	0.7500	0.3750	0.0792
56	Ni	12.1642	3.4755	1.7377	0.8750	0.2500	0.0792
57	Ni	6.9510	8.6887	1.7377	0.5000	0.6250	0.0792
58	Ni	8.6887	6.9510	1.7377	0.6250	0.5000	0.0792
59	Ni	6.9510	12.1642	1.7377	0.5000	0.8750	0.0792
60	Ni	8.6887	10.4265	1.7377	0.6250	0.7500	0.0792
61	Ni	10.4265	8.6887	1.7377	0.7500	0.6250	0.0792
62	Ni	12.1642	6.9510	1.7377	0.8750	0.5000	0.0792
63	Ni	10.4265	12.1642	1.7377	0.7500	0.8750	0.0792
64	Ni	12.1642	10.4265	1.7377	0.8750	0.7500	0.0792
65	Ni	0.0196	0.0058	3.4989	0.0014	0.0004	0.1594
66	Ni	1.7468	1.7657	3.4549	0.1257	0.1270	0.1574
67	Ni	0.0084	3.4734	3.5007	0.0006	0.2498	0.1595
68	Ni	1.7086	5.2177	3.4610	0.1229	0.3753	0.1577
69	Ni	3.4633	13.8982	3.5071	0.2491	0.9997	0.1598
70	Ni	5.2112	1.7401	3.5485	0.3748	0.1252	0.1617
71	Ni	3.4974	3.4508	3.4901	0.2516	0.2482	0.1590
72	Ni	5.2171	5.2200	3.4720	0.3753	0.3755	0.1582
73	Ni	13.8740	6.9484	3.5216	0.9980	0.4998	0.1604
74	Ni	1.7490	8.6582	3.4779	0.1258	0.6228	0.1584
75	Ni	13.8848	10.4194	3.5197	0.9988	0.7495	0.1603
76	Ni	1.7246	12.1495	3.5432	0.1241	0.8739	0.1614
77	Ni	3.4717	6.9817	3.4752	0.2497	0.5022	0.1583
78	Ni	5.1902	8.7109	3.4882	0.3733	0.6266	0.1589
79	Ni	3.4595	10.4059	3.5267	0.2489	0.7485	0.1607
80	Ni	5.2047	12.1672	3.4948	0.3744	0.8752	0.1592
81	Ni	6.9436	13.8969	3.5026	0.4995	0.9996	0.1596
82	Ni	8.6863	1.7219	3.4249	0.6248	0.1239	0.1560
83	Ni	6.9336	3.4640	3.4872	0.4988	0.2492	0.1589
84	Ni	8.6865	5.2147	3.5443	0.6248	0.3751	0.1615
85	Ni	10.4396	0.0016	3.5102	0.7509	0.0001	0.1599
86	Ni	12.1759	1.7198	3.5261	0.8758	0.1237	0.1606
87	Ni	10.4314	3.4750	3.4941	0.7504	0.2500	0.1592
88	Ni	12.1432	5.2257	3.5219	0.8735	0.3759	0.1604
89	Ni	6.9481	6.9287	3.4949	0.4998	0.4984	0.1592

90	Ni	8.6966	8.6820	3.4559	0.6256	0.6245	0.1574
91	Ni	6.9897	10.4735	3.4913	0.5028	0.7534	0.1591
92	Ni	8.7174	12.1947	3.5322	0.6271	0.8772	0.1609
93	Ni	10.4158	6.9288	3.5404	0.7492	0.4984	0.1613
94	Ni	12.1805	8.6758	3.5221	0.8762	0.6241	0.1605
95	Ni	10.4311	10.4483	3.4508	0.7503	0.7516	0.1572
96	Ni	12.1668	12.1667	3.4412	0.8752	0.8752	0.1568
97	Ni	0.0618	1.7747	5.2733	0.0044	0.1277	0.2402
98	Ni	1.7363	13.8737	5.2896	0.1249	0.9980	0.2410
99	Ni	13.8464	5.1917	5.1869	0.9960	0.3734	0.2363
100	Ni	1.7607	3.5744	5.4272	0.1267	0.2571	0.2472
101	Ni	3.4468	1.7379	5.2808	0.2479	0.1250	0.2406
102	Ni	5.1968	13.8903	5.2126	0.3738	0.9992	0.2375
103	Ni	3.5047	5.2157	5.0637	0.2521	0.3752	0.2307
104	Ni	5.2071	3.4246	5.3333	0.3746	0.2463	0.2430
105	Ni	0.0132	8.6784	5.2641	0.0009	0.6243	0.2398
106	Ni	1.7038	6.8836	5.3680	0.1226	0.4951	0.2445
107	Ni	13.8968	12.1477	5.2720	0.9996	0.8738	0.2402
108	Ni	1.7433	10.4232	5.2933	0.1254	0.7498	0.2411
109	Ni	3.4904	8.7001	5.4518	0.2511	0.6258	0.2484
110	Ni	5.2529	7.0011	5.2268	0.3779	0.5036	0.2381
111	Ni	3.4796	12.1516	5.2115	0.2503	0.8741	0.2374
112	Ni	5.2374	10.4495	5.1862	0.3767	0.7517	0.2363
113	Ni	6.9530	1.6900	5.2622	0.5001	0.1216	0.2397
114	Ni	8.6902	0.0034	5.2777	0.6251	0.0002	0.2404
115	Ni	6.9190	5.1948	5.2426	0.4977	0.3737	0.2388
116	Ni	8.6700	3.4330	5.1985	0.6237	0.2469	0.2368
117	Ni	10.4235	1.7347	5.2712	0.7498	0.1248	0.2401
118	Ni	12.2083	0.0484	5.2809	0.8782	0.0035	0.2406
119	Ni	10.4121	5.1272	5.3151	0.7490	0.3688	0.2421
120	Ni	12.1808	3.4282	5.1835	0.8762	0.2466	0.2361
121	Ni	6.9589	8.7563	5.0740	0.5006	0.6299	0.2312
122	Ni	8.6214	6.9905	5.3039	0.6202	0.5028	0.2416
123	Ni	6.9489	12.1671	5.2173	0.4998	0.8752	0.2377
124	Ni	8.7089	10.4683	5.2821	0.6265	0.7530	0.2406
125	Ni	10.4207	8.6751	5.1391	0.7496	0.6240	0.2341
126	Ni	12.1618	6.9569	5.3781	0.8748	0.5004	0.2450
127	Ni	10.4683	12.2337	5.2988	0.7530	0.8800	0.2414
128	Ni	12.1320	10.4263	5.3242	0.8727	0.7500	0.2426
129	O	6.1170	4.3172	6.8255	0.4400	0.3105	0.3109
130	O	4.4378	7.6406	6.8527	0.3192	0.5496	0.3122
131	O	6.2385	6.3927	8.7891	0.4487	0.4598	0.4004
132	O	8.4239	7.4134	7.1796	0.6059	0.5333	0.3271
133	O	10.2888	10.1134	6.3844	0.7401	0.7275	0.2908
134	O	10.3303	6.4820	9.0272	0.7431	0.4663	0.4112
135	O	8.1434	9.4931	9.0465	0.5858	0.6829	0.4121
136	Ce	6.4657	8.5883	7.9413	0.4651	0.6178	0.3618
137	Ce	4.3515	5.5327	7.7211	0.3130	0.3980	0.3517
138	Ce	8.0384	5.2618	7.9236	0.5782	0.3785	0.3610

139 Sm	9.9167	8.7934	8.1965	0.7133	0.6325	0.3734
140 C	10.9136	6.1636	7.9043	0.7850	0.4434	0.3601
141 O	11.7306	7.0093	7.3553	0.8438	0.5042	0.3351
142 O	10.5371	5.0607	7.3117	0.7580	0.3640	0.3331
143 O	2.2165	5.2543	6.4506	0.1594	0.3780	0.2939
144 O	1.7490	1.6812	6.1946	0.1258	0.1209	0.2822
145 O	8.6831	1.7678	6.1816	0.6246	0.1272	0.2816
146 O	1.6293	8.7553	6.2267	0.1172	0.6298	0.2837
147 O	12.2407	12.2788	6.1895	0.8805	0.8832	0.2820

/db/jmorales/CoNi-alloy/Profiles/Sm(subsurf)$Ce_{26}O_{53}$/CO2

a =			11.6082000732				
b =			11.608200073110298				
c =			22.898399353				
alpha =			90.0				
beta =			90.0				
gamma =			120.0				
	Atom	X	Y	Z	X	Y	Z
1	Sm	1.9457	5.5935	3.8487	0.4458	0.5564	0.1681
2	Ce	1.9347	1.1170	0.7898	0.2222	0.1111	0.0345
3	Ce	0.0000	4.4680	0.7898	0.2222	0.4444	0.0345
4	Ce	-1.9347	7.8190	0.7898	0.2222	0.7778	0.0345
5	Ce	5.8041	1.1170	0.7898	0.5556	0.1111	0.0345
6	Ce	3.8694	4.4680	0.7898	0.5556	0.4444	0.0345
7	Ce	1.9347	7.8190	0.7898	0.5556	0.7778	0.0345
8	Ce	9.6735	1.1170	0.7898	0.8889	0.1111	0.0345
9	Ce	7.7388	4.4680	0.7898	0.8889	0.4444	0.0345
10	Ce	5.8041	7.8190	0.7898	0.8889	0.7778	0.0345
11	Ce	0.0029	2.2480	3.9523	0.1121	0.2236	0.1726
12	Ce	-1.9132	5.5857	3.9476	0.1130	0.5556	0.1724
13	Ce	-3.8707	8.9319	3.9420	0.1108	0.8885	0.1722
14	Ce	3.8690	2.2468	3.9401	0.4450	0.2235	0.1721
15	Ce	0.0146	8.9099	3.9664	0.4444	0.8863	0.1732
16	Ce	7.7372	2.2426	3.9063	0.7781	0.2231	0.1706
17	Ce	5.7627	5.5962	3.9707	0.7748	0.5567	0.1734
18	Ce	3.8556	8.8950	3.9902	0.7746	0.8848	0.1743
19	Ce	0.0078	-0.0019	7.0944	0.0006	-0.0002	0.3098
20	Ce	9.6483	3.3479	7.0816	0.9977	0.3330	0.3093
21	Ce	7.7203	6.7073	7.0942	0.9987	0.6672	0.3098
22	Ce	3.8685	-0.0021	7.0755	0.3332	-0.0002	0.3090
23	Ce	1.9650	3.1778	7.1348	0.3273	0.3161	0.3116
24	Ce	-0.2522	6.7655	7.2140	0.3148	0.6730	0.3150
25	Ce	7.7477	0.1360	7.0744	0.6742	0.0135	0.3089
26	Ce	5.8123	3.3468	7.0829	0.6672	0.3329	0.3093
27	Ce	4.0827	6.7517	7.3342	0.6875	0.6716	0.3203
28	O	0.0000	0.0000	0.0000	0.0000	0.0000	0.0000
29	O	-1.9347	3.3510	0.0000	-0.0000	0.3333	0.0000
30	O	-3.8694	6.7020	0.0000	-0.0000	0.6667	0.0000
31	O	3.8694	0.0000	0.0000	0.3333	0.0000	0.0000
32	O	1.9347	3.3510	0.0000	0.3333	0.3333	0.0000
33	O	0.0000	6.7020	0.0000	0.3333	0.6667	0.0000
34	O	7.7388	0.0000	0.0000	0.6667	0.0000	0.0000
35	O	5.8041	3.3510	0.0000	0.6667	0.3333	0.0000
36	O	3.8694	6.7020	0.0000	0.6667	0.6667	0.0000
37	O	-0.0000	2.2340	1.5798	0.1111	0.2222	0.0690
38	O	-1.9347	5.5850	1.5798	0.1111	0.5556	0.0690
39	O	-3.8694	8.9360	1.5798	0.1111	0.8889	0.0690
40	O	3.8694	2.2340	1.5798	0.4444	0.2222	0.0690

41	O	1.9347	5.5850	1.5798	0.4444	0.5556	0.0690
42	O	-0.0000	8.9360	1.5798	0.4444	0.8889	0.0690
43	O	7.7388	2.2340	1.5798	0.7778	0.2222	0.0690
44	O	5.8041	5.5850	1.5798	0.7778	0.5556	0.0690
45	O	3.8694	8.9360	1.5798	0.7778	0.8889	0.0690
46	O	1.9347	1.1170	3.1593	0.2222	0.1111	0.1380
47	O	0.0000	4.4680	3.1593	0.2222	0.4444	0.1380
48	O	-1.9347	7.8190	3.1593	0.2222	0.7778	0.1380
49	O	5.8041	1.1170	3.1593	0.5556	0.1111	0.1380
50	O	3.8694	4.4680	3.1593	0.5556	0.4444	0.1380
51	O	1.9347	7.8190	3.1593	0.5556	0.7778	0.1380
52	O	9.6735	1.1170	3.1593	0.8889	0.1111	0.1380
53	O	7.7388	4.4680	3.1593	0.8889	0.4444	0.1380
54	O	5.8041	7.8190	3.1593	0.8889	0.7778	0.1380
55	O	-0.0136	-0.0129	4.7550	-0.0018	-0.0013	0.2077
56	O	-1.9174	3.3639	4.7670	0.0021	0.3346	0.2082
57	O	-3.8675	6.7086	4.7443	0.0005	0.6673	0.2072
58	O	-1.9111	10.0296	4.7362	0.3342	0.9977	0.2068
59	O	1.9423	3.4077	4.8819	0.3368	0.3390	0.2132
60	O	0.0432	6.6739	4.9075	0.3357	0.6639	0.2143
61	O	7.7258	0.0049	4.7732	0.6658	0.0005	0.2084
62	O	5.7859	3.3536	4.7606	0.6652	0.3336	0.2079
63	O	3.8346	6.6900	4.8714	0.6631	0.6655	0.2127
64	O	-0.0200	2.1674	6.3248	0.1061	0.2156	0.2762
65	O	-2.0111	5.5880	6.3388	0.1047	0.5559	0.2768
66	O	-3.8542	8.9698	6.2565	0.1141	0.8922	0.2732
67	O	3.8939	2.1335	6.2827	0.4416	0.2122	0.2744
68	O	-0.0133	8.9604	6.3644	0.4445	0.8913	0.2779
69	O	7.7464	2.2635	6.2461	0.7799	0.2252	0.2728
70	O	5.9030	5.5559	6.3395	0.7848	0.5527	0.2769
71	O	3.8552	9.0303	6.3887	0.7812	0.8983	0.2790
72	O	1.9490	1.0741	7.9357	0.2213	0.1068	0.3466
73	O	0.2227	4.5803	7.5640	0.2470	0.4556	0.3303
74	O	-2.0382	7.8534	7.9258	0.2150	0.7812	0.3461
75	O	5.8640	1.1697	7.8914	0.5633	0.1164	0.3446
76	O	3.7531	4.3815	7.7055	0.5412	0.4358	0.3365
77	O	1.8542	7.7658	7.9112	0.5460	0.7725	0.3455
78	O	9.6056	1.2184	7.9251	0.8881	0.1212	0.3461
79	O	7.7622	4.3976	7.9205	0.8874	0.4374	0.3459
80	O	5.9851	7.9127	7.9609	0.9091	0.7871	0.3477
81	C	1.8153	7.1383	9.1757	0.5114	0.7101	0.4007
82	O	0.6408	6.8333	9.5465	0.3951	0.6797	0.4169
83	O	2.9359	6.8915	9.6845	0.5957	0.6855	0.4229

/db/jmorales/CoNi-alloy/Profiles/Ni-Ce_3SmO_7/O-CO-O

a =	13.9019837372
b =	13.9019837372
c =	21.9509918687
alpha =	90.0
beta =	90.0
gamma =	90.0

	Atom	X	Y	Z	X	Y	Z
1	Ni	0.0000	0.0000	0.0000	0.0000	0.0000	0.0000
2	Ni	1.7377	1.7377	0.0000	0.1250	0.1250	0.0000
3	Ni	0.0000	3.4755	0.0000	0.0000	0.2500	0.0000
4	Ni	1.7377	5.2132	0.0000	0.1250	0.3750	0.0000
5	Ni	3.4755	0.0000	0.0000	0.2500	0.0000	0.0000
6	Ni	5.2132	1.7377	0.0000	0.3750	0.1250	0.0000
7	Ni	3.4755	3.4755	0.0000	0.2500	0.2500	0.0000
8	Ni	5.2132	5.2132	0.0000	0.3750	0.3750	0.0000
9	Ni	0.0000	6.9510	0.0000	0.0000	0.5000	0.0000
10	Ni	1.7377	8.6887	0.0000	0.1250	0.6250	0.0000
11	Ni	0.0000	10.4265	0.0000	0.0000	0.7500	0.0000
12	Ni	1.7377	12.1642	0.0000	0.1250	0.8750	0.0000
13	Ni	3.4755	6.9510	0.0000	0.2500	0.5000	0.0000
14	Ni	5.2132	8.6887	0.0000	0.3750	0.6250	0.0000
15	Ni	3.4755	10.4265	0.0000	0.2500	0.7500	0.0000
16	Ni	5.2132	12.1642	0.0000	0.3750	0.8750	0.0000
17	Ni	6.9510	0.0000	0.0000	0.5000	0.0000	0.0000
18	Ni	8.6887	1.7377	0.0000	0.6250	0.1250	0.0000
19	Ni	6.9510	3.4755	0.0000	0.5000	0.2500	0.0000
20	Ni	8.6887	5.2132	0.0000	0.6250	0.3750	0.0000
21	Ni	10.4265	0.0000	0.0000	0.7500	0.0000	0.0000
22	Ni	12.1642	1.7377	0.0000	0.8750	0.1250	0.0000
23	Ni	10.4265	3.4755	0.0000	0.7500	0.2500	0.0000
24	Ni	12.1642	5.2132	0.0000	0.8750	0.3750	0.0000
25	Ni	6.9510	6.9510	0.0000	0.5000	0.5000	0.0000
26	Ni	8.6887	8.6887	0.0000	0.6250	0.6250	0.0000
27	Ni	6.9510	10.4265	0.0000	0.5000	0.7500	0.0000
28	Ni	8.6887	12.1642	0.0000	0.6250	0.8750	0.0000
29	Ni	10.4265	6.9510	0.0000	0.7500	0.5000	0.0000
30	Ni	12.1642	8.6887	0.0000	0.8750	0.6250	0.0000
31	Ni	10.4265	10.4265	0.0000	0.7500	0.7500	0.0000
32	Ni	12.1642	12.1642	0.0000	0.8750	0.8750	0.0000
33	Ni	0.0000	1.7377	1.7377	0.0000	0.1250	0.0792
34	Ni	1.7377	0.0000	1.7377	0.1250	0.0000	0.0792
35	Ni	0.0000	5.2132	1.7377	0.0000	0.3750	0.0792
36	Ni	1.7377	3.4755	1.7377	0.1250	0.2500	0.0792
37	Ni	3.4755	1.7377	1.7377	0.2500	0.1250	0.0792
38	Ni	5.2132	0.0000	1.7377	0.3750	0.0000	0.0792
39	Ni	3.4755	5.2132	1.7377	0.2500	0.3750	0.0792
40	Ni	5.2132	3.4755	1.7377	0.3750	0.2500	0.0792

41	Ni	0.0000	8.6887	1.7377	0.0000	0.6250	0.0792
42	Ni	1.7377	6.9510	1.7377	0.1250	0.5000	0.0792
43	Ni	0.0000	12.1642	1.7377	0.0000	0.8750	0.0792
44	Ni	1.7377	10.4265	1.7377	0.1250	0.7500	0.0792
45	Ni	3.4755	8.6887	1.7377	0.2500	0.6250	0.0792
46	Ni	5.2132	6.9510	1.7377	0.3750	0.5000	0.0792
47	Ni	3.4755	12.1642	1.7377	0.2500	0.8750	0.0792
48	Ni	5.2132	10.4265	1.7377	0.3750	0.7500	0.0792
49	Ni	6.9510	1.7377	1.7377	0.5000	0.1250	0.0792
50	Ni	8.6887	0.0000	1.7377	0.6250	0.0000	0.0792
51	Ni	6.9510	5.2132	1.7377	0.5000	0.3750	0.0792
52	Ni	8.6887	3.4755	1.7377	0.6250	0.2500	0.0792
53	Ni	10.4265	1.7377	1.7377	0.7500	0.1250	0.0792
54	Ni	12.1642	0.0000	1.7377	0.8750	0.0000	0.0792
55	Ni	10.4265	5.2132	1.7377	0.7500	0.3750	0.0792
56	Ni	12.1642	3.4755	1.7377	0.8750	0.2500	0.0792
57	Ni	6.9510	8.6887	1.7377	0.5000	0.6250	0.0792
58	Ni	8.6887	6.9510	1.7377	0.6250	0.5000	0.0792
59	Ni	6.9510	12.1642	1.7377	0.5000	0.8750	0.0792
60	Ni	8.6887	10.4265	1.7377	0.6250	0.7500	0.0792
61	Ni	10.4265	8.6887	1.7377	0.7500	0.6250	0.0792
62	Ni	12.1642	6.9510	1.7377	0.8750	0.5000	0.0792
63	Ni	10.4265	12.1642	1.7377	0.7500	0.8750	0.0792
64	Ni	12.1642	10.4265	1.7377	0.8750	0.7500	0.0792
65	Ni	0.0061	0.0026	3.5035	0.0004	0.0002	0.1596
66	Ni	1.7371	1.7322	3.5057	0.1250	0.1246	0.1597
67	Ni	13.8916	3.4674	3.5025	0.9993	0.2494	0.1596
68	Ni	1.7068	5.2032	3.4635	0.1228	0.3743	0.1578
69	Ni	3.4776	13.8999	3.5096	0.2502	0.9999	0.1599
70	Ni	5.2153	1.7480	3.5249	0.3751	0.1257	0.1606
71	Ni	3.4884	3.4365	3.4933	0.2509	0.2472	0.1591
72	Ni	5.1946	5.2093	3.4706	0.3737	0.3747	0.1581
73	Ni	13.8854	6.9454	3.5043	0.9988	0.4996	0.1596
74	Ni	1.7478	8.6990	3.5130	0.1257	0.6257	0.1600
75	Ni	13.8856	10.4344	3.5186	0.9988	0.7506	0.1603
76	Ni	1.7434	12.1639	3.5072	0.1254	0.8750	0.1598
77	Ni	3.4637	6.9829	3.4782	0.2492	0.5023	0.1585
78	Ni	5.2215	8.7095	3.5065	0.3756	0.6265	0.1597
79	Ni	3.4767	10.4098	3.5308	0.2501	0.7488	0.1609
80	Ni	5.2123	12.1644	3.5047	0.3749	0.8750	0.1597
81	Ni	6.9471	13.9005	3.5008	0.4997	0.9999	0.1595
82	Ni	8.7010	1.7483	3.5049	0.6259	0.1258	0.1597
83	Ni	6.9555	3.4648	3.5038	0.5003	0.2492	0.1596
84	Ni	8.6872	5.2198	3.5337	0.6249	0.3755	0.1610
85	Ni	10.4308	13.8828	3.5186	0.7503	0.9986	0.1603
86	Ni	12.1596	1.7387	3.5034	0.8747	0.1251	0.1596
87	Ni	10.4297	3.5122	3.5341	0.7502	0.2526	0.1610
88	Ni	12.1360	5.2198	3.5540	0.8730	0.3755	0.1619
89	Ni	6.9240	6.9461	3.5014	0.4981	0.4997	0.1595

90	Ni	8.6842	8.6824	3.4600	0.6247	0.6245	0.1576
91	Ni	6.9734	10.4226	3.5211	0.5016	0.7497	0.1604
92	Ni	8.6893	12.1813	3.4908	0.6250	0.8762	0.1590
93	Ni	10.4287	6.9211	3.5500	0.7502	0.4978	0.1617
94	Ni	12.1877	8.6736	3.4888	0.8767	0.6239	0.1589
95	Ni	10.4402	10.4524	3.4271	0.7510	0.7519	0.1561
96	Ni	12.1711	12.1725	3.5045	0.8755	0.8756	0.1597
97	Ni	0.0059	1.7365	5.2241	0.0004	0.1249	0.2380
98	Ni	1.7330	13.8988	5.2241	0.1247	0.9998	0.2380
99	Ni	0.0258	5.1827	5.2074	0.0019	0.3728	0.2372
100	Ni	1.7474	3.4694	5.2163	0.1257	0.2496	0.2376
101	Ni	3.4609	1.7234	5.2315	0.2490	0.1240	0.2383
102	Ni	5.2118	13.9001	5.2264	0.3749	0.9999	0.2381
103	Ni	3.4241	5.1880	5.0728	0.2463	0.3732	0.2311
104	Ni	5.1781	3.4246	5.3588	0.3725	0.2463	0.2441
105	Ni	0.0369	8.7098	5.2039	0.0027	0.6265	0.2371
106	Ni	1.7133	6.9230	5.1807	0.1232	0.4980	0.2360
107	Ni	0.0002	12.1745	5.2213	0.0000	0.8757	0.2379
108	Ni	1.7265	10.4551	5.2251	0.1242	0.7521	0.2380
109	Ni	3.4278	8.7405	5.3794	0.2466	0.6287	0.2451
110	Ni	5.1593	6.9801	5.2226	0.3711	0.5021	0.2379
111	Ni	3.4836	12.1606	5.2317	0.2506	0.8747	0.2383
112	Ni	5.2388	10.4438	5.2138	0.3768	0.7512	0.2375
113	Ni	6.9629	1.7022	5.1995	0.5009	0.1224	0.2369
114	Ni	8.6862	13.9002	5.2131	0.6248	0.9999	0.2375
115	Ni	6.8687	5.2138	5.2017	0.4941	0.3750	0.2370
116	Ni	8.6833	3.4344	5.2775	0.6246	0.2470	0.2404
117	Ni	10.4444	1.7102	5.2085	0.7513	0.1230	0.2373
118	Ni	12.1810	0.0081	5.2177	0.8762	0.0006	0.2377
119	Ni	10.4452	5.2957	5.7995	0.7513	0.3809	0.2642
120	Ni	12.1798	3.4672	5.2201	0.8761	0.2494	0.2378
121	Ni	6.9766	8.7325	5.2608	0.5018	0.6282	0.2397
122	Ni	8.6839	6.9549	5.2360	0.6247	0.5003	0.2385
123	Ni	6.9499	12.1676	5.2242	0.4999	0.8752	0.2380
124	Ni	8.7084	10.4643	5.2139	0.6264	0.7527	0.2375
125	Ni	10.4350	8.7135	5.1069	0.7506	0.6268	0.2326
126	Ni	12.2156	6.9777	5.3199	0.8787	0.5019	0.2424
127	Ni	10.4414	12.1926	5.2854	0.7511	0.8770	0.2408
128	Ni	12.1772	10.4279	5.2702	0.8759	0.7501	0.2401
129	O	6.0228	4.4084	6.8264	0.4332	0.3171	0.3110
130	O	4.1835	7.6811	6.7980	0.3009	0.5525	0.3097
131	O	5.9016	6.5538	8.7878	0.4245	0.4714	0.4003
132	O	7.8201	7.7502	6.8597	0.5625	0.5575	0.3125
133	O	10.4175	10.4417	6.2910	0.7494	0.7511	0.2866
134	O	10.1092	6.6777	8.2299	0.7272	0.4803	0.3749
135	O	7.8282	9.5948	8.9621	0.5631	0.6902	0.4083
136	Ce	5.9863	8.7755	8.0486	0.4306	0.6312	0.3667
137	Ce	4.2089	5.5562	7.6570	0.3028	0.3997	0.3488
138	Ce	7.8682	5.5090	7.8415	0.5660	0.3963	0.3572

139 Sm	9.4775	8.9951	7.8306	0.6817	0.6470	0.3567
140 C	10.9649	6.3905	7.2604	0.7887	0.4597	0.3308
141 O	12.1409	6.8775	7.3188	0.8733	0.4947	0.3334
142 O	9.4099	4.0945	6.8151	0.6769	0.2945	0.3105

/db/jmorales/CoNi-alloy/Profiles/CoNi-Ce_3SmO_7/O-coverage/CO-O-O-coverage/CO-O-CoNi-Ce_3SmO_7-2O

a =			13.9033384264				
b =			13.9033384264				
c =			21.9516692133				
alpha =			90.0				
beta =			90.0				
gamma =			90.0				
	Atom	X	Y	Z	X	Y	Z
1	Ni	0.0000	0.0000	0.0000	0.0000	0.0000	0.0000
2	Ni	0.0000	3.4758	0.0000	0.0000	0.2500	0.0000
3	Ni	3.4758	0.0000	0.0000	0.2500	0.0000	0.0000
4	Ni	3.4758	3.4758	0.0000	0.2500	0.2500	0.0000
5	Ni	0.0000	6.9517	0.0000	0.0000	0.5000	0.0000
6	Ni	0.0000	10.4275	0.0000	0.0000	0.7500	0.0000
7	Ni	3.4758	6.9517	0.0000	0.2500	0.5000	0.0000
8	Ni	3.4758	10.4275	0.0000	0.2500	0.7500	0.0000
9	Ni	6.9517	0.0000	0.0000	0.5000	0.0000	0.0000
10	Ni	6.9517	3.4758	0.0000	0.5000	0.2500	0.0000
11	Ni	10.4275	0.0000	0.0000	0.7500	0.0000	0.0000
12	Ni	10.4275	3.4758	0.0000	0.7500	0.2500	0.0000
13	Ni	6.9517	6.9517	0.0000	0.5000	0.5000	0.0000
14	Ni	6.9517	10.4275	0.0000	0.5000	0.7500	0.0000
15	Ni	10.4275	6.9517	0.0000	0.7500	0.5000	0.0000
16	Ni	10.4275	10.4275	0.0000	0.7500	0.7500	0.0000
17	Ni	0.0000	1.7379	1.7379	0.0000	0.1250	0.0792
18	Ni	0.0000	5.2138	1.7379	0.0000	0.3750	0.0792
19	Ni	3.4758	1.7379	1.7379	0.2500	0.1250	0.0792
20	Ni	3.4758	5.2138	1.7379	0.2500	0.3750	0.0792
21	Ni	0.0000	8.6896	1.7379	0.0000	0.6250	0.0792
22	Ni	0.0000	12.1654	1.7379	0.0000	0.8750	0.0792
23	Ni	3.4758	8.6896	1.7379	0.2500	0.6250	0.0792
24	Ni	3.4758	12.1654	1.7379	0.2500	0.8750	0.0792
25	Ni	6.9517	1.7379	1.7379	0.5000	0.1250	0.0792
26	Ni	6.9517	5.2138	1.7379	0.5000	0.3750	0.0792
27	Ni	10.4275	1.7379	1.7379	0.7500	0.1250	0.0792
28	Ni	10.4275	5.2138	1.7379	0.7500	0.3750	0.0792
29	Ni	6.9517	8.6896	1.7379	0.5000	0.6250	0.0792
30	Ni	6.9517	12.1654	1.7379	0.5000	0.8750	0.0792
31	Ni	10.4275	8.6896	1.7379	0.7500	0.6250	0.0792
32	Ni	10.4275	12.1654	1.7379	0.7500	0.8750	0.0792
33	Ni	13.8962	13.8946	3.4515	0.9995	0.9994	0.1572
34	Ni	0.0053	3.4536	3.5161	0.0004	0.2484	0.1602
35	Ni	3.4758	13.8944	3.4402	0.2500	0.9994	0.1567
36	Ni	3.4603	3.4308	3.4798	0.2489	0.2468	0.1585
37	Ni	0.0062	6.9609	3.5098	0.0004	0.5007	0.1599
38	Ni	13.8810	10.4575	3.5082	0.9984	0.7522	0.1598
39	Ni	3.4695	6.9573	3.4892	0.2495	0.5004	0.1589

40	Ni	3.5018	10.4279	3.5373	0.2519	0.7500	0.1611
41	Ni	6.9558	0.0144	3.4653	0.5003	0.0010	0.1579
42	Ni	6.9513	3.4932	3.5199	0.5000	0.2512	0.1603
43	Ni	10.4317	0.0158	3.4486	0.7503	0.0011	0.1571
44	Ni	10.4319	3.4752	3.5115	0.7503	0.2500	0.1600
45	Ni	6.9548	6.9573	3.4973	0.5002	0.5004	0.1593
46	Ni	6.9698	10.4392	3.4839	0.5013	0.7508	0.1587
47	Ni	10.4399	6.9363	3.4864	0.7509	0.4989	0.1588
48	Ni	10.4207	10.4379	3.5036	0.7495	0.7507	0.1596
49	Ni	13.8971	1.7439	5.2525	0.9996	0.1254	0.2393
50	Ni	0.0171	5.2088	5.2115	0.0012	0.3746	0.2374
51	Ni	3.4892	1.7412	5.2539	0.2510	0.1252	0.2393
52	Ni	3.4454	5.1686	5.0809	0.2478	0.3718	0.2315
53	Ni	13.8752	8.7282	5.1884	0.9980	0.6278	0.2364
54	Ni	13.9006	12.1508	5.2639	0.9998	0.8740	0.2398
55	Ni	3.4498	8.6760	5.2312	0.2481	0.6240	0.2383
56	Ni	3.4939	12.1376	5.2686	0.2513	0.8730	0.2400
57	Ni	6.9171	1.7621	5.2429	0.4975	0.1267	0.2388
58	Ni	6.9683	5.1613	5.3631	0.5012	0.3712	0.2443
59	Ni	10.4529	1.8064	5.2530	0.7518	0.1299	0.2393
60	Ni	10.4253	5.2127	5.2120	0.7498	0.3749	0.2374
61	Ni	6.9716	8.6967	5.2155	0.5014	0.6255	0.2376
62	Ni	7.0369	12.2532	5.2459	0.5061	0.8813	0.2390
63	Ni	10.4407	8.6653	5.1848	0.7509	0.6233	0.2362
64	Ni	10.3407	12.1695	5.3565	0.7438	0.8753	0.2440
65	Co	1.7379	1.7379	0.0000	0.1250	0.1250	0.0000
66	Co	1.7379	5.2138	0.0000	0.1250	0.3750	0.0000
67	Co	5.2138	1.7379	0.0000	0.3750	0.1250	0.0000
68	Co	5.2138	5.2138	0.0000	0.3750	0.3750	0.0000
69	Co	1.7379	8.6896	0.0000	0.1250	0.6250	0.0000
70	Co	1.7379	12.1654	0.0000	0.1250	0.8750	0.0000
71	Co	5.2138	8.6896	0.0000	0.3750	0.6250	0.0000
72	Co	5.2138	12.1654	0.0000	0.3750	0.8750	0.0000
73	Co	8.6896	1.7379	0.0000	0.6250	0.1250	0.0000
74	Co	8.6896	5.2138	0.0000	0.6250	0.3750	0.0000
75	Co	12.1654	1.7379	0.0000	0.8750	0.1250	0.0000
76	Co	12.1654	5.2138	0.0000	0.8750	0.3750	0.0000
77	Co	8.6896	8.6896	0.0000	0.6250	0.6250	0.0000
78	Co	8.6896	12.1654	0.0000	0.6250	0.8750	0.0000
79	Co	12.1654	8.6896	0.0000	0.8750	0.6250	0.0000
80	Co	12.1654	12.1654	0.0000	0.8750	0.8750	0.0000
81	Co	1.7379	0.0000	1.7379	0.1250	0.0000	0.0792
82	Co	1.7379	3.4758	1.7379	0.1250	0.2500	0.0792
83	Co	5.2138	0.0000	1.7379	0.3750	0.0000	0.0792
84	Co	5.2138	3.4758	1.7379	0.3750	0.2500	0.0792
85	Co	1.7379	6.9517	1.7379	0.1250	0.5000	0.0792
86	Co	1.7379	10.4275	1.7379	0.1250	0.7500	0.0792
87	Co	5.2138	6.9517	1.7379	0.3750	0.5000	0.0792
88	Co	5.2138	10.4275	1.7379	0.3750	0.7500	0.0792

89	Co	8.6896	0.0000	1.7379	0.6250	0.0000	0.0792
90	Co	8.6896	3.4758	1.7379	0.6250	0.2500	0.0792
91	Co	12.1654	0.0000	1.7379	0.8750	0.0000	0.0792
92	Co	12.1654	3.4758	1.7379	0.8750	0.2500	0.0792
93	Co	8.6896	6.9517	1.7379	0.6250	0.5000	0.0792
94	Co	8.6896	10.4275	1.7379	0.6250	0.7500	0.0792
95	Co	12.1654	6.9517	1.7379	0.8750	0.5000	0.0792
96	Co	12.1654	10.4275	1.7379	0.8750	0.7500	0.0792
97	Co	1.7341	1.7179	3.5529	0.1247	0.1236	0.1619
98	Co	1.7040	5.2298	3.5183	0.1226	0.3762	0.1603
99	Co	5.2106	1.7323	3.5248	0.3748	0.1246	0.1606
100	Co	5.2350	5.1891	3.5118	0.3765	0.3732	0.1600
101	Co	1.7274	8.6953	3.5271	0.1242	0.6254	0.1607
102	Co	1.7444	12.1735	3.5461	0.1255	0.8756	0.1615
103	Co	5.2004	8.6823	3.5555	0.3740	0.6245	0.1620
104	Co	5.2396	12.1576	3.5678	0.3769	0.8744	0.1625
105	Co	8.6851	1.7508	3.5498	0.6247	0.1259	0.1617
106	Co	8.6700	5.2073	3.5660	0.6236	0.3745	0.1624
107	Co	12.1716	1.7414	3.5434	0.8754	0.1253	0.1614
108	Co	12.1760	5.2129	3.5530	0.8758	0.3749	0.1619
109	Co	8.6876	8.6641	3.5135	0.6249	0.6232	0.1601
110	Co	8.7116	12.1900	3.4800	0.6266	0.8768	0.1585
111	Co	12.1625	8.6915	3.5398	0.8748	0.6251	0.1613
112	Co	12.1261	12.1818	3.5906	0.8722	0.8762	0.1636
113	Co	1.7565	13.8924	5.3632	0.1263	0.9992	0.2443
114	Co	1.7425	3.4838	5.2061	0.1253	0.2506	0.2372
115	Co	5.2459	13.8946	5.3315	0.3773	0.9994	0.2429
116	Co	5.1631	3.4584	5.1835	0.3714	0.2487	0.2361
117	Co	1.7103	6.9596	5.3171	0.1230	0.5006	0.2422
118	Co	1.7252	10.4550	5.1698	0.1241	0.7520	0.2355
119	Co	5.2442	6.8743	5.1717	0.3772	0.4944	0.2356
120	Co	5.2372	10.4331	5.2311	0.3767	0.7504	0.2383
121	Co	8.6758	0.1224	5.3480	0.6240	0.0088	0.2436
122	Co	8.6943	3.4758	5.2173	0.6253	0.2500	0.2377
123	Co	12.1345	13.8868	5.3588	0.8728	0.9988	0.2441
124	Co	12.1850	3.5057	5.2164	0.8764	0.2522	0.2376
125	Co	8.7196	6.9661	5.1828	0.6272	0.5010	0.2361
126	Co	8.7414	10.3482	5.1939	0.6287	0.7443	0.2366
127	Co	12.1748	6.9444	5.2183	0.8757	0.4995	0.2377
128	Co	12.1467	10.4272	5.2097	0.8737	0.7500	0.2373
129	O	5.9300	6.0552	6.7912	0.4265	0.4355	0.3094
130	O	2.6242	7.7783	6.8160	0.1887	0.5595	0.3105
131	O	4.8042	7.7766	8.7923	0.3455	0.5593	0.4005
132	O	6.0597	9.6826	6.8282	0.4358	0.6964	0.3111
133	O	7.8000	9.6322	9.1096	0.5610	0.6928	0.4150
134	O	4.9367	11.3413	8.8899	0.3551	0.8157	0.4050
135	O	8.5600	11.6827	6.4155	0.6157	0.8403	0.2923
136	Ce	3.8372	9.7794	8.0125	0.2760	0.7034	0.3650
137	Ce	3.8242	6.0736	7.7136	0.2751	0.4368	0.3514

138	Ce	7.0687	7.8996	8.1072	0.5084	0.5682	0.3693
139	Sm	6.9917	11.4129	8.2147	0.5029	0.8209	0.3742
140	C	6.8879	0.1306	6.3577	0.4954	0.0094	0.2896
141	O	6.9960	0.0257	7.6014	0.5032	0.0018	0.3463
142	O	3.5204	13.8964	6.1675	0.2532	0.9995	0.2810
143	O	10.4095	0.1225	6.1994	0.7487	0.0088	0.2824
144	O	13.9023	13.8991	6.1867	0.9999	0.9997	0.2818

/db/jmorales/CoNi-alloy/Profiles/CoNi-Ce_3SmO_7/O

a =			13.9033384264				
b =			13.9033384264				
c =			21.9516692133				
alpha =			90.0				
beta =			90.0				
gamma =			90.0				
	Atom	X	Y	Z	X	Y	Z
1	Ni	0.0000	0.0000	0.0000	0.0000	0.0000	0.0000
2	Ni	0.0000	3.4758	0.0000	0.0000	0.2500	0.0000
3	Ni	3.4758	0.0000	0.0000	0.2500	0.0000	0.0000
4	Ni	3.4758	3.4758	0.0000	0.2500	0.2500	0.0000
5	Ni	0.0000	6.9517	0.0000	0.0000	0.5000	0.0000
6	Ni	0.0000	10.4275	0.0000	0.0000	0.7500	0.0000
7	Ni	3.4758	6.9517	0.0000	0.2500	0.5000	0.0000
8	Ni	3.4758	10.4275	0.0000	0.2500	0.7500	0.0000
9	Ni	6.9517	0.0000	0.0000	0.5000	0.0000	0.0000
10	Ni	6.9517	3.4758	0.0000	0.5000	0.2500	0.0000
11	Ni	10.4275	0.0000	0.0000	0.7500	0.0000	0.0000
12	Ni	10.4275	3.4758	0.0000	0.7500	0.2500	0.0000
13	Ni	6.9517	6.9517	0.0000	0.5000	0.5000	0.0000
14	Ni	6.9517	10.4275	0.0000	0.5000	0.7500	0.0000
15	Ni	10.4275	6.9517	0.0000	0.7500	0.5000	0.0000
16	Ni	10.4275	10.4275	0.0000	0.7500	0.7500	0.0000
17	Ni	0.0000	1.7379	1.7379	0.0000	0.1250	0.0792
18	Ni	0.0000	5.2138	1.7379	0.0000	0.3750	0.0792
19	Ni	3.4758	1.7379	1.7379	0.2500	0.1250	0.0792
20	Ni	3.4758	5.2138	1.7379	0.2500	0.3750	0.0792
21	Ni	0.0000	8.6896	1.7379	0.0000	0.6250	0.0792
22	Ni	0.0000	12.1654	1.7379	0.0000	0.8750	0.0792
23	Ni	3.4758	8.6896	1.7379	0.2500	0.6250	0.0792
24	Ni	3.4758	12.1654	1.7379	0.2500	0.8750	0.0792
25	Ni	6.9517	1.7379	1.7379	0.5000	0.1250	0.0792
26	Ni	6.9517	5.2138	1.7379	0.5000	0.3750	0.0792
27	Ni	10.4275	1.7379	1.7379	0.7500	0.1250	0.0792
28	Ni	10.4275	5.2138	1.7379	0.7500	0.3750	0.0792
29	Ni	6.9517	8.6896	1.7379	0.5000	0.6250	0.0792
30	Ni	6.9517	12.1654	1.7379	0.5000	0.8750	0.0792
31	Ni	10.4275	8.6896	1.7379	0.7500	0.6250	0.0792
32	Ni	10.4275	12.1654	1.7379	0.7500	0.8750	0.0792
33	Ni	0.0109	13.9000	3.5076	0.0008	0.9998	0.1598
34	Ni	-0.0005	3.4747	3.5013	-0.0000	0.2499	0.1595
35	Ni	3.4764	13.8991	3.4457	0.2500	0.9997	0.1570
36	Ni	3.4641	3.4331	3.4867	0.2492	0.2469	0.1588
37	Ni	0.0009	6.9608	3.5144	0.0001	0.5007	0.1601
38	Ni	13.8897	10.4339	3.4918	0.9990	0.7505	0.1591
39	Ni	3.4735	6.9576	3.4946	0.2498	0.5004	0.1592
40	Ni	3.4923	10.4181	3.5421	0.2512	0.7493	0.1614

41	Ni	6.9427	0.0153	3.4969	0.4994	0.0011	0.1593
42	Ni	6.9573	3.4902	3.5202	0.5004	0.2510	0.1604
43	Ni	10.4258	0.0036	3.5047	0.7499	0.0003	0.1597
44	Ni	10.4278	3.4751	3.5048	0.7500	0.2499	0.1597
45	Ni	6.9655	6.9644	3.4992	0.5010	0.5009	0.1594
46	Ni	6.9674	10.4377	3.4786	0.5011	0.7507	0.1585
47	Ni	10.4360	6.9469	3.4895	0.7506	0.4997	0.1590
48	Ni	10.4299	10.4417	3.4990	0.7502	0.7510	0.1594
49	Ni	0.0084	1.7413	5.2073	0.0006	0.1252	0.2372
50	Ni	0.0002	5.2138	5.2156	0.0000	0.3750	0.2376
51	Ni	3.4603	1.7607	5.2625	0.2489	0.1266	0.2397
52	Ni	3.4501	5.1685	5.0869	0.2481	0.3717	0.2317
53	Ni	-0.0301	8.7195	5.1927	-0.0022	0.6272	0.2366
54	Ni	0.0008	12.1644	5.2122	0.0001	0.8749	0.2374
55	Ni	3.4499	8.6756	5.2467	0.2481	0.6240	0.2390
56	Ni	3.4765	12.1232	5.2812	0.2500	0.8720	0.2406
57	Ni	6.9522	1.7401	5.2036	0.5000	0.1252	0.2370
58	Ni	6.9881	5.1688	5.3559	0.5026	0.3718	0.2440
59	Ni	10.4313	1.7294	5.2052	0.7503	0.1244	0.2371
60	Ni	10.4265	5.2150	5.2125	0.7499	0.3751	0.2375
61	Ni	7.0091	8.7100	5.2195	0.5041	0.6265	0.2378
62	Ni	6.9481	12.2049	5.1602	0.4997	0.8778	0.2351
63	Ni	10.4222	8.6976	5.2051	0.7496	0.6256	0.2371
64	Ni	10.4486	12.1991	5.2581	0.7515	0.8774	0.2395
65	Co	1.7379	1.7379	0.0000	0.1250	0.1250	0.0000
66	Co	1.7379	5.2138	0.0000	0.1250	0.3750	0.0000
67	Co	5.2138	1.7379	0.0000	0.3750	0.1250	0.0000
68	Co	5.2138	5.2138	0.0000	0.3750	0.3750	0.0000
69	Co	1.7379	8.6896	0.0000	0.1250	0.6250	0.0000
70	Co	1.7379	12.1654	0.0000	0.1250	0.8750	0.0000
71	Co	5.2138	8.6896	0.0000	0.3750	0.6250	0.0000
72	Co	5.2138	12.1654	0.0000	0.3750	0.8750	0.0000
73	Co	8.6896	1.7379	0.0000	0.6250	0.1250	0.0000
74	Co	8.6896	5.2138	0.0000	0.6250	0.3750	0.0000
75	Co	12.1654	1.7379	0.0000	0.8750	0.1250	0.0000
76	Co	12.1654	5.2138	0.0000	0.8750	0.3750	0.0000
77	Co	8.6896	8.6896	0.0000	0.6250	0.6250	0.0000
78	Co	8.6896	12.1654	0.0000	0.6250	0.8750	0.0000
79	Co	12.1654	8.6896	0.0000	0.8750	0.6250	0.0000
80	Co	12.1654	12.1654	0.0000	0.8750	0.8750	0.0000
81	Co	1.7379	0.0000	1.7379	0.1250	0.0000	0.0792
82	Co	1.7379	3.4758	1.7379	0.1250	0.2500	0.0792
83	Co	5.2138	0.0000	1.7379	0.3750	0.0000	0.0792
84	Co	5.2138	3.4758	1.7379	0.3750	0.2500	0.0792
85	Co	1.7379	6.9517	1.7379	0.1250	0.5000	0.0792
86	Co	1.7379	10.4275	1.7379	0.1250	0.7500	0.0792
87	Co	5.2138	6.9517	1.7379	0.3750	0.5000	0.0792
88	Co	5.2138	10.4275	1.7379	0.3750	0.7500	0.0792
89	Co	8.6896	0.0000	1.7379	0.6250	0.0000	0.0792

90	Co	8.6896	3.4758	1.7379	0.6250	0.2500	0.0792
91	Co	12.1654	0.0000	1.7379	0.8750	0.0000	0.0792
92	Co	12.1654	3.4758	1.7379	0.8750	0.2500	0.0792
93	Co	8.6896	6.9517	1.7379	0.6250	0.5000	0.0792
94	Co	8.6896	10.4275	1.7379	0.6250	0.7500	0.0792
95	Co	12.1654	6.9517	1.7379	0.8750	0.5000	0.0792
96	Co	12.1654	10.4275	1.7379	0.8750	0.7500	0.0792
97	Co	1.7338	1.7289	3.5504	0.1247	0.1244	0.1617
98	Co	1.7050	5.2370	3.5280	0.1226	0.3767	0.1607
99	Co	5.2120	1.7283	3.5538	0.3749	0.1243	0.1619
100	Co	5.2409	5.1900	3.5170	0.3770	0.3733	0.1602
101	Co	1.7297	8.6846	3.5312	0.1244	0.6246	0.1609
102	Co	1.7454	12.1696	3.5475	0.1255	0.8753	0.1616
103	Co	5.2061	8.6776	3.5612	0.3744	0.6241	0.1622
104	Co	5.2106	12.1631	3.5639	0.3748	0.8748	0.1624
105	Co	8.6887	1.7312	3.5517	0.6249	0.1245	0.1618
106	Co	8.6747	5.2046	3.5656	0.6239	0.3743	0.1624
107	Co	12.1697	1.7382	3.5527	0.8753	0.1250	0.1618
108	Co	12.1681	5.2129	3.5585	0.8752	0.3749	0.1621
109	Co	8.6947	8.7026	3.5390	0.6254	0.6259	0.1612
110	Co	8.7042	12.1867	3.4598	0.6261	0.8765	0.1576
111	Co	12.1533	8.6950	3.5475	0.8741	0.6254	0.1616
112	Co	12.1552	12.1747	3.5721	0.8743	0.8757	0.1627
113	Co	1.7549	13.8912	5.2858	0.1262	0.9991	0.2408
114	Co	1.7299	3.4951	5.2127	0.1244	0.2514	0.2375
115	Co	5.1856	0.0022	5.2783	0.3730	0.0002	0.2404
116	Co	5.1887	3.4481	5.1939	0.3732	0.2480	0.2366
117	Co	1.7096	6.9633	5.3265	0.1230	0.5008	0.2426
118	Co	1.7101	10.4499	5.1830	0.1230	0.7516	0.2361
119	Co	5.2493	6.8752	5.1712	0.3776	0.4945	0.2356
120	Co	5.2367	10.4165	5.2227	0.3767	0.7492	0.2379
121	Co	8.6757	-0.0002	5.2919	0.6240	-0.0000	0.2411
122	Co	8.7111	3.4519	5.2258	0.6265	0.2483	0.2381
123	Co	12.1900	0.0177	5.2175	0.8768	0.0013	0.2377
124	Co	12.1672	3.4694	5.2234	0.8751	0.2495	0.2380
125	Co	8.7292	6.9695	5.1766	0.6278	0.5013	0.2358
126	Co	8.7362	10.4882	5.2145	0.6284	0.7544	0.2375
127	Co	12.1600	6.9570	5.2207	0.8746	0.5004	0.2378
128	Co	12.1625	10.4321	5.2176	0.8748	0.7503	0.2377
129	O	5.9023	6.0068	6.7881	0.4245	0.4320	0.3092
130	O	2.6228	7.8231	6.8380	0.1886	0.5627	0.3115
131	O	4.8607	7.7756	8.7868	0.3496	0.5593	0.4003
132	O	6.1157	9.6175	6.8154	0.4399	0.6917	0.3105
133	O	8.0032	9.5147	8.9462	0.5756	0.6843	0.4075
134	O	5.1779	11.3413	8.8691	0.3724	0.8157	0.4040
135	O	8.6168	12.1672	6.3294	0.6198	0.8751	0.2883
136	Ce	3.9669	9.8138	8.0235	0.2853	0.7059	0.3655
137	Ce	3.7948	6.1038	7.7106	0.2729	0.4390	0.3513
138	Ce	7.1136	7.7678	8.0440	0.5116	0.5587	0.3664

139 Sm	7.1850	11.2638	8.0766	0.5168	0.8102	0.3679
140 O	3.4666	13.8978	6.2059	0.2493	0.9996	0.2827

/db/jmorales/CoNi-alloy/Profiles/Sm(surf)$Ce_{26}O_{54}$/O

a =	11.6082000732
b =	11.608200073110298
c =	22.898399353
alpha =	90.0
beta =	90.0
gamma =	120.0

	Atom	X	Y	Z	X	Y	Z
1	Sm	2.0485	3.2820	7.0654	0.3397	0.3265	0.3086
2	Ce	1.9347	1.1170	0.7898	0.2222	0.1111	0.0345
3	Ce	0.0000	4.4680	0.7898	0.2222	0.4444	0.0345
4	Ce	-1.9347	7.8190	0.7898	0.2222	0.7778	0.0345
5	Ce	5.8041	1.1170	0.7898	0.5556	0.1111	0.0345
6	Ce	3.8694	4.4680	0.7898	0.5556	0.4444	0.0345
7	Ce	1.9347	7.8190	0.7898	0.5556	0.7778	0.0345
8	Ce	9.6735	1.1170	0.7898	0.8889	0.1111	0.0345
9	Ce	7.7388	4.4680	0.7898	0.8889	0.4444	0.0345
10	Ce	5.8041	7.8190	0.7898	0.8889	0.7778	0.0345
11	Ce	0.0045	2.2405	3.9732	0.1118	0.2229	0.1735
12	Ce	-1.9202	5.5797	3.9647	0.1121	0.5550	0.1731
13	Ce	-3.8623	8.9362	3.9485	0.1117	0.8889	0.1724
14	Ce	3.8664	2.2367	3.9436	0.4443	0.2225	0.1722
15	Ce	1.9252	5.5775	3.9797	0.4433	0.5548	0.1738
16	Ce	-0.0000	8.9439	3.9297	0.4448	0.8897	0.1716
17	Ce	7.7318	2.2305	3.9271	0.7770	0.2219	0.1715
18	Ce	5.8071	5.5772	3.9504	0.7776	0.5548	0.1725
19	Ce	3.8652	8.9380	3.9576	0.7775	0.8891	0.1728
20	Ce	0.0183	-0.0062	7.1025	0.0013	-0.0006	0.3102
21	Ce	9.5714	3.2654	7.0703	0.9869	0.3248	0.3088
22	Ce	-3.8606	6.6982	7.1014	0.0006	0.6663	0.3101
23	Ce	3.8522	0.0115	7.0996	0.3324	0.0011	0.3100
24	Ce	0.0205	6.8509	7.0925	0.3425	0.6815	0.3097
25	Ce	7.7384	0.0080	7.1093	0.6670	0.0008	0.3105
26	Ce	5.7915	3.3598	7.1014	0.6660	0.3342	0.3101
27	Ce	3.8817	6.6899	7.1132	0.6671	0.6655	0.3106
28	O	0.0000	0.0000	0.0000	0.0000	0.0000	0.0000
29	O	-1.9347	3.3510	0.0000	-0.0000	0.3333	0.0000
30	O	-3.8694	6.7020	0.0000	-0.0000	0.6667	0.0000
31	O	3.8694	0.0000	0.0000	0.3333	0.0000	0.0000
32	O	1.9347	3.3510	0.0000	0.3333	0.3333	0.0000
33	O	0.0000	6.7020	0.0000	0.3333	0.6667	0.0000
34	O	7.7388	0.0000	0.0000	0.6667	0.0000	0.0000
35	O	5.8041	3.3510	0.0000	0.6667	0.3333	0.0000
36	O	3.8694	6.7020	0.0000	0.6667	0.6667	0.0000
37	O	-0.0000	2.2340	1.5798	0.1111	0.2222	0.0690
38	O	-1.9347	5.5850	1.5798	0.1111	0.5556	0.0690
39	O	-3.8694	8.9360	1.5798	0.1111	0.8889	0.0690
40	O	3.8694	2.2340	1.5798	0.4444	0.2222	0.0690

41	O	1.9347	5.5850	1.5798	0.4444	0.5556	0.0690
42	O	-0.0000	8.9360	1.5798	0.4444	0.8889	0.0690
43	O	7.7388	2.2340	1.5798	0.7778	0.2222	0.0690
44	O	5.8041	5.5850	1.5798	0.7778	0.5556	0.0690
45	O	3.8694	8.9360	1.5798	0.7778	0.8889	0.0690
46	O	1.9347	1.1170	3.1593	0.2222	0.1111	0.1380
47	O	0.0000	4.4680	3.1593	0.2222	0.4444	0.1380
48	O	-1.9347	7.8190	3.1593	0.2222	0.7778	0.1380
49	O	5.8041	1.1170	3.1593	0.5556	0.1111	0.1380
50	O	3.8694	4.4680	3.1593	0.5556	0.4444	0.1380
51	O	1.9347	7.8190	3.1593	0.5556	0.7778	0.1380
52	O	9.6735	1.1170	3.1593	0.8889	0.1111	0.1380
53	O	7.7388	4.4680	3.1593	0.8889	0.4444	0.1380
54	O	5.8041	7.8190	3.1593	0.8889	0.7778	0.1380
55	O	0.0055	0.0269	4.7698	0.0018	0.0027	0.2083
56	O	-1.9631	3.3466	4.7452	-0.0027	0.3329	0.2072
57	O	-3.8155	6.6690	4.7797	0.0030	0.6634	0.2087
58	O	3.8610	-0.0057	4.7475	0.3323	-0.0006	0.2073
59	O	1.9479	3.3481	4.7409	0.3343	0.3330	0.2070
60	O	-0.0075	6.7320	4.7403	0.3342	0.6697	0.2070
61	O	7.7466	-0.0039	4.7511	0.6671	-0.0004	0.2075
62	O	5.8059	3.3614	4.7476	0.6673	0.3344	0.2073
63	O	3.8407	6.6779	4.7860	0.6630	0.6643	0.2090
64	O	-0.0480	2.2960	6.3465	0.1101	0.2284	0.2772
65	O	-1.8053	5.5143	6.3917	0.1187	0.5485	0.2791
66	O	-3.8858	8.8997	6.2675	0.1079	0.8853	0.2737
67	O	3.9112	2.2109	6.2855	0.4469	0.2199	0.2745
68	O	1.8256	5.5840	6.3856	0.4350	0.5555	0.2789
69	O	0.0030	8.9684	6.2847	0.4463	0.8921	0.2745
70	O	7.7214	2.2214	6.2780	0.7757	0.2210	0.2742
71	O	5.8323	5.6107	6.2739	0.7815	0.5581	0.2740
72	O	3.8908	8.9303	6.2954	0.7793	0.8883	0.2749
73	O	1.9417	1.0046	7.8730	0.2172	0.0999	0.3438
74	O	-0.0036	4.1191	8.3995	0.2046	0.4097	0.3668
75	O	-1.9432	7.8590	7.8596	0.2235	0.7818	0.3432
76	O	5.8141	1.1219	7.8986	0.5567	0.1116	0.3449
77	O	3.9674	4.5164	7.8714	0.5664	0.4493	0.3438
78	O	1.9403	7.8873	7.8908	0.5594	0.7846	0.3446
79	O	9.6296	1.1053	7.8953	0.8845	0.1100	0.3448
80	O	7.7301	4.4498	7.8675	0.8872	0.4426	0.3436
81	O	5.8204	7.8052	7.8931	0.8896	0.7764	0.3447
82	O	-0.0532	5.3104	9.0387	0.2595	0.5282	0.3947

/db/jmorales/CoNi-alloy/Profiles/Ni-Ce_3SmO_7/O-coverage/TS-O-coverage/TS-Ni-Ce_3SmO_7-2O

a =			13.9019837372				
b =			13.9019837372				
c =			21.9509918687				
alpha =			90.0				
beta =			90.0				
gamma =			90.0				
	Atom	X	Y	Z	X	Y	Z
1	Ni	0.0000	0.0000	0.0000	0.0000	0.0000	0.0000
2	Ni	1.7377	1.7377	0.0000	0.1250	0.1250	0.0000
3	Ni	0.0000	3.4755	0.0000	0.0000	0.2500	0.0000
4	Ni	1.7377	5.2132	0.0000	0.1250	0.3750	0.0000
5	Ni	3.4755	0.0000	0.0000	0.2500	0.0000	0.0000
6	Ni	5.2132	1.7377	0.0000	0.3750	0.1250	0.0000
7	Ni	3.4755	3.4755	0.0000	0.2500	0.2500	0.0000
8	Ni	5.2132	5.2132	0.0000	0.3750	0.3750	0.0000
9	Ni	0.0000	6.9510	0.0000	0.0000	0.5000	0.0000
10	Ni	1.7377	8.6887	0.0000	0.1250	0.6250	0.0000
11	Ni	0.0000	10.4265	0.0000	0.0000	0.7500	0.0000
12	Ni	1.7377	12.1642	0.0000	0.1250	0.8750	0.0000
13	Ni	3.4755	6.9510	0.0000	0.2500	0.5000	0.0000
14	Ni	5.2132	8.6887	0.0000	0.3750	0.6250	0.0000
15	Ni	3.4755	10.4265	0.0000	0.2500	0.7500	0.0000
16	Ni	5.2132	12.1642	0.0000	0.3750	0.8750	0.0000
17	Ni	6.9510	0.0000	0.0000	0.5000	0.0000	0.0000
18	Ni	8.6887	1.7377	0.0000	0.6250	0.1250	0.0000
19	Ni	6.9510	3.4755	0.0000	0.5000	0.2500	0.0000
20	Ni	8.6887	5.2132	0.0000	0.6250	0.3750	0.0000
21	Ni	10.4265	0.0000	0.0000	0.7500	0.0000	0.0000
22	Ni	12.1642	1.7377	0.0000	0.8750	0.1250	0.0000
23	Ni	10.4265	3.4755	0.0000	0.7500	0.2500	0.0000
24	Ni	12.1642	5.2132	0.0000	0.8750	0.3750	0.0000
25	Ni	6.9510	6.9510	0.0000	0.5000	0.5000	0.0000
26	Ni	8.6887	8.6887	0.0000	0.6250	0.6250	0.0000
27	Ni	6.9510	10.4265	0.0000	0.5000	0.7500	0.0000
28	Ni	8.6887	12.1642	0.0000	0.6250	0.8750	0.0000
29	Ni	10.4265	6.9510	0.0000	0.7500	0.5000	0.0000
30	Ni	12.1642	8.6887	0.0000	0.8750	0.6250	0.0000
31	Ni	10.4265	10.4265	0.0000	0.7500	0.7500	0.0000
32	Ni	12.1642	12.1642	0.0000	0.8750	0.8750	0.0000
33	Ni	0.0000	1.7377	1.7377	0.0000	0.1250	0.0792
34	Ni	1.7377	0.0000	1.7377	0.1250	0.0000	0.0792
35	Ni	0.0000	5.2132	1.7377	0.0000	0.3750	0.0792
36	Ni	1.7377	3.4755	1.7377	0.1250	0.2500	0.0792
37	Ni	3.4755	1.7377	1.7377	0.2500	0.1250	0.0792
38	Ni	5.2132	0.0000	1.7377	0.3750	0.0000	0.0792
39	Ni	3.4755	5.2132	1.7377	0.2500	0.3750	0.0792

40	Ni	5.2132	3.4755	1.7377	0.3750	0.2500	0.0792
41	Ni	0.0000	8.6887	1.7377	0.0000	0.6250	0.0792
42	Ni	1.7377	6.9510	1.7377	0.1250	0.5000	0.0792
43	Ni	0.0000	12.1642	1.7377	0.0000	0.8750	0.0792
44	Ni	1.7377	10.4265	1.7377	0.1250	0.7500	0.0792
45	Ni	3.4755	8.6887	1.7377	0.2500	0.6250	0.0792
46	Ni	5.2132	6.9510	1.7377	0.3750	0.5000	0.0792
47	Ni	3.4755	12.1642	1.7377	0.2500	0.8750	0.0792
48	Ni	5.2132	10.4265	1.7377	0.3750	0.7500	0.0792
49	Ni	6.9510	1.7377	1.7377	0.5000	0.1250	0.0792
50	Ni	8.6887	0.0000	1.7377	0.6250	0.0000	0.0792
51	Ni	6.9510	5.2132	1.7377	0.5000	0.3750	0.0792
52	Ni	8.6887	3.4755	1.7377	0.6250	0.2500	0.0792
53	Ni	10.4265	1.7377	1.7377	0.7500	0.1250	0.0792
54	Ni	12.1642	0.0000	1.7377	0.8750	0.0000	0.0792
55	Ni	10.4265	5.2132	1.7377	0.7500	0.3750	0.0792
56	Ni	12.1642	3.4755	1.7377	0.8750	0.2500	0.0792
57	Ni	6.9510	8.6887	1.7377	0.5000	0.6250	0.0792
58	Ni	8.6887	6.9510	1.7377	0.6250	0.5000	0.0792
59	Ni	6.9510	12.1642	1.7377	0.5000	0.8750	0.0792
60	Ni	8.6887	10.4265	1.7377	0.6250	0.7500	0.0792
61	Ni	10.4265	8.6887	1.7377	0.7500	0.6250	0.0792
62	Ni	12.1642	6.9510	1.7377	0.8750	0.5000	0.0792
63	Ni	10.4265	12.1642	1.7377	0.7500	0.8750	0.0792
64	Ni	12.1642	10.4265	1.7377	0.8750	0.7500	0.0792
65	Ni	0.0150	0.0089	3.5070	0.0011	0.0006	0.1598
66	Ni	1.7383	1.7717	3.4589	0.1250	0.1274	0.1576
67	Ni	13.8833	3.4769	3.5104	0.9987	0.2501	0.1599
68	Ni	1.6930	5.1847	3.4601	0.1218	0.3729	0.1576
69	Ni	3.4695	0.0019	3.5108	0.2496	0.0001	0.1599
70	Ni	5.2051	1.7433	3.5457	0.3744	0.1254	0.1615
71	Ni	3.5049	3.4452	3.4788	0.2521	0.2478	0.1585
72	Ni	5.2410	5.2095	3.4716	0.3770	0.3747	0.1582
73	Ni	0.0006	6.9361	3.5134	0.0000	0.4989	0.1601
74	Ni	1.7538	8.6550	3.5470	0.1262	0.6226	0.1616
75	Ni	13.8979	10.4185	3.5189	0.9997	0.7494	0.1603
76	Ni	1.7436	12.1729	3.5160	0.1254	0.8756	0.1602
77	Ni	3.4765	6.9736	3.4645	0.2501	0.5016	0.1578
78	Ni	5.2313	8.7084	3.5000	0.3763	0.6264	0.1594
79	Ni	3.4664	10.3969	3.5264	0.2493	0.7479	0.1606
80	Ni	5.2073	12.1634	3.5016	0.3746	0.8749	0.1595
81	Ni	6.9384	13.8909	3.5030	0.4991	0.9992	0.1596
82	Ni	8.6903	1.7044	3.4892	0.6251	0.1226	0.1590
83	Ni	6.9512	3.4586	3.4986	0.5000	0.2488	0.1594
84	Ni	8.6638	5.2365	3.5091	0.6232	0.3767	0.1599
85	Ni	10.4431	13.8768	3.5217	0.7512	0.9982	0.1604
86	Ni	12.1819	1.7410	3.5234	0.8763	0.1252	0.1605
87	Ni	10.4157	3.4607	3.4858	0.7492	0.2489	0.1588
88	Ni	12.1510	5.2125	3.4921	0.8740	0.3749	0.1591

89	Ni	6.9458	6.9479	3.5082	0.4996	0.4998	0.1598
90	Ni	8.6968	8.6957	3.4682	0.6256	0.6255	0.1580
91	Ni	6.9766	10.4317	3.5121	0.5018	0.7504	0.1600
92	Ni	8.6952	12.1764	3.4922	0.6255	0.8759	0.1591
93	Ni	10.4204	6.9596	3.5194	0.7496	0.5006	0.1603
94	Ni	12.1867	8.6639	3.5039	0.8766	0.6232	0.1596
95	Ni	10.4506	10.4419	3.4314	0.7517	0.7511	0.1563
96	Ni	12.1801	12.1666	3.5036	0.8761	0.8752	0.1596
97	Ni	0.0417	1.7905	5.2816	0.0030	0.1288	0.2406
98	Ni	1.7374	13.8832	5.3009	0.1250	0.9986	0.2415
99	Ni	13.8261	5.1638	5.2995	0.9945	0.3714	0.2414
100	Ni	1.7522	3.5825	5.3985	0.1260	0.2577	0.2459
101	Ni	3.4499	1.7575	5.2850	0.2482	0.1264	0.2408
102	Ni	5.2019	13.8972	5.2246	0.3742	0.9997	0.2380
103	Ni	3.5318	5.1784	5.0488	0.2541	0.3725	0.2300
104	Ni	5.2596	3.4274	5.3615	0.3783	0.2465	0.2443
105	Ni	0.0346	8.6757	5.2123	0.0025	0.6241	0.2375
106	Ni	1.7336	6.8480	5.2921	0.1247	0.4926	0.2411
107	Ni	0.0035	12.1634	5.2122	0.0003	0.8749	0.2374
108	Ni	1.7300	10.4340	5.2214	0.1244	0.7505	0.2379
109	Ni	3.4510	8.7162	5.3763	0.2482	0.6270	0.2449
110	Ni	5.2126	6.9757	5.2038	0.3750	0.5018	0.2371
111	Ni	3.4814	12.1480	5.2224	0.2504	0.8738	0.2379
112	Ni	5.2272	10.4382	5.1950	0.3760	0.7508	0.2367
113	Ni	6.9809	1.6848	5.2062	0.5021	0.1212	0.2372
114	Ni	8.6877	13.8725	5.2194	0.6249	0.9979	0.2378
115	Ni	6.9564	5.2024	5.2746	0.5004	0.3742	0.2403
116	Ni	8.6952	3.4431	5.1640	0.6255	0.2477	0.2352
117	Ni	10.4080	1.6931	5.2072	0.7487	0.1218	0.2372
118	Ni	12.1990	0.0188	5.2154	0.8775	0.0014	0.2376
119	Ni	10.3697	5.1970	5.2463	0.7459	0.3738	0.2390
120	Ni	12.0715	3.4420	5.3114	0.8683	0.2476	0.2420
121	Ni	6.9859	8.7497	5.2398	0.5025	0.6294	0.2387
122	Ni	8.7117	6.9635	5.2744	0.6267	0.5009	0.2403
123	Ni	6.9389	12.1590	5.2239	0.4991	0.8746	0.2380
124	Ni	8.7263	10.4780	5.2065	0.6277	0.7537	0.2372
125	Ni	10.4629	8.7092	5.1719	0.7526	0.6265	0.2356
126	Ni	12.1444	6.9728	5.3596	0.8736	0.5016	0.2442
127	Ni	10.4684	12.1767	5.2832	0.7530	0.8759	0.2407
128	Ni	12.1954	10.4170	5.2779	0.8772	0.7493	0.2404
129	O	6.1212	4.3991	6.8068	0.4403	0.3164	0.3101
130	O	4.2435	7.6264	6.7580	0.3052	0.5486	0.3079
131	O	6.0674	6.6341	8.7891	0.4364	0.4772	0.4004
132	O	7.8719	7.9111	6.7972	0.5662	0.5691	0.3097
133	O	10.4366	10.3942	6.2652	0.7507	0.7477	0.2854
134	O	9.5153	7.1592	8.9697	0.6845	0.5150	0.4086
135	O	7.6545	9.9093	8.8708	0.5506	0.7128	0.4041
136	Ce	5.9641	8.8519	8.0426	0.4290	0.6367	0.3664
137	Ce	4.3647	5.5207	7.7415	0.3140	0.3971	0.3527

138	Ce	8.1751	5.7939	8.0813	0.5881	0.4168	0.3682
139	Sm	9.4371	9.1078	8.0773	0.6788	0.6551	0.3680
140	C	11.3515	4.5325	6.7430	0.8165	0.3260	0.3072
141	O	12.7243	5.7689	6.6384	0.9153	0.4150	0.3024
142	O	10.9757	4.3667	7.8817	0.7895	0.3141	0.3591
143	O	2.2407	5.2819	6.4139	0.1612	0.3799	0.2922
144	O	1.7394	1.6898	6.1840	0.1251	0.1215	0.2817

/db/jmorales/CoNi-alloy/Profiles/Ni-Ce_3SmO_7/O-coverage/TS-O-coverage/TS-Ni-Ce_3SmO_7-3O

a = 13.9019837372
b = 13.9019837372
c = 21.9509918687
alpha = 90.0
beta = 90.0
gamma = 90.0

	Atom	X	Y	Z	X	Y	Z
1	Ni	0.0000	0.0000	0.0000	0.0000	0.0000	0.0000
2	Ni	1.7377	1.7377	0.0000	0.1250	0.1250	0.0000
3	Ni	0.0000	3.4755	0.0000	0.0000	0.2500	0.0000
4	Ni	1.7377	5.2132	0.0000	0.1250	0.3750	0.0000
5	Ni	3.4755	0.0000	0.0000	0.2500	0.0000	0.0000
6	Ni	5.2132	1.7377	0.0000	0.3750	0.1250	0.0000
7	Ni	3.4755	3.4755	0.0000	0.2500	0.2500	0.0000
8	Ni	5.2132	5.2132	0.0000	0.3750	0.3750	0.0000
9	Ni	0.0000	6.9510	0.0000	0.0000	0.5000	0.0000
10	Ni	1.7377	8.6887	0.0000	0.1250	0.6250	0.0000
11	Ni	0.0000	10.4265	0.0000	0.0000	0.7500	0.0000
12	Ni	1.7377	12.1642	0.0000	0.1250	0.8750	0.0000
13	Ni	3.4755	6.9510	0.0000	0.2500	0.5000	0.0000
14	Ni	5.2132	8.6887	0.0000	0.3750	0.6250	0.0000
15	Ni	3.4755	10.4265	0.0000	0.2500	0.7500	0.0000
16	Ni	5.2132	12.1642	0.0000	0.3750	0.8750	0.0000
17	Ni	6.9510	0.0000	0.0000	0.5000	0.0000	0.0000
18	Ni	8.6887	1.7377	0.0000	0.6250	0.1250	0.0000
19	Ni	6.9510	3.4755	0.0000	0.5000	0.2500	0.0000
20	Ni	8.6887	5.2132	0.0000	0.6250	0.3750	0.0000
21	Ni	10.4265	0.0000	0.0000	0.7500	0.0000	0.0000
22	Ni	12.1642	1.7377	0.0000	0.8750	0.1250	0.0000
23	Ni	10.4265	3.4755	0.0000	0.7500	0.2500	0.0000
24	Ni	12.1642	5.2132	0.0000	0.8750	0.3750	0.0000
25	Ni	6.9510	6.9510	0.0000	0.5000	0.5000	0.0000
26	Ni	8.6887	8.6887	0.0000	0.6250	0.6250	0.0000
27	Ni	6.9510	10.4265	0.0000	0.5000	0.7500	0.0000
28	Ni	8.6887	12.1642	0.0000	0.6250	0.8750	0.0000
29	Ni	10.4265	6.9510	0.0000	0.7500	0.5000	0.0000
30	Ni	12.1642	8.6887	0.0000	0.8750	0.6250	0.0000
31	Ni	10.4265	10.4265	0.0000	0.7500	0.7500	0.0000
32	Ni	12.1642	12.1642	0.0000	0.8750	0.8750	0.0000
33	Ni	0.0000	1.7377	1.7377	0.0000	0.1250	0.0792
34	Ni	1.7377	0.0000	1.7377	0.1250	0.0000	0.0792
35	Ni	0.0000	5.2132	1.7377	0.0000	0.3750	0.0792
36	Ni	1.7377	3.4755	1.7377	0.1250	0.2500	0.0792
37	Ni	3.4755	1.7377	1.7377	0.2500	0.1250	0.0792
38	Ni	5.2132	0.0000	1.7377	0.3750	0.0000	0.0792
39	Ni	3.4755	5.2132	1.7377	0.2500	0.3750	0.0792

40	Ni	5.2132	3.4755	1.7377	0.3750	0.2500	0.0792
41	Ni	0.0000	8.6887	1.7377	0.0000	0.6250	0.0792
42	Ni	1.7377	6.9510	1.7377	0.1250	0.5000	0.0792
43	Ni	0.0000	12.1642	1.7377	0.0000	0.8750	0.0792
44	Ni	1.7377	10.4265	1.7377	0.1250	0.7500	0.0792
45	Ni	3.4755	8.6887	1.7377	0.2500	0.6250	0.0792
46	Ni	5.2132	6.9510	1.7377	0.3750	0.5000	0.0792
47	Ni	3.4755	12.1642	1.7377	0.2500	0.8750	0.0792
48	Ni	5.2132	10.4265	1.7377	0.3750	0.7500	0.0792
49	Ni	6.9510	1.7377	1.7377	0.5000	0.1250	0.0792
50	Ni	8.6887	0.0000	1.7377	0.6250	0.0000	0.0792
51	Ni	6.9510	5.2132	1.7377	0.5000	0.3750	0.0792
52	Ni	8.6887	3.4755	1.7377	0.6250	0.2500	0.0792
53	Ni	10.4265	1.7377	1.7377	0.7500	0.1250	0.0792
54	Ni	12.1642	0.0000	1.7377	0.8750	0.0000	0.0792
55	Ni	10.4265	5.2132	1.7377	0.7500	0.3750	0.0792
56	Ni	12.1642	3.4755	1.7377	0.8750	0.2500	0.0792
57	Ni	6.9510	8.6887	1.7377	0.5000	0.6250	0.0792
58	Ni	8.6887	6.9510	1.7377	0.6250	0.5000	0.0792
59	Ni	6.9510	12.1642	1.7377	0.5000	0.8750	0.0792
60	Ni	8.6887	10.4265	1.7377	0.6250	0.7500	0.0792
61	Ni	10.4265	8.6887	1.7377	0.7500	0.6250	0.0792
62	Ni	12.1642	6.9510	1.7377	0.8750	0.5000	0.0792
63	Ni	10.4265	12.1642	1.7377	0.7500	0.8750	0.0792
64	Ni	12.1642	10.4265	1.7377	0.8750	0.7500	0.0792
65	Ni	0.0125	0.0118	3.5063	0.0009	0.0008	0.1597
66	Ni	1.7373	1.7718	3.4597	0.1250	0.1274	0.1576
67	Ni	13.8814	3.4738	3.5109	0.9985	0.2499	0.1599
68	Ni	1.6904	5.1849	3.4585	0.1216	0.3730	0.1576
69	Ni	3.4688	0.0034	3.5095	0.2495	0.0002	0.1599
70	Ni	5.2151	1.7432	3.5544	0.3751	0.1254	0.1619
71	Ni	3.5029	3.4426	3.4769	0.2520	0.2476	0.1584
72	Ni	5.2351	5.2118	3.4673	0.3766	0.3749	0.1580
73	Ni	0.0005	6.9370	3.5130	0.0000	0.4990	0.1600
74	Ni	1.7522	8.6550	3.5451	0.1260	0.6226	0.1615
75	Ni	13.8992	10.4171	3.5174	0.9998	0.7493	0.1602
76	Ni	1.7423	12.1717	3.5153	0.1253	0.8755	0.1601
77	Ni	3.4748	6.9757	3.4648	0.2500	0.5018	0.1578
78	Ni	5.2314	8.7077	3.4997	0.3763	0.6264	0.1594
79	Ni	3.4660	10.3939	3.5252	0.2493	0.7477	0.1606
80	Ni	5.2042	12.1587	3.4981	0.3744	0.8746	0.1594
81	Ni	6.9365	13.8934	3.5023	0.4990	0.9994	0.1595
82	Ni	8.6910	1.7103	3.4281	0.6252	0.1230	0.1562
83	Ni	6.9485	3.4597	3.4924	0.4998	0.2489	0.1591
84	Ni	8.6648	5.2182	3.5167	0.6233	0.3754	0.1602
85	Ni	10.4447	13.8779	3.5185	0.7513	0.9983	0.1603
86	Ni	12.1652	1.7423	3.5371	0.8751	0.1253	0.1611
87	Ni	10.4156	3.4633	3.4839	0.7492	0.2491	0.1587
88	Ni	12.1539	5.2150	3.4894	0.8743	0.3751	0.1590

89	Ni	6.9471	6.9443	3.5062	0.4997	0.4995	0.1597
90	Ni	8.6973	8.6935	3.4646	0.6256	0.6253	0.1578
91	Ni	6.9801	10.4314	3.5112	0.5021	0.7504	0.1600
92	Ni	8.6952	12.1919	3.5023	0.6255	0.8770	0.1596
93	Ni	10.4196	6.9558	3.5152	0.7495	0.5003	0.1601
94	Ni	12.1872	8.6631	3.5032	0.8767	0.6232	0.1596
95	Ni	10.4475	10.4418	3.4288	0.7515	0.7511	0.1562
96	Ni	12.1831	12.1630	3.5007	0.8764	0.8749	0.1595
97	Ni	0.0355	1.7876	5.2820	0.0026	0.1286	0.2406
98	Ni	1.7368	13.8832	5.2996	0.1249	0.9986	0.2414
99	Ni	13.8243	5.1650	5.2990	0.9944	0.3715	0.2414
100	Ni	1.7479	3.5800	5.3978	0.1257	0.2575	0.2459
101	Ni	3.4508	1.7545	5.2838	0.2482	0.1262	0.2407
102	Ni	5.1961	13.8902	5.2167	0.3738	0.9992	0.2377
103	Ni	3.5248	5.1802	5.0452	0.2535	0.3726	0.2298
104	Ni	5.2396	3.4379	5.3420	0.3769	0.2473	0.2434
105	Ni	0.0320	8.6759	5.2116	0.0023	0.6241	0.2374
106	Ni	1.7295	6.8493	5.2898	0.1244	0.4927	0.2410
107	Ni	0.0038	12.1606	5.2108	0.0003	0.8747	0.2374
108	Ni	1.7298	10.4323	5.2198	0.1244	0.7504	0.2378
109	Ni	3.4522	8.7171	5.3750	0.2483	0.6270	0.2449
110	Ni	5.2113	6.9735	5.2015	0.3749	0.5016	0.2370
111	Ni	3.4796	12.1444	5.2191	0.2503	0.8736	0.2378
112	Ni	5.2293	10.4373	5.1932	0.3762	0.7508	0.2366
113	Ni	6.9651	1.6852	5.2665	0.5010	0.1212	0.2399
114	Ni	8.6903	13.8670	5.2847	0.6251	0.9975	0.2407
115	Ni	6.9484	5.1996	5.2620	0.4998	0.3740	0.2397
116	Ni	8.6968	3.4368	5.2255	0.6256	0.2472	0.2381
117	Ni	10.4180	1.6877	5.2736	0.7494	0.1214	0.2402
118	Ni	12.2016	0.0120	5.2094	0.8777	0.0009	0.2373
119	Ni	10.3775	5.1989	5.2389	0.7465	0.3740	0.2387
120	Ni	12.0782	3.4484	5.3034	0.8688	0.2481	0.2416
121	Ni	6.9892	8.7444	5.2346	0.5028	0.6290	0.2385
122	Ni	8.7137	6.9529	5.2649	0.6268	0.5001	0.2398
123	Ni	6.9377	12.1580	5.2158	0.4990	0.8745	0.2376
124	Ni	8.7274	10.4803	5.2060	0.6278	0.7539	0.2372
125	Ni	10.4586	8.7064	5.1637	0.7523	0.6263	0.2352
126	Ni	12.1419	6.9723	5.3600	0.8734	0.5015	0.2442
127	Ni	10.4716	12.1700	5.2701	0.7532	0.8754	0.2401
128	Ni	12.1934	10.4153	5.2772	0.8771	0.7492	0.2404
129	O	6.1069	4.3963	6.7943	0.4393	0.3162	0.3095
130	O	4.2379	7.6217	6.7550	0.3048	0.5482	0.3077
131	O	6.0607	6.6176	8.7808	0.4360	0.4760	0.4000
132	O	7.8658	7.8795	6.7841	0.5658	0.5668	0.3091
133	O	10.4429	10.3836	6.2587	0.7512	0.7469	0.2851
134	O	9.5142	7.1002	8.9437	0.6844	0.5107	0.4074
135	O	7.6843	9.8667	8.8600	0.5527	0.7097	0.4036
136	Ce	5.9771	8.8348	8.0380	0.4299	0.6355	0.3662
137	Ce	4.3522	5.5146	7.7389	0.3131	0.3967	0.3526

138	Ce	8.1590	5.7465	8.0637	0.5869	0.4134	0.3673
139	Sm	9.4537	9.0499	8.0536	0.6800	0.6510	0.3669
140	C	11.3515	4.5325	6.7430	0.8165	0.3260	0.3072
141	O	12.7243	5.7689	6.6384	0.9153	0.4150	0.3024
142	O	10.9757	4.3667	7.8817	0.7895	0.3141	0.3591
143	O	2.2317	5.2810	6.4094	0.1605	0.3799	0.2920
144	O	1.7362	1.6878	6.1832	0.1249	0.1214	0.2817
145	O	8.6764	1.7140	6.1527	0.6241	0.1233	0.2803

/db/jmorales/CoNi-alloy/metal-SDC-interface/Ni-Ce_3SmO_7/Ni-Ce_3SmO_7-conf4a

a = 13.9019837372
b = 13.9019837372
c = 21.9509918687
alpha = 90.0
beta = 90.0
gamma = 90.0

	Atom	X	Y	Z	X	Y	Z
1	Ni	0.0000	0.0000	0.0000	0.0000	0.0000	0.0000
2	Ni	1.7377	1.7377	0.0000	0.1250	0.1250	0.0000
3	Ni	0.0000	3.4755	0.0000	0.0000	0.2500	0.0000
4	Ni	1.7377	5.2132	0.0000	0.1250	0.3750	0.0000
5	Ni	3.4755	0.0000	0.0000	0.2500	0.0000	0.0000
6	Ni	5.2132	1.7377	0.0000	0.3750	0.1250	0.0000
7	Ni	3.4755	3.4755	0.0000	0.2500	0.2500	0.0000
8	Ni	5.2132	5.2132	0.0000	0.3750	0.3750	0.0000
9	Ni	0.0000	6.9510	0.0000	0.0000	0.5000	0.0000
10	Ni	1.7377	8.6887	0.0000	0.1250	0.6250	0.0000
11	Ni	0.0000	10.4265	0.0000	0.0000	0.7500	0.0000
12	Ni	1.7377	12.1642	0.0000	0.1250	0.8750	0.0000
13	Ni	3.4755	6.9510	0.0000	0.2500	0.5000	0.0000
14	Ni	5.2132	8.6887	0.0000	0.3750	0.6250	0.0000
15	Ni	3.4755	10.4265	0.0000	0.2500	0.7500	0.0000
16	Ni	5.2132	12.1642	0.0000	0.3750	0.8750	0.0000
17	Ni	6.9510	0.0000	0.0000	0.5000	0.0000	0.0000
18	Ni	8.6887	1.7377	0.0000	0.6250	0.1250	0.0000
19	Ni	6.9510	3.4755	0.0000	0.5000	0.2500	0.0000
20	Ni	8.6887	5.2132	0.0000	0.6250	0.3750	0.0000
21	Ni	10.4265	0.0000	0.0000	0.7500	0.0000	0.0000
22	Ni	12.1642	1.7377	0.0000	0.8750	0.1250	0.0000
23	Ni	10.4265	3.4755	0.0000	0.7500	0.2500	0.0000
24	Ni	12.1642	5.2132	0.0000	0.8750	0.3750	0.0000
25	Ni	6.9510	6.9510	0.0000	0.5000	0.5000	0.0000
26	Ni	8.6887	8.6887	0.0000	0.6250	0.6250	0.0000
27	Ni	6.9510	10.4265	0.0000	0.5000	0.7500	0.0000
28	Ni	8.6887	12.1642	0.0000	0.6250	0.8750	0.0000
29	Ni	10.4265	6.9510	0.0000	0.7500	0.5000	0.0000
30	Ni	12.1642	8.6887	0.0000	0.8750	0.6250	0.0000
31	Ni	10.4265	10.4265	0.0000	0.7500	0.7500	0.0000
32	Ni	12.1642	12.1642	0.0000	0.8750	0.8750	0.0000
33	Ni	0.0000	1.7377	1.7377	0.0000	0.1250	0.0792
34	Ni	1.7377	0.0000	1.7377	0.1250	0.0000	0.0792
35	Ni	0.0000	5.2132	1.7377	0.0000	0.3750	0.0792
36	Ni	1.7377	3.4755	1.7377	0.1250	0.2500	0.0792
37	Ni	3.4755	1.7377	1.7377	0.2500	0.1250	0.0792
38	Ni	5.2132	0.0000	1.7377	0.3750	0.0000	0.0792
39	Ni	3.4755	5.2132	1.7377	0.2500	0.3750	0.0792
40	Ni	5.2132	3.4755	1.7377	0.3750	0.2500	0.0792

41	Ni	0.0000	8.6887	1.7377	0.0000	0.6250	0.0792
42	Ni	1.7377	6.9510	1.7377	0.1250	0.5000	0.0792
43	Ni	0.0000	12.1642	1.7377	0.0000	0.8750	0.0792
44	Ni	1.7377	10.4265	1.7377	0.1250	0.7500	0.0792
45	Ni	3.4755	8.6887	1.7377	0.2500	0.6250	0.0792
46	Ni	5.2132	6.9510	1.7377	0.3750	0.5000	0.0792
47	Ni	3.4755	12.1642	1.7377	0.2500	0.8750	0.0792
48	Ni	5.2132	10.4265	1.7377	0.3750	0.7500	0.0792
49	Ni	6.9510	1.7377	1.7377	0.5000	0.1250	0.0792
50	Ni	8.6887	0.0000	1.7377	0.6250	0.0000	0.0792
51	Ni	6.9510	5.2132	1.7377	0.5000	0.3750	0.0792
52	Ni	8.6887	3.4755	1.7377	0.6250	0.2500	0.0792
53	Ni	10.4265	1.7377	1.7377	0.7500	0.1250	0.0792
54	Ni	12.1642	0.0000	1.7377	0.8750	0.0000	0.0792
55	Ni	10.4265	5.2132	1.7377	0.7500	0.3750	0.0792
56	Ni	12.1642	3.4755	1.7377	0.8750	0.2500	0.0792
57	Ni	6.9510	8.6887	1.7377	0.5000	0.6250	0.0792
58	Ni	8.6887	6.9510	1.7377	0.6250	0.5000	0.0792
59	Ni	6.9510	12.1642	1.7377	0.5000	0.8750	0.0792
60	Ni	8.6887	10.4265	1.7377	0.6250	0.7500	0.0792
61	Ni	10.4265	8.6887	1.7377	0.7500	0.6250	0.0792
62	Ni	12.1642	6.9510	1.7377	0.8750	0.5000	0.0792
63	Ni	10.4265	12.1642	1.7377	0.7500	0.8750	0.0792
64	Ni	12.1642	10.4265	1.7377	0.8750	0.7500	0.0792
65	Ni	13.9003	13.8993	3.5089	0.9999	0.9998	0.1599
66	Ni	1.7545	1.7094	3.4997	0.1262	0.1230	0.1594
67	Ni	13.8675	3.4670	3.4754	0.9975	0.2494	0.1583
68	Ni	1.7209	5.2404	3.4855	0.1238	0.3770	0.1588
69	Ni	3.4841	0.0135	3.5336	0.2506	0.0010	0.1610
70	Ni	5.2106	1.7339	3.5100	0.3748	0.1247	0.1599
71	Ni	3.4787	3.4765	3.4970	0.2502	0.2501	0.1593
72	Ni	5.1989	5.2065	3.5133	0.3740	0.3745	0.1600
73	Ni	0.0048	6.9555	3.5284	0.0003	0.5003	0.1607
74	Ni	1.7325	8.6675	3.5325	0.1246	0.6235	0.1609
75	Ni	0.0039	10.4271	3.5099	0.0003	0.7500	0.1599
76	Ni	1.7412	12.1656	3.5123	0.1252	0.8751	0.1600
77	Ni	3.4764	6.9618	3.5025	0.2501	0.5008	0.1596
78	Ni	5.2219	8.6879	3.4990	0.3756	0.6249	0.1594
79	Ni	3.4744	10.4301	3.5061	0.2499	0.7503	0.1597
80	Ni	5.2111	12.1619	3.5097	0.3748	0.8748	0.1599
81	Ni	6.9558	13.8948	3.5052	0.5003	0.9995	0.1597
82	Ni	8.6930	1.7398	3.5041	0.6253	0.1252	0.1596
83	Ni	6.9301	3.4895	3.5236	0.4985	0.2510	0.1605
84	Ni	8.6784	5.2030	3.5133	0.6243	0.3743	0.1601
85	Ni	10.4272	13.8988	3.5096	0.7501	0.9998	0.1599
86	Ni	12.1557	1.7293	3.5099	0.8744	0.1244	0.1599
87	Ni	10.4273	3.4767	3.5114	0.7501	0.2501	0.1600
88	Ni	12.1559	5.2161	3.5077	0.8744	0.3752	0.1598
89	Ni	6.9650	6.9443	3.4823	0.5010	0.4995	0.1586

90	Ni	8.7057	8.7051	3.4865	0.6262	0.6262	0.1588
91	Ni	6.9551	10.4425	3.4982	0.5003	0.7512	0.1594
92	Ni	8.6955	12.1637	3.5104	0.6255	0.8750	0.1599
93	Ni	10.4467	6.9510	3.4950	0.7515	0.5000	0.1592
94	Ni	12.1645	8.6920	3.5107	0.8750	0.6252	0.1599
95	Ni	10.4337	10.4306	3.5095	0.7505	0.7503	0.1599
96	Ni	12.1658	12.1645	3.5102	0.8751	0.8750	0.1599
97	Ni	0.0023	1.7382	5.2190	0.0002	0.1250	0.2378
98	Ni	1.7326	-0.0016	5.2306	0.1246	-0.0001	0.2383
99	Ni	13.8728	5.1827	5.2024	0.9979	0.3728	0.2370
100	Ni	1.6864	3.4523	5.0924	0.1213	0.2483	0.2320
101	Ni	3.4559	1.7164	5.3656	0.2486	0.1235	0.2444
102	Ni	5.2393	13.8791	5.2195	0.3769	0.9984	0.2378
103	Ni	3.4254	5.2245	5.2254	0.2464	0.3758	0.2381
104	Ni	5.2106	3.4611	5.2762	0.3748	0.2490	0.2404
105	Ni	13.8841	8.7067	5.2332	0.9987	0.6263	0.2384
106	Ni	1.6942	6.9993	5.3815	0.1219	0.5035	0.2452
107	Ni	0.0023	12.1607	5.2270	0.0002	0.8747	0.2381
108	Ni	1.7432	10.4181	5.2311	0.1254	0.7494	0.2383
109	Ni	3.4940	8.7037	5.2071	0.2513	0.6261	0.2372
110	Ni	5.2396	7.0166	5.2220	0.3769	0.5047	0.2379
111	Ni	3.4748	12.1609	5.2287	0.2500	0.8748	0.2382
112	Ni	5.2194	10.4145	5.2289	0.3754	0.7491	0.2382
113	Ni	6.9791	1.7347	5.2025	0.5020	0.1248	0.2370
114	Ni	8.6894	13.8998	5.2275	0.6250	0.9998	0.2381
115	Ni	6.9857	5.1929	5.2968	0.5025	0.3735	0.2413
116	Ni	8.6908	3.4676	5.2264	0.6251	0.2494	0.2381
117	Ni	10.4246	1.7341	5.2281	0.7499	0.1247	0.2382
118	Ni	12.1675	-0.0024	5.2281	0.8752	-0.0002	0.2382
119	Ni	10.4161	5.2208	5.2304	0.7493	0.3755	0.2383
120	Ni	12.1588	3.4653	5.2284	0.8746	0.2493	0.2382
121	Ni	6.9966	8.7150	5.1713	0.5033	0.6269	0.2356
122	Ni	8.7192	6.9749	5.1518	0.6272	0.5017	0.2347
123	Ni	6.9586	12.1538	5.2282	0.5005	0.8742	0.2382
124	Ni	8.7004	10.4274	5.2296	0.6258	0.7501	0.2382
125	Ni	10.4253	8.6879	5.2343	0.7499	0.6249	0.2385
126	Ni	12.1636	6.9517	5.2325	0.8750	0.5000	0.2384
127	Ni	10.4277	12.1674	5.2299	0.7501	0.8752	0.2383
128	Ni	12.1592	10.4310	5.2285	0.8746	0.7503	0.2382
129	O	4.3245	2.7147	6.7988	0.3111	0.1953	0.3097
130	O	6.1922	2.3685	9.5574	0.4454	0.1704	0.4354
131	O	2.4598	5.9344	6.8108	0.1769	0.4269	0.3103
132	O	4.1744	4.9440	8.7413	0.3003	0.3556	0.3982
133	O	5.9899	6.0522	6.7419	0.4309	0.4353	0.3071
134	O	7.7764	5.2254	8.7694	0.5594	0.3759	0.3995
135	O	5.8012	7.9155	8.7465	0.4173	0.5694	0.3985
136	Ce	4.2041	6.9935	7.8794	0.3024	0.5031	0.3590
137	Ce	2.5634	3.7528	7.7149	0.1844	0.2699	0.3515
138	Ce	6.2656	3.7413	8.2866	0.4507	0.2691	0.3775

139 Sm	7.6663	7.1025	7.8703	0.5515	0.5109	0.3585

/db/jmorales/CoNi-alloy/metal-SDC-interface/CoNi-Ce_3SmO_7/CoNi-Ce_3SmO_7-conf1b

a =				13.9033384264			
b =				13.9033384264			
c =				21.9516692133			
alpha =				90.0			
beta =				90.0			
gamma =				90.0			
	Atom	X	Y	Z	X	Y	Z
1	Ni	0.0000	0.0000	0.0000	0.0000	0.0000	0.0000
2	Ni	0.0000	3.4758	0.0000	0.0000	0.2500	0.0000
3	Ni	3.4758	0.0000	0.0000	0.2500	0.0000	0.0000
4	Ni	3.4758	3.4758	0.0000	0.2500	0.2500	0.0000
5	Ni	0.0000	6.9517	0.0000	0.0000	0.5000	0.0000
6	Ni	0.0000	10.4275	0.0000	0.0000	0.7500	0.0000
7	Ni	3.4758	6.9517	0.0000	0.2500	0.5000	0.0000
8	Ni	3.4758	10.4275	0.0000	0.2500	0.7500	0.0000
9	Ni	6.9517	0.0000	0.0000	0.5000	0.0000	0.0000
10	Ni	6.9517	3.4758	0.0000	0.5000	0.2500	0.0000
11	Ni	10.4275	0.0000	0.0000	0.7500	0.0000	0.0000
12	Ni	10.4275	3.4758	0.0000	0.7500	0.2500	0.0000
13	Ni	6.9517	6.9517	0.0000	0.5000	0.5000	0.0000
14	Ni	6.9517	10.4275	0.0000	0.5000	0.7500	0.0000
15	Ni	10.4275	6.9517	0.0000	0.7500	0.5000	0.0000
16	Ni	10.4275	10.4275	0.0000	0.7500	0.7500	0.0000
17	Ni	0.0000	1.7379	1.7379	0.0000	0.1250	0.0792
18	Ni	0.0000	5.2138	1.7379	0.0000	0.3750	0.0792
19	Ni	3.4758	1.7379	1.7379	0.2500	0.1250	0.0792
20	Ni	3.4758	5.2138	1.7379	0.2500	0.3750	0.0792
21	Ni	0.0000	8.6896	1.7379	0.0000	0.6250	0.0792
22	Ni	0.0000	12.1654	1.7379	0.0000	0.8750	0.0792
23	Ni	3.4758	8.6896	1.7379	0.2500	0.6250	0.0792
24	Ni	3.4758	12.1654	1.7379	0.2500	0.8750	0.0792
25	Ni	6.9517	1.7379	1.7379	0.5000	0.1250	0.0792
26	Ni	6.9517	5.2138	1.7379	0.5000	0.3750	0.0792
27	Ni	10.4275	1.7379	1.7379	0.7500	0.1250	0.0792
28	Ni	10.4275	5.2138	1.7379	0.7500	0.3750	0.0792
29	Ni	6.9517	8.6896	1.7379	0.5000	0.6250	0.0792
30	Ni	6.9517	12.1654	1.7379	0.5000	0.8750	0.0792
31	Ni	10.4275	8.6896	1.7379	0.7500	0.6250	0.0792
32	Ni	10.4275	12.1654	1.7379	0.7500	0.8750	0.0792
33	Ni	0.0017	13.8993	3.5056	0.0001	0.9997	0.1597
34	Ni	-0.0005	3.4745	3.5013	-0.0000	0.2499	0.1595
35	Ni	3.4776	13.8987	3.5048	0.2501	0.9997	0.1597
36	Ni	3.4635	3.4475	3.4659	0.2491	0.2480	0.1579
37	Ni	0.0014	6.9589	3.5153	0.0001	0.5005	0.1601
38	Ni	13.8915	10.4336	3.4942	0.9992	0.7504	0.1592
39	Ni	3.4751	6.9563	3.4948	0.2499	0.5003	0.1592

40	Ni	3.4924	10.4008	3.5239	0.2512	0.7481	0.1605
41	Ni	6.9554	0.0134	3.4919	0.5003	0.0010	0.1591
42	Ni	6.9569	3.4883	3.5182	0.5004	0.2509	0.1603
43	Ni	10.4250	0.0026	3.5064	0.7498	0.0002	0.1597
44	Ni	10.4298	3.4758	3.5031	0.7502	0.2500	0.1596
45	Ni	6.9667	6.9624	3.4984	0.5011	0.5008	0.1594
46	Ni	6.9659	10.4362	3.4798	0.5010	0.7506	0.1585
47	Ni	10.4360	6.9465	3.4903	0.7506	0.4996	0.1590
48	Ni	10.4309	10.4375	3.4982	0.7502	0.7507	0.1594
49	Ni	0.0077	1.7384	5.2082	0.0006	0.1250	0.2373
50	Ni	0.0027	5.2100	5.2171	0.0002	0.3747	0.2377
51	Ni	3.4599	1.7359	5.2077	0.2489	0.1249	0.2372
52	Ni	3.4482	5.1668	5.0822	0.2480	0.3716	0.2315
53	Ni	13.8791	8.7186	5.1948	0.9983	0.6271	0.2366
54	Ni	0.0012	12.1644	5.2140	0.0001	0.8749	0.2375
55	Ni	3.4505	8.6667	5.2446	0.2482	0.6234	0.2389
56	Ni	3.4809	12.1510	5.2208	0.2504	0.8740	0.2378
57	Ni	6.9545	1.7392	5.2063	0.5002	0.1251	0.2372
58	Ni	6.9886	5.1606	5.3555	0.5027	0.3712	0.2440
59	Ni	10.4302	1.7302	5.2055	0.7502	0.1244	0.2371
60	Ni	10.4260	5.2148	5.2125	0.7499	0.3751	0.2375
61	Ni	7.0058	8.7054	5.2230	0.5039	0.6261	0.2379
62	Ni	6.9450	12.2020	5.1637	0.4995	0.8776	0.2352
63	Ni	10.4220	8.6954	5.2058	0.7496	0.6254	0.2372
64	Ni	10.4493	12.1908	5.2537	0.7516	0.8768	0.2393
65	Co	1.7379	1.7379	0.0000	0.1250	0.1250	0.0000
66	Co	1.7379	5.2138	0.0000	0.1250	0.3750	0.0000
67	Co	5.2138	1.7379	0.0000	0.3750	0.1250	0.0000
68	Co	5.2138	5.2138	0.0000	0.3750	0.3750	0.0000
69	Co	1.7379	8.6896	0.0000	0.1250	0.6250	0.0000
70	Co	1.7379	12.1654	0.0000	0.1250	0.8750	0.0000
71	Co	5.2138	8.6896	0.0000	0.3750	0.6250	0.0000
72	Co	5.2138	12.1654	0.0000	0.3750	0.8750	0.0000
73	Co	8.6896	1.7379	0.0000	0.6250	0.1250	0.0000
74	Co	8.6896	5.2138	0.0000	0.6250	0.3750	0.0000
75	Co	12.1654	1.7379	0.0000	0.8750	0.1250	0.0000
76	Co	12.1654	5.2138	0.0000	0.8750	0.3750	0.0000
77	Co	8.6896	8.6896	0.0000	0.6250	0.6250	0.0000
78	Co	8.6896	12.1654	0.0000	0.6250	0.8750	0.0000
79	Co	12.1654	8.6896	0.0000	0.8750	0.6250	0.0000
80	Co	12.1654	12.1654	0.0000	0.8750	0.8750	0.0000
81	Co	1.7379	0.0000	1.7379	0.1250	0.0000	0.0792
82	Co	1.7379	3.4758	1.7379	0.1250	0.2500	0.0792
83	Co	5.2138	0.0000	1.7379	0.3750	0.0000	0.0792
84	Co	5.2138	3.4758	1.7379	0.3750	0.2500	0.0792
85	Co	1.7379	6.9517	1.7379	0.1250	0.5000	0.0792
86	Co	1.7379	10.4275	1.7379	0.1250	0.7500	0.0792
87	Co	5.2138	6.9517	1.7379	0.3750	0.5000	0.0792
88	Co	5.2138	10.4275	1.7379	0.3750	0.7500	0.0792

89	Co	8.6896	0.0000	1.7379	0.6250	0.0000	0.0792
90	Co	8.6896	3.4758	1.7379	0.6250	0.2500	0.0792
91	Co	12.1654	0.0000	1.7379	0.8750	0.0000	0.0792
92	Co	12.1654	3.4758	1.7379	0.8750	0.2500	0.0792
93	Co	8.6896	6.9517	1.7379	0.6250	0.5000	0.0792
94	Co	8.6896	10.4275	1.7379	0.6250	0.7500	0.0792
95	Co	12.1654	6.9517	1.7379	0.8750	0.5000	0.0792
96	Co	12.1654	10.4275	1.7379	0.8750	0.7500	0.0792
97	Co	1.7328	1.7309	3.5476	0.1246	0.1245	0.1616
98	Co	1.7031	5.2357	3.5268	0.1225	0.3766	0.1607
99	Co	5.2157	1.7301	3.5528	0.3751	0.1244	0.1618
100	Co	5.2409	5.1880	3.5164	0.3770	0.3731	0.1602
101	Co	1.7291	8.6845	3.5336	0.1244	0.6246	0.1610
102	Co	1.7446	12.1655	3.5477	0.1255	0.8750	0.1616
103	Co	5.2074	8.6765	3.5622	0.3745	0.6241	0.1623
104	Co	5.2128	12.1594	3.5620	0.3749	0.8746	0.1623
105	Co	8.6909	1.7310	3.5514	0.6251	0.1245	0.1618
106	Co	8.6747	5.2035	3.5661	0.6239	0.3743	0.1625
107	Co	12.1680	1.7377	3.5511	0.8752	0.1250	0.1618
108	Co	12.1690	5.2129	3.5618	0.8753	0.3749	0.1623
109	Co	8.6935	8.7013	3.5402	0.6253	0.6258	0.1613
110	Co	8.7051	12.1834	3.4584	0.6261	0.8763	0.1575
111	Co	12.1564	8.6937	3.5500	0.8744	0.6253	0.1617
112	Co	12.1549	12.1734	3.5716	0.8742	0.8756	0.1627
113	Co	1.7444	13.8927	5.2326	0.1255	0.9992	0.2384
114	Co	1.7407	3.4826	5.2170	0.1252	0.2505	0.2377
115	Co	5.2037	0.0064	5.2225	0.3743	0.0005	0.2379
116	Co	5.1734	3.4346	5.1985	0.3721	0.2470	0.2368
117	Co	1.7089	6.9628	5.3287	0.1229	0.5008	0.2427
118	Co	1.7295	10.4585	5.1917	0.1244	0.7522	0.2365
119	Co	5.2513	6.8679	5.1714	0.3777	0.4940	0.2356
120	Co	5.2270	10.4214	5.2300	0.3760	0.7496	0.2383
121	Co	8.6849	-0.0052	5.2973	0.6247	-0.0004	0.2413
122	Co	8.7140	3.4534	5.2249	0.6268	0.2484	0.2380
123	Co	12.1810	0.0152	5.2235	0.8761	0.0011	0.2380
124	Co	12.1650	3.4690	5.2241	0.8750	0.2495	0.2380
125	Co	8.7278	6.9672	5.1775	0.6277	0.5011	0.2359
126	Co	8.7342	10.4855	5.2136	0.6282	0.7542	0.2375
127	Co	12.1622	6.9574	5.2213	0.8748	0.5004	0.2379
128	Co	12.1624	10.4282	5.2173	0.8748	0.7501	0.2377
129	O	5.9010	5.9827	6.7864	0.4244	0.4303	0.3092
130	O	2.6347	7.8126	6.8415	0.1895	0.5619	0.3117
131	O	4.8654	7.7407	8.7933	0.3499	0.5568	0.4006
132	O	6.1055	9.5876	6.8193	0.4391	0.6896	0.3107
133	O	8.0125	9.4781	8.9366	0.5763	0.6817	0.4071
134	O	5.1879	11.3128	8.8828	0.3731	0.8137	0.4047
135	O	8.6072	12.1672	6.3321	0.6191	0.8751	0.2885
136	Ce	3.9735	9.7827	8.0340	0.2858	0.7036	0.3660
137	Ce	3.7933	6.0782	7.7072	0.2728	0.4372	0.3511

138 Ce	7.1162	7.7326	8.0438	0.5118	0.5562	0.3664
139 Sm	7.1884	11.2290	8.0710	0.5170	0.8076	0.3677

/db/jmorales/CoNi-alloy/metal-SDC-interface/Ni-Ce_3SmO_7/Ni-Ce_3SmO_7-conf5b

a =	13.9019837372
b =	13.9019837372
c =	21.9509918687
alpha =	90.0
beta =	90.0
gamma =	90.0

	Atom	X	Y	Z	X	Y	Z
1	Ni	0.0000	0.0000	0.0000	0.0000	0.0000	0.0000
2	Ni	1.7377	1.7377	0.0000	0.1250	0.1250	0.0000
3	Ni	0.0000	3.4755	0.0000	0.0000	0.2500	0.0000
4	Ni	1.7377	5.2132	0.0000	0.1250	0.3750	0.0000
5	Ni	3.4755	0.0000	0.0000	0.2500	0.0000	0.0000
6	Ni	5.2132	1.7377	0.0000	0.3750	0.1250	0.0000
7	Ni	3.4755	3.4755	0.0000	0.2500	0.2500	0.0000
8	Ni	5.2132	5.2132	0.0000	0.3750	0.3750	0.0000
9	Ni	0.0000	6.9510	0.0000	0.0000	0.5000	0.0000
10	Ni	1.7377	8.6887	0.0000	0.1250	0.6250	0.0000
11	Ni	0.0000	10.4265	0.0000	0.0000	0.7500	0.0000
12	Ni	1.7377	12.1642	0.0000	0.1250	0.8750	0.0000
13	Ni	3.4755	6.9510	0.0000	0.2500	0.5000	0.0000
14	Ni	5.2132	8.6887	0.0000	0.3750	0.6250	0.0000
15	Ni	3.4755	10.4265	0.0000	0.2500	0.7500	0.0000
16	Ni	5.2132	12.1642	0.0000	0.3750	0.8750	0.0000
17	Ni	6.9510	0.0000	0.0000	0.5000	0.0000	0.0000
18	Ni	8.6887	1.7377	0.0000	0.6250	0.1250	0.0000
19	Ni	6.9510	3.4755	0.0000	0.5000	0.2500	0.0000
20	Ni	8.6887	5.2132	0.0000	0.6250	0.3750	0.0000
21	Ni	10.4265	0.0000	0.0000	0.7500	0.0000	0.0000
22	Ni	12.1642	1.7377	0.0000	0.8750	0.1250	0.0000
23	Ni	10.4265	3.4755	0.0000	0.7500	0.2500	0.0000
24	Ni	12.1642	5.2132	0.0000	0.8750	0.3750	0.0000
25	Ni	6.9510	6.9510	0.0000	0.5000	0.5000	0.0000
26	Ni	8.6887	8.6887	0.0000	0.6250	0.6250	0.0000
27	Ni	6.9510	10.4265	0.0000	0.5000	0.7500	0.0000
28	Ni	8.6887	12.1642	0.0000	0.6250	0.8750	0.0000
29	Ni	10.4265	6.9510	0.0000	0.7500	0.5000	0.0000
30	Ni	12.1642	8.6887	0.0000	0.8750	0.6250	0.0000
31	Ni	10.4265	10.4265	0.0000	0.7500	0.7500	0.0000
32	Ni	12.1642	12.1642	0.0000	0.8750	0.8750	0.0000
33	Ni	0.0000	1.7377	1.7377	0.0000	0.1250	0.0792
34	Ni	1.7377	0.0000	1.7377	0.1250	0.0000	0.0792
35	Ni	0.0000	5.2132	1.7377	0.0000	0.3750	0.0792
36	Ni	1.7377	3.4755	1.7377	0.1250	0.2500	0.0792
37	Ni	3.4755	1.7377	1.7377	0.2500	0.1250	0.0792
38	Ni	5.2132	0.0000	1.7377	0.3750	0.0000	0.0792
39	Ni	3.4755	5.2132	1.7377	0.2500	0.3750	0.0792
40	Ni	5.2132	3.4755	1.7377	0.3750	0.2500	0.0792

41	Ni	0.0000	8.6887	1.7377	0.0000	0.6250	0.0792
42	Ni	1.7377	6.9510	1.7377	0.1250	0.5000	0.0792
43	Ni	0.0000	12.1642	1.7377	0.0000	0.8750	0.0792
44	Ni	1.7377	10.4265	1.7377	0.1250	0.7500	0.0792
45	Ni	3.4755	8.6887	1.7377	0.2500	0.6250	0.0792
46	Ni	5.2132	6.9510	1.7377	0.3750	0.5000	0.0792
47	Ni	3.4755	12.1642	1.7377	0.2500	0.8750	0.0792
48	Ni	5.2132	10.4265	1.7377	0.3750	0.7500	0.0792
49	Ni	6.9510	1.7377	1.7377	0.5000	0.1250	0.0792
50	Ni	8.6887	0.0000	1.7377	0.6250	0.0000	0.0792
51	Ni	6.9510	5.2132	1.7377	0.5000	0.3750	0.0792
52	Ni	8.6887	3.4755	1.7377	0.6250	0.2500	0.0792
53	Ni	10.4265	1.7377	1.7377	0.7500	0.1250	0.0792
54	Ni	12.1642	0.0000	1.7377	0.8750	0.0000	0.0792
55	Ni	10.4265	5.2132	1.7377	0.7500	0.3750	0.0792
56	Ni	12.1642	3.4755	1.7377	0.8750	0.2500	0.0792
57	Ni	6.9510	8.6887	1.7377	0.5000	0.6250	0.0792
58	Ni	8.6887	6.9510	1.7377	0.6250	0.5000	0.0792
59	Ni	6.9510	12.1642	1.7377	0.5000	0.8750	0.0792
60	Ni	8.6887	10.4265	1.7377	0.6250	0.7500	0.0792
61	Ni	10.4265	8.6887	1.7377	0.7500	0.6250	0.0792
62	Ni	12.1642	6.9510	1.7377	0.8750	0.5000	0.0792
63	Ni	10.4265	12.1642	1.7377	0.7500	0.8750	0.0792
64	Ni	12.1642	10.4265	1.7377	0.8750	0.7500	0.0792
65	Ni	13.8992	13.8995	3.5113	0.9998	0.9998	0.1600
66	Ni	1.7408	1.7332	3.5036	0.1252	0.1247	0.1596
67	Ni	13.8937	3.4706	3.5034	0.9994	0.2496	0.1596
68	Ni	1.7269	5.2207	3.5053	0.1242	0.3755	0.1597
69	Ni	3.4793	0.0003	3.5128	0.2503	0.0000	0.1600
70	Ni	5.2145	1.7507	3.5255	0.3751	0.1259	0.1606
71	Ni	3.4959	3.4918	3.5235	0.2515	0.2512	0.1605
72	Ni	5.2104	5.2254	3.5183	0.3748	0.3759	0.1603
73	Ni	0.0147	6.9461	3.5400	0.0011	0.4996	0.1613
74	Ni	1.7064	8.6703	3.5037	0.1227	0.6237	0.1596
75	Ni	13.8976	10.4284	3.5103	0.9997	0.7501	0.1599
76	Ni	1.7301	12.1736	3.5111	0.1244	0.8757	0.1599
77	Ni	3.4706	6.9560	3.4891	0.2497	0.5004	0.1589
78	Ni	5.2292	8.6993	3.4739	0.3761	0.6258	0.1583
79	Ni	3.4612	10.4576	3.4732	0.2490	0.7522	0.1582
80	Ni	5.2173	12.1757	3.5036	0.3753	0.8758	0.1596
81	Ni	6.9513	13.8980	3.5085	0.5000	0.9997	0.1598
82	Ni	8.6953	1.7253	3.5052	0.6255	0.1241	0.1597
83	Ni	6.9511	3.4659	3.5001	0.5000	0.2493	0.1595
84	Ni	8.6801	5.1981	3.5099	0.6244	0.3739	0.1599
85	Ni	10.4287	13.8936	3.5106	0.7502	0.9994	0.1599
86	Ni	12.1648	1.7294	3.5089	0.8750	0.1244	0.1599
87	Ni	10.4355	3.4700	3.5021	0.7506	0.2496	0.1595
88	Ni	12.1648	5.2158	3.5085	0.8750	0.3752	0.1598
89	Ni	6.9567	6.9246	3.5005	0.5004	0.4981	0.1595

90	Ni	8.6803	8.6881	3.5095	0.6244	0.6250	0.1599
91	Ni	6.9543	10.4263	3.5019	0.5002	0.7500	0.1595
92	Ni	8.6913	12.1653	3.5079	0.6252	0.8751	0.1598
93	Ni	10.4334	6.9520	3.5038	0.7505	0.5001	0.1596
94	Ni	12.1654	8.6850	3.5173	0.8751	0.6247	0.1602
95	Ni	10.4311	10.4219	3.5087	0.7503	0.7497	0.1598
96	Ni	12.1626	12.1611	3.5129	0.8749	0.8748	0.1600
97	Ni	13.9003	1.7371	5.2239	0.9999	0.1250	0.2380
98	Ni	1.7373	0.0038	5.2270	0.1250	0.0003	0.2381
99	Ni	13.8817	5.1898	5.2188	0.9985	0.3733	0.2377
100	Ni	1.7345	3.4499	5.1923	0.1248	0.2482	0.2365
101	Ni	3.4764	1.7422	5.2247	0.2501	0.1253	0.2380
102	Ni	5.2174	13.8962	5.2324	0.3753	0.9996	0.2384
103	Ni	3.4445	5.2374	5.2401	0.2478	0.3767	0.2387
104	Ni	5.2160	3.4520	5.3263	0.3752	0.2483	0.2426
105	Ni	-0.0022	8.6957	5.2376	-0.0002	0.6255	0.2386
106	Ni	1.7263	6.9872	5.4085	0.1242	0.5026	0.2464
107	Ni	13.9007	12.1612	5.2304	0.9999	0.8748	0.2383
108	Ni	1.7371	10.4234	5.2165	0.1250	0.7498	0.2376
109	Ni	3.4512	8.7378	5.0920	0.2483	0.6285	0.2320
110	Ni	5.2086	7.0003	5.2312	0.3747	0.5035	0.2383
111	Ni	3.4688	12.1643	5.2283	0.2495	0.8750	0.2382
112	Ni	5.1914	10.4513	5.1772	0.3734	0.7518	0.2359
113	Ni	6.9708	1.7102	5.2099	0.5014	0.1230	0.2373
114	Ni	8.6915	13.8845	5.2325	0.6252	0.9987	0.2384
115	Ni	6.9933	5.1711	5.2992	0.5030	0.3720	0.2414
116	Ni	8.7299	3.4503	5.2053	0.6280	0.2482	0.2371
117	Ni	10.4320	1.7230	5.2250	0.7504	0.1239	0.2380
118	Ni	12.1661	13.8985	5.2303	0.8751	0.9998	0.2383
119	Ni	10.4274	5.2123	5.2235	0.7501	0.3749	0.2380
120	Ni	12.1572	3.4679	5.2242	0.8745	0.2495	0.2380
121	Ni	7.0039	8.7061	5.2539	0.5038	0.6262	0.2393
122	Ni	8.7076	6.9215	5.1884	0.6264	0.4979	0.2364
123	Ni	6.9536	12.1439	5.2339	0.5002	0.8735	0.2384
124	Ni	8.6912	10.4386	5.2136	0.6252	0.7509	0.2375
125	Ni	10.4075	8.6884	5.2354	0.7486	0.6250	0.2385
126	Ni	12.1612	6.9532	5.2312	0.8748	0.5002	0.2383
127	Ni	10.4255	12.1587	5.2332	0.7499	0.8746	0.2384
128	Ni	12.1601	10.4239	5.2309	0.8747	0.7498	0.2383
129	O	6.0433	4.3949	6.7746	0.4347	0.3161	0.3086
130	O	3.4916	3.3133	9.4359	0.2512	0.2383	0.4299
131	O	2.7093	6.1102	6.7958	0.1949	0.4395	0.3096
132	O	4.9293	6.2839	8.7817	0.3546	0.4520	0.4001
133	O	5.9929	7.9907	6.7529	0.4311	0.5748	0.3076
134	O	7.8765	8.2502	8.8427	0.5666	0.5935	0.4028
135	O	4.9783	9.8359	8.6939	0.3581	0.7075	0.3961
136	Ce	3.8535	8.1528	7.9311	0.2772	0.5865	0.3613
137	Ce	3.9393	4.3974	7.9801	0.2834	0.3163	0.3635
138	Ce	7.1288	6.3780	8.0826	0.5128	0.4588	0.3682

139 Sm	6.9372	9.9104	7.9381	0.4990	0.7129	0.3616

/db/jmorales/CoNi-alloy/metal-SDC-interface/Ni-Ce_3SmO_7/Ni-Ce_3SmO_7-conf3a

a =				13.9019837372			
b =				13.9019837372			
c =				21.9509918687			
alpha =				90.0			
beta =				90.0			
gamma =				90.0			
	Atom	X	Y	Z	X	Y	Z
1	Ni	0.0000	0.0000	0.0000	0.0000	0.0000	0.0000
2	Ni	1.7377	1.7377	0.0000	0.1250	0.1250	0.0000
3	Ni	0.0000	3.4755	0.0000	0.0000	0.2500	0.0000
4	Ni	1.7377	5.2132	0.0000	0.1250	0.3750	0.0000
5	Ni	3.4755	0.0000	0.0000	0.2500	0.0000	0.0000
6	Ni	5.2132	1.7377	0.0000	0.3750	0.1250	0.0000
7	Ni	3.4755	3.4755	0.0000	0.2500	0.2500	0.0000
8	Ni	5.2132	5.2132	0.0000	0.3750	0.3750	0.0000
9	Ni	0.0000	6.9510	0.0000	0.0000	0.5000	0.0000
10	Ni	1.7377	8.6887	0.0000	0.1250	0.6250	0.0000
11	Ni	0.0000	10.4265	0.0000	0.0000	0.7500	0.0000
12	Ni	1.7377	12.1642	0.0000	0.1250	0.8750	0.0000
13	Ni	3.4755	6.9510	0.0000	0.2500	0.5000	0.0000
14	Ni	5.2132	8.6887	0.0000	0.3750	0.6250	0.0000
15	Ni	3.4755	10.4265	0.0000	0.2500	0.7500	0.0000
16	Ni	5.2132	12.1642	0.0000	0.3750	0.8750	0.0000
17	Ni	6.9510	0.0000	0.0000	0.5000	0.0000	0.0000
18	Ni	8.6887	1.7377	0.0000	0.6250	0.1250	0.0000
19	Ni	6.9510	3.4755	0.0000	0.5000	0.2500	0.0000
20	Ni	8.6887	5.2132	0.0000	0.6250	0.3750	0.0000
21	Ni	10.4265	0.0000	0.0000	0.7500	0.0000	0.0000
22	Ni	12.1642	1.7377	0.0000	0.8750	0.1250	0.0000
23	Ni	10.4265	3.4755	0.0000	0.7500	0.2500	0.0000
24	Ni	12.1642	5.2132	0.0000	0.8750	0.3750	0.0000
25	Ni	6.9510	6.9510	0.0000	0.5000	0.5000	0.0000
26	Ni	8.6887	8.6887	0.0000	0.6250	0.6250	0.0000
27	Ni	6.9510	10.4265	0.0000	0.5000	0.7500	0.0000
28	Ni	8.6887	12.1642	0.0000	0.6250	0.8750	0.0000
29	Ni	10.4265	6.9510	0.0000	0.7500	0.5000	0.0000
30	Ni	12.1642	8.6887	0.0000	0.8750	0.6250	0.0000
31	Ni	10.4265	10.4265	0.0000	0.7500	0.7500	0.0000
32	Ni	12.1642	12.1642	0.0000	0.8750	0.8750	0.0000
33	Ni	0.0000	1.7377	1.7377	0.0000	0.1250	0.0792
34	Ni	1.7377	0.0000	1.7377	0.1250	0.0000	0.0792
35	Ni	0.0000	5.2132	1.7377	0.0000	0.3750	0.0792
36	Ni	1.7377	3.4755	1.7377	0.1250	0.2500	0.0792
37	Ni	3.4755	1.7377	1.7377	0.2500	0.1250	0.0792
38	Ni	5.2132	0.0000	1.7377	0.3750	0.0000	0.0792
39	Ni	3.4755	5.2132	1.7377	0.2500	0.3750	0.0792
40	Ni	5.2132	3.4755	1.7377	0.3750	0.2500	0.0792

41	Ni	0.0000	8.6887	1.7377	0.0000	0.6250	0.0792
42	Ni	1.7377	6.9510	1.7377	0.1250	0.5000	0.0792
43	Ni	0.0000	12.1642	1.7377	0.0000	0.8750	0.0792
44	Ni	1.7377	10.4265	1.7377	0.1250	0.7500	0.0792
45	Ni	3.4755	8.6887	1.7377	0.2500	0.6250	0.0792
46	Ni	5.2132	6.9510	1.7377	0.3750	0.5000	0.0792
47	Ni	3.4755	12.1642	1.7377	0.2500	0.8750	0.0792
48	Ni	5.2132	10.4265	1.7377	0.3750	0.7500	0.0792
49	Ni	6.9510	1.7377	1.7377	0.5000	0.1250	0.0792
50	Ni	8.6887	0.0000	1.7377	0.6250	0.0000	0.0792
51	Ni	6.9510	5.2132	1.7377	0.5000	0.3750	0.0792
52	Ni	8.6887	3.4755	1.7377	0.6250	0.2500	0.0792
53	Ni	10.4265	1.7377	1.7377	0.7500	0.1250	0.0792
54	Ni	12.1642	0.0000	1.7377	0.8750	0.0000	0.0792
55	Ni	10.4265	5.2132	1.7377	0.7500	0.3750	0.0792
56	Ni	12.1642	3.4755	1.7377	0.8750	0.2500	0.0792
57	Ni	6.9510	8.6887	1.7377	0.5000	0.6250	0.0792
58	Ni	8.6887	6.9510	1.7377	0.6250	0.5000	0.0792
59	Ni	6.9510	12.1642	1.7377	0.5000	0.8750	0.0792
60	Ni	8.6887	10.4265	1.7377	0.6250	0.7500	0.0792
61	Ni	10.4265	8.6887	1.7377	0.7500	0.6250	0.0792
62	Ni	12.1642	6.9510	1.7377	0.8750	0.5000	0.0792
63	Ni	10.4265	12.1642	1.7377	0.7500	0.8750	0.0792
64	Ni	12.1642	10.4265	1.7377	0.8750	0.7500	0.0792
65	Ni	-0.0006	13.9007	3.5106	-0.0000	0.9999	0.1599
66	Ni	1.7420	1.7339	3.5047	0.1253	0.1247	0.1597
67	Ni	13.8958	3.4715	3.5025	0.9996	0.2497	0.1596
68	Ni	1.7249	5.2191	3.5021	0.1241	0.3754	0.1595
69	Ni	3.4794	13.9015	3.5115	0.2503	1.0000	0.1600
70	Ni	5.2057	1.7545	3.5352	0.3745	0.1262	0.1611
71	Ni	3.4953	3.4873	3.5315	0.2514	0.2508	0.1609
72	Ni	5.2087	5.2018	3.5119	0.3747	0.3742	0.1600
73	Ni	0.0054	6.9447	3.5247	0.0004	0.4995	0.1606
74	Ni	1.7106	8.6712	3.5054	0.1230	0.6237	0.1597
75	Ni	13.9008	10.4314	3.5084	0.9999	0.7504	0.1598
76	Ni	1.7325	12.1711	3.5101	0.1246	0.8755	0.1599
77	Ni	3.4773	6.9599	3.4841	0.2501	0.5006	0.1587
78	Ni	5.2217	8.7005	3.4804	0.3756	0.6258	0.1586
79	Ni	3.4682	10.4553	3.4848	0.2495	0.7521	0.1588
80	Ni	5.2197	12.1687	3.5074	0.3755	0.8753	0.1598
81	Ni	6.9501	13.9003	3.5093	0.4999	0.9999	0.1599
82	Ni	8.6904	1.7480	3.5219	0.6251	0.1257	0.1604
83	Ni	6.9331	3.4460	3.4930	0.4987	0.2479	0.1591
84	Ni	8.7206	5.2041	3.4232	0.6273	0.3743	0.1559
85	Ni	10.4230	13.8959	3.5096	0.7497	0.9996	0.1599
86	Ni	12.1670	1.7271	3.5076	0.8752	0.1242	0.1598
87	Ni	10.4331	3.4647	3.5049	0.7505	0.2492	0.1597
88	Ni	12.1429	5.2118	3.5251	0.8735	0.3749	0.1606
89	Ni	6.9604	6.9600	3.4627	0.5007	0.5006	0.1577

90	Ni	8.6828	8.6747	3.5304	0.6246	0.6240	0.1608
91	Ni	6.9536	10.4127	3.5212	0.5002	0.7490	0.1604
92	Ni	8.6842	12.1585	3.5113	0.6247	0.8746	0.1600
93	Ni	10.4365	6.9575	3.5035	0.7507	0.5005	0.1596
94	Ni	12.1650	8.6933	3.5090	0.8751	0.6253	0.1599
95	Ni	10.4209	10.4260	3.5103	0.7496	0.7500	0.1599
96	Ni	12.1613	12.1640	3.5107	0.8748	0.8750	0.1599
97	Ni	0.0029	1.7386	5.2281	0.0002	0.1251	0.2382
98	Ni	1.7391	0.0037	5.2295	0.1251	0.0003	0.2382
99	Ni	13.8766	5.1829	5.2154	0.9982	0.3728	0.2376
100	Ni	1.7405	3.4516	5.2097	0.1252	0.2483	0.2373
101	Ni	3.4666	1.7380	5.2291	0.2494	0.1250	0.2382
102	Ni	5.2102	13.8957	5.2263	0.3748	0.9995	0.2381
103	Ni	3.4539	5.2505	5.2198	0.2484	0.3777	0.2378
104	Ni	5.2523	3.4880	5.4262	0.3778	0.2509	0.2472
105	Ni	13.8818	8.7091	5.2289	0.9985	0.6265	0.2382
106	Ni	1.6887	7.0098	5.3641	0.1215	0.5042	0.2444
107	Ni	0.0025	12.1639	5.2276	0.0002	0.8750	0.2381
108	Ni	1.7413	10.4197	5.2218	0.1253	0.7495	0.2379
109	Ni	3.4683	8.7281	5.1067	0.2495	0.6278	0.2326
110	Ni	5.2196	6.9290	5.2101	0.3755	0.4984	0.2374
111	Ni	3.4698	12.1647	5.2292	0.2496	0.8750	0.2382
112	Ni	5.1936	10.4426	5.2099	0.3736	0.7512	0.2373
113	Ni	6.9613	1.7101	5.2201	0.5007	0.1230	0.2378
114	Ni	8.6923	13.8934	5.2265	0.6253	0.9994	0.2381
115	Ni	6.9919	5.2046	5.0941	0.5029	0.3744	0.2321
116	Ni	8.6936	3.4567	5.2694	0.6254	0.2486	0.2401
117	Ni	10.4304	1.7266	5.2219	0.7503	0.1242	0.2379
118	Ni	12.1640	13.8976	5.2261	0.8750	0.9997	0.2381
119	Ni	10.4356	5.1994	5.2886	0.7507	0.3740	0.2409
120	Ni	12.1647	3.4564	5.2214	0.8750	0.2486	0.2379
121	Ni	6.9521	8.6945	5.2739	0.5001	0.6254	0.2403
122	Ni	8.7263	6.9429	5.2406	0.6277	0.4994	0.2387
123	Ni	6.9548	12.1533	5.2386	0.5003	0.8742	0.2387
124	Ni	8.6782	10.4159	5.2427	0.6242	0.7492	0.2388
125	Ni	10.4272	8.7024	5.2310	0.7500	0.6260	0.2383
126	Ni	12.1666	6.9575	5.2232	0.8752	0.5005	0.2380
127	Ni	10.4173	12.1561	5.2332	0.7493	0.8744	0.2384
128	Ni	12.1596	10.4354	5.2261	0.8747	0.7506	0.2381
129	O	5.4388	3.6598	7.3268	0.3912	0.2633	0.3338
130	O	2.5554	6.0924	6.8052	0.1838	0.4382	0.3100
131	O	4.5917	5.9387	8.7340	0.3303	0.4272	0.3979
132	O	8.6386	5.1948	6.2578	0.6214	0.3737	0.2851
133	O	6.0995	7.6610	6.7926	0.4388	0.5511	0.3094
134	O	7.8582	7.3886	8.9855	0.5653	0.5315	0.4093
135	O	5.2774	9.4445	8.8400	0.3796	0.6794	0.4027
136	Ce	3.9473	8.0551	7.8496	0.2839	0.5794	0.3576
137	Ce	3.5184	4.3438	7.7669	0.2531	0.3125	0.3538
138	Ce	6.9209	5.6986	7.9923	0.4978	0.4099	0.3641

139 Sm	7.2494	9.2124	8.1227	0.5215	0.6627	0.3700

/db/jmorales/CoNi-alloy/metal-SDC-interface/CoNi-Ce_3SmO_7/CoNi-Ce_3SmO_7-conf4c

a =			13.9033384264				
b =			13.9033384264				
c =			21.9516692133				
alpha =			90.0				
beta =			90.0				
gamma =			90.0				
	Atom	X	Y	Z	X	Y	Z
1	Ni	0.0000	0.0000	0.0000	0.0000	0.0000	0.0000
2	Ni	0.0000	3.4758	0.0000	0.0000	0.2500	0.0000
3	Ni	3.4758	0.0000	0.0000	0.2500	0.0000	0.0000
4	Ni	3.4758	3.4758	0.0000	0.2500	0.2500	0.0000
5	Ni	0.0000	6.9517	0.0000	0.0000	0.5000	0.0000
6	Ni	0.0000	10.4275	0.0000	0.0000	0.7500	0.0000
7	Ni	3.4758	6.9517	0.0000	0.2500	0.5000	0.0000
8	Ni	3.4758	10.4275	0.0000	0.2500	0.7500	0.0000
9	Ni	6.9517	0.0000	0.0000	0.5000	0.0000	0.0000
10	Ni	6.9517	3.4758	0.0000	0.5000	0.2500	0.0000
11	Ni	10.4275	0.0000	0.0000	0.7500	0.0000	0.0000
12	Ni	10.4275	3.4758	0.0000	0.7500	0.2500	0.0000
13	Ni	6.9517	6.9517	0.0000	0.5000	0.5000	0.0000
14	Ni	6.9517	10.4275	0.0000	0.5000	0.7500	0.0000
15	Ni	10.4275	6.9517	0.0000	0.7500	0.5000	0.0000
16	Ni	10.4275	10.4275	0.0000	0.7500	0.7500	0.0000
17	Ni	0.0000	1.7379	1.7379	0.0000	0.1250	0.0792
18	Ni	0.0000	5.2138	1.7379	0.0000	0.3750	0.0792
19	Ni	3.4758	1.7379	1.7379	0.2500	0.1250	0.0792
20	Ni	3.4758	5.2138	1.7379	0.2500	0.3750	0.0792
21	Ni	0.0000	8.6896	1.7379	0.0000	0.6250	0.0792
22	Ni	0.0000	12.1654	1.7379	0.0000	0.8750	0.0792
23	Ni	3.4758	8.6896	1.7379	0.2500	0.6250	0.0792
24	Ni	3.4758	12.1654	1.7379	0.2500	0.8750	0.0792
25	Ni	6.9517	1.7379	1.7379	0.5000	0.1250	0.0792
26	Ni	6.9517	5.2138	1.7379	0.5000	0.3750	0.0792
27	Ni	10.4275	1.7379	1.7379	0.7500	0.1250	0.0792
28	Ni	10.4275	5.2138	1.7379	0.7500	0.3750	0.0792
29	Ni	6.9517	8.6896	1.7379	0.5000	0.6250	0.0792
30	Ni	6.9517	12.1654	1.7379	0.5000	0.8750	0.0792
31	Ni	10.4275	8.6896	1.7379	0.7500	0.6250	0.0792
32	Ni	10.4275	12.1654	1.7379	0.7500	0.8750	0.0792
33	Ni	-0.0009	0.0002	3.5042	-0.0001	0.0000	0.1596
34	Ni	13.8990	3.4750	3.4993	0.9997	0.2499	0.1594
35	Ni	3.4784	0.0036	3.5053	0.2502	0.0003	0.1597
36	Ni	3.4877	3.4918	3.5104	0.2508	0.2511	0.1599
37	Ni	0.0122	6.9460	3.5239	0.0009	0.4996	0.1605
38	Ni	13.9015	10.4282	3.5028	0.9999	0.7501	0.1596
39	Ni	3.4766	6.9404	3.4959	0.2501	0.4992	0.1593

40	Ni	3.4688	10.4603	3.4645	0.2495	0.7524	0.1578
41	Ni	6.9509	13.9012	3.5031	0.4999	0.9998	0.1596
42	Ni	6.9508	3.4699	3.4855	0.4999	0.2496	0.1588
43	Ni	10.4267	13.9009	3.5033	0.7499	0.9998	0.1596
44	Ni	10.4287	3.4729	3.5020	0.7501	0.2498	0.1595
45	Ni	6.9605	6.9419	3.4856	0.5006	0.4993	0.1588
46	Ni	6.9525	10.4190	3.5061	0.5001	0.7494	0.1597
47	Ni	10.4344	6.9549	3.4982	0.7505	0.5002	0.1594
48	Ni	10.4333	10.4262	3.4992	0.7504	0.7499	0.1594
49	Ni	13.8944	1.7347	5.2094	0.9994	0.1248	0.2373
50	Ni	13.8760	5.1897	5.2080	0.9980	0.3733	0.2372
51	Ni	3.4871	1.7485	5.2201	0.2508	0.1258	0.2378
52	Ni	3.4393	5.1981	5.2665	0.2474	0.3739	0.2399
53	Ni	0.0112	8.6902	5.2202	0.0008	0.6250	0.2378
54	Ni	0.0080	12.1645	5.2081	0.0006	0.8749	0.2373
55	Ni	3.4699	8.7347	5.0632	0.2496	0.6282	0.2307
56	Ni	3.4573	12.1737	5.2023	0.2487	0.8756	0.2370
57	Ni	6.9565	1.7350	5.2019	0.5003	0.1248	0.2370
58	Ni	7.0148	5.1966	5.2248	0.5045	0.3738	0.2380
59	Ni	10.4276	1.7303	5.2062	0.7500	0.1245	0.2372
60	Ni	10.4335	5.2051	5.2133	0.7504	0.3744	0.2375
61	Ni	6.9725	8.6970	5.2185	0.5015	0.6255	0.2377
62	Ni	6.9655	12.1545	5.2189	0.5010	0.8742	0.2377
63	Ni	10.4157	8.6933	5.2163	0.7492	0.6253	0.2376
64	Ni	10.4197	12.1639	5.2135	0.7494	0.8749	0.2375
65	Co	1.7379	1.7379	0.0000	0.1250	0.1250	0.0000
66	Co	1.7379	5.2138	0.0000	0.1250	0.3750	0.0000
67	Co	5.2138	1.7379	0.0000	0.3750	0.1250	0.0000
68	Co	5.2138	5.2138	0.0000	0.3750	0.3750	0.0000
69	Co	1.7379	8.6896	0.0000	0.1250	0.6250	0.0000
70	Co	1.7379	12.1654	0.0000	0.1250	0.8750	0.0000
71	Co	5.2138	8.6896	0.0000	0.3750	0.6250	0.0000
72	Co	5.2138	12.1654	0.0000	0.3750	0.8750	0.0000
73	Co	8.6896	1.7379	0.0000	0.6250	0.1250	0.0000
74	Co	8.6896	5.2138	0.0000	0.6250	0.3750	0.0000
75	Co	12.1654	1.7379	0.0000	0.8750	0.1250	0.0000
76	Co	12.1654	5.2138	0.0000	0.8750	0.3750	0.0000
77	Co	8.6896	8.6896	0.0000	0.6250	0.6250	0.0000
78	Co	8.6896	12.1654	0.0000	0.6250	0.8750	0.0000
79	Co	12.1654	8.6896	0.0000	0.8750	0.6250	0.0000
80	Co	12.1654	12.1654	0.0000	0.8750	0.8750	0.0000
81	Co	1.7379	0.0000	1.7379	0.1250	0.0000	0.0792
82	Co	1.7379	3.4758	1.7379	0.1250	0.2500	0.0792
83	Co	5.2138	0.0000	1.7379	0.3750	0.0000	0.0792
84	Co	5.2138	3.4758	1.7379	0.3750	0.2500	0.0792
85	Co	1.7379	6.9517	1.7379	0.1250	0.5000	0.0792
86	Co	1.7379	10.4275	1.7379	0.1250	0.7500	0.0792
87	Co	5.2138	6.9517	1.7379	0.3750	0.5000	0.0792
88	Co	5.2138	10.4275	1.7379	0.3750	0.7500	0.0792

89	Co	8.6896	0.0000	1.7379	0.6250	0.0000	0.0792
90	Co	8.6896	3.4758	1.7379	0.6250	0.2500	0.0792
91	Co	12.1654	0.0000	1.7379	0.8750	0.0000	0.0792
92	Co	12.1654	3.4758	1.7379	0.8750	0.2500	0.0792
93	Co	8.6896	6.9517	1.7379	0.6250	0.5000	0.0792
94	Co	8.6896	10.4275	1.7379	0.6250	0.7500	0.0792
95	Co	12.1654	6.9517	1.7379	0.8750	0.5000	0.0792
96	Co	12.1654	10.4275	1.7379	0.8750	0.7500	0.0792
97	Co	1.7433	1.7363	3.5488	0.1254	0.1249	0.1617
98	Co	1.7399	5.2181	3.5684	0.1251	0.3753	0.1626
99	Co	5.2127	1.7375	3.5498	0.3749	0.1250	0.1617
100	Co	5.2023	5.2243	3.4946	0.3742	0.3758	0.1592
101	Co	1.7053	8.6673	3.5278	0.1227	0.6234	0.1607
102	Co	1.7300	12.1763	3.5476	0.1244	0.8758	0.1616
103	Co	5.2286	8.7018	3.5054	0.3761	0.6259	0.1597
104	Co	5.2200	12.1792	3.5515	0.3754	0.8760	0.1618
105	Co	8.6896	1.7335	3.5507	0.6250	0.1247	0.1618
106	Co	8.6918	5.2068	3.5546	0.6252	0.3745	0.1619
107	Co	12.1641	1.7324	3.5468	0.8749	0.1246	0.1616
108	Co	12.1654	5.2122	3.5485	0.8750	0.3749	0.1617
109	Co	8.6862	8.6894	3.5476	0.6248	0.6250	0.1616
110	Co	8.6915	12.1697	3.5391	0.6251	0.8753	0.1612
111	Co	12.1686	8.6902	3.5640	0.8752	0.6250	0.1624
112	Co	12.1647	12.1661	3.5584	0.8749	0.8751	0.1621
113	Co	1.7433	0.0191	5.2317	0.1254	0.0014	0.2383
114	Co	1.7247	3.4265	5.1993	0.1240	0.2464	0.2369
115	Co	5.2173	0.0006	5.2327	0.3753	0.0000	0.2384
116	Co	5.2328	3.4672	5.2385	0.3764	0.2494	0.2386
117	Co	1.7530	6.9684	5.3687	0.1261	0.5012	0.2446
118	Co	1.7499	10.4150	5.2084	0.1259	0.7491	0.2373
119	Co	5.2213	6.9885	5.2245	0.3755	0.5027	0.2380
120	Co	5.1812	10.4707	5.1949	0.3727	0.7531	0.2367
121	Co	8.6826	13.8998	5.2293	0.6245	0.9997	0.2382
122	Co	8.6998	3.4622	5.2141	0.6257	0.2490	0.2375
123	Co	12.1632	13.8984	5.2251	0.8748	0.9996	0.2380
124	Co	12.1624	3.4662	5.2219	0.8748	0.2493	0.2379
125	Co	8.7301	6.9267	5.1961	0.6279	0.4982	0.2367
126	Co	8.6915	10.4344	5.2118	0.6251	0.7505	0.2374
127	Co	12.1639	6.9559	5.2191	0.8749	0.5003	0.2378
128	Co	12.1653	10.4264	5.2208	0.8750	0.7499	0.2378
129	O	5.3735	5.1278	6.3429	0.3865	0.3688	0.2889
130	O	6.0099	4.2671	8.8772	0.4323	0.3069	0.4044
131	O	2.6339	6.1954	6.8352	0.1894	0.4456	0.3114
132	O	4.6668	6.5808	8.8618	0.3357	0.4733	0.4037
133	O	6.0755	7.9044	6.8142	0.4370	0.5685	0.3104
134	O	7.7934	7.7776	9.0050	0.5605	0.5594	0.4102
135	O	5.2071	9.9920	8.5163	0.3745	0.7187	0.3880
136	Ce	3.8572	8.4207	7.8140	0.2774	0.6057	0.3560
137	Ce	3.9141	4.5776	8.1537	0.2815	0.3292	0.3714

138 Ce	6.8389	6.0875	8.2426	0.4919	0.4378	0.3755
139 Sm	7.1862	9.5969	7.9500	0.5169	0.6903	0.3622

/db/jmorales/CoNi-alloy/metal-SDC-interface/Ni-Ce_3SmO_7/Ni-Ce_3SmO_7-conf6b

a = 13.9019837372
b = 13.9019837372
c = 21.9509918687
alpha = 90.0
beta = 90.0
gamma = 90.0

	Atom	X	Y	Z	X	Y	Z
1	Ni	0.0000	0.0000	0.0000	0.0000	0.0000	0.0000
2	Ni	1.7377	1.7377	0.0000	0.1250	0.1250	0.0000
3	Ni	0.0000	3.4755	0.0000	0.0000	0.2500	0.0000
4	Ni	1.7377	5.2132	0.0000	0.1250	0.3750	0.0000
5	Ni	3.4755	0.0000	0.0000	0.2500	0.0000	0.0000
6	Ni	5.2132	1.7377	0.0000	0.3750	0.1250	0.0000
7	Ni	3.4755	3.4755	0.0000	0.2500	0.2500	0.0000
8	Ni	5.2132	5.2132	0.0000	0.3750	0.3750	0.0000
9	Ni	0.0000	6.9510	0.0000	0.0000	0.5000	0.0000
10	Ni	1.7377	8.6887	0.0000	0.1250	0.6250	0.0000
11	Ni	0.0000	10.4265	0.0000	0.0000	0.7500	0.0000
12	Ni	1.7377	12.1642	0.0000	0.1250	0.8750	0.0000
13	Ni	3.4755	6.9510	0.0000	0.2500	0.5000	0.0000
14	Ni	5.2132	8.6887	0.0000	0.3750	0.6250	0.0000
15	Ni	3.4755	10.4265	0.0000	0.2500	0.7500	0.0000
16	Ni	5.2132	12.1642	0.0000	0.3750	0.8750	0.0000
17	Ni	6.9510	0.0000	0.0000	0.5000	0.0000	0.0000
18	Ni	8.6887	1.7377	0.0000	0.6250	0.1250	0.0000
19	Ni	6.9510	3.4755	0.0000	0.5000	0.2500	0.0000
20	Ni	8.6887	5.2132	0.0000	0.6250	0.3750	0.0000
21	Ni	10.4265	0.0000	0.0000	0.7500	0.0000	0.0000
22	Ni	12.1642	1.7377	0.0000	0.8750	0.1250	0.0000
23	Ni	10.4265	3.4755	0.0000	0.7500	0.2500	0.0000
24	Ni	12.1642	5.2132	0.0000	0.8750	0.3750	0.0000
25	Ni	6.9510	6.9510	0.0000	0.5000	0.5000	0.0000
26	Ni	8.6887	8.6887	0.0000	0.6250	0.6250	0.0000
27	Ni	6.9510	10.4265	0.0000	0.5000	0.7500	0.0000
28	Ni	8.6887	12.1642	0.0000	0.6250	0.8750	0.0000
29	Ni	10.4265	6.9510	0.0000	0.7500	0.5000	0.0000
30	Ni	12.1642	8.6887	0.0000	0.8750	0.6250	0.0000
31	Ni	10.4265	10.4265	0.0000	0.7500	0.7500	0.0000
32	Ni	12.1642	12.1642	0.0000	0.8750	0.8750	0.0000
33	Ni	0.0000	1.7377	1.7377	0.0000	0.1250	0.0792
34	Ni	1.7377	0.0000	1.7377	0.1250	0.0000	0.0792
35	Ni	0.0000	5.2132	1.7377	0.0000	0.3750	0.0792
36	Ni	1.7377	3.4755	1.7377	0.1250	0.2500	0.0792
37	Ni	3.4755	1.7377	1.7377	0.2500	0.1250	0.0792
38	Ni	5.2132	0.0000	1.7377	0.3750	0.0000	0.0792
39	Ni	3.4755	5.2132	1.7377	0.2500	0.3750	0.0792
40	Ni	5.2132	3.4755	1.7377	0.3750	0.2500	0.0792

41	Ni	0.0000	8.6887	1.7377	0.0000	0.6250	0.0792
42	Ni	1.7377	6.9510	1.7377	0.1250	0.5000	0.0792
43	Ni	0.0000	12.1642	1.7377	0.0000	0.8750	0.0792
44	Ni	1.7377	10.4265	1.7377	0.1250	0.7500	0.0792
45	Ni	3.4755	8.6887	1.7377	0.2500	0.6250	0.0792
46	Ni	5.2132	6.9510	1.7377	0.3750	0.5000	0.0792
47	Ni	3.4755	12.1642	1.7377	0.2500	0.8750	0.0792
48	Ni	5.2132	10.4265	1.7377	0.3750	0.7500	0.0792
49	Ni	6.9510	1.7377	1.7377	0.5000	0.1250	0.0792
50	Ni	8.6887	0.0000	1.7377	0.6250	0.0000	0.0792
51	Ni	6.9510	5.2132	1.7377	0.5000	0.3750	0.0792
52	Ni	8.6887	3.4755	1.7377	0.6250	0.2500	0.0792
53	Ni	10.4265	1.7377	1.7377	0.7500	0.1250	0.0792
54	Ni	12.1642	0.0000	1.7377	0.8750	0.0000	0.0792
55	Ni	10.4265	5.2132	1.7377	0.7500	0.3750	0.0792
56	Ni	12.1642	3.4755	1.7377	0.8750	0.2500	0.0792
57	Ni	6.9510	8.6887	1.7377	0.5000	0.6250	0.0792
58	Ni	8.6887	6.9510	1.7377	0.6250	0.5000	0.0792
59	Ni	6.9510	12.1642	1.7377	0.5000	0.8750	0.0792
60	Ni	8.6887	10.4265	1.7377	0.6250	0.7500	0.0792
61	Ni	10.4265	8.6887	1.7377	0.7500	0.6250	0.0792
62	Ni	12.1642	6.9510	1.7377	0.8750	0.5000	0.0792
63	Ni	10.4265	12.1642	1.7377	0.7500	0.8750	0.0792
64	Ni	12.1642	10.4265	1.7377	0.8750	0.7500	0.0792
65	Ni	13.8958	13.8956	3.5051	0.9996	0.9995	0.1597
66	Ni	1.7471	1.7388	3.5169	0.1257	0.1251	0.1602
67	Ni	0.0112	3.4664	3.5178	0.0008	0.2493	0.1603
68	Ni	1.7220	5.2039	3.5077	0.1239	0.3743	0.1598
69	Ni	3.4765	0.0220	3.5349	0.2501	0.0016	0.1610
70	Ni	5.1969	1.7490	3.5356	0.3738	0.1258	0.1611
71	Ni	3.4839	3.4841	3.5178	0.2506	0.2506	0.1603
72	Ni	5.2212	5.2297	3.5009	0.3756	0.3762	0.1595
73	Ni	0.0166	6.9460	3.5394	0.0012	0.4996	0.1612
74	Ni	1.7054	8.6732	3.5040	0.1227	0.6239	0.1596
75	Ni	13.8953	10.4254	3.5091	0.9995	0.7499	0.1599
76	Ni	1.7322	12.1693	3.5055	0.1246	0.8754	0.1597
77	Ni	3.4708	6.9708	3.4694	0.2497	0.5014	0.1581
78	Ni	5.2307	8.7035	3.4709	0.3763	0.6261	0.1581
79	Ni	3.4622	10.4564	3.4727	0.2490	0.7521	0.1582
80	Ni	5.2144	12.1764	3.5020	0.3751	0.8759	0.1595
81	Ni	6.9518	13.8963	3.5060	0.5001	0.9996	0.1597
82	Ni	8.6989	1.7221	3.5040	0.6257	0.1239	0.1596
83	Ni	6.9591	3.4605	3.4902	0.5006	0.2489	0.1590
84	Ni	8.6808	5.2004	3.5120	0.6244	0.3741	0.1600
85	Ni	10.4266	13.8903	3.5096	0.7500	0.9992	0.1599
86	Ni	12.1586	1.7311	3.5019	0.8746	0.1245	0.1595
87	Ni	10.4330	3.4717	3.5016	0.7505	0.2497	0.1595
88	Ni	12.1652	5.2135	3.5073	0.8751	0.3750	0.1598
89	Ni	6.9631	6.9291	3.4969	0.5009	0.4984	0.1593

90	Ni	8.6823	8.6883	3.5092	0.6245	0.6250	0.1599
91	Ni	6.9564	10.4268	3.5027	0.5004	0.7500	0.1596
92	Ni	8.6910	12.1635	3.5073	0.6252	0.8749	0.1598
93	Ni	10.4338	6.9513	3.5040	0.7505	0.5000	0.1596
94	Ni	12.1634	8.6832	3.5165	0.8749	0.6246	0.1602
95	Ni	10.4303	10.4201	3.5085	0.7503	0.7495	0.1598
96	Ni	12.1593	12.1566	3.5099	0.8746	0.8744	0.1599
97	Ni	13.8649	1.7170	5.2081	0.9973	0.1235	0.2373
98	Ni	1.7161	13.8746	5.2134	0.1234	0.9980	0.2375
99	Ni	13.8747	5.1961	5.2148	0.9980	0.3738	0.2376
100	Ni	1.7014	3.4544	5.3412	0.1224	0.2485	0.2433
101	Ni	3.5035	1.7181	5.3904	0.2520	0.1236	0.2456
102	Ni	5.2273	13.8866	5.2310	0.3760	0.9989	0.2383
103	Ni	3.4764	5.2833	5.1719	0.2501	0.3800	0.2356
104	Ni	5.2601	3.4653	5.2708	0.3784	0.2493	0.2401
105	Ni	13.8977	8.6957	5.2382	0.9997	0.6255	0.2386
106	Ni	1.7243	6.9818	5.3958	0.1240	0.5022	0.2458
107	Ni	13.8926	12.1535	5.2265	0.9993	0.8742	0.2381
108	Ni	1.7374	10.4245	5.2168	0.1250	0.7499	0.2377
109	Ni	3.4531	8.7396	5.0943	0.2484	0.6287	0.2321
110	Ni	5.2140	7.0069	5.2183	0.3751	0.5040	0.2377
111	Ni	3.4712	12.1642	5.2254	0.2497	0.8750	0.2380
112	Ni	5.1928	10.4533	5.1788	0.3735	0.7519	0.2359
113	Ni	6.9748	1.7029	5.2021	0.5017	0.1225	0.2370
114	Ni	8.6932	13.8801	5.2338	0.6253	0.9984	0.2384
115	Ni	7.0017	5.1771	5.2969	0.5036	0.3724	0.2413
116	Ni	8.7282	3.4475	5.2048	0.6278	0.2480	0.2371
117	Ni	10.4332	1.7218	5.2255	0.7505	0.1239	0.2381
118	Ni	12.1590	13.8887	5.2254	0.8746	0.9990	0.2380
119	Ni	10.4264	5.2120	5.2267	0.7500	0.3749	0.2381
120	Ni	12.1570	3.4711	5.2200	0.8745	0.2497	0.2378
121	Ni	7.0024	8.7054	5.2491	0.5037	0.6262	0.2391
122	Ni	8.7097	6.9241	5.1920	0.6265	0.4981	0.2365
123	Ni	6.9559	12.1405	5.2354	0.5004	0.8733	0.2385
124	Ni	8.6923	10.4366	5.2148	0.6253	0.7507	0.2376
125	Ni	10.4075	8.6878	5.2372	0.7486	0.6249	0.2386
126	Ni	12.1601	6.9527	5.2320	0.8747	0.5001	0.2383
127	Ni	10.4239	12.1534	5.2350	0.7498	0.8742	0.2385
128	Ni	12.1582	10.4213	5.2315	0.8746	0.7496	0.2383
129	O	2.6904	2.7099	6.7238	0.1935	0.1949	0.3063
130	O	6.0109	4.3994	6.7936	0.4324	0.3165	0.3095
131	O	2.7651	6.1179	6.7921	0.1989	0.4401	0.3094
132	O	4.9249	6.2644	8.8393	0.3543	0.4506	0.4027
133	O	6.0037	7.9755	6.7680	0.4319	0.5737	0.3083
134	O	7.9074	8.2245	8.8477	0.5688	0.5916	0.4031
135	O	5.0240	9.8329	8.7165	0.3614	0.7073	0.3971
136	Ce	3.8918	8.1559	7.9293	0.2799	0.5867	0.3612
137	Ce	3.9297	4.4083	7.8216	0.2827	0.3171	0.3563
138	Ce	7.1247	6.3654	8.0780	0.5125	0.4579	0.3680

139 Sm	6.9749	9.8838	7.9220	0.5017	0.7110	0.3609

/db/jmorales/CoNi-alloy/metal-SDC-interface/Ni-Ce_3SmO_7/Ni-Ce_3SmO_7-conf1c

a =	13.9019837372
b =	13.9019837372
c =	21.9509918687
alpha =	90.0
beta =	90.0
gamma =	90.0

	Atom	X	Y	Z	X	Y	Z
1	Ni	0.0000	0.0000	0.0000	0.0000	0.0000	0.0000
2	Ni	1.7377	1.7377	0.0000	0.1250	0.1250	0.0000
3	Ni	0.0000	3.4755	0.0000	0.0000	0.2500	0.0000
4	Ni	1.7377	5.2132	0.0000	0.1250	0.3750	0.0000
5	Ni	3.4755	0.0000	0.0000	0.2500	0.0000	0.0000
6	Ni	5.2132	1.7377	0.0000	0.3750	0.1250	0.0000
7	Ni	3.4755	3.4755	0.0000	0.2500	0.2500	0.0000
8	Ni	5.2132	5.2132	0.0000	0.3750	0.3750	0.0000
9	Ni	0.0000	6.9510	0.0000	0.0000	0.5000	0.0000
10	Ni	1.7377	8.6887	0.0000	0.1250	0.6250	0.0000
11	Ni	0.0000	10.4265	0.0000	0.0000	0.7500	0.0000
12	Ni	1.7377	12.1642	0.0000	0.1250	0.8750	0.0000
13	Ni	3.4755	6.9510	0.0000	0.2500	0.5000	0.0000
14	Ni	5.2132	8.6887	0.0000	0.3750	0.6250	0.0000
15	Ni	3.4755	10.4265	0.0000	0.2500	0.7500	0.0000
16	Ni	5.2132	12.1642	0.0000	0.3750	0.8750	0.0000
17	Ni	6.9510	0.0000	0.0000	0.5000	0.0000	0.0000
18	Ni	8.6887	1.7377	0.0000	0.6250	0.1250	0.0000
19	Ni	6.9510	3.4755	0.0000	0.5000	0.2500	0.0000
20	Ni	8.6887	5.2132	0.0000	0.6250	0.3750	0.0000
21	Ni	10.4265	0.0000	0.0000	0.7500	0.0000	0.0000
22	Ni	12.1642	1.7377	0.0000	0.8750	0.1250	0.0000
23	Ni	10.4265	3.4755	0.0000	0.7500	0.2500	0.0000
24	Ni	12.1642	5.2132	0.0000	0.8750	0.3750	0.0000
25	Ni	6.9510	6.9510	0.0000	0.5000	0.5000	0.0000
26	Ni	8.6887	8.6887	0.0000	0.6250	0.6250	0.0000
27	Ni	6.9510	10.4265	0.0000	0.5000	0.7500	0.0000
28	Ni	8.6887	12.1642	0.0000	0.6250	0.8750	0.0000
29	Ni	10.4265	6.9510	0.0000	0.7500	0.5000	0.0000
30	Ni	12.1642	8.6887	0.0000	0.8750	0.6250	0.0000
31	Ni	10.4265	10.4265	0.0000	0.7500	0.7500	0.0000
32	Ni	12.1642	12.1642	0.0000	0.8750	0.8750	0.0000
33	Ni	0.0000	1.7377	1.7377	0.0000	0.1250	0.0792
34	Ni	1.7377	0.0000	1.7377	0.1250	0.0000	0.0792
35	Ni	0.0000	5.2132	1.7377	0.0000	0.3750	0.0792
36	Ni	1.7377	3.4755	1.7377	0.1250	0.2500	0.0792
37	Ni	3.4755	1.7377	1.7377	0.2500	0.1250	0.0792
38	Ni	5.2132	0.0000	1.7377	0.3750	0.0000	0.0792
39	Ni	3.4755	5.2132	1.7377	0.2500	0.3750	0.0792
40	Ni	5.2132	3.4755	1.7377	0.3750	0.2500	0.0792

41	Ni	0.0000	8.6887	1.7377	0.0000	0.6250	0.0792
42	Ni	1.7377	6.9510	1.7377	0.1250	0.5000	0.0792
43	Ni	0.0000	12.1642	1.7377	0.0000	0.8750	0.0792
44	Ni	1.7377	10.4265	1.7377	0.1250	0.7500	0.0792
45	Ni	3.4755	8.6887	1.7377	0.2500	0.6250	0.0792
46	Ni	5.2132	6.9510	1.7377	0.3750	0.5000	0.0792
47	Ni	3.4755	12.1642	1.7377	0.2500	0.8750	0.0792
48	Ni	5.2132	10.4265	1.7377	0.3750	0.7500	0.0792
49	Ni	6.9510	1.7377	1.7377	0.5000	0.1250	0.0792
50	Ni	8.6887	0.0000	1.7377	0.6250	0.0000	0.0792
51	Ni	6.9510	5.2132	1.7377	0.5000	0.3750	0.0792
52	Ni	8.6887	3.4755	1.7377	0.6250	0.2500	0.0792
53	Ni	10.4265	1.7377	1.7377	0.7500	0.1250	0.0792
54	Ni	12.1642	0.0000	1.7377	0.8750	0.0000	0.0792
55	Ni	10.4265	5.2132	1.7377	0.7500	0.3750	0.0792
56	Ni	12.1642	3.4755	1.7377	0.8750	0.2500	0.0792
57	Ni	6.9510	8.6887	1.7377	0.5000	0.6250	0.0792
58	Ni	8.6887	6.9510	1.7377	0.6250	0.5000	0.0792
59	Ni	6.9510	12.1642	1.7377	0.5000	0.8750	0.0792
60	Ni	8.6887	10.4265	1.7377	0.6250	0.7500	0.0792
61	Ni	10.4265	8.6887	1.7377	0.7500	0.6250	0.0792
62	Ni	12.1642	6.9510	1.7377	0.8750	0.5000	0.0792
63	Ni	10.4265	12.1642	1.7377	0.7500	0.8750	0.0792
64	Ni	12.1642	10.4265	1.7377	0.8750	0.7500	0.0792
65	Ni	0.0032	0.0026	3.5085	0.0002	0.0002	0.1598
66	Ni	1.7352	1.7354	3.5090	0.1248	0.1248	0.1599
67	Ni	0.0022	3.4785	3.5132	0.0002	0.2502	0.1600
68	Ni	1.7496	5.2215	3.5349	0.1259	0.3756	0.1610
69	Ni	3.4653	13.8923	3.5094	0.2493	0.9993	0.1599
70	Ni	5.1995	1.7039	3.4729	0.3740	0.1226	0.1582
71	Ni	3.4399	3.4929	3.4992	0.2474	0.2513	0.1594
72	Ni	5.2051	5.2142	3.4923	0.3744	0.3751	0.1591
73	Ni	13.9002	6.9481	3.5064	0.9999	0.4998	0.1597
74	Ni	1.7293	8.6974	3.5035	0.1244	0.6256	0.1596
75	Ni	13.8809	10.4340	3.5287	0.9985	0.7505	0.1608
76	Ni	1.7348	12.1610	3.5094	0.1248	0.8748	0.1599
77	Ni	3.4664	6.9472	3.5078	0.2493	0.4997	0.1598
78	Ni	5.2299	8.6667	3.5247	0.3762	0.6234	0.1606
79	Ni	3.4783	10.4277	3.5037	0.2502	0.7501	0.1596
80	Ni	5.2123	12.1616	3.5091	0.3749	0.8748	0.1599
81	Ni	6.9493	13.8995	3.5031	0.4999	0.9998	0.1596
82	Ni	8.6947	1.7430	3.5222	0.6254	0.1254	0.1605
83	Ni	6.9754	3.4605	3.4819	0.5018	0.2489	0.1586
84	Ni	8.7029	5.2204	3.5038	0.6260	0.3755	0.1596
85	Ni	10.4298	13.8861	3.5254	0.7502	0.9989	0.1606
86	Ni	12.1637	1.7409	3.5091	0.8750	0.1252	0.1599
87	Ni	10.4060	3.4700	3.5300	0.7485	0.2496	0.1608
88	Ni	12.1667	5.2103	3.5040	0.8752	0.3748	0.1596
89	Ni	6.9391	6.9277	3.5170	0.4991	0.4983	0.1602

90	Ni	8.6835	8.6924	3.4753	0.6246	0.6253	0.1583
91	Ni	6.9559	10.4156	3.5201	0.5004	0.7492	0.1604
92	Ni	8.6865	12.1829	3.4912	0.6248	0.8763	0.1590
93	Ni	10.4249	6.9750	3.5153	0.7499	0.5017	0.1601
94	Ni	12.1757	8.6941	3.4987	0.8758	0.6254	0.1594
95	Ni	10.4405	10.4395	3.4373	0.7510	0.7509	0.1566
96	Ni	12.1665	12.1691	3.5090	0.8752	0.8754	0.1599
97	Ni	13.8994	1.7376	5.2268	0.9998	0.1250	0.2381
98	Ni	1.7363	0.0016	5.2291	0.1249	0.0001	0.2382
99	Ni	-0.0001	5.2129	5.2291	-0.0000	0.3750	0.2382
100	Ni	1.7320	3.4665	5.2353	0.1246	0.2494	0.2385
101	Ni	3.4746	1.7385	5.2198	0.2499	0.1251	0.2378
102	Ni	5.2007	13.8980	5.2260	0.3741	0.9997	0.2381
103	Ni	3.4355	5.1993	5.3794	0.2471	0.3740	0.2451
104	Ni	5.1800	3.4300	5.0812	0.3726	0.2467	0.2315
105	Ni	13.8937	8.6924	5.2237	0.9994	0.6253	0.2380
106	Ni	1.7096	6.9818	5.2159	0.1230	0.5022	0.2376
107	Ni	0.0083	12.1709	5.2268	0.0006	0.8755	0.2381
108	Ni	1.7337	10.4244	5.2277	0.1247	0.7498	0.2382
109	Ni	3.4738	8.7132	5.2028	0.2499	0.6268	0.2370
110	Ni	5.1901	6.9268	5.2693	0.3733	0.4983	0.2400
111	Ni	3.4736	12.1582	5.2269	0.2499	0.8746	0.2381
112	Ni	5.2114	10.4196	5.2248	0.3749	0.7495	0.2380
113	Ni	6.9149	1.7112	5.2006	0.4974	0.1231	0.2369
114	Ni	8.6794	0.0072	5.2226	0.6243	0.0005	0.2379
115	Ni	6.9667	5.1629	5.2231	0.5011	0.3714	0.2379
116	Ni	8.7447	3.4345	5.3769	0.6290	0.2471	0.2450
117	Ni	10.4446	1.7154	5.2306	0.7513	0.1234	0.2383
118	Ni	12.1768	13.9010	5.2245	0.8759	0.9999	0.2380
119	Ni	10.4421	5.2326	5.2037	0.7511	0.3764	0.2371
120	Ni	12.1557	3.4807	5.2315	0.8744	0.2504	0.2383
121	Ni	6.9417	8.7069	5.2795	0.4993	0.6263	0.2405
122	Ni	8.7328	6.9684	5.2412	0.6282	0.5013	0.2388
123	Ni	6.9456	12.1649	5.2197	0.4996	0.8750	0.2378
124	Ni	8.6954	10.4605	5.1813	0.6255	0.7524	0.2360
125	Ni	10.4566	8.7155	5.2182	0.7522	0.6269	0.2377
126	Ni	12.1629	6.9481	5.2235	0.8749	0.4998	0.2380
127	Ni	10.4247	12.1842	5.2901	0.7499	0.8764	0.2410
128	Ni	12.1663	10.4456	5.2917	0.8751	0.7514	0.2411
129	O	7.6308	4.1497	6.7571	0.5489	0.2985	0.3078
130	O	4.3885	6.0915	6.8370	0.3157	0.4382	0.3115
131	O	6.6368	5.9627	8.7644	0.4774	0.4289	0.3993
132	O	7.8480	7.8094	6.8068	0.5645	0.5617	0.3101
133	O	9.7535	7.6635	8.9154	0.7016	0.5513	0.4062
134	O	6.9693	9.5394	8.8672	0.5013	0.6862	0.4040
135	O	10.3592	10.4246	6.2853	0.7452	0.7499	0.2863
136	Ce	5.7302	8.0240	8.0271	0.4122	0.5772	0.3657
137	Ce	5.5218	4.3166	7.6759	0.3972	0.3105	0.3497
138	Ce	8.8547	5.9384	8.0079	0.6369	0.4272	0.3648

139 Sm	8.9598	9.4309	8.0403	0.6445	0.6784	0.3663

/db/jmorales/CoNi-alloy/metal-SDC-interface/Ni-Ce_3SmO_7/Ni-Ce_3SmO_7-conf6a

a = 13.9019837372
b = 13.9019837372
c = 21.9509918687
alpha = 90.0
beta = 90.0
gamma = 90.0

	Atom	X	Y	Z	X	Y	Z
1	Ni	0.0000	0.0000	0.0000	0.0000	0.0000	0.0000
2	Ni	1.7377	1.7377	0.0000	0.1250	0.1250	0.0000
3	Ni	0.0000	3.4755	0.0000	0.0000	0.2500	0.0000
4	Ni	1.7377	5.2132	0.0000	0.1250	0.3750	0.0000
5	Ni	3.4755	0.0000	0.0000	0.2500	0.0000	0.0000
6	Ni	5.2132	1.7377	0.0000	0.3750	0.1250	0.0000
7	Ni	3.4755	3.4755	0.0000	0.2500	0.2500	0.0000
8	Ni	5.2132	5.2132	0.0000	0.3750	0.3750	0.0000
9	Ni	0.0000	6.9510	0.0000	0.0000	0.5000	0.0000
10	Ni	1.7377	8.6887	0.0000	0.1250	0.6250	0.0000
11	Ni	0.0000	10.4265	0.0000	0.0000	0.7500	0.0000
12	Ni	1.7377	12.1642	0.0000	0.1250	0.8750	0.0000
13	Ni	3.4755	6.9510	0.0000	0.2500	0.5000	0.0000
14	Ni	5.2132	8.6887	0.0000	0.3750	0.6250	0.0000
15	Ni	3.4755	10.4265	0.0000	0.2500	0.7500	0.0000
16	Ni	5.2132	12.1642	0.0000	0.3750	0.8750	0.0000
17	Ni	6.9510	0.0000	0.0000	0.5000	0.0000	0.0000
18	Ni	8.6887	1.7377	0.0000	0.6250	0.1250	0.0000
19	Ni	6.9510	3.4755	0.0000	0.5000	0.2500	0.0000
20	Ni	8.6887	5.2132	0.0000	0.6250	0.3750	0.0000
21	Ni	10.4265	0.0000	0.0000	0.7500	0.0000	0.0000
22	Ni	12.1642	1.7377	0.0000	0.8750	0.1250	0.0000
23	Ni	10.4265	3.4755	0.0000	0.7500	0.2500	0.0000
24	Ni	12.1642	5.2132	0.0000	0.8750	0.3750	0.0000
25	Ni	6.9510	6.9510	0.0000	0.5000	0.5000	0.0000
26	Ni	8.6887	8.6887	0.0000	0.6250	0.6250	0.0000
27	Ni	6.9510	10.4265	0.0000	0.5000	0.7500	0.0000
28	Ni	8.6887	12.1642	0.0000	0.6250	0.8750	0.0000
29	Ni	10.4265	6.9510	0.0000	0.7500	0.5000	0.0000
30	Ni	12.1642	8.6887	0.0000	0.8750	0.6250	0.0000
31	Ni	10.4265	10.4265	0.0000	0.7500	0.7500	0.0000
32	Ni	12.1642	12.1642	0.0000	0.8750	0.8750	0.0000
33	Ni	0.0000	1.7377	1.7377	0.0000	0.1250	0.0792
34	Ni	1.7377	0.0000	1.7377	0.1250	0.0000	0.0792
35	Ni	0.0000	5.2132	1.7377	0.0000	0.3750	0.0792
36	Ni	1.7377	3.4755	1.7377	0.1250	0.2500	0.0792
37	Ni	3.4755	1.7377	1.7377	0.2500	0.1250	0.0792
38	Ni	5.2132	0.0000	1.7377	0.3750	0.0000	0.0792
39	Ni	3.4755	5.2132	1.7377	0.2500	0.3750	0.0792
40	Ni	5.2132	3.4755	1.7377	0.3750	0.2500	0.0792

41	Ni	0.0000	8.6887	1.7377	0.0000	0.6250	0.0792
42	Ni	1.7377	6.9510	1.7377	0.1250	0.5000	0.0792
43	Ni	0.0000	12.1642	1.7377	0.0000	0.8750	0.0792
44	Ni	1.7377	10.4265	1.7377	0.1250	0.7500	0.0792
45	Ni	3.4755	8.6887	1.7377	0.2500	0.6250	0.0792
46	Ni	5.2132	6.9510	1.7377	0.3750	0.5000	0.0792
47	Ni	3.4755	12.1642	1.7377	0.2500	0.8750	0.0792
48	Ni	5.2132	10.4265	1.7377	0.3750	0.7500	0.0792
49	Ni	6.9510	1.7377	1.7377	0.5000	0.1250	0.0792
50	Ni	8.6887	0.0000	1.7377	0.6250	0.0000	0.0792
51	Ni	6.9510	5.2132	1.7377	0.5000	0.3750	0.0792
52	Ni	8.6887	3.4755	1.7377	0.6250	0.2500	0.0792
53	Ni	10.4265	1.7377	1.7377	0.7500	0.1250	0.0792
54	Ni	12.1642	0.0000	1.7377	0.8750	0.0000	0.0792
55	Ni	10.4265	5.2132	1.7377	0.7500	0.3750	0.0792
56	Ni	12.1642	3.4755	1.7377	0.8750	0.2500	0.0792
57	Ni	6.9510	8.6887	1.7377	0.5000	0.6250	0.0792
58	Ni	8.6887	6.9510	1.7377	0.6250	0.5000	0.0792
59	Ni	6.9510	12.1642	1.7377	0.5000	0.8750	0.0792
60	Ni	8.6887	10.4265	1.7377	0.6250	0.7500	0.0792
61	Ni	10.4265	8.6887	1.7377	0.7500	0.6250	0.0792
62	Ni	12.1642	6.9510	1.7377	0.8750	0.5000	0.0792
63	Ni	10.4265	12.1642	1.7377	0.7500	0.8750	0.0792
64	Ni	12.1642	10.4265	1.7377	0.8750	0.7500	0.0792
65	Ni	0.0046	13.8973	3.5129	0.0003	0.9997	0.1600
66	Ni	1.7053	1.7501	3.4391	0.1227	0.1259	0.1567
67	Ni	0.0129	3.4817	3.5340	0.0009	0.2504	0.1610
68	Ni	1.7274	5.2080	3.5133	0.1243	0.3746	0.1601
69	Ni	3.4624	13.8598	3.4697	0.2491	0.9970	0.1581
70	Ni	5.2137	1.7216	3.4945	0.3750	0.1238	0.1592
71	Ni	3.4710	3.4863	3.4783	0.2497	0.2508	0.1585
72	Ni	5.2102	5.2007	3.5122	0.3748	0.3741	0.1600
73	Ni	13.8894	6.9602	3.5027	0.9991	0.5007	0.1596
74	Ni	1.7379	8.6916	3.5023	0.1250	0.6252	0.1596
75	Ni	13.8992	10.4256	3.5086	0.9998	0.7499	0.1598
76	Ni	1.7291	12.1635	3.5249	0.1244	0.8749	0.1606
77	Ni	3.4906	6.9365	3.5167	0.2511	0.4990	0.1602
78	Ni	5.2097	8.6786	3.5090	0.3747	0.6243	0.1599
79	Ni	3.4773	10.4216	3.5075	0.2501	0.7497	0.1598
80	Ni	5.2208	12.1509	3.5036	0.3755	0.8740	0.1596
81	Ni	6.9522	0.0104	3.5275	0.5001	0.0008	0.1607
82	Ni	8.6616	1.7332	3.5362	0.6230	0.1247	0.1611
83	Ni	6.9554	3.4768	3.5082	0.5003	0.2501	0.1598
84	Ni	8.6833	5.2165	3.5059	0.6246	0.3752	0.1597
85	Ni	10.4293	0.0119	3.5128	0.7502	0.0009	0.1600
86	Ni	12.1964	1.7387	3.5400	0.8773	0.1251	0.1613
87	Ni	10.4328	3.4678	3.5068	0.7505	0.2494	0.1598
88	Ni	12.1568	5.2112	3.5076	0.8745	0.3749	0.1598
89	Ni	6.9536	6.9588	3.4822	0.5002	0.5006	0.1586

90	Ni	8.7093	8.7032	3.4864	0.6265	0.6260	0.1588
91	Ni	6.9569	10.4455	3.4942	0.5004	0.7514	0.1592
92	Ni	8.6924	12.1701	3.5123	0.6253	0.8754	0.1600
93	Ni	10.4403	6.9529	3.4990	0.7510	0.5001	0.1594
94	Ni	12.1586	8.6973	3.5114	0.8746	0.6256	0.1600
95	Ni	10.4279	10.4360	3.5103	0.7501	0.7507	0.1599
96	Ni	12.1614	12.1658	3.5089	0.8748	0.8751	0.1599
97	Ni	0.0450	1.7317	5.3111	0.0032	0.1246	0.2420
98	Ni	1.7460	13.8465	5.3036	0.1256	0.9960	0.2416
99	Ni	13.8636	5.2509	5.2111	0.9972	0.3777	0.2374
100	Ni	1.7021	3.5342	5.3908	0.1224	0.2542	0.2456
101	Ni	3.4238	1.6933	5.0919	0.2463	0.1218	0.2320
102	Ni	5.1822	13.8638	5.1908	0.3728	0.9973	0.2365
103	Ni	3.4716	5.2194	5.2528	0.2497	0.3754	0.2393
104	Ni	5.2120	3.4339	5.2214	0.3749	0.2470	0.2379
105	Ni	13.8969	8.6905	5.2274	0.9996	0.6251	0.2381
106	Ni	1.7309	6.9813	5.1973	0.1245	0.5022	0.2368
107	Ni	13.8886	12.1512	5.2262	0.9990	0.8741	0.2381
108	Ni	1.7355	10.4225	5.2278	0.1248	0.7497	0.2382
109	Ni	3.4698	8.6861	5.2250	0.2496	0.6248	0.2380
110	Ni	5.2132	6.9955	5.2790	0.3750	0.5032	0.2405
111	Ni	3.4851	12.1289	5.2213	0.2507	0.8725	0.2379
112	Ni	5.2294	10.4062	5.2302	0.3762	0.7485	0.2383
113	Ni	6.9683	1.7088	5.3965	0.5012	0.1229	0.2458
114	Ni	8.6974	13.8955	5.2362	0.6256	0.9995	0.2385
115	Ni	7.0004	5.2390	5.2473	0.5036	0.3769	0.2390
116	Ni	8.6992	3.4858	5.2064	0.6258	0.2507	0.2372
117	Ni	10.4222	1.7437	5.2328	0.7497	0.1254	0.2384
118	Ni	12.1663	0.0014	5.2284	0.8752	0.0001	0.2382
119	Ni	10.4176	5.2102	5.2286	0.7494	0.3748	0.2382
120	Ni	12.1671	3.4714	5.2308	0.8752	0.2497	0.2383
121	Ni	6.9874	8.7187	5.1466	0.5026	0.6272	0.2345
122	Ni	8.7178	6.9859	5.1751	0.6271	0.5025	0.2358
123	Ni	6.9518	12.1625	5.2289	0.5001	0.8749	0.2382
124	Ni	8.6909	10.4318	5.2352	0.6252	0.7504	0.2385
125	Ni	10.4204	8.7021	5.2329	0.7496	0.6260	0.2384
126	Ni	12.1454	6.9630	5.2285	0.8736	0.5009	0.2382
127	Ni	10.4320	12.1672	5.2295	0.7504	0.8752	0.2382
128	Ni	12.1611	10.4267	5.2297	0.8748	0.7500	0.2382
129	O	1.8260	1.6160	6.3131	0.1313	0.1162	0.2876
130	O	5.8381	2.4555	6.7895	0.4199	0.1766	0.3093
131	O	2.7123	4.4782	6.8573	0.1951	0.3221	0.3124
132	O	4.8525	4.2745	8.8394	0.3491	0.3075	0.4027
133	O	6.0569	6.0471	6.7934	0.4357	0.4350	0.3095
134	O	8.0248	6.0002	8.8283	0.5772	0.4316	0.4022
135	O	5.2941	7.8464	8.8330	0.3808	0.5644	0.4024
136	Ce	3.9923	6.3156	8.0676	0.2872	0.4543	0.3675
137	Ce	3.7125	2.6314	7.7235	0.2670	0.1893	0.3519
138	Ce	7.0776	4.2233	8.0489	0.5091	0.3038	0.3667

139 Sm	7.2071	7.7275	7.9120	0.5184	0.5559	0.3604

/db/jmorales/CoNi-alloy/metal-SDC-interface/Ni-Ce_3SmO_7/Ni-Ce_3SmO_7-conf2b

a =				13.9019837372			
b =				13.9019837372			
c =				21.9509918687			
alpha =				90.0			
beta =				90.0			
gamma =				90.0			
	Atom	X	Y	Z	X	Y	Z
1	Ni	0.0000	0.0000	0.0000	0.0000	0.0000	0.0000
2	Ni	1.7377	1.7377	0.0000	0.1250	0.1250	0.0000
3	Ni	0.0000	3.4755	0.0000	0.0000	0.2500	0.0000
4	Ni	1.7377	5.2132	0.0000	0.1250	0.3750	0.0000
5	Ni	3.4755	0.0000	0.0000	0.2500	0.0000	0.0000
6	Ni	5.2132	1.7377	0.0000	0.3750	0.1250	0.0000
7	Ni	3.4755	3.4755	0.0000	0.2500	0.2500	0.0000
8	Ni	5.2132	5.2132	0.0000	0.3750	0.3750	0.0000
9	Ni	0.0000	6.9510	0.0000	0.0000	0.5000	0.0000
10	Ni	1.7377	8.6887	0.0000	0.1250	0.6250	0.0000
11	Ni	0.0000	10.4265	0.0000	0.0000	0.7500	0.0000
12	Ni	1.7377	12.1642	0.0000	0.1250	0.8750	0.0000
13	Ni	3.4755	6.9510	0.0000	0.2500	0.5000	0.0000
14	Ni	5.2132	8.6887	0.0000	0.3750	0.6250	0.0000
15	Ni	3.4755	10.4265	0.0000	0.2500	0.7500	0.0000
16	Ni	5.2132	12.1642	0.0000	0.3750	0.8750	0.0000
17	Ni	6.9510	0.0000	0.0000	0.5000	0.0000	0.0000
18	Ni	8.6887	1.7377	0.0000	0.6250	0.1250	0.0000
19	Ni	6.9510	3.4755	0.0000	0.5000	0.2500	0.0000
20	Ni	8.6887	5.2132	0.0000	0.6250	0.3750	0.0000
21	Ni	10.4265	0.0000	0.0000	0.7500	0.0000	0.0000
22	Ni	12.1642	1.7377	0.0000	0.8750	0.1250	0.0000
23	Ni	10.4265	3.4755	0.0000	0.7500	0.2500	0.0000
24	Ni	12.1642	5.2132	0.0000	0.8750	0.3750	0.0000
25	Ni	6.9510	6.9510	0.0000	0.5000	0.5000	0.0000
26	Ni	8.6887	8.6887	0.0000	0.6250	0.6250	0.0000
27	Ni	6.9510	10.4265	0.0000	0.5000	0.7500	0.0000
28	Ni	8.6887	12.1642	0.0000	0.6250	0.8750	0.0000
29	Ni	10.4265	6.9510	0.0000	0.7500	0.5000	0.0000
30	Ni	12.1642	8.6887	0.0000	0.8750	0.6250	0.0000
31	Ni	10.4265	10.4265	0.0000	0.7500	0.7500	0.0000
32	Ni	12.1642	12.1642	0.0000	0.8750	0.8750	0.0000
33	Ni	0.0000	1.7377	1.7377	0.0000	0.1250	0.0792
34	Ni	1.7377	0.0000	1.7377	0.1250	0.0000	0.0792
35	Ni	0.0000	5.2132	1.7377	0.0000	0.3750	0.0792
36	Ni	1.7377	3.4755	1.7377	0.1250	0.2500	0.0792
37	Ni	3.4755	1.7377	1.7377	0.2500	0.1250	0.0792
38	Ni	5.2132	0.0000	1.7377	0.3750	0.0000	0.0792
39	Ni	3.4755	5.2132	1.7377	0.2500	0.3750	0.0792
40	Ni	5.2132	3.4755	1.7377	0.3750	0.2500	0.0792

41	Ni	0.0000	8.6887	1.7377	0.0000	0.6250	0.0792
42	Ni	1.7377	6.9510	1.7377	0.1250	0.5000	0.0792
43	Ni	0.0000	12.1642	1.7377	0.0000	0.8750	0.0792
44	Ni	1.7377	10.4265	1.7377	0.1250	0.7500	0.0792
45	Ni	3.4755	8.6887	1.7377	0.2500	0.6250	0.0792
46	Ni	5.2132	6.9510	1.7377	0.3750	0.5000	0.0792
47	Ni	3.4755	12.1642	1.7377	0.2500	0.8750	0.0792
48	Ni	5.2132	10.4265	1.7377	0.3750	0.7500	0.0792
49	Ni	6.9510	1.7377	1.7377	0.5000	0.1250	0.0792
50	Ni	8.6887	0.0000	1.7377	0.6250	0.0000	0.0792
51	Ni	6.9510	5.2132	1.7377	0.5000	0.3750	0.0792
52	Ni	8.6887	3.4755	1.7377	0.6250	0.2500	0.0792
53	Ni	10.4265	1.7377	1.7377	0.7500	0.1250	0.0792
54	Ni	12.1642	0.0000	1.7377	0.8750	0.0000	0.0792
55	Ni	10.4265	5.2132	1.7377	0.7500	0.3750	0.0792
56	Ni	12.1642	3.4755	1.7377	0.8750	0.2500	0.0792
57	Ni	6.9510	8.6887	1.7377	0.5000	0.6250	0.0792
58	Ni	8.6887	6.9510	1.7377	0.6250	0.5000	0.0792
59	Ni	6.9510	12.1642	1.7377	0.5000	0.8750	0.0792
60	Ni	8.6887	10.4265	1.7377	0.6250	0.7500	0.0792
61	Ni	10.4265	8.6887	1.7377	0.7500	0.6250	0.0792
62	Ni	12.1642	6.9510	1.7377	0.8750	0.5000	0.0792
63	Ni	10.4265	12.1642	1.7377	0.7500	0.8750	0.0792
64	Ni	12.1642	10.4265	1.7377	0.8750	0.7500	0.0792
65	Ni	0.0019	0.0009	3.5081	0.0001	0.0001	0.1598
66	Ni	1.7362	1.7349	3.5033	0.1249	0.1248	0.1596
67	Ni	13.8964	3.4758	3.5035	0.9996	0.2500	0.1596
68	Ni	1.7226	5.2302	3.5011	0.1239	0.3762	0.1595
69	Ni	3.4721	13.9013	3.5097	0.2498	1.0000	0.1599
70	Ni	5.2020	1.7451	3.5164	0.3742	0.1255	0.1602
71	Ni	3.4814	3.4872	3.5104	0.2504	0.2508	0.1599
72	Ni	5.2129	5.2121	3.5026	0.3750	0.3749	0.1596
73	Ni	0.0146	6.9532	3.5437	0.0011	0.5002	0.1614
74	Ni	1.7206	8.6708	3.5085	0.1238	0.6237	0.1598
75	Ni	0.0006	10.4217	3.5054	0.0000	0.7497	0.1597
76	Ni	1.7325	12.1698	3.5087	0.1246	0.8754	0.1598
77	Ni	3.4885	6.9682	3.4921	0.2509	0.5012	0.1591
78	Ni	5.2305	8.7038	3.4778	0.3762	0.6261	0.1584
79	Ni	3.4686	10.4548	3.4845	0.2495	0.7520	0.1587
80	Ni	5.2118	12.1754	3.5037	0.3749	0.8758	0.1596
81	Ni	6.9490	0.0018	3.5030	0.4999	0.0001	0.1596
82	Ni	8.6983	1.7298	3.5016	0.6257	0.1244	0.1595
83	Ni	6.9476	3.4518	3.4933	0.4998	0.2483	0.1591
84	Ni	8.6777	5.1873	3.5075	0.6242	0.3731	0.1598
85	Ni	10.4328	0.0001	3.5025	0.7505	0.0000	0.1596
86	Ni	12.1714	1.7342	3.5080	0.8755	0.1247	0.1598
87	Ni	10.4418	3.4697	3.5016	0.7511	0.2496	0.1595
88	Ni	12.1636	5.2219	3.5101	0.8750	0.3756	0.1599
89	Ni	6.9542	6.9186	3.4916	0.5002	0.4977	0.1591

90	Ni	8.6868	8.6891	3.5071	0.6249	0.6250	0.1598
91	Ni	6.9627	10.4291	3.5125	0.5008	0.7502	0.1600
92	Ni	8.6902	12.1614	3.5089	0.6251	0.8748	0.1599
93	Ni	10.4285	6.9690	3.5234	0.7501	0.5013	0.1605
94	Ni	12.1426	8.6872	3.5370	0.8734	0.6249	0.1611
95	Ni	10.4270	10.4233	3.5101	0.7500	0.7498	0.1599
96	Ni	12.1716	12.1649	3.5039	0.8755	0.8750	0.1596
97	Ni	0.0066	1.7438	5.2288	0.0005	0.1254	0.2382
98	Ni	1.7305	13.9004	5.2296	0.1245	0.9999	0.2382
99	Ni	13.8874	5.1979	5.2217	0.9990	0.3739	0.2379
100	Ni	1.7371	3.4652	5.2006	0.1250	0.2493	0.2369
101	Ni	3.4469	1.7349	5.2308	0.2479	0.1248	0.2383
102	Ni	5.2004	0.0115	5.2290	0.3741	0.0008	0.2382
103	Ni	3.4518	5.2475	5.1908	0.2483	0.3775	0.2365
104	Ni	5.1751	3.4141	5.2969	0.3723	0.2456	0.2413
105	Ni	-0.0027	8.6936	5.2333	-0.0002	0.6253	0.2384
106	Ni	1.7367	7.0012	5.4074	0.1249	0.5036	0.2463
107	Ni	0.0034	12.1610	5.2250	0.0002	0.8748	0.2380
108	Ni	1.7409	10.4200	5.2210	0.1252	0.7495	0.2378
109	Ni	3.4772	8.7395	5.1208	0.2501	0.6287	0.2333
110	Ni	5.2439	6.9795	5.2342	0.3772	0.5021	0.2385
111	Ni	3.4654	12.1702	5.2304	0.2493	0.8754	0.2383
112	Ni	5.1985	10.4546	5.2052	0.3739	0.7520	0.2371
113	Ni	6.9685	1.7116	5.2054	0.5013	0.1231	0.2371
114	Ni	8.6921	13.8958	5.2211	0.6252	0.9996	0.2379
115	Ni	6.9672	5.1188	5.2825	0.5012	0.3682	0.2406
116	Ni	8.7333	3.4455	5.2050	0.6282	0.2478	0.2371
117	Ni	10.4397	1.7220	5.2249	0.7509	0.1239	0.2380
118	Ni	12.1826	0.0035	5.2265	0.8763	0.0003	0.2381
119	Ni	10.4304	5.2069	5.2230	0.7503	0.3745	0.2379
120	Ni	12.1652	3.4711	5.2252	0.8751	0.2497	0.2380
121	Ni	6.9602	8.7057	5.2289	0.5007	0.6262	0.2382
122	Ni	8.6843	6.8803	5.1776	0.6247	0.4949	0.2359
123	Ni	6.9336	12.1851	5.2184	0.4987	0.8765	0.2377
124	Ni	8.6558	10.5033	5.2994	0.6226	0.7555	0.2414
125	Ni	10.3974	8.6833	5.4014	0.7479	0.6246	0.2461
126	Ni	12.1578	6.9564	5.2376	0.8745	0.5004	0.2386
127	Ni	10.4510	12.1991	5.2079	0.7518	0.8775	0.2373
128	Ni	12.1865	10.4387	5.2150	0.8766	0.7509	0.2376
129	O	5.9585	4.2979	6.8093	0.4286	0.3092	0.3102
130	O	2.8012	6.1810	6.8076	0.2015	0.4446	0.3101
131	O	4.9734	6.1390	8.8437	0.3577	0.4416	0.4029
132	O	6.2843	7.8023	6.8252	0.4520	0.5612	0.3109
133	O	9.4440	9.6051	6.6682	0.6793	0.6909	0.3038
134	O	8.0897	7.7207	9.0792	0.5819	0.5554	0.4136
135	O	5.4418	9.7509	8.7345	0.3914	0.7014	0.3979
136	Ce	4.1896	8.2386	7.9210	0.3014	0.5926	0.3608
137	Ce	3.8928	4.4896	7.8250	0.2800	0.3229	0.3565
138	Ce	7.2118	6.0194	8.1191	0.5188	0.4330	0.3699

139 Sm	7.5064	9.4883	8.0852	0.5400	0.6825	0.3683

/db/jmorales/CoNi-alloy/metal-SDC-interface/CoNi-Ce_3SmO_7/CoNi-Ce_3SmO_7-conf1a

a =			13.9033384264				
b =			13.9033384264				
c =			21.9516692133				
alpha =			90.0				
beta =			90.0				
gamma =			90.0				
	Atom	X	Y	Z	X	Y	Z
1	Ni	0.0000	0.0000	0.0000	0.0000	0.0000	0.0000
2	Ni	0.0000	3.4758	0.0000	0.0000	0.2500	0.0000
3	Ni	3.4758	0.0000	0.0000	0.2500	0.0000	0.0000
4	Ni	3.4758	3.4758	0.0000	0.2500	0.2500	0.0000
5	Ni	0.0000	6.9517	0.0000	0.0000	0.5000	0.0000
6	Ni	0.0000	10.4275	0.0000	0.0000	0.7500	0.0000
7	Ni	3.4758	6.9517	0.0000	0.2500	0.5000	0.0000
8	Ni	3.4758	10.4275	0.0000	0.2500	0.7500	0.0000
9	Ni	6.9517	0.0000	0.0000	0.5000	0.0000	0.0000
10	Ni	6.9517	3.4758	0.0000	0.5000	0.2500	0.0000
11	Ni	10.4275	0.0000	0.0000	0.7500	0.0000	0.0000
12	Ni	10.4275	3.4758	0.0000	0.7500	0.2500	0.0000
13	Ni	6.9517	6.9517	0.0000	0.5000	0.5000	0.0000
14	Ni	6.9517	10.4275	0.0000	0.5000	0.7500	0.0000
15	Ni	10.4275	6.9517	0.0000	0.7500	0.5000	0.0000
16	Ni	10.4275	10.4275	0.0000	0.7500	0.7500	0.0000
17	Ni	0.0000	1.7379	1.7379	0.0000	0.1250	0.0792
18	Ni	0.0000	5.2138	1.7379	0.0000	0.3750	0.0792
19	Ni	3.4758	1.7379	1.7379	0.2500	0.1250	0.0792
20	Ni	3.4758	5.2138	1.7379	0.2500	0.3750	0.0792
21	Ni	0.0000	8.6896	1.7379	0.0000	0.6250	0.0792
22	Ni	0.0000	12.1654	1.7379	0.0000	0.8750	0.0792
23	Ni	3.4758	8.6896	1.7379	0.2500	0.6250	0.0792
24	Ni	3.4758	12.1654	1.7379	0.2500	0.8750	0.0792
25	Ni	6.9517	1.7379	1.7379	0.5000	0.1250	0.0792
26	Ni	6.9517	5.2138	1.7379	0.5000	0.3750	0.0792
27	Ni	10.4275	1.7379	1.7379	0.7500	0.1250	0.0792
28	Ni	10.4275	5.2138	1.7379	0.7500	0.3750	0.0792
29	Ni	6.9517	8.6896	1.7379	0.5000	0.6250	0.0792
30	Ni	6.9517	12.1654	1.7379	0.5000	0.8750	0.0792
31	Ni	10.4275	8.6896	1.7379	0.7500	0.6250	0.0792
32	Ni	10.4275	12.1654	1.7379	0.7500	0.8750	0.0792
33	Ni	13.8992	-0.0013	3.5067	0.9997	-0.0001	0.1597
34	Ni	0.0050	3.4662	3.4947	0.0004	0.2493	0.1592
35	Ni	3.4827	0.0158	3.5308	0.2505	0.0011	0.1608
36	Ni	3.4868	3.4712	3.4427	0.2508	0.2497	0.1568
37	Ni	0.0115	6.9503	3.5248	0.0008	0.4999	0.1606
38	Ni	13.8998	10.4316	3.4998	0.9997	0.7503	0.1594
39	Ni	3.4564	6.9343	3.5435	0.2486	0.4987	0.1614

40	Ni	3.4806	10.4460	3.4909	0.2503	0.7513	0.1590
41	Ni	6.9517	13.8779	3.5313	0.5000	0.9982	0.1609
42	Ni	6.9403	3.4651	3.4960	0.4992	0.2492	0.1593
43	Ni	10.4250	0.0034	3.5059	0.7498	0.0002	0.1597
44	Ni	10.4247	3.4710	3.5048	0.7498	0.2497	0.1597
45	Ni	6.9480	6.9651	3.5088	0.4997	0.5010	0.1598
46	Ni	6.9553	10.4374	3.4445	0.5003	0.7507	0.1569
47	Ni	10.4315	6.9505	3.5006	0.7503	0.4999	0.1595
48	Ni	10.4217	10.4297	3.5057	0.7496	0.7502	0.1597
49	Ni	0.0087	1.7355	5.2079	0.0006	0.1248	0.2372
50	Ni	13.8847	5.1782	5.1885	0.9987	0.3724	0.2364
51	Ni	3.4743	1.7287	5.2546	0.2499	0.1243	0.2394
52	Ni	3.4859	5.1913	5.1924	0.2507	0.3734	0.2365
53	Ni	13.8769	8.7267	5.2105	0.9981	0.6277	0.2374
54	Ni	13.9015	12.1597	5.2086	0.9999	0.8746	0.2373
55	Ni	3.4532	8.7340	5.1374	0.2484	0.6282	0.2340
56	Ni	3.4764	12.1640	5.2150	0.2500	0.8749	0.2376
57	Ni	6.9487	1.7363	5.2157	0.4998	0.1249	0.2376
58	Ni	6.9595	5.2022	5.1609	0.5006	0.3742	0.2351
59	Ni	10.4227	1.7434	5.2111	0.7497	0.1254	0.2374
60	Ni	10.4297	5.2030	5.2082	0.7502	0.3742	0.2373
61	Ni	7.0091	8.6894	5.1655	0.5041	0.6250	0.2353
62	Ni	6.9604	12.1691	5.2680	0.5006	0.8753	0.2400
63	Ni	10.4185	8.6805	5.2088	0.7494	0.6243	0.2373
64	Ni	10.4245	12.1735	5.2086	0.7498	0.8756	0.2373
65	Co	1.7379	1.7379	0.0000	0.1250	0.1250	0.0000
66	Co	1.7379	5.2138	0.0000	0.1250	0.3750	0.0000
67	Co	5.2138	1.7379	0.0000	0.3750	0.1250	0.0000
68	Co	5.2138	5.2138	0.0000	0.3750	0.3750	0.0000
69	Co	1.7379	8.6896	0.0000	0.1250	0.6250	0.0000
70	Co	1.7379	12.1654	0.0000	0.1250	0.8750	0.0000
71	Co	5.2138	8.6896	0.0000	0.3750	0.6250	0.0000
72	Co	5.2138	12.1654	0.0000	0.3750	0.8750	0.0000
73	Co	8.6896	1.7379	0.0000	0.6250	0.1250	0.0000
74	Co	8.6896	5.2138	0.0000	0.6250	0.3750	0.0000
75	Co	12.1654	1.7379	0.0000	0.8750	0.1250	0.0000
76	Co	12.1654	5.2138	0.0000	0.8750	0.3750	0.0000
77	Co	8.6896	8.6896	0.0000	0.6250	0.6250	0.0000
78	Co	8.6896	12.1654	0.0000	0.6250	0.8750	0.0000
79	Co	12.1654	8.6896	0.0000	0.8750	0.6250	0.0000
80	Co	12.1654	12.1654	0.0000	0.8750	0.8750	0.0000
81	Co	1.7379	0.0000	1.7379	0.1250	0.0000	0.0792
82	Co	1.7379	3.4758	1.7379	0.1250	0.2500	0.0792
83	Co	5.2138	0.0000	1.7379	0.3750	0.0000	0.0792
84	Co	5.2138	3.4758	1.7379	0.3750	0.2500	0.0792
85	Co	1.7379	6.9517	1.7379	0.1250	0.5000	0.0792
86	Co	1.7379	10.4275	1.7379	0.1250	0.7500	0.0792
87	Co	5.2138	6.9517	1.7379	0.3750	0.5000	0.0792
88	Co	5.2138	10.4275	1.7379	0.3750	0.7500	0.0792

89	Co	8.6896	0.0000	1.7379	0.6250	0.0000	0.0792
90	Co	8.6896	3.4758	1.7379	0.6250	0.2500	0.0792
91	Co	12.1654	0.0000	1.7379	0.8750	0.0000	0.0792
92	Co	12.1654	3.4758	1.7379	0.8750	0.2500	0.0792
93	Co	8.6896	6.9517	1.7379	0.6250	0.5000	0.0792
94	Co	8.6896	10.4275	1.7379	0.6250	0.7500	0.0792
95	Co	12.1654	6.9517	1.7379	0.8750	0.5000	0.0792
96	Co	12.1654	10.4275	1.7379	0.8750	0.7500	0.0792
97	Co	1.7447	1.7179	3.5245	0.1255	0.1236	0.1606
98	Co	1.7327	5.2297	3.5446	0.1246	0.3761	0.1615
99	Co	5.2139	1.7383	3.5558	0.3750	0.1250	0.1620
100	Co	5.2148	5.2239	3.5377	0.3751	0.3757	0.1612
101	Co	1.7170	8.6743	3.5490	0.1235	0.6239	0.1617
102	Co	1.7391	12.1714	3.5525	0.1251	0.8754	0.1618
103	Co	5.2144	8.7044	3.5284	0.3750	0.6261	0.1607
104	Co	5.2181	12.1598	3.5638	0.3753	0.8746	0.1623
105	Co	8.6846	1.7389	3.5555	0.6246	0.1251	0.1620
106	Co	8.6991	5.2087	3.5320	0.6257	0.3746	0.1609
107	Co	12.1648	1.7354	3.5522	0.8750	0.1248	0.1618
108	Co	12.1610	5.2072	3.5432	0.8747	0.3745	0.1614
109	Co	8.7061	8.6932	3.5245	0.6262	0.6253	0.1606
110	Co	8.6887	12.1595	3.5563	0.6249	0.8746	0.1620
111	Co	12.1625	8.6889	3.5554	0.8748	0.6249	0.1620
112	Co	12.1610	12.1698	3.5533	0.8747	0.8753	0.1619
113	Co	1.7272	13.8947	5.2252	0.1242	0.9994	0.2380
114	Co	1.7419	3.4587	5.1816	0.1253	0.2488	0.2360
115	Co	5.2174	13.9011	5.2295	0.3753	0.9998	0.2382
116	Co	5.2206	3.4791	5.2581	0.3755	0.2502	0.2395
117	Co	1.6961	6.9548	5.3773	0.1220	0.5002	0.2450
118	Co	1.7468	10.4326	5.2091	0.1256	0.7504	0.2373
119	Co	5.2135	7.0004	5.2372	0.3750	0.5035	0.2386
120	Co	5.2288	10.4255	5.2609	0.3761	0.7499	0.2397
121	Co	8.6972	0.0112	5.2273	0.6256	0.0008	0.2381
122	Co	8.6650	3.4854	5.2220	0.6232	0.2507	0.2379
123	Co	12.1636	0.0046	5.2268	0.8749	0.0003	0.2381
124	Co	12.1738	3.4673	5.2207	0.8756	0.2494	0.2378
125	Co	8.7047	6.9198	5.2119	0.6261	0.4977	0.2374
126	Co	8.6936	10.4299	5.2747	0.6253	0.7502	0.2403
127	Co	12.1581	6.9419	5.2153	0.8745	0.4993	0.2376
128	Co	12.1477	10.4345	5.2179	0.8737	0.7505	0.2377
129	O	3.4629	3.5444	6.3823	0.2491	0.2549	0.2907
130	O	1.5244	6.8041	7.2659	0.1096	0.4894	0.3310
131	O	3.8270	5.7913	8.7133	0.2753	0.4165	0.3969
132	O	5.4586	7.4867	7.0637	0.3926	0.5385	0.3218
133	O	7.3289	6.8770	8.9923	0.5271	0.4946	0.4096
134	O	4.9254	9.2940	9.1319	0.3543	0.6685	0.4160
135	O	6.9765	10.3354	6.3503	0.5018	0.7434	0.2893
136	Ce	3.4332	8.1020	8.0442	0.2469	0.5827	0.3665
137	Ce	2.0608	4.7625	7.8450	0.1482	0.3425	0.3574

138 Ce	6.0755	5.4725	8.0360	0.4370	0.3936	0.3661
139 Sm	6.8031	8.8813	8.2867	0.4893	0.6388	0.3775

/db/jmorales/CoNi-alloy/metal-SDC-interface/Ni-Ce_3SmO_7/Ni-Ce_3SmO_7-conf2a

a =			13.9019837372				
b =			13.9019837372				
c =			21.9509918687				
alpha =			90.0				
beta =			90.0				
gamma =			90.0				
	Atom	X	Y	Z	X	Y	Z
1	Ni	0.0000	0.0000	0.0000	0.0000	0.0000	0.0000
2	Ni	1.7377	1.7377	0.0000	0.1250	0.1250	0.0000
3	Ni	0.0000	3.4755	0.0000	0.0000	0.2500	0.0000
4	Ni	1.7377	5.2132	0.0000	0.1250	0.3750	0.0000
5	Ni	3.4755	0.0000	0.0000	0.2500	0.0000	0.0000
6	Ni	5.2132	1.7377	0.0000	0.3750	0.1250	0.0000
7	Ni	3.4755	3.4755	0.0000	0.2500	0.2500	0.0000
8	Ni	5.2132	5.2132	0.0000	0.3750	0.3750	0.0000
9	Ni	0.0000	6.9510	0.0000	0.0000	0.5000	0.0000
10	Ni	1.7377	8.6887	0.0000	0.1250	0.6250	0.0000
11	Ni	0.0000	10.4265	0.0000	0.0000	0.7500	0.0000
12	Ni	1.7377	12.1642	0.0000	0.1250	0.8750	0.0000
13	Ni	3.4755	6.9510	0.0000	0.2500	0.5000	0.0000
14	Ni	5.2132	8.6887	0.0000	0.3750	0.6250	0.0000
15	Ni	3.4755	10.4265	0.0000	0.2500	0.7500	0.0000
16	Ni	5.2132	12.1642	0.0000	0.3750	0.8750	0.0000
17	Ni	6.9510	0.0000	0.0000	0.5000	0.0000	0.0000
18	Ni	8.6887	1.7377	0.0000	0.6250	0.1250	0.0000
19	Ni	6.9510	3.4755	0.0000	0.5000	0.2500	0.0000
20	Ni	8.6887	5.2132	0.0000	0.6250	0.3750	0.0000
21	Ni	10.4265	0.0000	0.0000	0.7500	0.0000	0.0000
22	Ni	12.1642	1.7377	0.0000	0.8750	0.1250	0.0000
23	Ni	10.4265	3.4755	0.0000	0.7500	0.2500	0.0000
24	Ni	12.1642	5.2132	0.0000	0.8750	0.3750	0.0000
25	Ni	6.9510	6.9510	0.0000	0.5000	0.5000	0.0000
26	Ni	8.6887	8.6887	0.0000	0.6250	0.6250	0.0000
27	Ni	6.9510	10.4265	0.0000	0.5000	0.7500	0.0000
28	Ni	8.6887	12.1642	0.0000	0.6250	0.8750	0.0000
29	Ni	10.4265	6.9510	0.0000	0.7500	0.5000	0.0000
30	Ni	12.1642	8.6887	0.0000	0.8750	0.6250	0.0000
31	Ni	10.4265	10.4265	0.0000	0.7500	0.7500	0.0000
32	Ni	12.1642	12.1642	0.0000	0.8750	0.8750	0.0000
33	Ni	0.0000	1.7377	1.7377	0.0000	0.1250	0.0792
34	Ni	1.7377	0.0000	1.7377	0.1250	0.0000	0.0792
35	Ni	0.0000	5.2132	1.7377	0.0000	0.3750	0.0792
36	Ni	1.7377	3.4755	1.7377	0.1250	0.2500	0.0792
37	Ni	3.4755	1.7377	1.7377	0.2500	0.1250	0.0792
38	Ni	5.2132	0.0000	1.7377	0.3750	0.0000	0.0792
39	Ni	3.4755	5.2132	1.7377	0.2500	0.3750	0.0792
40	Ni	5.2132	3.4755	1.7377	0.3750	0.2500	0.0792

41	Ni	0.0000	8.6887	1.7377	0.0000	0.6250	0.0792
42	Ni	1.7377	6.9510	1.7377	0.1250	0.5000	0.0792
43	Ni	0.0000	12.1642	1.7377	0.0000	0.8750	0.0792
44	Ni	1.7377	10.4265	1.7377	0.1250	0.7500	0.0792
45	Ni	3.4755	8.6887	1.7377	0.2500	0.6250	0.0792
46	Ni	5.2132	6.9510	1.7377	0.3750	0.5000	0.0792
47	Ni	3.4755	12.1642	1.7377	0.2500	0.8750	0.0792
48	Ni	5.2132	10.4265	1.7377	0.3750	0.7500	0.0792
49	Ni	6.9510	1.7377	1.7377	0.5000	0.1250	0.0792
50	Ni	8.6887	0.0000	1.7377	0.6250	0.0000	0.0792
51	Ni	6.9510	5.2132	1.7377	0.5000	0.3750	0.0792
52	Ni	8.6887	3.4755	1.7377	0.6250	0.2500	0.0792
53	Ni	10.4265	1.7377	1.7377	0.7500	0.1250	0.0792
54	Ni	12.1642	0.0000	1.7377	0.8750	0.0000	0.0792
55	Ni	10.4265	5.2132	1.7377	0.7500	0.3750	0.0792
56	Ni	12.1642	3.4755	1.7377	0.8750	0.2500	0.0792
57	Ni	6.9510	8.6887	1.7377	0.5000	0.6250	0.0792
58	Ni	8.6887	6.9510	1.7377	0.6250	0.5000	0.0792
59	Ni	6.9510	12.1642	1.7377	0.5000	0.8750	0.0792
60	Ni	8.6887	10.4265	1.7377	0.6250	0.7500	0.0792
61	Ni	10.4265	8.6887	1.7377	0.7500	0.6250	0.0792
62	Ni	12.1642	6.9510	1.7377	0.8750	0.5000	0.0792
63	Ni	10.4265	12.1642	1.7377	0.7500	0.8750	0.0792
64	Ni	12.1642	10.4265	1.7377	0.8750	0.7500	0.0792
65	Ni	0.0014	0.0014	3.5086	0.0001	0.0001	0.1598
66	Ni	1.7368	1.7532	3.5177	0.1249	0.1261	0.1603
67	Ni	13.8904	3.4725	3.5000	0.9992	0.2498	0.1594
68	Ni	1.7261	5.2158	3.4370	0.1242	0.3752	0.1566
69	Ni	3.4745	0.0008	3.5076	0.2499	0.0001	0.1598
70	Ni	5.2143	1.7489	3.5354	0.3751	0.1258	0.1611
71	Ni	3.5000	3.4603	3.5073	0.2518	0.2489	0.1598
72	Ni	5.1936	5.1903	3.5181	0.3736	0.3734	0.1603
73	Ni	13.8972	6.9536	3.5052	0.9997	0.5002	0.1597
74	Ni	1.7183	8.6722	3.5073	0.1236	0.6238	0.1598
75	Ni	0.0026	10.4277	3.5085	0.0002	0.7501	0.1598
76	Ni	1.7334	12.1696	3.5092	0.1247	0.8754	0.1599
77	Ni	3.4912	6.9603	3.4838	0.2511	0.5007	0.1587
78	Ni	5.2278	8.6955	3.5034	0.3760	0.6255	0.1596
79	Ni	3.4656	10.4465	3.4904	0.2493	0.7514	0.1590
80	Ni	5.2151	12.1718	3.5043	0.3751	0.8755	0.1596
81	Ni	6.9533	13.8997	3.5103	0.5002	0.9998	0.1599
82	Ni	8.6919	1.7352	3.5073	0.6252	0.1248	0.1598
83	Ni	6.9311	3.4490	3.5087	0.4986	0.2481	0.1598
84	Ni	8.7201	5.2297	3.4951	0.6273	0.3762	0.1592
85	Ni	10.4235	13.8965	3.5084	0.7498	0.9996	0.1598
86	Ni	12.1620	1.7305	3.5082	0.8748	0.1245	0.1598
87	Ni	10.4333	3.4737	3.5056	0.7505	0.2499	0.1597
88	Ni	12.1868	5.2108	3.5272	0.8766	0.3748	0.1607
89	Ni	6.9536	6.9621	3.4825	0.5002	0.5008	0.1586

90 Ni	8.6812	8.6899	3.4585	0.6245	0.6251	0.1576
91 Ni	6.9462	10.4150	3.5195	0.4997	0.7492	0.1603
92 Ni	8.6853	12.1380	3.5275	0.6248	0.8731	0.1607
93 Ni	10.4420	6.9586	3.5036	0.7511	0.5005	0.1596
94 Ni	12.1540	8.6967	3.5214	0.8743	0.6256	0.1604
95 Ni	10.4295	10.4240	3.5068	0.7502	0.7498	0.1598
96 Ni	12.1668	12.1666	3.5074	0.8752	0.8752	0.1598
97 Ni	13.8970	1.7363	5.2232	0.9996	0.1249	0.2379
98 Ni	1.7338	0.0076	5.2290	0.1247	0.0005	0.2382
99 Ni	-0.0015	5.2147	5.2802	-0.0001	0.3751	0.2405
100 Ni	1.7328	3.4601	5.2333	0.1246	0.2489	0.2384
101 Ni	3.4632	1.7270	5.2198	0.2491	0.1242	0.2378
102 Ni	5.2134	13.8951	5.2230	0.3750	0.9995	0.2379
103 Ni	3.4553	5.2304	5.1716	0.2485	0.3762	0.2356
104 Ni	5.2278	3.4697	5.4534	0.3760	0.2496	0.2484
105 Ni	13.8939	8.6951	5.2278	0.9994	0.6255	0.2382
106 Ni	1.7430	6.9847	5.2794	0.1254	0.5024	0.2405
107 Ni	0.0061	12.1693	5.2247	0.0004	0.8754	0.2380
108 Ni	1.7428	10.4196	5.2288	0.1254	0.7495	0.2382
109 Ni	3.4620	8.7104	5.1410	0.2490	0.6266	0.2342
110 Ni	5.2002	6.9052	5.2508	0.3741	0.4967	0.2392
111 Ni	3.4644	12.1728	5.2293	0.2492	0.8756	0.2382
112 Ni	5.1755	10.4528	5.2064	0.3723	0.7519	0.2372
113 Ni	6.9739	1.7186	5.2263	0.5016	0.1236	0.2381
114 Ni	8.6948	13.8884	5.2250	0.6254	0.9990	0.2380
115 Ni	6.9956	5.2002	5.0905	0.5032	0.3741	0.2319
116 Ni	8.6929	3.4826	5.2097	0.6253	0.2505	0.2373
117 Ni	10.4245	1.7356	5.2232	0.7499	0.1248	0.2379
118 Ni	12.1629	13.8980	5.2244	0.8749	0.9997	0.2380
119 Ni	10.4339	5.2081	5.2229	0.7505	0.3746	0.2379
120 Ni	12.1621	3.4683	5.2216	0.8748	0.2495	0.2379
121 Ni	6.9021	8.7094	5.3042	0.4965	0.6265	0.2416
122 Ni	8.7139	6.9565	5.2558	0.6268	0.5004	0.2394
123 Ni	6.9477	12.1582	5.2305	0.4998	0.8746	0.2383
124 Ni	8.6839	10.4171	5.2874	0.6247	0.7493	0.2409
125 Ni	10.4715	8.6952	5.2948	0.7532	0.6255	0.2412
126 Ni	12.1713	6.9499	5.2192	0.8755	0.4999	0.2378
127 Ni	10.4286	12.1600	5.2204	0.7502	0.8747	0.2378
128 Ni	12.1790	10.4435	5.2232	0.8761	0.7512	0.2379
129 O	5.1611	3.4806	7.3687	0.3712	0.2504	0.3357
130 O	1.7483	5.2106	6.2796	0.1258	0.3748	0.2861
131 O	4.2315	5.9969	8.5178	0.3044	0.4314	0.3880
132 O	6.0794	7.6263	6.8506	0.4373	0.5486	0.3121
133 O	8.8154	8.7234	6.3033	0.6341	0.6275	0.2872
134 O	7.7260	6.8074	9.0727	0.5557	0.4897	0.4133
135 O	5.5410	9.3537	8.9891	0.3986	0.6728	0.4095
136 Ce	4.0651	8.2747	7.9257	0.2924	0.5952	0.3611
137 Ce	3.1657	4.1438	7.8719	0.2277	0.2981	0.3586
138 Ce	6.5076	5.4384	7.8609	0.4681	0.3912	0.3581

139 Sm	7.5147	8.7459	8.3211	0.5406	0.6291	0.3791

/db/jmorales/CoNi-alloy/metal-SDC-interface/Ni-Ce_3SmO_7/Ni-Ce_3SmO_7-conf5c

a =			13.9019837372				
b =			13.9019837372				
c =			21.9509918687				
alpha =			90.0				
beta =			90.0				
gamma =			90.0				
	Atom	X	Y	Z	X	Y	Z
1	Ni	0.0000	0.0000	0.0000	0.0000	0.0000	0.0000
2	Ni	1.7377	1.7377	0.0000	0.1250	0.1250	0.0000
3	Ni	0.0000	3.4755	0.0000	0.0000	0.2500	0.0000
4	Ni	1.7377	5.2132	0.0000	0.1250	0.3750	0.0000
5	Ni	3.4755	0.0000	0.0000	0.2500	0.0000	0.0000
6	Ni	5.2132	1.7377	0.0000	0.3750	0.1250	0.0000
7	Ni	3.4755	3.4755	0.0000	0.2500	0.2500	0.0000
8	Ni	5.2132	5.2132	0.0000	0.3750	0.3750	0.0000
9	Ni	0.0000	6.9510	0.0000	0.0000	0.5000	0.0000
10	Ni	1.7377	8.6887	0.0000	0.1250	0.6250	0.0000
11	Ni	0.0000	10.4265	0.0000	0.0000	0.7500	0.0000
12	Ni	1.7377	12.1642	0.0000	0.1250	0.8750	0.0000
13	Ni	3.4755	6.9510	0.0000	0.2500	0.5000	0.0000
14	Ni	5.2132	8.6887	0.0000	0.3750	0.6250	0.0000
15	Ni	3.4755	10.4265	0.0000	0.2500	0.7500	0.0000
16	Ni	5.2132	12.1642	0.0000	0.3750	0.8750	0.0000
17	Ni	6.9510	0.0000	0.0000	0.5000	0.0000	0.0000
18	Ni	8.6887	1.7377	0.0000	0.6250	0.1250	0.0000
19	Ni	6.9510	3.4755	0.0000	0.5000	0.2500	0.0000
20	Ni	8.6887	5.2132	0.0000	0.6250	0.3750	0.0000
21	Ni	10.4265	0.0000	0.0000	0.7500	0.0000	0.0000
22	Ni	12.1642	1.7377	0.0000	0.8750	0.1250	0.0000
23	Ni	10.4265	3.4755	0.0000	0.7500	0.2500	0.0000
24	Ni	12.1642	5.2132	0.0000	0.8750	0.3750	0.0000
25	Ni	6.9510	6.9510	0.0000	0.5000	0.5000	0.0000
26	Ni	8.6887	8.6887	0.0000	0.6250	0.6250	0.0000
27	Ni	6.9510	10.4265	0.0000	0.5000	0.7500	0.0000
28	Ni	8.6887	12.1642	0.0000	0.6250	0.8750	0.0000
29	Ni	10.4265	6.9510	0.0000	0.7500	0.5000	0.0000
30	Ni	12.1642	8.6887	0.0000	0.8750	0.6250	0.0000
31	Ni	10.4265	10.4265	0.0000	0.7500	0.7500	0.0000
32	Ni	12.1642	12.1642	0.0000	0.8750	0.8750	0.0000
33	Ni	0.0000	1.7377	1.7377	0.0000	0.1250	0.0792
34	Ni	1.7377	0.0000	1.7377	0.1250	0.0000	0.0792
35	Ni	0.0000	5.2132	1.7377	0.0000	0.3750	0.0792
36	Ni	1.7377	3.4755	1.7377	0.1250	0.2500	0.0792
37	Ni	3.4755	1.7377	1.7377	0.2500	0.1250	0.0792
38	Ni	5.2132	0.0000	1.7377	0.3750	0.0000	0.0792
39	Ni	3.4755	5.2132	1.7377	0.2500	0.3750	0.0792
40	Ni	5.2132	3.4755	1.7377	0.3750	0.2500	0.0792

41	Ni	0.0000	8.6887	1.7377	0.0000	0.6250	0.0792
42	Ni	1.7377	6.9510	1.7377	0.1250	0.5000	0.0792
43	Ni	0.0000	12.1642	1.7377	0.0000	0.8750	0.0792
44	Ni	1.7377	10.4265	1.7377	0.1250	0.7500	0.0792
45	Ni	3.4755	8.6887	1.7377	0.2500	0.6250	0.0792
46	Ni	5.2132	6.9510	1.7377	0.3750	0.5000	0.0792
47	Ni	3.4755	12.1642	1.7377	0.2500	0.8750	0.0792
48	Ni	5.2132	10.4265	1.7377	0.3750	0.7500	0.0792
49	Ni	6.9510	1.7377	1.7377	0.5000	0.1250	0.0792
50	Ni	8.6887	0.0000	1.7377	0.6250	0.0000	0.0792
51	Ni	6.9510	5.2132	1.7377	0.5000	0.3750	0.0792
52	Ni	8.6887	3.4755	1.7377	0.6250	0.2500	0.0792
53	Ni	10.4265	1.7377	1.7377	0.7500	0.1250	0.0792
54	Ni	12.1642	0.0000	1.7377	0.8750	0.0000	0.0792
55	Ni	10.4265	5.2132	1.7377	0.7500	0.3750	0.0792
56	Ni	12.1642	3.4755	1.7377	0.8750	0.2500	0.0792
57	Ni	6.9510	8.6887	1.7377	0.5000	0.6250	0.0792
58	Ni	8.6887	6.9510	1.7377	0.6250	0.5000	0.0792
59	Ni	6.9510	12.1642	1.7377	0.5000	0.8750	0.0792
60	Ni	8.6887	10.4265	1.7377	0.6250	0.7500	0.0792
61	Ni	10.4265	8.6887	1.7377	0.7500	0.6250	0.0792
62	Ni	12.1642	6.9510	1.7377	0.8750	0.5000	0.0792
63	Ni	10.4265	12.1642	1.7377	0.7500	0.8750	0.0792
64	Ni	12.1642	10.4265	1.7377	0.8750	0.7500	0.0792
65	Ni	-0.0000	0.0005	3.5106	-0.0000	0.0000	0.1599
66	Ni	1.7376	1.7409	3.5086	0.1250	0.1252	0.1598
67	Ni	0.0036	3.4815	3.5121	0.0003	0.2504	0.1600
68	Ni	1.7618	5.2131	3.5501	0.1267	0.3750	0.1617
69	Ni	3.4698	13.8936	3.5084	0.2496	0.9994	0.1598
70	Ni	5.2078	1.7148	3.4877	0.3746	0.1234	0.1589
71	Ni	3.4736	3.4969	3.5365	0.2499	0.2515	0.1611
72	Ni	5.1816	5.2024	3.4754	0.3727	0.3742	0.1583
73	Ni	0.0030	6.9459	3.5140	0.0002	0.4996	0.1601
74	Ni	1.7299	8.6936	3.5078	0.1244	0.6254	0.1598
75	Ni	13.8993	10.4292	3.5123	0.9998	0.7502	0.1600
76	Ni	1.7346	12.1634	3.5119	0.1248	0.8749	0.1600
77	Ni	3.4409	6.9392	3.4970	0.2475	0.4991	0.1593
78	Ni	5.2206	8.7049	3.4887	0.3755	0.6262	0.1589
79	Ni	3.4748	10.4341	3.5073	0.2499	0.7505	0.1598
80	Ni	5.2217	12.1647	3.5131	0.3756	0.8750	0.1600
81	Ni	6.9526	0.0133	3.5305	0.5001	0.0010	0.1608
82	Ni	8.6950	1.7303	3.5081	0.6254	0.1245	0.1598
83	Ni	6.9664	3.4611	3.4267	0.5011	0.2490	0.1561
84	Ni	8.6974	5.2158	3.4955	0.6256	0.3752	0.1592
85	Ni	10.4325	13.8984	3.5103	0.7504	0.9997	0.1599
86	Ni	12.1649	1.7427	3.5101	0.8750	0.1254	0.1599
87	Ni	10.4108	3.4710	3.5252	0.7489	0.2497	0.1606
88	Ni	12.1664	5.2143	3.5080	0.8752	0.3751	0.1598
89	Ni	6.9612	6.9323	3.4917	0.5007	0.4987	0.1591

90	Ni	8.6885	8.6961	3.4973	0.6250	0.6255	0.1593
91	Ni	6.9526	10.4162	3.5171	0.5001	0.7493	0.1602
92	Ni	8.6864	12.1767	3.5022	0.6248	0.8759	0.1595
93	Ni	10.4174	6.9583	3.5138	0.7493	0.5005	0.1601
94	Ni	12.1694	8.6857	3.5077	0.8754	0.6248	0.1598
95	Ni	10.4371	10.4336	3.5006	0.7508	0.7505	0.1595
96	Ni	12.1660	12.1665	3.5111	0.8751	0.8752	0.1600
97	Ni	13.9004	1.7413	5.2252	0.9999	0.1253	0.2380
98	Ni	1.7360	0.0030	5.2259	0.1249	0.0002	0.2381
99	Ni	13.8981	5.2131	5.2307	0.9997	0.3750	0.2383
100	Ni	1.7362	3.4673	5.2352	0.1249	0.2494	0.2385
101	Ni	3.4753	1.7333	5.2164	0.2500	0.1247	0.2376
102	Ni	5.2087	13.8983	5.2271	0.3747	0.9997	0.2381
103	Ni	3.4930	5.2389	5.5110	0.2513	0.3768	0.2511
104	Ni	5.2130	3.4419	5.1529	0.3750	0.2476	0.2347
105	Ni	13.8945	8.6909	5.2271	0.9995	0.6252	0.2381
106	Ni	1.7302	6.9746	5.2344	0.1245	0.5017	0.2385
107	Ni	-0.0001	12.1642	5.2286	-0.0000	0.8750	0.2382
108	Ni	1.7408	10.4205	5.2276	0.1252	0.7496	0.2382
109	Ni	3.4868	8.6931	5.2003	0.2508	0.6253	0.2369
110	Ni	5.1856	6.9535	5.0780	0.3730	0.5002	0.2313
111	Ni	3.4726	12.1657	5.2266	0.2498	0.8751	0.2381
112	Ni	5.2088	10.4267	5.2309	0.3747	0.7500	0.2383
113	Ni	6.9590	1.7297	5.2808	0.5006	0.1244	0.2406
114	Ni	8.6968	13.8944	5.2278	0.6256	0.9995	0.2382
115	Ni	6.9272	5.1716	5.1865	0.4983	0.3720	0.2363
116	Ni	8.7134	3.4829	5.2963	0.6268	0.2505	0.2413
117	Ni	10.4332	1.7322	5.2260	0.7505	0.1246	0.2381
118	Ni	12.1654	-0.0000	5.2255	0.8751	-0.0000	0.2381
119	Ni	10.4290	5.2152	5.2128	0.7502	0.3751	0.2375
120	Ni	12.1569	3.4822	5.2319	0.8745	0.2505	0.2383
121	Ni	6.9256	8.7064	5.2752	0.4982	0.6263	0.2403
122	Ni	8.7190	6.9397	5.2477	0.6272	0.4992	0.2391
123	Ni	6.9605	12.1553	5.2339	0.5007	0.8744	0.2384
124	Ni	8.7058	10.4453	5.1890	0.6262	0.7514	0.2364
125	Ni	10.4380	8.7144	5.2077	0.7508	0.6268	0.2372
126	Ni	12.1534	6.9482	5.2324	0.8742	0.4998	0.2384
127	Ni	10.4259	12.1601	5.2351	0.7500	0.8747	0.2385
128	Ni	12.1636	10.4294	5.2320	0.8750	0.7502	0.2383
129	O	6.9898	3.4524	6.2735	0.5028	0.2483	0.2858
130	O	4.9823	2.7740	9.5712	0.3584	0.1995	0.4360
131	O	3.9761	5.5291	7.2832	0.2860	0.3977	0.3318
132	O	6.6159	5.5699	8.5659	0.4759	0.4007	0.3902
133	O	7.6578	7.6228	6.7352	0.5508	0.5483	0.3068
134	O	9.4514	7.6514	8.8867	0.6799	0.5504	0.4048
135	O	6.5118	9.1408	8.8003	0.4684	0.6575	0.4009
136	Ce	5.4340	7.4682	7.8908	0.3909	0.5372	0.3595
137	Ce	5.2311	3.8026	8.0262	0.3763	0.2735	0.3656
138	Ce	8.7985	5.8424	8.0149	0.6329	0.4203	0.3651

139 Sm	8.4491	9.3424	8.0260	0.6078	0.6720	0.3656

/db/jmorales/CoNi-alloy/metal-SDC-interface/CoNi-Ce_3SmO_7/CoNi-Ce_3SmO_7-conf1c

a = 13.9033384264
b = 13.9033384264
c = 21.9516692133
alpha = 90.0
beta = 90.0
gamma = 90.0

	Atom	X	Y	Z	X	Y	Z
1	Ni	0.0000	0.0000	0.0000	0.0000	0.0000	0.0000
2	Ni	0.0000	3.4758	0.0000	0.0000	0.2500	0.0000
3	Ni	3.4758	0.0000	0.0000	0.2500	0.0000	0.0000
4	Ni	3.4758	3.4758	0.0000	0.2500	0.2500	0.0000
5	Ni	0.0000	6.9517	0.0000	0.0000	0.5000	0.0000
6	Ni	0.0000	10.4275	0.0000	0.0000	0.7500	0.0000
7	Ni	3.4758	6.9517	0.0000	0.2500	0.5000	0.0000
8	Ni	3.4758	10.4275	0.0000	0.2500	0.7500	0.0000
9	Ni	6.9517	0.0000	0.0000	0.5000	0.0000	0.0000
10	Ni	6.9517	3.4758	0.0000	0.5000	0.2500	0.0000
11	Ni	10.4275	0.0000	0.0000	0.7500	0.0000	0.0000
12	Ni	10.4275	3.4758	0.0000	0.7500	0.2500	0.0000
13	Ni	6.9517	6.9517	0.0000	0.5000	0.5000	0.0000
14	Ni	6.9517	10.4275	0.0000	0.5000	0.7500	0.0000
15	Ni	10.4275	6.9517	0.0000	0.7500	0.5000	0.0000
16	Ni	10.4275	10.4275	0.0000	0.7500	0.7500	0.0000
17	Ni	0.0000	1.7379	1.7379	0.0000	0.1250	0.0792
18	Ni	0.0000	5.2138	1.7379	0.0000	0.3750	0.0792
19	Ni	3.4758	1.7379	1.7379	0.2500	0.1250	0.0792
20	Ni	3.4758	5.2138	1.7379	0.2500	0.3750	0.0792
21	Ni	0.0000	8.6896	1.7379	0.0000	0.6250	0.0792
22	Ni	0.0000	12.1654	1.7379	0.0000	0.8750	0.0792
23	Ni	3.4758	8.6896	1.7379	0.2500	0.6250	0.0792
24	Ni	3.4758	12.1654	1.7379	0.2500	0.8750	0.0792
25	Ni	6.9517	1.7379	1.7379	0.5000	0.1250	0.0792
26	Ni	6.9517	5.2138	1.7379	0.5000	0.3750	0.0792
27	Ni	10.4275	1.7379	1.7379	0.7500	0.1250	0.0792
28	Ni	10.4275	5.2138	1.7379	0.7500	0.3750	0.0792
29	Ni	6.9517	8.6896	1.7379	0.5000	0.6250	0.0792
30	Ni	6.9517	12.1654	1.7379	0.5000	0.8750	0.0792
31	Ni	10.4275	8.6896	1.7379	0.7500	0.6250	0.0792
32	Ni	10.4275	12.1654	1.7379	0.7500	0.8750	0.0792
33	Ni	13.9008	0.0014	3.5012	0.9998	0.0001	0.1595
34	Ni	13.8926	3.4815	3.4909	0.9992	0.2504	0.1590
35	Ni	3.4788	0.0012	3.5030	0.2502	0.0001	0.1596
36	Ni	3.4984	3.4523	3.4940	0.2516	0.2483	0.1592
37	Ni	0.0229	6.9566	3.5364	0.0016	0.5004	0.1611
38	Ni	0.0002	10.4233	3.5030	0.0000	0.7497	0.1596
39	Ni	3.4656	6.9685	3.4904	0.2493	0.5012	0.1590

40	Ni	3.4705	10.4505	3.4736	0.2496	0.7517	0.1582
41	Ni	6.9504	0.0064	3.5050	0.4999	0.0005	0.1597
42	Ni	6.9294	3.4380	3.4934	0.4984	0.2473	0.1591
43	Ni	10.4284	0.0023	3.5011	0.7501	0.0002	0.1595
44	Ni	10.4453	3.4695	3.4972	0.7513	0.2495	0.1593
45	Ni	6.9580	6.9550	3.4639	0.5005	0.5002	0.1578
46	Ni	6.9632	10.4147	3.5187	0.5008	0.7491	0.1603
47	Ni	10.4354	6.9818	3.5129	0.7506	0.5022	0.1600
48	Ni	10.4164	10.4196	3.5168	0.7492	0.7494	0.1602
49	Ni	0.0129	1.7468	5.2073	0.0009	0.1256	0.2372
50	Ni	0.0014	5.2071	5.2169	0.0001	0.3745	0.2377
51	Ni	3.4346	1.7249	5.2168	0.2470	0.1241	0.2376
52	Ni	3.4695	5.2115	5.0748	0.2495	0.3748	0.2312
53	Ni	0.0029	8.6967	5.2196	0.0002	0.6255	0.2378
54	Ni	0.0049	12.1642	5.2000	0.0004	0.8749	0.2369
55	Ni	3.4888	8.7398	5.1200	0.2509	0.6286	0.2332
56	Ni	3.4664	12.1738	5.2034	0.2493	0.8756	0.2370
57	Ni	6.9773	1.7282	5.2168	0.5018	0.1243	0.2376
58	Ni	6.9949	5.1793	5.0798	0.5031	0.3725	0.2314
59	Ni	10.4312	1.7372	5.2088	0.7503	0.1249	0.2373
60	Ni	10.4353	5.2065	5.2039	0.7506	0.3745	0.2371
61	Ni	6.8966	8.6881	5.2259	0.4960	0.6249	0.2381
62	Ni	6.9608	12.1652	5.2020	0.5007	0.8750	0.2370
63	Ni	10.4076	8.6593	5.3874	0.7486	0.6228	0.2454
64	Ni	10.4408	12.1983	5.1907	0.7510	0.8774	0.2365
65	Co	1.7379	1.7379	0.0000	0.1250	0.1250	0.0000
66	Co	1.7379	5.2138	0.0000	0.1250	0.3750	0.0000
67	Co	5.2138	1.7379	0.0000	0.3750	0.1250	0.0000
68	Co	5.2138	5.2138	0.0000	0.3750	0.3750	0.0000
69	Co	1.7379	8.6896	0.0000	0.1250	0.6250	0.0000
70	Co	1.7379	12.1654	0.0000	0.1250	0.8750	0.0000
71	Co	5.2138	8.6896	0.0000	0.3750	0.6250	0.0000
72	Co	5.2138	12.1654	0.0000	0.3750	0.8750	0.0000
73	Co	8.6896	1.7379	0.0000	0.6250	0.1250	0.0000
74	Co	8.6896	5.2138	0.0000	0.6250	0.3750	0.0000
75	Co	12.1654	1.7379	0.0000	0.8750	0.1250	0.0000
76	Co	12.1654	5.2138	0.0000	0.8750	0.3750	0.0000
77	Co	8.6896	8.6896	0.0000	0.6250	0.6250	0.0000
78	Co	8.6896	12.1654	0.0000	0.6250	0.8750	0.0000
79	Co	12.1654	8.6896	0.0000	0.8750	0.6250	0.0000
80	Co	12.1654	12.1654	0.0000	0.8750	0.8750	0.0000
81	Co	1.7379	0.0000	1.7379	0.1250	0.0000	0.0792
82	Co	1.7379	3.4758	1.7379	0.1250	0.2500	0.0792
83	Co	5.2138	0.0000	1.7379	0.3750	0.0000	0.0792
84	Co	5.2138	3.4758	1.7379	0.3750	0.2500	0.0792
85	Co	1.7379	6.9517	1.7379	0.1250	0.5000	0.0792
86	Co	1.7379	10.4275	1.7379	0.1250	0.7500	0.0792
87	Co	5.2138	6.9517	1.7379	0.3750	0.5000	0.0792
88	Co	5.2138	10.4275	1.7379	0.3750	0.7500	0.0792

89	Co	8.6896	0.0000	1.7379	0.6250	0.0000	0.0792
90	Co	8.6896	3.4758	1.7379	0.6250	0.2500	0.0792
91	Co	12.1654	0.0000	1.7379	0.8750	0.0000	0.0792
92	Co	12.1654	3.4758	1.7379	0.8750	0.2500	0.0792
93	Co	8.6896	6.9517	1.7379	0.6250	0.5000	0.0792
94	Co	8.6896	10.4275	1.7379	0.6250	0.7500	0.0792
95	Co	12.1654	6.9517	1.7379	0.8750	0.5000	0.0792
96	Co	12.1654	10.4275	1.7379	0.8750	0.7500	0.0792
97	Co	1.7380	1.7289	3.5277	0.1250	0.1244	0.1607
98	Co	1.7150	5.2437	3.5315	0.1234	0.3772	0.1609
99	Co	5.2089	1.7555	3.5935	0.3747	0.1263	0.1637
100	Co	5.2222	5.2007	3.5173	0.3756	0.3741	0.1602
101	Co	1.7209	8.6761	3.5478	0.1238	0.6240	0.1616
102	Co	1.7346	12.1687	3.5375	0.1248	0.8752	0.1611
103	Co	5.2191	8.6947	3.5066	0.3754	0.6254	0.1597
104	Co	5.2230	12.1796	3.5403	0.3757	0.8760	0.1613
105	Co	8.6945	1.7429	3.5466	0.6254	0.1254	0.1616
106	Co	8.7222	5.1961	3.4973	0.6273	0.3737	0.1593
107	Co	12.1709	1.7360	3.5555	0.8754	0.1249	0.1620
108	Co	12.1703	5.2227	3.5537	0.8754	0.3756	0.1619
109	Co	8.6864	8.6922	3.5732	0.6248	0.6252	0.1628
110	Co	8.6840	12.1534	3.5436	0.6246	0.8741	0.1614
111	Co	12.1430	8.6900	3.5906	0.8734	0.6250	0.1636
112	Co	12.1646	12.1646	3.5429	0.8749	0.8749	0.1614
113	Co	1.7317	-0.0019	5.2207	0.1246	-0.0001	0.2378
114	Co	1.7322	3.4834	5.1538	0.1246	0.2505	0.2348
115	Co	5.2135	13.8982	5.2194	0.3750	0.9996	0.2378
116	Co	5.1856	3.5295	5.4371	0.3730	0.2539	0.2477
117	Co	1.8311	6.9575	5.4860	0.1317	0.5004	0.2499
118	Co	1.7496	10.4281	5.2098	0.1258	0.7500	0.2373
119	Co	5.2204	6.9289	5.2004	0.3755	0.4984	0.2369
120	Co	5.1830	10.4648	5.1896	0.3728	0.7527	0.2364
121	Co	8.6889	0.0030	5.2121	0.6250	0.0002	0.2374
122	Co	8.6982	3.4821	5.2003	0.6256	0.2505	0.2369
123	Co	12.1734	0.0002	5.2214	0.8756	0.0000	0.2379
124	Co	12.1735	3.4794	5.2222	0.8756	0.2503	0.2379
125	Co	8.6926	6.8714	5.1666	0.6252	0.4942	0.2354
126	Co	8.6713	10.4376	5.3303	0.6237	0.7507	0.2428
127	Co	12.1660	6.9508	5.2149	0.8750	0.4999	0.2376
128	Co	12.2004	10.4397	5.2119	0.8775	0.7509	0.2374
129	O	4.9956	3.7171	7.2406	0.3593	0.2674	0.3298
130	O	2.1628	6.8471	7.2654	0.1556	0.4925	0.3310
131	O	4.5682	6.1604	8.7057	0.3286	0.4431	0.3966
132	O	6.1938	7.6204	6.7643	0.4455	0.5481	0.3081
133	O	8.0040	6.8620	8.8909	0.5757	0.4936	0.4050
134	O	5.7533	9.4705	8.8828	0.4138	0.6812	0.4047
135	O	9.4334	9.5917	6.7346	0.6785	0.6899	0.3068
136	Ce	4.1732	8.3411	7.9585	0.3002	0.5999	0.3625
137	Ce	3.0413	4.7941	7.8148	0.2187	0.3448	0.3560

138 Ce	6.6564	5.4718	7.9228	0.4788	0.3936	0.3609
139 Sm	7.6947	8.8326	8.1655	0.5534	0.6353	0.3720

/db/jmorales/CoNi-alloy/metal-SDC-interface/Ni-Ce_3SmO_7/Ni-Ce_3SmO_7-conf1b

a = 13.9019837372
b = 13.9019837372
c = 21.9509918687
alpha = 90.0
beta = 90.0
gamma = 90.0

	Atom	X	Y	Z	X	Y	Z
1	Ni	0.0000	0.0000	0.0000	0.0000	0.0000	0.0000
2	Ni	1.7377	1.7377	0.0000	0.1250	0.1250	0.0000
3	Ni	0.0000	3.4755	0.0000	0.0000	0.2500	0.0000
4	Ni	1.7377	5.2132	0.0000	0.1250	0.3750	0.0000
5	Ni	3.4755	0.0000	0.0000	0.2500	0.0000	0.0000
6	Ni	5.2132	1.7377	0.0000	0.3750	0.1250	0.0000
7	Ni	3.4755	3.4755	0.0000	0.2500	0.2500	0.0000
8	Ni	5.2132	5.2132	0.0000	0.3750	0.3750	0.0000
9	Ni	0.0000	6.9510	0.0000	0.0000	0.5000	0.0000
10	Ni	1.7377	8.6887	0.0000	0.1250	0.6250	0.0000
11	Ni	0.0000	10.4265	0.0000	0.0000	0.7500	0.0000
12	Ni	1.7377	12.1642	0.0000	0.1250	0.8750	0.0000
13	Ni	3.4755	6.9510	0.0000	0.2500	0.5000	0.0000
14	Ni	5.2132	8.6887	0.0000	0.3750	0.6250	0.0000
15	Ni	3.4755	10.4265	0.0000	0.2500	0.7500	0.0000
16	Ni	5.2132	12.1642	0.0000	0.3750	0.8750	0.0000
17	Ni	6.9510	0.0000	0.0000	0.5000	0.0000	0.0000
18	Ni	8.6887	1.7377	0.0000	0.6250	0.1250	0.0000
19	Ni	6.9510	3.4755	0.0000	0.5000	0.2500	0.0000
20	Ni	8.6887	5.2132	0.0000	0.6250	0.3750	0.0000
21	Ni	10.4265	0.0000	0.0000	0.7500	0.0000	0.0000
22	Ni	12.1642	1.7377	0.0000	0.8750	0.1250	0.0000
23	Ni	10.4265	3.4755	0.0000	0.7500	0.2500	0.0000
24	Ni	12.1642	5.2132	0.0000	0.8750	0.3750	0.0000
25	Ni	6.9510	6.9510	0.0000	0.5000	0.5000	0.0000
26	Ni	8.6887	8.6887	0.0000	0.6250	0.6250	0.0000
27	Ni	6.9510	10.4265	0.0000	0.5000	0.7500	0.0000
28	Ni	8.6887	12.1642	0.0000	0.6250	0.8750	0.0000
29	Ni	10.4265	6.9510	0.0000	0.7500	0.5000	0.0000
30	Ni	12.1642	8.6887	0.0000	0.8750	0.6250	0.0000
31	Ni	10.4265	10.4265	0.0000	0.7500	0.7500	0.0000
32	Ni	12.1642	12.1642	0.0000	0.8750	0.8750	0.0000
33	Ni	0.0000	1.7377	1.7377	0.0000	0.1250	0.0792
34	Ni	1.7377	0.0000	1.7377	0.1250	0.0000	0.0792
35	Ni	0.0000	5.2132	1.7377	0.0000	0.3750	0.0792
36	Ni	1.7377	3.4755	1.7377	0.1250	0.2500	0.0792
37	Ni	3.4755	1.7377	1.7377	0.2500	0.1250	0.0792
38	Ni	5.2132	0.0000	1.7377	0.3750	0.0000	0.0792
39	Ni	3.4755	5.2132	1.7377	0.2500	0.3750	0.0792
40	Ni	5.2132	3.4755	1.7377	0.3750	0.2500	0.0792

41	Ni	0.0000	8.6887	1.7377	0.0000	0.6250	0.0792
42	Ni	1.7377	6.9510	1.7377	0.1250	0.5000	0.0792
43	Ni	0.0000	12.1642	1.7377	0.0000	0.8750	0.0792
44	Ni	1.7377	10.4265	1.7377	0.1250	0.7500	0.0792
45	Ni	3.4755	8.6887	1.7377	0.2500	0.6250	0.0792
46	Ni	5.2132	6.9510	1.7377	0.3750	0.5000	0.0792
47	Ni	3.4755	12.1642	1.7377	0.2500	0.8750	0.0792
48	Ni	5.2132	10.4265	1.7377	0.3750	0.7500	0.0792
49	Ni	6.9510	1.7377	1.7377	0.5000	0.1250	0.0792
50	Ni	8.6887	0.0000	1.7377	0.6250	0.0000	0.0792
51	Ni	6.9510	5.2132	1.7377	0.5000	0.3750	0.0792
52	Ni	8.6887	3.4755	1.7377	0.6250	0.2500	0.0792
53	Ni	10.4265	1.7377	1.7377	0.7500	0.1250	0.0792
54	Ni	12.1642	0.0000	1.7377	0.8750	0.0000	0.0792
55	Ni	10.4265	5.2132	1.7377	0.7500	0.3750	0.0792
56	Ni	12.1642	3.4755	1.7377	0.8750	0.2500	0.0792
57	Ni	6.9510	8.6887	1.7377	0.5000	0.6250	0.0792
58	Ni	8.6887	6.9510	1.7377	0.6250	0.5000	0.0792
59	Ni	6.9510	12.1642	1.7377	0.5000	0.8750	0.0792
60	Ni	8.6887	10.4265	1.7377	0.6250	0.7500	0.0792
61	Ni	10.4265	8.6887	1.7377	0.7500	0.6250	0.0792
62	Ni	12.1642	6.9510	1.7377	0.8750	0.5000	0.0792
63	Ni	10.4265	12.1642	1.7377	0.7500	0.8750	0.0792
64	Ni	12.1642	10.4265	1.7377	0.8750	0.7500	0.0792
65	Ni	0.0018	0.0016	3.5084	0.0001	0.0001	0.1598
66	Ni	1.7406	1.7360	3.5023	0.1252	0.1249	0.1595
67	Ni	13.8976	3.4734	3.5029	0.9997	0.2498	0.1596
68	Ni	1.7235	5.2219	3.5008	0.1240	0.3756	0.1595
69	Ni	3.4758	13.9019	3.5083	0.2500	1.0000	0.1598
70	Ni	5.2117	1.7514	3.5175	0.3749	0.1260	0.1602
71	Ni	3.4903	3.4898	3.5140	0.2511	0.2510	0.1601
72	Ni	5.2140	5.2255	3.5138	0.3751	0.3759	0.1601
73	Ni	0.0159	6.9432	3.5407	0.0011	0.4994	0.1613
74	Ni	1.7074	8.6673	3.5032	0.1228	0.6235	0.1596
75	Ni	-0.0004	10.4264	3.5080	-0.0000	0.7500	0.1598
76	Ni	1.7284	12.1732	3.5093	0.1243	0.8756	0.1599
77	Ni	3.4728	6.9596	3.4851	0.2498	0.5006	0.1588
78	Ni	5.2292	8.6962	3.4700	0.3761	0.6255	0.1581
79	Ni	3.4585	10.4594	3.4674	0.2488	0.7524	0.1580
80	Ni	5.2294	12.1771	3.5131	0.3762	0.8759	0.1600
81	Ni	6.9518	13.8842	3.5176	0.5001	0.9987	0.1602
82	Ni	8.6950	1.7262	3.5007	0.6255	0.1242	0.1595
83	Ni	6.9517	3.4665	3.4958	0.5001	0.2494	0.1593
84	Ni	8.6781	5.1961	3.5104	0.6242	0.3738	0.1599
85	Ni	10.4359	-0.0014	3.5037	0.7507	-0.0001	0.1596
86	Ni	12.1703	1.7360	3.5047	0.8754	0.1249	0.1597
87	Ni	10.4378	3.4738	3.5004	0.7508	0.2499	0.1595
88	Ni	12.1653	5.2165	3.5084	0.8751	0.3752	0.1598
89	Ni	6.9593	6.9263	3.4972	0.5006	0.4982	0.1593

90	Ni	8.6791	8.7046	3.5209	0.6243	0.6261	0.1604
91	Ni	6.9592	10.4335	3.5095	0.5006	0.7505	0.1599
92	Ni	8.6916	12.1665	3.5075	0.6252	0.8752	0.1598
93	Ni	10.4303	6.9509	3.5011	0.7503	0.5000	0.1595
94	Ni	12.1666	8.6848	3.5115	0.8752	0.6247	0.1600
95	Ni	10.4162	10.4243	3.5184	0.7493	0.7498	0.1603
96	Ni	12.1680	12.1612	3.5075	0.8753	0.8748	0.1598
97	Ni	0.0026	1.7434	5.2263	0.0002	0.1254	0.2381
98	Ni	1.7365	0.0059	5.2277	0.1249	0.0004	0.2382
99	Ni	13.8835	5.1903	5.2216	0.9987	0.3734	0.2379
100	Ni	1.7376	3.4553	5.1990	0.1250	0.2485	0.2368
101	Ni	3.4667	1.7464	5.2286	0.2494	0.1256	0.2382
102	Ni	5.1987	0.0133	5.2252	0.3740	0.0010	0.2380
103	Ni	3.4522	5.2372	5.2018	0.2483	0.3767	0.2370
104	Ni	5.2134	3.4387	5.3067	0.3750	0.2474	0.2418
105	Ni	-0.0008	8.6911	5.2362	-0.0001	0.6252	0.2385
106	Ni	1.7298	6.9829	5.4074	0.1244	0.5023	0.2463
107	Ni	13.9004	12.1613	5.2273	0.9999	0.8748	0.2381
108	Ni	1.7394	10.4227	5.2179	0.1251	0.7497	0.2377
109	Ni	3.4511	8.7332	5.0907	0.2482	0.6282	0.2319
110	Ni	5.2118	6.9963	5.2356	0.3749	0.5033	0.2385
111	Ni	3.4678	12.1678	5.2234	0.2494	0.8753	0.2380
112	Ni	5.1681	10.4357	5.1629	0.3718	0.7507	0.2352
113	Ni	6.9645	1.7120	5.2005	0.5010	0.1231	0.2369
114	Ni	8.7122	0.0222	5.2221	0.6267	0.0016	0.2379
115	Ni	6.9936	5.1680	5.2941	0.5031	0.3717	0.2412
116	Ni	8.7281	3.4463	5.2033	0.6278	0.2479	0.2370
117	Ni	10.4448	1.7308	5.2219	0.7513	0.1245	0.2379
118	Ni	12.1783	0.0105	5.2257	0.8760	0.0008	0.2381
119	Ni	10.4261	5.2123	5.2248	0.7500	0.3749	0.2380
120	Ni	12.1609	3.4712	5.2252	0.8748	0.2497	0.2380
121	Ni	6.9907	8.6867	5.2489	0.5029	0.6249	0.2391
122	Ni	8.7084	6.9217	5.1864	0.6264	0.4979	0.2363
123	Ni	6.9062	12.1789	5.3567	0.4968	0.8761	0.2440
124	Ni	8.7209	10.4244	5.3250	0.6273	0.7499	0.2426
125	Ni	10.4199	8.6771	5.2253	0.7495	0.6242	0.2380
126	Ni	12.1632	6.9508	5.2296	0.8749	0.5000	0.2382
127	Ni	10.4729	12.1819	5.2190	0.7533	0.8763	0.2378
128	Ni	12.1658	10.4167	5.2226	0.8751	0.7493	0.2379
129	O	6.0290	4.3775	6.8057	0.4337	0.3149	0.3100
130	O	2.7288	6.0851	6.8230	0.1963	0.4377	0.3108
131	O	4.9326	6.2292	8.8152	0.3548	0.4481	0.4016
132	O	6.0170	7.9913	6.7864	0.4328	0.5748	0.3092
133	O	7.9482	8.1709	8.8908	0.5717	0.5878	0.4050
134	O	4.9498	9.8296	8.7512	0.3561	0.7071	0.3987
135	O	8.0045	11.4876	6.6620	0.5758	0.8263	0.3035
136	Ce	3.8776	8.1879	7.9056	0.2789	0.5890	0.3601
137	Ce	3.9908	4.4808	7.8371	0.2871	0.3223	0.3570
138	Ce	7.1869	6.3686	8.0507	0.5170	0.4581	0.3668

139 Sm	6.9702	9.8757	8.0146	0.5014	0.7104	0.3651

/db/jmorales/CoNi-alloy/metal-SDC-interface/CoNi-Ce_3SmO_7/CoNi-Ce_3SmO_7-conf6d

a =				13.9033384264			
b =				13.9033384264			
c =				21.9516692133			
alpha =				90.0			
beta =				90.0			
gamma =				90.0			
	Atom	X	Y	Z	X	Y	Z
1	Ni	0.0000	0.0000	0.0000	0.0000	0.0000	0.0000
2	Ni	0.0000	3.4758	0.0000	0.0000	0.2500	0.0000
3	Ni	3.4758	0.0000	0.0000	0.2500	0.0000	0.0000
4	Ni	3.4758	3.4758	0.0000	0.2500	0.2500	0.0000
5	Ni	0.0000	6.9517	0.0000	0.0000	0.5000	0.0000
6	Ni	0.0000	10.4275	0.0000	0.0000	0.7500	0.0000
7	Ni	3.4758	6.9517	0.0000	0.2500	0.5000	0.0000
8	Ni	3.4758	10.4275	0.0000	0.2500	0.7500	0.0000
9	Ni	6.9517	0.0000	0.0000	0.5000	0.0000	0.0000
10	Ni	6.9517	3.4758	0.0000	0.5000	0.2500	0.0000
11	Ni	10.4275	0.0000	0.0000	0.7500	0.0000	0.0000
12	Ni	10.4275	3.4758	0.0000	0.7500	0.2500	0.0000
13	Ni	6.9517	6.9517	0.0000	0.5000	0.5000	0.0000
14	Ni	6.9517	10.4275	0.0000	0.5000	0.7500	0.0000
15	Ni	10.4275	6.9517	0.0000	0.7500	0.5000	0.0000
16	Ni	10.4275	10.4275	0.0000	0.7500	0.7500	0.0000
17	Ni	0.0000	1.7379	1.7379	0.0000	0.1250	0.0792
18	Ni	0.0000	5.2138	1.7379	0.0000	0.3750	0.0792
19	Ni	3.4758	1.7379	1.7379	0.2500	0.1250	0.0792
20	Ni	3.4758	5.2138	1.7379	0.2500	0.3750	0.0792
21	Ni	0.0000	8.6896	1.7379	0.0000	0.6250	0.0792
22	Ni	0.0000	12.1654	1.7379	0.0000	0.8750	0.0792
23	Ni	3.4758	8.6896	1.7379	0.2500	0.6250	0.0792
24	Ni	3.4758	12.1654	1.7379	0.2500	0.8750	0.0792
25	Ni	6.9517	1.7379	1.7379	0.5000	0.1250	0.0792
26	Ni	6.9517	5.2138	1.7379	0.5000	0.3750	0.0792
27	Ni	10.4275	1.7379	1.7379	0.7500	0.1250	0.0792
28	Ni	10.4275	5.2138	1.7379	0.7500	0.3750	0.0792
29	Ni	6.9517	8.6896	1.7379	0.5000	0.6250	0.0792
30	Ni	6.9517	12.1654	1.7379	0.5000	0.8750	0.0792
31	Ni	10.4275	8.6896	1.7379	0.7500	0.6250	0.0792
32	Ni	10.4275	12.1654	1.7379	0.7500	0.8750	0.0792
33	Ni	13.8923	13.8942	3.4954	0.9992	0.9993	0.1592
34	Ni	-0.0005	3.4715	3.5009	-0.0000	0.2497	0.1595
35	Ni	3.4858	0.0220	3.5227	0.2507	0.0016	0.1605
36	Ni	3.4972	3.4884	3.5603	0.2515	0.2509	0.1622
37	Ni	0.0116	6.9499	3.5186	0.0008	0.4999	0.1603
38	Ni	-0.0006	10.4266	3.5039	-0.0000	0.7499	0.1596
39	Ni	3.4709	6.9546	3.4851	0.2496	0.5002	0.1588

40	Ni	3.4694	10.4508	3.4751	0.2495	0.7517	0.1583
41	Ni	6.9469	0.0018	3.5046	0.4997	0.0001	0.1596
42	Ni	6.9405	3.4451	3.4920	0.4992	0.2478	0.1591
43	Ni	10.4251	13.8955	3.5028	0.7498	0.9994	0.1596
44	Ni	10.4365	3.4659	3.4924	0.7506	0.2493	0.1591
45	Ni	6.9694	6.9610	3.4553	0.5013	0.5007	0.1574
46	Ni	6.9490	10.4128	3.5104	0.4998	0.7489	0.1599
47	Ni	10.4437	6.9625	3.4913	0.7512	0.5008	0.1590
48	Ni	10.4284	10.4246	3.5005	0.7501	0.7498	0.1595
49	Ni	13.8511	1.7056	5.1863	0.9962	0.1227	0.2363
50	Ni	13.8698	5.1914	5.1944	0.9976	0.3734	0.2366
51	Ni	3.4418	1.6698	5.3567	0.2476	0.1201	0.2440
52	Ni	3.4728	5.2714	5.2247	0.2498	0.3791	0.2380
53	Ni	0.0036	8.6965	5.2158	0.0003	0.6255	0.2376
54	Ni	13.8947	12.1533	5.2066	0.9994	0.8741	0.2372
55	Ni	3.4719	8.7285	5.1103	0.2497	0.6278	0.2328
56	Ni	3.4772	12.1707	5.2042	0.2501	0.8754	0.2371
57	Ni	6.9954	1.7125	5.2129	0.5031	0.1232	0.2375
58	Ni	7.0363	5.2209	5.0733	0.5061	0.3755	0.2311
59	Ni	10.4260	1.7300	5.2072	0.7499	0.1244	0.2372
60	Ni	10.4301	5.2060	5.2041	0.7502	0.3744	0.2371
61	Ni	6.9640	8.6971	5.2278	0.5009	0.6255	0.2382
62	Ni	6.9658	12.1510	5.2157	0.5010	0.8740	0.2376
63	Ni	10.4184	8.6970	5.2196	0.7493	0.6255	0.2378
64	Ni	10.4137	12.1534	5.2119	0.7490	0.8741	0.2374
65	Co	1.7379	1.7379	0.0000	0.1250	0.1250	0.0000
66	Co	1.7379	5.2138	0.0000	0.1250	0.3750	0.0000
67	Co	5.2138	1.7379	0.0000	0.3750	0.1250	0.0000
68	Co	5.2138	5.2138	0.0000	0.3750	0.3750	0.0000
69	Co	1.7379	8.6896	0.0000	0.1250	0.6250	0.0000
70	Co	1.7379	12.1654	0.0000	0.1250	0.8750	0.0000
71	Co	5.2138	8.6896	0.0000	0.3750	0.6250	0.0000
72	Co	5.2138	12.1654	0.0000	0.3750	0.8750	0.0000
73	Co	8.6896	1.7379	0.0000	0.6250	0.1250	0.0000
74	Co	8.6896	5.2138	0.0000	0.6250	0.3750	0.0000
75	Co	12.1654	1.7379	0.0000	0.8750	0.1250	0.0000
76	Co	12.1654	5.2138	0.0000	0.8750	0.3750	0.0000
77	Co	8.6896	8.6896	0.0000	0.6250	0.6250	0.0000
78	Co	8.6896	12.1654	0.0000	0.6250	0.8750	0.0000
79	Co	12.1654	8.6896	0.0000	0.8750	0.6250	0.0000
80	Co	12.1654	12.1654	0.0000	0.8750	0.8750	0.0000
81	Co	1.7379	0.0000	1.7379	0.1250	0.0000	0.0792
82	Co	1.7379	3.4758	1.7379	0.1250	0.2500	0.0792
83	Co	5.2138	0.0000	1.7379	0.3750	0.0000	0.0792
84	Co	5.2138	3.4758	1.7379	0.3750	0.2500	0.0792
85	Co	1.7379	6.9517	1.7379	0.1250	0.5000	0.0792
86	Co	1.7379	10.4275	1.7379	0.1250	0.7500	0.0792
87	Co	5.2138	6.9517	1.7379	0.3750	0.5000	0.0792
88	Co	5.2138	10.4275	1.7379	0.3750	0.7500	0.0792

89	Co	8.6896	0.0000	1.7379	0.6250	0.0000	0.0792
90	Co	8.6896	3.4758	1.7379	0.6250	0.2500	0.0792
91	Co	12.1654	0.0000	1.7379	0.8750	0.0000	0.0792
92	Co	12.1654	3.4758	1.7379	0.8750	0.2500	0.0792
93	Co	8.6896	6.9517	1.7379	0.6250	0.5000	0.0792
94	Co	8.6896	10.4275	1.7379	0.6250	0.7500	0.0792
95	Co	12.1654	6.9517	1.7379	0.8750	0.5000	0.0792
96	Co	12.1654	10.4275	1.7379	0.8750	0.7500	0.0792
97	Co	1.7462	1.7297	3.5370	0.1256	0.1244	0.1611
98	Co	1.7272	5.2055	3.5532	0.1242	0.3744	0.1619
99	Co	5.1884	1.7673	3.6240	0.3732	0.1271	0.1651
100	Co	5.2004	5.1960	3.5534	0.3740	0.3737	0.1619
101	Co	1.7132	8.6734	3.5354	0.1232	0.6238	0.1611
102	Co	1.7356	12.1683	3.5473	0.1248	0.8752	0.1616
103	Co	5.2198	8.7043	3.5093	0.3754	0.6261	0.1599
104	Co	5.2202	12.1708	3.5462	0.3755	0.8754	0.1615
105	Co	8.6948	1.7315	3.5443	0.6254	0.1245	0.1615
106	Co	8.7297	5.2103	3.4948	0.6279	0.3747	0.1592
107	Co	12.1584	1.7253	3.5407	0.8745	0.1241	0.1613
108	Co	12.1640	5.2140	3.5448	0.8749	0.3750	0.1615
109	Co	8.6886	8.6933	3.5486	0.6249	0.6253	0.1617
110	Co	8.6850	12.1647	3.5347	0.6247	0.8749	0.1610
111	Co	12.1676	8.6922	3.5638	0.8752	0.6252	0.1623
112	Co	12.1590	12.1566	3.5515	0.8745	0.8744	0.1618
113	Co	1.7085	13.8567	5.2064	0.1229	0.9966	0.2372
114	Co	1.6937	3.4275	5.2569	0.1218	0.2465	0.2395
115	Co	5.2359	13.8863	5.2222	0.3766	0.9988	0.2379
116	Co	5.3313	3.5532	5.4077	0.3835	0.2556	0.2463
117	Co	1.7229	6.9616	5.3335	0.1239	0.5007	0.2430
118	Co	1.7511	10.4198	5.2178	0.1259	0.7494	0.2377
119	Co	5.2511	6.9653	5.1551	0.3777	0.5010	0.2348
120	Co	5.1841	10.4535	5.2066	0.3729	0.7519	0.2372
121	Co	8.6928	13.8884	5.2253	0.6252	0.9989	0.2380
122	Co	8.7033	3.4812	5.1960	0.6260	0.2504	0.2367
123	Co	12.1509	13.8795	5.2255	0.8740	0.9983	0.2380
124	Co	12.1591	3.4714	5.2132	0.8745	0.2497	0.2375
125	Co	8.7413	6.9293	5.1843	0.6287	0.4984	0.2362
126	Co	8.6895	10.4244	5.2202	0.6250	0.7498	0.2378
127	Co	12.1658	6.9569	5.2211	0.8750	0.5004	0.2378
128	Co	12.1603	10.4250	5.2261	0.8746	0.7498	0.2381
129	O	2.4243	2.5776	6.7088	0.1744	0.1854	0.3056
130	O	5.4249	3.6917	7.2453	0.3902	0.2655	0.3301
131	O	2.6186	6.1466	6.8007	0.1883	0.4421	0.3098
132	O	4.7260	6.0411	8.8053	0.3399	0.4345	0.4011
133	O	6.1027	7.6508	6.7611	0.4389	0.5503	0.3080
134	O	8.0065	7.2804	8.8350	0.5759	0.5236	0.4025
135	O	5.4526	9.4587	8.8192	0.3922	0.6803	0.4018
136	Ce	4.0383	8.0619	7.9376	0.2905	0.5799	0.3616
137	Ce	3.4071	4.2916	7.9781	0.2451	0.3087	0.3634

138 Ce	6.8454	5.6511	7.9376	0.4924	0.4065	0.3616
139 Sm	7.4018	9.1422	8.0647	0.5324	0.6576	0.3674

/db/jmorales/CoNi-alloy/metal-SDC-interface/CoNi-Ce_3SmO_7/CoNi-Ce_3SmO_7-conf5b

a = 13.9033384264
b = 13.9033384264
c = 21.9516692133
alpha = 90.0
beta = 90.0
gamma = 90.0

	Atom	X	Y	Z	X	Y	Z
1	Ni	0.0000	0.0000	0.0000	0.0000	0.0000	0.0000
2	Ni	0.0000	3.4758	0.0000	0.0000	0.2500	0.0000
3	Ni	3.4758	0.0000	0.0000	0.2500	0.0000	0.0000
4	Ni	3.4758	3.4758	0.0000	0.2500	0.2500	0.0000
5	Ni	0.0000	6.9517	0.0000	0.0000	0.5000	0.0000
6	Ni	0.0000	10.4275	0.0000	0.0000	0.7500	0.0000
7	Ni	3.4758	6.9517	0.0000	0.2500	0.5000	0.0000
8	Ni	3.4758	10.4275	0.0000	0.2500	0.7500	0.0000
9	Ni	6.9517	0.0000	0.0000	0.5000	0.0000	0.0000
10	Ni	6.9517	3.4758	0.0000	0.5000	0.2500	0.0000
11	Ni	10.4275	0.0000	0.0000	0.7500	0.0000	0.0000
12	Ni	10.4275	3.4758	0.0000	0.7500	0.2500	0.0000
13	Ni	6.9517	6.9517	0.0000	0.5000	0.5000	0.0000
14	Ni	6.9517	10.4275	0.0000	0.5000	0.7500	0.0000
15	Ni	10.4275	6.9517	0.0000	0.7500	0.5000	0.0000
16	Ni	10.4275	10.4275	0.0000	0.7500	0.7500	0.0000
17	Ni	0.0000	1.7379	1.7379	0.0000	0.1250	0.0792
18	Ni	0.0000	5.2138	1.7379	0.0000	0.3750	0.0792
19	Ni	3.4758	1.7379	1.7379	0.2500	0.1250	0.0792
20	Ni	3.4758	5.2138	1.7379	0.2500	0.3750	0.0792
21	Ni	0.0000	8.6896	1.7379	0.0000	0.6250	0.0792
22	Ni	0.0000	12.1654	1.7379	0.0000	0.8750	0.0792
23	Ni	3.4758	8.6896	1.7379	0.2500	0.6250	0.0792
24	Ni	3.4758	12.1654	1.7379	0.2500	0.8750	0.0792
25	Ni	6.9517	1.7379	1.7379	0.5000	0.1250	0.0792
26	Ni	6.9517	5.2138	1.7379	0.5000	0.3750	0.0792
27	Ni	10.4275	1.7379	1.7379	0.7500	0.1250	0.0792
28	Ni	10.4275	5.2138	1.7379	0.7500	0.3750	0.0792
29	Ni	6.9517	8.6896	1.7379	0.5000	0.6250	0.0792
30	Ni	6.9517	12.1654	1.7379	0.5000	0.8750	0.0792
31	Ni	10.4275	8.6896	1.7379	0.7500	0.6250	0.0792
32	Ni	10.4275	12.1654	1.7379	0.7500	0.8750	0.0792
33	Ni	13.8998	0.0016	3.5045	0.9997	0.0001	0.1596
34	Ni	0.0034	3.4736	3.5052	0.0002	0.2498	0.1597
35	Ni	3.4744	13.8981	3.5063	0.2499	0.9996	0.1597
36	Ni	3.4478	3.4901	3.4935	0.2480	0.2510	0.1591
37	Ni	13.9031	6.9480	3.5061	1.0000	0.4997	0.1597
38	Ni	13.9008	10.4355	3.5044	0.9998	0.7506	0.1596
39	Ni	3.4751	6.9477	3.5073	0.2499	0.4997	0.1598

40	Ni	3.4718	10.4383	3.4885	0.2497	0.7508	0.1589
41	Ni	6.9610	13.8925	3.4986	0.5007	0.9992	0.1594
42	Ni	6.9808	3.4793	3.4968	0.5021	0.2503	0.1593
43	Ni	10.4188	0.0076	3.5073	0.7494	0.0005	0.1598
44	Ni	10.4066	3.4735	3.5281	0.7485	0.2498	0.1607
45	Ni	6.9170	6.9481	3.5082	0.4975	0.4997	0.1598
46	Ni	6.9389	10.4133	3.5001	0.4991	0.7490	0.1594
47	Ni	10.4631	6.9585	3.4683	0.7526	0.5005	0.1580
48	Ni	10.4475	10.4279	3.4905	0.7514	0.7500	0.1590
49	Ni	13.8961	1.7330	5.2086	0.9995	0.1246	0.2373
50	Ni	13.8991	5.2137	5.2090	0.9997	0.3750	0.2373
51	Ni	3.4821	1.7375	5.2028	0.2505	0.1250	0.2370
52	Ni	3.4599	5.1287	5.3328	0.2489	0.3689	0.2429
53	Ni	0.0007	8.6995	5.2083	0.0001	0.6257	0.2373
54	Ni	13.9024	12.1677	5.2101	0.9999	0.8752	0.2373
55	Ni	3.4577	8.7261	5.1769	0.2487	0.6276	0.2358
56	Ni	3.4677	12.1734	5.2087	0.2494	0.8756	0.2373
57	Ni	6.9278	1.7199	5.1925	0.4983	0.1237	0.2365
58	Ni	6.9847	5.2464	5.2396	0.5024	0.3773	0.2387
59	Ni	10.4090	1.7424	5.2246	0.7487	0.1253	0.2380
60	Ni	10.4527	5.2446	5.1882	0.7518	0.3772	0.2363
61	Ni	6.9656	8.7046	5.2334	0.5010	0.6261	0.2384
62	Ni	6.9627	12.1418	5.2150	0.5008	0.8733	0.2376
63	Ni	10.4195	8.6954	5.2032	0.7494	0.6254	0.2370
64	Ni	10.4210	12.1699	5.2085	0.7495	0.8753	0.2373
65	Co	1.7379	1.7379	0.0000	0.1250	0.1250	0.0000
66	Co	1.7379	5.2138	0.0000	0.1250	0.3750	0.0000
67	Co	5.2138	1.7379	0.0000	0.3750	0.1250	0.0000
68	Co	5.2138	5.2138	0.0000	0.3750	0.3750	0.0000
69	Co	1.7379	8.6896	0.0000	0.1250	0.6250	0.0000
70	Co	1.7379	12.1654	0.0000	0.1250	0.8750	0.0000
71	Co	5.2138	8.6896	0.0000	0.3750	0.6250	0.0000
72	Co	5.2138	12.1654	0.0000	0.3750	0.8750	0.0000
73	Co	8.6896	1.7379	0.0000	0.6250	0.1250	0.0000
74	Co	8.6896	5.2138	0.0000	0.6250	0.3750	0.0000
75	Co	12.1654	1.7379	0.0000	0.8750	0.1250	0.0000
76	Co	12.1654	5.2138	0.0000	0.8750	0.3750	0.0000
77	Co	8.6896	8.6896	0.0000	0.6250	0.6250	0.0000
78	Co	8.6896	12.1654	0.0000	0.6250	0.8750	0.0000
79	Co	12.1654	8.6896	0.0000	0.8750	0.6250	0.0000
80	Co	12.1654	12.1654	0.0000	0.8750	0.8750	0.0000
81	Co	1.7379	0.0000	1.7379	0.1250	0.0000	0.0792
82	Co	1.7379	3.4758	1.7379	0.1250	0.2500	0.0792
83	Co	5.2138	0.0000	1.7379	0.3750	0.0000	0.0792
84	Co	5.2138	3.4758	1.7379	0.3750	0.2500	0.0792
85	Co	1.7379	6.9517	1.7379	0.1250	0.5000	0.0792
86	Co	1.7379	10.4275	1.7379	0.1250	0.7500	0.0792
87	Co	5.2138	6.9517	1.7379	0.3750	0.5000	0.0792
88	Co	5.2138	10.4275	1.7379	0.3750	0.7500	0.0792

89 Co	8.6896	0.0000	1.7379	0.6250	0.0000	0.0792
90 Co	8.6896	3.4758	1.7379	0.6250	0.2500	0.0792
91 Co	12.1654	0.0000	1.7379	0.8750	0.0000	0.0792
92 Co	12.1654	3.4758	1.7379	0.8750	0.2500	0.0792
93 Co	8.6896	6.9517	1.7379	0.6250	0.5000	0.0792
94 Co	8.6896	10.4275	1.7379	0.6250	0.7500	0.0792
95 Co	12.1654	6.9517	1.7379	0.8750	0.5000	0.0792
96 Co	12.1654	10.4275	1.7379	0.8750	0.7500	0.0792
97 Co	1.7381	1.7292	3.5510	0.1250	0.1244	0.1618
98 Co	1.7515	5.2159	3.5733	0.1260	0.3752	0.1628
99 Co	5.2145	1.6989	3.5104	0.3751	0.1222	0.1599
100 Co	5.2136	5.2206	3.5447	0.3750	0.3755	0.1615
101 Co	1.7281	8.6903	3.5360	0.1243	0.6250	0.1611
102 Co	1.7332	12.1720	3.5516	0.1247	0.8755	0.1618
103 Co	5.2164	8.6791	3.5605	0.3752	0.6242	0.1622
104 Co	5.2170	12.1673	3.5521	0.3752	0.8751	0.1618
105 Co	8.6885	1.7606	3.5808	0.6249	0.1266	0.1631
106 Co	8.6949	5.1915	3.5146	0.6254	0.3734	0.1601
107 Co	12.1559	1.7405	3.5583	0.8743	0.1252	0.1621
108 Co	12.1725	5.2079	3.5415	0.8755	0.3746	0.1613
109 Co	8.6812	8.7111	3.5100	0.6244	0.6265	0.1599
110 Co	8.6905	12.1715	3.5303	0.6251	0.8754	0.1608
111 Co	12.1712	8.6955	3.5538	0.8754	0.6254	0.1619
112 Co	12.1630	12.1715	3.5565	0.8748	0.8754	0.1620
113 Co	1.7350	0.0021	5.2294	0.1248	0.0002	0.2382
114 Co	1.7152	3.4564	5.2262	0.1234	0.2486	0.2381
115 Co	5.2283	13.8937	5.2323	0.3760	0.9993	0.2384
116 Co	5.2089	3.4185	5.0724	0.3747	0.2459	0.2311
117 Co	1.7132	6.9772	5.2178	0.1232	0.5018	0.2377
118 Co	1.7415	10.4241	5.2232	0.1253	0.7498	0.2379
119 Co	5.1566	6.9057	5.2772	0.3709	0.4967	0.2404
120 Co	5.1791	10.4511	5.2102	0.3725	0.7517	0.2374
121 Co	8.6751	13.8941	5.2334	0.6240	0.9993	0.2384
122 Co	8.6820	3.5306	5.4262	0.6245	0.2539	0.2472
123 Co	12.1546	0.0044	5.2232	0.8742	0.0003	0.2379
124 Co	12.1582	3.4772	5.2199	0.8745	0.2501	0.2378
125 Co	8.7412	6.9687	5.0607	0.6287	0.5012	0.2305
126 Co	8.7156	10.4570	5.1646	0.6269	0.7521	0.2353
127 Co	12.1627	6.9581	5.2236	0.8748	0.5005	0.2380
128 Co	12.1612	10.4302	5.2281	0.8747	0.7502	0.2382
129 O	7.7853	4.4635	6.8293	0.5600	0.3210	0.3111
130 O	4.8888	3.1990	9.1751	0.3516	0.2301	0.4180
131 O	4.2503	6.0912	6.7957	0.3057	0.4381	0.3096
132 O	6.2726	6.2359	8.8749	0.4512	0.4485	0.4043
133 O	6.7757	8.2984	7.0578	0.4873	0.5969	0.3215
134 O	8.8253	8.7594	8.8479	0.6348	0.6300	0.4031
135 O	5.6354	9.8619	9.0163	0.4053	0.7093	0.4107
136 Ce	4.8376	7.9775	8.3084	0.3479	0.5738	0.3785
137 Ce	5.5682	4.3217	7.8392	0.4005	0.3108	0.3571

138 Ce	8.3452	6.8200	8.0242	0.6002	0.4905	0.3655
139 Sm	7.4949	10.2158	8.0637	0.5391	0.7348	0.3673

/db/jmorales/CoNi-alloy/metal-SDC-interface/CoNi-Ce_3SmO_7/CoNi-Ce_3SmO_7-conf4a

a =			13.9033384264				
b =			13.9033384264				
c =			21.9516692133				
alpha =			90.0				
beta =			90.0				
gamma =			90.0				
	Atom	X	Y	Z	X	Y	Z
1	Ni	0.0000	0.0000	0.0000	0.0000	0.0000	0.0000
2	Ni	0.0000	3.4758	0.0000	0.0000	0.2500	0.0000
3	Ni	3.4758	0.0000	0.0000	0.2500	0.0000	0.0000
4	Ni	3.4758	3.4758	0.0000	0.2500	0.2500	0.0000
5	Ni	0.0000	6.9517	0.0000	0.0000	0.5000	0.0000
6	Ni	0.0000	10.4275	0.0000	0.0000	0.7500	0.0000
7	Ni	3.4758	6.9517	0.0000	0.2500	0.5000	0.0000
8	Ni	3.4758	10.4275	0.0000	0.2500	0.7500	0.0000
9	Ni	6.9517	0.0000	0.0000	0.5000	0.0000	0.0000
10	Ni	6.9517	3.4758	0.0000	0.5000	0.2500	0.0000
11	Ni	10.4275	0.0000	0.0000	0.7500	0.0000	0.0000
12	Ni	10.4275	3.4758	0.0000	0.7500	0.2500	0.0000
13	Ni	6.9517	6.9517	0.0000	0.5000	0.5000	0.0000
14	Ni	6.9517	10.4275	0.0000	0.5000	0.7500	0.0000
15	Ni	10.4275	6.9517	0.0000	0.7500	0.5000	0.0000
16	Ni	10.4275	10.4275	0.0000	0.7500	0.7500	0.0000
17	Ni	0.0000	1.7379	1.7379	0.0000	0.1250	0.0792
18	Ni	0.0000	5.2138	1.7379	0.0000	0.3750	0.0792
19	Ni	3.4758	1.7379	1.7379	0.2500	0.1250	0.0792
20	Ni	3.4758	5.2138	1.7379	0.2500	0.3750	0.0792
21	Ni	0.0000	8.6896	1.7379	0.0000	0.6250	0.0792
22	Ni	0.0000	12.1654	1.7379	0.0000	0.8750	0.0792
23	Ni	3.4758	8.6896	1.7379	0.2500	0.6250	0.0792
24	Ni	3.4758	12.1654	1.7379	0.2500	0.8750	0.0792
25	Ni	6.9517	1.7379	1.7379	0.5000	0.1250	0.0792
26	Ni	6.9517	5.2138	1.7379	0.5000	0.3750	0.0792
27	Ni	10.4275	1.7379	1.7379	0.7500	0.1250	0.0792
28	Ni	10.4275	5.2138	1.7379	0.7500	0.3750	0.0792
29	Ni	6.9517	8.6896	1.7379	0.5000	0.6250	0.0792
30	Ni	6.9517	12.1654	1.7379	0.5000	0.8750	0.0792
31	Ni	10.4275	8.6896	1.7379	0.7500	0.6250	0.0792
32	Ni	10.4275	12.1654	1.7379	0.7500	0.8750	0.0792
33	Ni	13.8997	13.8988	3.5050	0.9997	0.9997	0.1597
34	Ni	13.8743	3.4695	3.4776	0.9979	0.2495	0.1584
35	Ni	3.4795	13.8968	3.5005	0.2503	0.9995	0.1595
36	Ni	3.5092	3.4857	3.5107	0.2524	0.2507	0.1599
37	Ni	0.0122	6.9495	3.5171	0.0009	0.4998	0.1602
38	Ni	0.0010	10.4267	3.5045	0.0001	0.7499	0.1596
39	Ni	3.4845	6.9495	3.5139	0.2506	0.4998	0.1601

40	Ni	3.4730	10.4385	3.4915	0.2498	0.7508	0.1591
41	Ni	6.9488	13.9012	3.5054	0.4998	0.9998	0.1597
42	Ni	6.9284	3.4736	3.5200	0.4983	0.2498	0.1604
43	Ni	10.4277	13.9006	3.5043	0.7500	0.9998	0.1596
44	Ni	10.4320	3.4745	3.5026	0.7503	0.2499	0.1596
45	Ni	6.9423	6.9436	3.4900	0.4993	0.4994	0.1590
46	Ni	6.9496	10.4518	3.4787	0.4999	0.7517	0.1585
47	Ni	10.4351	6.9503	3.5009	0.7505	0.4999	0.1595
48	Ni	10.4351	10.4294	3.5019	0.7505	0.7501	0.1595
49	Ni	13.9012	1.7330	5.2120	0.9998	0.1246	0.2374
50	Ni	13.8743	5.1840	5.1909	0.9979	0.3729	0.2365
51	Ni	3.4424	1.7182	5.1915	0.2476	0.1236	0.2365
52	Ni	3.4061	5.1972	5.2119	0.2450	0.3738	0.2374
53	Ni	0.0022	8.6918	5.2144	0.0002	0.6252	0.2375
54	Ni	0.0050	12.1615	5.2095	0.0004	0.8747	0.2373
55	Ni	3.4643	8.7222	5.1680	0.2492	0.6273	0.2354
56	Ni	3.4688	12.1624	5.2057	0.2495	0.8748	0.2371
57	Ni	6.9819	1.7153	5.2093	0.5022	0.1234	0.2373
58	Ni	7.0293	5.2160	5.1903	0.5056	0.3752	0.2364
59	Ni	10.4211	1.7304	5.2072	0.7495	0.1245	0.2372
60	Ni	10.4213	5.2163	5.2070	0.7496	0.3752	0.2372
61	Ni	6.9946	8.7272	5.0998	0.5031	0.6277	0.2323
62	Ni	6.9562	12.1618	5.2114	0.5003	0.8747	0.2374
63	Ni	10.4212	8.6950	5.2135	0.7495	0.6254	0.2375
64	Ni	10.4261	12.1607	5.2111	0.7499	0.8747	0.2374
65	Co	1.7379	1.7379	0.0000	0.1250	0.1250	0.0000
66	Co	1.7379	5.2138	0.0000	0.1250	0.3750	0.0000
67	Co	5.2138	1.7379	0.0000	0.3750	0.1250	0.0000
68	Co	5.2138	5.2138	0.0000	0.3750	0.3750	0.0000
69	Co	1.7379	8.6896	0.0000	0.1250	0.6250	0.0000
70	Co	1.7379	12.1654	0.0000	0.1250	0.8750	0.0000
71	Co	5.2138	8.6896	0.0000	0.3750	0.6250	0.0000
72	Co	5.2138	12.1654	0.0000	0.3750	0.8750	0.0000
73	Co	8.6896	1.7379	0.0000	0.6250	0.1250	0.0000
74	Co	8.6896	5.2138	0.0000	0.6250	0.3750	0.0000
75	Co	12.1654	1.7379	0.0000	0.8750	0.1250	0.0000
76	Co	12.1654	5.2138	0.0000	0.8750	0.3750	0.0000
77	Co	8.6896	8.6896	0.0000	0.6250	0.6250	0.0000
78	Co	8.6896	12.1654	0.0000	0.6250	0.8750	0.0000
79	Co	12.1654	8.6896	0.0000	0.8750	0.6250	0.0000
80	Co	12.1654	12.1654	0.0000	0.8750	0.8750	0.0000
81	Co	1.7379	0.0000	1.7379	0.1250	0.0000	0.0792
82	Co	1.7379	3.4758	1.7379	0.1250	0.2500	0.0792
83	Co	5.2138	0.0000	1.7379	0.3750	0.0000	0.0792
84	Co	5.2138	3.4758	1.7379	0.3750	0.2500	0.0792
85	Co	1.7379	6.9517	1.7379	0.1250	0.5000	0.0792
86	Co	1.7379	10.4275	1.7379	0.1250	0.7500	0.0792
87	Co	5.2138	6.9517	1.7379	0.3750	0.5000	0.0792
88	Co	5.2138	10.4275	1.7379	0.3750	0.7500	0.0792

89	Co	8.6896	0.0000	1.7379	0.6250	0.0000	0.0792
90	Co	8.6896	3.4758	1.7379	0.6250	0.2500	0.0792
91	Co	12.1654	0.0000	1.7379	0.8750	0.0000	0.0792
92	Co	12.1654	3.4758	1.7379	0.8750	0.2500	0.0792
93	Co	8.6896	6.9517	1.7379	0.6250	0.5000	0.0792
94	Co	8.6896	10.4275	1.7379	0.6250	0.7500	0.0792
95	Co	12.1654	6.9517	1.7379	0.8750	0.5000	0.0792
96	Co	12.1654	10.4275	1.7379	0.8750	0.7500	0.0792
97	Co	1.7366	1.7199	3.5160	0.1249	0.1237	0.1602
98	Co	1.7266	5.2119	3.5075	0.1242	0.3749	0.1598
99	Co	5.2156	1.7464	3.5706	0.3751	0.1256	0.1627
100	Co	5.2040	5.2110	3.6154	0.3743	0.3748	0.1647
101	Co	1.7279	8.6739	3.5438	0.1243	0.6239	0.1614
102	Co	1.7345	12.1644	3.5507	0.1248	0.8749	0.1617
103	Co	5.2056	8.6902	3.5294	0.3744	0.6250	0.1608
104	Co	5.2135	12.1655	3.5587	0.3750	0.8750	0.1621
105	Co	8.6893	1.7372	3.5428	0.6250	0.1249	0.1614
106	Co	8.6951	5.2132	3.5320	0.6254	0.3750	0.1609
107	Co	12.1622	1.7287	3.5541	0.8748	0.1243	0.1619
108	Co	12.1631	5.2158	3.5556	0.8748	0.3751	0.1620
109	Co	8.7172	8.6924	3.5079	0.6270	0.6252	0.1598
110	Co	8.6932	12.1645	3.5472	0.6253	0.8749	0.1616
111	Co	12.1687	8.6885	3.5580	0.8752	0.6249	0.1621
112	Co	12.1659	12.1652	3.5574	0.8750	0.8750	0.1621
113	Co	1.7355	0.0052	5.2253	0.1248	0.0004	0.2380
114	Co	1.7020	3.4150	5.1203	0.1224	0.2456	0.2333
115	Co	5.2079	13.8958	5.2202	0.3746	0.9995	0.2378
116	Co	5.2204	3.4622	5.3651	0.3755	0.2490	0.2444
117	Co	1.7129	6.9569	5.3048	0.1232	0.5004	0.2417
118	Co	1.7444	10.4123	5.2234	0.1255	0.7489	0.2379
119	Co	5.2656	7.0107	5.2784	0.3787	0.5042	0.2405
120	Co	5.2150	10.4157	5.2189	0.3751	0.7491	0.2377
121	Co	8.6913	13.8945	5.2227	0.6251	0.9994	0.2379
122	Co	8.7009	3.4727	5.2112	0.6258	0.2498	0.2374
123	Co	12.1650	13.8987	5.2301	0.8750	0.9997	0.2383
124	Co	12.1582	3.4608	5.2258	0.8745	0.2489	0.2381
125	Co	8.6986	6.9735	5.2058	0.6256	0.5016	0.2371
126	Co	8.6858	10.4218	5.2153	0.6247	0.7496	0.2376
127	Co	12.1646	6.9588	5.2230	0.8749	0.5005	0.2379
128	Co	12.1701	10.4248	5.2242	0.8753	0.7498	0.2380
129	O	5.0336	3.5686	7.1672	0.3620	0.2567	0.3265
130	O	6.7277	3.6402	9.9818	0.4839	0.2618	0.4547
131	O	2.3381	5.8556	6.7463	0.1682	0.4212	0.3073
132	O	3.9620	5.4708	9.1084	0.2850	0.3935	0.4149
133	O	5.4823	6.8565	7.0926	0.3943	0.4932	0.3231
134	O	7.5688	6.7694	8.9779	0.5444	0.4869	0.4090
135	O	5.0118	8.9837	8.8317	0.3605	0.6462	0.4023
136	Ce	3.5323	7.5664	8.1721	0.2541	0.5442	0.3723
137	Ce	2.9778	3.9259	7.9093	0.2142	0.2824	0.3603

138 Ce	6.3525	4.9854	8.7220	0.4569	0.3586	0.3973
139 Sm	6.8848	8.4743	7.9682	0.4952	0.6095	0.3630

/db/jmorales/CoNi-alloy/metal-SDC-interface/Ni-Ce_3SmO_7/Ni-Ce_3SmO_7-conf4c

a = 13.9019837372
b = 13.9019837372
c = 21.9509918687
alpha = 90.0
beta = 90.0
gamma = 90.0

	Atom	X	Y	Z	X	Y	Z
1	Ni	0.0000	0.0000	0.0000	0.0000	0.0000	0.0000
2	Ni	1.7377	1.7377	0.0000	0.1250	0.1250	0.0000
3	Ni	0.0000	3.4755	0.0000	0.0000	0.2500	0.0000
4	Ni	1.7377	5.2132	0.0000	0.1250	0.3750	0.0000
5	Ni	3.4755	0.0000	0.0000	0.2500	0.0000	0.0000
6	Ni	5.2132	1.7377	0.0000	0.3750	0.1250	0.0000
7	Ni	3.4755	3.4755	0.0000	0.2500	0.2500	0.0000
8	Ni	5.2132	5.2132	0.0000	0.3750	0.3750	0.0000
9	Ni	0.0000	6.9510	0.0000	0.0000	0.5000	0.0000
10	Ni	1.7377	8.6887	0.0000	0.1250	0.6250	0.0000
11	Ni	0.0000	10.4265	0.0000	0.0000	0.7500	0.0000
12	Ni	1.7377	12.1642	0.0000	0.1250	0.8750	0.0000
13	Ni	3.4755	6.9510	0.0000	0.2500	0.5000	0.0000
14	Ni	5.2132	8.6887	0.0000	0.3750	0.6250	0.0000
15	Ni	3.4755	10.4265	0.0000	0.2500	0.7500	0.0000
16	Ni	5.2132	12.1642	0.0000	0.3750	0.8750	0.0000
17	Ni	6.9510	0.0000	0.0000	0.5000	0.0000	0.0000
18	Ni	8.6887	1.7377	0.0000	0.6250	0.1250	0.0000
19	Ni	6.9510	3.4755	0.0000	0.5000	0.2500	0.0000
20	Ni	8.6887	5.2132	0.0000	0.6250	0.3750	0.0000
21	Ni	10.4265	0.0000	0.0000	0.7500	0.0000	0.0000
22	Ni	12.1642	1.7377	0.0000	0.8750	0.1250	0.0000
23	Ni	10.4265	3.4755	0.0000	0.7500	0.2500	0.0000
24	Ni	12.1642	5.2132	0.0000	0.8750	0.3750	0.0000
25	Ni	6.9510	6.9510	0.0000	0.5000	0.5000	0.0000
26	Ni	8.6887	8.6887	0.0000	0.6250	0.6250	0.0000
27	Ni	6.9510	10.4265	0.0000	0.5000	0.7500	0.0000
28	Ni	8.6887	12.1642	0.0000	0.6250	0.8750	0.0000
29	Ni	10.4265	6.9510	0.0000	0.7500	0.5000	0.0000
30	Ni	12.1642	8.6887	0.0000	0.8750	0.6250	0.0000
31	Ni	10.4265	10.4265	0.0000	0.7500	0.7500	0.0000
32	Ni	12.1642	12.1642	0.0000	0.8750	0.8750	0.0000
33	Ni	0.0000	1.7377	1.7377	0.0000	0.1250	0.0792
34	Ni	1.7377	0.0000	1.7377	0.1250	0.0000	0.0792
35	Ni	0.0000	5.2132	1.7377	0.0000	0.3750	0.0792
36	Ni	1.7377	3.4755	1.7377	0.1250	0.2500	0.0792
37	Ni	3.4755	1.7377	1.7377	0.2500	0.1250	0.0792
38	Ni	5.2132	0.0000	1.7377	0.3750	0.0000	0.0792
39	Ni	3.4755	5.2132	1.7377	0.2500	0.3750	0.0792
40	Ni	5.2132	3.4755	1.7377	0.3750	0.2500	0.0792

41	Ni	0.0000	8.6887	1.7377	0.0000	0.6250	0.0792
42	Ni	1.7377	6.9510	1.7377	0.1250	0.5000	0.0792
43	Ni	0.0000	12.1642	1.7377	0.0000	0.8750	0.0792
44	Ni	1.7377	10.4265	1.7377	0.1250	0.7500	0.0792
45	Ni	3.4755	8.6887	1.7377	0.2500	0.6250	0.0792
46	Ni	5.2132	6.9510	1.7377	0.3750	0.5000	0.0792
47	Ni	3.4755	12.1642	1.7377	0.2500	0.8750	0.0792
48	Ni	5.2132	10.4265	1.7377	0.3750	0.7500	0.0792
49	Ni	6.9510	1.7377	1.7377	0.5000	0.1250	0.0792
50	Ni	8.6887	0.0000	1.7377	0.6250	0.0000	0.0792
51	Ni	6.9510	5.2132	1.7377	0.5000	0.3750	0.0792
52	Ni	8.6887	3.4755	1.7377	0.6250	0.2500	0.0792
53	Ni	10.4265	1.7377	1.7377	0.7500	0.1250	0.0792
54	Ni	12.1642	0.0000	1.7377	0.8750	0.0000	0.0792
55	Ni	10.4265	5.2132	1.7377	0.7500	0.3750	0.0792
56	Ni	12.1642	3.4755	1.7377	0.8750	0.2500	0.0792
57	Ni	6.9510	8.6887	1.7377	0.5000	0.6250	0.0792
58	Ni	8.6887	6.9510	1.7377	0.6250	0.5000	0.0792
59	Ni	6.9510	12.1642	1.7377	0.5000	0.8750	0.0792
60	Ni	8.6887	10.4265	1.7377	0.6250	0.7500	0.0792
61	Ni	10.4265	8.6887	1.7377	0.7500	0.6250	0.0792
62	Ni	12.1642	6.9510	1.7377	0.8750	0.5000	0.0792
63	Ni	10.4265	12.1642	1.7377	0.7500	0.8750	0.0792
64	Ni	12.1642	10.4265	1.7377	0.8750	0.7500	0.0792
65	Ni	13.8998	13.8981	3.5109	0.9998	0.9997	0.1599
66	Ni	1.7284	1.7386	3.5006	0.1243	0.1251	0.1595
67	Ni	13.8972	3.4779	3.5088	0.9997	0.2502	0.1598
68	Ni	1.7487	5.2133	3.5287	0.1258	0.3750	0.1608
69	Ni	3.4717	13.8892	3.5002	0.2497	0.9991	0.1595
70	Ni	5.2229	1.7337	3.5038	0.3757	0.1247	0.1596
71	Ni	3.4786	3.4926	3.5193	0.2502	0.2512	0.1603
72	Ni	5.1906	5.1963	3.4987	0.3734	0.3738	0.1594
73	Ni	13.9000	6.9511	3.5101	0.9999	0.5000	0.1599
74	Ni	1.7356	8.6873	3.5077	0.1248	0.6249	0.1598
75	Ni	13.8987	10.4283	3.5113	0.9998	0.7501	0.1600
76	Ni	1.7315	12.1630	3.5122	0.1246	0.8749	0.1600
77	Ni	3.4784	6.9307	3.5284	0.2502	0.4985	0.1607
78	Ni	5.2019	8.7084	3.4806	0.3742	0.6264	0.1586
79	Ni	3.4694	10.4337	3.5073	0.2496	0.7505	0.1598
80	Ni	5.2202	12.1590	3.5098	0.3755	0.8746	0.1599
81	Ni	6.9475	0.0129	3.5316	0.4998	0.0009	0.1609
82	Ni	8.6700	1.7297	3.5194	0.6237	0.1244	0.1603
83	Ni	6.9608	3.4916	3.5039	0.5007	0.2512	0.1596
84	Ni	8.7090	5.2124	3.4741	0.6265	0.3749	0.1583
85	Ni	10.4237	-0.0001	3.5113	0.7498	-0.0000	0.1600
86	Ni	12.1657	1.7319	3.5128	0.8751	0.1246	0.1600
87	Ni	10.4473	3.4694	3.4963	0.7515	0.2496	0.1593
88	Ni	12.1637	5.2189	3.5104	0.8750	0.3754	0.1599
89	Ni	6.9582	6.9587	3.4246	0.5005	0.5006	0.1560

90	Ni	8.6970	8.7023	3.4942	0.6256	0.6260	0.1592
91	Ni	6.9541	10.4102	3.5322	0.5002	0.7488	0.1609
92	Ni	8.6820	12.1624	3.5141	0.6245	0.8749	0.1601
93	Ni	10.4257	6.9564	3.5160	0.7499	0.5004	0.1602
94	Ni	12.1634	8.6914	3.5099	0.8749	0.6252	0.1599
95	Ni	10.4284	10.4319	3.5092	0.7501	0.7504	0.1599
96	Ni	12.1624	12.1641	3.5121	0.8749	0.8750	0.1600
97	Ni	13.8985	1.7313	5.2283	0.9998	0.1245	0.2382
98	Ni	1.7422	0.0058	5.2295	0.1253	0.0004	0.2382
99	Ni	13.8936	5.2146	5.2209	0.9994	0.3751	0.2378
100	Ni	1.7243	3.4494	5.2194	0.1240	0.2481	0.2378
101	Ni	3.4533	1.7319	5.1796	0.2484	0.1246	0.2360
102	Ni	5.1885	13.8844	5.2151	0.3732	0.9987	0.2376
103	Ni	3.4540	5.2185	5.4046	0.2485	0.3754	0.2462
104	Ni	5.2320	3.4824	5.2263	0.3764	0.2505	0.2381
105	Ni	13.8924	8.6966	5.2266	0.9993	0.6256	0.2381
106	Ni	1.7208	6.9773	5.2266	0.1238	0.5019	0.2381
107	Ni	0.0002	12.1644	5.2300	0.0000	0.8750	0.2383
108	Ni	1.7388	10.4208	5.2275	0.1251	0.7496	0.2381
109	Ni	3.4776	8.6920	5.2119	0.2502	0.6252	0.2374
110	Ni	5.2083	6.9678	5.1512	0.3746	0.5012	0.2347
111	Ni	3.4687	12.1597	5.2252	0.2495	0.8747	0.2380
112	Ni	5.2039	10.4224	5.2313	0.3743	0.7497	0.2383
113	Ni	6.9894	1.7204	5.3735	0.5028	0.1237	0.2448
114	Ni	8.6942	13.8956	5.2312	0.6254	0.9995	0.2383
115	Ni	6.9826	5.2408	5.2050	0.5023	0.3770	0.2371
116	Ni	8.7200	3.4938	5.1578	0.6272	0.2513	0.2350
117	Ni	10.4170	1.7446	5.2275	0.7493	0.1255	0.2381
118	Ni	12.1594	13.9003	5.2281	0.8746	0.9999	0.2382
119	Ni	10.4306	5.2057	5.2295	0.7503	0.3745	0.2382
120	Ni	12.1657	3.4721	5.2294	0.8751	0.2498	0.2382
121	Ni	6.9527	8.6935	5.2417	0.5001	0.6253	0.2388
122	Ni	8.7178	6.9546	5.1795	0.6271	0.5003	0.2360
123	Ni	6.9527	12.1581	5.2334	0.5001	0.8746	0.2384
124	Ni	8.6956	10.4154	5.2416	0.6255	0.7492	0.2388
125	Ni	10.4204	8.6967	5.2319	0.7496	0.6256	0.2383
126	Ni	12.1582	6.9585	5.2342	0.8746	0.5005	0.2385
127	Ni	10.4237	12.1591	5.2291	0.7498	0.8746	0.2382
128	Ni	12.1597	10.4260	5.2317	0.8747	0.7500	0.2383
129	O	6.0789	2.6654	6.7446	0.4373	0.1917	0.3073
130	O	8.1508	2.6651	9.4544	0.5863	0.1917	0.4307
131	O	3.2732	5.1693	7.2780	0.2354	0.3718	0.3316
132	O	5.6699	4.7374	8.6849	0.4078	0.3408	0.3956
133	O	6.8572	6.9440	6.3321	0.4933	0.4995	0.2885
134	O	8.8143	5.8824	8.4245	0.6340	0.4231	0.3838
135	O	6.4855	8.0762	8.9533	0.4665	0.5809	0.4079
136	Ce	4.9639	6.8171	8.0381	0.3571	0.4904	0.3662
137	Ce	4.1757	3.2695	7.9153	0.3004	0.2352	0.3606
138	Ce	7.8452	3.9580	8.1241	0.5643	0.2847	0.3701

139 Sm	8.3346	7.8663	7.9843	0.5995	0.5658	0.3637

/db/jmorales/CoNi-alloy/metal-SDC-interface/CoNi-Ce_3SmO_7/CoNi-Ce_3SmO_7-conf-CoNi-b_

a =				13.9033384264			
b =				13.9033384264			
c =				21.9516692133			
alpha =				90.0			
beta =				90.0			
gamma =				90.0			
	Atom	X	Y	Z	X	Y	Z
1	Ni	0.0000	0.0000	0.0000	0.0000	0.0000	0.0000
2	Ni	0.0000	3.4758	0.0000	0.0000	0.2500	0.0000
3	Ni	3.4758	0.0000	0.0000	0.2500	0.0000	0.0000
4	Ni	3.4758	3.4758	0.0000	0.2500	0.2500	0.0000
5	Ni	0.0000	6.9517	0.0000	0.0000	0.5000	0.0000
6	Ni	0.0000	10.4275	0.0000	0.0000	0.7500	0.0000
7	Ni	3.4758	6.9517	0.0000	0.2500	0.5000	0.0000
8	Ni	3.4758	10.4275	0.0000	0.2500	0.7500	0.0000
9	Ni	6.9517	0.0000	0.0000	0.5000	0.0000	0.0000
10	Ni	6.9517	3.4758	0.0000	0.5000	0.2500	0.0000
11	Ni	10.4275	0.0000	0.0000	0.7500	0.0000	0.0000
12	Ni	10.4275	3.4758	0.0000	0.7500	0.2500	0.0000
13	Ni	6.9517	6.9517	0.0000	0.5000	0.5000	0.0000
14	Ni	6.9517	10.4275	0.0000	0.5000	0.7500	0.0000
15	Ni	10.4275	6.9517	0.0000	0.7500	0.5000	0.0000
16	Ni	10.4275	10.4275	0.0000	0.7500	0.7500	0.0000
17	Ni	0.0000	1.7379	1.7379	0.0000	0.1250	0.0792
18	Ni	0.0000	5.2138	1.7379	0.0000	0.3750	0.0792
19	Ni	3.4758	1.7379	1.7379	0.2500	0.1250	0.0792
20	Ni	3.4758	5.2138	1.7379	0.2500	0.3750	0.0792
21	Ni	0.0000	8.6896	1.7379	0.0000	0.6250	0.0792
22	Ni	0.0000	12.1654	1.7379	0.0000	0.8750	0.0792
23	Ni	3.4758	8.6896	1.7379	0.2500	0.6250	0.0792
24	Ni	3.4758	12.1654	1.7379	0.2500	0.8750	0.0792
25	Ni	6.9517	1.7379	1.7379	0.5000	0.1250	0.0792
26	Ni	6.9517	5.2138	1.7379	0.5000	0.3750	0.0792
27	Ni	10.4275	1.7379	1.7379	0.7500	0.1250	0.0792
28	Ni	10.4275	5.2138	1.7379	0.7500	0.3750	0.0792
29	Ni	6.9517	8.6896	1.7379	0.5000	0.6250	0.0792
30	Ni	6.9517	12.1654	1.7379	0.5000	0.8750	0.0792
31	Ni	10.4275	8.6896	1.7379	0.7500	0.6250	0.0792
32	Ni	10.4275	12.1654	1.7379	0.7500	0.8750	0.0792
33	Ni	13.8983	-0.0001	3.4976	0.9996	-0.0000	0.1593
34	Ni	0.0036	3.4697	3.5056	0.0003	0.2496	0.1597
35	Ni	3.4824	0.0293	3.5322	0.2505	0.0021	0.1609
36	Ni	3.4934	3.4882	3.5320	0.2513	0.2509	0.1609
37	Ni	0.0131	6.9457	3.5225	0.0009	0.4996	0.1605
38	Ni	-0.0018	10.4277	3.5023	-0.0001	0.7500	0.1595
39	Ni	3.4710	6.9720	3.4708	0.2496	0.5015	0.1581

40 Ni	3.4595	10.4550	3.4633	0.2488	0.7520	0.1578
41 Ni	6.9507	13.8938	3.4980	0.4999	0.9993	0.1594
42 Ni	6.9634	3.4714	3.4838	0.5008	0.2497	0.1587
43 Ni	10.4263	13.8934	3.5014	0.7499	0.9993	0.1595
44 Ni	10.4275	3.4744	3.4959	0.7500	0.2499	0.1593
45 Ni	6.9632	6.9341	3.5000	0.5008	0.4987	0.1594
46 Ni	6.9524	10.4211	3.5047	0.5001	0.7495	0.1597
47 Ni	10.4408	6.9553	3.4895	0.7510	0.5003	0.1590
48 Ni	10.4362	10.4238	3.4959	0.7506	0.7497	0.1593
49 Ni	13.8551	1.7155	5.1897	0.9965	0.1234	0.2364
50 Ni	13.8845	5.1888	5.2022	0.9986	0.3732	0.2370
51 Ni	3.5100	1.7064	5.3681	0.2525	0.1227	0.2445
52 Ni	3.4768	5.2734	5.1645	0.2501	0.3793	0.2353
53 Ni	0.0081	8.6933	5.2172	0.0006	0.6253	0.2377
54 Ni	13.8974	12.1591	5.2062	0.9996	0.8745	0.2372
55 Ni	3.4525	8.7454	5.0941	0.2483	0.6290	0.2321
56 Ni	3.4682	12.1742	5.2057	0.2494	0.8756	0.2371
57 Ni	6.9871	1.7062	5.1839	0.5025	0.1227	0.2362
58 Ni	7.0060	5.2050	5.2759	0.5039	0.3744	0.2403
59 Ni	10.4292	1.7178	5.2025	0.7501	0.1236	0.2370
60 Ni	10.4281	5.2103	5.2128	0.7500	0.3748	0.2375
61 Ni	6.9969	8.7147	5.2301	0.5033	0.6268	0.2383
62 Ni	6.9621	12.1391	5.2260	0.5007	0.8731	0.2381
63 Ni	10.4179	8.6945	5.2112	0.7493	0.6254	0.2374
64 Ni	10.4268	12.1592	5.2134	0.7499	0.8745	0.2375
65 Co	1.7379	1.7379	0.0000	0.1250	0.1250	0.0000
66 Co	1.7379	5.2138	0.0000	0.1250	0.3750	0.0000
67 Co	5.2138	1.7379	0.0000	0.3750	0.1250	0.0000
68 Co	5.2138	5.2138	0.0000	0.3750	0.3750	0.0000
69 Co	1.7379	8.6896	0.0000	0.1250	0.6250	0.0000
70 Co	1.7379	12.1654	0.0000	0.1250	0.8750	0.0000
71 Co	5.2138	8.6896	0.0000	0.3750	0.6250	0.0000
72 Co	5.2138	12.1654	0.0000	0.3750	0.8750	0.0000
73 Co	8.6896	1.7379	0.0000	0.6250	0.1250	0.0000
74 Co	8.6896	5.2138	0.0000	0.6250	0.3750	0.0000
75 Co	12.1654	1.7379	0.0000	0.8750	0.1250	0.0000
76 Co	12.1654	5.2138	0.0000	0.8750	0.3750	0.0000
77 Co	8.6896	8.6896	0.0000	0.6250	0.6250	0.0000
78 Co	8.6896	12.1654	0.0000	0.6250	0.8750	0.0000
79 Co	12.1654	8.6896	0.0000	0.8750	0.6250	0.0000
80 Co	12.1654	12.1654	0.0000	0.8750	0.8750	0.0000
81 Co	1.7379	0.0000	1.7379	0.1250	0.0000	0.0792
82 Co	1.7379	3.4758	1.7379	0.1250	0.2500	0.0792
83 Co	5.2138	0.0000	1.7379	0.3750	0.0000	0.0792
84 Co	5.2138	3.4758	1.7379	0.3750	0.2500	0.0792
85 Co	1.7379	6.9517	1.7379	0.1250	0.5000	0.0792
86 Co	1.7379	10.4275	1.7379	0.1250	0.7500	0.0792
87 Co	5.2138	6.9517	1.7379	0.3750	0.5000	0.0792
88 Co	5.2138	10.4275	1.7379	0.3750	0.7500	0.0792

89	Co	8.6896	0.0000	1.7379	0.6250	0.0000	0.0792
90	Co	8.6896	3.4758	1.7379	0.6250	0.2500	0.0792
91	Co	12.1654	0.0000	1.7379	0.8750	0.0000	0.0792
92	Co	12.1654	3.4758	1.7379	0.8750	0.2500	0.0792
93	Co	8.6896	6.9517	1.7379	0.6250	0.5000	0.0792
94	Co	8.6896	10.4275	1.7379	0.6250	0.7500	0.0792
95	Co	12.1654	6.9517	1.7379	0.8750	0.5000	0.0792
96	Co	12.1654	10.4275	1.7379	0.8750	0.7500	0.0792
97	Co	1.7540	1.7417	3.5607	0.1262	0.1253	0.1622
98	Co	1.7197	5.1993	3.5464	0.1237	0.3740	0.1616
99	Co	5.1939	1.7443	3.5829	0.3736	0.1255	0.1632
100	Co	5.2220	5.2329	3.5491	0.3756	0.3764	0.1617
101	Co	1.7066	8.6708	3.5293	0.1228	0.6237	0.1608
102	Co	1.7344	12.1779	3.5498	0.1247	0.8759	0.1617
103	Co	5.2271	8.7154	3.5044	0.3760	0.6269	0.1596
104	Co	5.2161	12.1780	3.5437	0.3752	0.8759	0.1614
105	Co	8.6985	1.7220	3.5384	0.6256	0.1239	0.1612
106	Co	8.6862	5.2115	3.5543	0.6248	0.3748	0.1619
107	Co	12.1567	1.7293	3.5348	0.8744	0.1244	0.1610
108	Co	12.1643	5.2104	3.5512	0.8749	0.3748	0.1618
109	Co	8.6895	8.6961	3.5398	0.6250	0.6255	0.1613
110	Co	8.6914	12.1656	3.5380	0.6251	0.8750	0.1612
111	Co	12.1697	8.6880	3.5631	0.8753	0.6249	0.1623
112	Co	12.1644	12.1619	3.5490	0.8749	0.8747	0.1617
113	Co	1.7090	13.8806	5.2134	0.1229	0.9984	0.2375
114	Co	1.7132	3.4203	5.3072	0.1232	0.2460	0.2418
115	Co	5.2447	13.8842	5.2310	0.3772	0.9986	0.2383
116	Co	5.2891	3.5071	5.2312	0.3804	0.2522	0.2383
117	Co	1.7403	6.9534	5.3386	0.1252	0.5001	0.2432
118	Co	1.7427	10.4254	5.2158	0.1253	0.7499	0.2376
119	Co	5.2232	7.0408	5.1777	0.3757	0.5064	0.2359
120	Co	5.1736	10.4670	5.1812	0.3721	0.7528	0.2360
121	Co	8.6861	13.8767	5.2330	0.6247	0.9981	0.2384
122	Co	8.7315	3.4463	5.2037	0.6280	0.2479	0.2371
123	Co	12.1607	13.8902	5.2238	0.8747	0.9991	0.2380
124	Co	12.1586	3.4713	5.2152	0.8745	0.2497	0.2376
125	Co	8.7399	6.9352	5.1822	0.6286	0.4988	0.2361
126	Co	8.7079	10.4481	5.2074	0.6263	0.7515	0.2372
127	Co	12.1681	6.9500	5.2182	0.8752	0.4999	0.2377
128	Co	12.1646	10.4269	5.2239	0.8749	0.7500	0.2380
129	O	2.6317	2.7237	6.7377	0.1893	0.1959	0.3069
130	O	5.9874	4.3488	6.8090	0.4306	0.3128	0.3102
131	O	2.7543	6.1466	6.7997	0.1981	0.4421	0.3098
132	O	4.8972	6.2385	8.8501	0.3522	0.4487	0.4032
133	O	6.0026	7.9275	6.7872	0.4317	0.5702	0.3092
134	O	7.8936	8.1522	8.8907	0.5677	0.5863	0.4050
135	O	5.0358	9.8394	8.6848	0.3622	0.7077	0.3956
136	Ce	3.8870	8.1478	7.9414	0.2796	0.5860	0.3618
137	Ce	3.8866	4.3992	7.8195	0.2795	0.3164	0.3562

138 Ce	7.0961	6.3053	8.1019	0.5104	0.4535	0.3691
139 Sm	7.0067	9.8218	7.9403	0.5040	0.7064	0.3617

/db/jmorales/CoNi-alloy/metal-SDC-interface/CoNi-Ce_3SmO_7/CoNi-Ce_3SmO_7-conf2a

a = 13.9033384264
b = 13.9033384264
c = 21.9516692133
alpha = 90.0
beta = 90.0
gamma = 90.0

	Atom	X	Y	Z	X	Y	Z
1	Ni	0.0000	0.0000	0.0000	0.0000	0.0000	0.0000
2	Ni	0.0000	3.4758	0.0000	0.0000	0.2500	0.0000
3	Ni	3.4758	0.0000	0.0000	0.2500	0.0000	0.0000
4	Ni	3.4758	3.4758	0.0000	0.2500	0.2500	0.0000
5	Ni	0.0000	6.9517	0.0000	0.0000	0.5000	0.0000
6	Ni	0.0000	10.4275	0.0000	0.0000	0.7500	0.0000
7	Ni	3.4758	6.9517	0.0000	0.2500	0.5000	0.0000
8	Ni	3.4758	10.4275	0.0000	0.2500	0.7500	0.0000
9	Ni	6.9517	0.0000	0.0000	0.5000	0.0000	0.0000
10	Ni	6.9517	3.4758	0.0000	0.5000	0.2500	0.0000
11	Ni	10.4275	0.0000	0.0000	0.7500	0.0000	0.0000
12	Ni	10.4275	3.4758	0.0000	0.7500	0.2500	0.0000
13	Ni	6.9517	6.9517	0.0000	0.5000	0.5000	0.0000
14	Ni	6.9517	10.4275	0.0000	0.5000	0.7500	0.0000
15	Ni	10.4275	6.9517	0.0000	0.7500	0.5000	0.0000
16	Ni	10.4275	10.4275	0.0000	0.7500	0.7500	0.0000
17	Ni	0.0000	1.7379	1.7379	0.0000	0.1250	0.0792
18	Ni	0.0000	5.2138	1.7379	0.0000	0.3750	0.0792
19	Ni	3.4758	1.7379	1.7379	0.2500	0.1250	0.0792
20	Ni	3.4758	5.2138	1.7379	0.2500	0.3750	0.0792
21	Ni	0.0000	8.6896	1.7379	0.0000	0.6250	0.0792
22	Ni	0.0000	12.1654	1.7379	0.0000	0.8750	0.0792
23	Ni	3.4758	8.6896	1.7379	0.2500	0.6250	0.0792
24	Ni	3.4758	12.1654	1.7379	0.2500	0.8750	0.0792
25	Ni	6.9517	1.7379	1.7379	0.5000	0.1250	0.0792
26	Ni	6.9517	5.2138	1.7379	0.5000	0.3750	0.0792
27	Ni	10.4275	1.7379	1.7379	0.7500	0.1250	0.0792
28	Ni	10.4275	5.2138	1.7379	0.7500	0.3750	0.0792
29	Ni	6.9517	8.6896	1.7379	0.5000	0.6250	0.0792
30	Ni	6.9517	12.1654	1.7379	0.5000	0.8750	0.0792
31	Ni	10.4275	8.6896	1.7379	0.7500	0.6250	0.0792
32	Ni	10.4275	12.1654	1.7379	0.7500	0.8750	0.0792
33	Ni	13.8980	13.8980	3.5032	0.9996	0.9996	0.1596
34	Ni	13.8721	3.4707	3.4712	0.9978	0.2496	0.1581
35	Ni	3.4795	13.8970	3.4994	0.2503	0.9995	0.1594
36	Ni	3.5085	3.4891	3.5173	0.2523	0.2510	0.1602
37	Ni	0.0070	6.9515	3.5141	0.0005	0.5000	0.1601
38	Ni	0.0016	10.4239	3.5044	0.0001	0.7497	0.1596
39	Ni	3.4901	6.9534	3.5144	0.2510	0.5001	0.1601

40	Ni	3.4756	10.4359	3.4884	0.2500	0.7506	0.1589
41	Ni	6.9483	-0.0008	3.5019	0.4998	-0.0001	0.1595
42	Ni	6.9265	3.4610	3.5093	0.4982	0.2489	0.1599
43	Ni	10.4288	13.9014	3.5016	0.7501	0.9999	0.1595
44	Ni	10.4347	3.4695	3.4995	0.7505	0.2495	0.1594
45	Ni	6.9591	6.9473	3.4901	0.5005	0.4997	0.1590
46	Ni	6.9573	10.4434	3.4898	0.5004	0.7511	0.1590
47	Ni	10.4227	6.9586	3.5063	0.7497	0.5005	0.1597
48	Ni	10.4233	10.4227	3.5070	0.7497	0.7497	0.1598
49	Ni	0.0008	1.7327	5.2104	0.0001	0.1246	0.2374
50	Ni	13.8767	5.1823	5.1831	0.9981	0.3727	0.2361
51	Ni	3.4273	1.7193	5.1992	0.2465	0.1237	0.2368
52	Ni	3.4179	5.2123	5.2156	0.2458	0.3749	0.2376
53	Ni	0.0008	8.6927	5.2148	0.0001	0.6252	0.2376
54	Ni	0.0057	12.1574	5.2069	0.0004	0.8744	0.2372
55	Ni	3.4750	8.7181	5.1726	0.2499	0.6270	0.2356
56	Ni	3.4669	12.1667	5.2052	0.2494	0.8751	0.2371
57	Ni	6.9885	1.7127	5.2067	0.5026	0.1232	0.2372
58	Ni	7.0158	5.1923	5.1533	0.5046	0.3735	0.2348
59	Ni	10.4214	1.7321	5.2071	0.7496	0.1246	0.2372
60	Ni	10.4177	5.2133	5.2043	0.7493	0.3750	0.2371
61	Ni	6.9635	8.7385	5.1466	0.5009	0.6285	0.2345
62	Ni	6.9520	12.1616	5.2080	0.5000	0.8747	0.2372
63	Ni	10.4339	8.6950	5.2613	0.7505	0.6254	0.2397
64	Ni	10.4275	12.1614	5.2056	0.7500	0.8747	0.2371
65	Co	1.7379	1.7379	0.0000	0.1250	0.1250	0.0000
66	Co	1.7379	5.2138	0.0000	0.1250	0.3750	0.0000
67	Co	5.2138	1.7379	0.0000	0.3750	0.1250	0.0000
68	Co	5.2138	5.2138	0.0000	0.3750	0.3750	0.0000
69	Co	1.7379	8.6896	0.0000	0.1250	0.6250	0.0000
70	Co	1.7379	12.1654	0.0000	0.1250	0.8750	0.0000
71	Co	5.2138	8.6896	0.0000	0.3750	0.6250	0.0000
72	Co	5.2138	12.1654	0.0000	0.3750	0.8750	0.0000
73	Co	8.6896	1.7379	0.0000	0.6250	0.1250	0.0000
74	Co	8.6896	5.2138	0.0000	0.6250	0.3750	0.0000
75	Co	12.1654	1.7379	0.0000	0.8750	0.1250	0.0000
76	Co	12.1654	5.2138	0.0000	0.8750	0.3750	0.0000
77	Co	8.6896	8.6896	0.0000	0.6250	0.6250	0.0000
78	Co	8.6896	12.1654	0.0000	0.6250	0.8750	0.0000
79	Co	12.1654	8.6896	0.0000	0.8750	0.6250	0.0000
80	Co	12.1654	12.1654	0.0000	0.8750	0.8750	0.0000
81	Co	1.7379	0.0000	1.7379	0.1250	0.0000	0.0792
82	Co	1.7379	3.4758	1.7379	0.1250	0.2500	0.0792
83	Co	5.2138	0.0000	1.7379	0.3750	0.0000	0.0792
84	Co	5.2138	3.4758	1.7379	0.3750	0.2500	0.0792
85	Co	1.7379	6.9517	1.7379	0.1250	0.5000	0.0792
86	Co	1.7379	10.4275	1.7379	0.1250	0.7500	0.0792
87	Co	5.2138	6.9517	1.7379	0.3750	0.5000	0.0792
88	Co	5.2138	10.4275	1.7379	0.3750	0.7500	0.0792

89	Co	8.6896	0.0000	1.7379	0.6250	0.0000	0.0792
90	Co	8.6896	3.4758	1.7379	0.6250	0.2500	0.0792
91	Co	12.1654	0.0000	1.7379	0.8750	0.0000	0.0792
92	Co	12.1654	3.4758	1.7379	0.8750	0.2500	0.0792
93	Co	8.6896	6.9517	1.7379	0.6250	0.5000	0.0792
94	Co	8.6896	10.4275	1.7379	0.6250	0.7500	0.0792
95	Co	12.1654	6.9517	1.7379	0.8750	0.5000	0.0792
96	Co	12.1654	10.4275	1.7379	0.8750	0.7500	0.0792
97	Co	1.7377	1.7161	3.5139	0.1250	0.1234	0.1601
98	Co	1.7268	5.2207	3.5114	0.1242	0.3755	0.1600
99	Co	5.2100	1.7448	3.5767	0.3747	0.1255	0.1629
100	Co	5.2028	5.2046	3.5996	0.3742	0.3743	0.1640
101	Co	1.7291	8.6703	3.5460	0.1244	0.6236	0.1615
102	Co	1.7355	12.1627	3.5468	0.1248	0.8748	0.1616
103	Co	5.2200	8.6888	3.5537	0.3754	0.6249	0.1619
104	Co	5.2150	12.1675	3.5561	0.3751	0.8751	0.1620
105	Co	8.6914	1.7365	3.5420	0.6251	0.1249	0.1614
106	Co	8.7059	5.2120	3.5168	0.6262	0.3749	0.1602
107	Co	12.1643	1.7287	3.5575	0.8749	0.1243	0.1621
108	Co	12.1539	5.2187	3.5509	0.8742	0.3754	0.1618
109	Co	8.7068	8.6909	3.4583	0.6262	0.6251	0.1575
110	Co	8.6917	12.1597	3.5485	0.6251	0.8746	0.1616
111	Co	12.1547	8.6887	3.5754	0.8742	0.6249	0.1629
112	Co	12.1640	12.1619	3.5552	0.8749	0.8747	0.1620
113	Co	1.7295	-0.0019	5.2244	0.1244	-0.0001	0.2380
114	Co	1.6952	3.4269	5.1109	0.1219	0.2465	0.2328
115	Co	5.2134	13.8918	5.2190	0.3750	0.9992	0.2377
116	Co	5.2190	3.4519	5.3707	0.3754	0.2483	0.2447
117	Co	1.7041	6.9571	5.3097	0.1226	0.5004	0.2419
118	Co	1.7508	10.4108	5.2222	0.1259	0.7488	0.2379
119	Co	5.2837	6.9783	5.2570	0.3800	0.5019	0.2395
120	Co	5.1987	10.4328	5.2167	0.3739	0.7504	0.2376
121	Co	8.6930	13.8886	5.2184	0.6252	0.9989	0.2377
122	Co	8.6958	3.4781	5.2057	0.6254	0.2502	0.2371
123	Co	12.1615	13.8931	5.2282	0.8747	0.9993	0.2382
124	Co	12.1584	3.4708	5.2215	0.8745	0.2496	0.2379
125	Co	8.6901	6.9614	5.2686	0.6250	0.5007	0.2400
126	Co	8.6933	10.4319	5.2839	0.6253	0.7503	0.2407
127	Co	12.1678	6.9474	5.2159	0.8752	0.4997	0.2376
128	Co	12.1702	10.4315	5.2175	0.8753	0.7503	0.2377
129	O	4.9463	3.3152	7.2135	0.3558	0.2384	0.3286
130	O	2.4406	5.9711	6.7758	0.1755	0.4295	0.3087
131	O	4.1606	5.4565	8.9992	0.2993	0.3925	0.4100
132	O	5.6970	6.9834	7.1061	0.4098	0.5023	0.3237
133	O	8.6270	8.7006	6.3135	0.6205	0.6258	0.2876
134	O	7.6064	6.5836	9.1323	0.5471	0.4735	0.4160
135	O	5.1903	8.9878	8.9727	0.3733	0.6465	0.4087
136	Ce	3.7192	7.6562	8.1484	0.2675	0.5507	0.3712
137	Ce	2.9318	3.9833	7.8466	0.2109	0.2865	0.3575

138 Ce	6.3747	5.0395	8.2160	0.4585	0.3625	0.3743
139 Sm	7.1304	8.4281	8.2202	0.5129	0.6062	0.3745

/db/jmorales/CoNi-alloy/metal-SDC-interface/CoNi-Ce_3SmO_7/CoNi-Ce_3SmO_7-conf6c

a = 13.9033384264
b = 13.9033384264
c = 21.9516692133
alpha = 90.0
beta = 90.0
gamma = 90.0

	Atom	X	Y	Z	X	Y	Z
1	Ni	0.0000	0.0000	0.0000	0.0000	0.0000	0.0000
2	Ni	0.0000	3.4758	0.0000	0.0000	0.2500	0.0000
3	Ni	3.4758	0.0000	0.0000	0.2500	0.0000	0.0000
4	Ni	3.4758	3.4758	0.0000	0.2500	0.2500	0.0000
5	Ni	0.0000	6.9517	0.0000	0.0000	0.5000	0.0000
6	Ni	0.0000	10.4275	0.0000	0.0000	0.7500	0.0000
7	Ni	3.4758	6.9517	0.0000	0.2500	0.5000	0.0000
8	Ni	3.4758	10.4275	0.0000	0.2500	0.7500	0.0000
9	Ni	6.9517	0.0000	0.0000	0.5000	0.0000	0.0000
10	Ni	6.9517	3.4758	0.0000	0.5000	0.2500	0.0000
11	Ni	10.4275	0.0000	0.0000	0.7500	0.0000	0.0000
12	Ni	10.4275	3.4758	0.0000	0.7500	0.2500	0.0000
13	Ni	6.9517	6.9517	0.0000	0.5000	0.5000	0.0000
14	Ni	6.9517	10.4275	0.0000	0.5000	0.7500	0.0000
15	Ni	10.4275	6.9517	0.0000	0.7500	0.5000	0.0000
16	Ni	10.4275	10.4275	0.0000	0.7500	0.7500	0.0000
17	Ni	0.0000	1.7379	1.7379	0.0000	0.1250	0.0792
18	Ni	0.0000	5.2138	1.7379	0.0000	0.3750	0.0792
19	Ni	3.4758	1.7379	1.7379	0.2500	0.1250	0.0792
20	Ni	3.4758	5.2138	1.7379	0.2500	0.3750	0.0792
21	Ni	0.0000	8.6896	1.7379	0.0000	0.6250	0.0792
22	Ni	0.0000	12.1654	1.7379	0.0000	0.8750	0.0792
23	Ni	3.4758	8.6896	1.7379	0.2500	0.6250	0.0792
24	Ni	3.4758	12.1654	1.7379	0.2500	0.8750	0.0792
25	Ni	6.9517	1.7379	1.7379	0.5000	0.1250	0.0792
26	Ni	6.9517	5.2138	1.7379	0.5000	0.3750	0.0792
27	Ni	10.4275	1.7379	1.7379	0.7500	0.1250	0.0792
28	Ni	10.4275	5.2138	1.7379	0.7500	0.3750	0.0792
29	Ni	6.9517	8.6896	1.7379	0.5000	0.6250	0.0792
30	Ni	6.9517	12.1654	1.7379	0.5000	0.8750	0.0792
31	Ni	10.4275	8.6896	1.7379	0.7500	0.6250	0.0792
32	Ni	10.4275	12.1654	1.7379	0.7500	0.8750	0.0792
33	Ni	13.8979	13.9017	3.4971	0.9996	0.9999	0.1593
34	Ni	0.0039	3.4702	3.5061	0.0003	0.2496	0.1597
35	Ni	3.4817	0.0277	3.5316	0.2504	0.0020	0.1609
36	Ni	3.4915	3.4869	3.5308	0.2511	0.2508	0.1608
37	Ni	0.0142	6.9462	3.5237	0.0010	0.4996	0.1605
38	Ni	13.9017	10.4269	3.5027	0.9999	0.7500	0.1596
39	Ni	3.4720	6.9730	3.4720	0.2497	0.5015	0.1582

40	Ni	3.4619	10.4539	3.4656	0.2490	0.7519	0.1579
41	Ni	6.9507	13.8937	3.4981	0.4999	0.9993	0.1594
42	Ni	6.9630	3.4685	3.4829	0.5008	0.2495	0.1587
43	Ni	10.4272	13.8930	3.5018	0.7500	0.9993	0.1595
44	Ni	10.4298	3.4737	3.4955	0.7502	0.2498	0.1592
45	Ni	6.9644	6.9314	3.4975	0.5009	0.4985	0.1593
46	Ni	6.9521	10.4202	3.5050	0.5000	0.7495	0.1597
47	Ni	10.4405	6.9544	3.4898	0.7509	0.5002	0.1590
48	Ni	10.4358	10.4230	3.4959	0.7506	0.7497	0.1593
49	Ni	13.8551	1.7138	5.1894	0.9965	0.1233	0.2364
50	Ni	13.8861	5.1900	5.2033	0.9988	0.3733	0.2370
51	Ni	3.5055	1.7026	5.3656	0.2521	0.1225	0.2444
52	Ni	3.4765	5.2718	5.1641	0.2500	0.3792	0.2352
53	Ni	0.0084	8.6929	5.2181	0.0006	0.6252	0.2377
54	Ni	13.8969	12.1572	5.2063	0.9995	0.8744	0.2372
55	Ni	3.4570	8.7445	5.0995	0.2486	0.6289	0.2323
56	Ni	3.4685	12.1738	5.2058	0.2495	0.8756	0.2371
57	Ni	6.9873	1.7054	5.1836	0.5026	0.1227	0.2361
58	Ni	7.0065	5.1952	5.2711	0.5039	0.3737	0.2401
59	Ni	10.4314	1.7174	5.2028	0.7503	0.1235	0.2370
60	Ni	10.4284	5.2096	5.2128	0.7501	0.3747	0.2375
61	Ni	6.9957	8.7117	5.2259	0.5032	0.6266	0.2381
62	Ni	6.9640	12.1403	5.2251	0.5009	0.8732	0.2380
63	Ni	10.4164	8.6935	5.2116	0.7492	0.6253	0.2374
64	Ni	10.4255	12.1580	5.2140	0.7499	0.8745	0.2375
65	Co	1.7379	1.7379	0.0000	0.1250	0.1250	0.0000
66	Co	1.7379	5.2138	0.0000	0.1250	0.3750	0.0000
67	Co	5.2138	1.7379	0.0000	0.3750	0.1250	0.0000
68	Co	5.2138	5.2138	0.0000	0.3750	0.3750	0.0000
69	Co	1.7379	8.6896	0.0000	0.1250	0.6250	0.0000
70	Co	1.7379	12.1654	0.0000	0.1250	0.8750	0.0000
71	Co	5.2138	8.6896	0.0000	0.3750	0.6250	0.0000
72	Co	5.2138	12.1654	0.0000	0.3750	0.8750	0.0000
73	Co	8.6896	1.7379	0.0000	0.6250	0.1250	0.0000
74	Co	8.6896	5.2138	0.0000	0.6250	0.3750	0.0000
75	Co	12.1654	1.7379	0.0000	0.8750	0.1250	0.0000
76	Co	12.1654	5.2138	0.0000	0.8750	0.3750	0.0000
77	Co	8.6896	8.6896	0.0000	0.6250	0.6250	0.0000
78	Co	8.6896	12.1654	0.0000	0.6250	0.8750	0.0000
79	Co	12.1654	8.6896	0.0000	0.8750	0.6250	0.0000
80	Co	12.1654	12.1654	0.0000	0.8750	0.8750	0.0000
81	Co	1.7379	0.0000	1.7379	0.1250	0.0000	0.0792
82	Co	1.7379	3.4758	1.7379	0.1250	0.2500	0.0792
83	Co	5.2138	0.0000	1.7379	0.3750	0.0000	0.0792
84	Co	5.2138	3.4758	1.7379	0.3750	0.2500	0.0792
85	Co	1.7379	6.9517	1.7379	0.1250	0.5000	0.0792
86	Co	1.7379	10.4275	1.7379	0.1250	0.7500	0.0792
87	Co	5.2138	6.9517	1.7379	0.3750	0.5000	0.0792
88	Co	5.2138	10.4275	1.7379	0.3750	0.7500	0.0792

89	Co	8.6896	0.0000	1.7379	0.6250	0.0000	0.0792
90	Co	8.6896	3.4758	1.7379	0.6250	0.2500	0.0792
91	Co	12.1654	0.0000	1.7379	0.8750	0.0000	0.0792
92	Co	12.1654	3.4758	1.7379	0.8750	0.2500	0.0792
93	Co	8.6896	6.9517	1.7379	0.6250	0.5000	0.0792
94	Co	8.6896	10.4275	1.7379	0.6250	0.7500	0.0792
95	Co	12.1654	6.9517	1.7379	0.8750	0.5000	0.0792
96	Co	12.1654	10.4275	1.7379	0.8750	0.7500	0.0792
97	Co	1.7524	1.7402	3.5600	0.1260	0.1252	0.1622
98	Co	1.7200	5.2009	3.5468	0.1237	0.3741	0.1616
99	Co	5.1928	1.7424	3.5824	0.3735	0.1253	0.1632
100	Co	5.2211	5.2316	3.5479	0.3755	0.3763	0.1616
101	Co	1.7082	8.6706	3.5313	0.1229	0.6236	0.1609
102	Co	1.7343	12.1753	3.5499	0.1247	0.8757	0.1617
103	Co	5.2275	8.7143	3.5060	0.3760	0.6268	0.1597
104	Co	5.2166	12.1765	3.5449	0.3752	0.8758	0.1615
105	Co	8.6996	1.7214	3.5381	0.6257	0.1238	0.1612
106	Co	8.6870	5.2093	3.5541	0.6248	0.3747	0.1619
107	Co	12.1579	1.7286	3.5349	0.8745	0.1243	0.1610
108	Co	12.1653	5.2111	3.5518	0.8750	0.3748	0.1618
109	Co	8.6899	8.6941	3.5375	0.6250	0.6253	0.1612
110	Co	8.6917	12.1663	3.5368	0.6252	0.8751	0.1611
111	Co	12.1694	8.6877	3.5634	0.8753	0.6249	0.1623
112	Co	12.1636	12.1601	3.5497	0.8749	0.8746	0.1617
113	Co	1.7079	13.8779	5.2133	0.1228	0.9982	0.2375
114	Co	1.7130	3.4203	5.3057	0.1232	0.2460	0.2417
115	Co	5.2439	13.8839	5.2300	0.3772	0.9986	0.2382
116	Co	5.2846	3.5033	5.2266	0.3801	0.2520	0.2381
117	Co	1.7451	6.9547	5.3442	0.1255	0.5002	0.2435
118	Co	1.7445	10.4240	5.2163	0.1255	0.7497	0.2376
119	Co	5.2279	7.0346	5.1766	0.3760	0.5060	0.2358
120	Co	5.1763	10.4659	5.1871	0.3723	0.7528	0.2363
121	Co	8.6877	13.8778	5.2326	0.6249	0.9982	0.2384
122	Co	8.7343	3.4437	5.2034	0.6282	0.2477	0.2370
123	Co	12.1611	13.8882	5.2244	0.8747	0.9989	0.2380
124	Co	12.1597	3.4719	5.2151	0.8746	0.2497	0.2376
125	Co	8.7397	6.9319	5.1816	0.6286	0.4986	0.2360
126	Co	8.7065	10.4463	5.2049	0.6262	0.7513	0.2371
127	Co	12.1683	6.9503	5.2184	0.8752	0.4999	0.2377
128	Co	12.1633	10.4253	5.2241	0.8748	0.7498	0.2380
129	O	2.6277	2.7188	6.7368	0.1890	0.1955	0.3069
130	O	5.9782	4.3308	6.8079	0.4300	0.3115	0.3101
131	O	2.7677	6.1482	6.8027	0.1991	0.4422	0.3099
132	O	4.9126	6.2134	8.8584	0.3533	0.4469	0.4035
133	O	6.0383	7.8870	6.7881	0.4343	0.5673	0.3092
134	O	7.9505	8.0820	8.8807	0.5718	0.5813	0.4046
135	O	5.1105	9.8076	8.6906	0.3676	0.7054	0.3959
136	Ce	3.9318	8.1380	7.9452	0.2828	0.5853	0.3619
137	Ce	3.8790	4.3908	7.8232	0.2790	0.3158	0.3564

138 Ce	7.1122	6.2474	8.1058	0.5115	0.4493	0.3693
139 Sm	7.0801	9.7655	7.9408	0.5092	0.7024	0.3617

/db/jmorales/CoNi-alloy/metal-SDC-interface/CoNi-Ce_3SmO_7/CoNi-Ce_3SmO_7-conf4b

a =				13.9033384264			
b =				13.9033384264			
c =				21.9516692133			
alpha =				90.0			
beta =				90.0			
gamma =				90.0			
	Atom	X	Y	Z	X	Y	Z
1	Ni	0.0000	0.0000	0.0000	0.0000	0.0000	0.0000
2	Ni	0.0000	3.4758	0.0000	0.0000	0.2500	0.0000
3	Ni	3.4758	0.0000	0.0000	0.2500	0.0000	0.0000
4	Ni	3.4758	3.4758	0.0000	0.2500	0.2500	0.0000
5	Ni	0.0000	6.9517	0.0000	0.0000	0.5000	0.0000
6	Ni	0.0000	10.4275	0.0000	0.0000	0.7500	0.0000
7	Ni	3.4758	6.9517	0.0000	0.2500	0.5000	0.0000
8	Ni	3.4758	10.4275	0.0000	0.2500	0.7500	0.0000
9	Ni	6.9517	0.0000	0.0000	0.5000	0.0000	0.0000
10	Ni	6.9517	3.4758	0.0000	0.5000	0.2500	0.0000
11	Ni	10.4275	0.0000	0.0000	0.7500	0.0000	0.0000
12	Ni	10.4275	3.4758	0.0000	0.7500	0.2500	0.0000
13	Ni	6.9517	6.9517	0.0000	0.5000	0.5000	0.0000
14	Ni	6.9517	10.4275	0.0000	0.5000	0.7500	0.0000
15	Ni	10.4275	6.9517	0.0000	0.7500	0.5000	0.0000
16	Ni	10.4275	10.4275	0.0000	0.7500	0.7500	0.0000
17	Ni	0.0000	1.7379	1.7379	0.0000	0.1250	0.0792
18	Ni	0.0000	5.2138	1.7379	0.0000	0.3750	0.0792
19	Ni	3.4758	1.7379	1.7379	0.2500	0.1250	0.0792
20	Ni	3.4758	5.2138	1.7379	0.2500	0.3750	0.0792
21	Ni	0.0000	8.6896	1.7379	0.0000	0.6250	0.0792
22	Ni	0.0000	12.1654	1.7379	0.0000	0.8750	0.0792
23	Ni	3.4758	8.6896	1.7379	0.2500	0.6250	0.0792
24	Ni	3.4758	12.1654	1.7379	0.2500	0.8750	0.0792
25	Ni	6.9517	1.7379	1.7379	0.5000	0.1250	0.0792
26	Ni	6.9517	5.2138	1.7379	0.5000	0.3750	0.0792
27	Ni	10.4275	1.7379	1.7379	0.7500	0.1250	0.0792
28	Ni	10.4275	5.2138	1.7379	0.7500	0.3750	0.0792
29	Ni	6.9517	8.6896	1.7379	0.5000	0.6250	0.0792
30	Ni	6.9517	12.1654	1.7379	0.5000	0.8750	0.0792
31	Ni	10.4275	8.6896	1.7379	0.7500	0.6250	0.0792
32	Ni	10.4275	12.1654	1.7379	0.7500	0.8750	0.0792
33	Ni	-0.0009	-0.0000	3.5042	-0.0001	-0.0000	0.1596
34	Ni	13.8995	3.4758	3.4996	0.9997	0.2500	0.1594
35	Ni	3.4782	0.0036	3.5056	0.2502	0.0003	0.1597
36	Ni	3.4873	3.4919	3.5119	0.2508	0.2512	0.1600
37	Ni	0.0114	6.9466	3.5226	0.0008	0.4996	0.1605
38	Ni	13.9014	10.4278	3.5029	0.9999	0.7500	0.1596
39	Ni	3.4774	6.9405	3.4952	0.2501	0.4992	0.1592

40	Ni	3.4695	10.4605	3.4644	0.2495	0.7524	0.1578
41	Ni	6.9510	13.9015	3.5033	0.5000	0.9999	0.1596
42	Ni	6.9504	3.4686	3.4868	0.4999	0.2495	0.1588
43	Ni	10.4270	13.9008	3.5033	0.7500	0.9998	0.1596
44	Ni	10.4286	3.4728	3.5022	0.7501	0.2498	0.1595
45	Ni	6.9608	6.9406	3.4864	0.5007	0.4992	0.1588
46	Ni	6.9528	10.4188	3.5064	0.5001	0.7494	0.1597
47	Ni	10.4340	6.9545	3.4987	0.7505	0.5002	0.1594
48	Ni	10.4329	10.4259	3.4990	0.7504	0.7499	0.1594
49	Ni	13.8948	1.7352	5.2092	0.9994	0.1248	0.2373
50	Ni	13.8767	5.1910	5.2076	0.9981	0.3734	0.2372
51	Ni	3.4861	1.7476	5.2214	0.2507	0.1257	0.2379
52	Ni	3.4375	5.2005	5.2661	0.2472	0.3741	0.2399
53	Ni	0.0095	8.6903	5.2189	0.0007	0.6251	0.2377
54	Ni	0.0084	12.1640	5.2083	0.0006	0.8749	0.2373
55	Ni	3.4728	8.7344	5.0637	0.2498	0.6282	0.2307
56	Ni	3.4574	12.1729	5.2024	0.2487	0.8755	0.2370
57	Ni	6.9561	1.7344	5.2029	0.5003	0.1247	0.2370
58	Ni	7.0144	5.1930	5.2261	0.5045	0.3735	0.2381
59	Ni	10.4287	1.7303	5.2068	0.7501	0.1245	0.2372
60	Ni	10.4331	5.2046	5.2136	0.7504	0.3743	0.2375
61	Ni	6.9701	8.6961	5.2198	0.5013	0.6255	0.2378
62	Ni	6.9664	12.1550	5.2182	0.5011	0.8742	0.2377
63	Ni	10.4149	8.6933	5.2167	0.7491	0.6253	0.2376
64	Ni	10.4191	12.1631	5.2140	0.7494	0.8748	0.2375
65	Co	1.7379	1.7379	0.0000	0.1250	0.1250	0.0000
66	Co	1.7379	5.2138	0.0000	0.1250	0.3750	0.0000
67	Co	5.2138	1.7379	0.0000	0.3750	0.1250	0.0000
68	Co	5.2138	5.2138	0.0000	0.3750	0.3750	0.0000
69	Co	1.7379	8.6896	0.0000	0.1250	0.6250	0.0000
70	Co	1.7379	12.1654	0.0000	0.1250	0.8750	0.0000
71	Co	5.2138	8.6896	0.0000	0.3750	0.6250	0.0000
72	Co	5.2138	12.1654	0.0000	0.3750	0.8750	0.0000
73	Co	8.6896	1.7379	0.0000	0.6250	0.1250	0.0000
74	Co	8.6896	5.2138	0.0000	0.6250	0.3750	0.0000
75	Co	12.1654	1.7379	0.0000	0.8750	0.1250	0.0000
76	Co	12.1654	5.2138	0.0000	0.8750	0.3750	0.0000
77	Co	8.6896	8.6896	0.0000	0.6250	0.6250	0.0000
78	Co	8.6896	12.1654	0.0000	0.6250	0.8750	0.0000
79	Co	12.1654	8.6896	0.0000	0.8750	0.6250	0.0000
80	Co	12.1654	12.1654	0.0000	0.8750	0.8750	0.0000
81	Co	1.7379	0.0000	1.7379	0.1250	0.0000	0.0792
82	Co	1.7379	3.4758	1.7379	0.1250	0.2500	0.0792
83	Co	5.2138	0.0000	1.7379	0.3750	0.0000	0.0792
84	Co	5.2138	3.4758	1.7379	0.3750	0.2500	0.0792
85	Co	1.7379	6.9517	1.7379	0.1250	0.5000	0.0792
86	Co	1.7379	10.4275	1.7379	0.1250	0.7500	0.0792
87	Co	5.2138	6.9517	1.7379	0.3750	0.5000	0.0792
88	Co	5.2138	10.4275	1.7379	0.3750	0.7500	0.0792

89	Co	8.6896	0.0000	1.7379	0.6250	0.0000	0.0792
90	Co	8.6896	3.4758	1.7379	0.6250	0.2500	0.0792
91	Co	12.1654	0.0000	1.7379	0.8750	0.0000	0.0792
92	Co	12.1654	3.4758	1.7379	0.8750	0.2500	0.0792
93	Co	8.6896	6.9517	1.7379	0.6250	0.5000	0.0792
94	Co	8.6896	10.4275	1.7379	0.6250	0.7500	0.0792
95	Co	12.1654	6.9517	1.7379	0.8750	0.5000	0.0792
96	Co	12.1654	10.4275	1.7379	0.8750	0.7500	0.0792
97	Co	1.7434	1.7361	3.5481	0.1254	0.1249	0.1616
98	Co	1.7399	5.2184	3.5682	0.1251	0.3753	0.1625
99	Co	5.2120	1.7373	3.5508	0.3749	0.1250	0.1618
100	Co	5.2024	5.2226	3.4948	0.3742	0.3756	0.1592
101	Co	1.7062	8.6679	3.5268	0.1227	0.6234	0.1607
102	Co	1.7301	12.1757	3.5477	0.1244	0.8757	0.1616
103	Co	5.2294	8.7017	3.5057	0.3761	0.6259	0.1597
104	Co	5.2199	12.1795	3.5514	0.3754	0.8760	0.1618
105	Co	8.6900	1.7329	3.5506	0.6250	0.1246	0.1617
106	Co	8.6910	5.2062	3.5556	0.6251	0.3745	0.1620
107	Co	12.1642	1.7326	3.5467	0.8749	0.1246	0.1616
108	Co	12.1651	5.2125	3.5496	0.8750	0.3749	0.1617
109	Co	8.6856	8.6888	3.5480	0.6247	0.6249	0.1616
110	Co	8.6920	12.1699	3.5383	0.6252	0.8753	0.1612
111	Co	12.1676	8.6904	3.5639	0.8752	0.6251	0.1624
112	Co	12.1647	12.1657	3.5593	0.8750	0.8750	0.1621
113	Co	1.7426	0.0186	5.2315	0.1253	0.0013	0.2383
114	Co	1.7253	3.4271	5.1991	0.1241	0.2465	0.2368
115	Co	5.2169	-0.0002	5.2332	0.3752	-0.0000	0.2384
116	Co	5.2312	3.4664	5.2412	0.3763	0.2493	0.2388
117	Co	1.7526	6.9738	5.3638	0.1261	0.5016	0.2443
118	Co	1.7509	10.4129	5.2083	0.1259	0.7489	0.2373
119	Co	5.2226	6.9849	5.2252	0.3756	0.5024	0.2380
120	Co	5.1826	10.4703	5.1950	0.3728	0.7531	0.2367
121	Co	8.6835	13.9002	5.2288	0.6246	0.9998	0.2382
122	Co	8.7002	3.4608	5.2147	0.6258	0.2489	0.2376
123	Co	12.1638	13.8978	5.2248	0.8749	0.9996	0.2380
124	Co	12.1631	3.4661	5.2221	0.8748	0.2493	0.2379
125	Co	8.7291	6.9264	5.1968	0.6278	0.4982	0.2367
126	Co	8.6908	10.4334	5.2122	0.6251	0.7504	0.2374
127	Co	12.1629	6.9564	5.2195	0.8748	0.5003	0.2378
128	Co	12.1649	10.4257	5.2217	0.8750	0.7499	0.2379
129	O	5.3644	5.1306	6.3412	0.3858	0.3690	0.2889
130	O	5.9808	4.2471	8.8718	0.4302	0.3055	0.4042
131	O	2.6299	6.2061	6.8339	0.1892	0.4464	0.3113
132	O	4.6598	6.5739	8.8642	0.3352	0.4728	0.4038
133	O	6.0828	7.8922	6.8177	0.4375	0.5676	0.3106
134	O	7.8004	7.7422	9.0111	0.5610	0.5569	0.4105
135	O	5.2338	9.9771	8.5285	0.3764	0.7176	0.3885
136	Ce	3.8703	8.4228	7.8170	0.2784	0.6058	0.3561
137	Ce	3.8879	4.5771	8.1466	0.2796	0.3292	0.3711

138 Ce	6.8262	6.0627	8.2485	0.4910	0.4361	0.3758
139 Sm	7.2084	9.5687	7.9583	0.5185	0.6882	0.3625

/db/jmorales/CoNi-alloy/metal-SDC-interface/CoNi-Ce$_3$SmO$_7$/CoNi-Ce$_3$SmO$_7$-conf3b

a =				13.9033384264			
b =				13.9033384264			
c =				21.9516692133			
alpha =				90.0			
beta =				90.0			
gamma =				90.0			
	Atom	X	Y	Z	X	Y	Z
1	Ni	0.0000	0.0000	0.0000	0.0000	0.0000	0.0000
2	Ni	0.0000	3.4758	0.0000	0.0000	0.2500	0.0000
3	Ni	3.4758	0.0000	0.0000	0.2500	0.0000	0.0000
4	Ni	3.4758	3.4758	0.0000	0.2500	0.2500	0.0000
5	Ni	0.0000	6.9517	0.0000	0.0000	0.5000	0.0000
6	Ni	0.0000	10.4275	0.0000	0.0000	0.7500	0.0000
7	Ni	3.4758	6.9517	0.0000	0.2500	0.5000	0.0000
8	Ni	3.4758	10.4275	0.0000	0.2500	0.7500	0.0000
9	Ni	6.9517	0.0000	0.0000	0.5000	0.0000	0.0000
10	Ni	6.9517	3.4758	0.0000	0.5000	0.2500	0.0000
11	Ni	10.4275	0.0000	0.0000	0.7500	0.0000	0.0000
12	Ni	10.4275	3.4758	0.0000	0.7500	0.2500	0.0000
13	Ni	6.9517	6.9517	0.0000	0.5000	0.5000	0.0000
14	Ni	6.9517	10.4275	0.0000	0.5000	0.7500	0.0000
15	Ni	10.4275	6.9517	0.0000	0.7500	0.5000	0.0000
16	Ni	10.4275	10.4275	0.0000	0.7500	0.7500	0.0000
17	Ni	0.0000	1.7379	1.7379	0.0000	0.1250	0.0792
18	Ni	0.0000	5.2138	1.7379	0.0000	0.3750	0.0792
19	Ni	3.4758	1.7379	1.7379	0.2500	0.1250	0.0792
20	Ni	3.4758	5.2138	1.7379	0.2500	0.3750	0.0792
21	Ni	0.0000	8.6896	1.7379	0.0000	0.6250	0.0792
22	Ni	0.0000	12.1654	1.7379	0.0000	0.8750	0.0792
23	Ni	3.4758	8.6896	1.7379	0.2500	0.6250	0.0792
24	Ni	3.4758	12.1654	1.7379	0.2500	0.8750	0.0792
25	Ni	6.9517	1.7379	1.7379	0.5000	0.1250	0.0792
26	Ni	6.9517	5.2138	1.7379	0.5000	0.3750	0.0792
27	Ni	10.4275	1.7379	1.7379	0.7500	0.1250	0.0792
28	Ni	10.4275	5.2138	1.7379	0.7500	0.3750	0.0792
29	Ni	6.9517	8.6896	1.7379	0.5000	0.6250	0.0792
30	Ni	6.9517	12.1654	1.7379	0.5000	0.8750	0.0792
31	Ni	10.4275	8.6896	1.7379	0.7500	0.6250	0.0792
32	Ni	10.4275	12.1654	1.7379	0.7500	0.8750	0.0792
33	Ni	0.0012	0.0013	3.5017	0.0001	0.0001	0.1595
34	Ni	-0.0007	3.4696	3.5005	-0.0001	0.2495	0.1595
35	Ni	3.4740	-0.0011	3.5031	0.2499	-0.0001	0.1596
36	Ni	3.4770	3.4559	3.4803	0.2501	0.2486	0.1585
37	Ni	13.9013	6.9528	3.5084	0.9999	0.5001	0.1598
38	Ni	0.0002	10.4311	3.5018	0.0000	0.7503	0.1595
39	Ni	3.4625	6.9634	3.4789	0.2490	0.5008	0.1585

40	Ni	3.4696	10.4101	3.5127	0.2496	0.7487	0.1600
41	Ni	6.9515	-0.0002	3.5006	0.5000	-0.0000	0.1595
42	Ni	6.9444	3.4915	3.5077	0.4995	0.2511	0.1598
43	Ni	10.4304	0.0035	3.5033	0.7502	0.0003	0.1596
44	Ni	10.4274	3.4940	3.5205	0.7500	0.2513	0.1604
45	Ni	6.9524	6.9470	3.5569	0.5001	0.4997	0.1620
46	Ni	6.9538	10.4171	3.4883	0.5002	0.7493	0.1589
47	Ni	10.4336	6.9490	3.4957	0.7504	0.4998	0.1592
48	Ni	10.4514	10.4328	3.4750	0.7517	0.7504	0.1583
49	Ni	0.0055	1.7314	5.2083	0.0004	0.1245	0.2373
50	Ni	0.0092	5.2006	5.2079	0.0007	0.3741	0.2372
51	Ni	3.4693	1.7272	5.2109	0.2495	0.1242	0.2374
52	Ni	3.4533	5.1688	5.1176	0.2484	0.3718	0.2331
53	Ni	-0.0002	8.6990	5.2075	-0.0000	0.6257	0.2372
54	Ni	-0.0017	12.1723	5.2089	-0.0001	0.8755	0.2373
55	Ni	3.4672	8.7679	5.3298	0.2494	0.6306	0.2428
56	Ni	3.4756	12.1575	5.2067	0.2500	0.8744	0.2372
57	Ni	6.9442	1.7210	5.1963	0.4995	0.1238	0.2367
58	Ni	6.8856	5.1902	5.3394	0.4952	0.3733	0.2432
59	Ni	10.4356	1.7355	5.2057	0.7506	0.1248	0.2371
60	Ni	10.4408	5.1340	5.3538	0.7510	0.3693	0.2439
61	Ni	7.0015	8.7565	5.2752	0.5036	0.6298	0.2403
62	Ni	6.9623	12.1611	5.2021	0.5008	0.8747	0.2370
63	Ni	10.4378	8.7441	5.1680	0.7507	0.6289	0.2354
64	Ni	10.4278	12.1716	5.2110	0.7500	0.8754	0.2374
65	Co	1.7379	1.7379	0.0000	0.1250	0.1250	0.0000
66	Co	1.7379	5.2138	0.0000	0.1250	0.3750	0.0000
67	Co	5.2138	1.7379	0.0000	0.3750	0.1250	0.0000
68	Co	5.2138	5.2138	0.0000	0.3750	0.3750	0.0000
69	Co	1.7379	8.6896	0.0000	0.1250	0.6250	0.0000
70	Co	1.7379	12.1654	0.0000	0.1250	0.8750	0.0000
71	Co	5.2138	8.6896	0.0000	0.3750	0.6250	0.0000
72	Co	5.2138	12.1654	0.0000	0.3750	0.8750	0.0000
73	Co	8.6896	1.7379	0.0000	0.6250	0.1250	0.0000
74	Co	8.6896	5.2138	0.0000	0.6250	0.3750	0.0000
75	Co	12.1654	1.7379	0.0000	0.8750	0.1250	0.0000
76	Co	12.1654	5.2138	0.0000	0.8750	0.3750	0.0000
77	Co	8.6896	8.6896	0.0000	0.6250	0.6250	0.0000
78	Co	8.6896	12.1654	0.0000	0.6250	0.8750	0.0000
79	Co	12.1654	8.6896	0.0000	0.8750	0.6250	0.0000
80	Co	12.1654	12.1654	0.0000	0.8750	0.8750	0.0000
81	Co	1.7379	0.0000	1.7379	0.1250	0.0000	0.0792
82	Co	1.7379	3.4758	1.7379	0.1250	0.2500	0.0792
83	Co	5.2138	0.0000	1.7379	0.3750	0.0000	0.0792
84	Co	5.2138	3.4758	1.7379	0.3750	0.2500	0.0792
85	Co	1.7379	6.9517	1.7379	0.1250	0.5000	0.0792
86	Co	1.7379	10.4275	1.7379	0.1250	0.7500	0.0792
87	Co	5.2138	6.9517	1.7379	0.3750	0.5000	0.0792
88	Co	5.2138	10.4275	1.7379	0.3750	0.7500	0.0792

89	Co	8.6896	0.0000	1.7379	0.6250	0.0000	0.0792
90	Co	8.6896	3.4758	1.7379	0.6250	0.2500	0.0792
91	Co	12.1654	0.0000	1.7379	0.8750	0.0000	0.0792
92	Co	12.1654	3.4758	1.7379	0.8750	0.2500	0.0792
93	Co	8.6896	6.9517	1.7379	0.6250	0.5000	0.0792
94	Co	8.6896	10.4275	1.7379	0.6250	0.7500	0.0792
95	Co	12.1654	6.9517	1.7379	0.8750	0.5000	0.0792
96	Co	12.1654	10.4275	1.7379	0.8750	0.7500	0.0792
97	Co	1.7357	1.7356	3.5485	0.1248	0.1248	0.1617
98	Co	1.7172	5.2124	3.5177	0.1235	0.3749	0.1602
99	Co	5.2126	1.7277	3.5425	0.3749	0.1243	0.1614
100	Co	5.2361	5.2281	3.5450	0.3766	0.3760	0.1615
101	Co	1.7512	8.6894	3.5698	0.1260	0.6250	0.1626
102	Co	1.7383	12.1662	3.5465	0.1250	0.8751	0.1616
103	Co	5.2279	8.7061	3.5487	0.3760	0.6262	0.1617
104	Co	5.2157	12.1823	3.5216	0.3751	0.8762	0.1604
105	Co	8.6892	1.7370	3.5513	0.6250	0.1249	0.1618
106	Co	8.6685	5.2088	3.5804	0.6235	0.3746	0.1631
107	Co	12.1660	1.7369	3.5474	0.8750	0.1249	0.1616
108	Co	12.1481	5.2151	3.5764	0.8738	0.3751	0.1629
109	Co	8.6788	8.6735	3.5213	0.6242	0.6238	0.1604
110	Co	8.6913	12.1950	3.5107	0.6251	0.8771	0.1599
111	Co	12.1762	8.6864	3.5425	0.8758	0.6248	0.1614
112	Co	12.1692	12.1736	3.5487	0.8753	0.8756	0.1617
113	Co	1.7400	13.8956	5.2251	0.1252	0.9994	0.2380
114	Co	1.7454	3.4754	5.2225	0.1255	0.2500	0.2379
115	Co	5.2129	13.8924	5.2269	0.3749	0.9992	0.2381
116	Co	5.1808	3.4361	5.2092	0.3726	0.2471	0.2373
117	Co	1.7181	6.9271	5.2193	0.1236	0.4982	0.2378
118	Co	1.7150	10.4411	5.2313	0.1234	0.7510	0.2383
119	Co	5.1463	6.9866	5.1920	0.3702	0.5025	0.2365
120	Co	5.2204	10.4840	5.1727	0.3755	0.7541	0.2356
121	Co	8.6895	13.8972	5.2314	0.6250	0.9996	0.2383
122	Co	8.6719	3.4194	5.2156	0.6237	0.2459	0.2376
123	Co	12.1653	0.0010	5.2272	0.8750	0.0001	0.2381
124	Co	12.1858	3.4630	5.2232	0.8765	0.2491	0.2379
125	Co	8.7518	6.9389	5.2444	0.6295	0.4991	0.2389
126	Co	8.7326	10.4773	5.1137	0.6281	0.7536	0.2330
127	Co	12.1902	6.9688	5.2263	0.8768	0.5012	0.2381
128	Co	12.1646	10.4359	5.2247	0.8749	0.7506	0.2380
129	O	6.6382	5.0064	7.2208	0.4775	0.3601	0.3289
130	O	4.0922	7.5308	6.7177	0.2943	0.5417	0.3060
131	O	5.7045	7.0481	9.0954	0.4103	0.5069	0.4143
132	O	9.6496	6.2068	6.6713	0.6940	0.4464	0.3039
133	O	7.2946	8.4797	7.1416	0.5247	0.6099	0.3253
134	O	9.1766	8.3975	9.2022	0.6600	0.6040	0.4192
135	O	6.7399	10.6166	8.8908	0.4848	0.7636	0.4050
136	Ce	5.3051	9.1701	8.1724	0.3816	0.6596	0.3723
137	Ce	4.6259	5.5557	7.8581	0.3327	0.3996	0.3580

138 Ce	8.0846	6.6675	8.4260	0.5815	0.4796	0.3838
139 Sm	8.6289	10.0970	8.0867	0.6206	0.7262	0.3684

/db/jmorales/CoNi-alloy/metal-SDC-interface/CoNi-Ce_3SmO_7/CoNi-Ce_3SmO_7-conf5a

a =			13.9033384264				
b =			13.9033384264				
c =			21.9516692133				
alpha =			90.0				
beta =			90.0				
gamma =			90.0				
	Atom	X	Y	Z	X	Y	Z
1	Ni	0.0000	0.0000	0.0000	0.0000	0.0000	0.0000
2	Ni	0.0000	3.4758	0.0000	0.0000	0.2500	0.0000
3	Ni	3.4758	0.0000	0.0000	0.2500	0.0000	0.0000
4	Ni	3.4758	3.4758	0.0000	0.2500	0.2500	0.0000
5	Ni	0.0000	6.9517	0.0000	0.0000	0.5000	0.0000
6	Ni	0.0000	10.4275	0.0000	0.0000	0.7500	0.0000
7	Ni	3.4758	6.9517	0.0000	0.2500	0.5000	0.0000
8	Ni	3.4758	10.4275	0.0000	0.2500	0.7500	0.0000
9	Ni	6.9517	0.0000	0.0000	0.5000	0.0000	0.0000
10	Ni	6.9517	3.4758	0.0000	0.5000	0.2500	0.0000
11	Ni	10.4275	0.0000	0.0000	0.7500	0.0000	0.0000
12	Ni	10.4275	3.4758	0.0000	0.7500	0.2500	0.0000
13	Ni	6.9517	6.9517	0.0000	0.5000	0.5000	0.0000
14	Ni	6.9517	10.4275	0.0000	0.5000	0.7500	0.0000
15	Ni	10.4275	6.9517	0.0000	0.7500	0.5000	0.0000
16	Ni	10.4275	10.4275	0.0000	0.7500	0.7500	0.0000
17	Ni	0.0000	1.7379	1.7379	0.0000	0.1250	0.0792
18	Ni	0.0000	5.2138	1.7379	0.0000	0.3750	0.0792
19	Ni	3.4758	1.7379	1.7379	0.2500	0.1250	0.0792
20	Ni	3.4758	5.2138	1.7379	0.2500	0.3750	0.0792
21	Ni	0.0000	8.6896	1.7379	0.0000	0.6250	0.0792
22	Ni	0.0000	12.1654	1.7379	0.0000	0.8750	0.0792
23	Ni	3.4758	8.6896	1.7379	0.2500	0.6250	0.0792
24	Ni	3.4758	12.1654	1.7379	0.2500	0.8750	0.0792
25	Ni	6.9517	1.7379	1.7379	0.5000	0.1250	0.0792
26	Ni	6.9517	5.2138	1.7379	0.5000	0.3750	0.0792
27	Ni	10.4275	1.7379	1.7379	0.7500	0.1250	0.0792
28	Ni	10.4275	5.2138	1.7379	0.7500	0.3750	0.0792
29	Ni	6.9517	8.6896	1.7379	0.5000	0.6250	0.0792
30	Ni	6.9517	12.1654	1.7379	0.5000	0.8750	0.0792
31	Ni	10.4275	8.6896	1.7379	0.7500	0.6250	0.0792
32	Ni	10.4275	12.1654	1.7379	0.7500	0.8750	0.0792
33	Ni	13.8999	13.8987	3.5032	0.9997	0.9997	0.1596
34	Ni	13.8986	3.4795	3.4973	0.9997	0.2503	0.1593
35	Ni	3.4746	13.8904	3.4920	0.2499	0.9991	0.1591
36	Ni	3.4906	3.4765	3.5097	0.2511	0.2500	0.1599
37	Ni	13.8848	6.9470	3.4926	0.9987	0.4997	0.1591
38	Ni	13.9030	10.4278	3.5057	1.0000	0.7500	0.1597
39	Ni	3.4922	6.9456	3.4992	0.2512	0.4996	0.1594

40	Ni	3.4739	10.4363	3.4941	0.2499	0.7506	0.1592
41	Ni	6.9518	13.9012	3.5055	0.5000	0.9998	0.1597
42	Ni	6.9328	3.4676	3.5102	0.4986	0.2494	0.1599
43	Ni	10.4288	13.9032	3.5028	0.7501	1.0000	0.1596
44	Ni	10.4356	3.4777	3.5047	0.7506	0.2501	0.1597
45	Ni	6.9418	6.9539	3.4960	0.4993	0.5002	0.1593
46	Ni	6.9556	10.4372	3.4886	0.5003	0.7507	0.1589
47	Ni	10.4348	6.9494	3.5084	0.7505	0.4998	0.1598
48	Ni	10.4261	10.4271	3.5067	0.7499	0.7500	0.1597
49	Ni	0.0004	1.7317	5.2024	0.0000	0.1246	0.2370
50	Ni	0.0327	5.2179	5.2827	0.0024	0.3753	0.2407
51	Ni	3.4404	1.7149	5.1566	0.2475	0.1233	0.2349
52	Ni	3.3981	5.2164	5.2011	0.2444	0.3752	0.2369
53	Ni	13.9015	8.6895	5.2131	0.9999	0.6250	0.2375
54	Ni	13.9014	12.1660	5.2080	0.9999	0.8750	0.2372
55	Ni	3.4561	8.7156	5.1792	0.2486	0.6269	0.2359
56	Ni	3.4810	12.1564	5.2063	0.2504	0.8744	0.2372
57	Ni	6.9923	1.7065	5.2115	0.5029	0.1227	0.2374
58	Ni	6.9997	5.2108	5.1684	0.5035	0.3748	0.2354
59	Ni	10.4212	1.7398	5.2067	0.7495	0.1251	0.2372
60	Ni	10.4196	5.2184	5.2118	0.7494	0.3753	0.2374
61	Ni	6.9635	8.7190	5.1635	0.5009	0.6271	0.2352
62	Ni	6.9473	12.1634	5.2123	0.4997	0.8749	0.2374
63	Ni	10.4236	8.6872	5.2103	0.7497	0.6248	0.2374
64	Ni	10.4286	12.1594	5.2098	0.7501	0.8746	0.2373
65	Co	1.7379	1.7379	0.0000	0.1250	0.1250	0.0000
66	Co	1.7379	5.2138	0.0000	0.1250	0.3750	0.0000
67	Co	5.2138	1.7379	0.0000	0.3750	0.1250	0.0000
68	Co	5.2138	5.2138	0.0000	0.3750	0.3750	0.0000
69	Co	1.7379	8.6896	0.0000	0.1250	0.6250	0.0000
70	Co	1.7379	12.1654	0.0000	0.1250	0.8750	0.0000
71	Co	5.2138	8.6896	0.0000	0.3750	0.6250	0.0000
72	Co	5.2138	12.1654	0.0000	0.3750	0.8750	0.0000
73	Co	8.6896	1.7379	0.0000	0.6250	0.1250	0.0000
74	Co	8.6896	5.2138	0.0000	0.6250	0.3750	0.0000
75	Co	12.1654	1.7379	0.0000	0.8750	0.1250	0.0000
76	Co	12.1654	5.2138	0.0000	0.8750	0.3750	0.0000
77	Co	8.6896	8.6896	0.0000	0.6250	0.6250	0.0000
78	Co	8.6896	12.1654	0.0000	0.6250	0.8750	0.0000
79	Co	12.1654	8.6896	0.0000	0.8750	0.6250	0.0000
80	Co	12.1654	12.1654	0.0000	0.8750	0.8750	0.0000
81	Co	1.7379	0.0000	1.7379	0.1250	0.0000	0.0792
82	Co	1.7379	3.4758	1.7379	0.1250	0.2500	0.0792
83	Co	5.2138	0.0000	1.7379	0.3750	0.0000	0.0792
84	Co	5.2138	3.4758	1.7379	0.3750	0.2500	0.0792
85	Co	1.7379	6.9517	1.7379	0.1250	0.5000	0.0792
86	Co	1.7379	10.4275	1.7379	0.1250	0.7500	0.0792
87	Co	5.2138	6.9517	1.7379	0.3750	0.5000	0.0792
88	Co	5.2138	10.4275	1.7379	0.3750	0.7500	0.0792

89	Co	8.6896	0.0000	1.7379	0.6250	0.0000	0.0792
90	Co	8.6896	3.4758	1.7379	0.6250	0.2500	0.0792
91	Co	12.1654	0.0000	1.7379	0.8750	0.0000	0.0792
92	Co	12.1654	3.4758	1.7379	0.8750	0.2500	0.0792
93	Co	8.6896	6.9517	1.7379	0.6250	0.5000	0.0792
94	Co	8.6896	10.4275	1.7379	0.6250	0.7500	0.0792
95	Co	12.1654	6.9517	1.7379	0.8750	0.5000	0.0792
96	Co	12.1654	10.4275	1.7379	0.8750	0.7500	0.0792
97	Co	1.7278	1.7290	3.5188	0.1243	0.1244	0.1603
98	Co	1.7276	5.2194	3.4565	0.1243	0.3754	0.1575
99	Co	5.2185	1.7442	3.5669	0.3753	0.1254	0.1625
100	Co	5.1992	5.1945	3.5949	0.3740	0.3736	0.1638
101	Co	1.7355	8.6914	3.5391	0.1248	0.6251	0.1612
102	Co	1.7358	12.1630	3.5557	0.1248	0.8748	0.1620
103	Co	5.2086	8.6912	3.5317	0.3746	0.6251	0.1609
104	Co	5.2171	12.1737	3.5402	0.3752	0.8756	0.1613
105	Co	8.6909	1.7384	3.5448	0.6251	0.1250	0.1615
106	Co	8.7027	5.2202	3.5258	0.6259	0.3755	0.1606
107	Co	12.1664	1.7370	3.5559	0.8751	0.1249	0.1620
108	Co	12.1889	5.2125	3.5921	0.8767	0.3749	0.1636
109	Co	8.7023	8.6857	3.5352	0.6259	0.6247	0.1610
110	Co	8.6912	12.1640	3.5518	0.6251	0.8749	0.1618
111	Co	12.1653	8.6919	3.5559	0.8750	0.6252	0.1620
112	Co	12.1622	12.1631	3.5534	0.8748	0.8748	0.1619
113	Co	1.7416	0.0060	5.2222	0.1253	0.0004	0.2379
114	Co	1.7156	3.4545	5.2059	0.1234	0.2485	0.2372
115	Co	5.1999	13.8990	5.2152	0.3740	0.9997	0.2376
116	Co	5.2424	3.4389	5.3641	0.3771	0.2473	0.2444
117	Co	1.7149	6.9818	5.1617	0.1233	0.5022	0.2351
118	Co	1.7361	10.4197	5.2296	0.1249	0.7494	0.2382
119	Co	5.2340	6.9615	5.2285	0.3765	0.5007	0.2382
120	Co	5.2191	10.4235	5.1989	0.3754	0.7497	0.2368
121	Co	8.6991	13.8900	5.2241	0.6257	0.9990	0.2380
122	Co	8.6941	3.4897	5.2064	0.6253	0.2510	0.2372
123	Co	12.1660	0.0003	5.2248	0.8750	0.0000	0.2380
124	Co	12.1642	3.4697	5.2205	0.8749	0.2496	0.2378
125	Co	8.6727	6.9567	5.2234	0.6238	0.5004	0.2380
126	Co	8.6808	10.4208	5.2235	0.6244	0.7495	0.2380
127	Co	12.1691	6.9573	5.2257	0.8753	0.5004	0.2381
128	Co	12.1692	10.4236	5.2281	0.8753	0.7497	0.2382
129	O	5.2032	3.3278	7.1758	0.3742	0.2393	0.3269
130	O	2.6376	1.9382	9.2448	0.1897	0.1394	0.4211
131	O	1.8224	4.9908	6.4569	0.1311	0.3590	0.2941
132	O	3.7711	5.1634	8.8775	0.2712	0.3714	0.4044
133	O	4.8365	6.9958	7.0401	0.3479	0.5032	0.3207
134	O	6.7454	7.3041	9.0026	0.4852	0.5253	0.4101
135	O	3.7166	8.7501	8.8397	0.2673	0.6294	0.4027
136	Ce	2.7082	7.0359	8.0537	0.1948	0.5061	0.3669
137	Ce	3.0545	3.2128	7.9385	0.2197	0.2311	0.3616

138 Ce	6.0269	5.3855	8.2382	0.4335	0.3874	0.3753
139 Sm	5.6913	8.8609	8.0469	0.4093	0.6373	0.3666

/db/jmorales/CoNi-alloy/metal-SDC-interface/CoNi-Ce_3SmO_7/CoNi-Ce_3SmO_7-conf6b

a =			13.9033384264				
b =			13.9033384264				
c =			21.9516692133				
alpha =			90.0				
beta =			90.0				
gamma =			90.0				
	Atom	X	Y	Z	X	Y	Z
1	Ni	0.0000	0.0000	0.0000	0.0000	0.0000	0.0000
2	Ni	0.0000	3.4758	0.0000	0.0000	0.2500	0.0000
3	Ni	3.4758	0.0000	0.0000	0.2500	0.0000	0.0000
4	Ni	3.4758	3.4758	0.0000	0.2500	0.2500	0.0000
5	Ni	0.0000	6.9517	0.0000	0.0000	0.5000	0.0000
6	Ni	0.0000	10.4275	0.0000	0.0000	0.7500	0.0000
7	Ni	3.4758	6.9517	0.0000	0.2500	0.5000	0.0000
8	Ni	3.4758	10.4275	0.0000	0.2500	0.7500	0.0000
9	Ni	6.9517	0.0000	0.0000	0.5000	0.0000	0.0000
10	Ni	6.9517	3.4758	0.0000	0.5000	0.2500	0.0000
11	Ni	10.4275	0.0000	0.0000	0.7500	0.0000	0.0000
12	Ni	10.4275	3.4758	0.0000	0.7500	0.2500	0.0000
13	Ni	6.9517	6.9517	0.0000	0.5000	0.5000	0.0000
14	Ni	6.9517	10.4275	0.0000	0.5000	0.7500	0.0000
15	Ni	10.4275	6.9517	0.0000	0.7500	0.5000	0.0000
16	Ni	10.4275	10.4275	0.0000	0.7500	0.7500	0.0000
17	Ni	0.0000	1.7379	1.7379	0.0000	0.1250	0.0792
18	Ni	0.0000	5.2138	1.7379	0.0000	0.3750	0.0792
19	Ni	3.4758	1.7379	1.7379	0.2500	0.1250	0.0792
20	Ni	3.4758	5.2138	1.7379	0.2500	0.3750	0.0792
21	Ni	0.0000	8.6896	1.7379	0.0000	0.6250	0.0792
22	Ni	0.0000	12.1654	1.7379	0.0000	0.8750	0.0792
23	Ni	3.4758	8.6896	1.7379	0.2500	0.6250	0.0792
24	Ni	3.4758	12.1654	1.7379	0.2500	0.8750	0.0792
25	Ni	6.9517	1.7379	1.7379	0.5000	0.1250	0.0792
26	Ni	6.9517	5.2138	1.7379	0.5000	0.3750	0.0792
27	Ni	10.4275	1.7379	1.7379	0.7500	0.1250	0.0792
28	Ni	10.4275	5.2138	1.7379	0.7500	0.3750	0.0792
29	Ni	6.9517	8.6896	1.7379	0.5000	0.6250	0.0792
30	Ni	6.9517	12.1654	1.7379	0.5000	0.8750	0.0792
31	Ni	10.4275	8.6896	1.7379	0.7500	0.6250	0.0792
32	Ni	10.4275	12.1654	1.7379	0.7500	0.8750	0.0792
33	Ni	0.0028	0.0025	3.5037	0.0002	0.0002	0.1596
34	Ni	13.8930	3.4678	3.5076	0.9993	0.2494	0.1598
35	Ni	3.4745	0.0336	3.5376	0.2499	0.0024	0.1612
36	Ni	3.4851	3.4475	3.4422	0.2507	0.2480	0.1568
37	Ni	13.8920	6.9564	3.5026	0.9992	0.5003	0.1596
38	Ni	0.0032	10.4317	3.5054	0.0002	0.7503	0.1597
39	Ni	3.4574	6.9541	3.4876	0.2487	0.5002	0.1589

40	Ni	3.4731	10.4017	3.5351	0.2498	0.7481	0.1610
41	Ni	6.9509	-0.0006	3.5016	0.4999	-0.0000	0.1595
42	Ni	6.9497	3.4728	3.5024	0.4999	0.2498	0.1595
43	Ni	10.4328	0.0005	3.5028	0.7504	0.0000	0.1596
44	Ni	10.4266	3.4832	3.4991	0.7499	0.2505	0.1594
45	Ni	6.9419	6.9448	3.5204	0.4993	0.4995	0.1604
46	Ni	6.9517	10.4221	3.4986	0.5000	0.7496	0.1594
47	Ni	10.4264	6.9494	3.5006	0.7499	0.4998	0.1595
48	Ni	10.4444	10.4454	3.4765	0.7512	0.7513	0.1584
49	Ni	13.9004	1.7338	5.2111	0.9998	0.1247	0.2374
50	Ni	13.8896	5.2184	5.2053	0.9990	0.3753	0.2371
51	Ni	3.4724	1.7452	5.2723	0.2498	0.1255	0.2402
52	Ni	3.4322	5.1936	5.0892	0.2469	0.3735	0.2318
53	Ni	13.8912	8.6917	5.2112	0.9991	0.6252	0.2374
54	Ni	13.8990	12.1696	5.2095	0.9997	0.8753	0.2373
55	Ni	3.4626	8.7353	5.3578	0.2491	0.6283	0.2441
56	Ni	3.4809	12.1617	5.2168	0.2504	0.8747	0.2376
57	Ni	6.9836	1.7119	5.1946	0.5023	0.1231	0.2366
58	Ni	6.9363	5.2205	5.2434	0.4989	0.3755	0.2389
59	Ni	10.4240	1.7314	5.2067	0.7497	0.1245	0.2372
60	Ni	10.4160	5.2117	5.2180	0.7492	0.3749	0.2377
61	Ni	6.9793	8.7462	5.2145	0.5020	0.6291	0.2375
62	Ni	6.9524	12.1615	5.2059	0.5000	0.8747	0.2372
63	Ni	10.4452	8.7287	5.1438	0.7513	0.6278	0.2343
64	Ni	10.4382	12.1688	5.2129	0.7508	0.8752	0.2375
65	Co	1.7379	1.7379	0.0000	0.1250	0.1250	0.0000
66	Co	1.7379	5.2138	0.0000	0.1250	0.3750	0.0000
67	Co	5.2138	1.7379	0.0000	0.3750	0.1250	0.0000
68	Co	5.2138	5.2138	0.0000	0.3750	0.3750	0.0000
69	Co	1.7379	8.6896	0.0000	0.1250	0.6250	0.0000
70	Co	1.7379	12.1654	0.0000	0.1250	0.8750	0.0000
71	Co	5.2138	8.6896	0.0000	0.3750	0.6250	0.0000
72	Co	5.2138	12.1654	0.0000	0.3750	0.8750	0.0000
73	Co	8.6896	1.7379	0.0000	0.6250	0.1250	0.0000
74	Co	8.6896	5.2138	0.0000	0.6250	0.3750	0.0000
75	Co	12.1654	1.7379	0.0000	0.8750	0.1250	0.0000
76	Co	12.1654	5.2138	0.0000	0.8750	0.3750	0.0000
77	Co	8.6896	8.6896	0.0000	0.6250	0.6250	0.0000
78	Co	8.6896	12.1654	0.0000	0.6250	0.8750	0.0000
79	Co	12.1654	8.6896	0.0000	0.8750	0.6250	0.0000
80	Co	12.1654	12.1654	0.0000	0.8750	0.8750	0.0000
81	Co	1.7379	0.0000	1.7379	0.1250	0.0000	0.0792
82	Co	1.7379	3.4758	1.7379	0.1250	0.2500	0.0792
83	Co	5.2138	0.0000	1.7379	0.3750	0.0000	0.0792
84	Co	5.2138	3.4758	1.7379	0.3750	0.2500	0.0792
85	Co	1.7379	6.9517	1.7379	0.1250	0.5000	0.0792
86	Co	1.7379	10.4275	1.7379	0.1250	0.7500	0.0792
87	Co	5.2138	6.9517	1.7379	0.3750	0.5000	0.0792
88	Co	5.2138	10.4275	1.7379	0.3750	0.7500	0.0792

89	Co	8.6896	0.0000	1.7379	0.6250	0.0000	0.0792
90	Co	8.6896	3.4758	1.7379	0.6250	0.2500	0.0792
91	Co	12.1654	0.0000	1.7379	0.8750	0.0000	0.0792
92	Co	12.1654	3.4758	1.7379	0.8750	0.2500	0.0792
93	Co	8.6896	6.9517	1.7379	0.6250	0.5000	0.0792
94	Co	8.6896	10.4275	1.7379	0.6250	0.7500	0.0792
95	Co	12.1654	6.9517	1.7379	0.8750	0.5000	0.0792
96	Co	12.1654	10.4275	1.7379	0.8750	0.7500	0.0792
97	Co	1.7370	1.7516	3.5631	0.1249	0.1260	0.1623
98	Co	1.6958	5.1959	3.5016	0.1220	0.3737	0.1595
99	Co	5.2151	1.7477	3.5723	0.3751	0.1257	0.1627
100	Co	5.2244	5.2076	3.5145	0.3758	0.3746	0.1601
101	Co	1.7504	8.6933	3.5743	0.1259	0.6253	0.1628
102	Co	1.7443	12.1685	3.5557	0.1255	0.8752	0.1620
103	Co	5.2120	8.7102	3.5411	0.3749	0.6265	0.1613
104	Co	5.2064	12.1829	3.5351	0.3745	0.8763	0.1610
105	Co	8.6943	1.7319	3.5406	0.6253	0.1246	0.1613
106	Co	8.6733	5.2316	3.5632	0.6238	0.3763	0.1623
107	Co	12.1632	1.7367	3.5568	0.8748	0.1249	0.1620
108	Co	12.1605	5.2173	3.5591	0.8746	0.3753	0.1621
109	Co	8.6996	8.7025	3.4919	0.6257	0.6259	0.1591
110	Co	8.6904	12.1847	3.5216	0.6251	0.8764	0.1604
111	Co	12.1842	8.6937	3.5232	0.8764	0.6253	0.1605
112	Co	12.1707	12.1721	3.5469	0.8754	0.8755	0.1616
113	Co	1.7285	13.8973	5.2264	0.1243	0.9996	0.2381
114	Co	1.7209	3.4810	5.2811	0.1238	0.2504	0.2406
115	Co	5.2271	13.8962	5.2280	0.3760	0.9995	0.2382
116	Co	5.2306	3.4656	5.3183	0.3762	0.2493	0.2423
117	Co	1.6932	6.9323	5.1825	0.1218	0.4986	0.2361
118	Co	1.7162	10.4417	5.2327	0.1234	0.7510	0.2384
119	Co	5.1526	6.9764	5.1820	0.3706	0.5018	0.2361
120	Co	5.2402	10.4525	5.2126	0.3769	0.7518	0.2375
121	Co	8.6925	13.8844	5.2258	0.6252	0.9986	0.2381
122	Co	8.7210	3.4709	5.1944	0.6273	0.2496	0.2366
123	Co	12.1668	0.0012	5.2302	0.8751	0.0001	0.2383
124	Co	12.1603	3.4807	5.2253	0.8746	0.2503	0.2380
125	Co	8.7435	6.9681	5.2374	0.6289	0.5012	0.2386
126	Co	8.7285	10.4727	5.1548	0.6278	0.7533	0.2348
127	Co	12.1415	6.9692	5.2327	0.8733	0.5013	0.2384
128	Co	12.1586	10.4281	5.2288	0.8745	0.7500	0.2382
129	O	3.3711	3.6168	6.3815	0.2425	0.2601	0.2907
130	O	6.2651	4.4003	6.9094	0.4506	0.3165	0.3148
131	O	4.1885	7.5592	6.8246	0.3013	0.5437	0.3109
132	O	6.0157	6.6184	8.7408	0.4327	0.4760	0.3982
133	O	7.7879	7.7877	6.8007	0.5601	0.5601	0.3098
134	O	9.5573	7.1362	8.8600	0.6874	0.5133	0.4036
135	O	7.4876	9.7379	8.7387	0.5385	0.7004	0.3981
136	Ce	5.9043	8.7405	7.8651	0.4247	0.6287	0.3583
137	Ce	4.4561	5.3979	7.6140	0.3205	0.3882	0.3469

138 Ce	8.1249	5.7431	8.0242	0.5844	0.4131	0.3655
139 Sm	9.3801	9.0040	7.8959	0.6747	0.6476	0.3597

/db/jmorales/CoNi-alloy/metal-SDC-interface/Ni-Ce_3SmO_7/Ni-Ce_3SmO_7-conf3c

a =			13.9019837372				
b =			13.9019837372				
c =			21.9509918687				
alpha =			90.0				
beta =			90.0				
gamma =			90.0				
	Atom	X	Y	Z	X	Y	Z
1	Ni	0.0000	0.0000	0.0000	0.0000	0.0000	0.0000
2	Ni	1.7377	1.7377	0.0000	0.1250	0.1250	0.0000
3	Ni	0.0000	3.4755	0.0000	0.0000	0.2500	0.0000
4	Ni	1.7377	5.2132	0.0000	0.1250	0.3750	0.0000
5	Ni	3.4755	0.0000	0.0000	0.2500	0.0000	0.0000
6	Ni	5.2132	1.7377	0.0000	0.3750	0.1250	0.0000
7	Ni	3.4755	3.4755	0.0000	0.2500	0.2500	0.0000
8	Ni	5.2132	5.2132	0.0000	0.3750	0.3750	0.0000
9	Ni	0.0000	6.9510	0.0000	0.0000	0.5000	0.0000
10	Ni	1.7377	8.6887	0.0000	0.1250	0.6250	0.0000
11	Ni	0.0000	10.4265	0.0000	0.0000	0.7500	0.0000
12	Ni	1.7377	12.1642	0.0000	0.1250	0.8750	0.0000
13	Ni	3.4755	6.9510	0.0000	0.2500	0.5000	0.0000
14	Ni	5.2132	8.6887	0.0000	0.3750	0.6250	0.0000
15	Ni	3.4755	10.4265	0.0000	0.2500	0.7500	0.0000
16	Ni	5.2132	12.1642	0.0000	0.3750	0.8750	0.0000
17	Ni	6.9510	0.0000	0.0000	0.5000	0.0000	0.0000
18	Ni	8.6887	1.7377	0.0000	0.6250	0.1250	0.0000
19	Ni	6.9510	3.4755	0.0000	0.5000	0.2500	0.0000
20	Ni	8.6887	5.2132	0.0000	0.6250	0.3750	0.0000
21	Ni	10.4265	0.0000	0.0000	0.7500	0.0000	0.0000
22	Ni	12.1642	1.7377	0.0000	0.8750	0.1250	0.0000
23	Ni	10.4265	3.4755	0.0000	0.7500	0.2500	0.0000
24	Ni	12.1642	5.2132	0.0000	0.8750	0.3750	0.0000
25	Ni	6.9510	6.9510	0.0000	0.5000	0.5000	0.0000
26	Ni	8.6887	8.6887	0.0000	0.6250	0.6250	0.0000
27	Ni	6.9510	10.4265	0.0000	0.5000	0.7500	0.0000
28	Ni	8.6887	12.1642	0.0000	0.6250	0.8750	0.0000
29	Ni	10.4265	6.9510	0.0000	0.7500	0.5000	0.0000
30	Ni	12.1642	8.6887	0.0000	0.8750	0.6250	0.0000
31	Ni	10.4265	10.4265	0.0000	0.7500	0.7500	0.0000
32	Ni	12.1642	12.1642	0.0000	0.8750	0.8750	0.0000
33	Ni	0.0000	1.7377	1.7377	0.0000	0.1250	0.0792
34	Ni	1.7377	0.0000	1.7377	0.1250	0.0000	0.0792
35	Ni	0.0000	5.2132	1.7377	0.0000	0.3750	0.0792
36	Ni	1.7377	3.4755	1.7377	0.1250	0.2500	0.0792
37	Ni	3.4755	1.7377	1.7377	0.2500	0.1250	0.0792
38	Ni	5.2132	0.0000	1.7377	0.3750	0.0000	0.0792
39	Ni	3.4755	5.2132	1.7377	0.2500	0.3750	0.0792
40	Ni	5.2132	3.4755	1.7377	0.3750	0.2500	0.0792

41	Ni	0.0000	8.6887	1.7377	0.0000	0.6250	0.0792
42	Ni	1.7377	6.9510	1.7377	0.1250	0.5000	0.0792
43	Ni	0.0000	12.1642	1.7377	0.0000	0.8750	0.0792
44	Ni	1.7377	10.4265	1.7377	0.1250	0.7500	0.0792
45	Ni	3.4755	8.6887	1.7377	0.2500	0.6250	0.0792
46	Ni	5.2132	6.9510	1.7377	0.3750	0.5000	0.0792
47	Ni	3.4755	12.1642	1.7377	0.2500	0.8750	0.0792
48	Ni	5.2132	10.4265	1.7377	0.3750	0.7500	0.0792
49	Ni	6.9510	1.7377	1.7377	0.5000	0.1250	0.0792
50	Ni	8.6887	0.0000	1.7377	0.6250	0.0000	0.0792
51	Ni	6.9510	5.2132	1.7377	0.5000	0.3750	0.0792
52	Ni	8.6887	3.4755	1.7377	0.6250	0.2500	0.0792
53	Ni	10.4265	1.7377	1.7377	0.7500	0.1250	0.0792
54	Ni	12.1642	0.0000	1.7377	0.8750	0.0000	0.0792
55	Ni	10.4265	5.2132	1.7377	0.7500	0.3750	0.0792
56	Ni	12.1642	3.4755	1.7377	0.8750	0.2500	0.0792
57	Ni	6.9510	8.6887	1.7377	0.5000	0.6250	0.0792
58	Ni	8.6887	6.9510	1.7377	0.6250	0.5000	0.0792
59	Ni	6.9510	12.1642	1.7377	0.5000	0.8750	0.0792
60	Ni	8.6887	10.4265	1.7377	0.6250	0.7500	0.0792
61	Ni	10.4265	8.6887	1.7377	0.7500	0.6250	0.0792
62	Ni	12.1642	6.9510	1.7377	0.8750	0.5000	0.0792
63	Ni	10.4265	12.1642	1.7377	0.7500	0.8750	0.0792
64	Ni	12.1642	10.4265	1.7377	0.8750	0.7500	0.0792
65	Ni	0.0015	13.8995	3.5093	0.0001	0.9998	0.1599
66	Ni	1.7343	1.7468	3.5038	0.1247	0.1256	0.1596
67	Ni	13.8803	3.4794	3.5322	0.9984	0.2503	0.1609
68	Ni	1.7587	5.2014	3.5426	0.1265	0.3741	0.1614
69	Ni	3.4684	13.8963	3.5030	0.2495	0.9996	0.1596
70	Ni	5.2198	1.7291	3.5021	0.3755	0.1244	0.1595
71	Ni	3.4956	3.4965	3.5391	0.2514	0.2515	0.1612
72	Ni	5.2052	5.1965	3.5073	0.3744	0.3738	0.1598
73	Ni	0.0051	6.9480	3.5118	0.0004	0.4998	0.1600
74	Ni	1.7344	8.6895	3.5087	0.1248	0.6251	0.1598
75	Ni	13.8985	10.4262	3.5113	0.9998	0.7500	0.1600
76	Ni	1.7303	12.1649	3.5119	0.1245	0.8750	0.1600
77	Ni	3.4474	6.9290	3.5038	0.2480	0.4984	0.1596
78	Ni	5.2008	8.7230	3.4771	0.3741	0.6275	0.1584
79	Ni	3.4637	10.4355	3.5069	0.2491	0.7507	0.1598
80	Ni	5.2118	12.1616	3.5089	0.3749	0.8748	0.1599
81	Ni	6.9456	0.0024	3.5233	0.4996	0.0002	0.1605
82	Ni	8.6761	1.7063	3.4881	0.6241	0.1227	0.1589
83	Ni	6.9695	3.4781	3.4856	0.5013	0.2502	0.1588
84	Ni	8.6946	5.2324	3.4673	0.6254	0.3764	0.1580
85	Ni	10.4334	0.0113	3.5256	0.7505	0.0008	0.1606
86	Ni	12.1669	1.7339	3.5124	0.8752	0.1247	0.1600
87	Ni	10.4531	3.4689	3.4266	0.7519	0.2495	0.1561
88	Ni	12.1671	5.2178	3.5083	0.8752	0.3753	0.1598
89	Ni	6.9514	6.9700	3.4675	0.5000	0.5014	0.1580

90	Ni	8.6926	8.6860	3.5091	0.6253	0.6248	0.1599
91	Ni	6.9571	10.4389	3.5042	0.5004	0.7509	0.1596
92	Ni	8.6933	12.1603	3.5088	0.6253	0.8747	0.1598
93	Ni	10.4204	6.9398	3.5295	0.7496	0.4992	0.1608
94	Ni	12.1632	8.6859	3.5091	0.8749	0.6248	0.1599
95	Ni	10.4280	10.4250	3.5094	0.7501	0.7499	0.1599
96	Ni	12.1636	12.1610	3.5110	0.8750	0.8748	0.1599
97	Ni	0.0031	1.7334	5.2259	0.0002	0.1247	0.2381
98	Ni	1.7392	0.0065	5.2291	0.1251	0.0005	0.2382
99	Ni	13.8973	5.2149	5.2264	0.9997	0.3751	0.2381
100	Ni	1.7357	3.4598	5.2285	0.1249	0.2489	0.2382
101	Ni	3.4628	1.7358	5.2043	0.2491	0.1249	0.2371
102	Ni	5.1888	13.8827	5.2157	0.3732	0.9986	0.2376
103	Ni	3.5123	5.2307	5.4596	0.2526	0.3763	0.2487
104	Ni	5.2729	3.4431	5.2353	0.3793	0.2477	0.2385
105	Ni	13.8981	8.6887	5.2272	0.9997	0.6250	0.2381
106	Ni	1.7277	6.9620	5.2276	0.1243	0.5008	0.2381
107	Ni	13.9008	12.1650	5.2291	0.9999	0.8751	0.2382
108	Ni	1.7337	10.4242	5.2276	0.1247	0.7498	0.2381
109	Ni	3.4754	8.6927	5.2127	0.2500	0.6253	0.2375
110	Ni	5.1951	7.0009	5.0855	0.3737	0.5036	0.2317
111	Ni	3.4655	12.1639	5.2262	0.2493	0.8750	0.2381
112	Ni	5.2013	10.4295	5.2295	0.3741	0.7502	0.2382
113	Ni	7.0049	1.6715	5.3442	0.5039	0.1202	0.2435
114	Ni	8.7005	13.8695	5.2211	0.6258	0.9977	0.2379
115	Ni	6.9271	5.2464	5.2087	0.4983	0.3774	0.2373
116	Ni	8.7131	3.4707	5.1010	0.6268	0.2497	0.2324
117	Ni	10.4087	1.7314	5.2893	0.7487	0.1245	0.2410
118	Ni	12.1676	13.9011	5.2247	0.8752	0.9999	0.2380
119	Ni	10.4426	5.2029	5.2584	0.7512	0.3743	0.2396
120	Ni	12.1494	3.4656	5.2972	0.8739	0.2493	0.2413
121	Ni	6.9231	8.7215	5.1923	0.4980	0.6274	0.2365
122	Ni	8.6920	6.9848	5.2545	0.6252	0.5024	0.2394
123	Ni	6.9504	12.1594	5.2284	0.5000	0.8747	0.2382
124	Ni	8.6972	10.4164	5.2351	0.6256	0.7493	0.2385
125	Ni	10.4252	8.6856	5.2312	0.7499	0.6248	0.2383
126	Ni	12.1603	6.9585	5.2317	0.8747	0.5005	0.2383
127	Ni	10.4359	12.1564	5.2287	0.7507	0.8744	0.2382
128	Ni	12.1624	10.4173	5.2337	0.8749	0.7493	0.2384
129	O	6.2838	2.6862	6.7540	0.4520	0.1932	0.3077
130	O	3.7521	5.3181	7.3275	0.2699	0.3825	0.3338
131	O	6.1399	4.6636	8.8069	0.4417	0.3355	0.4012
132	O	10.2998	3.4771	6.2631	0.7409	0.2501	0.2853
133	O	7.7460	6.1094	6.7658	0.5572	0.4395	0.3082
134	O	9.4825	5.6297	8.9193	0.6821	0.4050	0.4063
135	O	7.2273	8.0428	8.8240	0.5199	0.5785	0.4020
136	Ce	5.6758	6.7948	7.9071	0.4083	0.4888	0.3602
137	Ce	4.4931	3.3817	7.9954	0.3232	0.2433	0.3642
138	Ce	8.3000	4.1103	7.9465	0.5970	0.2957	0.3620

139 Sm	9.1189	7.5349	8.0776	0.6559	0.5420	0.3680

/db/jmorales/CoNi-alloy/metal-SDC-interface/Ni-Ce_3SmO_7/Ni-Ce_3SmO_7-conf6c

a = 13.9019837372
b = 13.9019837372
c = 21.9509918687
alpha = 90.0
beta = 90.0
gamma = 90.0

	Atom	X	Y	Z	X	Y	Z
1	Ni	0.0000	0.0000	0.0000	0.0000	0.0000	0.0000
2	Ni	1.7377	1.7377	0.0000	0.1250	0.1250	0.0000
3	Ni	0.0000	3.4755	0.0000	0.0000	0.2500	0.0000
4	Ni	1.7377	5.2132	0.0000	0.1250	0.3750	0.0000
5	Ni	3.4755	0.0000	0.0000	0.2500	0.0000	0.0000
6	Ni	5.2132	1.7377	0.0000	0.3750	0.1250	0.0000
7	Ni	3.4755	3.4755	0.0000	0.2500	0.2500	0.0000
8	Ni	5.2132	5.2132	0.0000	0.3750	0.3750	0.0000
9	Ni	0.0000	6.9510	0.0000	0.0000	0.5000	0.0000
10	Ni	1.7377	8.6887	0.0000	0.1250	0.6250	0.0000
11	Ni	0.0000	10.4265	0.0000	0.0000	0.7500	0.0000
12	Ni	1.7377	12.1642	0.0000	0.1250	0.8750	0.0000
13	Ni	3.4755	6.9510	0.0000	0.2500	0.5000	0.0000
14	Ni	5.2132	8.6887	0.0000	0.3750	0.6250	0.0000
15	Ni	3.4755	10.4265	0.0000	0.2500	0.7500	0.0000
16	Ni	5.2132	12.1642	0.0000	0.3750	0.8750	0.0000
17	Ni	6.9510	0.0000	0.0000	0.5000	0.0000	0.0000
18	Ni	8.6887	1.7377	0.0000	0.6250	0.1250	0.0000
19	Ni	6.9510	3.4755	0.0000	0.5000	0.2500	0.0000
20	Ni	8.6887	5.2132	0.0000	0.6250	0.3750	0.0000
21	Ni	10.4265	0.0000	0.0000	0.7500	0.0000	0.0000
22	Ni	12.1642	1.7377	0.0000	0.8750	0.1250	0.0000
23	Ni	10.4265	3.4755	0.0000	0.7500	0.2500	0.0000
24	Ni	12.1642	5.2132	0.0000	0.8750	0.3750	0.0000
25	Ni	6.9510	6.9510	0.0000	0.5000	0.5000	0.0000
26	Ni	8.6887	8.6887	0.0000	0.6250	0.6250	0.0000
27	Ni	6.9510	10.4265	0.0000	0.5000	0.7500	0.0000
28	Ni	8.6887	12.1642	0.0000	0.6250	0.8750	0.0000
29	Ni	10.4265	6.9510	0.0000	0.7500	0.5000	0.0000
30	Ni	12.1642	8.6887	0.0000	0.8750	0.6250	0.0000
31	Ni	10.4265	10.4265	0.0000	0.7500	0.7500	0.0000
32	Ni	12.1642	12.1642	0.0000	0.8750	0.8750	0.0000
33	Ni	0.0000	1.7377	1.7377	0.0000	0.1250	0.0792
34	Ni	1.7377	0.0000	1.7377	0.1250	0.0000	0.0792
35	Ni	0.0000	5.2132	1.7377	0.0000	0.3750	0.0792
36	Ni	1.7377	3.4755	1.7377	0.1250	0.2500	0.0792
37	Ni	3.4755	1.7377	1.7377	0.2500	0.1250	0.0792
38	Ni	5.2132	0.0000	1.7377	0.3750	0.0000	0.0792
39	Ni	3.4755	5.2132	1.7377	0.2500	0.3750	0.0792
40	Ni	5.2132	3.4755	1.7377	0.3750	0.2500	0.0792

41	Ni	0.0000	8.6887	1.7377	0.0000	0.6250	0.0792
42	Ni	1.7377	6.9510	1.7377	0.1250	0.5000	0.0792
43	Ni	0.0000	12.1642	1.7377	0.0000	0.8750	0.0792
44	Ni	1.7377	10.4265	1.7377	0.1250	0.7500	0.0792
45	Ni	3.4755	8.6887	1.7377	0.2500	0.6250	0.0792
46	Ni	5.2132	6.9510	1.7377	0.3750	0.5000	0.0792
47	Ni	3.4755	12.1642	1.7377	0.2500	0.8750	0.0792
48	Ni	5.2132	10.4265	1.7377	0.3750	0.7500	0.0792
49	Ni	6.9510	1.7377	1.7377	0.5000	0.1250	0.0792
50	Ni	8.6887	0.0000	1.7377	0.6250	0.0000	0.0792
51	Ni	6.9510	5.2132	1.7377	0.5000	0.3750	0.0792
52	Ni	8.6887	3.4755	1.7377	0.6250	0.2500	0.0792
53	Ni	10.4265	1.7377	1.7377	0.7500	0.1250	0.0792
54	Ni	12.1642	0.0000	1.7377	0.8750	0.0000	0.0792
55	Ni	10.4265	5.2132	1.7377	0.7500	0.3750	0.0792
56	Ni	12.1642	3.4755	1.7377	0.8750	0.2500	0.0792
57	Ni	6.9510	8.6887	1.7377	0.5000	0.6250	0.0792
58	Ni	8.6887	6.9510	1.7377	0.6250	0.5000	0.0792
59	Ni	6.9510	12.1642	1.7377	0.5000	0.8750	0.0792
60	Ni	8.6887	10.4265	1.7377	0.6250	0.7500	0.0792
61	Ni	10.4265	8.6887	1.7377	0.7500	0.6250	0.0792
62	Ni	12.1642	6.9510	1.7377	0.8750	0.5000	0.0792
63	Ni	10.4265	12.1642	1.7377	0.7500	0.8750	0.0792
64	Ni	12.1642	10.4265	1.7377	0.8750	0.7500	0.0792
65	Ni	13.8967	13.8957	3.5017	0.9996	0.9996	0.1595
66	Ni	1.7494	1.7291	3.5175	0.1258	0.1244	0.1602
67	Ni	0.0009	3.4758	3.5074	0.0001	0.2500	0.1598
68	Ni	1.7551	5.2081	3.5396	0.1262	0.3746	0.1613
69	Ni	3.4852	0.0014	3.5170	0.2507	0.0001	0.1602
70	Ni	5.2226	1.7466	3.5182	0.3757	0.1256	0.1603
71	Ni	3.4603	3.4657	3.5077	0.2489	0.2493	0.1598
72	Ni	5.2088	5.2324	3.4696	0.3747	0.3764	0.1581
73	Ni	0.0001	6.9454	3.5164	0.0000	0.4996	0.1602
74	Ni	1.7317	8.6878	3.5089	0.1246	0.6249	0.1599
75	Ni	13.8977	10.4193	3.5095	0.9997	0.7495	0.1599
76	Ni	1.7322	12.1583	3.5050	0.1246	0.8746	0.1597
77	Ni	3.4435	6.9351	3.5040	0.2477	0.4989	0.1596
78	Ni	5.2003	8.7185	3.4721	0.3741	0.6271	0.1582
79	Ni	3.4704	10.4315	3.5056	0.2496	0.7504	0.1597
80	Ni	5.2148	12.1867	3.5346	0.3751	0.8766	0.1610
81	Ni	6.9349	0.0114	3.5356	0.4988	0.0008	0.1611
82	Ni	8.6974	1.7232	3.4902	0.6256	0.1240	0.1590
83	Ni	6.9590	3.4921	3.5013	0.5006	0.2512	0.1595
84	Ni	8.7007	5.1914	3.4969	0.6259	0.3734	0.1593
85	Ni	10.4371	13.8864	3.5038	0.7508	0.9989	0.1596
86	Ni	12.1711	1.7341	3.5014	0.8755	0.1247	0.1595
87	Ni	10.4188	3.4631	3.5118	0.7494	0.2491	0.1600
88	Ni	12.1717	5.2140	3.5035	0.8755	0.3751	0.1596
89	Ni	6.9686	6.9655	3.4706	0.5013	0.5010	0.1581

90	Ni	8.6944	8.6887	3.5027	0.6254	0.6250	0.1596
91	Ni	6.9525	10.4390	3.5018	0.5001	0.7509	0.1595
92	Ni	8.6899	12.1586	3.5059	0.6251	0.8746	0.1597
93	Ni	10.4197	6.9505	3.5096	0.7495	0.5000	0.1599
94	Ni	12.1683	8.6822	3.5087	0.8753	0.6245	0.1598
95	Ni	10.4288	10.4258	3.5073	0.7502	0.7499	0.1598
96	Ni	12.1645	12.1526	3.5096	0.8750	0.8742	0.1599
97	Ni	13.8953	1.7335	5.2197	0.9995	0.1247	0.2378
98	Ni	1.7014	13.8814	5.2078	0.1224	0.9985	0.2372
99	Ni	13.8981	5.2151	5.2314	0.9997	0.3751	0.2383
100	Ni	1.7107	3.4582	5.2146	0.1231	0.2488	0.2376
101	Ni	3.4395	1.7166	5.3412	0.2474	0.1235	0.2433
102	Ni	5.2427	13.8833	5.3904	0.3771	0.9987	0.2456
103	Ni	3.4634	5.2424	5.3962	0.2491	0.3771	0.2458
104	Ni	5.2155	3.5448	5.1722	0.3752	0.2550	0.2356
105	Ni	13.8967	8.6837	5.2305	0.9996	0.6246	0.2383
106	Ni	1.7347	6.9573	5.2378	0.1248	0.5005	0.2386
107	Ni	13.8970	12.1510	5.2246	0.9996	0.8740	0.2380
108	Ni	1.7293	10.4159	5.2257	0.1244	0.7492	0.2381
109	Ni	3.4758	8.6865	5.2163	0.2500	0.6248	0.2376
110	Ni	5.1912	7.0017	5.0943	0.3734	0.5036	0.2321
111	Ni	3.4554	12.1377	5.2125	0.2486	0.8731	0.2375
112	Ni	5.2096	10.4265	5.2246	0.3747	0.7500	0.2380
113	Ni	6.9994	1.7276	5.2711	0.5035	0.1243	0.2401
114	Ni	8.7129	13.8671	5.2017	0.6267	0.9975	0.2370
115	Ni	6.9514	5.2696	5.2188	0.5000	0.3791	0.2377
116	Ni	8.7408	3.4406	5.2967	0.6287	0.2475	0.2413
117	Ni	10.4669	1.7097	5.2043	0.7529	0.1230	0.2371
118	Ni	12.1716	13.8860	5.2253	0.8755	0.9988	0.2380
119	Ni	10.4479	5.1867	5.1917	0.7515	0.3731	0.2365
120	Ni	12.1643	3.4745	5.2264	0.8750	0.2499	0.2381
121	Ni	6.9310	8.7156	5.1773	0.4986	0.6269	0.2359
122	Ni	8.7401	6.9676	5.2499	0.6287	0.5012	0.2392
123	Ni	6.9654	12.1493	5.2303	0.5010	0.8739	0.2383
124	Ni	8.6937	10.4028	5.2353	0.6254	0.7483	0.2385
125	Ni	10.4302	8.6988	5.2151	0.7503	0.6257	0.2376
126	Ni	12.1460	6.9505	5.2366	0.8737	0.5000	0.2386
127	Ni	10.4316	12.1424	5.2330	0.7504	0.8734	0.2384
128	Ni	12.1622	10.4160	5.2344	0.8749	0.7492	0.2385
129	O	4.4289	0.9719	6.7237	0.3186	0.0699	0.3063
130	O	7.7533	2.6615	6.7932	0.5577	0.1914	0.3095
131	O	4.5054	4.3772	6.7932	0.3241	0.3149	0.3095
132	O	6.6654	4.5235	8.8397	0.4795	0.3254	0.4027
133	O	7.7366	6.2407	6.7688	0.5565	0.4489	0.3084
134	O	9.6423	6.4925	8.8468	0.6936	0.4670	0.4030
135	O	6.7545	8.0907	8.7227	0.4859	0.5820	0.3974
136	Ce	5.6261	6.4121	7.9337	0.4047	0.4612	0.3614
137	Ce	5.6728	2.6667	7.8211	0.4081	0.1918	0.3563
138	Ce	8.8643	4.6318	8.0761	0.6376	0.3332	0.3679

139 Sm	8.7032	8.1495	7.9233	0.6260	0.5862	0.3610

/db/jmorales/CoNi-alloy/metal-SDC-interface/Ni-Ce_3SmO_7/Ni-Ce_3SmO_7-conf-CoNi-b

a = 13.901983737
b = 13.901983737
c = 21.950991869
alpha = 90.0
beta = 90.0
gamma = 90.0

	Atom	X	Y	Z	X	Y	Z
1	Ni	0.0000	0.0000	0.0000	0.0000	0.0000	0.0000
2	Ni	1.7378	1.7378	0.0000	0.1250	0.1250	0.0000
3	Ni	0.0000	3.4755	0.0000	0.0000	0.2500	0.0000
4	Ni	1.7378	5.2132	0.0000	0.1250	0.3750	0.0000
5	Ni	3.4755	0.0000	0.0000	0.2500	0.0000	0.0000
6	Ni	5.2132	1.7378	0.0000	0.3750	0.1250	0.0000
7	Ni	3.4755	3.4755	0.0000	0.2500	0.2500	0.0000
8	Ni	5.2132	5.2132	0.0000	0.3750	0.3750	0.0000
9	Ni	0.0000	6.9510	0.0000	0.0000	0.5000	0.0000
10	Ni	1.7378	8.6887	0.0000	0.1250	0.6250	0.0000
11	Ni	0.0000	10.4265	0.0000	0.0000	0.7500	0.0000
12	Ni	1.7378	12.1642	0.0000	0.1250	0.8750	0.0000
13	Ni	3.4755	6.9510	0.0000	0.2500	0.5000	0.0000
14	Ni	5.2132	8.6887	0.0000	0.3750	0.6250	0.0000
15	Ni	3.4755	10.4265	0.0000	0.2500	0.7500	0.0000
16	Ni	5.2132	12.1642	0.0000	0.3750	0.8750	0.0000
17	Ni	6.9510	0.0000	0.0000	0.5000	0.0000	0.0000
18	Ni	8.6887	1.7378	0.0000	0.6250	0.1250	0.0000
19	Ni	6.9510	3.4755	0.0000	0.5000	0.2500	0.0000
20	Ni	8.6887	5.2132	0.0000	0.6250	0.3750	0.0000
21	Ni	10.4265	0.0000	0.0000	0.7500	0.0000	0.0000
22	Ni	12.1642	1.7378	0.0000	0.8750	0.1250	0.0000
23	Ni	10.4265	3.4755	0.0000	0.7500	0.2500	0.0000
24	Ni	12.1642	5.2132	0.0000	0.8750	0.3750	0.0000
25	Ni	6.9510	6.9510	0.0000	0.5000	0.5000	0.0000
26	Ni	8.6887	8.6887	0.0000	0.6250	0.6250	0.0000
27	Ni	6.9510	10.4265	0.0000	0.5000	0.7500	0.0000
28	Ni	8.6887	12.1642	0.0000	0.6250	0.8750	0.0000
29	Ni	10.4265	6.9510	0.0000	0.7500	0.5000	0.0000
30	Ni	12.1642	8.6887	0.0000	0.8750	0.6250	0.0000
31	Ni	10.4265	10.4265	0.0000	0.7500	0.7500	0.0000
32	Ni	12.1642	12.1642	0.0000	0.8750	0.8750	0.0000
33	Ni	0.0000	1.7378	1.7378	0.0000	0.1250	0.0792
34	Ni	1.7378	0.0000	1.7378	0.1250	0.0000	0.0792
35	Ni	0.0000	5.2132	1.7378	0.0000	0.3750	0.0792
36	Ni	1.7378	3.4755	1.7378	0.1250	0.2500	0.0792
37	Ni	3.4755	1.7378	1.7378	0.2500	0.1250	0.0792
38	Ni	5.2132	0.0000	1.7378	0.3750	0.0000	0.0792
39	Ni	3.4755	5.2132	1.7378	0.2500	0.3750	0.0792

40	Ni	5.2132	3.4755	1.7378	0.3750	0.2500	0.0792
41	Ni	0.0000	8.6887	1.7378	0.0000	0.6250	0.0792
42	Ni	1.7378	6.9510	1.7378	0.1250	0.5000	0.0792
43	Ni	0.0000	12.1642	1.7378	0.0000	0.8750	0.0792
44	Ni	1.7378	10.4265	1.7378	0.1250	0.7500	0.0792
45	Ni	3.4755	8.6887	1.7378	0.2500	0.6250	0.0792
46	Ni	5.2132	6.9510	1.7378	0.3750	0.5000	0.0792
47	Ni	3.4755	12.1642	1.7378	0.2500	0.8750	0.0792
48	Ni	5.2132	10.4265	1.7378	0.3750	0.7500	0.0792
49	Ni	6.9510	1.7378	1.7378	0.5000	0.1250	0.0792
50	Ni	8.6887	0.0000	1.7378	0.6250	0.0000	0.0792
51	Ni	6.9510	5.2132	1.7378	0.5000	0.3750	0.0792
52	Ni	8.6887	3.4755	1.7378	0.6250	0.2500	0.0792
53	Ni	10.4265	1.7378	1.7378	0.7500	0.1250	0.0792
54	Ni	12.1642	0.0000	1.7378	0.8750	0.0000	0.0792
55	Ni	10.4265	5.2132	1.7378	0.7500	0.3750	0.0792
56	Ni	12.1642	3.4755	1.7378	0.8750	0.2500	0.0792
57	Ni	6.9510	8.6887	1.7378	0.5000	0.6250	0.0792
58	Ni	8.6887	6.9510	1.7378	0.6250	0.5000	0.0792
59	Ni	6.9510	12.1642	1.7378	0.5000	0.8750	0.0792
60	Ni	8.6887	10.4265	1.7378	0.6250	0.7500	0.0792
61	Ni	10.4265	8.6887	1.7378	0.7500	0.6250	0.0792
62	Ni	12.1642	6.9510	1.7378	0.8750	0.5000	0.0792
63	Ni	10.4265	12.1642	1.7378	0.7500	0.8750	0.0792
64	Ni	12.1642	10.4265	1.7378	0.8750	0.7500	0.0792
65	Ni	13.8991	13.8997	3.5092	0.9998	0.9998	0.1599
66	Ni	1.7273	1.7292	3.5094	0.1242	0.1244	0.1599
67	Ni	13.8996	3.4738	3.5091	0.9998	0.2499	0.1599
68	Ni	1.7034	5.2312	3.5006	0.1225	0.3763	0.1595
69	Ni	3.4738	13.9001	3.5083	0.2499	0.9999	0.1598
70	Ni	5.2109	1.7356	3.5031	0.3748	0.1248	0.1596
71	Ni	3.4617	3.4439	3.4747	0.2490	0.2477	0.1583
72	Ni	5.2378	5.2003	3.4848	0.3768	0.3741	0.1588
73	Ni	0.0133	6.9598	3.5360	0.0010	0.5006	0.1611
74	Ni	1.7302	8.6852	3.5081	0.1245	0.6247	0.1598
75	Ni	13.8933	10.4354	3.5033	0.9994	0.7506	0.1596
76	Ni	1.7400	12.1662	3.5044	0.1252	0.8751	0.1596
77	Ni	3.4699	6.9520	3.4936	0.2496	0.5001	0.1592
78	Ni	5.2023	8.6675	3.5147	0.3742	0.6235	0.1601
79	Ni	3.4924	10.4063	3.5242	0.2512	0.7485	0.1605
80	Ni	5.2191	12.1548	3.5199	0.3754	0.8743	0.1604
81	Ni	6.9488	0.0199	3.4903	0.4998	0.0014	0.1590
82	Ni	8.6922	1.7238	3.5263	0.6252	0.1240	0.1606
83	Ni	6.9557	3.4822	3.5233	0.5003	0.2505	0.1605
84	Ni	8.6660	5.2083	3.5330	0.6234	0.3746	0.1610
85	Ni	10.4297	0.0074	3.5090	0.7502	0.0005	0.1599
86	Ni	12.1678	1.7418	3.5081	0.8753	0.1253	0.1598
87	Ni	10.4259	3.4804	3.5092	0.7500	0.2504	0.1599
88	Ni	12.1672	5.2174	3.5138	0.8752	0.3753	0.1601

89	Ni	6.9640	6.9588	3.5049	0.5009	0.5006	0.1597
90	Ni	8.6869	8.7139	3.5155	0.6249	0.6268	0.1602
91	Ni	6.9468	10.4309	3.4742	0.4997	0.7503	0.1583
92	Ni	8.7047	12.1783	3.4352	0.6262	0.8760	0.1565
93	Ni	10.4293	6.9481	3.5044	0.7502	0.4998	0.1596
94	Ni	12.1634	8.6860	3.5065	0.8749	0.6248	0.1597
95	Ni	10.4390	10.4327	3.4984	0.7509	0.7504	0.1594
96	Ni	12.1433	12.1740	3.5296	0.8735	0.8757	0.1608
97	Ni	13.9007	1.7406	5.2292	0.9999	0.1252	0.2382
98	Ni	1.7353	13.8972	5.2269	0.1248	0.9997	0.2381
99	Ni	13.8983	5.2062	5.2360	0.9997	0.3745	0.2385
100	Ni	1.7370	3.4773	5.2209	0.1249	0.2501	0.2378
101	Ni	3.4626	1.7337	5.2258	0.2491	0.1247	0.2381
102	Ni	5.2081	0.0006	5.2184	0.3746	0.0000	0.2377
103	Ni	3.4449	5.1697	5.0858	0.2478	0.3719	0.2317
104	Ni	5.1765	3.4495	5.2009	0.3724	0.2481	0.2369
105	Ni	13.8742	8.7199	5.2157	0.9980	0.6272	0.2376
106	Ni	1.7025	6.9380	5.3794	0.1225	0.4991	0.2451
107	Ni	13.8980	12.1628	5.2281	0.9997	0.8749	0.2382
108	Ni	1.7359	10.4519	5.2022	0.1249	0.7518	0.2370
109	Ni	3.4560	8.6686	5.2670	0.2486	0.6236	0.2399
110	Ni	5.2325	6.9053	5.2245	0.3764	0.4967	0.2380
111	Ni	3.4731	12.1586	5.2243	0.2498	0.8746	0.2380
112	Ni	5.2043	10.4492	5.2786	0.3744	0.7516	0.2405
113	Ni	6.9411	1.7462	5.2227	0.4993	0.1256	0.2379
114	Ni	8.6876	0.0239	5.2883	0.6249	0.0017	0.2409
115	Ni	7.0007	5.1731	5.3846	0.5036	0.3721	0.2453
116	Ni	8.7052	3.4555	5.2318	0.6262	0.2486	0.2383
117	Ni	10.4387	1.7395	5.2241	0.7509	0.1251	0.2380
118	Ni	12.1733	0.0083	5.2272	0.8757	0.0006	0.2381
119	Ni	10.4182	5.2196	5.2318	0.7494	0.3755	0.2383
120	Ni	12.1626	3.4772	5.2271	0.8749	0.2501	0.2381
121	Ni	6.9928	8.7088	5.2388	0.5030	0.6264	0.2387
122	Ni	8.7051	6.9718	5.2006	0.6262	0.5015	0.2369
123	Ni	6.9603	12.2013	5.1768	0.5007	0.8777	0.2358
124	Ni	8.7195	10.4543	5.2173	0.6272	0.7520	0.2377
125	Ni	10.4254	8.6870	5.2215	0.7499	0.6249	0.2379
126	Ni	12.1645	6.9513	5.2292	0.8750	0.5000	0.2382
127	Ni	10.4297	12.1854	5.2908	0.7502	0.8765	0.2410
128	Ni	12.1561	10.4314	5.2229	0.8744	0.7504	0.2379
129	O	5.9071	5.9158	6.7849	0.4249	0.4255	0.3091
130	O	2.6650	7.8272	6.8272	0.1917	0.5630	0.3110
131	O	4.8734	7.7159	8.7863	0.3506	0.5550	0.4003
132	O	6.1101	9.5575	6.8065	0.4395	0.6875	0.3101
133	O	8.0215	9.4456	8.9169	0.5770	0.6794	0.4062
134	O	5.2187	11.3018	8.8564	0.3754	0.8130	0.4035
135	O	8.6275	12.1695	6.2805	0.6206	0.8754	0.2861
136	Ce	3.9894	9.7715	8.0309	0.2870	0.7029	0.3659
137	Ce	3.7988	6.0623	7.6930	0.2733	0.4361	0.3505

138 Ce	7.1215	7.6991	8.0279	0.5123	0.5538	0.3657
139 Sm	7.2129	11.1966	8.0376	0.5188	0.8054	0.3662

/db/jmorales/CoNi-alloy/metal-SDC-interface/CoNi-Ce_3SmO_7/CoNi-Ce_3SmO_7-conf3a

a =			13.9033384264				
b =			13.9033384264				
c =			21.9516692133				
alpha =			90.0				
beta =			90.0				
gamma =			90.0				
	Atom	X	Y	Z	X	Y	Z
1	Ni	0.0000	0.0000	0.0000	0.0000	0.0000	0.0000
2	Ni	0.0000	3.4758	0.0000	0.0000	0.2500	0.0000
3	Ni	3.4758	0.0000	0.0000	0.2500	0.0000	0.0000
4	Ni	3.4758	3.4758	0.0000	0.2500	0.2500	0.0000
5	Ni	0.0000	6.9517	0.0000	0.0000	0.5000	0.0000
6	Ni	0.0000	10.4275	0.0000	0.0000	0.7500	0.0000
7	Ni	3.4758	6.9517	0.0000	0.2500	0.5000	0.0000
8	Ni	3.4758	10.4275	0.0000	0.2500	0.7500	0.0000
9	Ni	6.9517	0.0000	0.0000	0.5000	0.0000	0.0000
10	Ni	6.9517	3.4758	0.0000	0.5000	0.2500	0.0000
11	Ni	10.4275	0.0000	0.0000	0.7500	0.0000	0.0000
12	Ni	10.4275	3.4758	0.0000	0.7500	0.2500	0.0000
13	Ni	6.9517	6.9517	0.0000	0.5000	0.5000	0.0000
14	Ni	6.9517	10.4275	0.0000	0.5000	0.7500	0.0000
15	Ni	10.4275	6.9517	0.0000	0.7500	0.5000	0.0000
16	Ni	10.4275	10.4275	0.0000	0.7500	0.7500	0.0000
17	Ni	0.0000	1.7379	1.7379	0.0000	0.1250	0.0792
18	Ni	0.0000	5.2138	1.7379	0.0000	0.3750	0.0792
19	Ni	3.4758	1.7379	1.7379	0.2500	0.1250	0.0792
20	Ni	3.4758	5.2138	1.7379	0.2500	0.3750	0.0792
21	Ni	0.0000	8.6896	1.7379	0.0000	0.6250	0.0792
22	Ni	0.0000	12.1654	1.7379	0.0000	0.8750	0.0792
23	Ni	3.4758	8.6896	1.7379	0.2500	0.6250	0.0792
24	Ni	3.4758	12.1654	1.7379	0.2500	0.8750	0.0792
25	Ni	6.9517	1.7379	1.7379	0.5000	0.1250	0.0792
26	Ni	6.9517	5.2138	1.7379	0.5000	0.3750	0.0792
27	Ni	10.4275	1.7379	1.7379	0.7500	0.1250	0.0792
28	Ni	10.4275	5.2138	1.7379	0.7500	0.3750	0.0792
29	Ni	6.9517	8.6896	1.7379	0.5000	0.6250	0.0792
30	Ni	6.9517	12.1654	1.7379	0.5000	0.8750	0.0792
31	Ni	10.4275	8.6896	1.7379	0.7500	0.6250	0.0792
32	Ni	10.4275	12.1654	1.7379	0.7500	0.8750	0.0792
33	Ni	13.8983	13.8980	3.5026	0.9996	0.9996	0.1596
34	Ni	13.8748	3.4685	3.4717	0.9979	0.2495	0.1582
35	Ni	3.4803	13.8936	3.4966	0.2503	0.9993	0.1593
36	Ni	3.5055	3.4901	3.5097	0.2521	0.2510	0.1599
37	Ni	0.0109	6.9520	3.5133	0.0008	0.5000	0.1600
38	Ni	0.0021	10.4280	3.5017	0.0001	0.7500	0.1595
39	Ni	3.4877	6.9523	3.5174	0.2509	0.5000	0.1602

40	Ni	3.4734	10.4378	3.4891	0.2498	0.7507	0.1589
41	Ni	6.9507	13.9005	3.5016	0.4999	0.9998	0.1595
42	Ni	6.9344	3.4757	3.5371	0.4988	0.2500	0.1611
43	Ni	10.4254	13.9013	3.5015	0.7499	0.9999	0.1595
44	Ni	10.4198	3.4698	3.5099	0.7494	0.2496	0.1599
45	Ni	6.9386	6.9306	3.5004	0.4991	0.4985	0.1595
46	Ni	6.9495	10.4503	3.4789	0.4998	0.7516	0.1585
47	Ni	10.4405	6.9555	3.4908	0.7509	0.5003	0.1590
48	Ni	10.4336	10.4319	3.5004	0.7504	0.7503	0.1595
49	Ni	0.0010	1.7303	5.2097	0.0001	0.1245	0.2373
50	Ni	13.8690	5.1840	5.1846	0.9975	0.3729	0.2362
51	Ni	3.4269	1.7069	5.1866	0.2465	0.1228	0.2363
52	Ni	3.4083	5.2090	5.2104	0.2451	0.3747	0.2374
53	Ni	0.0044	8.6977	5.2137	0.0003	0.6256	0.2375
54	Ni	0.0034	12.1615	5.2099	0.0002	0.8747	0.2373
55	Ni	3.4621	8.7285	5.1743	0.2490	0.6278	0.2357
56	Ni	3.4713	12.1617	5.2055	0.2497	0.8747	0.2371
57	Ni	6.9654	1.6862	5.2050	0.5010	0.1213	0.2371
58	Ni	7.0239	5.2425	5.2395	0.5052	0.3771	0.2387
59	Ni	10.4189	1.7296	5.2061	0.7494	0.1244	0.2372
60	Ni	10.4633	5.2404	5.1919	0.7526	0.3769	0.2365
61	Ni	6.9903	8.7308	5.1125	0.5028	0.6280	0.2329
62	Ni	6.9536	12.1635	5.2136	0.5001	0.8749	0.2375
63	Ni	10.4306	8.7007	5.2121	0.7502	0.6258	0.2374
64	Ni	10.4261	12.1621	5.2107	0.7499	0.8748	0.2374
65	Co	1.7379	1.7379	0.0000	0.1250	0.1250	0.0000
66	Co	1.7379	5.2138	0.0000	0.1250	0.3750	0.0000
67	Co	5.2138	1.7379	0.0000	0.3750	0.1250	0.0000
68	Co	5.2138	5.2138	0.0000	0.3750	0.3750	0.0000
69	Co	1.7379	8.6896	0.0000	0.1250	0.6250	0.0000
70	Co	1.7379	12.1654	0.0000	0.1250	0.8750	0.0000
71	Co	5.2138	8.6896	0.0000	0.3750	0.6250	0.0000
72	Co	5.2138	12.1654	0.0000	0.3750	0.8750	0.0000
73	Co	8.6896	1.7379	0.0000	0.6250	0.1250	0.0000
74	Co	8.6896	5.2138	0.0000	0.6250	0.3750	0.0000
75	Co	12.1654	1.7379	0.0000	0.8750	0.1250	0.0000
76	Co	12.1654	5.2138	0.0000	0.8750	0.3750	0.0000
77	Co	8.6896	8.6896	0.0000	0.6250	0.6250	0.0000
78	Co	8.6896	12.1654	0.0000	0.6250	0.8750	0.0000
79	Co	12.1654	8.6896	0.0000	0.8750	0.6250	0.0000
80	Co	12.1654	12.1654	0.0000	0.8750	0.8750	0.0000
81	Co	1.7379	0.0000	1.7379	0.1250	0.0000	0.0792
82	Co	1.7379	3.4758	1.7379	0.1250	0.2500	0.0792
83	Co	5.2138	0.0000	1.7379	0.3750	0.0000	0.0792
84	Co	5.2138	3.4758	1.7379	0.3750	0.2500	0.0792
85	Co	1.7379	6.9517	1.7379	0.1250	0.5000	0.0792
86	Co	1.7379	10.4275	1.7379	0.1250	0.7500	0.0792
87	Co	5.2138	6.9517	1.7379	0.3750	0.5000	0.0792
88	Co	5.2138	10.4275	1.7379	0.3750	0.7500	0.0792

89	Co	8.6896	0.0000	1.7379	0.6250	0.0000	0.0792
90	Co	8.6896	3.4758	1.7379	0.6250	0.2500	0.0792
91	Co	12.1654	0.0000	1.7379	0.8750	0.0000	0.0792
92	Co	12.1654	3.4758	1.7379	0.8750	0.2500	0.0792
93	Co	8.6896	6.9517	1.7379	0.6250	0.5000	0.0792
94	Co	8.6896	10.4275	1.7379	0.6250	0.7500	0.0792
95	Co	12.1654	6.9517	1.7379	0.8750	0.5000	0.0792
96	Co	12.1654	10.4275	1.7379	0.8750	0.7500	0.0792
97	Co	1.7342	1.7173	3.5085	0.1247	0.1235	0.1598
98	Co	1.7230	5.2160	3.5097	0.1239	0.3752	0.1599
99	Co	5.2130	1.7493	3.5708	0.3749	0.1258	0.1627
100	Co	5.2161	5.2100	3.6310	0.3752	0.3747	0.1654
101	Co	1.7299	8.6718	3.5449	0.1244	0.6237	0.1615
102	Co	1.7348	12.1649	3.5500	0.1248	0.8750	0.1617
103	Co	5.2069	8.6930	3.5318	0.3745	0.6252	0.1609
104	Co	5.2143	12.1638	3.5565	0.3750	0.8749	0.1620
105	Co	8.6904	1.7558	3.5498	0.6251	0.1263	0.1617
106	Co	8.7045	5.2075	3.5234	0.6261	0.3746	0.1605
107	Co	12.1605	1.7252	3.5523	0.8746	0.1241	0.1618
108	Co	12.1636	5.2147	3.5431	0.8749	0.3751	0.1614
109	Co	8.7138	8.6934	3.5104	0.6267	0.6253	0.1599
110	Co	8.6916	12.1626	3.5480	0.6251	0.8748	0.1616
111	Co	12.1703	8.6949	3.5535	0.8754	0.6254	0.1619
112	Co	12.1653	12.1655	3.5572	0.8750	0.8750	0.1620
113	Co	1.7315	-0.0011	5.2231	0.1245	-0.0001	0.2379
114	Co	1.6974	3.4197	5.1134	0.1221	0.2460	0.2329
115	Co	5.2061	13.8941	5.2171	0.3745	0.9993	0.2377
116	Co	5.1581	3.4262	5.3705	0.3710	0.2464	0.2447
117	Co	1.7051	6.9575	5.3043	0.1226	0.5004	0.2416
118	Co	1.7438	10.4169	5.2218	0.1254	0.7492	0.2379
119	Co	5.2655	7.0194	5.2774	0.3787	0.5049	0.2404
120	Co	5.2148	10.4167	5.2206	0.3751	0.7492	0.2378
121	Co	8.6931	13.8961	5.2151	0.6253	0.9995	0.2376
122	Co	8.7219	3.4767	5.3526	0.6273	0.2501	0.2438
123	Co	12.1617	13.9010	5.2265	0.8747	0.9998	0.2381
124	Co	12.1591	3.4549	5.2130	0.8745	0.2485	0.2375
125	Co	8.7147	7.0105	5.1824	0.6268	0.5042	0.2361
126	Co	8.6865	10.4227	5.2192	0.6248	0.7497	0.2378
127	Co	12.1720	6.9679	5.2171	0.8755	0.5012	0.2377
128	Co	12.1713	10.4275	5.2239	0.8754	0.7500	0.2380
129	O	4.9987	3.4585	7.2135	0.3595	0.2488	0.3286
130	O	2.3875	5.9080	6.7643	0.1717	0.4249	0.3081
131	O	4.0122	5.4541	9.1128	0.2886	0.3923	0.4151
132	O	7.9044	4.3796	6.6963	0.5685	0.3150	0.3050
133	O	5.4856	6.9443	7.1419	0.3946	0.4995	0.3253
134	O	7.5092	6.9192	9.0684	0.5401	0.4977	0.4131
135	O	4.9371	9.0609	8.8866	0.3551	0.6517	0.4048
136	Ce	3.5128	7.5811	8.2081	0.2527	0.5453	0.3739
137	Ce	2.9566	3.9494	7.8791	0.2127	0.2841	0.3589

138 Ce	6.4085	5.1549	8.3902	0.4609	0.3708	0.3822
139 Sm	6.8242	8.6149	8.0218	0.4908	0.6196	0.3654

/db/jmorales/CoNi-alloy/metal-SDC-interface/CoNi-Ce_3SmO_7/CoNi-Ce_3SmO_7-conf5d

a =				13.9033384264			
b =				13.9033384264			
c =				21.9516692133			
alpha =				90.0			
beta =				90.0			
gamma =				90.0			
	Atom	X	Y	Z	X	Y	Z
1	Ni	0.0000	0.0000	0.0000	0.0000	0.0000	0.0000
2	Ni	0.0000	3.4758	0.0000	0.0000	0.2500	0.0000
3	Ni	3.4758	0.0000	0.0000	0.2500	0.0000	0.0000
4	Ni	3.4758	3.4758	0.0000	0.2500	0.2500	0.0000
5	Ni	0.0000	6.9517	0.0000	0.0000	0.5000	0.0000
6	Ni	0.0000	10.4275	0.0000	0.0000	0.7500	0.0000
7	Ni	3.4758	6.9517	0.0000	0.2500	0.5000	0.0000
8	Ni	3.4758	10.4275	0.0000	0.2500	0.7500	0.0000
9	Ni	6.9517	0.0000	0.0000	0.5000	0.0000	0.0000
10	Ni	6.9517	3.4758	0.0000	0.5000	0.2500	0.0000
11	Ni	10.4275	0.0000	0.0000	0.7500	0.0000	0.0000
12	Ni	10.4275	3.4758	0.0000	0.7500	0.2500	0.0000
13	Ni	6.9517	6.9517	0.0000	0.5000	0.5000	0.0000
14	Ni	6.9517	10.4275	0.0000	0.5000	0.7500	0.0000
15	Ni	10.4275	6.9517	0.0000	0.7500	0.5000	0.0000
16	Ni	10.4275	10.4275	0.0000	0.7500	0.7500	0.0000
17	Ni	0.0000	1.7379	1.7379	0.0000	0.1250	0.0792
18	Ni	0.0000	5.2138	1.7379	0.0000	0.3750	0.0792
19	Ni	3.4758	1.7379	1.7379	0.2500	0.1250	0.0792
20	Ni	3.4758	5.2138	1.7379	0.2500	0.3750	0.0792
21	Ni	0.0000	8.6896	1.7379	0.0000	0.6250	0.0792
22	Ni	0.0000	12.1654	1.7379	0.0000	0.8750	0.0792
23	Ni	3.4758	8.6896	1.7379	0.2500	0.6250	0.0792
24	Ni	3.4758	12.1654	1.7379	0.2500	0.8750	0.0792
25	Ni	6.9517	1.7379	1.7379	0.5000	0.1250	0.0792
26	Ni	6.9517	5.2138	1.7379	0.5000	0.3750	0.0792
27	Ni	10.4275	1.7379	1.7379	0.7500	0.1250	0.0792
28	Ni	10.4275	5.2138	1.7379	0.7500	0.3750	0.0792
29	Ni	6.9517	8.6896	1.7379	0.5000	0.6250	0.0792
30	Ni	6.9517	12.1654	1.7379	0.5000	0.8750	0.0792
31	Ni	10.4275	8.6896	1.7379	0.7500	0.6250	0.0792
32	Ni	10.4275	12.1654	1.7379	0.7500	0.8750	0.0792
33	Ni	13.9027	-0.0002	3.5058	1.0000	-0.0000	0.1597
34	Ni	0.0024	3.4780	3.5080	0.0002	0.2502	0.1598
35	Ni	3.4661	13.8940	3.5048	0.2493	0.9993	0.1597
36	Ni	3.4364	3.4940	3.4924	0.2472	0.2513	0.1591
37	Ni	13.9028	6.9504	3.5072	1.0000	0.4999	0.1598
38	Ni	13.9009	10.4330	3.5033	0.9998	0.7504	0.1596
39	Ni	3.4658	6.9503	3.5018	0.2493	0.4999	0.1595

40	Ni	3.4822	10.4311	3.4981	0.2505	0.7503	0.1594
41	Ni	6.9556	13.8995	3.5046	0.5003	0.9997	0.1596
42	Ni	6.9766	3.4628	3.4784	0.5018	0.2491	0.1585
43	Ni	10.4295	0.0041	3.5055	0.7501	0.0003	0.1597
44	Ni	10.4186	3.4744	3.5098	0.7494	0.2499	0.1599
45	Ni	6.9363	6.9290	3.5314	0.4989	0.4984	0.1609
46	Ni	6.9408	10.4090	3.5081	0.4992	0.7487	0.1598
47	Ni	10.4284	6.9549	3.4956	0.7501	0.5002	0.1592
48	Ni	10.4455	10.4400	3.4756	0.7513	0.7509	0.1583
49	Ni	13.8975	1.7369	5.2096	0.9996	0.1249	0.2373
50	Ni	13.8963	5.2171	5.2125	0.9995	0.3752	0.2375
51	Ni	3.4725	1.7356	5.2021	0.2498	0.1248	0.2370
52	Ni	3.4671	5.1740	5.3421	0.2494	0.3721	0.2434
53	Ni	13.8937	8.7005	5.2056	0.9993	0.6258	0.2371
54	Ni	0.0000	12.1636	5.2122	0.0000	0.8749	0.2374
55	Ni	3.4680	8.7226	5.1912	0.2494	0.6274	0.2365
56	Ni	3.4747	12.1600	5.2082	0.2499	0.8746	0.2373
57	Ni	6.9182	1.7093	5.1927	0.4976	0.1229	0.2366
58	Ni	6.9527	5.1551	5.2222	0.5001	0.3708	0.2379
59	Ni	10.4342	1.7314	5.2121	0.7505	0.1245	0.2374
60	Ni	10.4388	5.2310	5.1926	0.7508	0.3762	0.2365
61	Ni	6.9591	8.7141	5.2734	0.5005	0.6268	0.2402
62	Ni	6.9576	12.1555	5.2064	0.5004	0.8743	0.2372
63	Ni	10.4440	8.7295	5.1502	0.7512	0.6279	0.2346
64	Ni	10.4269	12.1688	5.2090	0.7500	0.8752	0.2373
65	Co	1.7379	1.7379	0.0000	0.1250	0.1250	0.0000
66	Co	1.7379	5.2138	0.0000	0.1250	0.3750	0.0000
67	Co	5.2138	1.7379	0.0000	0.3750	0.1250	0.0000
68	Co	5.2138	5.2138	0.0000	0.3750	0.3750	0.0000
69	Co	1.7379	8.6896	0.0000	0.1250	0.6250	0.0000
70	Co	1.7379	12.1654	0.0000	0.1250	0.8750	0.0000
71	Co	5.2138	8.6896	0.0000	0.3750	0.6250	0.0000
72	Co	5.2138	12.1654	0.0000	0.3750	0.8750	0.0000
73	Co	8.6896	1.7379	0.0000	0.6250	0.1250	0.0000
74	Co	8.6896	5.2138	0.0000	0.6250	0.3750	0.0000
75	Co	12.1654	1.7379	0.0000	0.8750	0.1250	0.0000
76	Co	12.1654	5.2138	0.0000	0.8750	0.3750	0.0000
77	Co	8.6896	8.6896	0.0000	0.6250	0.6250	0.0000
78	Co	8.6896	12.1654	0.0000	0.6250	0.8750	0.0000
79	Co	12.1654	8.6896	0.0000	0.8750	0.6250	0.0000
80	Co	12.1654	12.1654	0.0000	0.8750	0.8750	0.0000
81	Co	1.7379	0.0000	1.7379	0.1250	0.0000	0.0792
82	Co	1.7379	3.4758	1.7379	0.1250	0.2500	0.0792
83	Co	5.2138	0.0000	1.7379	0.3750	0.0000	0.0792
84	Co	5.2138	3.4758	1.7379	0.3750	0.2500	0.0792
85	Co	1.7379	6.9517	1.7379	0.1250	0.5000	0.0792
86	Co	1.7379	10.4275	1.7379	0.1250	0.7500	0.0792
87	Co	5.2138	6.9517	1.7379	0.3750	0.5000	0.0792
88	Co	5.2138	10.4275	1.7379	0.3750	0.7500	0.0792

89	Co	8.6896	0.0000	1.7379	0.6250	0.0000	0.0792
90	Co	8.6896	3.4758	1.7379	0.6250	0.2500	0.0792
91	Co	12.1654	0.0000	1.7379	0.8750	0.0000	0.0792
92	Co	12.1654	3.4758	1.7379	0.8750	0.2500	0.0792
93	Co	8.6896	6.9517	1.7379	0.6250	0.5000	0.0792
94	Co	8.6896	10.4275	1.7379	0.6250	0.7500	0.0792
95	Co	12.1654	6.9517	1.7379	0.8750	0.5000	0.0792
96	Co	12.1654	10.4275	1.7379	0.8750	0.7500	0.0792
97	Co	1.7330	1.7311	3.5524	0.1246	0.1245	0.1618
98	Co	1.7512	5.2200	3.5822	0.1260	0.3754	0.1632
99	Co	5.2059	1.6924	3.5031	0.3744	0.1217	0.1596
100	Co	5.2064	5.2130	3.5257	0.3745	0.3749	0.1606
101	Co	1.7336	8.6937	3.5457	0.1247	0.6253	0.1615
102	Co	1.7345	12.1642	3.5524	0.1248	0.8749	0.1618
103	Co	5.2338	8.6731	3.5665	0.3764	0.6238	0.1625
104	Co	5.2179	12.1638	3.5495	0.3753	0.8749	0.1617
105	Co	8.6926	1.7525	3.5707	0.6252	0.1261	0.1627
106	Co	8.6940	5.2167	3.5527	0.6253	0.3752	0.1618
107	Co	12.1658	1.7423	3.5541	0.8750	0.1253	0.1619
108	Co	12.1709	5.2189	3.5477	0.8754	0.3754	0.1616
109	Co	8.6813	8.7000	3.5028	0.6244	0.6257	0.1596
110	Co	8.6887	12.1893	3.5203	0.6249	0.8767	0.1604
111	Co	12.1800	8.6909	3.5254	0.8760	0.6251	0.1606
112	Co	12.1684	12.1710	3.5512	0.8752	0.8754	0.1618
113	Co	1.7354	13.9000	5.2308	0.1248	0.9998	0.2383
114	Co	1.7235	3.4669	5.2320	0.1240	0.2494	0.2383
115	Co	5.2042	13.8964	5.2271	0.3743	0.9995	0.2381
116	Co	5.1790	3.4140	5.0541	0.3725	0.2456	0.2302
117	Co	1.7035	6.9784	5.2211	0.1225	0.5019	0.2378
118	Co	1.7386	10.4263	5.2232	0.1251	0.7499	0.2379
119	Co	5.1598	6.9027	5.2370	0.3711	0.4965	0.2386
120	Co	5.2051	10.4218	5.2243	0.3744	0.7496	0.2380
121	Co	8.6890	13.8997	5.2220	0.6250	0.9997	0.2379
122	Co	8.7203	3.4717	5.3331	0.6272	0.2497	0.2429
123	Co	12.1654	13.9002	5.2259	0.8750	0.9998	0.2381
124	Co	12.1518	3.4877	5.2219	0.8740	0.2509	0.2379
125	Co	8.7318	6.9929	5.2091	0.6280	0.5030	0.2373
126	Co	8.7201	10.4639	5.1324	0.6272	0.7526	0.2338
127	Co	12.1451	6.9620	5.2282	0.8735	0.5007	0.2382
128	Co	12.1582	10.4285	5.2230	0.8745	0.7501	0.2379
129	O	7.6150	4.0650	6.7425	0.5477	0.2924	0.3072
130	O	4.6234	3.2862	9.2414	0.3325	0.2364	0.4210
131	O	4.3944	6.1437	6.8001	0.3161	0.4419	0.3098
132	O	6.6275	5.9273	8.7808	0.4767	0.4263	0.4000
133	O	7.8043	7.6842	6.7845	0.5613	0.5527	0.3091
134	O	9.7840	7.5997	8.8134	0.7037	0.5466	0.4015
135	O	7.0479	9.4593	8.8362	0.5069	0.6804	0.4025
136	Ce	5.7348	7.9285	8.1069	0.4125	0.5703	0.3693
137	Ce	5.4097	4.2190	7.8225	0.3891	0.3035	0.3563

138 Ce	8.8175	5.8197	8.0695	0.6342	0.4186	0.3676
139 Sm	8.9642	9.3362	7.9356	0.6447	0.6715	0.3615

/db/jmorales/CoNi-alloy/metal-SDC-interface/CoNi-Ce_3SmO_7/CoNi-Ce_3SmO_7-conf6a

a =			13.9033384264				
b =			13.9033384264				
c =			21.9516692133				
alpha =			90.0				
beta =			90.0				
gamma =			90.0				
	Atom	X	Y	Z	X	Y	Z
1	Ni	0.0000	0.0000	0.0000	0.0000	0.0000	0.0000
2	Ni	0.0000	3.4758	0.0000	0.0000	0.2500	0.0000
3	Ni	3.4758	0.0000	0.0000	0.2500	0.0000	0.0000
4	Ni	3.4758	3.4758	0.0000	0.2500	0.2500	0.0000
5	Ni	0.0000	6.9517	0.0000	0.0000	0.5000	0.0000
6	Ni	0.0000	10.4275	0.0000	0.0000	0.7500	0.0000
7	Ni	3.4758	6.9517	0.0000	0.2500	0.5000	0.0000
8	Ni	3.4758	10.4275	0.0000	0.2500	0.7500	0.0000
9	Ni	6.9517	0.0000	0.0000	0.5000	0.0000	0.0000
10	Ni	6.9517	3.4758	0.0000	0.5000	0.2500	0.0000
11	Ni	10.4275	0.0000	0.0000	0.7500	0.0000	0.0000
12	Ni	10.4275	3.4758	0.0000	0.7500	0.2500	0.0000
13	Ni	6.9517	6.9517	0.0000	0.5000	0.5000	0.0000
14	Ni	6.9517	10.4275	0.0000	0.5000	0.7500	0.0000
15	Ni	10.4275	6.9517	0.0000	0.7500	0.5000	0.0000
16	Ni	10.4275	10.4275	0.0000	0.7500	0.7500	0.0000
17	Ni	0.0000	1.7379	1.7379	0.0000	0.1250	0.0792
18	Ni	0.0000	5.2138	1.7379	0.0000	0.3750	0.0792
19	Ni	3.4758	1.7379	1.7379	0.2500	0.1250	0.0792
20	Ni	3.4758	5.2138	1.7379	0.2500	0.3750	0.0792
21	Ni	0.0000	8.6896	1.7379	0.0000	0.6250	0.0792
22	Ni	0.0000	12.1654	1.7379	0.0000	0.8750	0.0792
23	Ni	3.4758	8.6896	1.7379	0.2500	0.6250	0.0792
24	Ni	3.4758	12.1654	1.7379	0.2500	0.8750	0.0792
25	Ni	6.9517	1.7379	1.7379	0.5000	0.1250	0.0792
26	Ni	6.9517	5.2138	1.7379	0.5000	0.3750	0.0792
27	Ni	10.4275	1.7379	1.7379	0.7500	0.1250	0.0792
28	Ni	10.4275	5.2138	1.7379	0.7500	0.3750	0.0792
29	Ni	6.9517	8.6896	1.7379	0.5000	0.6250	0.0792
30	Ni	6.9517	12.1654	1.7379	0.5000	0.8750	0.0792
31	Ni	10.4275	8.6896	1.7379	0.7500	0.6250	0.0792
32	Ni	10.4275	12.1654	1.7379	0.7500	0.8750	0.0792
33	Ni	0.0058	0.0031	3.5157	0.0004	0.0002	0.1602
34	Ni	13.8735	3.4653	3.4768	0.9979	0.2492	0.1584
35	Ni	3.4708	-0.0002	3.5075	0.2496	-0.0000	0.1598
36	Ni	3.5071	3.4795	3.5073	0.2523	0.2503	0.1598
37	Ni	0.0096	6.9543	3.5124	0.0007	0.5002	0.1600
38	Ni	0.0004	10.4280	3.5046	0.0000	0.7500	0.1597
39	Ni	3.4830	6.9509	3.5119	0.2505	0.4999	0.1600

40	Ni	3.4701	10.4386	3.4902	0.2496	0.7508	0.1590
41	Ni	6.9510	13.8999	3.5040	0.4999	0.9997	0.1596
42	Ni	6.9335	3.4657	3.5078	0.4987	0.2493	0.1598
43	Ni	10.4288	13.9010	3.5054	0.7501	0.9998	0.1597
44	Ni	10.4301	3.4730	3.5013	0.7502	0.2498	0.1595
45	Ni	6.9424	6.9495	3.4887	0.4993	0.4998	0.1589
46	Ni	6.9481	10.4478	3.4812	0.4997	0.7515	0.1586
47	Ni	10.4306	6.9539	3.5025	0.7502	0.5002	0.1596
48	Ni	10.4324	10.4292	3.5033	0.7504	0.7501	0.1596
49	Ni	13.8756	1.7401	5.2660	0.9980	0.1252	0.2399
50	Ni	13.8656	5.1888	5.1797	0.9973	0.3732	0.2360
51	Ni	3.4721	1.7256	5.2134	0.2497	0.1241	0.2375
52	Ni	3.4147	5.1906	5.1981	0.2456	0.3733	0.2368
53	Ni	0.0023	8.6922	5.2146	0.0002	0.6252	0.2376
54	Ni	0.0076	12.1720	5.2089	0.0005	0.8755	0.2373
55	Ni	3.4543	8.7223	5.1737	0.2484	0.6274	0.2357
56	Ni	3.4706	12.1684	5.2072	0.2496	0.8752	0.2372
57	Ni	6.9929	1.7102	5.2054	0.5030	0.1230	0.2371
58	Ni	7.0089	5.2054	5.1591	0.5041	0.3744	0.2350
59	Ni	10.4153	1.7347	5.2120	0.7491	0.1248	0.2374
60	Ni	10.4200	5.2178	5.2075	0.7495	0.3753	0.2372
61	Ni	6.9890	8.7277	5.1219	0.5027	0.6277	0.2333
62	Ni	6.9550	12.1607	5.2140	0.5002	0.8747	0.2375
63	Ni	10.4194	8.6943	5.2158	0.7494	0.6253	0.2376
64	Ni	10.4248	12.1605	5.2115	0.7498	0.8746	0.2374
65	Co	1.7379	1.7379	0.0000	0.1250	0.1250	0.0000
66	Co	1.7379	5.2138	0.0000	0.1250	0.3750	0.0000
67	Co	5.2138	1.7379	0.0000	0.3750	0.1250	0.0000
68	Co	5.2138	5.2138	0.0000	0.3750	0.3750	0.0000
69	Co	1.7379	8.6896	0.0000	0.1250	0.6250	0.0000
70	Co	1.7379	12.1654	0.0000	0.1250	0.8750	0.0000
71	Co	5.2138	8.6896	0.0000	0.3750	0.6250	0.0000
72	Co	5.2138	12.1654	0.0000	0.3750	0.8750	0.0000
73	Co	8.6896	1.7379	0.0000	0.6250	0.1250	0.0000
74	Co	8.6896	5.2138	0.0000	0.6250	0.3750	0.0000
75	Co	12.1654	1.7379	0.0000	0.8750	0.1250	0.0000
76	Co	12.1654	5.2138	0.0000	0.8750	0.3750	0.0000
77	Co	8.6896	8.6896	0.0000	0.6250	0.6250	0.0000
78	Co	8.6896	12.1654	0.0000	0.6250	0.8750	0.0000
79	Co	12.1654	8.6896	0.0000	0.8750	0.6250	0.0000
80	Co	12.1654	12.1654	0.0000	0.8750	0.8750	0.0000
81	Co	1.7379	0.0000	1.7379	0.1250	0.0000	0.0792
82	Co	1.7379	3.4758	1.7379	0.1250	0.2500	0.0792
83	Co	5.2138	0.0000	1.7379	0.3750	0.0000	0.0792
84	Co	5.2138	3.4758	1.7379	0.3750	0.2500	0.0792
85	Co	1.7379	6.9517	1.7379	0.1250	0.5000	0.0792
86	Co	1.7379	10.4275	1.7379	0.1250	0.7500	0.0792
87	Co	5.2138	6.9517	1.7379	0.3750	0.5000	0.0792
88	Co	5.2138	10.4275	1.7379	0.3750	0.7500	0.0792

89	Co	8.6896	0.0000	1.7379	0.6250	0.0000	0.0792
90	Co	8.6896	3.4758	1.7379	0.6250	0.2500	0.0792
91	Co	12.1654	0.0000	1.7379	0.8750	0.0000	0.0792
92	Co	12.1654	3.4758	1.7379	0.8750	0.2500	0.0792
93	Co	8.6896	6.9517	1.7379	0.6250	0.5000	0.0792
94	Co	8.6896	10.4275	1.7379	0.6250	0.7500	0.0792
95	Co	12.1654	6.9517	1.7379	0.8750	0.5000	0.0792
96	Co	12.1654	10.4275	1.7379	0.8750	0.7500	0.0792
97	Co	1.7344	1.7121	3.4533	0.1247	0.1231	0.1573
98	Co	1.7222	5.2143	3.4962	0.1239	0.3750	0.1593
99	Co	5.2135	1.7443	3.5780	0.3750	0.1255	0.1630
100	Co	5.2007	5.2044	3.5936	0.3741	0.3743	0.1637
101	Co	1.7283	8.6751	3.5464	0.1243	0.6240	0.1616
102	Co	1.7357	12.1800	3.5614	0.1248	0.8761	0.1622
103	Co	5.2056	8.6947	3.5364	0.3744	0.6254	0.1611
104	Co	5.2104	12.1683	3.5554	0.3748	0.8752	0.1620
105	Co	8.6931	1.7363	3.5388	0.6253	0.1249	0.1612
106	Co	8.7039	5.2165	3.5222	0.6260	0.3752	0.1605
107	Co	12.1723	1.7295	3.5745	0.8755	0.1244	0.1628
108	Co	12.1601	5.2174	3.5498	0.8746	0.3753	0.1617
109	Co	8.7131	8.6901	3.5177	0.6267	0.6250	0.1602
110	Co	8.6914	12.1630	3.5463	0.6251	0.8748	0.1615
111	Co	12.1655	8.6920	3.5611	0.8750	0.6252	0.1622
112	Co	12.1677	12.1667	3.5579	0.8752	0.8751	0.1621
113	Co	1.7352	0.0474	5.3152	0.1248	0.0034	0.2421
114	Co	1.6966	3.4137	5.1087	0.1220	0.2455	0.2327
115	Co	5.2191	13.8861	5.2106	0.3754	0.9988	0.2374
116	Co	5.2505	3.4658	5.3551	0.3776	0.2493	0.2439
117	Co	1.7106	6.9438	5.2932	0.1230	0.4994	0.2411
118	Co	1.7400	10.4262	5.2174	0.1251	0.7499	0.2377
119	Co	5.2615	6.9908	5.2556	0.3784	0.5028	0.2394
120	Co	5.2099	10.4228	5.2171	0.3747	0.7497	0.2377
121	Co	8.6938	13.8892	5.2248	0.6253	0.9990	0.2380
122	Co	8.6925	3.4831	5.2087	0.6252	0.2505	0.2373
123	Co	12.1572	13.8944	5.2267	0.8744	0.9994	0.2381
124	Co	12.1467	3.4746	5.2188	0.8736	0.2499	0.2377
125	Co	8.6807	6.9596	5.2170	0.6244	0.5006	0.2377
126	Co	8.6838	10.4239	5.2214	0.6246	0.7497	0.2379
127	Co	12.1647	6.9564	5.2221	0.8750	0.5003	0.2379
128	Co	12.1742	10.4323	5.2269	0.8756	0.7503	0.2381
129	O	1.8214	1.8765	6.3607	0.1310	0.1350	0.2898
130	O	4.9985	3.3443	7.1931	0.3595	0.2405	0.3277
131	O	2.3015	5.7814	6.7263	0.1655	0.4158	0.3064
132	O	4.0009	5.3526	9.0438	0.2878	0.3850	0.4120
133	O	5.3244	6.9750	7.1244	0.3830	0.5017	0.3245
134	O	7.4066	6.8808	8.9544	0.5327	0.4949	0.4079
135	O	4.7515	8.9639	8.9457	0.3418	0.6447	0.4075
136	Ce	3.3557	7.4721	8.2271	0.2414	0.5374	0.3748
137	Ce	2.9068	3.7640	7.7973	0.2091	0.2707	0.3552

138 Ce	6.2443	5.1684	8.2533	0.4491	0.3717	0.3760
139 Sm	6.6408	8.6239	8.0279	0.4776	0.6203	0.3657

/db/jmorales/CoNi-alloy/metal-SDC-interface/CoNi-Ce_3SmO_7/CoNi-Ce_3SmO_7-conf4d

a = 13.9033384264
b = 13.9033384264
c = 21.9516692133
alpha = 90.0
beta = 90.0
gamma = 90.0

	Atom	X	Y	Z	X	Y	Z
1	Ni	0.0000	0.0000	0.0000	0.0000	0.0000	0.0000
2	Ni	0.0000	3.4758	0.0000	0.0000	0.2500	0.0000
3	Ni	3.4758	0.0000	0.0000	0.2500	0.0000	0.0000
4	Ni	3.4758	3.4758	0.0000	0.2500	0.2500	0.0000
5	Ni	0.0000	6.9517	0.0000	0.0000	0.5000	0.0000
6	Ni	0.0000	10.4275	0.0000	0.0000	0.7500	0.0000
7	Ni	3.4758	6.9517	0.0000	0.2500	0.5000	0.0000
8	Ni	3.4758	10.4275	0.0000	0.2500	0.7500	0.0000
9	Ni	6.9517	0.0000	0.0000	0.5000	0.0000	0.0000
10	Ni	6.9517	3.4758	0.0000	0.5000	0.2500	0.0000
11	Ni	10.4275	0.0000	0.0000	0.7500	0.0000	0.0000
12	Ni	10.4275	3.4758	0.0000	0.7500	0.2500	0.0000
13	Ni	6.9517	6.9517	0.0000	0.5000	0.5000	0.0000
14	Ni	6.9517	10.4275	0.0000	0.5000	0.7500	0.0000
15	Ni	10.4275	6.9517	0.0000	0.7500	0.5000	0.0000
16	Ni	10.4275	10.4275	0.0000	0.7500	0.7500	0.0000
17	Ni	0.0000	1.7379	1.7379	0.0000	0.1250	0.0792
18	Ni	0.0000	5.2138	1.7379	0.0000	0.3750	0.0792
19	Ni	3.4758	1.7379	1.7379	0.2500	0.1250	0.0792
20	Ni	3.4758	5.2138	1.7379	0.2500	0.3750	0.0792
21	Ni	0.0000	8.6896	1.7379	0.0000	0.6250	0.0792
22	Ni	0.0000	12.1654	1.7379	0.0000	0.8750	0.0792
23	Ni	3.4758	8.6896	1.7379	0.2500	0.6250	0.0792
24	Ni	3.4758	12.1654	1.7379	0.2500	0.8750	0.0792
25	Ni	6.9517	1.7379	1.7379	0.5000	0.1250	0.0792
26	Ni	6.9517	5.2138	1.7379	0.5000	0.3750	0.0792
27	Ni	10.4275	1.7379	1.7379	0.7500	0.1250	0.0792
28	Ni	10.4275	5.2138	1.7379	0.7500	0.3750	0.0792
29	Ni	6.9517	8.6896	1.7379	0.5000	0.6250	0.0792
30	Ni	6.9517	12.1654	1.7379	0.5000	0.8750	0.0792
31	Ni	10.4275	8.6896	1.7379	0.7500	0.6250	0.0792
32	Ni	10.4275	12.1654	1.7379	0.7500	0.8750	0.0792
33	Ni	13.8981	0.0081	3.5083	0.9996	0.0006	0.1598
34	Ni	13.8850	3.4782	3.5191	0.9987	0.2502	0.1603
35	Ni	3.4706	-0.0010	3.5068	0.2496	-0.0001	0.1598
36	Ni	3.4652	3.4919	3.5167	0.2492	0.2512	0.1602
37	Ni	13.9014	6.9442	3.5050	0.9999	0.4995	0.1597
38	Ni	0.0007	10.4369	3.5020	0.0000	0.7507	0.1595
39	Ni	3.4606	6.9502	3.4981	0.2489	0.4999	0.1594

40	Ni	3.4802	10.4330	3.4978	0.2503	0.7504	0.1593
41	Ni	6.9539	0.0119	3.5249	0.5002	0.0009	0.1606
42	Ni	6.9850	3.4577	3.4348	0.5024	0.2487	0.1565
43	Ni	10.4313	0.0125	3.5205	0.7503	0.0009	0.1604
44	Ni	10.4226	3.4706	3.4611	0.7496	0.2496	0.1577
45	Ni	6.9275	6.9366	3.5262	0.4983	0.4989	0.1606
46	Ni	6.9449	10.4012	3.5210	0.4995	0.7481	0.1604
47	Ni	10.4363	6.9504	3.4962	0.7506	0.4999	0.1593
48	Ni	10.4391	10.4437	3.4786	0.7508	0.7512	0.1585
49	Ni	13.8815	1.7511	5.2210	0.9984	0.1260	0.2378
50	Ni	13.8952	5.2116	5.2178	0.9994	0.3748	0.2377
51	Ni	3.4829	1.7448	5.2124	0.2505	0.1255	0.2374
52	Ni	3.4505	5.1900	5.3165	0.2482	0.3733	0.2422
53	Ni	13.8995	8.7071	5.2034	0.9997	0.6263	0.2370
54	Ni	0.0030	12.1670	5.2122	0.0002	0.8751	0.2374
55	Ni	3.4536	8.7202	5.1963	0.2484	0.6272	0.2367
56	Ni	3.4774	12.1661	5.2089	0.2501	0.8750	0.2373
57	Ni	6.9709	1.7148	5.2310	0.5014	0.1233	0.2383
58	Ni	6.9549	5.1733	5.1573	0.5002	0.3721	0.2349
59	Ni	10.4122	1.6934	5.2423	0.7489	0.1218	0.2388
60	Ni	10.4153	5.2662	5.1961	0.7491	0.3788	0.2367
61	Ni	6.9655	8.6973	5.2878	0.5010	0.6256	0.2409
62	Ni	6.9453	12.1471	5.2140	0.4995	0.8737	0.2375
63	Ni	10.4431	8.7240	5.1485	0.7511	0.6275	0.2345
64	Ni	10.4307	12.1647	5.2141	0.7502	0.8749	0.2375
65	Co	1.7379	1.7379	0.0000	0.1250	0.1250	0.0000
66	Co	1.7379	5.2138	0.0000	0.1250	0.3750	0.0000
67	Co	5.2138	1.7379	0.0000	0.3750	0.1250	0.0000
68	Co	5.2138	5.2138	0.0000	0.3750	0.3750	0.0000
69	Co	1.7379	8.6896	0.0000	0.1250	0.6250	0.0000
70	Co	1.7379	12.1654	0.0000	0.1250	0.8750	0.0000
71	Co	5.2138	8.6896	0.0000	0.3750	0.6250	0.0000
72	Co	5.2138	12.1654	0.0000	0.3750	0.8750	0.0000
73	Co	8.6896	1.7379	0.0000	0.6250	0.1250	0.0000
74	Co	8.6896	5.2138	0.0000	0.6250	0.3750	0.0000
75	Co	12.1654	1.7379	0.0000	0.8750	0.1250	0.0000
76	Co	12.1654	5.2138	0.0000	0.8750	0.3750	0.0000
77	Co	8.6896	8.6896	0.0000	0.6250	0.6250	0.0000
78	Co	8.6896	12.1654	0.0000	0.6250	0.8750	0.0000
79	Co	12.1654	8.6896	0.0000	0.8750	0.6250	0.0000
80	Co	12.1654	12.1654	0.0000	0.8750	0.8750	0.0000
81	Co	1.7379	0.0000	1.7379	0.1250	0.0000	0.0792
82	Co	1.7379	3.4758	1.7379	0.1250	0.2500	0.0792
83	Co	5.2138	0.0000	1.7379	0.3750	0.0000	0.0792
84	Co	5.2138	3.4758	1.7379	0.3750	0.2500	0.0792
85	Co	1.7379	6.9517	1.7379	0.1250	0.5000	0.0792
86	Co	1.7379	10.4275	1.7379	0.1250	0.7500	0.0792
87	Co	5.2138	6.9517	1.7379	0.3750	0.5000	0.0792
88	Co	5.2138	10.4275	1.7379	0.3750	0.7500	0.0792

89	Co	8.6896	0.0000	1.7379	0.6250	0.0000	0.0792
90	Co	8.6896	3.4758	1.7379	0.6250	0.2500	0.0792
91	Co	12.1654	0.0000	1.7379	0.8750	0.0000	0.0792
92	Co	12.1654	3.4758	1.7379	0.8750	0.2500	0.0792
93	Co	8.6896	6.9517	1.7379	0.6250	0.5000	0.0792
94	Co	8.6896	10.4275	1.7379	0.6250	0.7500	0.0792
95	Co	12.1654	6.9517	1.7379	0.8750	0.5000	0.0792
96	Co	12.1654	10.4275	1.7379	0.8750	0.7500	0.0792
97	Co	1.7311	1.7403	3.5584	0.1245	0.1252	0.1621
98	Co	1.7456	5.2072	3.5758	0.1255	0.3745	0.1629
99	Co	5.2156	1.7037	3.5097	0.3751	0.1225	0.1599
100	Co	5.1935	5.2082	3.5126	0.3735	0.3746	0.1600
101	Co	1.7326	8.6944	3.5419	0.1246	0.6253	0.1614
102	Co	1.7360	12.1751	3.5538	0.1249	0.8757	0.1619
103	Co	5.2279	8.6724	3.5765	0.3760	0.6238	0.1629
104	Co	5.2190	12.1640	3.5555	0.3754	0.8749	0.1620
105	Co	8.6958	1.7358	3.5473	0.6254	0.1248	0.1616
106	Co	8.6934	5.2207	3.5076	0.6253	0.3755	0.1598
107	Co	12.1547	1.7641	3.5748	0.8742	0.1269	0.1628
108	Co	12.1694	5.2067	3.5535	0.8753	0.3745	0.1619
109	Co	8.6838	8.7037	3.5070	0.6246	0.6260	0.1598
110	Co	8.6829	12.1746	3.5316	0.6245	0.8757	0.1609
111	Co	12.1838	8.6947	3.5252	0.8763	0.6254	0.1606
112	Co	12.1694	12.1700	3.5545	0.8753	0.8753	0.1619
113	Co	1.7335	0.0202	5.2314	0.1247	0.0015	0.2383
114	Co	1.7187	3.4574	5.2282	0.1236	0.2487	0.2382
115	Co	5.2032	0.0041	5.2289	0.3742	0.0003	0.2382
116	Co	5.2452	3.4243	5.1041	0.3773	0.2463	0.2325
117	Co	1.6923	6.9697	5.2153	0.1217	0.5013	0.2376
118	Co	1.7400	10.4335	5.2215	0.1252	0.7504	0.2379
119	Co	5.1546	6.9172	5.2367	0.3707	0.4975	0.2386
120	Co	5.2004	10.4108	5.2322	0.3740	0.7488	0.2384
121	Co	8.6866	13.8648	5.2272	0.6248	0.9972	0.2381
122	Co	8.7213	3.4656	5.2615	0.6273	0.2493	0.2397
123	Co	12.1746	13.8968	5.2291	0.8757	0.9995	0.2382
124	Co	12.0716	3.4974	5.3361	0.8683	0.2516	0.2431
125	Co	8.7247	6.9754	5.1806	0.6275	0.5017	0.2360
126	Co	8.7219	10.4516	5.1782	0.6273	0.7517	0.2359
127	Co	12.1663	6.9803	5.2211	0.8751	0.5021	0.2378
128	Co	12.1574	10.4253	5.2255	0.8744	0.7498	0.2380
129	O	6.9903	3.6570	6.4785	0.5028	0.2630	0.2951
130	O	10.3510	3.7823	6.4410	0.7445	0.2720	0.2934
131	O	4.3587	6.1866	6.8614	0.3135	0.4450	0.3126
132	O	6.5823	5.6742	8.7398	0.4734	0.4081	0.3981
133	O	7.8351	7.5345	6.7923	0.5635	0.5419	0.3094
134	O	9.8768	7.0277	8.7121	0.7104	0.5055	0.3969
135	O	7.3727	9.1899	8.9739	0.5303	0.6610	0.4088
136	Ce	5.9183	7.8585	8.1208	0.4257	0.5652	0.3699
137	Ce	5.1571	4.2845	7.7518	0.3709	0.3082	0.3531

138 Ce	8.8209	5.3911	7.8394	0.6344	0.3878	0.3571
139 Sm	9.2292	8.9138	7.9562	0.6638	0.6411	0.3624

/db/jmorales/CoNi-alloy/metal-SDC-interface/Ni-Ce_3SmO_7/Ni-Ce_3SmO_7-conf-CoNi

a =	13.9019837372
b =	13.9019837372
c =	21.9509918687
alpha =	90.0
beta =	90.0
gamma =	90.0

	Atom	X	Y	Z	X	Y	Z
1	Ni	0.0000	0.0000	0.0000	0.0000	0.0000	0.0000
2	Ni	1.7377	1.7377	0.0000	0.1250	0.1250	0.0000
3	Ni	0.0000	3.4755	0.0000	0.0000	0.2500	0.0000
4	Ni	1.7377	5.2132	0.0000	0.1250	0.3750	0.0000
5	Ni	3.4755	0.0000	0.0000	0.2500	0.0000	0.0000
6	Ni	5.2132	1.7377	0.0000	0.3750	0.1250	0.0000
7	Ni	3.4755	3.4755	0.0000	0.2500	0.2500	0.0000
8	Ni	5.2132	5.2132	0.0000	0.3750	0.3750	0.0000
9	Ni	0.0000	6.9510	0.0000	0.0000	0.5000	0.0000
10	Ni	1.7377	8.6887	0.0000	0.1250	0.6250	0.0000
11	Ni	0.0000	10.4265	0.0000	0.0000	0.7500	0.0000
12	Ni	1.7377	12.1642	0.0000	0.1250	0.8750	0.0000
13	Ni	3.4755	6.9510	0.0000	0.2500	0.5000	0.0000
14	Ni	5.2132	8.6887	0.0000	0.3750	0.6250	0.0000
15	Ni	3.4755	10.4265	0.0000	0.2500	0.7500	0.0000
16	Ni	5.2132	12.1642	0.0000	0.3750	0.8750	0.0000
17	Ni	6.9510	0.0000	0.0000	0.5000	0.0000	0.0000
18	Ni	8.6887	1.7377	0.0000	0.6250	0.1250	0.0000
19	Ni	6.9510	3.4755	0.0000	0.5000	0.2500	0.0000
20	Ni	8.6887	5.2132	0.0000	0.6250	0.3750	0.0000
21	Ni	10.4265	0.0000	0.0000	0.7500	0.0000	0.0000
22	Ni	12.1642	1.7377	0.0000	0.8750	0.1250	0.0000
23	Ni	10.4265	3.4755	0.0000	0.7500	0.2500	0.0000
24	Ni	12.1642	5.2132	0.0000	0.8750	0.3750	0.0000
25	Ni	6.9510	6.9510	0.0000	0.5000	0.5000	0.0000
26	Ni	8.6887	8.6887	0.0000	0.6250	0.6250	0.0000
27	Ni	6.9510	10.4265	0.0000	0.5000	0.7500	0.0000
28	Ni	8.6887	12.1642	0.0000	0.6250	0.8750	0.0000
29	Ni	10.4265	6.9510	0.0000	0.7500	0.5000	0.0000
30	Ni	12.1642	8.6887	0.0000	0.8750	0.6250	0.0000
31	Ni	10.4265	10.4265	0.0000	0.7500	0.7500	0.0000
32	Ni	12.1642	12.1642	0.0000	0.8750	0.8750	0.0000
33	Ni	0.0000	1.7377	1.7377	0.0000	0.1250	0.0792
34	Ni	1.7377	0.0000	1.7377	0.1250	0.0000	0.0792
35	Ni	0.0000	5.2132	1.7377	0.0000	0.3750	0.0792
36	Ni	1.7377	3.4755	1.7377	0.1250	0.2500	0.0792
37	Ni	3.4755	1.7377	1.7377	0.2500	0.1250	0.0792
38	Ni	5.2132	0.0000	1.7377	0.3750	0.0000	0.0792
39	Ni	3.4755	5.2132	1.7377	0.2500	0.3750	0.0792
40	Ni	5.2132	3.4755	1.7377	0.3750	0.2500	0.0792

41	Ni	0.0000	8.6887	1.7377	0.0000	0.6250	0.0792
42	Ni	1.7377	6.9510	1.7377	0.1250	0.5000	0.0792
43	Ni	0.0000	12.1642	1.7377	0.0000	0.8750	0.0792
44	Ni	1.7377	10.4265	1.7377	0.1250	0.7500	0.0792
45	Ni	3.4755	8.6887	1.7377	0.2500	0.6250	0.0792
46	Ni	5.2132	6.9510	1.7377	0.3750	0.5000	0.0792
47	Ni	3.4755	12.1642	1.7377	0.2500	0.8750	0.0792
48	Ni	5.2132	10.4265	1.7377	0.3750	0.7500	0.0792
49	Ni	6.9510	1.7377	1.7377	0.5000	0.1250	0.0792
50	Ni	8.6887	0.0000	1.7377	0.6250	0.0000	0.0792
51	Ni	6.9510	5.2132	1.7377	0.5000	0.3750	0.0792
52	Ni	8.6887	3.4755	1.7377	0.6250	0.2500	0.0792
53	Ni	10.4265	1.7377	1.7377	0.7500	0.1250	0.0792
54	Ni	12.1642	0.0000	1.7377	0.8750	0.0000	0.0792
55	Ni	10.4265	5.2132	1.7377	0.7500	0.3750	0.0792
56	Ni	12.1642	3.4755	1.7377	0.8750	0.2500	0.0792
57	Ni	6.9510	8.6887	1.7377	0.5000	0.6250	0.0792
58	Ni	8.6887	6.9510	1.7377	0.6250	0.5000	0.0792
59	Ni	6.9510	12.1642	1.7377	0.5000	0.8750	0.0792
60	Ni	8.6887	10.4265	1.7377	0.6250	0.7500	0.0792
61	Ni	10.4265	8.6887	1.7377	0.7500	0.6250	0.0792
62	Ni	12.1642	6.9510	1.7377	0.8750	0.5000	0.0792
63	Ni	10.4265	12.1642	1.7377	0.7500	0.8750	0.0792
64	Ni	12.1642	10.4265	1.7377	0.8750	0.7500	0.0792
65	Ni	0.0027	0.0027	3.5083	0.0002	0.0002	0.1598
66	Ni	1.7353	1.7351	3.5085	0.1248	0.1248	0.1598
67	Ni	13.8930	3.4655	3.5086	0.9994	0.2493	0.1598
68	Ni	1.7052	5.1996	3.4730	0.1227	0.3740	0.1582
69	Ni	3.4785	0.0018	3.5119	0.2502	0.0001	0.1600
70	Ni	5.2217	1.7491	3.5330	0.3756	0.1258	0.1610
71	Ni	3.4927	3.4400	3.4981	0.2512	0.2474	0.1594
72	Ni	5.2140	5.2055	3.4923	0.3751	0.3744	0.1591
73	Ni	13.8992	6.9495	3.5022	0.9998	0.4999	0.1595
74	Ni	1.7432	8.6938	3.5214	0.1254	0.6254	0.1604
75	Ni	13.8868	10.4292	3.5251	0.9989	0.7502	0.1606
76	Ni	1.7411	12.1631	3.5086	0.1252	0.8749	0.1598
77	Ni	3.4614	6.9753	3.4832	0.2490	0.5017	0.1587
78	Ni	5.2205	8.7015	3.5040	0.3755	0.6259	0.1596
79	Ni	3.4703	10.4036	3.5308	0.2496	0.7484	0.1609
80	Ni	5.2105	12.1663	3.5030	0.3748	0.8751	0.1596
81	Ni	6.9486	13.9002	3.5058	0.4998	0.9999	0.1597
82	Ni	8.6982	1.7293	3.5026	0.6257	0.1244	0.1596
83	Ni	6.9472	3.4668	3.5075	0.4997	0.2494	0.1598
84	Ni	8.6680	5.2301	3.5248	0.6235	0.3762	0.1606
85	Ni	10.4348	13.8808	3.5280	0.7506	0.9985	0.1607
86	Ni	12.1612	1.7348	3.5088	0.8748	0.1248	0.1598
87	Ni	10.4281	3.4785	3.5033	0.7501	0.2502	0.1596
88	Ni	12.1617	5.2126	3.5088	0.8748	0.3750	0.1598
89	Ni	6.9289	6.9394	3.5157	0.4984	0.4992	0.1602

90	Ni	8.6923	8.6831	3.4762	0.6253	0.6246	0.1584
91	Ni	6.9756	10.4244	3.5147	0.5018	0.7498	0.1601
92	Ni	8.6947	12.1754	3.4989	0.6254	0.8758	0.1594
93	Ni	10.4155	6.9562	3.5206	0.7492	0.5004	0.1604
94	Ni	12.1825	8.6868	3.4910	0.8763	0.6249	0.1590
95	Ni	10.4394	10.4401	3.4368	0.7509	0.7510	0.1566
96	Ni	12.1692	12.1660	3.5089	0.8754	0.8751	0.1599
97	Ni	0.0025	1.7356	5.2282	0.0002	0.1248	0.2382
98	Ni	1.7380	13.8988	5.2256	0.1250	0.9998	0.2381
99	Ni	13.8984	5.2009	5.2249	0.9997	0.3741	0.2380
100	Ni	1.7390	3.4740	5.2190	0.1251	0.2499	0.2378
101	Ni	3.4666	1.7310	5.2335	0.2494	0.1245	0.2384
102	Ni	5.2131	-0.0002	5.2275	0.3750	-0.0000	0.2381
103	Ni	3.4303	5.1810	5.0813	0.2468	0.3727	0.2315
104	Ni	5.1978	3.4355	5.3750	0.3739	0.2471	0.2449
105	Ni	0.0077	8.6797	5.2222	0.0006	0.6243	0.2379
106	Ni	1.7105	6.9148	5.1995	0.1230	0.4974	0.2369
107	Ni	0.0001	12.1752	5.2236	0.0000	0.8758	0.2380
108	Ni	1.7173	10.4426	5.2305	0.1235	0.7512	0.2383
109	Ni	3.4349	8.7376	5.3800	0.2471	0.6285	0.2451
110	Ni	5.1660	6.9660	5.2221	0.3716	0.5011	0.2379
111	Ni	3.4805	12.1560	5.2300	0.2504	0.8744	0.2383
112	Ni	5.2316	10.4427	5.1994	0.3763	0.7512	0.2369
113	Ni	6.9816	1.7096	5.2144	0.5022	0.1230	0.2375
114	Ni	8.6929	13.8944	5.2229	0.6253	0.9995	0.2379
115	Ni	6.9280	5.1900	5.2683	0.4983	0.3733	0.2400
116	Ni	8.7144	3.4734	5.2027	0.6268	0.2499	0.2370
117	Ni	10.4248	1.7332	5.2270	0.7499	0.1247	0.2381
118	Ni	12.1715	0.0075	5.2261	0.8755	0.0005	0.2381
119	Ni	10.4205	5.2112	5.2246	0.7496	0.3749	0.2380
120	Ni	12.1591	3.4735	5.2260	0.8746	0.2499	0.2381
121	Ni	6.9678	8.7303	5.2396	0.5012	0.6280	0.2387
122	Ni	8.7072	6.9414	5.2814	0.6263	0.4993	0.2406
123	Ni	6.9486	12.1626	5.2219	0.4998	0.8749	0.2379
124	Ni	8.7156	10.4559	5.2177	0.6269	0.7521	0.2377
125	Ni	10.4611	8.6955	5.1815	0.7525	0.6255	0.2360
126	Ni	12.1648	6.9460	5.2195	0.8750	0.4996	0.2378
127	Ni	10.4457	12.1664	5.2916	0.7514	0.8752	0.2411
128	Ni	12.1846	10.4248	5.2900	0.8765	0.7499	0.2410
129	O	6.0852	4.3903	6.8265	0.4377	0.3158	0.3110
130	O	4.1629	7.6327	6.7758	0.2994	0.5490	0.3087
131	O	5.9596	6.6067	8.7799	0.4287	0.4752	0.4000
132	O	7.8078	7.8466	6.8076	0.5616	0.5644	0.3101
133	O	10.4249	10.3605	6.2835	0.7499	0.7453	0.2863
134	O	9.5368	6.9696	8.8726	0.6860	0.5013	0.4042
135	O	7.6736	9.7625	8.9154	0.5520	0.7022	0.4061
136	Ce	5.9357	8.8538	8.0210	0.4270	0.6369	0.3654
137	Ce	4.3158	5.5215	7.6861	0.3104	0.3972	0.3501
138	Ce	8.0203	5.7308	8.0348	0.5769	0.4122	0.3660

139 Sm	9.4319	8.9601	8.0432	0.6785	0.6445	0.3664

/db/jmorales/CoNi-alloy/metal-SDC-interface/CoNi-Ce_3SmO_7/CoNi-Ce_3SmO_7-conf-CoNi

a = 13.9033384264
b = 13.9033384264
c = 21.9516692133
alpha = 90.0
beta = 90.0
gamma = 90.0

	Atom	X	Y	Z	X	Y	Z
1	Ni	0.0000	0.0000	0.0000	0.0000	0.0000	0.0000
2	Ni	0.0000	3.4758	0.0000	0.0000	0.2500	0.0000
3	Ni	3.4758	0.0000	0.0000	0.2500	0.0000	0.0000
4	Ni	3.4758	3.4758	0.0000	0.2500	0.2500	0.0000
5	Ni	0.0000	6.9517	0.0000	0.0000	0.5000	0.0000
6	Ni	0.0000	10.4275	0.0000	0.0000	0.7500	0.0000
7	Ni	3.4758	6.9517	0.0000	0.2500	0.5000	0.0000
8	Ni	3.4758	10.4275	0.0000	0.2500	0.7500	0.0000
9	Ni	6.9517	0.0000	0.0000	0.5000	0.0000	0.0000
10	Ni	6.9517	3.4758	0.0000	0.5000	0.2500	0.0000
11	Ni	10.4275	0.0000	0.0000	0.7500	0.0000	0.0000
12	Ni	10.4275	3.4758	0.0000	0.7500	0.2500	0.0000
13	Ni	6.9517	6.9517	0.0000	0.5000	0.5000	0.0000
14	Ni	6.9517	10.4275	0.0000	0.5000	0.7500	0.0000
15	Ni	10.4275	6.9517	0.0000	0.7500	0.5000	0.0000
16	Ni	10.4275	10.4275	0.0000	0.7500	0.7500	0.0000
17	Ni	0.0000	1.7379	1.7379	0.0000	0.1250	0.0792
18	Ni	0.0000	5.2138	1.7379	0.0000	0.3750	0.0792
19	Ni	3.4758	1.7379	1.7379	0.2500	0.1250	0.0792
20	Ni	3.4758	5.2138	1.7379	0.2500	0.3750	0.0792
21	Ni	0.0000	8.6896	1.7379	0.0000	0.6250	0.0792
22	Ni	0.0000	12.1654	1.7379	0.0000	0.8750	0.0792
23	Ni	3.4758	8.6896	1.7379	0.2500	0.6250	0.0792
24	Ni	3.4758	12.1654	1.7379	0.2500	0.8750	0.0792
25	Ni	6.9517	1.7379	1.7379	0.5000	0.1250	0.0792
26	Ni	6.9517	5.2138	1.7379	0.5000	0.3750	0.0792
27	Ni	10.4275	1.7379	1.7379	0.7500	0.1250	0.0792
28	Ni	10.4275	5.2138	1.7379	0.7500	0.3750	0.0792
29	Ni	6.9517	8.6896	1.7379	0.5000	0.6250	0.0792
30	Ni	6.9517	12.1654	1.7379	0.5000	0.8750	0.0792
31	Ni	10.4275	8.6896	1.7379	0.7500	0.6250	0.0792
32	Ni	10.4275	12.1654	1.7379	0.7500	0.8750	0.0792
33	Ni	0.0159	0.0007	3.5135	0.0011	0.0001	0.1601
34	Ni	0.0108	3.4753	3.5236	0.0008	0.2500	0.1605
35	Ni	3.4528	13.8712	3.4716	0.2483	0.9977	0.1581
36	Ni	3.4726	3.4872	3.4911	0.2498	0.2508	0.1590
37	Ni	13.8894	6.9594	3.4922	0.9990	0.5006	0.1591
38	Ni	13.9014	10.4241	3.5041	0.9999	0.7498	0.1596
39	Ni	3.4944	6.9322	3.5164	0.2513	0.4986	0.1602

40	Ni	3.4831	10.4165	3.5031	0.2505	0.7492	0.1596
41	Ni	6.9540	0.0159	3.5195	0.5002	0.0011	0.1603
42	Ni	6.9565	3.4836	3.5067	0.5003	0.2506	0.1597
43	Ni	10.4322	0.0051	3.5073	0.7503	0.0004	0.1598
44	Ni	10.4370	3.4664	3.4970	0.7507	0.2493	0.1593
45	Ni	6.9596	6.9547	3.4745	0.5006	0.5002	0.1583
46	Ni	6.9504	10.4423	3.4845	0.4999	0.7511	0.1587
47	Ni	10.4424	6.9495	3.4850	0.7511	0.4998	0.1588
48	Ni	10.4334	10.4382	3.4989	0.7504	0.7508	0.1594
49	Ni	0.0391	1.7323	5.2776	0.0028	0.1246	0.2404
50	Ni	13.8785	5.2459	5.1871	0.9982	0.3773	0.2363
51	Ni	3.4434	1.6968	5.0843	0.2477	0.1220	0.2316
52	Ni	3.4553	5.2112	5.2274	0.2485	0.3748	0.2381
53	Ni	13.8981	8.6917	5.2117	0.9996	0.6252	0.2374
54	Ni	0.0041	12.1618	5.2049	0.0003	0.8747	0.2371
55	Ni	3.4789	8.6781	5.2199	0.2502	0.6242	0.2378
56	Ni	3.4766	12.1407	5.2015	0.2501	0.8732	0.2370
57	Ni	6.9701	1.7005	5.3688	0.5013	0.1223	0.2446
58	Ni	7.0058	5.2358	5.2380	0.5039	0.3766	0.2386
59	Ni	10.4248	1.7391	5.2157	0.7498	0.1251	0.2376
60	Ni	10.4202	5.2153	5.2079	0.7495	0.3751	0.2372
61	Ni	6.9923	8.7121	5.1452	0.5029	0.6266	0.2344
62	Ni	6.9603	12.1668	5.2085	0.5006	0.8751	0.2373
63	Ni	10.4237	8.7076	5.2123	0.7497	0.6263	0.2374
64	Ni	10.4268	12.1657	5.2087	0.7499	0.8750	0.2373
65	Co	1.7379	1.7379	0.0000	0.1250	0.1250	0.0000
66	Co	1.7379	5.2138	0.0000	0.1250	0.3750	0.0000
67	Co	5.2138	1.7379	0.0000	0.3750	0.1250	0.0000
68	Co	5.2138	5.2138	0.0000	0.3750	0.3750	0.0000
69	Co	1.7379	8.6896	0.0000	0.1250	0.6250	0.0000
70	Co	1.7379	12.1654	0.0000	0.1250	0.8750	0.0000
71	Co	5.2138	8.6896	0.0000	0.3750	0.6250	0.0000
72	Co	5.2138	12.1654	0.0000	0.3750	0.8750	0.0000
73	Co	8.6896	1.7379	0.0000	0.6250	0.1250	0.0000
74	Co	8.6896	5.2138	0.0000	0.6250	0.3750	0.0000
75	Co	12.1654	1.7379	0.0000	0.8750	0.1250	0.0000
76	Co	12.1654	5.2138	0.0000	0.8750	0.3750	0.0000
77	Co	8.6896	8.6896	0.0000	0.6250	0.6250	0.0000
78	Co	8.6896	12.1654	0.0000	0.6250	0.8750	0.0000
79	Co	12.1654	8.6896	0.0000	0.8750	0.6250	0.0000
80	Co	12.1654	12.1654	0.0000	0.8750	0.8750	0.0000
81	Co	1.7379	0.0000	1.7379	0.1250	0.0000	0.0792
82	Co	1.7379	3.4758	1.7379	0.1250	0.2500	0.0792
83	Co	5.2138	0.0000	1.7379	0.3750	0.0000	0.0792
84	Co	5.2138	3.4758	1.7379	0.3750	0.2500	0.0792
85	Co	1.7379	6.9517	1.7379	0.1250	0.5000	0.0792
86	Co	1.7379	10.4275	1.7379	0.1250	0.7500	0.0792
87	Co	5.2138	6.9517	1.7379	0.3750	0.5000	0.0792
88	Co	5.2138	10.4275	1.7379	0.3750	0.7500	0.0792

89	Co	8.6896	0.0000	1.7379	0.6250	0.0000	0.0792
90	Co	8.6896	3.4758	1.7379	0.6250	0.2500	0.0792
91	Co	12.1654	0.0000	1.7379	0.8750	0.0000	0.0792
92	Co	12.1654	3.4758	1.7379	0.8750	0.2500	0.0792
93	Co	8.6896	6.9517	1.7379	0.6250	0.5000	0.0792
94	Co	8.6896	10.4275	1.7379	0.6250	0.7500	0.0792
95	Co	12.1654	6.9517	1.7379	0.8750	0.5000	0.0792
96	Co	12.1654	10.4275	1.7379	0.8750	0.7500	0.0792
97	Co	1.7071	1.7516	3.4553	0.1228	0.1260	0.1574
98	Co	1.7288	5.2129	3.5327	0.1243	0.3749	0.1609
99	Co	5.2248	1.7091	3.5327	0.3758	0.1229	0.1609
100	Co	5.2082	5.2072	3.5617	0.3746	0.3745	0.1623
101	Co	1.7433	8.6900	3.5484	0.1254	0.6250	0.1616
102	Co	1.7355	12.1517	3.5469	0.1248	0.8740	0.1616
103	Co	5.2114	8.6829	3.5531	0.3748	0.6245	0.1619
104	Co	5.2166	12.1519	3.5519	0.3752	0.8740	0.1618
105	Co	8.6702	1.7293	3.5685	0.6236	0.1244	0.1626
106	Co	8.6886	5.2132	3.5433	0.6249	0.3750	0.1614
107	Co	12.1937	1.7346	3.5891	0.8770	0.1248	0.1635
108	Co	12.1590	5.2133	3.5534	0.8745	0.3750	0.1619
109	Co	8.7119	8.7051	3.5121	0.6266	0.6261	0.1600
110	Co	8.6917	12.1669	3.5470	0.6252	0.8751	0.1616
111	Co	12.1609	8.6966	3.5549	0.8747	0.6255	0.1619
112	Co	12.1697	12.1697	3.5543	0.8753	0.8753	0.1619
113	Co	1.7578	13.8678	5.3066	0.1264	0.9974	0.2417
114	Co	1.7014	3.5343	5.3688	0.1224	0.2542	0.2446
115	Co	5.1800	13.8516	5.1875	0.3726	0.9963	0.2363
116	Co	5.2343	3.3977	5.1734	0.3765	0.2444	0.2357
117	Co	1.7246	6.9902	5.1882	0.1240	0.5028	0.2363
118	Co	1.7409	10.4183	5.2267	0.1252	0.7493	0.2381
119	Co	5.2311	6.9672	5.2358	0.3762	0.5011	0.2385
120	Co	5.2311	10.4106	5.2353	0.3762	0.7488	0.2385
121	Co	8.7056	13.8947	5.2291	0.6261	0.9994	0.2382
122	Co	8.7186	3.4833	5.1891	0.6271	0.2505	0.2364
123	Co	12.1691	13.8976	5.2211	0.8753	0.9996	0.2378
124	Co	12.1696	3.4758	5.2167	0.8753	0.2500	0.2376
125	Co	8.7314	6.9993	5.1655	0.6280	0.5034	0.2353
126	Co	8.6843	10.4225	5.2235	0.6246	0.7496	0.2380
127	Co	12.1542	6.9615	5.2251	0.8742	0.5007	0.2380
128	Co	12.1692	10.4342	5.2269	0.8753	0.7505	0.2381
129	O	1.8384	1.6254	6.3502	0.1322	0.1169	0.2893
130	O	5.8148	2.5069	6.7940	0.4182	0.1803	0.3095
131	O	2.6934	4.4616	6.8724	0.1937	0.3209	0.3131
132	O	4.8309	4.2818	8.8622	0.3475	0.3080	0.4037
133	O	6.0228	6.0607	6.8190	0.4332	0.4359	0.3106
134	O	8.0024	6.0266	8.8425	0.5756	0.4335	0.4028
135	O	5.2450	7.8502	8.8640	0.3772	0.5646	0.4038
136	Ce	3.9578	6.3077	8.0833	0.2847	0.4537	0.3682
137	Ce	3.6936	2.6262	7.7451	0.2657	0.1889	0.3528

138 Ce	7.0536	4.2458	8.0728	0.5073	0.3054	0.3678
139 Sm	7.1548	7.7484	7.9410	0.5146	0.5573	0.3617

/db/jmorales/CoNi-alloy/metal-SDC-interface/CoNi-Ce_3SmO_7/CoNi-Ce_3SmO_7-conf3d

a = 13.9033384264
b = 13.9033384264
c = 21.9516692133
alpha = 90.0
beta = 90.0
gamma = 90.0

	Atom	X	Y	Z	X	Y	Z
1	Ni	0.0000	0.0000	0.0000	0.0000	0.0000	0.0000
2	Ni	0.0000	3.4758	0.0000	0.0000	0.2500	0.0000
3	Ni	3.4758	0.0000	0.0000	0.2500	0.0000	0.0000
4	Ni	3.4758	3.4758	0.0000	0.2500	0.2500	0.0000
5	Ni	0.0000	6.9517	0.0000	0.0000	0.5000	0.0000
6	Ni	0.0000	10.4275	0.0000	0.0000	0.7500	0.0000
7	Ni	3.4758	6.9517	0.0000	0.2500	0.5000	0.0000
8	Ni	3.4758	10.4275	0.0000	0.2500	0.7500	0.0000
9	Ni	6.9517	0.0000	0.0000	0.5000	0.0000	0.0000
10	Ni	6.9517	3.4758	0.0000	0.5000	0.2500	0.0000
11	Ni	10.4275	0.0000	0.0000	0.7500	0.0000	0.0000
12	Ni	10.4275	3.4758	0.0000	0.7500	0.2500	0.0000
13	Ni	6.9517	6.9517	0.0000	0.5000	0.5000	0.0000
14	Ni	6.9517	10.4275	0.0000	0.5000	0.7500	0.0000
15	Ni	10.4275	6.9517	0.0000	0.7500	0.5000	0.0000
16	Ni	10.4275	10.4275	0.0000	0.7500	0.7500	0.0000
17	Ni	0.0000	1.7379	1.7379	0.0000	0.1250	0.0792
18	Ni	0.0000	5.2138	1.7379	0.0000	0.3750	0.0792
19	Ni	3.4758	1.7379	1.7379	0.2500	0.1250	0.0792
20	Ni	3.4758	5.2138	1.7379	0.2500	0.3750	0.0792
21	Ni	0.0000	8.6896	1.7379	0.0000	0.6250	0.0792
22	Ni	0.0000	12.1654	1.7379	0.0000	0.8750	0.0792
23	Ni	3.4758	8.6896	1.7379	0.2500	0.6250	0.0792
24	Ni	3.4758	12.1654	1.7379	0.2500	0.8750	0.0792
25	Ni	6.9517	1.7379	1.7379	0.5000	0.1250	0.0792
26	Ni	6.9517	5.2138	1.7379	0.5000	0.3750	0.0792
27	Ni	10.4275	1.7379	1.7379	0.7500	0.1250	0.0792
28	Ni	10.4275	5.2138	1.7379	0.7500	0.3750	0.0792
29	Ni	6.9517	8.6896	1.7379	0.5000	0.6250	0.0792
30	Ni	6.9517	12.1654	1.7379	0.5000	0.8750	0.0792
31	Ni	10.4275	8.6896	1.7379	0.7500	0.6250	0.0792
32	Ni	10.4275	12.1654	1.7379	0.7500	0.8750	0.0792
33	Ni	13.8984	0.0090	3.5086	0.9996	0.0006	0.1598
34	Ni	13.8859	3.4783	3.5191	0.9987	0.2502	0.1603
35	Ni	3.4705	13.9024	3.5067	0.2496	0.9999	0.1597
36	Ni	3.4650	3.4925	3.5168	0.2492	0.2512	0.1602
37	Ni	13.9009	6.9451	3.5051	0.9998	0.4995	0.1597
38	Ni	13.9032	10.4373	3.5026	1.0000	0.7507	0.1596
39	Ni	3.4613	6.9509	3.4994	0.2490	0.4999	0.1594

40	Ni	3.4805	10.4327	3.4986	0.2503	0.7504	0.1594
41	Ni	6.9541	0.0125	3.5273	0.5002	0.0009	0.1607
42	Ni	6.9830	3.4575	3.4341	0.5023	0.2487	0.1564
43	Ni	10.4320	0.0144	3.5219	0.7503	0.0010	0.1604
44	Ni	10.4231	3.4703	3.4605	0.7497	0.2496	0.1576
45	Ni	6.9279	6.9361	3.5262	0.4983	0.4989	0.1606
46	Ni	6.9457	10.4017	3.5223	0.4996	0.7481	0.1605
47	Ni	10.4361	6.9499	3.4958	0.7506	0.4999	0.1592
48	Ni	10.4389	10.4443	3.4787	0.7508	0.7512	0.1585
49	Ni	13.8843	1.7511	5.2213	0.9986	0.1259	0.2379
50	Ni	13.8952	5.2120	5.2173	0.9994	0.3749	0.2377
51	Ni	3.4826	1.7451	5.2119	0.2505	0.1255	0.2374
52	Ni	3.4481	5.1969	5.3162	0.2480	0.3738	0.2422
53	Ni	13.8987	8.7066	5.2048	0.9997	0.6262	0.2371
54	Ni	0.0028	12.1675	5.2125	0.0002	0.8752	0.2375
55	Ni	3.4531	8.7212	5.1962	0.2484	0.6273	0.2367
56	Ni	3.4770	12.1650	5.2086	0.2501	0.8750	0.2373
57	Ni	6.9685	1.7166	5.2335	0.5012	0.1235	0.2384
58	Ni	6.9539	5.1720	5.1573	0.5002	0.3720	0.2349
59	Ni	10.4127	1.6966	5.2437	0.7489	0.1220	0.2389
60	Ni	10.4157	5.2625	5.1936	0.7491	0.3785	0.2366
61	Ni	6.9680	8.6956	5.2888	0.5012	0.6254	0.2409
62	Ni	6.9456	12.1479	5.2150	0.4996	0.8737	0.2376
63	Ni	10.4432	8.7244	5.1470	0.7511	0.6275	0.2345
64	Ni	10.4311	12.1655	5.2139	0.7503	0.8750	0.2375
65	Co	1.7379	1.7379	0.0000	0.1250	0.1250	0.0000
66	Co	1.7379	5.2138	0.0000	0.1250	0.3750	0.0000
67	Co	5.2138	1.7379	0.0000	0.3750	0.1250	0.0000
68	Co	5.2138	5.2138	0.0000	0.3750	0.3750	0.0000
69	Co	1.7379	8.6896	0.0000	0.1250	0.6250	0.0000
70	Co	1.7379	12.1654	0.0000	0.1250	0.8750	0.0000
71	Co	5.2138	8.6896	0.0000	0.3750	0.6250	0.0000
72	Co	5.2138	12.1654	0.0000	0.3750	0.8750	0.0000
73	Co	8.6896	1.7379	0.0000	0.6250	0.1250	0.0000
74	Co	8.6896	5.2138	0.0000	0.6250	0.3750	0.0000
75	Co	12.1654	1.7379	0.0000	0.8750	0.1250	0.0000
76	Co	12.1654	5.2138	0.0000	0.8750	0.3750	0.0000
77	Co	8.6896	8.6896	0.0000	0.6250	0.6250	0.0000
78	Co	8.6896	12.1654	0.0000	0.6250	0.8750	0.0000
79	Co	12.1654	8.6896	0.0000	0.8750	0.6250	0.0000
80	Co	12.1654	12.1654	0.0000	0.8750	0.8750	0.0000
81	Co	1.7379	0.0000	1.7379	0.1250	0.0000	0.0792
82	Co	1.7379	3.4758	1.7379	0.1250	0.2500	0.0792
83	Co	5.2138	0.0000	1.7379	0.3750	0.0000	0.0792
84	Co	5.2138	3.4758	1.7379	0.3750	0.2500	0.0792
85	Co	1.7379	6.9517	1.7379	0.1250	0.5000	0.0792
86	Co	1.7379	10.4275	1.7379	0.1250	0.7500	0.0792
87	Co	5.2138	6.9517	1.7379	0.3750	0.5000	0.0792
88	Co	5.2138	10.4275	1.7379	0.3750	0.7500	0.0792

89	Co	8.6896	0.0000	1.7379	0.6250	0.0000	0.0792
90	Co	8.6896	3.4758	1.7379	0.6250	0.2500	0.0792
91	Co	12.1654	0.0000	1.7379	0.8750	0.0000	0.0792
92	Co	12.1654	3.4758	1.7379	0.8750	0.2500	0.0792
93	Co	8.6896	6.9517	1.7379	0.6250	0.5000	0.0792
94	Co	8.6896	10.4275	1.7379	0.6250	0.7500	0.0792
95	Co	12.1654	6.9517	1.7379	0.8750	0.5000	0.0792
96	Co	12.1654	10.4275	1.7379	0.8750	0.7500	0.0792
97	Co	1.7314	1.7405	3.5576	0.1245	0.1252	0.1621
98	Co	1.7448	5.2077	3.5753	0.1255	0.3746	0.1629
99	Co	5.2155	1.7038	3.5106	0.3751	0.1225	0.1599
100	Co	5.1921	5.2100	3.5128	0.3734	0.3747	0.1600
101	Co	1.7318	8.6954	3.5420	0.1246	0.6254	0.1614
102	Co	1.7358	12.1751	3.5538	0.1249	0.8757	0.1619
103	Co	5.2290	8.6720	3.5779	0.3761	0.6237	0.1630
104	Co	5.2194	12.1650	3.5553	0.3754	0.8750	0.1620
105	Co	8.6952	1.7353	3.5471	0.6254	0.1248	0.1616
106	Co	8.6931	5.2200	3.5074	0.6253	0.3754	0.1598
107	Co	12.1557	1.7643	3.5754	0.8743	0.1269	0.1629
108	Co	12.1702	5.2061	3.5523	0.8753	0.3744	0.1618
109	Co	8.6834	8.7036	3.5070	0.6246	0.6260	0.1598
110	Co	8.6835	12.1761	3.5338	0.6246	0.8758	0.1610
111	Co	12.1840	8.6954	3.5251	0.8763	0.6254	0.1606
112	Co	12.1691	12.1710	3.5543	0.8753	0.8754	0.1619
113	Co	1.7334	0.0192	5.2316	0.1247	0.0014	0.2383
114	Co	1.7201	3.4588	5.2274	0.1237	0.2488	0.2381
115	Co	5.2017	0.0039	5.2292	0.3741	0.0003	0.2382
116	Co	5.2420	3.4273	5.1044	0.3770	0.2465	0.2325
117	Co	1.6914	6.9703	5.2144	0.1217	0.5013	0.2375
118	Co	1.7389	10.4335	5.2221	0.1251	0.7504	0.2379
119	Co	5.1557	6.9178	5.2396	0.3708	0.4976	0.2387
120	Co	5.2017	10.4119	5.2314	0.3741	0.7489	0.2383
121	Co	8.6873	13.8678	5.2282	0.6248	0.9974	0.2382
122	Co	8.7189	3.4641	5.2574	0.6271	0.2492	0.2395
123	Co	12.1757	13.8978	5.2304	0.8757	0.9996	0.2383
124	Co	12.0749	3.4969	5.3342	0.8685	0.2515	0.2430
125	Co	8.7251	6.9745	5.1797	0.6276	0.5016	0.2360
126	Co	8.7225	10.4519	5.1785	0.6274	0.7518	0.2359
127	Co	12.1647	6.9797	5.2204	0.8749	0.5020	0.2378
128	Co	12.1572	10.4259	5.2262	0.8744	0.7499	0.2381
129	O	6.9844	3.6487	6.4722	0.5024	0.2624	0.2948
130	O	4.3546	6.2066	6.8674	0.3132	0.4464	0.3128
131	O	6.5755	5.6682	8.7288	0.4729	0.4077	0.3976
132	O	10.3491	3.7749	6.4374	0.7444	0.2715	0.2933
133	O	7.8390	7.5272	6.7967	0.5638	0.5414	0.3096
134	O	9.8800	7.0191	8.7076	0.7106	0.5048	0.3967
135	O	7.3761	9.2064	8.9510	0.5305	0.6622	0.4078
136	Ce	5.9241	7.8564	8.1318	0.4261	0.5651	0.3704
137	Ce	5.1438	4.2846	7.7375	0.3700	0.3082	0.3525

138 Ce	8.8180	5.3846	7.8335	0.6342	0.3873	0.3569
139 Sm	9.2414	8.9060	7.9455	0.6647	0.6406	0.3620

/db/jmorales/CoNi-alloy/metal-SDC-interface/Ni-Ce_3SmO_7/Ni-Ce_3SmO_7-conf4b

a =			13.9019837372				
b =			13.9019837372				
c =			21.9509918687				
alpha =			90.0				
beta =			90.0				
gamma =			90.0				
	Atom	X	Y	Z	X	Y	Z
1	Ni	0.0000	0.0000	0.0000	0.0000	0.0000	0.0000
2	Ni	1.7377	1.7377	0.0000	0.1250	0.1250	0.0000
3	Ni	0.0000	3.4755	0.0000	0.0000	0.2500	0.0000
4	Ni	1.7377	5.2132	0.0000	0.1250	0.3750	0.0000
5	Ni	3.4755	0.0000	0.0000	0.2500	0.0000	0.0000
6	Ni	5.2132	1.7377	0.0000	0.3750	0.1250	0.0000
7	Ni	3.4755	3.4755	0.0000	0.2500	0.2500	0.0000
8	Ni	5.2132	5.2132	0.0000	0.3750	0.3750	0.0000
9	Ni	0.0000	6.9510	0.0000	0.0000	0.5000	0.0000
10	Ni	1.7377	8.6887	0.0000	0.1250	0.6250	0.0000
11	Ni	0.0000	10.4265	0.0000	0.0000	0.7500	0.0000
12	Ni	1.7377	12.1642	0.0000	0.1250	0.8750	0.0000
13	Ni	3.4755	6.9510	0.0000	0.2500	0.5000	0.0000
14	Ni	5.2132	8.6887	0.0000	0.3750	0.6250	0.0000
15	Ni	3.4755	10.4265	0.0000	0.2500	0.7500	0.0000
16	Ni	5.2132	12.1642	0.0000	0.3750	0.8750	0.0000
17	Ni	6.9510	0.0000	0.0000	0.5000	0.0000	0.0000
18	Ni	8.6887	1.7377	0.0000	0.6250	0.1250	0.0000
19	Ni	6.9510	3.4755	0.0000	0.5000	0.2500	0.0000
20	Ni	8.6887	5.2132	0.0000	0.6250	0.3750	0.0000
21	Ni	10.4265	0.0000	0.0000	0.7500	0.0000	0.0000
22	Ni	12.1642	1.7377	0.0000	0.8750	0.1250	0.0000
23	Ni	10.4265	3.4755	0.0000	0.7500	0.2500	0.0000
24	Ni	12.1642	5.2132	0.0000	0.8750	0.3750	0.0000
25	Ni	6.9510	6.9510	0.0000	0.5000	0.5000	0.0000
26	Ni	8.6887	8.6887	0.0000	0.6250	0.6250	0.0000
27	Ni	6.9510	10.4265	0.0000	0.5000	0.7500	0.0000
28	Ni	8.6887	12.1642	0.0000	0.6250	0.8750	0.0000
29	Ni	10.4265	6.9510	0.0000	0.7500	0.5000	0.0000
30	Ni	12.1642	8.6887	0.0000	0.8750	0.6250	0.0000
31	Ni	10.4265	10.4265	0.0000	0.7500	0.7500	0.0000
32	Ni	12.1642	12.1642	0.0000	0.8750	0.8750	0.0000
33	Ni	0.0000	1.7377	1.7377	0.0000	0.1250	0.0792
34	Ni	1.7377	0.0000	1.7377	0.1250	0.0000	0.0792
35	Ni	0.0000	5.2132	1.7377	0.0000	0.3750	0.0792
36	Ni	1.7377	3.4755	1.7377	0.1250	0.2500	0.0792
37	Ni	3.4755	1.7377	1.7377	0.2500	0.1250	0.0792
38	Ni	5.2132	0.0000	1.7377	0.3750	0.0000	0.0792
39	Ni	3.4755	5.2132	1.7377	0.2500	0.3750	0.0792
40	Ni	5.2132	3.4755	1.7377	0.3750	0.2500	0.0792

41	Ni	0.0000	8.6887	1.7377	0.0000	0.6250	0.0792
42	Ni	1.7377	6.9510	1.7377	0.1250	0.5000	0.0792
43	Ni	0.0000	12.1642	1.7377	0.0000	0.8750	0.0792
44	Ni	1.7377	10.4265	1.7377	0.1250	0.7500	0.0792
45	Ni	3.4755	8.6887	1.7377	0.2500	0.6250	0.0792
46	Ni	5.2132	6.9510	1.7377	0.3750	0.5000	0.0792
47	Ni	3.4755	12.1642	1.7377	0.2500	0.8750	0.0792
48	Ni	5.2132	10.4265	1.7377	0.3750	0.7500	0.0792
49	Ni	6.9510	1.7377	1.7377	0.5000	0.1250	0.0792
50	Ni	8.6887	0.0000	1.7377	0.6250	0.0000	0.0792
51	Ni	6.9510	5.2132	1.7377	0.5000	0.3750	0.0792
52	Ni	8.6887	3.4755	1.7377	0.6250	0.2500	0.0792
53	Ni	10.4265	1.7377	1.7377	0.7500	0.1250	0.0792
54	Ni	12.1642	0.0000	1.7377	0.8750	0.0000	0.0792
55	Ni	10.4265	5.2132	1.7377	0.7500	0.3750	0.0792
56	Ni	12.1642	3.4755	1.7377	0.8750	0.2500	0.0792
57	Ni	6.9510	8.6887	1.7377	0.5000	0.6250	0.0792
58	Ni	8.6887	6.9510	1.7377	0.6250	0.5000	0.0792
59	Ni	6.9510	12.1642	1.7377	0.5000	0.8750	0.0792
60	Ni	8.6887	10.4265	1.7377	0.6250	0.7500	0.0792
61	Ni	10.4265	8.6887	1.7377	0.7500	0.6250	0.0792
62	Ni	12.1642	6.9510	1.7377	0.8750	0.5000	0.0792
63	Ni	10.4265	12.1642	1.7377	0.7500	0.8750	0.0792
64	Ni	12.1642	10.4265	1.7377	0.8750	0.7500	0.0792
65	Ni	13.9001	13.8985	3.5118	0.9999	0.9997	0.1600
66	Ni	1.7387	1.7285	3.5045	0.1251	0.1243	0.1597
67	Ni	13.8927	3.4659	3.5050	0.9993	0.2493	0.1597
68	Ni	1.7214	5.2185	3.5144	0.1238	0.3754	0.1601
69	Ni	3.4831	0.0070	3.5174	0.2506	0.0005	0.1602
70	Ni	5.2084	1.7674	3.5379	0.3747	0.1271	0.1612
71	Ni	3.4651	3.4964	3.5132	0.2492	0.2515	0.1600
72	Ni	5.2036	5.2234	3.4623	0.3743	0.3757	0.1577
73	Ni	0.0139	6.9480	3.5438	0.0010	0.4998	0.1614
74	Ni	1.7063	8.6742	3.5005	0.1227	0.6240	0.1595
75	Ni	13.8999	10.4325	3.5092	0.9998	0.7504	0.1599
76	Ni	1.7354	12.1749	3.5107	0.1248	0.8758	0.1599
77	Ni	3.4591	6.9407	3.4893	0.2488	0.4993	0.1590
78	Ni	5.2299	8.6682	3.4934	0.3762	0.6235	0.1591
79	Ni	3.4704	10.4570	3.4751	0.2496	0.7522	0.1583
80	Ni	5.2217	12.1790	3.5052	0.3756	0.8761	0.1597
81	Ni	6.9462	0.0060	3.5160	0.4997	0.0004	0.1602
82	Ni	8.6934	1.7259	3.5081	0.6253	0.1242	0.1598
83	Ni	6.9615	3.4650	3.4918	0.5008	0.2492	0.1591
84	Ni	8.7008	5.2050	3.5057	0.6259	0.3744	0.1597
85	Ni	10.4227	13.8926	3.5138	0.7497	0.9993	0.1601
86	Ni	12.1620	1.7255	3.5106	0.8748	0.1241	0.1599
87	Ni	10.4360	3.4674	3.5044	0.7507	0.2494	0.1596
88	Ni	12.1639	5.2146	3.5089	0.8750	0.3751	0.1598
89	Ni	6.9750	6.9381	3.4792	0.5017	0.4991	0.1585

90	Ni	8.6860	8.6916	3.5099	0.6248	0.6252	0.1599
91	Ni	6.9506	10.4206	3.5064	0.5000	0.7496	0.1597
92	Ni	8.6836	12.1665	3.5123	0.6246	0.8752	0.1600
93	Ni	10.4398	6.9570	3.5052	0.7510	0.5004	0.1597
94	Ni	12.1657	8.6921	3.5159	0.8751	0.6252	0.1602
95	Ni	10.4253	10.4278	3.5103	0.7499	0.7501	0.1599
96	Ni	12.1598	12.1633	3.5131	0.8747	0.8749	0.1600
97	Ni	13.8918	1.7254	5.2287	0.9993	0.1241	0.2382
98	Ni	1.7445	0.0087	5.2308	0.1255	0.0006	0.2383
99	Ni	13.8778	5.1879	5.2228	0.9983	0.3732	0.2379
100	Ni	1.7146	3.4318	5.2119	0.1233	0.2469	0.2374
101	Ni	3.4809	1.7579	5.2406	0.2504	0.1264	0.2387
102	Ni	5.2166	0.0030	5.2372	0.3752	0.0002	0.2386
103	Ni	3.3700	5.2062	5.2787	0.2424	0.3745	0.2405
104	Ni	5.2016	3.5046	5.2664	0.3742	0.2521	0.2399
105	Ni	13.8954	8.7056	5.2353	0.9995	0.6262	0.2385
106	Ni	1.7015	6.9857	5.4103	0.1224	0.5025	0.2465
107	Ni	0.0058	12.1636	5.2292	0.0004	0.8750	0.2382
108	Ni	1.7429	10.4215	5.2155	0.1254	0.7496	0.2376
109	Ni	3.4638	8.7276	5.0981	0.2492	0.6278	0.2322
110	Ni	5.2176	6.9018	5.2351	0.3753	0.4965	0.2385
111	Ni	3.4735	12.1640	5.2249	0.2499	0.8750	0.2380
112	Ni	5.2001	10.4480	5.1838	0.3741	0.7515	0.2362
113	Ni	6.9524	1.7455	5.2261	0.5001	0.1256	0.2381
114	Ni	8.6790	13.8992	5.2357	0.6243	0.9998	0.2385
115	Ni	7.0804	5.1860	5.2333	0.5093	0.3730	0.2384
116	Ni	8.7263	3.4454	5.2176	0.6277	0.2478	0.2377
117	Ni	10.4345	1.7202	5.2283	0.7506	0.1237	0.2382
118	Ni	12.1618	13.8878	5.2322	0.8748	0.9990	0.2384
119	Ni	10.4311	5.2070	5.2314	0.7503	0.3746	0.2383
120	Ni	12.1587	3.4638	5.2267	0.8746	0.2492	0.2381
121	Ni	6.9911	8.6894	5.2453	0.5029	0.6250	0.2390
122	Ni	8.7383	6.9430	5.2083	0.6286	0.4994	0.2373
123	Ni	6.9513	12.1451	5.2440	0.5000	0.8736	0.2389
124	Ni	8.6776	10.4306	5.2325	0.6242	0.7503	0.2384
125	Ni	10.4195	8.7066	5.2380	0.7495	0.6263	0.2386
126	Ni	12.1613	6.9548	5.2338	0.8748	0.5003	0.2384
127	Ni	10.4103	12.1574	5.2386	0.7488	0.8745	0.2386
128	Ni	12.1613	10.4362	5.2300	0.8748	0.7507	0.2383
129	O	5.5475	5.1292	6.3619	0.3990	0.3690	0.2898
130	O	6.1630	4.3493	8.9122	0.4433	0.3129	0.4060
131	O	2.7387	6.2123	6.8670	0.1970	0.4469	0.3128
132	O	4.7094	6.5810	8.8071	0.3388	0.4734	0.4012
133	O	6.0556	7.9171	6.8003	0.4356	0.5695	0.3098
134	O	7.7434	7.9375	8.9778	0.5570	0.5710	0.4090
135	O	4.9116	9.7674	8.5198	0.3533	0.7026	0.3881
136	Ce	3.8439	8.1939	7.6315	0.2765	0.5894	0.3477
137	Ce	4.0814	4.4970	8.0977	0.2936	0.3235	0.3689
138	Ce	6.9549	6.1583	8.2052	0.5003	0.4430	0.3738

139 Sm	6.9767	9.6814	7.9279	0.5018	0.6964	0.3612

/db/jmorales/CoNi-alloy/metal-SDC-interface/CoNi-Ce_3SmO_7/CoNi-Ce_3SmO_7-conf2c

a = 13.9033384264
b = 13.9033384264
c = 21.9516692133
alpha = 90.0
beta = 90.0
gamma = 90.0

	Atom	X	Y	Z	X	Y	Z
1	Ni	0.0000	0.0000	0.0000	0.0000	0.0000	0.0000
2	Ni	0.0000	3.4758	0.0000	0.0000	0.2500	0.0000
3	Ni	3.4758	0.0000	0.0000	0.2500	0.0000	0.0000
4	Ni	3.4758	3.4758	0.0000	0.2500	0.2500	0.0000
5	Ni	0.0000	6.9517	0.0000	0.0000	0.5000	0.0000
6	Ni	0.0000	10.4275	0.0000	0.0000	0.7500	0.0000
7	Ni	3.4758	6.9517	0.0000	0.2500	0.5000	0.0000
8	Ni	3.4758	10.4275	0.0000	0.2500	0.7500	0.0000
9	Ni	6.9517	0.0000	0.0000	0.5000	0.0000	0.0000
10	Ni	6.9517	3.4758	0.0000	0.5000	0.2500	0.0000
11	Ni	10.4275	0.0000	0.0000	0.7500	0.0000	0.0000
12	Ni	10.4275	3.4758	0.0000	0.7500	0.2500	0.0000
13	Ni	6.9517	6.9517	0.0000	0.5000	0.5000	0.0000
14	Ni	6.9517	10.4275	0.0000	0.5000	0.7500	0.0000
15	Ni	10.4275	6.9517	0.0000	0.7500	0.5000	0.0000
16	Ni	10.4275	10.4275	0.0000	0.7500	0.7500	0.0000
17	Ni	0.0000	1.7379	1.7379	0.0000	0.1250	0.0792
18	Ni	0.0000	5.2138	1.7379	0.0000	0.3750	0.0792
19	Ni	3.4758	1.7379	1.7379	0.2500	0.1250	0.0792
20	Ni	3.4758	5.2138	1.7379	0.2500	0.3750	0.0792
21	Ni	0.0000	8.6896	1.7379	0.0000	0.6250	0.0792
22	Ni	0.0000	12.1654	1.7379	0.0000	0.8750	0.0792
23	Ni	3.4758	8.6896	1.7379	0.2500	0.6250	0.0792
24	Ni	3.4758	12.1654	1.7379	0.2500	0.8750	0.0792
25	Ni	6.9517	1.7379	1.7379	0.5000	0.1250	0.0792
26	Ni	6.9517	5.2138	1.7379	0.5000	0.3750	0.0792
27	Ni	10.4275	1.7379	1.7379	0.7500	0.1250	0.0792
28	Ni	10.4275	5.2138	1.7379	0.7500	0.3750	0.0792
29	Ni	6.9517	8.6896	1.7379	0.5000	0.6250	0.0792
30	Ni	6.9517	12.1654	1.7379	0.5000	0.8750	0.0792
31	Ni	10.4275	8.6896	1.7379	0.7500	0.6250	0.0792
32	Ni	10.4275	12.1654	1.7379	0.7500	0.8750	0.0792
33	Ni	0.0000	13.9027	3.5011	0.0000	1.0000	0.1595
34	Ni	13.8867	3.4724	3.4863	0.9988	0.2498	0.1588
35	Ni	3.4786	0.0007	3.5023	0.2502	0.0001	0.1595
36	Ni	3.5002	3.4939	3.5324	0.2517	0.2513	0.1609
37	Ni	0.0031	6.9560	3.5144	0.0002	0.5003	0.1601
38	Ni	0.0002	10.4270	3.5047	0.0000	0.7500	0.1597
39	Ni	3.4784	6.9578	3.4935	0.2502	0.5004	0.1591

40	Ni	3.4720	10.4492	3.4768	0.2497	0.7516	0.1584
41	Ni	6.9478	0.0051	3.5042	0.4997	0.0004	0.1596
42	Ni	6.9316	3.4378	3.4891	0.4986	0.2473	0.1589
43	Ni	10.4285	13.9027	3.4996	0.7501	1.0000	0.1594
44	Ni	10.4436	3.4657	3.4954	0.7512	0.2493	0.1592
45	Ni	6.9618	6.9543	3.4559	0.5007	0.5002	0.1574
46	Ni	6.9625	10.4142	3.5228	0.5008	0.7490	0.1605
47	Ni	10.4392	6.9817	3.5124	0.7508	0.5022	0.1600
48	Ni	10.4148	10.4211	3.5172	0.7491	0.7495	0.1602
49	Ni	0.0043	1.7360	5.2121	0.0003	0.1249	0.2374
50	Ni	13.8811	5.1910	5.1972	0.9984	0.3734	0.2368
51	Ni	3.4403	1.7314	5.2169	0.2474	0.1245	0.2377
52	Ni	3.4385	5.2413	5.2130	0.2473	0.3770	0.2375
53	Ni	0.0021	8.6961	5.2139	0.0001	0.6255	0.2375
54	Ni	0.0071	12.1668	5.2046	0.0005	0.8751	0.2371
55	Ni	3.4881	8.7317	5.1290	0.2509	0.6280	0.2337
56	Ni	3.4669	12.1731	5.2055	0.2494	0.8756	0.2371
57	Ni	6.9882	1.7177	5.2129	0.5026	0.1235	0.2375
58	Ni	7.0053	5.1810	5.0646	0.5039	0.3726	0.2307
59	Ni	10.4254	1.7326	5.2079	0.7499	0.1246	0.2372
60	Ni	10.4384	5.2048	5.2052	0.7508	0.3744	0.2371
61	Ni	6.9076	8.6922	5.2274	0.4968	0.6252	0.2381
62	Ni	6.9580	12.1659	5.2049	0.5005	0.8750	0.2371
63	Ni	10.4035	8.6569	5.3875	0.7483	0.6226	0.2454
64	Ni	10.4390	12.1970	5.1915	0.7508	0.8773	0.2365
65	Co	1.7379	1.7379	0.0000	0.1250	0.1250	0.0000
66	Co	1.7379	5.2138	0.0000	0.1250	0.3750	0.0000
67	Co	5.2138	1.7379	0.0000	0.3750	0.1250	0.0000
68	Co	5.2138	5.2138	0.0000	0.3750	0.3750	0.0000
69	Co	1.7379	8.6896	0.0000	0.1250	0.6250	0.0000
70	Co	1.7379	12.1654	0.0000	0.1250	0.8750	0.0000
71	Co	5.2138	8.6896	0.0000	0.3750	0.6250	0.0000
72	Co	5.2138	12.1654	0.0000	0.3750	0.8750	0.0000
73	Co	8.6896	1.7379	0.0000	0.6250	0.1250	0.0000
74	Co	8.6896	5.2138	0.0000	0.6250	0.3750	0.0000
75	Co	12.1654	1.7379	0.0000	0.8750	0.1250	0.0000
76	Co	12.1654	5.2138	0.0000	0.8750	0.3750	0.0000
77	Co	8.6896	8.6896	0.0000	0.6250	0.6250	0.0000
78	Co	8.6896	12.1654	0.0000	0.6250	0.8750	0.0000
79	Co	12.1654	8.6896	0.0000	0.8750	0.6250	0.0000
80	Co	12.1654	12.1654	0.0000	0.8750	0.8750	0.0000
81	Co	1.7379	0.0000	1.7379	0.1250	0.0000	0.0792
82	Co	1.7379	3.4758	1.7379	0.1250	0.2500	0.0792
83	Co	5.2138	0.0000	1.7379	0.3750	0.0000	0.0792
84	Co	5.2138	3.4758	1.7379	0.3750	0.2500	0.0792
85	Co	1.7379	6.9517	1.7379	0.1250	0.5000	0.0792
86	Co	1.7379	10.4275	1.7379	0.1250	0.7500	0.0792
87	Co	5.2138	6.9517	1.7379	0.3750	0.5000	0.0792
88	Co	5.2138	10.4275	1.7379	0.3750	0.7500	0.0792

89	Co	8.6896	0.0000	1.7379	0.6250	0.0000	0.0792
90	Co	8.6896	3.4758	1.7379	0.6250	0.2500	0.0792
91	Co	12.1654	0.0000	1.7379	0.8750	0.0000	0.0792
92	Co	12.1654	3.4758	1.7379	0.8750	0.2500	0.0792
93	Co	8.6896	6.9517	1.7379	0.6250	0.5000	0.0792
94	Co	8.6896	10.4275	1.7379	0.6250	0.7500	0.0792
95	Co	12.1654	6.9517	1.7379	0.8750	0.5000	0.0792
96	Co	12.1654	10.4275	1.7379	0.8750	0.7500	0.0792
97	Co	1.7404	1.7309	3.5309	0.1252	0.1245	0.1608
98	Co	1.7230	5.2252	3.5302	0.1239	0.3758	0.1608
99	Co	5.2058	1.7583	3.5904	0.3744	0.1265	0.1636
100	Co	5.1902	5.1952	3.5546	0.3733	0.3737	0.1619
101	Co	1.7190	8.6753	3.5395	0.1236	0.6240	0.1612
102	Co	1.7353	12.1659	3.5452	0.1248	0.8750	0.1615
103	Co	5.2206	8.6972	3.5094	0.3755	0.6255	0.1599
104	Co	5.2206	12.1754	3.5463	0.3755	0.8757	0.1615
105	Co	8.6945	1.7392	3.5392	0.6254	0.1251	0.1612
106	Co	8.7286	5.1977	3.4932	0.6278	0.3738	0.1591
107	Co	12.1689	1.7312	3.5538	0.8753	0.1245	0.1619
108	Co	12.1632	5.2154	3.5467	0.8748	0.3751	0.1616
109	Co	8.6886	8.6928	3.5698	0.6249	0.6252	0.1626
110	Co	8.6817	12.1523	3.5430	0.6244	0.8741	0.1614
111	Co	12.1388	8.6970	3.5908	0.8731	0.6255	0.1636
112	Co	12.1659	12.1638	3.5456	0.8750	0.8749	0.1615
113	Co	1.7381	0.0017	5.2239	0.1250	0.0001	0.2380
114	Co	1.7154	3.4470	5.1655	0.1234	0.2479	0.2353
115	Co	5.2104	13.8997	5.2202	0.3748	0.9997	0.2378
116	Co	5.2437	3.4934	5.3990	0.3772	0.2513	0.2459
117	Co	1.7146	6.9778	5.3210	0.1233	0.5019	0.2424
118	Co	1.7534	10.4143	5.2201	0.1261	0.7491	0.2378
119	Co	5.2629	6.9359	5.1808	0.3785	0.4989	0.2360
120	Co	5.1834	10.4567	5.2044	0.3728	0.7521	0.2371
121	Co	8.6909	13.8996	5.2119	0.6251	0.9997	0.2374
122	Co	8.7018	3.4816	5.1979	0.6259	0.2504	0.2368
123	Co	12.1702	13.9017	5.2213	0.8753	0.9999	0.2379
124	Co	12.1663	3.4680	5.2235	0.8751	0.2494	0.2380
125	Co	8.6985	6.8718	5.1620	0.6256	0.4943	0.2352
126	Co	8.6714	10.4414	5.3340	0.6237	0.7510	0.2430
127	Co	12.1710	6.9524	5.2197	0.8754	0.5000	0.2378
128	Co	12.1948	10.4431	5.2171	0.8771	0.7511	0.2377
129	O	5.2544	3.5682	7.2727	0.3779	0.2566	0.3313
130	O	2.6369	6.2196	6.7704	0.1897	0.4473	0.3084
131	O	4.6757	5.9722	8.7655	0.3363	0.4295	0.3993
132	O	6.2467	7.5432	6.7798	0.4493	0.5425	0.3089
133	O	9.4070	9.5697	6.7410	0.6766	0.6883	0.3071
134	O	8.0612	6.8750	8.9190	0.5798	0.4945	0.4063
135	O	5.6777	9.3364	8.8785	0.4084	0.6715	0.4045
136	Ce	4.2196	8.1169	7.9381	0.3035	0.5838	0.3616
137	Ce	3.3235	4.3756	7.9363	0.2390	0.3147	0.3615

138 Ce	6.8167	5.4148	7.9140	0.4903	0.3895	0.3605
139 Sm	7.6810	8.8200	8.1729	0.5525	0.6344	0.3723

/db/jmorales/CoNi-alloy/metal-SDC-interface/CoNi-Ce_3SmO_7/CoNi-Ce_3SmO_7-conf3c

a = 13.9033384264
b = 13.9033384264
c = 21.9516692133
alpha = 90.0
beta = 90.0
gamma = 90.0

	Atom	X	Y	Z	X	Y	Z
1	Ni	0.0000	0.0000	0.0000	0.0000	0.0000	0.0000
2	Ni	0.0000	3.4758	0.0000	0.0000	0.2500	0.0000
3	Ni	3.4758	0.0000	0.0000	0.2500	0.0000	0.0000
4	Ni	3.4758	3.4758	0.0000	0.2500	0.2500	0.0000
5	Ni	0.0000	6.9517	0.0000	0.0000	0.5000	0.0000
6	Ni	0.0000	10.4275	0.0000	0.0000	0.7500	0.0000
7	Ni	3.4758	6.9517	0.0000	0.2500	0.5000	0.0000
8	Ni	3.4758	10.4275	0.0000	0.2500	0.7500	0.0000
9	Ni	6.9517	0.0000	0.0000	0.5000	0.0000	0.0000
10	Ni	6.9517	3.4758	0.0000	0.5000	0.2500	0.0000
11	Ni	10.4275	0.0000	0.0000	0.7500	0.0000	0.0000
12	Ni	10.4275	3.4758	0.0000	0.7500	0.2500	0.0000
13	Ni	6.9517	6.9517	0.0000	0.5000	0.5000	0.0000
14	Ni	6.9517	10.4275	0.0000	0.5000	0.7500	0.0000
15	Ni	10.4275	6.9517	0.0000	0.7500	0.5000	0.0000
16	Ni	10.4275	10.4275	0.0000	0.7500	0.7500	0.0000
17	Ni	0.0000	1.7379	1.7379	0.0000	0.1250	0.0792
18	Ni	0.0000	5.2138	1.7379	0.0000	0.3750	0.0792
19	Ni	3.4758	1.7379	1.7379	0.2500	0.1250	0.0792
20	Ni	3.4758	5.2138	1.7379	0.2500	0.3750	0.0792
21	Ni	0.0000	8.6896	1.7379	0.0000	0.6250	0.0792
22	Ni	0.0000	12.1654	1.7379	0.0000	0.8750	0.0792
23	Ni	3.4758	8.6896	1.7379	0.2500	0.6250	0.0792
24	Ni	3.4758	12.1654	1.7379	0.2500	0.8750	0.0792
25	Ni	6.9517	1.7379	1.7379	0.5000	0.1250	0.0792
26	Ni	6.9517	5.2138	1.7379	0.5000	0.3750	0.0792
27	Ni	10.4275	1.7379	1.7379	0.7500	0.1250	0.0792
28	Ni	10.4275	5.2138	1.7379	0.7500	0.3750	0.0792
29	Ni	6.9517	8.6896	1.7379	0.5000	0.6250	0.0792
30	Ni	6.9517	12.1654	1.7379	0.5000	0.8750	0.0792
31	Ni	10.4275	8.6896	1.7379	0.7500	0.6250	0.0792
32	Ni	10.4275	12.1654	1.7379	0.7500	0.8750	0.0792
33	Ni	13.9014	0.0008	3.5040	0.9999	0.0001	0.1596
34	Ni	13.8936	3.4726	3.4917	0.9993	0.2498	0.1591
35	Ni	3.4827	0.0039	3.5058	0.2505	0.0003	0.1597
36	Ni	3.4941	3.4906	3.5105	0.2513	0.2511	0.1599
37	Ni	0.0122	6.9495	3.5211	0.0009	0.4998	0.1604
38	Ni	-0.0018	10.4295	3.5031	-0.0001	0.7501	0.1596
39	Ni	3.4744	6.9635	3.4883	0.2499	0.5008	0.1589

40	Ni	3.4660	10.4559	3.4673	0.2493	0.7520	0.1580
41	Ni	6.9527	13.9000	3.5010	0.5001	0.9998	0.1595
42	Ni	6.9560	3.4696	3.4990	0.5003	0.2496	0.1594
43	Ni	10.4286	13.9017	3.5019	0.7501	0.9999	0.1595
44	Ni	10.4254	3.4825	3.5070	0.7498	0.2505	0.1598
45	Ni	6.9691	6.9299	3.4998	0.5013	0.4984	0.1594
46	Ni	6.9537	10.4200	3.5069	0.5001	0.7495	0.1598
47	Ni	10.4291	6.9473	3.5003	0.7501	0.4997	0.1595
48	Ni	10.4363	10.4249	3.4959	0.7506	0.7498	0.1593
49	Ni	0.0010	1.7348	5.2106	0.0001	0.1248	0.2374
50	Ni	13.8964	5.1902	5.1989	0.9995	0.3733	0.2368
51	Ni	3.4773	1.7425	5.2194	0.2501	0.1253	0.2378
52	Ni	3.4357	5.2351	5.1872	0.2471	0.3765	0.2363
53	Ni	0.0080	8.6970	5.2176	0.0006	0.6255	0.2377
54	Ni	0.0020	12.1667	5.2103	0.0001	0.8751	0.2374
55	Ni	3.4638	8.7477	5.1077	0.2491	0.6292	0.2327
56	Ni	3.4666	12.1730	5.2052	0.2493	0.8755	0.2371
57	Ni	6.9882	1.7178	5.1931	0.5026	0.1235	0.2366
58	Ni	6.9317	5.1831	5.2603	0.4986	0.3728	0.2396
59	Ni	10.4251	1.7346	5.2023	0.7498	0.1248	0.2370
60	Ni	10.4636	5.2082	5.2659	0.7526	0.3746	0.2399
61	Ni	6.9840	8.7120	5.2241	0.5023	0.6266	0.2380
62	Ni	6.9668	12.1518	5.2209	0.5011	0.8740	0.2378
63	Ni	10.4180	8.6931	5.2070	0.7493	0.6253	0.2372
64	Ni	10.4247	12.1642	5.2116	0.7498	0.8749	0.2374
65	Co	1.7379	1.7379	0.0000	0.1250	0.1250	0.0000
66	Co	1.7379	5.2138	0.0000	0.1250	0.3750	0.0000
67	Co	5.2138	1.7379	0.0000	0.3750	0.1250	0.0000
68	Co	5.2138	5.2138	0.0000	0.3750	0.3750	0.0000
69	Co	1.7379	8.6896	0.0000	0.1250	0.6250	0.0000
70	Co	1.7379	12.1654	0.0000	0.1250	0.8750	0.0000
71	Co	5.2138	8.6896	0.0000	0.3750	0.6250	0.0000
72	Co	5.2138	12.1654	0.0000	0.3750	0.8750	0.0000
73	Co	8.6896	1.7379	0.0000	0.6250	0.1250	0.0000
74	Co	8.6896	5.2138	0.0000	0.6250	0.3750	0.0000
75	Co	12.1654	1.7379	0.0000	0.8750	0.1250	0.0000
76	Co	12.1654	5.2138	0.0000	0.8750	0.3750	0.0000
77	Co	8.6896	8.6896	0.0000	0.6250	0.6250	0.0000
78	Co	8.6896	12.1654	0.0000	0.6250	0.8750	0.0000
79	Co	12.1654	8.6896	0.0000	0.8750	0.6250	0.0000
80	Co	12.1654	12.1654	0.0000	0.8750	0.8750	0.0000
81	Co	1.7379	0.0000	1.7379	0.1250	0.0000	0.0792
82	Co	1.7379	3.4758	1.7379	0.1250	0.2500	0.0792
83	Co	5.2138	0.0000	1.7379	0.3750	0.0000	0.0792
84	Co	5.2138	3.4758	1.7379	0.3750	0.2500	0.0792
85	Co	1.7379	6.9517	1.7379	0.1250	0.5000	0.0792
86	Co	1.7379	10.4275	1.7379	0.1250	0.7500	0.0792
87	Co	5.2138	6.9517	1.7379	0.3750	0.5000	0.0792
88	Co	5.2138	10.4275	1.7379	0.3750	0.7500	0.0792

89	Co	8.6896	0.0000	1.7379	0.6250	0.0000	0.0792
90	Co	8.6896	3.4758	1.7379	0.6250	0.2500	0.0792
91	Co	12.1654	0.0000	1.7379	0.8750	0.0000	0.0792
92	Co	12.1654	3.4758	1.7379	0.8750	0.2500	0.0792
93	Co	8.6896	6.9517	1.7379	0.6250	0.5000	0.0792
94	Co	8.6896	10.4275	1.7379	0.6250	0.7500	0.0792
95	Co	12.1654	6.9517	1.7379	0.8750	0.5000	0.0792
96	Co	12.1654	10.4275	1.7379	0.8750	0.7500	0.0792
97	Co	1.7439	1.7319	3.5401	0.1254	0.1246	0.1613
98	Co	1.7225	5.2214	3.5220	0.1239	0.3756	0.1604
99	Co	5.2157	1.7426	3.5645	0.3751	0.1253	0.1624
100	Co	5.2120	5.2219	3.5685	0.3749	0.3756	0.1626
101	Co	1.7124	8.6722	3.5346	0.1232	0.6237	0.1610
102	Co	1.7326	12.1741	3.5520	0.1246	0.8756	0.1618
103	Co	5.2276	8.7108	3.5080	0.3760	0.6265	0.1598
104	Co	5.2211	12.1767	3.5520	0.3755	0.8758	0.1618
105	Co	8.6952	1.7436	3.5483	0.6254	0.1254	0.1616
106	Co	8.6833	5.2010	3.4871	0.6246	0.3741	0.1589
107	Co	12.1630	1.7342	3.5513	0.8748	0.1247	0.1618
108	Co	12.1560	5.2150	3.5668	0.8743	0.3751	0.1625
109	Co	8.6867	8.6861	3.5447	0.6248	0.6247	0.1615
110	Co	8.6932	12.1669	3.5352	0.6253	0.8751	0.1610
111	Co	12.1664	8.6869	3.5617	0.8751	0.6248	0.1623
112	Co	12.1666	12.1661	3.5574	0.8751	0.8750	0.1621
113	Co	1.7410	0.0114	5.2293	0.1252	0.0008	0.2382
114	Co	1.7308	3.4374	5.1816	0.1245	0.2472	0.2360
115	Co	5.2272	0.0007	5.2304	0.3760	0.0000	0.2383
116	Co	5.2165	3.4575	5.2653	0.3752	0.2487	0.2399
117	Co	1.7392	6.9709	5.3483	0.1251	0.5014	0.2436
118	Co	1.7442	10.4237	5.2172	0.1254	0.7497	0.2377
119	Co	5.2237	7.0297	5.1754	0.3757	0.5056	0.2358
120	Co	5.1844	10.4631	5.1945	0.3729	0.7526	0.2366
121	Co	8.6922	13.8957	5.2225	0.6252	0.9995	0.2379
122	Co	8.7291	3.4902	5.2814	0.6278	0.2510	0.2406
123	Co	12.1610	0.0028	5.2258	0.8747	0.0002	0.2381
124	Co	12.1767	3.4609	5.2156	0.8758	0.2489	0.2376
125	Co	8.7394	6.9129	5.2280	0.6286	0.4972	0.2382
126	Co	8.7060	10.4399	5.2022	0.6262	0.7509	0.2370
127	Co	12.1787	6.9632	5.2160	0.8760	0.5008	0.2376
128	Co	12.1617	10.4220	5.2241	0.8747	0.7496	0.2380
129	O	5.8616	4.2716	6.8753	0.4216	0.3072	0.3132
130	O	2.7274	6.1444	6.8314	0.1962	0.4419	0.3112
131	O	4.8561	6.1468	8.8711	0.3493	0.4421	0.4041
132	O	8.7075	5.2601	6.3485	0.6263	0.3783	0.2892
133	O	6.0967	7.8051	6.8016	0.4385	0.5614	0.3098
134	O	7.9988	7.9688	8.9095	0.5753	0.5732	0.4059
135	O	5.1858	9.7541	8.7006	0.3730	0.7016	0.3964
136	Ce	3.9728	8.1347	7.9178	0.2857	0.5851	0.3607
137	Ce	3.8377	4.4486	7.8708	0.2760	0.3200	0.3586

138 Ce	7.1638	6.1289	8.1281	0.5153	0.4408	0.3703
139 Sm	7.1603	9.6587	7.9571	0.5150	0.6947	0.3625

/db/jmorales/CoNi-alloy/metal-SDC-interface/CoNi-Ce_3SmO_7/CoNi-Ce_3SmO_7-conf2b

a = 13.9033384264
b = 13.9033384264
c = 21.9516692133
alpha = 90.0
beta = 90.0
gamma = 90.0

	Atom	X	Y	Z	X	Y	Z
1	Ni	0.0000	0.0000	0.0000	0.0000	0.0000	0.0000
2	Ni	0.0000	3.4758	0.0000	0.0000	0.2500	0.0000
3	Ni	3.4758	0.0000	0.0000	0.2500	0.0000	0.0000
4	Ni	3.4758	3.4758	0.0000	0.2500	0.2500	0.0000
5	Ni	0.0000	6.9517	0.0000	0.0000	0.5000	0.0000
6	Ni	0.0000	10.4275	0.0000	0.0000	0.7500	0.0000
7	Ni	3.4758	6.9517	0.0000	0.2500	0.5000	0.0000
8	Ni	3.4758	10.4275	0.0000	0.2500	0.7500	0.0000
9	Ni	6.9517	0.0000	0.0000	0.5000	0.0000	0.0000
10	Ni	6.9517	3.4758	0.0000	0.5000	0.2500	0.0000
11	Ni	10.4275	0.0000	0.0000	0.7500	0.0000	0.0000
12	Ni	10.4275	3.4758	0.0000	0.7500	0.2500	0.0000
13	Ni	6.9517	6.9517	0.0000	0.5000	0.5000	0.0000
14	Ni	6.9517	10.4275	0.0000	0.5000	0.7500	0.0000
15	Ni	10.4275	6.9517	0.0000	0.7500	0.5000	0.0000
16	Ni	10.4275	10.4275	0.0000	0.7500	0.7500	0.0000
17	Ni	0.0000	1.7379	1.7379	0.0000	0.1250	0.0792
18	Ni	0.0000	5.2138	1.7379	0.0000	0.3750	0.0792
19	Ni	3.4758	1.7379	1.7379	0.2500	0.1250	0.0792
20	Ni	3.4758	5.2138	1.7379	0.2500	0.3750	0.0792
21	Ni	0.0000	8.6896	1.7379	0.0000	0.6250	0.0792
22	Ni	0.0000	12.1654	1.7379	0.0000	0.8750	0.0792
23	Ni	3.4758	8.6896	1.7379	0.2500	0.6250	0.0792
24	Ni	3.4758	12.1654	1.7379	0.2500	0.8750	0.0792
25	Ni	6.9517	1.7379	1.7379	0.5000	0.1250	0.0792
26	Ni	6.9517	5.2138	1.7379	0.5000	0.3750	0.0792
27	Ni	10.4275	1.7379	1.7379	0.7500	0.1250	0.0792
28	Ni	10.4275	5.2138	1.7379	0.7500	0.3750	0.0792
29	Ni	6.9517	8.6896	1.7379	0.5000	0.6250	0.0792
30	Ni	6.9517	12.1654	1.7379	0.5000	0.8750	0.0792
31	Ni	10.4275	8.6896	1.7379	0.7500	0.6250	0.0792
32	Ni	10.4275	12.1654	1.7379	0.7500	0.8750	0.0792
33	Ni	-0.0008	0.0030	3.5031	-0.0001	0.0002	0.1596
34	Ni	13.8924	3.4692	3.5019	0.9992	0.2495	0.1595
35	Ni	3.4780	0.0025	3.5070	0.2502	0.0002	0.1598
36	Ni	3.4933	3.4440	3.4861	0.2513	0.2477	0.1588
37	Ni	13.9007	6.9569	3.5021	0.9998	0.5004	0.1595
38	Ni	13.9000	10.4314	3.5064	0.9998	0.7503	0.1597
39	Ni	3.4594	6.9762	3.4726	0.2488	0.5018	0.1582

40	Ni	3.4690	10.4107	3.5198	0.2495	0.7488	0.1603
41	Ni	6.9547	-0.0014	3.4996	0.5002	-0.0001	0.1594
42	Ni	6.9443	3.4765	3.5035	0.4995	0.2500	0.1596
43	Ni	10.4321	13.8822	3.5246	0.7503	0.9985	0.1606
44	Ni	10.4248	3.4876	3.5000	0.7498	0.2508	0.1594
45	Ni	6.9348	6.9438	3.5264	0.4988	0.4994	0.1606
46	Ni	6.9677	10.4207	3.5026	0.5012	0.7495	0.1596
47	Ni	10.4218	6.9628	3.5172	0.7496	0.5008	0.1602
48	Ni	10.4419	10.4390	3.4397	0.7510	0.7508	0.1567
49	Ni	0.0019	1.7429	5.2110	0.0001	0.1254	0.2374
50	Ni	0.0055	5.2025	5.2080	0.0004	0.3742	0.2372
51	Ni	3.4749	1.7364	5.2163	0.2499	0.1249	0.2376
52	Ni	3.4252	5.1898	5.0761	0.2464	0.3733	0.2312
53	Ni	13.8967	8.6856	5.2077	0.9995	0.6247	0.2372
54	Ni	13.8999	12.1756	5.2085	0.9997	0.8757	0.2373
55	Ni	3.4631	8.7678	5.3596	0.2491	0.6306	0.2442
56	Ni	3.4794	12.1600	5.2107	0.2503	0.8746	0.2374
57	Ni	6.9788	1.7209	5.1980	0.5020	0.1238	0.2368
58	Ni	6.9181	5.2078	5.2555	0.4976	0.3746	0.2394
59	Ni	10.4216	1.7348	5.2056	0.7496	0.1248	0.2371
60	Ni	10.4122	5.2205	5.2216	0.7489	0.3755	0.2379
61	Ni	6.9817	8.7381	5.2270	0.5022	0.6285	0.2381
62	Ni	6.9485	12.1660	5.2060	0.4998	0.8750	0.2372
63	Ni	10.4596	8.6990	5.1660	0.7523	0.6257	0.2353
64	Ni	10.4447	12.1907	5.2603	0.7512	0.8768	0.2396
65	Co	1.7379	1.7379	0.0000	0.1250	0.1250	0.0000
66	Co	1.7379	5.2138	0.0000	0.1250	0.3750	0.0000
67	Co	5.2138	1.7379	0.0000	0.3750	0.1250	0.0000
68	Co	5.2138	5.2138	0.0000	0.3750	0.3750	0.0000
69	Co	1.7379	8.6896	0.0000	0.1250	0.6250	0.0000
70	Co	1.7379	12.1654	0.0000	0.1250	0.8750	0.0000
71	Co	5.2138	8.6896	0.0000	0.3750	0.6250	0.0000
72	Co	5.2138	12.1654	0.0000	0.3750	0.8750	0.0000
73	Co	8.6896	1.7379	0.0000	0.6250	0.1250	0.0000
74	Co	8.6896	5.2138	0.0000	0.6250	0.3750	0.0000
75	Co	12.1654	1.7379	0.0000	0.8750	0.1250	0.0000
76	Co	12.1654	5.2138	0.0000	0.8750	0.3750	0.0000
77	Co	8.6896	8.6896	0.0000	0.6250	0.6250	0.0000
78	Co	8.6896	12.1654	0.0000	0.6250	0.8750	0.0000
79	Co	12.1654	8.6896	0.0000	0.8750	0.6250	0.0000
80	Co	12.1654	12.1654	0.0000	0.8750	0.8750	0.0000
81	Co	1.7379	0.0000	1.7379	0.1250	0.0000	0.0792
82	Co	1.7379	3.4758	1.7379	0.1250	0.2500	0.0792
83	Co	5.2138	0.0000	1.7379	0.3750	0.0000	0.0792
84	Co	5.2138	3.4758	1.7379	0.3750	0.2500	0.0792
85	Co	1.7379	6.9517	1.7379	0.1250	0.5000	0.0792
86	Co	1.7379	10.4275	1.7379	0.1250	0.7500	0.0792
87	Co	5.2138	6.9517	1.7379	0.3750	0.5000	0.0792
88	Co	5.2138	10.4275	1.7379	0.3750	0.7500	0.0792

89	Co	8.6896	0.0000	1.7379	0.6250	0.0000	0.0792
90	Co	8.6896	3.4758	1.7379	0.6250	0.2500	0.0792
91	Co	12.1654	0.0000	1.7379	0.8750	0.0000	0.0792
92	Co	12.1654	3.4758	1.7379	0.8750	0.2500	0.0792
93	Co	8.6896	6.9517	1.7379	0.6250	0.5000	0.0792
94	Co	8.6896	10.4275	1.7379	0.6250	0.7500	0.0792
95	Co	12.1654	6.9517	1.7379	0.8750	0.5000	0.0792
96	Co	12.1654	10.4275	1.7379	0.8750	0.7500	0.0792
97	Co	1.7377	1.7345	3.5534	0.1250	0.1248	0.1619
98	Co	1.7032	5.2054	3.4996	0.1225	0.3744	0.1594
99	Co	5.2193	1.7431	3.5693	0.3754	0.1254	0.1626
100	Co	5.2181	5.2181	3.5278	0.3753	0.3753	0.1607
101	Co	1.7524	8.6940	3.5779	0.1260	0.6253	0.1630
102	Co	1.7392	12.1688	3.5520	0.1251	0.8752	0.1618
103	Co	5.2168	8.7154	3.5475	0.3752	0.6269	0.1616
104	Co	5.2142	12.1775	3.5304	0.3750	0.8759	0.1608
105	Co	8.6931	1.7351	3.5391	0.6253	0.1248	0.1612
106	Co	8.6664	5.2373	3.5747	0.6233	0.3767	0.1628
107	Co	12.1604	1.7401	3.5567	0.8746	0.1252	0.1620
108	Co	12.1614	5.2161	3.5598	0.8747	0.3752	0.1622
109	Co	8.6981	8.7036	3.5038	0.6256	0.6260	0.1596
110	Co	8.6949	12.1679	3.5424	0.6254	0.8752	0.1614
111	Co	12.1855	8.6966	3.5247	0.8764	0.6255	0.1606
112	Co	12.1679	12.1590	3.5569	0.8752	0.8745	0.1620
113	Co	1.7384	0.0005	5.2250	0.1250	0.0000	0.2380
114	Co	1.7460	3.4775	5.2124	0.1256	0.2501	0.2374
115	Co	5.2177	13.8993	5.2239	0.3753	0.9997	0.2380
116	Co	5.1967	3.4792	5.3184	0.3738	0.2502	0.2423
117	Co	1.7095	6.9220	5.2026	0.1230	0.4979	0.2370
118	Co	1.7030	10.4443	5.2269	0.1225	0.7512	0.2381
119	Co	5.1347	6.9910	5.1884	0.3693	0.5028	0.2364
120	Co	5.2416	10.4690	5.1887	0.3770	0.7530	0.2364
121	Co	8.6796	13.9004	5.2217	0.6243	0.9998	0.2379
122	Co	8.7206	3.4764	5.1976	0.6272	0.2500	0.2368
123	Co	12.1811	0.0126	5.2266	0.8761	0.0009	0.2381
124	Co	12.1538	3.4856	5.2283	0.8742	0.2507	0.2382
125	Co	8.7097	6.9747	5.2493	0.6264	0.5017	0.2391
126	Co	8.7348	10.4718	5.2114	0.6283	0.7532	0.2374
127	Co	12.1684	6.9479	5.2182	0.8752	0.4997	0.2377
128	Co	12.1586	10.4306	5.2814	0.8745	0.7502	0.2406
129	O	6.0804	4.3517	6.8498	0.4373	0.3130	0.3120
130	O	4.1656	7.6038	6.7783	0.2996	0.5469	0.3088
131	O	5.9567	6.5938	8.7949	0.4284	0.4743	0.4006
132	O	7.8089	7.8197	6.8248	0.5617	0.5624	0.3109
133	O	10.4210	10.3368	6.3453	0.7495	0.7435	0.2891
134	O	9.5301	6.9421	8.9021	0.6855	0.4993	0.4055
135	O	7.6712	9.7494	8.9248	0.5518	0.7012	0.4066
136	Ce	5.9370	8.8396	8.0307	0.4270	0.6358	0.3658
137	Ce	4.3196	5.4999	7.7036	0.3107	0.3956	0.3509

138 Ce	8.0122	5.7113	8.0511	0.5763	0.4108	0.3668
139 Sm	9.4353	8.9339	8.0715	0.6786	0.6426	0.3677

/db/jmorales/CoNi-alloy/metal-SDC-interface/Ni-Ce_3SmO_7/Ni-Ce_3SmO_7-conf2c

a =			13.9019837372				
b =			13.9019837372				
c =			21.9509918687				
alpha =			90.0				
beta =			90.0				
gamma =			90.0				
	Atom	X	Y	Z	X	Y	Z
1	Ni	0.0000	0.0000	0.0000	0.0000	0.0000	0.0000
2	Ni	1.7377	1.7377	0.0000	0.1250	0.1250	0.0000
3	Ni	0.0000	3.4755	0.0000	0.0000	0.2500	0.0000
4	Ni	1.7377	5.2132	0.0000	0.1250	0.3750	0.0000
5	Ni	3.4755	0.0000	0.0000	0.2500	0.0000	0.0000
6	Ni	5.2132	1.7377	0.0000	0.3750	0.1250	0.0000
7	Ni	3.4755	3.4755	0.0000	0.2500	0.2500	0.0000
8	Ni	5.2132	5.2132	0.0000	0.3750	0.3750	0.0000
9	Ni	0.0000	6.9510	0.0000	0.0000	0.5000	0.0000
10	Ni	1.7377	8.6887	0.0000	0.1250	0.6250	0.0000
11	Ni	0.0000	10.4265	0.0000	0.0000	0.7500	0.0000
12	Ni	1.7377	12.1642	0.0000	0.1250	0.8750	0.0000
13	Ni	3.4755	6.9510	0.0000	0.2500	0.5000	0.0000
14	Ni	5.2132	8.6887	0.0000	0.3750	0.6250	0.0000
15	Ni	3.4755	10.4265	0.0000	0.2500	0.7500	0.0000
16	Ni	5.2132	12.1642	0.0000	0.3750	0.8750	0.0000
17	Ni	6.9510	0.0000	0.0000	0.5000	0.0000	0.0000
18	Ni	8.6887	1.7377	0.0000	0.6250	0.1250	0.0000
19	Ni	6.9510	3.4755	0.0000	0.5000	0.2500	0.0000
20	Ni	8.6887	5.2132	0.0000	0.6250	0.3750	0.0000
21	Ni	10.4265	0.0000	0.0000	0.7500	0.0000	0.0000
22	Ni	12.1642	1.7377	0.0000	0.8750	0.1250	0.0000
23	Ni	10.4265	3.4755	0.0000	0.7500	0.2500	0.0000
24	Ni	12.1642	5.2132	0.0000	0.8750	0.3750	0.0000
25	Ni	6.9510	6.9510	0.0000	0.5000	0.5000	0.0000
26	Ni	8.6887	8.6887	0.0000	0.6250	0.6250	0.0000
27	Ni	6.9510	10.4265	0.0000	0.5000	0.7500	0.0000
28	Ni	8.6887	12.1642	0.0000	0.6250	0.8750	0.0000
29	Ni	10.4265	6.9510	0.0000	0.7500	0.5000	0.0000
30	Ni	12.1642	8.6887	0.0000	0.8750	0.6250	0.0000
31	Ni	10.4265	10.4265	0.0000	0.7500	0.7500	0.0000
32	Ni	12.1642	12.1642	0.0000	0.8750	0.8750	0.0000
33	Ni	0.0000	1.7377	1.7377	0.0000	0.1250	0.0792
34	Ni	1.7377	0.0000	1.7377	0.1250	0.0000	0.0792
35	Ni	0.0000	5.2132	1.7377	0.0000	0.3750	0.0792
36	Ni	1.7377	3.4755	1.7377	0.1250	0.2500	0.0792
37	Ni	3.4755	1.7377	1.7377	0.2500	0.1250	0.0792
38	Ni	5.2132	0.0000	1.7377	0.3750	0.0000	0.0792
39	Ni	3.4755	5.2132	1.7377	0.2500	0.3750	0.0792
40	Ni	5.2132	3.4755	1.7377	0.3750	0.2500	0.0792

41	Ni	0.0000	8.6887	1.7377	0.0000	0.6250	0.0792
42	Ni	1.7377	6.9510	1.7377	0.1250	0.5000	0.0792
43	Ni	0.0000	12.1642	1.7377	0.0000	0.8750	0.0792
44	Ni	1.7377	10.4265	1.7377	0.1250	0.7500	0.0792
45	Ni	3.4755	8.6887	1.7377	0.2500	0.6250	0.0792
46	Ni	5.2132	6.9510	1.7377	0.3750	0.5000	0.0792
47	Ni	3.4755	12.1642	1.7377	0.2500	0.8750	0.0792
48	Ni	5.2132	10.4265	1.7377	0.3750	0.7500	0.0792
49	Ni	6.9510	1.7377	1.7377	0.5000	0.1250	0.0792
50	Ni	8.6887	0.0000	1.7377	0.6250	0.0000	0.0792
51	Ni	6.9510	5.2132	1.7377	0.5000	0.3750	0.0792
52	Ni	8.6887	3.4755	1.7377	0.6250	0.2500	0.0792
53	Ni	10.4265	1.7377	1.7377	0.7500	0.1250	0.0792
54	Ni	12.1642	0.0000	1.7377	0.8750	0.0000	0.0792
55	Ni	10.4265	5.2132	1.7377	0.7500	0.3750	0.0792
56	Ni	12.1642	3.4755	1.7377	0.8750	0.2500	0.0792
57	Ni	6.9510	8.6887	1.7377	0.5000	0.6250	0.0792
58	Ni	8.6887	6.9510	1.7377	0.6250	0.5000	0.0792
59	Ni	6.9510	12.1642	1.7377	0.5000	0.8750	0.0792
60	Ni	8.6887	10.4265	1.7377	0.6250	0.7500	0.0792
61	Ni	10.4265	8.6887	1.7377	0.7500	0.6250	0.0792
62	Ni	12.1642	6.9510	1.7377	0.8750	0.5000	0.0792
63	Ni	10.4265	12.1642	1.7377	0.7500	0.8750	0.0792
64	Ni	12.1642	10.4265	1.7377	0.8750	0.7500	0.0792
65	Ni	-0.0007	13.8993	3.5089	-0.0000	0.9998	0.1599
66	Ni	1.7356	1.7367	3.5086	0.1248	0.1249	0.1598
67	Ni	0.0065	3.4777	3.5129	0.0005	0.2502	0.1600
68	Ni	1.7632	5.2099	3.5545	0.1268	0.3748	0.1619
69	Ni	3.4736	13.8991	3.5082	0.2499	0.9998	0.1598
70	Ni	5.2299	1.7135	3.4913	0.3762	0.1233	0.1591
71	Ni	3.4455	3.4933	3.4964	0.2478	0.2513	0.1593
72	Ni	5.2038	5.2327	3.4873	0.3743	0.3764	0.1589
73	Ni	0.0105	6.9420	3.5115	0.0008	0.4994	0.1600
74	Ni	1.7338	8.6921	3.5087	0.1247	0.6252	0.1598
75	Ni	13.8805	10.4271	3.5359	0.9985	0.7500	0.1611
76	Ni	1.7313	12.1632	3.5113	0.1245	0.8749	0.1600
77	Ni	3.4404	6.9299	3.5059	0.2475	0.4985	0.1597
78	Ni	5.1945	8.7219	3.4674	0.3737	0.6274	0.1580
79	Ni	3.4665	10.4386	3.5073	0.2494	0.7509	0.1598
80	Ni	5.2154	12.1681	3.5108	0.3752	0.8753	0.1599
81	Ni	6.9573	0.0212	3.5342	0.5005	0.0015	0.1610
82	Ni	8.6882	1.7300	3.5094	0.6250	0.1244	0.1599
83	Ni	6.9804	3.4918	3.5051	0.5021	0.2512	0.1597
84	Ni	8.6892	5.2015	3.5030	0.6250	0.3742	0.1596
85	Ni	10.4320	13.8704	3.5291	0.7504	0.9977	0.1608
86	Ni	12.1653	1.7351	3.5050	0.8751	0.1248	0.1597
87	Ni	10.4045	3.4629	3.5177	0.7484	0.2491	0.1603
88	Ni	12.1730	5.2082	3.5001	0.8756	0.3746	0.1594
89	Ni	6.9586	6.9586	3.4701	0.5005	0.5005	0.1581

90	Ni	8.6864	8.6908	3.4782	0.6248	0.6252	0.1585
91	Ni	6.9638	10.4407	3.5080	0.5009	0.7510	0.1598
92	Ni	8.6904	12.1653	3.5047	0.6251	0.8751	0.1597
93	Ni	10.4254	6.9596	3.5047	0.7499	0.5006	0.1597
94	Ni	12.1821	8.6852	3.4978	0.8763	0.6247	0.1593
95	Ni	10.4343	10.4353	3.4447	0.7506	0.7506	0.1569
96	Ni	12.1612	12.1545	3.5220	0.8748	0.8743	0.1604
97	Ni	13.8984	1.7341	5.2238	0.9997	0.1247	0.2380
98	Ni	1.7395	0.0059	5.2257	0.1251	0.0004	0.2381
99	Ni	-0.0006	5.2122	5.2307	-0.0000	0.3749	0.2383
100	Ni	1.7383	3.4607	5.2331	0.1250	0.2489	0.2384
101	Ni	3.4909	1.7404	5.2107	0.2511	0.1252	0.2374
102	Ni	5.2098	0.0060	5.2321	0.3748	0.0004	0.2384
103	Ni	3.5487	5.2184	5.5217	0.2553	0.3754	0.2515
104	Ni	5.2037	3.4664	5.0643	0.3743	0.2493	0.2307
105	Ni	0.0066	8.6749	5.2286	0.0005	0.6240	0.2382
106	Ni	1.7422	6.9526	5.2386	0.1253	0.5001	0.2387
107	Ni	-0.0001	12.1683	5.2313	-0.0000	0.8753	0.2383
108	Ni	1.7341	10.4254	5.2279	0.1247	0.7499	0.2382
109	Ni	3.4736	8.6931	5.2162	0.2499	0.6253	0.2376
110	Ni	5.1800	7.0093	5.0883	0.3726	0.5042	0.2318
111	Ni	3.4728	12.1685	5.2246	0.2498	0.8753	0.2380
112	Ni	5.2071	10.4266	5.2223	0.3746	0.7500	0.2379
113	Ni	6.9612	1.7147	5.3615	0.5007	0.1233	0.2442
114	Ni	8.7041	13.8811	5.2176	0.6261	0.9985	0.2377
115	Ni	6.9044	5.2832	5.2872	0.4966	0.3800	0.2409
116	Ni	8.7168	3.4616	5.3179	0.6270	0.2490	0.2423
117	Ni	10.4493	1.7189	5.2132	0.7516	0.1236	0.2375
118	Ni	12.1668	13.8936	5.2254	0.8752	0.9994	0.2380
119	Ni	10.4452	5.1945	5.1829	0.7513	0.3736	0.2361
120	Ni	12.1611	3.4751	5.2232	0.8748	0.2500	0.2379
121	Ni	6.9187	8.7117	5.1652	0.4977	0.6267	0.2353
122	Ni	8.7513	6.9827	5.2240	0.6295	0.5023	0.2380
123	Ni	6.9500	12.1668	5.2218	0.4999	0.8752	0.2379
124	Ni	8.6703	10.4120	5.2531	0.6237	0.7490	0.2393
125	Ni	10.4458	8.6885	5.2003	0.7514	0.6250	0.2369
126	Ni	12.1606	6.9418	5.2194	0.8747	0.4993	0.2378
127	Ni	10.4165	12.1268	5.3139	0.7493	0.8723	0.2421
128	Ni	12.1568	10.4082	5.3070	0.8745	0.7487	0.2418
129	O	7.6206	2.8527	6.8163	0.5482	0.2052	0.3105
130	O	4.1802	5.0180	7.3051	0.3007	0.3610	0.3328
131	O	6.6597	4.8492	8.7240	0.4791	0.3488	0.3974
132	O	7.7337	6.4306	6.7259	0.5563	0.4626	0.3064
133	O	10.3122	10.2861	6.3137	0.7418	0.7399	0.2876
134	O	9.7693	6.7200	8.7273	0.7027	0.4834	0.3976
135	O	7.0101	8.5541	8.5862	0.5043	0.6153	0.3912
136	Ce	5.7076	6.9686	7.8932	0.4106	0.5013	0.3596
137	Ce	5.5568	3.4157	7.5774	0.3997	0.2457	0.3452
138	Ce	8.9408	4.8872	7.9939	0.6431	0.3515	0.3642

139 Sm	8.9776	8.4701	7.8001	0.6458	0.6093	0.3553

/db/jmorales/CoNi-alloy/metal-SDC-interface/Ni-Ce_3SmO_7/Ni-Ce_3SmO_7-conf3b

a = 13.9019837372
b = 13.9019837372
c = 21.9509918687
alpha = 90.0
beta = 90.0
gamma = 90.0

	Atom	X	Y	Z	X	Y	Z
1	Ni	0.0000	0.0000	0.0000	0.0000	0.0000	0.0000
2	Ni	1.7377	1.7377	0.0000	0.1250	0.1250	0.0000
3	Ni	0.0000	3.4755	0.0000	0.0000	0.2500	0.0000
4	Ni	1.7377	5.2132	0.0000	0.1250	0.3750	0.0000
5	Ni	3.4755	0.0000	0.0000	0.2500	0.0000	0.0000
6	Ni	5.2132	1.7377	0.0000	0.3750	0.1250	0.0000
7	Ni	3.4755	3.4755	0.0000	0.2500	0.2500	0.0000
8	Ni	5.2132	5.2132	0.0000	0.3750	0.3750	0.0000
9	Ni	0.0000	6.9510	0.0000	0.0000	0.5000	0.0000
10	Ni	1.7377	8.6887	0.0000	0.1250	0.6250	0.0000
11	Ni	0.0000	10.4265	0.0000	0.0000	0.7500	0.0000
12	Ni	1.7377	12.1642	0.0000	0.1250	0.8750	0.0000
13	Ni	3.4755	6.9510	0.0000	0.2500	0.5000	0.0000
14	Ni	5.2132	8.6887	0.0000	0.3750	0.6250	0.0000
15	Ni	3.4755	10.4265	0.0000	0.2500	0.7500	0.0000
16	Ni	5.2132	12.1642	0.0000	0.3750	0.8750	0.0000
17	Ni	6.9510	0.0000	0.0000	0.5000	0.0000	0.0000
18	Ni	8.6887	1.7377	0.0000	0.6250	0.1250	0.0000
19	Ni	6.9510	3.4755	0.0000	0.5000	0.2500	0.0000
20	Ni	8.6887	5.2132	0.0000	0.6250	0.3750	0.0000
21	Ni	10.4265	0.0000	0.0000	0.7500	0.0000	0.0000
22	Ni	12.1642	1.7377	0.0000	0.8750	0.1250	0.0000
23	Ni	10.4265	3.4755	0.0000	0.7500	0.2500	0.0000
24	Ni	12.1642	5.2132	0.0000	0.8750	0.3750	0.0000
25	Ni	6.9510	6.9510	0.0000	0.5000	0.5000	0.0000
26	Ni	8.6887	8.6887	0.0000	0.6250	0.6250	0.0000
27	Ni	6.9510	10.4265	0.0000	0.5000	0.7500	0.0000
28	Ni	8.6887	12.1642	0.0000	0.6250	0.8750	0.0000
29	Ni	10.4265	6.9510	0.0000	0.7500	0.5000	0.0000
30	Ni	12.1642	8.6887	0.0000	0.8750	0.6250	0.0000
31	Ni	10.4265	10.4265	0.0000	0.7500	0.7500	0.0000
32	Ni	12.1642	12.1642	0.0000	0.8750	0.8750	0.0000
33	Ni	0.0000	1.7377	1.7377	0.0000	0.1250	0.0792
34	Ni	1.7377	0.0000	1.7377	0.1250	0.0000	0.0792
35	Ni	0.0000	5.2132	1.7377	0.0000	0.3750	0.0792
36	Ni	1.7377	3.4755	1.7377	0.1250	0.2500	0.0792
37	Ni	3.4755	1.7377	1.7377	0.2500	0.1250	0.0792
38	Ni	5.2132	0.0000	1.7377	0.3750	0.0000	0.0792
39	Ni	3.4755	5.2132	1.7377	0.2500	0.3750	0.0792
40	Ni	5.2132	3.4755	1.7377	0.3750	0.2500	0.0792

41	Ni	0.0000	8.6887	1.7377	0.0000	0.6250	0.0792
42	Ni	1.7377	6.9510	1.7377	0.1250	0.5000	0.0792
43	Ni	0.0000	12.1642	1.7377	0.0000	0.8750	0.0792
44	Ni	1.7377	10.4265	1.7377	0.1250	0.7500	0.0792
45	Ni	3.4755	8.6887	1.7377	0.2500	0.6250	0.0792
46	Ni	5.2132	6.9510	1.7377	0.3750	0.5000	0.0792
47	Ni	3.4755	12.1642	1.7377	0.2500	0.8750	0.0792
48	Ni	5.2132	10.4265	1.7377	0.3750	0.7500	0.0792
49	Ni	6.9510	1.7377	1.7377	0.5000	0.1250	0.0792
50	Ni	8.6887	0.0000	1.7377	0.6250	0.0000	0.0792
51	Ni	6.9510	5.2132	1.7377	0.5000	0.3750	0.0792
52	Ni	8.6887	3.4755	1.7377	0.6250	0.2500	0.0792
53	Ni	10.4265	1.7377	1.7377	0.7500	0.1250	0.0792
54	Ni	12.1642	0.0000	1.7377	0.8750	0.0000	0.0792
55	Ni	10.4265	5.2132	1.7377	0.7500	0.3750	0.0792
56	Ni	12.1642	3.4755	1.7377	0.8750	0.2500	0.0792
57	Ni	6.9510	8.6887	1.7377	0.5000	0.6250	0.0792
58	Ni	8.6887	6.9510	1.7377	0.6250	0.5000	0.0792
59	Ni	6.9510	12.1642	1.7377	0.5000	0.8750	0.0792
60	Ni	8.6887	10.4265	1.7377	0.6250	0.7500	0.0792
61	Ni	10.4265	8.6887	1.7377	0.7500	0.6250	0.0792
62	Ni	12.1642	6.9510	1.7377	0.8750	0.5000	0.0792
63	Ni	10.4265	12.1642	1.7377	0.7500	0.8750	0.0792
64	Ni	12.1642	10.4265	1.7377	0.8750	0.7500	0.0792
65	Ni	-0.0000	-0.0003	3.5114	-0.0000	-0.0000	0.1600
66	Ni	1.7446	1.7340	3.5049	0.1255	0.1247	0.1597
67	Ni	13.8926	3.4712	3.5020	0.9993	0.2497	0.1595
68	Ni	1.7254	5.2179	3.5042	0.1241	0.3753	0.1596
69	Ni	3.4812	0.0031	3.5147	0.2504	0.0002	0.1601
70	Ni	5.2075	1.7598	3.5410	0.3746	0.1266	0.1613
71	Ni	3.4989	3.4912	3.5368	0.2517	0.2511	0.1611
72	Ni	5.2087	5.2015	3.5074	0.3747	0.3742	0.1598
73	Ni	0.0086	6.9438	3.5305	0.0006	0.4995	0.1608
74	Ni	1.7096	8.6722	3.5048	0.1230	0.6238	0.1597
75	Ni	-0.0003	10.4324	3.5096	-0.0000	0.7504	0.1599
76	Ni	1.7328	12.1743	3.5124	0.1246	0.8757	0.1600
77	Ni	3.4711	6.9542	3.4833	0.2497	0.5002	0.1587
78	Ni	5.2170	8.7058	3.4717	0.3753	0.6262	0.1582
79	Ni	3.4672	10.4591	3.4843	0.2494	0.7523	0.1587
80	Ni	5.2195	12.1726	3.5082	0.3754	0.8756	0.1598
81	Ni	6.9500	13.9017	3.5112	0.4999	1.0000	0.1600
82	Ni	8.6908	1.7484	3.5253	0.6251	0.1258	0.1606
83	Ni	6.9396	3.4467	3.4896	0.4992	0.2479	0.1590
84	Ni	8.7231	5.2067	3.4257	0.6275	0.3745	0.1561
85	Ni	10.4237	13.8962	3.5106	0.7498	0.9996	0.1599
86	Ni	12.1660	1.7275	3.5085	0.8751	0.1243	0.1598
87	Ni	10.4334	3.4670	3.5069	0.7505	0.2494	0.1598
88	Ni	12.1412	5.2132	3.5297	0.8733	0.3750	0.1608
89	Ni	6.9607	6.9615	3.4571	0.5007	0.5008	0.1575

90	Ni	8.6881	8.6757	3.5255	0.6250	0.6241	0.1606
91	Ni	6.9545	10.4191	3.5153	0.5003	0.7495	0.1601
92	Ni	8.6861	12.1609	3.5109	0.6248	0.8748	0.1599
93	Ni	10.4379	6.9581	3.5046	0.7508	0.5005	0.1597
94	Ni	12.1676	8.6926	3.5102	0.8752	0.6253	0.1599
95	Ni	10.4237	10.4265	3.5101	0.7498	0.7500	0.1599
96	Ni	12.1621	12.1643	3.5113	0.8748	0.8750	0.1600
97	Ni	0.0009	1.7371	5.2293	0.0001	0.1250	0.2382
98	Ni	1.7427	0.0100	5.2323	0.1254	0.0007	0.2384
99	Ni	13.8793	5.1847	5.2187	0.9984	0.3729	0.2377
100	Ni	1.7343	3.4501	5.1991	0.1248	0.2482	0.2369
101	Ni	3.4721	1.7502	5.2395	0.2498	0.1259	0.2387
102	Ni	5.2101	13.9000	5.2314	0.3748	0.9999	0.2383
103	Ni	3.4465	5.2464	5.2321	0.2479	0.3774	0.2384
104	Ni	5.2628	3.4984	5.4276	0.3786	0.2516	0.2473
105	Ni	13.8886	8.7067	5.2317	0.9990	0.6263	0.2383
106	Ni	1.6971	7.0029	5.3790	0.1221	0.5037	0.2450
107	Ni	0.0032	12.1662	5.2291	0.0002	0.8751	0.2382
108	Ni	1.7414	10.4213	5.2221	0.1253	0.7496	0.2379
109	Ni	3.4648	8.7303	5.0998	0.2492	0.6280	0.2323
110	Ni	5.2063	6.9330	5.1900	0.3745	0.4987	0.2364
111	Ni	3.4692	12.1671	5.2312	0.2495	0.8752	0.2383
112	Ni	5.1921	10.4462	5.2078	0.3735	0.7514	0.2372
113	Ni	6.9619	1.7105	5.2231	0.5008	0.1230	0.2379
114	Ni	8.6930	13.8931	5.2291	0.6253	0.9994	0.2382
115	Ni	6.9883	5.2146	5.0916	0.5027	0.3751	0.2320
116	Ni	8.6955	3.4630	5.2695	0.6255	0.2491	0.2401
117	Ni	10.4300	1.7281	5.2234	0.7503	0.1243	0.2380
118	Ni	12.1626	13.8981	5.2278	0.8749	0.9997	0.2382
119	Ni	10.4361	5.2037	5.2918	0.7507	0.3743	0.2411
120	Ni	12.1651	3.4569	5.2247	0.8751	0.2487	0.2380
121	Ni	6.9559	8.7056	5.2425	0.5004	0.6262	0.2388
122	Ni	8.7249	6.9479	5.2453	0.6276	0.4998	0.2390
123	Ni	6.9551	12.1525	5.2421	0.5003	0.8742	0.2388
124	Ni	8.6803	10.4185	5.2420	0.6244	0.7494	0.2388
125	Ni	10.4281	8.7036	5.2312	0.7501	0.6261	0.2383
126	Ni	12.1686	6.9598	5.2274	0.8753	0.5006	0.2381
127	Ni	10.4181	12.1560	5.2342	0.7494	0.8744	0.2385
128	Ni	12.1620	10.4344	5.2276	0.8748	0.7506	0.2382
129	O	5.6508	3.8688	7.2776	0.4065	0.2783	0.3315
130	O	2.6082	6.1158	6.8145	0.1876	0.4399	0.3104
131	O	4.7286	6.0425	8.7356	0.3401	0.4346	0.3980
132	O	8.6131	5.2121	6.2828	0.6196	0.3749	0.2862
133	O	6.0762	7.6927	6.7628	0.4371	0.5534	0.3081
134	O	7.8245	7.3081	8.8473	0.5628	0.5257	0.4030
135	O	5.3099	9.5114	8.7774	0.3819	0.6842	0.3999
136	Ce	3.9362	8.0813	7.8364	0.2831	0.5813	0.3570
137	Ce	3.5346	4.3105	7.8650	0.2543	0.3101	0.3583
138	Ce	6.8384	5.7574	7.8630	0.4919	0.4141	0.3582

139 Sm	7.2426	9.2473	8.0315	0.5210	0.6652	0.3659

/db/jmorales/CoNi-alloy/metal-SDC-interface/CoNi-Ce_3SmO_7/CoNi-Ce_3SmO_7-conf2d

a =			13.9033384264				
b =			13.9033384264				
c =			21.9516692133				
alpha =			90.0				
beta =			90.0				
gamma =			90.0				
	Atom	X	Y	Z	X	Y	Z
1	Ni	0.0000	0.0000	0.0000	0.0000	0.0000	0.0000
2	Ni	0.0000	3.4758	0.0000	0.0000	0.2500	0.0000
3	Ni	3.4758	0.0000	0.0000	0.2500	0.0000	0.0000
4	Ni	3.4758	3.4758	0.0000	0.2500	0.2500	0.0000
5	Ni	0.0000	6.9517	0.0000	0.0000	0.5000	0.0000
6	Ni	0.0000	10.4275	0.0000	0.0000	0.7500	0.0000
7	Ni	3.4758	6.9517	0.0000	0.2500	0.5000	0.0000
8	Ni	3.4758	10.4275	0.0000	0.2500	0.7500	0.0000
9	Ni	6.9517	0.0000	0.0000	0.5000	0.0000	0.0000
10	Ni	6.9517	3.4758	0.0000	0.5000	0.2500	0.0000
11	Ni	10.4275	0.0000	0.0000	0.7500	0.0000	0.0000
12	Ni	10.4275	3.4758	0.0000	0.7500	0.2500	0.0000
13	Ni	6.9517	6.9517	0.0000	0.5000	0.5000	0.0000
14	Ni	6.9517	10.4275	0.0000	0.5000	0.7500	0.0000
15	Ni	10.4275	6.9517	0.0000	0.7500	0.5000	0.0000
16	Ni	10.4275	10.4275	0.0000	0.7500	0.7500	0.0000
17	Ni	0.0000	1.7379	1.7379	0.0000	0.1250	0.0792
18	Ni	0.0000	5.2138	1.7379	0.0000	0.3750	0.0792
19	Ni	3.4758	1.7379	1.7379	0.2500	0.1250	0.0792
20	Ni	3.4758	5.2138	1.7379	0.2500	0.3750	0.0792
21	Ni	0.0000	8.6896	1.7379	0.0000	0.6250	0.0792
22	Ni	0.0000	12.1654	1.7379	0.0000	0.8750	0.0792
23	Ni	3.4758	8.6896	1.7379	0.2500	0.6250	0.0792
24	Ni	3.4758	12.1654	1.7379	0.2500	0.8750	0.0792
25	Ni	6.9517	1.7379	1.7379	0.5000	0.1250	0.0792
26	Ni	6.9517	5.2138	1.7379	0.5000	0.3750	0.0792
27	Ni	10.4275	1.7379	1.7379	0.7500	0.1250	0.0792
28	Ni	10.4275	5.2138	1.7379	0.7500	0.3750	0.0792
29	Ni	6.9517	8.6896	1.7379	0.5000	0.6250	0.0792
30	Ni	6.9517	12.1654	1.7379	0.5000	0.8750	0.0792
31	Ni	10.4275	8.6896	1.7379	0.7500	0.6250	0.0792
32	Ni	10.4275	12.1654	1.7379	0.7500	0.8750	0.0792
33	Ni	-0.0020	13.9012	3.5016	-0.0001	0.9998	0.1595
34	Ni	-0.0002	3.4826	3.5136	-0.0000	0.2505	0.1601
35	Ni	3.4626	13.8722	3.4651	0.2490	0.9978	0.1579
36	Ni	3.4725	3.4800	3.4926	0.2498	0.2503	0.1591
37	Ni	13.8904	6.9580	3.4941	0.9991	0.5005	0.1592
38	Ni	-0.0002	10.4237	3.5058	-0.0000	0.7497	0.1597
39	Ni	3.4906	6.9251	3.5222	0.2511	0.4981	0.1605

40	Ni	3.4778	10.4226	3.5046	0.2501	0.7496	0.1597
41	Ni	6.9570	0.0114	3.5186	0.5004	0.0008	0.1603
42	Ni	6.9648	3.4857	3.5000	0.5009	0.2507	0.1594
43	Ni	10.4280	0.0002	3.5038	0.7500	0.0000	0.1596
44	Ni	10.4356	3.4708	3.4905	0.7506	0.2496	0.1590
45	Ni	6.9644	6.9596	3.4807	0.5009	0.5006	0.1586
46	Ni	6.9563	10.4393	3.4927	0.5003	0.7508	0.1591
47	Ni	10.4295	6.9603	3.4992	0.7501	0.5006	0.1594
48	Ni	10.4229	10.4295	3.5068	0.7497	0.7501	0.1597
49	Ni	0.0014	1.7329	5.2159	0.0001	0.1246	0.2376
50	Ni	13.8778	5.2429	5.1935	0.9982	0.3771	0.2366
51	Ni	3.4444	1.6895	5.0779	0.2477	0.1215	0.2313
52	Ni	3.4459	5.1891	5.2405	0.2478	0.3732	0.2387
53	Ni	0.0001	8.6886	5.2143	0.0000	0.6249	0.2375
54	Ni	0.0053	12.1654	5.2089	0.0004	0.8750	0.2373
55	Ni	3.4798	8.6757	5.2192	0.2503	0.6240	0.2378
56	Ni	3.4593	12.1630	5.2077	0.2488	0.8748	0.2372
57	Ni	6.9862	1.6871	5.3544	0.5025	0.1213	0.2439
58	Ni	7.0005	5.2263	5.2228	0.5035	0.3759	0.2379
59	Ni	10.4247	1.7395	5.2122	0.7498	0.1251	0.2374
60	Ni	10.4217	5.2188	5.2056	0.7496	0.3754	0.2371
61	Ni	6.9440	8.7241	5.1653	0.4994	0.6275	0.2353
62	Ni	6.9558	12.1659	5.2074	0.5003	0.8750	0.2372
63	Ni	10.4480	8.7147	5.2551	0.7515	0.6268	0.2394
64	Ni	10.4280	12.1564	5.2066	0.7500	0.8744	0.2372
65	Co	1.7379	1.7379	0.0000	0.1250	0.1250	0.0000
66	Co	1.7379	5.2138	0.0000	0.1250	0.3750	0.0000
67	Co	5.2138	1.7379	0.0000	0.3750	0.1250	0.0000
68	Co	5.2138	5.2138	0.0000	0.3750	0.3750	0.0000
69	Co	1.7379	8.6896	0.0000	0.1250	0.6250	0.0000
70	Co	1.7379	12.1654	0.0000	0.1250	0.8750	0.0000
71	Co	5.2138	8.6896	0.0000	0.3750	0.6250	0.0000
72	Co	5.2138	12.1654	0.0000	0.3750	0.8750	0.0000
73	Co	8.6896	1.7379	0.0000	0.6250	0.1250	0.0000
74	Co	8.6896	5.2138	0.0000	0.6250	0.3750	0.0000
75	Co	12.1654	1.7379	0.0000	0.8750	0.1250	0.0000
76	Co	12.1654	5.2138	0.0000	0.8750	0.3750	0.0000
77	Co	8.6896	8.6896	0.0000	0.6250	0.6250	0.0000
78	Co	8.6896	12.1654	0.0000	0.6250	0.8750	0.0000
79	Co	12.1654	8.6896	0.0000	0.8750	0.6250	0.0000
80	Co	12.1654	12.1654	0.0000	0.8750	0.8750	0.0000
81	Co	1.7379	0.0000	1.7379	0.1250	0.0000	0.0792
82	Co	1.7379	3.4758	1.7379	0.1250	0.2500	0.0792
83	Co	5.2138	0.0000	1.7379	0.3750	0.0000	0.0792
84	Co	5.2138	3.4758	1.7379	0.3750	0.2500	0.0792
85	Co	1.7379	6.9517	1.7379	0.1250	0.5000	0.0792
86	Co	1.7379	10.4275	1.7379	0.1250	0.7500	0.0792
87	Co	5.2138	6.9517	1.7379	0.3750	0.5000	0.0792
88	Co	5.2138	10.4275	1.7379	0.3750	0.7500	0.0792

89	Co	8.6896	0.0000	1.7379	0.6250	0.0000	0.0792
90	Co	8.6896	3.4758	1.7379	0.6250	0.2500	0.0792
91	Co	12.1654	0.0000	1.7379	0.8750	0.0000	0.0792
92	Co	12.1654	3.4758	1.7379	0.8750	0.2500	0.0792
93	Co	8.6896	6.9517	1.7379	0.6250	0.5000	0.0792
94	Co	8.6896	10.4275	1.7379	0.6250	0.7500	0.0792
95	Co	12.1654	6.9517	1.7379	0.8750	0.5000	0.0792
96	Co	12.1654	10.4275	1.7379	0.8750	0.7500	0.0792
97	Co	1.7010	1.7574	3.5235	0.1223	0.1264	0.1605
98	Co	1.7268	5.2089	3.5314	0.1242	0.3747	0.1609
99	Co	5.2396	1.7093	3.5143	0.3769	0.1229	0.1601
100	Co	5.2058	5.1995	3.5615	0.3744	0.3740	0.1622
101	Co	1.7431	8.6907	3.5466	0.1254	0.6251	0.1616
102	Co	1.7312	12.1564	3.5475	0.1245	0.8743	0.1616
103	Co	5.2129	8.6840	3.5617	0.3749	0.6246	0.1623
104	Co	5.2166	12.1571	3.5536	0.3752	0.8744	0.1619
105	Co	8.6732	1.7283	3.5665	0.6238	0.1243	0.1625
106	Co	8.6917	5.2245	3.5413	0.6251	0.3758	0.1613
107	Co	12.1675	1.7363	3.5609	0.8752	0.1249	0.1622
108	Co	12.1559	5.2180	3.5501	0.8743	0.3753	0.1617
109	Co	8.7042	8.7058	3.4595	0.6261	0.6262	0.1576
110	Co	8.6897	12.1583	3.5520	0.6250	0.8745	0.1618
111	Co	12.1533	8.6972	3.5715	0.8741	0.6255	0.1627
112	Co	12.1657	12.1654	3.5524	0.8750	0.8750	0.1618
113	Co	1.7387	0.0046	5.2160	0.1251	0.0003	0.2376
114	Co	1.7057	3.4834	5.3220	0.1227	0.2505	0.2424
115	Co	5.1735	13.8605	5.1983	0.3721	0.9969	0.2368
116	Co	5.2460	3.3878	5.1692	0.3773	0.2437	0.2355
117	Co	1.7288	6.9838	5.1897	0.1243	0.5023	0.2364
118	Co	1.7429	10.4166	5.2331	0.1254	0.7492	0.2384
119	Co	5.2224	6.9449	5.2277	0.3756	0.4995	0.2381
120	Co	5.2036	10.4338	5.2230	0.3743	0.7505	0.2379
121	Co	8.7129	13.8815	5.2257	0.6267	0.9984	0.2381
122	Co	8.7260	3.4913	5.1788	0.6276	0.2511	0.2359
123	Co	12.1627	13.8958	5.2250	0.8748	0.9995	0.2380
124	Co	12.1613	3.4805	5.2204	0.8747	0.2503	0.2378
125	Co	8.7337	7.0069	5.2152	0.6282	0.5040	0.2376
126	Co	8.6833	10.4188	5.3001	0.6245	0.7494	0.2414
127	Co	12.1615	6.9523	5.2177	0.8747	0.5000	0.2377
128	Co	12.1790	10.4421	5.2240	0.8760	0.7510	0.2380
129	O	5.8754	2.4920	6.7846	0.4226	0.1792	0.3091
130	O	2.6228	4.3358	6.8363	0.1886	0.3119	0.3114
131	O	4.8411	4.2602	8.7918	0.3482	0.3064	0.4005
132	O	6.0929	6.1044	6.8162	0.4382	0.4391	0.3105
133	O	8.6009	8.6861	6.3376	0.6186	0.6247	0.2887
134	O	7.9857	5.9872	8.9430	0.5744	0.4306	0.4074
135	O	5.1698	7.8399	8.8654	0.3718	0.5639	0.4039
136	Ce	3.9555	6.3067	8.0238	0.2845	0.4536	0.3655
137	Ce	3.7698	2.6007	7.7093	0.2711	0.1871	0.3512

138 Ce	7.0926	4.2460	8.0409	0.5101	0.3054	0.3663
139 Sm	7.1756	7.7417	8.0680	0.5161	0.5568	0.3675

/db/jmorales/CoNi-alloy/metal-SDC-interface/CoNi-Ce_3SmO_7/CoNi-Ce_3SmO_7-conf1d

a =			13.9033384264				
b =			13.9033384264				
c =			21.9516692133				
alpha =			90.0				
beta =			90.0				
gamma =			90.0				
	Atom	X	Y	Z	X	Y	Z
1	Ni	0.0000	0.0000	0.0000	0.0000	0.0000	0.0000
2	Ni	0.0000	3.4758	0.0000	0.0000	0.2500	0.0000
3	Ni	3.4758	0.0000	0.0000	0.2500	0.0000	0.0000
4	Ni	3.4758	3.4758	0.0000	0.2500	0.2500	0.0000
5	Ni	0.0000	6.9517	0.0000	0.0000	0.5000	0.0000
6	Ni	0.0000	10.4275	0.0000	0.0000	0.7500	0.0000
7	Ni	3.4758	6.9517	0.0000	0.2500	0.5000	0.0000
8	Ni	3.4758	10.4275	0.0000	0.2500	0.7500	0.0000
9	Ni	6.9517	0.0000	0.0000	0.5000	0.0000	0.0000
10	Ni	6.9517	3.4758	0.0000	0.5000	0.2500	0.0000
11	Ni	10.4275	0.0000	0.0000	0.7500	0.0000	0.0000
12	Ni	10.4275	3.4758	0.0000	0.7500	0.2500	0.0000
13	Ni	6.9517	6.9517	0.0000	0.5000	0.5000	0.0000
14	Ni	6.9517	10.4275	0.0000	0.5000	0.7500	0.0000
15	Ni	10.4275	6.9517	0.0000	0.7500	0.5000	0.0000
16	Ni	10.4275	10.4275	0.0000	0.7500	0.7500	0.0000
17	Ni	0.0000	1.7379	1.7379	0.0000	0.1250	0.0792
18	Ni	0.0000	5.2138	1.7379	0.0000	0.3750	0.0792
19	Ni	3.4758	1.7379	1.7379	0.2500	0.1250	0.0792
20	Ni	3.4758	5.2138	1.7379	0.2500	0.3750	0.0792
21	Ni	0.0000	8.6896	1.7379	0.0000	0.6250	0.0792
22	Ni	0.0000	12.1654	1.7379	0.0000	0.8750	0.0792
23	Ni	3.4758	8.6896	1.7379	0.2500	0.6250	0.0792
24	Ni	3.4758	12.1654	1.7379	0.2500	0.8750	0.0792
25	Ni	6.9517	1.7379	1.7379	0.5000	0.1250	0.0792
26	Ni	6.9517	5.2138	1.7379	0.5000	0.3750	0.0792
27	Ni	10.4275	1.7379	1.7379	0.7500	0.1250	0.0792
28	Ni	10.4275	5.2138	1.7379	0.7500	0.3750	0.0792
29	Ni	6.9517	8.6896	1.7379	0.5000	0.6250	0.0792
30	Ni	6.9517	12.1654	1.7379	0.5000	0.8750	0.0792
31	Ni	10.4275	8.6896	1.7379	0.7500	0.6250	0.0792
32	Ni	10.4275	12.1654	1.7379	0.7500	0.8750	0.0792
33	Ni	0.0004	-0.0004	3.5012	0.0000	-0.0000	0.1595
34	Ni	13.8884	3.4742	3.4868	0.9989	0.2499	0.1588
35	Ni	3.4794	-0.0001	3.5019	0.2503	-0.0000	0.1595
36	Ni	3.4989	3.4968	3.5359	0.2517	0.2515	0.1611
37	Ni	0.0039	6.9564	3.5149	0.0003	0.5003	0.1601
38	Ni	0.0021	10.4250	3.5047	0.0001	0.7498	0.1597
39	Ni	3.4800	6.9573	3.4947	0.2503	0.5004	0.1592

40	Ni	3.4724	10.4480	3.4768	0.2498	0.7515	0.1584
41	Ni	6.9484	0.0052	3.5042	0.4998	0.0004	0.1596
42	Ni	6.9316	3.4382	3.4886	0.4986	0.2473	0.1589
43	Ni	10.4289	-0.0007	3.5000	0.7501	-0.0001	0.1594
44	Ni	10.4444	3.4652	3.4946	0.7512	0.2492	0.1592
45	Ni	6.9657	6.9533	3.4542	0.5010	0.5001	0.1574
46	Ni	6.9633	10.4137	3.5232	0.5008	0.7490	0.1605
47	Ni	10.4384	6.9816	3.5126	0.7508	0.5022	0.1600
48	Ni	10.4159	10.4212	3.5163	0.7492	0.7495	0.1602
49	Ni	0.0066	1.7367	5.2101	0.0005	0.1249	0.2373
50	Ni	13.8823	5.1931	5.1983	0.9985	0.3735	0.2368
51	Ni	3.4393	1.7308	5.2163	0.2474	0.1245	0.2376
52	Ni	3.4412	5.2483	5.2206	0.2475	0.3775	0.2378
53	Ni	0.0025	8.6943	5.2145	0.0002	0.6253	0.2375
54	Ni	0.0079	12.1663	5.2032	0.0006	0.8751	0.2370
55	Ni	3.4942	8.7302	5.1310	0.2513	0.6279	0.2337
56	Ni	3.4672	12.1727	5.2051	0.2494	0.8755	0.2371
57	Ni	6.9884	1.7193	5.2122	0.5026	0.1237	0.2374
58	Ni	7.0066	5.1799	5.0644	0.5040	0.3726	0.2307
59	Ni	10.4252	1.7323	5.2074	0.7498	0.1246	0.2372
60	Ni	10.4388	5.2046	5.2047	0.7508	0.3743	0.2371
61	Ni	6.9102	8.6899	5.2291	0.4970	0.6250	0.2382
62	Ni	6.9585	12.1655	5.2049	0.5005	0.8750	0.2371
63	Ni	10.4061	8.6576	5.3870	0.7485	0.6227	0.2454
64	Ni	10.4404	12.1981	5.1909	0.7509	0.8774	0.2365
65	Co	1.7379	1.7379	0.0000	0.1250	0.1250	0.0000
66	Co	1.7379	5.2138	0.0000	0.1250	0.3750	0.0000
67	Co	5.2138	1.7379	0.0000	0.3750	0.1250	0.0000
68	Co	5.2138	5.2138	0.0000	0.3750	0.3750	0.0000
69	Co	1.7379	8.6896	0.0000	0.1250	0.6250	0.0000
70	Co	1.7379	12.1654	0.0000	0.1250	0.8750	0.0000
71	Co	5.2138	8.6896	0.0000	0.3750	0.6250	0.0000
72	Co	5.2138	12.1654	0.0000	0.3750	0.8750	0.0000
73	Co	8.6896	1.7379	0.0000	0.6250	0.1250	0.0000
74	Co	8.6896	5.2138	0.0000	0.6250	0.3750	0.0000
75	Co	12.1654	1.7379	0.0000	0.8750	0.1250	0.0000
76	Co	12.1654	5.2138	0.0000	0.8750	0.3750	0.0000
77	Co	8.6896	8.6896	0.0000	0.6250	0.6250	0.0000
78	Co	8.6896	12.1654	0.0000	0.6250	0.8750	0.0000
79	Co	12.1654	8.6896	0.0000	0.8750	0.6250	0.0000
80	Co	12.1654	12.1654	0.0000	0.8750	0.8750	0.0000
81	Co	1.7379	0.0000	1.7379	0.1250	0.0000	0.0792
82	Co	1.7379	3.4758	1.7379	0.1250	0.2500	0.0792
83	Co	5.2138	0.0000	1.7379	0.3750	0.0000	0.0792
84	Co	5.2138	3.4758	1.7379	0.3750	0.2500	0.0792
85	Co	1.7379	6.9517	1.7379	0.1250	0.5000	0.0792
86	Co	1.7379	10.4275	1.7379	0.1250	0.7500	0.0792
87	Co	5.2138	6.9517	1.7379	0.3750	0.5000	0.0792
88	Co	5.2138	10.4275	1.7379	0.3750	0.7500	0.0792

89	Co	8.6896	0.0000	1.7379	0.6250	0.0000	0.0792
90	Co	8.6896	3.4758	1.7379	0.6250	0.2500	0.0792
91	Co	12.1654	0.0000	1.7379	0.8750	0.0000	0.0792
92	Co	12.1654	3.4758	1.7379	0.8750	0.2500	0.0792
93	Co	8.6896	6.9517	1.7379	0.6250	0.5000	0.0792
94	Co	8.6896	10.4275	1.7379	0.6250	0.7500	0.0792
95	Co	12.1654	6.9517	1.7379	0.8750	0.5000	0.0792
96	Co	12.1654	10.4275	1.7379	0.8750	0.7500	0.0792
97	Co	1.7414	1.7317	3.5302	0.1252	0.1246	0.1608
98	Co	1.7243	5.2286	3.5366	0.1240	0.3761	0.1611
99	Co	5.2055	1.7589	3.5902	0.3744	0.1265	0.1636
100	Co	5.1918	5.1949	3.5544	0.3734	0.3736	0.1619
101	Co	1.7224	8.6730	3.5451	0.1239	0.6238	0.1615
102	Co	1.7363	12.1655	3.5448	0.1249	0.8750	0.1615
103	Co	5.2222	8.6964	3.5108	0.3756	0.6255	0.1599
104	Co	5.2217	12.1745	3.5460	0.3756	0.8757	0.1615
105	Co	8.6947	1.7397	3.5390	0.6254	0.1251	0.1612
106	Co	8.7295	5.1959	3.4929	0.6279	0.3737	0.1591
107	Co	12.1694	1.7327	3.5547	0.8753	0.1246	0.1619
108	Co	12.1628	5.2165	3.5458	0.8748	0.3752	0.1615
109	Co	8.6903	8.6933	3.5710	0.6250	0.6253	0.1627
110	Co	8.6823	12.1511	3.5442	0.6245	0.8740	0.1615
111	Co	12.1393	8.6955	3.5897	0.8731	0.6254	0.1635
112	Co	12.1669	12.1636	3.5449	0.8751	0.8749	0.1615
113	Co	1.7384	0.0013	5.2223	0.1250	0.0001	0.2379
114	Co	1.7157	3.4503	5.1686	0.1234	0.2482	0.2355
115	Co	5.2114	13.8998	5.2186	0.3748	0.9997	0.2377
116	Co	5.2403	3.4941	5.3985	0.3769	0.2513	0.2459
117	Co	1.7193	6.9773	5.3289	0.1237	0.5018	0.2428
118	Co	1.7558	10.4123	5.2205	0.1263	0.7489	0.2378
119	Co	5.2679	6.9362	5.1765	0.3789	0.4989	0.2358
120	Co	5.1847	10.4573	5.2043	0.3729	0.7521	0.2371
121	Co	8.6909	13.8998	5.2115	0.6251	0.9997	0.2374
122	Co	8.7025	3.4815	5.1976	0.6259	0.2504	0.2368
123	Co	12.1713	13.9021	5.2202	0.8754	0.9999	0.2378
124	Co	12.1672	3.4691	5.2227	0.8751	0.2495	0.2379
125	Co	8.6992	6.8718	5.1635	0.6257	0.4943	0.2352
126	Co	8.6727	10.4393	5.3335	0.6238	0.7509	0.2430
127	Co	12.1718	6.9520	5.2202	0.8755	0.5000	0.2378
128	Co	12.1973	10.4426	5.2156	0.8773	0.7511	0.2376
129	O	5.2450	3.5649	7.2667	0.3773	0.2564	0.3310
130	O	2.6693	6.2539	6.7919	0.1920	0.4498	0.3094
131	O	4.6945	5.9529	8.7879	0.3377	0.4282	0.4003
132	O	6.2556	7.5337	6.7795	0.4499	0.5419	0.3088
133	O	8.1021	6.8498	8.8946	0.5827	0.4927	0.4052
134	O	5.7332	9.3346	8.8859	0.4124	0.6714	0.4048
135	O	9.4383	9.5955	6.7413	0.6788	0.6902	0.3071
136	Ce	4.2428	8.1198	7.9658	0.3052	0.5840	0.3629
137	Ce	3.3248	4.3896	7.9475	0.2391	0.3157	0.3620

138 Ce	6.8238	5.4023	7.9180	0.4908	0.3886	0.3607
139 Sm	7.7173	8.8013	8.1600	0.5551	0.6330	0.3717

/db/jmorales/CoNi-alloy/metal-SDC-interface/CoNi-Ce_3SmO_7/CoNi-Ce_3SmO_7-conf5c

a = 13.9033384264
b = 13.9033384264
c = 21.9516692133
alpha = 90.0
beta = 90.0
gamma = 90.0

	Atom	X	Y	Z	X	Y	Z
1	Ni	0.0000	0.0000	0.0000	0.0000	0.0000	0.0000
2	Ni	0.0000	3.4758	0.0000	0.0000	0.2500	0.0000
3	Ni	3.4758	0.0000	0.0000	0.2500	0.0000	0.0000
4	Ni	3.4758	3.4758	0.0000	0.2500	0.2500	0.0000
5	Ni	0.0000	6.9517	0.0000	0.0000	0.5000	0.0000
6	Ni	0.0000	10.4275	0.0000	0.0000	0.7500	0.0000
7	Ni	3.4758	6.9517	0.0000	0.2500	0.5000	0.0000
8	Ni	3.4758	10.4275	0.0000	0.2500	0.7500	0.0000
9	Ni	6.9517	0.0000	0.0000	0.5000	0.0000	0.0000
10	Ni	6.9517	3.4758	0.0000	0.5000	0.2500	0.0000
11	Ni	10.4275	0.0000	0.0000	0.7500	0.0000	0.0000
12	Ni	10.4275	3.4758	0.0000	0.7500	0.2500	0.0000
13	Ni	6.9517	6.9517	0.0000	0.5000	0.5000	0.0000
14	Ni	6.9517	10.4275	0.0000	0.5000	0.7500	0.0000
15	Ni	10.4275	6.9517	0.0000	0.7500	0.5000	0.0000
16	Ni	10.4275	10.4275	0.0000	0.7500	0.7500	0.0000
17	Ni	0.0000	1.7379	1.7379	0.0000	0.1250	0.0792
18	Ni	0.0000	5.2138	1.7379	0.0000	0.3750	0.0792
19	Ni	3.4758	1.7379	1.7379	0.2500	0.1250	0.0792
20	Ni	3.4758	5.2138	1.7379	0.2500	0.3750	0.0792
21	Ni	0.0000	8.6896	1.7379	0.0000	0.6250	0.0792
22	Ni	0.0000	12.1654	1.7379	0.0000	0.8750	0.0792
23	Ni	3.4758	8.6896	1.7379	0.2500	0.6250	0.0792
24	Ni	3.4758	12.1654	1.7379	0.2500	0.8750	0.0792
25	Ni	6.9517	1.7379	1.7379	0.5000	0.1250	0.0792
26	Ni	6.9517	5.2138	1.7379	0.5000	0.3750	0.0792
27	Ni	10.4275	1.7379	1.7379	0.7500	0.1250	0.0792
28	Ni	10.4275	5.2138	1.7379	0.7500	0.3750	0.0792
29	Ni	6.9517	8.6896	1.7379	0.5000	0.6250	0.0792
30	Ni	6.9517	12.1654	1.7379	0.5000	0.8750	0.0792
31	Ni	10.4275	8.6896	1.7379	0.7500	0.6250	0.0792
32	Ni	10.4275	12.1654	1.7379	0.7500	0.8750	0.0792
33	Ni	0.0003	0.0008	3.5059	0.0000	0.0001	0.1597
34	Ni	13.8915	3.4718	3.4931	0.9991	0.2497	0.1591
35	Ni	3.4809	0.0055	3.5074	0.2504	0.0004	0.1598
36	Ni	3.5007	3.4945	3.5220	0.2518	0.2513	0.1604
37	Ni	0.0099	6.9479	3.5230	0.0007	0.4997	0.1605
38	Ni	13.9027	10.4282	3.5046	1.0000	0.7501	0.1597
39	Ni	3.4728	6.9514	3.4941	0.2498	0.5000	0.1592

40	Ni	3.4595	10.4539	3.4645	0.2488	0.7519	0.1578
41	Ni	6.9497	13.8941	3.5005	0.4999	0.9993	0.1595
42	Ni	6.9551	3.4706	3.4911	0.5002	0.2496	0.1590
43	Ni	10.4300	13.8972	3.5018	0.7502	0.9996	0.1595
44	Ni	10.4299	3.4735	3.4992	0.7502	0.2498	0.1594
45	Ni	6.9616	6.9270	3.4985	0.5007	0.4982	0.1594
46	Ni	6.9523	10.4201	3.5045	0.5000	0.7495	0.1596
47	Ni	10.4398	6.9546	3.4902	0.7509	0.5002	0.1590
48	Ni	10.4377	10.4261	3.4977	0.7507	0.7499	0.1593
49	Ni	13.8966	1.7380	5.2081	0.9995	0.1250	0.2373
50	Ni	13.8812	5.1867	5.1996	0.9984	0.3731	0.2369
51	Ni	3.4918	1.7514	5.2169	0.2512	0.1260	0.2377
52	Ni	3.4407	5.2279	5.2217	0.2475	0.3760	0.2379
53	Ni	0.0092	8.6902	5.2193	0.0007	0.6250	0.2378
54	Ni	0.0055	12.1655	5.2086	0.0004	0.8750	0.2373
55	Ni	3.4540	8.7409	5.0937	0.2484	0.6287	0.2320
56	Ni	3.4595	12.1709	5.2033	0.2488	0.8754	0.2370
57	Ni	6.9774	1.7053	5.1878	0.5019	0.1227	0.2363
58	Ni	6.9951	5.1909	5.2758	0.5031	0.3734	0.2403
59	Ni	10.4294	1.7198	5.2035	0.7501	0.1237	0.2370
60	Ni	10.4307	5.2093	5.2126	0.7502	0.3747	0.2375
61	Ni	6.9979	8.7112	5.2231	0.5033	0.6266	0.2379
62	Ni	6.9599	12.1475	5.2203	0.5006	0.8737	0.2378
63	Ni	10.4123	8.6947	5.2109	0.7489	0.6254	0.2374
64	Ni	10.4279	12.1632	5.2136	0.7500	0.8748	0.2375
65	Co	1.7379	1.7379	0.0000	0.1250	0.1250	0.0000
66	Co	1.7379	5.2138	0.0000	0.1250	0.3750	0.0000
67	Co	5.2138	1.7379	0.0000	0.3750	0.1250	0.0000
68	Co	5.2138	5.2138	0.0000	0.3750	0.3750	0.0000
69	Co	1.7379	8.6896	0.0000	0.1250	0.6250	0.0000
70	Co	1.7379	12.1654	0.0000	0.1250	0.8750	0.0000
71	Co	5.2138	8.6896	0.0000	0.3750	0.6250	0.0000
72	Co	5.2138	12.1654	0.0000	0.3750	0.8750	0.0000
73	Co	8.6896	1.7379	0.0000	0.6250	0.1250	0.0000
74	Co	8.6896	5.2138	0.0000	0.6250	0.3750	0.0000
75	Co	12.1654	1.7379	0.0000	0.8750	0.1250	0.0000
76	Co	12.1654	5.2138	0.0000	0.8750	0.3750	0.0000
77	Co	8.6896	8.6896	0.0000	0.6250	0.6250	0.0000
78	Co	8.6896	12.1654	0.0000	0.6250	0.8750	0.0000
79	Co	12.1654	8.6896	0.0000	0.8750	0.6250	0.0000
80	Co	12.1654	12.1654	0.0000	0.8750	0.8750	0.0000
81	Co	1.7379	0.0000	1.7379	0.1250	0.0000	0.0792
82	Co	1.7379	3.4758	1.7379	0.1250	0.2500	0.0792
83	Co	5.2138	0.0000	1.7379	0.3750	0.0000	0.0792
84	Co	5.2138	3.4758	1.7379	0.3750	0.2500	0.0792
85	Co	1.7379	6.9517	1.7379	0.1250	0.5000	0.0792
86	Co	1.7379	10.4275	1.7379	0.1250	0.7500	0.0792
87	Co	5.2138	6.9517	1.7379	0.3750	0.5000	0.0792
88	Co	5.2138	10.4275	1.7379	0.3750	0.7500	0.0792

89	Co	8.6896	0.0000	1.7379	0.6250	0.0000	0.0792
90	Co	8.6896	3.4758	1.7379	0.6250	0.2500	0.0792
91	Co	12.1654	0.0000	1.7379	0.8750	0.0000	0.0792
92	Co	12.1654	3.4758	1.7379	0.8750	0.2500	0.0792
93	Co	8.6896	6.9517	1.7379	0.6250	0.5000	0.0792
94	Co	8.6896	10.4275	1.7379	0.6250	0.7500	0.0792
95	Co	12.1654	6.9517	1.7379	0.8750	0.5000	0.0792
96	Co	12.1654	10.4275	1.7379	0.8750	0.7500	0.0792
97	Co	1.7459	1.7370	3.5492	0.1256	0.1249	0.1617
98	Co	1.7257	5.2193	3.5335	0.1241	0.3754	0.1610
99	Co	5.2148	1.7460	3.5608	0.3751	0.1256	0.1622
100	Co	5.2094	5.2227	3.5687	0.3747	0.3756	0.1626
101	Co	1.7071	8.6661	3.5306	0.1228	0.6233	0.1608
102	Co	1.7319	12.1757	3.5492	0.1246	0.8757	0.1617
103	Co	5.2251	8.7087	3.5063	0.3758	0.6264	0.1597
104	Co	5.2166	12.1736	3.5494	0.3752	0.8756	0.1617
105	Co	8.6956	1.7252	3.5399	0.6254	0.1241	0.1613
106	Co	8.6858	5.2072	3.5556	0.6247	0.3745	0.1620
107	Co	12.1646	1.7303	3.5473	0.8749	0.1245	0.1616
108	Co	12.1629	5.2112	3.5508	0.8748	0.3748	0.1618
109	Co	8.6899	8.6943	3.5350	0.6250	0.6253	0.1610
110	Co	8.6929	12.1671	3.5382	0.6252	0.8751	0.1612
111	Co	12.1681	8.6895	3.5648	0.8752	0.6250	0.1624
112	Co	12.1675	12.1653	3.5565	0.8751	0.8750	0.1620
113	Co	1.7426	0.0160	5.2298	0.1253	0.0011	0.2382
114	Co	1.7259	3.4418	5.1815	0.1241	0.2476	0.2360
115	Co	5.2199	13.8926	5.2289	0.3754	0.9992	0.2382
116	Co	5.2426	3.4791	5.2836	0.3771	0.2502	0.2407
117	Co	1.7403	6.9546	5.3673	0.1252	0.5002	0.2445
118	Co	1.7426	10.4193	5.2145	0.1253	0.7494	0.2375
119	Co	5.2282	7.0328	5.1838	0.3760	0.5058	0.2361
120	Co	5.1743	10.4628	5.1846	0.3722	0.7525	0.2362
121	Co	8.6883	13.8837	5.2277	0.6249	0.9986	0.2381
122	Co	8.7343	3.4472	5.2056	0.6282	0.2479	0.2371
123	Co	12.1675	13.8985	5.2268	0.8752	0.9997	0.2381
124	Co	12.1599	3.4669	5.2233	0.8746	0.2494	0.2379
125	Co	8.7354	6.9271	5.1773	0.6283	0.4982	0.2358
126	Co	8.7037	10.4504	5.2019	0.6260	0.7516	0.2370
127	Co	12.1647	6.9531	5.2189	0.8749	0.5001	0.2377
128	Co	12.1670	10.4263	5.2239	0.8751	0.7499	0.2380
129	O	6.0289	4.3233	6.7854	0.4336	0.3110	0.3091
130	O	3.5163	3.2381	9.4053	0.2529	0.2329	0.4285
131	O	2.6823	6.1329	6.8071	0.1929	0.4411	0.3101
132	O	4.9134	6.2355	8.7824	0.3534	0.4485	0.4001
133	O	6.0336	7.8997	6.7669	0.4340	0.5682	0.3083
134	O	7.9172	8.1189	8.8687	0.5694	0.5840	0.4040
135	O	5.0520	9.8219	8.6347	0.3634	0.7064	0.3934
136	Ce	3.8882	8.1330	7.9282	0.2797	0.5850	0.3612
137	Ce	3.9012	4.3679	7.9672	0.2806	0.3142	0.3629

138 Ce	7.1204	6.2651	8.1084	0.5121	0.4506	0.3694
139 Sm	7.0372	9.7987	7.9430	0.5062	0.7048	0.3618

/db/jmorales/CoNi-alloy/metal-SDC-interface/Ni-Ce_3SmO_7/Ni-Ce_3SmO_7-conf5a

a =	13.9019837372
b =	13.9019837372
c =	21.9509918687
alpha =	90.0
beta =	90.0
gamma =	90.0

	Atom	X	Y	Z	X	Y	Z
1	Ni	0.0000	0.0000	0.0000	0.0000	0.0000	0.0000
2	Ni	1.7377	1.7377	0.0000	0.1250	0.1250	0.0000
3	Ni	0.0000	3.4755	0.0000	0.0000	0.2500	0.0000
4	Ni	1.7377	5.2132	0.0000	0.1250	0.3750	0.0000
5	Ni	3.4755	0.0000	0.0000	0.2500	0.0000	0.0000
6	Ni	5.2132	1.7377	0.0000	0.3750	0.1250	0.0000
7	Ni	3.4755	3.4755	0.0000	0.2500	0.2500	0.0000
8	Ni	5.2132	5.2132	0.0000	0.3750	0.3750	0.0000
9	Ni	0.0000	6.9510	0.0000	0.0000	0.5000	0.0000
10	Ni	1.7377	8.6887	0.0000	0.1250	0.6250	0.0000
11	Ni	0.0000	10.4265	0.0000	0.0000	0.7500	0.0000
12	Ni	1.7377	12.1642	0.0000	0.1250	0.8750	0.0000
13	Ni	3.4755	6.9510	0.0000	0.2500	0.5000	0.0000
14	Ni	5.2132	8.6887	0.0000	0.3750	0.6250	0.0000
15	Ni	3.4755	10.4265	0.0000	0.2500	0.7500	0.0000
16	Ni	5.2132	12.1642	0.0000	0.3750	0.8750	0.0000
17	Ni	6.9510	0.0000	0.0000	0.5000	0.0000	0.0000
18	Ni	8.6887	1.7377	0.0000	0.6250	0.1250	0.0000
19	Ni	6.9510	3.4755	0.0000	0.5000	0.2500	0.0000
20	Ni	8.6887	5.2132	0.0000	0.6250	0.3750	0.0000
21	Ni	10.4265	0.0000	0.0000	0.7500	0.0000	0.0000
22	Ni	12.1642	1.7377	0.0000	0.8750	0.1250	0.0000
23	Ni	10.4265	3.4755	0.0000	0.7500	0.2500	0.0000
24	Ni	12.1642	5.2132	0.0000	0.8750	0.3750	0.0000
25	Ni	6.9510	6.9510	0.0000	0.5000	0.5000	0.0000
26	Ni	8.6887	8.6887	0.0000	0.6250	0.6250	0.0000
27	Ni	6.9510	10.4265	0.0000	0.5000	0.7500	0.0000
28	Ni	8.6887	12.1642	0.0000	0.6250	0.8750	0.0000
29	Ni	10.4265	6.9510	0.0000	0.7500	0.5000	0.0000
30	Ni	12.1642	8.6887	0.0000	0.8750	0.6250	0.0000
31	Ni	10.4265	10.4265	0.0000	0.7500	0.7500	0.0000
32	Ni	12.1642	12.1642	0.0000	0.8750	0.8750	0.0000
33	Ni	0.0000	1.7377	1.7377	0.0000	0.1250	0.0792
34	Ni	1.7377	0.0000	1.7377	0.1250	0.0000	0.0792
35	Ni	0.0000	5.2132	1.7377	0.0000	0.3750	0.0792
36	Ni	1.7377	3.4755	1.7377	0.1250	0.2500	0.0792
37	Ni	3.4755	1.7377	1.7377	0.2500	0.1250	0.0792
38	Ni	5.2132	0.0000	1.7377	0.3750	0.0000	0.0792
39	Ni	3.4755	5.2132	1.7377	0.2500	0.3750	0.0792
40	Ni	5.2132	3.4755	1.7377	0.3750	0.2500	0.0792

41	Ni	0.0000	8.6887	1.7377	0.0000	0.6250	0.0792
42	Ni	1.7377	6.9510	1.7377	0.1250	0.5000	0.0792
43	Ni	0.0000	12.1642	1.7377	0.0000	0.8750	0.0792
44	Ni	1.7377	10.4265	1.7377	0.1250	0.7500	0.0792
45	Ni	3.4755	8.6887	1.7377	0.2500	0.6250	0.0792
46	Ni	5.2132	6.9510	1.7377	0.3750	0.5000	0.0792
47	Ni	3.4755	12.1642	1.7377	0.2500	0.8750	0.0792
48	Ni	5.2132	10.4265	1.7377	0.3750	0.7500	0.0792
49	Ni	6.9510	1.7377	1.7377	0.5000	0.1250	0.0792
50	Ni	8.6887	0.0000	1.7377	0.6250	0.0000	0.0792
51	Ni	6.9510	5.2132	1.7377	0.5000	0.3750	0.0792
52	Ni	8.6887	3.4755	1.7377	0.6250	0.2500	0.0792
53	Ni	10.4265	1.7377	1.7377	0.7500	0.1250	0.0792
54	Ni	12.1642	0.0000	1.7377	0.8750	0.0000	0.0792
55	Ni	10.4265	5.2132	1.7377	0.7500	0.3750	0.0792
56	Ni	12.1642	3.4755	1.7377	0.8750	0.2500	0.0792
57	Ni	6.9510	8.6887	1.7377	0.5000	0.6250	0.0792
58	Ni	8.6887	6.9510	1.7377	0.6250	0.5000	0.0792
59	Ni	6.9510	12.1642	1.7377	0.5000	0.8750	0.0792
60	Ni	8.6887	10.4265	1.7377	0.6250	0.7500	0.0792
61	Ni	10.4265	8.6887	1.7377	0.7500	0.6250	0.0792
62	Ni	12.1642	6.9510	1.7377	0.8750	0.5000	0.0792
63	Ni	10.4265	12.1642	1.7377	0.7500	0.8750	0.0792
64	Ni	12.1642	10.4265	1.7377	0.8750	0.7500	0.0792
65	Ni	0.0032	13.8979	3.5097	0.0002	0.9997	0.1599
66	Ni	1.7334	1.7503	3.5138	0.1247	0.1259	0.1601
67	Ni	13.8803	3.4718	3.4956	0.9984	0.2497	0.1592
68	Ni	1.7237	5.2027	3.4287	0.1240	0.3742	0.1562
69	Ni	3.4751	13.8985	3.5057	0.2500	0.9998	0.1597
70	Ni	5.2205	1.7534	3.5330	0.3755	0.1261	0.1609
71	Ni	3.5023	3.4656	3.5035	0.2519	0.2493	0.1596
72	Ni	5.1979	5.1954	3.5279	0.3739	0.3737	0.1607
73	Ni	13.8793	6.9524	3.4913	0.9984	0.5001	0.1591
74	Ni	1.7469	8.6749	3.5247	0.1257	0.6240	0.1606
75	Ni	0.0056	10.4257	3.5110	0.0004	0.7499	0.1599
76	Ni	1.7434	12.1604	3.5148	0.1254	0.8747	0.1601
77	Ni	3.4821	6.9567	3.4821	0.2505	0.5004	0.1586
78	Ni	5.2171	8.6983	3.4916	0.3753	0.6257	0.1591
79	Ni	3.4698	10.4104	3.5184	0.2496	0.7488	0.1603
80	Ni	5.2123	12.1790	3.4968	0.3749	0.8761	0.1593
81	Ni	6.9499	0.0004	3.5104	0.4999	0.0000	0.1599
82	Ni	8.6879	1.7385	3.5082	0.6249	0.1251	0.1598
83	Ni	6.9317	3.4690	3.5235	0.4986	0.2495	0.1605
84	Ni	8.7006	5.2136	3.4971	0.6259	0.3750	0.1593
85	Ni	10.4267	13.8993	3.5091	0.7500	0.9998	0.1599
86	Ni	12.1598	1.7298	3.5081	0.8747	0.1244	0.1598
87	Ni	10.4292	3.4755	3.5084	0.7502	0.2500	0.1598
88	Ni	12.1773	5.2124	3.5339	0.8759	0.3749	0.1610
89	Ni	6.9428	6.9562	3.4989	0.4994	0.5004	0.1594

90	Ni	8.7009	8.6890	3.5031	0.6259	0.6250	0.1596
91	Ni	6.9662	10.4353	3.4971	0.5011	0.7506	0.1593
92	Ni	8.6913	12.1665	3.5088	0.6252	0.8752	0.1598
93	Ni	10.4326	6.9503	3.5114	0.7504	0.4999	0.1600
94	Ni	12.1597	8.6960	3.5089	0.8747	0.6255	0.1598
95	Ni	10.4268	10.4300	3.5129	0.7500	0.7503	0.1600
96	Ni	12.1660	12.1620	3.5108	0.8751	0.8748	0.1599
97	Ni	13.8986	1.7333	5.2209	0.9998	0.1247	0.2378
98	Ni	1.7429	0.0026	5.2285	0.1254	0.0002	0.2382
99	Ni	13.8875	5.2006	5.2937	0.9990	0.3741	0.2412
100	Ni	1.7200	3.4606	5.2146	0.1237	0.2489	0.2376
101	Ni	3.4628	1.7292	5.1965	0.2491	0.1244	0.2367
102	Ni	5.2084	13.9000	5.2265	0.3747	0.9999	0.2381
103	Ni	3.4380	5.2056	5.1894	0.2473	0.3745	0.2364
104	Ni	5.2462	3.4790	5.4538	0.3774	0.2503	0.2485
105	Ni	0.0028	8.6885	5.2232	0.0002	0.6250	0.2379
106	Ni	1.6937	6.9376	5.1778	0.1218	0.4990	0.2359
107	Ni	0.0039	12.1639	5.2270	0.0003	0.8750	0.2381
108	Ni	1.7388	10.4199	5.2354	0.1251	0.7495	0.2385
109	Ni	3.4756	8.7015	5.2939	0.2500	0.6259	0.2412
110	Ni	5.2580	6.9335	5.2322	0.3782	0.4987	0.2384
111	Ni	3.4853	12.1532	5.2305	0.2507	0.8742	0.2383
112	Ni	5.2365	10.4515	5.1673	0.3767	0.7518	0.2354
113	Ni	6.9706	1.7188	5.2287	0.5014	0.1236	0.2382
114	Ni	8.6922	13.8947	5.2251	0.6253	0.9995	0.2380
115	Ni	6.9812	5.2265	5.1705	0.5022	0.3760	0.2355
116	Ni	8.6905	3.4806	5.2195	0.6251	0.2504	0.2378
117	Ni	10.4224	1.7342	5.2240	0.7497	0.1247	0.2380
118	Ni	12.1633	-0.0012	5.2239	0.8749	-0.0001	0.2380
119	Ni	10.4258	5.2140	5.2298	0.7500	0.3751	0.2383
120	Ni	12.1556	3.4631	5.2249	0.8744	0.2491	0.2380
121	Ni	6.9703	8.7150	5.1941	0.5014	0.6269	0.2366
122	Ni	8.6833	6.9520	5.2219	0.6246	0.5001	0.2379
123	Ni	6.9483	12.1635	5.2331	0.4998	0.8749	0.2384
124	Ni	8.6888	10.4310	5.2313	0.6250	0.7503	0.2383
125	Ni	10.4260	8.6889	5.2280	0.7500	0.6250	0.2382
126	Ni	12.1651	6.9607	5.2292	0.8751	0.5007	0.2382
127	Ni	10.4282	12.1637	5.2284	0.7501	0.8750	0.2382
128	Ni	12.1678	10.4242	5.2271	0.8753	0.7498	0.2381
129	O	5.1254	3.6477	7.2913	0.3687	0.2624	0.3322
130	O	2.5032	2.4458	9.4045	0.1801	0.1759	0.4284
131	O	1.7311	5.1835	6.3119	0.1245	0.3729	0.2875
132	O	3.5549	5.6158	8.7062	0.2557	0.4040	0.3966
133	O	4.3265	7.6683	6.7488	0.3112	0.5516	0.3074
134	O	6.3351	7.8587	8.7835	0.4557	0.5653	0.4001
135	O	3.2738	9.0983	8.8762	0.2355	0.6545	0.4044
136	Ce	2.3135	7.4015	8.0394	0.1664	0.5324	0.3662
137	Ce	2.9852	3.5116	7.9438	0.2147	0.2526	0.3619
138	Ce	5.7431	5.9037	8.0043	0.4131	0.4247	0.3646

139 Sm	5.1891	9.4243	7.9872	0.3733	0.6779	0.3639

/db/jmorales/CoNi-alloy/metal-SDC-interface/Ni-Ce_3SmO_7/Ni-Ce_3SmO_7-conf1a

a = 13.9019837372
b = 13.9019837372
c = 21.9509918687
alpha = 90.0
beta = 90.0
gamma = 90.0

	Atom	X	Y	Z	X	Y	Z
1	Ni	0.0000	0.0000	0.0000	0.0000	0.0000	0.0000
2	Ni	1.7377	1.7377	0.0000	0.1250	0.1250	0.0000
3	Ni	0.0000	3.4755	0.0000	0.0000	0.2500	0.0000
4	Ni	1.7377	5.2132	0.0000	0.1250	0.3750	0.0000
5	Ni	3.4755	0.0000	0.0000	0.2500	0.0000	0.0000
6	Ni	5.2132	1.7377	0.0000	0.3750	0.1250	0.0000
7	Ni	3.4755	3.4755	0.0000	0.2500	0.2500	0.0000
8	Ni	5.2132	5.2132	0.0000	0.3750	0.3750	0.0000
9	Ni	0.0000	6.9510	0.0000	0.0000	0.5000	0.0000
10	Ni	1.7377	8.6887	0.0000	0.1250	0.6250	0.0000
11	Ni	0.0000	10.4265	0.0000	0.0000	0.7500	0.0000
12	Ni	1.7377	12.1642	0.0000	0.1250	0.8750	0.0000
13	Ni	3.4755	6.9510	0.0000	0.2500	0.5000	0.0000
14	Ni	5.2132	8.6887	0.0000	0.3750	0.6250	0.0000
15	Ni	3.4755	10.4265	0.0000	0.2500	0.7500	0.0000
16	Ni	5.2132	12.1642	0.0000	0.3750	0.8750	0.0000
17	Ni	6.9510	0.0000	0.0000	0.5000	0.0000	0.0000
18	Ni	8.6887	1.7377	0.0000	0.6250	0.1250	0.0000
19	Ni	6.9510	3.4755	0.0000	0.5000	0.2500	0.0000
20	Ni	8.6887	5.2132	0.0000	0.6250	0.3750	0.0000
21	Ni	10.4265	0.0000	0.0000	0.7500	0.0000	0.0000
22	Ni	12.1642	1.7377	0.0000	0.8750	0.1250	0.0000
23	Ni	10.4265	3.4755	0.0000	0.7500	0.2500	0.0000
24	Ni	12.1642	5.2132	0.0000	0.8750	0.3750	0.0000
25	Ni	6.9510	6.9510	0.0000	0.5000	0.5000	0.0000
26	Ni	8.6887	8.6887	0.0000	0.6250	0.6250	0.0000
27	Ni	6.9510	10.4265	0.0000	0.5000	0.7500	0.0000
28	Ni	8.6887	12.1642	0.0000	0.6250	0.8750	0.0000
29	Ni	10.4265	6.9510	0.0000	0.7500	0.5000	0.0000
30	Ni	12.1642	8.6887	0.0000	0.8750	0.6250	0.0000
31	Ni	10.4265	10.4265	0.0000	0.7500	0.7500	0.0000
32	Ni	12.1642	12.1642	0.0000	0.8750	0.8750	0.0000
33	Ni	0.0000	1.7377	1.7377	0.0000	0.1250	0.0792
34	Ni	1.7377	0.0000	1.7377	0.1250	0.0000	0.0792
35	Ni	0.0000	5.2132	1.7377	0.0000	0.3750	0.0792
36	Ni	1.7377	3.4755	1.7377	0.1250	0.2500	0.0792
37	Ni	3.4755	1.7377	1.7377	0.2500	0.1250	0.0792
38	Ni	5.2132	0.0000	1.7377	0.3750	0.0000	0.0792
39	Ni	3.4755	5.2132	1.7377	0.2500	0.3750	0.0792
40	Ni	5.2132	3.4755	1.7377	0.3750	0.2500	0.0792

41	Ni	0.0000	8.6887	1.7377	0.0000	0.6250	0.0792
42	Ni	1.7377	6.9510	1.7377	0.1250	0.5000	0.0792
43	Ni	0.0000	12.1642	1.7377	0.0000	0.8750	0.0792
44	Ni	1.7377	10.4265	1.7377	0.1250	0.7500	0.0792
45	Ni	3.4755	8.6887	1.7377	0.2500	0.6250	0.0792
46	Ni	5.2132	6.9510	1.7377	0.3750	0.5000	0.0792
47	Ni	3.4755	12.1642	1.7377	0.2500	0.8750	0.0792
48	Ni	5.2132	10.4265	1.7377	0.3750	0.7500	0.0792
49	Ni	6.9510	1.7377	1.7377	0.5000	0.1250	0.0792
50	Ni	8.6887	0.0000	1.7377	0.6250	0.0000	0.0792
51	Ni	6.9510	5.2132	1.7377	0.5000	0.3750	0.0792
52	Ni	8.6887	3.4755	1.7377	0.6250	0.2500	0.0792
53	Ni	10.4265	1.7377	1.7377	0.7500	0.1250	0.0792
54	Ni	12.1642	0.0000	1.7377	0.8750	0.0000	0.0792
55	Ni	10.4265	5.2132	1.7377	0.7500	0.3750	0.0792
56	Ni	12.1642	3.4755	1.7377	0.8750	0.2500	0.0792
57	Ni	6.9510	8.6887	1.7377	0.5000	0.6250	0.0792
58	Ni	8.6887	6.9510	1.7377	0.6250	0.5000	0.0792
59	Ni	6.9510	12.1642	1.7377	0.5000	0.8750	0.0792
60	Ni	8.6887	10.4265	1.7377	0.6250	0.7500	0.0792
61	Ni	10.4265	8.6887	1.7377	0.7500	0.6250	0.0792
62	Ni	12.1642	6.9510	1.7377	0.8750	0.5000	0.0792
63	Ni	10.4265	12.1642	1.7377	0.7500	0.8750	0.0792
64	Ni	12.1642	10.4265	1.7377	0.8750	0.7500	0.0792
65	Ni	13.9016	13.9003	3.5089	1.0000	0.9999	0.1599
66	Ni	1.7324	1.7512	3.5108	0.1246	0.1260	0.1599
67	Ni	13.8846	3.4752	3.4978	0.9987	0.2500	0.1593
68	Ni	1.7259	5.2116	3.4412	0.1241	0.3749	0.1568
69	Ni	3.4745	13.8987	3.5007	0.2499	0.9998	0.1595
70	Ni	5.2244	1.7467	3.5262	0.3758	0.1256	0.1606
71	Ni	3.5052	3.4644	3.5025	0.2521	0.2492	0.1596
72	Ni	5.1977	5.1992	3.5310	0.3739	0.3740	0.1609
73	Ni	13.8936	6.9477	3.5060	0.9994	0.4998	0.1597
74	Ni	1.7242	8.6746	3.5097	0.1240	0.6240	0.1599
75	Ni	13.9019	10.4278	3.5092	1.0000	0.7501	0.1599
76	Ni	1.7337	12.1643	3.5097	0.1247	0.8750	0.1599
77	Ni	3.4800	6.9506	3.4757	0.2503	0.5000	0.1583
78	Ni	5.2143	8.6953	3.4784	0.3751	0.6255	0.1585
79	Ni	3.4859	10.4453	3.5068	0.2507	0.7514	0.1598
80	Ni	5.2175	12.1697	3.5050	0.3753	0.8754	0.1597
81	Ni	6.9527	13.8786	3.5318	0.5001	0.9983	0.1609
82	Ni	8.6876	1.7371	3.5076	0.6249	0.1250	0.1598
83	Ni	6.9310	3.4638	3.5164	0.4986	0.2492	0.1602
84	Ni	8.7048	5.2109	3.4910	0.6262	0.3748	0.1590
85	Ni	10.4287	-0.0005	3.5077	0.7502	-0.0000	0.1598
86	Ni	12.1623	1.7342	3.5084	0.8749	0.1247	0.1598
87	Ni	10.4345	3.4768	3.5093	0.7506	0.2501	0.1599
88	Ni	12.1889	5.2128	3.5412	0.8768	0.3750	0.1613
89	Ni	6.9461	6.9679	3.4999	0.4997	0.5012	0.1594

90	Ni	8.7034	8.6879	3.4985	0.6261	0.6249	0.1594
91	Ni	6.9573	10.4364	3.4461	0.5005	0.7507	0.1570
92	Ni	8.6900	12.1612	3.5104	0.6251	0.8748	0.1599
93	Ni	10.4392	6.9456	3.5080	0.7509	0.4996	0.1598
94	Ni	12.1642	8.6938	3.5097	0.8750	0.6254	0.1599
95	Ni	10.4100	10.4267	3.5308	0.7488	0.7500	0.1608
96	Ni	12.1635	12.1610	3.5095	0.8749	0.8748	0.1599
97	Ni	13.8981	1.7323	5.2224	0.9997	0.1246	0.2379
98	Ni	1.7387	0.0075	5.2267	0.1251	0.0005	0.2381
99	Ni	0.0150	5.2115	5.2991	0.0011	0.3749	0.2414
100	Ni	1.7264	3.4494	5.2135	0.1242	0.2481	0.2375
101	Ni	3.4627	1.7235	5.1940	0.2491	0.1240	0.2366
102	Ni	5.2045	0.0009	5.2206	0.3744	0.0001	0.2378
103	Ni	3.4508	5.2132	5.1915	0.2482	0.3750	0.2365
104	Ni	5.2574	3.4622	5.4350	0.3782	0.2490	0.2476
105	Ni	0.0009	8.6903	5.2317	0.0001	0.6251	0.2383
106	Ni	1.7366	6.9669	5.2423	0.1249	0.5011	0.2388
107	Ni	0.0013	12.1647	5.2254	0.0001	0.8750	0.2380
108	Ni	1.7397	10.4220	5.2236	0.1251	0.7497	0.2380
109	Ni	3.4545	8.7075	5.1445	0.2485	0.6263	0.2344
110	Ni	5.2083	6.9456	5.2384	0.3746	0.4996	0.2386
111	Ni	3.4705	12.1702	5.2220	0.2496	0.8754	0.2379
112	Ni	5.2031	10.4311	5.2465	0.3743	0.7503	0.2390
113	Ni	6.9779	1.7101	5.2272	0.5019	0.1230	0.2381
114	Ni	8.7036	13.8950	5.2248	0.6261	0.9995	0.2380
115	Ni	6.9862	5.2204	5.1456	0.5025	0.3755	0.2344
116	Ni	8.6890	3.4821	5.2175	0.6250	0.2505	0.2377
117	Ni	10.4245	1.7383	5.2232	0.7499	0.1250	0.2379
118	Ni	12.1619	0.0009	5.2245	0.8748	0.0001	0.2380
119	Ni	10.4272	5.2133	5.2322	0.7501	0.3750	0.2384
120	Ni	12.1620	3.4666	5.2292	0.8748	0.2494	0.2382
121	Ni	6.9768	8.6939	5.1971	0.5019	0.6254	0.2368
122	Ni	8.6941	6.9406	5.2138	0.6254	0.4992	0.2375
123	Ni	6.9478	12.1596	5.2982	0.4998	0.8747	0.2414
124	Ni	8.6954	10.4236	5.2938	0.6255	0.7498	0.2412
125	Ni	10.4322	8.6777	5.2256	0.7504	0.6242	0.2381
126	Ni	12.1675	6.9554	5.2302	0.8752	0.5003	0.2383
127	Ni	10.4326	12.1665	5.2237	0.7504	0.8752	0.2380
128	Ni	12.1633	10.4246	5.2278	0.8749	0.7499	0.2382
129	O	5.2100	3.4282	7.3251	0.3748	0.2466	0.3337
130	O	1.8154	5.1577	6.3497	0.1306	0.3710	0.2893
131	O	3.8522	5.5456	8.7191	0.2771	0.3989	0.3972
132	O	5.1805	7.4673	7.0414	0.3726	0.5371	0.3208
133	O	7.0799	7.3085	9.0520	0.5093	0.5257	0.4124
134	O	4.1688	9.1114	9.0099	0.2999	0.6554	0.4105
135	O	6.9089	10.3601	6.3064	0.4970	0.7452	0.2873
136	Ce	3.0607	7.6115	7.9885	0.2202	0.5475	0.3639
137	Ce	3.1113	3.6611	7.7874	0.2238	0.2634	0.3548
138	Ce	6.1927	5.5922	8.0294	0.4455	0.4023	0.3658

139 Sm	6.1961	9.0787	8.2726	0.4457	0.6530	0.3769
